# Synthesis of Hetero-bifunctional, End-Capped Oligo-EDOT Derivatives

**DOI:** 10.1016/j.chempr.2016.12.003

**Published:** 2017-01-12

**Authors:** Christopher D. Spicer, Marsilea A. Booth, Damia Mawad, Astrid Armgarth, Christian B. Nielsen, Molly M. Stevens

**Affiliations:** 1 Departments of Materials and Bioengineering, Institute of Biomedical Engineering, Imperial College London, London SW7 2AZ, UK; 2 Materials Research Institute and School of Biological and Chemical Sciences, Queen Mary University of London, London E1 4NS, UK

**Keywords:** thiophene, oligomer, conjugated polymer, C-H activation, end capping, synthesis, SDG3: Good health and well-being, SDG7: Affordable and clean energy

## Abstract

Conjugated oligomers of 3,4-ethylenedioxythiophene (EDOT) are attractive materials for tissue engineering applications and as model systems for studying the properties of the widely used polymer poly(3,4-ethylenedioxythiophene). We report here the facile synthesis of a series of keto-acid end-capped oligo-EDOT derivatives (n = 2–7) through a combination of a glyoxylation end-capping strategy and iterative direct arylation chain extension. Importantly, these structures not only represent the longest oligo-EDOTs reported but are also bench stable, in contrast to previous reports on such oligomers. The constructs reported here can undergo subsequent derivatization for integration into higher-order architectures, such as those required for tissue engineering applications. The synthesis of hetero-bifunctional constructs, as well as those containing mixed-monomer units, is also reported, allowing further complexity to be installed in a controlled manner. Finally, we describe the optical and electrochemical properties of these oligomers and demonstrate the importance of the keto-acid in determining their characteristics.

## Highlights

• A keto-acid end-capping strategy has been used to create stable oligo-EDOTs
• Oligomers can be synthesized in a facile manner via iterative direct arylation
• Hetero-bifunctional and mixed-monomer constructs can be controllably synthesized
• The keto-acid end group determines oligomer optical and electrochemical properties

## The Bigger Picture

The production of materials that can aid the repair, regrowth, or replacement of damaged tissue is a key challenge in tissue engineering. In this context, conjugated polymers have been proposed as attractive materials for the engineering of electroactive tissues such as the heart. Although there has been much progress in the field, the use of conjugated polymers is still hindered by their high heterogeneity, stiffness, poor solubility, and lack of chemical functionality. Therefore, there is a pressing need to produce new routes to create constructs such as those reported here, which offer homogeneity, stability, ease of synthesis, and most importantly, flexibility of design. This versatility allows the incorporation of conjugated structures into the higher-order biomaterial architectures required for tissue engineering, as well as tunable solubility and material properties. It is anticipated that this report will open the door to an exciting new chapter in the use of EDOT in biology.

## Graphical Abstract

**Figure:**
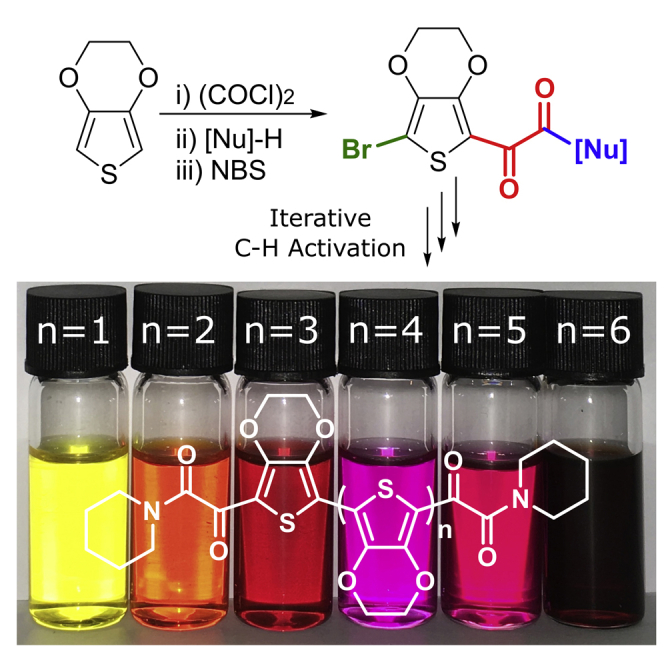

## Teaser

The synthesis and characterization of a series of keto-acid end-capped conjugated oligomers (n = 2–7) based around the monomer EDOT is reported. The use of direct arylation chain extension allows the synthesis of stable structures, which represent the longest reported EDOT oligomers to date with tunable properties based around the versatile end-capping group and monomer composition. These constructs can undergo subsequent derivatization, allowing them to be integrated into functional materials, such as those required for tissue engineering applications.

## Introduction

Conjugated polymers (CPs) are promising materials for tissue engineering applications.^1^, ^2^, ^3^, ^4^ However, further developments are required in order to allow their full potential to be realized in the biomedical field. Although initial investigations have shown CPs to be able to modulate cellular growth,^5^ migration,^6^ and differentiation,^7^, ^8^ as well as protein adhesion and conformation,^9^ difficulties remain as a consequence of their poor material characteristics, difficult processing, and lack of biodegradability.^1^, ^2^, ^10^ Further, the production of constructs bearing reactive functionalities for integration into more complex scaffold architectures remains challenging.^2^

In order to address these issues, there is increasing interest in the use of oligomers rather than polymeric systems. Although oligomers are often more synthetically complex,^11^ they offer the benefits of a defined molecular structure, improved solubility, tunability, and additional chemical functionality.^2^, ^12^ Oligomers can also act as mono-disperse model systems for studying the electronic and optical properties of the parent polymer, for which such investigations can be hindered.^13^

**Figure 1:**
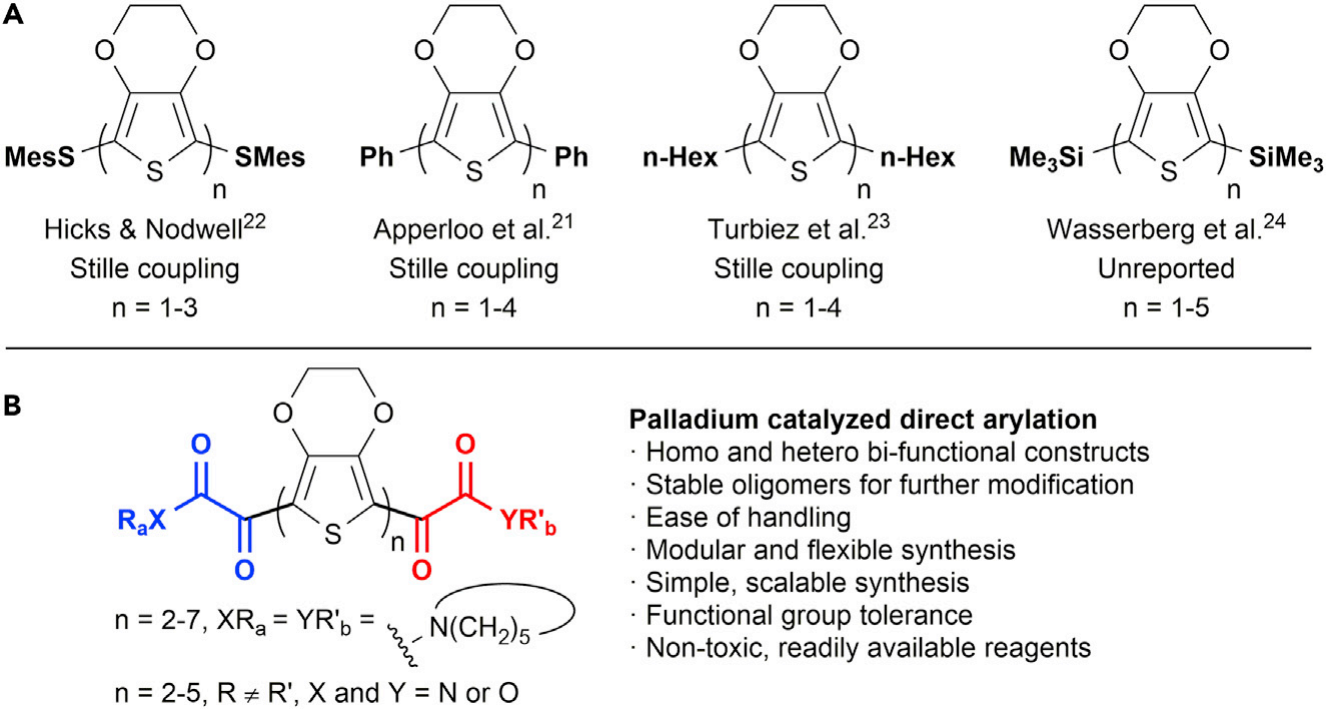
Previous EDOT End Caps and the Concept of This Report (A) Previous reports of the synthesis of EDOT oligomers. (B) Keto-acid-capped oligomers presented in this work. These oligomers were synthesized by direct arylation.

**Scheme 1:**
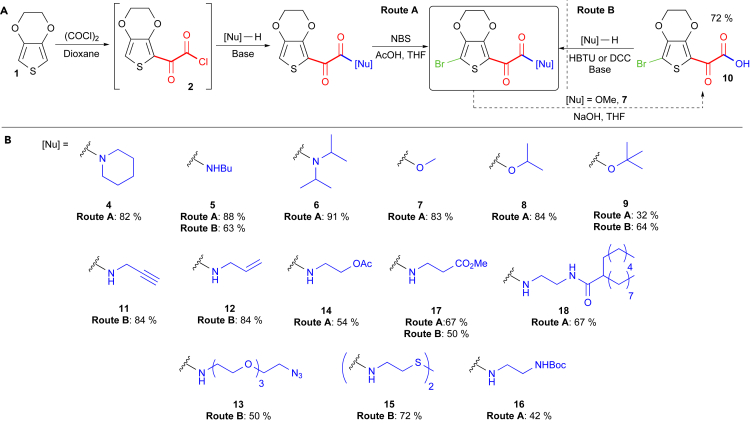
EDOT Glyoxylation and Functionalization (A) Treatment of EDOT with oxalyl chloride and subsequent treatment with the desired nucleophile generates functional end-capped derivatives, which can then undergo bromination (route A). Alternatively, amide or ester coupling can be undertaken from a common intermediate **10** to give the desired monomers (route B). (B) Functionalized brominated monomers synthesized.

Poly(3,4-ethylenedioxythiophene) (PEDOT) is a particularly attractive material for tissue engineering because of its electrical and chemical stability and high conductivity when doped with polymeric ionomers such as polystyrene sulfonate.^14^, ^15^ Although the synthesis of thiophene-based oligomers has been widely reported,^11^, ^16^, ^17^, ^18^, ^19^ those of EDOT (**1**; Scheme 1) have generated comparatively little interest, largely as a consequence of the poor oxidative stability and low solubility of the oligomers.^20^, ^21^ Mesityl,^22^ phenyl,^21^ *n*-hexyl,^23^ and trimethylsilyl^24^ capping groups have all been reported. However, longer oligomers were found to be unstable in solution, very poorly soluble, and difficult to purify, limiting their utility. Indeed, there remains only a single report on the synthesis of a pentameric species, but no synthetic details were reported^24^ (Figure 1A). Furthermore, the end caps utilized offer no opportunities for further chemical derivatization and subsequent incorporation into more complex structures.

Here, we report the facile synthesis and characterization of bench-stable oligo-EDOT derivatives, up to n = 7, produced via a glyoxylation keto-acid end-capping strategy and iterative C–H activation chemistry. Importantly, this allows the production of hetero-bifunctional constructs with a wide range of functional handles for further modification (Figure 1B). These motifs allow additional integration into more challenging substrates, such as those required for tissue engineering applications.

## Results and Discussion

### Oligomer Synthesis

Our initial designs were inspired by reports of thiophene glyoxylation with oxalyl chloride.^25^ We reasoned that the intermediate glyoxylyl chloride **2** could be reacted in situ with a range of nucleophiles to generate α-functionalized EDOT derivatives (Scheme 1). Importantly, the choice of nucleophile would have little influence on aromatic stability, allowing for a range of diverse constructs to be produced. After treatment of EDOT with 1 equiv of oxalyl chloride, the intermediate chloride **2** reacted smoothly with piperidine to generate the tertiary keto-amide **3** (Scheme 1A; Figures S6 and S7) in good yield. Subsequent bromination with *N*-bromosuccinimide yielded the di-functional monomer **4** on a multi-gram scale (Figures S8 and S9).^22^

A range of functionalized monomers could be produced by this method, including secondary amines (**5**), hindered tertiary amines (**6**), esters (**7**, **8**, and **9**), and monomers bearing functional groups for further modifications (Scheme 1, route A; Figures S10–S19, S73, S74, S91, S92, S99, S100, and S129–S132). In addition, hydrolysis of brominated-EDOT methyl ester **7** and subsequent amide or ester coupling allowed the synthesis of a range of di-functional monomers from a common intermediate **10** (Scheme 1, route B; Figures S20 and S21). Thus, monomers containing orthogonal reactive groups for further conjugation, such as alkynes (**11**), alkenes (**12**), azides (**13**), and protected alcohols (**14**), thiols (**15**), and amines (**16**), could all be produced in good yields in a simple fashion (Figures S22–S37, S125–S128, and S133–S150).^26^

**Scheme 2:**
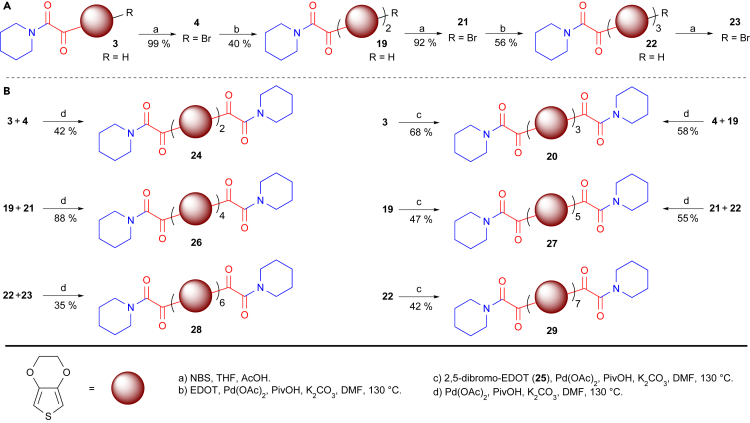
Synthesis of Piperidine End-Capped Homo-bifunctional EDOT Oligomers (A) Chain extension of mono-functional (n = 1–3) piperidine-capped oligomers. (B) Convergent coupling to generate bifunctional piperidine end-capped constructs (n = 2–7).

Next, we investigated the chain extension of brominated monomer **4** to form dimer **19**. The most popular strategies for undertaking such reactions utilize Kumada,^27^ Negishi,^28^ or Stille^29^ couplings. However, problems such as poor functional-group tolerance, monomer instability, and high reagent toxicity result in significant limitations, particularly for use in biological applications.^22^, ^30^, ^31^ As such, we chose to investigate the use of direct arylation, which has emerged in recent years as a powerful tool for constructing conjugated systems.^32^, ^33^ Pleasingly, **4** was found to be partially converted to **19** in the presence of 1.5 equiv of EDOT **1** in *N,N*-dimethylformamide (DMF) at 130°C for 1 hr (Scheme 2A; Figures S38 and S39). Importantly, the reaction was catalyzed by a readily available combination of Pd(OAc)_2_, pivalic acid, and potassium carbonate, thus negating the need for expensive or air-sensitive catalysts and ligands or the use of specialist techniques.^34^

Investigating the reaction further, we found yields to be increased significantly through the use of 4 equiv of EDOT, the excess of which could be readily re-isolated through column chromatography. At lower loadings, a significant amount of the symmetrical di-capped trimer **20** was produced as a result of further reaction of **19** with **4** (Figures S40 and S41). Although small amounts of this side product were still produced at higher EDOT loadings, yields were significantly lowered, and separation was readily achieved. Further iterations of bromination and direct arylation allowed the production of brominated dimer **21** and trimer **22** on a gram scale, both of which were found to be bench stable (Figures S42–S45). Bromination to form brominated trimer **23** was also possible, although its low solubility and stability prevented characterization and required its immediate use once prepared, as discussed later.

With these mono-capped building blocks in hand, we investigated the synthesis of di-capped oligomers (Scheme 2B). Heating a mixture of brominated and non-brominated monomers **4** and **3** (1.1 equiv) under the same conditions required for chain extension cleanly produced di-capped dimer **24** (Figures S46 and S47). Similarly, trimer **20** was produced from **4** and dimer **19**. Alternatively, **20** could be produced from the reaction of 2 equiv of either monomer **3** or brominated monomer **4** with 2,5-dibromo-EDOT **25** or EDOT **1**, respectively, in an optimized version of the previously discussed chain-extension side reaction.

By suitable choice of starting materials, di-capped oligomers (n = 2–5; **24**, **20**, **26**, and **27**) were all readily produced and easily isolated by column chromatography (Figures S48–S50). Extending the scope further to the use of brominated trimer **23**, used immediately without purification, allowed the synthesis of hexamer **28**, whereas coupling of trimer **22** with 2,5-dibromo-EDOT **25** allowed the synthesis of heptamer **29**, the first time the synthesis of EDOT oligomers of such lengths has been reported (Figures S51 and S52). Oligomers up to n = 6 were found to be bench and air stable and therefore could be easily handled, purified, and analyzed; no change in structure was observed by UV-Vis or ^1^H-NMR spectroscopy after 2 months of storage at room temperature. Heptamer **29** was produced with reduced purity (∼80% as judged by ^1^H NMR) but retained stability. Although oligomers of n = 2–5 were also found to be stable in solution, after long periods in chlorinated solvents (>2 weeks), a broadened UV-Vis absorption indicated that hexamer **28** and heptamer **29** had undergone partial degradation.

Oligomer solubility was found to decrease with increasing chain length, and aggregation in solution became significant at longer lengths. However, it remained high enough to allow manipulation in solution and the use of typical synthetic techniques such as phase extraction and column chromatography. Oligomers of n = 2–5 were soluble at concentrations of >20 mM in dichloromethane (DCM), and hexamer **28** was soluble at concentrations of >5 mM, whereas heptamer **29** could be solubilized at concentrations up to 0.5 mM. It is important to note that solubility is strongly influenced by the choice of end group and can be readily improved by the introduction of a flexible solubilizing linker to the functional group of interest, as discussed later. Finally, we analyzed oligomers **20** and **26** by inductively coupled plasma mass spectrometry (ICP-MS) to determine the levels of residual palladium present. As for other heavy metals, palladium contamination in pharmaceuticals and biomedical devices is tightly regulated because of the potential for toxic side effects. Palladium contamination was found to be at a low level of 7.4 ± 0.5 ppm for trimer **20** and 1.2 ± 0.5 ppm for tetramer **26**. Although it is difficult to make a direct comparison between a substrate intended for applications in tissue engineering and an active pharmaceutical ingredient (API), it is useful to note that these low levels of contamination are below the 10 ppm limit set by the International Council on Harmonisation of Technical Requirements for Registration of Pharmaceuticals for Human Use and the US Pharmacopeia for acceptable levels of palladium in APIs.^35^ Furthermore, because no extensive effort was taken to remove palladium from the samples, it is likely that these levels could be reduced further. For example, the use of palladium chelators during purification or the use of heterogeneous catalysts would be expected to lead to a significant reduction in contamination in any structures intended for biological applications.^36^, ^37^, ^38^

Although the ability to create symmetrical oligo-EDOTs with non-functional end groups is a useful tool for modeling the properties of PEDOT, the true utility of the method described above for the synthesis of di-piperidine-capped oligomers is in the synthesis of hetero-bifunctional constructs, which can be selectively derivatized at both ends, allowing their integration into more complex architectures. To demonstrate this, we first synthesized a series of unsymmetrical oligomers capped with a piperidine motif at one terminus and diisopropylamine at the other (see Scheme S1). Coupling differently terminated oligomers as described above produced oligomers of n = 2–5 (**30**–**33**) in a limited number of steps (Figures S53–S59 and S93–S98).

During these experiments, a number of observations were made. Firstly, although a temperature of 130°C was required for the chain extension and oligomer synthesis with brominated piperidine-based species, for diisopropyl-functionalized oligomers, 90°C was found to be sufficient to give complete conversion within 1 hr of reaction, leading to cleaner reaction products. Indeed, for all other end-capping groups investigated during this work, 90°C was high enough to facilitate reaction.^39^ Secondly, although couplings generally proceeded cleanly, the amount of side products produced increased with increasing oligomer length. The major side product was found to stem from the instability of the brominated species, resulting in partial dehalogenation and subsequent homo-coupling and, to a lesser extent, homo-coupling of the non-brominated reaction partner. Such side reactions have been studied extensively^40^ and are also known to occur during Stille and Suzuki polymerizations.^41^ Although outside the scope of this work (which focuses on the use of unoptimized, simple, and cheap catalyst systems), it is likely that such products could be minimized through judicious choice of both metal and ligand.^42^

To create functional oligomers primed for further reaction and derivatization, we considered that a number of common reactive handles would not be amenable to the chain extension and bromination procedures described above.^43^ It would therefore be advantageous to be able to install functionality at a late stage after oligomer synthesis. Thus, we investigated the use of orthogonal ester-protecting groups to provide latent functionality. Initial attempts to react methyl ester **7** with an excess of EDOT **1** led not only to chain extension but also to a significant amount (∼40%) of ester cleavage (see Scheme S2A). However, switching to *iso*-propyl ester **8** lead to a clean conversion to dimer **34** at 90°C, followed by subsequent bromination and extension to yield trimer **35** (reaction at 130°C as described for piperidine oligomers led to complete ester cleavage; see Scheme S2B; Figures S60–S63, S101, and S102). Similarly, the orthogonally protected *tert*-butyl ester **9** could undergo iterative chain extension and bromination to yield brominated dimer **36** (Figures S64, S65, and S111–S114).

**Scheme 3:**
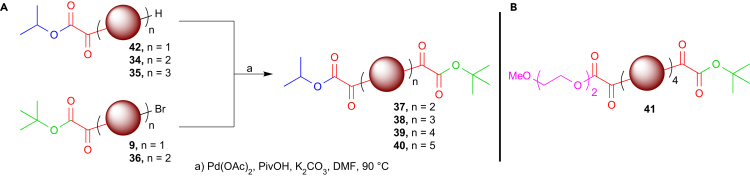
Synthesis of Orthogonal-Ester-Functionalized Hetero-bifunctional Oligomers (A) Synthesis of unsymmetrical, orthogonally protected oligo-EDOT diesters **37**–**40** with *iso*-propyl and *tert*-butyl end groups. (B) Triethylene glycol ester-capped tetra-EDOT **41** with improved solubility.

With these substrates in hand, we were able to synthesize di-capped, orthogonally protected oligomers **37**–**40** with n = 2–5 in a short number of steps and in good yields (Scheme 3A; see Scheme S2C for full details and Figures S66–S71). Although the synthesis of tetramer **39** and pentamer **40** was confirmed by mass spectrometry, the propensity of the constructs to aggregate in solution prevented analysis by ^13^C NMR. As an alternative, constructs possessing a solubility-enhancing triethylene glycol chain could also be produced as discussed above (**41**; Scheme 3B; Figures S72 and S103–S110). Here, the significant difference in end-group polarity greatly aided purification, offering a potential means of enhancing purity during particularly difficult separations. This representative example demonstrates an important advantage of the synthesis reported in this work; because the choice of end group is an important determinant in the material properties of the synthesized constructs, simply choosing an appropriate end cap can alter factors such as the solubility of the material to reflect the desired application.

Amide coupling after sequential ester deprotection, first in the presence of trifluoroacetic acid to remove the *tert*-butyl group and then in the presence of sodium hydroxide to cleave the *iso*-propyl ester, allowed the subsequent synthesis of unsymmetrical constructs bearing reactive functionality for further modification (see Scheme S3; Figures S115 and S116). As a result of the mild amide- or ester-forming conditions required, this method is applicable to the late-stage hetero-functionalization of the oligomers reported with a wide range of reactive or functional groups, such as those shown in Scheme 1. The potential applications of this methodology are diverse. The ability to create hetero-bifunctional oligomers of a tunable length and bearing handles for further modification allows the modular synthesis of more complex structures. For example, the integration of such constructs into biologically active scaffolds^2^ or the production of amphiphilic, self-assembling morphologies^44^, ^45^ offers exciting possibilities in the fields of both the material and biomedical sciences.

**Scheme 4:**
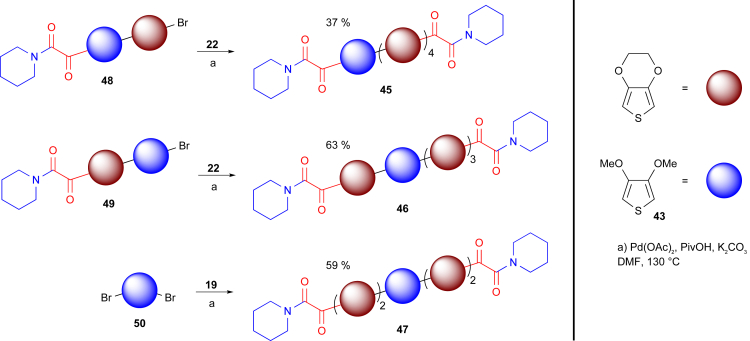
Synthesis of Dimethoxythiophene-Containing Isomers Pentameric EDOT oligomers containing a single DMT unit were synthesized for the generation of the structural isomers **45**, **46**, and **47**.

Finally, we wished to investigate the application of our methodology to the synthesis of mixed oligomers composed of different monomer units, which could possess interesting properties. In particular, we considered the rigidity of EDOT oligomers, which are known to lead to highly planar structures with enhanced π conjugation.^23^ We reasoned that disrupting planarity in a controlled fashion could tune the properties of the resultant material. Structurally related dialkoxythiophene monomers such as 3,4-dimethoxythiophene (DMT, **43**) and 3,4-propylenedioxythiophene (ProDOT, **44**) were found to be suitable substrates for our glyoxylation and chain-extension procedures. We therefore introduced a single DMT moiety in an EDOT-pentameric structure to create three structural isomers: **45**, **46**, and **47** (Scheme 4; see Scheme S4 for full details and Figures S75–S83 and S117–S124). The simple manner in which such compounds can be created allows the rapid construction of a library of dialkoxythiophene-based constructs for investigating the effects of structure, substituents, and isomerization on the chemical and electrical properties of CPs.

### Oligomer Characterization

**Figure 2:**
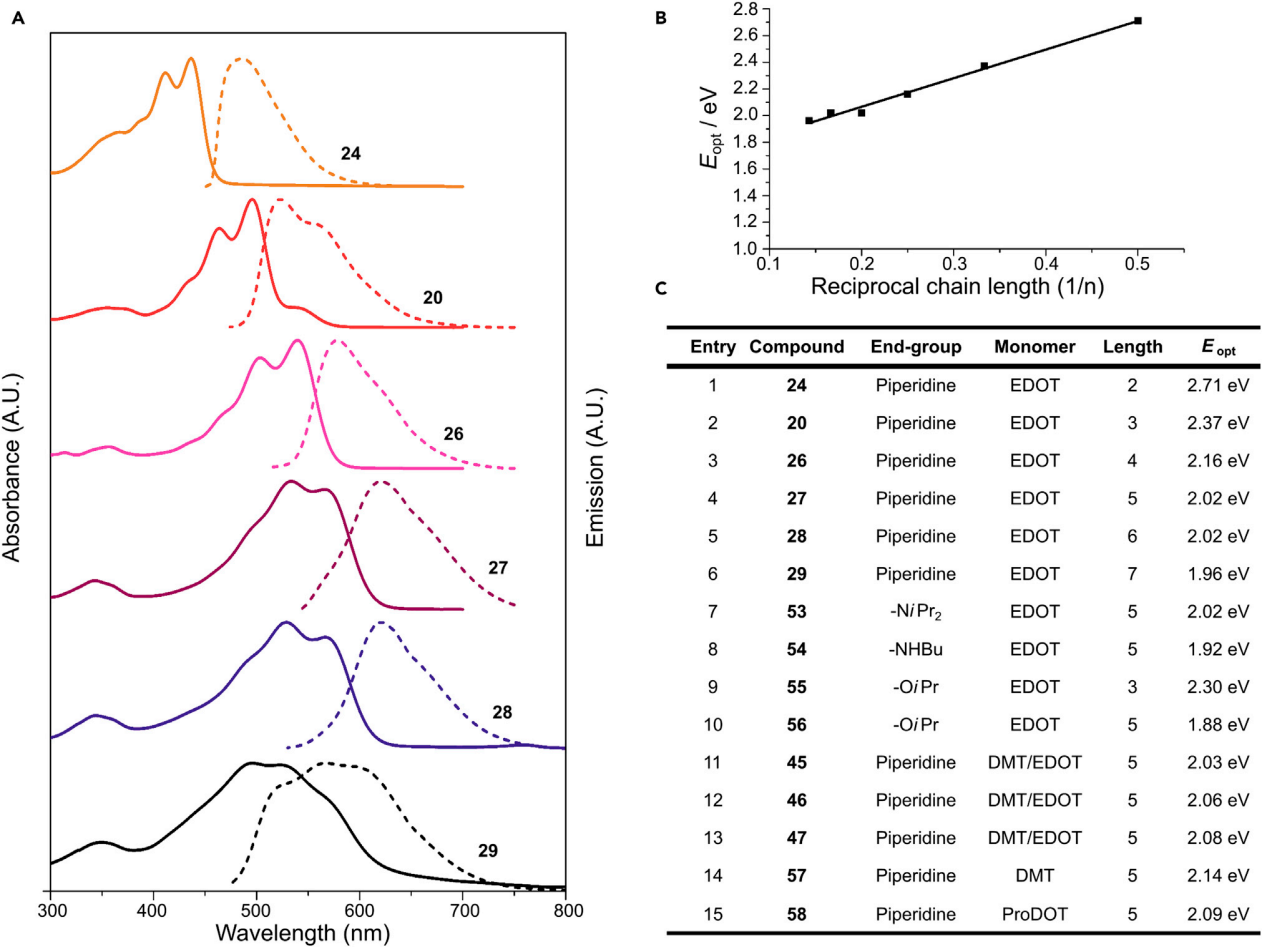
Oligomer Optical Characterization (A) Normalized UV-Vis (solid line) and fluorescence (dashed line) spectra of di-piperidine-capped oligomers **24**, **20**, and **26**–**29**. (B) Correlation of inverse chain length and *E*_opt_ for oligomers **24**, **20**, and **26**–**29** (adjusted R^2^ = 0.9828). (C) Summary of *E*_opt_ for a series of di-functionalized oligomers.

Solutions of the di-piperidine-capped oligomers described above (**24**, **20**, and **26**–**29**) in DCM were analyzed by UV-Vis and fluorescence spectroscopy. Within the range investigated, the optical properties of the materials were found to be independent of concentration, indicating that aggregation was not occurring. As expected, a gradual red shift in the onset of absorbance was observed with increasing chain length (Figure 2), although a blue shift in absorbance maxima for heptamer **29** was observed, most likely because of the presence of impurities in the sample. Furthermore, the spectra possessed well-defined vibronic structures, a widely reported feature of EDOT oligomers not shared by unsubstituted thiophene structures.^21^, ^23^, ^46^ When compared with the parent C–H capped oligomers biEDOT **51** and terEDOT **52**, mono-piperidine-capped dimer **19** and trimer **22** displayed a large red shift in absorbance (see Figure S1). This effect was even more pronounced for the di-capped oligomers **24** and **20**. A red shift in absorbance of >100 nm indicated that conjugation of the thiophene core with the keto-acid end group, to create an acceptor-donor-acceptor triad, played a major role in influencing the properties of the synthesized oligomers, leading to a significant narrowing of the optical gap (*E*_opt_).^47^, ^48^

When compared with those of previously reported EDOT end-capped oligomers, the absorption spectra were strongly red shifted in relation to the corresponding mesityl, phenyl, hexyl, and trimethylsilyl structures highlighted in Figure 1.^21^, ^22^, ^23^, ^24^ The remarkably low-energy *E*_opt_ of the structures reported here is considered to be a consequence of the lowering in energy of the lowest unoccupied molecular orbital (LUMO) as a result of the electron-withdrawing nature of the keto-acid moiety, as discussed later. Oligomer capping with primary amines to yield secondary amides was found to result in a further lowering of *E*_opt_ (Figure 2C, entry 8; Figures S2 and S151–S158). This effect was enhanced through capping with more electron-poor ester groups, resulting in an *E*_opt_ as low as 1.88 eV for the *iso*-propyl ester di-capped pentamer **56** (Figure 2C, entry 10; Figures S84–S86 and S159–S161).

The constrained six-membered ring of EDOT is known to result in favorable attractive intramolecular S–O interactions between repeating units.^23^, ^49^ This effect is reduced upon the introduction of the more structurally flexible methoxy units of DMT. Therefore, as predicted, the introduction of a single DMT residue into an EDOT pentamer led to an increase in *E*_opt_ as a result of disruption of the highly planar EDOT-repeating structure. This effect was found to be position dependent such that the length of the longest continuous EDOT chain determined the degree of disruption. When compared with the pentaEDOT oligomer **27**, DMT-containing isomer **45** (four continuous residues) exhibited a Δ*E*_opt_ = +0.013 eV, whereas isomer **47** (two continuous residues) possessed an increased Δ*E*_opt_ = +0.057 eV (Figure 2C, entries 11–13). This widening of the optical gap was further enhanced in an oligomer consisting of end-capped penta-DMT **57** (Δ*E*_opt_ = +0.122 eV) or the analogous penta-ProDOT oligomer **58** (Δ*E*_opt_ = +0.44 eV) (Figure 2C, entries 14 and 15; Figures S87, S88, and S164–S173). These results support our hypothesis that the oligomer properties can be tuned through the suitable choice and positioning of alternative monomer units.

**Figure 3:**
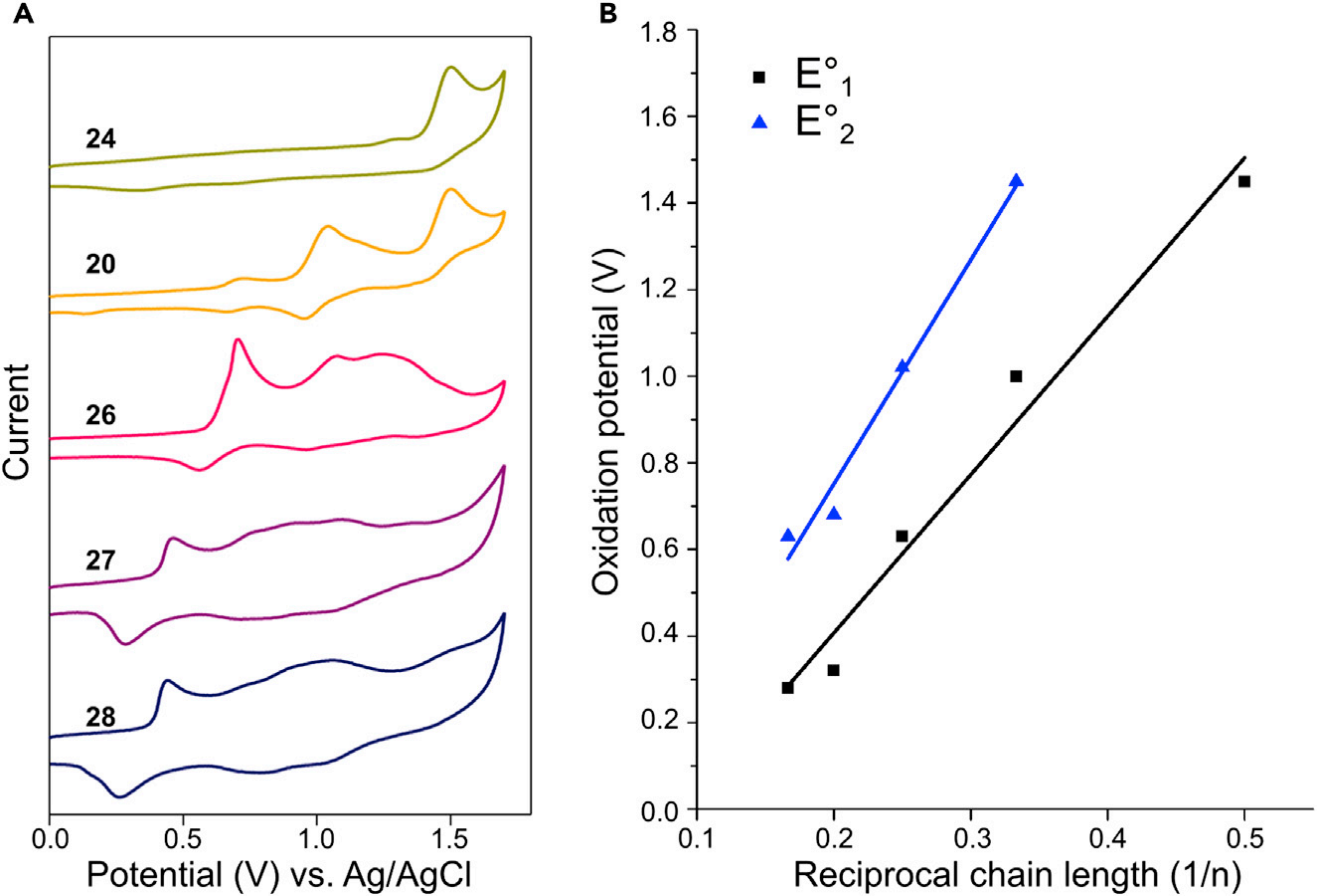
Cyclic Voltammetry Characterization (A) Cyclic voltammograms of piperidine-capped oligomers **24**, **20**, and **26**–**28**. CVs were recorded at a scan rate of 100 mV s^−1^ with oligomer concentrations of 1 mM in DCM containing 0.1 M Bu_4_NPF_6_. (B) Correlation of inverse chain length and first and second oxidation potentials for oligomers **24**, **20**, and **26**–**28** (adjusted R^2^ = 0.9680 and 0.9725, respectively).

Next, we investigated the solution electrochemical properties of selected oligomers by cyclic voltammetry. Di-piperidine-capped oligomers **24**, **20**, and **26**–**28** (n = 2–6) were all investigated. However, because of the low solubility of EDOT-heptamer **29** and its reduced purity, weak signal intensity was observed during measurements, and therefore this structure was not further investigated. Cyclic voltammograms (CVs) demonstrated a decrease in the first oxidation potential with increasing chain length, supporting the results obtained by UV-Vis spectroscopy (Figure 3A). Linear correlations were found between the first and second oxidation potentials and the inverse chain length (Figure 3B; see Table S1). The oxidation of oligomers **24**, **20**, and **26** (n = 2–4) was electrochemically quasi-reversible, whereas pentamer **27** and hexamer **28** displayed improved electrochemical reversibility (Figure 3). Furthermore, CVs of penta-DMT **57** and penta-ProDOT **58** allowed comparison with penta-EDOT **27** (see Figure S3). As was seen for the optical gap, the first oxidation potential was found to follow the trend EDOT < ProDOT < DMT. These results further support the higher effective conjugation of EDOT oligomers and a degree of planarity disruption induced by the high torsional strain of DMT-based structures.^50^ The ease with which the oxidation potentials can be tuned, through both alteration of oligomer length and monomer composition, offers intriguing possibilities for applications not only in tissue engineering but also in creating sensitive and selective organic bioelectronics.^51^, ^52^

**Figure 4:**
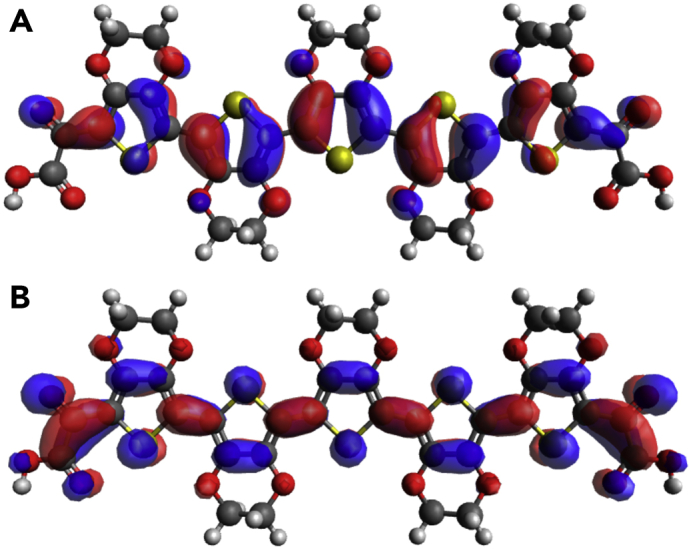
DFT Orbital Projections (A) HOMO orbital distribution of carboxy-capped pentamer **59**. (B) LUMO orbital distribution.

Finally, we undertook computational density functional theory (DFT) calculations to further probe the influence of the keto-acid end groups on oligomer properties.^53^ The trends observed in the calculated HOMO-LUMO gaps during these studies reproduced the structural and length dependencies observed during experimental measurements. Initial calculations on carboxy-terminated EDOT pentamer **59** validated our hypothesis that the keto-acid end group played an important role in extending π conjugation (Figure 4). This was particularly true for the LUMO—the electron-withdrawing nature of the end group led to a large orbital localization across the ketone group. Partial distribution of the LUMO across the terminal carboxyl indicated that the choice of an ester or amide linkage might influence the electrical properties of oligomeric constructs. Thus, compared with an analogous amide substrate, the presence of a more electron-deficient ester group would be expected to lower the LUMO level, leading to a decreased HOMO-LUMO gap (see Figure S4). This supports our experimental observation of a lower *E*_opt_ for *iso*-propyl ester di-capped oligomers than for amide-capped structures.

DFT also provided rationale for the increase in *E*_opt_ observed for tertiary-amide-capped structures. To accommodate the steric bulk of both the piperidine and diisopropylamine substituents, the dicarbonyl groups were found to be significantly disrupted from the antiperiplanar orientation observed for other substituents. This led to dihedral angles of as little as 131° for diisopropyl-capped dimer **60** and 142° for piperidine-capped dimer **24** (see Figures S5, S89, S90, S162, and S163). As a result, conjugation was partially disrupted, leading to an increase in the HOMO-LUMO gap, supporting the observed increase in *E*_opt_. Replacement of EDOT with DMT or ProDOT offered two different mechanisms by which disruption of the expected planar configuration could potentially occur. In the case of DMT, the high torsional strain of consecutive units was found to lead to a slight twisting of the backbone for longer oligomer structures, therefore decreasing effective conjugation. In contrast, calculations predicted a slight deflection of the alkoxy substituents in the ProDOT structure (174° and 180° dihedral angle in EDOT and DMT, respectively) to accommodate an expanded seven-membered ring. The resultant cumulative decrease in electron donation from these substituents might explain the slight increase in *E*_opt_ observed for the ProDOT derivatives described above.

### Conclusions

We have developed a glyoxylation end-capping strategy that allows the rapid installation of keto-amides and keto-esters at the end of oligomeric-EDOT chains. The resultant materials retain solubility and are bench stable, in contrast to previous reports of oligo-EDOT derivatives. These developments allow us to report the synthesis of hexa- and heptameric EDOT constructs for the first time. Furthermore, the use of iterative chain extension allows the construction of hetero-bifunctional constructs bearing orthogonally reactive handles for further modification. Characterization of the structures produced demonstrated the important role played by the keto-acid end group in determining oligomer properties. The remarkably low optical gap observed for the oligomeric structures was attributed to the important role played by the extended conjugated system, particularly in lowering the LUMO energy, as demonstrated by DFT calculations. Notably, through suitable choice of oligomer length, end group, and monomer composition, the optical, electronic, and physical properties of a construct can be readily tuned both across a wide range and with fine control. This ability to undertake a flexible and modular approach to structural design creates intriguing opportunities in the synthesis of novel materials. Work to explore the full possibilities of this powerful methodology is currently ongoing in our group for the integration of tunable conjugated materials into tissue engineering scaffolds.

## Experimental Procedures

### General Method for EDOT Glyoxylation

Oxalyl chloride (850 μL, 10 mmol) was added drop-wise to a solution of EDOT (1.05 mL, 10 mmol) in dioxane (30 mL). The mixture was heated to 100°C for 1 hr and then allowed to cool to room temperature. The requisite amine (15 mmol) and base (50 mmol) were then added, and the mixture was stirred for 3 hr. After this time, the mixture was diluted with DCM (150 mL) and washed with water (100 mL), and the organics were dried with MgSO_4_, filtered, and concentrated in vacuo. The residue was purified by flash column chromatography, and pure fractions were concentrated in vacuo.

### General Method for Monomer Bromination

EDOT derivative (5 mmol) was dissolved in a mixture of tetrahydrofuran (THF, 5 mL) and acetic acid (3 mL). If solubility was poor, a further 25 mL of THF was added. The mixture was placed in the dark, and *N*-bromosuccinimide (6 mmol) was added. After being stirred for 2 hr, the mixture was either precipitated in water (150 mL), causing precipitation of the product, which could be collected by filtration, or diluted with DCM (150 mL) and washed with saturated NaHCO_3_ (3 × 100 mL), dried with MgSO_4_, filtered, and concentrated in vacuo. Column chromatography was then undertaken if required, although the products were usually sufficiently pure for further use.

### General Method for Chain Extension

Brominated monomer (1 mmol), pivalic acid (0.5 mmol), palladium(II) acetate (0.05 mmol), and potassium carbonate (10 mmol) were charged under nitrogen. Dry DMF (2 mL) and EDOT (4 mmol) were then added, and the mixture was heated to 90°C for 2 hr. After cooling to room temperature, the mixture was diluted with DCM (50 mL) and washed with water (2 × 50 mL) and brine (50 mL). The organics were dried with MgSO_4_, filtered, and concentrated in vacuo. The residue was purified by flash column chromatography, and pure fractions were concentrated in vacuo.

### General Method for Oligomer Synthesis

Brominated oligomer (1 mmol), hydrogen-capped oligomer (1.2 mmol), pivalic acid (0.5 mmol), palladium(II) acetate (0.05 mmol), and potassium carbonate (10 mmol) were charged under nitrogen. Dry DMF (2 mL) was added, and the mixture was heated to 90°C for 2 hr. After cooling to room temperature, the mixture was diluted with DCM (50 mL) and washed with water (2 × 50 mL) and brine (50 mL). The organics were dried with MgSO_4_, filtered, and concentrated in vacuo. The residue was purified by flash column chromatography, and pure fractions were concentrated in vacuo.

## Author Contributions

C.D.S. performed all experiments and wrote the manuscript. M.A.B. performed CV measurements. A.A. performed UV-Vis measurements. C.D.S., M.A.B., A.A., and C.B.N. analyzed and interpreted data. C.D.S., D.M., and M.M.S. developed the ideas. All authors commented on the manuscript. M.M.S. supervised the project.

## Supplemental Information

Document S1. Supplemental Experimental Procedures, Figures S1–S173, Table S1, and Schemes S1–S4

Table S2. Compound Table

Document S2. Article plus Supplemental Information
