# Supplementary material for: Synthesis of Hetero-bifunctional, End-Capped Oligo-EDOT Derivatives
Source: Chem. 2017 Jan 12;2(1):125–38. doi: 10.1016/j.chempr.2016.12.003 (PMC5268340; doi:10.1016/j.chempr.2016.12.003)
Supplement: Document S1. Supplemental Experimental Procedures, Figures S1–S173, Table S1, and Schemes S1–S4 [file mmc1.pdf]

Chem, Volume *2*

Supplemental Information

Synthesis of Hetero-bifunctional, End-Capped Oligo-EDOT Derivatives

Christopher D. Spicer, Marsilea A. Booth, Damia Mawad, Astrid Armgarth, Christian B. Nielsen, and Molly M. Stevens

Table of contents

**S2**	Supplemental figures
**S5**	Supplemental schemes
**S7**	Supplemental table
**S7**	General considerations
**S9**	Cyclic voltammetry
**S9**	DFT calculations
**S10**	Amine synthesis
**S13**	Thiophene synthesis
**S16**	EDOT Functionalisation
**S21**	Monomer bromination
**S25**	Monomer manipulation
**S31**	Chain extension protocol
**S32**	Chain extension-dimers
**S36**	Dimer bromination
**S38**	Chain extension-trimers
**S40**	Trimer bromination
**S40**	Oligomer synthesis protocol
**S41**	Dimer synthesis
**S42**	Trimer synthesis
**S44**	Tetramer synthesis
**S46**	Pentamer synthesis
**S49**	Hexamer synthesis
**S49**	Heptamer synthesis
**S49**	Oligomer manipulation
**S53**	3,4-Dimethoxythiophene functionalisation
**S60**	3,4-Propylenedioxythiophene functionalisation
**S63**	References
**S64**	NMR spectra of novel compounds

S2

Figure S1. Normalised UV-Vis (solid) and fluorescence (dashed) spectra of piperidine-capped oligomers.

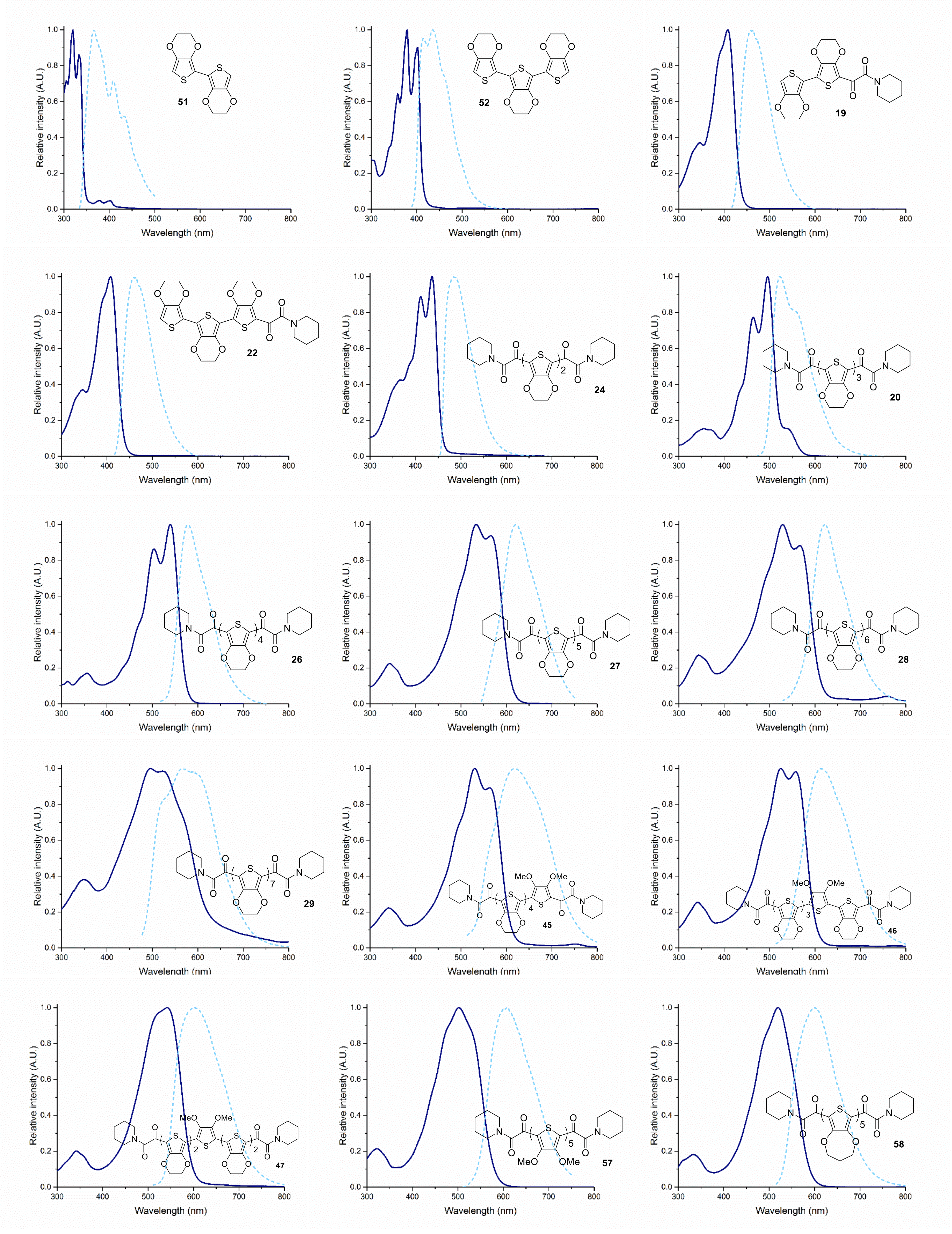

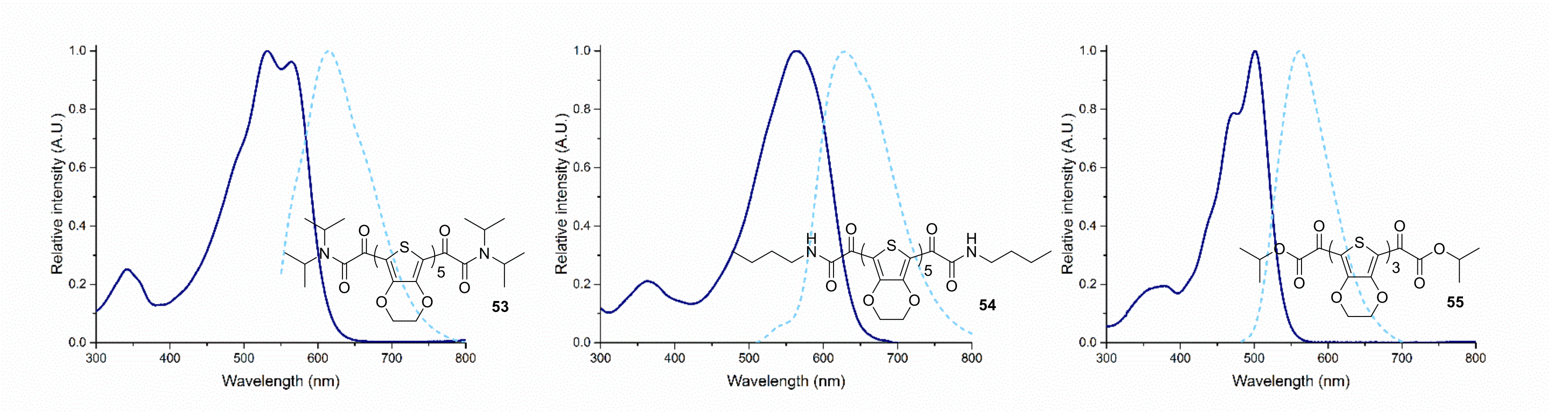

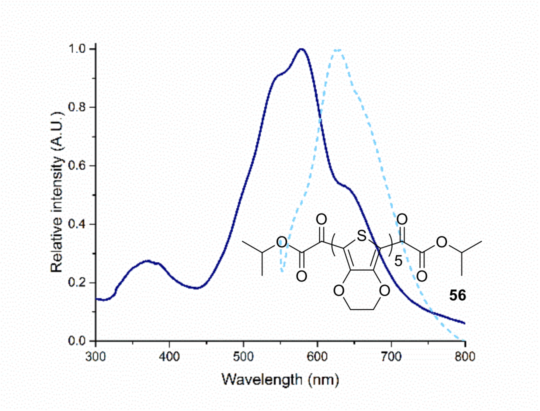

Figure S2. Normalised UV-Vis (solid) and fluorescence (dashed) spectra of diisopropylamine, *n*-butylamine ad *iso*-propanol capped oligomers.

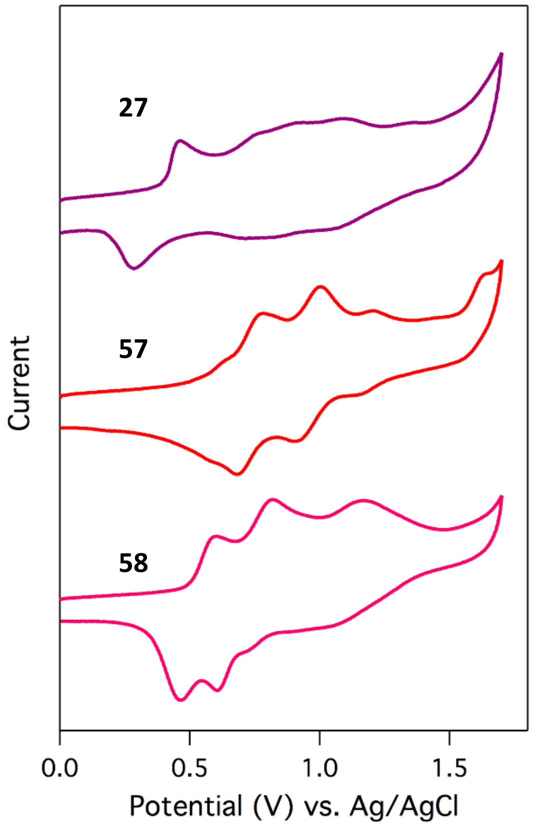

Figure S3. Cyclic voltammograms of EDOT (**27**), DMT (**57**) and ProDOT (**58**) pentamers. Voltamograms were recorded at a concentration of 1 mM in DCM containing 1 M Bu_4NPF_6.

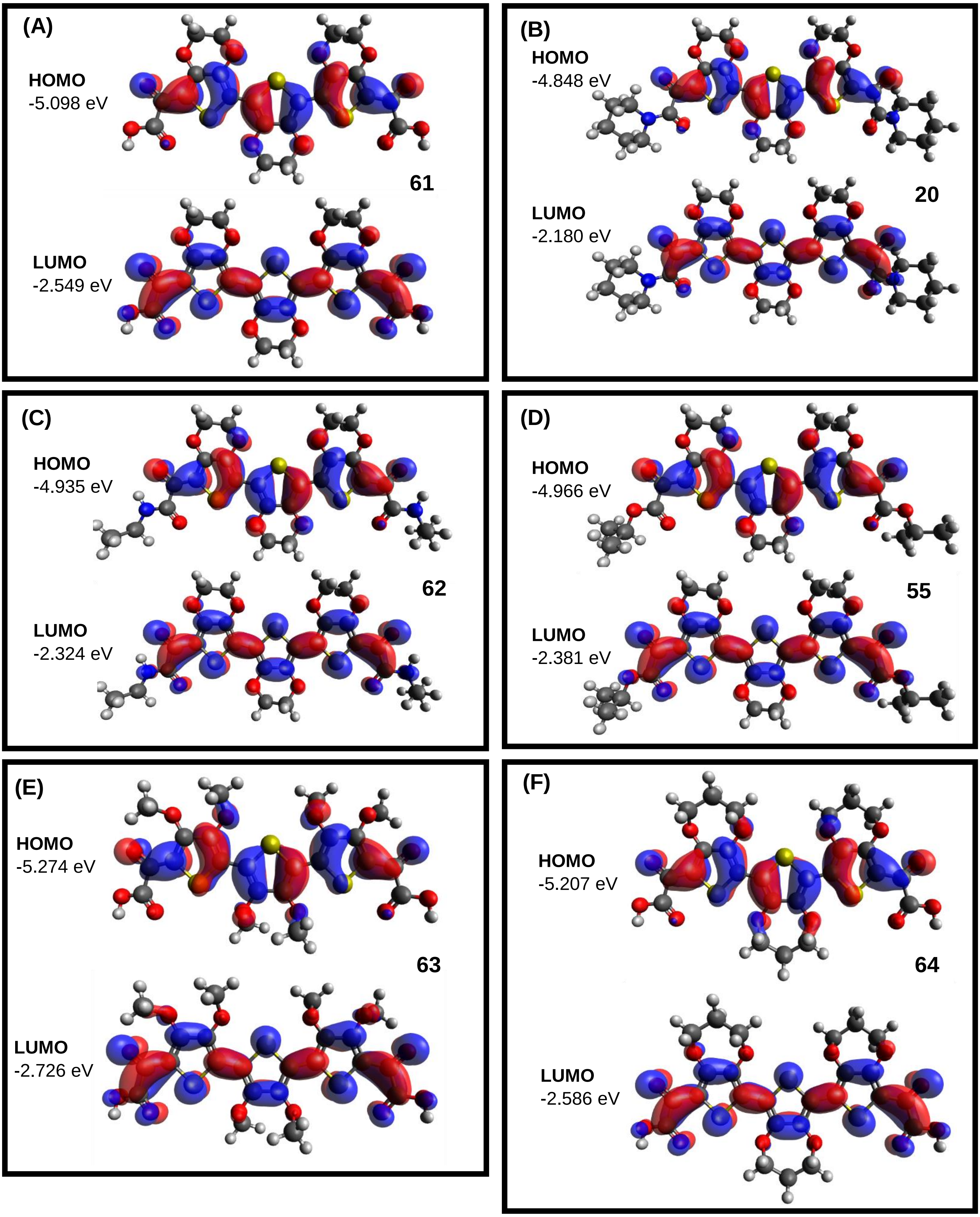

Figure S4. DFT HOMO/LUMO distributions and optimised geometries for model oligomers. Trimers were modelled in order to reduce computational time. (A) Carboxy-terminated EDOT trimer **61**. (B) Piperidine capped EDOT trimer **20**. (C) Ethylamine-capped EDOT trimer **62**. An ethyl group was modelled in place of the butyl group used experimentally to reduce computational time. (D) *Iso*-propyl ester-capped EDOT trimer **55**. (E) Carboxy-terminated DMT trimer **63**. (F) Carboxy-terminated ProDOT trimer **64.**

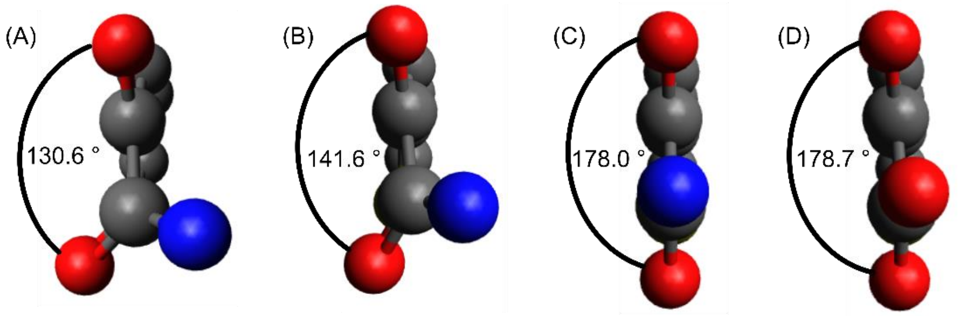

Figure S5. DFT optimised geometries demonstrating the dicarbonyl dihedral angle. Amide/ester substituents, dialkoxy-rings and further EDOT residues have been removed for visual clarity. (A) Diisopropylamine capped dimer **60**. (B) Piperidine capped dimer **24**. (C) Ethylamine capped trimer **62**. (D) *Iso*-propyl ester capped trimer **38**.

Supplemental schemes

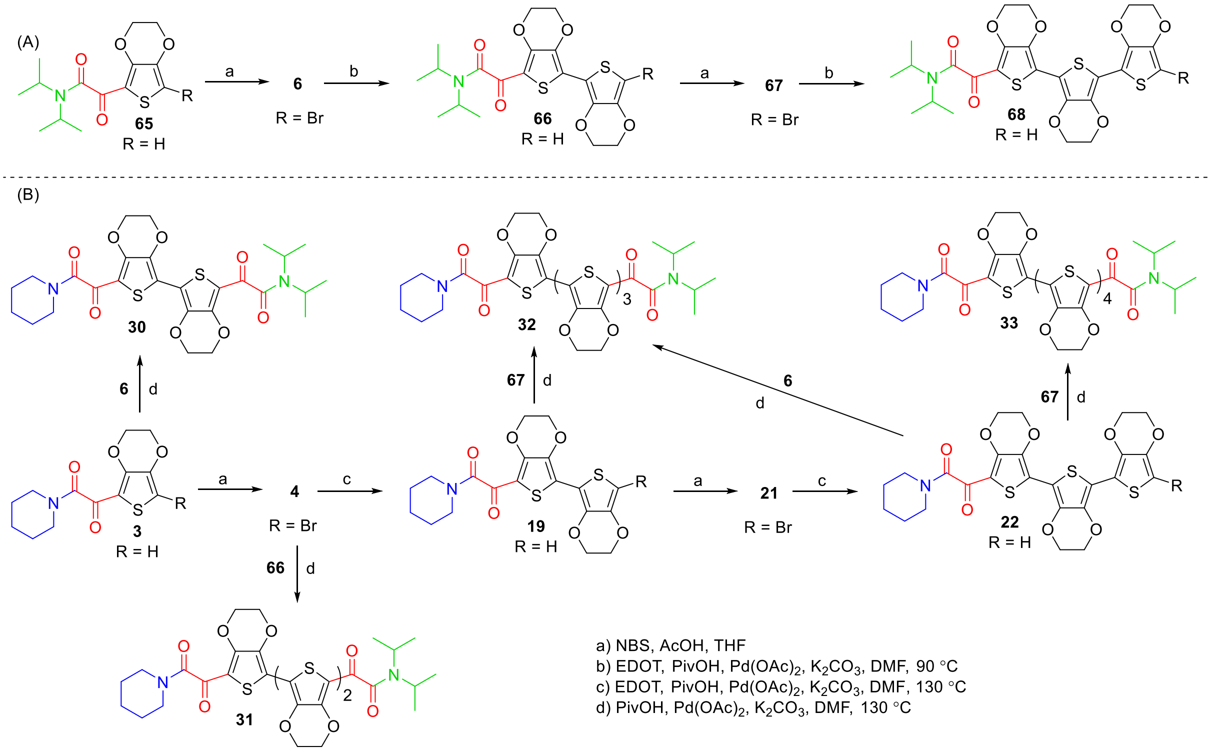

Scheme S1. (A) Synthesis of mono-capped diisopropylamine-EDOT oligomers; (B) Synthesis of unsymmetrical, bifunctional piperidine-diisopropylamine EDOT oligomers.

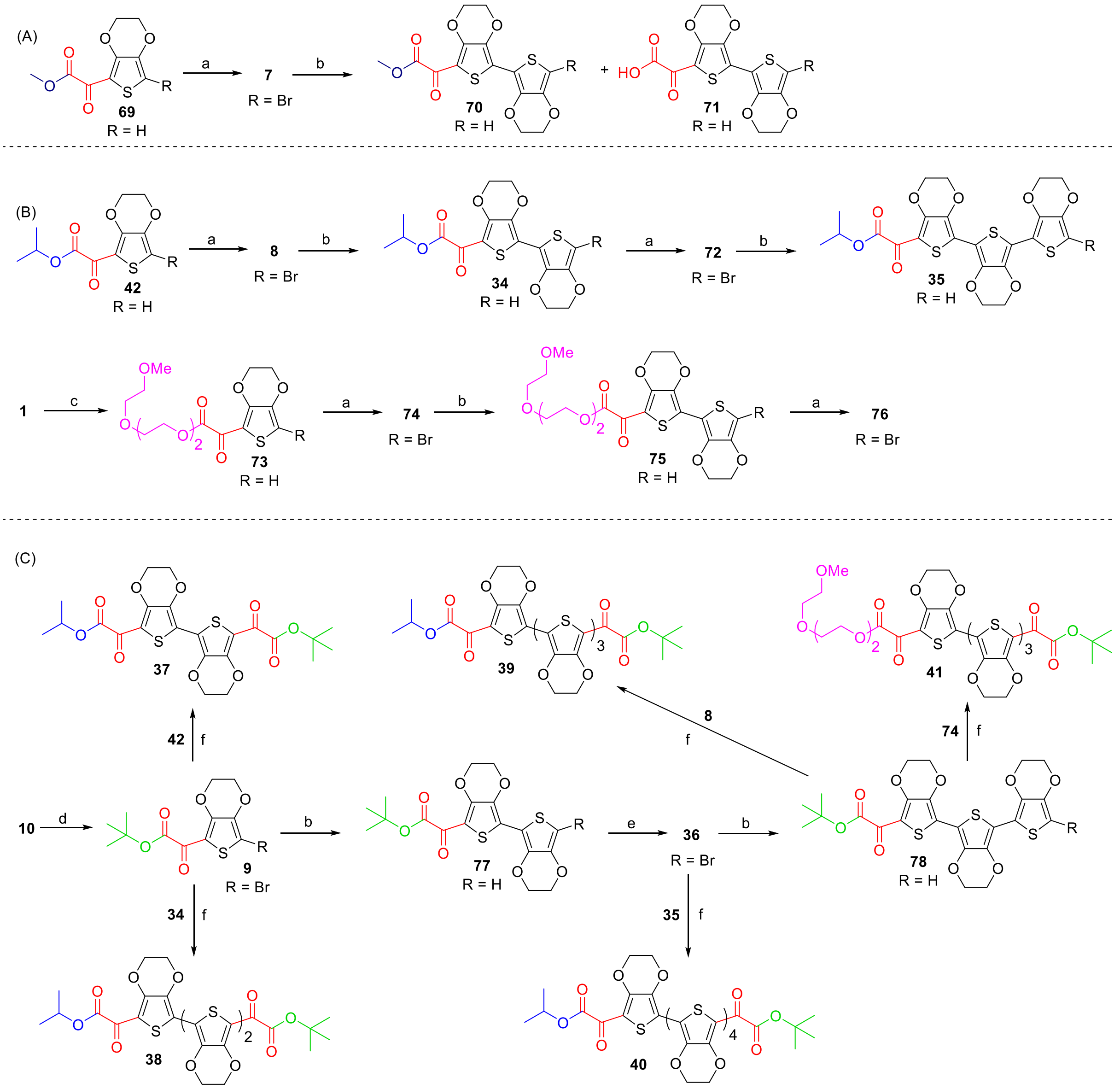

a) NBS, AcOH, THF; b) EDOT, PivOH, $Pd(OAc)_2$, K_2CO_3, DMF, 90 °C; c) i) $(COCl)_2$, dioxane, ii) HO-TEG-OMe, NEt_3; d) tBuOH, DCC, DMAP, DCM; e) NBS, DMF; f) PivOH, $Pd(OAc)_2$, K_2CO_3, DMF, 90 °C;

Scheme S2. a) Partial methyl ester cleavage under chain extension conditions; b) Synthesis of mono-capped ester-oligomers; c) Synthesis of hetero-bifunctional di-ester oligomers.

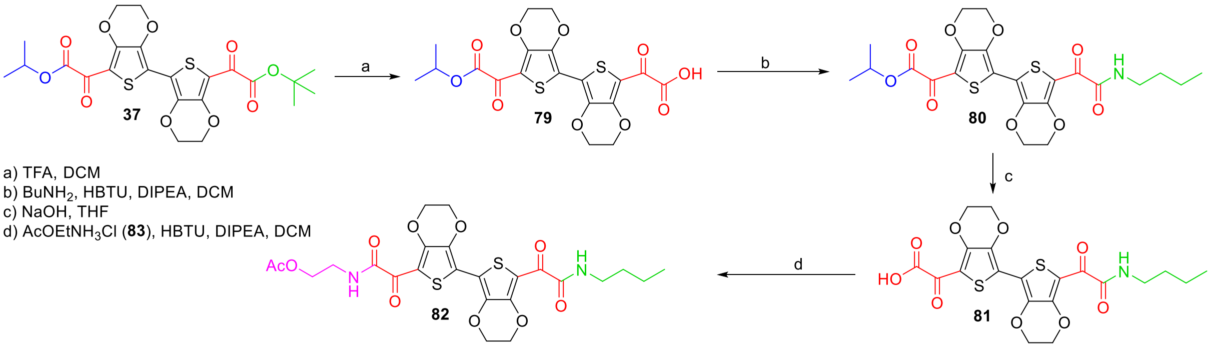

Scheme S3. Orthogonal ester deprotection and sequential amide coupling to generate hetero-bifunctional oligomer bearing reactive groups for further modification.

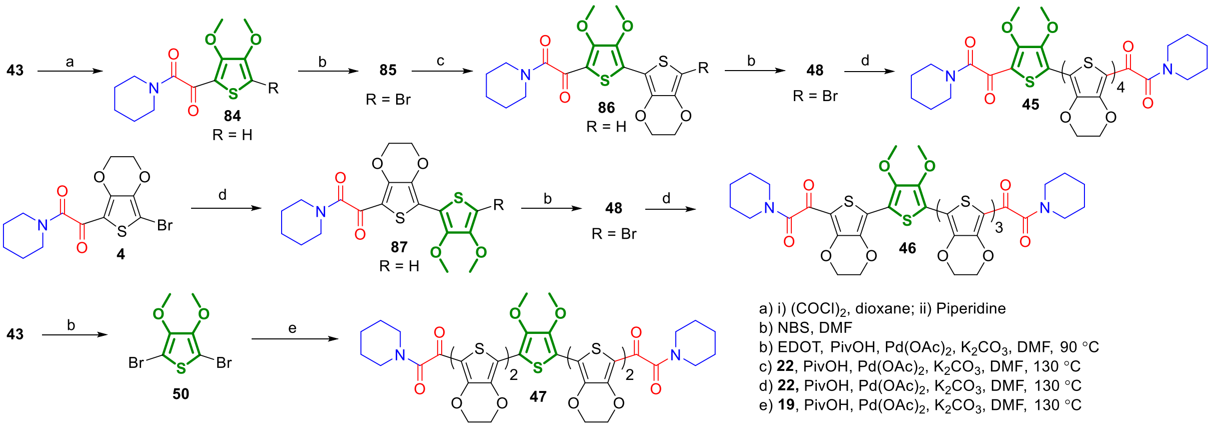

Scheme S4. Synthesis of dimethoxythiophene substituted oligomers.

Entry	Compound	Length	Monomer	$E°_1$ (V)	$E°_2$ (V)
1	**24**	2	EDOT	1.45	
2	**20**	3	EDOT	1.00	1.45
3	**26**	4	EDOT	0.63	1.02
4	**27**	5	EDOT	0.32	0.68
5	**28**	6	EDOT	0.28	0.63
6	**57**	5	DMT	0.69	0.95
7	**59**	5	ProDOT	0.48	0.76

Table S1. Summary of oligomer first and second oxidation potentials, calculated by cyclic voltammetry. Potentials are corrected by the internal standard Fc/Fc$^+$ and are vs. Ag/AgCl.

General Considerations

Proton nuclear magnetic resonance (^{1}H NMR) spectra were recorded on a Bruker AV-400 (400 MHz) spectrometer. Carbon nuclear magnetic resonance (^{13}C NMR) spectra were recorded on a Bruker AV-400 (100 MHz) spectrometer. NMR shifts were assigned using COSY, HSQC and HMBC spectra. All

chemical shifts are quoted on the δ scale in ppm using residual solvent as the internal standard (^{1}H NMR: $CDCl_3$ = 7.26; MeOD = 3.31 DMSO-d_6 = 2.50 and ^{13}C NMR: $CDCl_3$ = 77.16, MeOD = 49.00, DMSO-d_6 = 39.52). Coupling constants (*J*) are reported in Hz with the following splitting abbreviations: s = singlet, d = doublet, t = triplet, q = quartet, sept = septet, m = multiplet, app = apparent, br = broad. Melting points (m.p.) were recorded on a Zeiss Axio Imager: Z1M microscope equipped with a Linkam LTS 420 temperature controlled microscope stage and are uncorrected. Infrared (IR) spectra were recorded on a Perkin Elmer Spectrum 100 spectrophotometer with a Universal ATR Sampling Accessory. Absorption maxima ($υ_{max}$) are reported in wavenumbers (cm^{-1}). UV-Vis spectra were recorded on a Perkin Elmer Lambda 25 spectrophotometer in a quartz cuvette with a pathlength of 1 cm. All measurements were undertaken in DCM at a concentration sufficient to give an absorbance reading of 0.5-1 Au. Fluorescence spectra were recorded on a Horiba Fluorolog fluorimeter in a quartz fluorescence cuvette. All measurements were undertaken in DCM at a concentration sufficient to give a relative emission intensity of 10^6-$2.5x10^7$ C.P.S. Low resolution mass spectra (LRMS) were recorded on an Agilent 6130 Quadrupole mass spectrometer using electrospray ionization (ESI), connected to an Agilent 1260 Infinity liquid chromatography set-up with a Phenomenex Gemini-NX-C16 column. High resolution mass spectra (HRMS) were recorded on a Waters LCT Premier (ES-ToF) spectrometer connected to an Aquity-iClass UPLC. Matrix-assisted laser desorption-ionization (MALDI) spectra were recorded on a Micromass MALDI-ToF spectrometer. Nominal and exact m/z values are reported in Daltons. ICP-MS was performed on a Varian 820-MS ICP-mass spectrometer. Samples were digested in 69 % AnalaR nitric acid (Sigma-Aldrich) and then diluted to 0.1 % analyte in 2 % nitric acid. Analysis was undertaken using the isotopes Pd-105, Pd-106, and Pd-108, using a 10 ppb palladium standard as a calibrant. Thin layer chromatography (TLC) was carried out using aluminium backed sheets coated with 60 F_{254} silica gel (Merck). Visualization of the silica plates was achieved using a UV lamp ($λ_{max}$ = 254, 302, or 366 nm), and/or ammonium molybdate (5 % in 2M H_2SO_4), and/or potassium permanganate (5 % $KMnO_4$ in 1M NaOH with 5 % potassium carbonate). Flash column chromatography was carried out using Geduran Si 60 (40-63 μm) (Merck). Mobile phases are reported as % volume of more polar solvent in less polar solvent. Anhydrous solvents were purchased from Sigma-Aldrich and used as supplied. All other solvents were used as supplied (Analytical or HPLC grade), without prior purification. Reagents were purchased from Sigma-Aldrich and used as supplied, unless otherwise indicated. 3,4-Ethylenedioxythiophene and palladium (II) acetate were purchased from Alfa Aesar. 3,4-

Dibromothiophene was purchased from Apollo scientific. Brine refers to a saturated solution of sodium chloride. Anhydrous magnesium sulfate ($MgSO_4$) was used as the drying agent after reaction workup unless otherwise stated.

Cyclic voltammetry

Cyclic voltammetry studies were performed using in-house potentiostats and a PowerLab 8/35, controlled by EChem (eDAQ). A three-electrode system was employed with a glassy carbon working electrode, and Ag/AgCl reference electrode (3M, NaCl, +0.197 V vs. SHE) and a stainless steel counter electrode. Ferrocene was added as an internal standard. Measurements were undertaken on 1 mM solutions of oligomers in DCM, containing 0.1 M Bu_4NPF_6 as an electrolyte. The spectra given in Figure 3 and supplemental Figure 2 are background subtracted. Oxidation potentials were calculated using the equation $E°_x = (E_{p,a} - E_{p,c})/2$ where $E_{p,a}$ is the anodic potential and $E_{p,c}$ is the cathodic potential.

DFT calculations

Molecules were built using the Avogadro software and geometries were manipulated to provide the lowest free energy configuration using a UFF forcefield. Single point energy and geometrical optimisation calculations were then undertaken utilising the Gaussian 9.0 software (B3LYP/6-31G*) in order to estimate the HOMO and LUMO orbital distributions. Alkyl end-groups were minimised in order to reduce computational complexity in a number of calculations.

Amine Synthesis

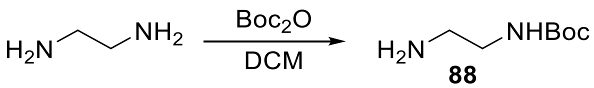

88

A solution of di-*tert*-butyl dicarbonate (3.27 g, 15 mmol) in DCM (200 mL) was added dropwise to a solution of ethylenediamine (6.01 mL, 90 mmol) in DCM (50 mL) over a period of 5 hrs. After stirring for a further 12 hrs the mixture was washed with K_2CO_3 (2 M, 2 x 200 mL), dried with $MgSO_4$, filtered and concentrated *in vacuo* to give the DP as a colourless oil. A yield of 1.74 g, 10.8 mmol (72 %) was obtained. Spectroscopic data were consistent with those previously reported.[1] ^1H NMR (400 MHz, $CDCl_3$): δ = 5.04 (1H, br s, -N<u>H</u>), 3.01-3.19 (2H, m, -C<u>H</u>$_2$NHBoc), 2.78 (2H, t, J = 5.9 Hz, -C<u>H</u>$_2$NH$_2$), 1.44 (9H, s, <u>Boc</u>) ppm;

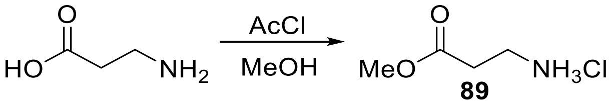

89

Acetyl chloride (14 mL, 200 mmol) was added dropwise to methanol (250 mL) at 0 ºC over a period of 15 min. After stirring for a further 15 min, β-alanine (1.78 g, 20 mmol) was added and the mixture stirred for 18 hrs. The solvent was then removed *in vacuo* and the residue triturated for 15 min in diethyl ether (2 x 100 mL) and DCM (100 mL), filtered and dried *in vacuo*. The DP was obtained as a white solid. A yield of 2.58 g, 18.6 mmol (93 %) was obtained. Spectroscopic data were consistent with those previously reported.[2] ^{1}H NMR (400 MHz, MeOD): δ = 3.76 (3H, s, -O<u>Me</u>), 3.23 (2H, t, *J* = 6.5 Hz, <u>H</u>$_\beta$), 2.78 (2H, t, *J* = 6.5 Hz, <u>H</u>$_\alpha$) ppm;

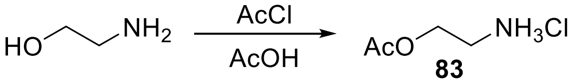

83

Acetyl chloride (5.7 mL, 80 mmol) was added to a solution of ethanolamine (2.4 mL, 40 mmol) in acetic acid (20 mL). After stirring for 18 hrs the mixture was poured into diethyl ether (200 mL). The resultant precipitate was collected by filtration, washed with diethyl ether (100 mL) and dried *in vacuo* to give the DP as a white sold. A yield of 5.2 g, 37.4 mmol (93 %) was obtained. Spectroscopic data were consistent with those previously reported.[3] ^{1}H NMR (400 MHz, DMSO-d$_6$): δ = 8.24 (3H, br s, -N<u>H</u>$_3$Cl), 4.20 (2H, t, *J* = 5.2 Hz, -C<u>H</u>$_2$NH$_3$Cl), 3.05 (2H, t, *J* = 5.2 Hz, -C<u>H</u>$_2$OAc), 2.06 (3H, s, -O<u>Ac</u>) ppm;

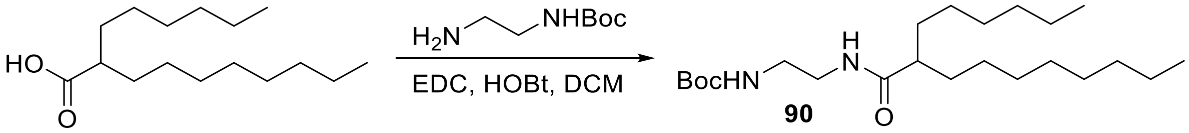

90

EDC (4.8 g, 25 mmol) and hydroxybenzotriazole (3.8 g, 25 mmol) were added to a solution of 2-Hexyldecanoic acid (5.1 mL, 16.7 mmol) and amine **88** (3.2 g, 20 mmol) in DCM (100 mL). The mixture was stirred for 18 hrs then washed with water (100 mL) and brine (100 mL). The organics were dried with MgSO$_4$, filtered and concentrated onto silica. The residue was purified by flash column chromatography eluting with 20-50 % EtOAc:Hexane. Pure fractions were concentrated *in vacuo* to give the DP as a white solid. A yield of 3 g, 7.5 mmol (45 %) was obtained. ^{1}H NMR (400 MHz, CDCl$_3$): δ = 6.28 (1H, br s, -N<u>H</u>COAlk), 4.99 (1H, br s, -N<u>H</u>Boc), 3.38 (2H, dt, *J* = 6.4, 5.1 Hz, -C<u>H</u>$_2$NHCOAlk), 3.29 (2H, t, *J* = 5.1 Hz, -C<u>H</u>$_2$NHBoc), 1.97-2.09 (1H, m, <u>H</u>$_\alpha$), 1.51-1.58 (2H, m, <u>H</u>$_\beta$), 1.35-1.51 (9H, m, <u>Alkyl</u>

and Boc), 1.15-1.35 (20H, m, Alkyl), 0.79-0.85 (6H, m, -CH_3) ppm; ^{13}C NMR (100 MHz, $CDCl_3$): δ = 177.47 (-NHCOAlkyl), 157.00 (-NHCO_2*t*Bu), 79.82 (-CMe_3),47.78 (C_α), 40.86 (-CH_2NHCOAlk), 40.39 (-CH_2NHBoc), 32.91 (Alkyl), 29.69 (Alkyl), 29.34 (Alkyl), 29.29 (Alkyl), 28.37 (Alkyl), 22.66 (Alkyl), 22.62 (Alkyl), 14.10 (-CH_3), 14.07 (-CH_3) ppm; IR (υ_{max}, solid): 3346, 3305, 2952, 2849, 1686, 1645, 1530, 1390, 1367, 1317, 1283, 1251, 1236, 1169 cm^{-1}; HRMS *m/z* (ESI+): Found: 399.3568 (M+H), Calc.: 399.3587; m.p. = 101-102 °C;

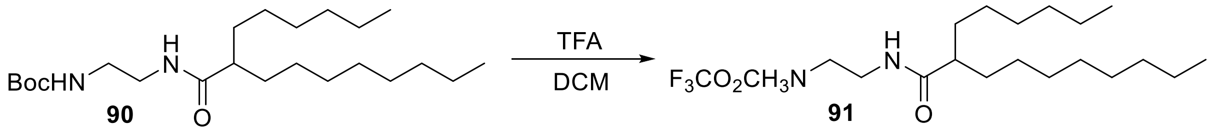

Trifluoroacetic acid (10 mL) was added to a suspension of amine **90** (3 g, 7.5 mmol) in DCM (50 mL) causing dissolution. After 3 hrs the mixture was concentrated *in vacuo* and azeotroped with toluene (2 x 50 mL) to give the DP as a white solid. NMR showed that some TFA remained in the product, but it was used in the following step without further purification. A yield of 3.09 g, 7.5 mmol (99 %) was obtained. ^{1}H NMR (400 MHz, $CDCl_3$): δ = 7.78 (3H, br s, -NH_3), 7.24-7.29 (1H, m, -NHCOAlk), 3.56-3.65 (2H, m, -CH_2NHCOAlk), 3.22-3.33 (2H, m, , -C$H_2$$NH_3$), 2.13-2.26 (1H, m, H_α), 1.38-1.55 (4H, m, H_β), 1.11-1.37 (20H, m, Alkyl), 0.82-0.92 (6H, m, -CH_3) ppm; ^{13}C NMR (100 MHz, $CDCl_3$): δ = 181.57 (-NHCOAlkyl), 47.32 (C_α), 41.07 (-CH_2NH_3), 38.21 (-CH_2NHCOAlk), 32.38 (Alkyl), 31.74 (Alkyl), 31.46 (Alkyl), 29.42 (Alkyl), 29.25 (Alkyl), 29.17 (Alkyl), 29.06 (Alkyl), 27.34 (Alkyl), 27.29 (Alkyl), 22.56 (Alkyl), 22.47 (Alkyl), 13.94 (-CH_3), 13.81 (-CH_3) ppm; IR (υ_{max}, solid): 3285, 2926, 2853, 1691, 1647, 1607, 1544, 1467, 1433, 1359, 1202, 1170, 1130 cm^{-1}; HRMS *m/z* (ESI+): Found: 299.3052 (M+H), Calc.: 299.3062; m.p. = 53-55 °C;

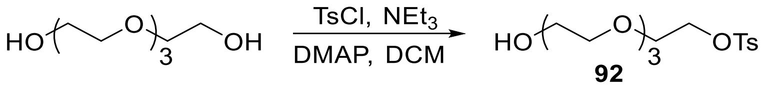

A solution of *p*-toluenesulfonyl chloride (3.8 g, 20 mmol) in DCM (200 mL) was added drop-wise over a period of 6 hrs to a mixture of tetraethylene glycol (17 mL, 100 mmol), triethylamine (3.1 mL, 22 mmol) and DMAP (244 mg, 2 mmol) in DCM (100 mL). After addition was complete the mixture was stirred for a further 10 hrs. The reaction was then washed with water (2 x 150 mL) hydrochloric acid (1 M, 2 x 150 mL), and brine (150 mL), dried with $MgSO_4$, filtered and concentrated *in vacuo* to give the DP as a

colourless oil. A yield of 6.4 g, 18.4 mmol (92 %) was obtained. The product was used in the subsequent step without further purification or analysis.

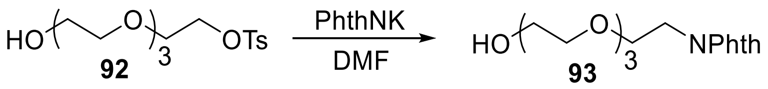

TEG tosylate **92** (6.4 g, 18.4 mmol) was dissolved in dry DMF (30 mL) under nitrogen and potassium phthalimide (4.1 g, 22 mmol) was added. After heating to 100 °C for 18 hrs, the mixture was cooled to rt and concentrated *in vacuo*. The residue was re-suspended in ethyl acetate (200 mL) and the organics washed with water (150 mL), hydrochloric acid (1 M, 2 x 150 mL) and brine (150 mL), dried with $MgSO_4$, filtered and concentrated *in vacuo*. The residue was purified by flash column chromatography eluting with 70-100 % EtOAc:Petrol. Pure fractions were concentrated *in vacuo* to give the DP as a colourless oil. A yield of 3.4 g, 10.5 mmol (57 %) was obtained. Spectroscopic data were consistent with those previously reported.[4] ^{1}H NMR (400 MHz, $CDCl_3$): δ = 7.79-7.86 (2H, m, <u>Phth</u>), 7.67-7.73 (2H, m, <u>Phth</u>), 3.84-3.93 (2H, m, TEG), 3.51-3.76 (14H, m, TEG) ppm;

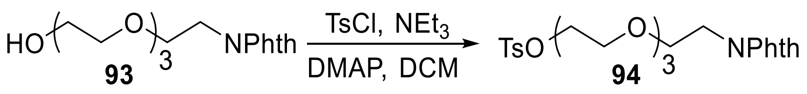

Protected TEG-amine **93** (3.4 g, 10.5 mmol) and DMAP (128 mg, 1.05 mmol) were dissolved in dry DCM (25 mL) under nitrogen and cooled to 0 ˚C. Triethylamine (2.9 mL, 21 mmol) and *p*-toluenesulfonyl chloride (2.4 g, 12.6 mmol) were then added and the reaction stirred for 4 hrs at rt. The mixture was then washed with water (100 mL), hydrochloric acid (1 M, 2 x 100 mL) and brine (100 mL), dried with $MgSO_4$, filtered and concentrated *in vacuo* to give the DP as a yellow oil. A yield of 4.76 g, 9.98 mmol (95 %) was obtained. The product was used in the subsequent step without further purification or analysis.

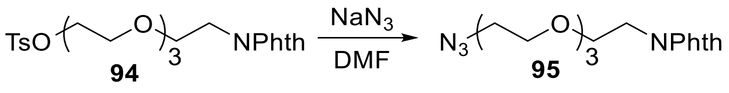

Sodium azide (89 mg, 1.37 mmol) was added to a solution of TEG-tosylate **94** (530 mg, 1.14 mmol) in dry DMF (5 mL) under nitrogen and heated to 70 °C for 48 hrs. After cooling to rt, the mixture was diluted with ethyl acetate (150 mL) and the organics washed with water (100 mL), hydrochloric acid (1 M, 100 mL) and brine (100 mL), dired with $MgSO_4$, filtered and concentrated *in vacuo*. The residue was

purified by flash column chromatography eluting with 50-60 % EtOAc:Hexane. Pure fractions were concentrated *in vacuo* to give the DP as a yellow oil. A yield of 1.8 g, 5.2 mmol (49 %) was obtained. Spectroscopic data were consistent with those previously reported.[5] ^{1}H NMR (400 MHz, $CDCl_3$): δ = 7.82-7.89 (2H, m, Phth), 7.69-7.75 (2H, m, Phth), 3.91 (2H, t, *J* = 5.7 Hz, TEG), 3.75 (2H, t, *J* = 5.6 Hz, TEG), 3.57-3.69 (10H, m, TEG), 3.37 (2H, t, *J* = 5.3 Hz, TEG) ppm;

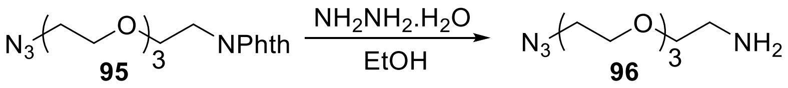

Hydrazine monohydrate (0.95 mL, 19.5 mmol) was added to a solution of TEG-azide **95** (1.7 g, 4.9 mmol) in ethanol (40 mL) under nitrogen and the mixture refluxed for 18 hrs. After cooling to rt, the resultant white precipitate was removed by filtration and the filtrate concentrated *in vacuo*. The residue was stirred in ether (20 mL) for 1 hr and the organics then filtered and concentrated *in vacuo* to give the DP as a light yellow oil. A yield of 1.06 g, 4.85 mmol (99 %) was obtained. Spectroscopic data were consistent with those previously reported.[5] ^{1}H NMR (400 MHz, $CDCl_3$): δ = 3.62-3.72 (10H, m, TEG), 3.52 (2H, t, *J* = 5.3 Hz, TEG), 3.40 (2H, t, *J* = 5.1 Hz, TEG), 2.87 (2H, t, *J* = 5.3 Hz, TEG) ppm;

Thiophene synthesis

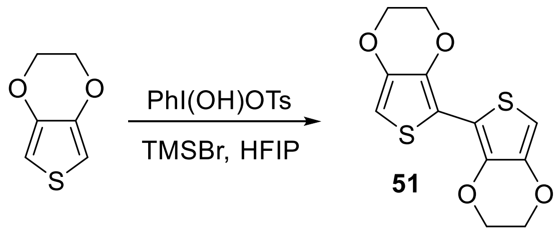

Koser's reagent (196 mg, 0.5 mmol) was added to a solution of EDOT (107 µL, 1 mmol) in HFIP (5 mL). Bromotrimethylsilane (132 mg, 1 mmol) was then added and the mixture stirred for 5 hrs. The reaction was then diluted with DCM (50 mL) and the organics washed with sat. $NaHCO_3$ (50 mL), dried with $MgSO_4$, filtered and concentrated *in vacuo*. The residue was purified by flash column chromatography eluting with 20-30 % EtOAc:Hexane. Pure fractions were concentrated *in vacuo* to give the DP as a grey solid. A yield of 65 mg, 0.23 mmol (46 %) was obtained. Spectroscopic data were consistent with those previously reported.[6] ^{1}H NMR (400 MHz, $CDCl_3$): δ = 6.29 (2H, s, ArH), 4.32-4.39 (4H, m, -OCH$_2$-), 4.23-4.30 (4H, m, -OCH$_2$-) ppm;

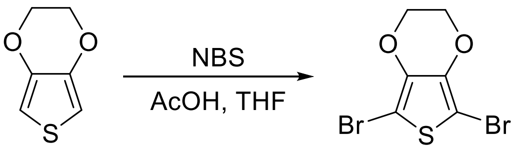

25

N-Bromosuccinimide (2.6 g, 14.7 mmol) was added to a solution of EDOT (1 g, 7 mmol) in acetic acid (15 mL) and THF (15 mL) in the dark and stirred for 4 hrs. The mixture was poured into water (150 mL) and the resultant precipitate collected by filtration, washed with water (2 x 50 mL) and the dissolved in DCM. The organics were dried with $MgSO_4$, filtered and concentrated *in vacuo* to give the DP as a light yellow solid. A yield of 1.9 g, 6.3 mmol (91 %) was obtained. Spectroscopic data were consistent with those previously reported.[7] ^{1}H NMR (400 MHz, $CDCl_3$): δ = 4.29 (4H, s, -OC<u>H</u>$_2$-) ppm;

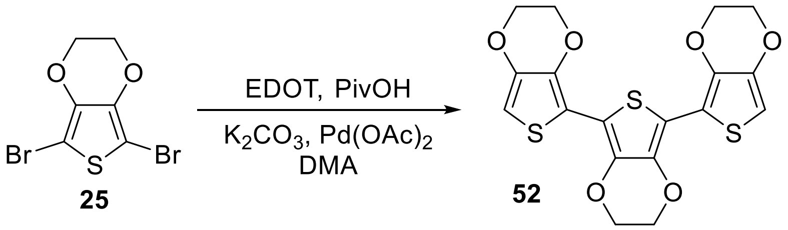

2,5-Dibromo-EDOT **25** (100 mg, 336 µmol), EDOT (143 µL, 1344 µmol), pivalic acid (10 mg, 101 µmol), palladium (**II**) acetate (4 mg, 17 µmol) and potassium carbonate (92 mg, 672 µmol) were heated to 110 ˚C in dry dimethylacetamide (500 µL) under nitrogen for 18 hrs. After cooling to rt, the mixture was diluted with DCM (50 mL), washed with water (50 mL), dried with $MgSO_4$, filtered and concentrated *in vacuo*. The residue was purified by flash column chromatography eluting with 5-20 % EtOAc:Hexane. Pure fractions were concentrated *in vacuo* to give the DP as a yellow solid. A yield of 17 mg, 40 µmol (12 %) was obtained. Spectroscopic data were consistent with those previously reported.[8] ^{1}H NMR (400 MHz, $CDCl_3$): δ = 6.30 (2H, s, Ar<u>H</u>), 4.28-4.44 (8H, m, -OC<u>H</u>$_2$-), 4.19-4.28 (4H, m, -OC<u>H</u>$_2$-) ppm;

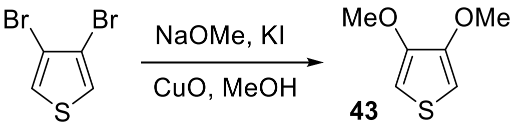

Sodium methoxide solution (25 %, 4.8 mL, 20.6 mmol) was degassed by bubbling through nitrogen for 10 min. Potassium iodide (8 mg, 0.04 mmol), copper (II) oxide (342 mg, 4.3 mmol) and 3,4-dibromothiophene (455 µL, 4.1 mmol) were added and the mixture refluxed for 18 hrs. After cooling to rt, the methanol was removed *in vacuo* and the residue diluted with water (50 mL) and extracted with

diethyl ether (2 x 50 mL). The combined organics were dried with $MgSO_4$, filtered and concentrated *in vacuo* The residue was purified by flash column chromatography, eluting with 0-5 % EtOAc:Hexane. Pure fractions were concentrated *in vacuo* to give the DP as a colourless oil. A yield of 578 mg, 4.0 mmol (98 %) was obtained. Spectroscopic data were consistent with those previously reported.[9] ^{1}H NMR (400 MHz, $CDCl_3$): δ = 6.19 (2H, s, ArH), 3.86 (6H, s, -OMe) ppm;

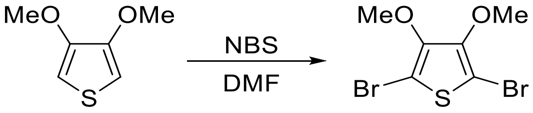

43 **50**

N-Bromosuccinimide (3.26 g, 18.3 mmol) was added to a solution of 3,4-dimethoxythiophene **43** (1 mL, 8.4 mmol) in DMF (5 mL) in the dark and stirred for 1 hr. The mixture was then diluted with diethyl ether (100 mL) and washed with water (100 mL) and brine (2 x 100 mL), dried with $MgSO_4$, filtered and concentrated *in vacuo* to give the DP as a dark brown oil. A yield of 2.7 g, 8.1 mmol (96 %) was obtained. ^{1}H NMR (400 MHz, $CDCl_3$): δ = 3.93 (6H, s, -OMe) ppm; ^{13}C NMR (400 MHz, $CDCl_3$): δ = 147.98 (ArC3), 94.80 (ArC2), 60.95 (-OMe) ppm; IR ($υ_{max}$, solid): 3002, 2939, 2882, 2830, 1566, 1495, 1450, 1424, 1345, 1211, 1204, 1152, 1040, 1009 cm^{-1}; HRMS *m/z* (CI+): Found: 302.8495 (M+H), Calc.: 302.8513;

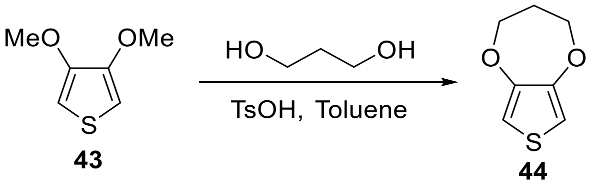

43 **44**

A mixture of 3,4-dimethoxythiophene **43** (2.06 mL, 17.4 mmol), 1,3-propanediol (6.2 mL, 87 mmol), *p*-toluenesulfonic acid monohydrate (299 mg, 1.74 mmol), and anhydrous toluene (250 mL) was degassed via argon bubbling for 30 min. The mixture was then heated to 100 °C for 48 hrs. At this point crude NMR indicated some starting material remained and so a further portion of 1,3-propanediol (5 mL, 69 mmol) and *p*-toluenesulfonic acid (299 mg, 1.74 mmol) were added and heating continued for a further 48 hrs. The mixture was then cooled to room temperature and concentrated *in vacuo*. The residue was diluted with diethyl ether (200 mL) and the organics washed with sodium hydroxide (0.5 M, 100 mL) and water (100 mL), dried with $MgSO_4$, filtered and concentrated onto silica. The residue was purified by flash column chromatography eluting with 10-20 % Et_2O:Hexane. Pure fractions were concentrated *in vacuo* to give the DP as a white solid. A yield of 1.9 g, 12.1 mmol (70 %) was obtained.

The Spectroscopic data were consistent with those previously reported.[10] ^{1}H NMR (400 MHz, $CDCl_3$): δ = 6.55 (2H, s, ArH), 4.10 (4H, t, *J* = 5.3 Hz, -OCH_2-), 2.17-2.27 (2H, m, -OCH_2CH_2-) ppm;

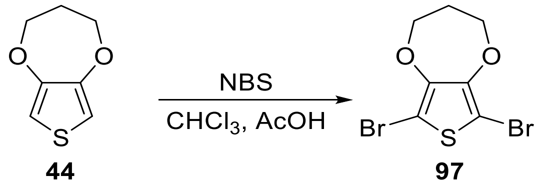

N-Bromosuccinimide (502 mg, 2.82 mmol) was added to a solution of ProDOT **44** (200 mg, 1.28 mmol) in a mixture of chloroform (50 mL) and acetic acid (10 mL) in the dark and stirred for 1 hr. The mixture was then washed with sodium hydroxide (2 M, 3 x 75 mL) and water (100 mL), dried with $MgSO_4$, filtered and concentrated *in vacuo* to give the DP as a colourless oil which solidified at -20 °C. A yield of 405 mg, 1.28 mmol (99 %) was obtained. Spectroscopic data were consistent with those previously reported.[10] ^{1}H NMR (400 MHz, $CDCl_3$): δ = 4.20 (4H, t, *J* = 5.4 Hz, -OCH_2-), 2.24-2.34 (2H, m, -OCH_2CH_2-) ppm;

EDOT functionalisation

Numbering system for functionalised-EDOT NMR assignments

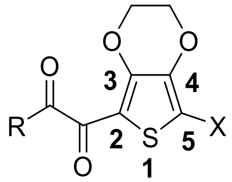

Method A: Oxalyl chloride (850 µL, 10 mmol) was added drop-wise to a solution of EDOT (1.05 mL, 10 mmol) in dioxane (30 mL). The mixture was heated to 100 ºC for 1 hr then allowed to cool to room temperature. The requisite amine (50 mmol) was then added and the mixture stirred for 3 hrs. After this time the mixture was diluted with DCM (150 mL), washed with water (100 mL), and the organics dried with $MgSO_4$, filtered and concentrated *in vacuo*. The residue was purified by flash column chromatography and pure fractions were concentrated *in vacuo*.

Method B: Oxalyl chloride (850 µL, 10 mmol) was added drop-wise to a solution of EDOT (1.05 mL, 10 mmol) in dioxane (30 mL). The mixture was heated to 100 ºC for 1 hr then allowed to cool to room temperature. The requisite amine (15 mmol) and triethylamine (7 mL, 50 mmol) were then added and

the mixture stirred for 3 hrs. After this time the mixture was diluted with DCM (150 mL), washed with water (100 mL), and the organics dried with $MgSO_4$, filtered and concentrated *in vacuo*. The residue was purified by flash column chromatography and pure fractions were concentrated *in vacuo*.

Method C: Oxalyl chloride (850 µL, 10 mmol) was added drop-wise to a solution of EDOT (1.05 mL, 10 mmol) in dioxane (30 mL). The mixture was heated to 100 ºC for 1 hr then allowed to cool to room temperature. The requisite alcohol (30 mL) and triethylamine (7 mL, 50 mmol) was then added and the mixture stirred for 3 hrs. Excess alcohol was then removed *in vacuo* and the mixture diluted with DCM (150 mL), washed with water (100 mL), and the organics dried with $MgSO_4$, filtered and concentrated *in vacuo*. The residue was purified by flash column chromatography and pure fractions were concentrated *in vacuo*.

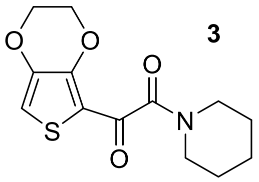

3

Method A with piperidine: Column eluted with 50-60 % EtOAc:Hexane. Yield of 2.34 g, 8.3 mmol (83 %) as a light yellow oil. ^{1}H NMR (400 MHz, $CDCl_3$): δ = 6.85 (1H, s, ArH5), 4.33-4.42 (2H, m, ArC4-OCH_2-), 4.22-4.29 (2H, m, ArC3-OCH_2-), 3.53-3.68 (2H, m, -CH_2N-), 3.30-3.42 (2H, m, -CH_2N-), 1.51-1.77 (6H, m, -CH_2CH_2CH_2N-) ppm; ^{13}C NMR (100 MHz, $CDCl_3$): δ = 182.17 (-COCONR_2), 165.09 (-CONR_2), 147.06 (ArC4), 141.79 (ArC3), 115.90 (ArC2), 111.76 (ArC5), 65.53 (ArC4-OCH_2-), 64.06 (ArC3-OCH_2-), 47.19 (-CH_2N-), 46.97 (-CH_2N-), 26.03 (-CH_2CH_2N-), 25.23 (-CH_2CH_2N-), 24.49 (-CH_2CH_2CH_2N-) ppm; IR (υ_{max}, solid): 3094, 2948, 2926, 2858, 1630, 1620, 1485, 1352, 1432, 1368, 1354, 1254, 1226, 1186, 1178, 1125, 1060, 1032, 1007 cm^{-1}; HRMS *m/z* (ESI+): Found: 282.0797 (M+H), Calc.: 282.0800; m.p. = 140-141 °C;

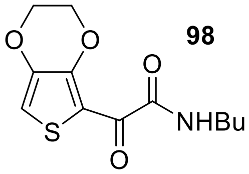

98

Method A with *n*-butylamine: Column eluted with 30-40 % EtOAc:Hexane. Yield of 2.4 g, 8.9 mmol (89 %) as a yellow solid. ^{1}H NMR (400 MHz, $CDCl_3$): δ = 7.40 (1H, br t, J = 6.8 Hz, -NH), 6.87 (1H, s, ArH5), 4.37-4.48 (2H, m, ArC4-OCH_2-), 4.17-4.31 (2H, m, ArC3-OCH_2-), 3.34 (2H, dt, J_1 = J_2 = 6.8 Hz, -NHCH_2-), 1.56 (2H, tt, J = 6.8, 7.3 Hz, -NHCH_2CH_2-), 1.37 (2H, tq, J_1 = J_2 = 7.3 Hz, -CH_2CH_3), 0.93 (3H, t, J = 7.3 Hz, -CH_3) ppm; ^{13}C NMR (100 MHz, $CDCl_3$): δ = 176.19 (-COCONHBu), 161.40 (-CONHBu), 150.17 (ArC4), 141.45 (ArC3), 115.43 (ArC5), 110.74 (ArC2), 65.57 (ArC4-OCH_2-), 63.87 (ArC3-OCH_2-), 39.15 (-NHCH_2-), 31.28 (-NHCH_2CH_2-), 21.02 (-CH_2CH_3), 14.18 (-CH_3) ppm; IR (υ_{max}, solid): 3366, 2952, 2924, 2871, 1682, 1619,

1531, 1472, 1450, 1440, 1416, 1378, 1359, 1291, 1236, 1183, 1175, 1075 cm^{-1}; HRMS *m/z* (ESI+): Found: 270.0810 (M+H), Calc.: 270.0800; m.p. = 85-87 °C;

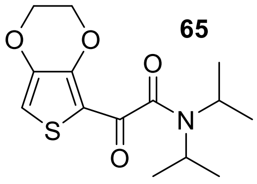

65

Method A with diisopropylamine: Column eluted with 30-50 % EtOAc:Hexane. Yield of 2.75 g, 9.2 mmol (92 %) as a yellow solid. ^{1}H NMR (400 MHz, $CDCl_3$): δ = 6.82 (1H, s, ArH5), 4.29-4.38 (2H, m, ArC4-OCH_2-), 4.20-4.27 (2H, m, ArC3-OCH_2-), 3.81 (1H, sept, *J* = 6.8 Hz, -$CHMe_2$), 3.54 (1H, sept, *J* = 6.8 Hz, -$CHMe_2$), 1.52 (6H, d, *J* = 6.8 Hz, -$CHMe_2$), 1.20 (6H, d, *J* = 6.8 Hz, -$CHMe_2$) ppm; ^{13}C NMR (100 MHz, $CDCl_3$): δ = 181.90 (-$COCONR_2$), 166.26 (-$CONR_2$), 146.74 (ArC4), 141.66 (ArC3), 116.02 (ArC2), 111.30 (ArC5), 65.14 (ArC4-OCH_2-), 64.04 (ArC3-OCH_2-), 50.26 (-$CHMe_2$), 45.79 (-$CHMe_2$), 20.56 (-$CHMe_2$), 20.03 (-$CHMe_2$) ppm; IR (υ_{max}, solid): 3101, 3073, 2968, 2933, 2878, 1626, 157, 1489, 1451, 1431, 1371, 1359, 1359, 1274, 1180, 1149, 1117, 1068, 1042, 1019 cm^{-1}; HRMS *m/z* (ESI+): Found: 298.1120 (M+H), Calc.: 298.1113; m.p. = 140-142 °C;

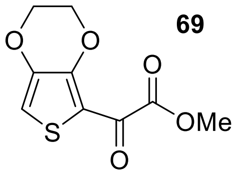

69

Method C with methanol: Column chromatography was not required to generate pure product. Yield of 2.02 g, 8.9 mmol (89 %) as a yellow solid. ^{1}H NMR (400 MHz, $CDCl_3$): δ = 6.87 (1H, s, ArH5), 4.35-4.42 (2H, m, ArC4-OCH_2-), 4.23-4.39 (2H, m, ArC3-OCH_2-), 3.93 (3H, s, -OMe) ppm; ^{13}C NMR (100 MHz, $CDCl_3$): δ = 175.04 (-$COCO_2Me$), 163.38 (-CO_2Me), 148.92 (ArC4), 141.78 (ArC3), 113.48 (ArC2), 113.20 (ArC5), 65.64 (ArC4-OCH_2-), 63.98 (ArC3-OCH_2-), 53.00 (-OMe) ppm; IR (υ_{max}, solid): 3100, 2962, 2944, 1729, 1651, 1475, 1440, 1428, 1362, 1316, 1272, 1247, 1227, 1177, 1126, 1069, 1027 cm^{-1}; HRMS *m/z* (ESI+): Found: 229.0165 (M+H), Calc.: 229.0166; m.p. = 105-106 °C;

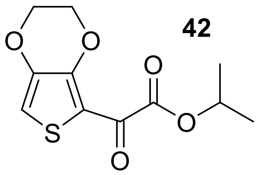

42

Method C with *i*-propanol: Column eluted with 30 % EtOAc:Hexane. Yield of 2.33 g, 9.1 mmol (91 %) as a light yellow oil which solidified on standing. ^{1}H NMR (400 MHz, $CDCl_3$): δ = 6.85 (1H, s, ArH5), 5.23 (1H, sept, *J* = 6.3 Hz, -$CHMe_2$), 4.32-4.41 (2H, m, ArC4-OCH_2-), 4.21-4.31 (2H, m, ArC3-OCH_2-), 1.37 (6H, d, *J* = 6.3 Hz, -$CHMe_2$) ppm; ^{13}C NMR (100 MHz, $CDCl_3$): δ = 176.37 (-$COCO_2$*i*Pr), 162.98 (-CO_2*i*Pr), 148.38 (ArC4), 141.71 (ArC3), 114.02 (ArC2), 112.62 (ArC5), 70.56 (-$CHMe_2$), 65.40 (ArC4-OCH_2-), 63.99 (ArC3-OCH_2-), 21.57 (-$CHMe_2$) ppm; IR (υ_{max}, solid): 2991, 2941, 1731, 1636, 1488, 1439, 1356, 1314, 1246, 1225, 1180, 1104, 1062, 1016 cm^{-1}; HRMS *m/z* (ESI+): Found: 257.0485 (M+H), Calc.: 257.0484; m.p. = 74-77 °C;

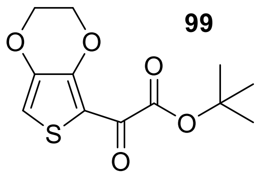

99 Method C with *t*-butanol: Column eluted with 30-50 % EtOAc:Hexane. Yield of 914 mg, 3.38 mmol (38 %) as a yellow oil which solidified on standing. ^{1}H NMR (400 MHz, $CDCl_3$): δ = 6.85 (1H, s, ArH5), 4.34-4.43 (2H, m, ArC4-OCH_2-), 4.21-4.33 (2H, m, ArC3-OCH_2-), 1.60 (9H, s, -O*t*Bu) ppm; ^{13}C NMR (100 MHz, $CDCl_3$): δ = 176.81 (-$COCO_2$*t*Bu), 162.70 (-CO_2*t*Bu), 148.09 (ArC4), 141.65 (ArC3), 114.14 (ArC2), 112.23 (ArC5), 84.34 (-CMe_3), 65.30 (ArC4-OCH_2-), 64.01 (ArC3-OCH_2-), 27.94 (-CMe_3) ppm; IR ($υ_{max}$, solid): 2980, 2935, 1721, 1632, 1488, 1452, 1436, 1359, 1337, 1251, 1228, 1173, 1155, 1127, 1064 cm^{-1}; HRMS *m/z* (ESI+): Found: 271.0643 (M+H), Calc.: 271.0640; m.p. = 64-66 °C;

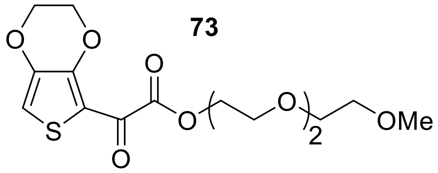

73 Method C with triethylene glycol monomethyl ether: Column eluted with 70-100 % EtOAc:Hexane. Yield of 3.3 g, 9.2 mmol (92 %) as a yellow oil. ^{1}H NMR (400 MHz, $CDCl_3$): δ = 6.85 (1H, s, ArH5), 4.43-4.47 (2H, m, TEG), 4.34-4.38 (2H, m, ArC4-OCH_2-), 4.21-4.25 (2H, m, ArC3-OCH_2-), 3.77-3.81 (2H, m, TEG), 3.65-3.69 (2H, m, TEG), 3.59-3.65 (4H, m, TEG), 3.49-3.53 (2H, m, TEG), 3.34 (3H, s, -OMe) ppm; ^{13}C NMR (100 MHz, $CDCl_3$): δ = 175.37 (-$COCO_2$R), 163.11 (-CO_2R), 148.78 (ArC4), 141.77 (ArC3), 113.71 (ArC2), 112.96 (ArC5), 71.87 (TEG), 70.70 (TEG), 70.59 (TEG), 70.54 (TEG), 68.55 (TEG), 65.61 (ArC4-OCH_2-), 65.31 (TEG), 64.02 (ArC3-OCH_2-), 58.97 (-OMe) ppm; IR ($υ_{max}$, oil): 2876, 1740, 1638, 1488, 1452, 1434, 1360, 1317, 1248, 1220, 1174, 1121, 1099, 1064, 1025 cm^{-1}; HRMS *m/z* (ESI+): Found: 361.0966 (M+H), Calc.: 361.0957;

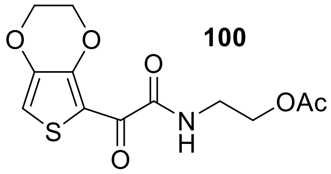

100 Method B with amine **83**: Column eluted with 50-70 % EtOAc:Hexane. Yield of 1.9 g, 6.3 mmol (63 %) as a yellow solid. ^{1}H NMR (400 MHz, $CDCl_3$): δ = 7.65 (1H, br t, *J* = 6.2 Hz, -NH), 6.89 (1H, s, ArH5), 4.41-4.47 (2H, m, ArC4-OCH_2-), 4.24-4.28 (2H, m, ArC3-OCH_2-), 4.22 (2H, t, *J* = 5.4 Hz, -CH_2OAc), 3.63 (2H, dt, *J* = 6.2, 5.4 Hz, -$NHCH_2$-), 2.08 (3H, s, -OAc) ppm; ^{13}C NMR (100 MHz, $CDCl_3$): δ = 175.67 (-COCONHR), 170.81 ($MeCO_2$-), 161.64 (-CONHR), 150.39 (ArC4), 141.54 (ArC3), 115.60 (ArC5), 110.64 (ArC2), 65.60 (ArC4-OCH_2-), 63.89 (ArC3-OCH_2-), 62.60 (-CH_2OAc), 38.44 (-$NHCH_2$-), 20.79 ($MeCO_2$-) ppm; IR ($υ_{max}$, solid): 3346, 3085, 2924, 1718, 1682, 1637, 1524, 1473, 1464, 1441, 1422, 1369, 1357, 1266, 1253, 1241, 1190, 1114, 1069, 1051 cm^{-1}; HRMS *m/z* (ESI+): Found: 300.0534 (M+H), Calc.: 300.0542; m.p. = 105-108 °C;

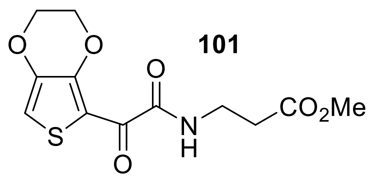

101

Method B using only 6 mmol of amine **89**: Column eluted 30-50 % EtOAc:Hexane. Yield of 1.3 g, 4.2 mmol (72 %) as a yellow solid. ^{1}H NMR (400 MHz, $CDCl_3$): δ = 7.80 (1H, br t, *J* = 6.4 Hz, -NH), 6.89 (1H, s, ArH5), 4.42-4.50 (2H, m, ArC4-OCH_2-), 4.23-4.29 (2H, m, ArC3-OCH_2-), 3.72 (3H, s, -CO_2Me), 3.65 (2H, dt, J_1 = J_2 = 6.4 Hz, -$NHCH_2$-), 2.64 (2H, t, *J* = 6.4 Hz, -CH_2CO_2Me) ppm; ^{13}C NMR (100 MHz, $CDCl_3$): δ = 175.78 (-COCONHR), 172.09 (-CO_2Me), 161.53 (-CONHR), 150.28 (ArC4), 141.52 (ArC3), 115.39 (ArC5), 110.71 (ArC2), 65.59 (ArC4-OCH_2-), 63.90 (ArC3-OCH_2-), 51.94 (-CO_2Me), 34.90 (-$NHCH_2$-), 33.58 (-CH_2CO_2Me) ppm; IR ($υ_{max}$, solid): 2938, 2856, 1716, 1683, 1634, 1620, 1606, 1546, 1467, 1435, 1375, 1361, 1217, 1196, 1171, 1155, 1132, 1069 cm^{-1}; HRMS *m/z* (ESI+): Found: 300.0550 (M+H), Calc.: 300.0542; m.p. = 120-125 °C;

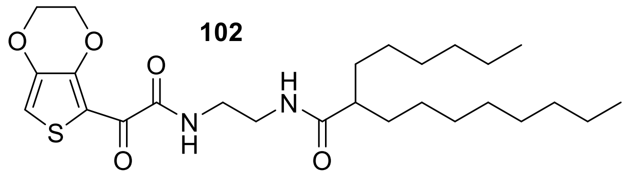

102

Method B with amine **91**: Column eluted with 60-80 % EtOAc:Hexane. Yield of 3.4 g, 6.8 mmol (68 %) as a light yellow solid. ^{1}H NMR (400 MHz, $CDCl_3$): δ = 7.82 (1H, br t, *J* = 5.9 Hz, -NHCOCOAr), 6.88 (1H, s, ArH5), 6.06 (1H, br t, *J* = 5.1 Hz, -NHCOAlk), 4.38-4.47 (2H, m, ArC4-OCH_2-), 4.28-4.37 (2H, m, ArC3-OCH_2-), 3.39-3.59 (4H, m, -$NHCH_2CH_2NH$-), 1.95-2.05 (1H, m, $H_α$), 1.48-1.63 (2H, m, $H_β$), 1.31-1.45 (2H, m, $H_β$), 1.11-1.31 (20H, m, Alkyl), 0.81-0.92 (6H, m, -CH_3) ppm; ^{13}C NMR (100 MHz, $CDCl_3$): δ = 176.84 (-NHCOAlkyl) 175.46 (-COCONHR), 162.52 (-COCONHR), 150.37 (ArC4), 141.55 (ArC3), 115.39 (ArC5), 110.61 (ArC2), 65.56 (ArC4-OCH_2-), 63.89 (ArC3-OCH_2-), 48.11 ($C_α$), 39.47 (-$NHCH_2$-), 39.41 (-$NHCH_2$-), 33.01 ($C_β$), 31.84 (Alkyl), 31.67 (Alkyl), 29.44 (Alkyl), 29.35 (Alkyl), 29.32 (Alkyl), 22.66 (Alkyl), 22.63 (Alkyl), 14.11 (-CH_3), 14.06 (-CH_3) ppm; IR ($υ_{max}$, solid): 3280, 2923, 2852, 1638, 1469, 1423, 1360, 1252, 1216, 1174, 1117, 1080, 1065 cm^{-1}; HRMS *m/z* (ESI+): Found: 495.2877 (M+H), Calc.: 495.2893; m.p. = 141-143 °C;

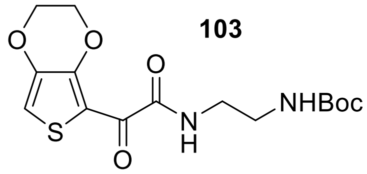

103

Method B with amine **88**: Column eluted with 30-60 % EtOAc:Hexane. Yield of 1.7 g, 4.8 mmol (48 %) as a yellow solid. ^{1}H NMR (400 MHz, $CDCl_3$): δ = 7.80 (1H, br app s, -NHCOCOAr), 6.88 (1H, s, ArH5), 4.96 (1H, br app s, -NHBoc), 4.36-4.48 (2H, m, ArC4-OCH_2-), 4.21-4.29 (2H, m, ArC3-OCH_2-), 3.49 (2H td, *J* = 6.6, 5.0 Hz, -$CH_2NHCOCOAr$), 3.35 (2H, dt, J_1 = J_2 = 6.6 Hz, -CH_2NHBoc), 1.43 (9H, s, Boc) ppm; ^{13}C NMR (100 MHz, $CDCl_3$): δ = 175.80 (-COCONHR), 162.09 (-COCONHR), 156.22 (-CO_2*t*Bu), 150.27

(ArC4), 141.50 (ArC3), 115.40 (ArC5), 110.70 (ArC2), 79.71 (-CMe$_3$), 65.56 (ArC4-OCH$_2$-), 63.89 (ArC3-OCH$_2$-), 39.93 (-CH$_2$NH-), 28.35 (-CMe$_3$) ppm; IR (υ_{max}, solid): 3360, 3309, 2980, 2933, 2873, 1735, 1703, 1677, 1621, 1508, 1470, 1439, 1426, 1358, 1264, 1246, 1224, 1163, 1142, 1118, 1071 cm^{-1}; HRMS *m/z* (ESI+): Found: 357.1137 (M+H), Calc.: 357.1120; m.p. = 81-83 °C;

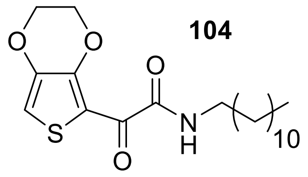

104

Method B with dodecylamine: Column eluted with 20-30 % EtOAc:Hexane. Yield of 3.6 g, 9.4 mmol (94 %) as a yellow solid. ^{1}H NMR (400 MHz, CDCl$_3$): δ = 7.41 (1H, br t, *J* = 6.1 Hz, -NH), 6.89 (1H, s, ArH5), 4.39-4.49 (2H, m, ArC4-OCH$_2$-), 4.21-4.32 (2H, m, ArC3-OCH$_2$-), 3.28-3.41 (2H, m, -NHCH$_2$-), 1.54-1.63 (2H, m, -NHCH$_2$CH$_2$-), 1.24-1.41 (18H, m, Alkyl), 0.89 (3H, t, *J* = 6.8 Hz, -CH$_3$) ppm; ^{13}C NMR (100 MHz, CDCl$_3$): δ = 176.21 (-COCONH-), 161.37 (-CONH-), 150.20 (ArC4), 141.46 (ArC3), 115.49 (ArC5), 110.77 (ArC2), 65.59 (ArC4-OCH$_2$-), 63.89 (ArC3-OCH$_2$-), 39.47 (-NHCH$_2$-), 31.91 (-NHCH$_2$CH$_2$-), 29.62 (Alkyl), 29.55 (Alkyl), 29.50 (Alkyl), 29.34 (Alkyl), 29.26 (Alkyl), 29.22 (Alkyl), 26.86 (Alkyl), 22.69 (Alkyl), 14.13 (-CH$_3$) ppm; IR (υ_{max}, solid): 3326, 2921, 2848, 1657, 1633, 1530, 1482, 1462, 1450, 1425, 1367, 1176, 1079, 1069 cm^{-1}; HRMS *m/z* (ESI+): Found: 382.2053 (M+H), Calc.: 382.2052; m.p. = 77-78 °C;

Monomer bromination

Bromination procedure A: EDOT derivative (5 mmol) was dissolved in a mixture of THF (5 mL) and acetic acid (3 mL). If solubility was poor a further 25 mL of THF was added. The mixture was placed in the dark and *N*-bromosuccinimide (6 mmol) was added. After stirring for 2 hrs the mixture was poured into water (150 mL) causing precipitation of the product. The solid was collected by filtration or centrifugation (5000 rpm, 10 min), washed with water (50 mL) and then dissolved in DCM. The organics were dried with $MgSO_4$, filtered and concentrated *in vacuo*. Column chromatography was then undertaken if required.

Bromination procedure B: Reaction procedure A was followed. After pouring into water the product was extracted with DCM (200 mL). The organics were washed with sat. $NaHCO_3$ (2 x 100 mL), dried with $MgSO_4$, filtered and concentrated *in vacuo*. Column chromatography was then undertaken if required.

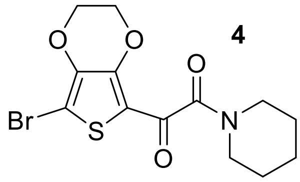

4

Method B on 3.9 mmol scale: Yield of 1.4 g, 3.9 mmol (99 %) as a yellow solid. ^{1}H NMR (400 MHz, $CDCl_3$): δ = 4.35-4.42 (2H, m, ArC4-$OC\underline{H}_2$-), 4.30-4.35 (2H, m, ArC3-$OC\underline{H}_2$-), 3.60-3.65 (2H, m, -$C\underline{H}_2N$-), 3.34-3.39 (2H, m, -$C\underline{H}_2N$-), 1.52-1.74 (6H, m, -$C\underline{H}_2C\underline{H}_2CH_2N$-) ppm; ^{13}C NMR (100 MHz, $CDCl_3$): δ = 180.86 (-$\underline{C}$OCON-), 164.67 (-$\underline{C}$ON-), 146.29 (Ar$\underline{C}$4), 140.27 (Ar$\underline{C}$3), 115.91 (Ar$\underline{C}$2), 102.66 (Ar$\underline{C}$5), 65.54 (ArC4-$O\underline{C}H_2$-), 64.49 (ArC3-$O\underline{C}H_2$-), 46.99 (-$C\underline{H}_2N$-), 42.33 (-$C\underline{H}_2N$-), 26.09 (-$\underline{C}H_2CH_2N$-), 25.24 (-$\underline{C}H_2CH_2N$-), 24.45 (-$\underline{C}H_2CH_2CH_2N$-) ppm; IR ($υ_{max}$, solid): 3342, 1946, 2859, 1627, 1491, 1478, 1425, 1354, 1252, 1124, 1080 cm^{-1}; HRMS *m/z* (ESI+): Found: 359.9904/361.9890 (M+H), Calc.: 359.9905/361.9885; m.p. = 157-160 °C;

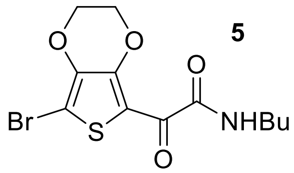

5

Method A on 18.6 mmol scale: Yield of 6.4 g, 18.5 mmol (99 %) as a yellow solid. ^{1}H NMR (400 MHz, $CDCl_3$): δ = 7.38 (1H, br t, *J* = 6.2 Hz, -N$\underline{H}$), 4.41-4.50 (2H, m, ArC4-$OC\underline{H}_2$-), 4.32-4.39 (2H, m, ArC3-$OC\underline{H}_2$-), 3.36 (2H, td, *J* = 7.1, 6.2 Hz, -$NHC\underline{H}_2$-), 1.58 (2H, tt, *J* = 7.6, 7.1 Hz, -$NHCH_2C\underline{H}_2$-), 1.39 (2H, tq, *J* = 7.6, 7.3 Hz, -$C\underline{H}_2CH_3$), 0.96 (3H, t, *J* = 7.3 Hz, -$C\underline{H}_3$) ppm; ^{13}C NMR (100 MHz, $CDCl_3$): δ = 175.05 (-$\underline{C}$OCONHBu), 161.29 (-$\underline{C}$ONHBu), 149.44 (Ar$\underline{C}$4), 139.99 (Ar$\underline{C}$3), 110.71 (Ar$\underline{C}$2), 106.88 (Ar$\underline{C}$5), 65.58 (ArC4-$O\underline{C}H_2$-), 64.35 (ArC3-$O\underline{C}H_2$-), 39.25 (-$NH\underline{C}H_2$-), 31.27 (-$NHCH_2\underline{C}H_2$-), 20.03 (-$\underline{C}H_2CH_3$), 13.69 (-$\underline{C}H_3$) ppm; IR ($υ_{max}$, solid): 3308, 2954, 2930, 2869, 1659, 1526, 1545, 1480, 1456, 1442, 1418, 1367, 1355, 1267, 1247, 1229, 1142, 1084 cm^{-1}; HRMS *m/z* (ESI+): Found: 347.9906/349.9891 (M+H), Calc.: 347.9905/349.9884; m.p. = 160-164 °C;

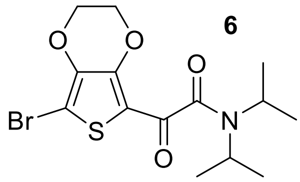

6

Method B on 10 mmol scale: Yield of 3.75 g, 9.9 mmol (99 %) as a yellow oil which solidified on standing. ^{1}H NMR (400 MHz, $CDCl_3$): δ = 4.34 (4H, app s, -$OC\underline{H}_2$-), 3.81 (1H, sept, *J* = 6.6 Hz, -$C\underline{H}Me_2$), 3.55 (1H, sept, *J* = 6.6 Hz, -$C\underline{H}Me_2$), 1.51 (6H, d, *J* = 6.6 Hz, -$CH\underline{Me}_2$), 1.21 (6H, d, *J* = 6.6 Hz, -$CH\underline{Me}_2$) ppm; ^{13}C NMR (100 MHz, $CDCl_3$): δ = 180.70 (-$\underline{C}$OCON-), 165.88 (-$\underline{C}$ON-), 145.91 (Ar$\underline{C}$4), 140.15 (Ar$\underline{C}$3), 116.07 (Ar$\underline{C}$2), 102.04 (Ar$\underline{C}$5), 65.15 (ArC4-$O\underline{C}H_2$-), 64.49 (ArC3-$O\underline{C}H_2$-), 50.29 (-$\underline{C}HMe_2$), 45.85 (-$\underline{C}HMe_2$), 20.60 (-$CH\underline{Me}_2$), 19.98 (-$CH\underline{Me}_2$) ppm; IR ($υ_{max}$, solid): 3360, 3297, 2924, 2853, 1631, 1545, 1475, 1422, 1355, 1270, 1249, 1233, 147, 1085 cm^{-1}; HRMS *m/z* (ESI+): Found: 376.0246/378.0227 (M+H), Calc.: 376.0243/378.0198; m.p. = 137-139 °C;

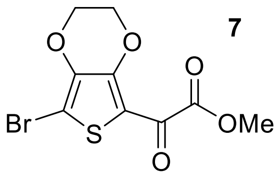

7 Method A, washing DCM with sat. $NaHCO_3$ (50 mL) before drying and concentrating. Yield of 1.42 g, 4.6 mmol (93 %) as a yellow solid. ^{1}H NMR (400 MHz, $CDCl_3$): δ = 4.40-4.45 (2H, m, ArC4-OCH$_2$-), 4.31-4.37 (2H, m, ArC3-OCH$_2$-), 3.93 (3H, s, -OMe) ppm; ^{13}C NMR (100 MHz, $CDCl_3$): δ = 173.24 (-COCO$_2$Me), 162.88 (-CO$_2$Me), 148.35 (ArC4), 140.33 (ArC3), 113.26 (ArC2), 104.38 (ArC5), 65.60 (ArC4-OCH$_2$-), 64.43 (ArC3-OCH$_2$-), 53.20 (-OMe) ppm; IR (υ_{max}, solid): 3360, 3296, 2923, 2852, 1680, 1632, 1544, 1475, 1421, 1357, 1248, 1235, 1089 cm^{-1}; HRMS *m/z* (ESI+): Found: 306.9282/308.9269 (M+H), Calc.: 306.9276/308.9255; m.p. = 133-137 °C;

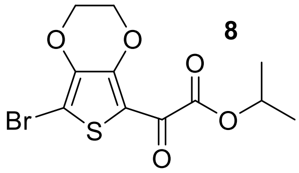

8 Method B on 13.6 mmol scale: Yield of 4.2 g, 12.5 mmol (92 %) as a yellow oil which slowly solidified on standing. ^{1}H NMR (400 MHz, $CDCl_3$): δ = 5.23 (1H, sept, *J* = 6.3 Hz, -CHMe$_2$), 4.38-4.42 (2H, m, ArC4-OCH$_2$-), 4.33-4.38 (2H, m, ArC3-OCH$_2$-), 1.38 (6H, d, *J* = 6.3 Hz, CHMe$_2$) ppm; ^{13}C NMR (100 MHz, $CDCl_3$): δ = 174.56 (-COCO$_2$*t*Bu), 162.37 (-CO$_2$*t*Bu), 147.83 (ArC4), 140.23 (ArC3), 113.78 (ArC2), 103.77 (ArC5), 70.95 (-CHMe$_2$), 65.39 (ArC4-OCH$_2$-), 64.44 (ArC3-OCH$_2$-), 21.58 (-CHMe$_2$) ppm; IR (υ_{max}, solid): 2982, 2934, 2876, 1735, 1713, 1641, 1480, 1472, 1430, 1356, 1314, 1246, 1221, 1174, 1131, 1099, 1075, 1035 cm^{-1}; HRMS *m/z* (ESI+): Found: 334.9583/336.9562 (M+H), Calc.: 334.9589/336.9568; m.p. = 71-73 °C;

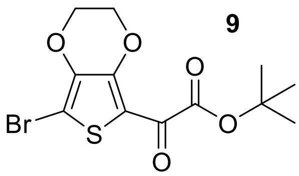

9 Method A on 1.22 mmol scale. DMF was used as solvent in place of THF/AcOH to prevent hydrolysis of the *t*-butyl group. Yield of 360 mg, 1.03 mmol (85 %) as a yellow solid. ^{1}H NMR (400 MHz, $CDCl_3$): δ = 4.37-4.41 (2H, m, ArC4-OCH$_2$-), 4.33-4.37 (2H, m, ArC3-OCH$_2$-), 1.60 (9H, s, -O*t*Bu) ppm; ^{13}C NMR (100 MHz, $CDCl_3$): δ = 175.21 (-COCO$_2$*t*Bu), 162.11 (-CO$_2$*t*Bu), 147.51 (ArC4), 140.18 (ArC3), 113.88 (ArC2), 103.23 (ArC5), 84.67 (-CMe$_3$), 65.31 (ArC4-OCH$_2$-), 64.45 (ArC3-OCH$_2$-), 27.80 (-CMe$_3$) ppm; IR (υ_{max}, solid): 2980, 2934, 1715, 1647, 1485, 1473, 1428, 1367, 1357, 1331, 1248, 1226, 1165, 1128, 1080, 1033 cm^{-1}; HRMS *m/z* (ESI+): Found: 348.9734/350.9714 (M+H), Calc.: 348.9745/350.9725; m.p. = 104-106 °C;

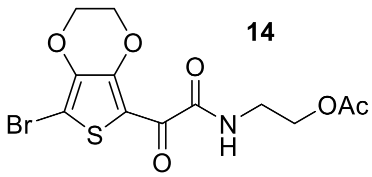

14 Method B on 1.87 mmol scale: Purified by column chromatography eluting with 50 % EtOAc:Hexane. Yield of 602 mg, 1.59 mmol (85 %) as a yellow solid. ^{1}H NMR (400 MHz, $CDCl_3$): δ = 7.64 (1H, br t, *J* = 6.3 Hz, -NH), 4.42-4.49 (2H, m, ArC4-OCH$_2$-), 4.30-4.40 (2H, m, ArC3-OCH$_2$-), 4.22 (2H, t, *J* = 5.4 Hz,

-CH_2OAc), 3.62 (2H, dt, *J* = 6.3, 5.4 Hz, -NHCH_2-), 2.08 (3H, s, -OAc) ppm; ^{13}C NMR (100 MHz, $CDCl_3$): δ = 174.48 (-COCONH-), 170.83 (MeCO_2-), 161.53 (-CONH-), 149.63 (ArC4), 140.07 (ArC3), 110.58 (ArC2), 106.99 (ArC5), 65.58 (ArC4-OCH_2-), 64.35 (ArC3-OCH_2-), 62.54 (-CH_2OAc), 38.54 (-NHCH_2-), 20.80 (MeCO_2-) ppm; IR (υ_{max}, solid): 3345, 2947, 1723, 1680, 1626, 1543, 1474, 1442, 1422, 1355, 1245, 1221, 1200, 1148, 1081, 1037 cm^{-1}; HRMS *m/z* (ESI+): Found: 377.9654/379.9630 (M+H), Calc.: 377.9647/379.9630; m.p. = 143-147 °C;

17

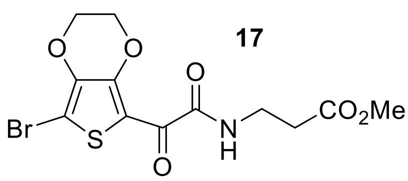

Method A, washing DCM with sat. $NaHCO_3$ (50 mL) before drying and concentrating. Yield of 1.72 g, 4.6 mmol (93 %) as a yellow solid. ^{1}H NMR (400 MHz, $CDCl_3$): δ = 7.79 (1H, br s, -NH), 4.40-4.49 (2H, m, ArC4-OCH_2-), 4.30-4.39 (2H, m, ArC3-OCH_2-), 3.73 (3H, s, -CO_2Me), 3.65 (2H, dt, $J_1 = J_2$ = 6.3 Hz, -NHCH_2-), 2.63 (2H, t, *J* = 6.4 Hz, -CH_2CO_2Me) ppm; ^{13}C NMR (100 MHz, $CDCl_3$): δ = 174.61 (-COCONH-), 172.06 (-CO_2Me), 161.40 (-CONH-), 149.50 (ArC4), 140.04 (ArC3), 110.66 (ArC2), 106.75 (ArC5), 65.56 (ArC4-OCH_2-), 64.36 (ArC3-OCH_2-), 51.97 (-CO_2Me), 34.95 (-NHCH_2-), 33.52 (-CH_2CO_2Me) ppm; IR (υ_{max}, solid): 3344, 2951, 1725, 1680, 1627, 1546, 1475, 1423, 1357, 1321, 1295, 1199, 1176, 1079 cm^{-1}; HRMS *m/z* (ESI+): Found: 377.9645/379.9630 (M+H), Calc.: 377.9647/379.9626; m.p. = 199-203 °C (Degrades);

18

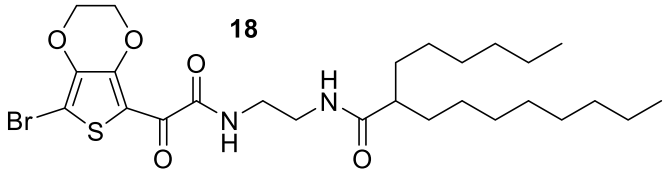

Method A on 3.4 mmol scale: Yield of 1.9 g, 3.3 mmol (98 %) as a yellow solid. ^{1}H NMR (400 MHz, $CDCl_3$): δ = 7.82 (1H, br t, *J* = 5.6 Hz, -NHCOCO-), 6.06 (1H, br t, *J* = 5.1 Hz, -NHCOAlk), 4.40-4.48 (2H, m, ArC4-OCH_2-), 4.30-4.38 (2H, m, ArC3-OCH_2-), 3.45-3.56 (4H, m, -NHCH_2CH_2NH-), 1.95-2.05 (1H, m, H_α), 1.51-1.62 (2H, m, H_β), 1.33-1.44 (2H, m, H_β), 1.15-1.32 (20H, m, Alkyl), 0.81-0.91 (6H, m, -CH_3) ppm; ^{13}C NMR (100 MHz, $CDCl_3$): δ = 176.92 (-NHCOAlkyl) 174.28 (-COCONH-), 162.37 (-COCONH-), 149.58 (ArC4), 140.06 (ArC3), 110.54 (ArC2), 106.76 (ArC5), 65.54 (ArC4-OCH_2-), 64.53 (ArC3-OCH_2-), 48.11 (C_α), 39.64 (-NHCH_2-), 39.32 (-NHCH_2-), 33.00 (C_β), 31.86 (Alkyl), 29.35 (Alkyl), 27.65 (Alkyl), 22.67 (Alkyl), 22.64 (Alkyl), 14.12 (-CH_3), 14.07 (-CH_3) ppm; IR (υ_{max}, solid): 3360, 3294, 2923, 2852, 1680, 1635, 1544, 1474, 1444, 1421, 1358, 1296, 1248, 1235, 1145, 1089 cm^{-1}; HRMS *m/z* (ESI+): Found: 573.1982/575.1966 (M+H), Calc.: 573.1998/575.1978; m.p. = 159-162 °C;

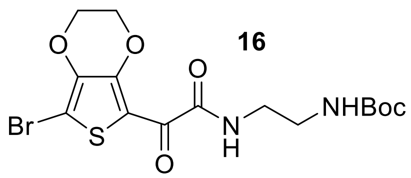

Method A on 1.96 mmol scale. DMF was used as solvent in place of THF/AcOH to prevent Boc cleavage. Purification by flash column chromatography was undertaken eluting with 30-60 % EtOAc:Hexane Yield of 746 mg, 1.71 mmol (87 %) as a yellow solid. ^{1}H NMR (400 MHz, CDCl$_3$): δ = 7.81 (1H, app s, -COCONHR), 4.93 (1H, app s, -NHBoc), 4.41-4.49 (2H, m, ArC4-OCH$_2$-), 4.29-4.39 (2H, m, ArC3-OCH$_2$-), 3.49 (2H, dt, J_1 = J_2 = 7.1 Hz, -COCONHCH$_2$-), 3.36 (2H, dt, J_1 = J_2 = 7.1 Hz, -CH$_2$NHBoc), 1.45 (9H, s, Boc) ppm; ^{13}C NMR (100 MHz, CDCl$_3$): δ = 174.60 (-COCONH-), 161.93 (-CONH-), 156.25 (-CO$_2$*t*Bu), 149.50 (ArC4), 140.03 (ArC3), 110.63 (ArC2), 106.75 (ArC5), 79.78 (-CMe$_3$), 65.55 (ArC4-OCH$_2$-), 64.35 (ArC3-OCH$_2$-), 40.09 (-CH$_2$NH-), 39.93 (-CH$_2$NH-), 28.36 (-CMe$_3$) ppm; IR (υ_{max}, solid): 2981, 2942, 2879, 1684, 1640, 1528, 1478, 1421, 1367, 1276, 1247, 1235, 1167, 1144, 1089 cm^{-1}; HRMS *m/z* (ESI+): Found: 435.0232/437.0220 (M+H), Calc.: 435.0225/437.0205; m.p. = 162-167 °C;

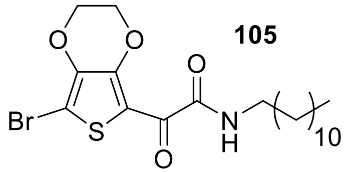

Method A on 4.2 mmol scale: Yield of 1.2 g, 2.6 mmol (62 %) as a yellow solid. ^{1}H NMR (400 MHz, CDCl$_3$): δ = 7.37 (1H, br t, J = 6.1 Hz, -NH), 4.41-4.50 (2H, m, ArC4-OCH$_2$-), 4.31-4.38 (2H, m, ArC3-OCH$_2$-), 3.34 (2H, dt, J_1 = J_2 = 6.9 Hz, -NHCH$_2$-), 1.52-1.66 (2H, m, -NHCH$_2$CH$_2$-), 1.20-1.38 (18H, m, Alkyl), 0.89 (3H, t, J = 6.7 Hz, -CH$_3$) ppm; ^{13}C NMR (100 MHz, CDCl$_3$): δ = 175.05 (-COCONH-), 161.26 (-CONH-), 149.41 (ArC4), 139.98 (ArC3), 110.72 (ArC2), 106.84 (ArC5), 65.57 (ArC4-OCH$_2$-), 64.35 (ArC3-OCH$_2$-), 39.54 (-NHCH$_2$-), 31.91 (-NHCH$_2$CH$_2$-), 29.62 (Alkyl), 29.54 (Alkyl), 29.49 (Alkyl), 29.34 (Alkyl), 29.21 (Alkyl), 26.84 (Alkyl), 22.69 (Alkyl), 14.13 (-CH$_3$) ppm; IR (υ_{max}, solid): 2919, 2869, 2849, 1628, 1468, 1435, 1424, 1356, 1264, 1222, 1211, 1077, 1064, 1042 cm^{-1}; HRMS *m/z* (ESI+): Found: 460.1165/462.1144 (M+H), Calc.: 460.1157/462.1136; m.p. = 132-134 °C;

Monomer manipulations

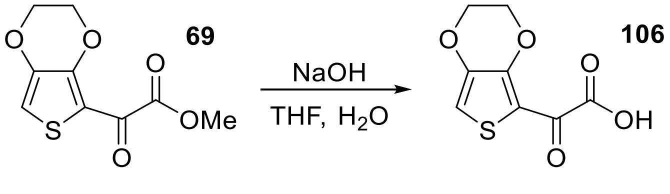

Sodium hydroxide (2 M, 5 mL, 10 mmol) was added to a solution of methoxy-EDOT **69** (0.5 g, 2.2 mmol) in THF (10 mL) and the mixture stirred for 2 hrs. The THF was then removed *in vacuo* and the residue

acidified with hydrochloric acid (1 M). The resultant precipitate was extracted with ethyl acetate (2 x 50 mL) and the organics dried with $MgSO_4$, filtered and concentrated *in vacuo* to give the DP as a yellow solid. ^1H NMR (400 MHz, DMSO-d_6): δ = 14.32 (1H, br s, -CO_2H), 7.36 (1H, s, ArH5), 4.34-4.42 (2H, m, ArC4-OCH_2-), 4.22-4.32 (2H, m, ArC3-OCH_2-) ppm; ^{13}C NMR (100 MHz, DMSO-d_6): δ = 178.41 (-COCO_2H), 165.80 (-CO_2H), 148.96 (ArC4), 142.15 (ArC3), 113.57 (ArC5), 113.22 (ArC2), 66.09 (ArC4-OCH_2-), 64.34 (ArC3-OCH_2-) ppm; IR ($υ_{max}$, solid): 2953, 1754, 1621, 1487, 1454, 1436, 1366, 1335, 1262, 1253, 12284, 1070 cm^{-1}; HRMS *m/z* (ESI-): Found: 212.9866 (M-H), Calc.: 292.9858; m.p. = 219-221 °C (Degrades);

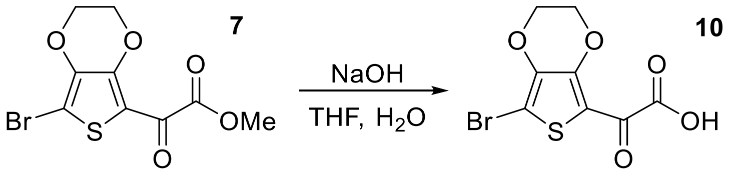

Sodium hydroxide (2 M, 15 mL, 30 mmol) was added to a solution of bromo-methoxy-EDOT **7** (2.8 g, 9.3 mmol) in THF (50 mL) and the mixture stirred for 2 hrs. The THF was then removed *in vacuo* and the residue acidified with hydrochloric acid (1 M). The resultant precipitate was collected by filtration, washed with water (2 x 50 mL) and dried *in vacuo* to give the DP as a yellow-green solid. A yield of 1.96 g, 6.7 mmol (72 %) was obtained. ^1H NMR (400 MHz, MeOD): δ = 4.40-4.45 (2H, m, ArC4-OCH_2-), 4.34-4.40 (2H, m, ArC3-OCH_2-) ppm; ^{13}C NMR (100 MHz, MeOD): δ = 175.42 (-COCO_2H), 164.33 (-CO_2H), 148.66 (ArC4), 140.65 (ArC3), 112.53 (ArC2), 103.07 (ArC5), 65.56 (ArC4-OCH_2-), 64.44 (ArC3-OCH_2-) ppm; IR ($υ_{max}$, solid): 3257, 2949, 1749, 1615, 1479, 1451, 1438, 1372, 1357, 1336, 1269, 1167, 1083, 1005 cm^{-1}; HRMS *m/z* (ESI-): Found: 290.8956/292.8888 (M-H), Calc.: 290.8956/292.8948; m.p. = 222-227 °C;

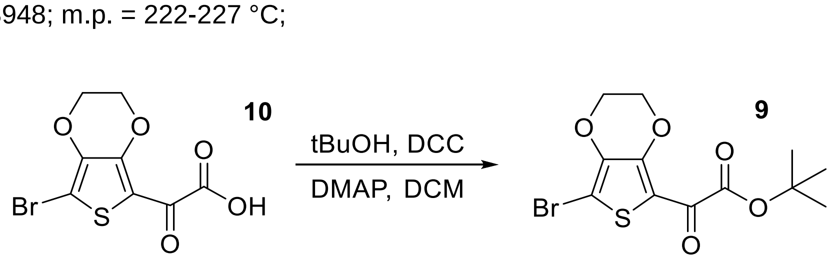

N,N'-Dicyclohexylcarbodiimide (2.12 g, 10.3 mmol) and 4-dimethylaminopyridine (83 mg, 0.68 mmol) were added to a suspension of carboxy-EDOT **10** (2 g, 6.8 mmol) in DCM (100 mL) and *tert*-butanol (5 mL). The mixture initially solubilised and then a white precipitate gradually formed. After stirring for 18 hrs the reaction was filtered and concentrated *in vacuo*. The residue was purified by flash column

chromatography eluting with 20-30 % EtOAc:Hexane. Pure fractions were concentrated *in vacuo* to give the DP as a yellow oil which solidified on standing. A yield of 1.9 g, 5.5 mmol (81 %) was obtained. Spectroscopic data were consistent with those reported above.

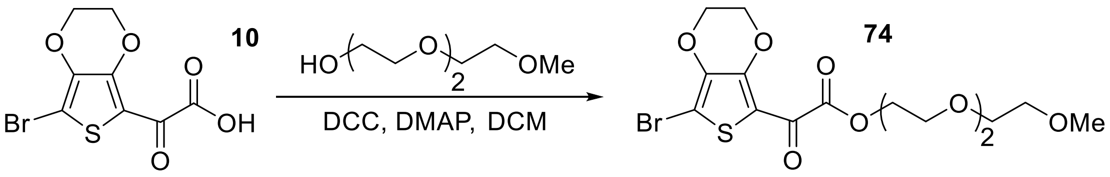

N,N'-Dicyclohexylcarbodiimide (2.32 g, 11.2 mmol) and 4-dimethylaminopyridine (92 mg, 0.75 mmol) were added to a suspension of carboxy-EDOT **10** (2.2 g, 7.5 mmol) in DCM (100 mL). The mixture initially solubilised and then a white precipitate gradually formed. After stirring for 18 hrs the reaction was filtered and concentrated *in vacuo*. The residue was purified by flash column chromatography eluting with 40-90 % EtOAc:Hexane. Product containing fractions were concentrated *in vacuo* and the residue triturated in water (3 x 70 mL), dissolved in DCM, dried with $MgSO_4$, filtered and concentrated *in vacuo* to give the DP as a yellow oil. A yield of 1.7 g, 3.88 mmol (52 %) was obtained. ^{1}H NMR (400 MHz, $CDCl_3$): δ = 4.45-4.49 (2H, m, TEG), 4.39-4.43 (2H, m, ArC4-OC<u>H</u>$_2$-), 4.32-4.37 (2H, m, ArC3-OC<u>H</u>$_2$-), 3.80-3.84 (2H, m, TEG), 3.67-3.72 (2H, m, TEG), 3.62-3.67 (4H, m, TEG), 3.52-3.56 (2H, m, TEG), 3.38 (3H, s, -O<u>Me</u>) ppm; ^{13}C NMR (100 MHz, $CDCl_3$): δ = 173.65 (-<u>C</u>OCO$_2$R), 162.59 (-<u>C</u>O$_2$R), 148.19 (Ar<u>C</u>4), 140.31 (Ar<u>C</u>3), 113.54 (Ar<u>C</u>5), 104.08 (Ar<u>C</u>2), 71.91 (TEG), 70.74 (TEG), 70.63 (TEG), 70.58 (TEG), 68.51 (TEG), 65.59 (ArC4-O<u>C</u>H$_2$-), 65.55 (TEG), 64.48 (ArC3-O<u>C</u>H$_2$-), 59.02 (-O<u>Me</u>) ppm; IR ($υ_{max}$, film): 2876, 1728, 1642, 1494, 1479, 1427, 1355, 1314, 1245, 1215, 1124, 1101, 1084, 1028 cm^{-1}; HRMS *m/z* (ESI+): Found: 460.9871/462.9862 (M+Na), Calc.: 460.9882/462.9861;

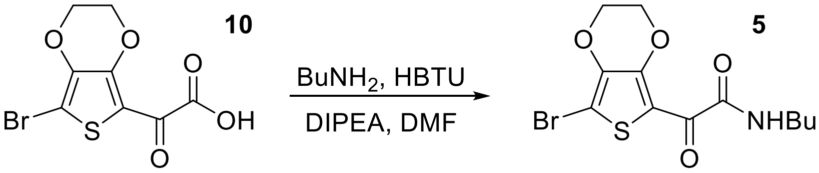

Bromo-carboxy-EDOT **10** (29 mg, 100 µmol) and HBTU (57 mg, 150 µmol) were dissolved in DMF (0.5 mL). *n*-Butylamine (12 µL, 120 µmol) and DIPEA (35 µL, 200 µmol) were added and the mixture stirred for 18 hrs. The mixture was then diluted with DCM (50 mL) and the organics washed with sat. $NaHCO_3$ (30 mL) and hydrochloric acid (1 M, 30 mL), dried with $MgSO_4$, filtered and concentrated *in vacuo*. The residue was purified by flash column chromatography eluting with 30-50 % EtOAc:Hexane. Pure

fractions were concentrated *in vacuo* to give the DP as a yellow solid. A yield of 22 mg, 63 µmol (63 %) was obtained. Spectroscopic data were consistent with those reported above.

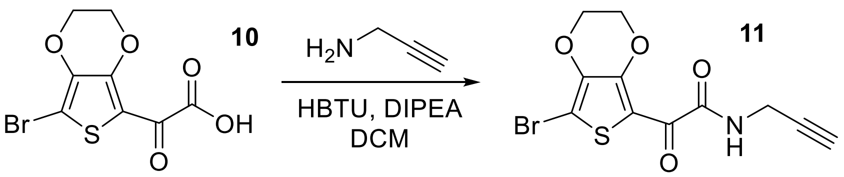

Bromo-carboxy-EDOT **10** (200 mg, 0.68 mmol) and HBTU (519 mg, 1.37 mmol) were suspended in DCM (5 mL). Propargylamine (88 µL, 1.37 mmol) and DIPEA (355 µL, 2.04 mmol) were added and the mixture stirred for 18 hrs. The mixture was then diluted with DCM (50 mL) and the organics washed with water (30 mL), dried with $MgSO_4$, filtered and concentrated *in vacuo*. The residue was purified by flash column chromatography eluting with 40-50 % EtOAc:Hexane. Pure fractions were concentrated *in vacuo* to give the DP as a yellow solid. A yield of 178 mg, 0.57 mmol (84 %) was obtained. ^{1}H NMR (400 MHz, $CDCl_3$): δ = 7.54 (1H, br s, -N<u>H</u>), 4.42-4.48 (2H, m, ArC4-OC<u>H</u>$_2$-), 4.29-4.39 (2H, m, ArC3-OC<u>H</u>$_2$-), 4.12 (2H, dd, *J* = 5.7, 2.2 Hz, -C<u>H</u>$_2$NH-), 2.30 (1H, t, *J* = 2.2 Hz, -C≡C<u>H</u>) ppm; ^{13}C NMR (100 MHz, $CDCl_3$): δ = 174.04 (-<u>C</u>OCONH-), 160.98 (-<u>C</u>ONH-), 149.66 (Ar<u>C</u>4), 140.09 (Ar<u>C</u>3), 110.55 (Ar<u>C</u>2), 107.09 (Ar<u>C</u>5), 78.08 (-<u>C</u>≡CH), 72.40 (-C≡<u>C</u>H), 65.60 (ArC4-O<u>C</u>H$_2$-), 64.35 (ArC3-O<u>C</u>H$_2$-), 29.20 (-<u>C</u>H$_2$NH-) ppm; IR ($υ_{max}$, solid): 3290, 3288, 1686, 1634, 1539, 1475, 1454, 1417, 1358, 1265, 1221, 1087 cm^{-1}; HRMS *m/z* (ESI+): Found: 329.9429/331.9406 (M+H), Calc.: 329.9430/331.9410; m.p. = 183-186 °C (Degrades);

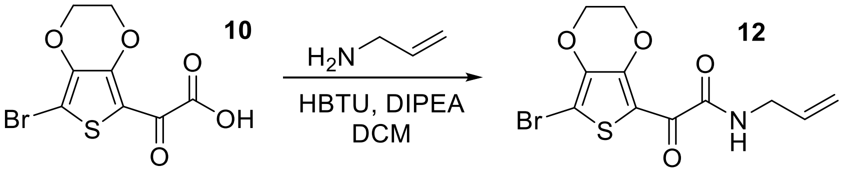

Bromo-carboxy-EDOT **10** (100 mg, 342 µmol) and HBTU (259 mg, 685 µmol) were suspended in DCM (5 mL). Allylamine (51 µL, 685 µmol) and DIPEA (238 µL, 1.37 mmol) were added and the mixture stirred for 18 hrs. The mixture was then diluted with DCM (50 mL) and the organics washed with hydrochloric acid (1 M, 30 mL), dried with $MgSO_4$, filtered and concentrated *in vacuo*. The residue was purified by flash column chromatography eluting with 20-60 % EtOAc:Hexane. Pure fractions were concentrated *in vacuo* to give the DP as a light yellow solid. A yield of 76 mg, 229 µmol (67 %) was obtained. ^{1}H NMR (400 MHz, $CDCl_3$): δ = 7.47 (1H, br s, -N<u>H</u>), 5.88 (1H, ddt, *J* = 17.2, 10.3, 5.6 Hz, -

CH=CH2), 5.27 (1H, ddt, J_1 = 17.2, J_2 = J_3 = 1.5 Hz, -CH=CH_2), 5.22 (1H, ddt, J_1 = 10.3 Hz, J_2 = J_3 = 1.5 Hz, -CH=CH_2), 4.42-4.51 (2H, m, ArC4-OCH_2-), 4.31-4.37 (2H, m, ArC3-OCH_2-), 3.99 (2H, tt, J = 5.6, 1.5 Hz, -CH_2NH-) ppm; ^{13}C NMR (100 MHz, $CDCl_3$): δ = 174.73 (-COCONH-), 161.19 (-CONH-), 149.54 (ArC4), 140.04 (ArC3), 132.76 (-CH=CH2), 117.32 (-CH=CH_2), 110.68 (ArC2), 106.99 (ArC5), 65.60 (ArC4-OCH_2-), 64.35 (ArC3-OCH_2-), 41.76 (-CH_2NH-) ppm; IR ($υ_{max}$, solid): 3010, 1683, 1626, 1477, 1417, 1386, 1354, 1258, 1246, 1227, 1141, 1085 cm^{-1}; HRMS *m/z* (ESI+): Found: 331.9601/333.9595 (M+H), Calc.: 331.9592/333.9572; m.p. = 140-143 °C;

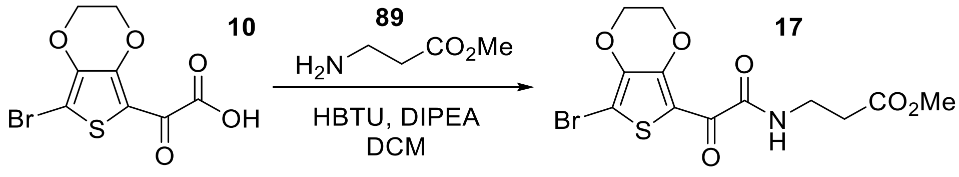

Bromo-carboxy-EDOT **10** (300 mg, 1.02 mmol) and HBTU (777 mg, 2.05 mmol) were dissolved in DCM (10 mL). Amine **89** (287 mg, 2.05 mmol) and DIPEA (889 µL, 5.10 mmol) were added and the mixture stirred for 18 hrs. The mixture was then diluted with DCM (50 mL) and the organics washed with water (30 mL), dried with $MgSO_4$, filtered and concentrated *in vacuo*. The residue was purified by flash column chromatography eluting with 40-50 % EtOAc:Hexane. Pure fractions were concentrated *in vacuo* to give the DP as a yellow solid. A yield of 194 mg, 0.51 mmol (50 %) was obtained. Spectroscopic data were consistent with those reported above.

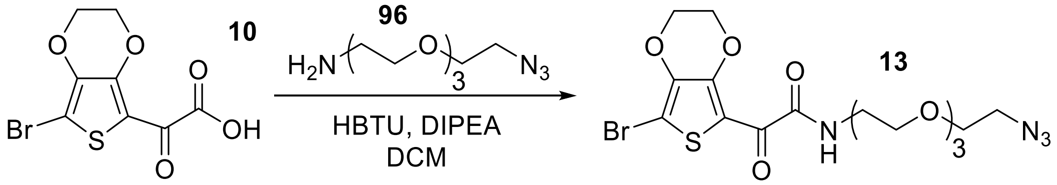

Bromo-carboxy-EDOT **10** (244 mg, 0.83 mmol) and HBTU (629 mg, 1.66 mmol) were dissolved in DCM (15 mL). Amine **96** (218 mg, 1 mmol) and DIPEA (578 µL, 3.32 mmol) were added and the mixture stirred for 5 hrs. The mixture was then diluted with DCM (50 mL) and the organics washed with water (30 mL), dried with $MgSO_4$, filtered and concentrated *in vacuo*. The residue was purified by flash column chromatography eluting with 60-90 % EtOAc:Hexane. Pure fractions were concentrated *in vacuo* to give the DP as a yellow oil. A yield of 204 mg, 0.42 mmol (50 %) was obtained. ^{1}H NMR (400 MHz, MeOD): δ = 4.41-4.45 (2H, m, ArC4-OCH_2-), 4.34-4.38 (2H, m, ArC3-OCH_2-), 3.62-3.69 (12H, m, TEG), 3.50 (2H, t, J = 5.4 Hz, -CH_2NH-), 3.40 (2H, t, J = 5.5 Hz, -CH_2N_3) ppm; ^{13}C NMR (100 MHz, MeOD): δ

=175.30 (-COCONHR), 162.88 (-CONHR), 149.55 (ArC4), 140.40 (ArC3), 110.63 (ArC5), 104.69 (ArC2), 70.14 (TEG), 70.09 (TEG), 70.02 (TEG), 69.88 (TEG), 69.62 (TEG), 68.84 (TEG), 65.49 (ArC4-OCH_2-), 64.35 (TEG), 50.37 (-CH_2N_3), 38.88 (-CH_2NH-) ppm; IR (υ_{max}, oil): 2926, 2875, 2102, 1682, 1638, 1478, 1443, 1419, 1357, 1238, 1107, 1080, 1067 cm^{-1}; HRMS *m/z* (ESI+): Found: 514.9980/516.9934 (M+Na), Calc.: 515.0206/517.0186;

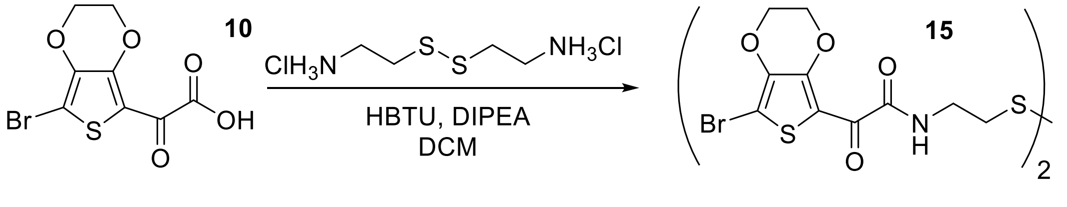

Bromo-carboxy-EDOT **10** (293 mg, 1 mmol) and HBTU (758 mg, 2 mmol) were dissolved in DCM (20 mL). Cystamine dihydrochloride (113g, 0.5 mmol) and DIPEA (872 µL, 5 mmol) were added and the mixture stirred for 3 hrs. The mixture was then diluted with DCM (10 mL) and the organics washed with water (30 mL), dried with $MgSO_4$, filtered and concentrated onto silica. The residue was purified by flash column chromatography eluting with 50-100 % EtOAc:Hexane. Pure fractions were concentrated *in vacuo* to give the DP as a yellow solid. A yield of 253 mg, 0.36 mmol (72 %) was obtained. ^{1}H NMR (400 MHz, DMSO-d_6): δ = 9.03 (2H, t, *J* = 5.9 Hz, -NH), 4.39-4.38 (4H, m, ArC4-OCH_2-), 4.29-4.39 (4H, m, ArC4-OCH_2-), 3.48 (4H, td, *J* = 6.8, 5.9 Hz, -CH_2NH-), 2.89 (4H, t, *J* = 6.8 Hz, -CH_2S-) ppm; ^{13}C NMR (100 MHz, DMSO-d_6): δ = 175.44 (-COCONR_2), 162.44 (-CONR_2), 149.74 (ArC4), 140.67 (ArC3), 110.45 (ArC2), 104.07 (ArC5), 65.77 (ArC4-OCH_2-), 64.70 (ArC3-OCH_2-), 38.62 (-CH_2NH-), 36.89 (-CH_2S-) ppm; IR (υ_{max}, solid): 2994, 1682, 1637,1626, 1528, 1477, 1418, 1350, 1279, 1244, 1168, 1142, 1087 cm^{-1}; HRMS *m/z* (ESI+): Found: 702.8389 (M+H), Calc.: 702.8371; m.p. = 232-235 °C;

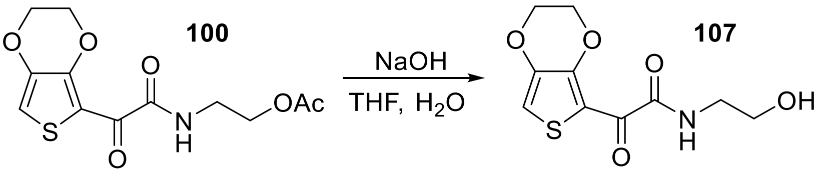

Sodium hydroxide (18 mL, 36 mmol) was added to a solution of acetoxy-EDOT **100** (2.7 g, 9 mmol) in THF (50 mL) and the mixture stirred for 2 hr. The THF was then removed *in vacuo* and the mixture diluted with DCM (100 mL). The organics were washed with hydrochlodic acid (1 M, 50 mL), dried with $MgSO_4$, filtered and concentrated *in vacuo*. The residue was purified by flash column chromatography eluting with 70-100 % EtOAc:Hexane. Pure fractions were concentrated *in vacuo* to give the DP as a

light yellow solid. A yield of 758 mg, 2.9 mmol (33 %) was obtained. ^{1}H NMR (400 MHz, DMSO-d$_6$): δ = 8.67 (1H, br t, *J* = 5.9 Hz, -NH), 7.25 (1H, s, ArH5), 4.75 (1H, t, *J* = 5.4 Hz -OH), 4.34-4.41 (2H, m, ArC4-OCH$_2$-), 4.22-4.31 (2H, m, ArC3-OCH$_2$-), 3.48 (2H, dt, *J* = 5.9, 5.7 Hz, -CH$_2$OH), 3.24 (2H, td, *J* = 5.7, 5.4 Hz, -NHCH$_2$-) ppm; ^{13}C NMR (100 MHz, DMSO-d$_6$): δ = 177.75 (-COCONH-), 163.21 (-CONH-), 149.86 (ArC4), 141.85 (ArC3), 114.46 (ArC5), 111.27 (ArC2), 65.69 (ArC4-OCH$_2$-), 64.18 (ArC3-OCH$_2$-), 59.69 (-CH$_2$OH), 41.95 (-NHCH$_2$-) ppm; IR (υ_{max}, solid): 3491, 3330, 1672, 1631, 1618, 1533, 1472, 1446, 1418, 1370, 1359, 1176, 1075, 1065, 1052, 1033 cm^{-1}; HRMS *m/z* (ESI+): Found: 258.0435 (M+H), Calc.: 258.0436; m.p. = 152-157 °C;

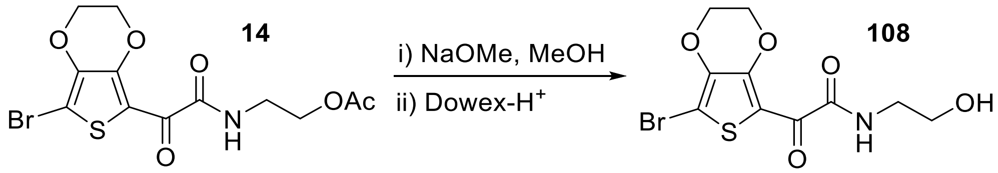

Sodium methoxide (1.14 g, 21.1 mmol) was added to a solution of Bromo-acetoxy-EDOT **14** (2 g, 5.3 mmol) in methanol (50 mL) and the mixture stirred for 1 hr, during which time a yellow precipitate formed. The reaction was neutralised with pre-activated Dowex-50WX8 and stirred for 10 min. The mixture was then filtered and the residue washed extensively with DCM (~ 150 mL). The filtrate was then concentrated *in vacuo* to give the DP as a yellow solid. A yield of 1.1 g, 3.3 mmol (62 %) was obtained. ^{1}H NMR (400 MHz, DMSO-d$_6$): δ = 8.76 (1H, br t, *J* = 6.0 Hz, -NH), 4.75 (1H, br s, -OH), 4.39-4.46 (2H, m, ArC4-OCH$_2$-), 4.32-4.39 (2H, m, ArC3-OCH$_2$-), 3.47 (2H, t, *J* = 6.1 Hz, -CH$_2$OH), 3.23 (2H, td, *J* = 6.1, 6.0 Hz, -NHCH$_2$-) ppm; ^{13}C NMR (100 MHz, DMSO-d$_6$): δ = 175.73 (-COCONH-), 162.49 (-CONH-), 149.70 (ArC4), 140.71 (ArC3), 110.45 (ArC2), 103.93 (ArC5), 65.77 (ArC4-OCH$_2$-), 64.72 (ArC3-OCH$_2$-), 59.58 (-CH$_2$OH), 42.10 (-NHCH$_2$-) ppm; IR (υ_{max}, solid): 2931, 2860, 1607, 1467, 1422, 1358, 1307, 1246, 1216, 1066 cm^{-1}; HRMS *m/z* (ESI+): Found: 335.9552/337.9520 (M+H), Calc.: 335.9541/337.9521; m.p. = 161-167 °C;

Chain extension protocol

Brominated monomer (1 mmol), pivalic acid (0.5 mmol), palladium (II) acetate (0.05 mmol) and potassium carbonate (10 mmol) were charged under nitrogen. Dry DMF (2 mL) and EDOT (4 mmol) were then added and the mixture heated to 90 ºC for 2 hrs. After cooling to rt the mixture was diluted with DCM (50 mL) and washed with water (2 x 50 mL) and brine (50 mL). The organics were dried with

$MgSO_4$, filtered and concentrated *in vacuo*. The residue was purified by flash column chromatography and pure fractions were concentrated *in vacuo*.

Numbering system for monofunctional-oligomer NMR assignments

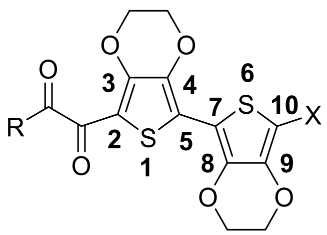

Chain extension- Dimers

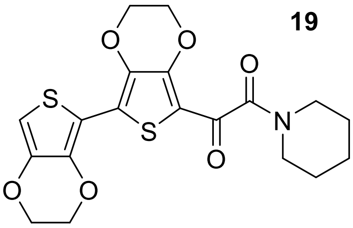

19 Run on 2.78 mmol scale at 130 ºC. Column eluted with 2 % NEt_3 in 50-90 % EtOAc:Hexane. A yield of 470 mg, 1.11 mmol (40 %) was obtained as a yellow solid. ^1H NMR (400 MHz, $CDCl_3$): δ = 6.48 (1H, s, ArH10), 4.36-4.45 (6H, m, -OC<u>H</u>$_2$-), 4.24-4.31 (2H, m, -OC<u>H</u>$_2$-), 3.65 (2H, t, *J* = 5.2 Hz, -NC<u>H</u>$_2$-), 3.39 (2H, t, *J* = 5.2 Hz, -NC<u>H</u>$_2$-), 1.53-1.76 (6H, m, -C<u>H</u>$_2$C<u>H</u>$_2$CH$_2$N-) ppm; ^{13}C NMR (100 MHz, $CDCl_3$): δ = 181.76 (-<u>C</u>OCON-), 165.52 (-<u>C</u>ON-), 146.55 (Ar<u>C</u>$_β$), 141.37 (Ar<u>C</u>$_β$), 140.30 (Ar<u>C</u>9), 136.11 (Ar<u>C</u>$_β$), 122.92 (Ar<u>C</u>$_α$), 112.24 (Ar<u>C</u>$_α$), 108.99 (Ar<u>C</u>7), 101.87 (Ar<u>C</u>10), 65.50 (-O<u>C</u>H$_2$-), 65.28 (-O<u>C</u>H$_2$-), 64.53 (-O<u>C</u>H$_2$-), 64.48 (-O<u>C</u>H$_2$-), 47.04 (-<u>C</u>H$_2$N-), 42.19 (-<u>C</u>H$_2$N-), 26.06 (-<u>C</u>H$_2$CH$_2$N-), 25.27 (-<u>C</u>H$_2$CH$_2$N-), 24.55 (-<u>C</u>H$_2$CH$_2$CH$_2$N-) ppm; IR ($υ_{max}$, solid): 3305, 2931, 2867, 1659, 1637, 1626, 1603, 1556, 1467, 1452, 1440, 1427, 1358, 1265, 1250, 1224, 1086, 1038 cm^{-1}; HRMS *m/z* (ESI+): Found: 422.0730 (M+H), Calc.: 422.0732; m.p. = 276-277 °C;

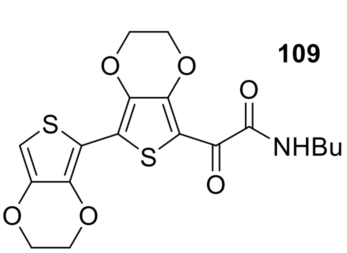

109 Run on 0.6 mmol scale. Column eluted with 2 % NEt_3 in 40-70 % EtOAc:Hexane. A yield of 158 mg, 0.39 mmol (67 %) was obtained as a yellow solid. ^1H NMR (400 MHz, $CDCl_3$): δ = 7.47 (1H, br t, *J* = 6.9 Hz, -N<u>H</u>), 6.49 (1H, s, Ar<u>H</u>10), 4.47-4.56 (2H, m, -OC<u>H</u>$_2$-), 4.35-4.47 (4H, m, -OC<u>H</u>$_2$-), 4.20-4.33 (2H, m, -OC<u>H</u>$_2$-), 3.39 (2H, dt, J_1 = J_2 = 6.9 Hz, -NHC<u>H</u>$_2$-), 1.52-1.66 (2H, m, -NHCH$_2$C<u>H</u>$_2$-), 1.32-1.47 (2H, m, -C<u>H</u>$_2$CH$_3$), 0.95 (3H, t, *J* = 7.3 Hz, -C<u>H</u>$_3$) ppm; ^{13}C NMR (100 MHz, $CDCl_3$): δ = 175.33 (-<u>C</u>OCONH-), 162.03 (-<u>C</u>ONH-), 149.94 (Ar<u>C</u>$_β$), 141.40 (Ar<u>C</u>$_β$), 140.74 (Ar<u>C</u>$_β$), 136.21 (Ar<u>C</u>$_β$), 126.41 (Ar<u>C</u>$_α$), 109.35 (Ar<u>C</u>$_α$), 108.14 (Ar<u>C</u>$_α$), 102.26 (Ar<u>C</u>10), 65.59 (-O<u>C</u>H$_2$-), 65.32 (-O<u>C</u>H$_2$-), 64.53 (-O<u>C</u>H$_2$-), 64.33 (-O<u>C</u>H$_2$-), 39.15 (-NH<u>C</u>H$_2$-), 31.35 (-NHCH$_2$<u>C</u>H$_2$-), 20.07 (-<u>C</u>H$_2$CH$_3$),

13.72 (-CH_3) ppm; IR (υ_{max}, solid): 3280, 2959, 2932, 2875, 2850, 1674, 1622, 1490, 1469, 1455, 1441, 1424, 1364, 1300, 1262, 1236, 1170, 1085, 1058, 1045 cm^{-1}; HRMS *m/z* (ESI+): Found: 410.0732 (M+H), Calc.: 410.0732; m.p. = 186-187 °C;

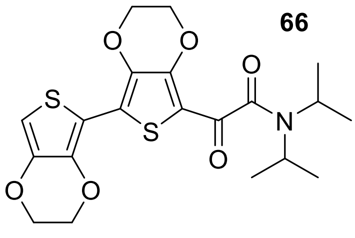
66

Run on 2.6 mmol scale. Column eluted with 2 % NEt_3 in 60 % EtOAc:Hexane. A yield of 480 mg, 1.09 mmol (41 %) was obtained as a yellow solid. ^{1}H NMR (400 MHz, $CDCl_3$): δ = 6.46 (1H, s, ArH10), 4.32-4.42 (6H, m, -OCH_2-), 4.23-4.30 (2H, m, -OCH_2-), 3.85 (1H, sept, *J* = 6.6 Hz, -CHMe$_2$), 3.53 (1H, sept, *J* = 6.8 Hz, -CHMe$_2$), 1.52 (6H, d, *J* = 6.8 Hz, -CHMe$_2$), 1.19 (6H, d, *J* = 6.6 Hz, -CHMe$_2$) ppm; ^{13}C NMR (100 MHz, $CDCl_3$): δ = 181.69 (-COCON-), 166.68 (-CON-), 146.19 (ArC$_\beta$), 141.35 (ArC$_\beta$), 140.15 (ArC9), 135.98 (ArC$_\beta$), 122.43 (ArC$_\alpha$), 112.40 (ArC$_\alpha$), 109.04 (ArC7), 101.64 (ArC10), 65.25 (-OCH_2-), 65.09 (-OCH_2-), 64.53 (-OCH_2-), 64.47 (-OCH_2-), 50.24 (-CHMe$_2$), 45.70 (-CHMe$_2$), 20.57 (-CHMe$_2$), 20.06 (-CHMe$_2$) ppm; IR (υ_{max}, solid): 2926, 2853, 1691, 1647, 1607, 1544, 1467, 1433, 1359, 1202, 1171, 1130 cm^{-1}; HRMS *m/z* (ESI+): Found: 438.1053 (M+H), Calc.: 438.1045; m.p. = 265-267 °C;

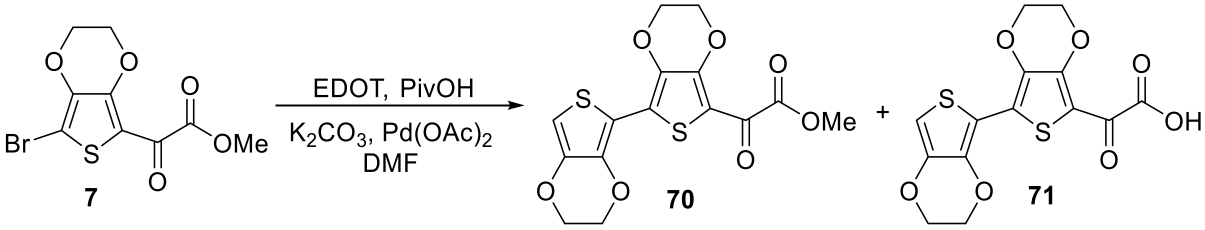

Run on 0.3 mmol scale. Analysis by LC-MS indicated that while complete chain extension of **7** had occurred, a significant degree of ester cleavage had occurred to generate a mixture of **70** and **71** (~1.5:1 ratio). Subsequent experiments were therefore undertaken with alternative esters which did not undergo cleavage under the reaction conditions.

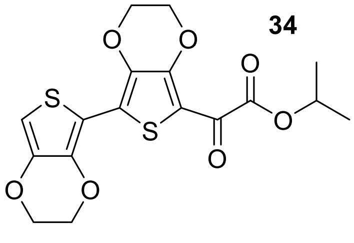
34

Run on 0.30 mmol scale. Column eluted with 40-60 % EtOAc:Hexane. A yield of 80 mg, 0.20 mmol (66 %) was obtained as a yellow oil. If the reaction was undertaken at 130 °C, ester cleavage was observed as described above for methyl ester **3**. ^{1}H NMR (400 MHz, DMSO-d_6): δ = 6.90 (1H, s, ArH10), 5.15 (1H, sept, *J* = 6.5 Hz, -CHMe$_2$), 4.37-4.60 (6H, m, -OCH_2-), 4.20-4.37 (2H, m, -OCH_2-), 1.31 (6H, d, *J* = 6.5 Hz, -CHMe$_2$) ppm; ^{13}C NMR (400 MHz, DMSO-d_6): δ = 176.57 (-COCO$_2$*i*Pr), 163.99 (-CO$_2$*i*Pr), 148.67 (ArC$_\beta$), 141.66 (ArC9), 141.19 (ArC$_\beta$), 136.53 (ArC$_\beta$), 122.87

(ArC$_{\alpha}$), 109.63 (ArC$_{\alpha}$), 108.08 (ArC7), 103.59 (ArC10), 70.58 (-CHMe$_2$), 66.07 (-OCH$_2$-), 65.14 (-OCH$_2$-), 64.71 (-OCH$_2$-), 21.74 (-CHMe$_2$) ppm; IR (υ_{max}, solid): 3345, 2980, 2939, 2880, 1731, 1704, 1646, 1637, 1489, 1468, 1438, 1357, 1312, 1263, 1244, 1216, 1175, 1104, 1057, 1028, 1010 cm^{-1}; HRMS *m/z* (ESI+): Found: 397.0435 (M+H), Calc.: 397.0416; m.p. = 168-172 °C;

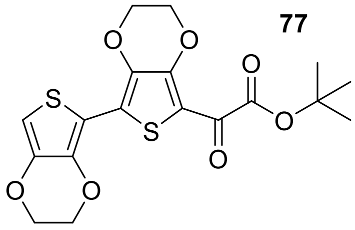

77

Run on 0.57 mmol scale. Column eluted with 2 % NEt_3 in 50-70 % EtOAc:Hexane. A yield of 190 mg, 0.46 mmol (81 %) was obtained as a yellow solid. ^{1}H NMR (400 MHz, DMSO-d$_6$): δ = 6.90 (1H, s, ArH10), 4.40-4.50 (6H, m, -OCH$_2$-), 4.25-4.32 (2H, m, -OCH$_2$-), 1.54 (9H, s, -O*t*Bu) ppm; ^{13}C NMR (100 MHz, $CDCl_3$): δ = 176.87 (-COCO$_2$*t*Bu), 163.75 (-CO$_2$*t*Bu), 148.40 (ArC$_{\beta}$), 141.66 (ArC$_{\beta}$), 141.09 (ArC9), 136.50 (ArC$_{\beta}$), 122.52 (ArC$_{\alpha}$), 109.69 (ArC$_{\alpha}$), 108.10 (ArC7), 103.44 (ArC10), 84.39 (-CMe$_3$), 66.05 (-OCH$_2$-), 65.97 (-OCH$_2$-), 65.15 (-OCH$_2$-), 64.71 (-OCH$_2$-), 27.97 (-CMe$_3$) ppm; IR (υ_{max}, solid): 2935, 1714, 1611, 1506, 1466, 1441, 1361, 1270, 1247, 1228, 1159, 1140, 1118, 1095, 1062 cm^{-1}; HRMS *m/z* (ESI+): Found: 411.0577 (M+H), Calc.: 411.0572; m.p. = 188-191 °C;

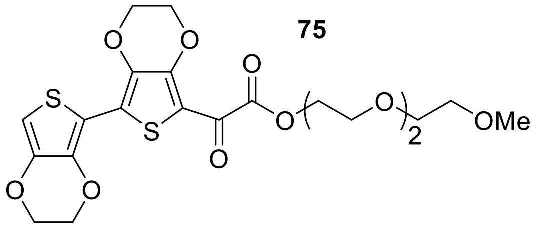

75

Run on 2.28 mmol scale. Column eluted with 2 % NEt_3 in 60-100 % EtOAc:Hexane. A yield of 270 mg, 0.59 mmol (26 %) was obtained as a yellow oil which solidified on standing. ^{1}H NMR (400 MHz, $CDCl_3$): δ = 6.48 (1H, s, ArH10), 4.43-4.48 (2H, m, TEG), 4.34-4.43 (6H, m, -OCH$_2$-), 4.19-4.31 (2H, m, -OCH$_2$-), 3.75-3.85 (2H, m, TEG), 3.66-3.71 (2H, m, TEG), 3.60-3.66 (4H, m, TEG), 3.46-3.58 (2H, m, TEG), 3.35 (3H, s, -OMe) ppm; ^{13}C NMR (400 MHz, $CDCl_3$): δ = 174.73 (-COCO$_2$R), 163.67 (-CO$_2$R), 148.30 (ArC$_{\beta}$), 141.36 (ArC$_{\beta}$), 140.58 (ArC$_{\beta}$), 136.13 (ArC$_{\beta}$), 124.17 (ArC$_{\alpha}$), 110.42 (ArC$_{\alpha}$), 108.97 (ArC$_{\alpha}$), 102.34 (ArC10), 71.58 (TEG), 70.74 (TEG), 70.59 (TEG), 70.55 (TEG), 68.60 (TEG), 65.59 (-OCH$_2$-), 65.35 (-OCH$_2$-), 65.20 (-OCH$_2$-), 64.49 (-OCH$_2$-/TEG), 64.48 (-OCH$_2$-/TEG), 58.98 (-OMe) ppm; IR (υ_{max}, oil): 2920, 2876, 1724, 1623, 1612, 1472, 1441, 1359, 1262, 1245, 1215, 1118, 1089, 1062, 1037, 1013 cm^{-1}; HRMS *m/z* (ESI+): Found: 501.0897 (M+H), Calc.: 501.0889; m.p. = 80-81 °C;

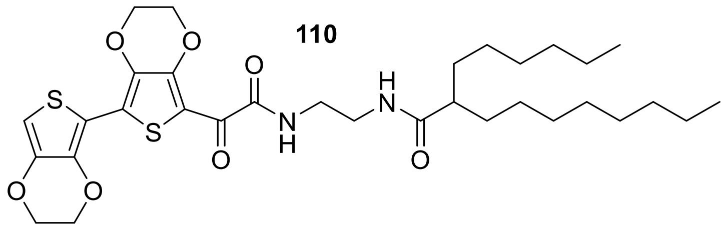

110

Run on 2.02 mmol scale. Column eluted with 2 % NEt_3 in 60-90 % EtOAc:Hexane. A yield of 290 mg, 0.46 mmol (23 %) was obtained as a yellow solid. ^{1}H NMR (400 MHz, $CDCl_3$): δ = 7.78 (1H, br t, *J* = 6.0 Hz, -N<u>H</u>COCO-), 6.52 (1H, s, Ar<u>H</u>10), 5.97 (1H, br t, *J* = 5.2 Hz, -N<u>H</u>COAlk), 4.48-4.52 (2H, m, -OC<u>H</u>$_2$-), 4.38-4.46 (2H, m, -OC<u>H</u>$_2$-), 4.26-4.31 (2H, m, -OC<u>H</u>$_2$-), 3.49-3.59 (4H, m, -NHC<u>H</u>$_2$C<u>H</u>$_2$NH-), 1.95-2.05 (1H, m, <u>H</u>$_α$), 1.52-1.64 (2H, m, <u>H</u>$_β$), 1.34-1.46 (2H, m, <u>H</u>$_β$), 1.16-1.31 (20H, m, <u>Alkyl</u>), 0.82-0.90 (6H, m, -C<u>H</u>$_3$) ppm; ^{13}C NMR (100 MHz, $CDCl_3$): δ = 176.79 (-NH<u>C</u>OAlkyl) 174.56 (-<u>C</u>OCONH-), 163.09 (-CO<u>C</u>ONH-), 150.13 (Ar<u>C</u>$_β$), 141.40 (Ar<u>C</u>$_β$), 140.78 (Ar<u>C</u>$_β$), 136.27 (Ar<u>C</u>$_β$), 109.33 (Ar<u>C</u>$_α$), 107.97 (Ar<u>C</u>$_α$), 102.49 (Ar<u>C</u>10), 65.58 (-O<u>C</u>H$_2$-), 65.35 (-O<u>C</u>H$_2$-), 64.52 (-O<u>C</u>H$_2$-), 64.33 (-O<u>C</u>H$_2$-), 48.12 (<u>C</u>$_α$), 39.35 (-NH<u>C</u>H$_2$-), 39.32 (-NH<u>C</u>H$_2$-), 33.02 (<u>C</u>$_β$), 31.86 (<u>Alkyl</u>), 31.68 (<u>Alkyl</u>), 29.71 (<u>Alkyl</u>), 29.46 (<u>Alkyl</u>), 29.34 (<u>Alkyl</u>), 27.71 (<u>Alkyl</u>), 27.65 (<u>Alkyl</u>), 22.65 (<u>Alkyl</u>), 14.12 (-<u>C</u>H$_3$), 14.07 (-<u>C</u>H$_3$) ppm; IR ($υ_{max}$, solid): 3324, 3287, 2923, 2870, 2853, 1671, 1641, 1623, 1471, 1440, 1428, 1369, 1251, 1207, 1181, 1095, 1064 cm^{-1}; HRMS *m/z* (ESI+): Found: 635.2820 (M+H), Calc.: 635.2825; m.p. = 228-230 °C;

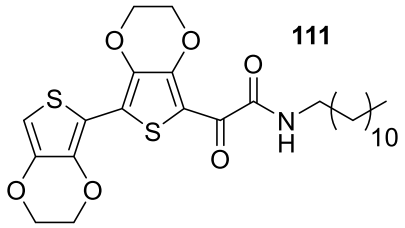

111

Run on 2.17 mmol scale. A significant amount of product was lost during work-up due to its high insolubility. The product was loaded onto silica prior to column chromatography, eluting with 2 % NEt_3 in 20-50 % EtOAc:Hexane. Yield of 210 mg, 0.40 mmol (18 %) as a yellow solid. ^{1}H NMR (400 MHz, $CDCl_3$): δ = 7.47 (1H, br t, *J* = 6.8 Hz, -N<u>H</u>), 6.47 (1H, s, Ar<u>C</u>10), 4.43-4.52 (2H, m, -OC<u>H</u>$_2$-), 4.35-4.44 (4H, m, -OC<u>H</u>$_2$-), 4.20-4.30 (2H, m, ArC3-OC<u>H</u>$_2$-), 3.35 (2H, dt, J_1 = J_2 = 6.8 Hz, -NHC<u>H</u>$_2$-), 1.52-1.62 (2H, m, -NHCH$_2$C<u>H</u>$_2$-), 1.23-1.40 (18H, m, <u>Alkyl</u>), 0.87 (3H, t, *J* = 6.7 Hz, -C<u>H</u>$_3$) ppm; ^{13}C NMR (100 MHz, $CDCl_3$): δ = 175.31 (-<u>C</u>OCONH-), 161.99 (-<u>C</u>ONH-), 149.92 (Ar<u>C</u>$_β$), 141.38 (Ar<u>C</u>$_β$), 140.72 (Ar<u>C</u>$_β$), 136.19 (Ar<u>C</u>$_β$), 126.38 (Ar<u>C</u>$_α$), 109.33 (Ar<u>C</u>$_α$), 108.12 (Ar<u>C</u>$_α$), 102.24 (Ar<u>C</u>10), 65.58 (-O<u>C</u>H$_2$-), 65.31 (-O<u>C</u>H$_2$-), 64.52 (-O<u>C</u>H$_2$-), 64.33 (-O<u>C</u>H$_2$-), 39.44 (-NH<u>C</u>H$_2$-), 31.90 (-NHCH$_2$<u>C</u>H$_2$-), 29.62 (<u>Alkyl</u>), 29.55 (<u>Alkyl</u>), 29.51 (<u>Alkyl</u>), 29.34 (<u>Alkyl</u>), 29.31 (<u>Alkyl</u>), 26.90 (<u>Alkyl</u>), 22.68 (<u>Alkyl</u>), 14.13 (-<u>C</u>H$_3$) ppm; IR ($υ_{max}$, solid): 2919, 2850, 1677, 1624, 1493, 1469, 1432, 1364, 1301, 1171, 1090, 1058 cm^{-1}; HRMS *m/z* (ESI+): Found: 522.1988 (M+H), Calc.: 522.1984; m.p. = 180-183 °C;

Dimer bromination

Bromination procedure A: Di-EDOT derivative (1 mmol) was dissolved in a mixture of THF (10 mL) and acetic acid (2 mL). If solubility was poor a further 25 mL of THF was added. The mixture was placed in the dark and *N*-bromosuccinimide (1.2 mmol) was added. After stirring for 2 hrs the mixture was poured into water (50 mL) causing precipitation of the product. The solid was collected by filtration or centrifugation (5000 rpm, 10 min), washed with water (50 mL) and then dissolved in DCM. The organics were dried with $MgSO_4$, filtered and concentrated *in vacuo*. Column chromatography was then undertaken if required.

Bromination procedure B: Reaction procedure A was followed. After pouring into water the product was extracted with DCM (200 mL). The organics were washed with sat. $NaHCO_3$ (2 x 100 mL), dried with $MgSO_4$, filtered and concentrated *in vacuo*. Column chromatography was then undertaken if required.

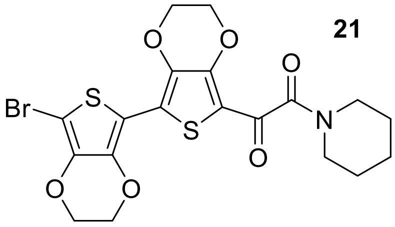

21

Method B on 0.48 mmol scale. The product was sufficiently pure for further applications. An analytical sample was obtained via column chromatography eluting with 2 % NEt_3 in 50-90 % EtOAc:Hexane. Yield of 220 mg, 0.44 mmol (92 %) as a dark green-yellow solid. ^{1}H NMR (400 MHz, $CDCl_3$): δ = 4.31-4.45 (8H, m, -OC$\underline{H}_2$-), 3.65 (2H, t, *J* = 5.2 Hz, -C$\underline{H}_2$N-), 3.39 (2H, t, *J* = 5.3 Hz, -C$\underline{H}_2$N-), 1.62-1.74 (4H, m, -C$\underline{H}_2$C$\underline{H}_2$CH$_2$N-), 1.53-1.61 (2H, m, -C$\underline{H}_2$CH$_2$N-) ppm; ^{13}C NMR (100 MHz, $CDCl_3$): δ = 181.73 (-$\underline{C}$OCON-), 165.40 (-$\underline{C}$ON-), 146.41 (Ar$\underline{C}_\beta$), 139.76 (Ar$\underline{C}_\beta$), 139.25 (Ar$\underline{C}_\beta$), 136.23 (Ar$\underline{C}_\beta$), 121.91 (Ar$\underline{C}_\alpha$), 112.48 (Ar$\underline{C}_\alpha$), 109.25 (Ar$\underline{C}_\alpha$), 90.81 (Ar$\underline{C}$10), 65.51 (-O$\underline{C}$H$_2$-), 65.22 (-O$\underline{C}$H$_2$-), 64.98 (-O$\underline{C}$H$_2$-), 64.57 (-O$\underline{C}$H$_2$-), 47.04 (-$\underline{C}$H$_2$N-), 42.22 (-$\underline{C}$H$_2$N-), 26.08 (-$\underline{C}$H$_2$$\underline{C}H_2CH_2$N-), 25.27 (-$\underline{C}H_2$$\underline{C}H_2CH_2$N-), 24.54 (-$\underline{C}H_2$$\underline{C}H_2CH_2$N-) ppm; IR ($\upsilon_{max}$, solid): 2978, 2929, 2858, 1645, 1601, 1509, 1472, 1445, 1358, 1315, 1257, 1250, 1222, 1122, 1075 cm^{-1}; HRMS *m/z* (ESI+): Found: 499.9841/501.9814 (M+H), Calc.: 499.9837/501.9816; m.p. = 238-240 °C;

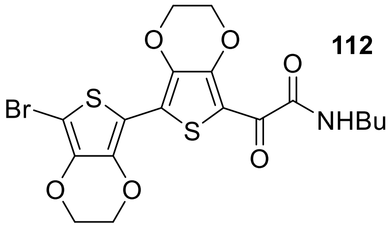

112

Method A on 2.44 mmol scale. A yield of 854 mg, 1.75 mmol (72 %) was obtained as a dark yellow solid. ^{1}H NMR (400 MHz, $CDCl_3$): δ = 7.45 (1H, br t, *J* = 6.3 Hz, -N$\underline{H}$), 4.48-4.53 (2H, m, -OC$\underline{H}_2$-), 4.38-4.46 (4H, m, -OC$\underline{H}_2$-), 4.33-4.38 (2H, m, -OC$\underline{H}_2$-), 3.38 (2H, td, *J* = 6.3, 6.1 Hz, -C$\underline{H}_2$NH-), 1.54-1.66 (2H, m, -C$\underline{H}_2$CH$_2$NH-), 1.33-1.47 (2H, m, -C$\underline{H}_2$CH$_3$), 0.96 (3H, t, *J* =

7.3 Hz, -CH_3) ppm; ^{13}C NMR (100 MHz, $CDCl_3$): δ = 175.37 (-COCONH-), 161.96 (-CON-), 149.81 (ArC_{β}), 139.80 (ArC_{β}), 139.68 (ArC_{β}), 136.31 (ArC_{β}), 125.39 (ArC_{α}), 109.61 (ArC_{α}), 108.31 (ArC_{α}), 91.21 (ArC10), 65.59 (-OCH_2-), 65.25 (-OCH_2-), 64.99 (-OCH_2-), 64.42 (-OCH_2-), 39.17 (-CH_2NH-), 31.34 (-CH_2CH_2NH-), 20.07 (-CH_2CH_3), 13.71 (-CH_3) ppm; IR ($υ_{max}$, solid): 3383, 2951, 2927, 2867, 1674, 1625, 1494, 1467, 1440, 1426, 1355, 1296, 1072, 1057, 1029 cm^{-1}; HRMS *m/z* (ESI+): Found: 487.9843/489.9798 (M+H), Calc.: 487.9837/489.9817; m.p. = 259-267 °C (Degrades);

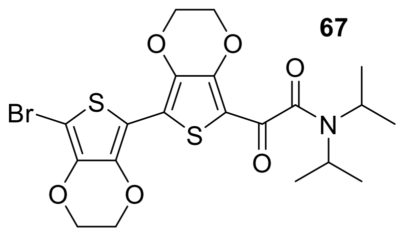
67

Method B on 0.56 mmol scale. The product was sufficiently pure for further applications. An analytical sample was obtained via column chromatography eluting with 2 % NEt_3 in 30-60 % EtOAc:Hexane. A yield of 252 mg, 0.49 mmol (87 %) was obtained as a yellow solid. ^1H NMR (400 MHz, $CDCl_3$): δ = 4.29-4.45 (8H, m, -OCH_2-), 3.85 (1H, sept, *J* = 6.5 Hz, -CHMe$_2$), 3.54 (1H, sept, *J* = 6.8 Hz, -CHMe$_2$), 1.52 (6H, d, *J* = 6.8 Hz, -CHMe_2), 1.20 (6H, d, *J* = 6.8 Hz, -CHMe_2) ppm; ^{13}C NMR (100 MHz, $CDCl_3$): δ = 181.67 (-COCON-), 166.57 (-CON-), 146.03 (ArC_{β}), 139.74 (ArC_{β}), 139.10 (ArC_{β}), 136.11 (ArC_{β}), 121.42 (ArC_{α}), 112.67 (ArC_{α}), 109.32 (ArC_{α}), 90.52 (ArC10), 65.20 (-OCH_2-), 65.10 (-OCH_2-), 65.00 (-OCH_2-), 64.57 (-OCH_2-), 50.26 (-CHMe_2), 45.74 (-CHMe_2), 20.61 (-CHMe_2), 20.07 (-CHMe_2) ppm; IR ($υ_{max}$, solid): 2971, 2933, 2872, 1711, 1637, 1606, 1470, 1438, 1362, 1289, 1270, 1238, 1209, 1179, 1162, 1115, 1079, 1045 cm^{-1}; HRMS *m/z* (ESI+): Found: 516.0.170/518.0129 (M+H), Calc.: 516.0150/518.0130; m.p. = 281-282 °C;

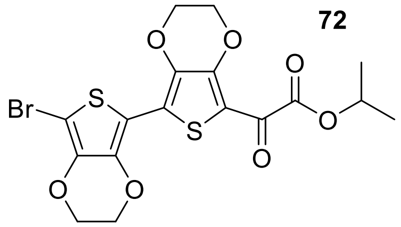
72

Method B on 0.83 mmol scale. The product was sufficiently pure for further applications. An analytical sample was obtained via column chromatography eluting with 2 % NEt_3 in 20-50 % EtOAc:Hexane. A yield of 320 mg, 0.67 mmol (81 %) was obtained as a green-yellow solid. ^1H NMR (400 MHz, $CDCl_3$): δ = 5.26 (1H, sept, *J* = 6.3 Hz, -CHMe$_2$), 4.38-4.45 (6H, m, -OCH_2-), 4.33-4.37 (2H, m, -OCH_2-), 1.40 (6H, d, *J* = 6.3 Hz, -CHMe_2) ppm; ^{13}C NMR (100 MHz, $CDCl_3$): δ = 175.60 (-$COCO_2$*i*Pr), 163.31 (-CO_2*i*Pr), 147.76 (ArC_{β}), 139.73 (ArC_{β}), 139.39 (ArC_{β}), 136.13 (ArC_{β}), 122.83 (ArC_{α}), 110.84 (ArC_{α}), 109.23 (ArC_{α}), 91.12 (ArC10), 70.40 (-CHMe_2), 65.32 (-OCH_2-), 65.22 (-OCH_2-), 64.92 (-OCH_2-), 64.48 (-OCH_2-), 21.58 (-CHMe_2) ppm; IR ($υ_{max}$, solid): 2981, 2933, 2869, 1737, 1714, 1649, 1621, 1484, 1469, 1457, 1429, 1357, 1220, 1099, 1075 cm^{-1}; HRMS *m/z* (ESI+): Found: 474.9525/476.9513 (M+H), Calc.: 474.9521/476/9500; m.p. = 178-186 °C;

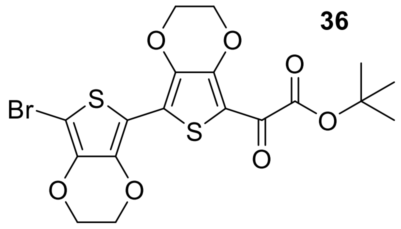

36

Method A on 1.22 mmol scale. DMF was used as solvent in place of THF/AcOH to prevent hydrolysis of the *t*-butyl group. The product was sufficiently pure for further applications. An analytical sample was obtained via column chromatography eluting with 2 % NEt_3 in 20-50 % EtOAc:Hexane. A yield of 544 mg, 1.11 mmol (92 %) was obtained as a yellow solid. ^{1}H NMR (400 MHz, $CDCl_3$): δ = 4.37-4.43 (6H, m, -OC$\underline{H}_2$-), 4.32-4.36 (2H, m, -OC$\underline{H}_2$-), 1.61 (9H, s, -$CO_2$$\underline{tBu}$) ppm; ^{13}C NMR (100 MHz, $CDCl_3$): δ = 176.36 (-$\underline{C}OCO_2$*t*Bu), 163.12 (-$\underline{C}O_2$*t*Bu), 147.47 (Ar$\underline{C}_\beta$), 139.75 (Ar$\underline{C}_\beta$), 139.31 (Ar$\underline{C}_\beta$), 136.12 (Ar$\underline{C}_\beta$), 122.41 (Ar$\underline{C}_\alpha$), 110.98 (Ar$\underline{C}_\alpha$), 109.30 (Ar$\underline{C}_\alpha$), 90.91 (Ar$\underline{C}$10), 65.27 (-O$\underline{C}H_2$-), 64.97 (-O$\underline{C}H_2$-), 64.53 (-O$\underline{C}H_2$-), 27.97 (-C$\underline{Me}_3$) ppm; IR (υ_{max}, solid): 2983, 2934, 2871, 1737, 1611, 1508, 1469, 1455, 1359, 1335, 1263, 1223, 1164, 1146, 1117, 1074 cm^{-1}; HRMS *m/z* (ESI+): Found: 488.9661/490.9648 (M+H), Calc.: 488.9657/490.9636; m.p. > 350 °C;

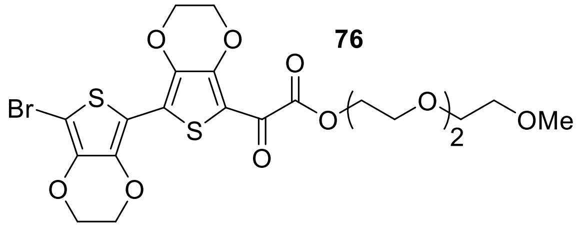

76

Method B on 0.26 mmol scale. The product was sufficiently pure for further applications. An analytical sample was obtained via column chromatography eluting with 2 % NEt_3 in 70-100 % EtOAc:Hexane. A yield of 150 mg, 0.24 mmol (93 %) was obtained as a green-yellow solid. ^{1}H NMR (400 MHz, $CDCl_3$): δ = 4.45-4.50 (2H, m, TEG), 4.36-4.45 (6H, m, -OC$\underline{H}_2$-), 4.32-4.46 (2H, m, -OC$\underline{H}_2$-), 3.79-3.85 (2H, m, TEG), 3.68-3.72 (2H, m, TEG), 3.61-3.68 (4H, m, TEG), 3.51-3.56 (2H, m, TEG), 3.36 (3H, s, -O$\underline{Me}$) ppm; ^{13}C NMR (400 MHz, $CDCl_3$): δ = 174.67 (-$\underline{C}OCO_2$R), 163.54 (-$\underline{C}O_2$R), 148.17 (Ar$\underline{C}_\beta$), 139.77 (Ar$\underline{C}_\beta$), 139.53 (Ar$\underline{C}_\beta$), 136.24 (Ar$\underline{C}_\beta$), 123.15 (Ar$\underline{C}_\alpha$), 110.62 (Ar$\underline{C}_\alpha$), 109.23 (Ar$\underline{C}_\alpha$), 91.28 (Ar$\underline{C}$10), 71.89 (TEG), 70.74 (TEG), 70.60 (TEG), 70.56 (TEG), 68.60 (TEG), 65.59 (-O$\underline{C}H_2$-), 65.30 (-O$\underline{C}H_2$-/TEG), 65.27 (-O$\underline{C}H_2$-/TEG), 64.96 (-O$\underline{C}H_2$-/TEG), 64.57 (-O$\underline{C}H_2$-), 59.01 (-O$\underline{Me}$) ppm; IR (υ_{max}, film): 2879, 2160, 1725, 1634, 1470, 1450, 1362, 1263, 1216, 1076 cm^{-1}; HRMS *m/z* (ESI+): Found: 578.9994/581.0024 (M+H), Calc.: 578.9997/580.9977; m.p. = 115-120 °C;

Chain extension-Trimers

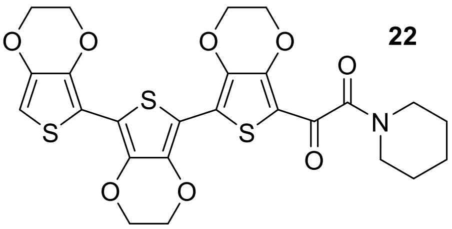

22

Run on 0.9 mmol scale at 130 ˚C. Column eluted with 2 % NEt_3 in 70-100 % EtOAc:Hexane. A yield of 284 mg, 0.51 mmol (56 %) was obtained as an orange solid. ^{1}H NMR (400 MHz, $CDCl_3$): δ = 6.37 (1H, s, Ar$\underline{H}$15), 4.36-4.49 (10H, m, -

OCH$_2$-), 4.23-4.30 (2H, m, -OCH$_2$-), 3.61-3.70 (2H, m, -CH$_2$N-), 3.36-3.40 (2H, m, -CH$_2$N-), 1.62-1.74 (4H, m, -CH$_2$CH$_2$CH$_2$-), 1.55-1.61 (2H, m, -CH$_2$CH$_2$N-) ppm; ^{13}C NMR (100 MHz, CDCl$_3$): δ = 181.43 (-COCONR$_2$), 166.64 (-CONR$_2$), 146.61 (ArC$_β$), 141.30 (ArC$_β$), 140.16 (ArC$_β$), 137.80 (ArC$_β$), 136.54 (ArC$_β$), 135.73 (ArC$_β$), 123.36 (ArC$_α$), 112.96 (ArC$_α$), 109.67 (ArC$_α$), 106.90 (ArC$_α$), 99.22 (ArC15), 65.52 (-OCH$_2$-), 65.25 (-OCH$_2$-), 64.91 (-OCH$_2$-), 64.59 (-OCH$_2$-), 47.06 (-CH$_2$N-), 42.18 (-CH$_2$N-), 26.07 (-CH$_2$CH$_2$CH$_2$N-), 25.28 (-CH$_2$CH$_2$CH$_2$N-), 24.56 (-CH$_2$CH$_2$CH$_2$N-) ppm; HRMS *m/z* (ESI+): Found: 562.0667 (M+H), Calc.: 562.0664; IR (υ$_{max}$, solid): 2924, 2862, 1650, 1607, 1510, 1466, 1433, 1313, 1254, 1223, 1176, 1121, 1101, 1077, 1059, cm^{-1}; m.p. = 319-321 °C;

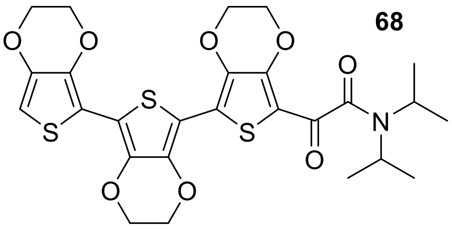

68

Run on 0.9 mmol scale. Column eluted with 2 % NEt$_3$ in 70-100 % EtOAc:Hexane. A yield of 252 mg, 0.44 mmol (48 %) was obtained as an orange solid. ^{1}H NMR (400 MHz, CDCl$_3$): δ = 6.35 (1H, s, ArH15), 4.30-4.48 (10H, m, -OCH$_2$-), 4.20-4.30 (2H, m, -OCH$_2$-), 3.85 (1H, sept, *J* = 5.7 Hz, -CHMe$_2$), 3.53 (1H, sept, *J* = 6.8 Hz, -CHMe$_2$), 1.52 (6H, d, *J* = 6.8 Hz, -CHMe$_2$), 1.20 (6H, d, *J* = 5.7 Hz, -CHMe$_2$) ppm; ^{13}C NMR (100 MHz, CDCl$_3$): δ = 181.42 (-COCONR$_2$), 166.80 (-CONR$_2$), 146.25 (ArC$_β$), 141.30 (ArC$_β$), 139.99 (ArC$_β$), 137.74 (ArC$_β$), 136.53 (ArC$_β$), 135.61 (ArC$_β$), 122.83 (ArC$_α$), 112.69 (ArC$_α$), 112.65 (ArC$_α$), 109.67 (ArC$_α$), 106.97 (ArC$_α$), 99.10 (ArC15), 65.24 (-OCH$_2$-), 65.12 (-OCH$_2$-), 65.08 (-OCH$_2$-), 64.92 (-OCH$_2$-), 64.59 (-OCH$_2$-), 50.26 (-CHMe$_2$), 45.68 (-CHMe$_2$), 20.61 (-CHMe$_2$), 20.09 (-CHMe$_2$) ppm; IR (υ$_{max}$, solid): 2973, 2932, 2872, 1634, 1614, 1509 ,1466, 1431, 1358, 1267, 1077, 1062, 1043 cm^{-1}; HRMS *m/z* (ESI+): Found: 578.0968 (M+H), Calc.: 578.0977; m.p. = 218-220 °C (Degrades);

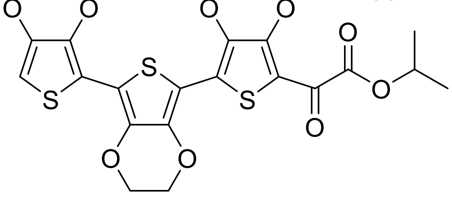

35

Run on 1.26 mmol scale. Column eluted with 2 % NEt$_3$ in 50-100 % EtOAc:Hexane. A yield of 270 mg, 0.5 mmol (40 %) was obtained as a red solid. ^{1}H NMR (400 MHz, CDCl$_3$): δ = 6.38 (1H, s, ArH15), 5.26 (1H, sept, *J* = 6.3 Hz, -CHMe$_2$), 4.37-4.50 (10H, m, -OCH$_2$-), 4.24-4.32 (2H, m, -OCH$_2$-), 1.40 (6H, d, *J* = 6.3 Hz, -CHMe$_2$) ppm; ^{13}C NMR (100 MHz, CDCl$_3$): δ = 174.97 (-COCO$_2$*i*Pr), 163.41 (-CO$_2$*i*Pr), 147.81 (ArC$_β$), 141.31 (ArC$_β$), 140.38 (ArC$_β$), 137.88 (ArC$_β$), 136.56 (ArC$_β$), 135.57 (ArC$_β$), 124.27 (ArC$_α$), 113.23 (ArC$_α$), 109.97 (ArC$_α$), 109.88 (ArC$_α$), 106.95 (ArC$_α$), 99.34 (ArC15), 70.29 (-CHMe$_2$), 65.36 (-OCH$_2$-), 65.31 (-OCH$_2$-), 65.24 (-OCH$_2$-), 64.90 (-OCH$_2$-), 64.58 (-OCH$_2$-), 64.54 (-OCH$_2$-), 21.63 (-CHMe$_2$) ppm; IR (υ$_{max}$, solid): 2926,

1721, 1645, 1606, 1508, 1465, 1432, 1397, 1360, 1319, 1252, 1217, 1175, 1083, 1061, 1014 cm^{-1}; HRMS *m/z* (ESI+): Found: 537.0359 (M+H), Calc.: 537.0350; m.p. = 267-268 °C;

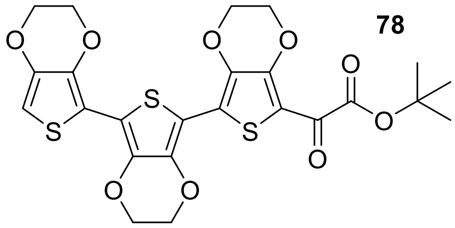

78

Run on 365 μmol scale. Column eluted with 2 % NEt_3 in 50-80 % EtOAc:Hexane. A yield of 74 mg, 135 μmol (37 %) was obtained as an orange solid. ^{1}H NMR (400 MHz, $CDCl_3$): δ = 6.37 (1H, s, ArH15), 4.36-4.49 (10H, m, $-OCH_2-$), 4.20-4.30 (2H, m, $-OCH_2-$), 1.61 (9H, s, -O*t*Bu) ppm; ^{13}C NMR (100 MHz, $CDCl_3$): δ = 176.05 ($-COCO_2$*t*Bu), 163.35 ($-CO_2$*t*Bu), 147.66 (ArC_β), 141.30 (ArC_β), 140.24 (ArC_β), 137.83 (ArC_β), 136.54 (ArC_β), 135.55 (ArC_β), 122.52 (ArC_α), 113.09 (ArC_α), 110.69 (ArC_α), 109.66 (ArC_α), 106.97 (ArC_α), 99.26 (ArC15), 83.89 ($-CMe_3$), 65.30 ($-OCH_2-$), 65.25 ($-OCH_2-$), 64.90 ($-OCH_2-$), 64.59 ($-OCH_2-$), 64.55 ($-OCH_2-$), 27.98 ($-CMe_3$) ppm; IR (υ_{max}, solid): 2978, 2931, 2873, 1720, 1607, 1509, 1430, 1360, 1252, 1222, 1162, 1145, 1123, 1083, 1025 cm^{-1}; HRMS *m/z* (ESI+): Found: 551.0491 (M+H), Calc.: 551.0504; m.p. = 214 °C (Degrades);

Trimer bromination

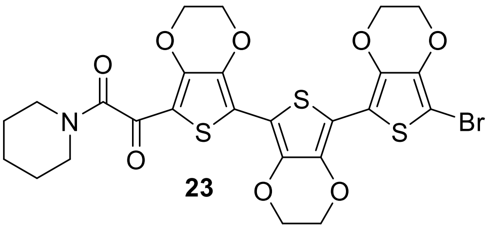

23

Run on 106 μmol scale, following ‘dimer bromination procedure A’. After precipitating in water (2 x 15 mL) and collecting by centrifugation (5000 rpm, 10 min), the product was used immediately without further purification or analysis due to its instability.

Oligomer synthesis protocol

Brominated oligomer (1 mmol), hydrogen-capped oligomer (1.2 mmol), pivalic acid (0.5 mmol), palladium (II) acetate (0.05 mmol) and potassium carbonate (10 mmol) were charged under nitrogen. Dry DMF (2 mL) was added and the mixture heated to 90 ºC for 2 hrs. After cooling to rt the mixture was diluted with DCM (50 mL) and washed with water (2 x 50 mL) and brine (50 mL). The organics were dried with $MgSO_4$, filtered and concentrated *in vacuo*. The residue was purified by flash column chromatography and pure fractions were concentrated *in vacuo*.

Numbering system for bifunctional-oligomer NMR assignments

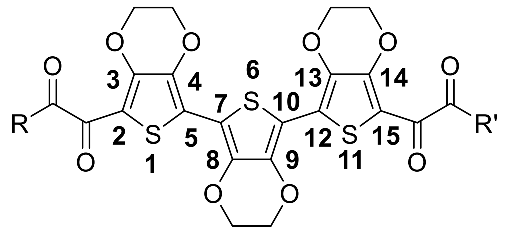

Dimer synthesis

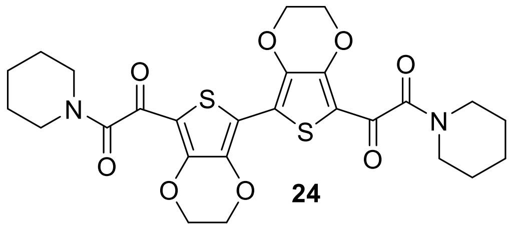

24

Run on 278 µmol scale at 130 °C with brominated piperidine-EDOT monomer **4** and piperidine-EDOT monomer **3**. Column eluted with 50-100 % EtOAc:Hexane. Yield of 65 mg, 116 mmol (42 %) as a yellow solid. ^{1}H NMR (400 MHz, $CDCl_3$): δ = 4.43 (8H, s, -OCH_2-), 3.64 (4H, t, *J* = 5.2 Hz, -CH_2N-), 3.38 (4H, t, *J* = 5.4 Hz, -CH_2N-), 1.62-1.74 (8H, m, -CH_2CH_2CH_2N-), 1.54-1.62 (2H, m, -CH_2CH_2N-) ppm; ^{13}C NMR (100 MHz, $CDCl_3$): δ = 182.19 (-COCONR$_2$), 165.03 (-CONR$_2$), 146.08 (ArC3), 139.05 (ArC4), 119.75 (ArC_α), 115.13 (ArC_α), 65.49 (-OCH$_2$), 64.77 (-OCH$_2$), 47.02 (-CH$_2$N-), 42.28 (-CH$_2$N-), 26.08 (-CH$_2$CH$_2$CH$_2$N-), 25.24 (-CH$_2$CH$_2$CH$_2$N-), 24.48 (-CH$_2$CH$_2$CH$_2$N-) ppm; IR (υ_{max}, solid): 2935, 2921, 2854, 1644, 1606, 1556, 1476, 1440, 1358, 1304, 1261, 1253, 1223, 1150, 1119, 1072, 1006 cm^{-1}; HRMS *m/z* (ESI+): Found: 561.1383 (M+H), Calc.: 561.1365; m.p. > 350 °C;

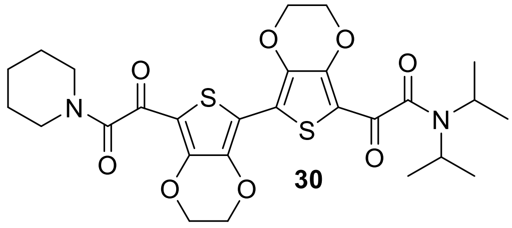

30

Run on 139 µmol scale with brominated piperidine-EDOT monomer **4** and diisopropyl-EDOT monomer **65**. Column eluted with 60-100 % EtOAc:Hexane. Yield of 59 mg, 102 µmol (74 %) as a yellow oily solid. ^{1}H NMR (400 MHz, $CDCl_3$): δ = 4.35-4.49 (8H, m, -OCH_2-), 3.82 (1H, sept, *J* = 6.6 Hz, -CHMe$_2$), 3.64 (2H, t, *J* = 5.3 Hz, -NCH_2-), 3.55 (1H, sept, *J* = 6.8 Hz, -CHMe$_2$), 3.38 (2H, t, *J* = 5.3 Hz, -NCH_2-), 1.55-1.85 (6H, m, -CH_2CH_2CH_2N-), 1.52 (6H, d, *J* = 6.8 Hz, -CHMe$_2$), 1.21 (6H, d, *J* = 6.6 Hz, -CHMe$_2$) ppm; ^{13}C NMR (100 MHz, $CDCl_3$): δ = 182.19 (-COCON-), 182.00 (-COCON-), 166.21 (-CON^{i}Pr), 165.07 (-COPip), 146.12 (ArC_β), 145.69 (ArC_β), 138.98 (ArC_β), 138.94 (ArC_β), 119.93 (ArC_α), 119.33 (ArC_α), 115.43 (ArC_α), 115.01 (ArC_α), 65.49 (-OCH$_2$), 65.08 (-OCH$_2$), 64.75 (-OCH$_2$), 50.31 (-CHMe$_2$), 47.02 (-CH$_2$N-), 45.82 (-CHMe$_2$), 42.27 (-CH$_2$N-), 26.07 (-CH$_2$CH$_2$CH$_2$N-), 25.25 (-CH$_2$CH$_2$CH$_2$N-), 24.49 (-CH$_2$CH$_2$CH$_2$N-), 20.63 (-CHMe$_2$), 20.03 (-CHMe$_2$) ppm; IR (υ_{max}, solid): 2971, 2937, 2860, 1634, 1614, 1551, 1473,

1434, 1357, 1280, 1254, 1233, 1216, 1115, 1074, 1042 cm^{-1}; HRMS *m/z* (ESI+): Found: 577.1693 (M+H), Calc.: 577.1678; m.p. = 312-314 °C;

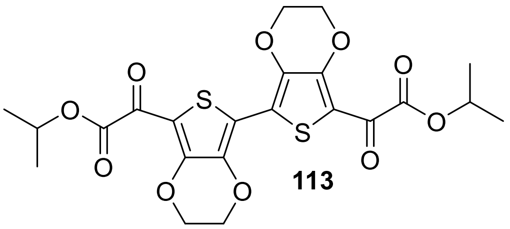

113

Run on 290 μmol scale with brominated isopropyl ester-EDOT monomer **8** and isopropyl ester-EDOT monomer **42**. Column eluted with 20-50 % EtOAc:Hexane. Yield of 44 mg, 86 mmol (30 %) as a yellow-orange solid. The product was highly insoluble in all standard NMR solvents and ^{13}C NMR analysis could not therefore be undertaken. ^{1}H NMR (400 MHz, DMSO-d$_6$): δ = 5.16 (2H, sept, *J* = 6.4 Hz, -C$\underline{H}$Me$_2$), 4.48-4.57 (8H, m, -OC$\underline{H}_2$-), 1.32 (12H, d, *J* = 6.4 Hz, -CH$\underline{Me}_2$) ppm; IR (υ_{max}, solid): 2923, 2872, 2853, 1732, 1635, 1473, 1432, 1366, 1275, 1227, 1103, 1075, 1021 cm^{-1}; HRMS *m/z* (ESI+): Found: 511.0739 (M+H), Calc.: 511.0733; m.p. = 225-228 °C;

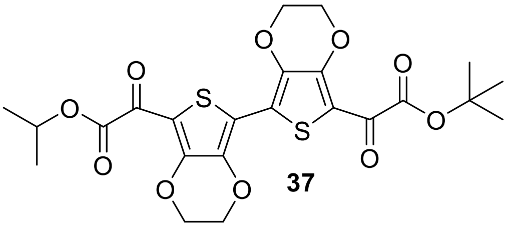

37

Run on 286 μmol scale with brominated *tert*-butyl ester-EDOT monomer **9** and isopropyl ester-EDOT monomer **42**. Column eluted with 20-50 % EtOAc:Hexane. Yield of 100 mg, 188 mmol (66 %) as a yellow solid. ^{1}H NMR (400 MHz, CDCl$_3$): δ = 5.26 (1H, sept, *J* = 6.3 Hz, -C$\underline{H}$Me$_2$), 4.37-4.51 (8H, m, -OC$\underline{H}_2$-), 1.61 (9H, s, -C$\underline{Me}_3$), 1.40 (6H, d, *J* = 6.3 Hz, -CH$\underline{Me}_2$) ppm; ^{13}C NMR (100 MHz, CDCl$_3$): δ = 176.86 (-$\underline{C}$OCO$_2$R), 176.25 (-$\underline{C}$OCO$_2$R), 162.98 (-$\underline{C}$O$_2$R), 162.70 (-$\underline{C}$O$_2$R), 147.40 (Ar$\underline{C}_\beta$), 147.06 (Ar$\underline{C}_\beta$), 139.06 (Ar$\underline{C}_\beta$), 138.97 (Ar$\underline{C}_\beta$), 120.63 (Ar$\underline{C}_\alpha$), 120.11 (Ar$\underline{C}_\alpha$), 113.80 (Ar$\underline{C}_\alpha$), 113.53 (Ar$\underline{C}_\alpha$), 84.47 (-$\underline{C}$Me$_3$), 70.74 (-$\underline{C}$HMe$_2$), 65.32 (-O$\underline{C}$H$_2$), 65.22 (-O$\underline{C}$H$_2$), 64.75 (-O$\underline{C}$H$_2$), 27.95 (-C$\underline{Me}_3$), 21.61 (-CH$\underline{Me}_2$) ppm; IR (υ_{max}, solid): 2981, 2935, 1732, 1620, 1547, 1472, 1439, 1360, 1317, 1245, 1222, 1162, 1121, 1099, 1075, 1027 cm^{-1}; HRMS *m/z* (ESI+): Found: 525.0880 (M+H), Calc.: 525.0889; m.p. = 270-272 °C (Degrades);

Trimer Synthesis

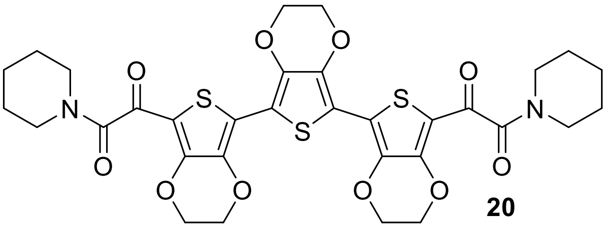

20

Run on 142 μmol scale at 130 °C with dibromo-EDOT **25** and piperidine-EDOT monomer **3**. Column eluted with 0-8 % MeOH:EtOAc. Yield of 68 mg, 97 mmol (68 %) as a red solid. Alternatively, the product could be accessed through the coupling of brominated piperidine-EDOT

monomer **4** and piperidine-EDOT dimer **19** in a 58 % yield. ^{1}H NMR (400 MHz, $CDCl_3$): δ = 4.33-4.57 (12H, s, -OC$\underline{H}_2$-), 3.64 (4H, t, *J* = 5.2 Hz, -C$\underline{H}_2$N-), 3.38 (4H, t, *J* = 5.4 Hz, -C$\underline{H}_2$N-), 1.61-1.74 (8H, m, -C$\underline{H}_2$C$\underline{H}_2$CH$_2$N-), 1.52-1.61 (4H, m, -C$\underline{H}_2$CH$_2$N-) ppm; ^{13}C NMR (100 MHz, $CDCl_3$): δ = 181.68 (-$\underline{C}$OCONR$_2$), 165.40 (-$\underline{C}$ONR$_2$), 146.41 (Ar$\underline{C}$3), 139.71 (Ar$\underline{C}_\beta$), 136.73 (Ar$\underline{C}_\beta$), 122.00 (Ar$\underline{C}_\alpha$), 113.24 (Ar$\underline{C}_\alpha$), 110.94 (Ar$\underline{C}_\alpha$), 65.51 (-O$\underline{C}$H$_2$), 65.22 (-O$\underline{C}$H$_2$-), 64.72 (-O$\underline{C}$H$_2$), 47.05 (-$\underline{C}$H$_2$N-), 42.22 (-$\underline{C}$H$_2$N-), 26.07 (-$\underline{C}$H$_2\underline{C}$H$_2$CH$_2$N-), 25.26 (-$\underline{C}$H$_2\underline{C}$H$_2$CH$_2$N-), 24.52 (-$\underline{C}$H$_2\underline{C}$H$_2$CH$_2$N-) ppm; IR ($υ_{max}$, solid): 2923, 2853, 1642 1622, 1614, 1486, 1431, 1361, 1308, 1251, 1217, 1117, 1096, 1065 cm^{-1}; HRMS *m/z* (ESI+): Found: 701.1299 (M+H), Calc.: 701.1292; m.p. = 315-318 °C;

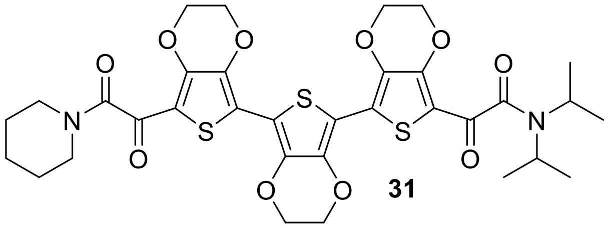

31

Run on 100 µmol scale with brominated piperidine-EDOT monomer **4** and diisopropyl-EDOT dimer **66**. Colum eluted with 60-100 % EtOAc:Hexane. Yield of 51 mg, 72 µmol (72 %) as an orange solid. ^{1}H NMR (400 MHz, $CDCl_3$): δ = 4.33-4.52 (12H, m, -OC$\underline{H}_2$-), 3.87 (1H, sept, *J* = 6.6 Hz, -C$\underline{H}$Me$_2$), 3.65 (2H, t, *J* = 5.3 Hz, -NC$\underline{H}_2$-), 3.55 (1H, sept, *J* = 6.8 Hz, -C$\underline{H}$Me$_2$), 3.40 (2H, t, *J* = 5.3 Hz, -NC$\underline{H}_2$-), 1.62-1.78 (6H, m, -C$\underline{H}_2$C$\underline{H}_2$CH$_2$N-), 1.57-1.64 (2H, m, -C$\underline{H}_2$C$\underline{H}_2$CH$_2$N-), 1.53 (6H, d, *J* = 6.8 Hz, -CH$\underline{Me}_2$), 1.21 (6H, d, *J* = 6.6 Hz, -CH$\underline{Me}_2$) ppm; ^{13}C NMR (100 MHz, $CDCl_3$): δ = 181.65 (-$\underline{C}$OCON-), 181.60 (-$\underline{C}$OCON-), 166.55 (-$\underline{C}$ON^{i}Pr), 165.39 (-$\underline{C}$OPip), 146.39 (Ar$\underline{C}_\beta$), 145.98 (Ar$\underline{C}_\beta$), 139.74 (Ar$\underline{C}_\beta$), 139.57 (Ar$\underline{C}_\beta$), 136.67 (Ar$\underline{C}_\beta$), 136.61 (Ar$\underline{C}_\beta$), 122.07 (Ar$\underline{C}_\alpha$), 121.49 (Ar$\underline{C}_\alpha$), 113.55 (Ar$\underline{C}_\alpha$), 113.23 (Ar$\underline{C}_\alpha$), 111.05 (Ar$\underline{C}_\alpha$), 110.71 (Ar$\underline{C}_\alpha$), 65.48 (-O$\underline{C}$H$_2$), 65.21 (-O$\underline{C}$H$_2$), 65.17 (-O$\underline{C}$H$_2$), 65.07 (-O$\underline{C}$H$_2$-), 64.69 (-O$\underline{C}$H$_2$-), 50.26 (-$\underline{C}$HMe$_2$), 47.06 (-$\underline{C}$H$_2$N-), 45.75 (-$\underline{C}$HMe$_2$), 42.24 (-$\underline{C}$H$_2$N-), 26.09 (-$\underline{C}$H$_2\underline{C}$H$_2$CH$_2$N-), 25.28 (-$\underline{C}$H$_2\underline{C}$H$_2$CH$_2$N-), 24.54 (-$\underline{C}$H$_2\underline{C}$H$_2$CH$_2$N-), 20.62 (-CH$\underline{Me}_2$), 20.07 (-CH$\underline{Me}_2$) ppm; IR ($υ_{max}$, solid): 2930, 2863, 1720, 1631, 1610, 1545, 1507, 1465, 1424, 1359, 1314, 1251, 1216, 1150, 1116, 1067, 1014 cm^{-1}; HRMS *m/z* (ESI+): Found: 717.1616 (M+H), Calc.: 717.1610; m.p. = 324-326 °C;

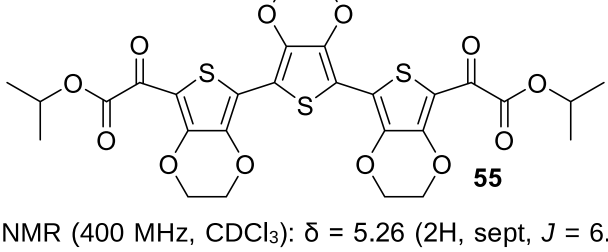

55

Run on 156 µmol scale with dibromo-EDOT **25** and isopropyl ester-EDOT monomer **42**. Column eluted with 30-80 % EtOAc:Hexane. Yield of 74 mg, 114 mmol (73 %) as a deep red solid. ^{1}H NMR (400 MHz, $CDCl_3$): δ = 5.26 (2H, sept, *J* = 6.2 Hz, -C$\underline{H}$Me$_2$), 4.40-4.51 (12H, m, -OC$\underline{H}_2$-), 1.61

(9H, s, -CMe$_3$), 1.40 (12H, d, *J* = 6.2 Hz, -CHMe$_2$); ^{13}C NMR (100 MHz, $CDCl_3$): δ = 175.58 (-COCO$_2$*i*Pr), 163.31 (-CO$_2$*i*Pr), 147.77 (ArC3), 139.91 (ArC$_β$), 136.72 (ArC$_β$), 122.90 (ArC$_α$), 111.77 (ArC$_α$), 111.20 (ArC$_α$), 70.50 (-CHMe$_2$), 65.33 (-OCH$_2$), 65.24 (-OCH$_2$-), 64.66 (-OCH$_2$), 21.63 (-CMe$_2$) ppm; IR (υ$_{max}$, solid): 2938, 2858, 1716, 1683, 1634, 1621, 1471, 1427, 1375, 1360, 1314, 1299, 1217, 1197, 1172, 1133, 1117, 1069 cm^{-1}; HRMS *m/z* (ESI+): Found: 651.0677 (M+H), Calc.: 651.0665; m.p. = 293-296 °C;

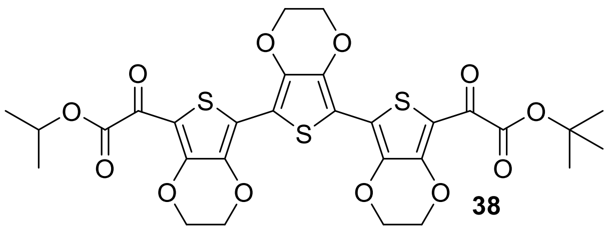

38

Run on 210 µmol scale with brominated *tert*-butyl ester-EDOT monomer **9** and isopropyl ester-EDOT dimer **34**. Column eluted with 40-80 % EtOAc:Hexane. Yield of 114 mg, 171 mmol (82 %) as a deep red solid. ^{1}H NMR (400 MHz, $CDCl_3$): δ = 5.25 (1H, sept, *J* = 6.2 Hz, -CHMe$_2$), 4.39-4.49 (12H, m, -OCH$_2$-), 1.61 (9H, s, -CMe$_3$), 1.39 (6H, d, *J* = 6.2 Hz, -CHMe$_2$) ppm; ^{13}C NMR (100 MHz, $CDCl_3$): δ = 176.26 (-COCO$_2$R), 175.58 (-COCO$_2$R), 163.35 (-CO$_2$R), 163.06 (-CO$_2$R), 147.78 (ArC$_β$), 147.44 (ArC$_β$), 139.91 (ArC$_β$), 139.77 (ArC$_β$), 136.68 (ArC$_β$), 122.95 (ArC$_α$), 122.42 (ArC$_α$), 111.87 (ArC$_α$), 111.27 (ArC$_α$) 111.02 (ArC$_α$), 84.14 (-CMe$_3$), 70.49 (-CHMe$_2$), 65.36 (-OCH$_2$), 65.25 (-OCH$_2$-), 64.67 (-OCH$_2$), 27.96 (-CMe$_3$), 21.63 (-CMe$_2$) ppm; IR (υ$_{max}$, solid): 2981, 2934, 2876, 1736, 1613, 1469, 1434, 1359, 1335, 1265, 1244, 1221, 1163, 1135, 1117, 1097, 1069 cm^{-1}; HRMS *m/z* (ESI+): Found: 664.9289 (M+H), Calc.: 665.0821; m.p. = 256-257 °C;

Tetramer Synthesis

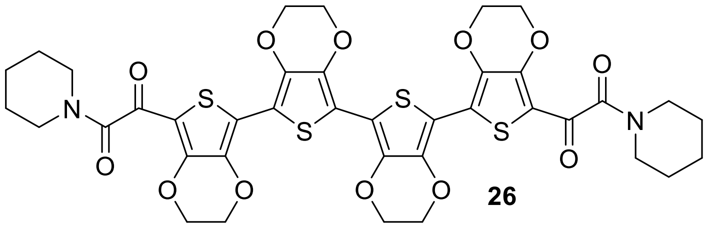

26

Run on 200 µmol scale with brominated piperidine-EDOT dimer **21** and piperidine-EDOT dimer **19**. Column eluted with 0-5 % MeOH:DCM. Yield of 148 mg, 176 µmol (88 %) as a deep red/purple solid. ^{1}H NMR (400 MHz, $CDCl_3$): δ = 4.14-4.64 (16H, m, -OCH$_2$-), 3.58-3.72 (4H, m, -NCH$_2$-), 3.29-3.48 (4H,m, -NCH$_2$-), 1.39-1.81 (12H, m, -CH$_2$CH$_2$CH$_2$N-) ppm; ^{13}C NMR (125 MHz, $CDCl_3$): 181.76 (-COCONR$_2$), 165.35 (-CONR$_2$), 146.33 (ArC$_β$), 142.93 (ArC$_β$), 139.34 (ArC$_β$), 136.68 (ArC$_β$), 121.80 (ArC$_α$), 112.86 (ArC$_α$), 111.91 (ArC$_α$), 108.77 (ArC$_α$), 65.44 (-OCH$_2$-), 64.97 (-OCH$_2$-), 64.92 (-OCH$_2$-), 64.54 (-OCH$_2$-), 47.02 (-CH$_2$N-), 42.21 (-CH$_2$N-), 26.04 (-CH$_2$CH$_2$CH$_2$N-), 25.24 (-CH$_2$CH$_2$CH$_2$N-), 24.50 (-CH$_2$CH$_2$CH$_2$N-) ppm; IR (υ$_{max}$, solid): 2931, 2853,

1652, 1601, 1574, 1488, 1463, 1428, 1358, 1308, 1279, 1263, 1250, 1219, 1135, 1116, 1108, 1067, 1022 cm^{-1}; HRMS *m/z* (ESI+): Found: 841.1229 (M+H), Calc.: 841.1232; m.p. > 350 °C;

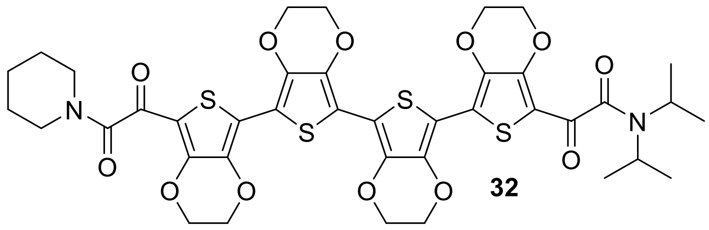

32

Run on 237 µmol scale with brominated diisopropyl-EDOT dimer **67** and piperidine-EDOT dimer **19**. Column eluted with 0-5 % MeOH:DCM. Yield of 136 mg, 158 µmol (67 %) as a deep red/purple solid. ^{1}H NMR (400 MHz, $CDCl_3$): δ = 4.18-4.66 (16H, m, -OC$\underline{H}_2$-), 3.86 (1H, sept, *J* = 6.6 Hz, -C$\underline{H}$Me$_2$), 3.64 (2H, t, *J* = 5.0 Hz, -NC$\underline{H}_2$-), 3.54 (1H, sept, *J* = 6.7 Hz, -C$\underline{H}$Me$_2$), 3.39 (2H, t, *J* = 5.4 Hz, -NC$\underline{H}_2$-), 1.60-1.74 (4H, m, -C$\underline{H}_2$C$\underline{H}_2$CH$_2$N-), 1.55-1.60 (2H, m, -C$\underline{H}_2$CH$_2$N-), 1.52 (6H, d, *J* = 6.7 Hz, -CH$\underline{Me}_2$), 1.20 (6H, d, *J* = 6.6 Hz, -CH$\underline{Me}_2$) ppm; ^{13}C NMR (125 MHz, $CDCl_3$): 181.72 (-$\underline{C}$OCONR$_2$), 181.49 (-$\underline{C}$OCONR$_2$), 166.67 (-$\underline{C}$ONR$_2$), 165.53 (-$\underline{C}$ONR$_2$), 146.52 (Ar$\underline{C}_\beta$), 141.32 (Ar$\underline{C}_\beta$), 140.27 (Ar$\underline{C}_\beta$), 137.24 (Ar$\underline{C}_\beta$), 136.07 (Ar$\underline{C}_\beta$), 122.88 (Ar$\underline{C}_\alpha$), 112.15 (Ar$\underline{C}_\alpha$), 108.94 (Ar$\underline{C}_\alpha$), 101.83 (Ar$\underline{C}_\alpha$), 65.47 (-O$\underline{C}$H$_2$-), 65.18 (-O$\underline{C}$H$_2$-), 65.10 (-O$\underline{C}$H$_2$-), 64.60 (-O$\underline{C}$H$_2$-), 50.21 (-$\underline{C}$HMe$_2$), 47.01 (-$\underline{C}$H$_2$N-), 45.66 (-$\underline{C}$HMe$_2$), 42.14 (-$\underline{C}$H$_2$N-), 26.02 (-$\underline{C}$H$_2\underline{C}$H$_2$CH$_2$N-), 25.22 (-$\underline{C}$H$_2\underline{C}$H$_2$CH$_2$N-), 24.50 (-$\underline{C}$H$_2\underline{C}$H$_2$CH$_2$N-), 20.56 (-CH$\underline{Me}_2$), 20.03 (-CH$\underline{Me}_2$) ppm; IR (υ_{max}, solid): 2933, 1634, 1594, 1542, 1493, 1467, 1428, 1359, 1251, 1220, 1152, 1136, 1116, 1094, 1067 cm^{-1}; HRMS *m/z* (ESI+): Found: 857.1528 (M+H), Calc.: 857.1542; m.p. > 350 °C;

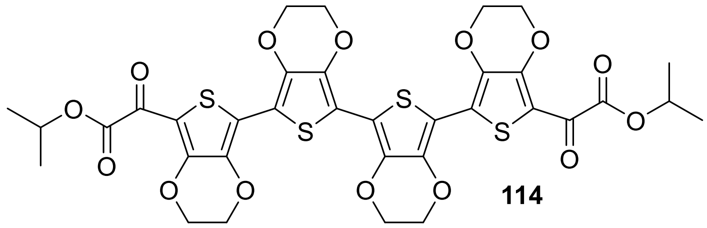

114

Run on 210 µmol scale with brominated *iso*-propyl ester-EDOT dimer **72** and *iso*-propyl ester-EDOT dimer **34**. Column eluted with 0-5 % MeOH:DCM. Yield of 122 mg, 154 µmol (74 %) as a purple solid. The low solubility of the product in $CDCl_3$ and propensity to aggregate in solution prevented the aromatic peaks being resolved in the ^{13}C NMR spectra and so analysis was not undertaken. ^{1}H NMR (400 MHz, $CDCl_3$): δ = 5.27 (2H, sept, *J* = 6.5 Hz, -C$\underline{H}$Me$_2$), 4.32-4.54 (16H, m, -OC$\underline{H}_2$-), 1.41 (12H, d, *J* = 6.5 Hz, -CH$\underline{Me}_2$) ppm; IR (υ_{max}, solid): 2932, 2869, 1734, 1601, 1490, 1464, 1428, 1359, 1313, 1214, 1139, 1098, 1066, 1022 cm^{-1}; HRMS *m/z* (ESI+): Found: 791.0597 (M+H), Calc.: 791.0597; m.p. > 350 °C;

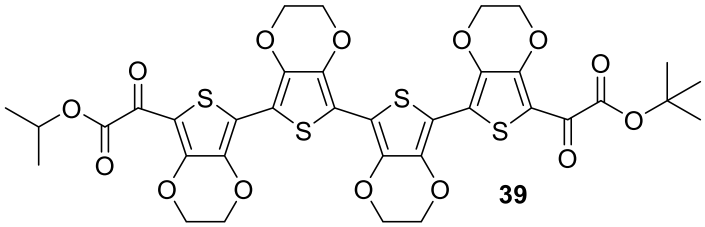

39

Run on 91 µmol scale with brominated *iso*-propyl ester-EDOT monomer **8** and *tert*-butyl ester-EDOT trimer **78**. Column eluted with 1-4 % MeOH:DCM. Yield of 63 mg, 78 µmol (86 %) as a purple solid. The product was highly insoluble in all commonly used NMR solvents and quickly aggregated in solution preventing full analysis. Thus, the purity of the construct could not be determined. However, the presence of the product as the major species was confirmed by HRMS. ^{1}H NMR (400 MHz, $CDCl_3$): δ = 5.28 (1H, sept, *J* = 6.2 Hz, -C<u>H</u>Me_2), 4.34-4.58 (16H, m, -OC<u>H</u>$_2$-), 1.63 (9H, s, -C<u>Me</u>$_3$), 1.41 (6H, d, *J* = 6.2 Hz, -CH<u>Me</u>$_2$) ppm; IR (υ_{max}, solid): 2979, 2938, 2869, 1722, 1605, 1439, 1454, 1434, 1361, 1221, 1139, 1092, 1069 cm^{-1}; HRMS *m/z* (ESI+): Found: 805.0746 (M+H), Calc.: 805.0753; m.p. > 350 °C;

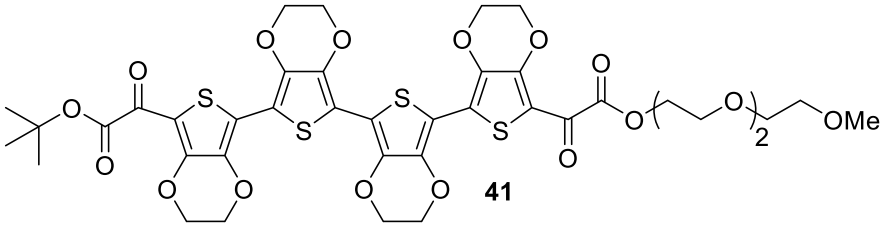

41

Run on 100 µmol scale with brominated TEG-EDOT monomer **74** and *tert*-butyl ester-EDOT trimer **78**. Column eluted with 1-5 % MeOH:DCM. Yield of 67 mg, 74 µmol (74 %) as a purple solid. Despite significantly improved solubility when compared to **39**, the aromatic peaks could not be resolved by ^{13}C NMR and so analysis was not undertaken ^{1}H NMR (400 MHz, $CDCl_3$): δ = 4.35-4.55 (16H, m, -OC<u>H</u>$_2$-), 3.84 (2H, t, *J* = 5.0 Hz, TEG), 3.62-3.75 (6H, m, TEG), 3.53-3.59 (2H, m, TEG), 3.39 (3H, s, -O<u>Me</u>), 1.60 (9H, s, -O<u>*t*Bu</u>) ppm; IR (υ_{max}, solid): 2946, 2928, 2873, 1732, 1600, 1428, 1358, 1319, 1217, 1203, 1138, 1117, 1092, 1064, 1021 cm^{-1}; HRMS *m/z* (ESI+): Found: 909.1221 (M+H), Calc.: 909.1227; m.p. > 350 °C;

Pentamer Synthesis

Aromatic peaks could not be resolved in the ^{13}C NMR spectra of all oligomers of 5 repeating units and longer, even after long scan times at elevated temperatures. Details are not therefore given for all subsequent oligomers.

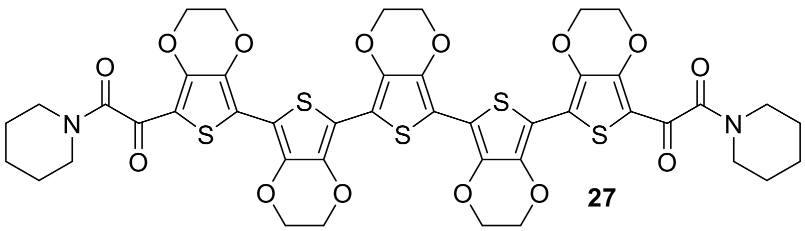

27

Run on 108 µmol scale with dibromo-EDOT **25** and piperidine-EDOT dimer **19**. Column eluted with 0-20 % MeOH:EtOAc. Yield of 58 mg, 59 µmol (55 %) as a purple solid. Alternatively, the product could be accessed through the coupling of brominated piperidine-EDOT dimer **21** and piperidine-EDOT trimer **22**. ^{1}H NMR (400 MHz, $CDCl_3$): δ = 4.40-4.49 (20H, s, -OC$\underline{H}_2$-), 3.65 (4H, t, *J* = 5.1 Hz, -C$\underline{H}_2$N-), 3.41 (4H, t, *J* = 5.3 Hz, -C$\underline{H}_2$N-), 1.65-1.73 (8H, m, -C$\underline{H}_2$C$\underline{H}_2$CH$_2$N-), 1.53-1.62 (4H, m, -C$\underline{H}_2$CH$_2$N-) ppm; IR (υ_{max}, solid): 2923, 2855, 1621, 1433, 1360, 1261, 1219, 1116, 1069 cm^{-1}; MS *m/z* (MALDI+): Found: 980.8 (M+H), Calc.: 981.1; m.p. > 350 °C;

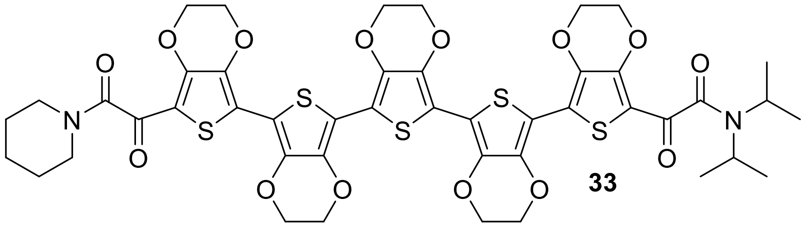

33

Run on 89 µmol scale with brominated diisopropyl-EDOT dimer **67** and piperidine-EDOT trimer **22**. Column eluted with 2-5 % MeOH:DCM. Yield of 39 mg, 39 µmol (44 %) as a purple solid. ^{1}H NMR (400 MHz, $CDCl_3$): δ = 4.19-4.56 (20H, s, -OC$\underline{H}_2$-), 3.88 (1H, sept, *J* = 6.9 Hz, -C$\underline{H}$Me$_2$), 3.66 (2H, t, *J* = 5.1 Hz, -C$\underline{H}_2$N-), 3.55 (1H, sept, *J* = 6.9 Hz, -C$\underline{H}$Me$_2$), 3.41 (2H, t, *J* = 5.4 Hz, -C$\underline{H}_2$N-), 1.63-1.74 (4H, m, -C$\underline{H}_2$C$\underline{H}_2$CH$_2$N-), 1.49-1.63 (8H, m, -C$\underline{H}_2$CH$_2$N- and –CH$\underline{Me}_2$), 1.22 (6H, d, *J* = 6.9 Hz, -CH$\underline{Me}_2$) ppm; IR (υ_{max}, solid): 2944, 2857, 1634, 1603, 1469, 1435, 1358, 1311, 1262, 1223, 1116, 1088, 1042 cm^{-1}; MS *m/z* (MALDI+): Found: 996.8 (M+H), Calc.: 997.1; m.p. > 350 °C;

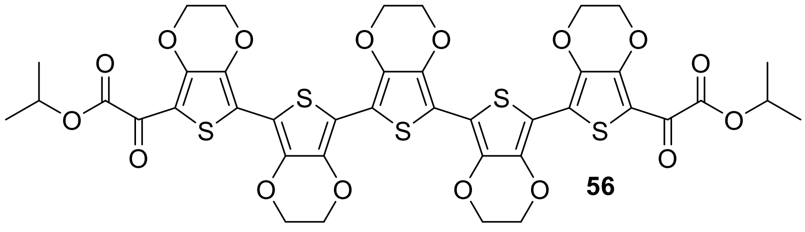

56

Run on 105 µmol scale with EDOT **1** and brominated *iso*-propyl ester-EDOT dimer **72**. Column eluted with 1-4 % MeOH:DCM. Yield of 56 mg, 64 µmol (61 %) as a purple solid. ^{1}H NMR (400 MHz, $CDCl_3$): δ = 5.27 (2H, sept, *J* = 6.2 Hz, -C$\underline{H}$Me$_2$), 4.39-4.54 (20H, m, -OC$\underline{H}_2$-), 1.41 (12H, d, *J* = 6.2 Hz, -CH$\underline{Me}_2$) ppm; IR (υ_{max}, film): 2920, 2850, 1737, 1603, 1462, 1430, 1360, 1257, 1211, 1098, 1066 cm^{-1}; MS *m/z* (MALDI+): Found: 930.6 (M+H), Calc.: 931.0; m.p. > 350 °C;

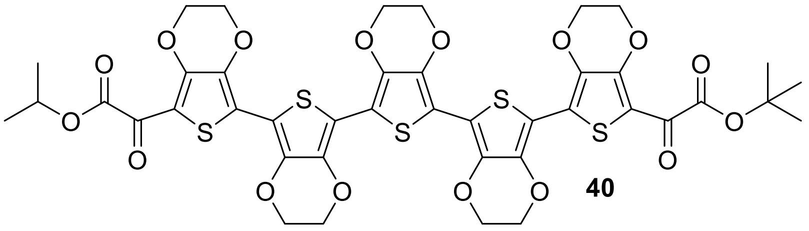

40

Run on 143 µmol scale with brominated *tert*-butyl ester-EDOT dimer **36** and *iso*-propyl ester-EDOT trimer **35**. Column eluted with 1-4 % MeOH:DCM. Yield of 56 mg, 60 µmol (42%) as a purple solid. ^{1}H NMR (400 MHz, $CDCl_3$): δ = 5.26 (1H, sept, *J* = 6.3 Hz, -C$\underline{H}$Me$_2$), 4.33-4.53 (20H, m, -OC$\underline{H}_2$-), 1.62 (9H, s, -C$\underline{Me}_3$), 1.41 (6H, d, *J* = 6.3 Hz, -CH$\underline{Me}_2$) ppm; IR (υ_{max}, solid): 2932, 1735, 1613, 1462, 1431, 1359, 1257, 1218, 1144, 1098, 1067 cm^{-1}; LRMS *m/z* (ESI+): Found: 945.0 (M+H), Calc.: 945.1; m.p. > 350 °C;

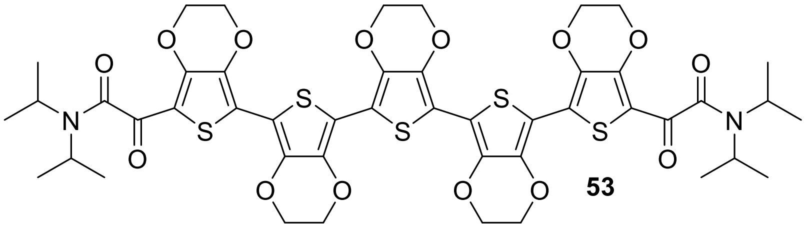

53

Run on 190 µmol scale with EDOT **1** and brominated diisopropylamine-EDOT dimer **67**. Column eluted with -4 % MeOH:DCM. Yield of 47 mg, 46 µmol (48%) as a purple solid. ^{1}H NMR (400 MHz, $CDCl_3$): δ = 4.22-4.54 (20H, m, -OC$\underline{H}_2$-), 3.82-3.95 (2H, m, -C$\underline{H}$Me$_2$), 3.50-3.60 (2H, m, -C$\underline{H}$Me$_2$), 1.54 (12H, d, *J* = 6.8 Hz, -CH$\underline{Me}_2$), 1.21 (12H, d, *J* = 7.0 Hz, -CH$\underline{Me}_2$) ppm; HRMS *m/z* (ESI+): Found: 1013.1821 (M+H), Calc.: 1013.1787; m.p. > 350 °C;

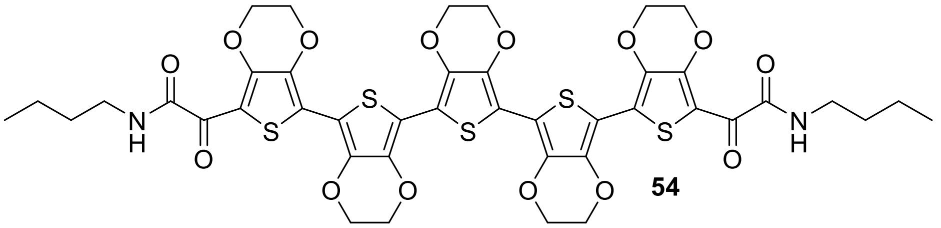

54

Run on 73 µmol scale with dibromo-EDOT **25** and *n*-butylamine-EDOT dimer **109**. Column eluted with 2-5 % MeOH:DCM. Yield of 12 mg, 12 µmol (34 %) as a purple solid. ^{1}H NMR (400 MHz, $CDCl_3$): δ = 7.46 (2H, t, *J* = 6.3 Hz, -N$\underline{H}$), 4.21-4.57 (20H, m, -OC$\underline{H}_2$-), 3.38 (4H, dt, J_1 = J_2 = 6.8 Hz, -NHC$\underline{H}_2$-), 1.53-1.68 (4H, m, -NHCH$_2$C$\underline{H}_2$-), 1.34-1.47 (4H, m, -C$\underline{H}_2$CH$_3$), 0.96 (6H, t, *J* = 7.3 Hz, -C$\underline{H}_3$) ppm; MS *m/z* (MALDI+): Found: 957.0 (M+H), Calc.: 957.1; m.p. > 350 °C;

Hexamer synthesis

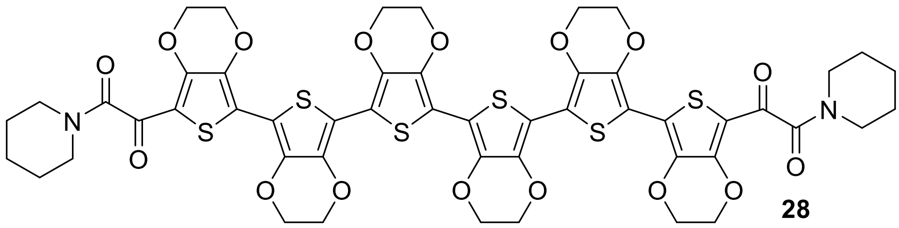

28

Run on 53 µmol scale with brominated piperidine-EDOT trimer **23** and piperidine-EDOT trimer **22**. Column eluted with 2-5 % MeOH:DCM. Yield of 21 mg, 18 µmol (35 %) as a purple solid. ^{1}H NMR (400 MHz, $CDCl_3$): δ = 4.13-4.61 (24H, s, -OC<u>H</u>$_2$-), 3.56-3.74 (4H, m, -C<u>H</u>$_2$N-), 3.33-3.53 (4H, m, -C<u>H</u>$_2$N-), 1.65-1.78 (8H, m, -C<u>H</u>$_2$C<u>H</u>$_2$CH$_2$N-), 1.49-1.65 (4H, m, -C<u>H</u>$_2$CH$_2$N-) ppm; IR ($υ_{max}$, solid): 2921, 2856, 1614, 1542, 1428, 1357, 1311, 1259, 1215, 1188, 1115, 1065 cm^{-1}; MS *m/z* (MALDI+): Found: 1120.8 (M+H), Calc.: 1121.1; m.p. > 350 °C;

Heptamer synthesis

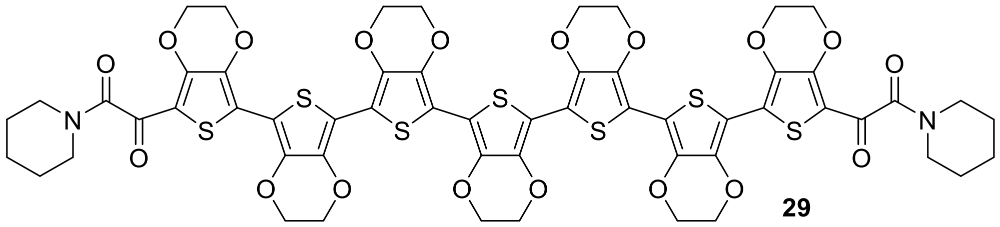

29

Run on 89 µmol scale with dibromo-EDOT **25** and piperidine-EDOT trimer **22**. Column eluted with 0-5 % MeOH:DCM. Yield of 46 mg, 37 µmol (42%) as a purple solid. ^{1}H NMR (400 MHz, $CDCl_3$): δ = 4.06-4.59 (28H, s, -OC<u>H</u>$_2$-), 3.61-3.71 (4H, t, -C<u>H</u>$_2$N-), 3.35-3.44 (4H, t, -C<u>H</u>$_2$N-), 1.53-1.76 (12H, m, -C<u>H</u>$_2$C<u>H</u>$_2$CH$_2$N-) ppm; IR ($υ_{max}$, solid): 2921, 2852, 1727, 1626, 1468, 1437, 1361, 1313, 1282, 1253, 1221, 1117, 1072 cm^{-1}; MS *m/z* (MALDI+): Found: 1260.8 (M+H), Calc.: 1261.1; m.p. > 350 °C;

Oligomer manipulations

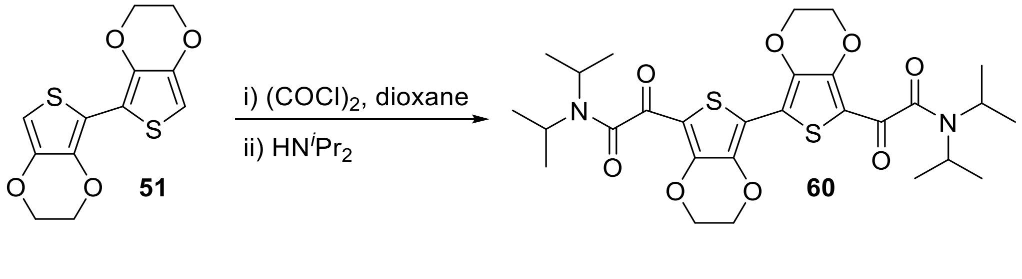

Oxalyl chloride (50 µL, 590 µmol) was added dropwise to a solution of di-EDOT **51** (10 mg, 35 µmol) in dioxane (2 mL) and heated to 100 ºC for 2 hrs. After cooling to rt, diisopropylamine (200 µL, 1425 µmol) was added and the mixture stirred for 20 min. The mixture was then diluted with DCM (20 mL) and the organics washed with water (10 mL), dried with $MgSO_4$, filtered and concentrated *in vacuo*. The residue was purified by flash column chromatography eluting with 70-100 % EtOAc:Hexane. Pure fractions were concentrated *in vacuo* to give the DP as a yellow solid. A yield of 12 mg, 21 µmol (60 %) was obtained. ^{1}H NMR (400 MHz, $CDCl_3$): δ = 4.41-4.46 (4H, m, -OCH_2-), 4.37-4.42 (4H, m, -OCH_2-), 3.83 (2H, sept, *J* = 6.6 Hz, -CHMe$_2$), 3.57 (2H, sept, *J* = 6.8 Hz, -CHMe$_2$), 1.53 (12H, d, *J* = 6.8 Hz, -CHMe$_2$), 1.22 (12H, d, *J* = 6.6 Hz, -CHMe$_2$) ppm; ^{13}C NMR (100 MHz, $CDCl_3$): δ = 181.97 (-COCON-), 166.23 (-CON-), 145.69 (ArC3), 138.85 (ArC4), 119.52 (ArC$_\alpha$), 115.34 (ArC$_\alpha$), 65.07 (-OCH_2-), 64.71 (-OCH_2-), 50.29 (-CHMe$_2$), 45.83 (-CHMe$_2$), 20.63 (-CHMe$_2$), 20.04 (-CHMe$_2$) ppm; IR (υ_{max}, solid): 2974, 1636, 1614, 1555, 1478, 1440, 1364, 1273, 1237, 1210, 1149, 1077 cm^{-1}; HRMS *m/z* (ESI+): Found: 593.1999 (M+H), Calc.: 593.1991; m.p. > 350 °C;

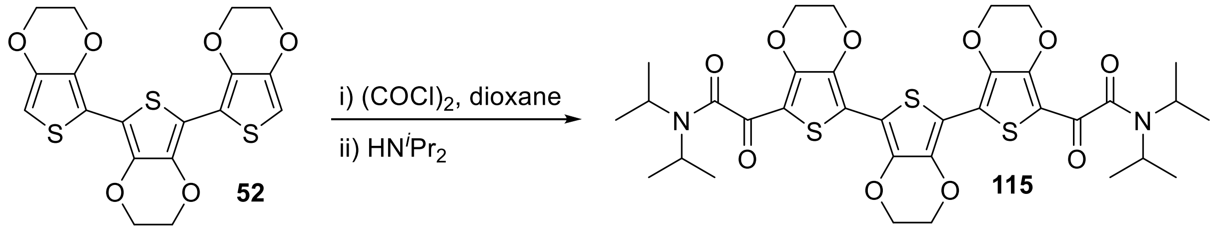

Oxalyl chloride (80 µL, 944 µmol) was added dropwise to a solution of tri-EDOT **52** (20 mg, 47 µmol) in dioxane (2 mL) and heated to 100 ºC for 2 hrs. After cooling to rt, diisopropylamine (400 µL, 2851 µmol) was added and the mixture stirred for 20 min. The mixture was then diluted with DCM (20 mL) and the organics washed with water (10 mL), dried with $MgSO_4$, filtered and concentrated *in vacuo*. The residue was purified by flash column chromatography eluting with 2 % NEt_3 in 60-100 % EtOAc:Hexane. Pure fractions were concentrated *in vacuo* to give the DP as an orange solid. A yield of 25 mg, 34 µmol (74 %) was obtained. ^{1}H NMR (400 MHz, $CDCl_3$): δ = 4.41-4.48 (8H, m, -OCH_2-), 4.35-4.41 (4H, m, -OCH_2-), 3.87 (2H, sept, *J* = 6.6 Hz, -CHMe$_2$), 3.55 (2H, sept, *J* = 6.8 Hz, -CHMe$_2$), 1.54 (12H, d, *J* = 6.8 Hz, -CHMe$_2$), 1.22 (12H, d, *J* = 6.6 Hz, -CHMe$_2$) ppm; ^{13}C NMR (100 MHz, $CDCl_3$): δ = 181.59 (-COCON-), 166.56 (-CON-), 145.99 (ArC$_\alpha$), 139.59 (ArC$_\alpha$), 136.55 (ArC$_\alpha$), 121.56 (ArC$_\alpha$), 113.48 (ArC$_\alpha$), 110.82 (ArC$_\alpha$), 65.18 (-OCH_2-), 65.07 (-OCH_2-), 64.68 (-OCH_2-), 50.25 (-CHMe$_2$), 45.74 (-CHMe$_2$), 20.62 (-CHMe$_2$), 20.07 (-CHMe$_2$) ppm; IR (υ_{max}, solid): 3361, 3297, 2925, 2852, 1634, 1547, 1470, 1424, 1358,

1297, 1268, 1255, 1235, 1209, 1090, 1070, 1042 cm^{-1}; HRMS *m/z* (ESI+): Found: 733.1931 (M+H), Calc.: 733.1923; m.p. > 350 °C;

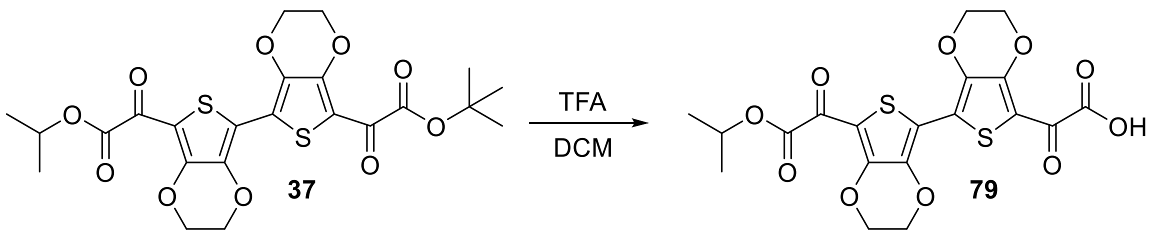

Trifluoroacetic acid (5 mL) was added to a solution of hetero-bifunctional EDOT-dimer **37** (200 mg, 382 μmol) in DCM (5 mL). After stirring for 2 hrs, complete cleavage of the *tert*-butyl ester was observed by LC-MS with no *iso*-propyl ester cleavage. The mixture was concentrated *in vacuo* and azeotroped with toluene (2 x 20 mL) to afford the DP as an orange solid which was used directly in further manipulations.

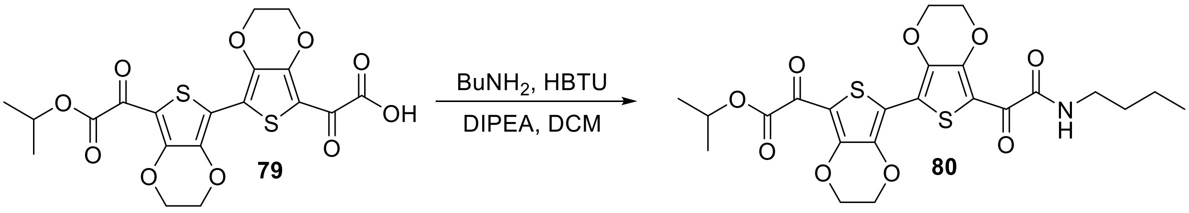

HBTU (162 mg, 427 μmol) and DIPEA (149 μL, 855 μmol) were added to a suspension of EDOT dimer **79** (100 mg, 213 μmol) in DCM (10 mL). After 5 min complete dissolution had occurred at which point *n*-butylamine (42 μL, 427 μmol) was added. After stirring for 18 hrs, the mixture was diluted with DCM (50 mL) and the organics washed with hydrochloric acid (1 M, 50 mL), dried with $MgSO_4$, filtered and concentrated *in vacuo*. The residue was purified by flash column chromatography eluting with 50-70 % EtOAc:Hexane. Pure fractions were concentrated *in vacuo* to give the DP as an orange-yellow solid. A yield of 40 mg, 76 μmol (36 %) was obtained. ^{1}H NMR (400 MHz, $CDCl_3$): 7.43 (1H, br t, *J* = 6.2 Hz, -N<u>H</u>), 5.27 (1H, sept, *J* = 6.3 Hz, -C<u>H</u>Me_2), 4.36-4.58 (8H, m, -OC<u>H</u>$_2$-), 3.38 (2H, td, *J* = 6.9, 6.2 Hz, -C<u>H</u>$_2$NH-), 1.54-1.67 (2H, m, -C<u>H</u>$_2$$CH_2$NH-), 1.31-1.47 (8H, m, -C<u>H</u>$_2$$CH_3$ and –CH<u>Me</u>$_2$), 0.96 (3H, t, *J* = 7.3 Hz, -CH_2C<u>H</u>$_3$) ppm; ^{13}C NMR (100 MHz, $CDCl_3$): δ = 176.08 (-<u>C</u>OCO_2*i*Pr/-<u>C</u>OCONHBu), 162.99 (-<u>C</u>O_2*i*Pr), 161.50 (-<u>C</u>ONHBu), 149.37 (Ar<u>C</u>$_β$), 147.46 (Ar<u>C</u>$_β$), 139.28 (Ar<u>C</u>$_β$), 139.04 (Ar<u>C</u>$_β$), 122.86 (Ar<u>C</u>$_α$), 120.99 (Ar<u>C</u>$_α$), 113.66 (Ar<u>C</u>$_α$), 110.83 (Ar<u>C</u>$_α$), 70.70 (-<u>C</u>HMe_2), 65.52 (-O<u>C</u>H$_2$-), 65.32 (-O<u>C</u>H$_2$-), 64.72 (-O<u>C</u>H$_2$-), 64.62 (-O<u>C</u>H$_2$-), 39.25 (-<u>C</u>H$_2$NH-), 31.30 (-<u>C</u>H$_2$$CH_2$NH-), 21.62 (-CH<u>Me</u>$_2$), 20.07 (-<u>C</u>H$_2$$CH_3$), 13.72 (-$CH_2$<u>C</u>H$_3$) ppm; IR ($υ_{max}$, film): 2959, 2935, 2876, 1720, 1677, 1635, 1470, 1441, 1359,

1260, 1221, 1091, 1072 cm^{-1}; HRMS *m/z* (ESI+): Found: 524.1052 (M+H), Calc.: 524.1049; m.p. = 262-265 °C;

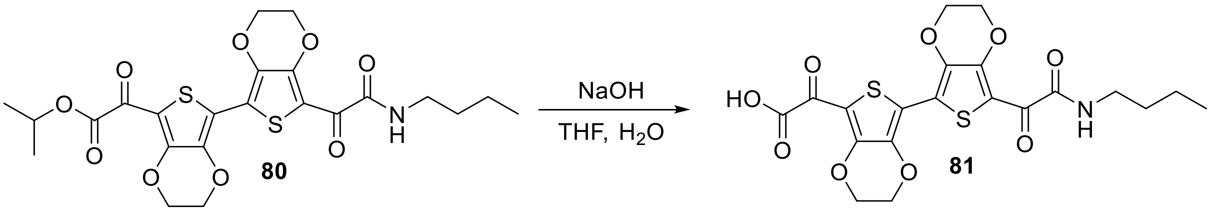

Sodium hydroxide (2 M, 3 mL) was added to a solution of hetero-bifunctional EDOT-dimer **80** (40 mg, 76 µmol) in THF (10 mL). After stirring for 2 hrs the mixture was diluted with DCM (100 mL) and washed with hydrochloric acid (1 M, 100 mL). The organics were dried with $MgSO_4$, filtered and concentrated *in vacuo* to afford the DP as an orange solid which was used directly in further manipulations.

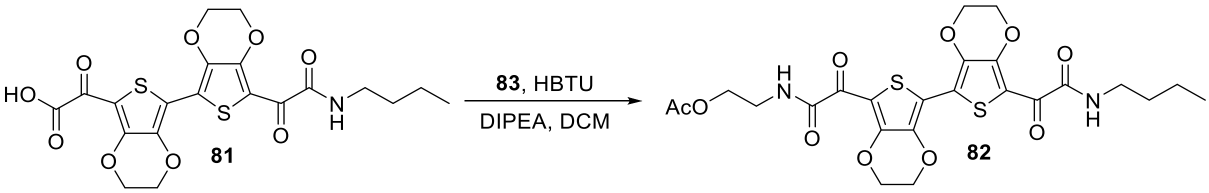

HBTU (58 mg, 152 µmol) and DIPEA (66 µL, 380 µmol) were added to a suspension of EDOT dimer **81** (36 mg, 76 µmol) in DCM (10 mL). After 5 min complete dissolution had occurred at which point amine **83** (21 mg, 152 µmol) was added. After stirring for 4 hrs, the mixture was diluted with DCM (50 mL) and the organics washed with hydrochloric acid (1 M, 50 mL) and sat. $NaHCO_3$ (50 mL), dried with $MgSO_4$, filtered and concentrated *in vacuo*. The residue was purified by flash column chromatography eluting with 50-70 % EtOAc:Hexane. Pure fractions were concentrated *in vacuo* to give the DP as an orange-yellow solid. A yield of 41 mg, 72 µmol (95 %) was obtained. ^{1}H NMR (400 MHz, $CDCl_3$): δ = 7.67 (1H, br t, *J* = 6.2 Hz, -N$\underline{H}$EtOAc), 7.43 (1H, br t, *J* = 6.4 Hz, -N$\underline{H}$Bu), 4.44-4.56 (8H, m, -OC$\underline{H}_2$-), 4.25 (2H, t, *J* = 5.3 Hz, -C$\underline{H}_2$OAc), 3.66 (2H, dt, *J* = 6.2, 5.3 Hz, -C$\underline{H}_2$CH$_2$OAc), 3.39 (2H, dt, J_1 = J_2 = 7.0 Hz, -C$\underline{H}_2$CH$_2$CH$_2$CH$_3$), 2.11 (3H, s, -O$\underline{Ac}$), 1.54-1.67 (2H, m, -C$\underline{H}_2$CH$_2$CH$_3$), 1.35-1.44 (2H, m, -C$\underline{H}_2$CH$_3$), 0.96 (3H, t, *J* = 7.3 Hz, -CH$_2$C$\underline{H}_3$) ppm; ^{13}C NMR (100 MHz, $CDCl_3$): δ = 176.11 (-$\underline{C}$OCONHR), 175.51 (-$\underline{C}$OCONHR), 170.8 (-O$\underline{C}$OCH$_3$), 161.80 (-$\underline{C}$ONHR), 161.52 (-$\underline{C}$ONHR), 149.61 (Ar$\underline{C}_β$), 149.38 (Ar$\underline{C}_β$), 139.33 (Ar$\underline{C}_β$), 139.29 (Ar$\underline{C}_β$), 123.14 (Ar$\underline{C}_α$), 111.02 (Ar$\underline{C}_α$), 110.75 (Ar$\underline{C}_α$), 65.55 (-O$\underline{C}$H$_2$-), 65.51 (-O$\underline{C}$H$_2$-), 64.63 (-O$\underline{C}$H$_2$-), 62.60 (-$\underline{C}$H$_2$OAc), 39.25 (-$\underline{C}$H$_2$NH-), 38.50 (-$\underline{C}$H$_2$CH$_2$OAc), 31.31 (-$\underline{C}$H$_2$CH$_2$NH-), 20.81 (-CO$\underline{C}$H$_3$), 20.07 (-$\underline{C}$H$_2$CH$_3$), 13.70 (-CH$_2$$\underline{C}H_3$) ppm; IR ($υ_{max}$, solid): 3306, 2926,

2855, 1736, 1659, 1624, 1523, 1477, 1427, 1363, 1293, 1228, 1139, 1084, 1054, 1023 cm^{-1}; HRMS *m/z* (ESI+): Found: 567.1115 (M+H), Calc.: 567.1110; m.p. > 350 ˚C;

3,4-Dimethoxythiophene functionalisation

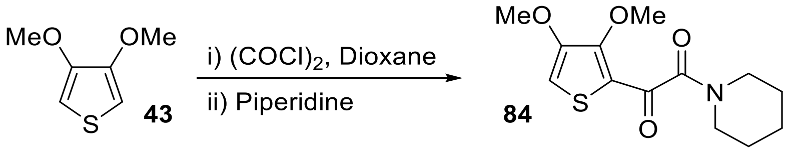

Oxalyl chloride (1.42 mL, 16.8 mmol) was added drop-wise to a solution of 3,4-dimethoxythiophene **43** (2 mL, 16.8 mmol) in dioxane (30 mL). The mixture was heated to 100 ºC for 90 min then allowed to cool to room temperature. Piperidine (8.3 mL, 84 mmol) was then added and the mixture stirred for 3 hrs. After this time the mixture was diluted with DCM (200 mL), and the organics washed with water (100 mL) and brine (100 mL), dried with $MgSO_4$, filtered and concentrated *in vacuo*. The residue was purified by flash column chromatography eluting with 20-50 % EtOAc:Hexane. Pure fractions were concentrated *in vacuo* to give the DP as a cream solid upon standing. A yield of 2.9 g, 10.2 mmol (61 %) was obtained. ^1H NMR (400 MHz, $CDCl_3$): δ = 6.72 (1H, s, ArH5), 3.96 (3H, s, ArC4-OMe), 3.86 (3H, s, ArC3-OMe), 3.62 (2H, dd, *J* = 6.3, 4.1 Hz, -NCH_2-), 3.26-3.34 (2H, m, -NCH_2-), 1.61-1.71 (4H, m, -$CH_2CH_2CH_2N$-), 1.54-1.60 (2H, m, -CH_2CH_2N-) ppm; ^{13}C NMR (100 MHz, $CDCl_3$): δ = 183.89 (-COCON-), 165.47 (-CON-), 152.32 (ArC4), 150.61 (ArC3), 123.15 (ArC2), 107.63 (ArC5), 61.00 (ArC4-OMe), 57.67 (ArC3-OMe), 46.86 (-CH_2N-), 42.02 (-CH_2N-), 25.71 (-$CH_2CH_2CH_2N$-), 25.10 (-$CH_2CH_2CH_2N$-), 24.49 (-$CH_2CH_2CH_2N$-) ppm; IR ($υ_{max}$, solid): 3078, 2934, 2865, 1631, 1491, 1453, 1427, 1398, 1370, 1301, 1281, 1264, 1254, 1240, 1213, 1145, 1124, 1052 cm^{-1}; HRMS *m/z* (ESI+): Found: 284.0951 (M+H), Calc.: 284.0957; m.p. = 93-94 °C;

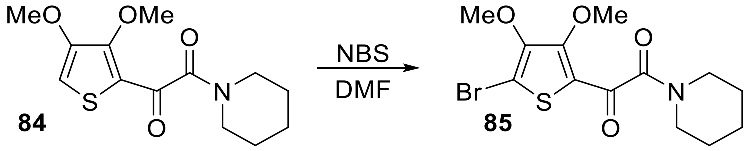

N-Bromosuccinimide (1.13 g, 6.4 mmol) was added in the dark to a solution of dimethoxythiophene derivative **84** (1.5 g, 5.3 mmol) in DMF (8 mL) (N.b. The reaction was initially run in a mixture of THF and AcOH as described above. However the reaction was extremely sluggish, even after addition of a further equivalent of NBS). After stirring for 1 hr the mixture was diluted with DCM (150 mL) and the

organics washed with water (100 mL) and sat. $NaHCO_3$ (2 x 100 mL), dried with $MgSO_4$, filtered and concentrated *in vacuo*. The residue was purified by flash column chromatography eluting with 20-50 % EtOAc:Hexane. Pure fractions were concentrated *in vacuo* and the residue dissolved in diethyl ether (100 mL). The organics were washed with brine (2 x 100 mL), dried with $MgSO_4$, filtered and concentrated *in vacuo* to give the DP as a light yellow oil. A yield of 1.8 g, 5.0 mmol (94 %) was obtained. ^{1}H NMR (400 MHz, $CDCl_3$): δ = 4.00 (3H, s, ArC4-OMe), 3.94 (3H, s, ArC3-OMe), 3.63 (2H, dd, *J* = 6.3, 4.3 Hz, -NCH$_2$-), 3.27-3.35 (2H, m, -NCH$_2$-), 1.62-1.76 (4H, m, -CH$_2$CH$_2$CH$_2$N-), 1.54-1.62 (2H, m, -CH$_2$CH$_2$N-) ppm; ^{13}C NMR (100 MHz, $CDCl_3$): δ = 182.23 (-COCON-), 164.97 (-CON-), 154.66 (ArC4), 148.56 (ArC3), 122.81 (ArC2), 112.39 (ArC5), 65.85 (ArC4-OMe), 61.18 (ArC3-OMe), 46.93 (-CH$_2$N-), 42.14 (-CH$_2$N-), 25.84 (-CH$_2$CH$_2$CH$_2$N-), 25.14 (-CH$_2$CH$_2$CH$_2$N-), 24.47 (-CH$_2$CH$_2$CH$_2$N-) ppm; IR ($υ_{max}$, oil): 2939, 2857, 1636, 1486, 1444, 1417, 1374, 1295, 1249, 1228, 1120, 1049, 1000 cm^{-1}; HRMS *m/z* (ESI+): Found: 362.0070/364.0040 (M+H), Calc.: 362.0062/364.0042;

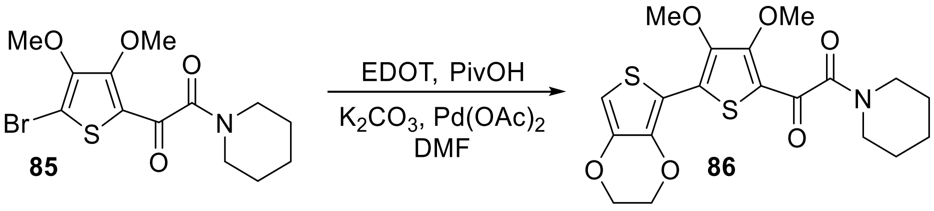

Brominated-dimethoxy thiophene **85** (0.5 g, 1.76 mmol), pivalic acid (90 mg, 0.88 mmol), palladium (II) acetate (20 mg, 0.09 mmol) and potassium carbonate (2.4 g, 17.6 mmol) were charged under nitrogen. Dry DMF (7 mL) and EDOT **1** (0.75 mL, 7.07 mmol) were then added and the mixture heated to 90 ºC for 1 hr. After cooling to rt the mixture was diluted with DCM (150 mL) and washed with water (2 x 100 mL) and brine (100 mL). The organics were dried with $MgSO_4$, filtered and concentrated *in vacuo*. The residue was purified by flash column chromatography eluting with 2 % NEt_3 in 20-70 % EtOAc:Hexane. Pure fractions were concentrated *in vacuo* and the residue dissolved in diethyl ether (100 mL). The organics were washed with brine (2 x 100 mL), dried with $MgSO_4$, filtered and concentrated *in vacuo* to give the DP as a yellow oil. A yield of 490 mg, 1.35 mmol (77 %) was obtained. ^{1}H NMR (400 MHz, $CDCl_3$): δ = 6.47 (1H, s, ArH10), 4.36-4.43 (2H, m, -OCH$_2$-), 4.21-4.30 (2H, m, -OCH$_2$-), 4.01 (3H, s, ArC4-OMe), 3.90 (3H, s, ArC3-OMe), 3.65 (2H, dd, *J* = 6.3, 4.1 Hz, -NCH$_2$-), 3.29-3.39 (2H, m, -NCH$_2$-), 1.62-1.73 (4H, m, -CH$_2$CH$_2$CH$_2$N-), 1.55-1.62 (2H, m, -CH$_2$CH$_2$N-) ppm; ^{13}C NMR (100 MHz, $CDCl_3$): δ = 183.07 (-COCON-), 165.68 (-CON-), 155.50 (ArC4), 144.10 (ArC3), 141.36 (ArC8), 140.72 (ArC9), 131.18 (ArC5), 118.96 (ArC2), 108.78 (ArC7), 102.12 (ArC10), 65.34 (ArC9-OCH$_2$-), 64.52 (ArC8-

O$\underline{C}H_2$-), 61.01 (ArC4-O$\underline{Me}$), 60.55 (ArC3-O$\underline{Me}$), 47.02 (-C$\underline{H}_2$N-), 42.07 (-C$\underline{H}_2$N-), 25.84 (-$\underline{C}H_2\underline{C}H_2CH_2$N-), 25.17 (-$\underline{C}H_2\underline{C}H_2CH_2$N-), 24.55 (-$\underline{C}H_2\underline{C}H_2CH_2$N-) ppm; IR ($\upsilon_{max}$, solid): 2926, 2856, 2228, 1638, 1595, 1572, 1533, 1500, 1469, 1432, 1395, 1364, 1324, 1282, 1262, 1248, 1221, 1183, 1150, 1137, 1114, 1078, 1051, 1015 cm^{-1}; HRMS *m/z* (ESI+): Found: 424.0880 (M+H), Calc.: 424.0889; m.p. = 82-87 °C;

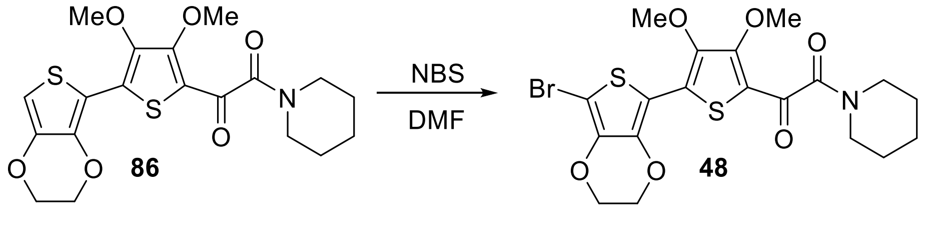

N-Bromosuccinimide (227 mg, 1.28 mmol) was added to a solution of EDOT-dimethoxythiophene dimer **86** (450 mg, 1.06 mmol) in DMF (5 mL) in the dark. After 1 hr the mixture was diluted with diethyl ether (100 mL) and the organics washed with water (100 mL) and brine (2 x 100 mL), dried with $MgSO_4$, filtered and concentrated *in vacuo*. The product was sufficiently pure for further applications. An analytical sample was obtained via column chromatography eluting with 2 % NEt_3 in 50 % EtOAc:Hexane. A yield of 410 mg, 0.82 mmol (64 %) was obtained as a yellow solid. ^{1}H NMR (400 MHz, $CDCl_3$): δ = 4.39-4.43 (2H, m, -OC$\underline{H}_2$-), 4.34-4.38 (2H, m, -OC$\underline{H}_2$-), 4.02 (3H, s, ArC4-O$\underline{Me}$), 3.92 (3H, s, ArC3-O$\underline{Me}$), 3.66 (2H, t, *J* = 5.1 Hz, -NC$\underline{H}_2$-), 3.32-3.40 (2H, m, -NC$\underline{H}_2$-), 1.63-1.74 (4H, m, -C$\underline{H}_2$C$\underline{H}_2CH_2$N-), 1.56-1.63 (2H, m, -C$\underline{H}_2CH_2$N-) ppm; ^{13}C NMR (100 MHz, $CDCl_3$): δ = 183.05 (-$\underline{C}$OCON-), 165.58 (-$\underline{C}$ON-), 155.26 (Ar$\underline{C}$4), 144.18 (Ar$\underline{C}$3), 139.75 (Ar$\underline{C}_\beta$), 139.70 (Ar$\underline{C}_\beta$), 130.16 (Ar$\underline{C}$5), 119.33 (Ar$\underline{C}$2), 109.05 (Ar$\underline{C}$7), 91.26 (Ar$\underline{C}$10), 65.28 (ArC9-O$\underline{C}H_2$-), 64.99 (ArC8-O$\underline{C}H_2$-), 61.09 (ArC4-O$\underline{Me}$), 60.57 (ArC3-O$\underline{Me}$), 47.03 (-C$\underline{H}_2$N-), 42.11 (-C$\underline{H}_2$N-), 25.87 (-$\underline{C}H_2\underline{C}H_2CH_2$N-), 25.18 (-$\underline{C}H_2\underline{C}H_2CH_2$N-), 24.55 (-$\underline{C}H_2\underline{C}H_2CH_2$N-) ppm; IR ($\upsilon_{max}$, solid): 2937, 2844, 1651, 1606, 1540, 1429, 1395, 1355, 1318, 1264, 1218, 1087, 1052 cm^{-1}; HRMS *m/z* (ESI+): Found: 501.9980/503.9965 (M+H), Calc.: 501.9994/503.9974; m.p. = 222-225 °C;

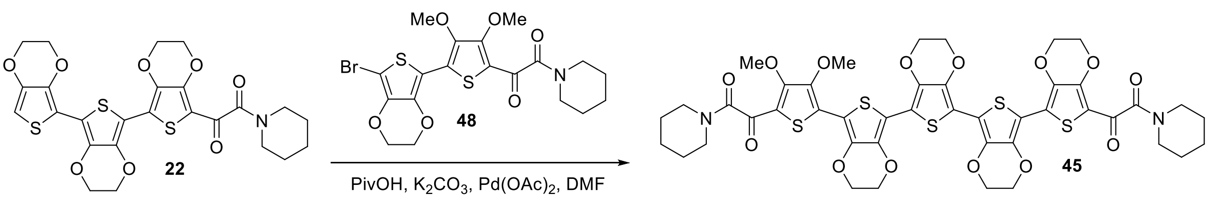

Piperidine-EDOT trimer **22** (80 mg, 142 µmol), brominated EDOT-dimethoxythiophene dimer **48** (50 mg, 159 µmol), pivalic acid (7 mg, 71 µmol), palladium (II) acetate (3 mg, 14 µmol) and potassium

carbonate (195 mg, 1420 μmol) were charged under nitrogen. Dry DMF (3 mL) was then added and the mixture heated to 130 ºC for 1 hr. After cooling to rt the mixture was diluted with DCM (50 mL) and washed with water (2 x 50 mL) and brine (50 mL). The organics were dried with $MgSO_4$, filtered and concentrated *in vacuo*. The residue was purified by flash column chromatography eluting with 2 % MeOH:DCM. Pure fractions were concentrated *in vacuo* and the residue triturated in diethyl ether (2 x 50 mL) to give the DP as a purple solid. A yield of 51 mg, 52 μmol (37 %) was obtained. ^{1}H NMR (400 MHz, $CDCl_3$): δ = 4.26-4.58 (16H, m, -OC<u>H</u>$_2$-), 4.04 (3H, s, -O<u>Me</u>), 3.96 (3H, s, -O<u>Me</u>), 3.63-3.71 (4H, m, -C<u>H</u>$_2$N-), 3.34-3.44 (2H, m, -C<u>H</u>$_2$N-), 1.65-1.74 (8H, m, -C<u>H</u>$_2$C<u>H</u>$_2$CH$_2$N-), 1.56-1.64 (4H, m, -C<u>H</u>$_2$CH$_2$N-) ppm; IR (υ$_{max}$, solid): 2927, 2857, 1622, 1434, 1394, 1359, 1316, 1218, 1151, 1068, 1057, 1013 cm^{-1}; HRMS *m/z* (ESI+): Found: 983.1295 (M+H), Calc.: 983.1318; m.p. > 350 °C;

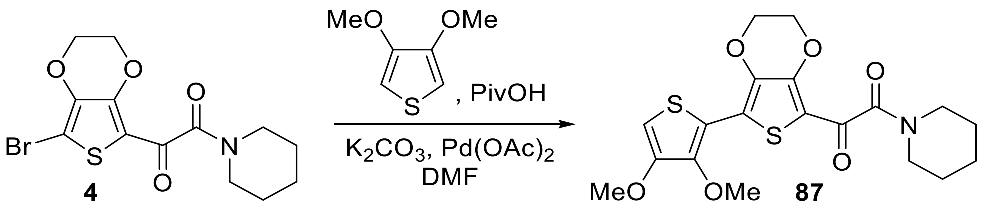

Brominated piperidine-EDOT monomer **4** (0.5 g, 1.4 mmol), pivalic acid (71 mg, 0.7 mmol), palladium (II) acetate (16 mg, 0.07 mmol) and potassium carbonate (1.9 g, 14 mmol) were charged under nitrogen. Dry DMF (5 mL) and 3,4-dimethoxythiophene **43** (0.67 mL, 5.6 mmol) were then added and the mixture heated to 130 ºC for 1 hr. After cooling to rt the mixture was diluted with DCM (150 mL) and washed with water (2 x 100 mL) and brine (100 mL). The organics were dried with $MgSO_4$, filtered and concentrated *in vacuo*. The residue was purified by flash column chromatography eluting with 2 % NEt_3 in 50-80 % EtOAc:Hexane. Pure fractions were concentrated *in vacuo* and the residue triturated in diethyl ether (2 x 100 mL) to give the DP as an orange solid. A yield of 330 mg, 0.78 mmol (56 %) was obtained. ^{1}H NMR (400 MHz, $CDCl_3$): δ = 6.32 (1H, s, Ar<u>H</u>10), 4.41 (4H, m, -OC<u>H</u>$_2$-), 3.99 (3H, s, ArC9-O<u>Me</u>), 3.88 (3H, s, ArC8-O<u>Me</u>), 3.65 (2H, t, *J* = 5.2 Hz, -NC<u>H</u>$_2$-), 3.34-3.43 (2H, m, -NC<u>H</u>$_2$-), 1.62-1.73 (4H, m, -C<u>H</u>$_2$C<u>H</u>$_2$CH$_2$N-), 1.54-1.62 (2H, m, -C<u>H</u>$_2$CH$_2$N-) ppm; ^{13}C NMR (100 MHz, $CDCl_3$): δ = 182.18 (-<u>C</u>OCON-), 165.53 (-<u>C</u>ON-), 150.53 (Ar<u>C</u>8), 146.46 (Ar<u>C</u>$_β$), 145.13 (Ar<u>C</u>9), 136.85 (Ar<u>C</u>$_β$), 122.56 (Ar<u>C</u>5), 116.42 (Ar<u>C</u>7), 112.80 (Ar<u>C</u>2), 98.09 (Ar<u>C</u>10), 65.48 (-O<u>C</u>H$_2$-), 64.51 (-O<u>C</u>H$_2$-), 60.13 (ArC9-O<u>Me</u>), 57.40 (ArC8-O<u>Me</u>), 47.02 (-C<u>H</u>$_2$N-), 42.15 (-C<u>H</u>$_2$N-), 26.03 (-<u>C</u>H$_2$CH$_2$CH$_2$N-), 25.25 (-<u>C</u>H$_2$<u>C</u>H$_2$CH$_2$N-), 24.55 (-<u>C</u>H$_2$<u>C</u>H$_2$CH$_2$N-) ppm; IR (υ$_{max}$, solid): 3107, 2931, 1636, 1605, 1548, 1504,

1471, 1445, 1403, 1363, 1314, 1262, 1251, 1224, 1208, 11187, 1091, 1041, 1003 cm^{-1}; HRMS *m/z* (ESI+): Found: 424.0877 (M+H), Calc.: 424.0889; m.p. = 214-218 °C;

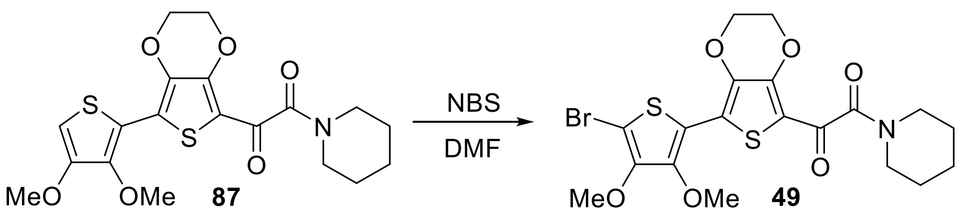

N-Bromosuccinimide (151 mg, 0.85 mmol) was added to a solution of dimethoxythiophene-EDOT dimer **87** (300 mg, 0.71 mmol) in DMF (3 mL) in the dark. After 1 hr the mixture was diluted with DCM (100 mL) and the organics washed with water (100 mL) and brine (2 x 100 mL), dried with $MgSO_4$, filtered and concentrated *in vacuo*. The product was sufficiently pure for further applications. An analytical sample was obtained via column chromatography eluting with 2 % NEt_3 in 60-90 % EtOAc:Hexane. A yield of 411 mg, 0.82 mmol (96 %) was obtained as a yellow oil. ^{1}H NMR (400 MHz, $CDCl_3$): δ = 4.34-4.43 (4H, m, -OC<u>H</u>$_2$-), 3.96 (3H, s, -O<u>Me</u>), 3.93 (3H, s, -O<u>Me</u>), 3.61 (2H, t, *J* = 5.3 Hz, -NC<u>H</u>$_2$-), 3.31-3.39 (2H, m, -NC<u>H</u>$_2$-), 1.60-1.72 (4H, m, -C<u>H</u>$_2$C<u>H</u>$_2$CH$_2$N-), 1.51-1.59 (2H, m, -C<u>H</u>$_2$CH$_2$N-) ppm; ^{13}C NMR (100 MHz, $CDCl_3$): δ = 182.13 (-<u>C</u>OCON-), 165.37 (-<u>C</u>ON-), 148.23 (Ar<u>C</u>$_\beta$), 146.84 (Ar<u>C</u>$_\beta$), 146.37 (Ar<u>C</u>$_\beta$), 136.88 (Ar<u>C</u>$_\beta$), 121.35 (Ar<u>C</u>$_\alpha$), 116.66 (Ar<u>C</u>$_\alpha$), 112.81 (Ar<u>C</u>$_\alpha$), 99.82 (Ar<u>C</u>10), 65.53 (-O<u>C</u>H$_2$-), 64.63 (-O<u>C</u>H$_2$-), 60.92 (-O<u>Me</u>), 60.39 (-O<u>Me</u>), 46.98 (-C<u>H</u>$_2$N-), 42.13 (-C<u>H</u>$_2$N-), 26.02 (-<u>C</u>H$_2$<u>C</u>H$_2$CH$_2$N-), 25.22 (-<u>C</u>H$_2$<u>C</u>H$_2$CH$_2$N-), 24.49 (-<u>C</u>H$_2$<u>C</u>H$_2$CH$_2$N-) ppm; IR (υ_{max}, solid): 2935, 2852, 1651, 1604, 1550, 1504, 1472, 1441, 1361, 1312, 1281, 1263, 1221, 1113, 1089, 1047, 1009 cm^{-1}; HRMS *m/z* (ESI+): Found: 501.9970/503.9957 (M+H), Calc.: 501.9994/503.9974; m.p. = 206-211 °C;

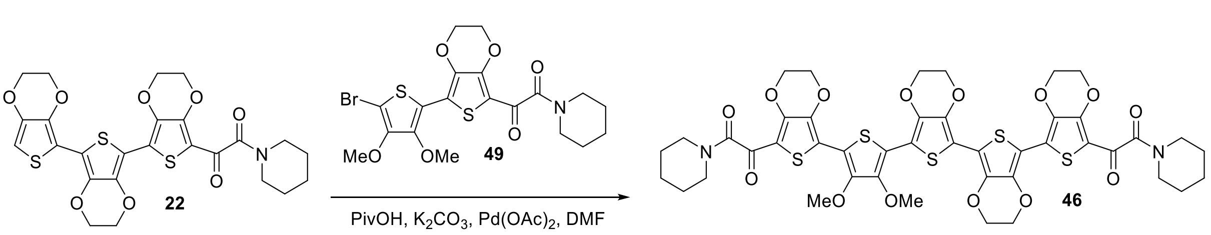

Piperidine-EDOT trimer **22** (50 mg, 89 µmol), brominated dimethoxythiophene-EDOT dimer **49** (50 mg, 100 µmol), pivalic acid (5 mg, 44 µmol), palladium (II) acetate (2 mg, 9 µmol) and potassium carbonate (123 mg, 890 µmol) were charged under nitrogen. Dry DMF (3 mL) was then added and the mixture heated to 130 °C for 1.5 hrs. After cooling to rt the mixture was diluted with DCM (50 mL) and washed with water (2 x 50 mL) and brine (50 mL). The organics were dried with $MgSO_4$, filtered and

concentrated *in vacuo*. The residue was purified by flash column chromatography eluting with 2-5 % MeOH:DCM. Pure fractions were concentrated *in vacuo* and the residue triturated in diethyl ether (2 x 50 mL) to give the DP as a purple solid. A yield of 55 mg, 56 μmol (63 %) was obtained. ^{1}H NMR (400 MHz, $CDCl_3$): δ = 4.33-4.54 (16H, m, -OC$\underline{H}_2$-), 3.94-4.10 (6H, m, -O$\underline{Me}$), 3.62-3.70 (4H, m, -C$\underline{H}_2$N-), 3.36-3.46 (2H, m, -C$\underline{H}_2$N-), 1.63-1.75 (8H, m, -C$\underline{H}_2$C$\underline{H}_2$CH_2N-), 1.55-1.63 (4H, m, -C$\underline{H}_2$CH_2N-) ppm; IR (υ_{max}, solid): 2924, 2872, 2855, 1732, 1634, 1601, 1468, 1427, 1359, 1248, 1217, 1138, 1117, 1066, 1021 cm^{-1}; HRMS *m/z* (ESI+): Found: 983.1321 (M+H), Calc.: 983.1318; m.p. > 350 °C;

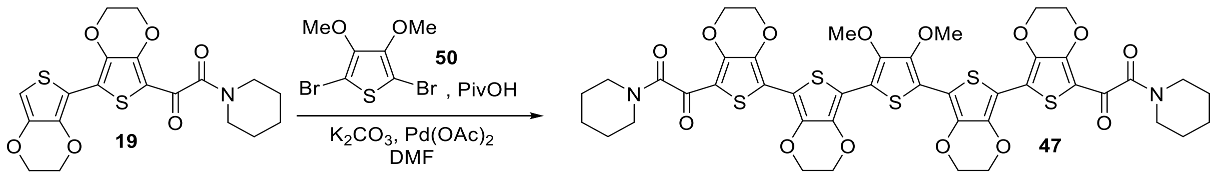

Piperidine-EDOT dimer **19** (40 mg, 95 μmol), pivalic acid (5 mg, 48 μmol), palladium (**II**) acetate (2 mg, 10 μmol) and potassium carbonate (131 mg, 950 μmol) were charged under nitrogen. Dry DMF (1.5 mL) and 2,5-dibromo-3,4-dimethoxythiophene **50** (14 mg, 48 μmol) were then added and the mixture heated to 130 ºC for 1 hr. After cooling to rt the mixture was diluted with DCM (50 mL) and washed with water (2 x 50 mL) and brine (50 mL). The organics were dried with $MgSO_4$, filtered and concentrated *in vacuo*. The residue was purified by flash column chromatography eluting with 1-5 % MeOH:DCM. Pure fractions were concentrated *in vacuo* and the residue triturated in diethyl ether (2 x 50 mL) to give the DP as a purple solid. A yield of 28 mg, 28 μmol (59 %) was obtained. ^{1}H NMR (400 MHz, $CDCl_3$): δ = 4.33-4.54 (16H, m, -OC$\underline{H}_2$-), 4.02 (6H, s, ArC4-O$\underline{Me}$), 3.65 (2H, t, *J* = 6.0 Hz, -NC$\underline{H}_2$-), 3.40 (2H, t, *J* = 5.5 Hz, -NC$\underline{H}_2$-), 1.62-1.75 (8H, m, -C$\underline{H}_2$C$\underline{H}_2$CH_2N-), 1.54-1.62 (4H, m, -C$\underline{H}_2$CH_2N-) ppm; IR (υ_{max}, solid): 2931, 2854, 1650, 1602, 1540, 1488, 1463, 1421, 1358, 1309, 1249, 1217, 1135, 1116, 1067, 1022 cm^{-1}; HRMS *m/z* (ESI+): Found: 983.1467 (M+H), Calc.: 983.1466; m.p. > 350 °C;

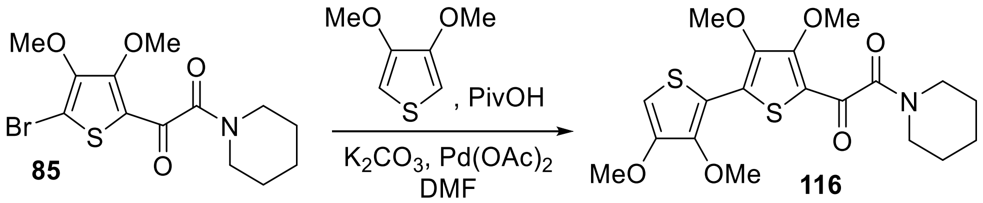

Brominated piperidine-DMT monomer **85** (400 mg, 1.1 mmol), pivalic acid (56 mg, 0.55 mmol), palladium (II) acetate (12 mg, 0.06 mmol) and potassium carbonate (1.5 g, 11 mmol) were charged under nitrogen. Dry DMF (5 mL) and 3,4-dimethoxythiophene **45** (523 μL, 4.4 mmol) were then added

and the mixture heated to 90 ºC for 1 hr. After cooling to rt the mixture was diluted with DCM (150 mL) and washed with water (2 x 100 mL) and brine (100 mL). The organics were dried with $MgSO_4$, filtered and concentrated *in vacuo*. The residue was purified by flash column chromatography eluting with 50 % EtOAc:Hexane. Pure fractions were concentrated *in vacuo* and the residue triturated in diethyl ether (2 x 100 mL) to give the DP as a yellow oil. A yield of 170 mg, 0.40 mmol (36 %) was obtained. The product was utilised directly in the subsequent formation of penta-DMT construct **57** without further purification or analysis.

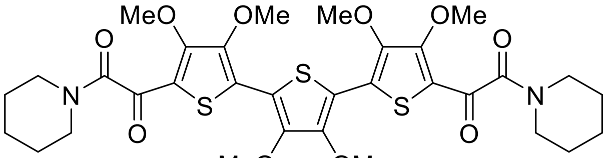

117

117 was obtained as a side product from the above reaction for the synthesis of **116**. The product was obtained as an orange solid. A yield of 110 mg, 156 µmol (28 %) was obtained. ^{1}H NMR (400 MHz, $CDCl_3$): δ = 4.00-4.01 (12H, m, $-OC\underline{H}_3$), 3.96 (6H, ArC8-$OC\underline{H}_3$), 3.65 (4H, t, J = 5.1 Hz, $-NC\underline{H}_2-$), 3.29-3.42 (4H, m, $-NC\underline{H}_2-$), 1.64-1.75 (8H, m, $-NCH_2C\underline{H}_2C\underline{H}_2-$), 1.54-1.64 (4H, m, $-NCH_2C\underline{H}_2-$) ppm; ^{13}C NMR (100 MHz, $CDCl_3$): δ = 183.27 ($-\underline{C}OCONR_2$), 165.50 ($-\underline{C}ONR_2$), 155.30 ($Ar\underline{C}_\beta$), 148.18 ($Ar\underline{C}_\beta$), 145.43 ($Ar\underline{C}_\beta$), 129.62 ($Ar\underline{C}_\alpha$), 120.75 ($Ar\underline{C}_\alpha$), 118.27 ($Ar\underline{C}_\alpha$), 109.19 ($Ar\underline{C}_\alpha$), 61.11 ($-O\underline{C}H_3$), 60.46 (ArC8-$O\underline{C}H_3$), 60.20 ($-O\underline{C}H_3$), 47.02 ($-N\underline{C}H_2-$), 42.11 ($-N\underline{C}H_2-$), 25.86 ($-NCH_2\underline{C}H_2-$), 25.17 ($-NCH_2\underline{C}H_2\underline{C}H_2-$), 24.53 ($-NCH_2\underline{C}H_2\underline{C}H_2-$) ppm; IR ($\upsilon_{max}$, solid): 2934, 2852, 1626, 1466, 1441, 1390, 1321, 1283, 1250, 1227, 1194, 1110, 1015 cm^{-1}; HRMS *m/z* (ESI+): Found: 743.1775 (M+H), Calc.: 743.1767; m.p. = 115-120 °C;

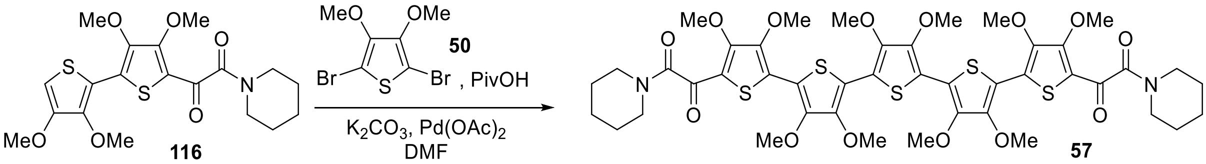

Piperidine-DMT dimer **116** (170 mg, 400 µmol), pivalic acid (20 mg, 200 µmol), palladium (II) acetate (9 mg, 40 µmol) and potassium carbonate (552 mg, 4 mmol) were charged under nitrogen. Dry DMF (3 mL) and 2,5-dibromo-3,4-dimethoxythiophene **50** (84 mg, 280 µmol) were then added and the mixture heated to 90 ºC for 1 hr. After cooling to rt the mixture was diluted with DCM (100 mL) and washed with water (2 x 75 mL) and brine (75 mL). The organics were dried with $MgSO_4$, filtered and concentrated *in vacuo*. The residue was purified by flash column chromatography eluting with 50-100 % EtOAc:Hexane. Pure fractions were concentrated *in vacuo* and the residue triturated in water (2 x 80 mL) to give the

DP as a red solid. A yield of 98 mg, 99 μmol (49 %) was obtained. ^{1}H NMR (400 MHz, $CDCl_3$): δ = 4.01-4.07 (24H, m, -OC$\underline{H}_3$), 3.98 (6H, s, -OC$\underline{H}_3$), 3.65-3.71 (4H, m, -NC$\underline{H}_2$-), 3.38 (4H, t, *J* = 5.3 Hz, -NC$\underline{H}_2$-), 1.65-1.75 (8H, m, -NCH$_2$C$\underline{H}_2$C$\underline{H}_2$-), 1.57-1.65 (4H, m, -NCH$_2$C$\underline{H}_2$-) ppm; IR ($υ_{max}$, solid): 2932, 2851, 1624, 1462, 1392, 1318, 1281, 1256, 1221, 1194, 1104, 1007 cm^{-1}; HRMS *m/z* (ESI+): Found: 991.1965 (M+H), Calc.: 991.1944; m.p. = 220-224 °C;

3,4-Propylenedioxythiophene functionalisation

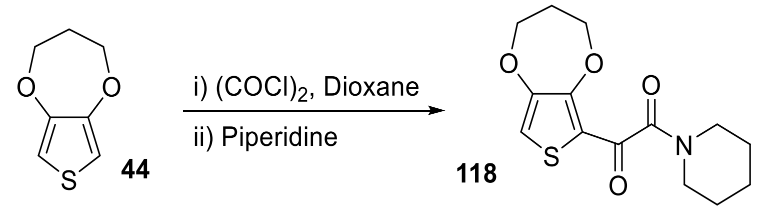

Oxalyl chloride (163 µL, 1.92 mmol) was added drop-wise to a solution of 3,4-propylenedioxythiophene **44** (300 mg, 1.92 mmol) in dioxane (10 mL). The mixture was heated to 100 ºC for 90 min then allowed to cool to room temperature. Piperidine (948 µL, 9.6 mmol) was then added and the mixture stirred for 3 hrs. After this time the mixture was diluted with DCM (200 mL), and the organics washed with water (100 mL) and brine (100 mL), dried with $MgSO_4$, filtered and concentrated *in vacuo*. The residue was purified by flash column chromatography eluting with 20-50 % EtOAc:Hexane. Pure fractions were concentrated *in vacuo* to give the DP as a white solid. A yield of 520 mg, 1.76 mmol (92 %) was obtained. ^{1}H NMR (400 MHz, $CDCl_3$): δ = 7.06 (1H, s, Ar$\underline{H}$), 4.20 (2H, t, *J* = 5.2 Hz, ArC4-OC$\underline{H}_2$-), 4.12 (2H, t, *J* = 5.3 Hz, ArC3-OC$\underline{H}_2$-), 3.58-3.68 (2H, m, -NC$\underline{H}_2$-), 3.31-3.39 (2H, m, -N$\underline{C}$H$_2$-), 2.22-2.23 (2H, m, -OCH$_2$C$\underline{H}_2$-), 1.55-1.67 (6H, m, -NCH$_2$C$\underline{H}_2$C$\underline{H}_2$-) ppm; ^{13}C NMR (100 MHz, $CDCl_3$): δ = 183.46 (-$\underline{C}$OCON-), 165.56 (-$\underline{C}$ON-), 155.44 (Ar$\underline{C}$4), 150.29 (Ar$\underline{C}$3), 121.66 (Ar$\underline{C}$2), 118.04 (Ar$\underline{C}$5), 71.72 (ArC4-O$\underline{C}$H$_2$-), 71.23 (ArC3-O$\underline{C}$H$_2$-), 46.90 (-C$\underline{H}_2$N-), 42.04 (-C$\underline{H}_2$N-), 33.08 (-OCH$_2\underline{C}$H$_2$-), 25.85 (-$\underline{C}$H$_2\underline{C}$H$_2$CH$_2$N-), 25.23 (-$\underline{C}$H$_2\underline{C}$H$_2$CH$_2$N-), 24.51 (-$\underline{C}$H$_2\underline{C}$H$_2$CH$_2$N-) ppm; IR ($υ_{max}$, solid): 3076, 2944, 2855, 1738, 1622, 1482, 1459, 1396, 1382, 1370, 1356, 1305, 1282, 1270, 1251, 1229, 1197, 1046, 1016 cm^{-1}; HRMS *m/z* (ESI+): Found: 296.0957 (M+H), Calc.: 296.0957; m.p. = 135-137 °C;

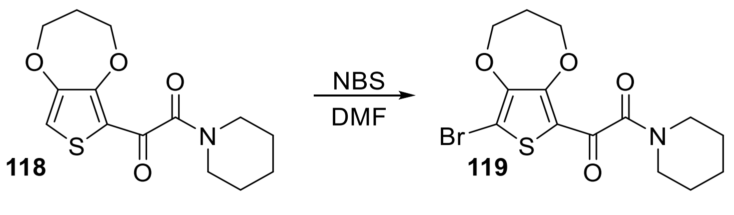

N-Bromosuccinimide (362 mg, 2.03 mmol) was added in the dark to a solution of ProDOT derivative **118** (500 mg, 1.69 mmol) in a mixture of acetic acid (15 mL) and THF (10 mL). After stirring for 18 hrs

the mixture was diluted with DCM (150 mL) and the organics washed with water (100 mL) and sat. $NaHCO_3$ (2 x 100 mL), dried with $MgSO_4$, filtered and concentrated *in vacuo*. The residue was purified by flash column chromatography eluting with 30-50 % EtOAc:Hexane. Pure fractions were concentrated *in vacuo* to give the DP as a light yellow oil. A yield of 508 mg, 1.36 mmol (81 %) was obtained. ^{1}H NMR (400 MHz, $CDCl_3$): δ = 4.26-4.15 (4H, m, -OC$\underline{H}_2$-), 3.62 (2H, t, *J* = 5.4 Hz, -NC$\underline{H}_2$-), 3.29-3.38 (2H, m, -N$\underline{C}H_2$-), 2.31 (2H, tt, *J* = 6.0, 4.7 Hz, -OCH$_2$C$\underline{H}_2$-), 1.53-1.7 (6H, m, -NCH$_2$C$\underline{H}_2$C$\underline{H}_2$-) ppm; ^{13}C NMR (100 MHz, $CDCl_3$): δ = 182.27 (-$\underline{C}$OCON-), 165.16 (-$\underline{C}$ON-), 154.24 (Ar$\underline{C}_\beta$), 148.27 (Ar$\underline{C}_\beta$), 120.99 (Ar$\underline{C}$2), 109.64 (Ar$\underline{C}$5), 72.01 (-O$\underline{C}$H$_2$-), 71.48 (-O$\underline{C}$H$_2$-), 46.92 (-C$\underline{H}_2$N-), 42.11 (-C$\underline{H}_2$N-), 32.86 (-OCH$_2\underline{C}$H$_2$-), 25.88 (-$\underline{C}$H$_2\underline{C}$H$_2$CH$_2$N-), 25.22 (-$\underline{C}$H$_2\underline{C}$H$_2$CH$_2$N-), 24.46 (-$\underline{C}$H$_2\underline{C}$H$_2$CH$_2$N-) ppm; IR (υ_{max}, solid): 2969, 2944, 2854, 1738, 1627, 1483, 1448, 1394, 1351, 1295, 1265, 1217, 1121, 1105, 1073, 1013 cm^{-1}; HRMS *m/z* (ESI+): Found: 374.0070/376.0040 (M+H), Calc.: 374.0062/374.0036; m.p. = 87-92 °C;

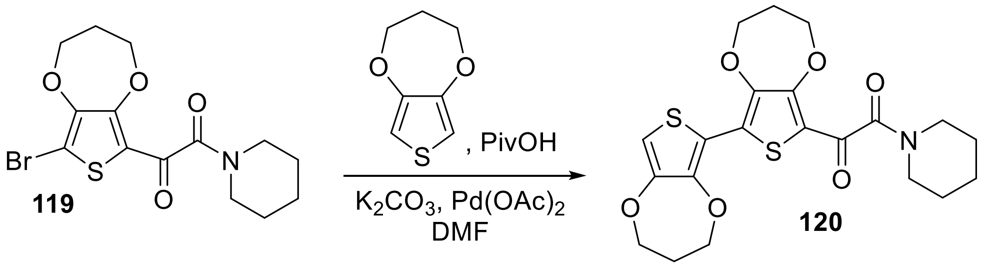

Brominated piperidine-ProDOT monomer **119** (200 mg, 0.53 mmol), pivalic acid (27 mg, 0.26 mmol), palladium (II) acetate (6 mg, 0.03 mmol), potassium carbonate (731 mg, 5.3 mmol) and ProDOT **44** (250 mg, 1.60 mmol) were charged under nitrogen. Dry DMF (4 mL) was then added and the mixture heated to 90 ºC for 1 hr. After cooling to rt the mixture was diluted with DCM (150 mL) and washed with water (2 x 100 mL) and brine (100 mL). The organics were dried with $MgSO_4$, filtered and concentrated *in vacuo*. The residue was purified by flash column chromatography eluting first with 40-80 % EtOAc:Hexane to elute the DP, then 0-10 % MeOH:EtOAc to elute SP **???** (see below). Pure fractions were concentrated *in vacuo* and the residue triturated in diethyl ether (2 x 100 mL) to give the DP as a yellow solid. A yield of 152 mg, 0.34 mmol (64 %) was obtained. ^{1}H NMR (400 MHz, $CDCl_3$): δ = 6.61 (1H, s, Ar$\underline{H}$10), 4.23 (2H, dd, *J* = 5.9, 4.4 Hz, -OC$\underline{H}_2$-), 4.16-4.21 (4H, m, -OC$\underline{H}_2$-), 4.10 (2H, dd, *J* = 5.8, 4.4 Hz, -OC$\underline{H}_2$-), 3.61 (2H, t, *J* = 5.3 Hz, -NC$\underline{H}_2$-), 3.28-3.37 (2H, m, -NC$\underline{H}_2$-), 2.21-2.35 (4H, m, -OCH$_2$C$\underline{H}_2$-), 1.60-1.72 (4H, m, -C$\underline{H}_2$C$\underline{H}_2$CH$_2$N-), 1.54-1.60 (2H, m, -C$\underline{H}_2$CH$_2$N-) ppm; ^{13}C NMR (100 MHz, $CDCl_3$): δ = 183.47 (-$\underline{C}$OCON-), 165.91 (-$\underline{C}$ON-), 155.03 (Ar$\underline{C}_\beta$), 149.95 (Ar$\underline{C}_\beta$), 148.55 (Ar$\underline{C}_\beta$),

144.61 (ArC_{β}), 128.53 (ArC_{α}), 117.25 (ArC_{α}), 115.04 (ArC_{α}), 108.07 (ArC_{α}), 71.77 (-OCH_2-), 71.33 (-OCH_2-), 71.18 (-OCH_2-), 71.08 (-OCH_2-), 46.93 (-CH_2N-), 41.97 (-CH_2N-), 33.48 (-OCH$_2$CH$_2$-), 33.02 (-OCH$_2$CH$_2$-), 25.84 (-CH$_2$CH$_2$CH$_2$N-), 25.23 (-CH$_2$CH$_2$CH$_2$N-), 24.52 (-CH$_2$CH$_2$CH$_2$N-) ppm; IR (υ_{max}, solid): 2969, 2922, 2864, 1738, 1631, 1603, 1535, 1495, 1470, 1439, 1421, 1398, 1357, 1319, 1285, 1267, 1250, 1216, 1180, 1079, 1047, 1023 cm^{-1}; HRMS *m/z* (ESI+): Found: 450.1059 (M+H), Calc.: 450.1045; m.p. = 184-190 °C;

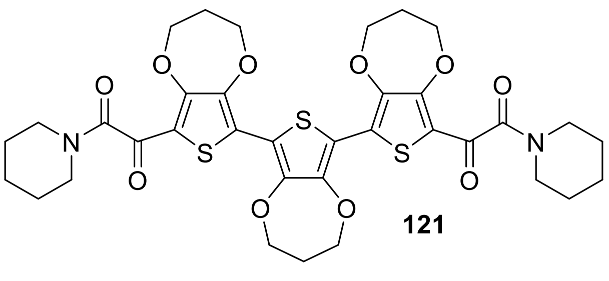

121

121 was obtained as a side product from the above reaction for the synthesis of **120**. The product was obtained as an orange solid. A yield of 20 mg, 27 µmol (10 %) was obtained. ^{1}H NMR (400 MHz, CDCl$_3$): δ = 4.15-4.34 (12H, m, -OCH_2-), 3.61 (4H, t, *J* = 5.3 Hz, -NCH_2-), 3.35 (4H, t, *J* = 5.4 Hz, -NCH_2-), 2.26-2.39 (6H, m, -OCH$_2$CH_2-), 1.54-1.73 (12H, m, -NCH$_2$CH_2CH_2-) ppm; ^{13}C NMR (100 MHz, CDCl$_3$): δ = 183.37 (-COCONR$_2$), 165.81 (-CONR$_2$), 154.85 (ArC_{β}), 147.95 (ArC_{β}), 145.20 (ArC_{β}), 127.76 (ArC_{α}), 118.31 (ArC_{α}), 116.40 (ArC_{α}), 71.80 (-OCH_2-), 71.35 (-OCH_2-), 71.20 (-OCH_2-), 46.95 (-NCH_2-), 42.00 (-NCH_2-), 33.10 (-OCH$_2$CH$_2$-), 32.98 (-OCH$_2$CH$_2$-), 25.87 (-NCH$_2$CH$_2$-), 25.24 (-NCH$_2$CH$_2$CH$_2$-), 24.51 (-NCH$_2$CH$_2$CH$_2$-) ppm; IR (υ_{max}, solid): 2934, 2852, 1738, 1617, 1472, 1438, 1406, 1371, 1313, 1245, 1216, 1128, 1050, 1012 cm^{-1}; HRMS *m/z* (ESI+): Found: 743.1470 (M+H), Calc.: 743.1767; m.p. = 198-202 °C;

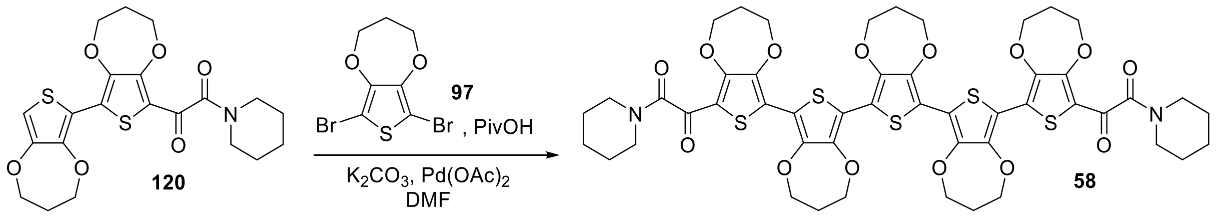

Piperidine-ProDOT dimer **120** (110 mg, 245 µmol), pivalic acid (15 mg, 123 µmol), palladium (II) acetate (6 mg, 25 µmol), potassium carbonate (338 mg, 2.45 mmol), and 2,5-dibromoProDOT **97** (53 mg, 171 µmol) were charged under nitrogen. Dry DMF (3 mL) was then added and the mixture heated to 130 ºC for 1 hr. After cooling to rt the mixture was diluted with DCM (100 mL) and washed with water (2 x 75 mL) and brine (75 mL). The organics were dried with MgSO$_4$, filtered and concentrated *in vacuo*. The residue was purified by flash column chromatography eluting with 1-4 % MeOH:DCM. Pure fractions were concentrated *in vacuo* and the residue triturated in diethyl ether (2 x 80 mL) to give the DP as a

red-purple solid. A yield of 77 mg, 73 μmol (60 %) was obtained. ^{1}H NMR (400 MHz, $CDCl_3$): δ = 4.17-4.35 (20H, m, -OC$\underline{H}_2$-), 3.60-3.71 (2H, m, -NC$\underline{H}_2$-), 3.33-3.43 (2H, m, -NC$\underline{H}_2$-), 2.27-2.43 (10H, m, -OCH_2C$\underline{H}_2$-), 1.54-1.80 (12H, m, -C$\underline{H}_2$C$\underline{H}_2$$CH_2$N-) ppm; IR ($\upsilon_{max}$, solid): 2939, 2854, 1738, 1618, 1471, 1407, 1356, 1312, 1267, 1216, 1132, 1108, 1041 cm^{-1}; HRMS *m/z* (ESI+): Found: 1083.1930 (M+H), Calc.: 1083.1928; m.p. > 350 °C;

References

1. Aubineau, T.; Cossy, J. *Chem. Commun.*, **2013**, *49* (32), 3303.
2. Stover, J. S.; Shi, J.; Jin, W.; Vogt, P. K.; Boger, D. L. *J. Am. Chem. Soc.*, **2009**, *131* (9), 3342.
3. Liu, J.; Kolar, C.; Lawson, T. A.; Gmeiner, W. H. *J. Org. Chem.*, **2001**, *66* (17), 5655.
4. Lankshear, M. D.; Dudley, I. M.; Chan, K-M.; Cowley, A. R.; Santos, S. M.; Felix, V.; Beer, P. D. *Chem. Eur. J.*, **2008**, *14* (7), 2248.
5. Bertozzi, C. R.; Bednarski, M. D. *J. Org. Chem.*, **1991**, *56*, 4326.
6. Morimoto, K.; Nakae, T.; Yamaoka, N.; Dohi, T.; Kita, Y. *Eur. J. Org. Chem*., **2011**, *31*, 6326.
7. Turbiez, M.; Frère, P.; Roncali, J. *J. Org. Chem*., **2003**, *68* (13), 5357.
8. Goto, H. *J. Mater. Chem*., **2009**, *19* (28), 4914.
9. Goldoni, F.; Langeveld-Voss, B. M. W.; Meijer, E. W. *Synth. Commun*., **1998**, *28* (12), 2237.
10. Kim, B.; Koh, J. K.; Kim, K.; Chi, W. S.; Kim, J. H.; Kim, E. *ChemSusChem*, **2012**, *5*, 2173-2180.

^1H NMR (400 MHz, $CDCl_3$)

Figure S6. ^1H NMR of **3**

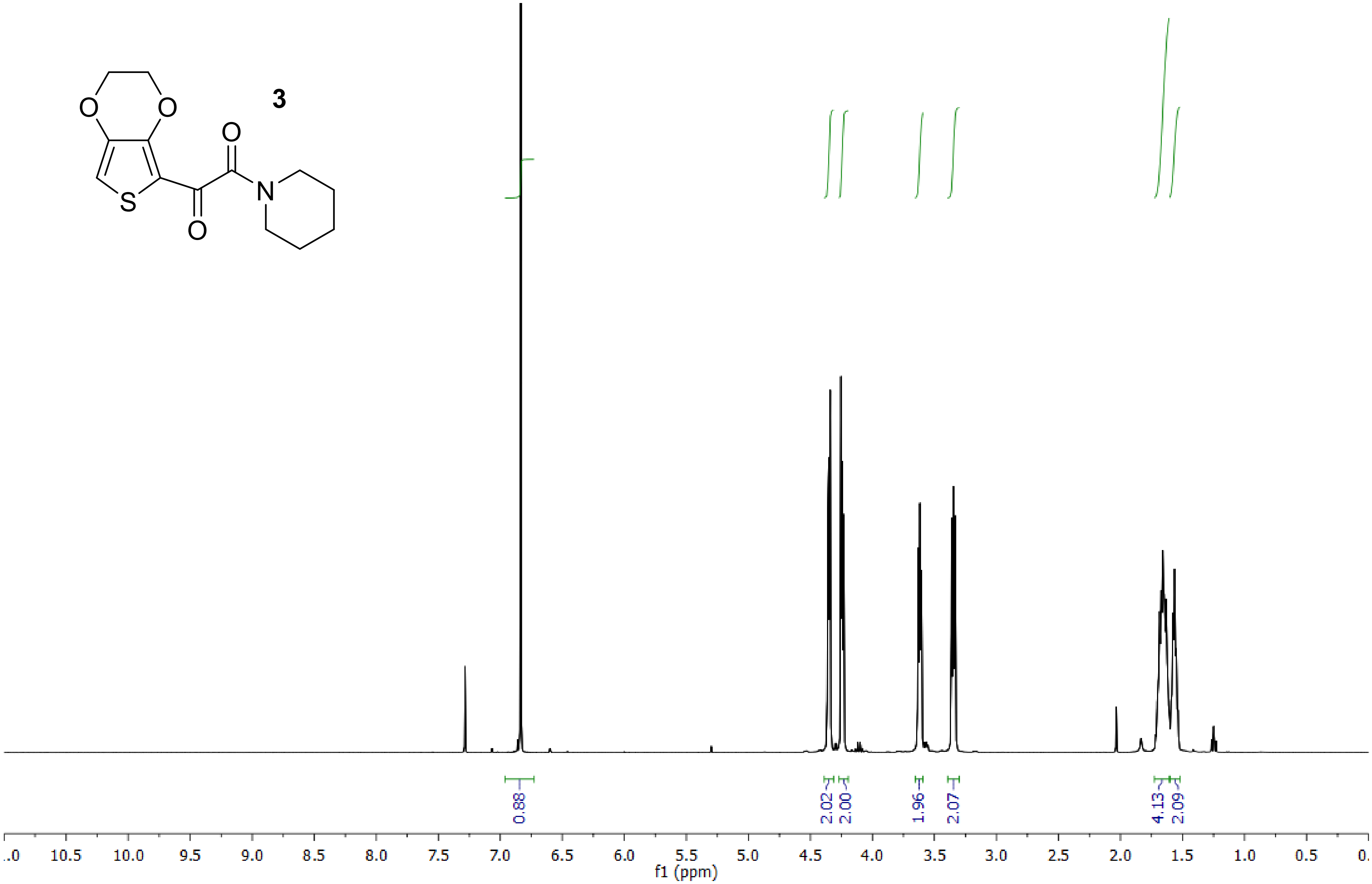

^{13}C NMR (100 MHz, $CDCl_3$)

Figure S7. ^{13}C NMR of **3**

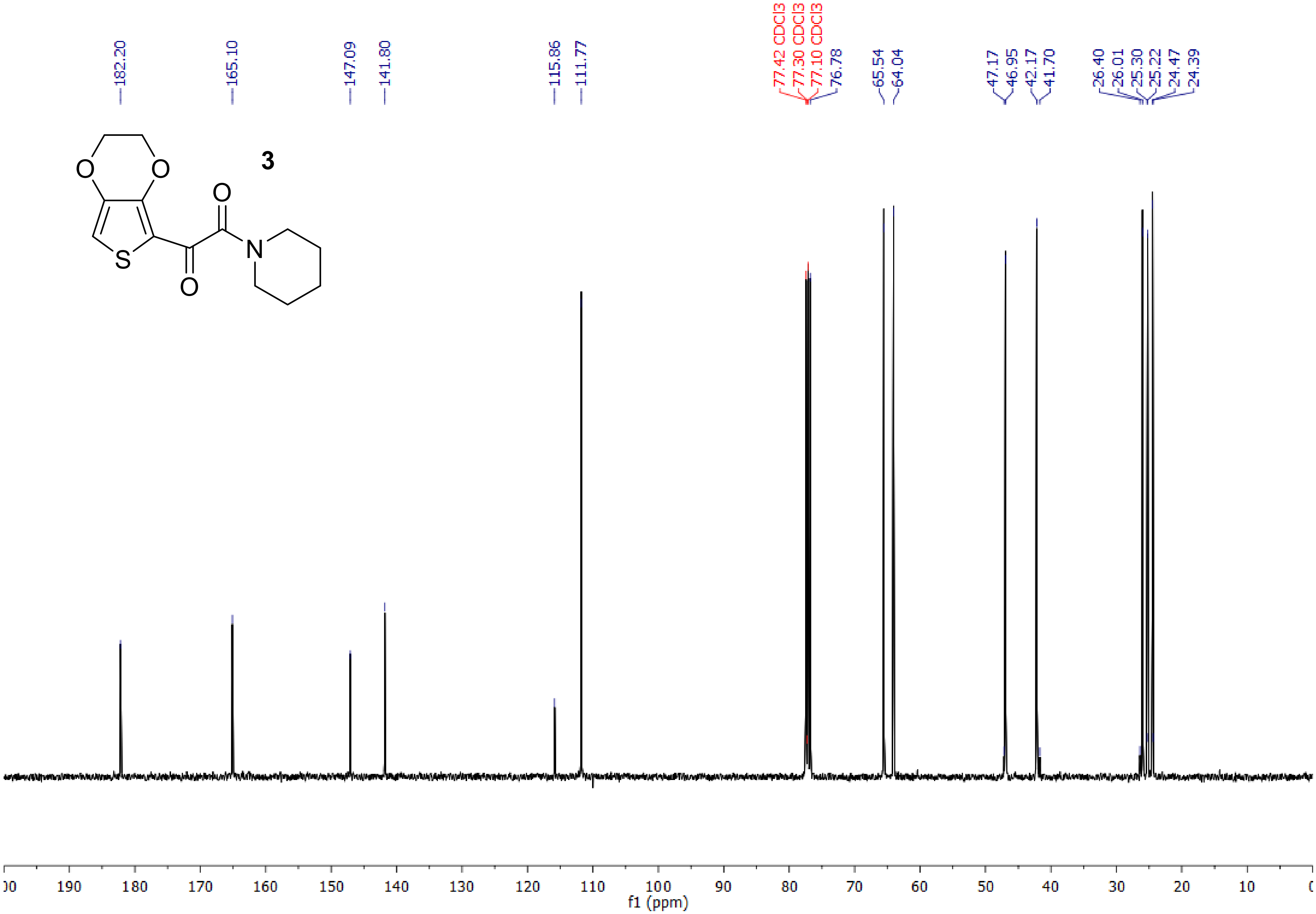

^{1}H NMR (400 MHz, $CDCl_3$)

Figure S8. ^{1}H NMR of **4**

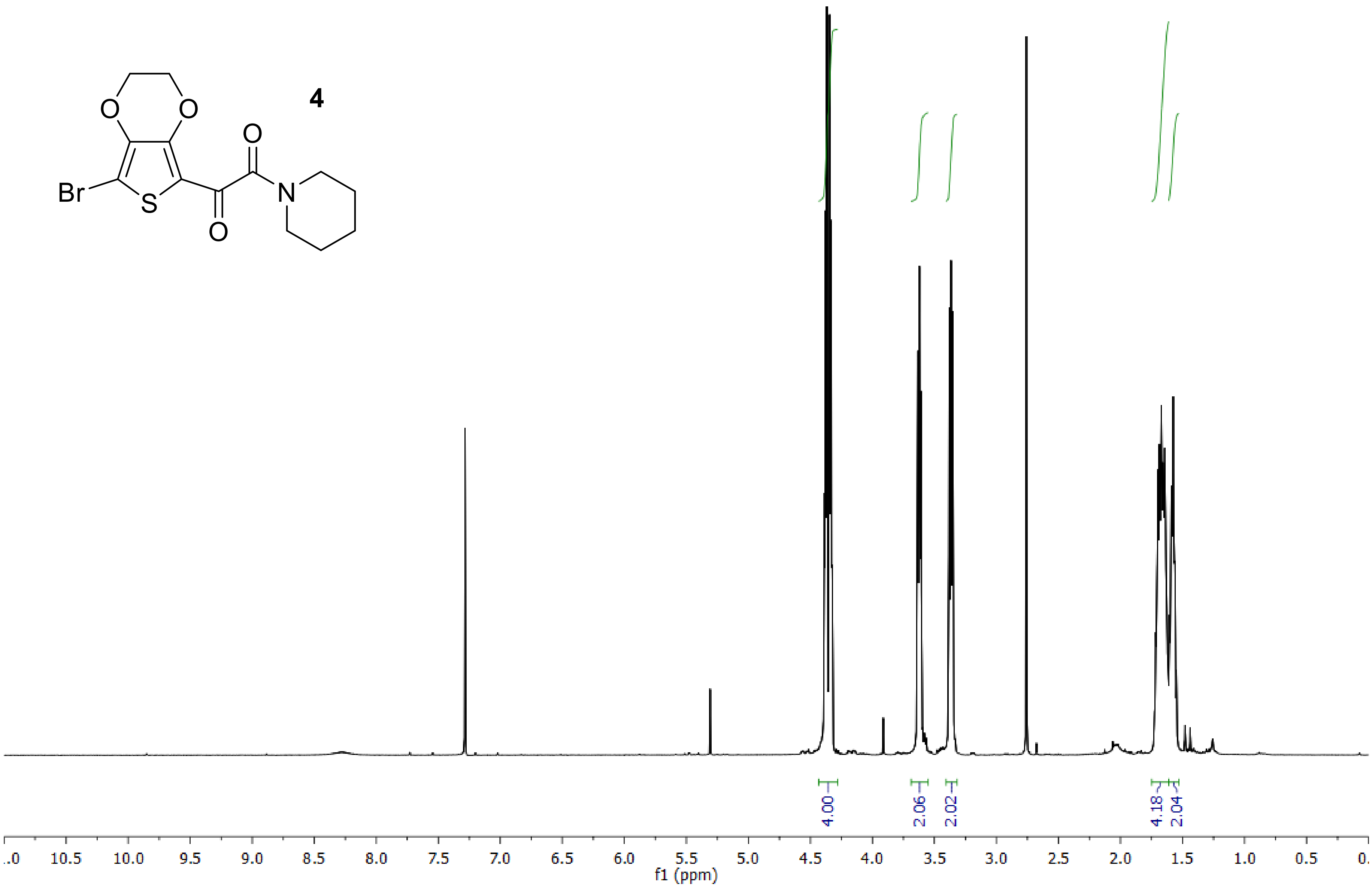

^{13}C NMR (100 MHz, CDCl$_3$)

Figure S9. ^{13}C NMR of **4**

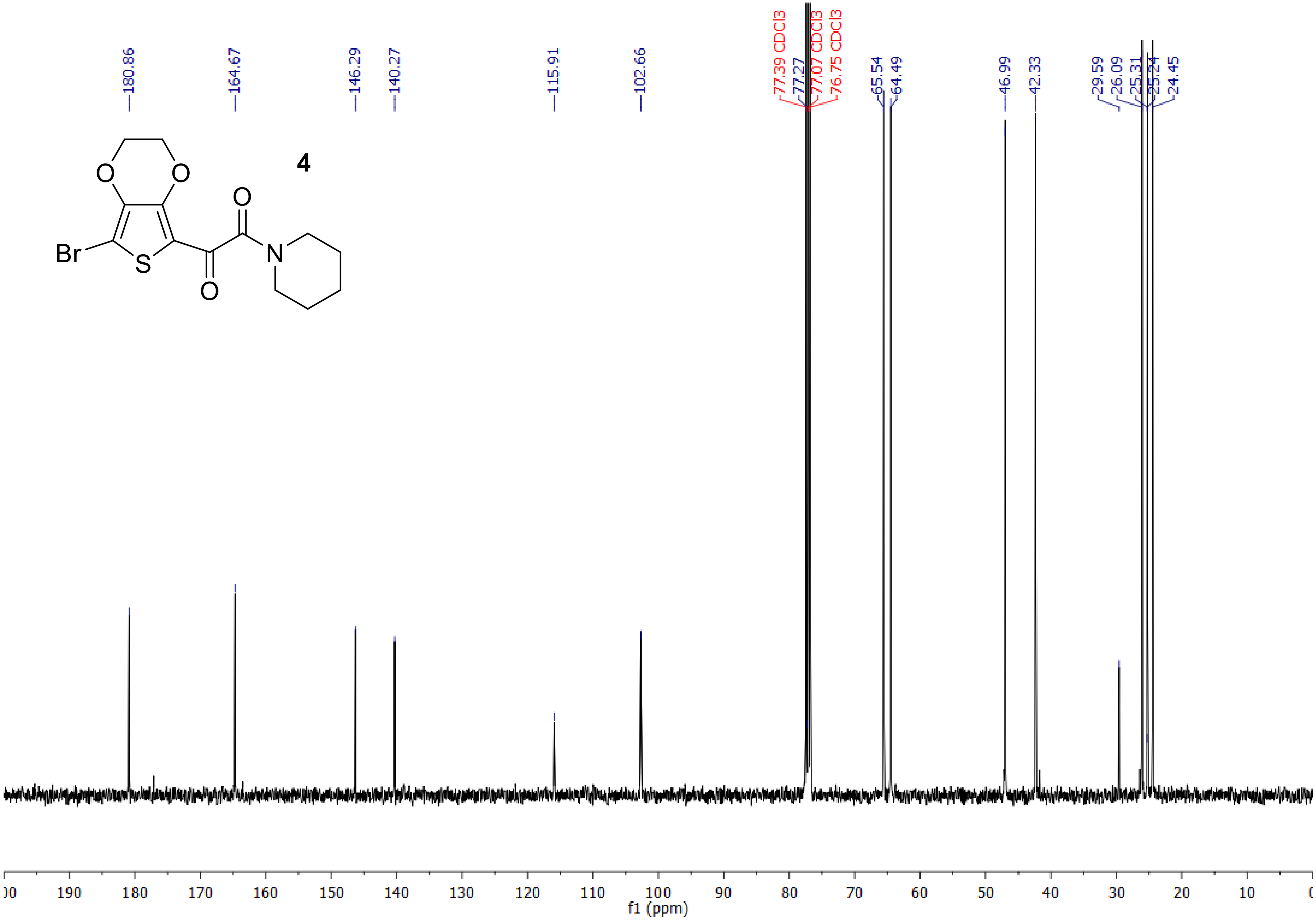

^1H NMR (400 MHz, $CDCl_3$)

Figure S10. ^1H NMR of **5**

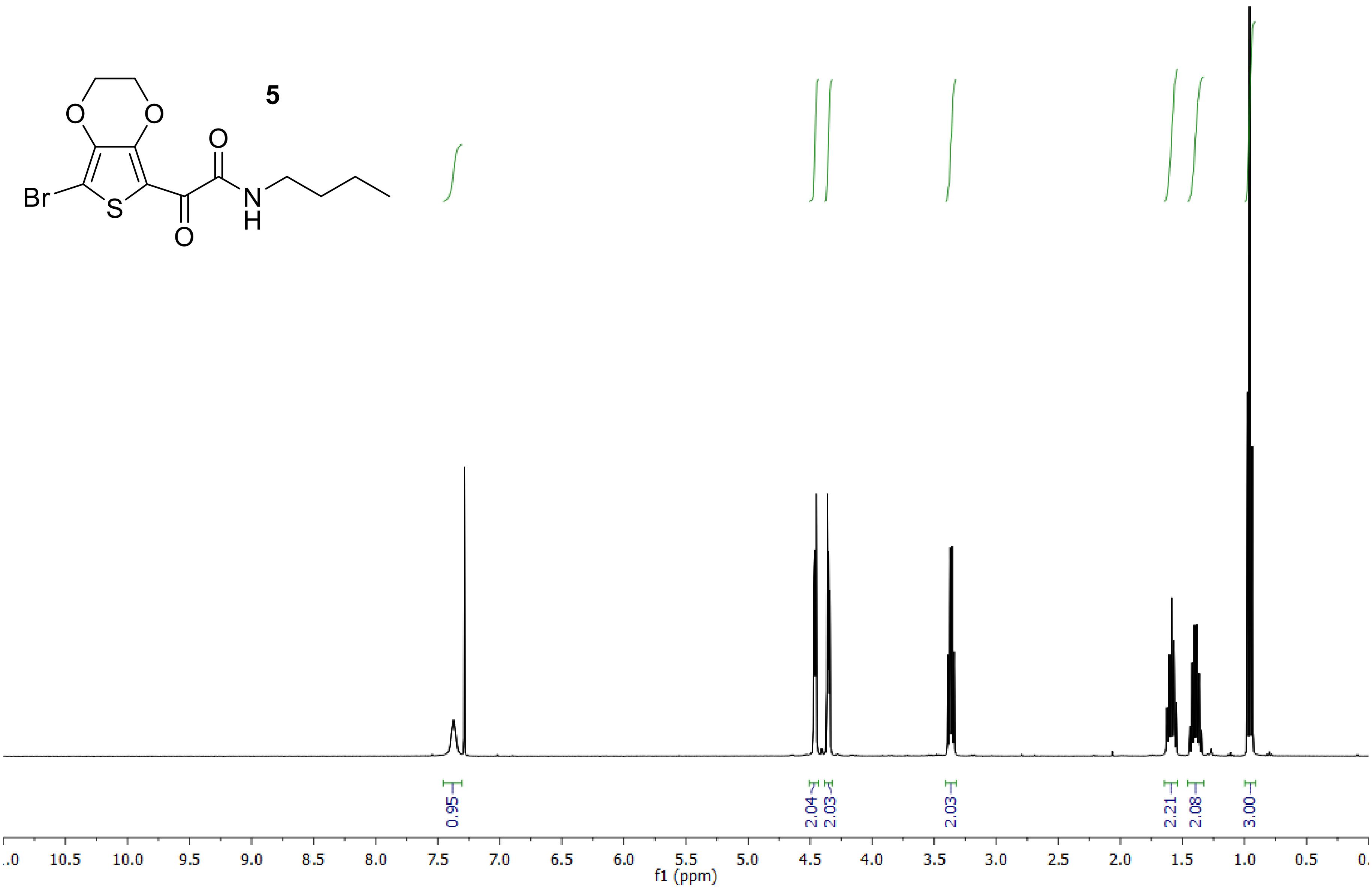

^{13}C NMR (100 MHz, $CDCl_3$)

Figure S11. ^{13}C NMR of **5**

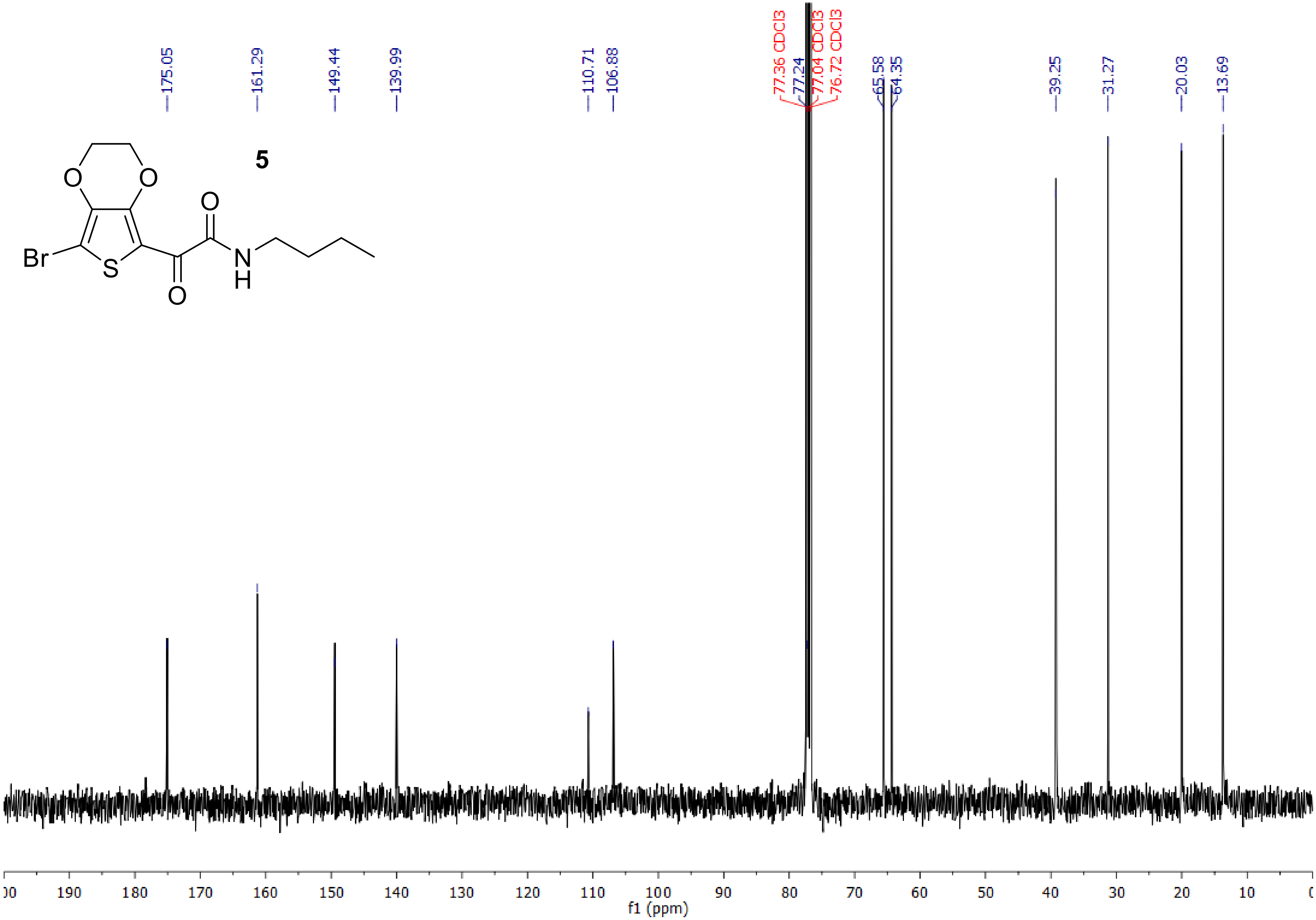

^1H NMR (400 MHz, $CDCl_3$)

Figure S12. ^1H NMR of **6**

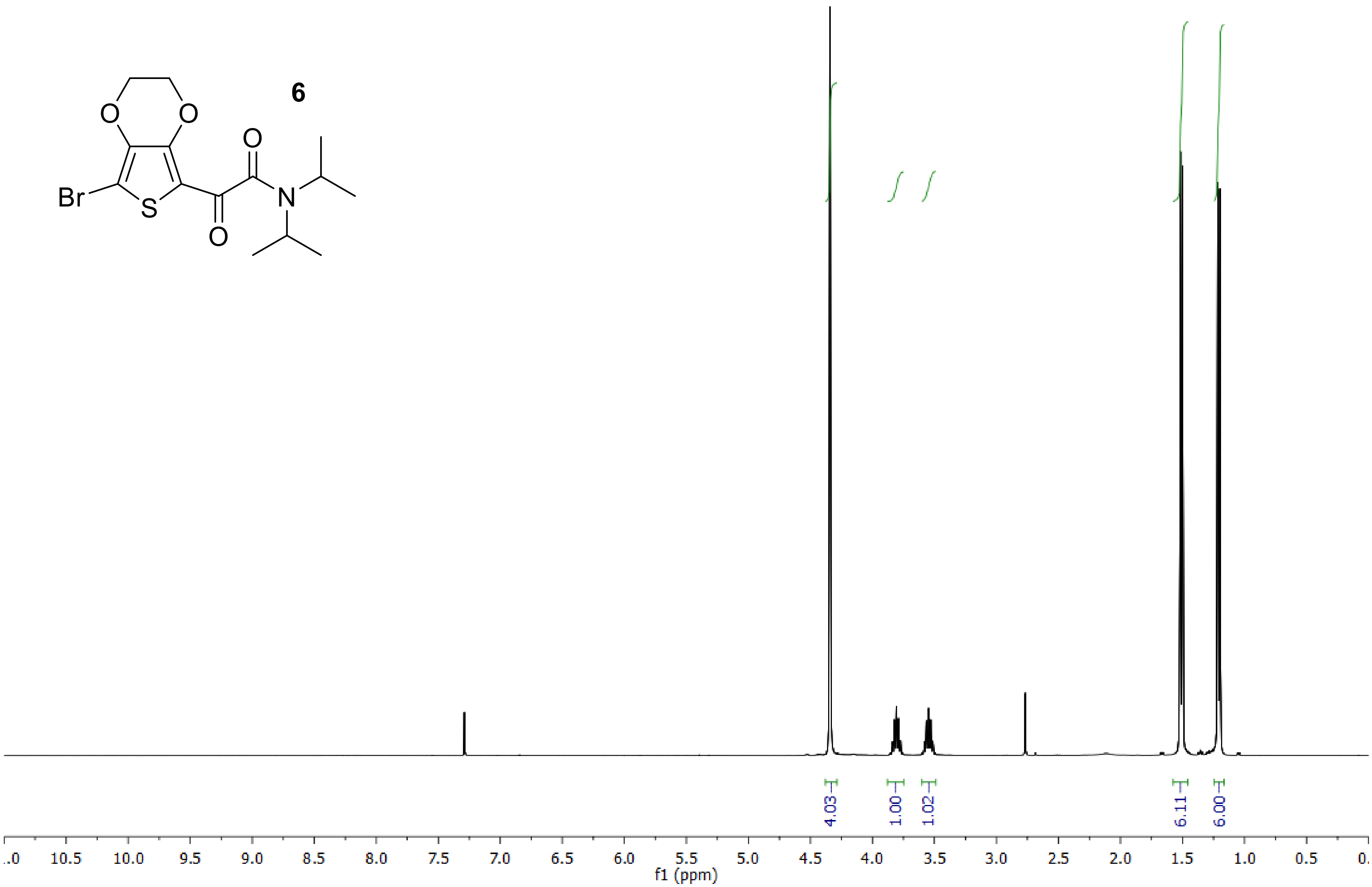

^{13}C NMR (100 MHz, $CDCl_3$)

Figure S13. ^{13}C NMR of **6**

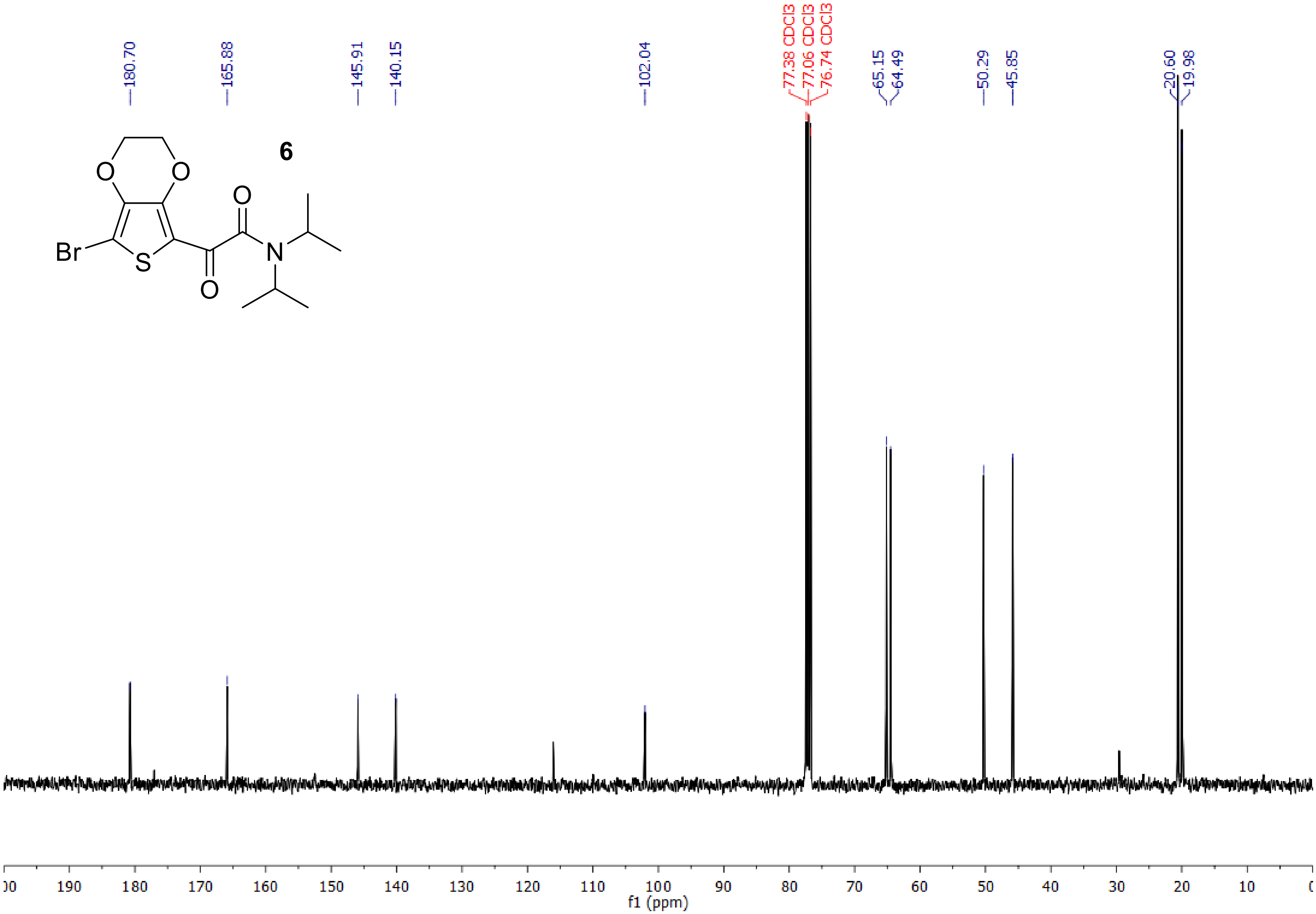

^1H NMR (400 MHz, $CDCl_3$)

Figure S14. ^1H NMR of **7**

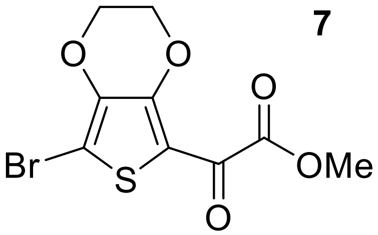

7

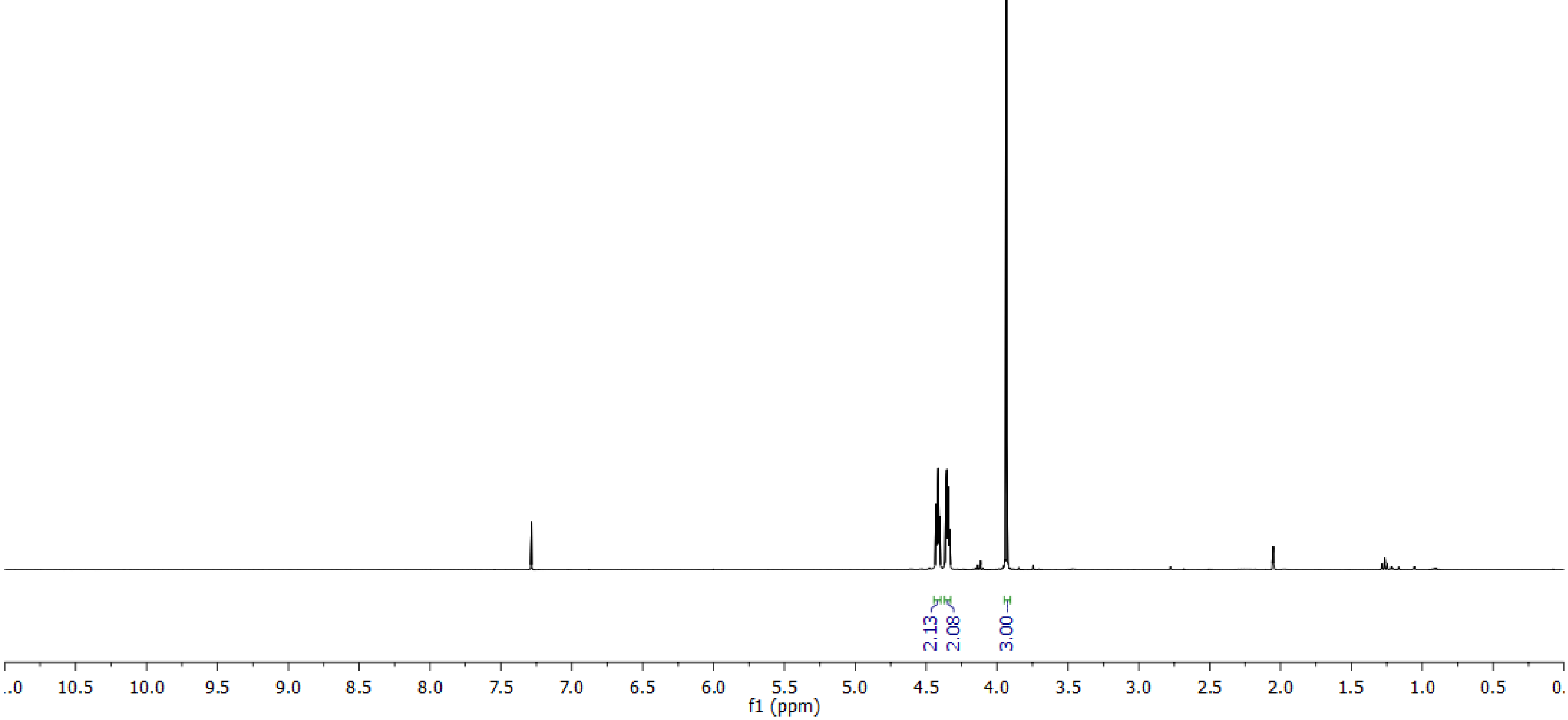

^{13}C NMR (100 MHz, $CDCl_3$)

Figure S15. ^{13}C NMR of **7**

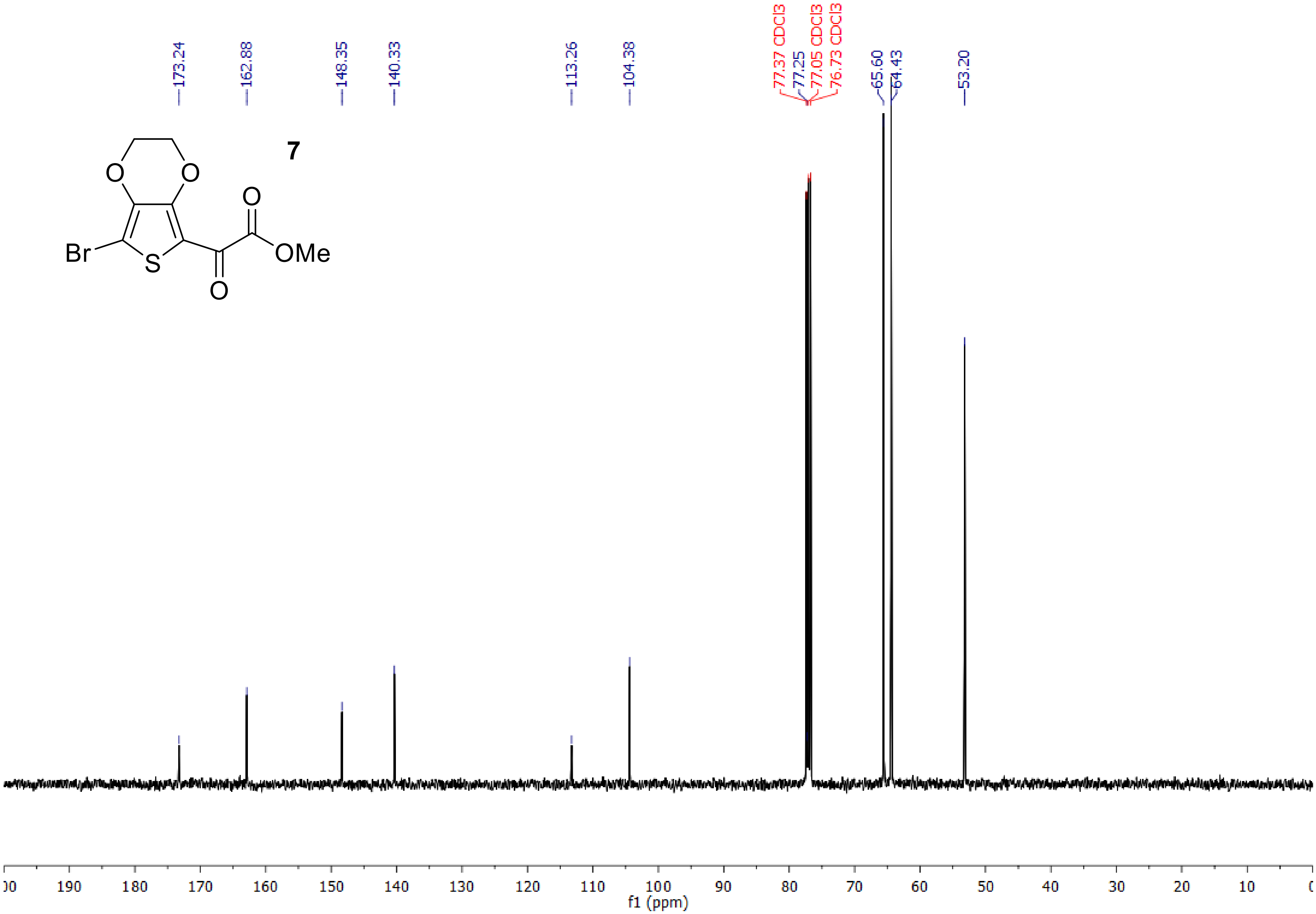

^{1}H NMR (400 MHz, $CDCl_3$)

Figure S16. ^{1}H NMR of **8**

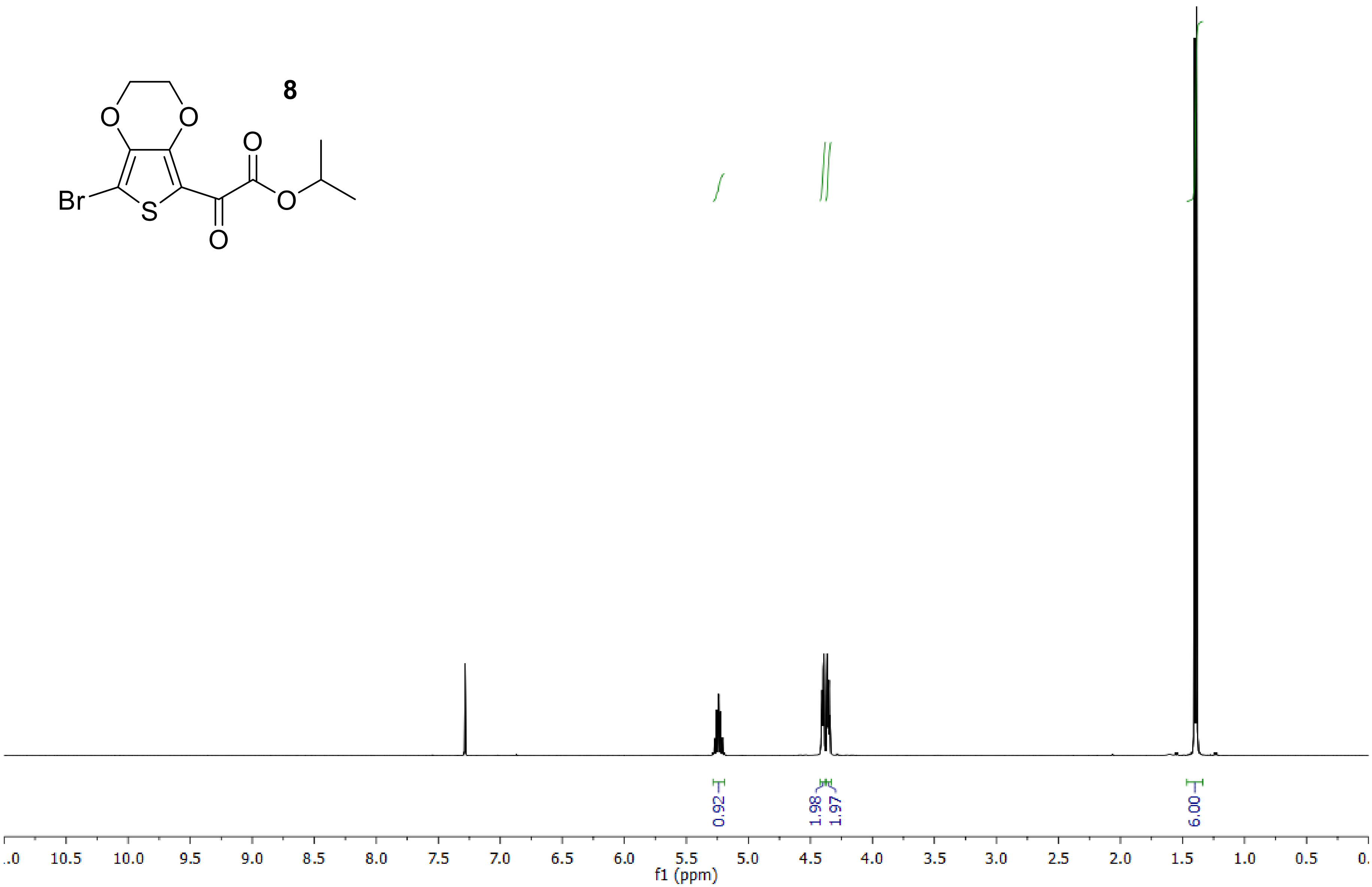

^{13}C NMR (100 MHz, $CDCl_3$)

Figure S17. ^{13}C NMR of **8**

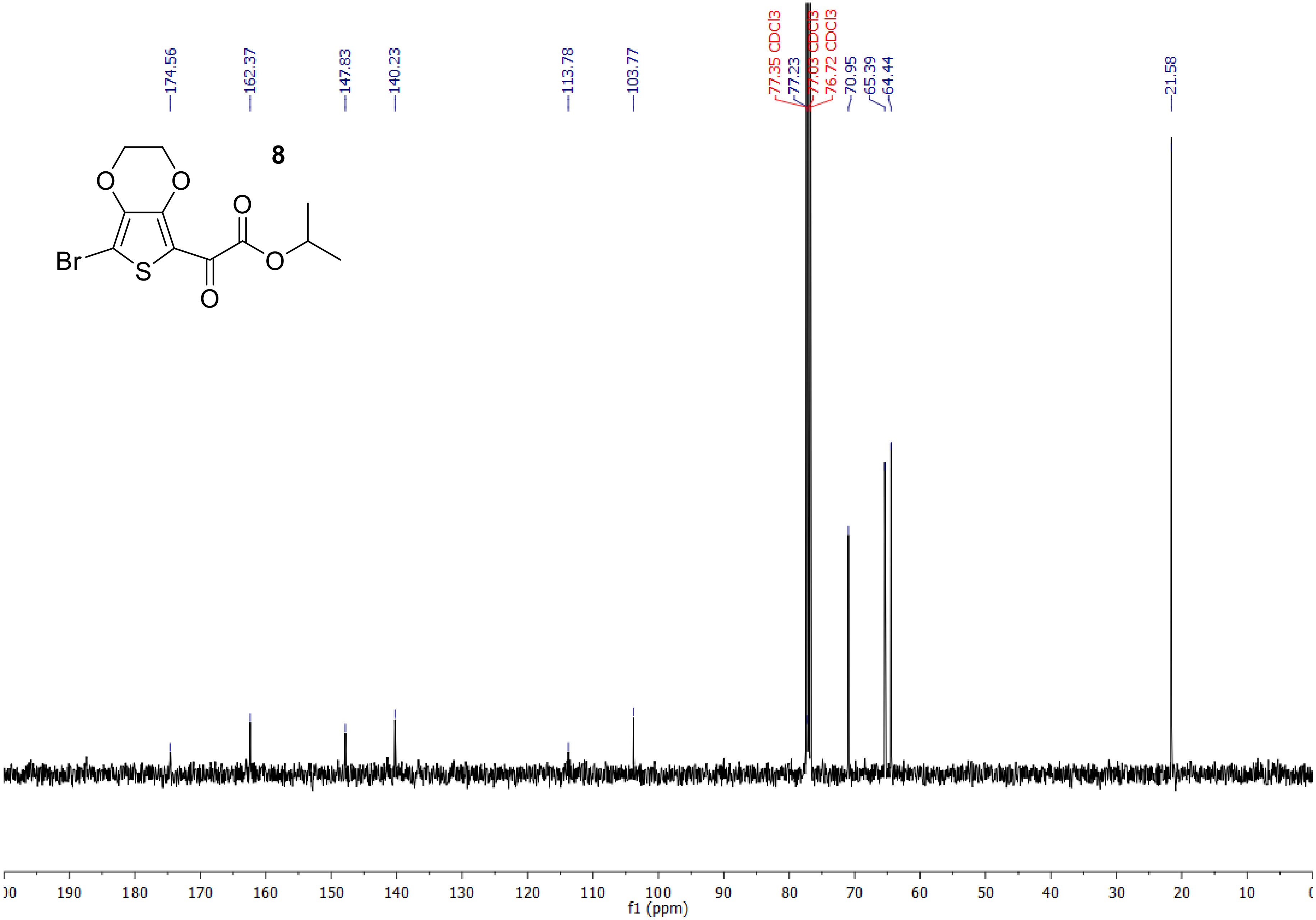

^{1}H NMR (400 MHz, $CDCl_3$)

Figure S18. ^{1}H NMR of **9**

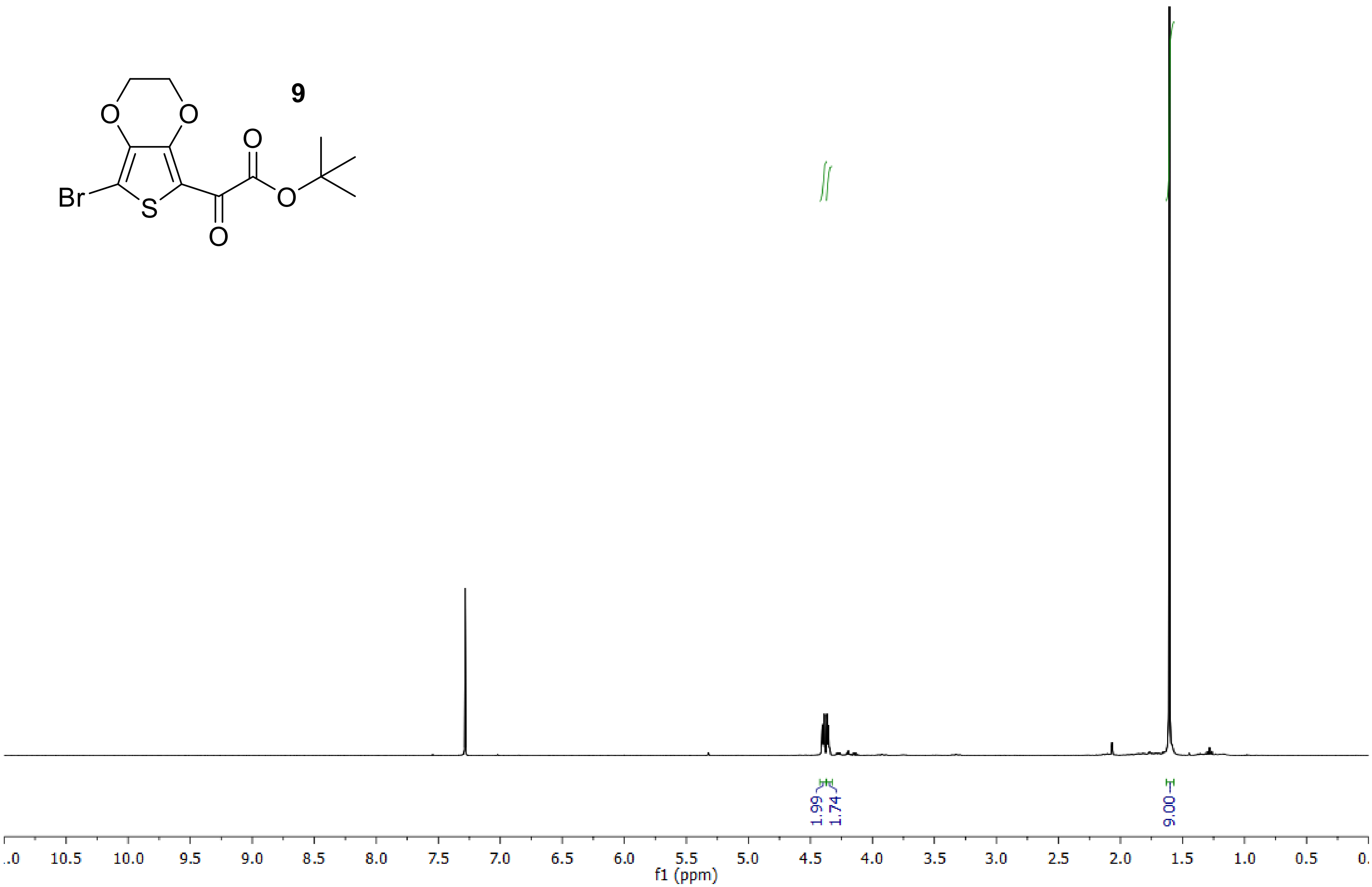

^{13}C NMR (100 MHz, $CDCl_3$)

Figure S19. ^{13}C NMR of **9**

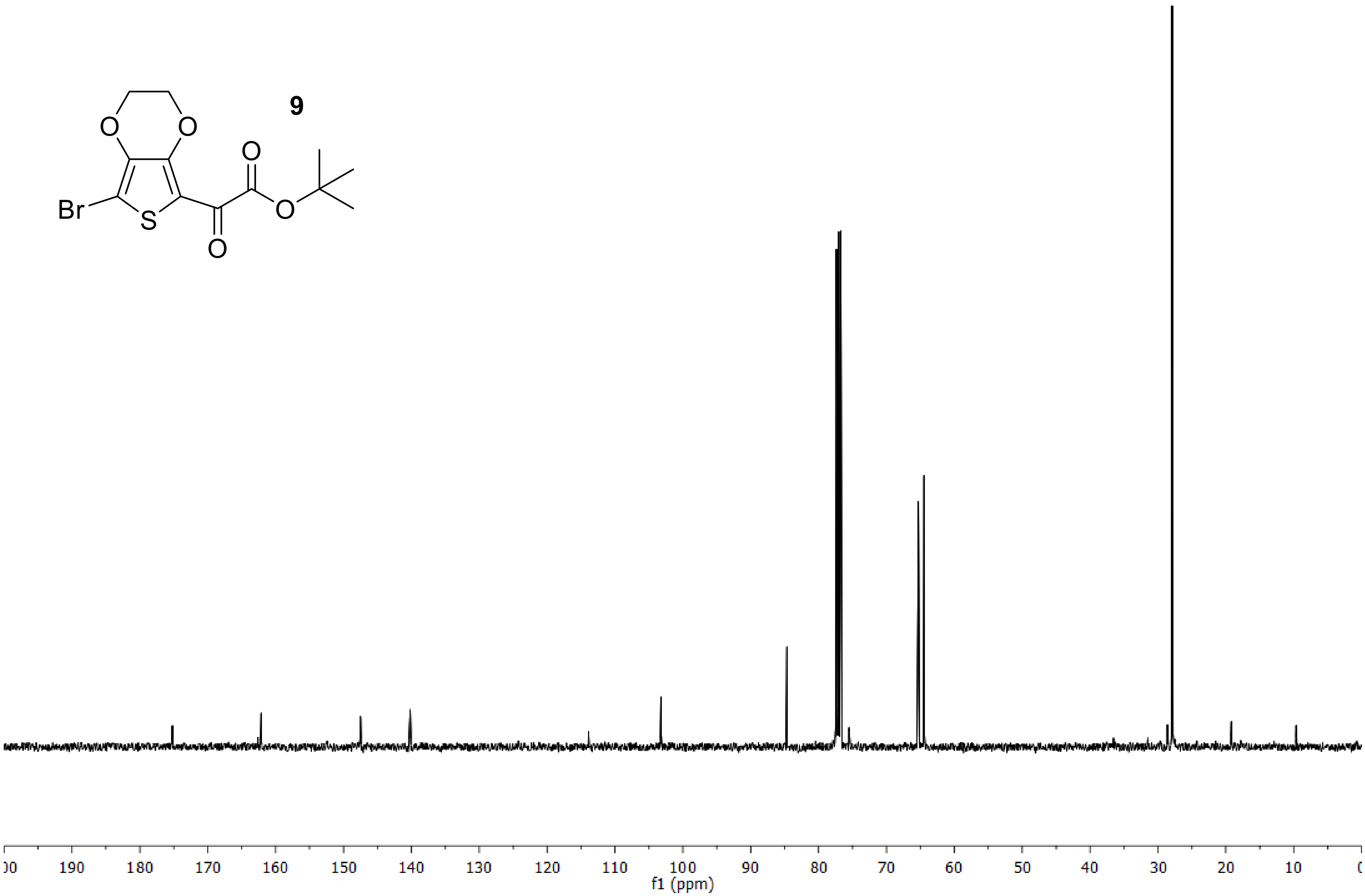

^{1}H NMR (400 MHz, MeOD)

Figure S20. ^{1}H NMR of **10**

10

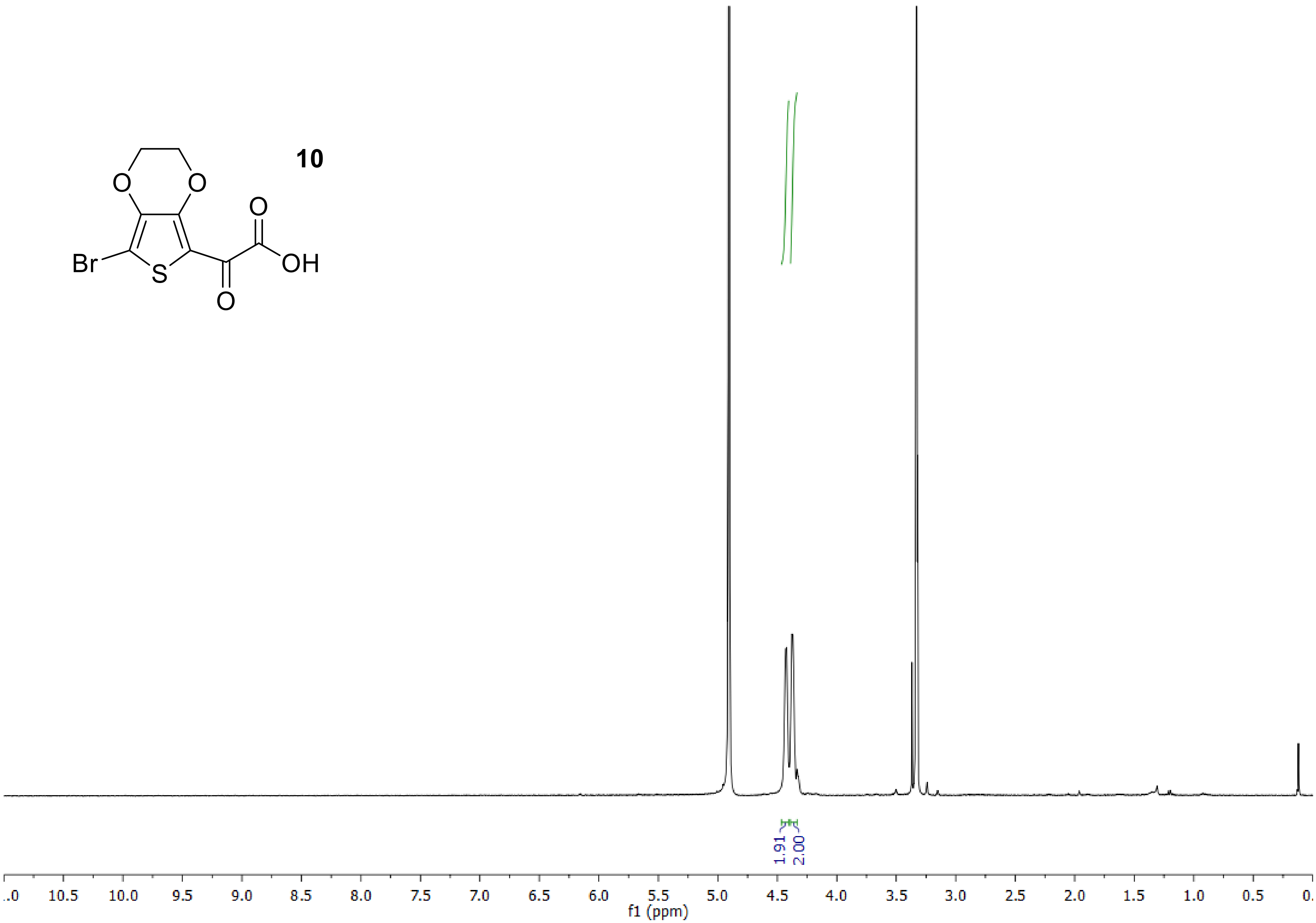

^{13}C NMR (100 MHz, MeOD)

Figure S21. ^{13}C NMR of **10**

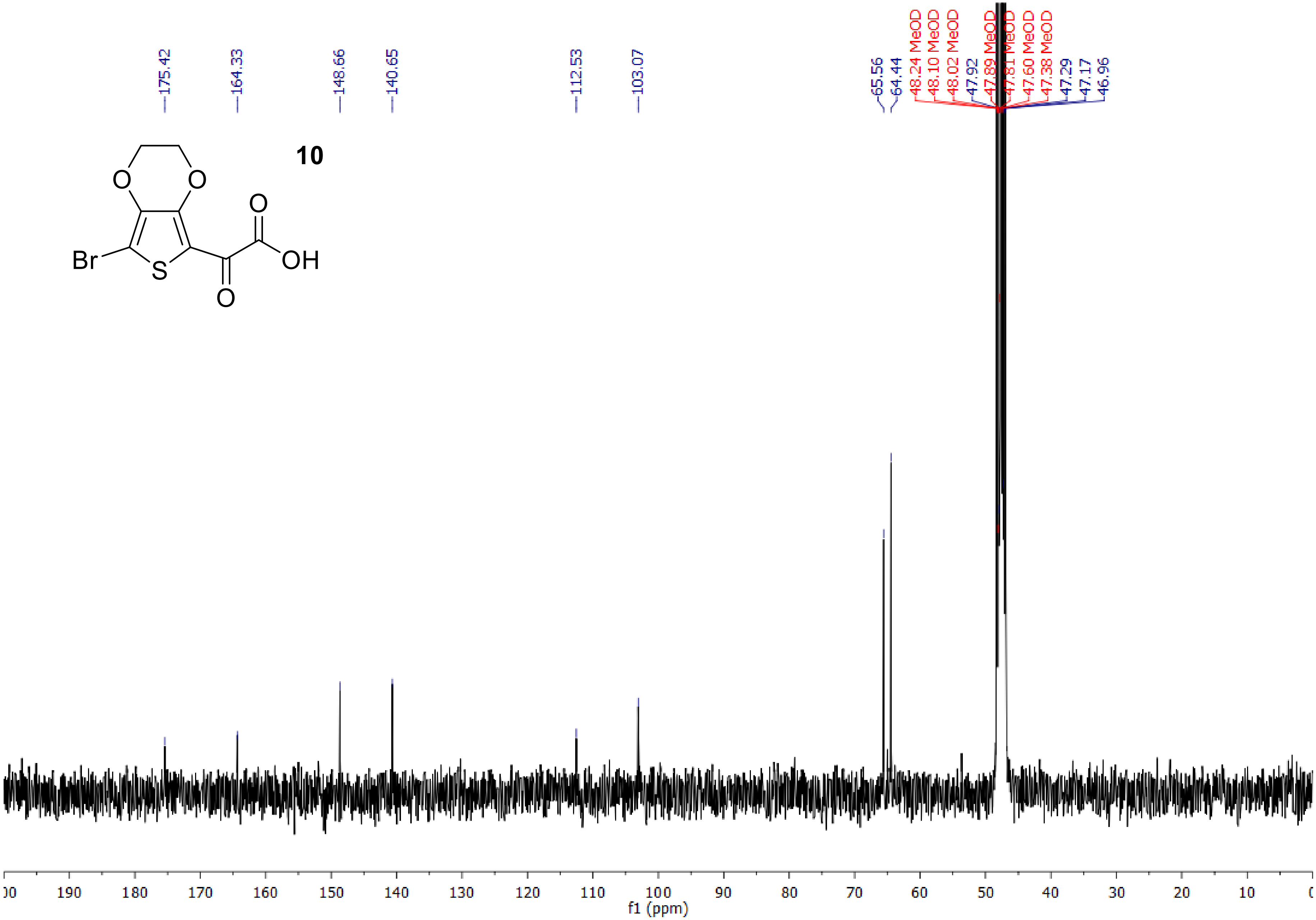

^{1}H NMR (400 MHz, $CDCl_3$)

Figure S22. ^{1}H NMR of **11**

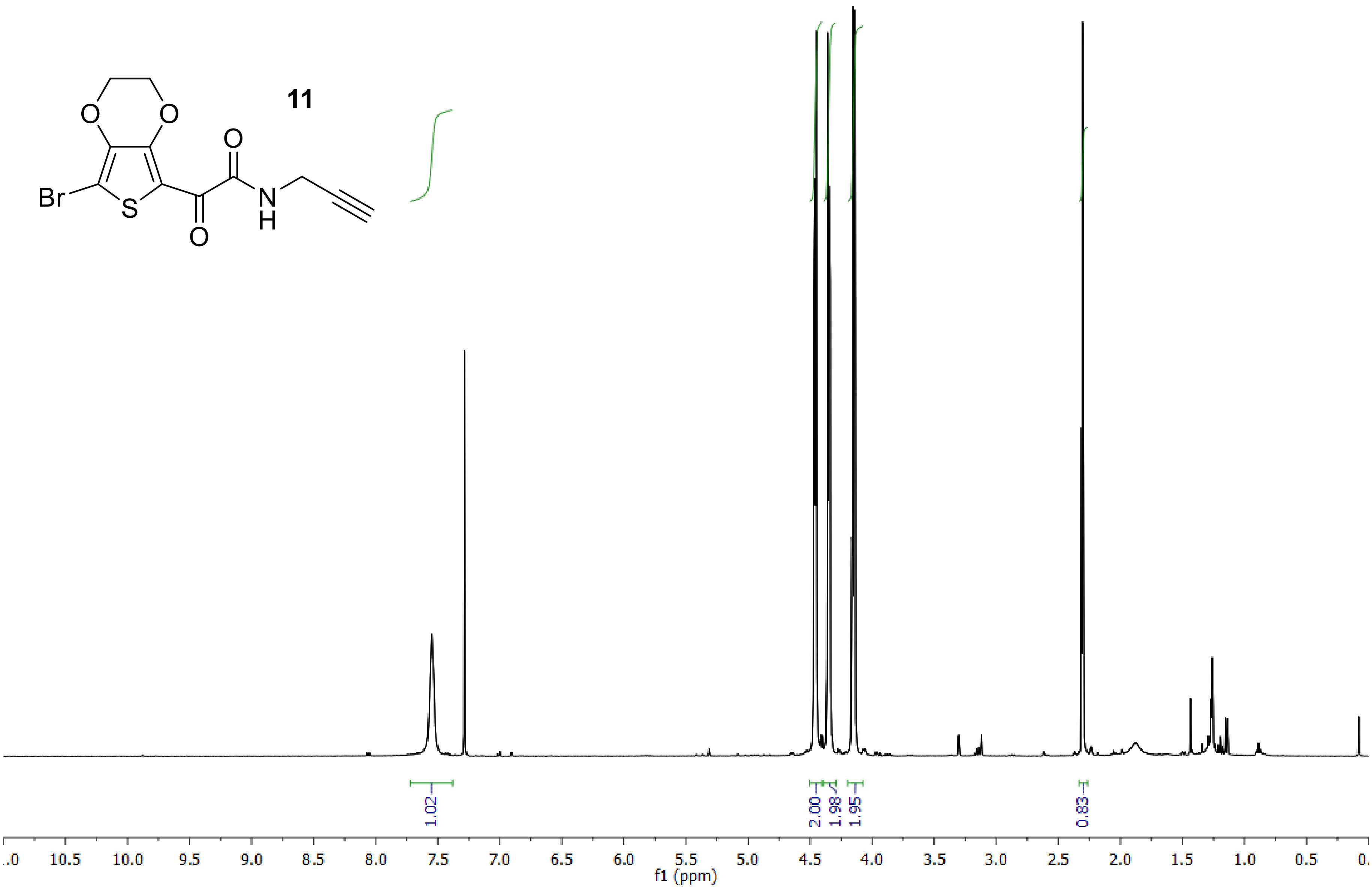

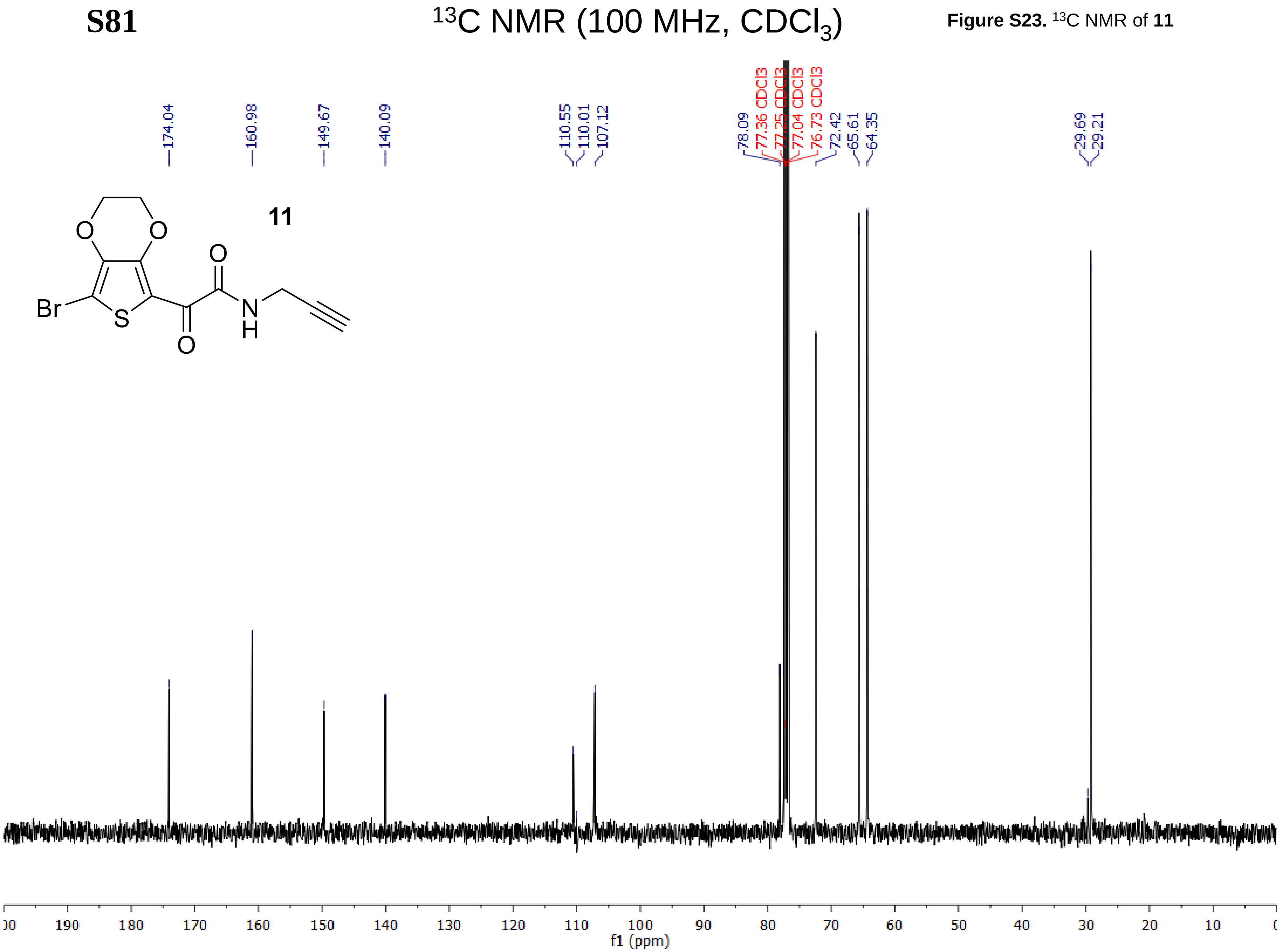

Figure S23. ^{13}C NMR of **11**

¹H NMR (400 MHz, $CDCl_3$)

Figure S24. ¹H NMR of **12**

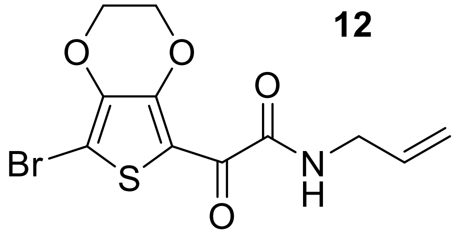

12

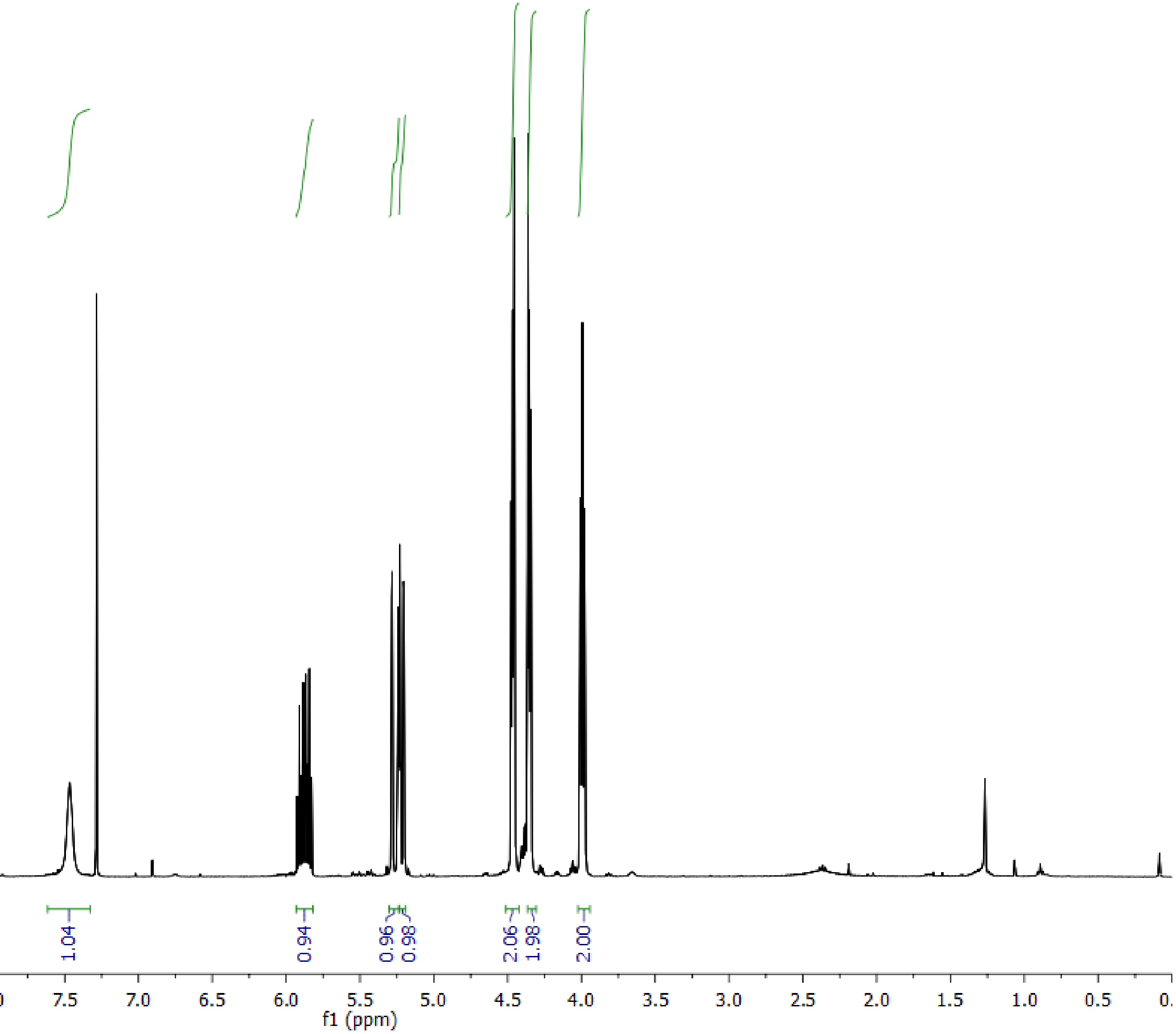

^{13}C NMR (100 MHz, $CDCl_3$)

Figure S25. ^{13}C NMR of **12**

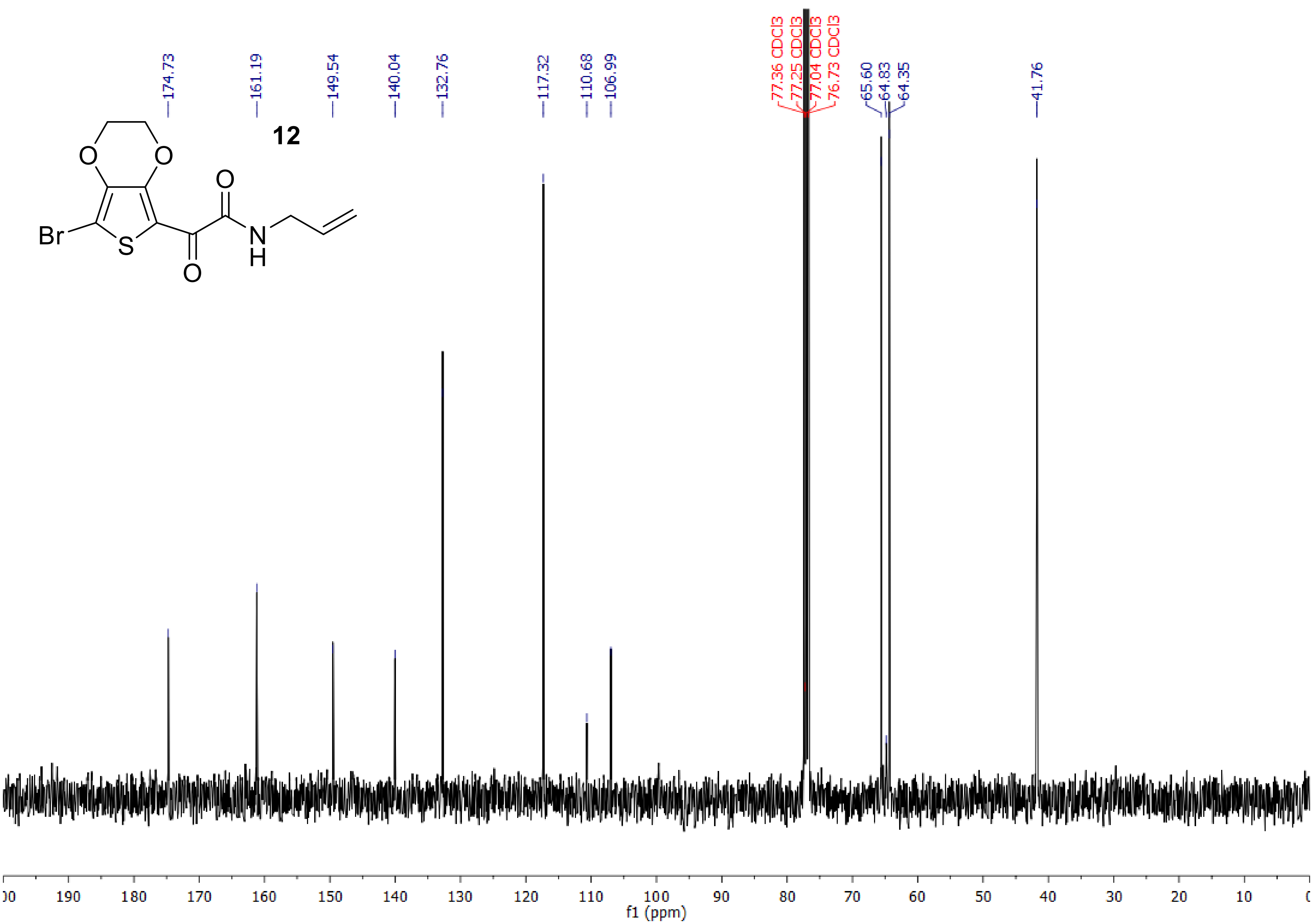

^{1}H NMR (400 MHz, MeOD)

Figure S26. ^{1}H NMR of **13**

13

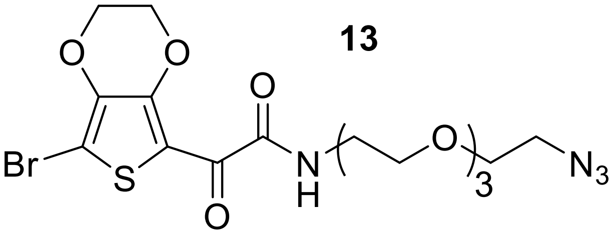

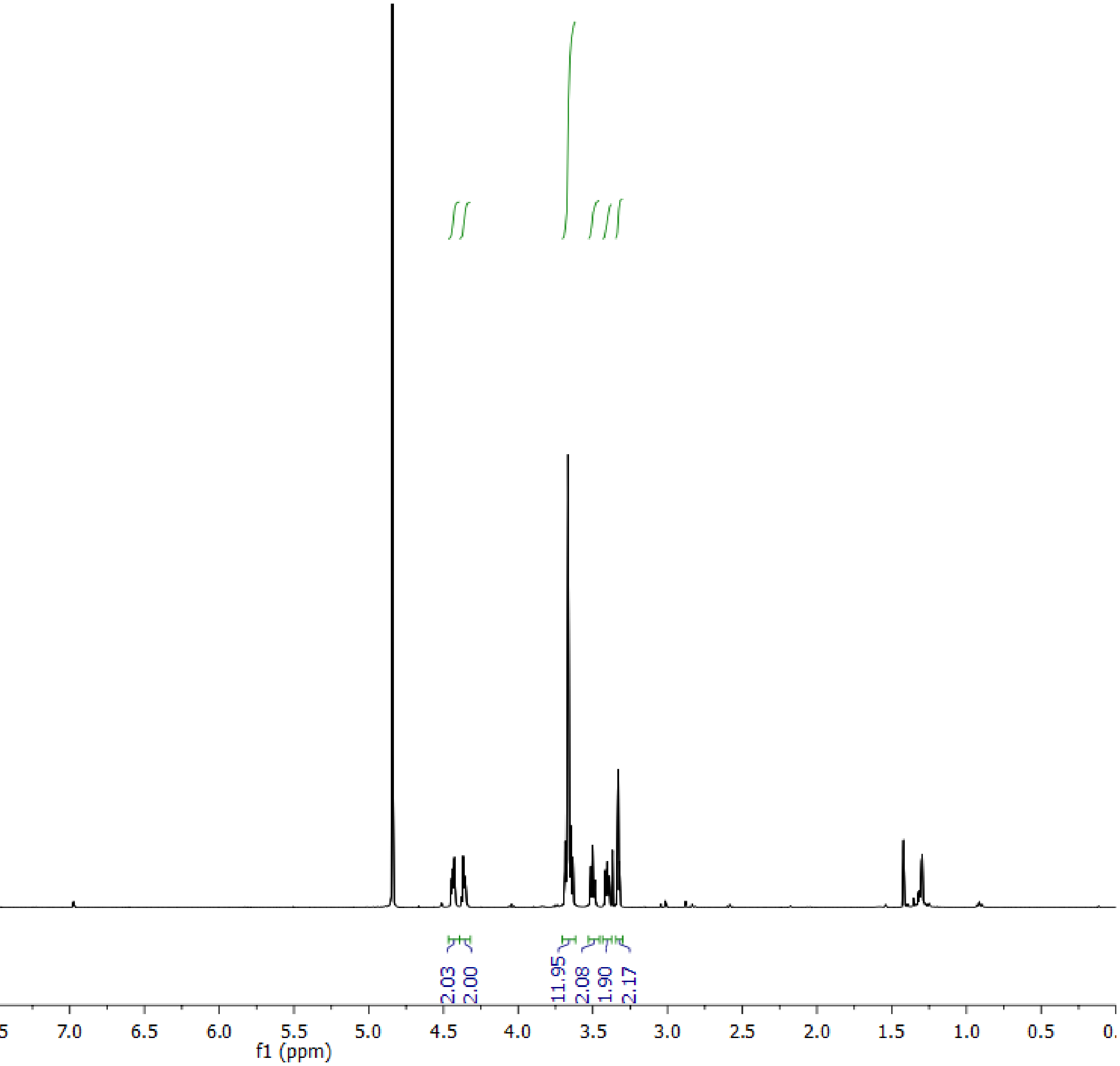

^{13}C NMR (100 MHz, MeOD)

Figure S27. ^{13}C NMR of **13**

13

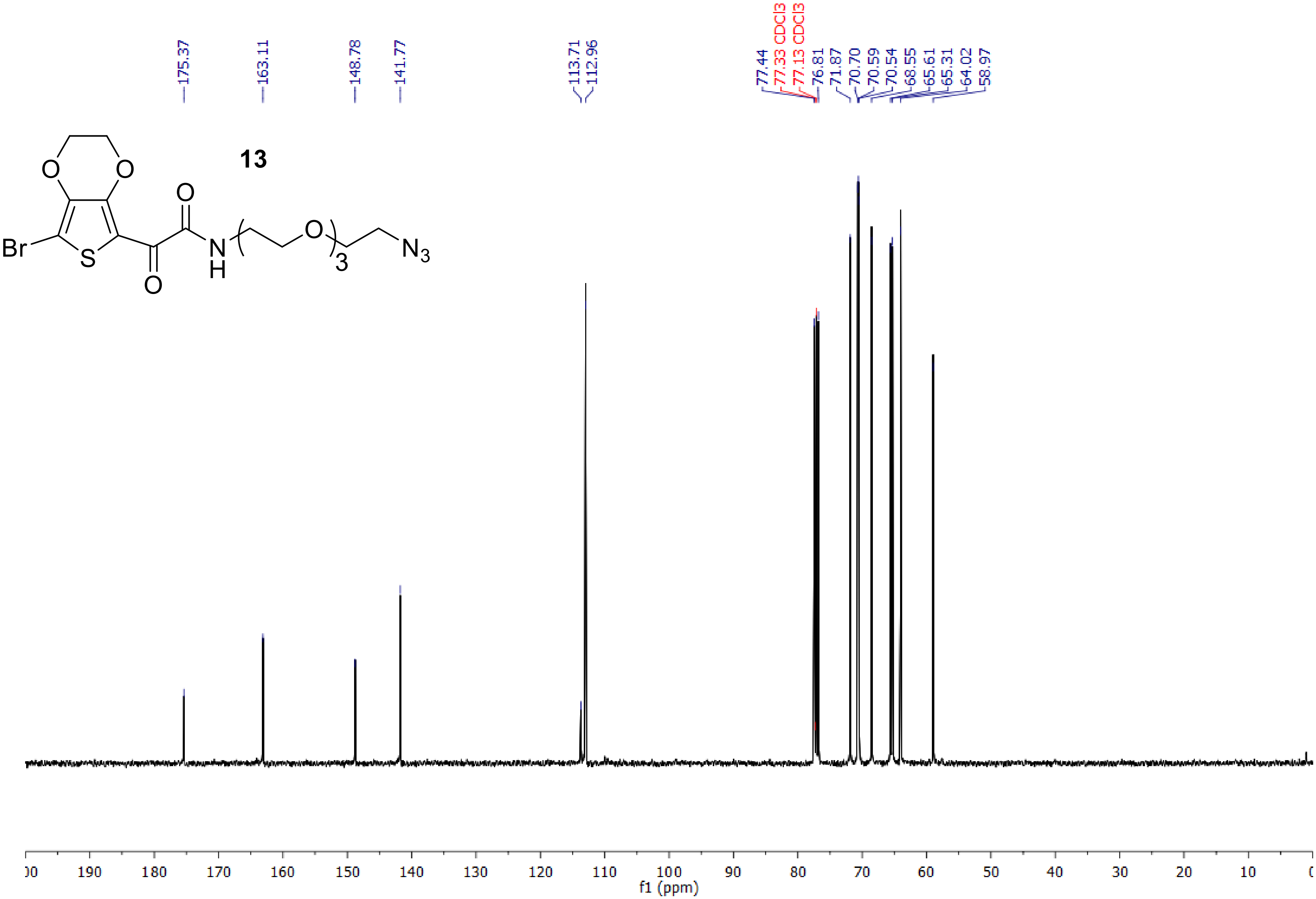

^{1}H NMR (400 MHz, $CDCl_3$)

Figure S28. ^{1}H NMR of **14**

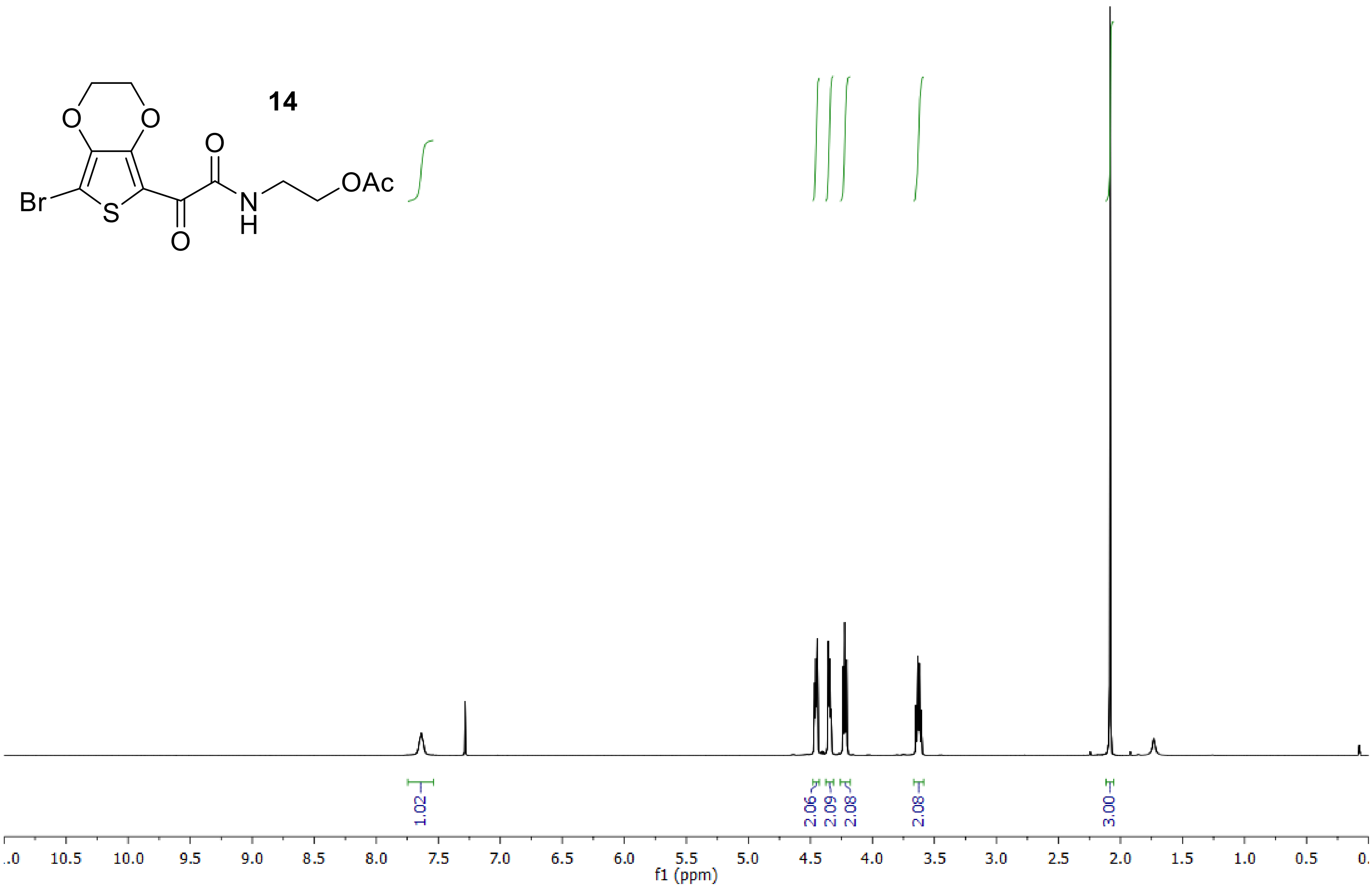

^{13}C NMR (100 MHz, $CDCl_3$)

Figure S29. ^{13}C NMR of **14**

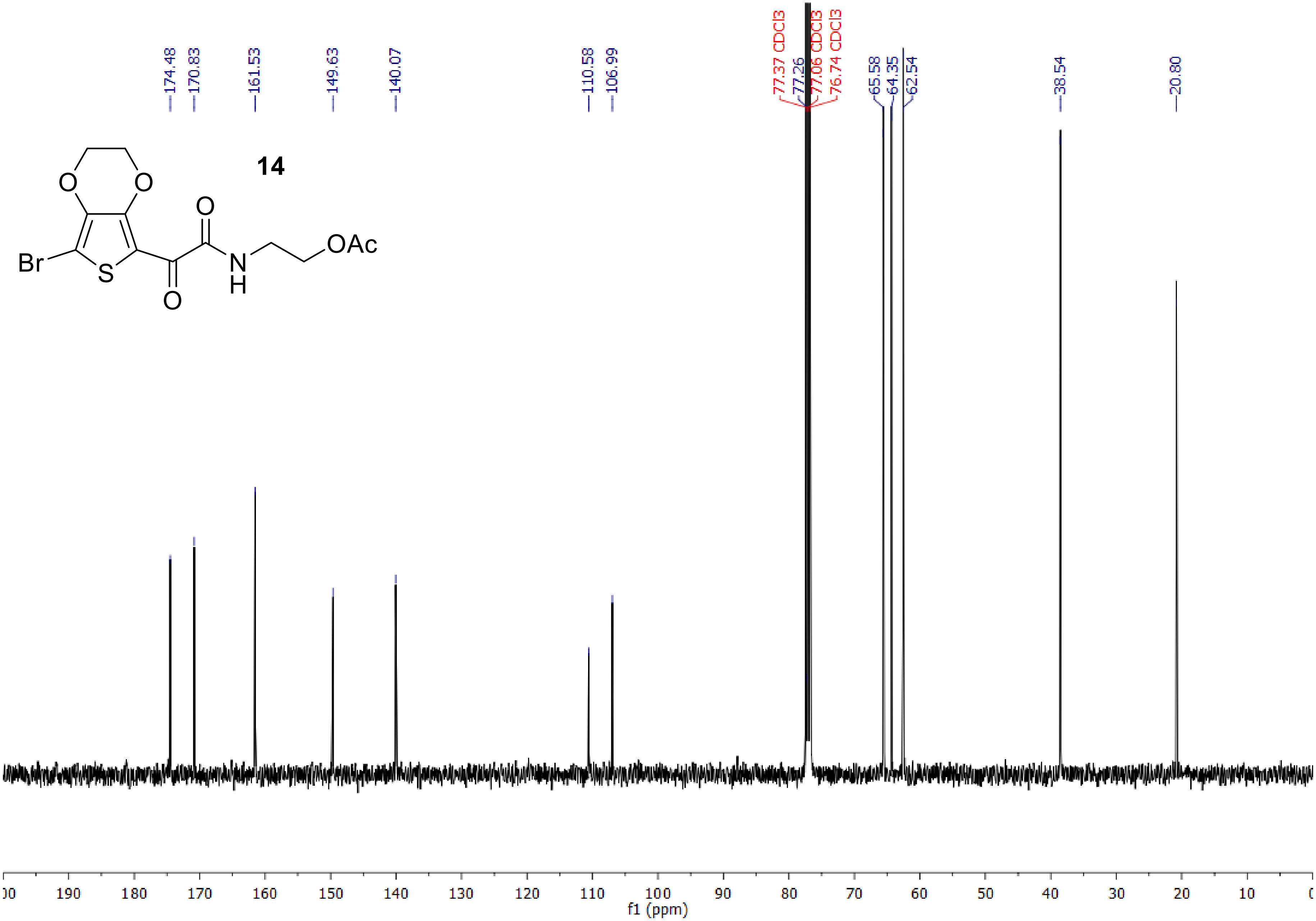

^{1}H NMR (400 MHz, DMSO-d_6)

Figure S30. ^{1}H NMR of **15**

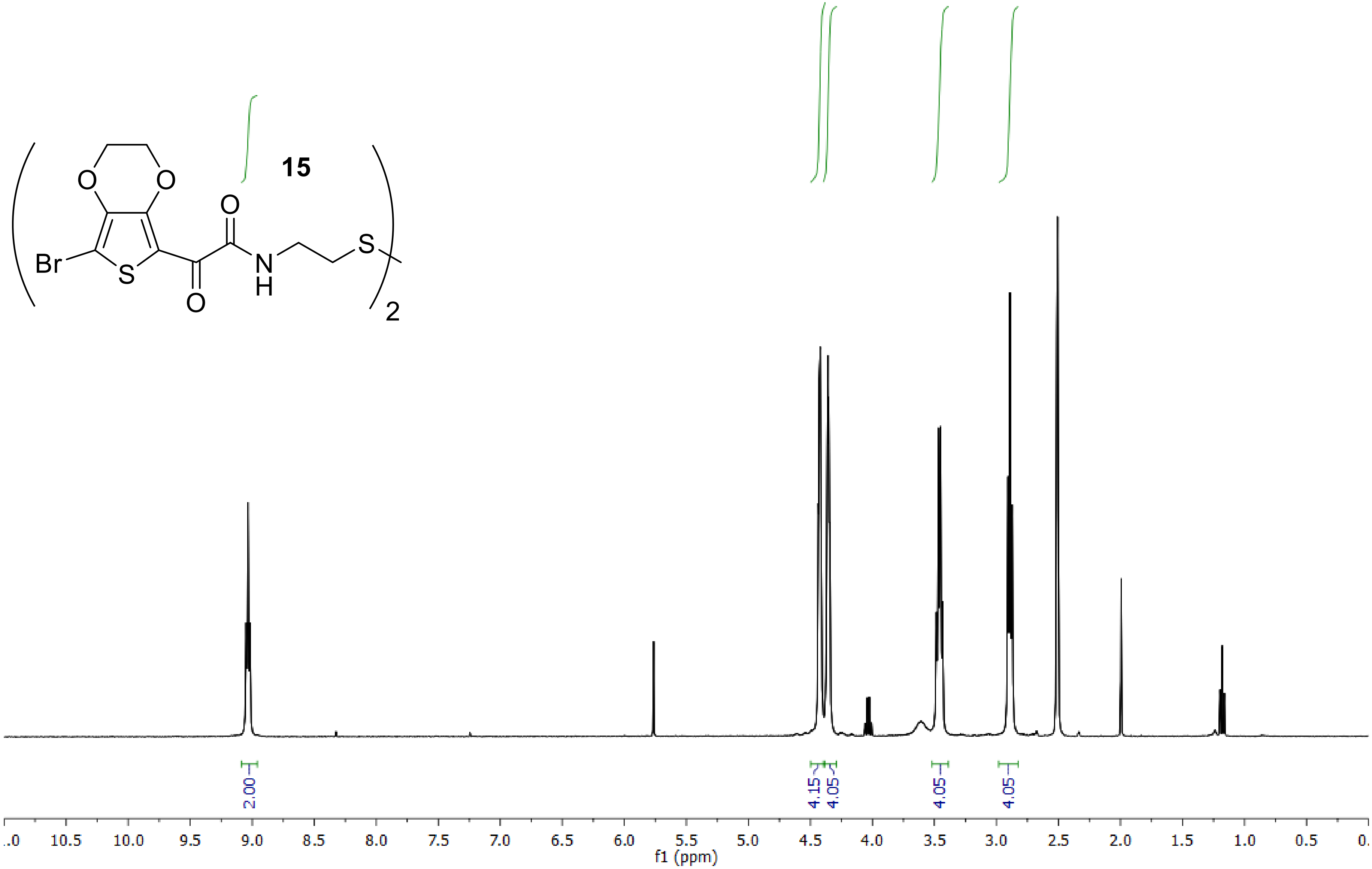

^{13}C NMR (100 MHz, DMSO-d_6)

Figure S31. ^{13}C NMR of **15**

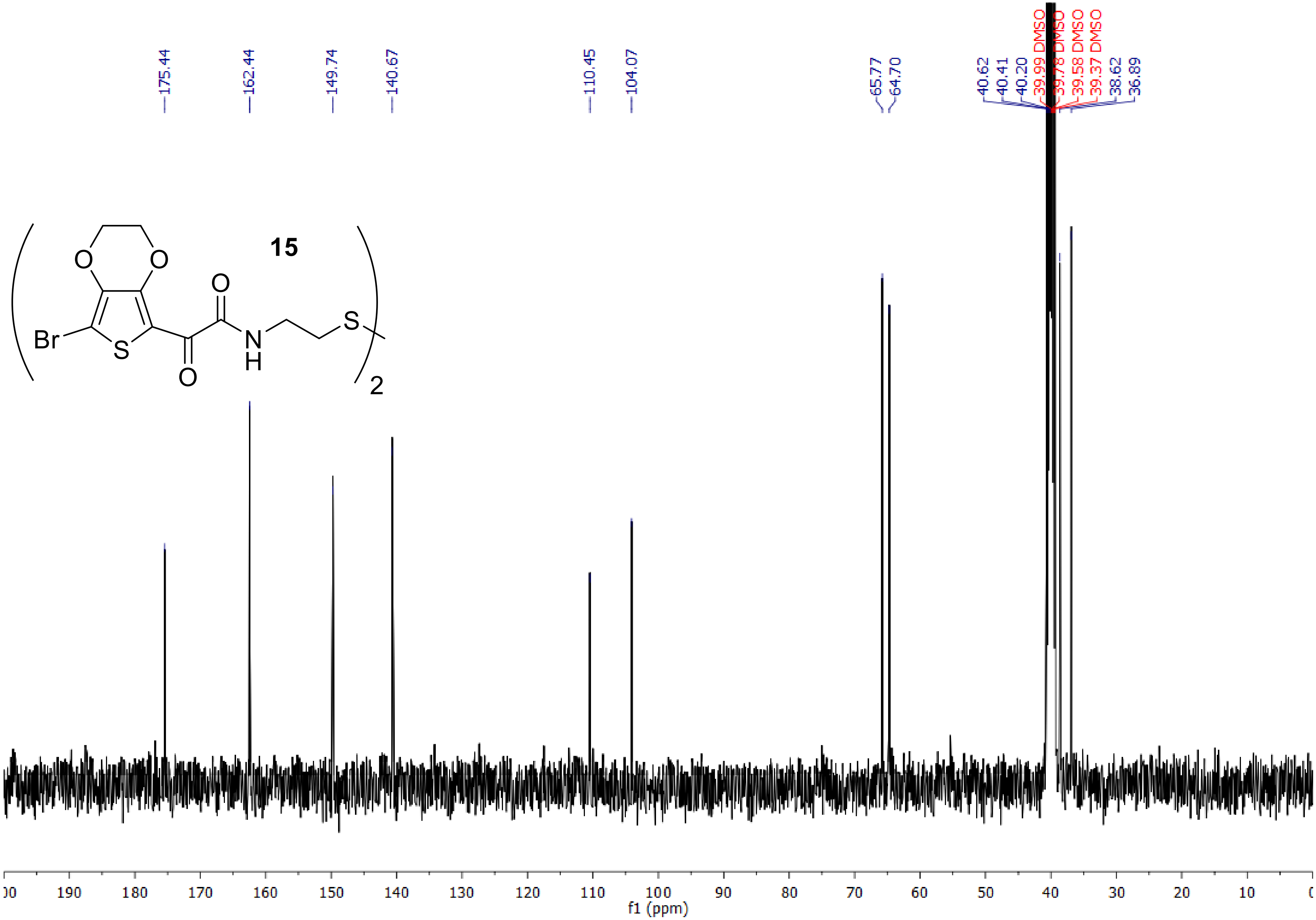

^{1}H NMR (400 MHz, $CDCl_3$)

Figure S32. ^{1}H NMR of **16**

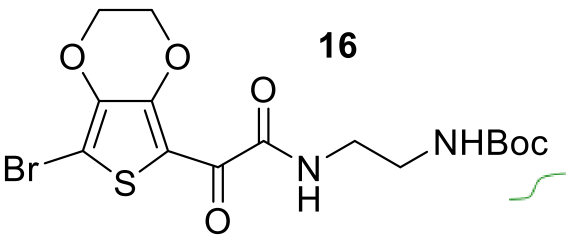

16

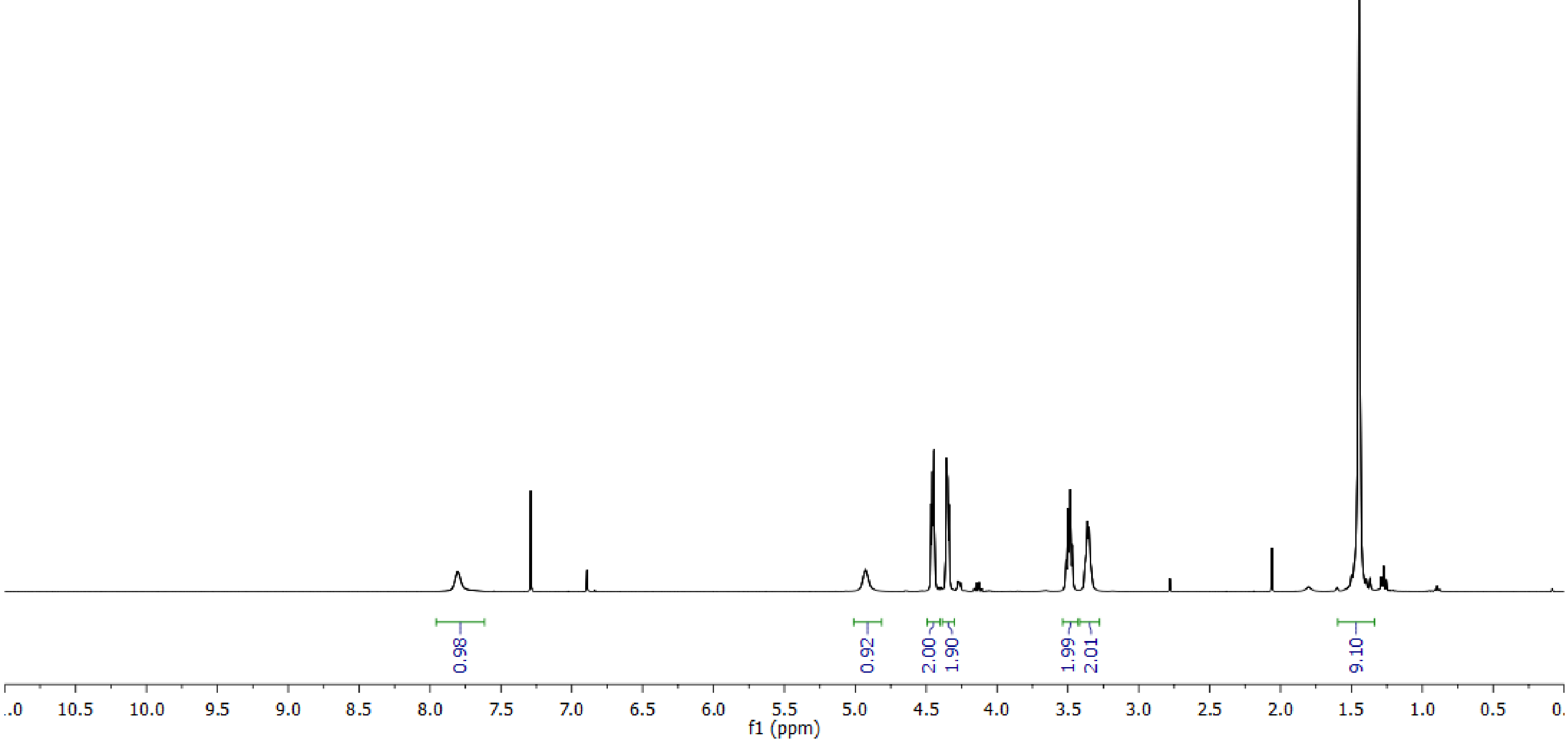

^{13}C NMR (100 MHz, $CDCl_3$)

Figure S33. ^{13}C NMR of **16**

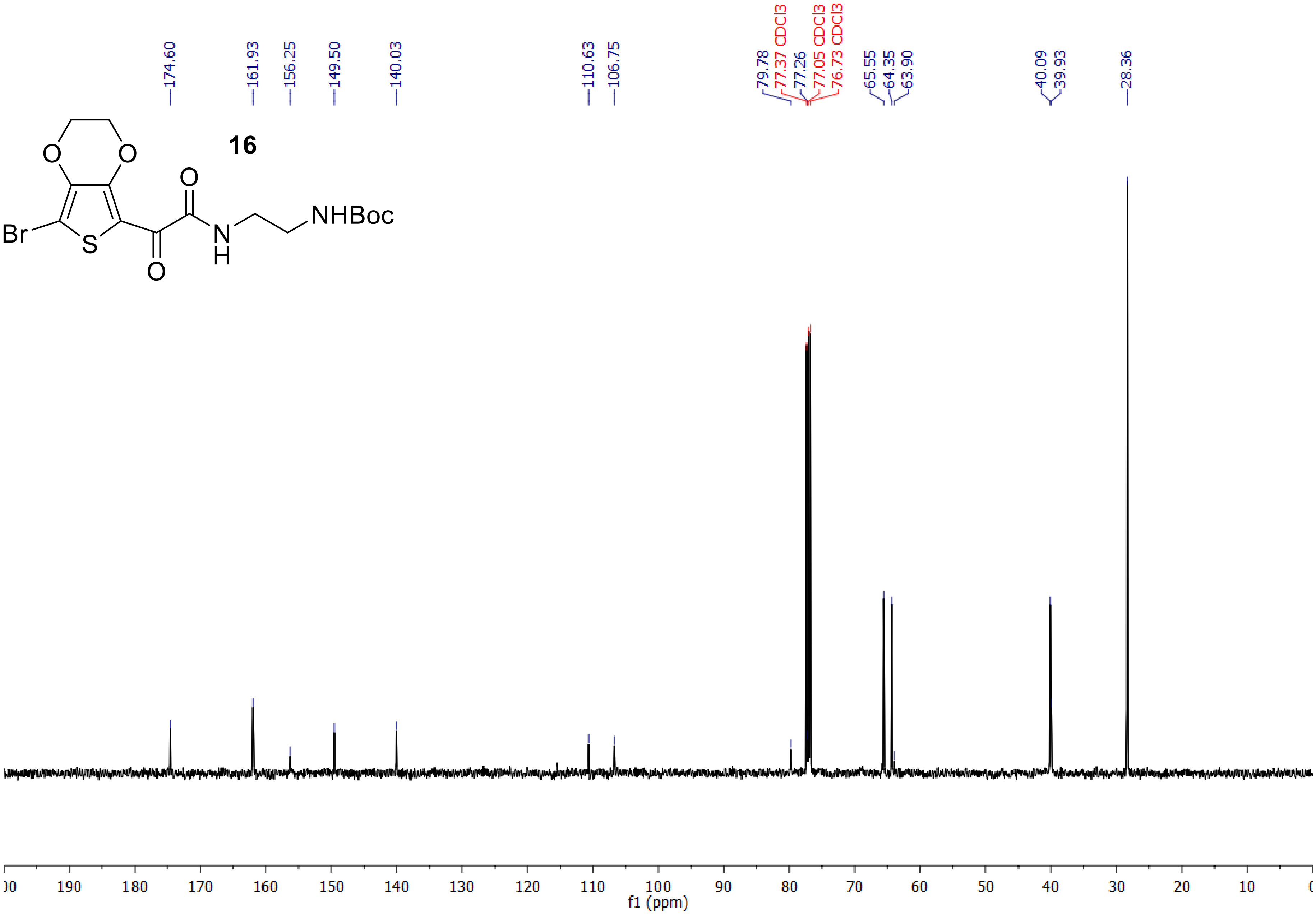

^{1}H NMR (400 MHz, $CDCl_3$)

Figure S34. ^{1}H NMR of **17**

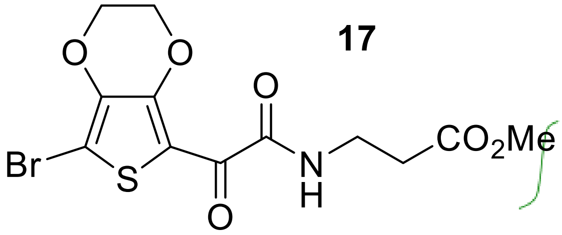

17

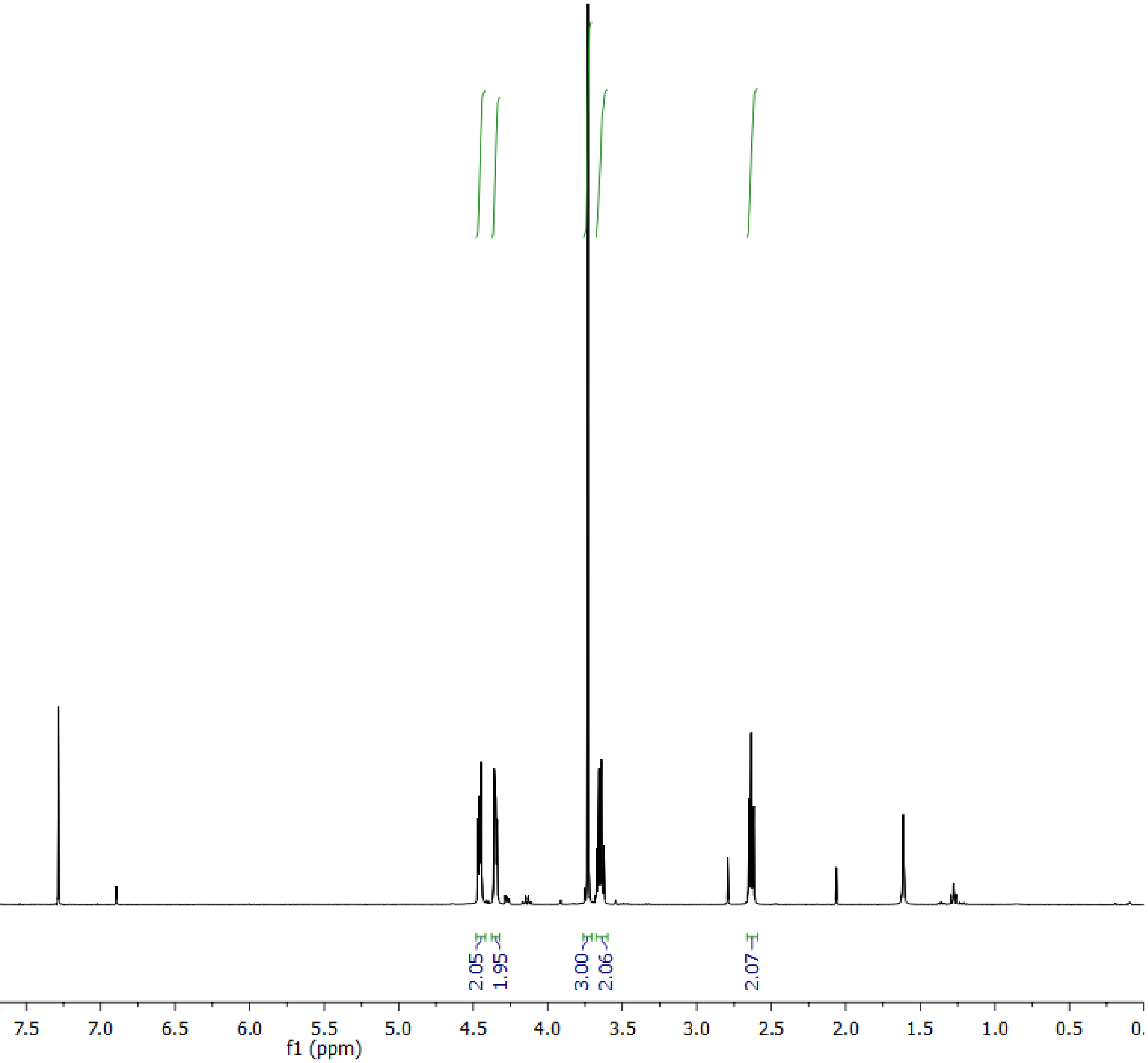

^{13}C NMR (100 MHz, $CDCl_3$)

Figure S35. ^{13}C NMR of **17**

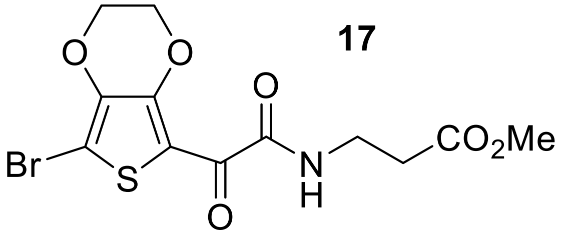

17

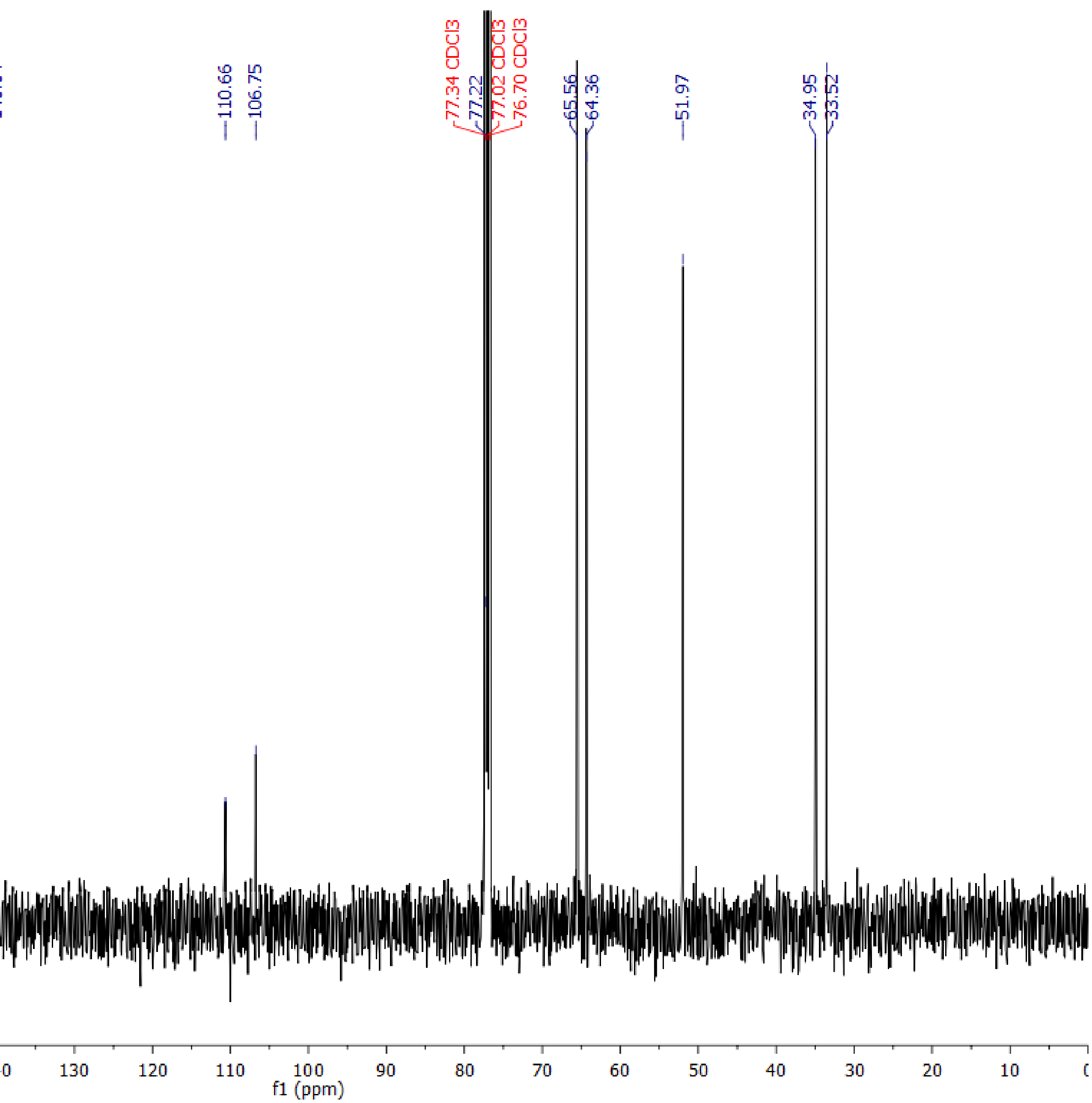

^{1}H NMR (400 MHz, $CDCl_3$)

Figure S36. ^{1}H NMR of **18**

18

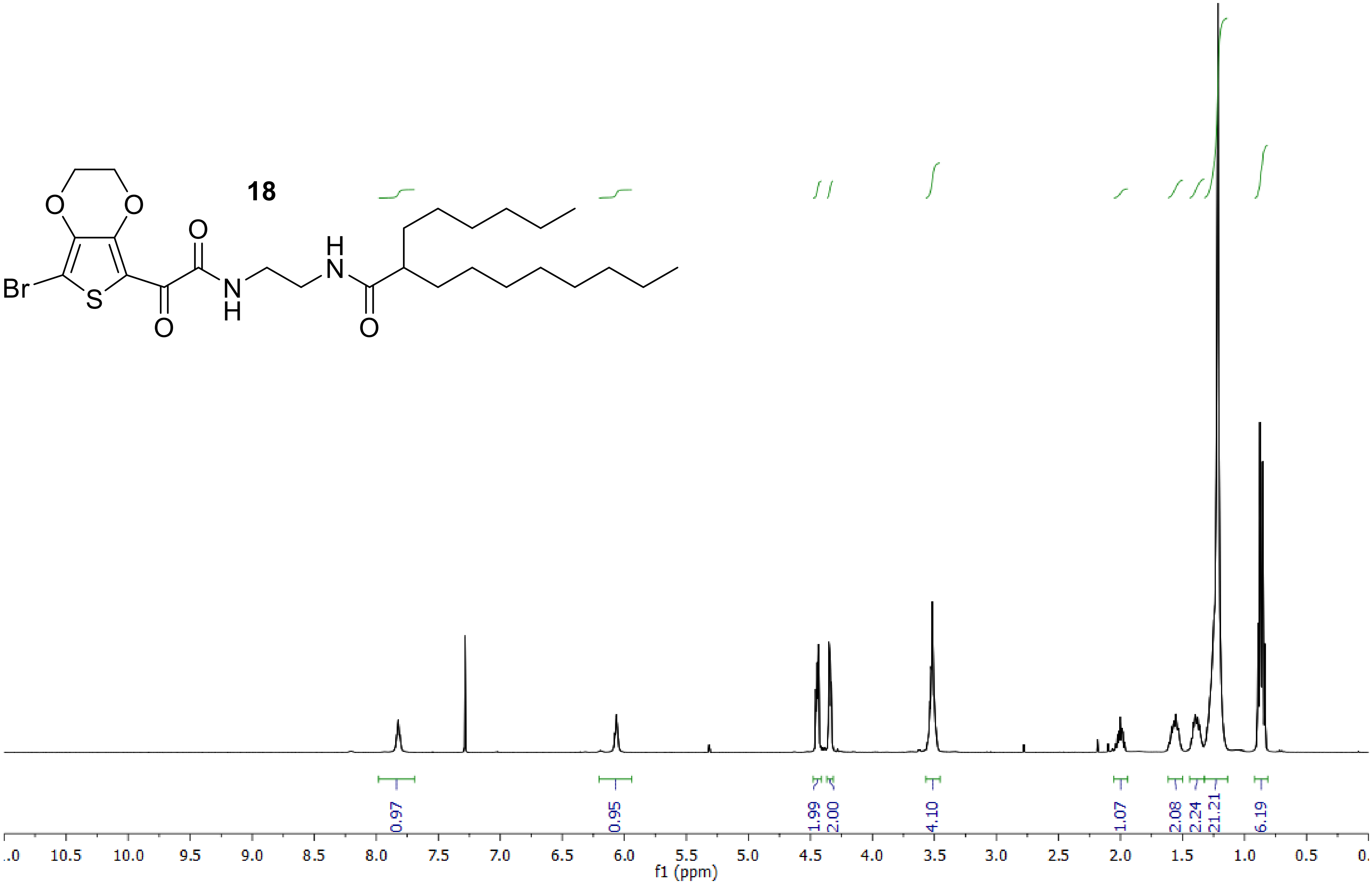

^{13}C NMR (100 MHz, $CDCl_3$)

Figure S37. ^{13}C NMR of **18**

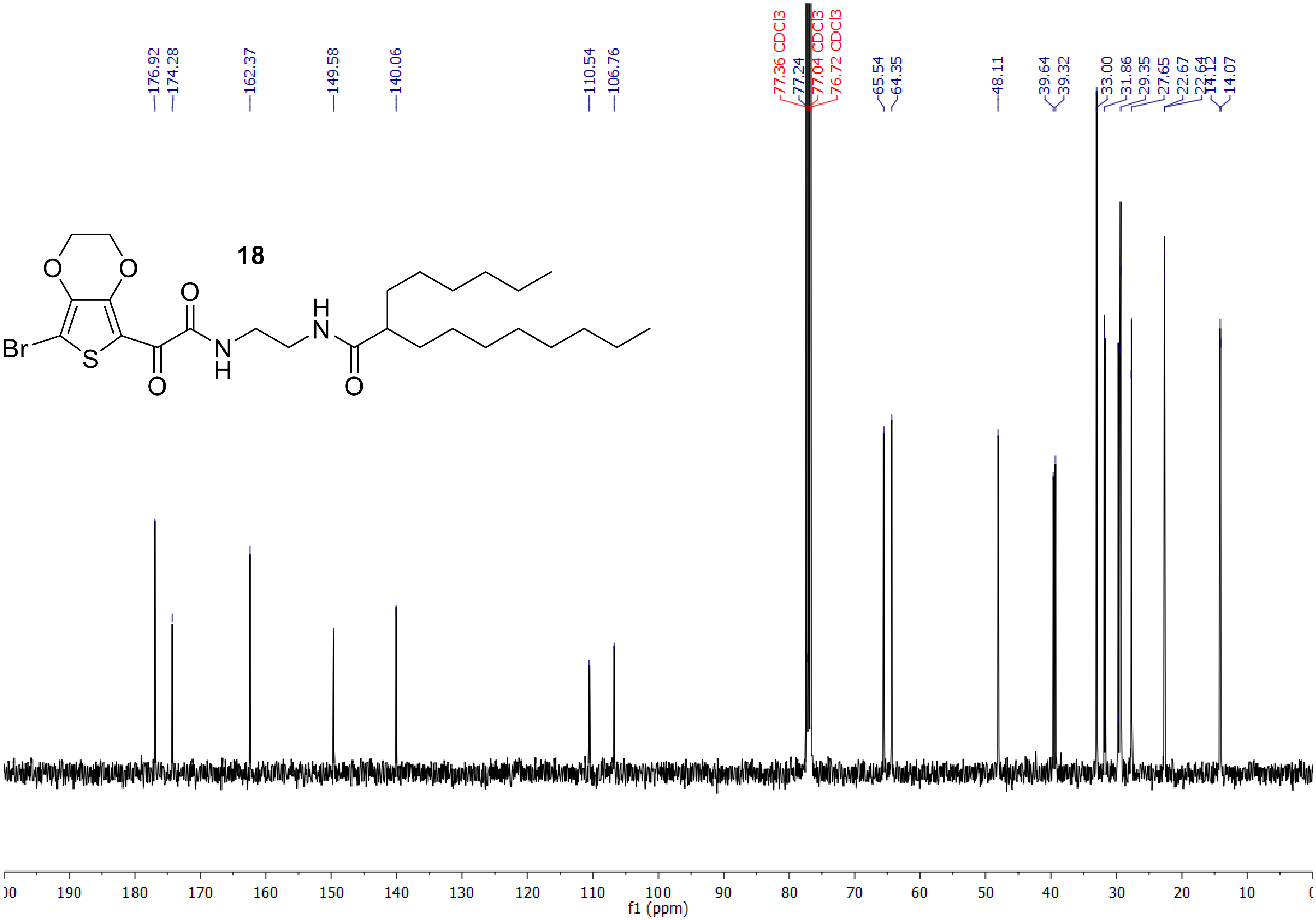

^{1}H NMR (400 MHz, $CDCl_3$)

Figure S38. ^{1}H NMR of **19**

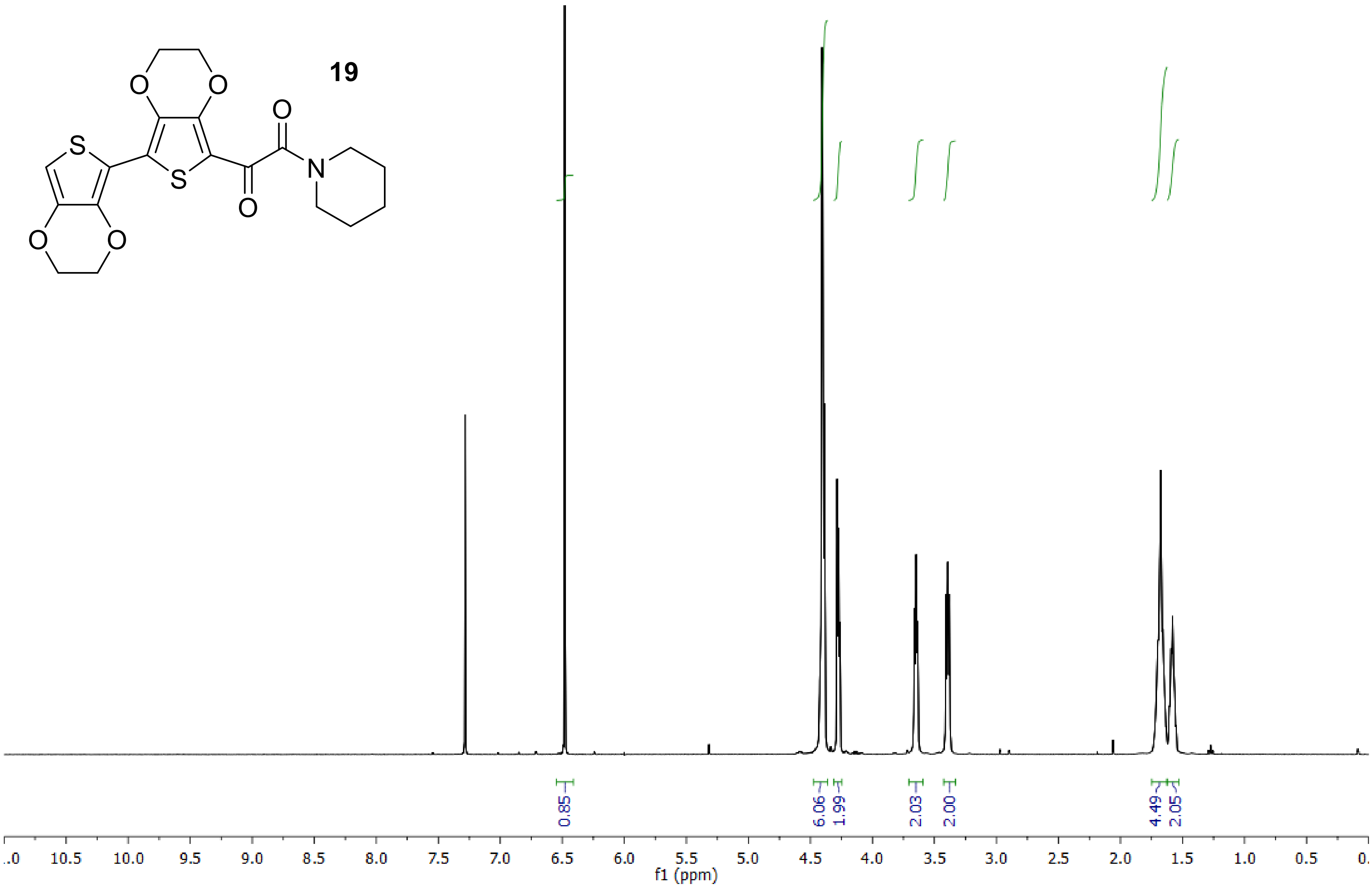

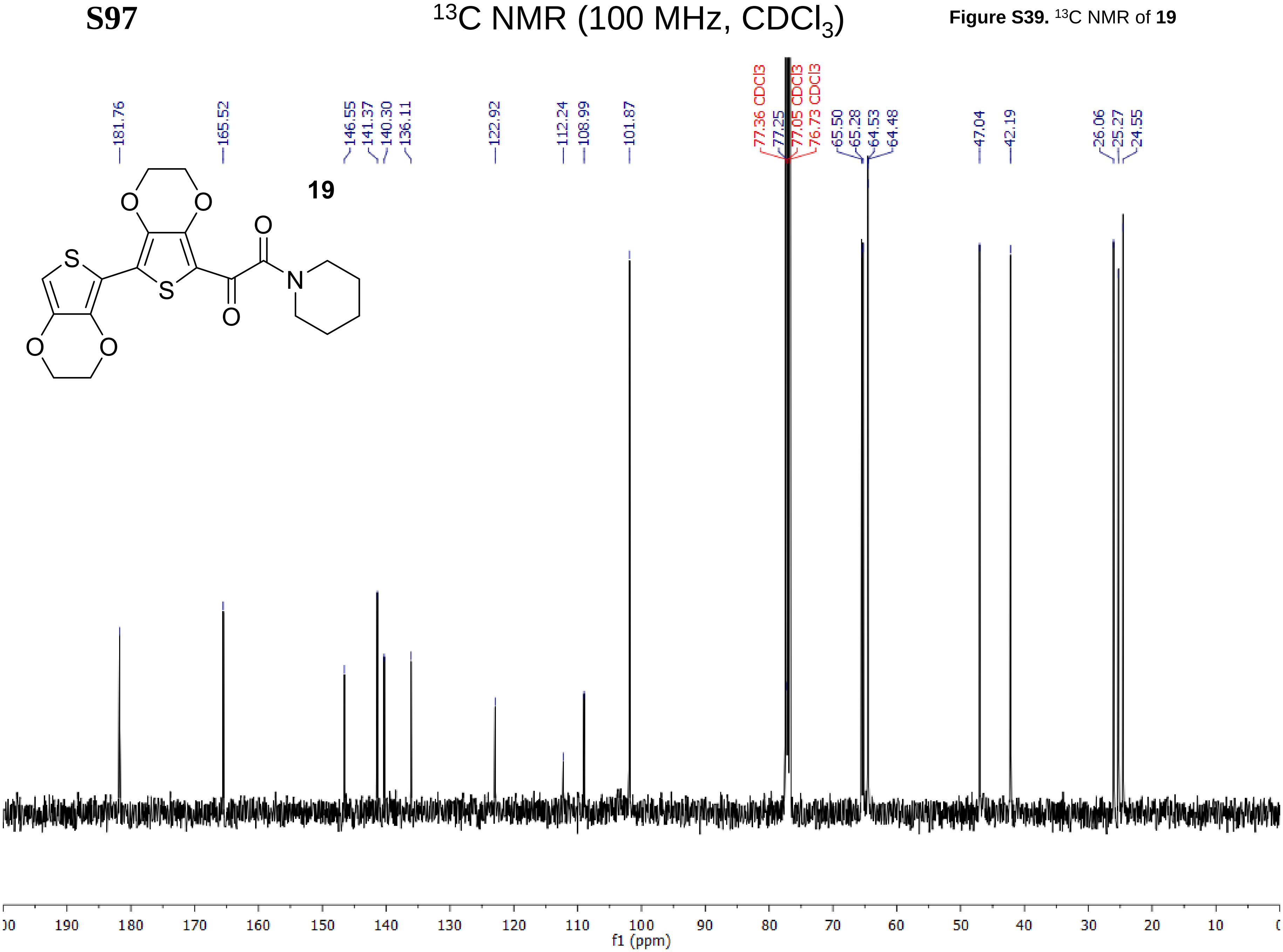

Figure S39. ^{13}C NMR of **19**

^{1}H NMR (400 MHz, $CDCl_3$)

Figure S40. ^{1}H NMR of **98**

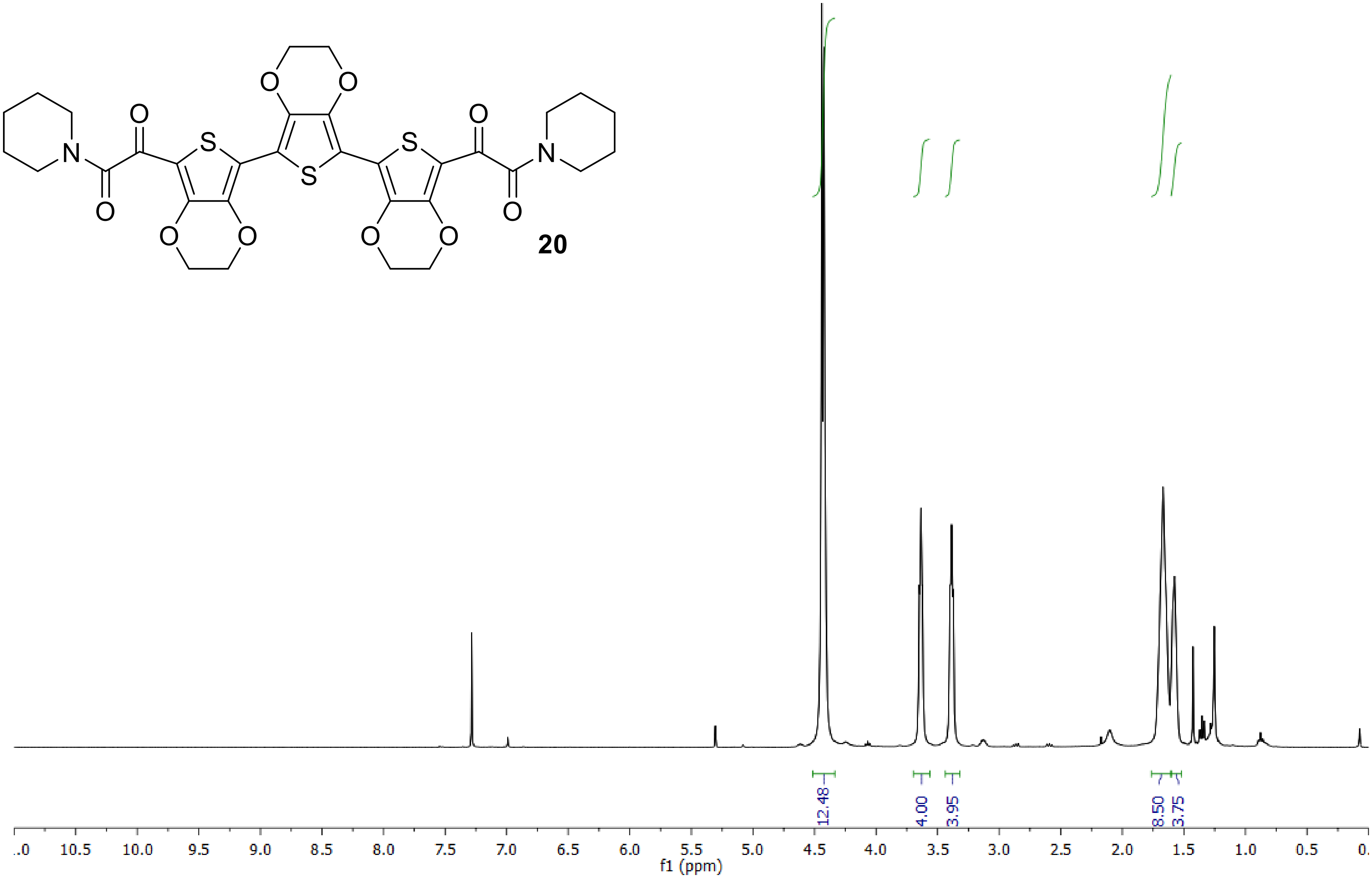

^{13}C NMR (100 MHz, $CDCl_3$)

Figure S41. ^{13}C NMR of **20**

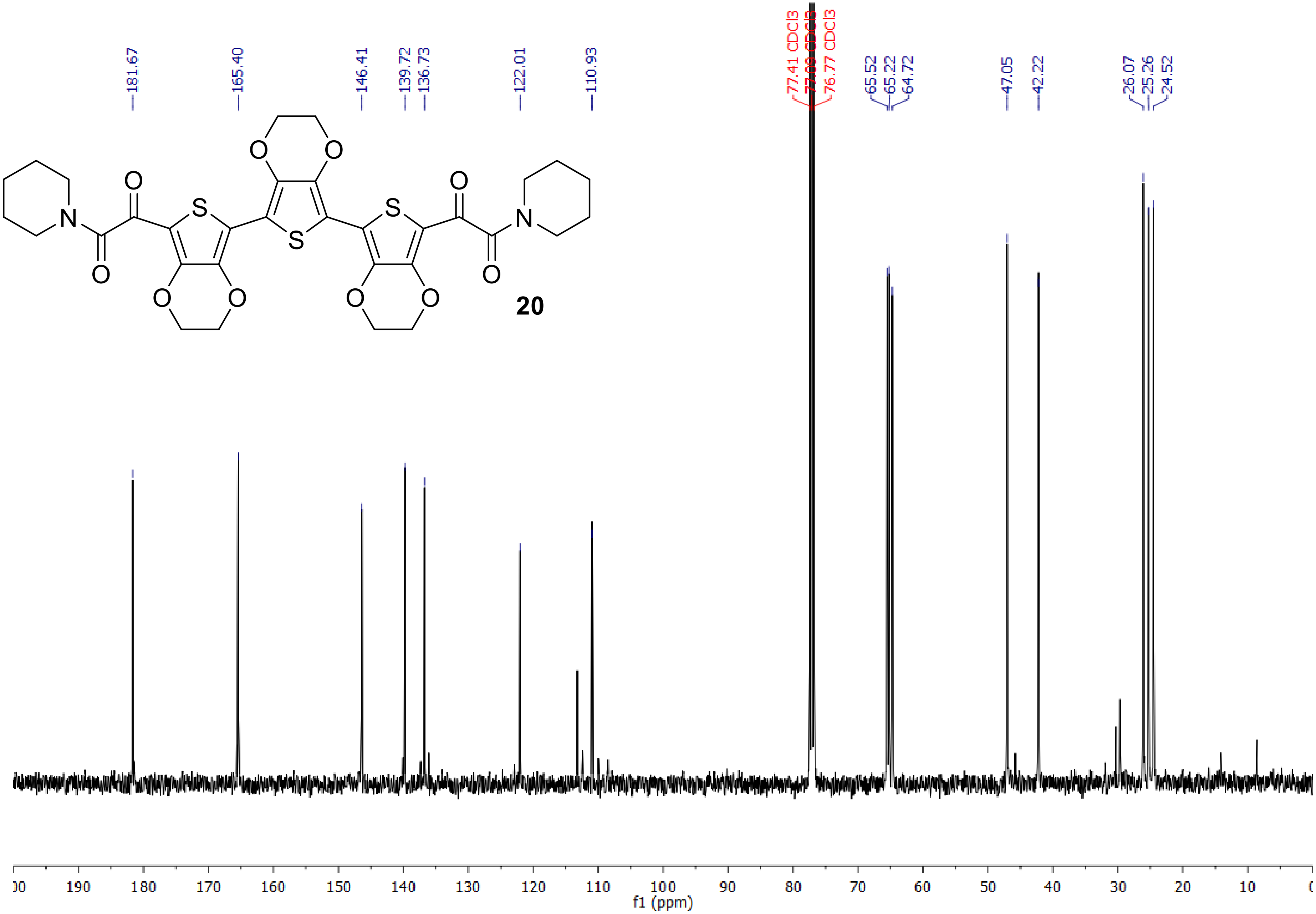

^1H NMR (400 MHz, $CDCl_3$)

Figure S42. ^1H NMR of **21**

21

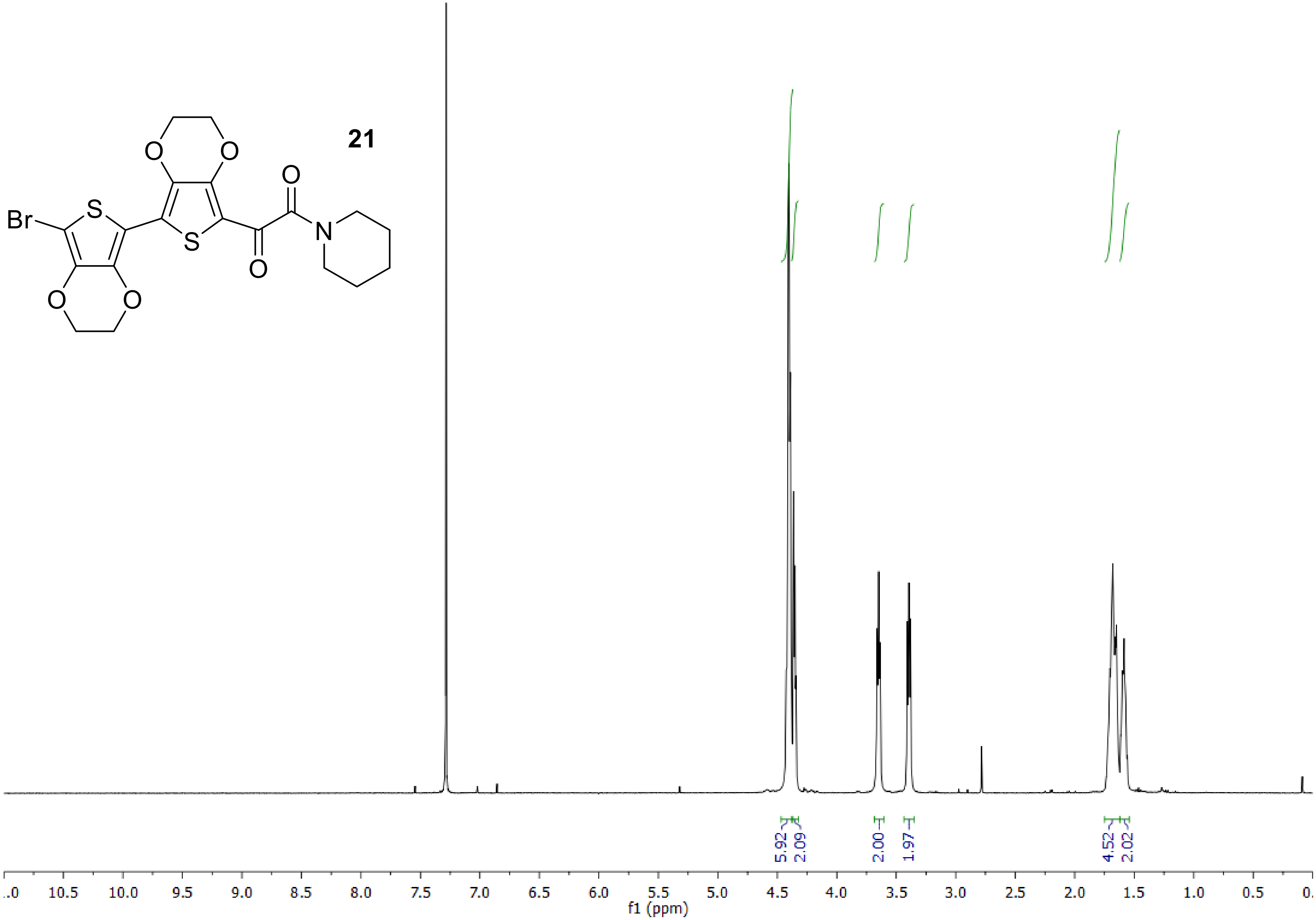

^{13}C NMR (100 MHz, $CDCl_3$)

Figure S43. ^{13}C NMR of **21**

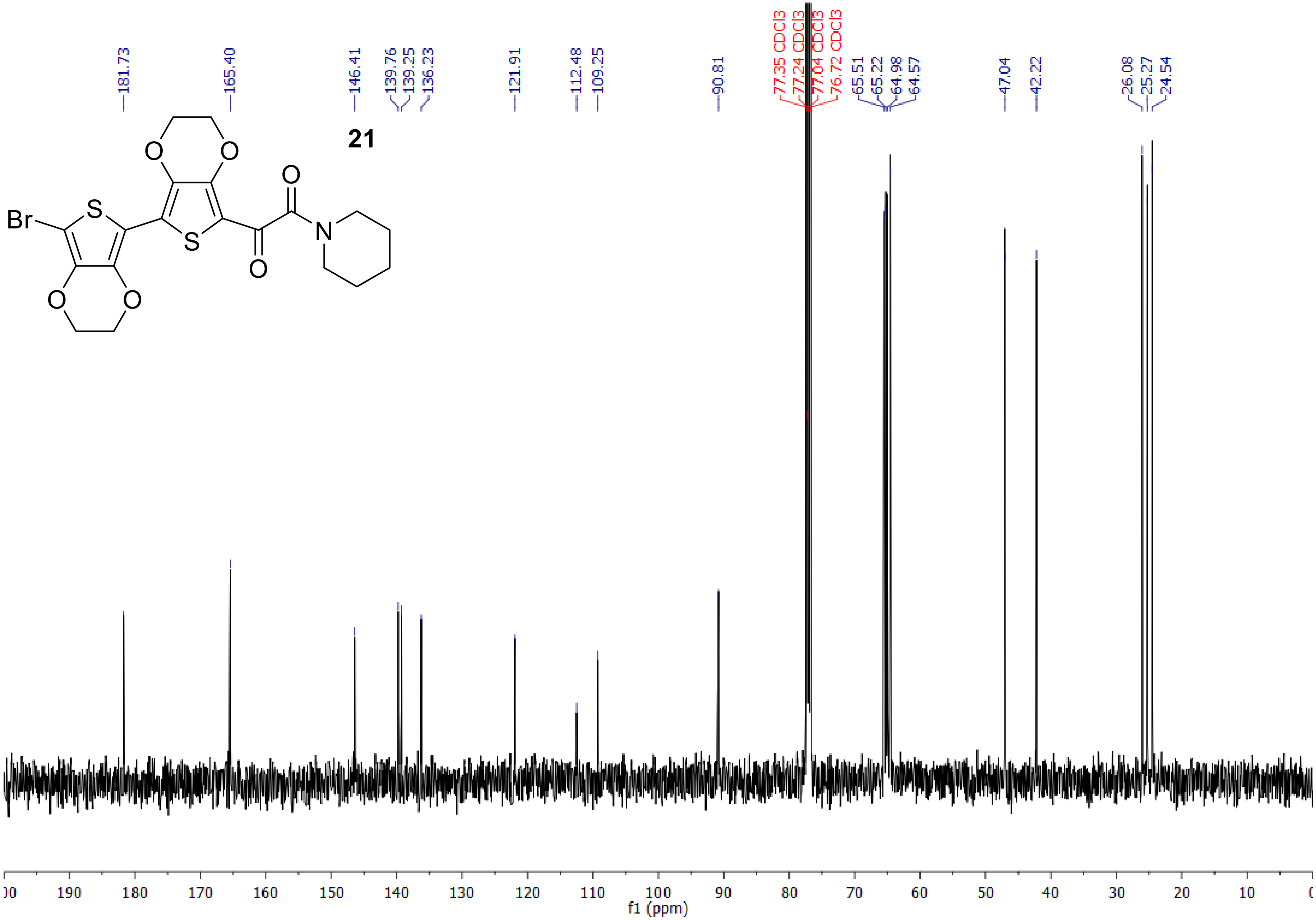

Figure S44. ^{1}H NMR of **22**

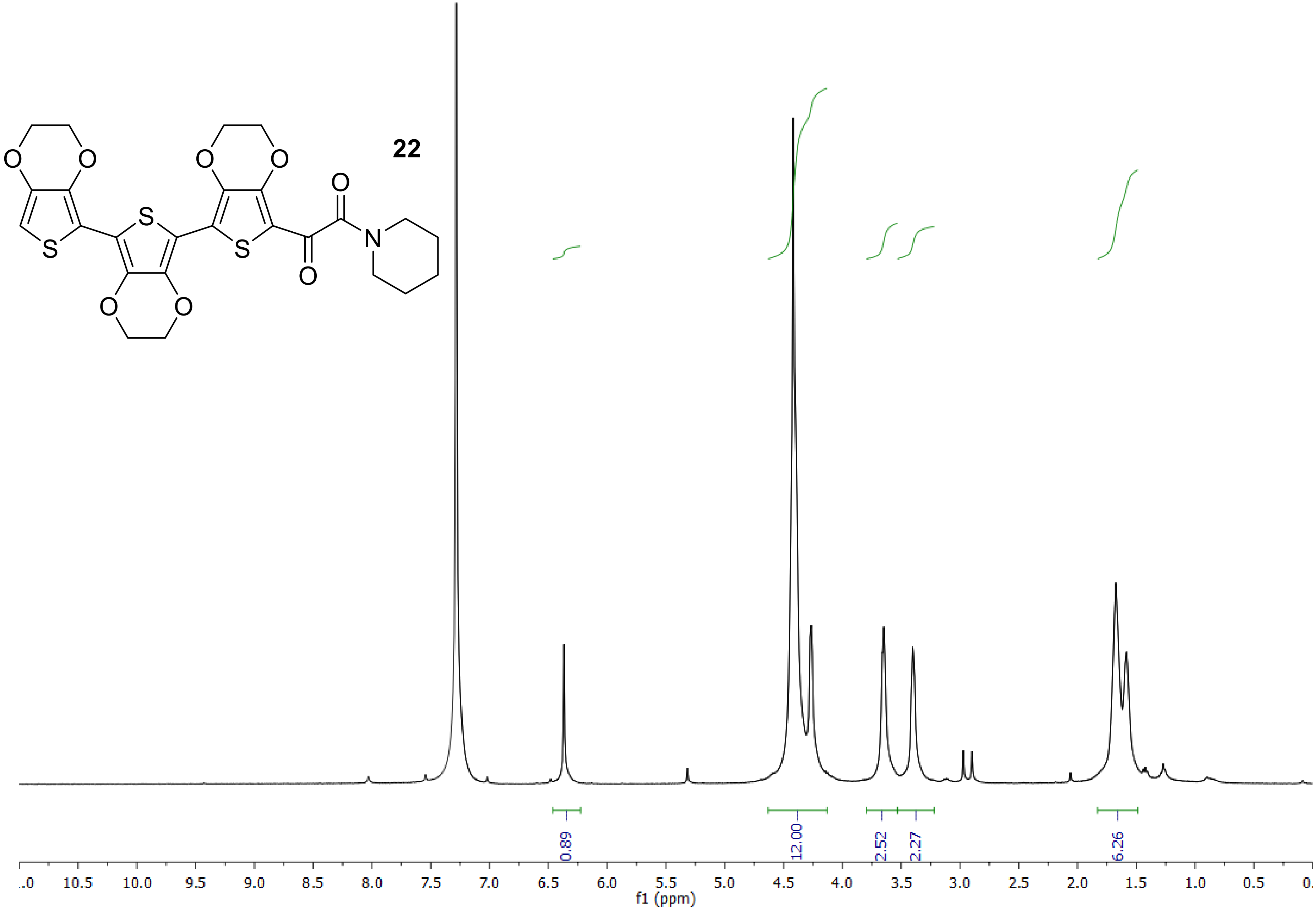

^{13}C NMR (100 MHz, $CDCl_3$)

Figure S45. ^{13}C NMR of **22**

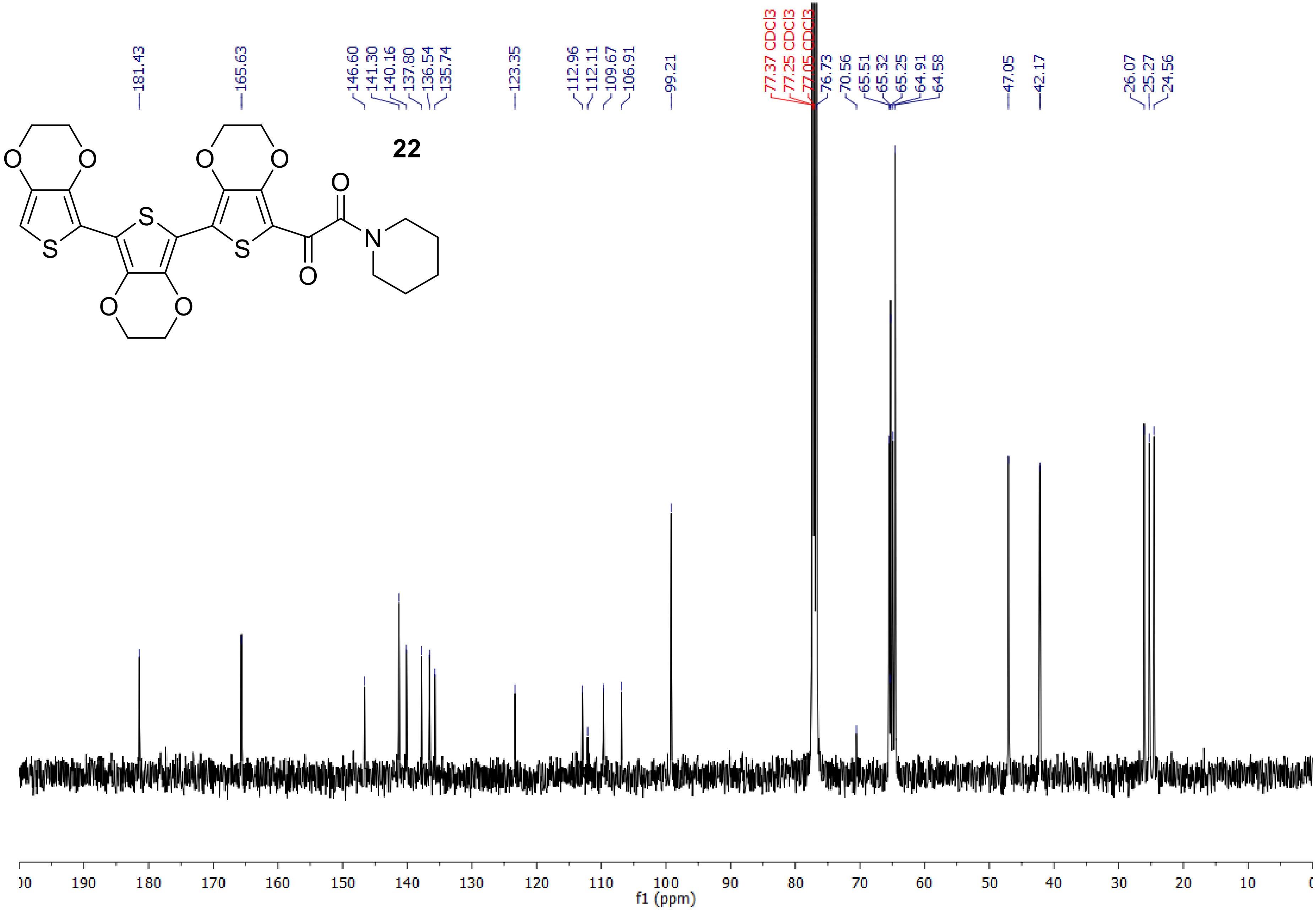

^{1}H NMR (400 MHz, $CDCl_3$)

Figure S46. ^{1}H NMR of **24**

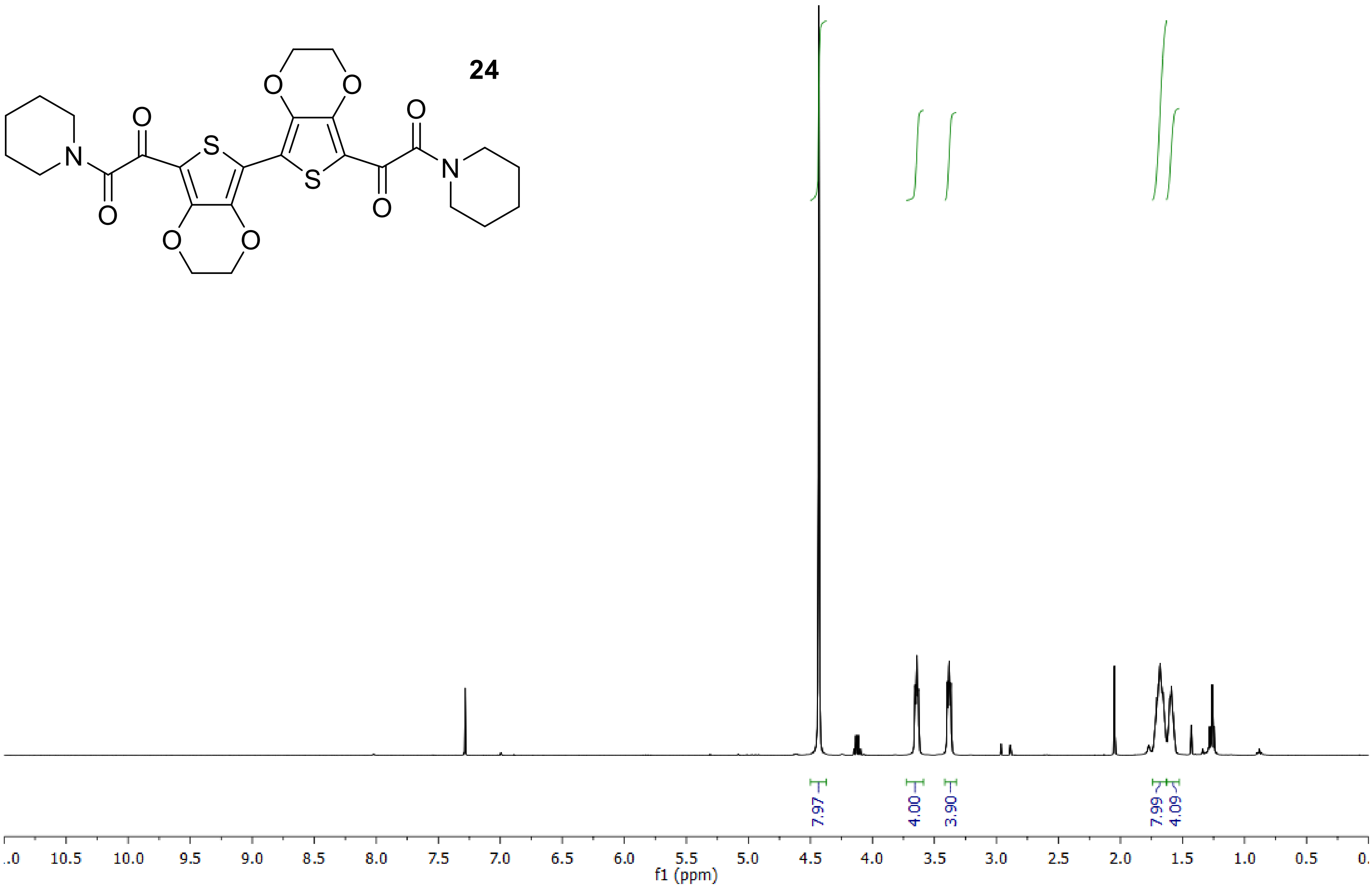

^{13}C NMR (100 MHz, $CDCl_3$)

Figure S47. ^{13}C NMR of **24**

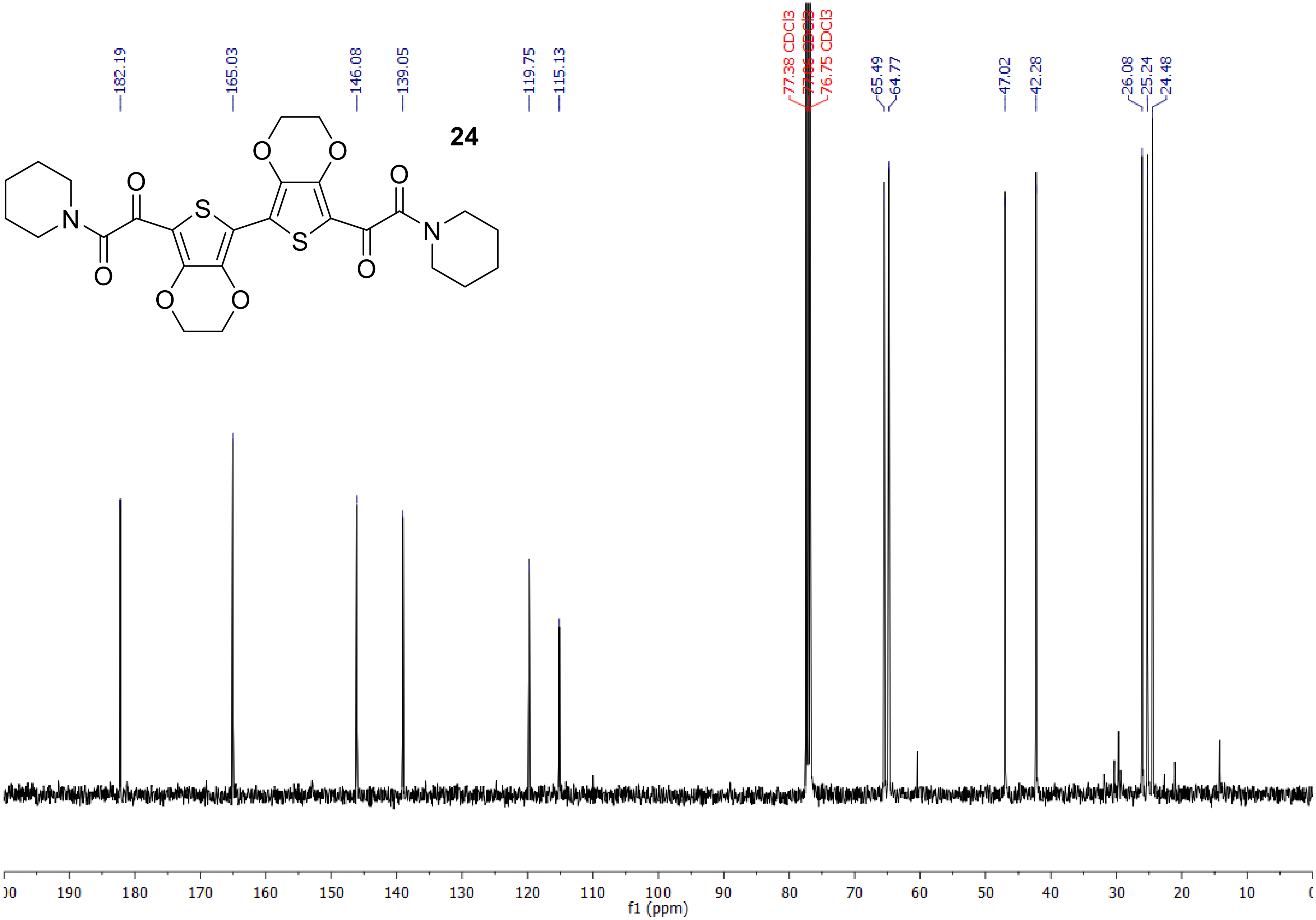

^{1}H NMR (400 MHz, $CDCl_3$)

Figure S48. ^{1}H NMR of **26**

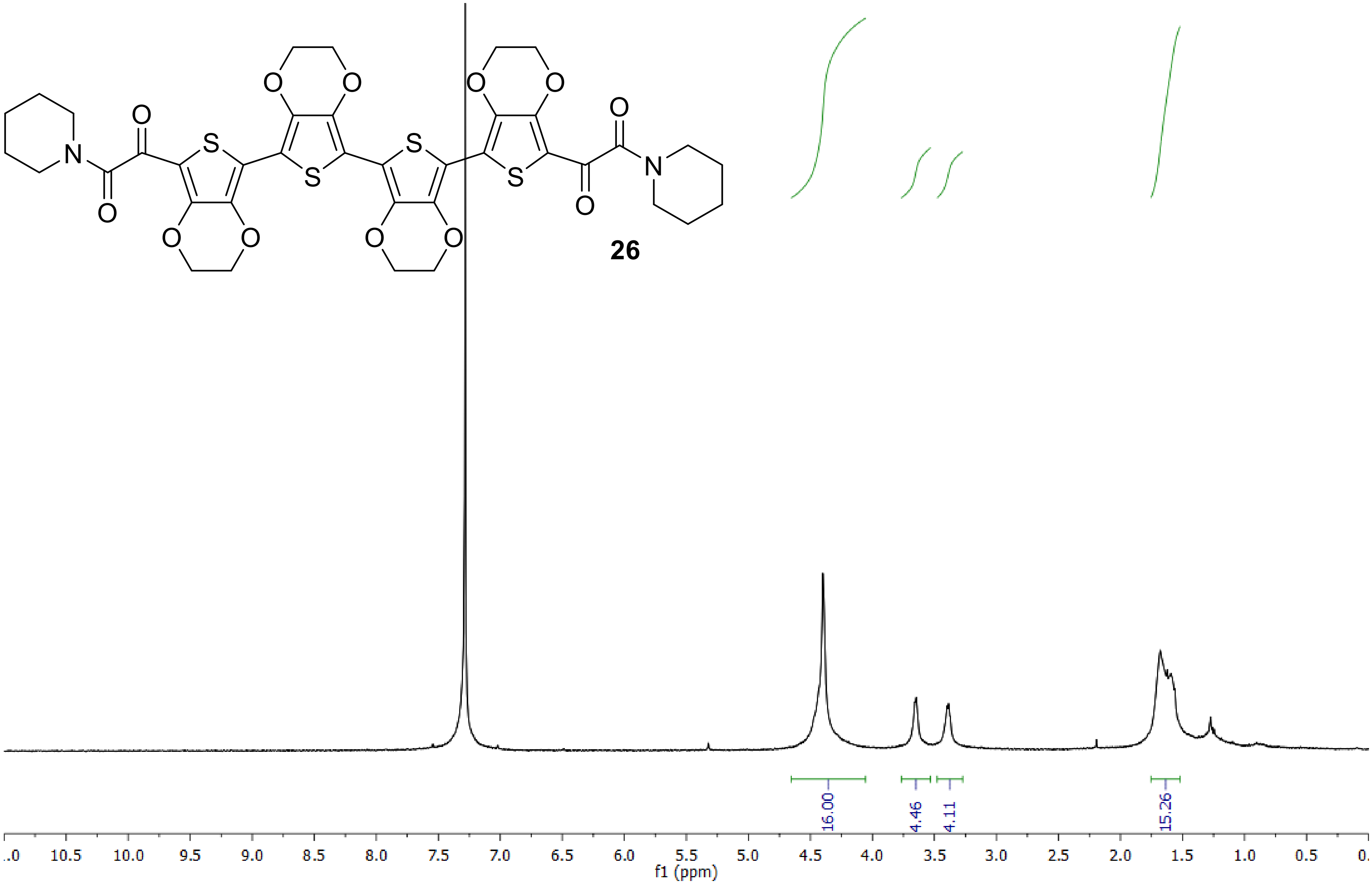

^{13}C NMR (125 MHz, $CDCl_3$)

Figure S49. ^{13}C NMR of **26**

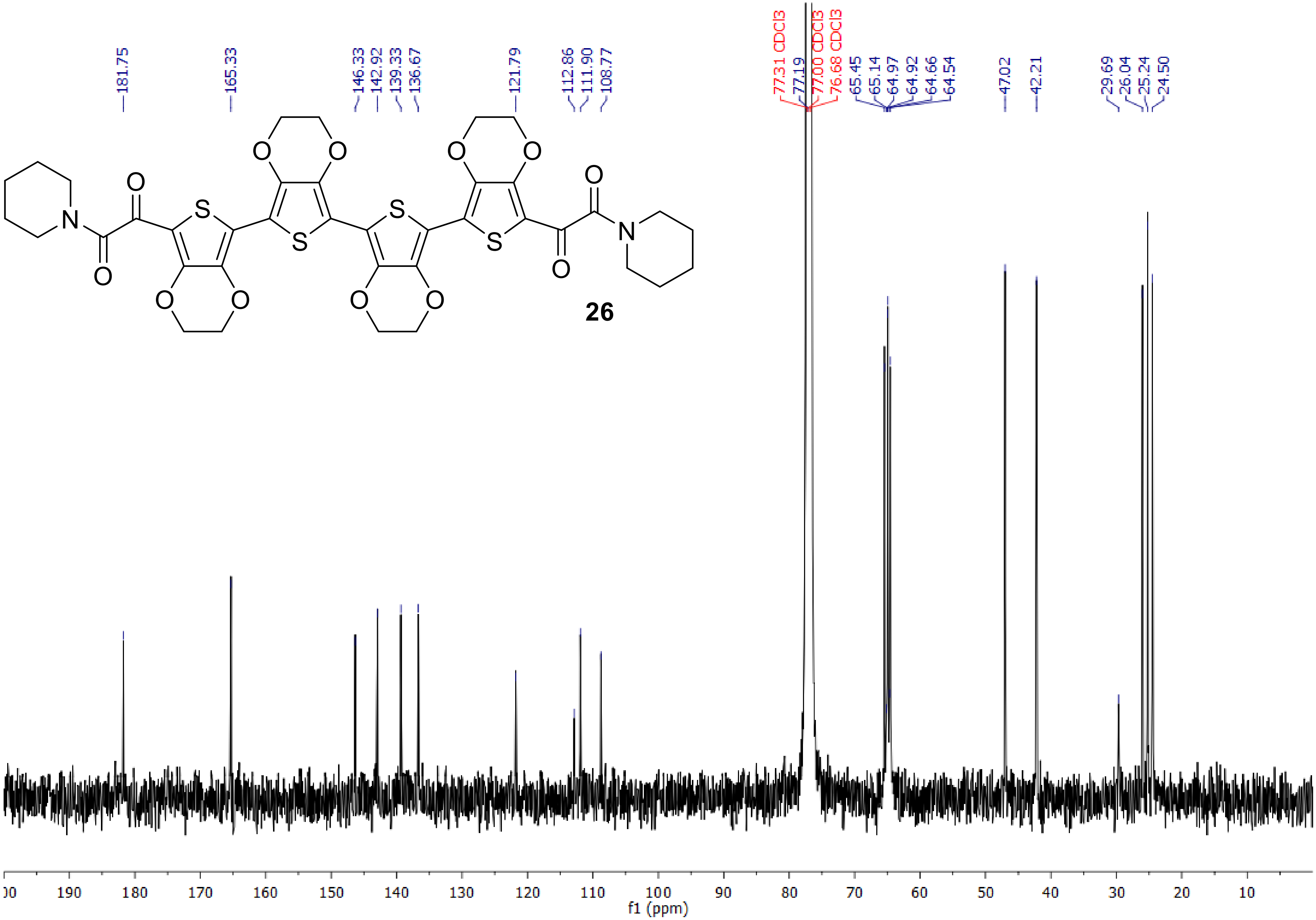

^{1}H NMR (400 MHz, $CDCl_3$)

Figure S50. ^{1}H NMR of **27**

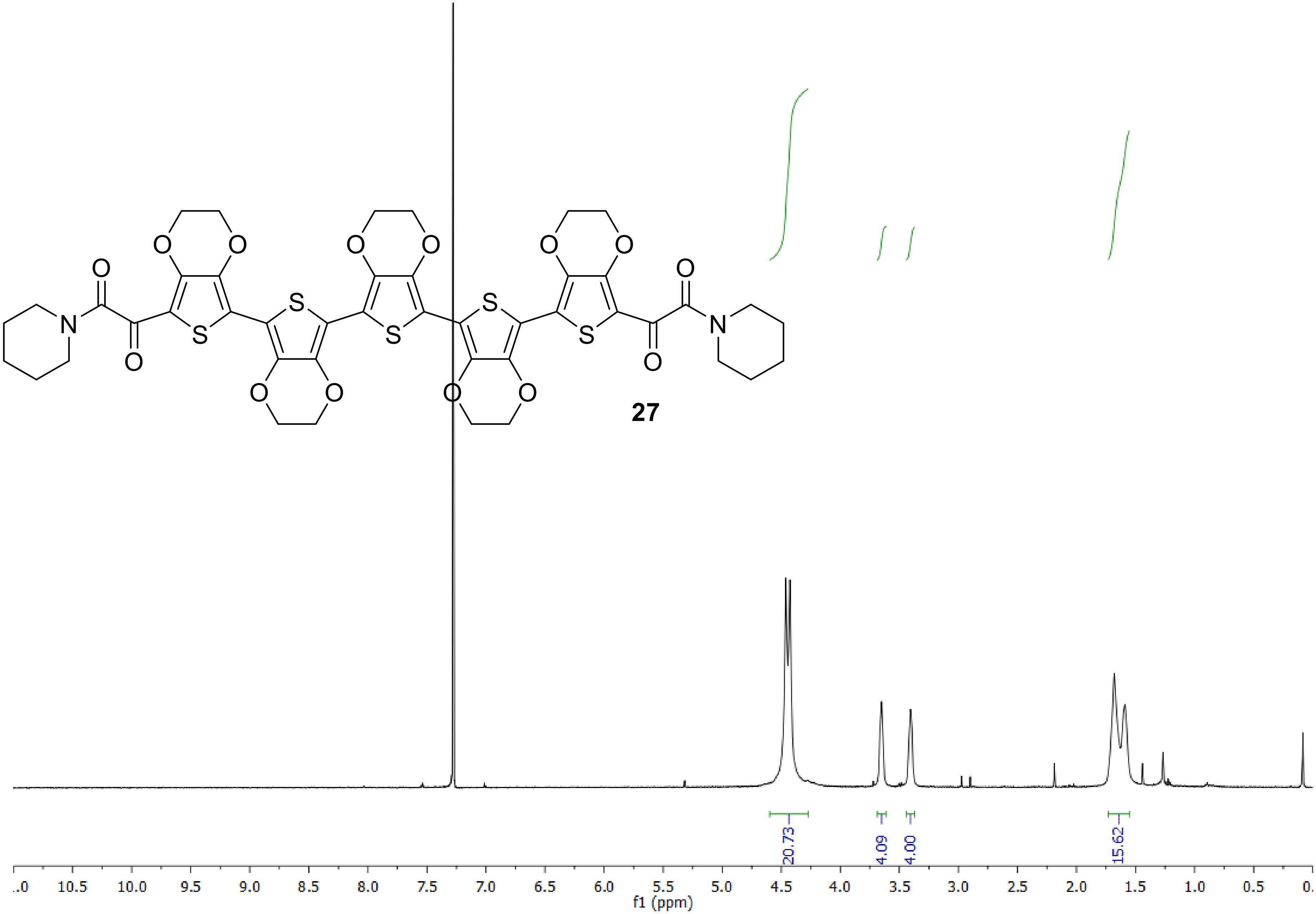

^{1}H NMR (400 MHz, $CDCl_3$)

Figure S51. ^{1}H NMR of **28**

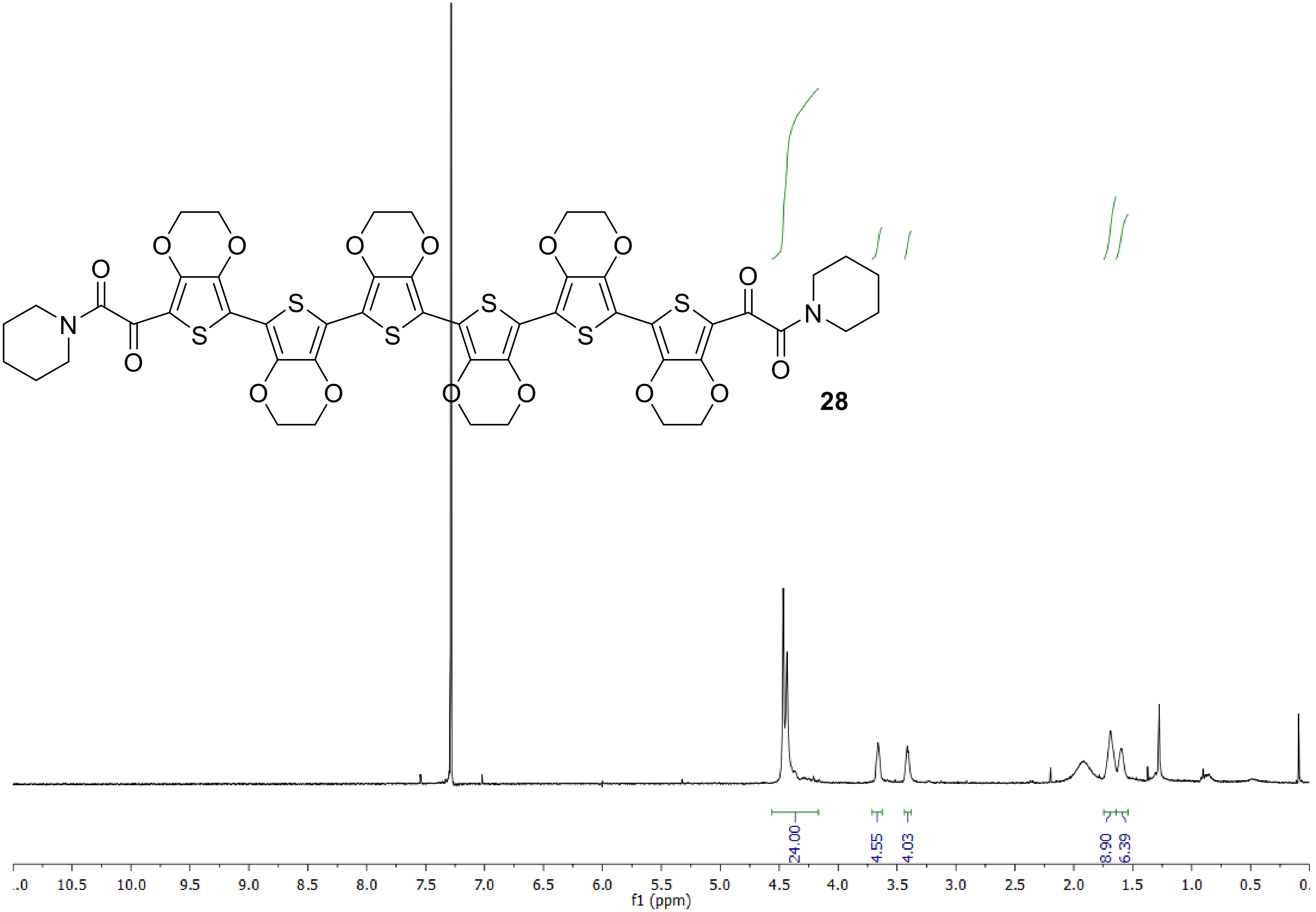

^{1}H NMR (400 MHz, $CDCl_3$)

Figure S52. ^{1}H NMR of **29**

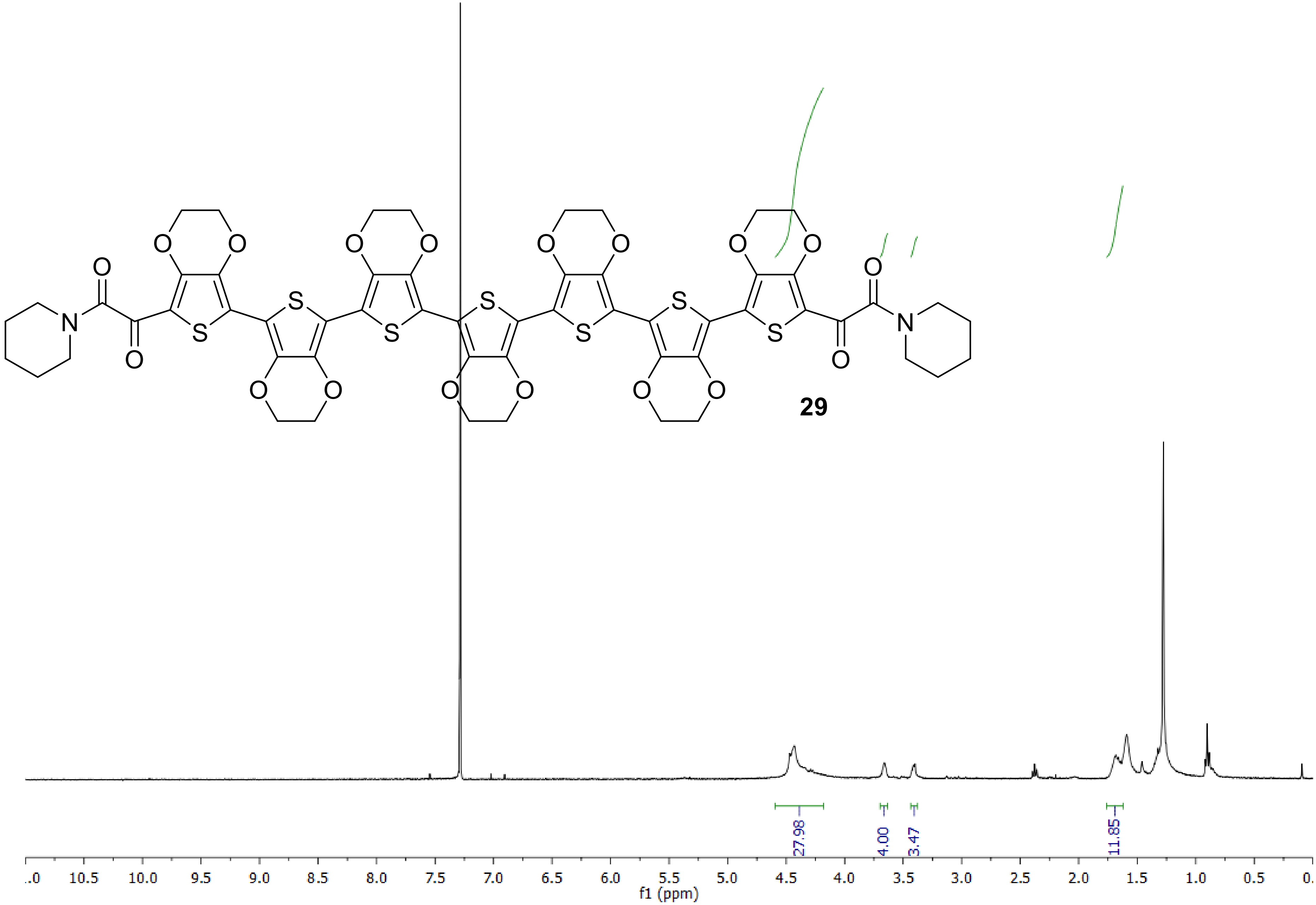

^{1}H NMR (400 MHz, $CDCl_3$)

Figure S53. ^{1}H NMR of **30**

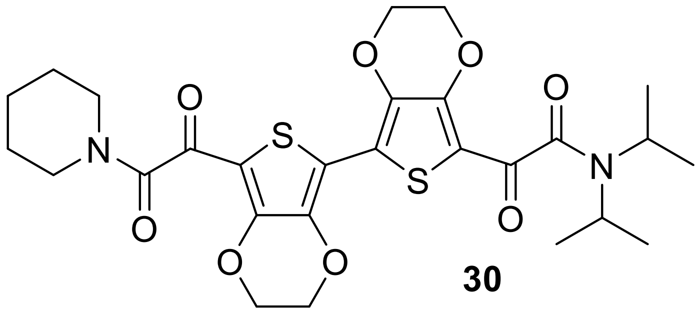

30

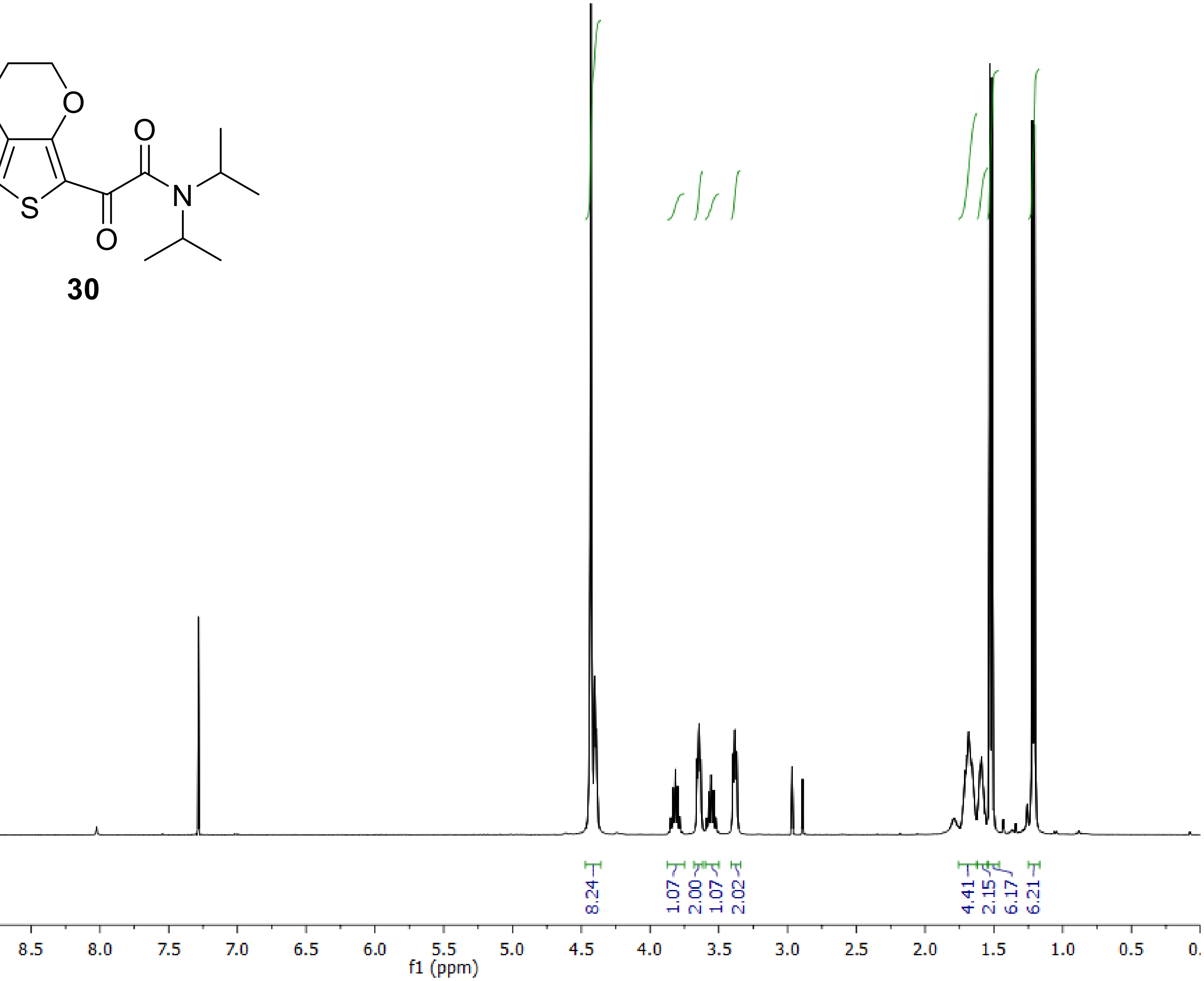

^{13}C NMR (100 MHz, $CDCl_3$)

Figure S54. ^{13}C NMR of **30**

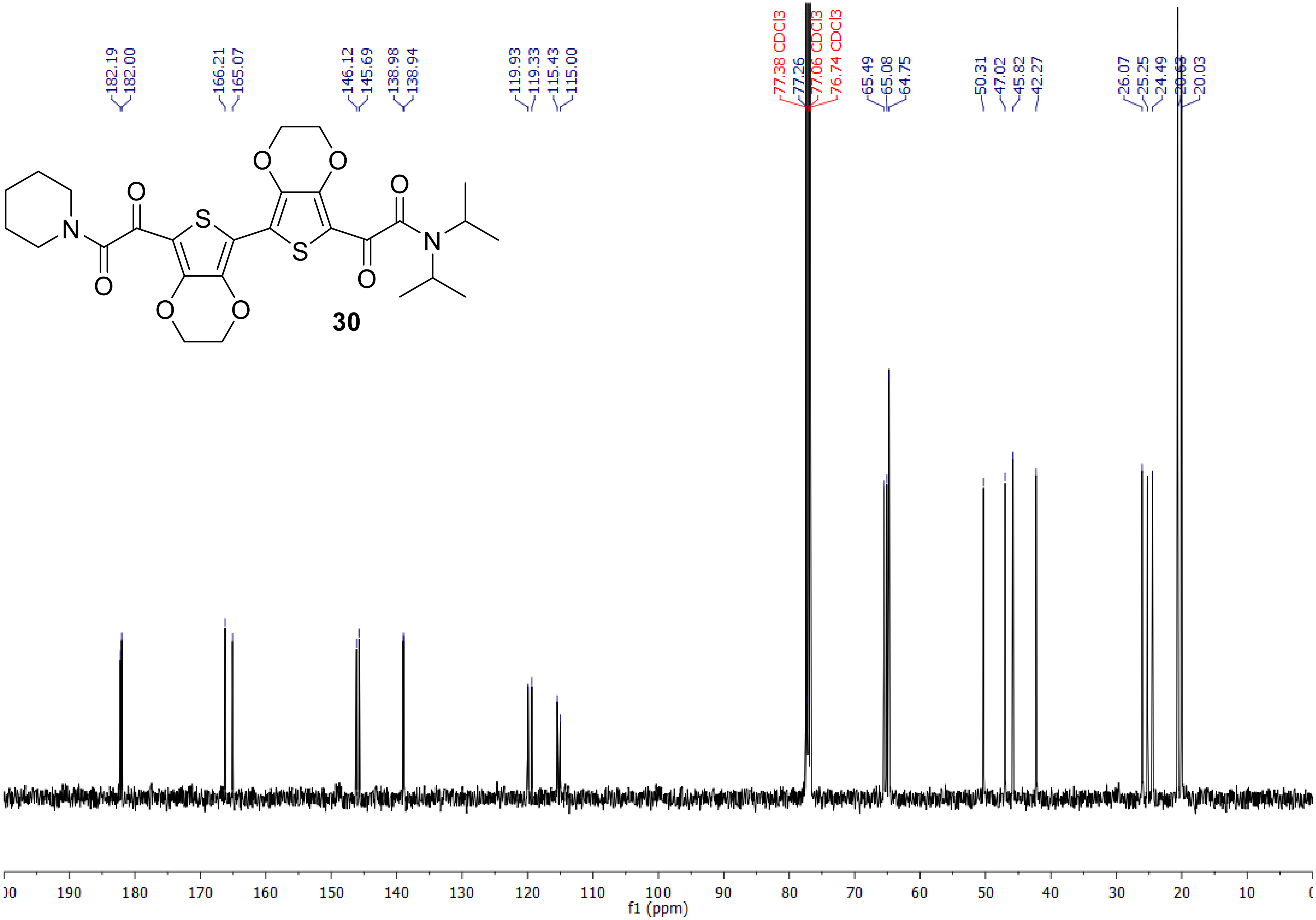

^1H NMR (400 MHz, $CDCl_3$)

Figure S55. ^1H NMR of **31**

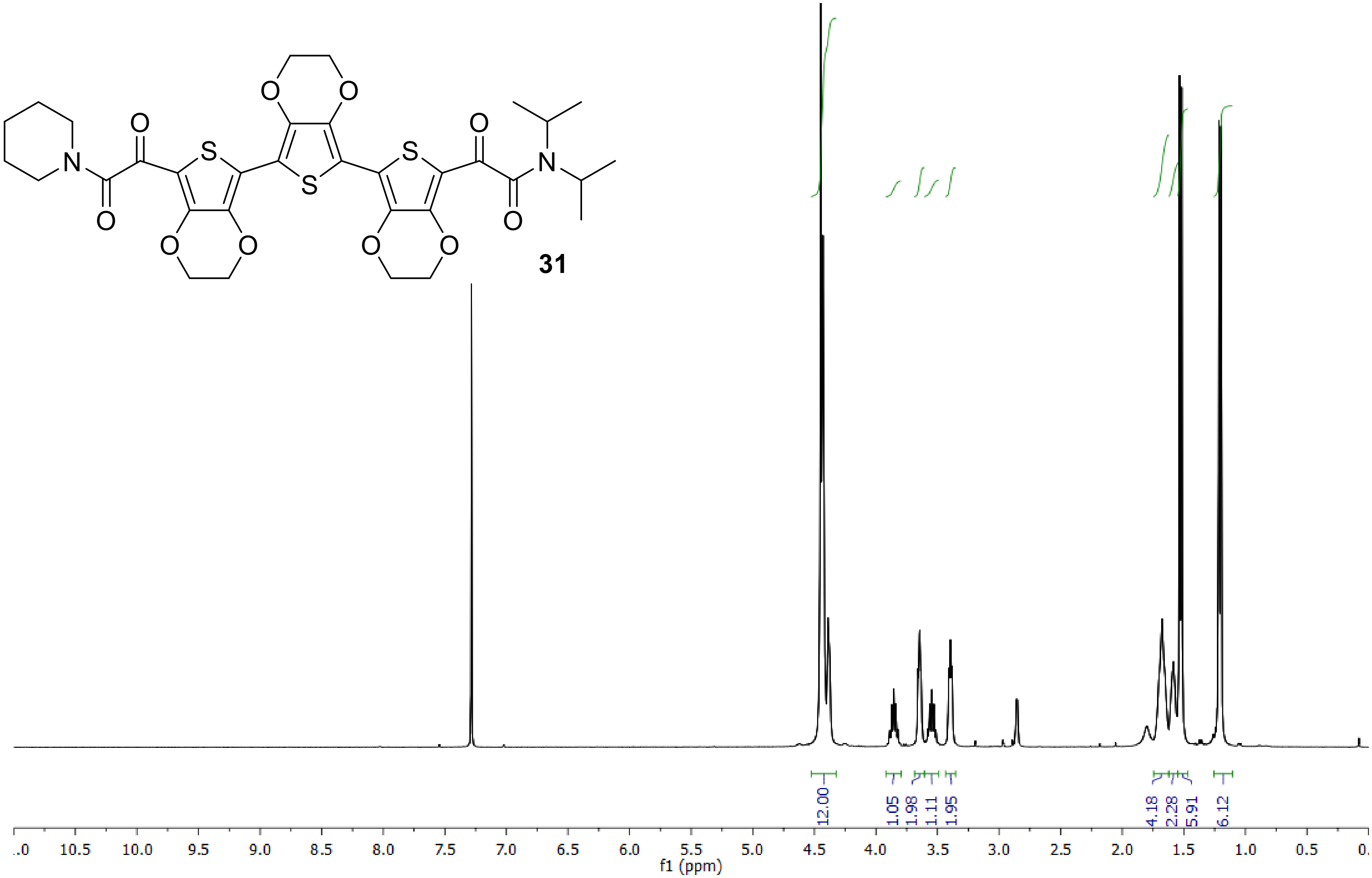

^{13}C NMR (100 MHz, $CDCl_3$)

Figure S56. ^{13}C NMR of **31**

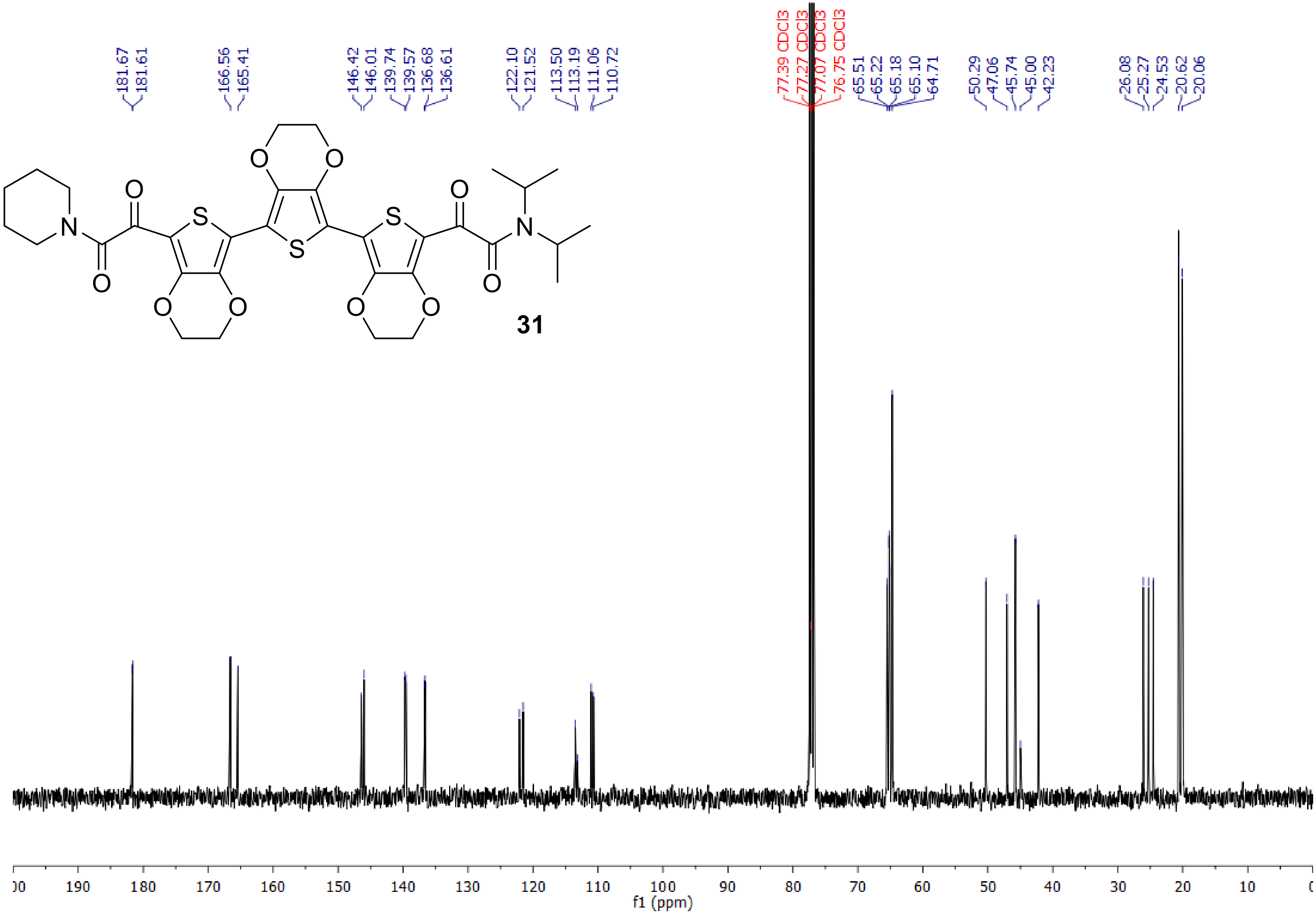

^{1}H NMR (400 MHz, $CDCl_3$)

Figure S57. ^{1}H NMR of **32**

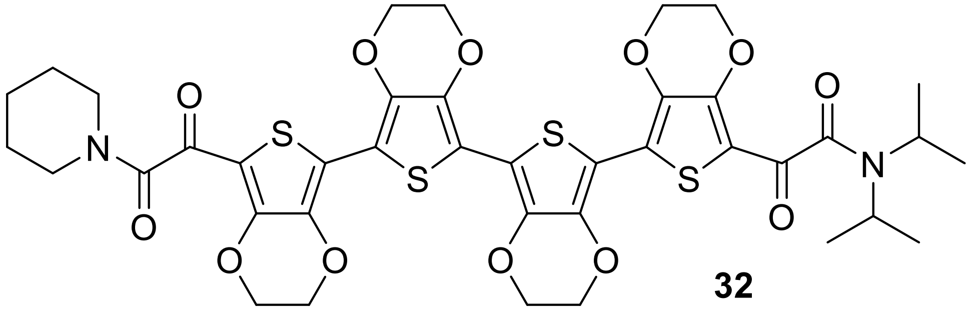

32

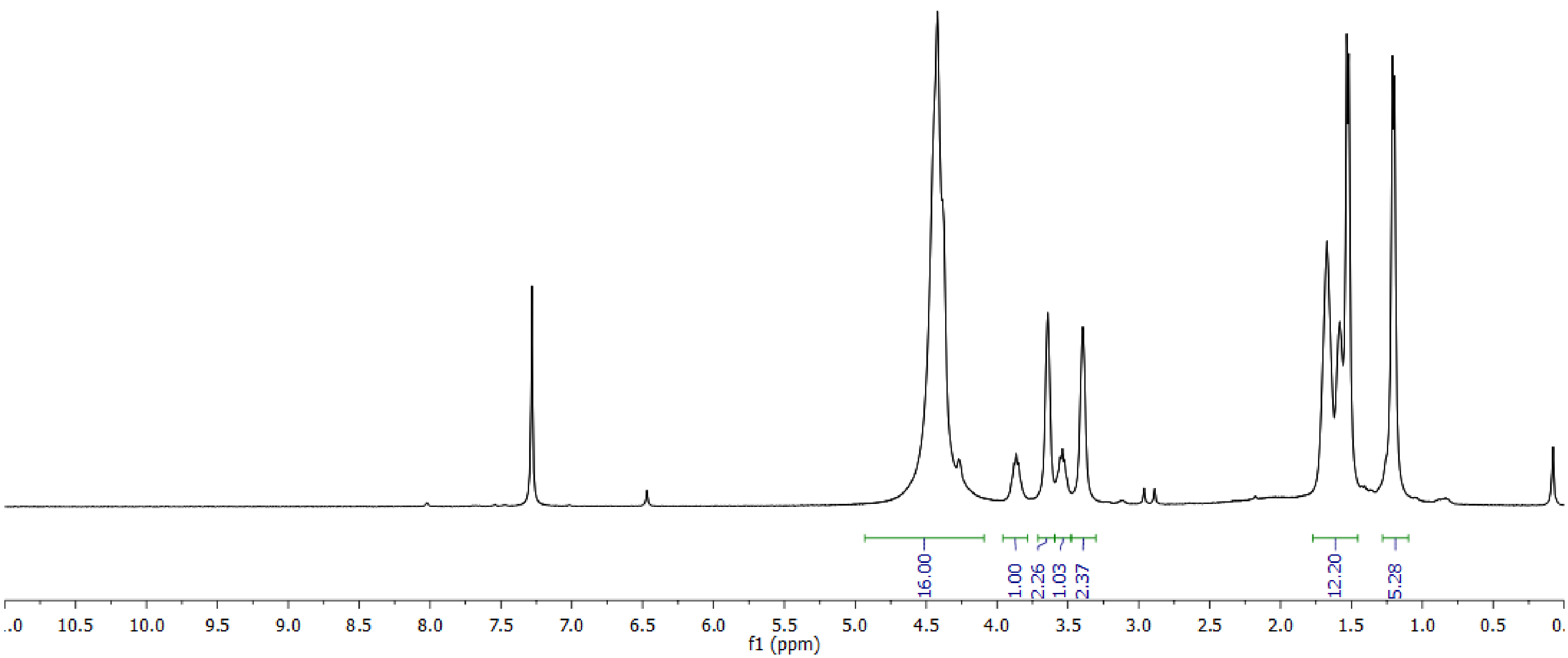

^{13}C NMR (125 MHz, $CDCl_3$)

Figure S58. ^{13}C NMR of **32**

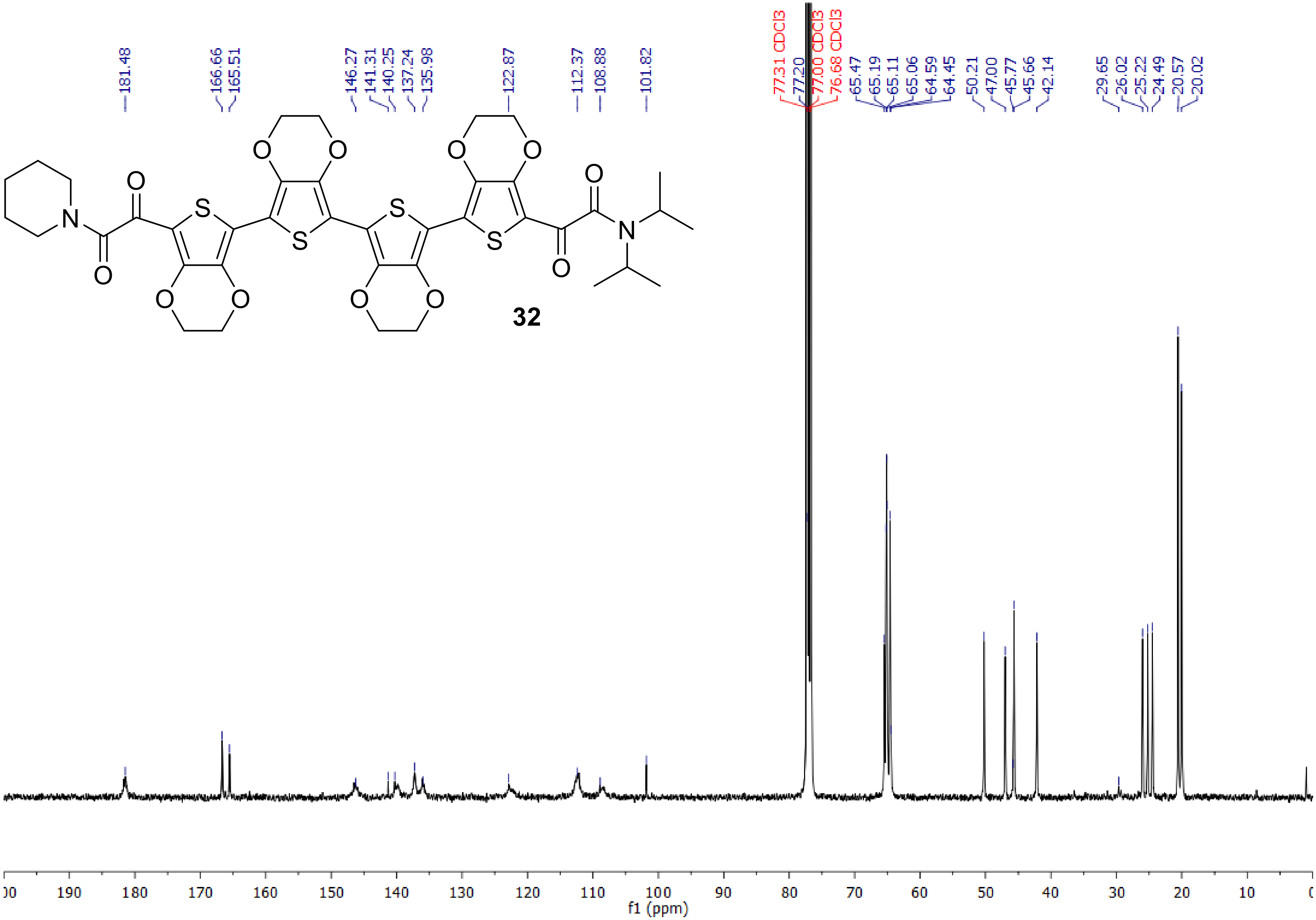

^{1}H NMR (400 MHz, $CDCl_3$)

Figure S59. ^{1}H NMR of **33**

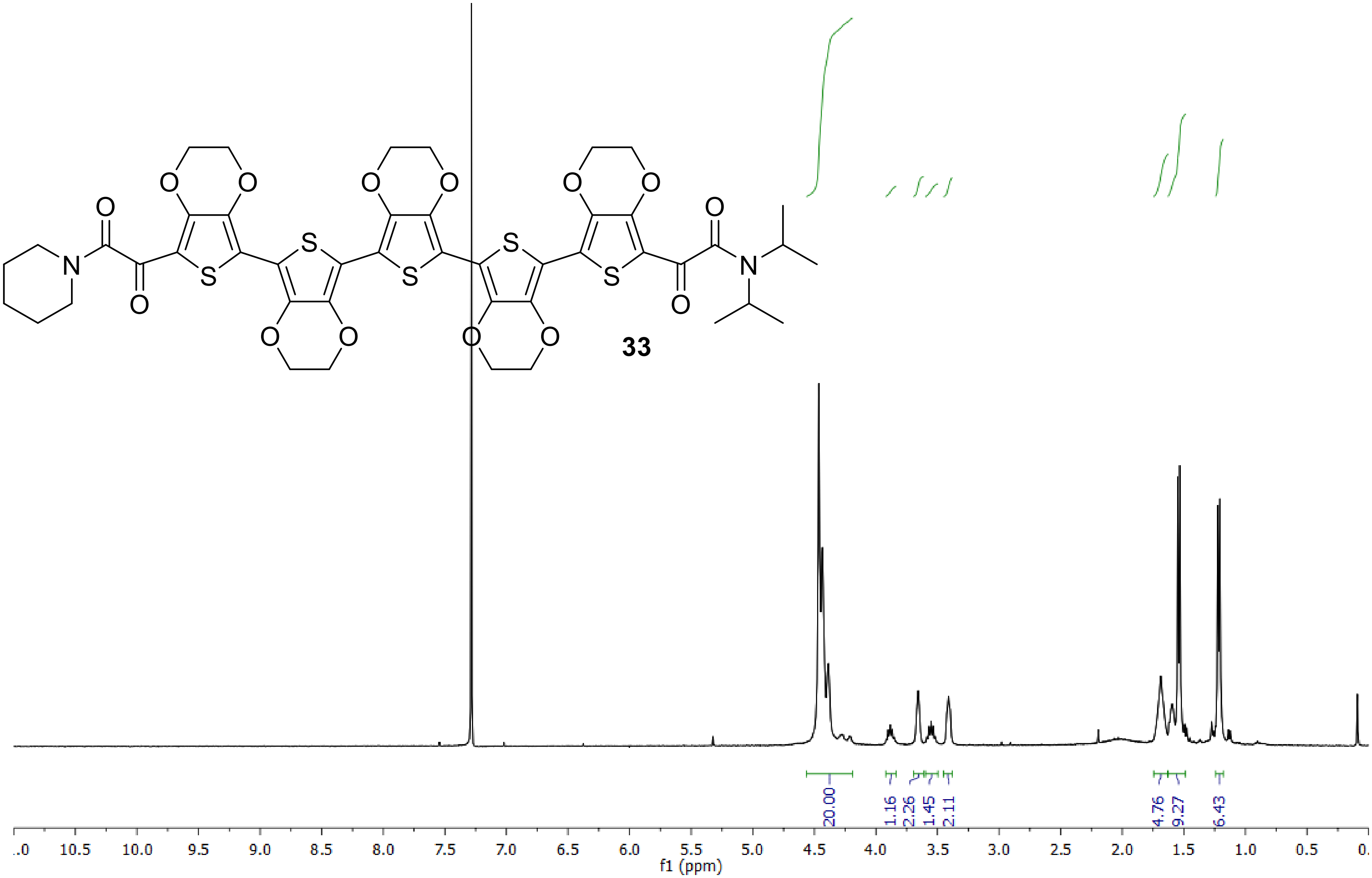

^{1}H NMR (400 MHz, DMSO)

Figure S60. ^{1}H NMR of **34**

34

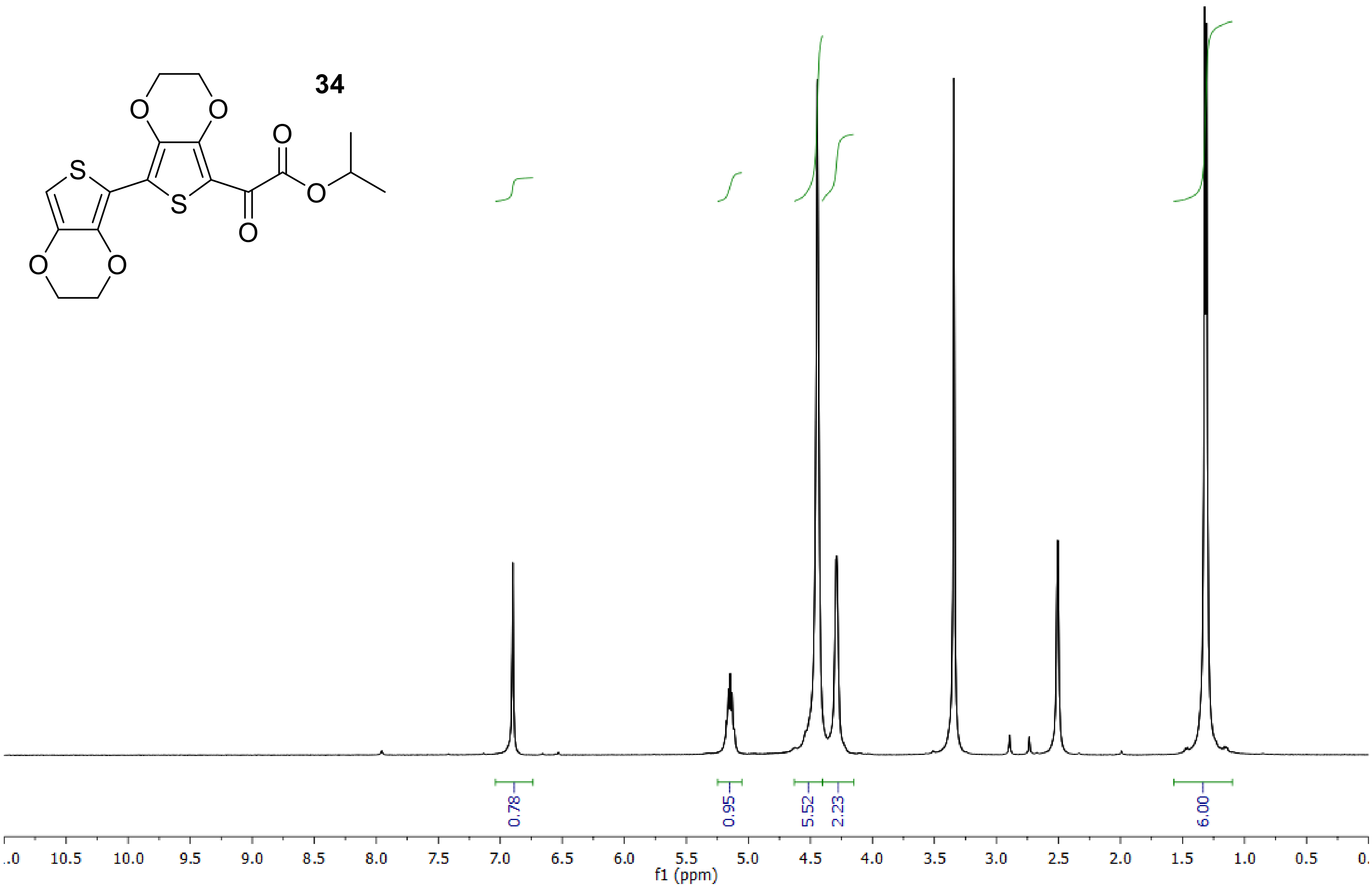

^{13}C NMR (100 MHz, DMSO)

Figure S61. ^{13}C NMR of **34**

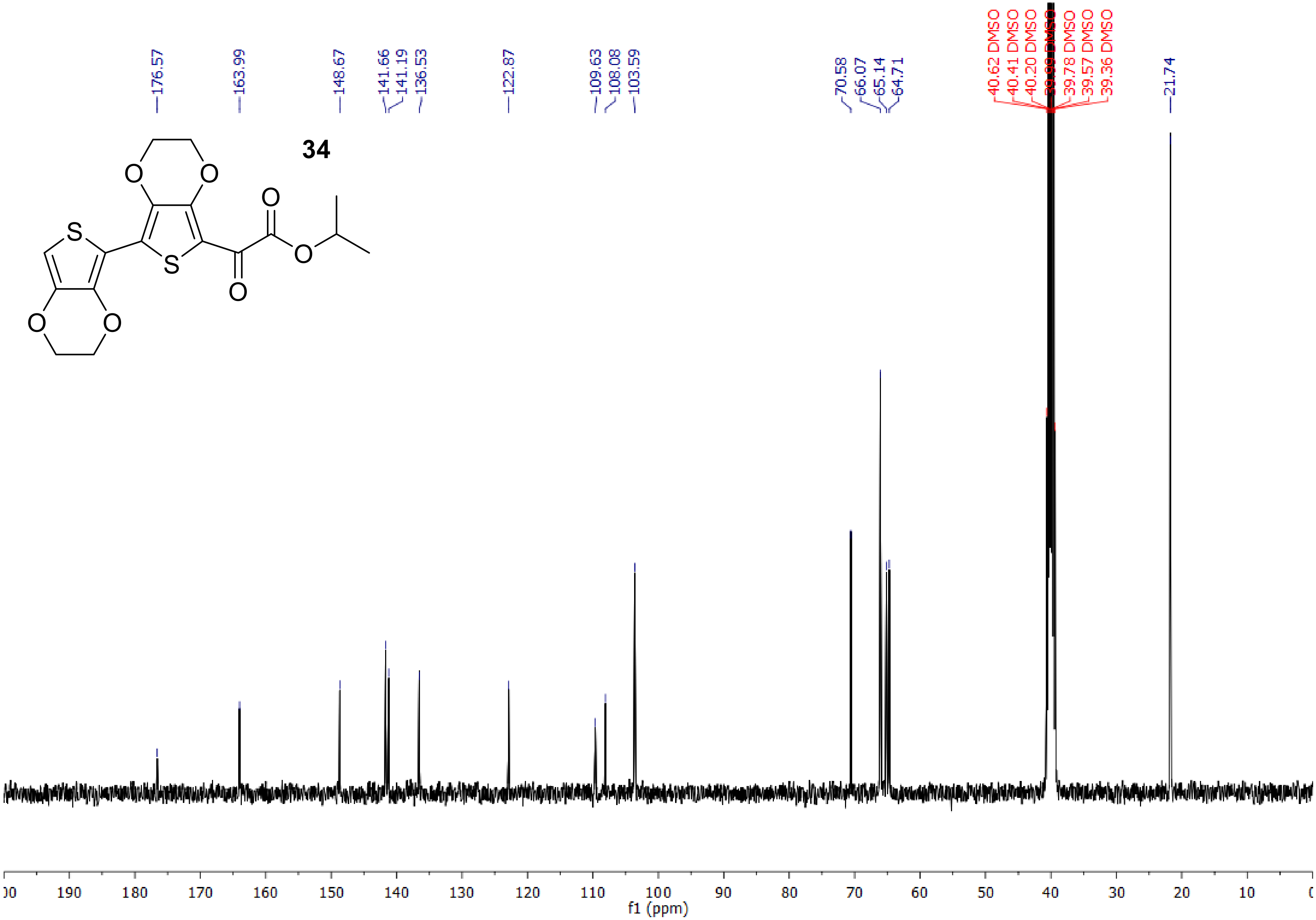

^1H NMR (400 MHz, $CDCl_3$)

Figure S62. ^1H NMR of **35**

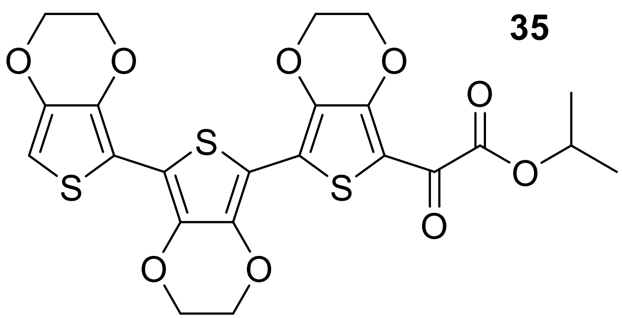

35

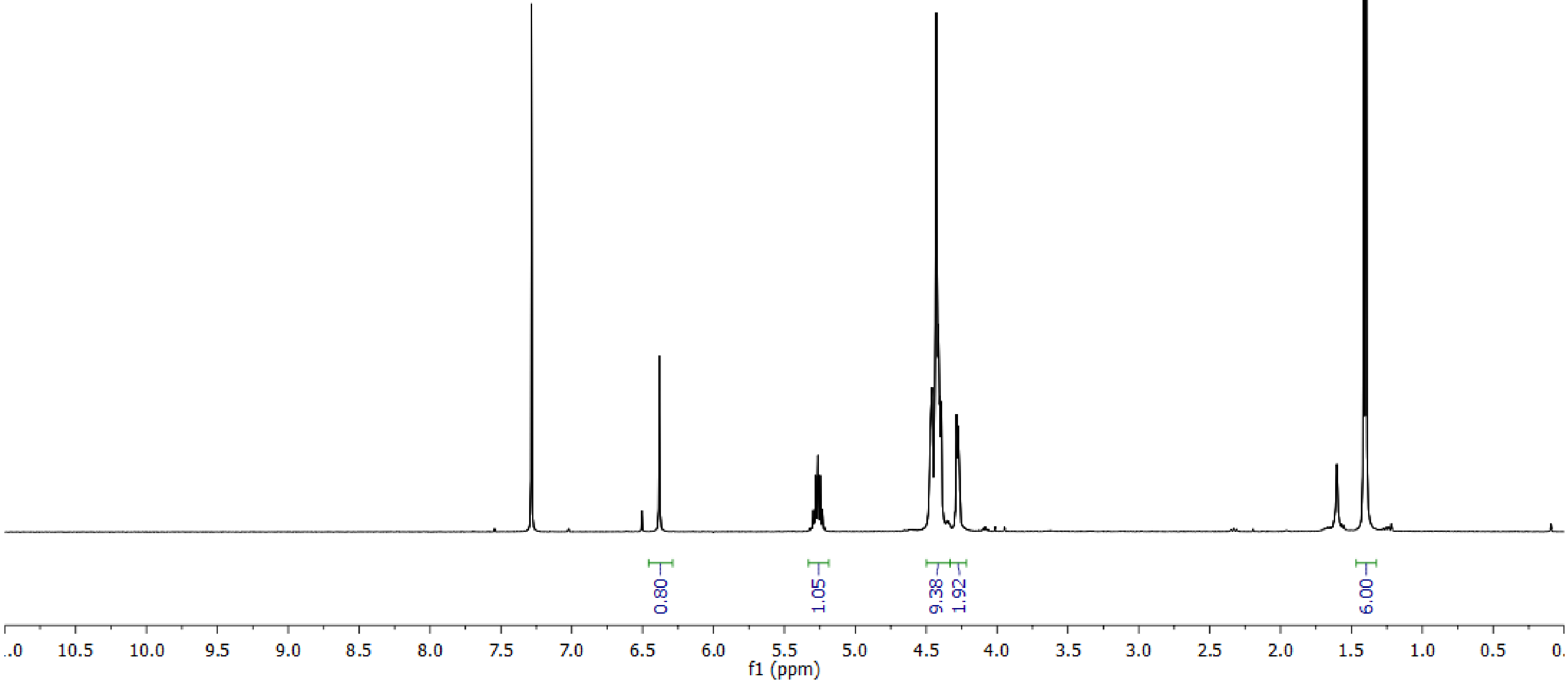

^{13}C NMR (100 MHz, $CDCl_3$)

Figure S63. ^{13}C NMR of **35**

35

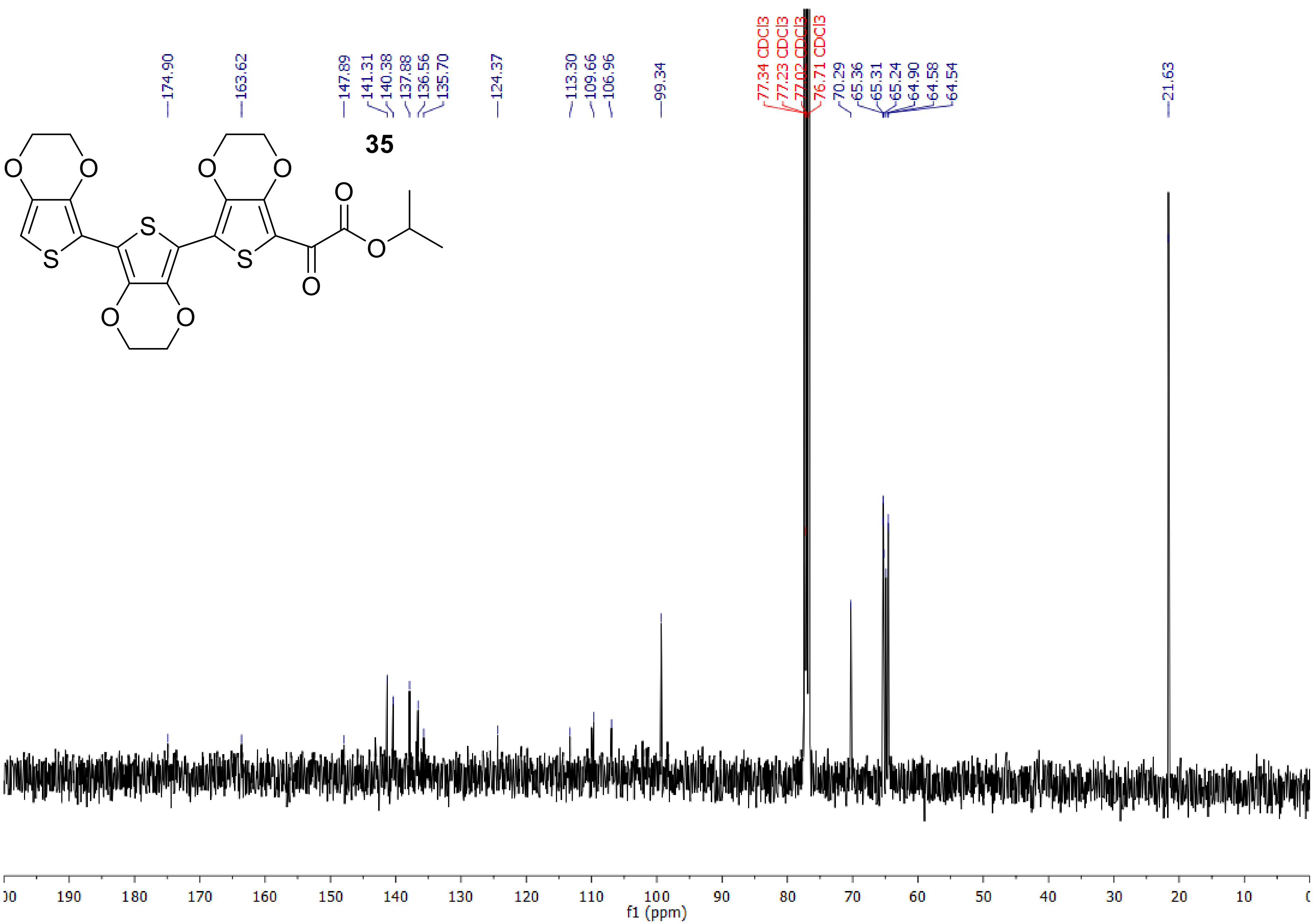

^{1}H NMR (400 MHz, $CDCl_3$)

Figure S64. ^{1}H NMR of **36**

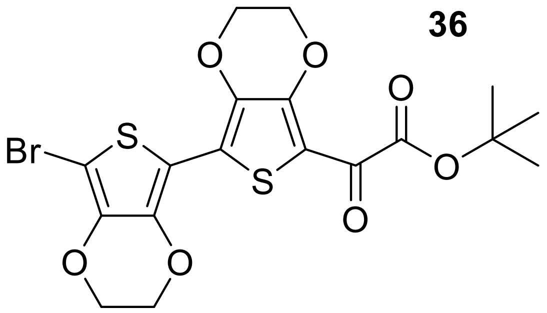

36

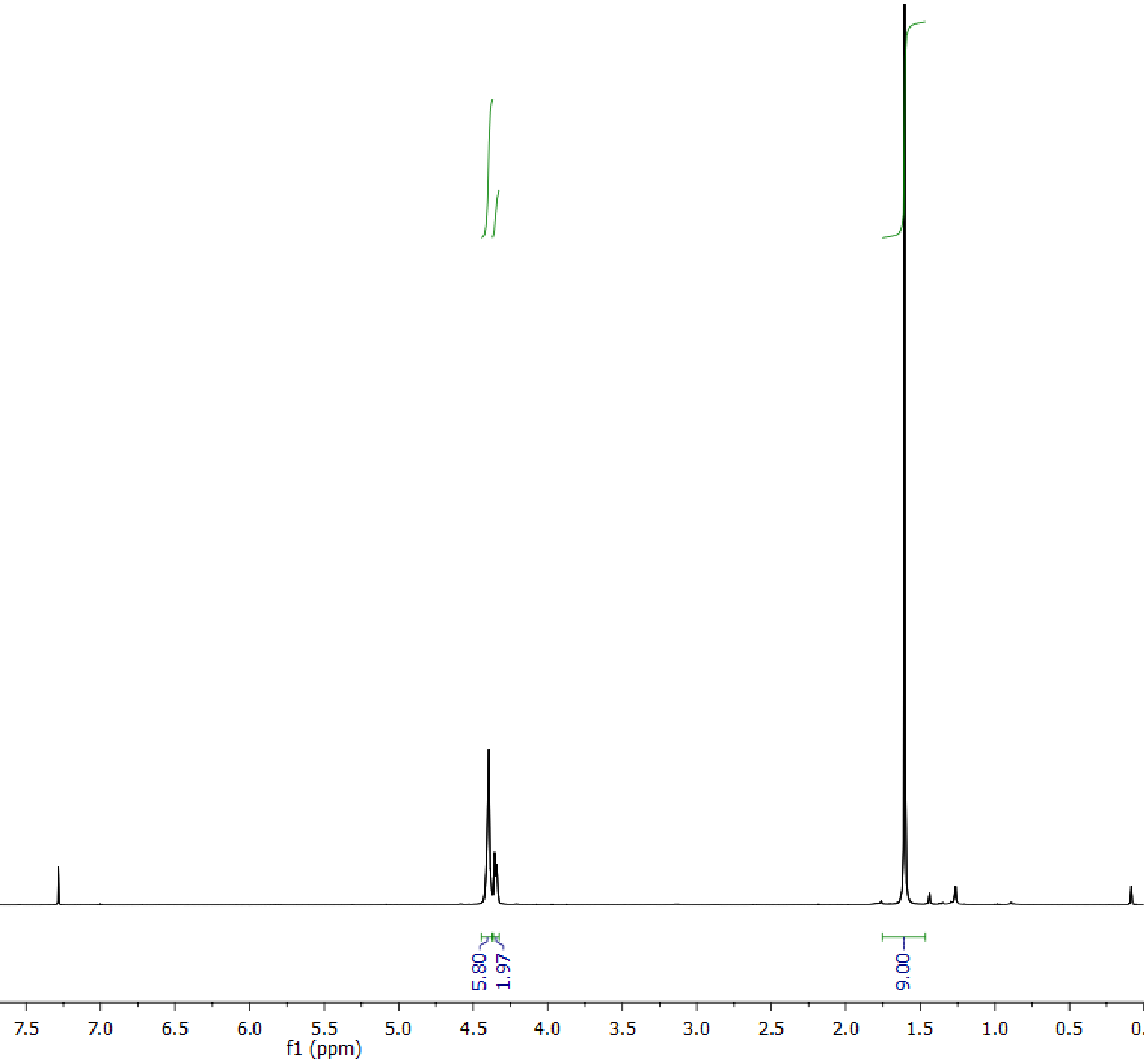

^{13}C NMR (100 MHz, $CDCl_3$)

Figure S65. ^{13}C NMR of **36**

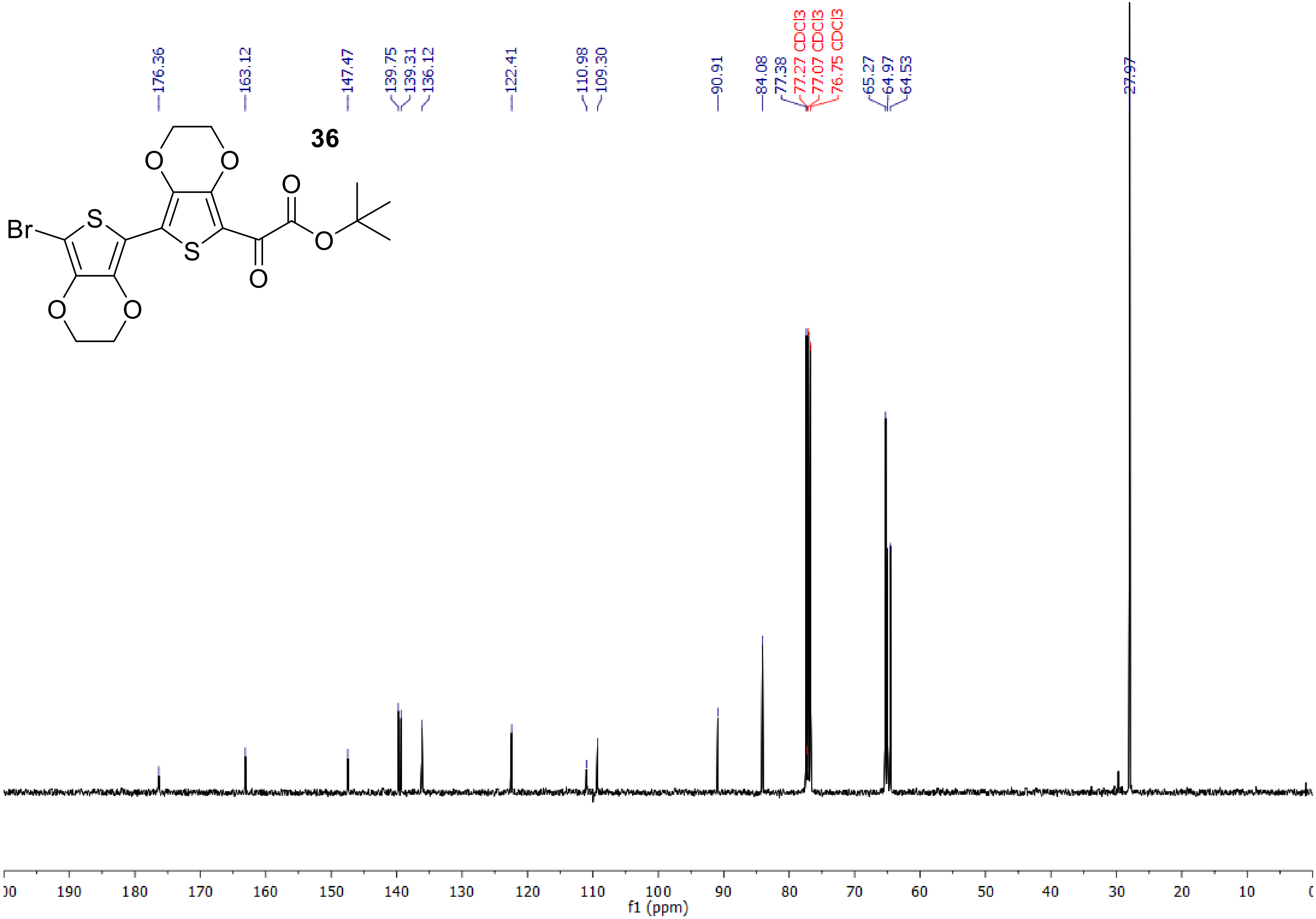

36

^{1}H NMR (400 MHz, $CDCl_3$)

Figure S66. ^{1}H NMR of **37**

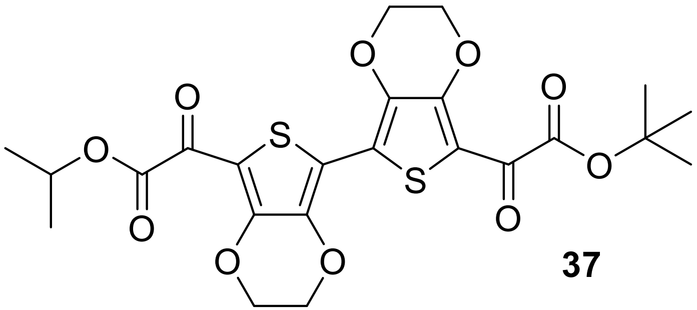

37

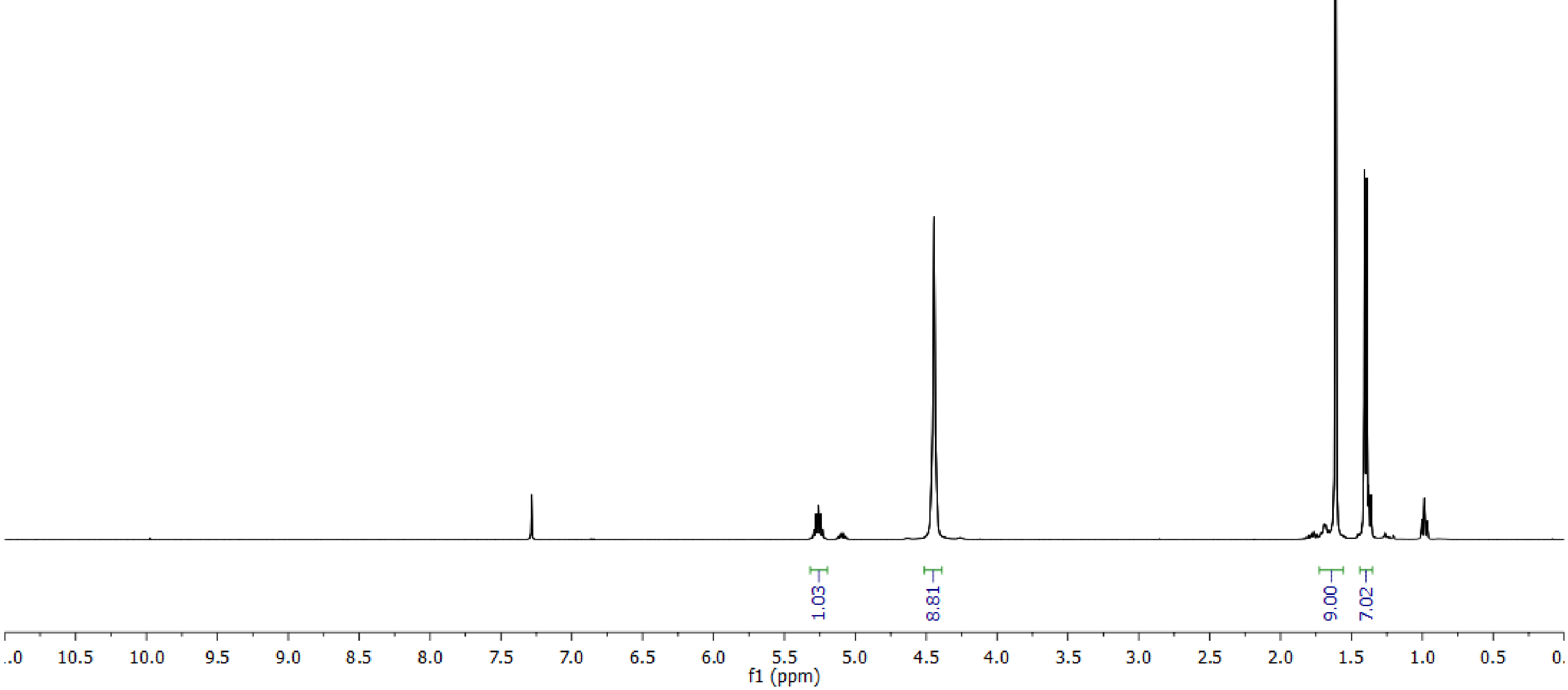

^{13}C NMR (100 MHz, $CDCl_3$)

Figure S67. ^{13}C NMR of **37**

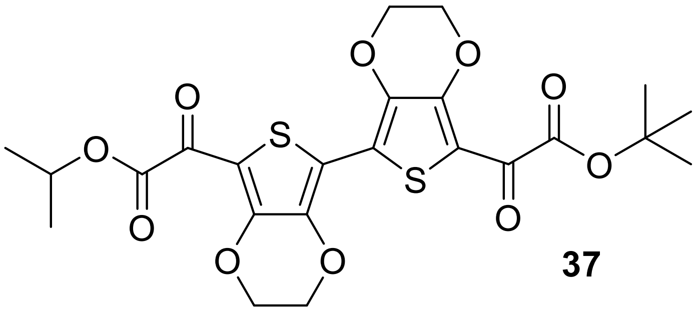

37

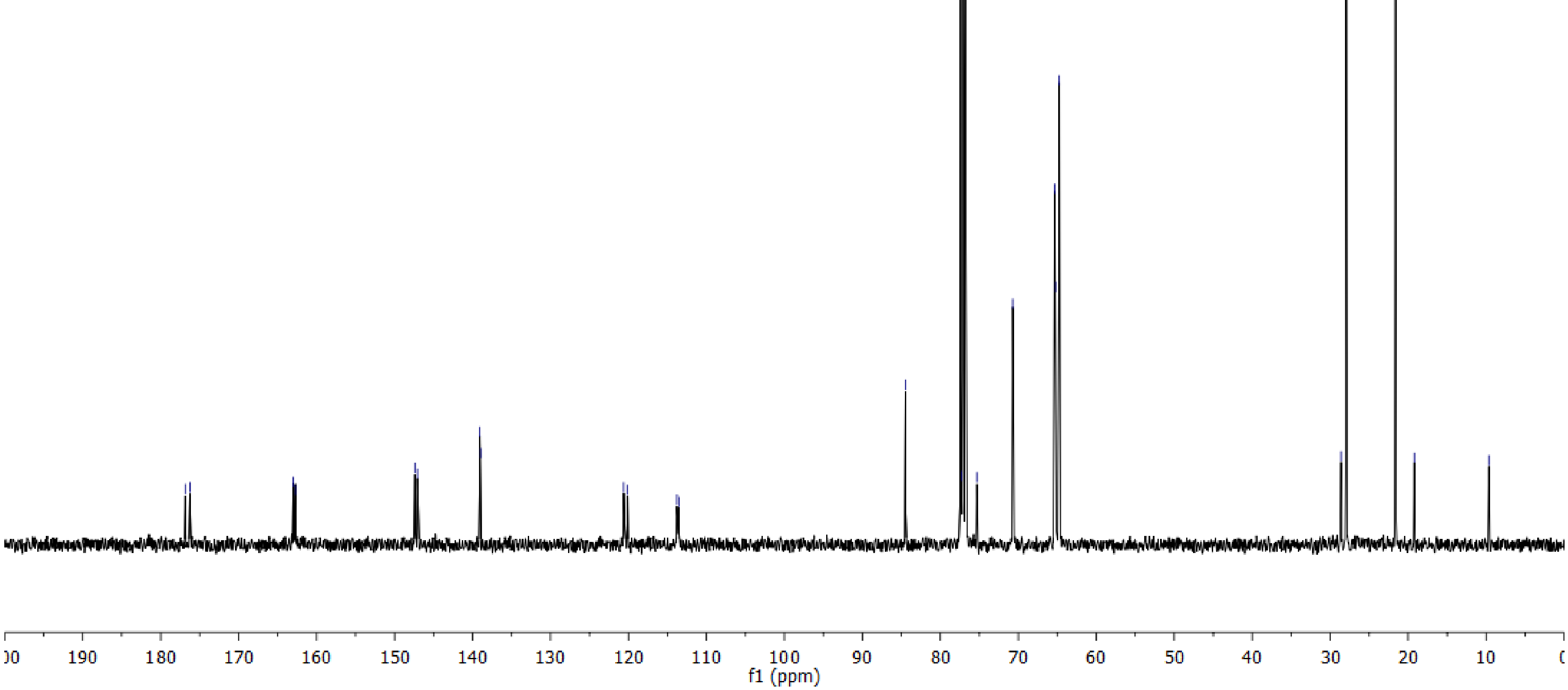

^{1}H NMR (400 MHz, $CDCl_3$)

Figure S68. ^{1}H NMR of **39**

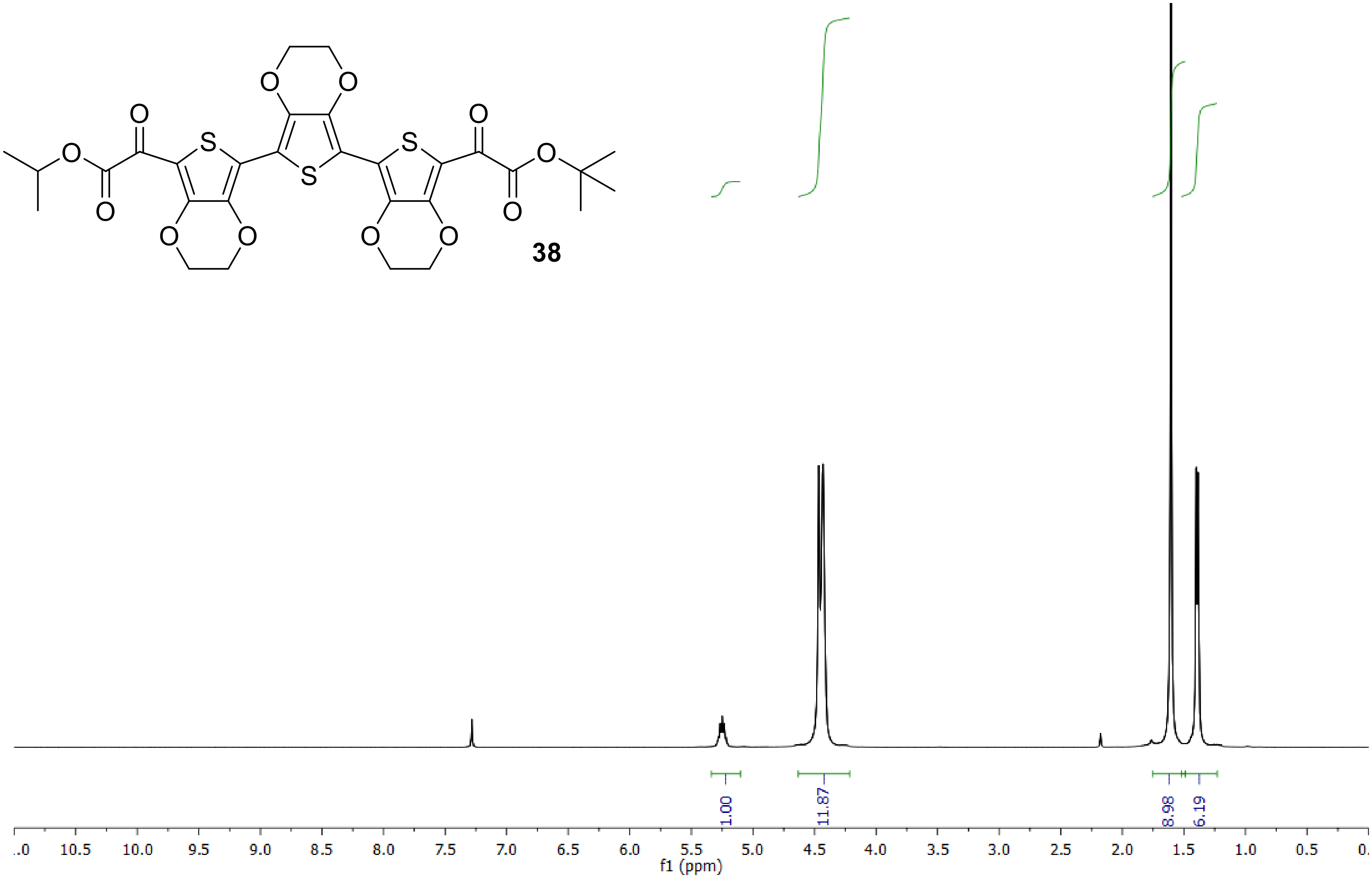

^{13}C NMR (100 MHz, $CDCl_3$)

Figure S69. ^{13}C NMR of **38**

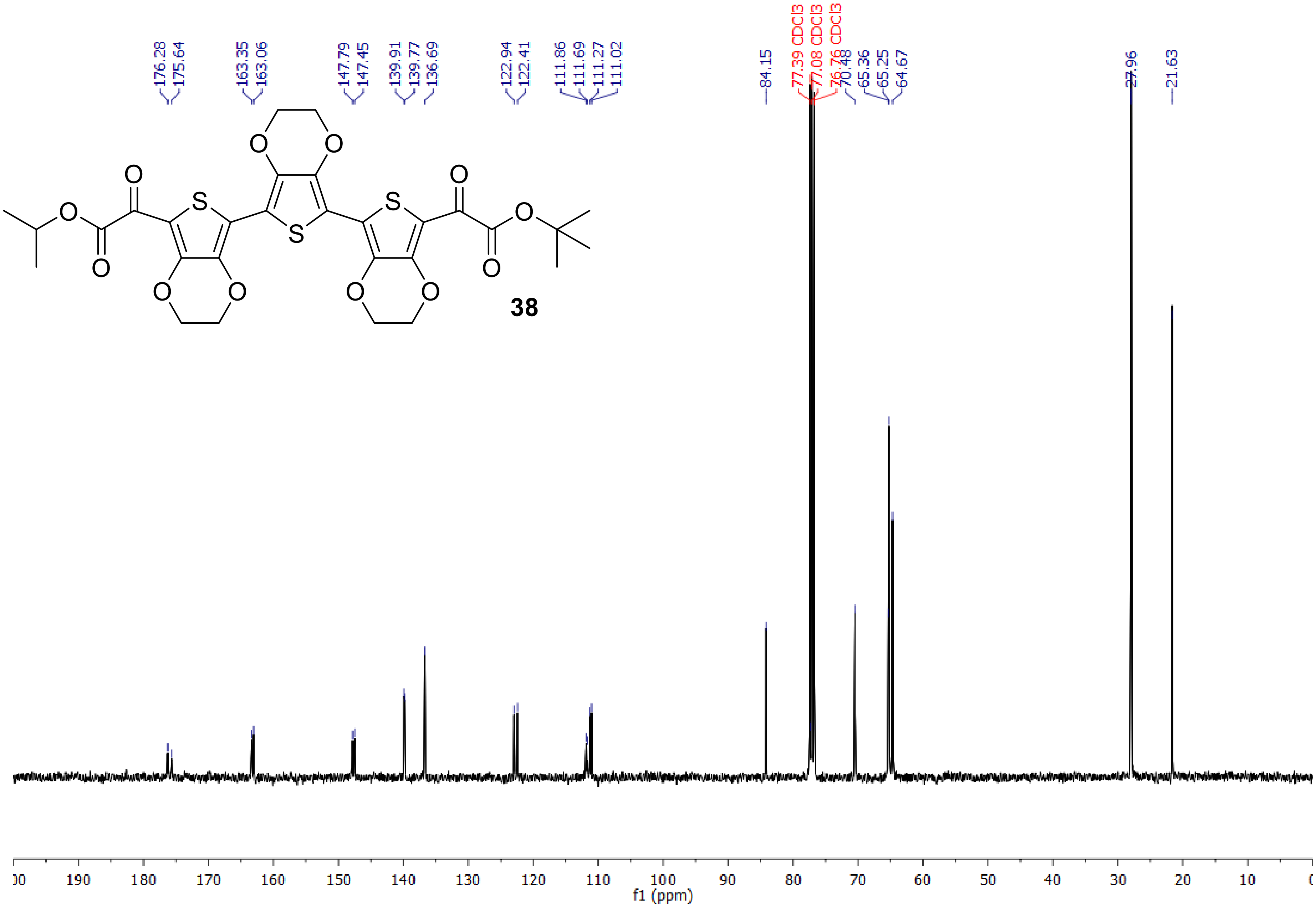

^{1}H NMR (400 MHz, $CDCl_3$)

Figure S70. ^{1}H NMR of **39**

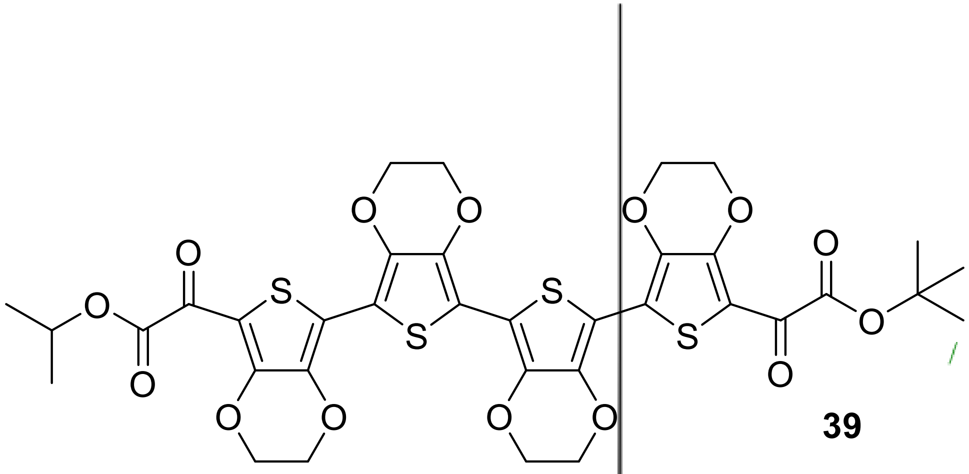

39

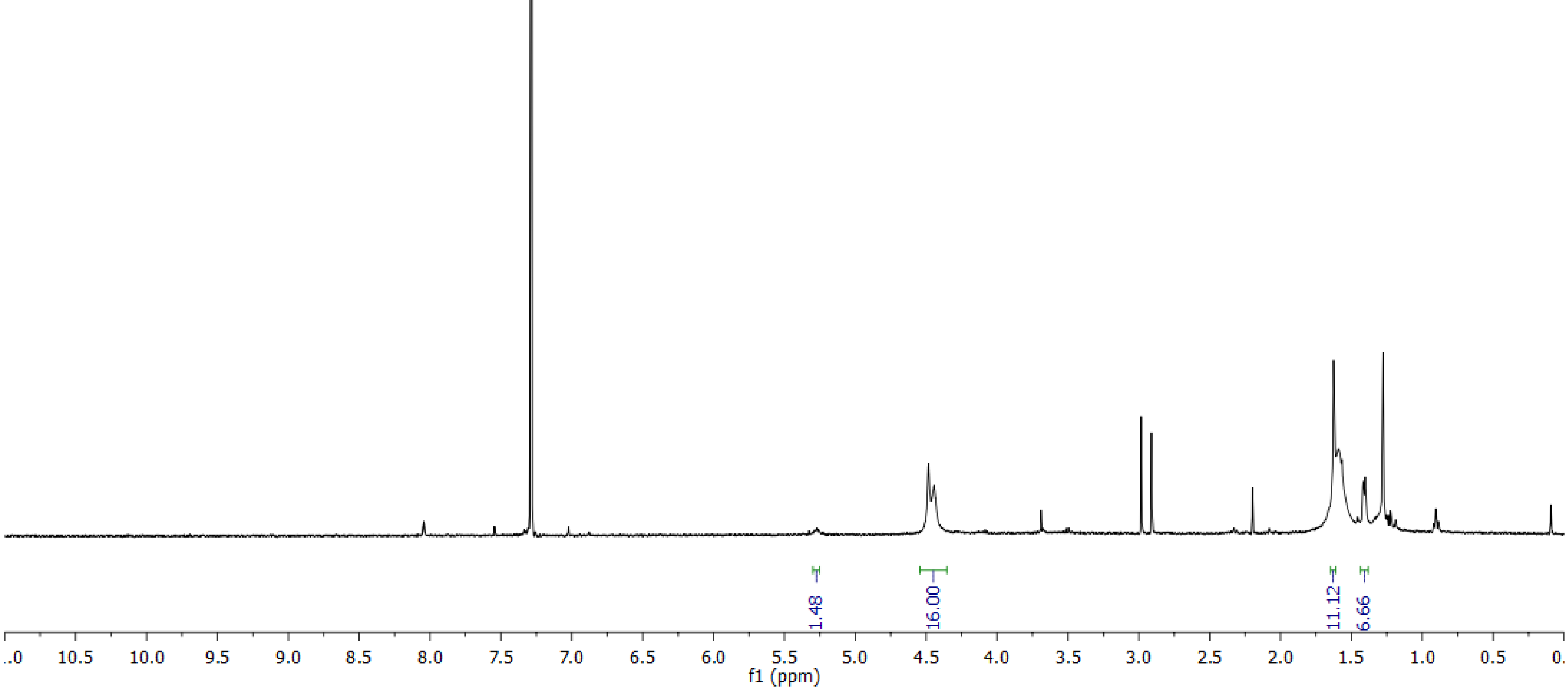

^{1}H NMR (400 MHz, $CDCl_3$)

Figure S71. ^{1}H NMR of **40**

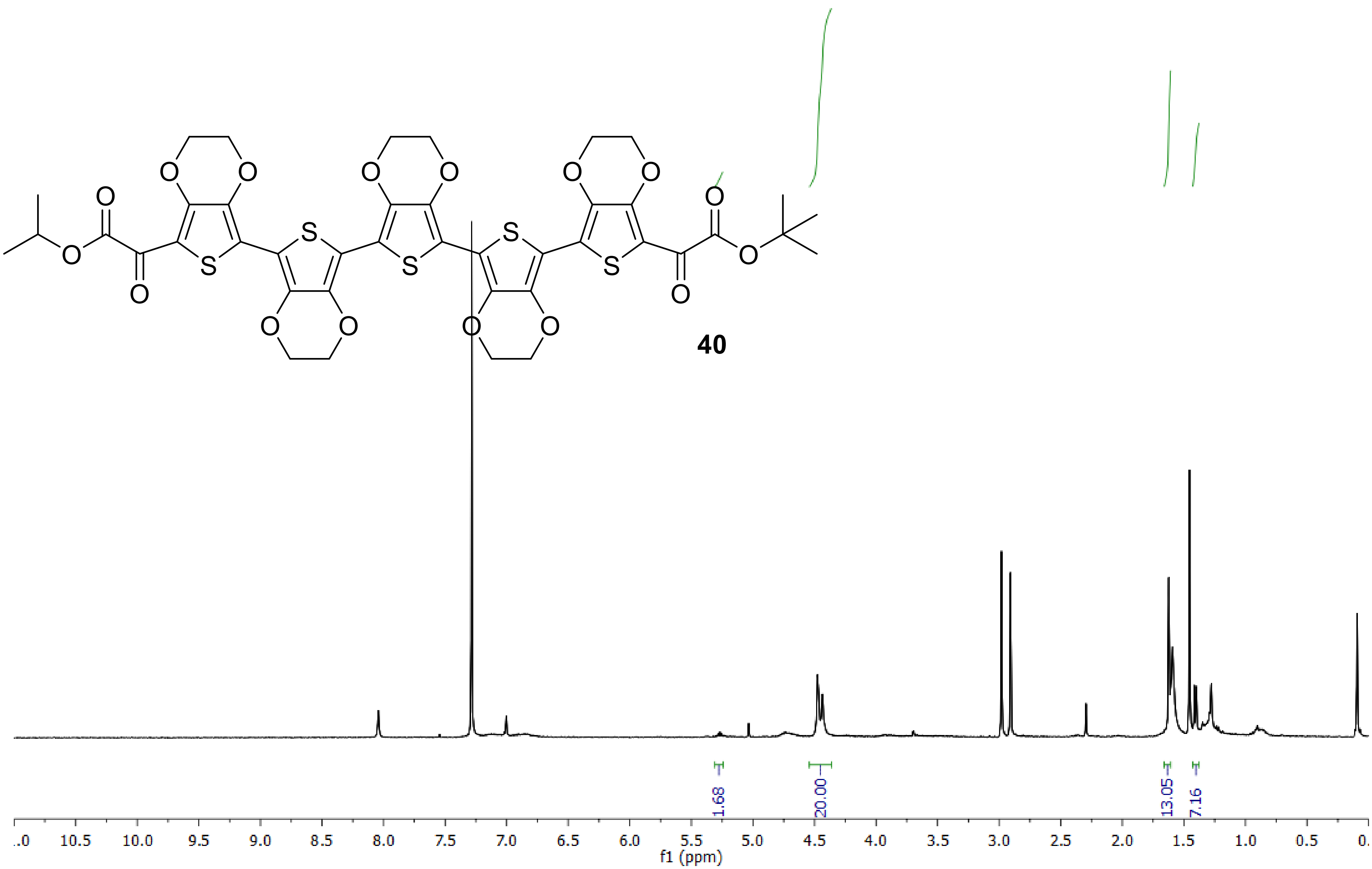

S130

^{1}H NMR (400 MHz, $CDCl_3$)

Figure S72. ^{1}H NMR of **41**

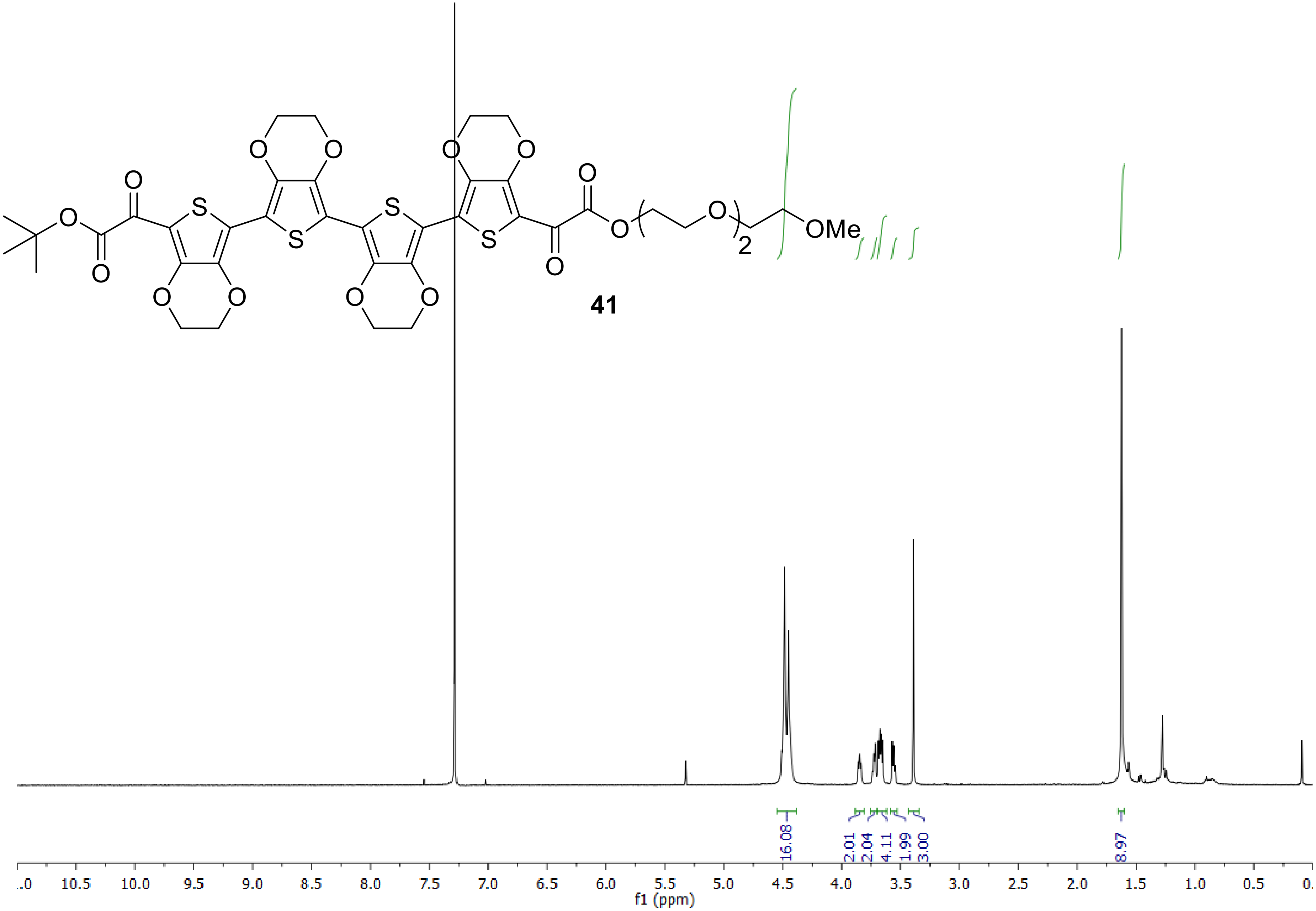

^{1}H NMR (400 MHz, $CDCl_3$)

Figure S73. ^{1}H NMR of **42**

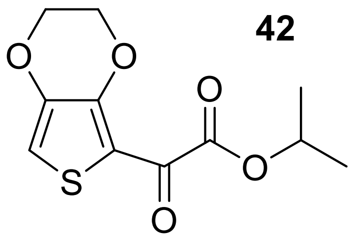

42

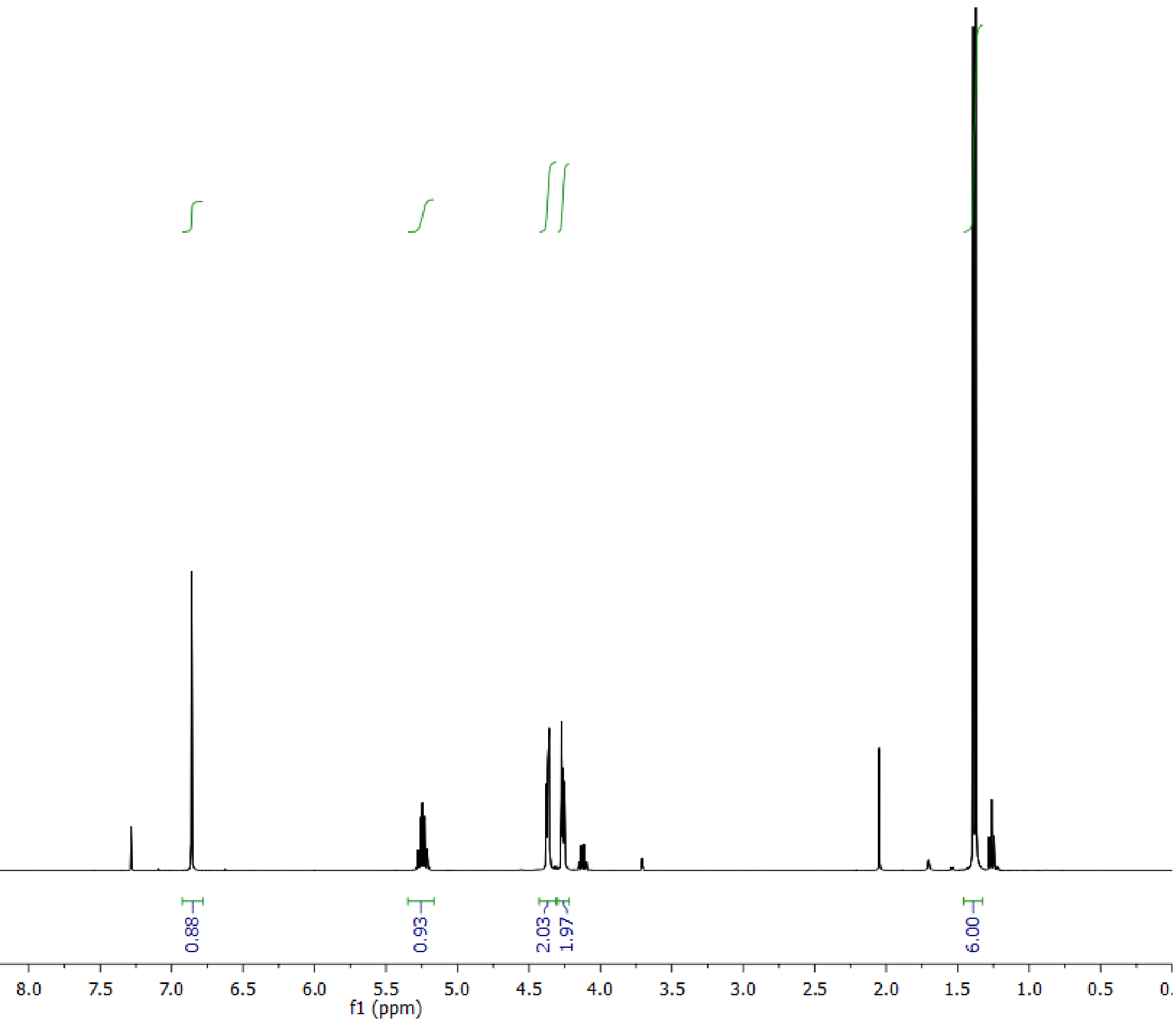

^{13}C NMR (100 MHz, $CDCl_3$)

Figure S74. ^{13}C NMR of **42**

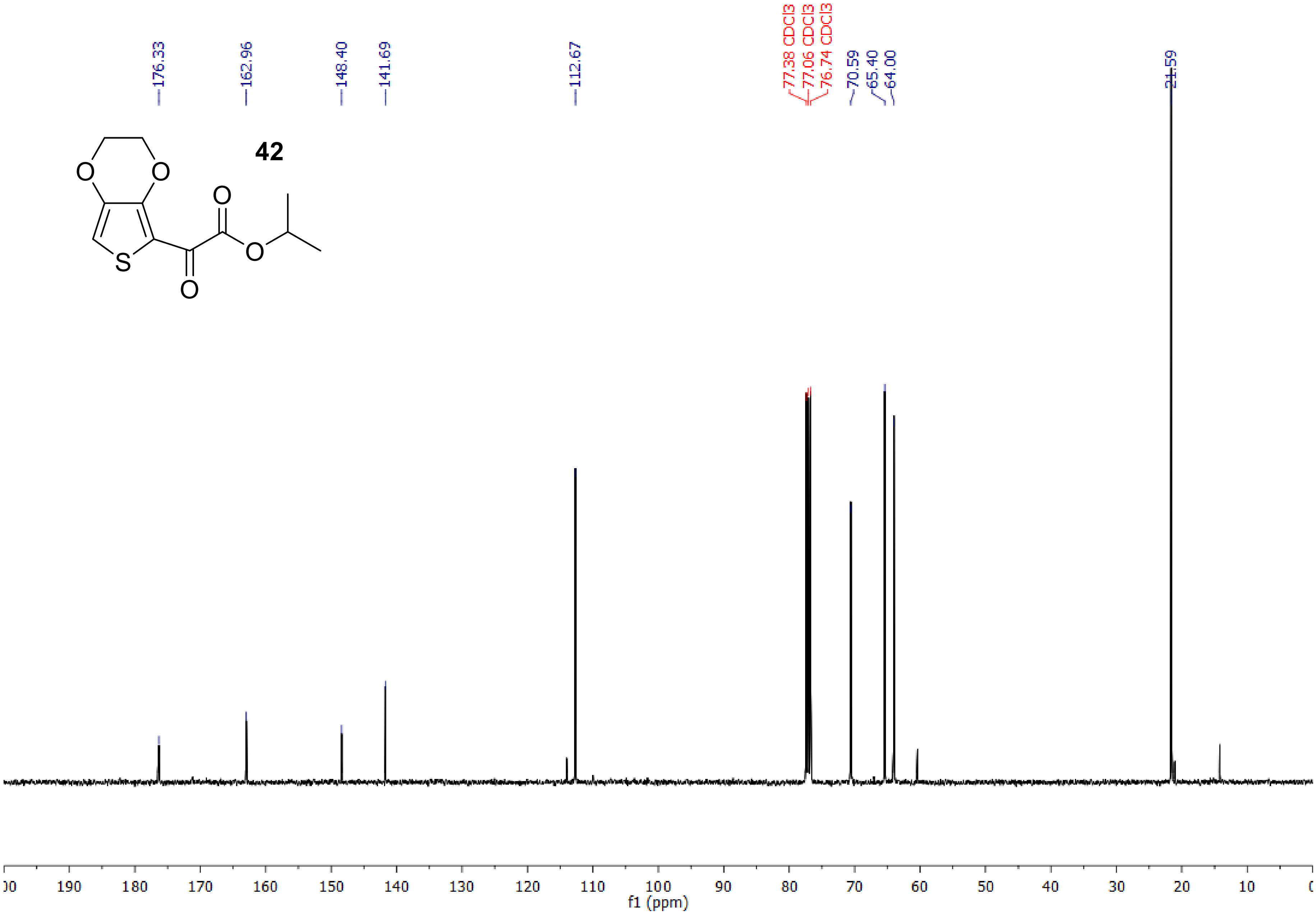

^{1}H NMR (400 MHz, $CDCl_3$)

Figure S75. ^{1}H NMR of **45**

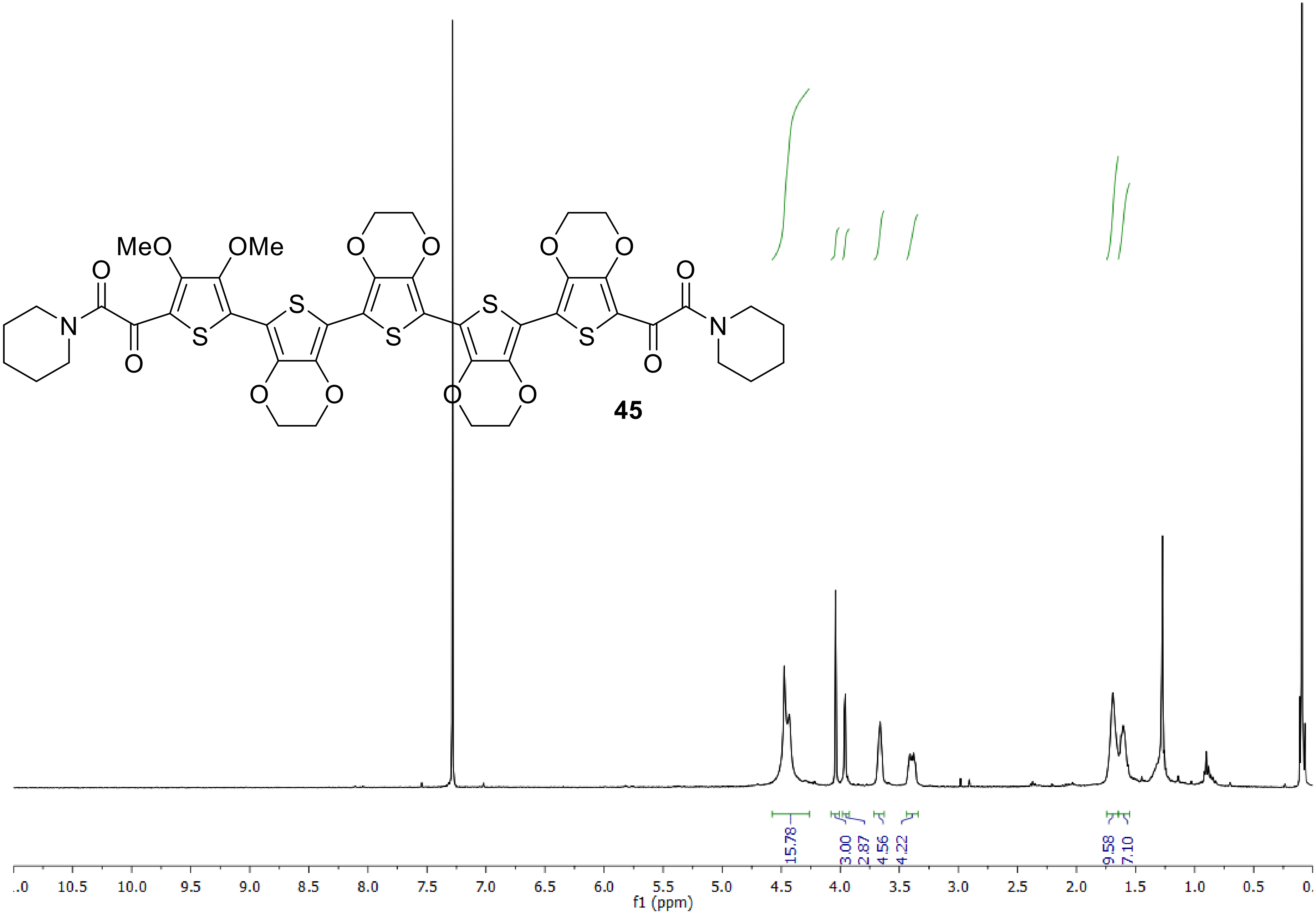

^{1}H NMR (400 MHz, $CDCl_3$)

Figure S76. ^{1}H NMR of **46**

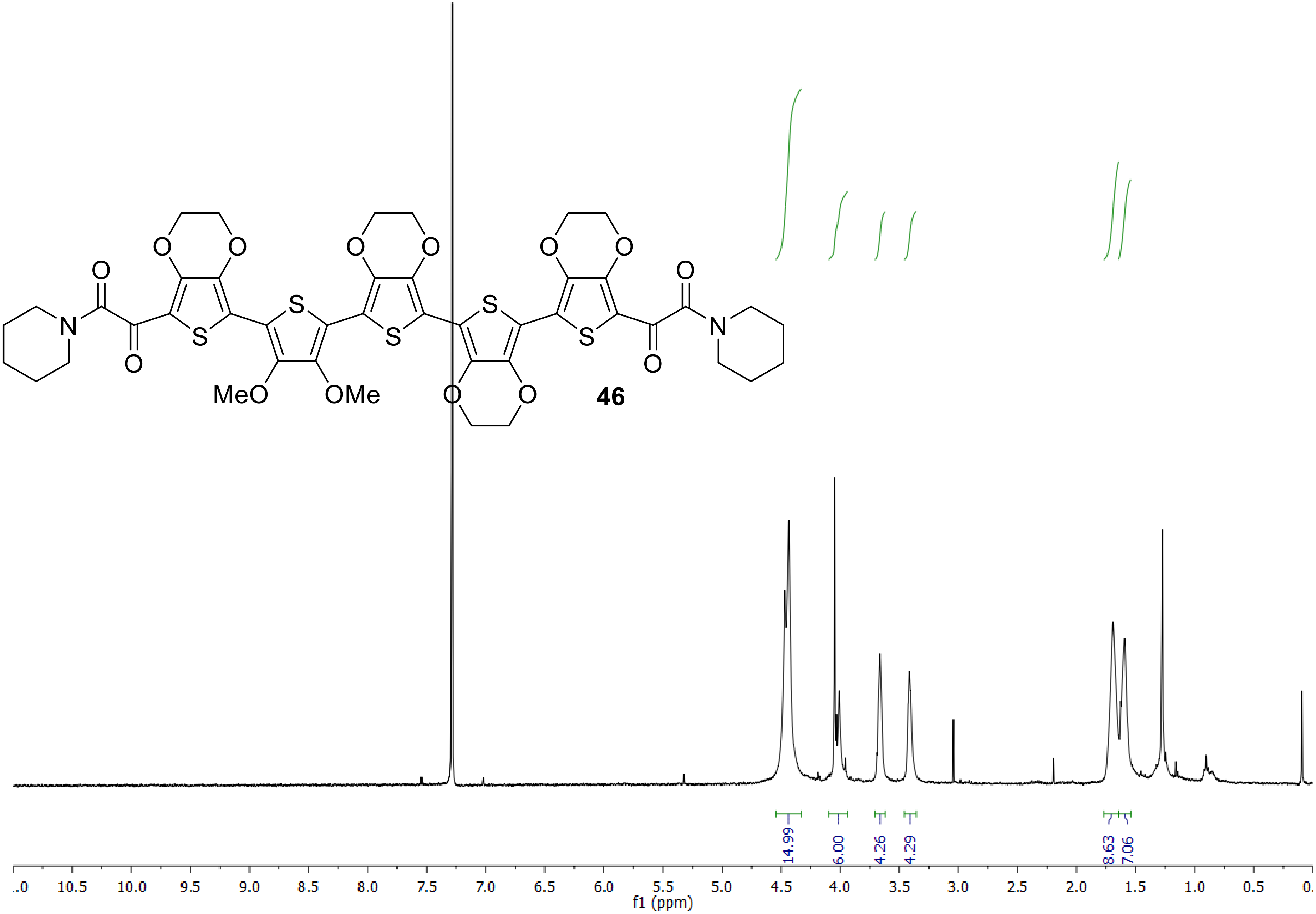

^{1}H NMR (400 MHz, $CDCl_3$)

Figure S77. ^{1}H NMR of **47**

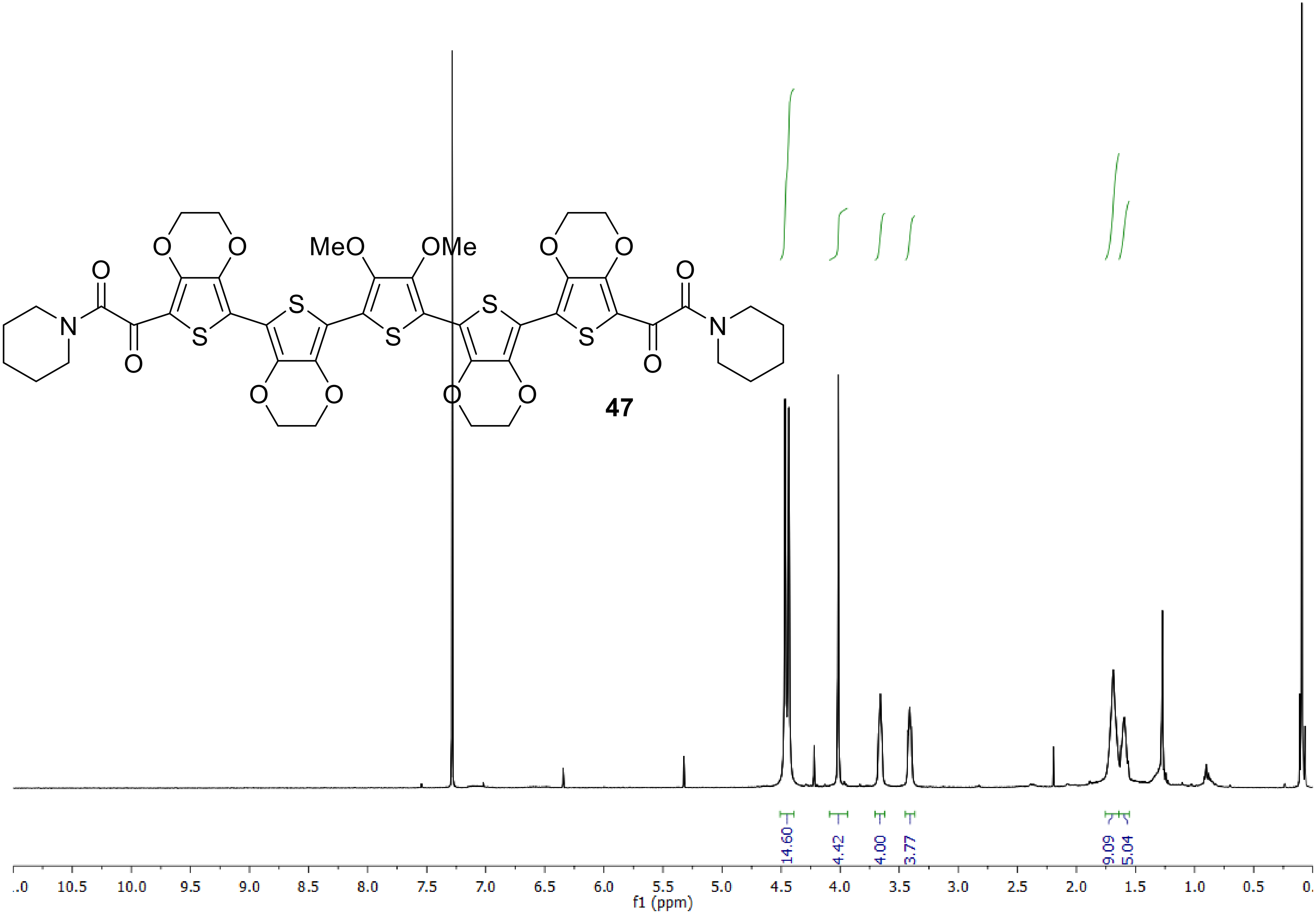

^{1}H NMR (400 MHz, $CDCl_3$)

Figure S78. ^{1}H NMR of **48**

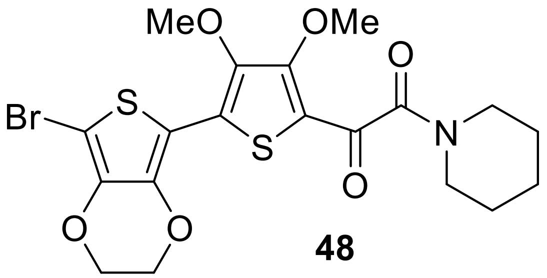

48

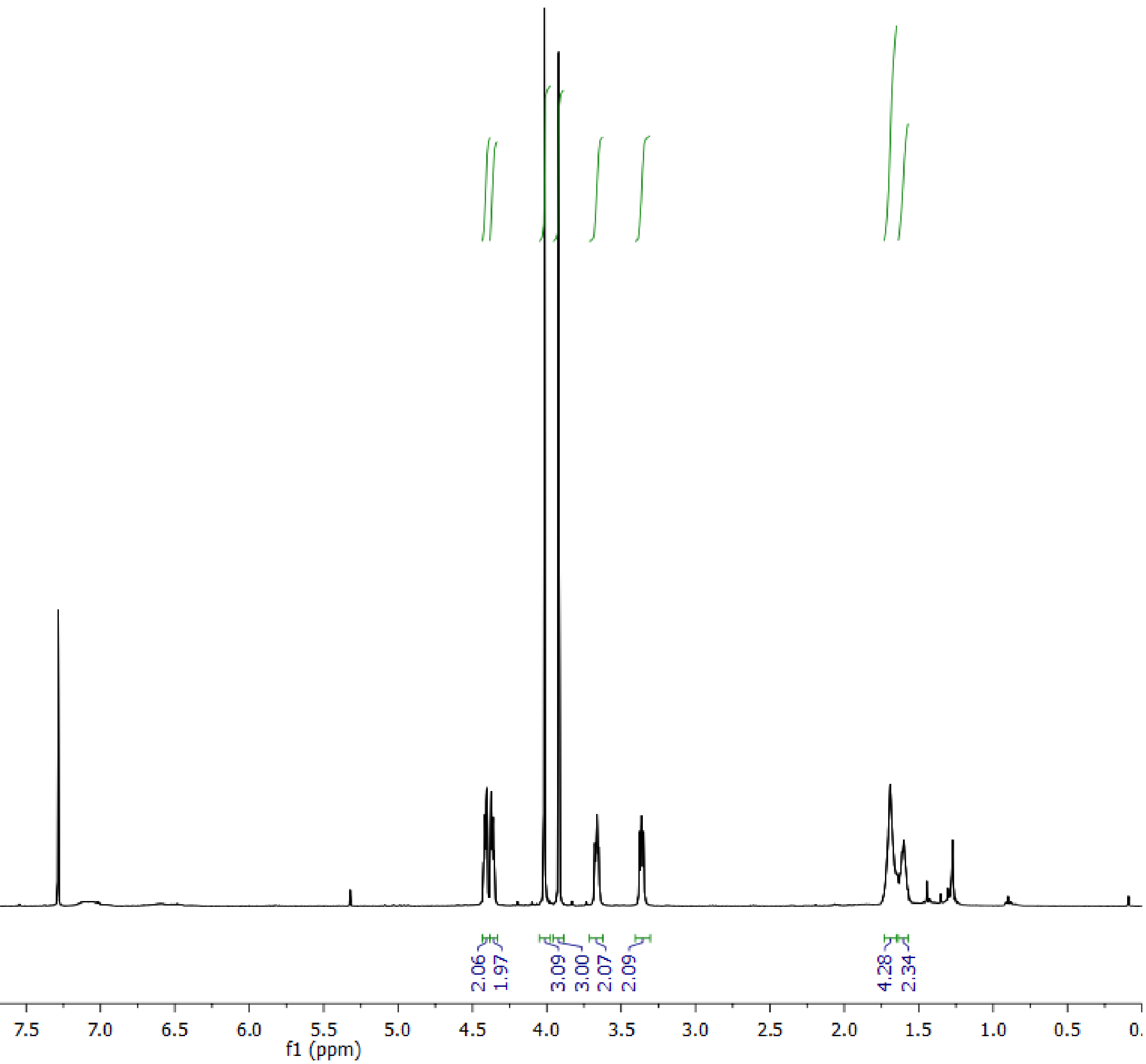

13C NMR (100 MHz, $CDCl_3$)

Figure S79. ^{13}C NMR of **48**

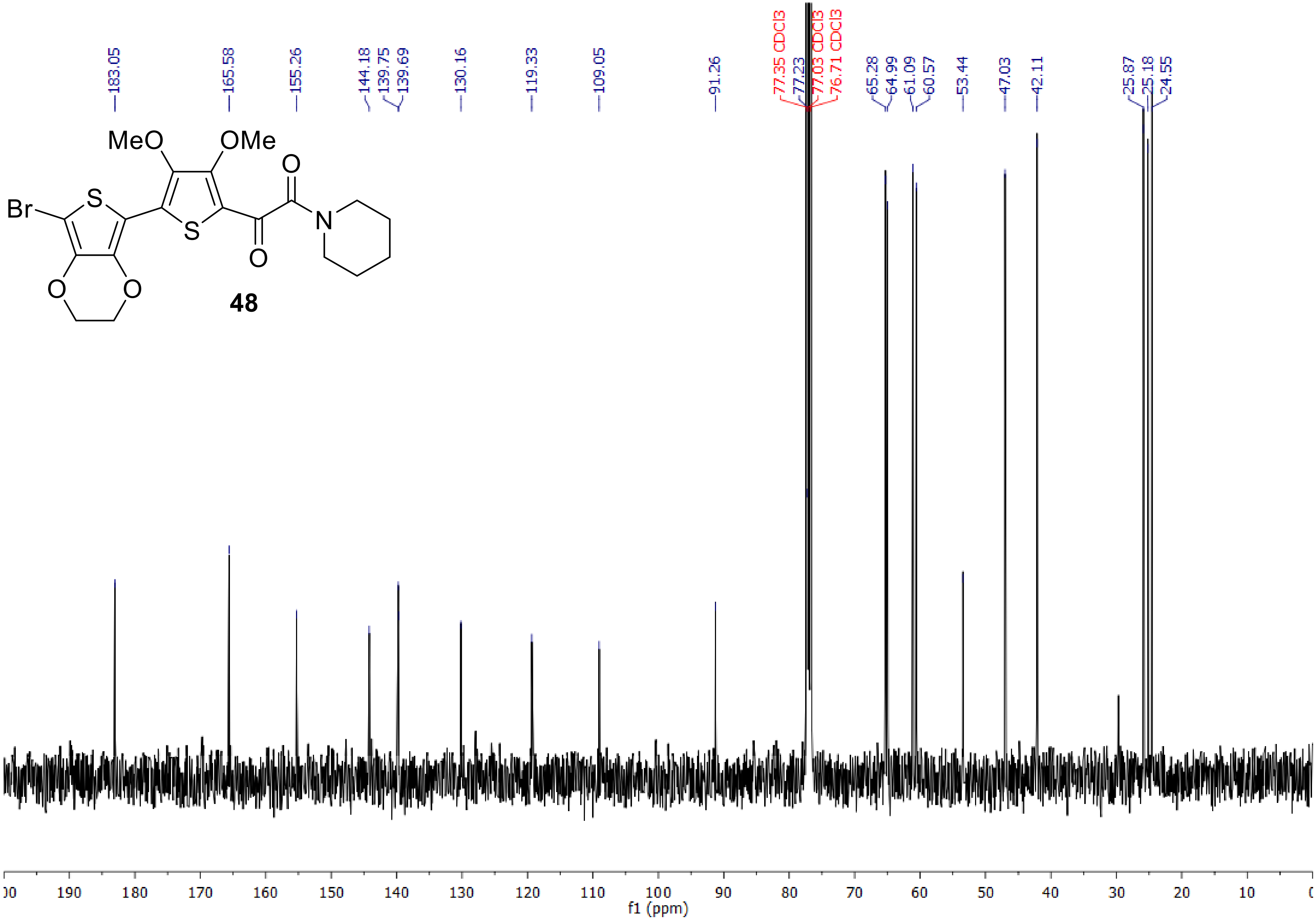

^{1}H NMR (400 MHz, $CDCl_3$)

Figure S80. ^{1}H NMR of **49**

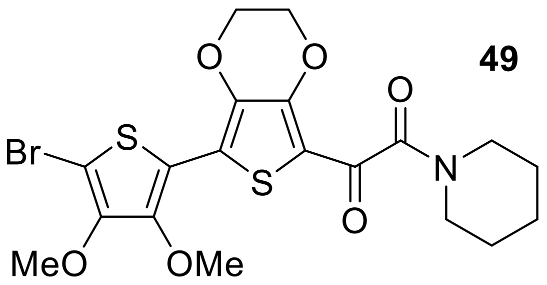

49

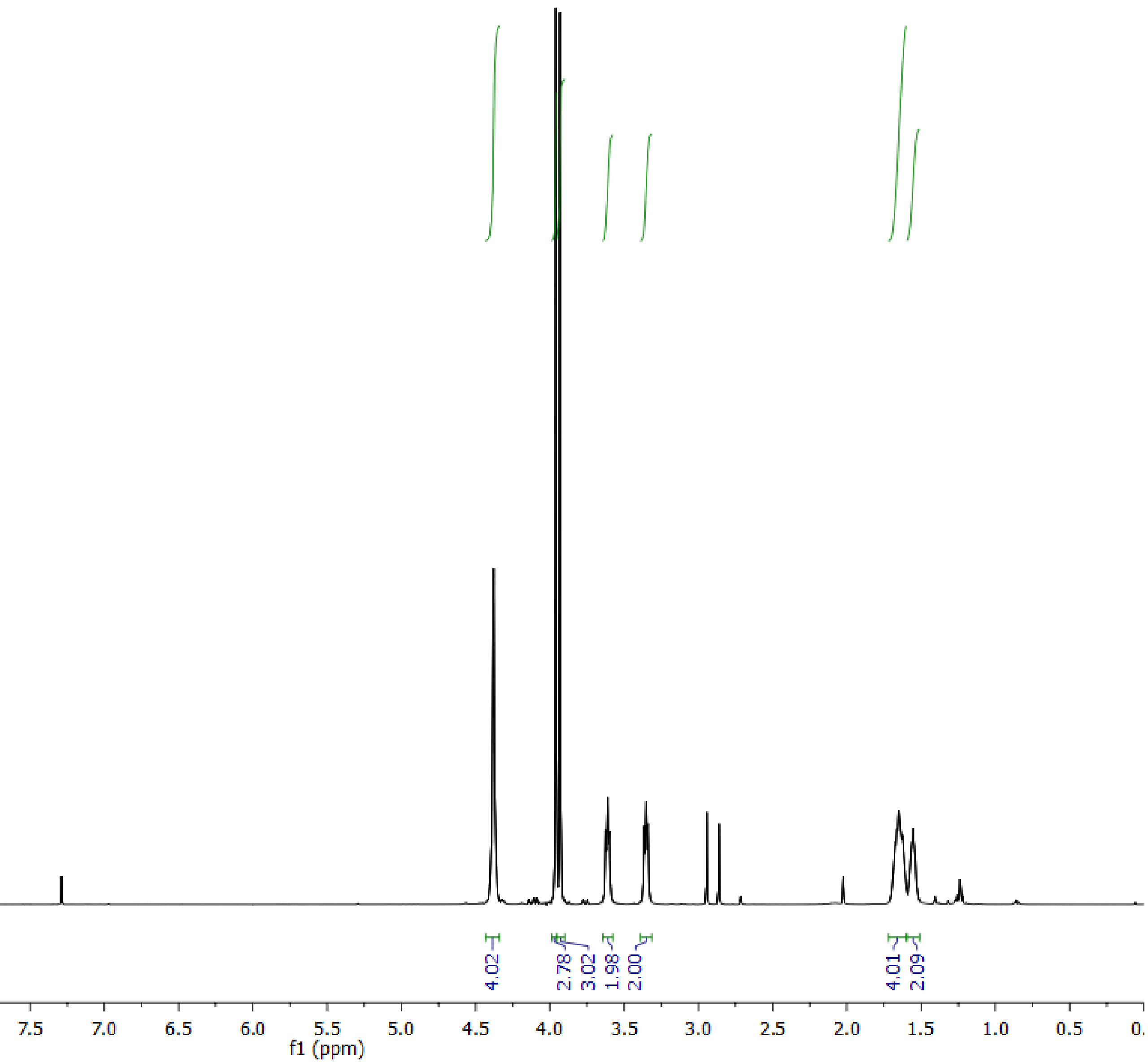

^{13}C NMR (100 MHz, $CDCl_3$)

Figure S81. ^{13}C NMR of **49**

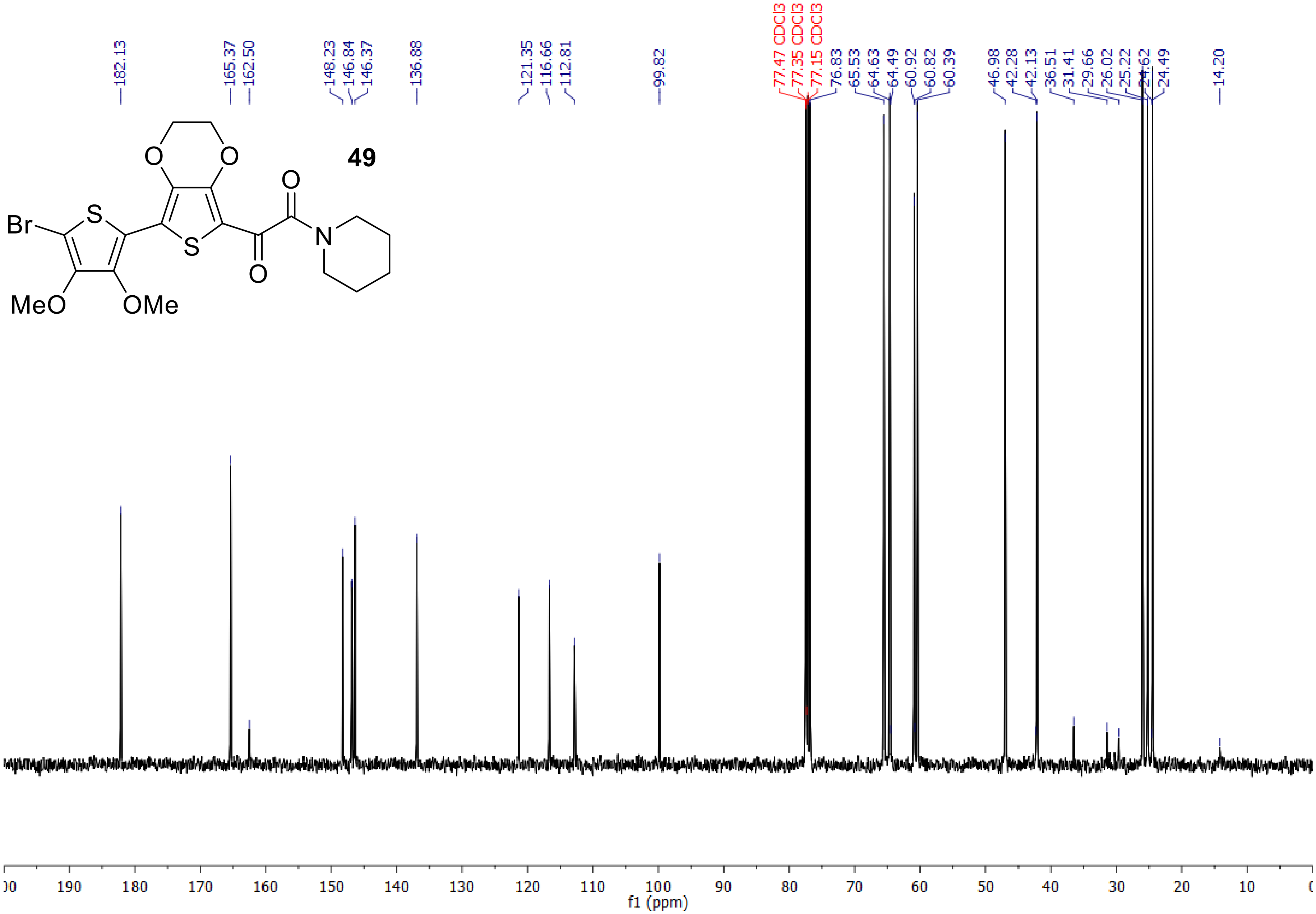

^1H NMR (400 MHz, $CDCl_3$)

Figure S82. ^1H NMR of **50**

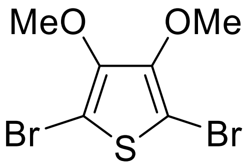

50

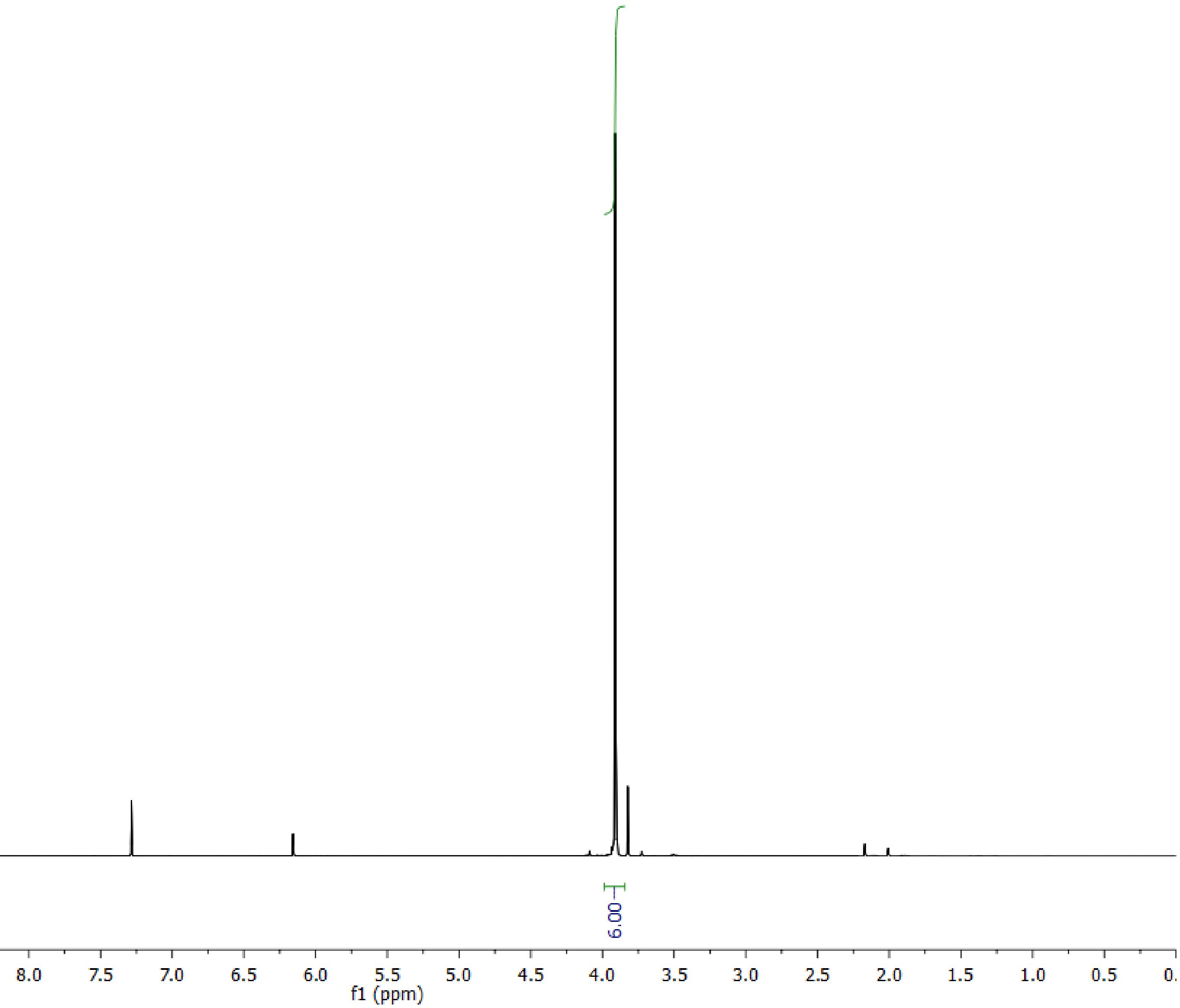

^{13}C NMR (100 MHz, $CDCl_3$)

Figure S83. ^{13}C NMR of **50**

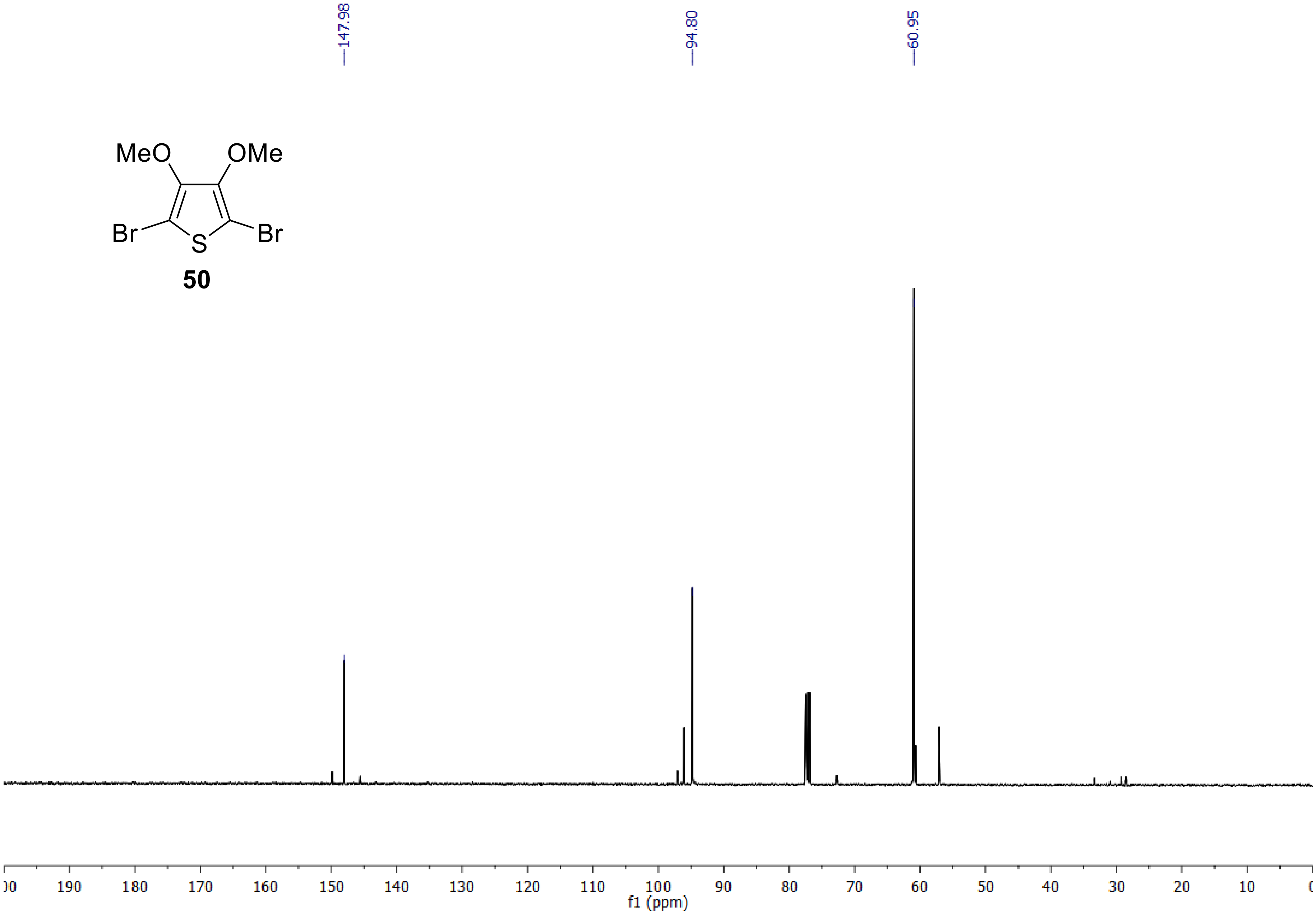

^{1}H NMR (400 MHz, $CDCl_3$)

Figure S84. ^{1}H NMR of **55**

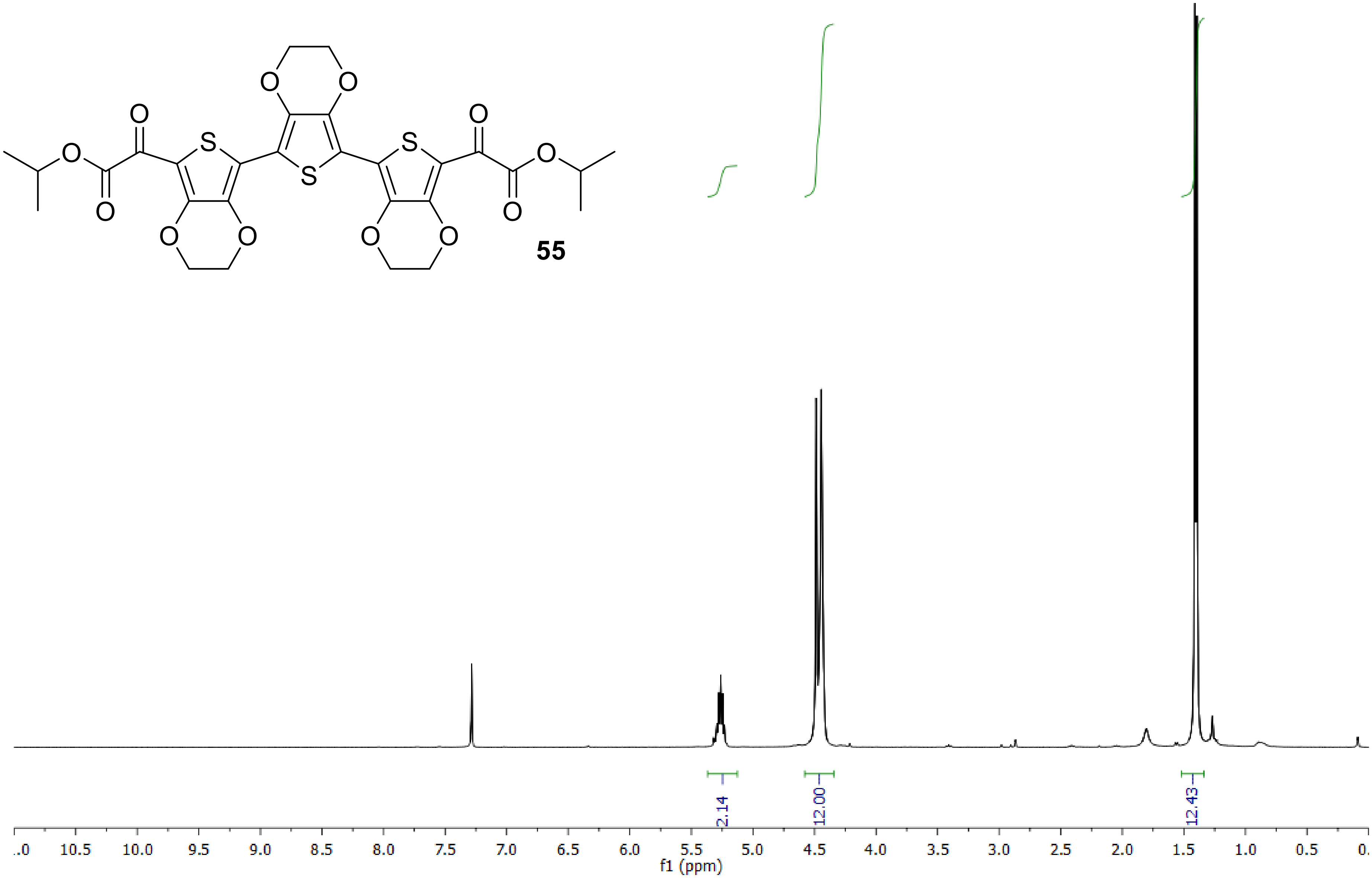

S143 ^{13}C NMR (100 MHz, $CDCl_3$) **Figure S85.** ^{13}C NMR of **55**

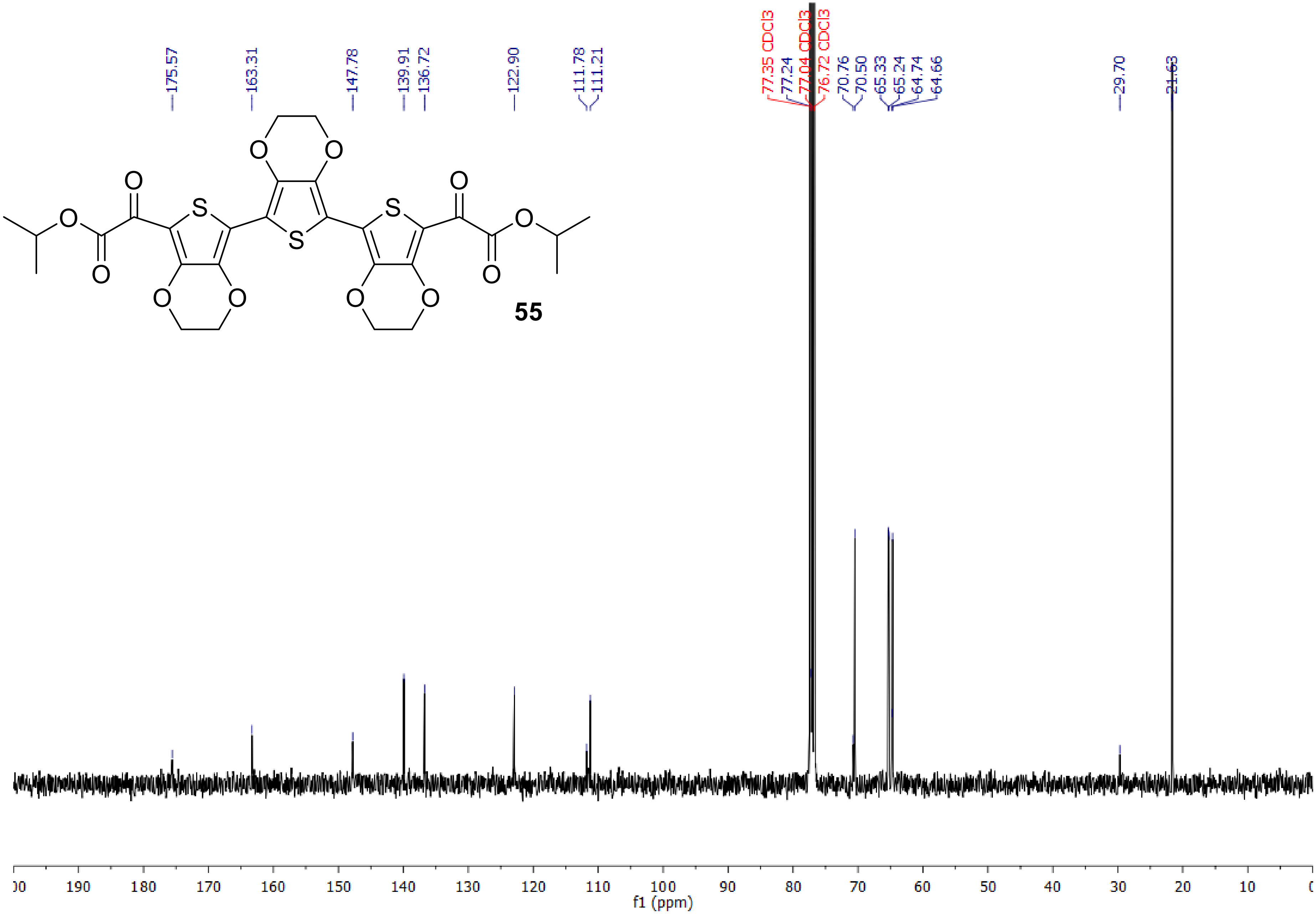

^1H NMR (400 MHz, $CDCl_3$)

Figure S86. ^1H NMR of **56**

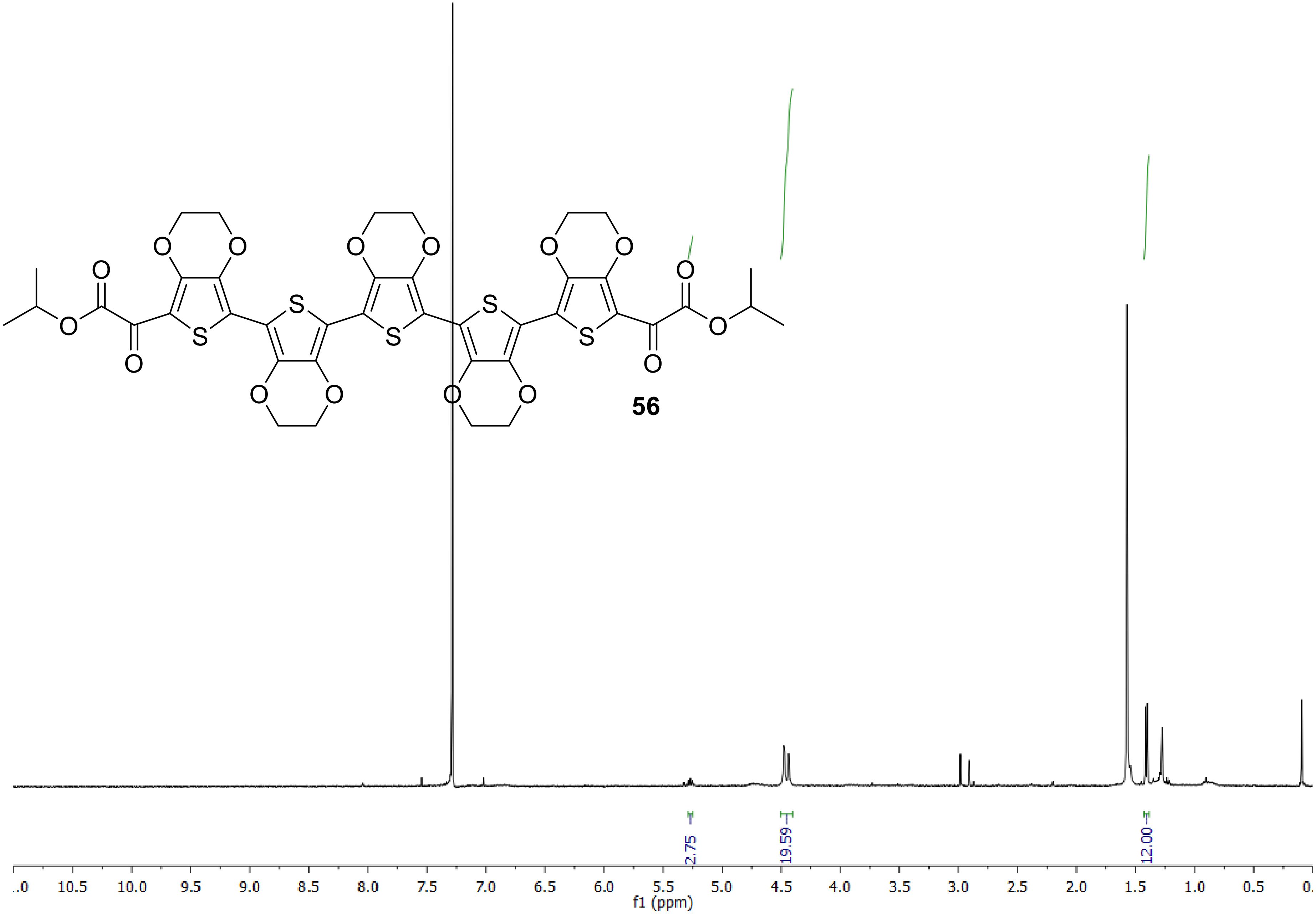

^{1}H NMR (400 MHz, $CDCl_3$)

Figure S87. ^{1}H NMR of **57**

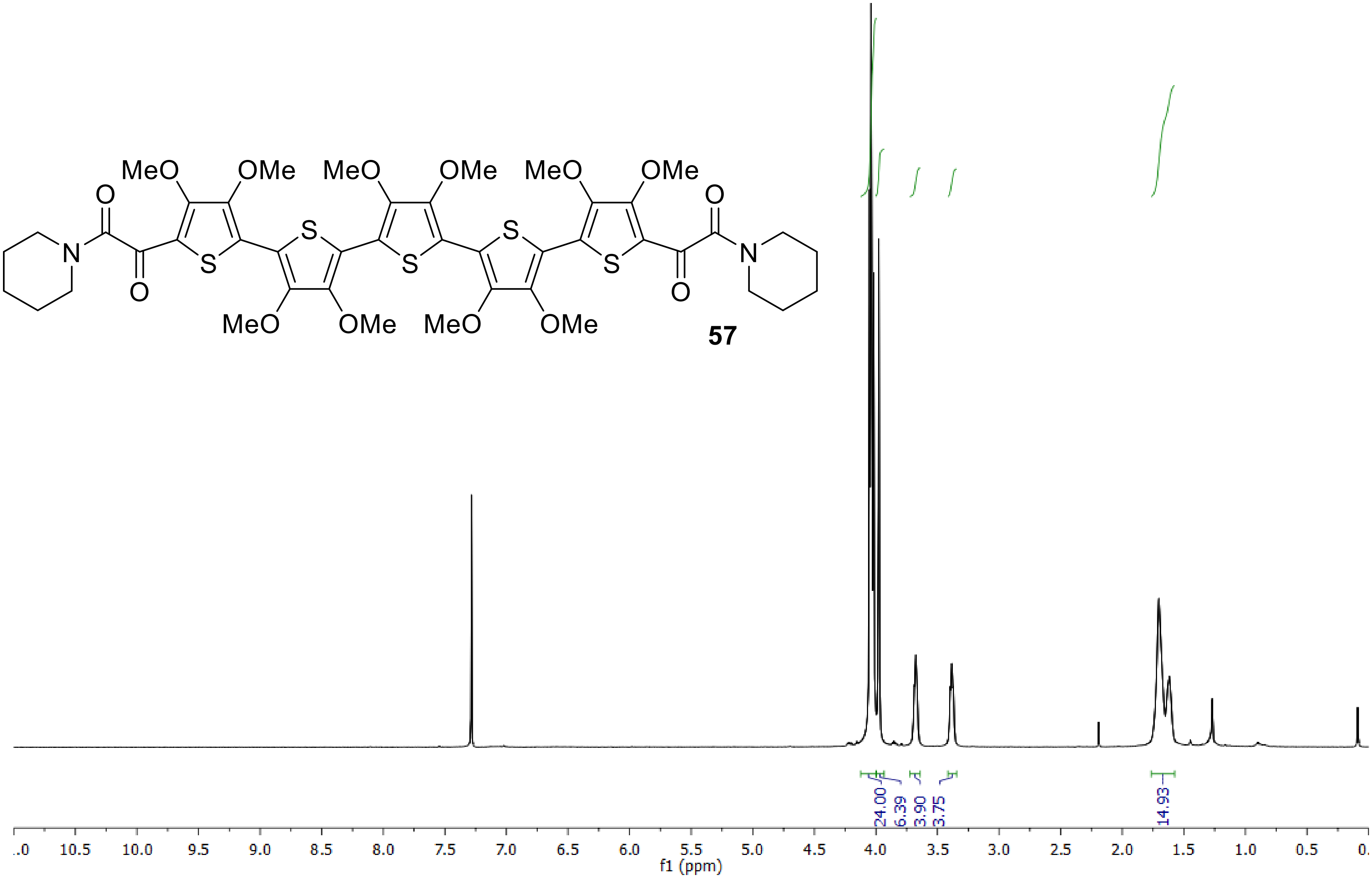

^1H NMR (400 MHz, $CDCl_3$)

Figure S88. ^1H NMR of **58**

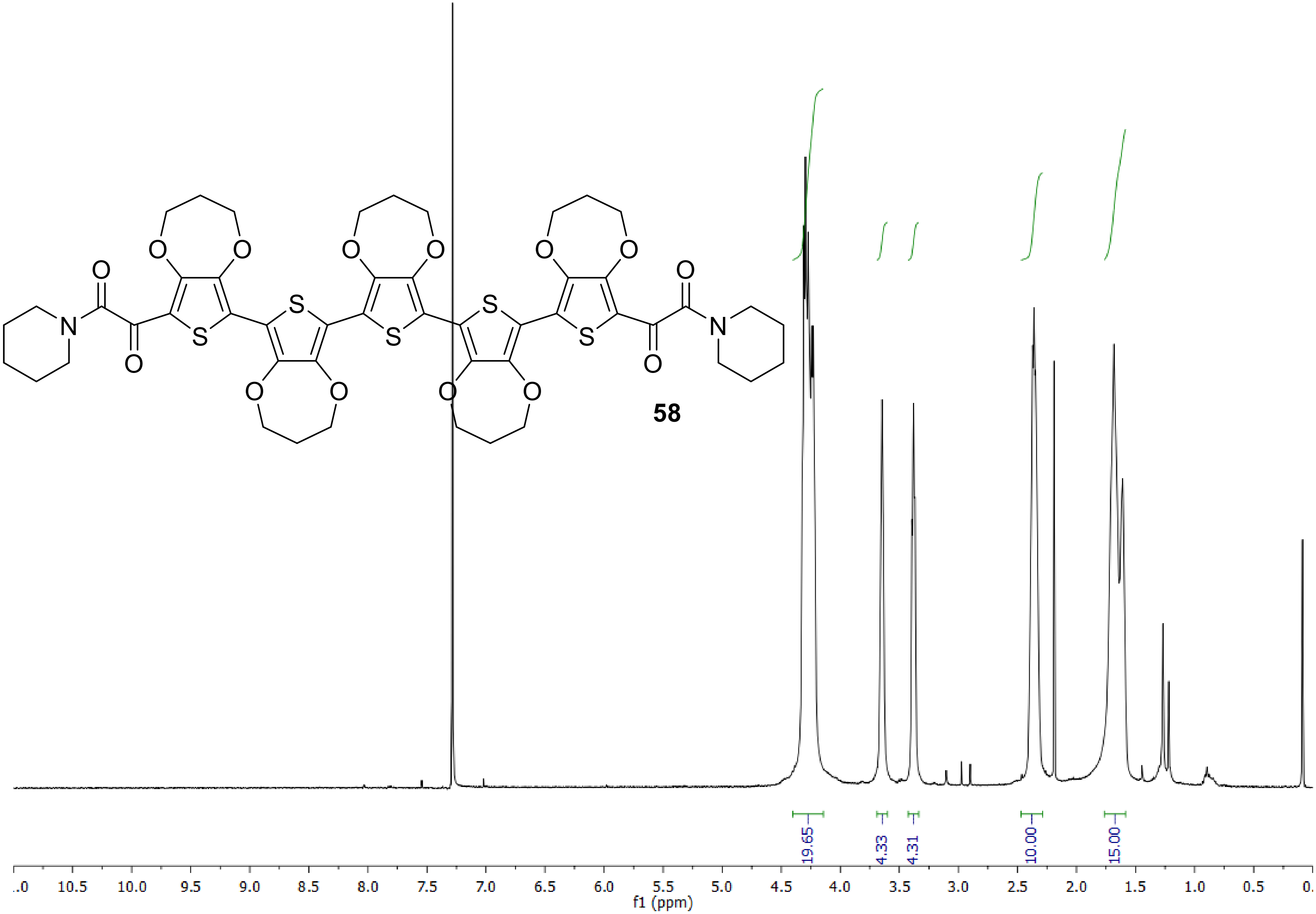

^{1}H NMR (400 MHz, $CDCl_3$)

Figure S89. ^{1}H NMR of **60**

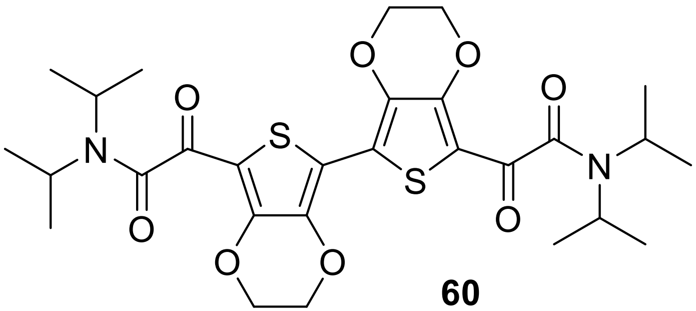

60

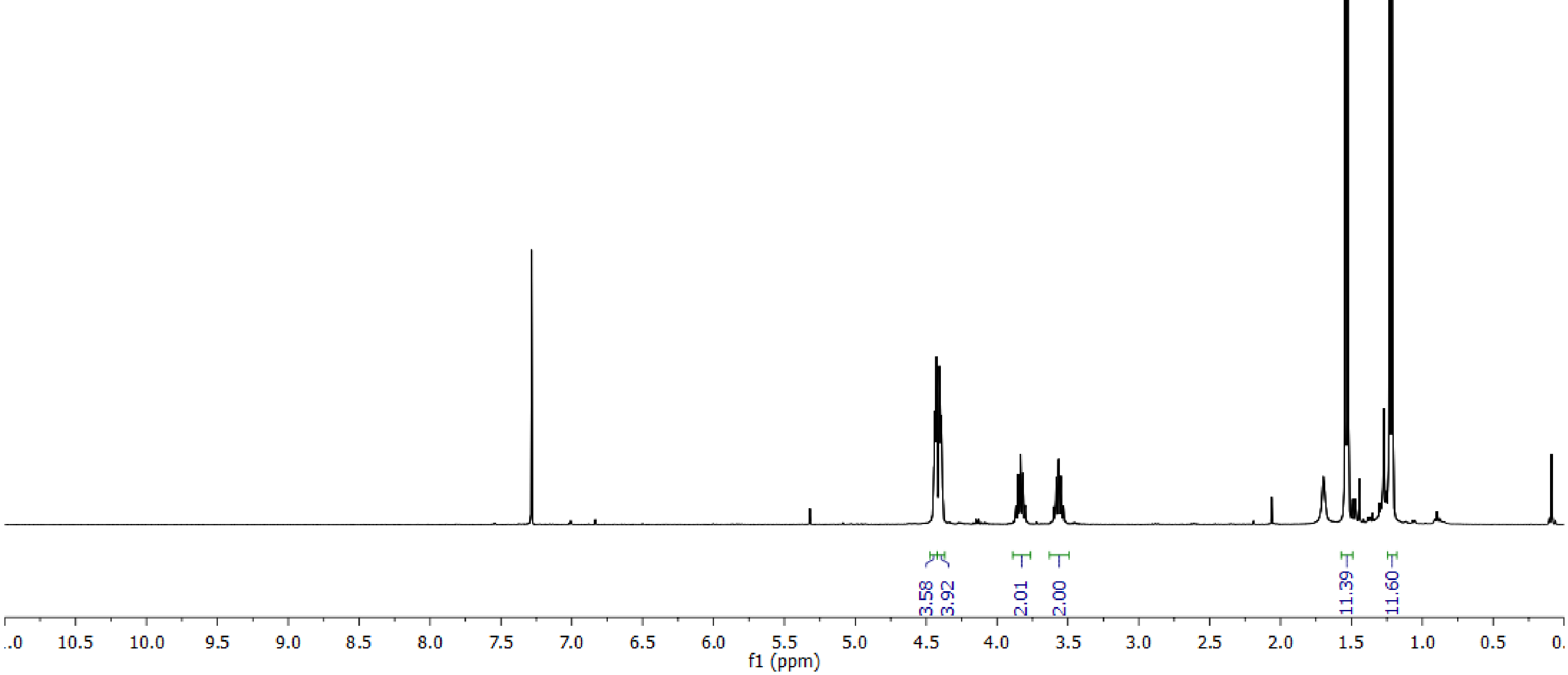

^{13}C NMR (100 MHz, $CDCl_3$)

Figure S90. ^{13}C NMR of **60**

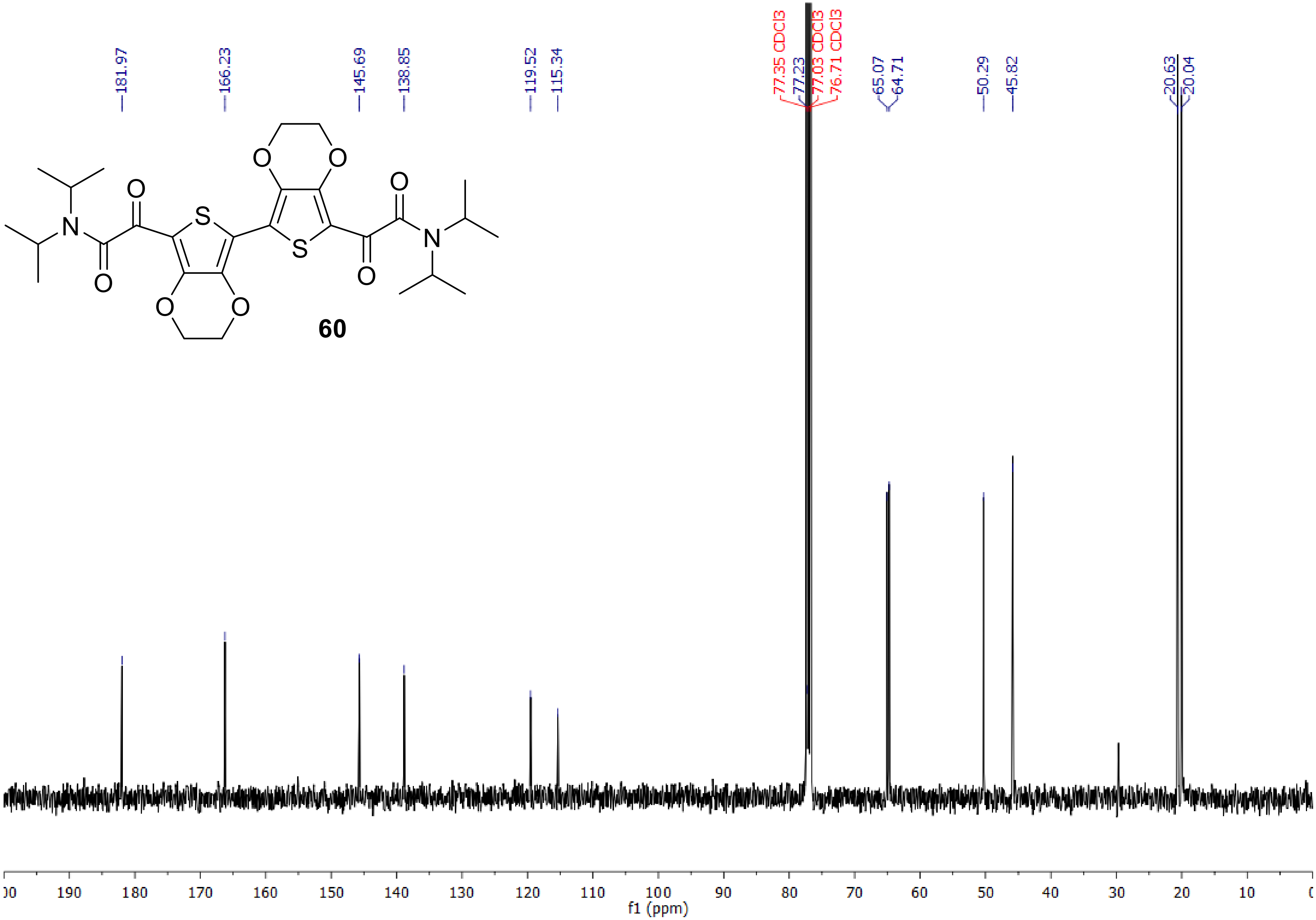

^{1}H NMR (400 MHz, $CDCl_3$)

Figure S91. ^{1}H NMR of **65**

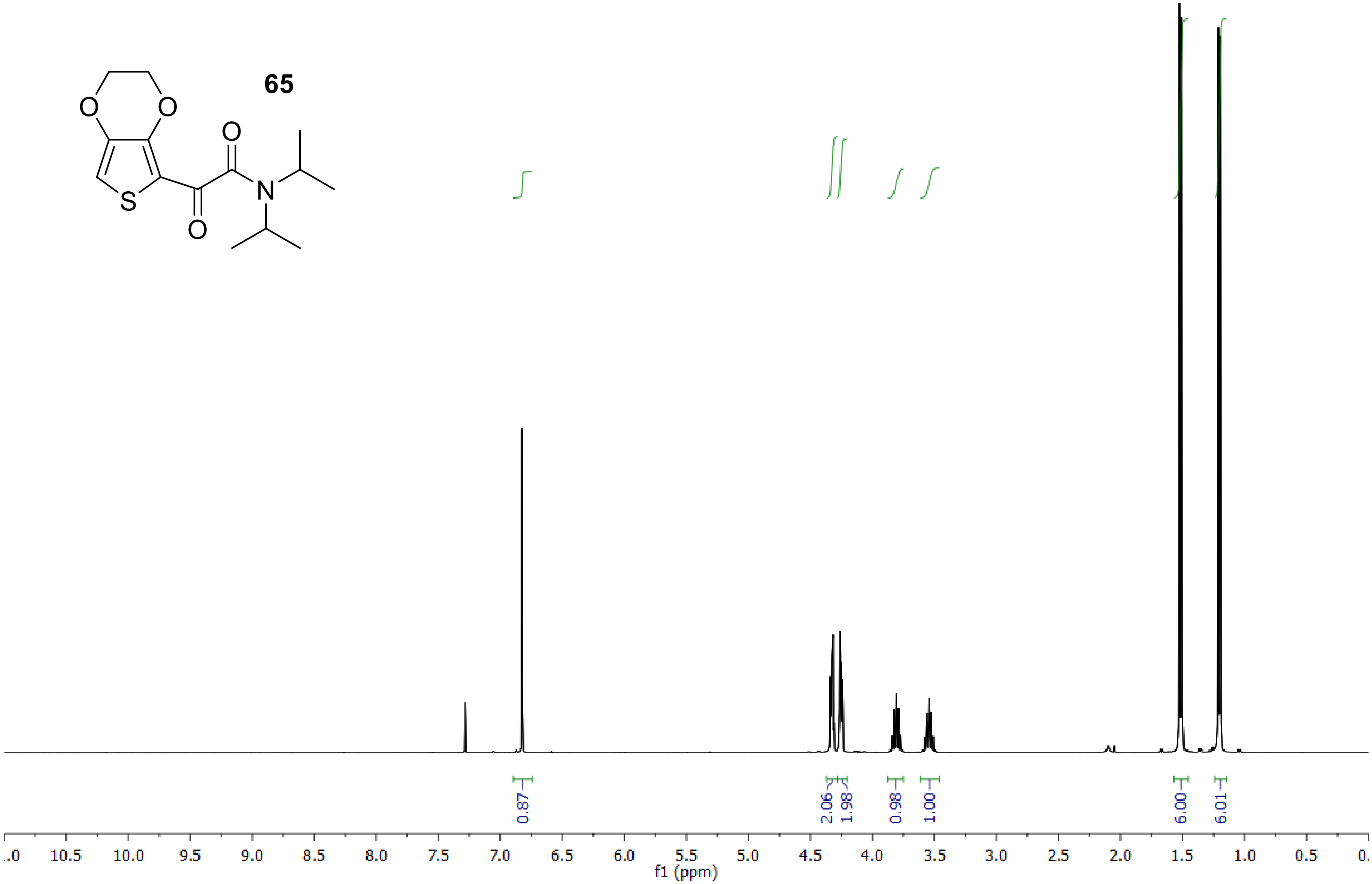

^{13}C NMR (100 MHz, $CDCl_3$)

Figure S92. ^{13}C NMR of **65**

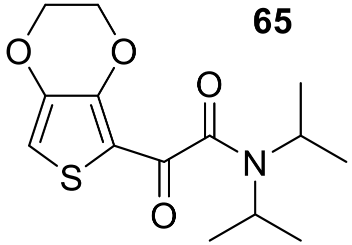

65

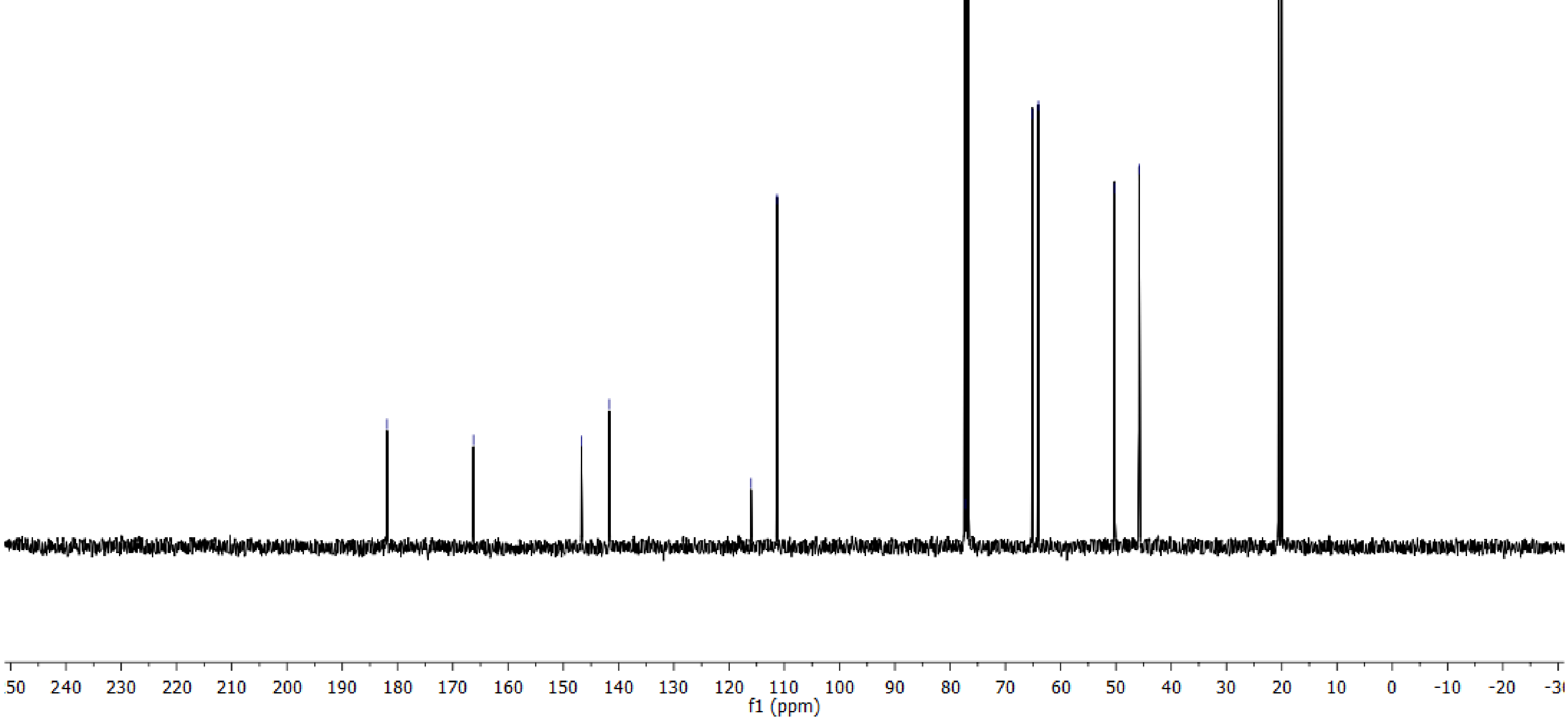

^{1}H NMR (400 MHz, $CDCl_3$)

Figure S93. ^{1}H NMR of **66**

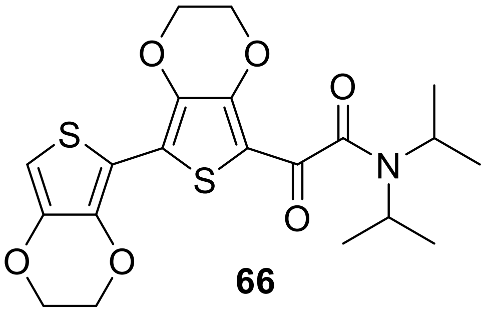

66

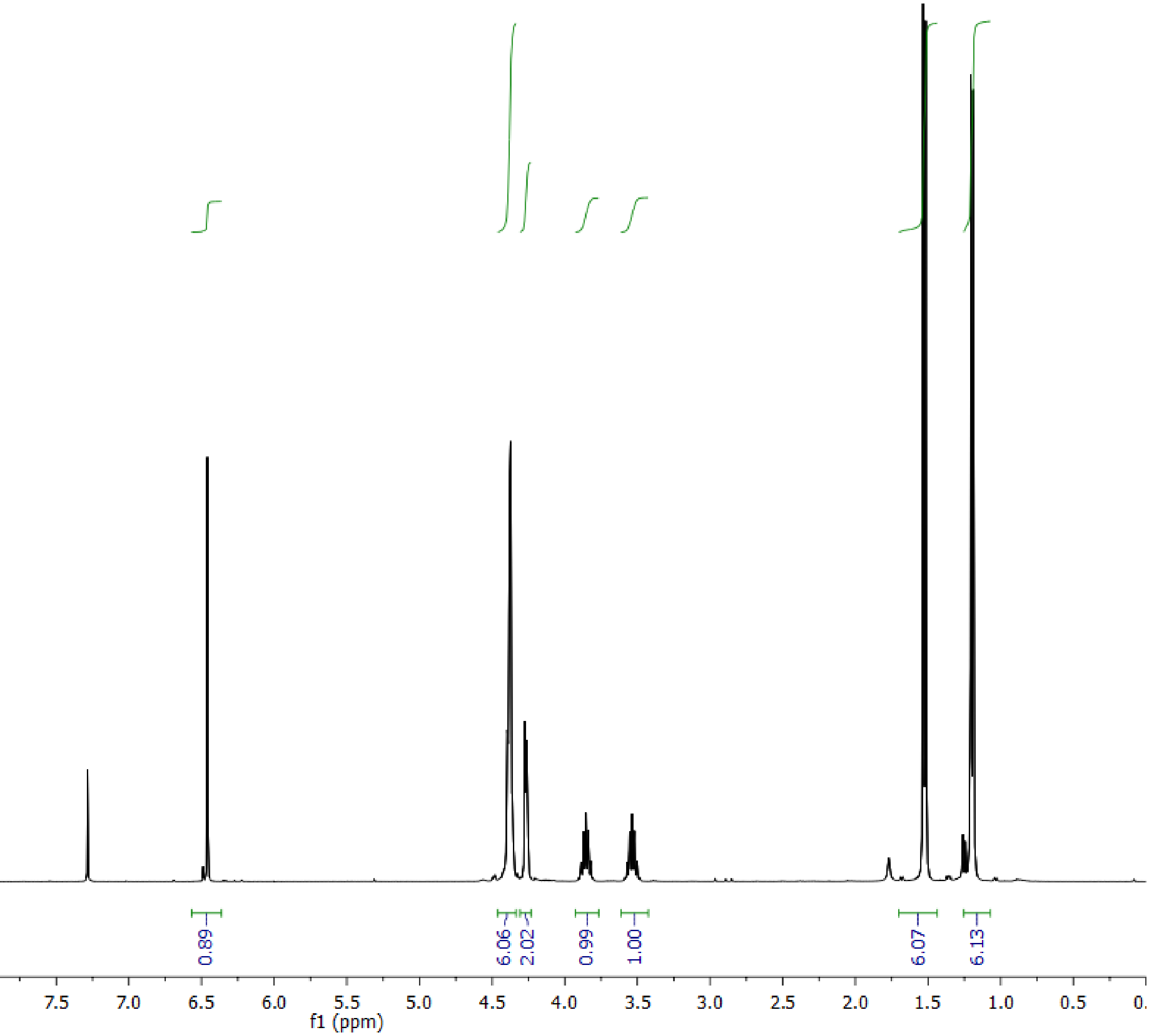

^{13}C NMR (100 MHz, $CDCl_3$)

Figure S94. ^{13}C NMR of **66**

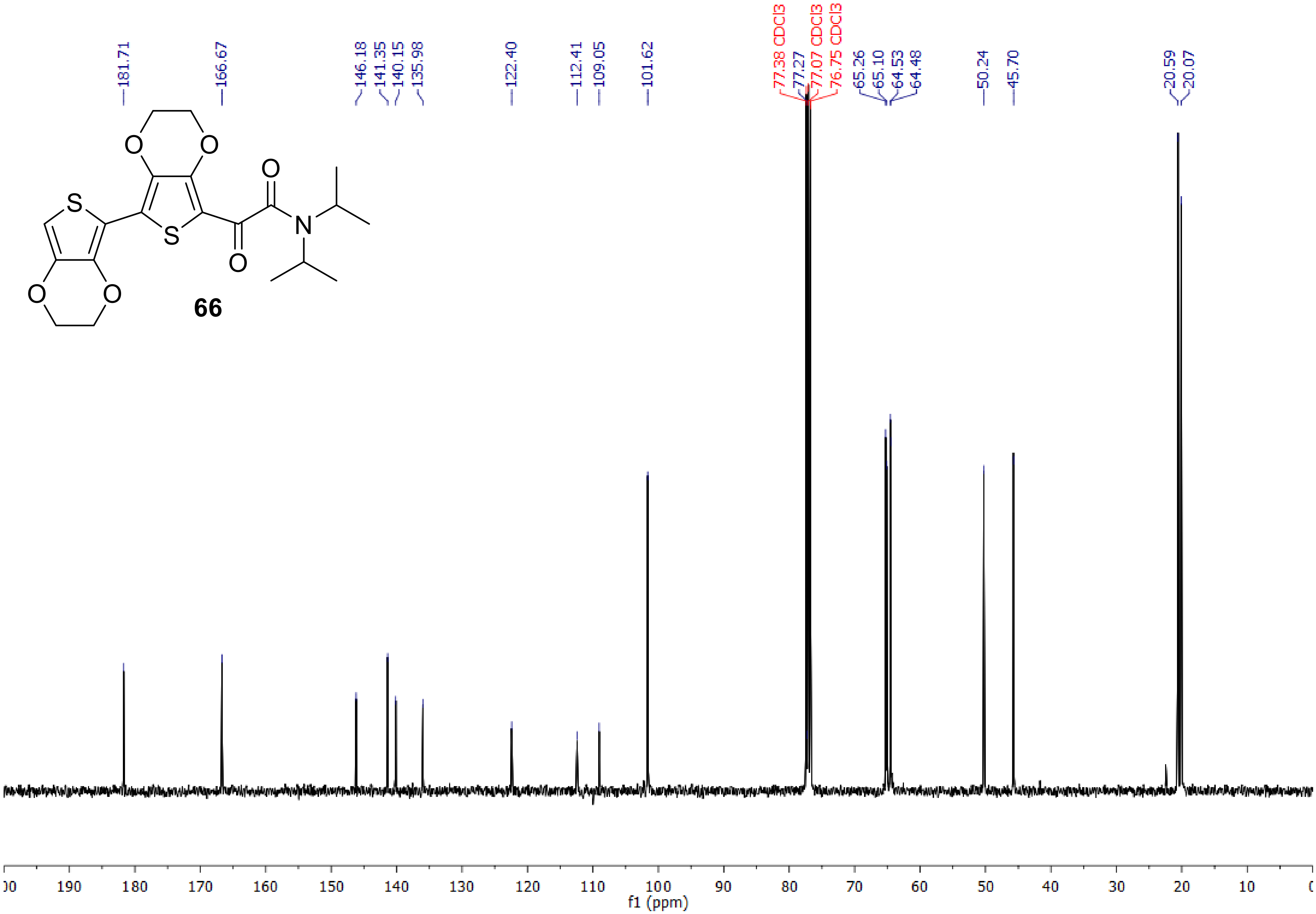

^{1}H NMR (400 MHz, $CDCl_3$)

Figure S95. ^{1}H NMR of **67**

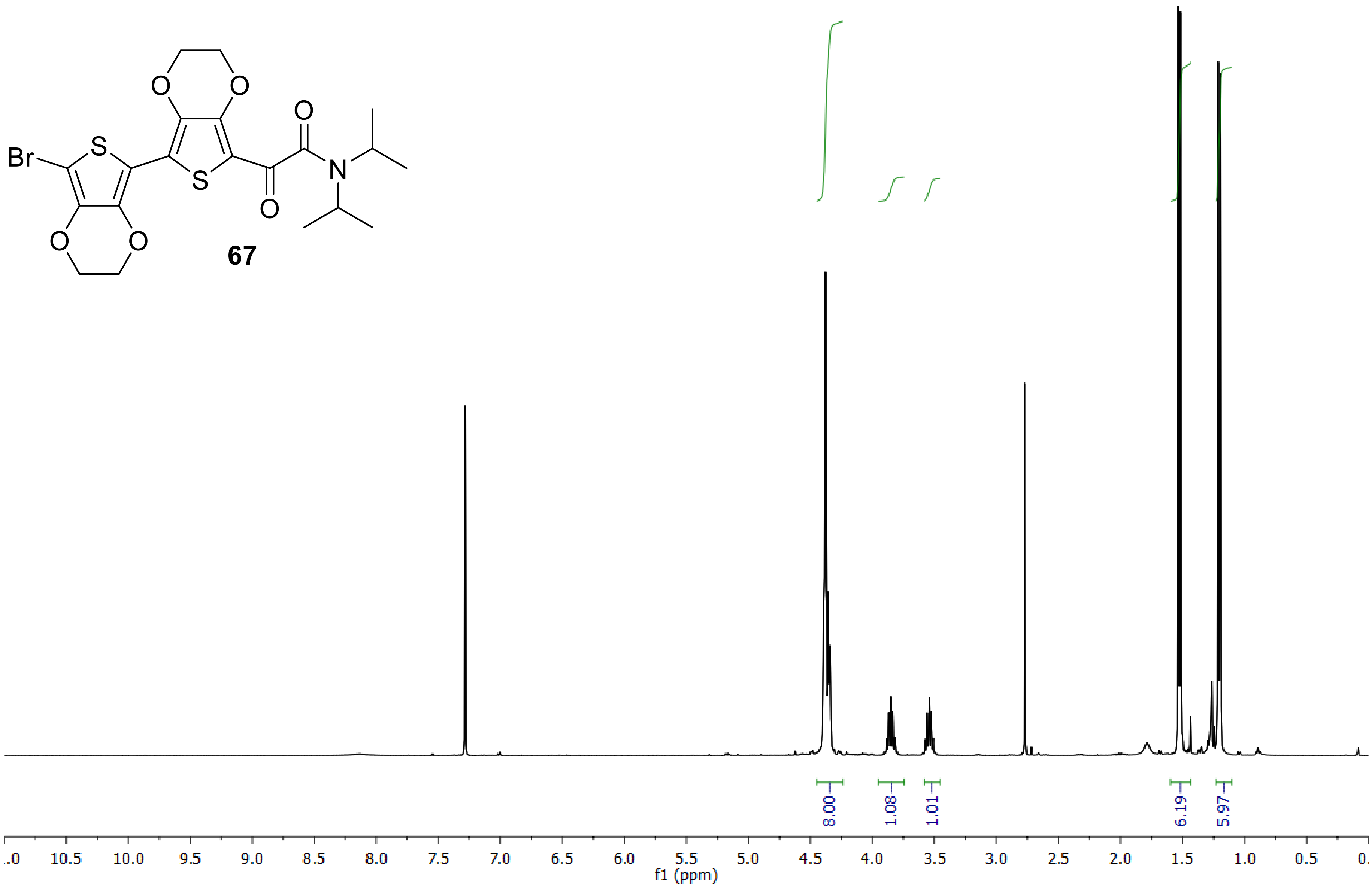

^{13}C NMR (100 MHz, $CDCl_3$)

Figure S96. ^{13}C NMR of **67**

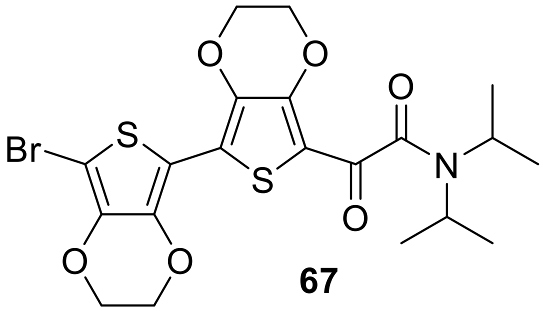

67

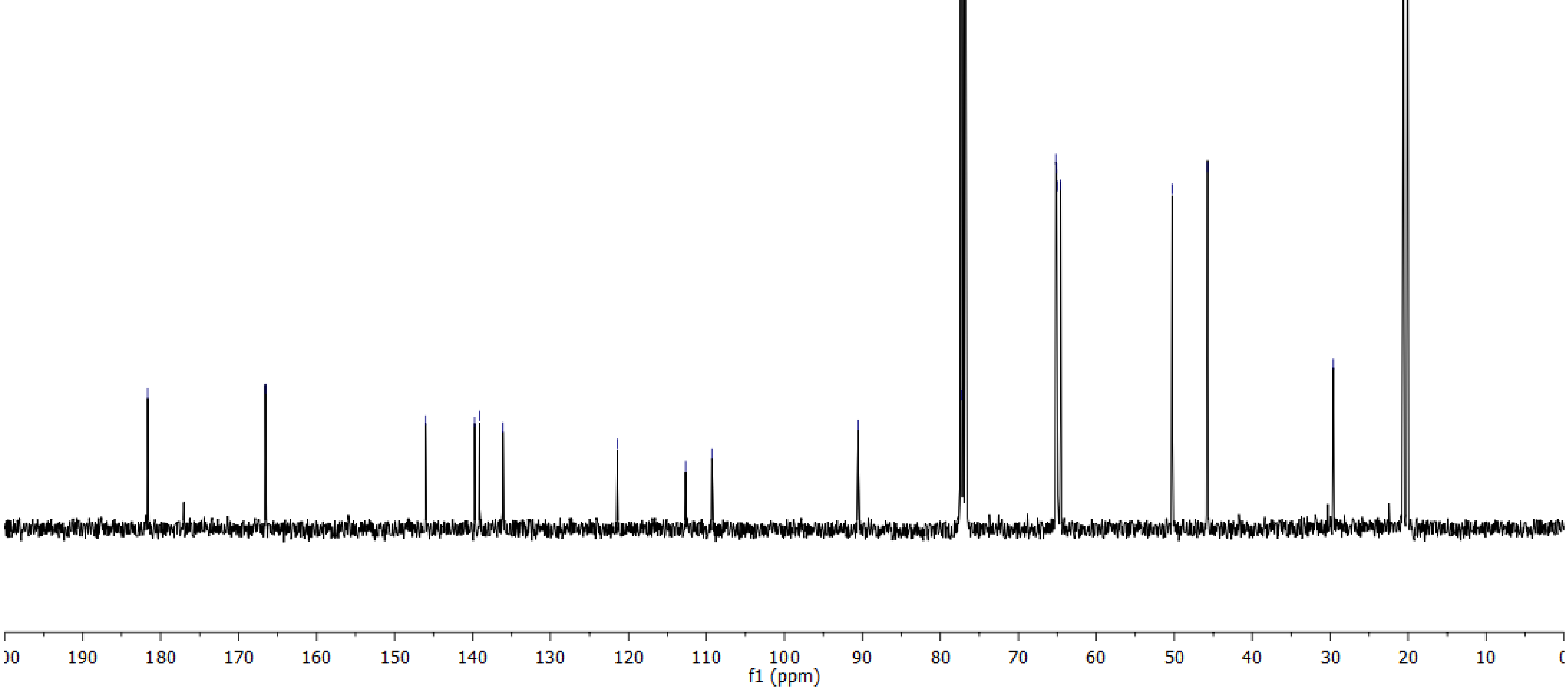

^{1}H NMR (400 MHz, $CDCl_3$)

Figure S97. ^{1}H NMR of **68**

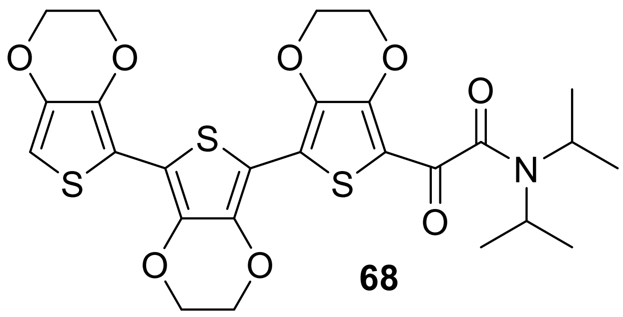

68

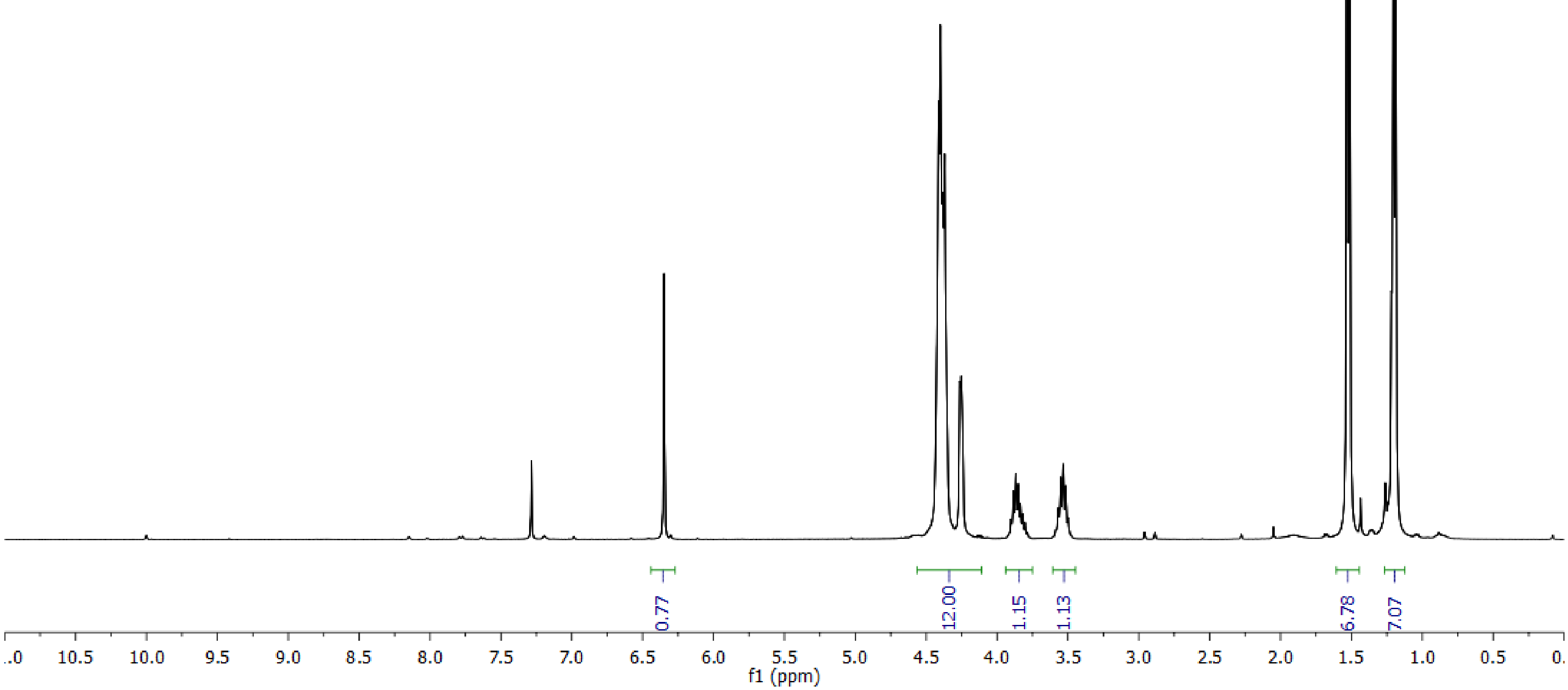

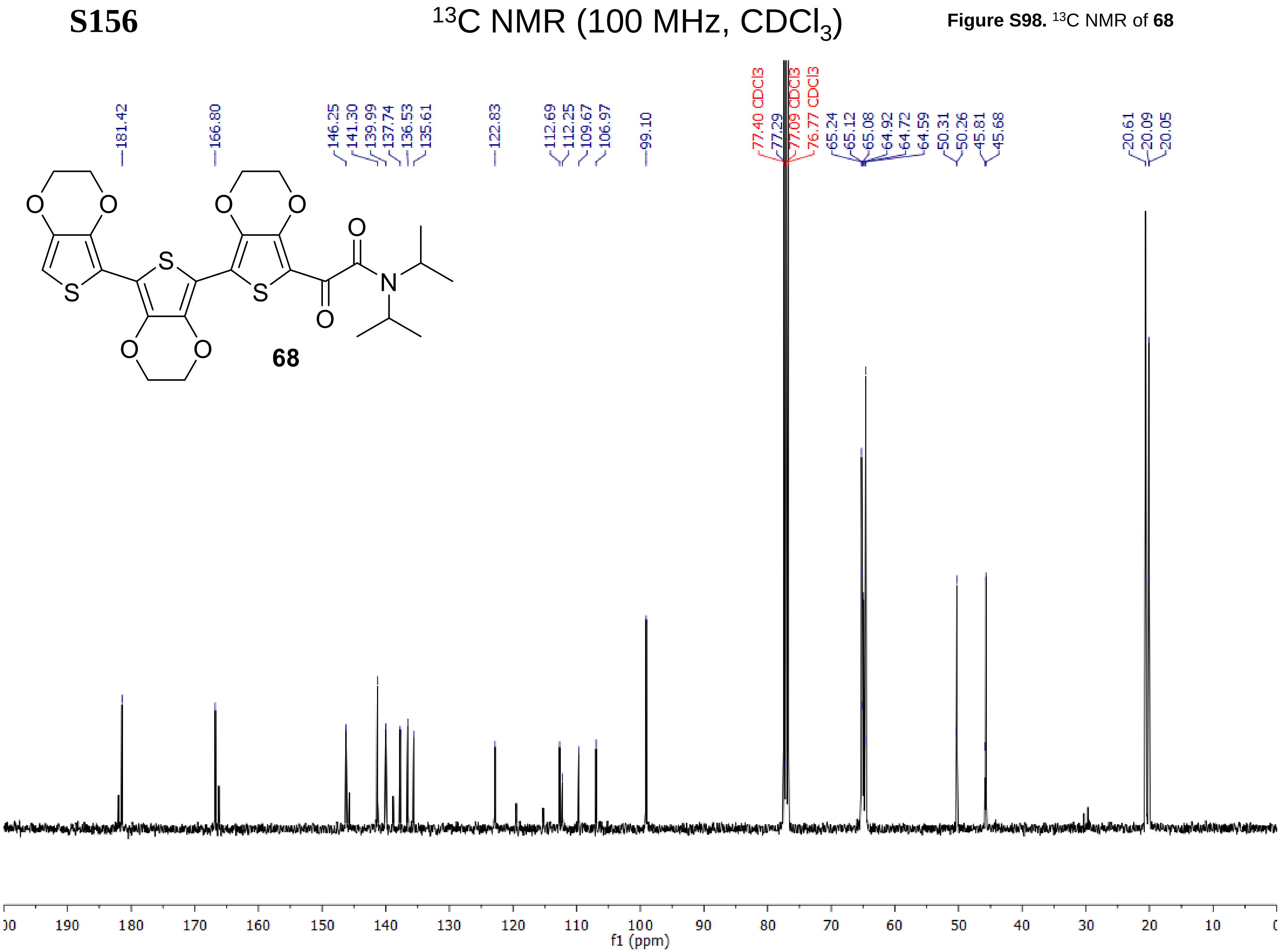

Figure S98. ^{13}C NMR of **68**

^{1}H NMR (400 MHz, $CDCl_3$)

Figure S99. ^{1}H NMR of **69**

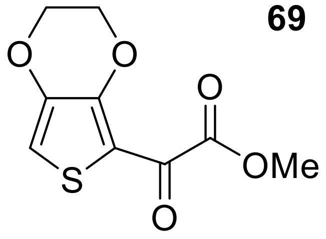

69

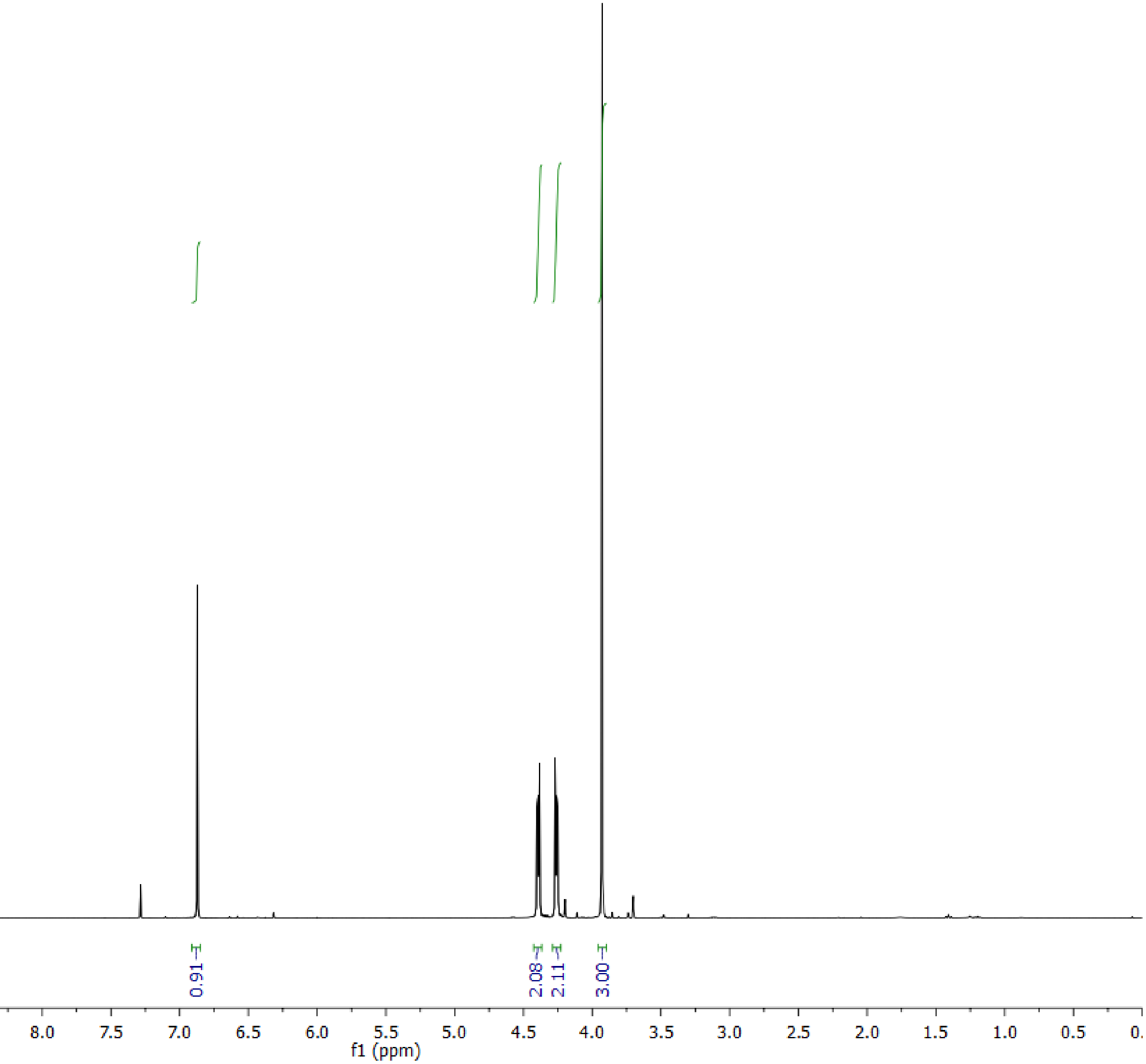

^{13}C NMR (100 MHz, $CDCl_3$)

Figure S100. ^{13}C NMR of **69**

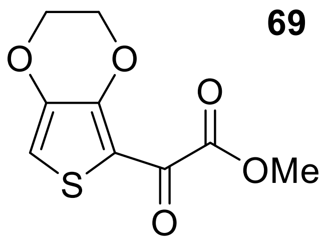

69

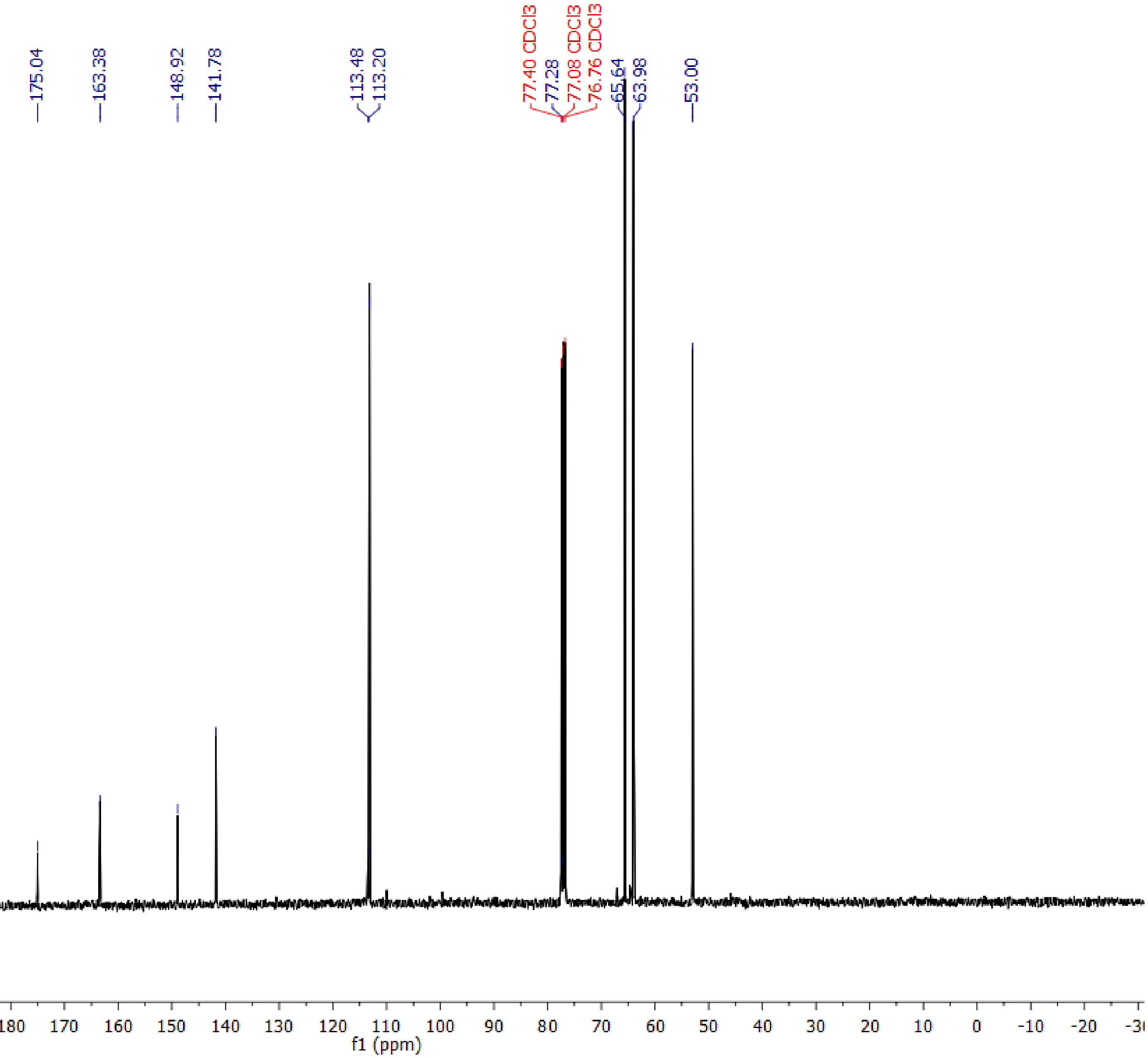

^{1}H NMR (400 MHz, $CDCl_3$)

Figure S101. ^{1}H NMR of **72**

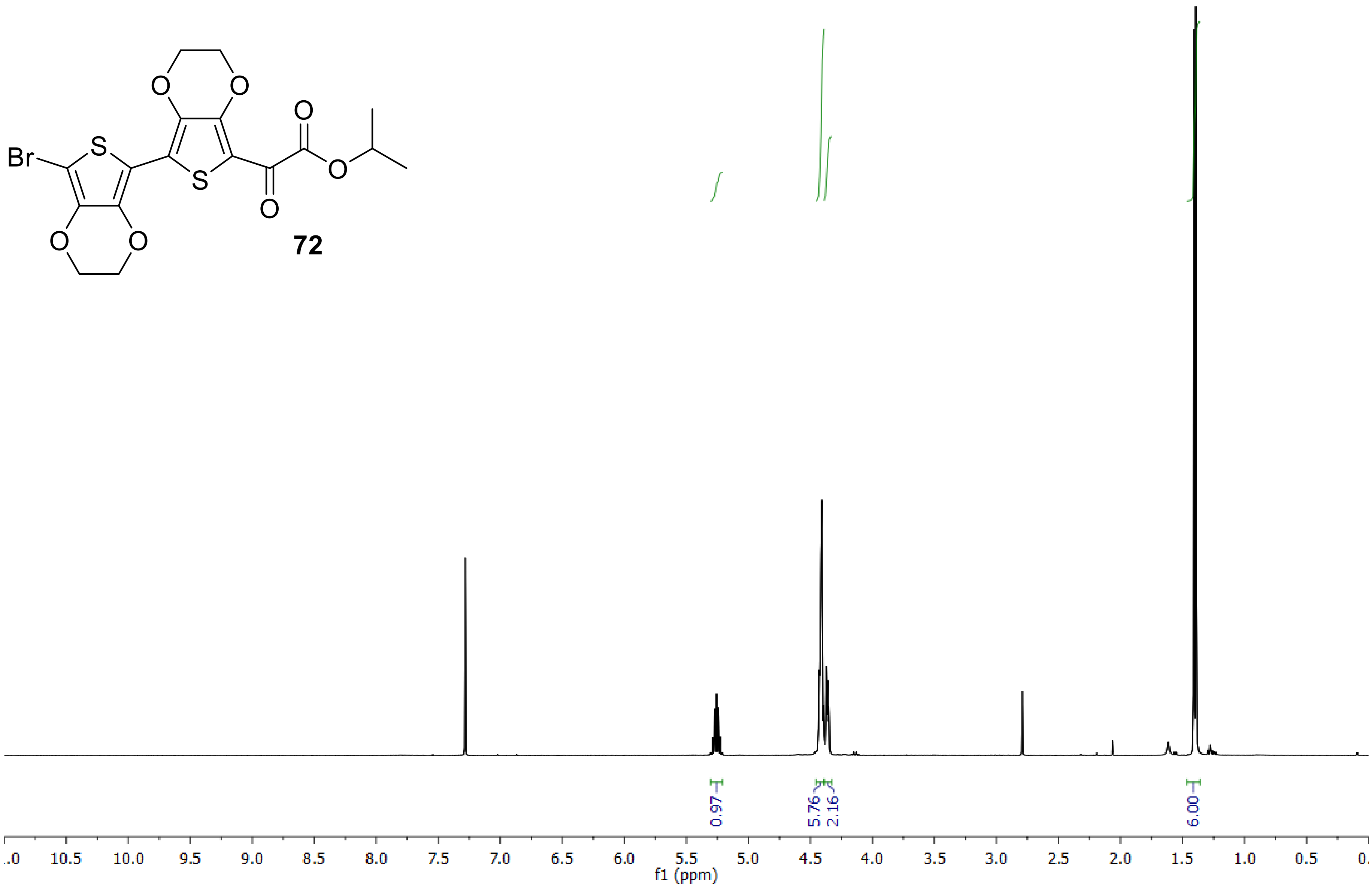

^{13}C NMR (100 MHz, $CDCl_3$)

Figure S102. ^{13}C NMR of **72**

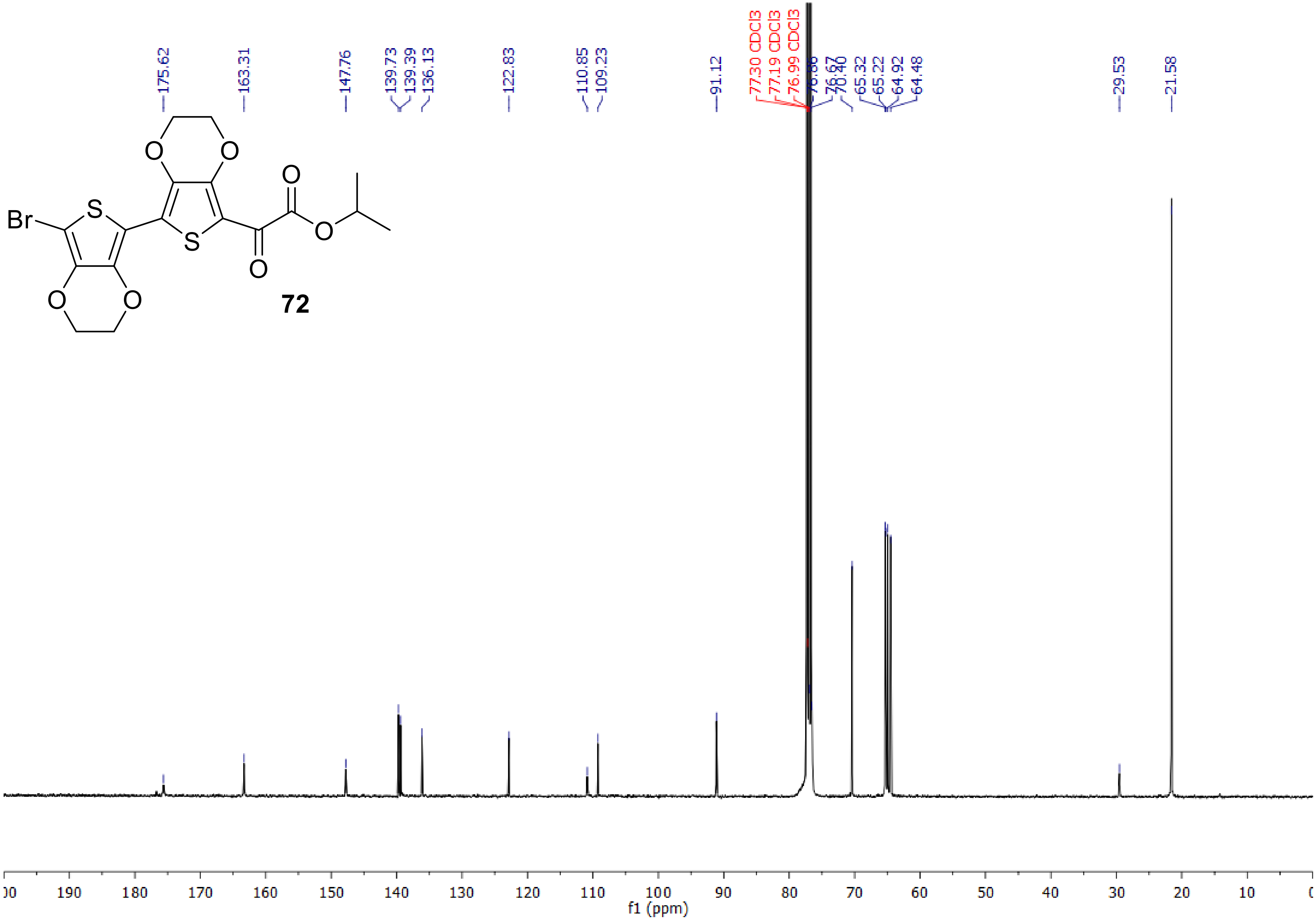

^{1}H NMR (400 MHz, $CDCl_3$)

Figure S103. ^{1}H NMR of **73**

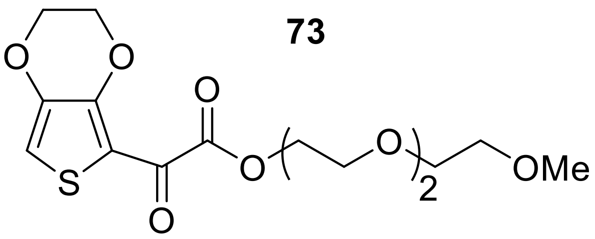

73

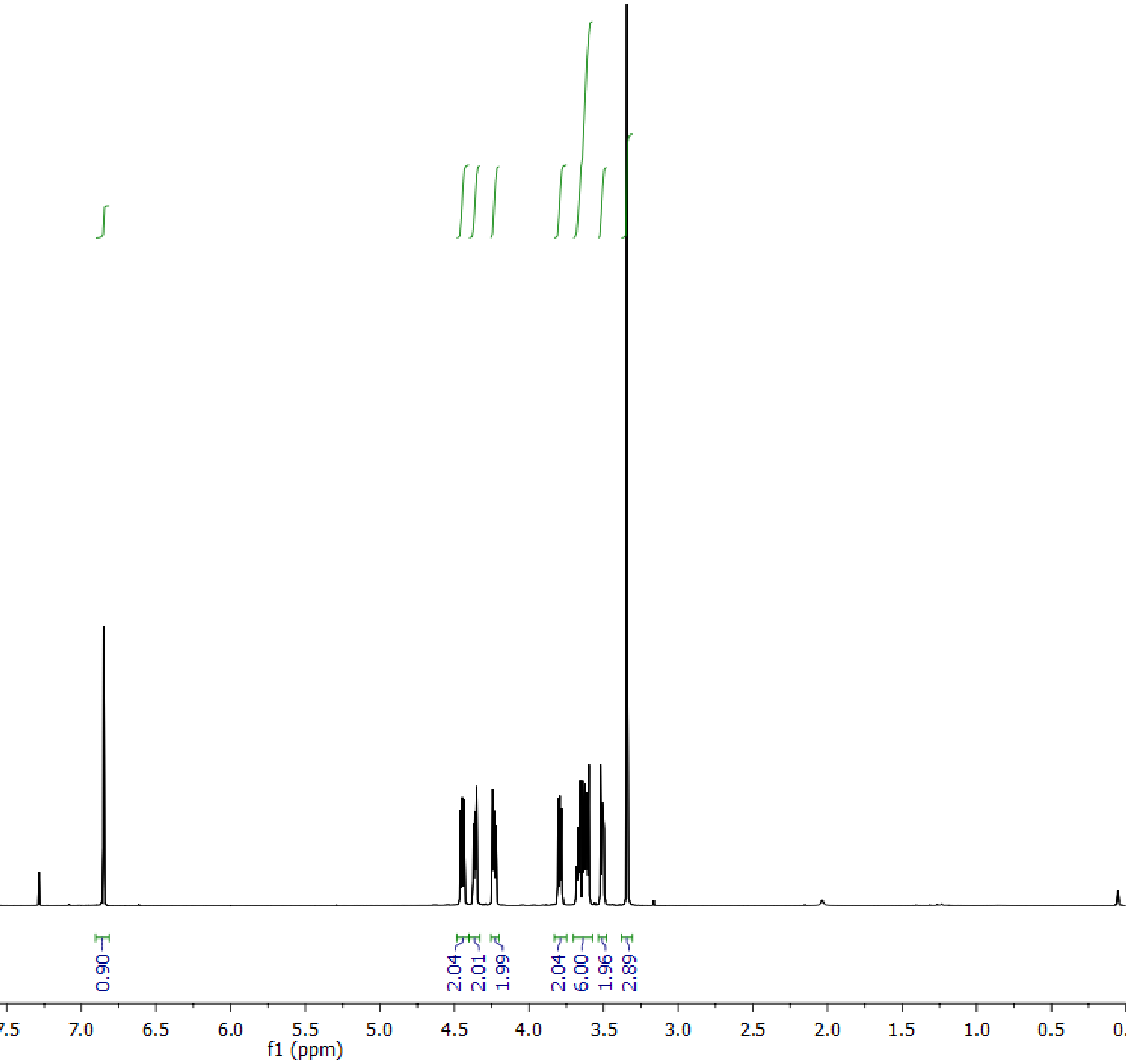

^{13}C NMR (100 MHz, $CDCl_3$)

Figure S104. ^{13}C NMR of **73**

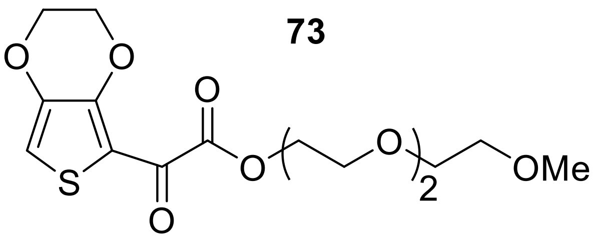

73

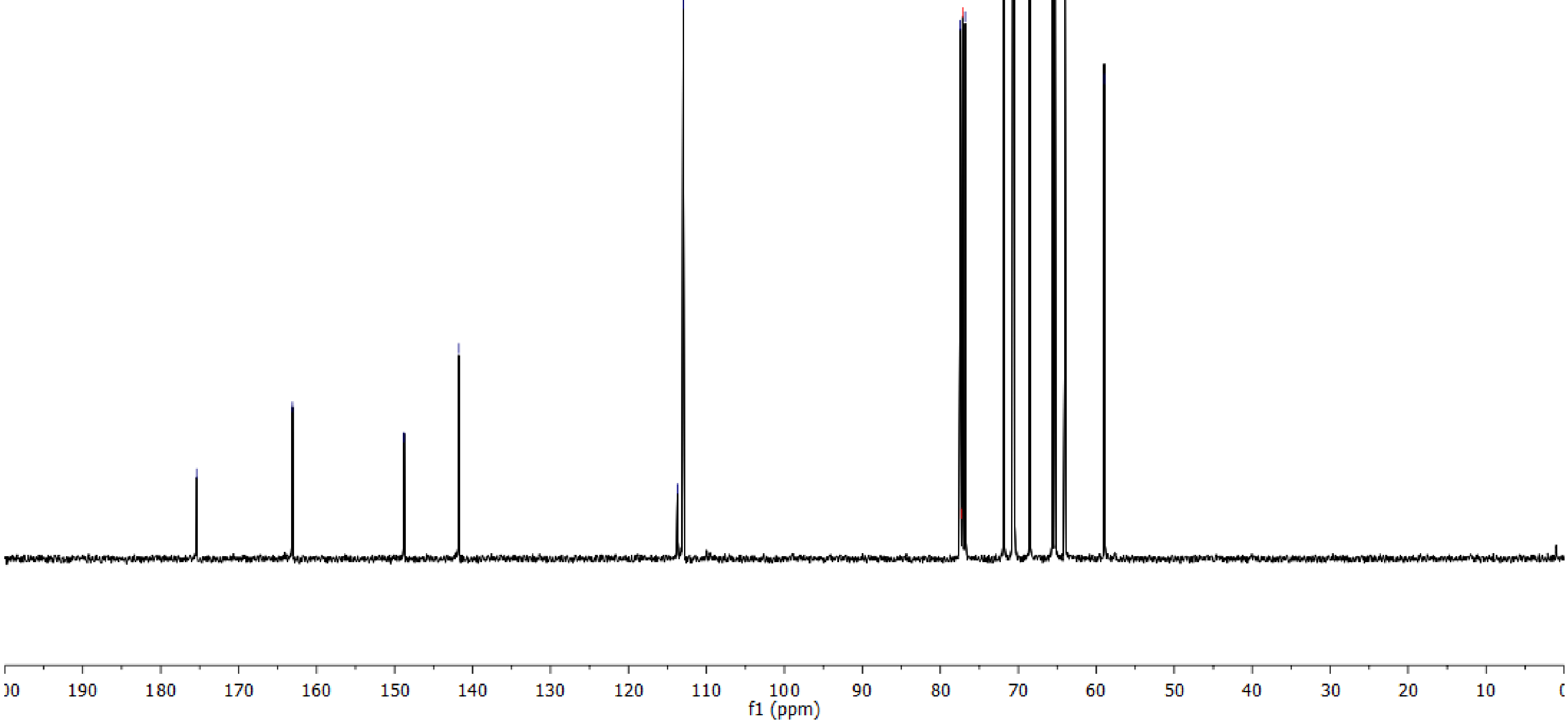

^{1}H NMR (400 MHz, $CDCl_3$)

Figure S105. ^{1}H NMR of **74**

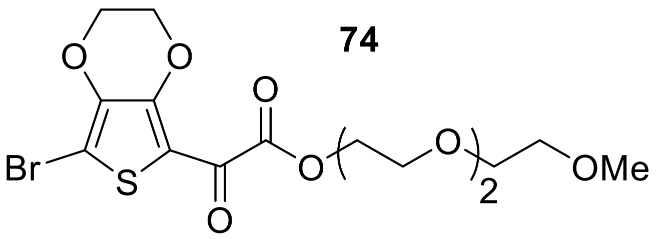

74

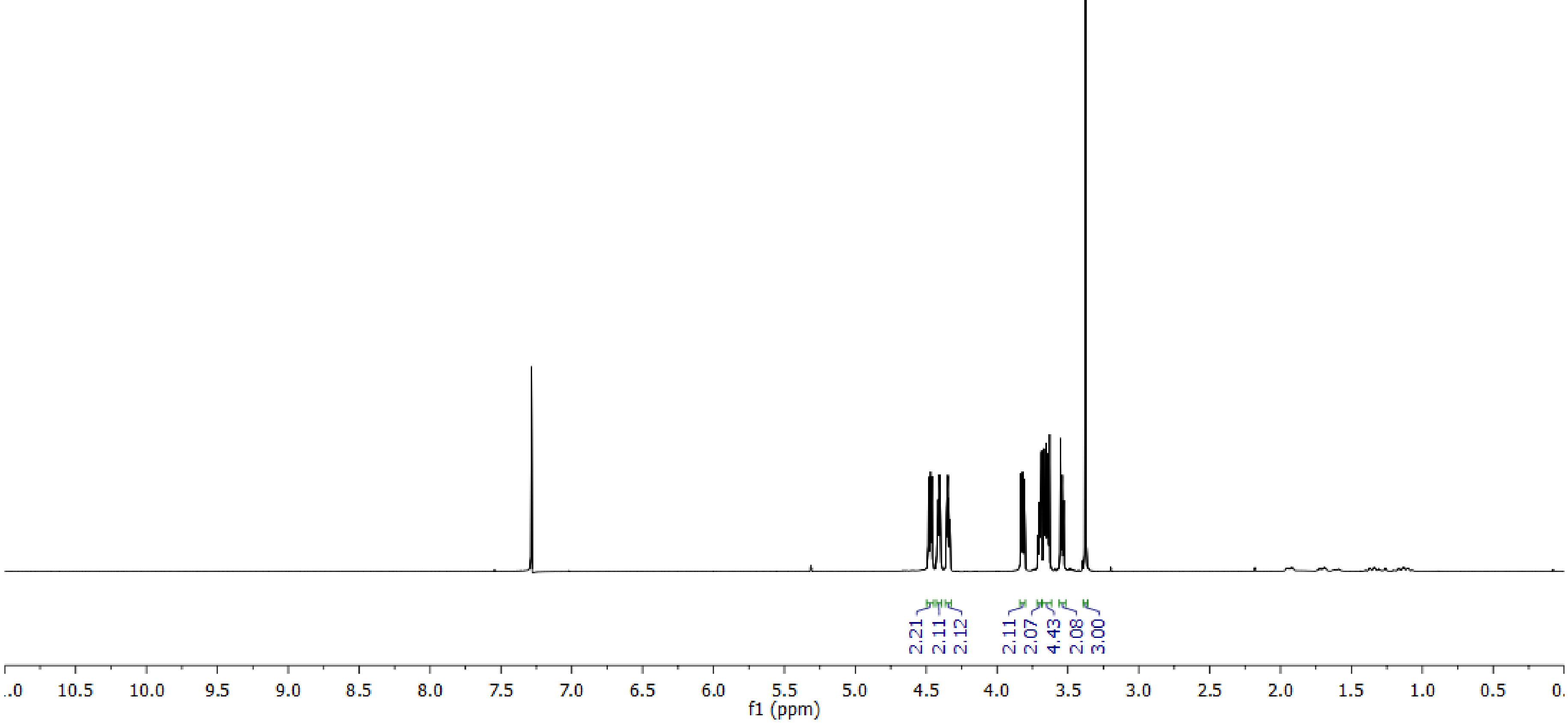

^{13}C NMR (100 MHz, $CDCl_3$)

Figure S106. ^{13}C NMR of **74**

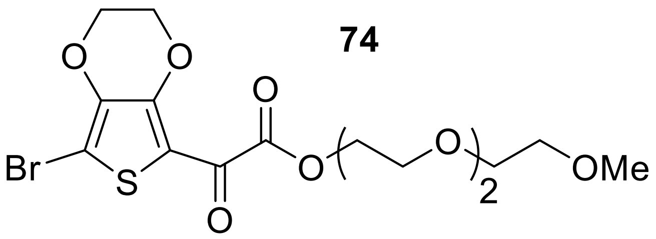

74

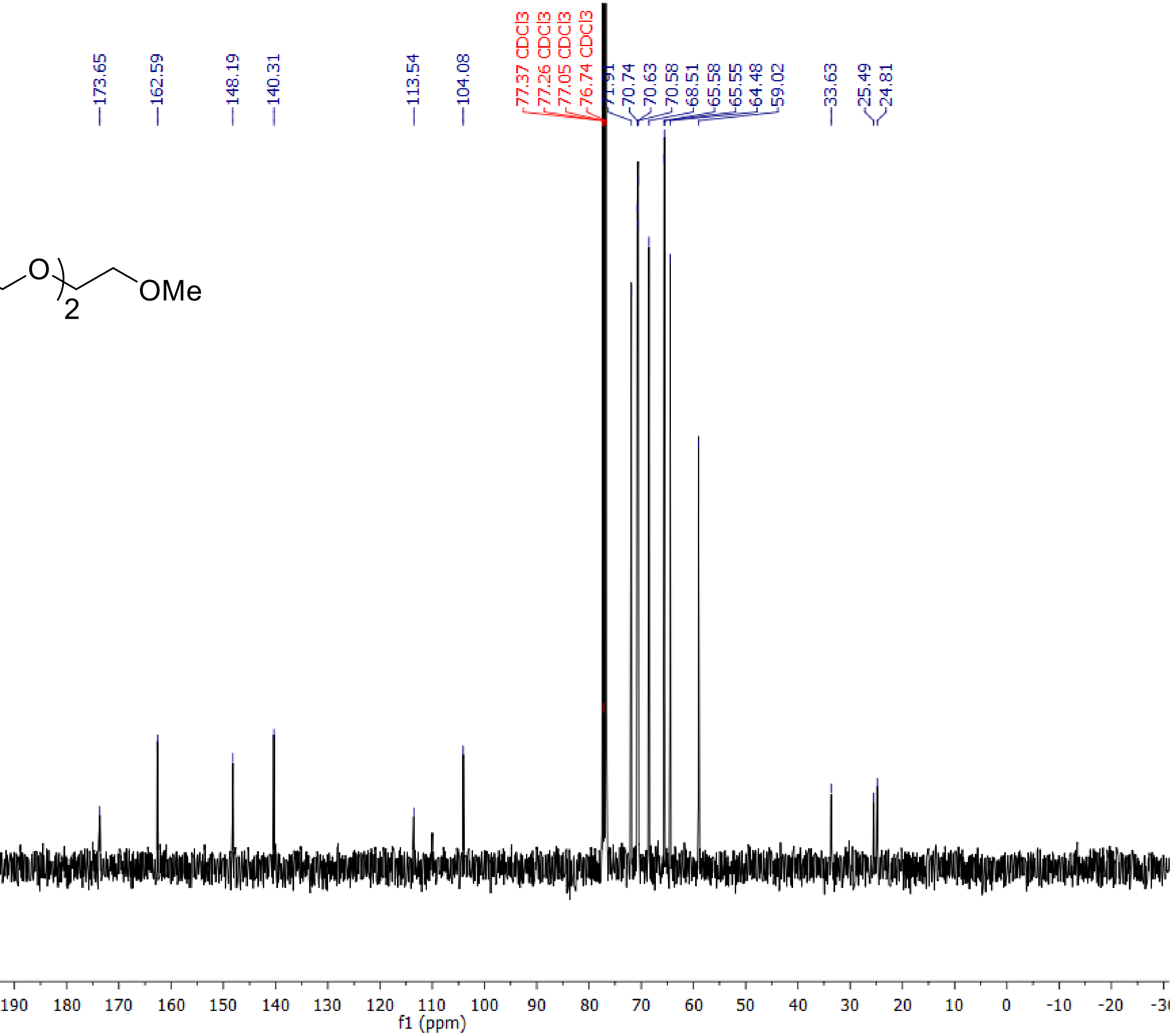

^{1}H NMR (400 MHz, $CDCl_3$)

Figure S107. ^{1}H NMR of **75**

75

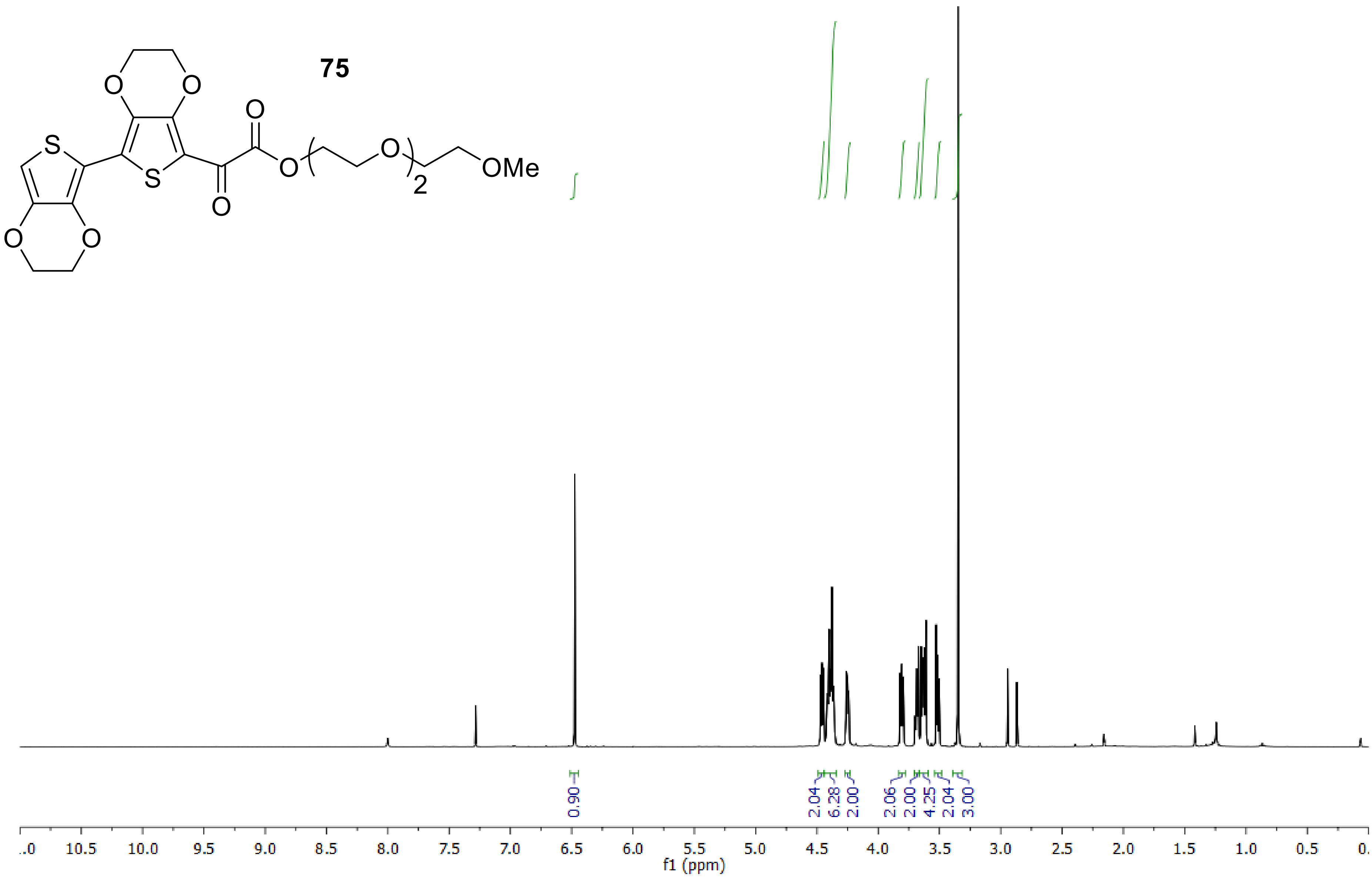

^{13}C NMR (100 MHz, $CDCl_3$)

Figure S108. ^{13}C NMR of **75**

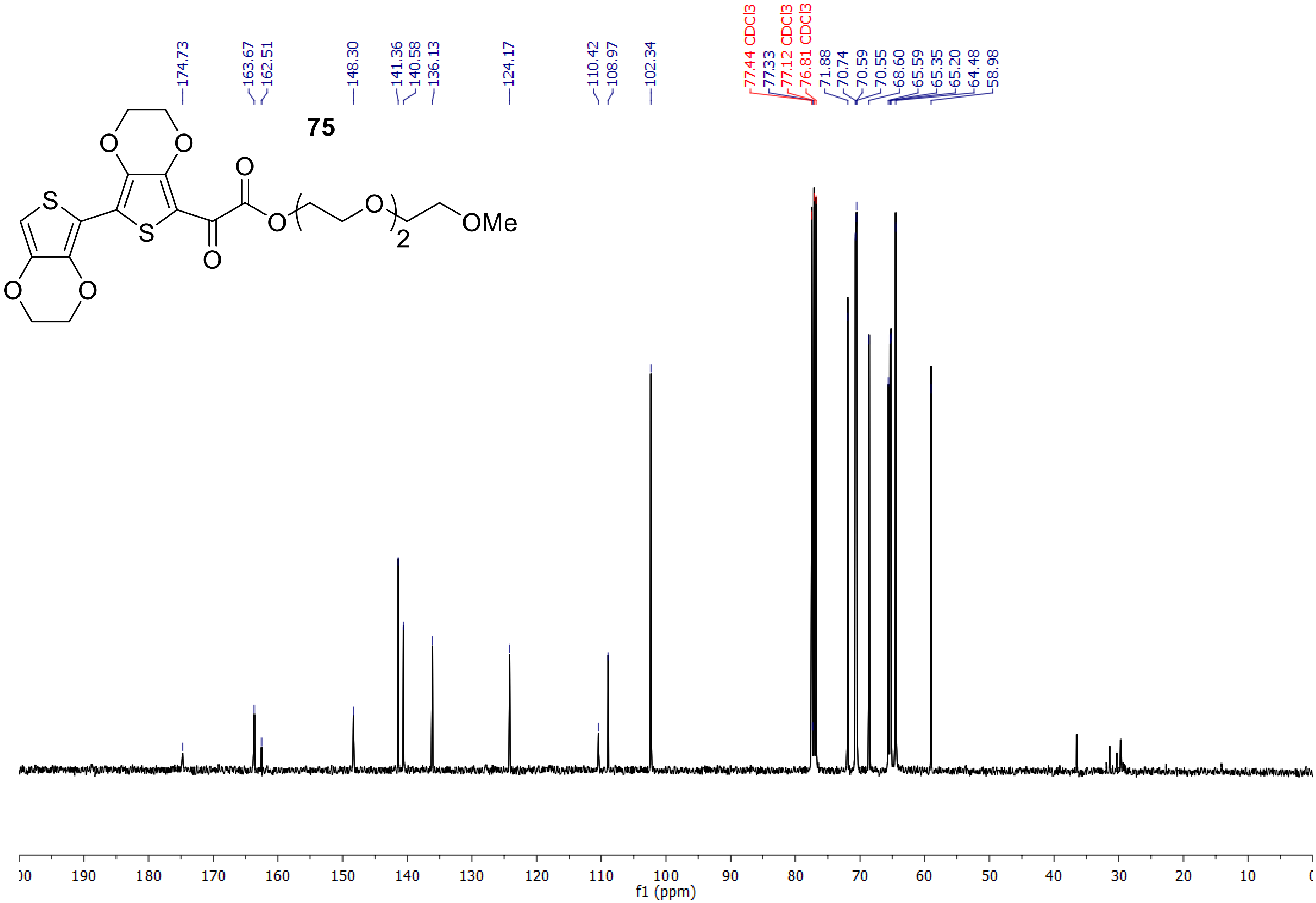

^1H NMR (400 MHz, $CDCl_3$)

Figure S109. ^1H NMR of **76**

76

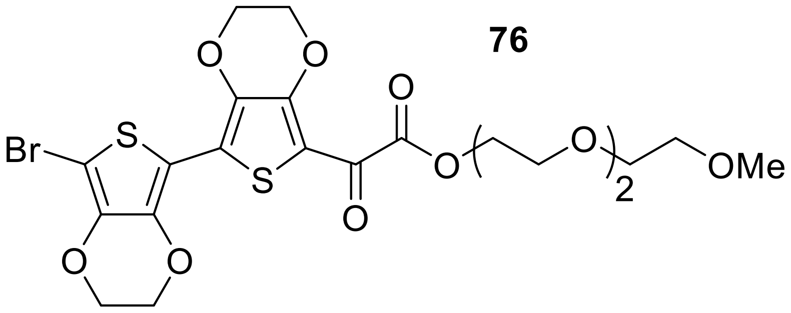

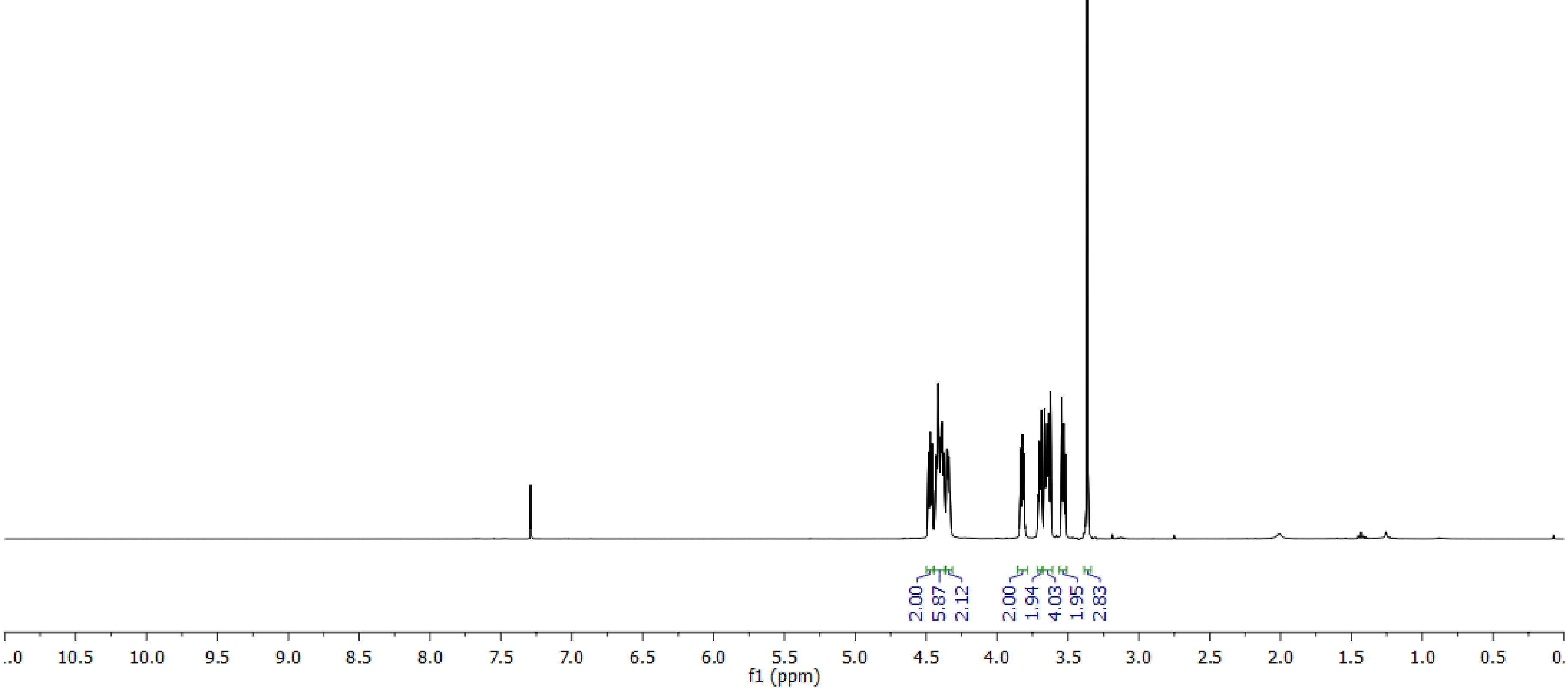

^{13}C NMR (100 MHz, $CDCl_3$)

Figure S110. ^{13}C NMR of **76**

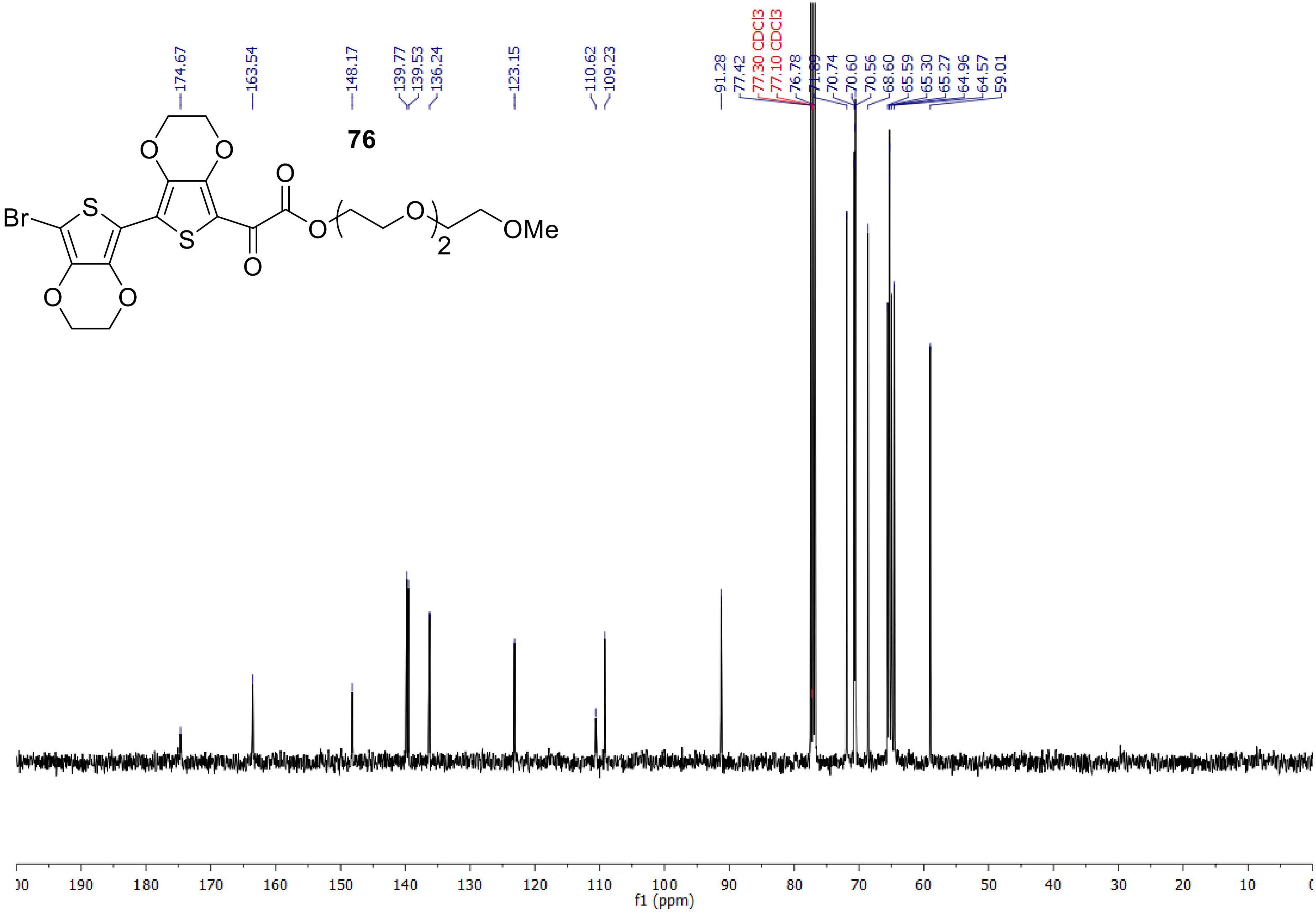

^{1}H NMR (400 MHz, DMSO-d$_6$)

Figure S111. ^{1}H NMR of **77**

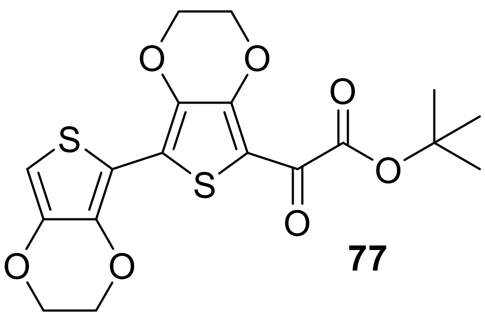

77

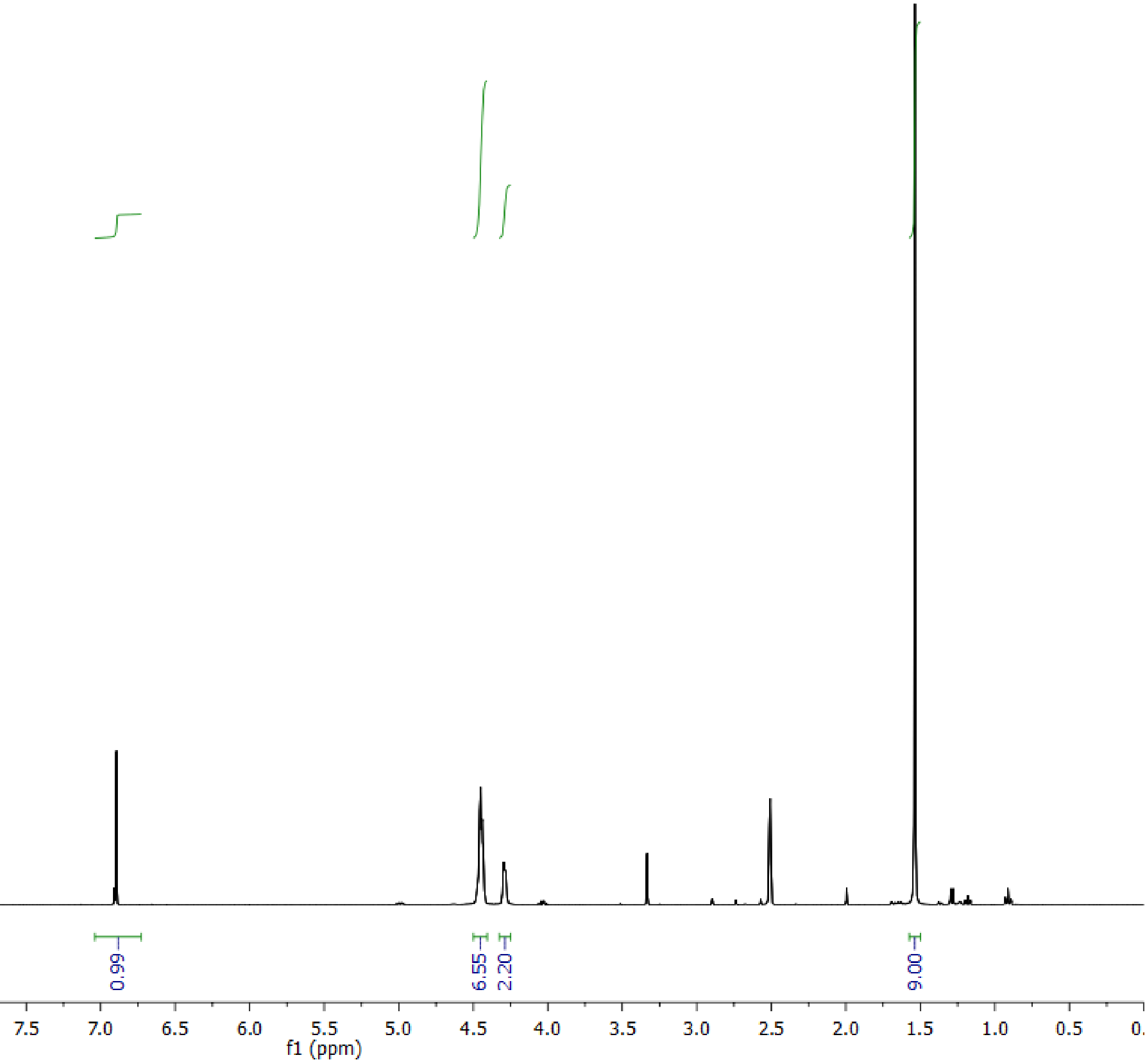

^{13}C NMR (100 MHz, DMSO-d$_6$)

Figure S112. ^{13}C NMR of **77**

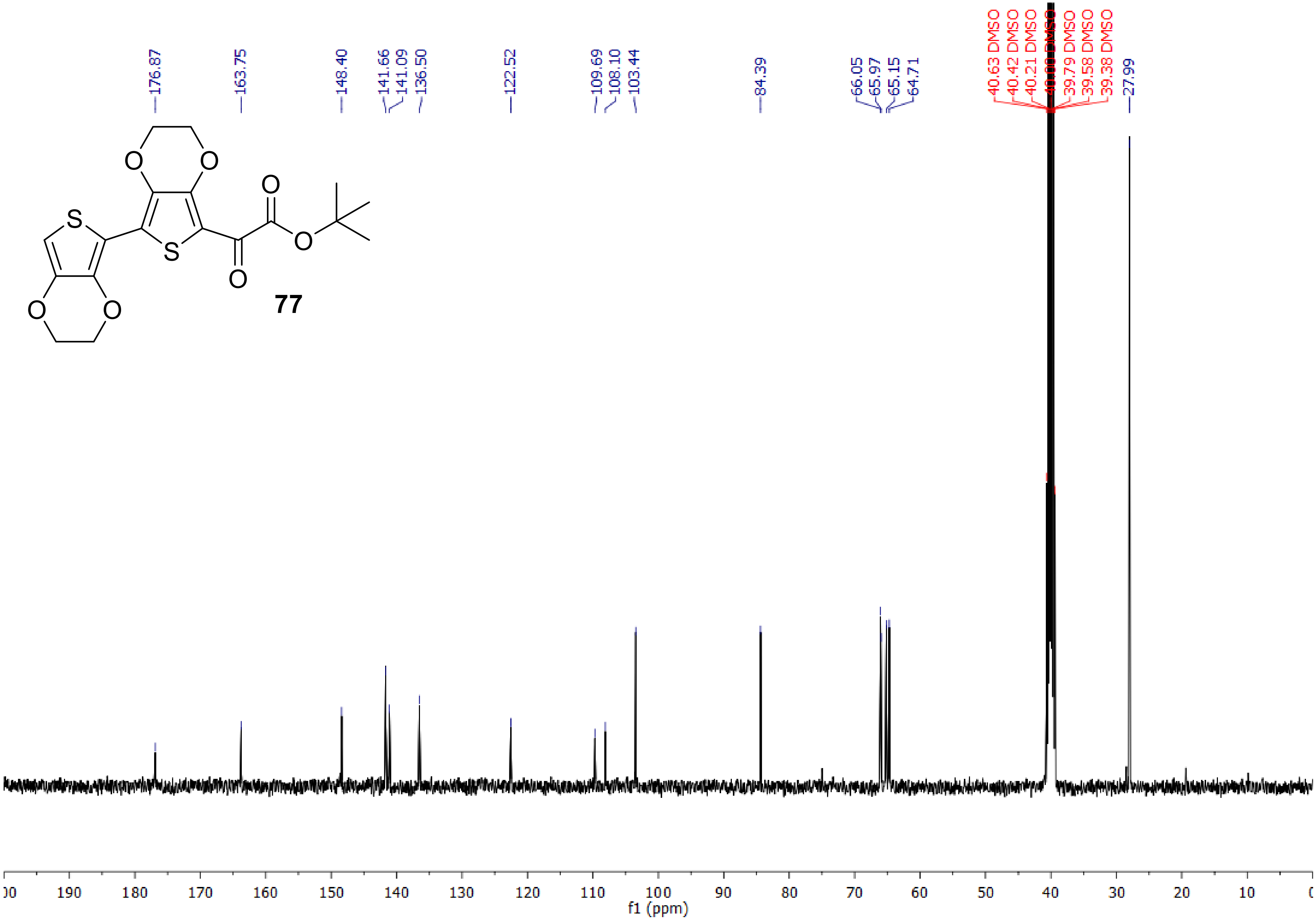

^{1}H NMR (400 MHz, $CDCl_3$)

Figure S113. ^{1}H NMR of **78**

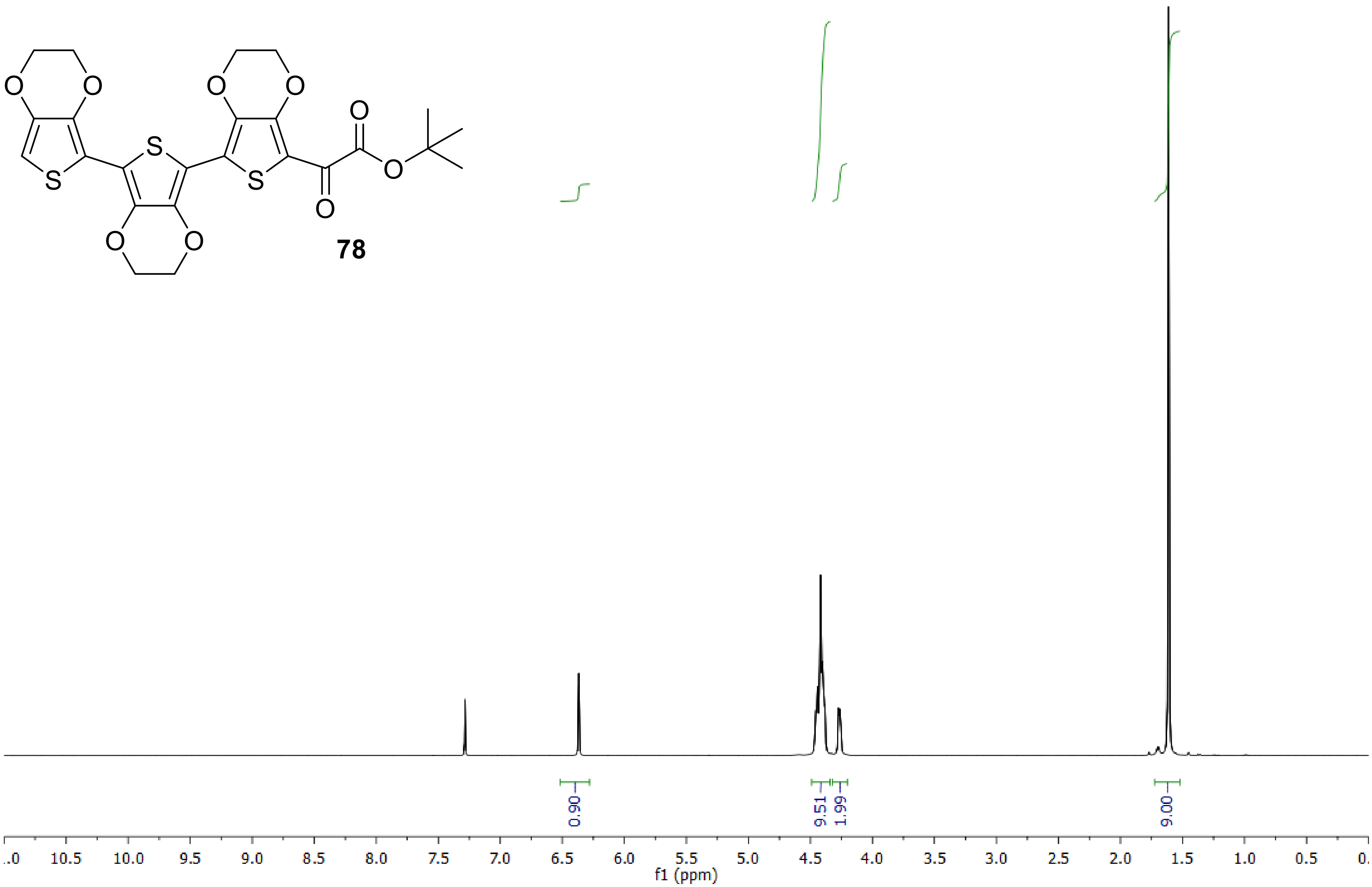

^{13}C NMR (100 MHz, $CDCl_3$)

Figure S114. ^{13}C NMR of **78**

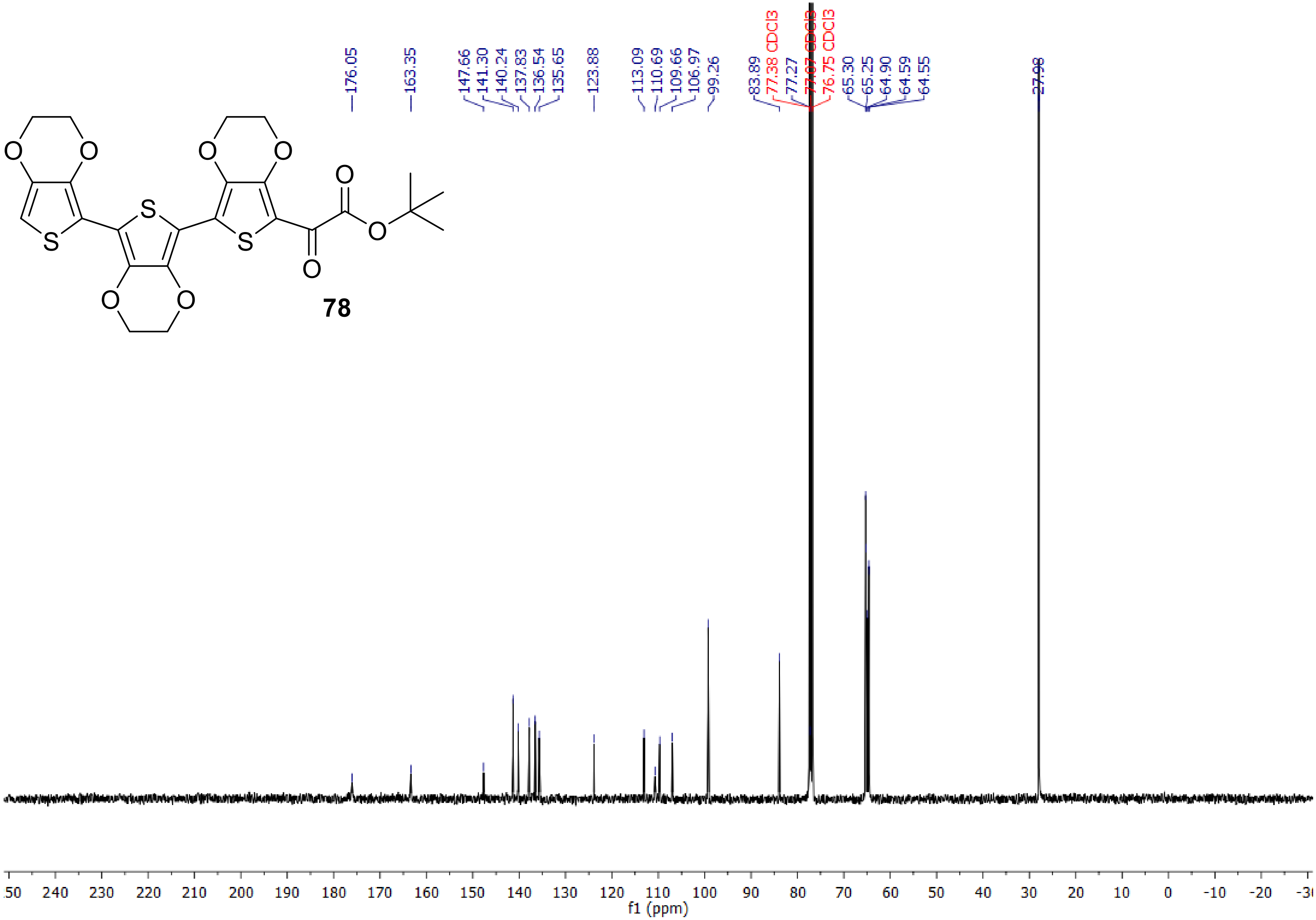

^{1}H NMR (400 MHz, $CDCl_3$)

Figure S115. ^{1}H NMR of **80**

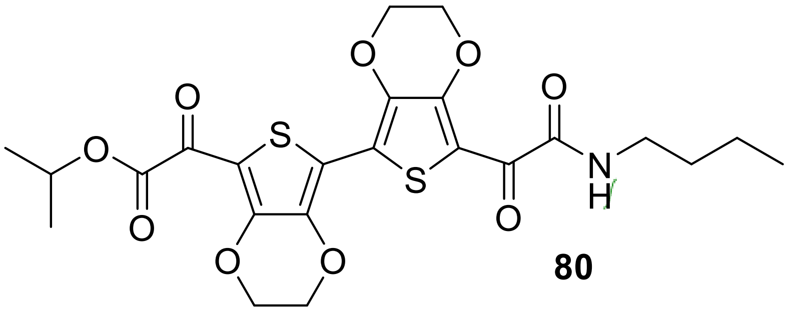

80

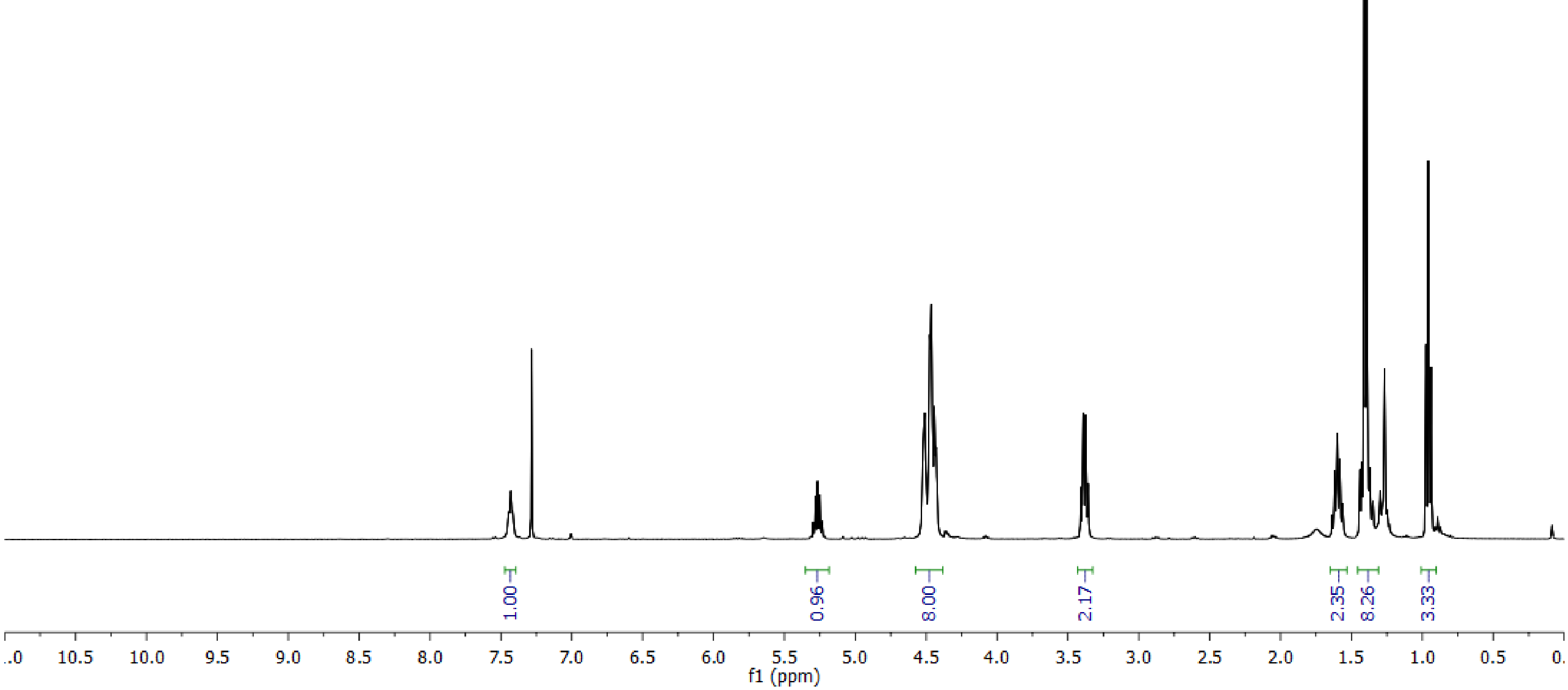

^{13}C NMR (100 MHz, $CDCl_3$)

Figure S116. ^{13}C NMR of **80**

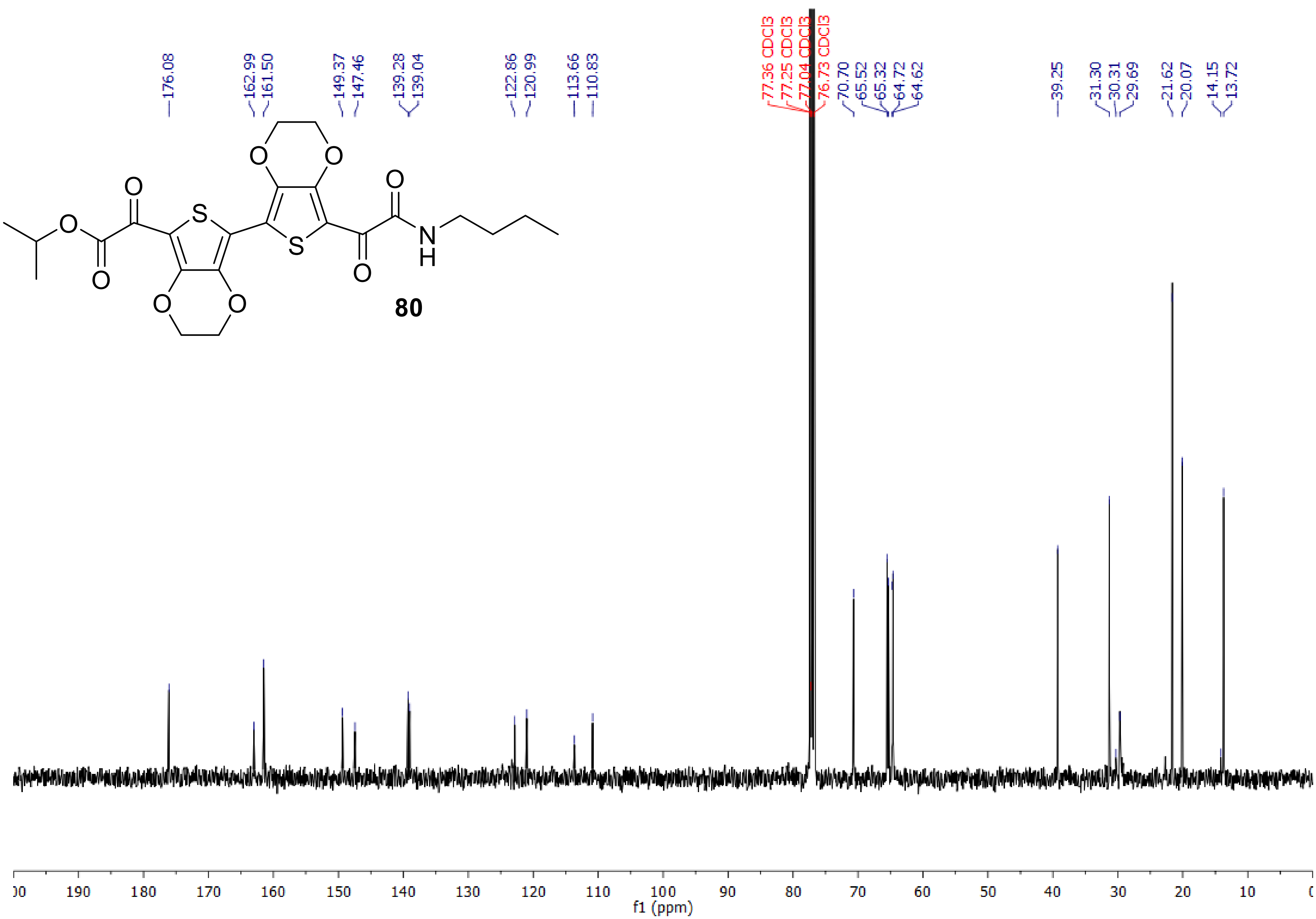

^{1}H NMR (400 MHz, $CDCl_3$)

Figure S117. ^{1}H NMR of **84**

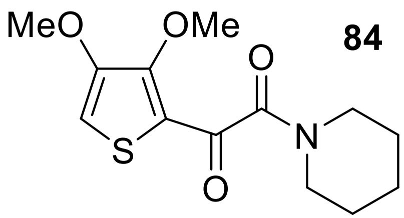

84

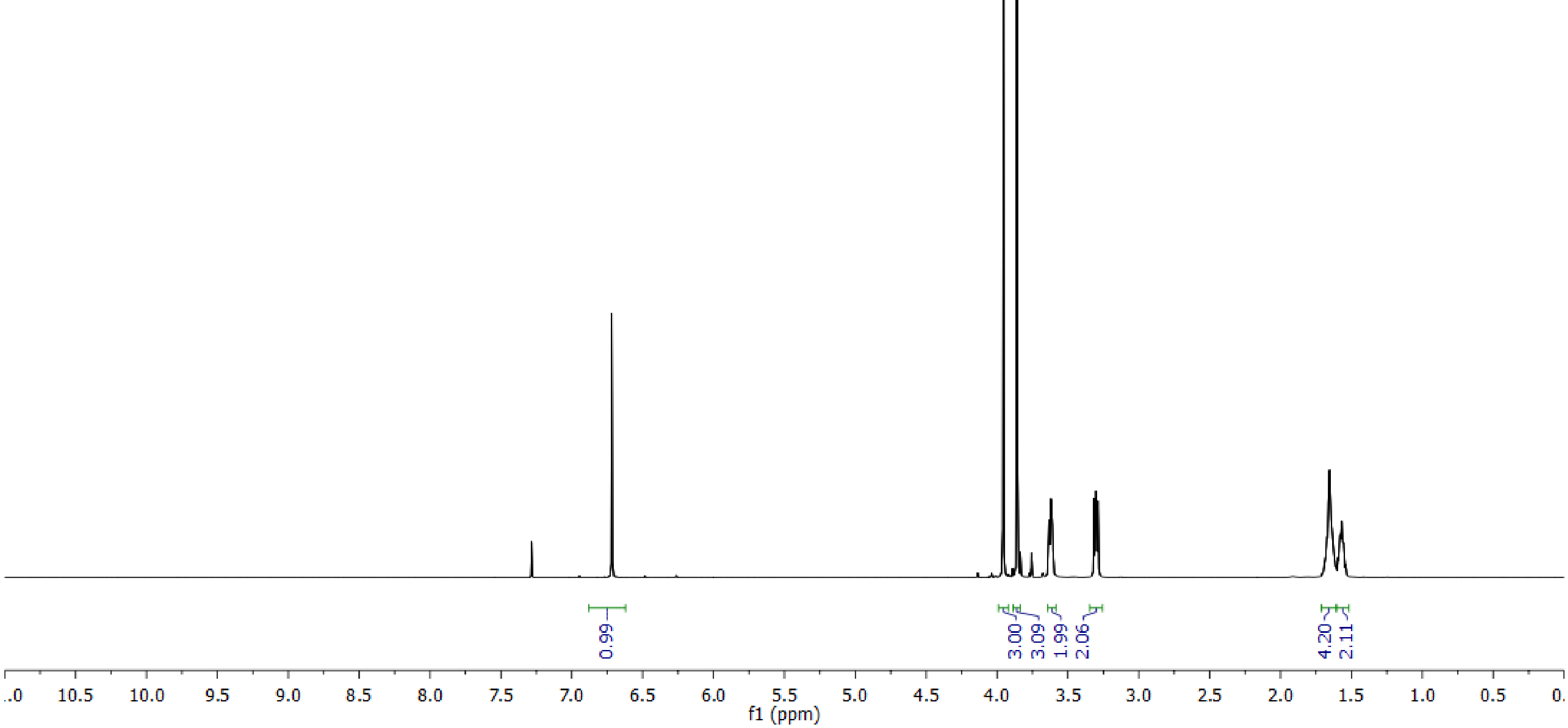

^{13}C NMR (100 MHz, $CDCl_3$)

Figure S118. ^{13}C NMR of **84**

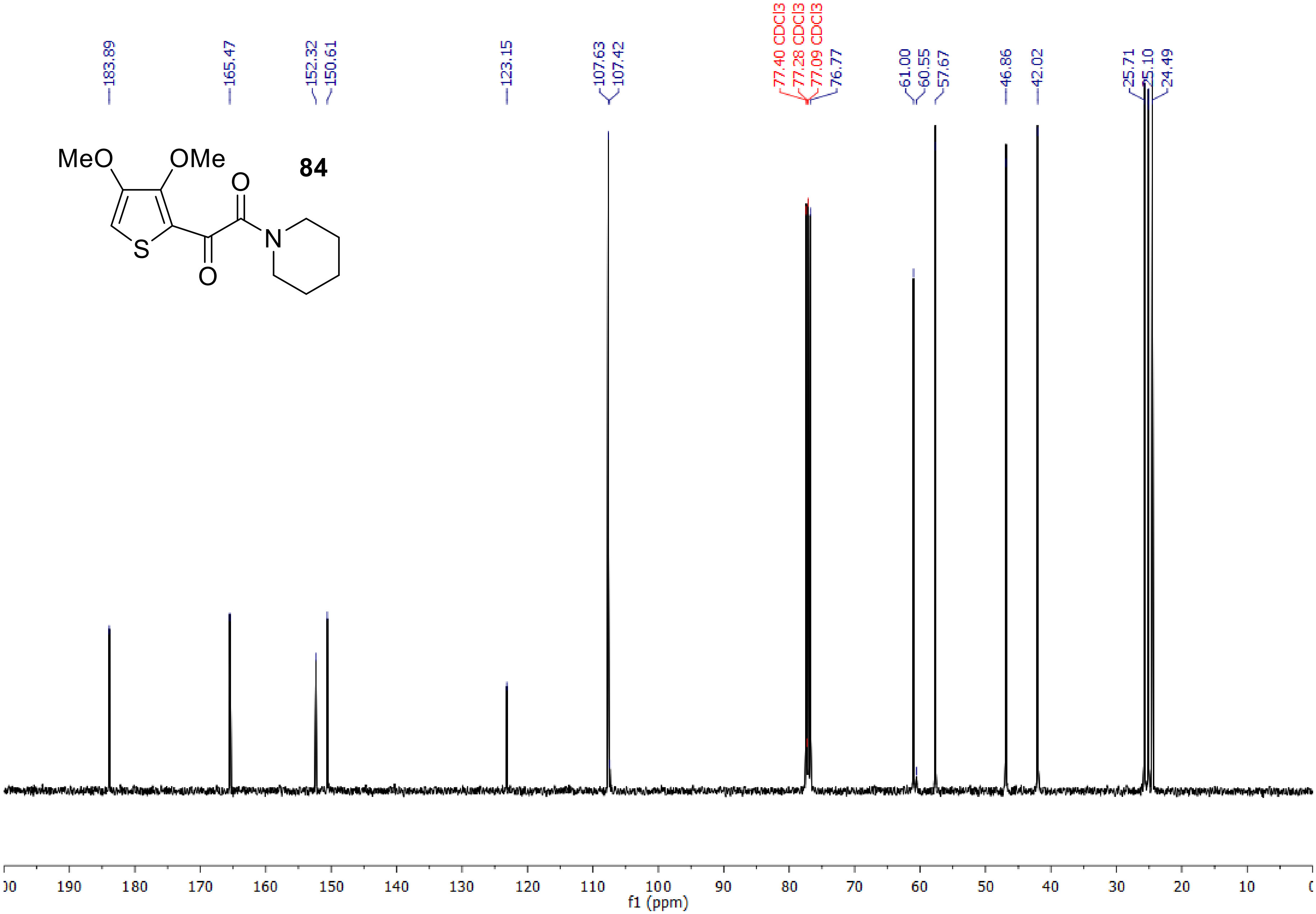

^{1}H NMR (400 MHz, $CDCl_3$)

Figure S119. ^{1}H NMR of **85**

85

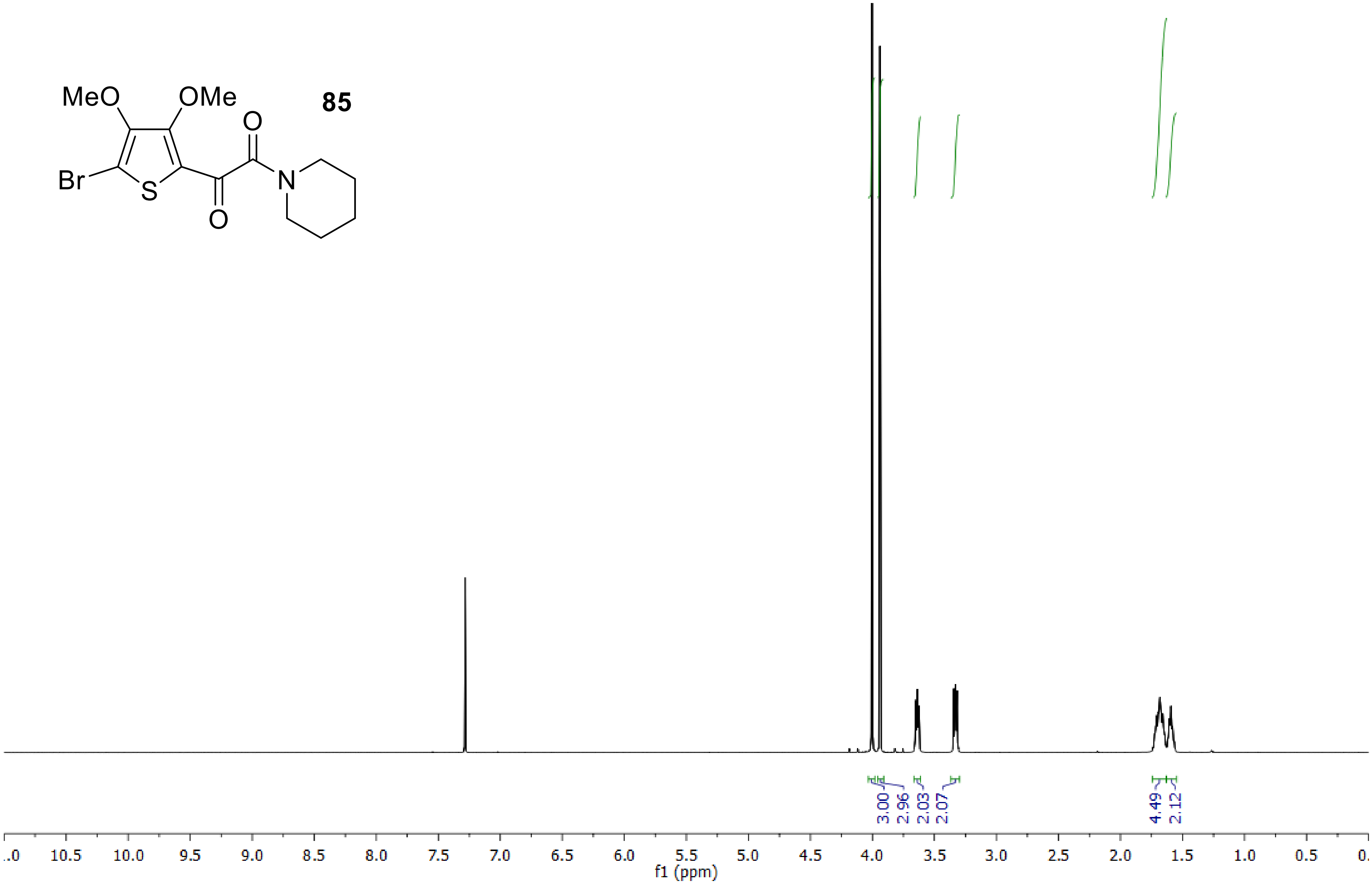

^{13}C NMR (100 MHz, $CDCl_3$)

Figure S120. ^{13}C NMR of **85**

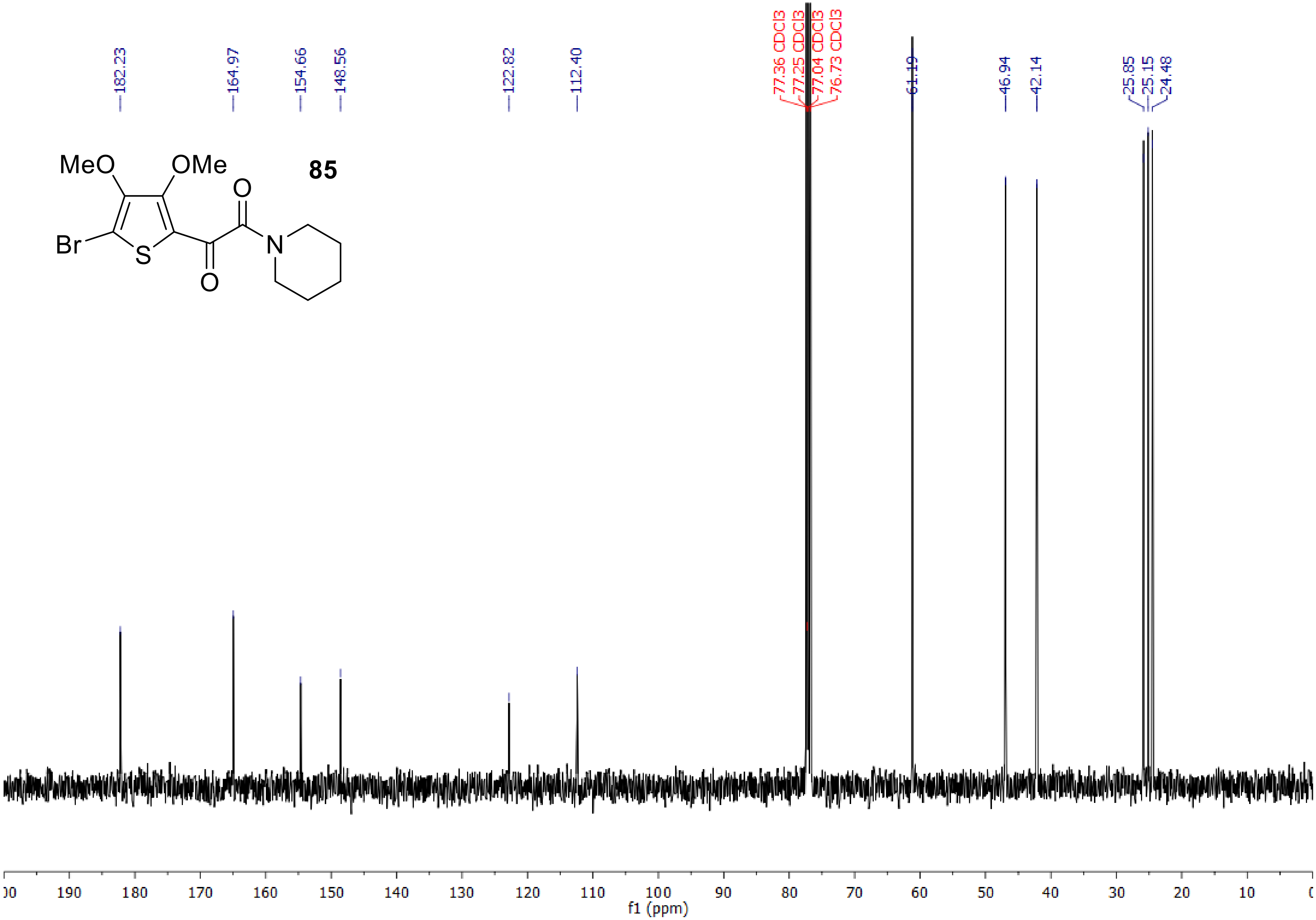

^{1}H NMR (400 MHz, $CDCl_3$)

Figure S121. ^{1}H NMR of **86**

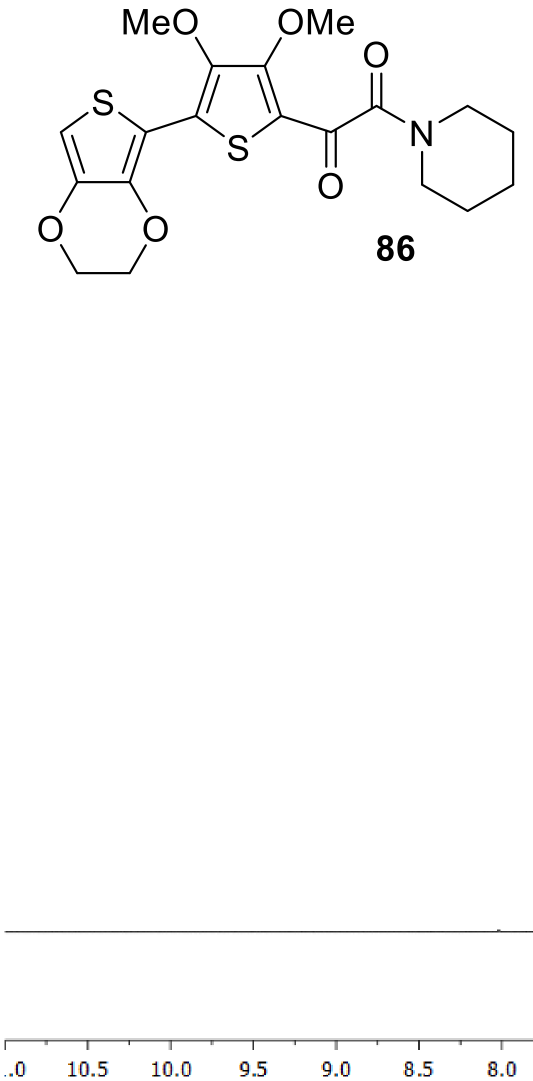

86

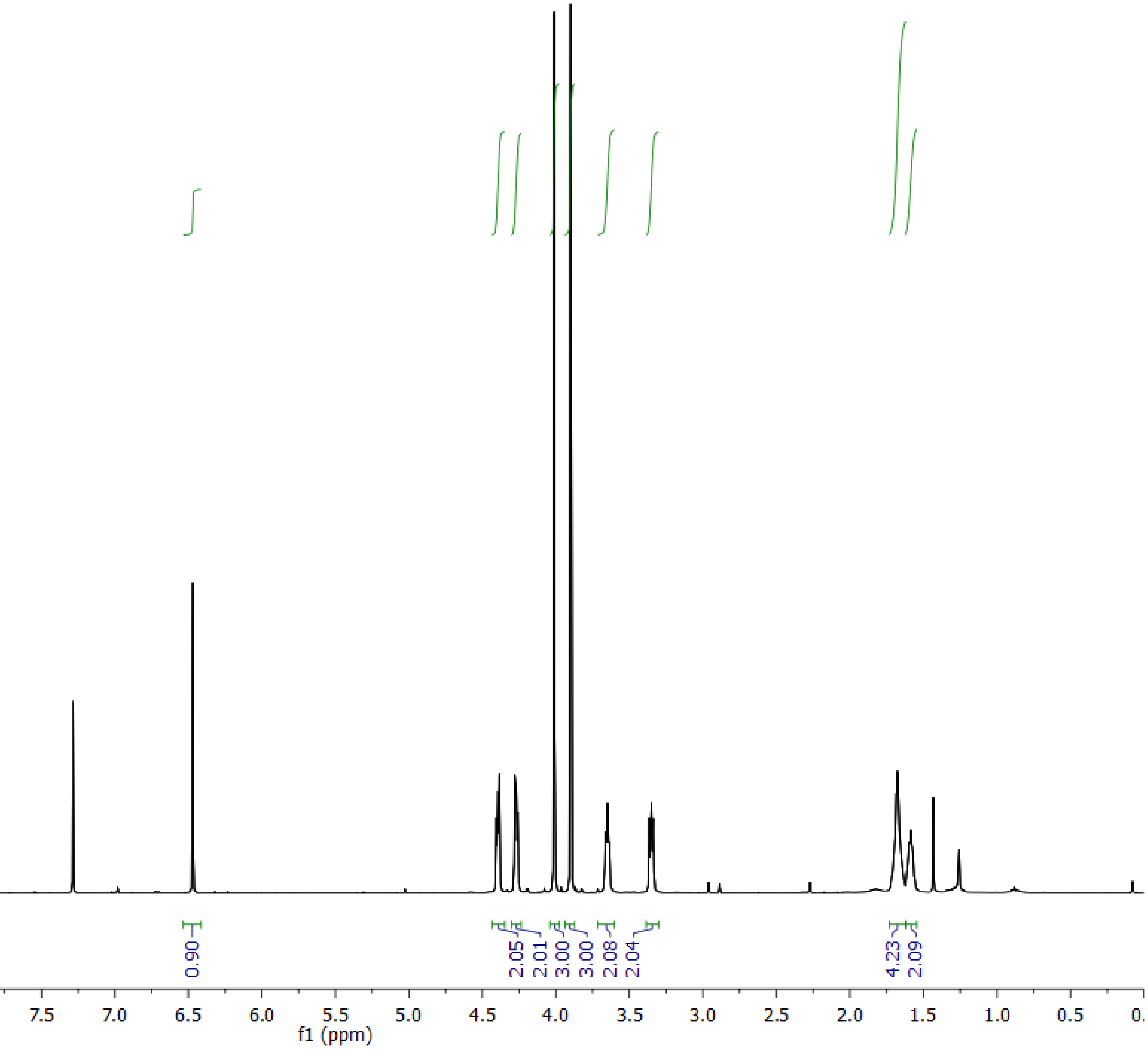

^{13}C NMR (100 MHz, $CDCl_3$)

Figure S122. ^{13}C NMR of **86**

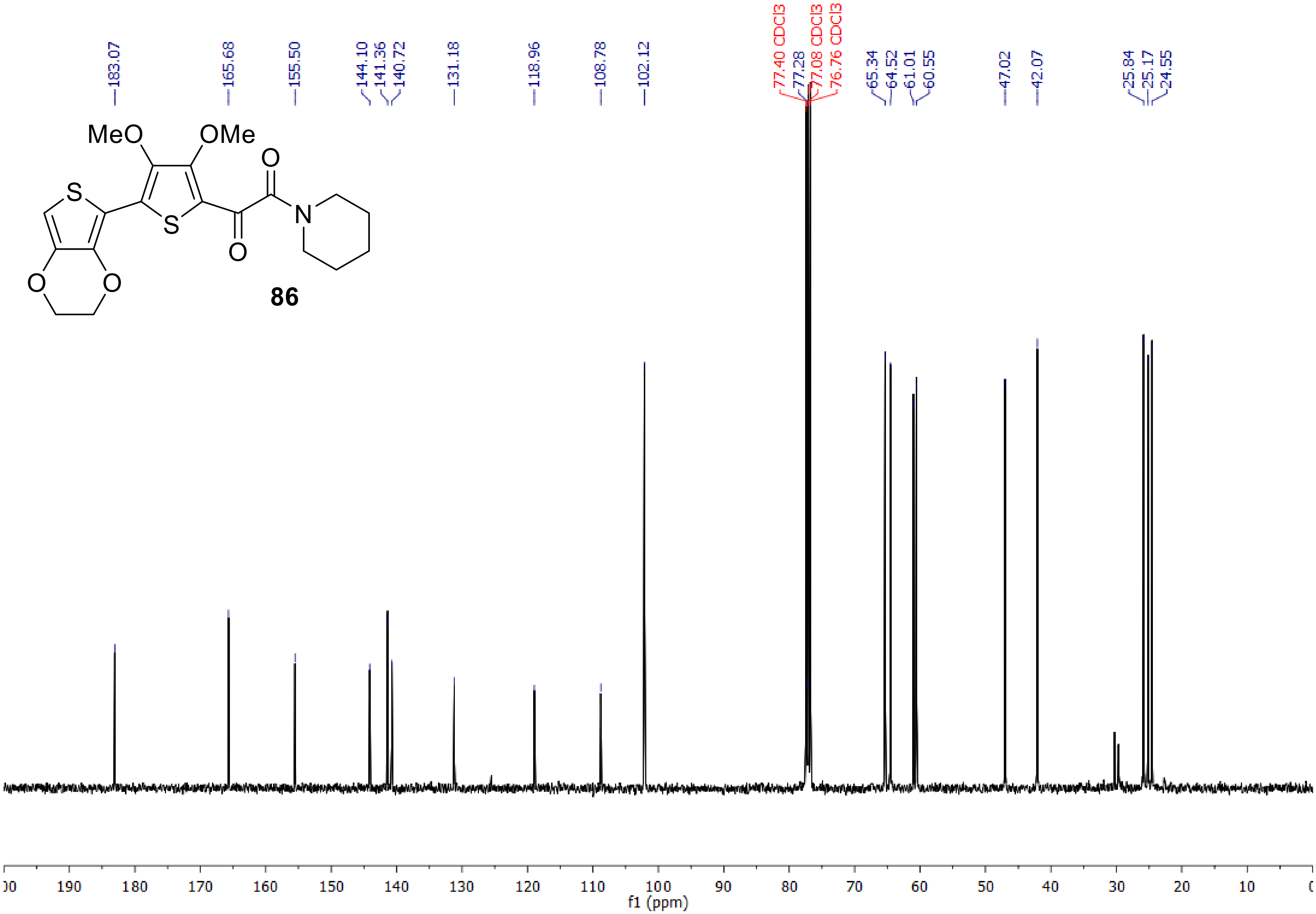

^{1}H NMR (400 MHz, $CDCl_3$)

Figure S123. ^{1}H NMR of **87**

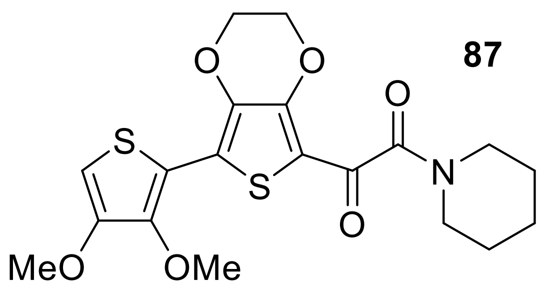

87

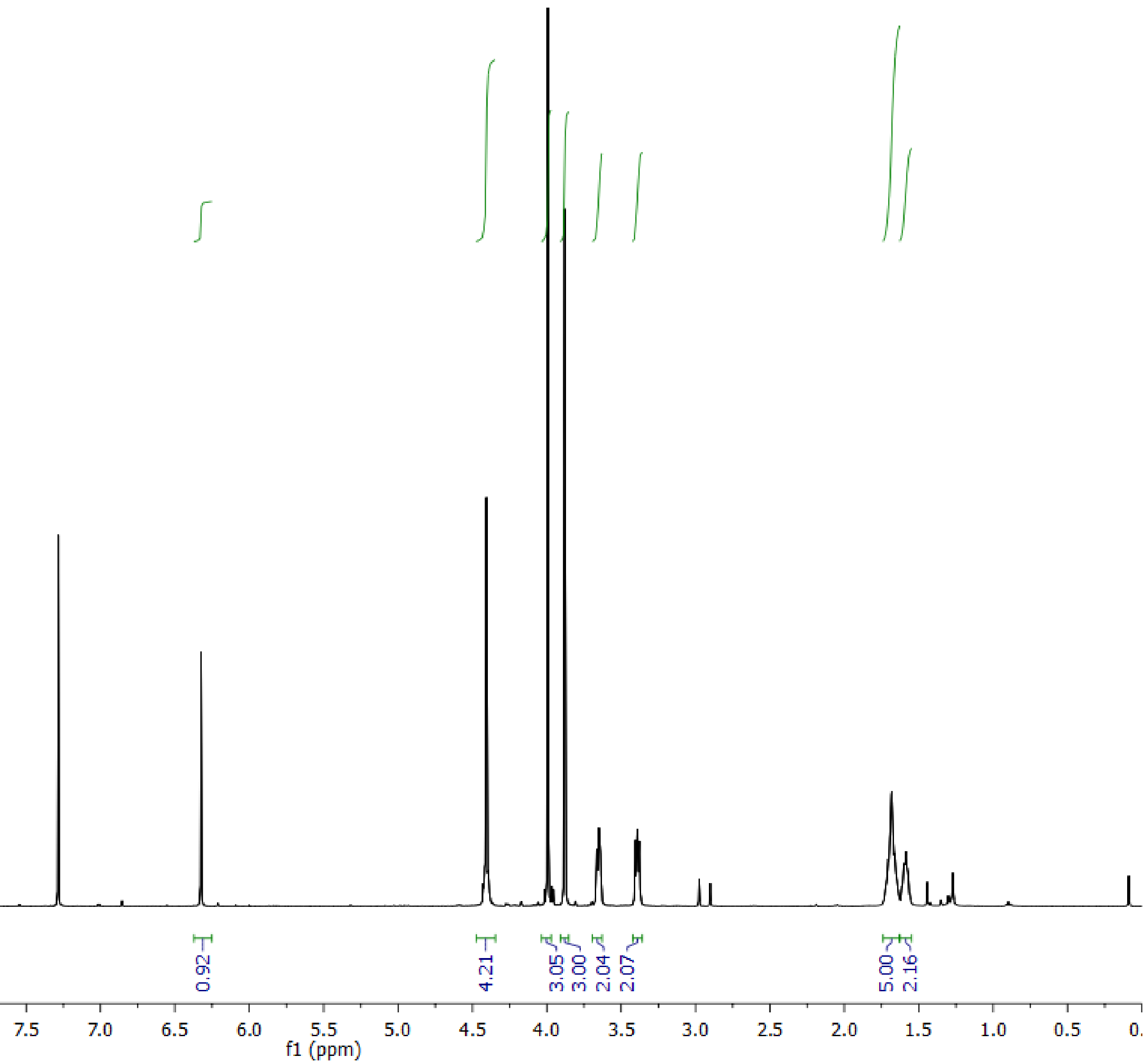

^{13}C NMR (100 MHz, $CDCl_3$)

Figure S124. ^{13}C NMR of **87**

87

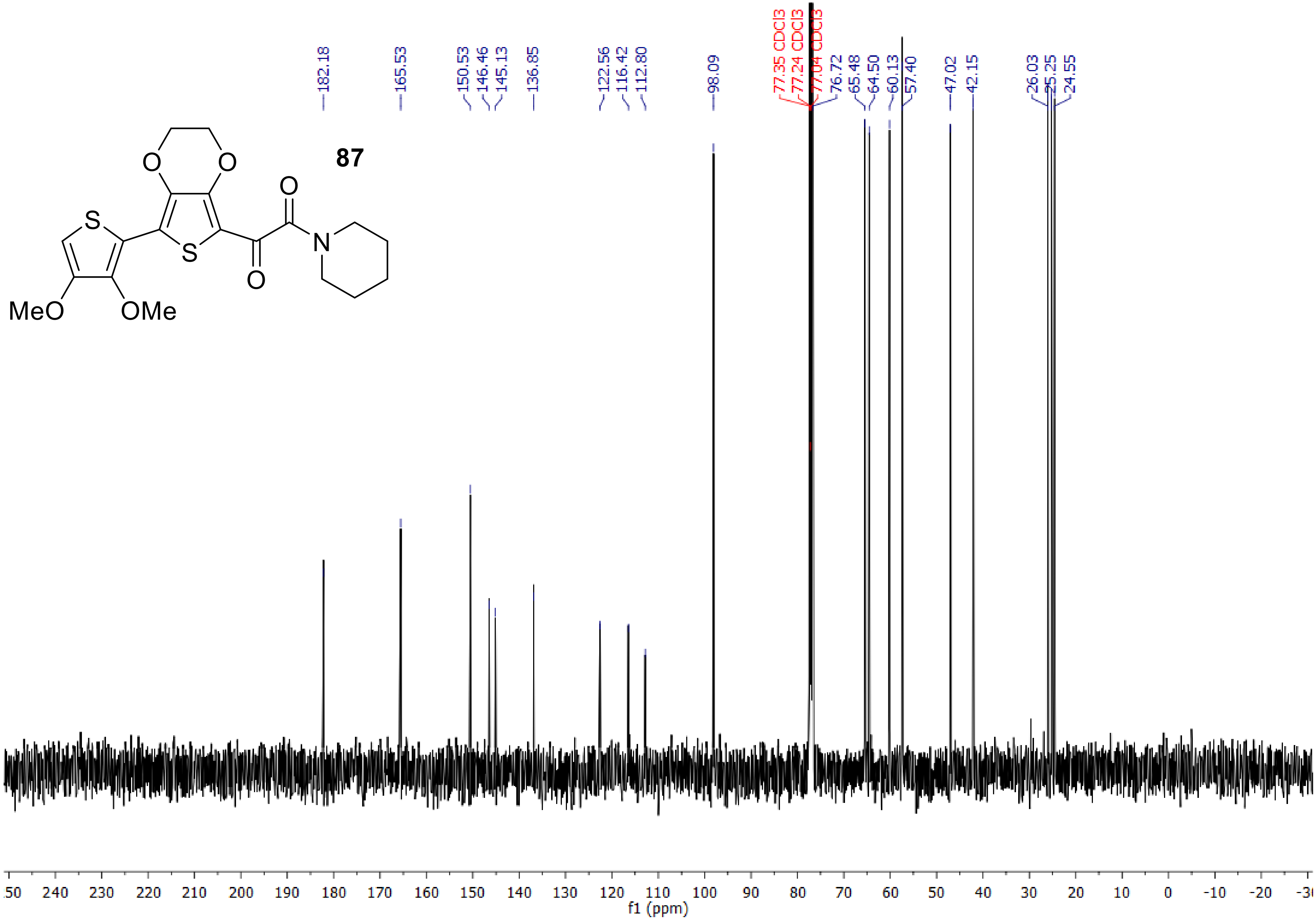

^{1}H NMR (400 MHz, $CDCl_3$)

Figure S125. ^{1}H NMR of **90**

90

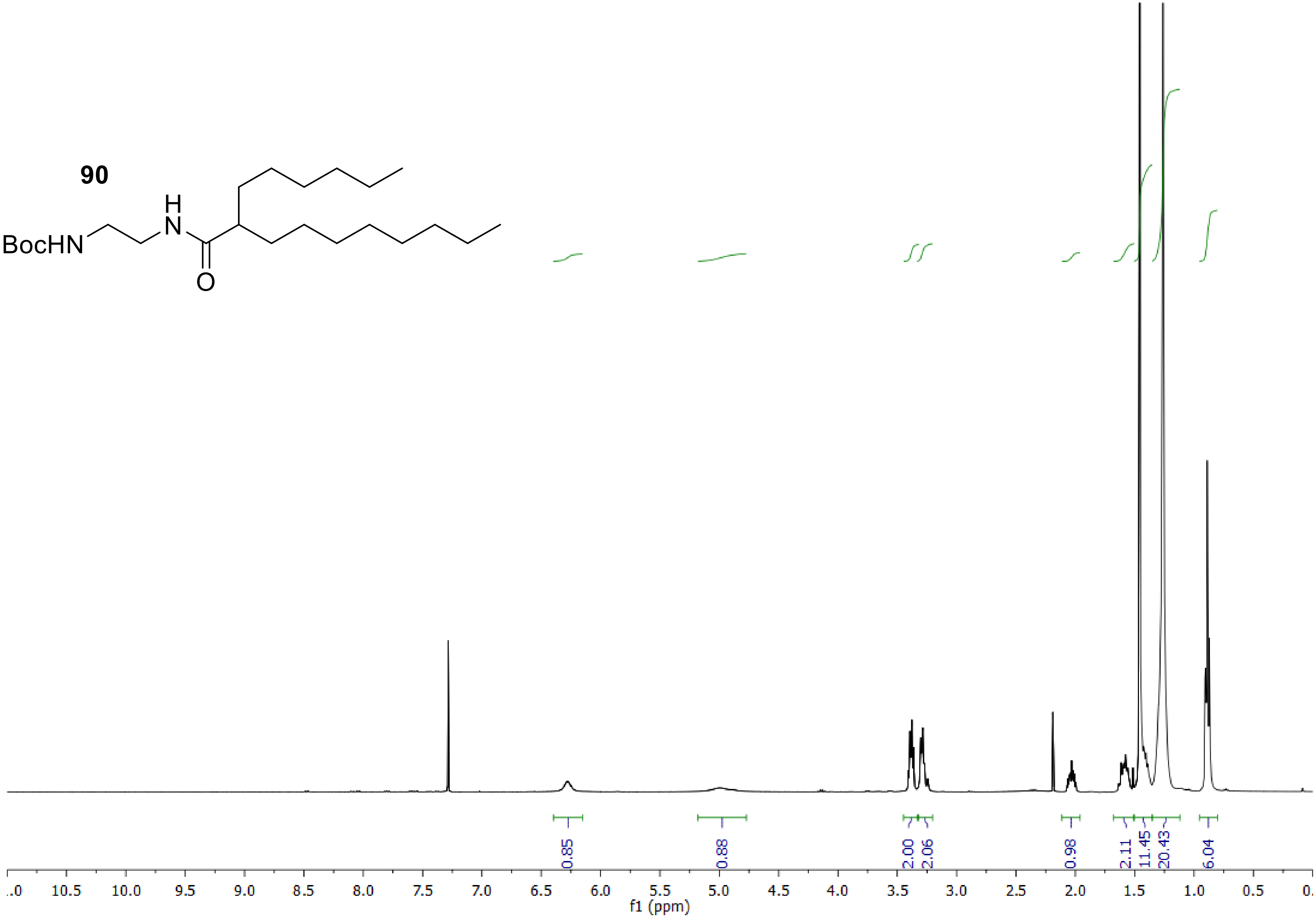

^{13}C NMR (100 MHz, $CDCl_3$)

Figure S126. ^{13}C NMR of **90**

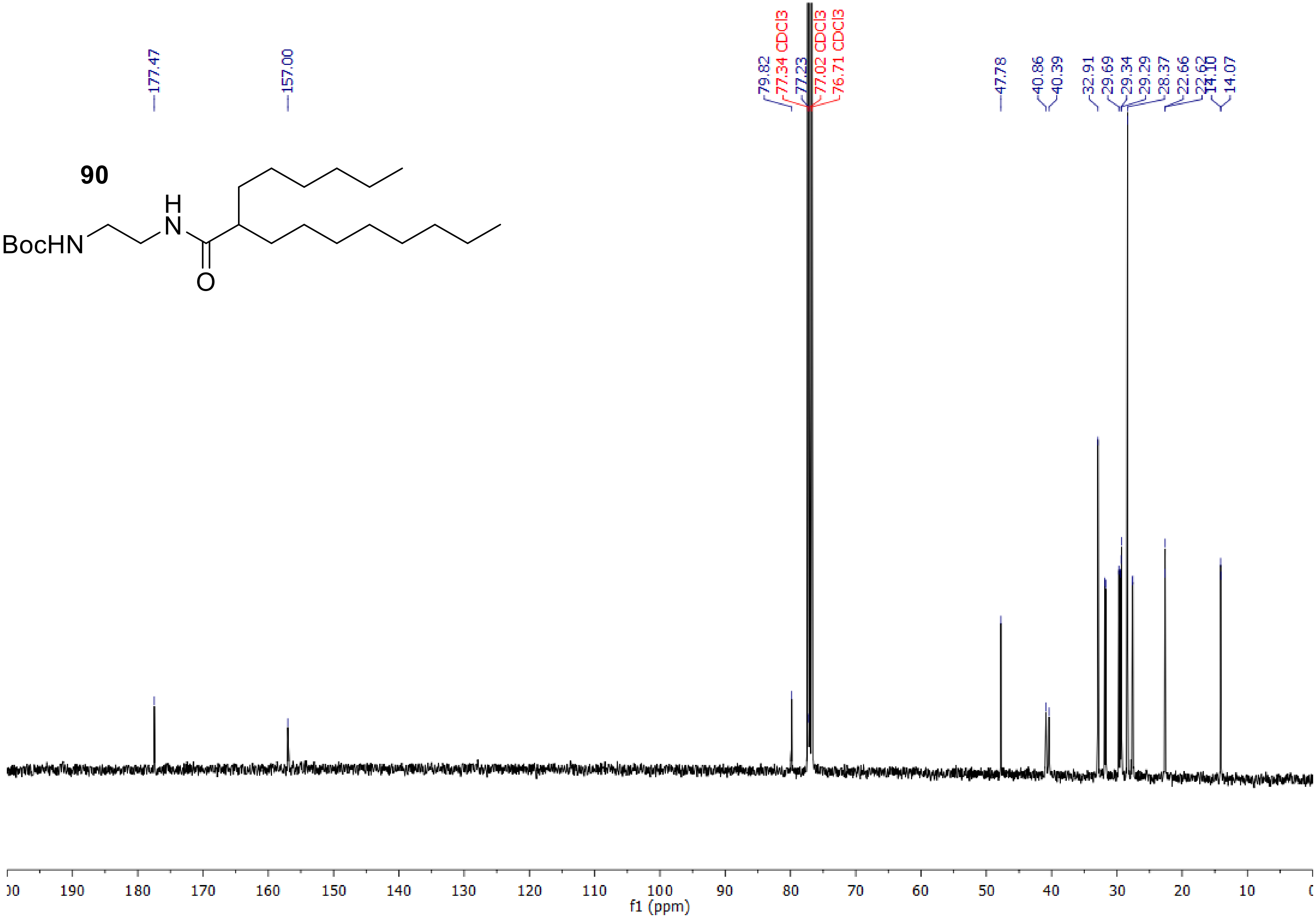

^1H NMR (400 MHz, $CDCl_3$)

Figure S127. ^1H NMR of **91**

91

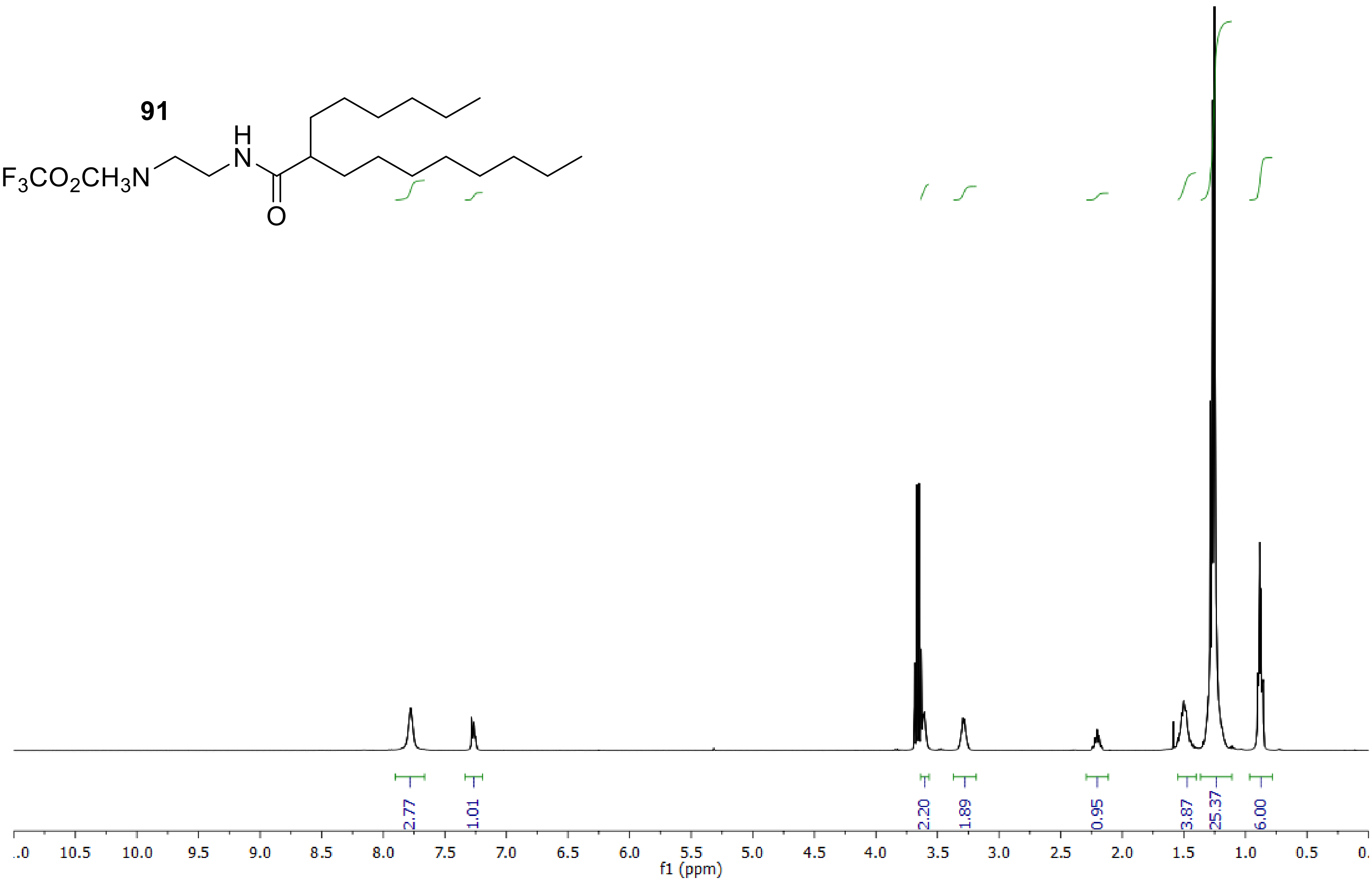

^{13}C NMR (100 MHz, $CDCl_3$)

Figure S128. ^{13}C NMR of **91**

91

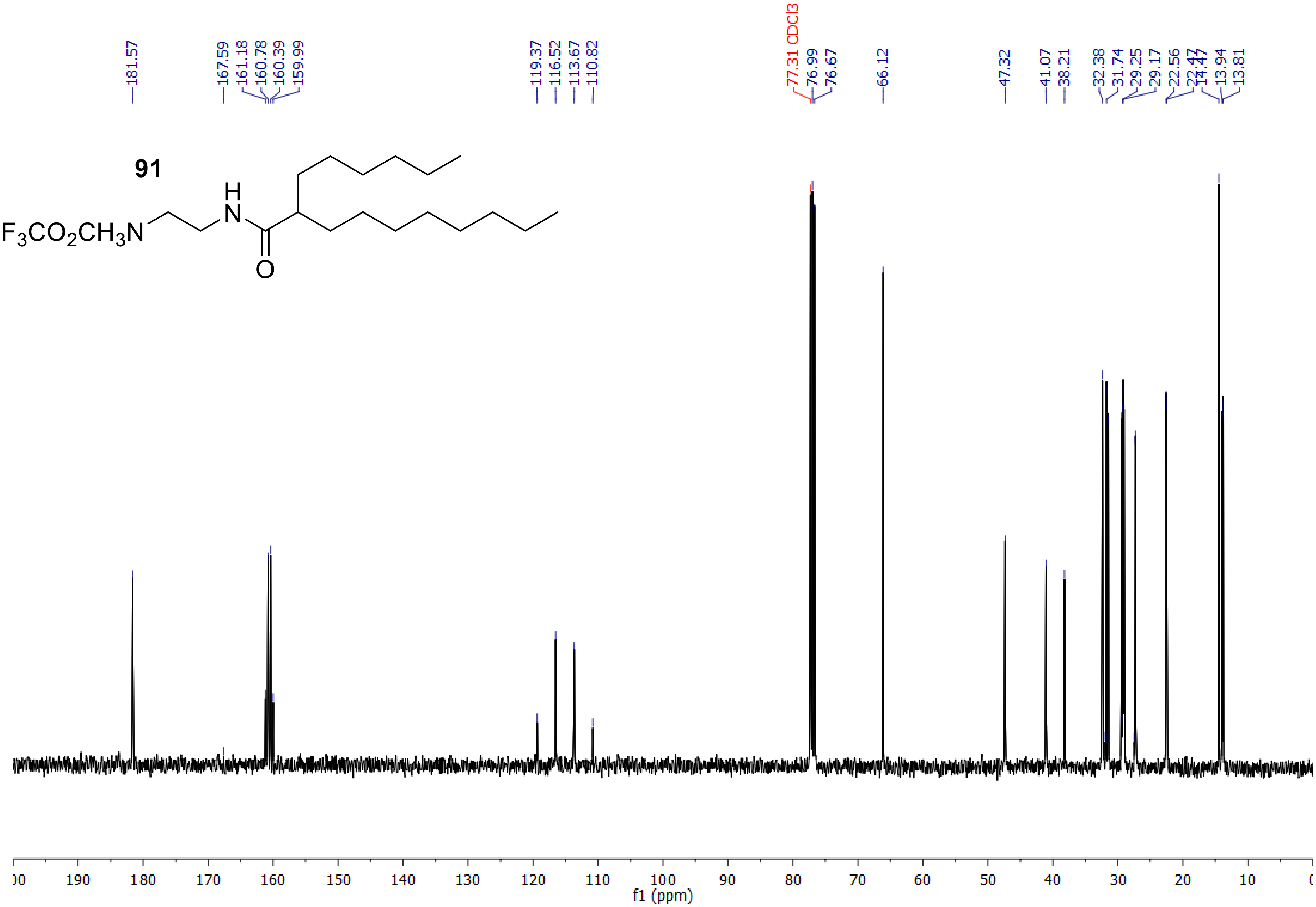

^{1}H NMR (400 MHz, $CDCl_3$)

Figure S129. ^{1}H NMR of **98**

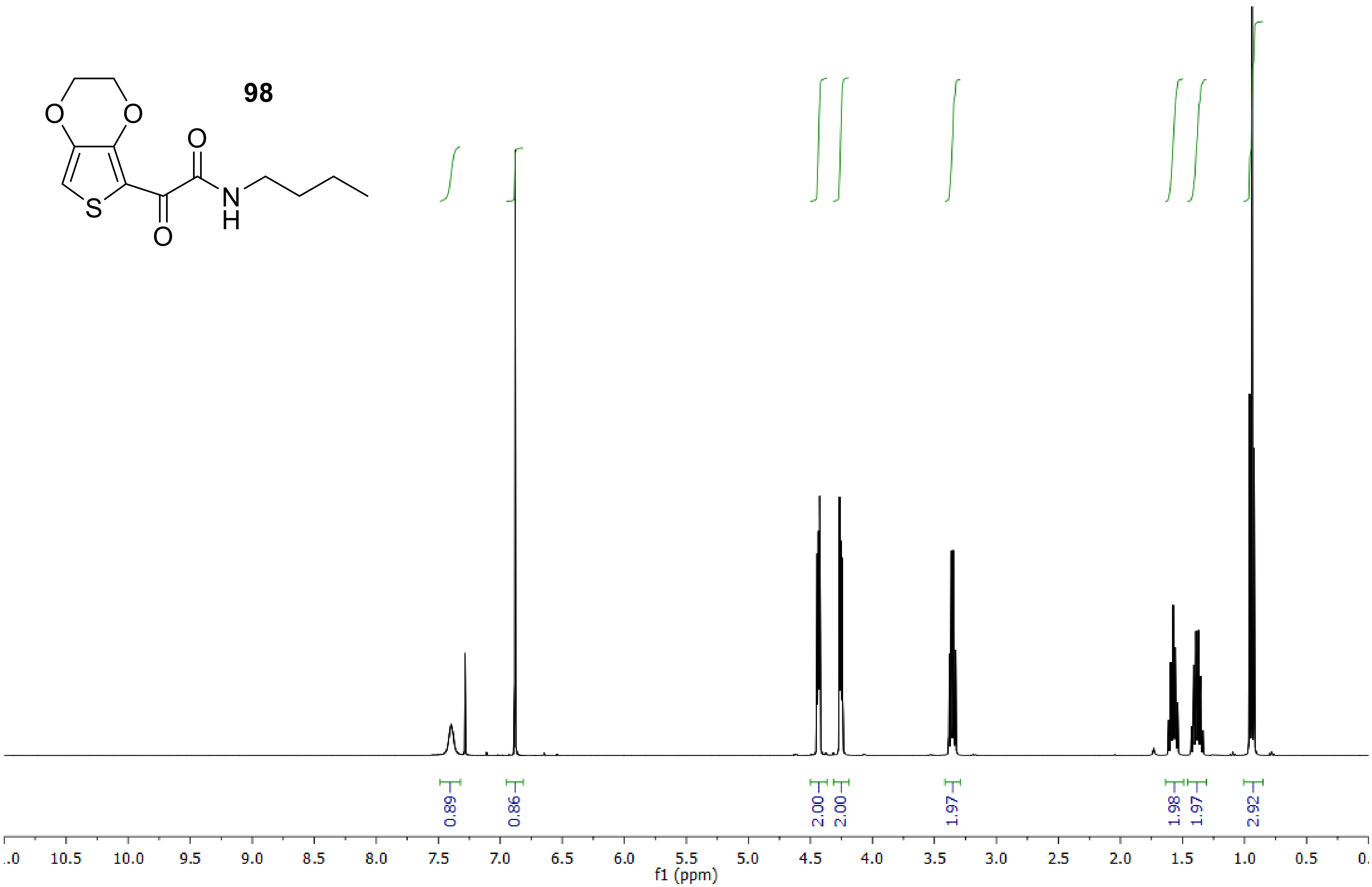

^{13}C NMR (100 MHz, $CDCl_3$)

Figure S130. ^{13}C NMR of **98**

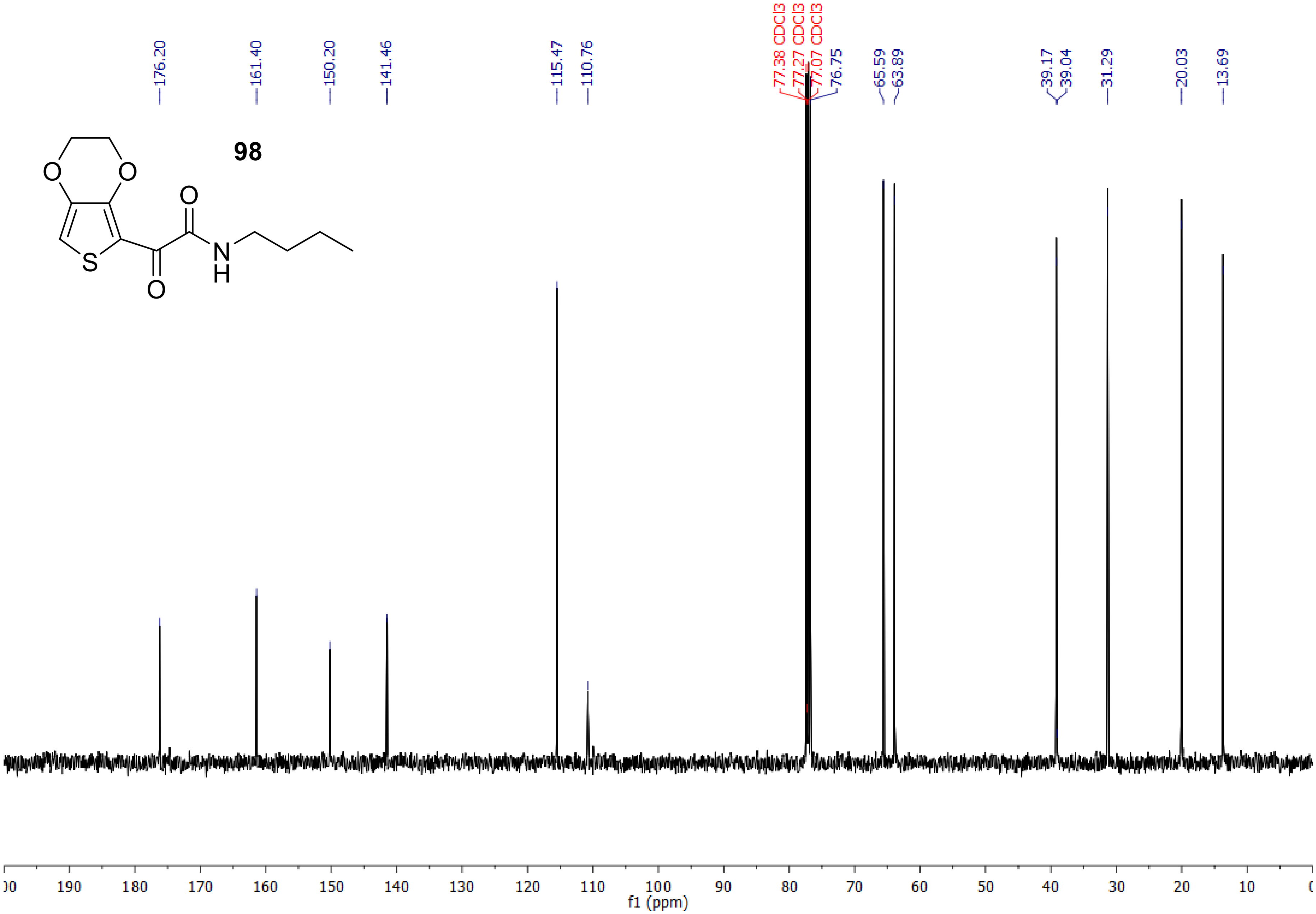

^{1}H NMR (400 MHz, $CDCl_3$)

Figure S131. ^{1}H NMR of **99**

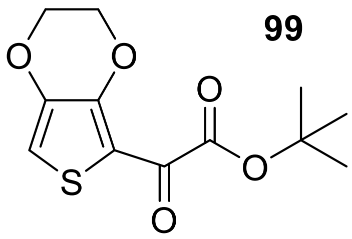

99

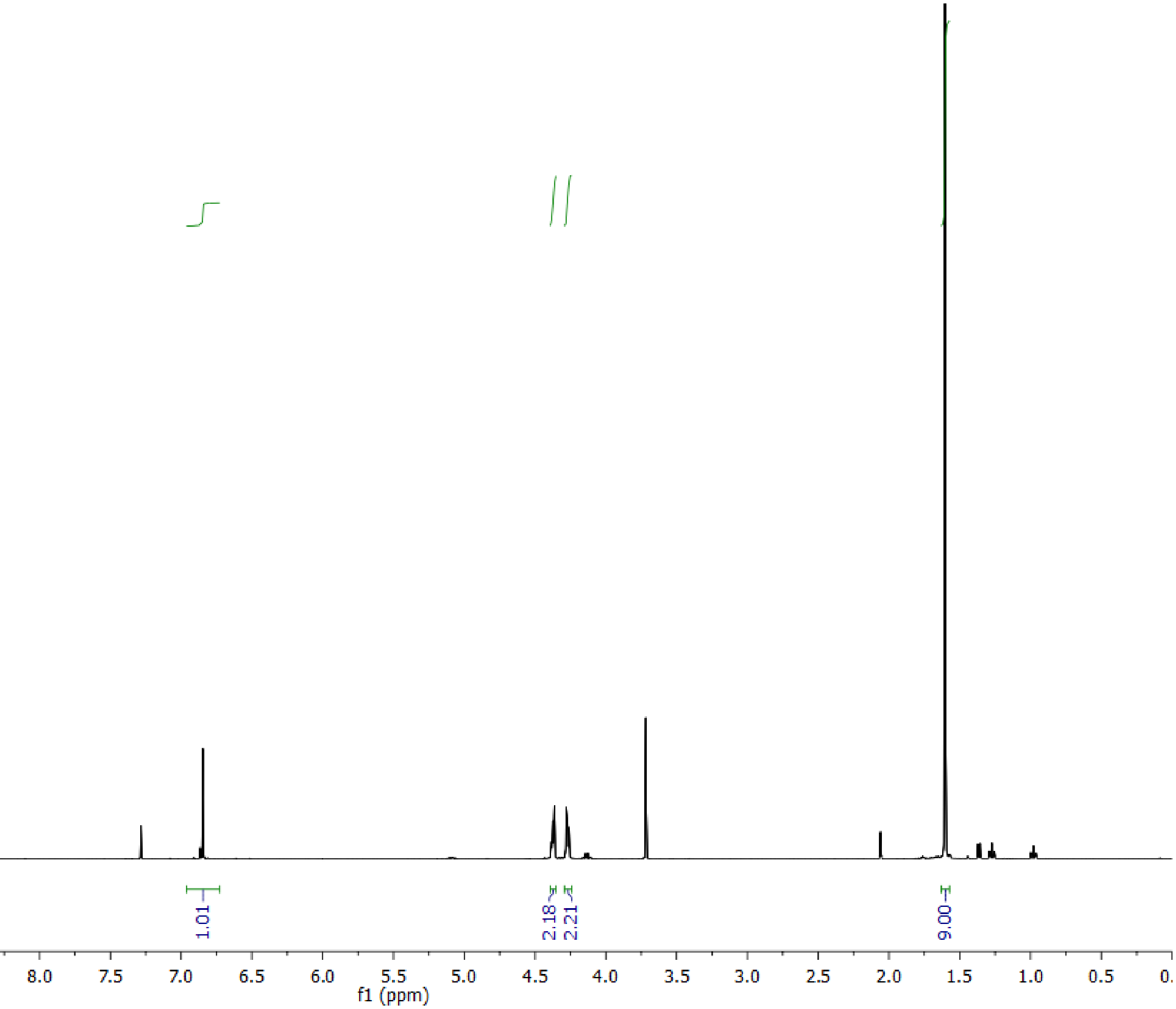

^{13}C NMR (100 MHz, $CDCl_3$)

Figure S132. ^{13}C NMR of **99**

99

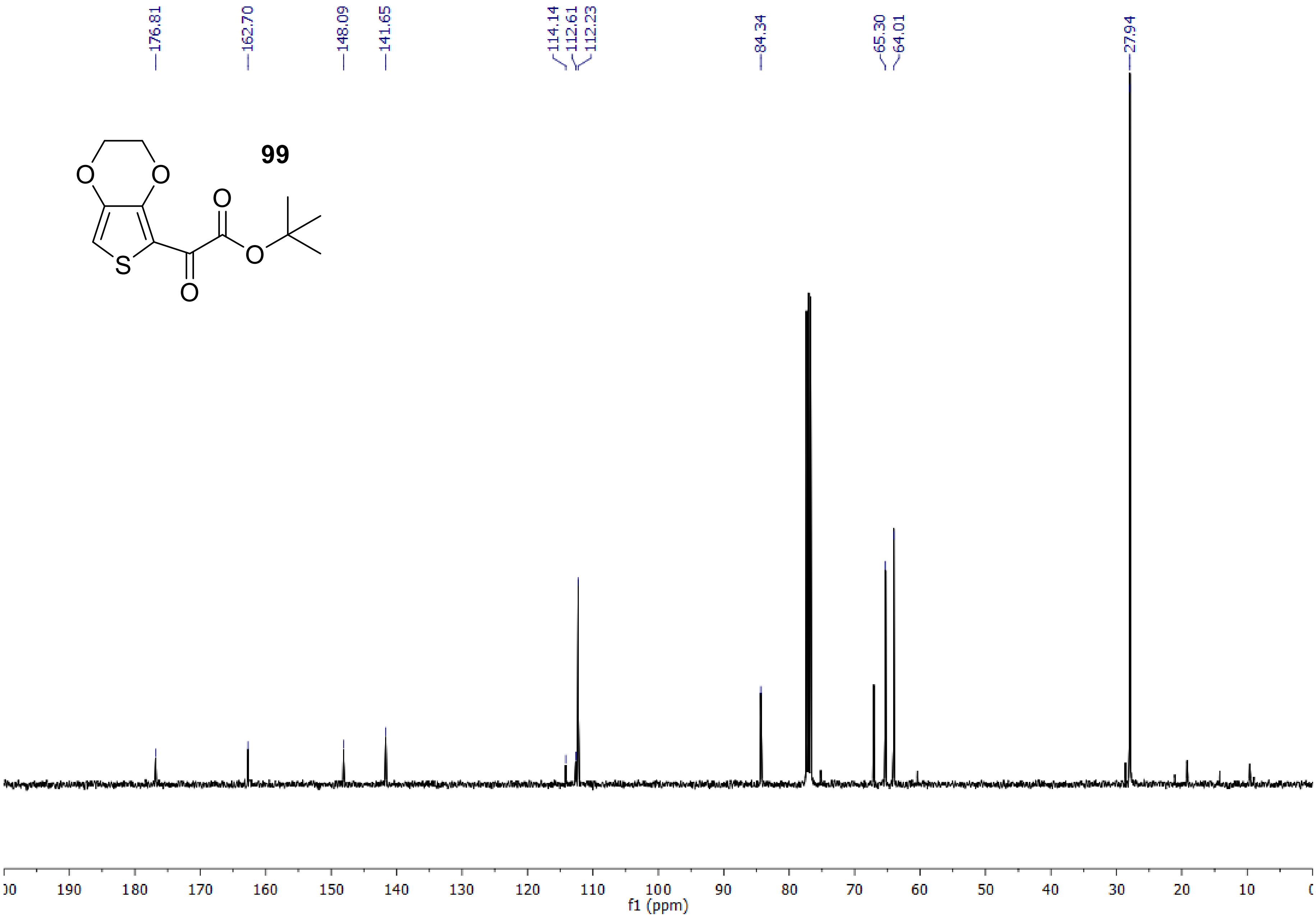

^{1}H NMR (400 MHz, $CDCl_3$)

Figure S133. ^{1}H NMR of **100**

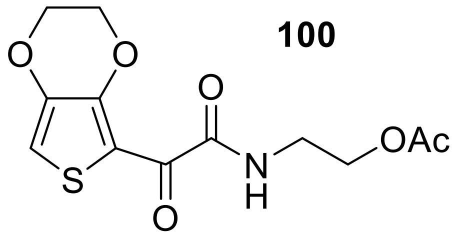

100

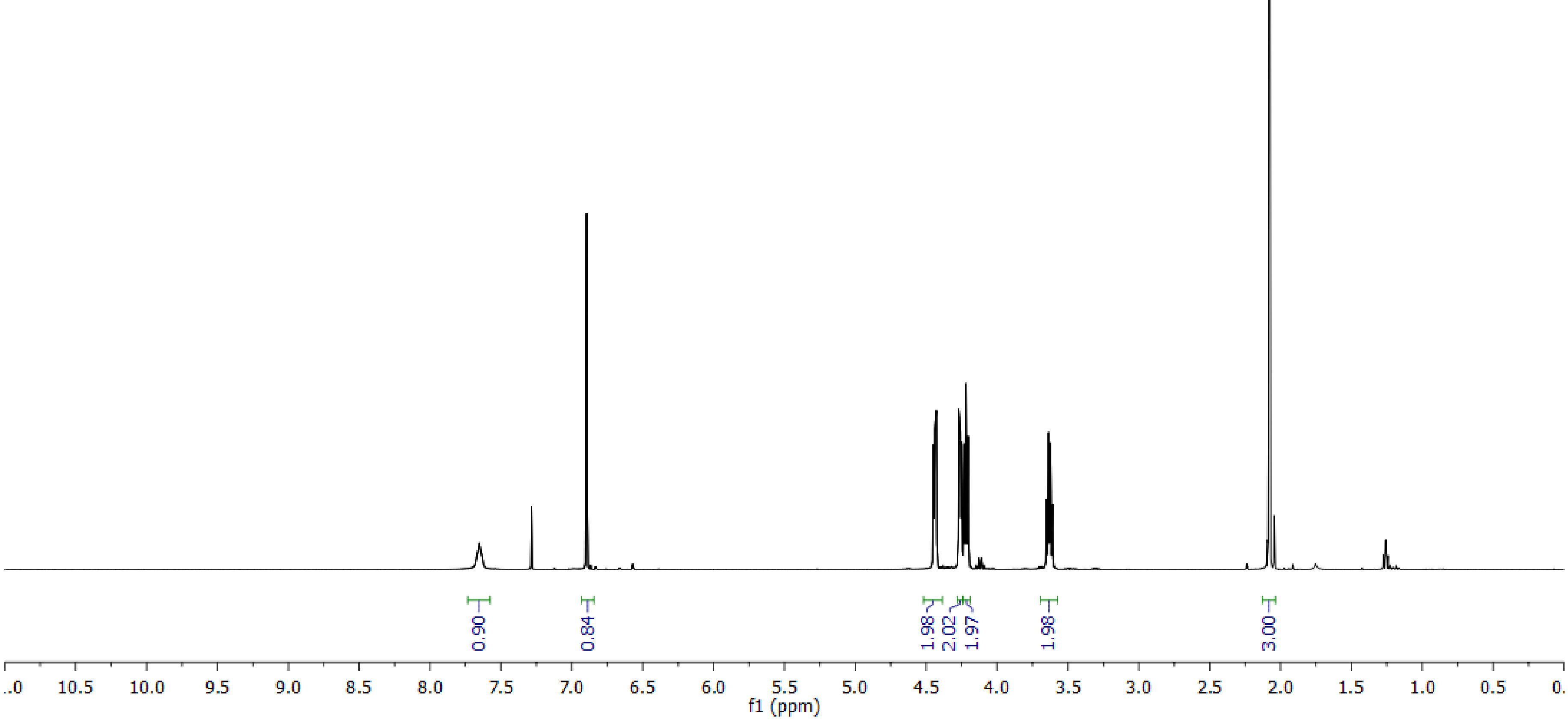

^{13}C NMR (100 MHz, $CDCl_3$)

Figure S134. ^{13}C NMR of **100**

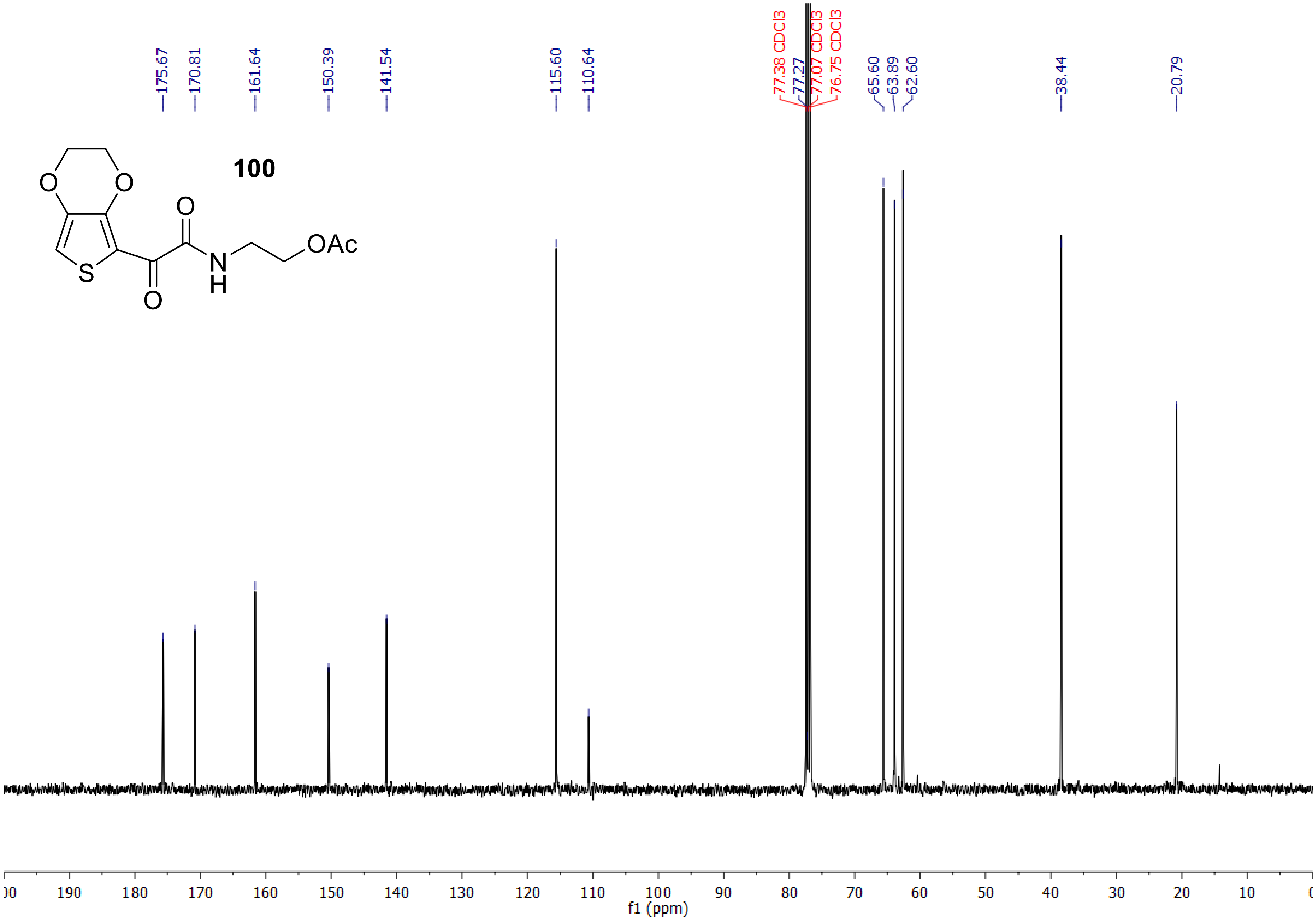

^{1}H NMR (400 MHz, $CDCl_3$)

Figure S135. ^{1}H NMR of **101**

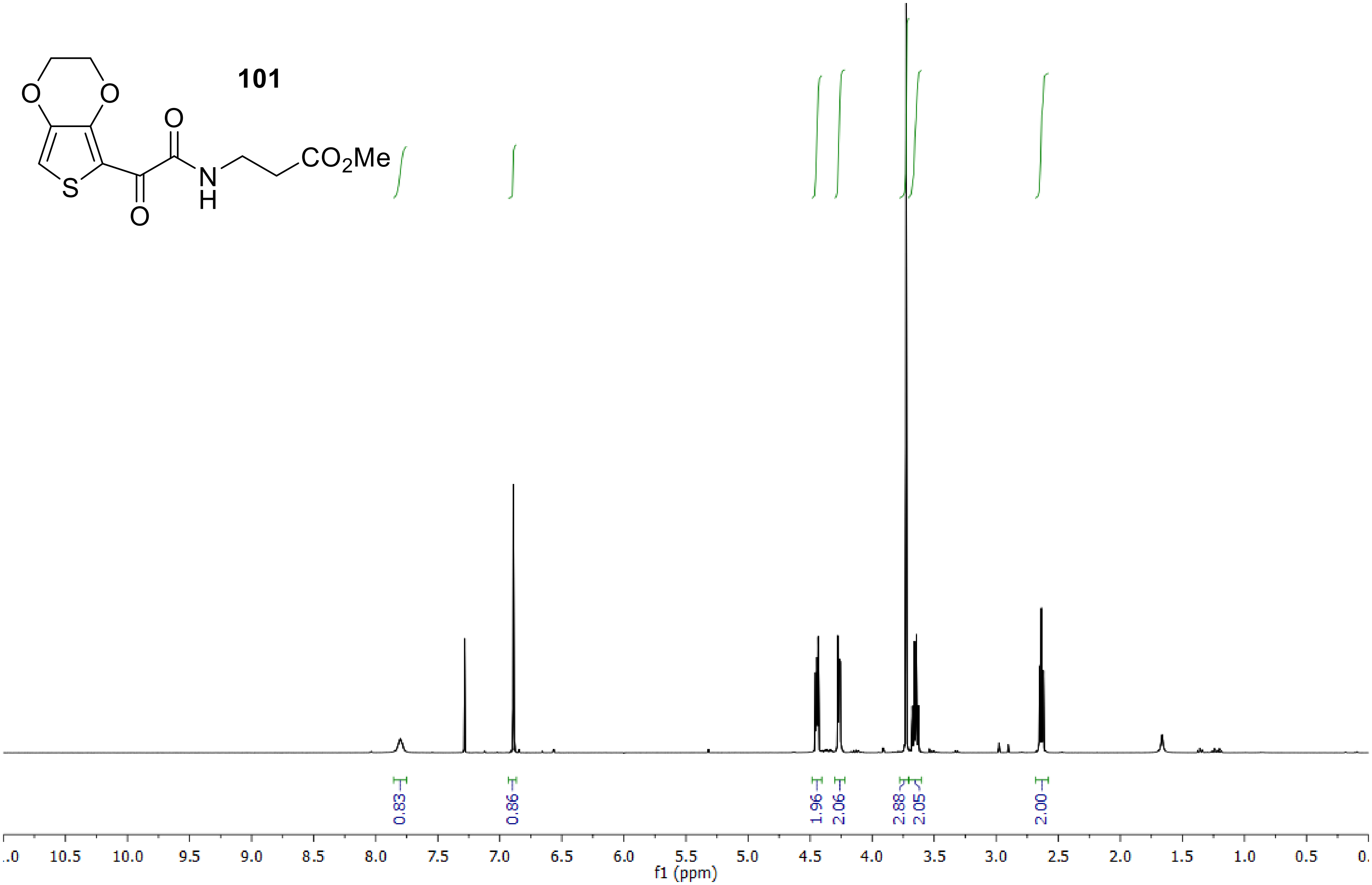

^{13}C NMR (100 MHz, $CDCl_3$)

Figure S136. ^{13}C NMR of **101**

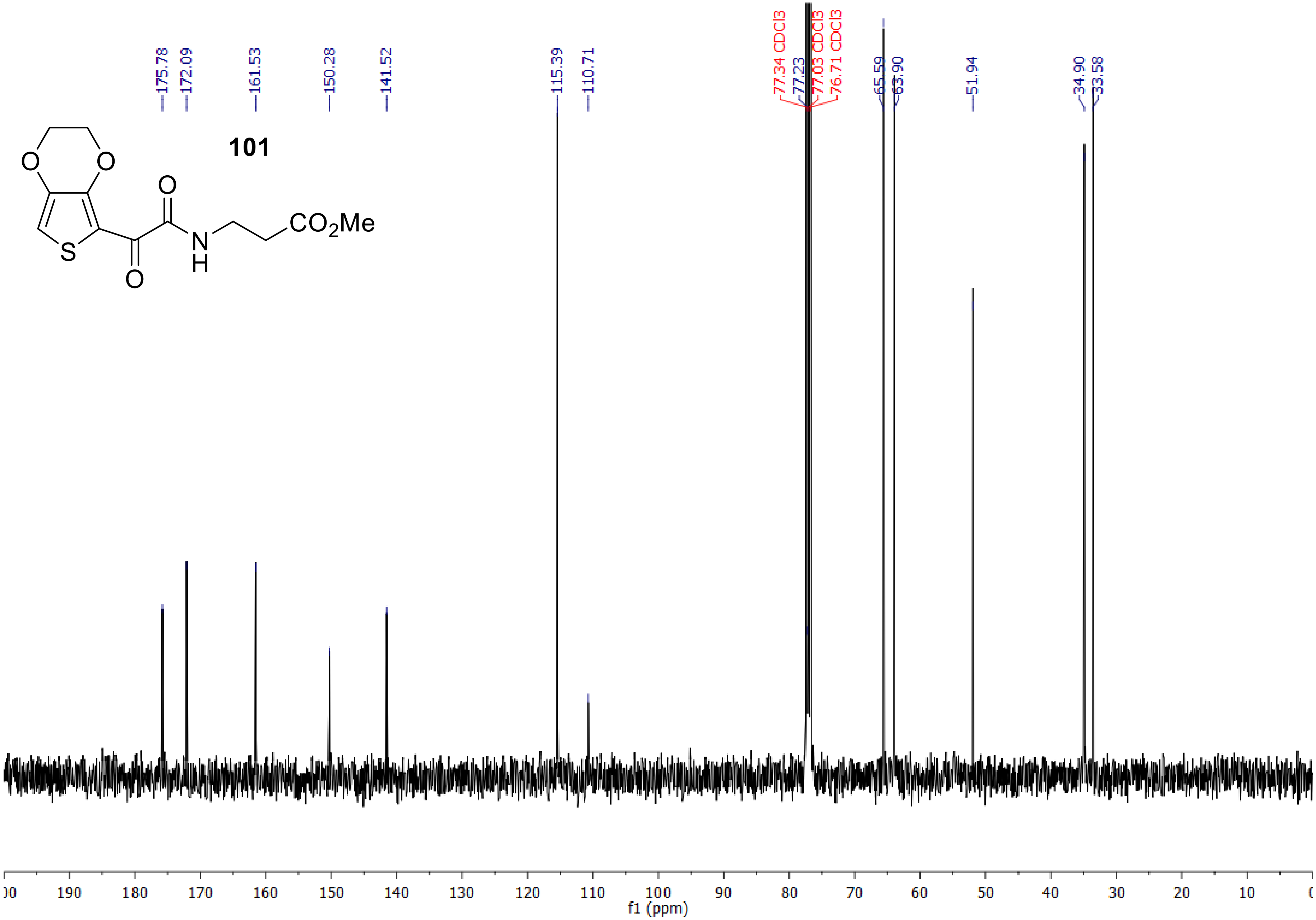

^{1}H NMR (400 MHz, $CDCl_3$)

Figure S137. ^{1}H NMR of **102**

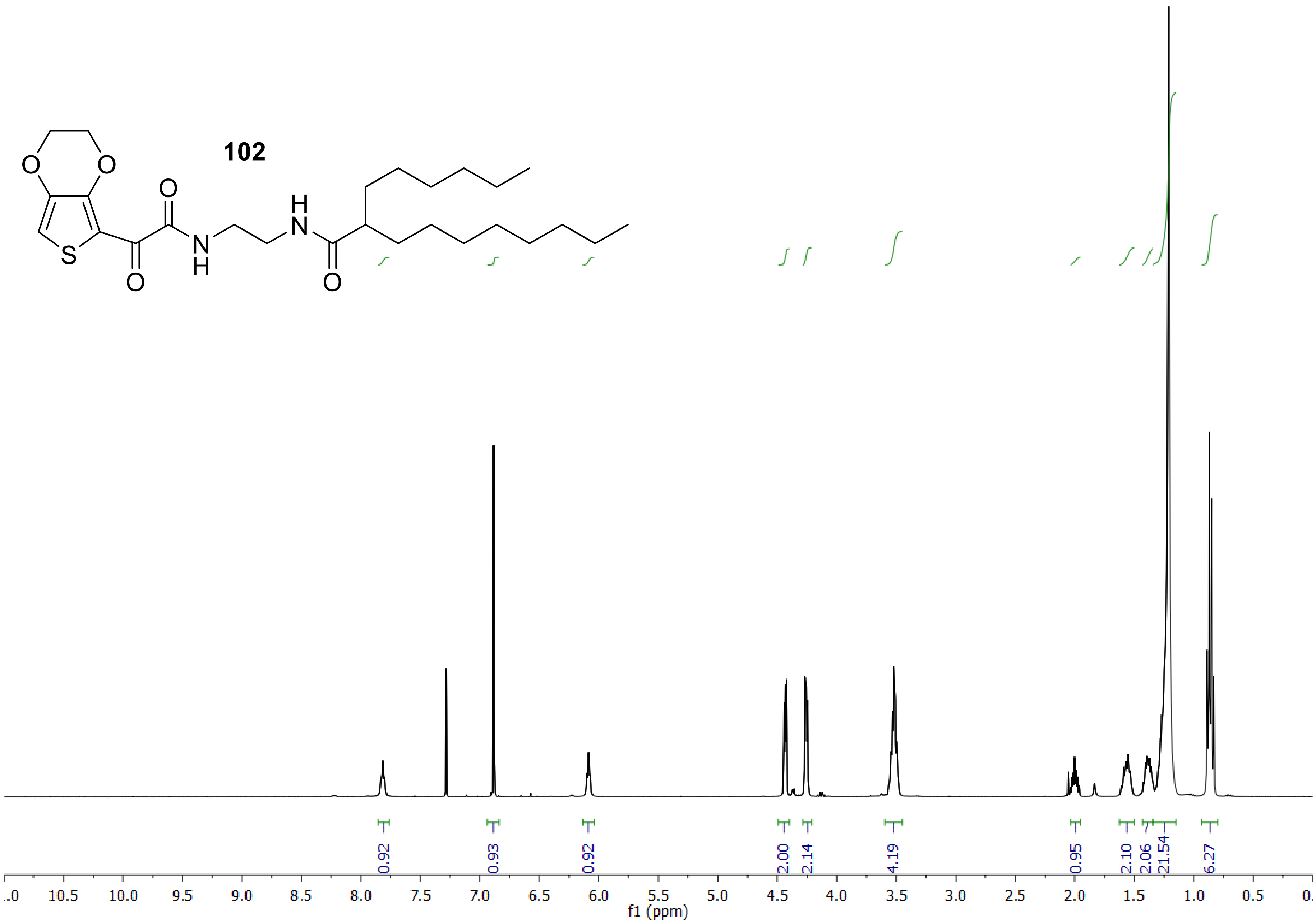

^{13}C NMR (100 MHz, $CDCl_3$)

Figure S138. ^{13}C NMR of **102**

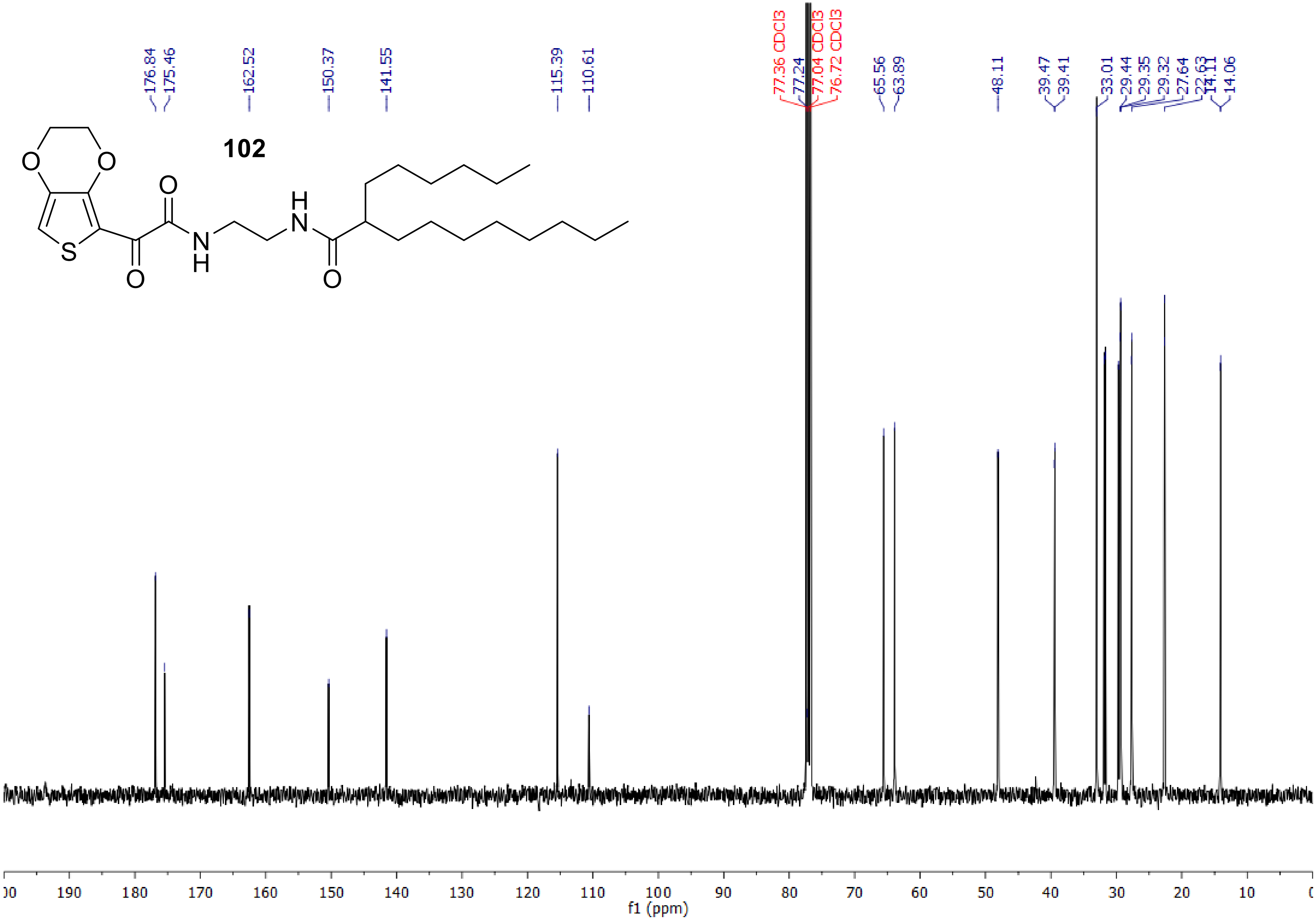

^{1}H NMR (400 MHz, $CDCl_3$)

Figure S139. ^{1}H NMR of **103**

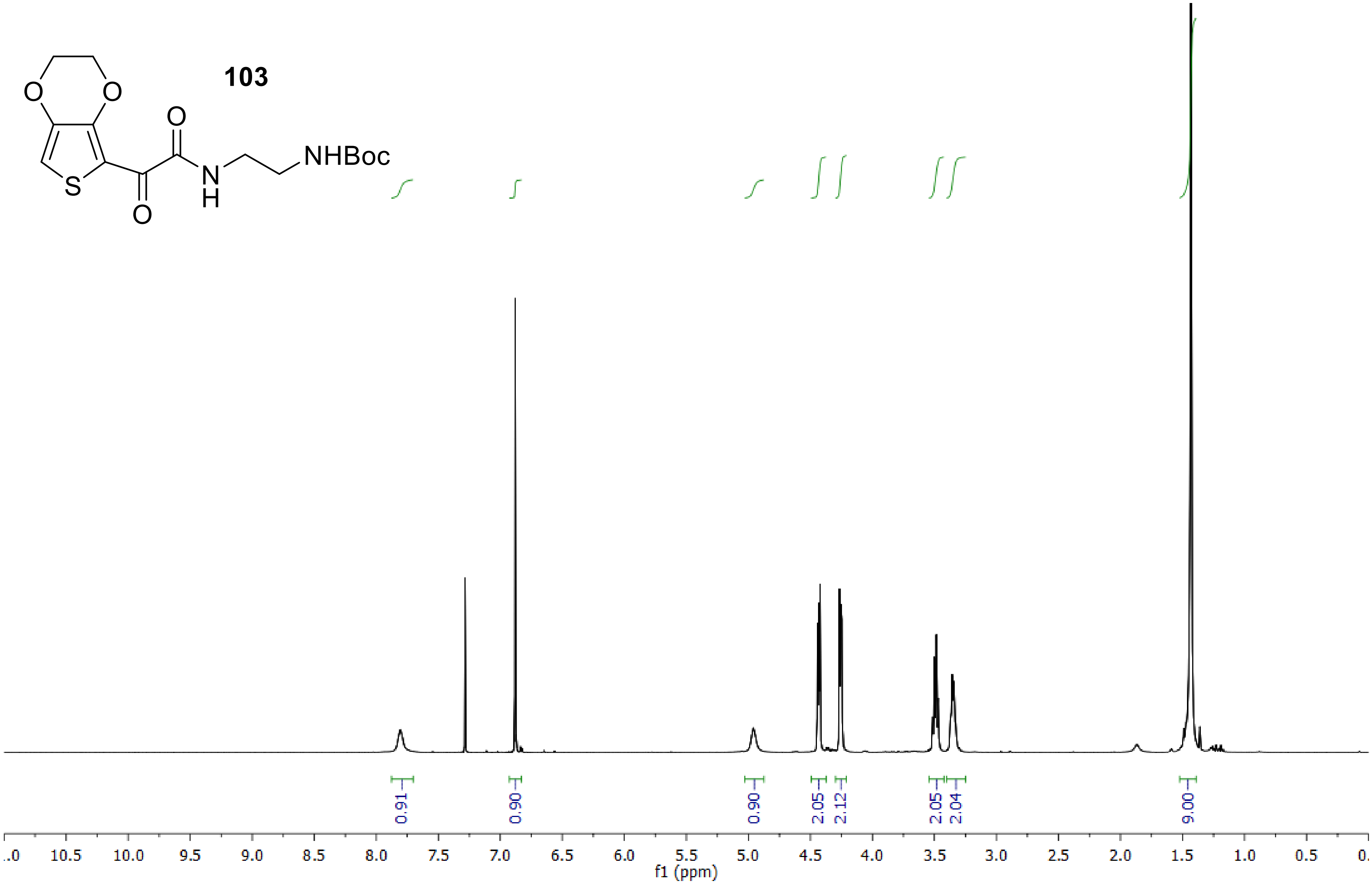

^{13}C NMR (100 MHz, $CDCl_3$)

Figure S140. ^{13}C NMR of **103**

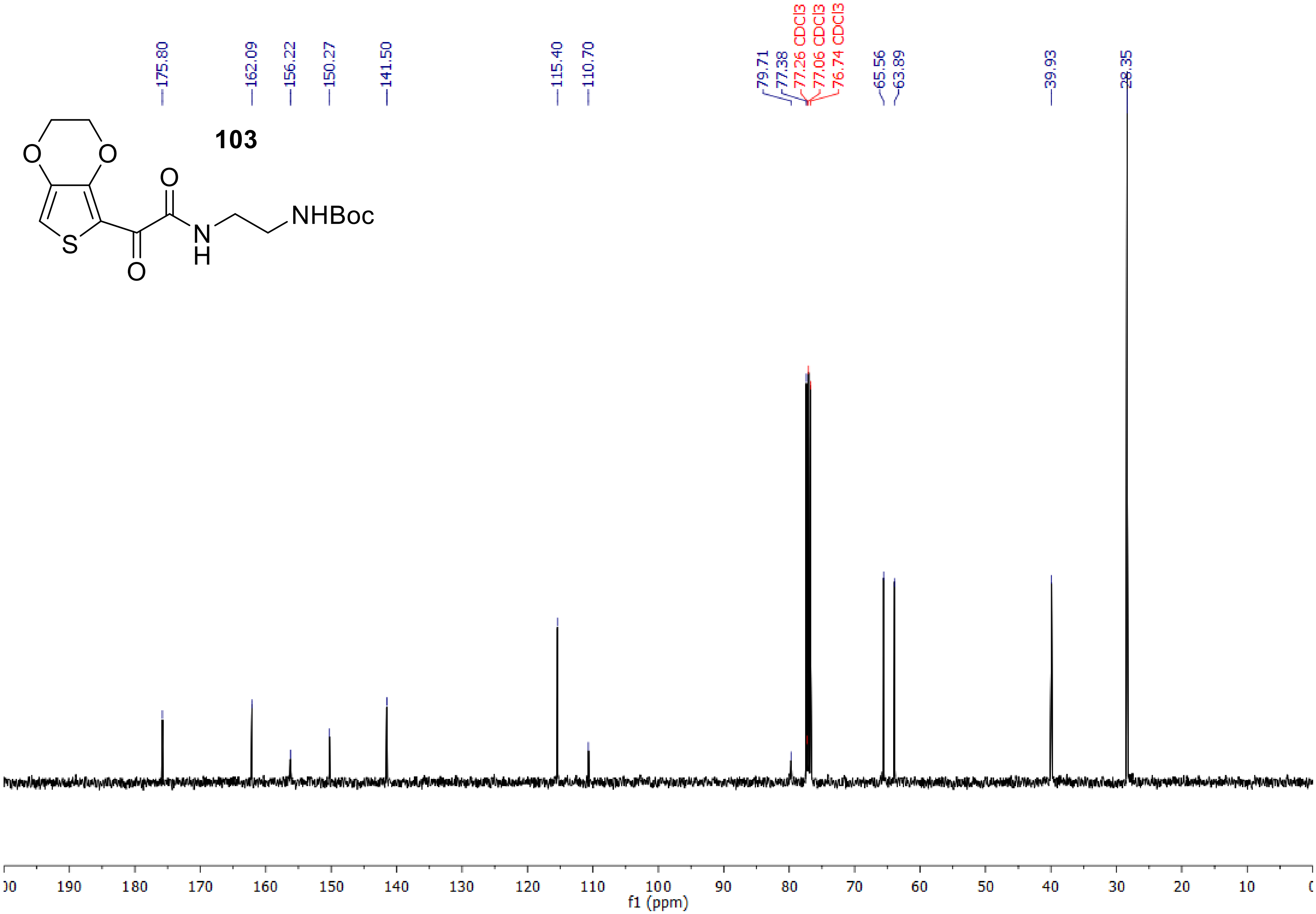

^{1}H NMR (400 MHz, $CDCl_3$)

Figure S141. ^{1}H NMR of **104**

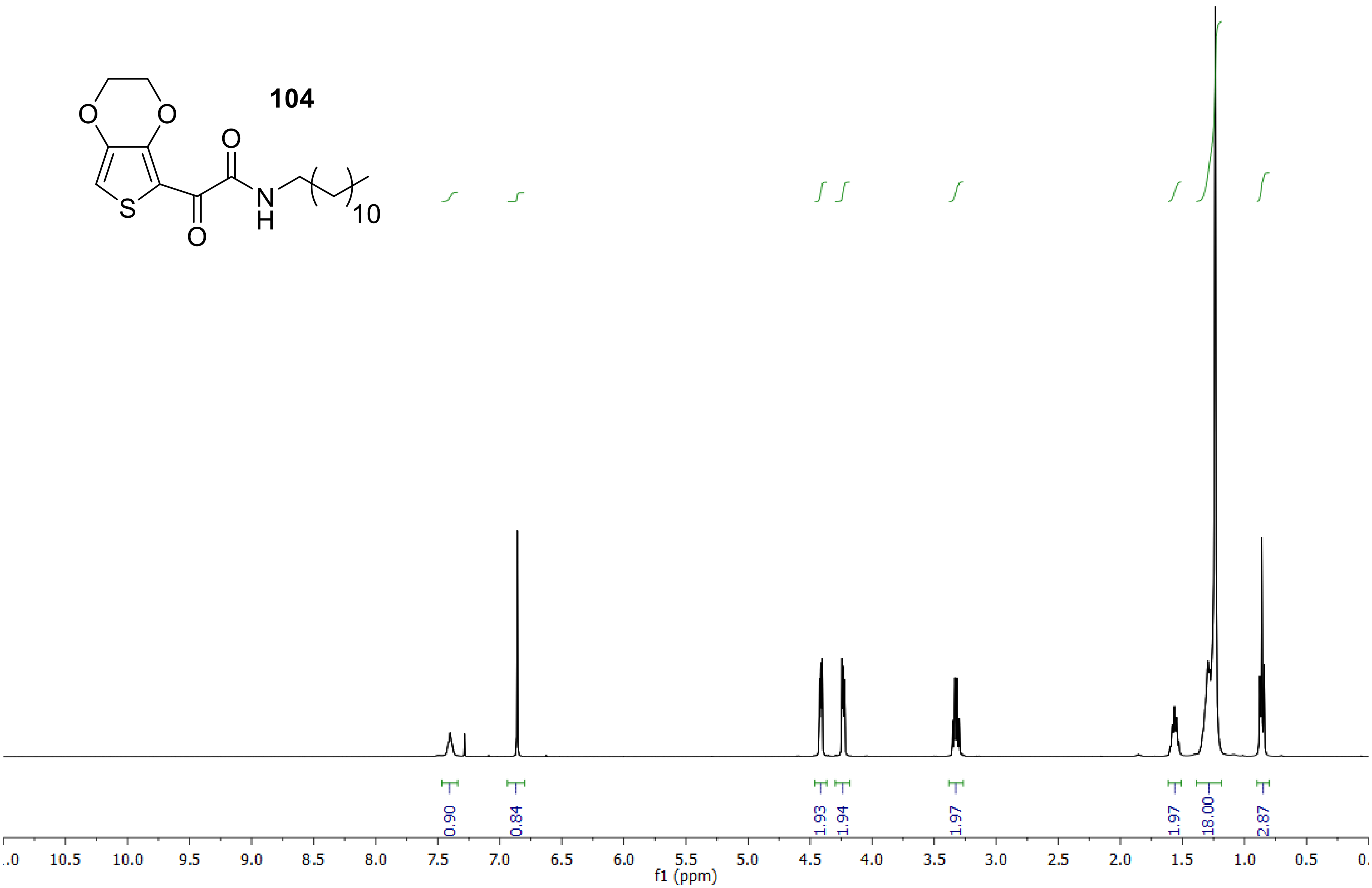

^{13}C NMR (100 MHz, $CDCl_3$)

Figure S142. ^{13}C NMR of **104**

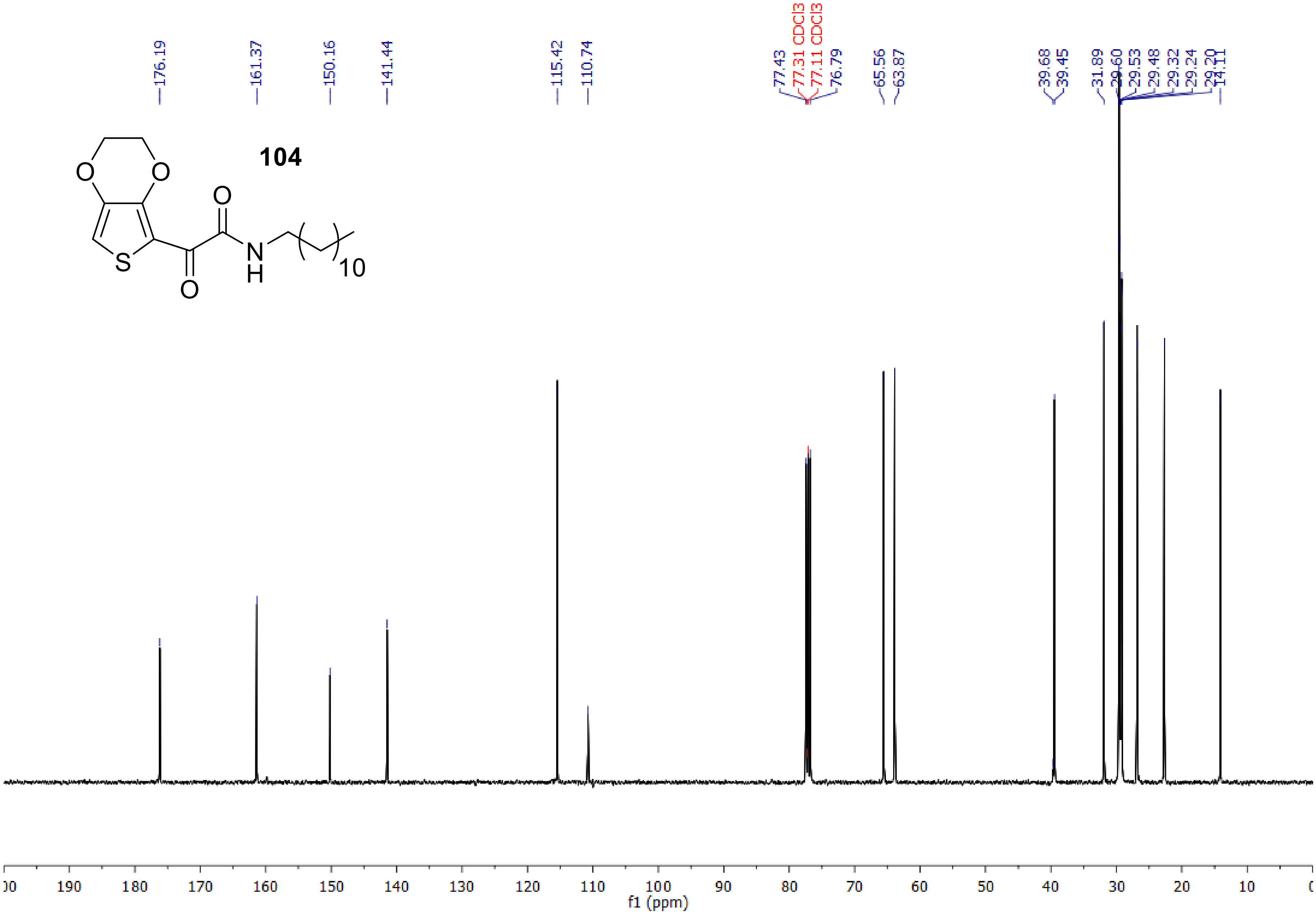

^{1}H NMR (400 MHz, $CDCl_3$)

Figure S143. ^{1}H NMR of **105**

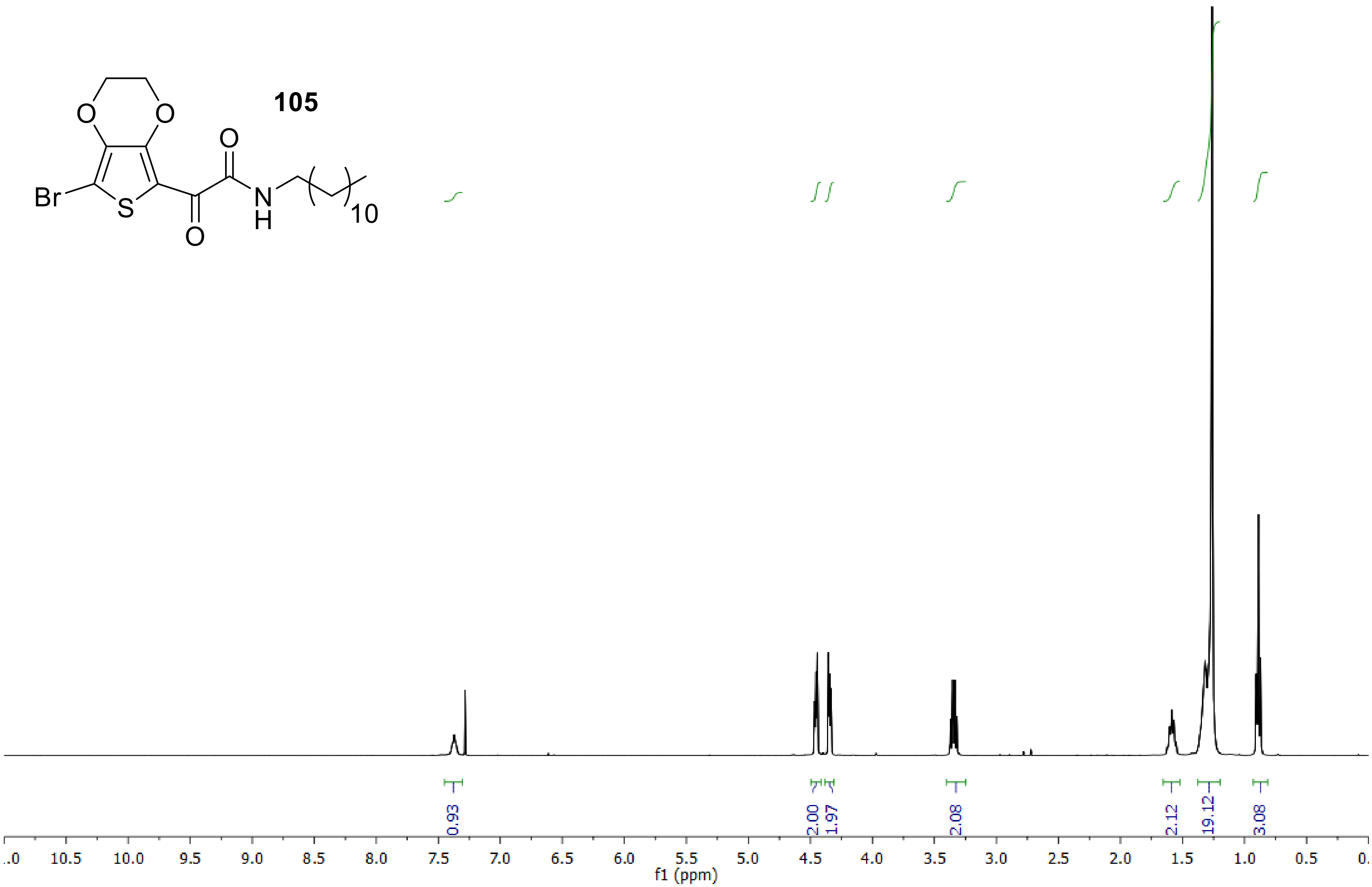

^{13}C NMR (100 MHz, $CDCl_3$)

Figure S144. ^{13}C NMR of **105**

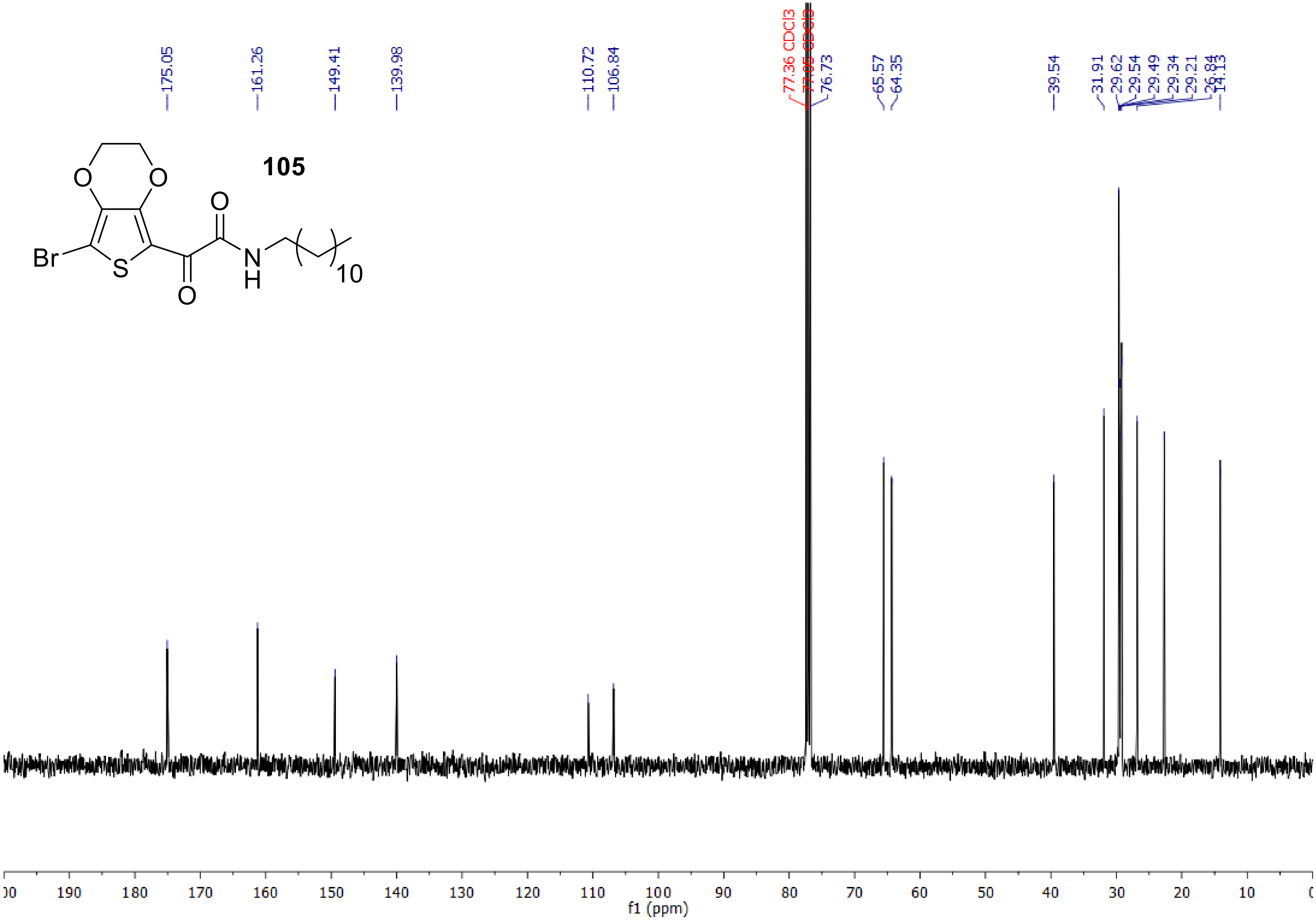

^{1}H NMR (400 MHz, DMSO-d$_6$)

Figure S145. ^{1}H NMR of **106**

106

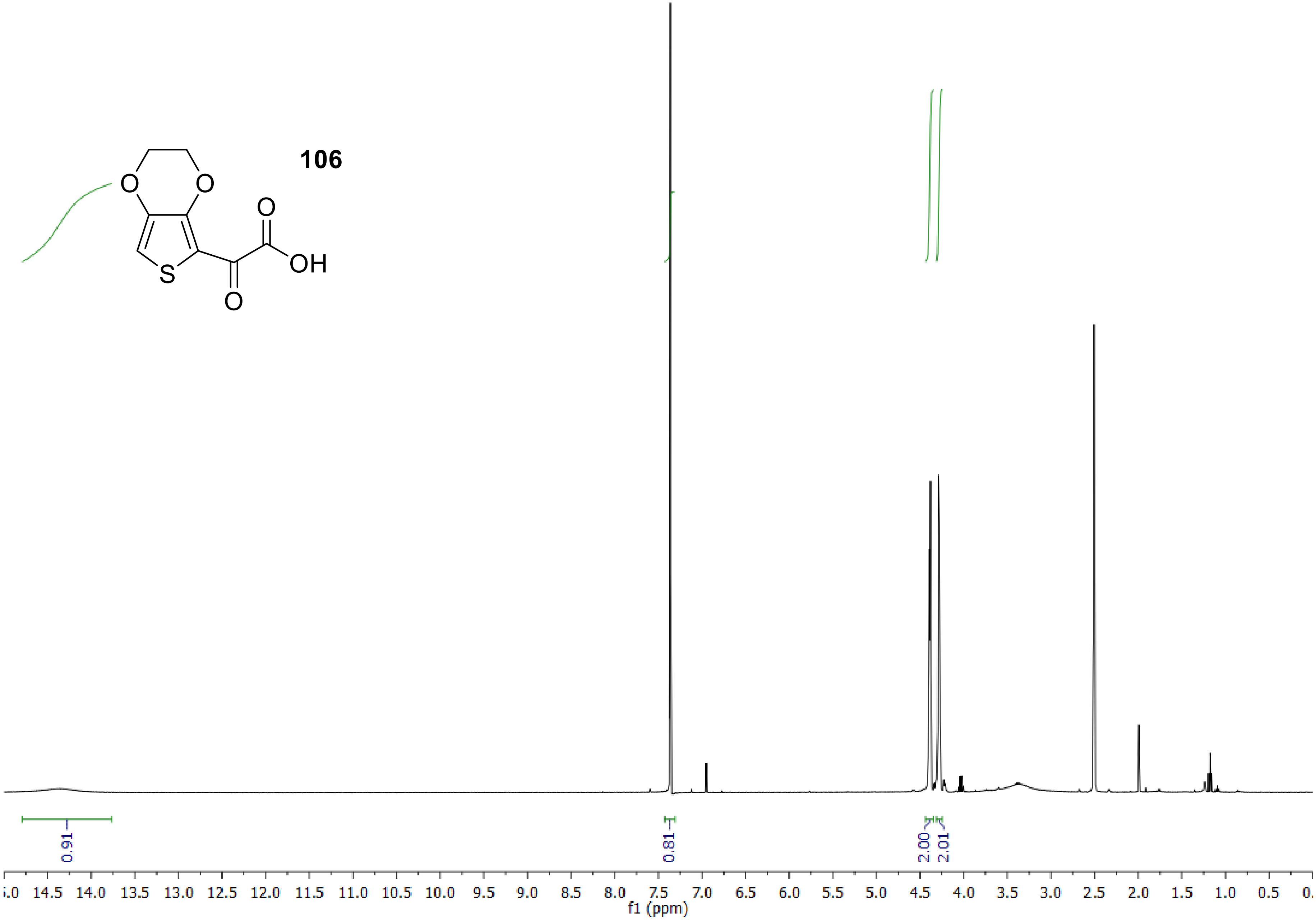

^{13}C NMR (100 MHz, DMSO-d_6)

Figure S146. ^{13}C NMR of **106**

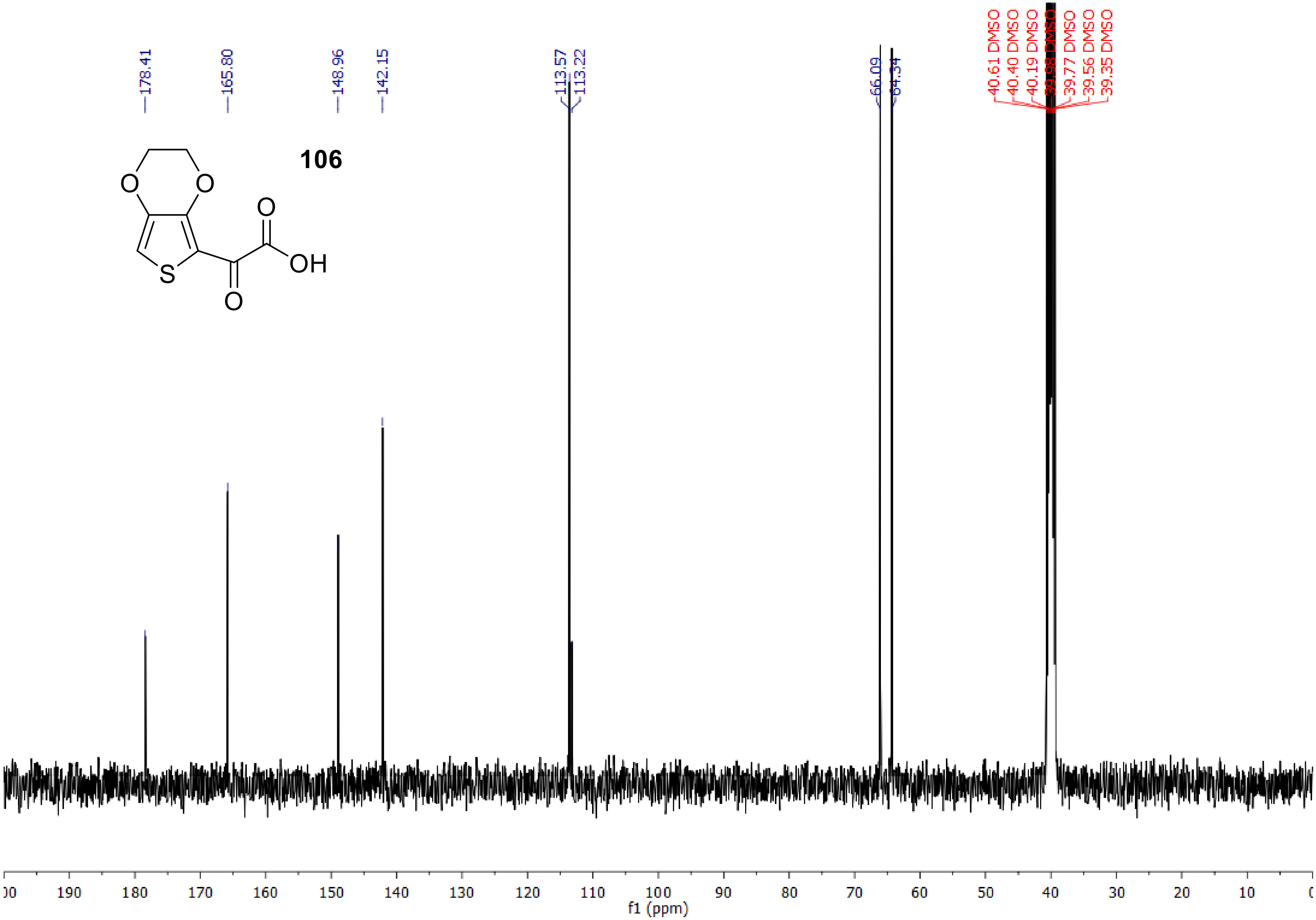

^{1}H NMR (400 MHz, DMSO-d_6)

Figure S147. ^{1}H NMR of **107**

107

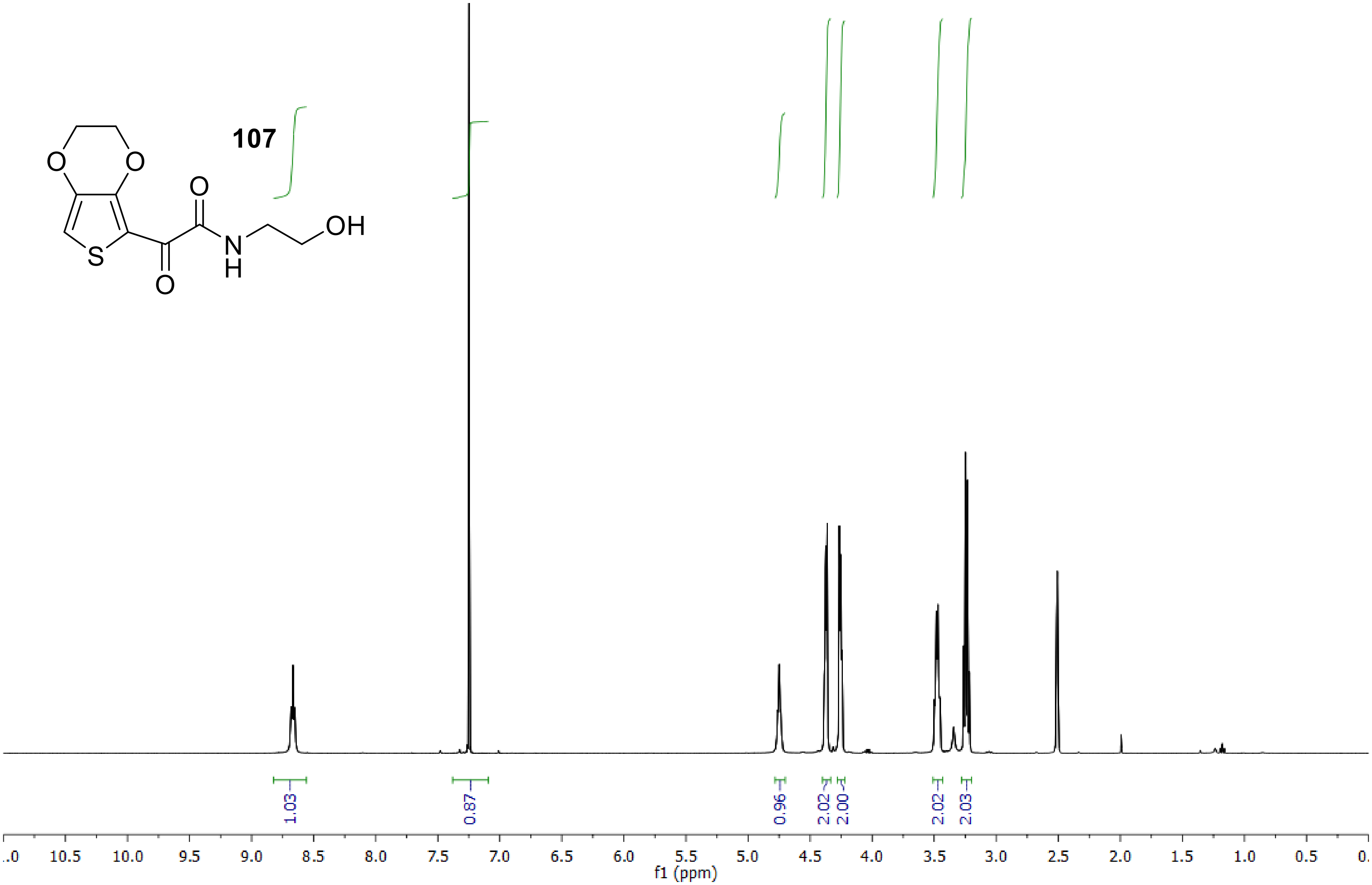

^{13}C NMR (100 MHz, DMSO-d_6)

Figure S148. ^{13}C NMR of **107**

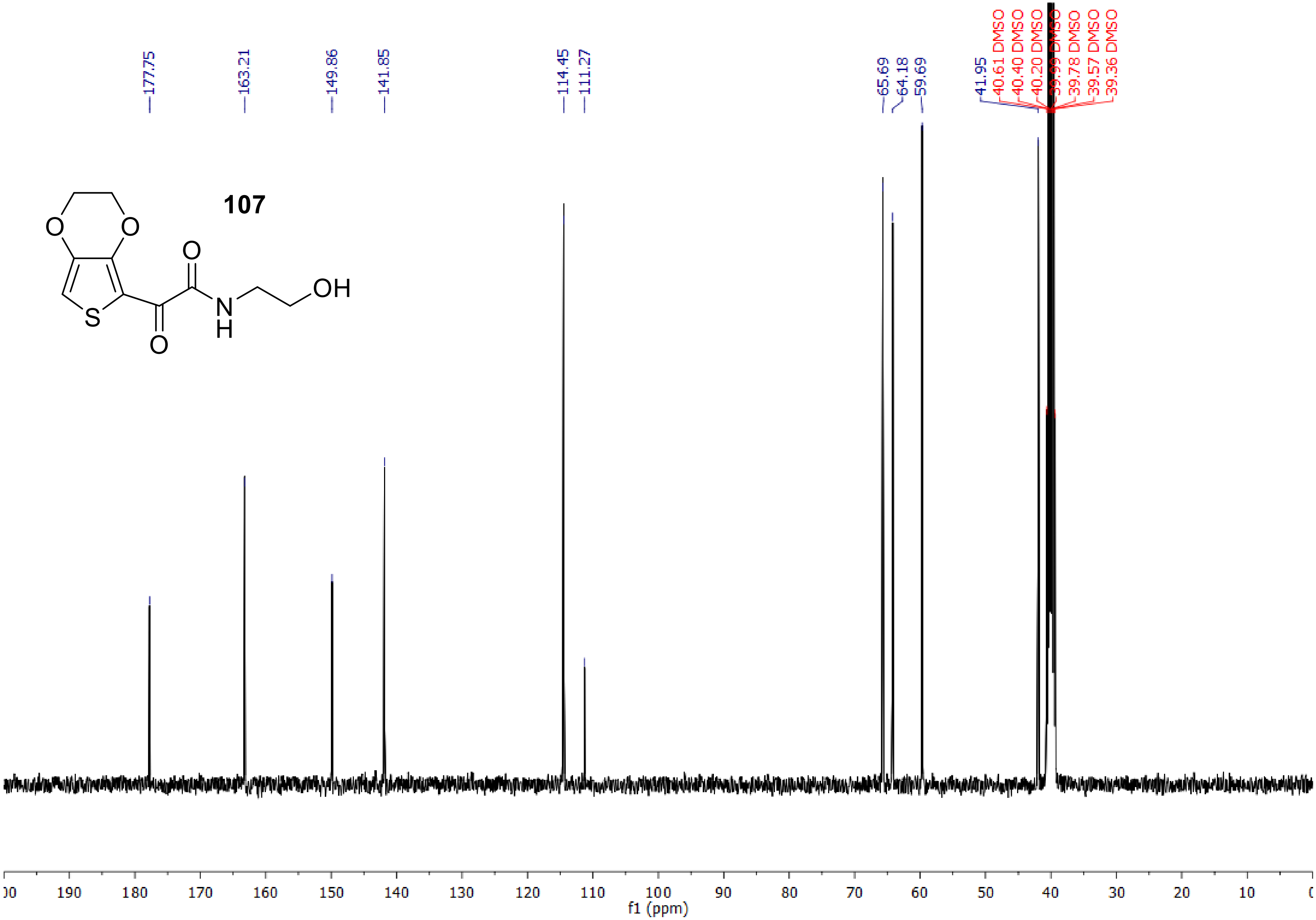

^{1}H NMR (400 MHz, DMSO-d$_6$)

Figure S149. ^{1}H NMR of **108**

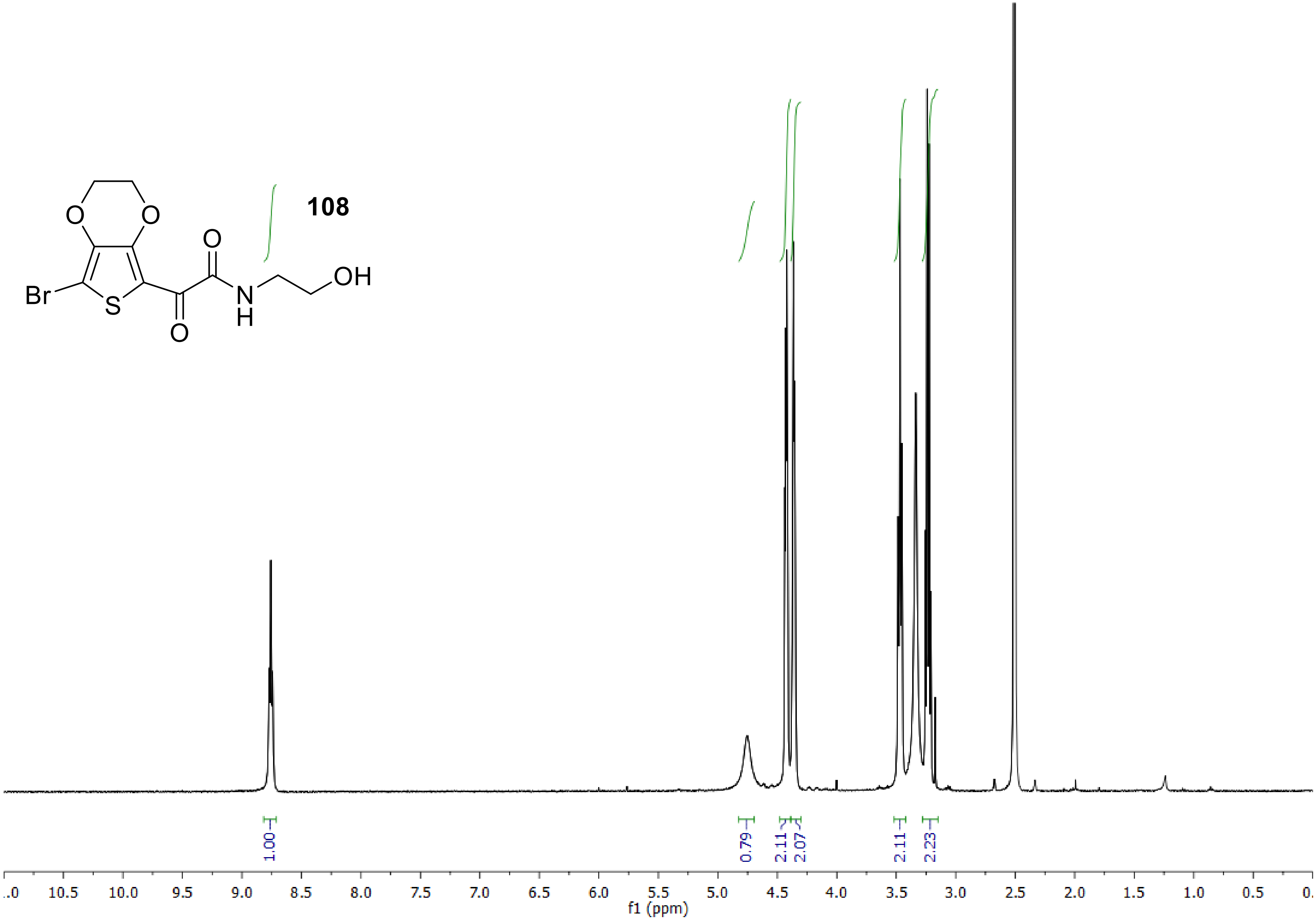

^{13}C NMR (100 MHz, DMSO-d_6)

Figure S150. ^{13}C NMR of **108**

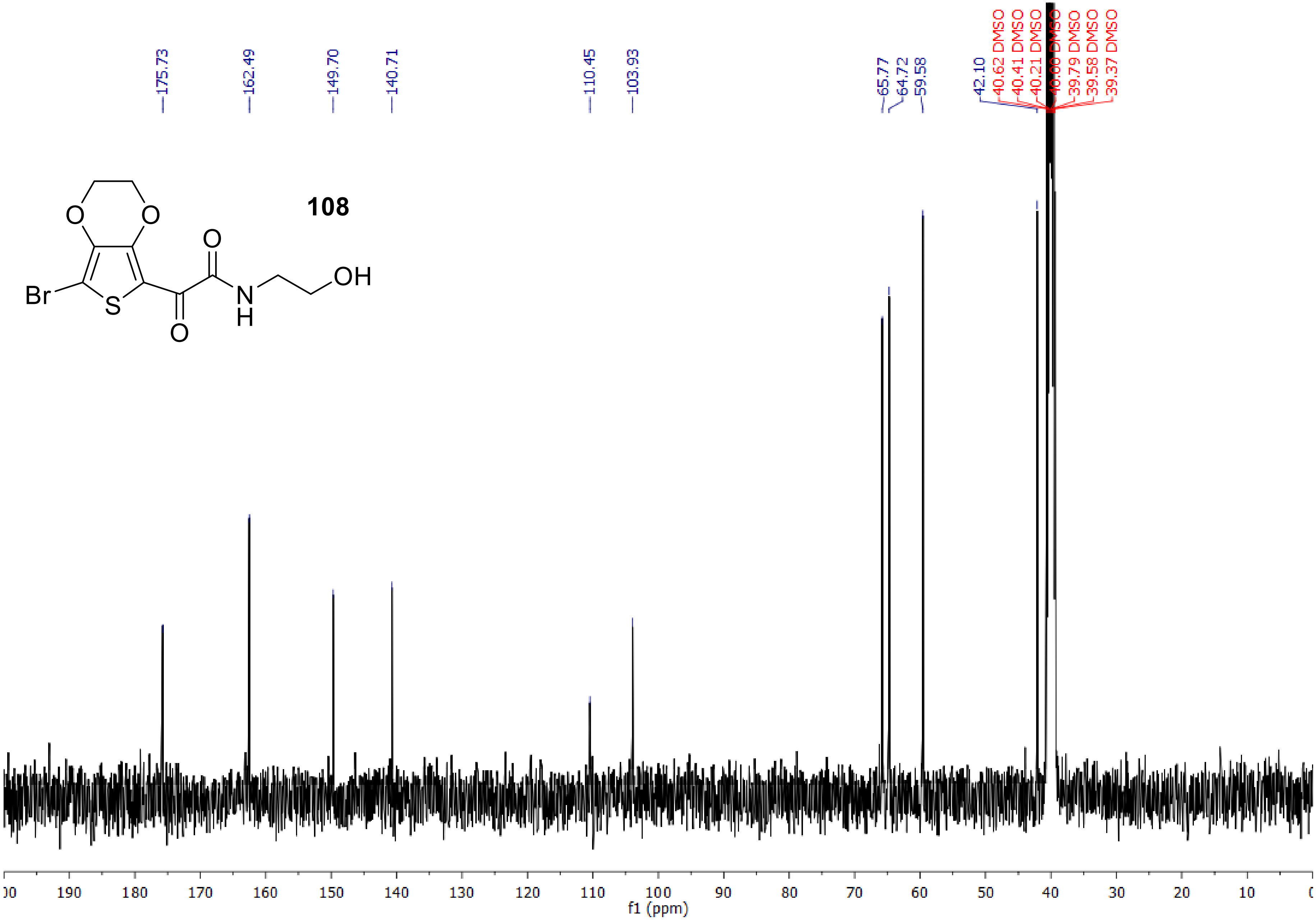

^{1}H NMR (400 MHz, $CDCl_3$)

Figure S151. ^{1}H NMR of **109**

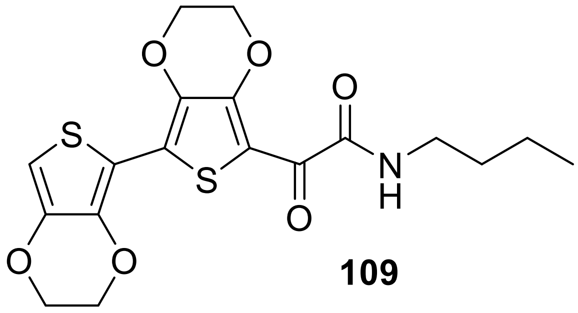

109

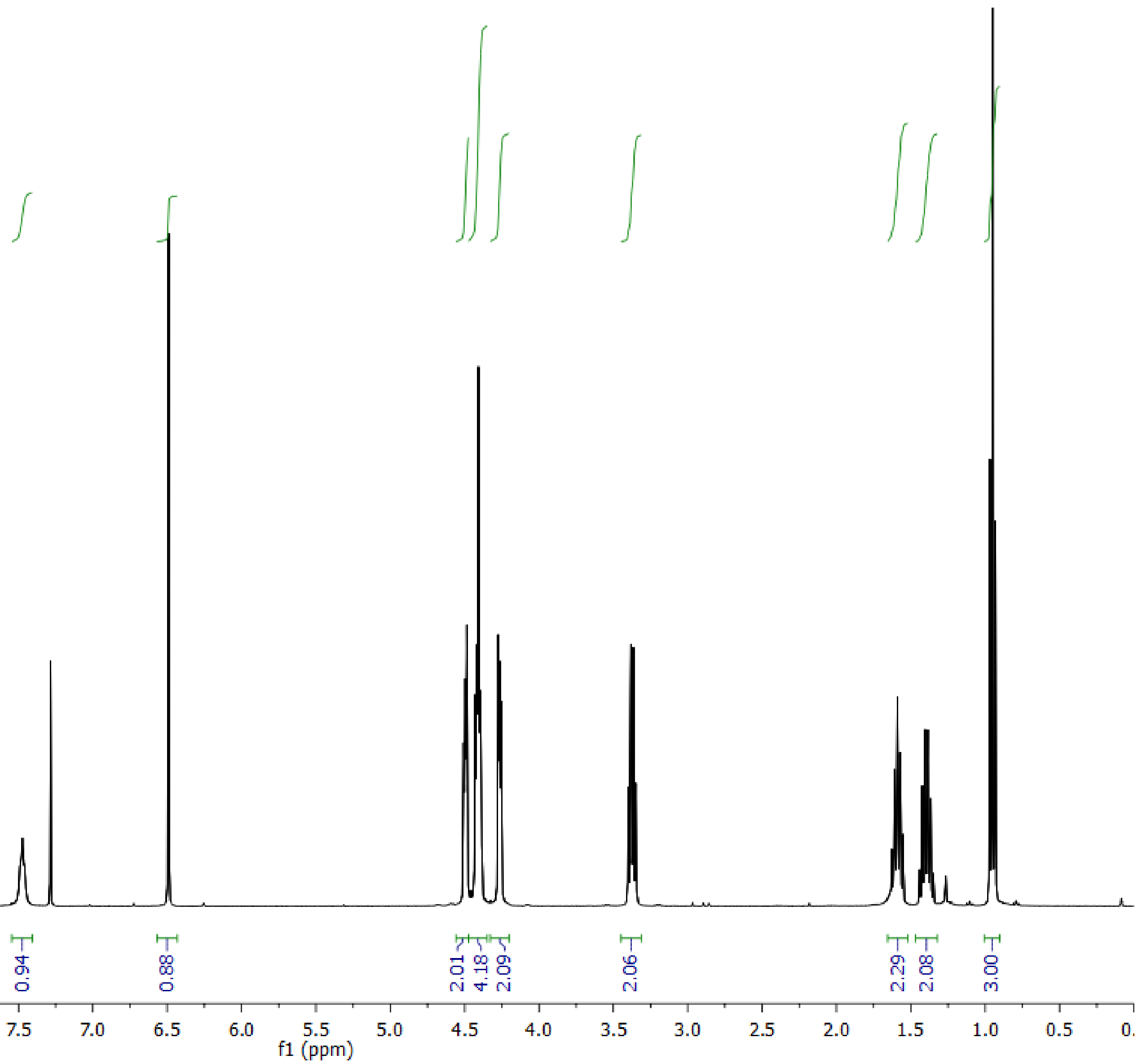

^{13}C NMR (100 MHz, $CDCl_3$)

Figure S152. ^{13}C NMR of **109**

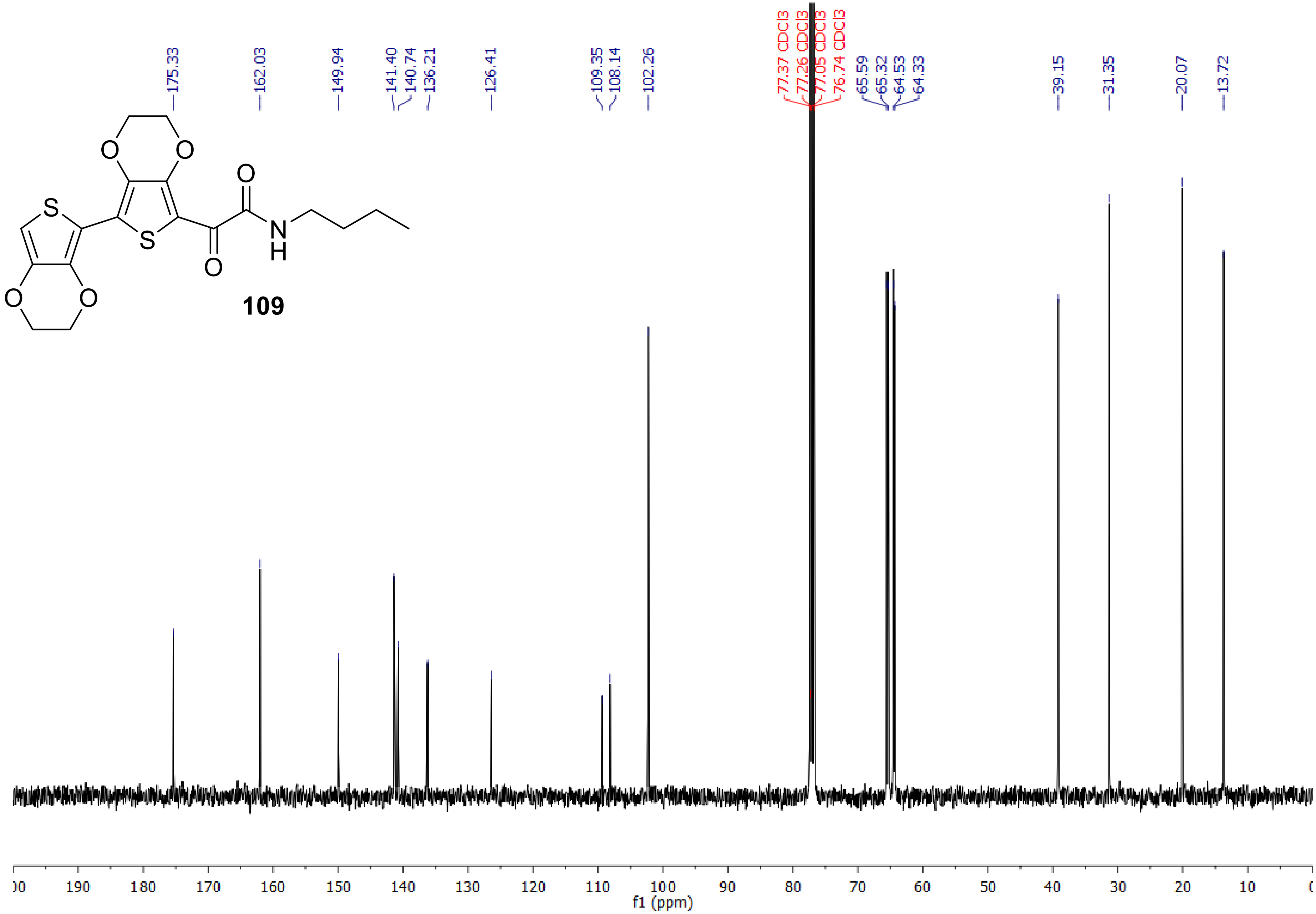

^{1}H NMR (400 MHz, $CDCl_3$)

Figure S153. ^{1}H NMR of **110**

110

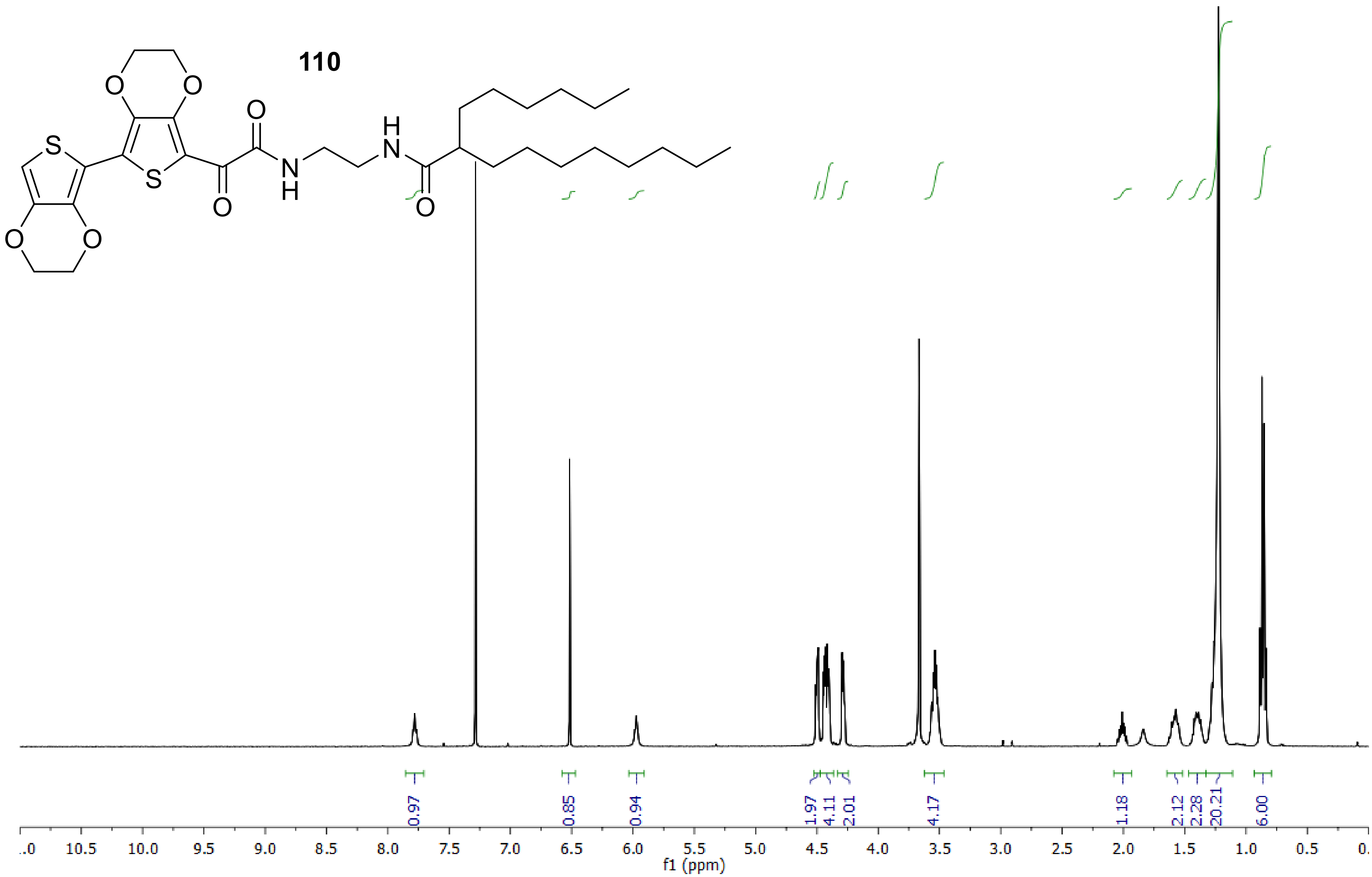

^{13}C NMR (100 MHz, $CDCl_3$)

Figure S154. ^{13}C NMR of **110**

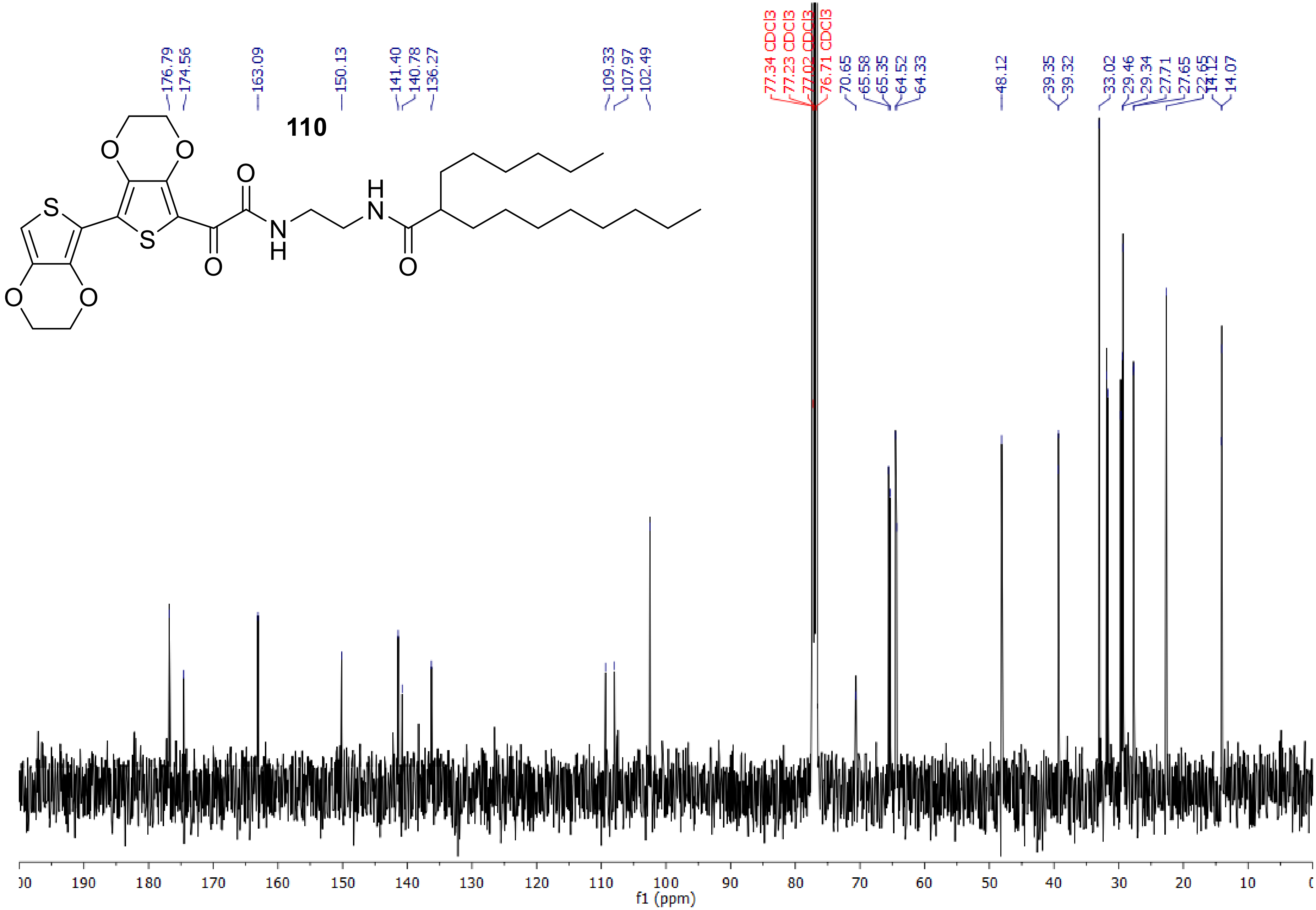

^{1}H NMR (400 MHz, $CDCl_3$)

Figure S155. ^{1}H NMR of **111**

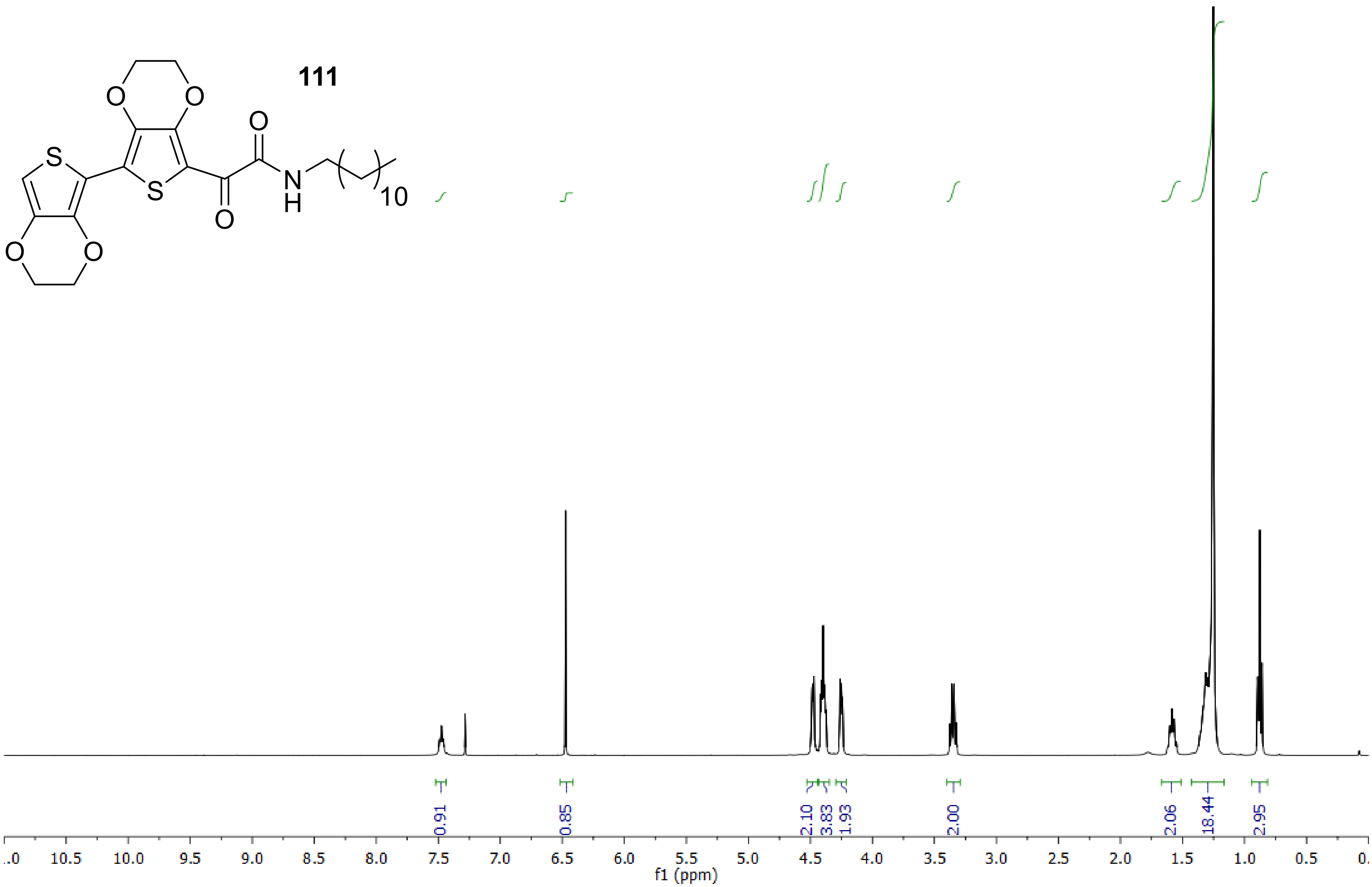

^{13}C NMR (100 MHz, $CDCl_3$)

Figure S156. ^{13}C NMR of **111**

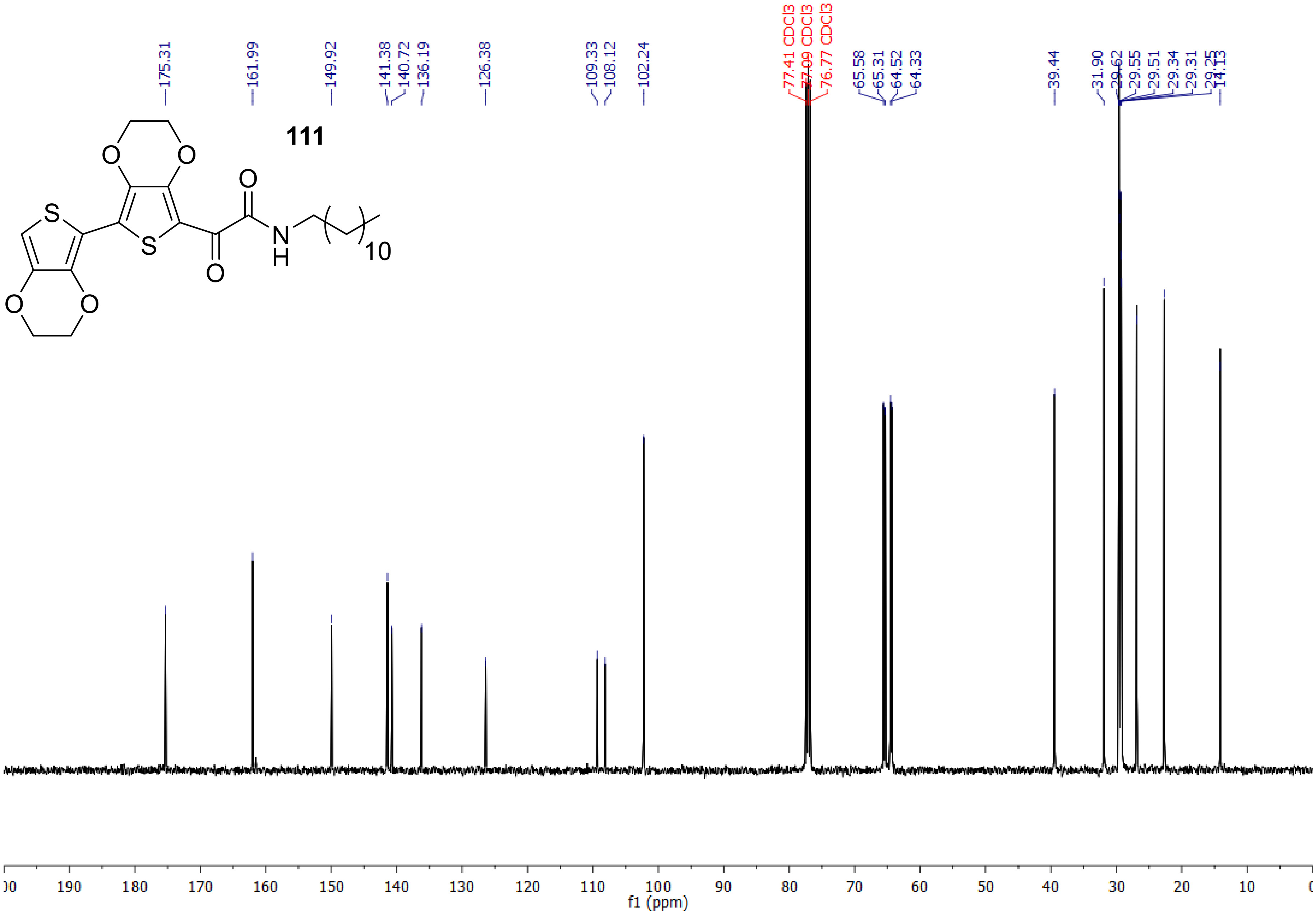

^{1}H NMR (400 MHz, $CDCl_3$)

Figure S157. ^{1}H NMR of **112**

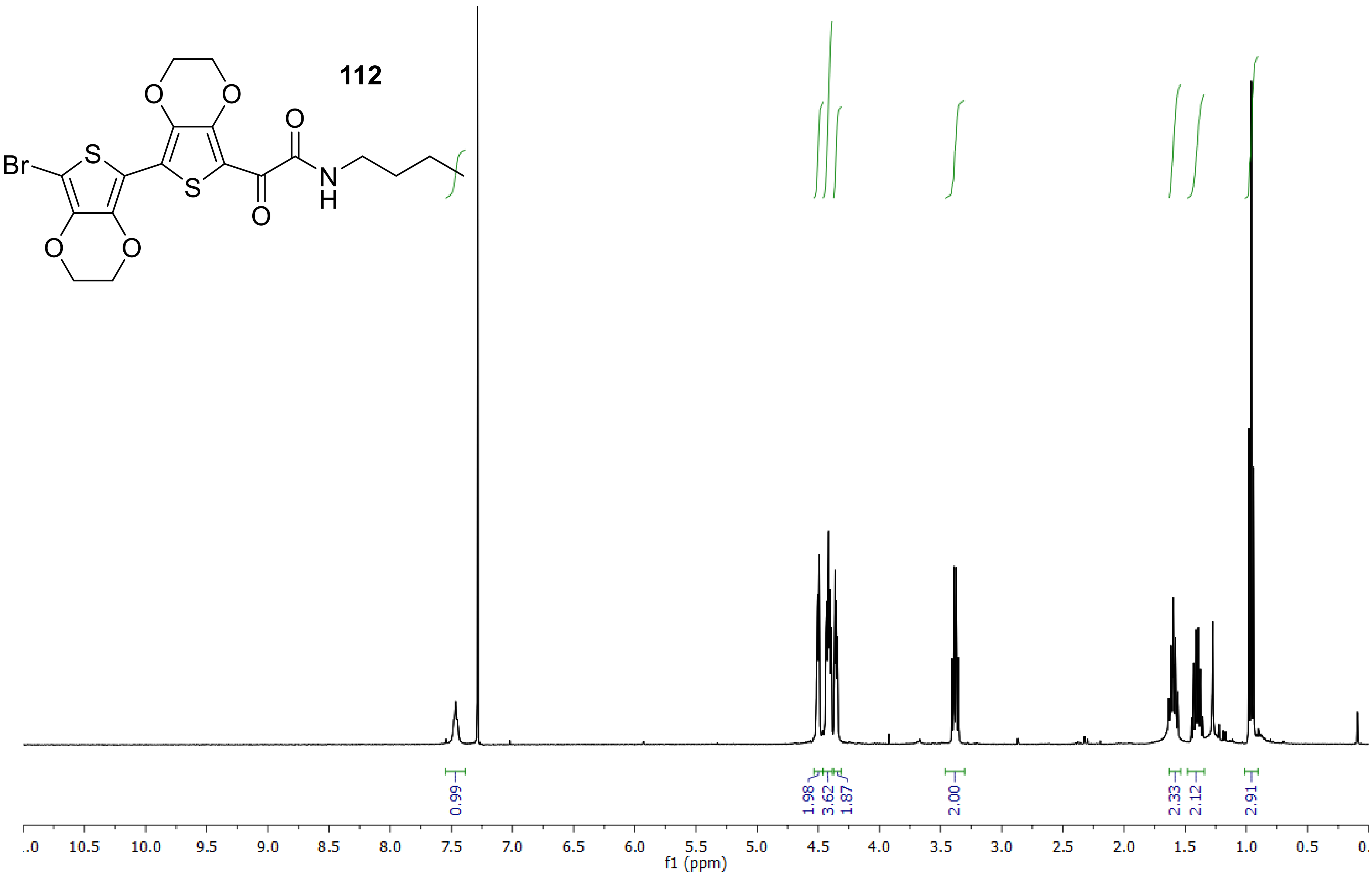

^{13}C NMR (100 MHz, $CDCl_3$)

Figure S158. ^{13}C NMR of **112**

112

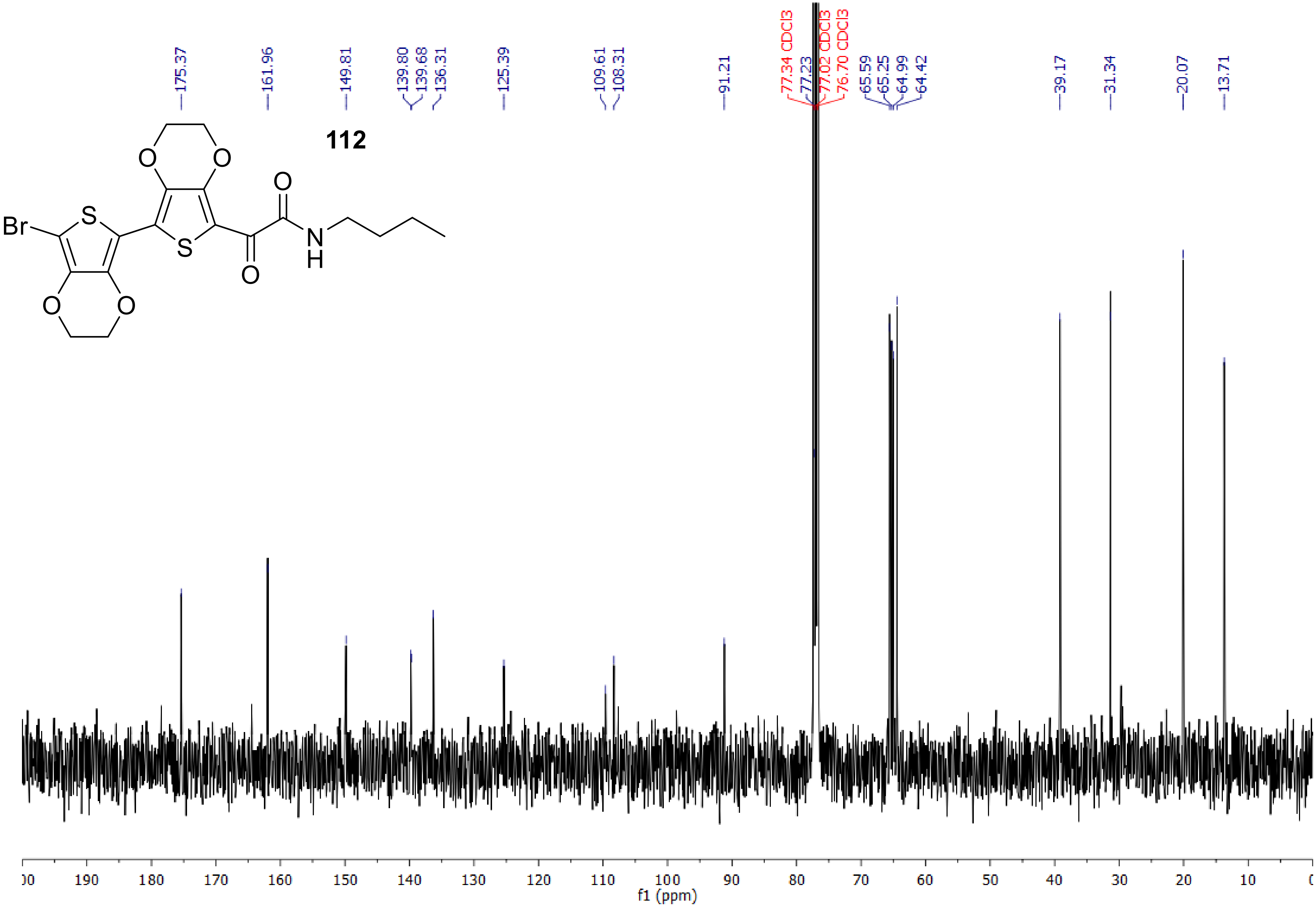

¹H NMR (400 MHz, DMSO)

Figure S159. ¹H NMR of **113**

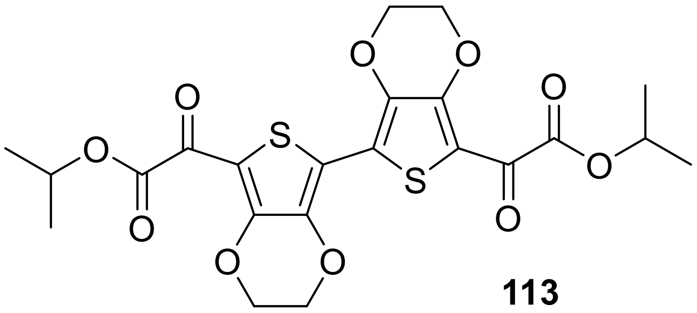

113

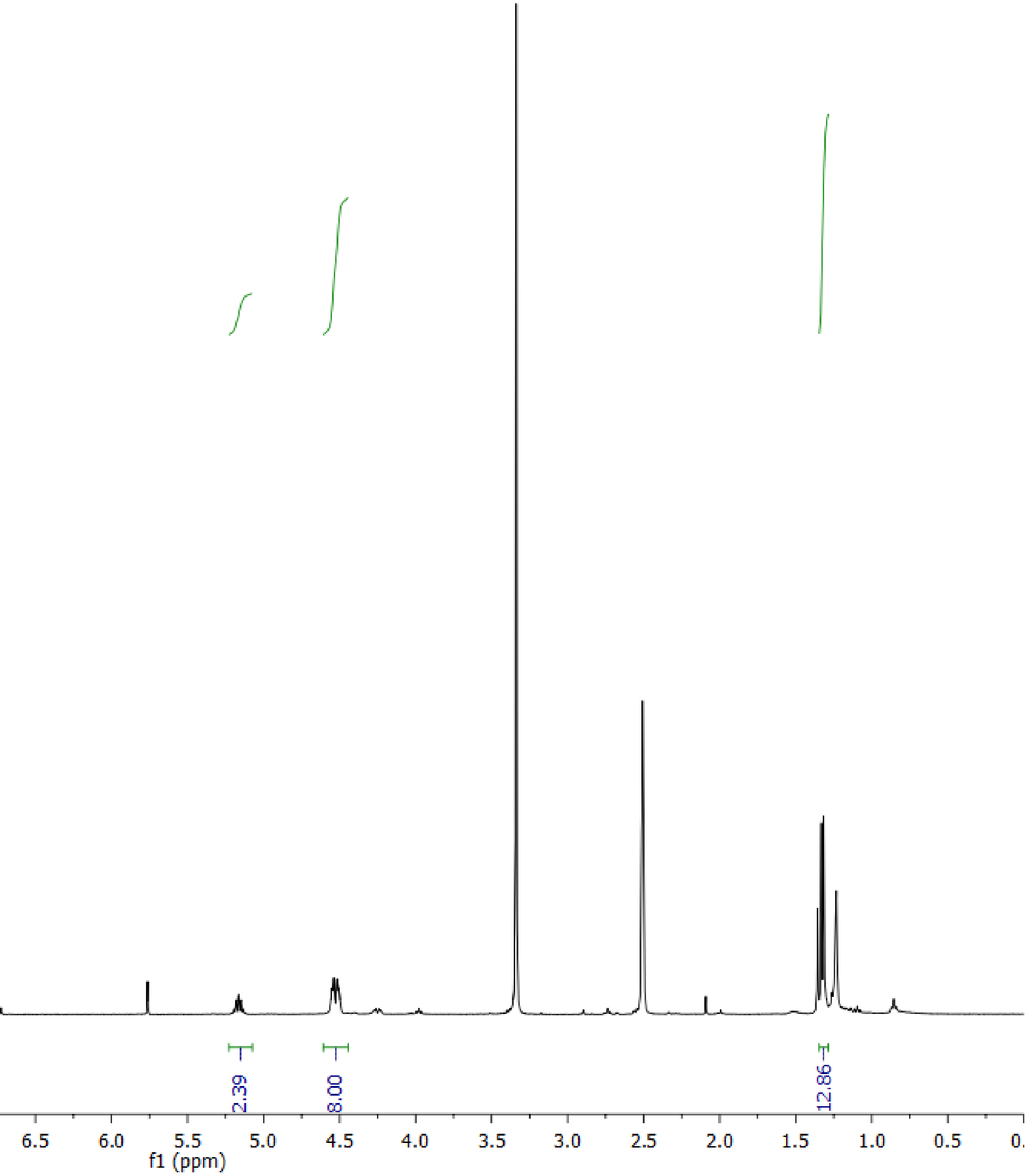

^{1}H NMR (400 MHz, $CDCl_3$)

Figure S160. ^{1}H NMR of **114**

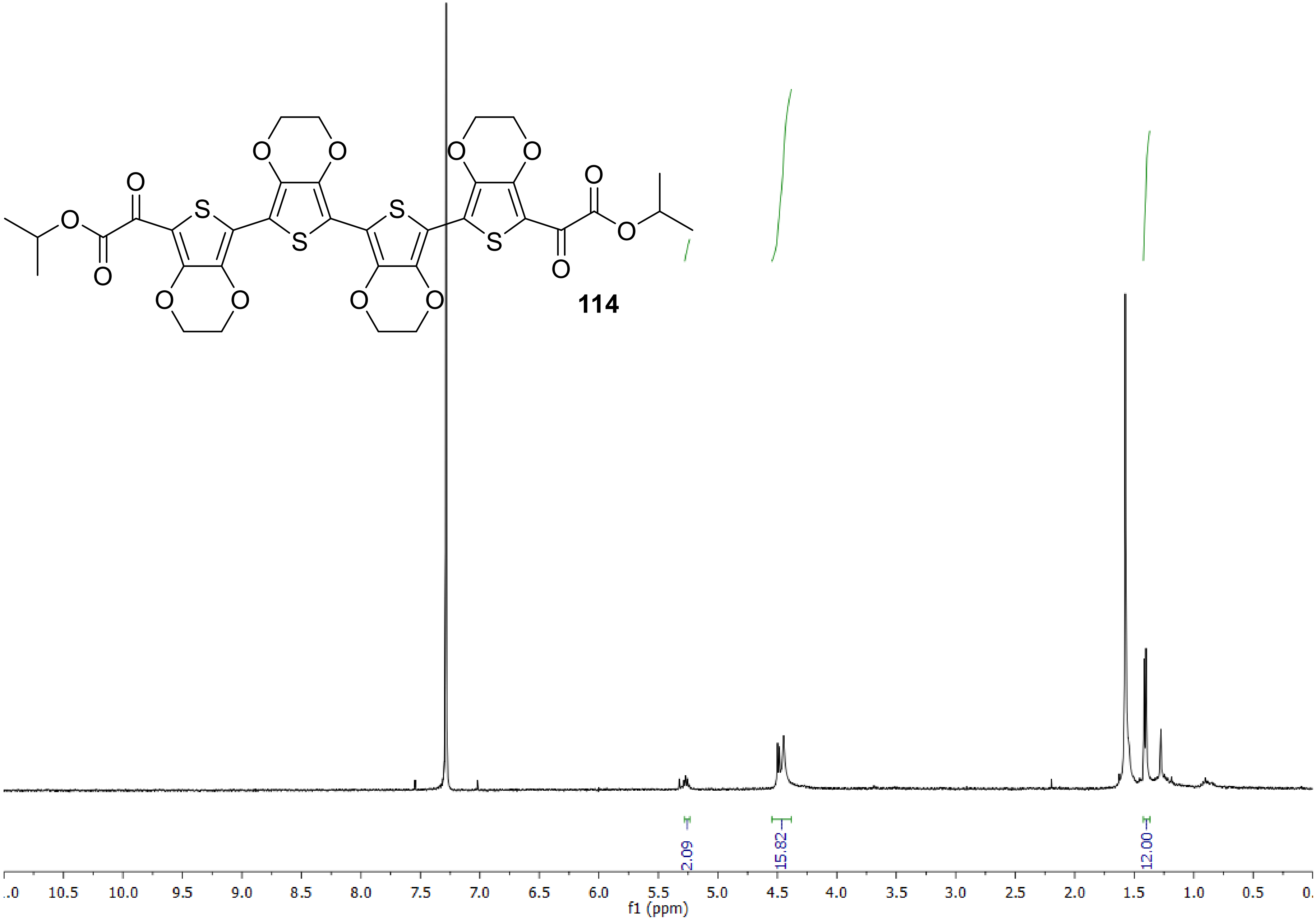

^{13}C NMR (125 MHz, $CDCl_3$)

Figure S161. ^{13}C NMR of **114**

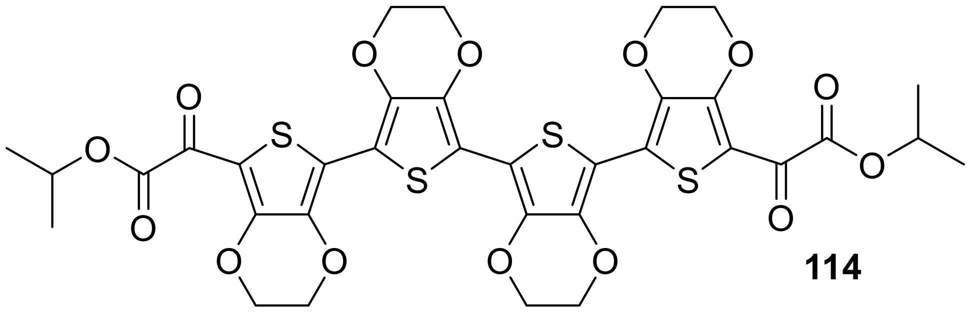

114

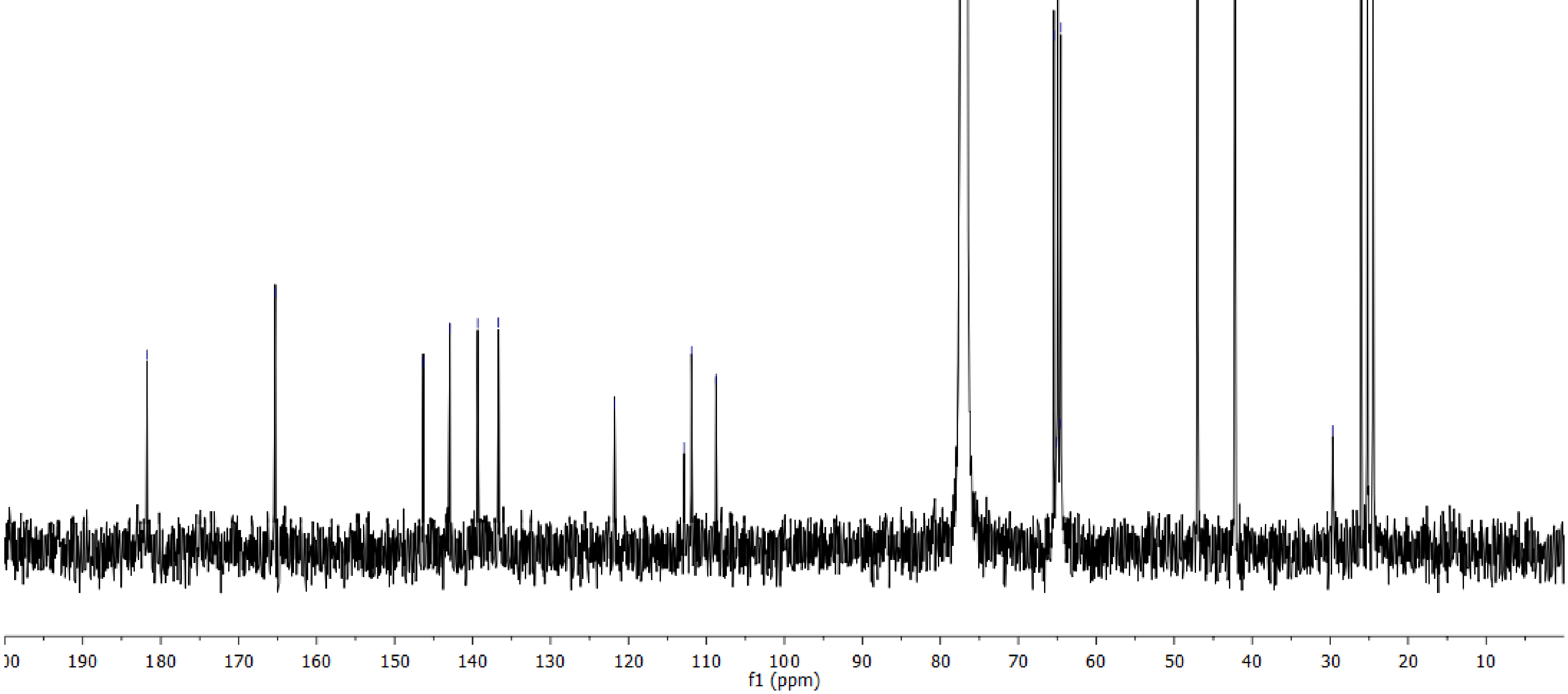

^1H NMR (400 MHz, $CDCl_3$)

Figure S162. ^1H NMR of **115**

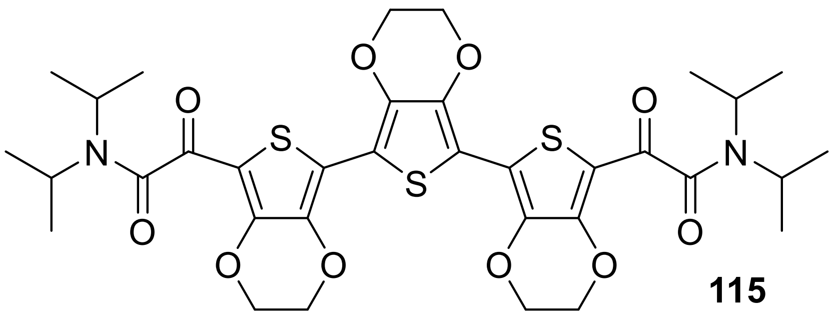

115

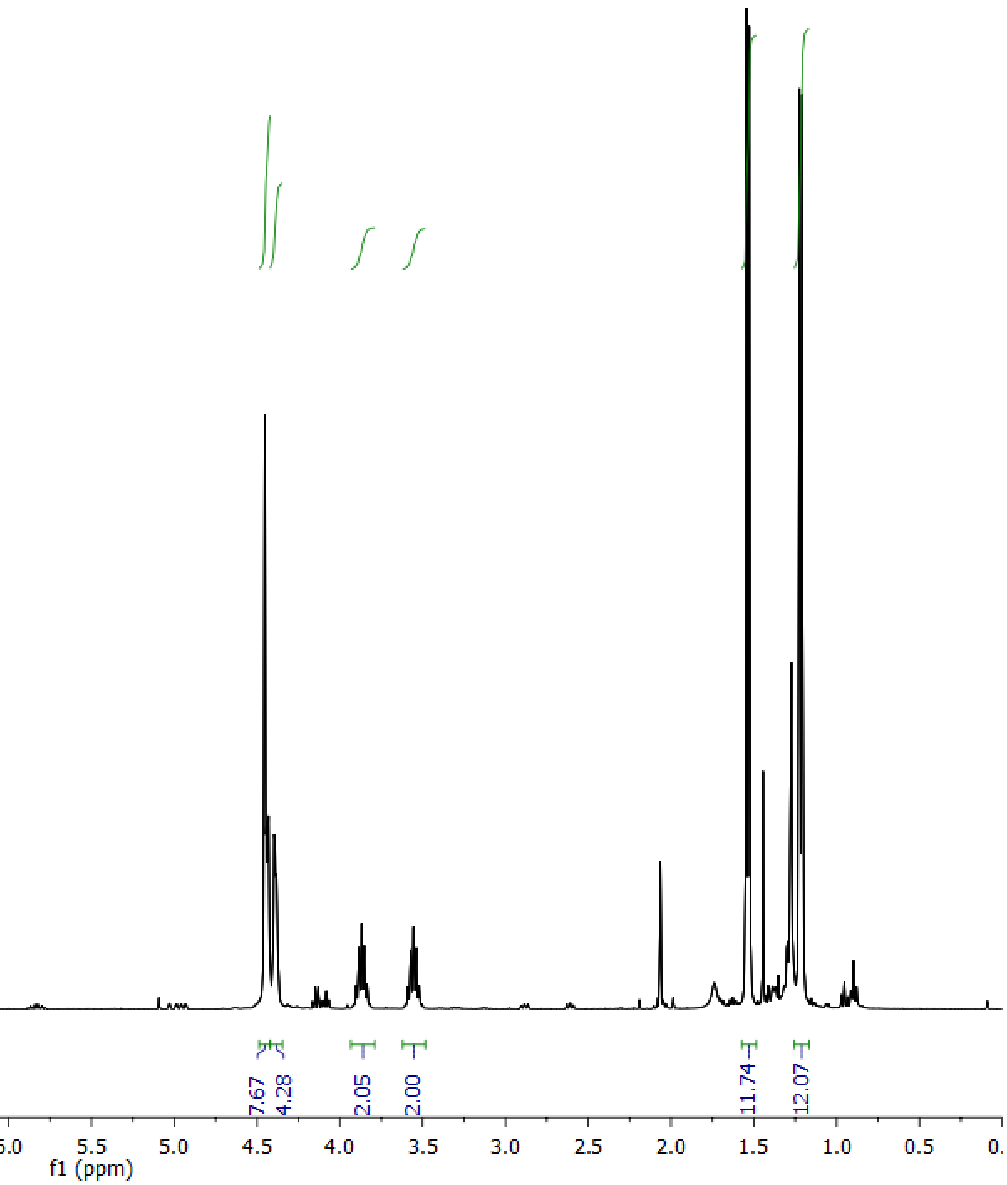

^{13}C NMR (100 MHz, $CDCl_3$)

Figure S163. ^{13}C NMR of **115**

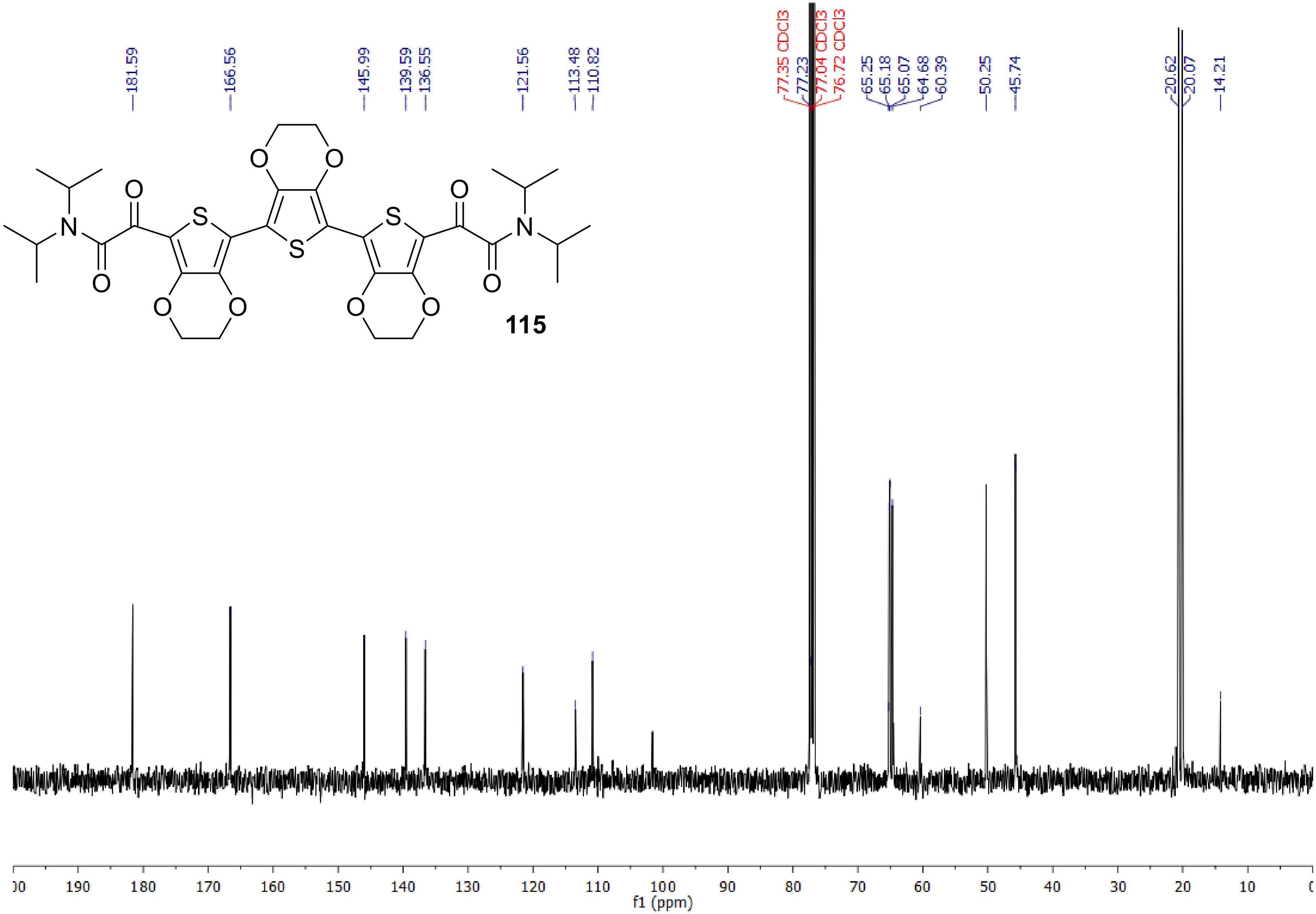

^{1}H NMR (400 MHz, $CDCl_3$)

Figure S164. ^{1}H NMR of **117**

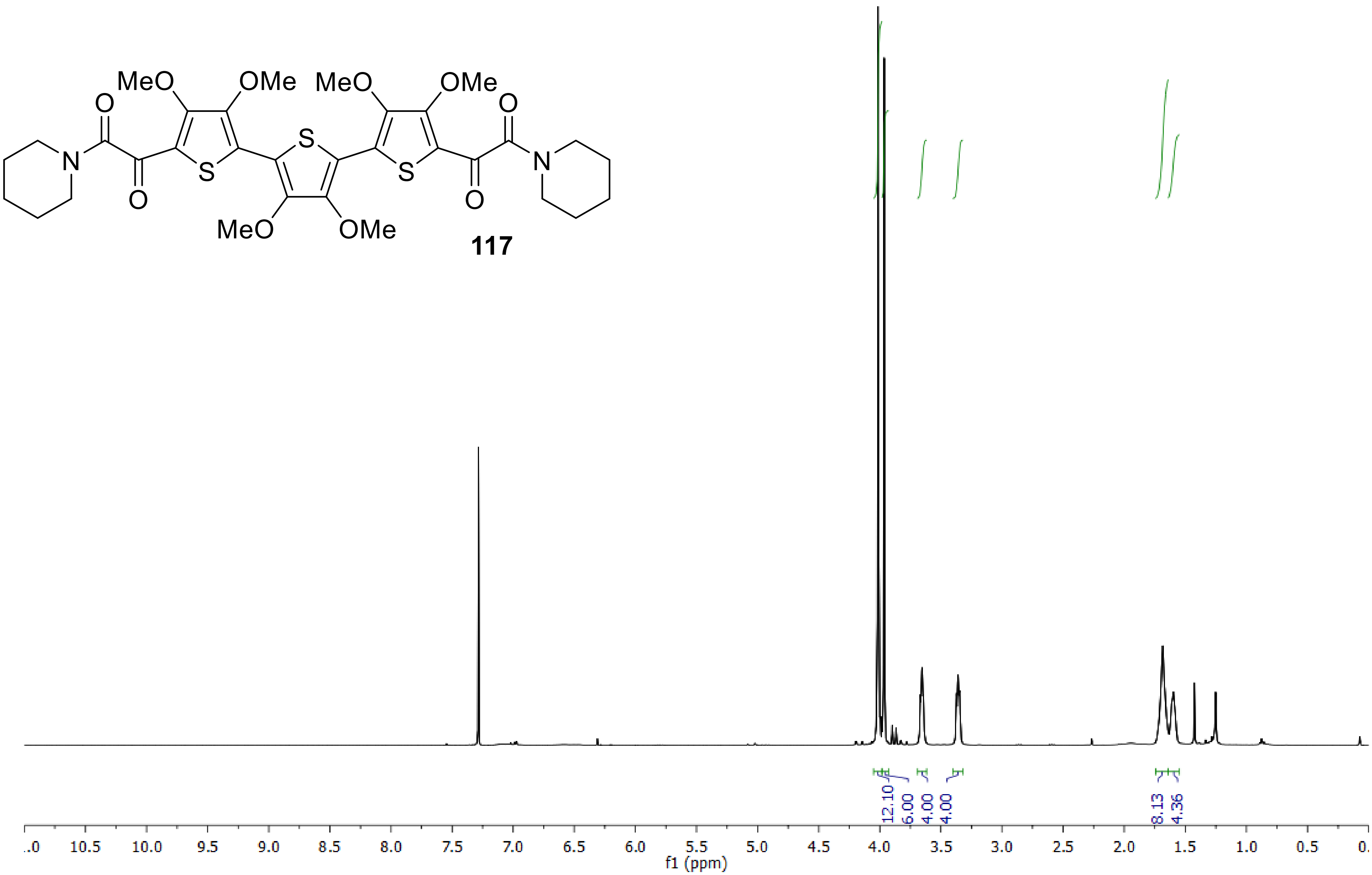

^{13}C NMR (100 MHz, $CDCl_3$)

Figure S165. ^{13}C NMR of **117**

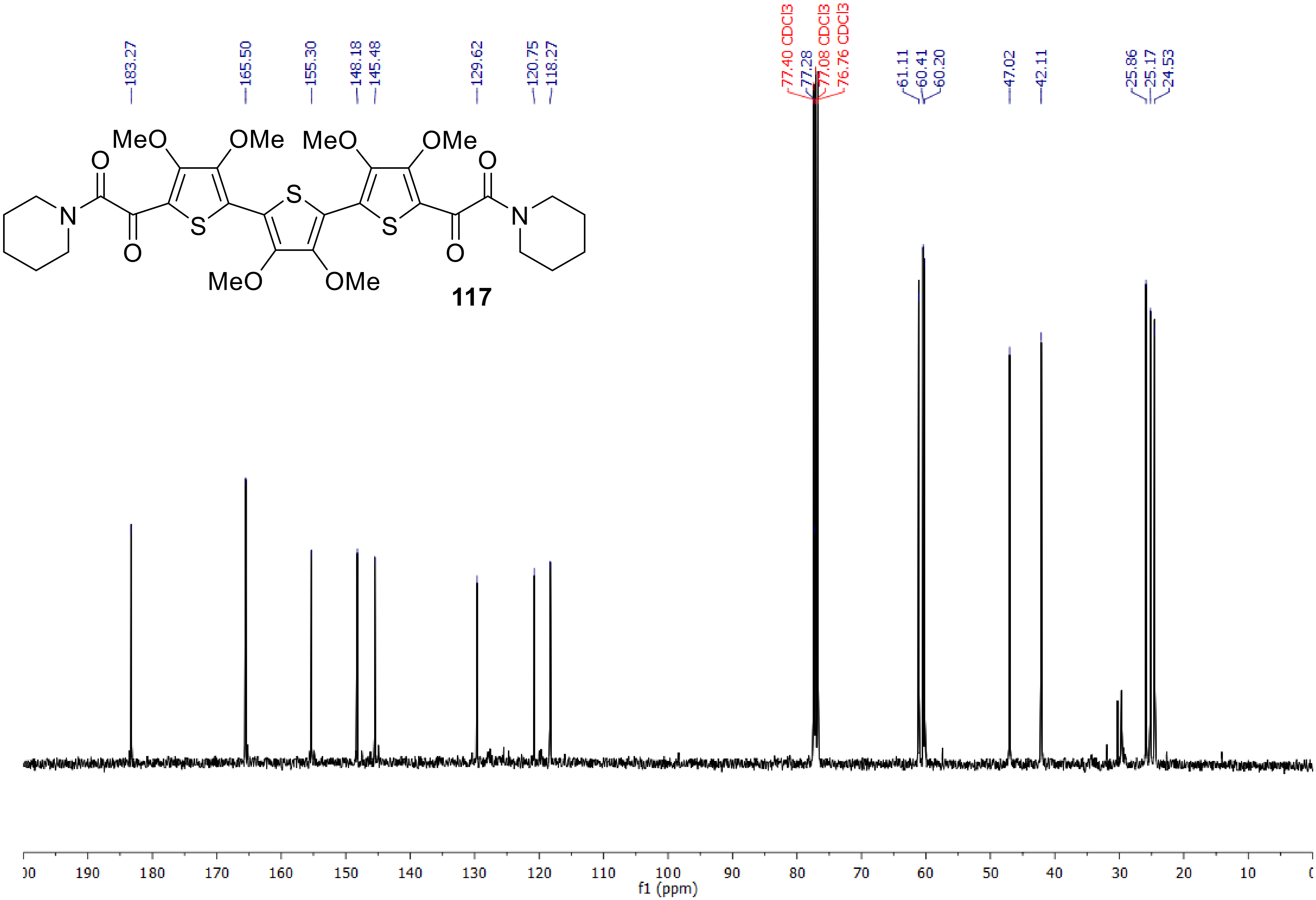

^{1}H NMR (400 MHz, $CDCl_3$)

Figure S166. ^{1}H NMR of **118**

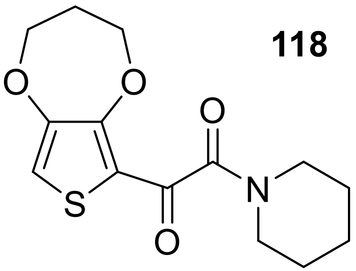

118

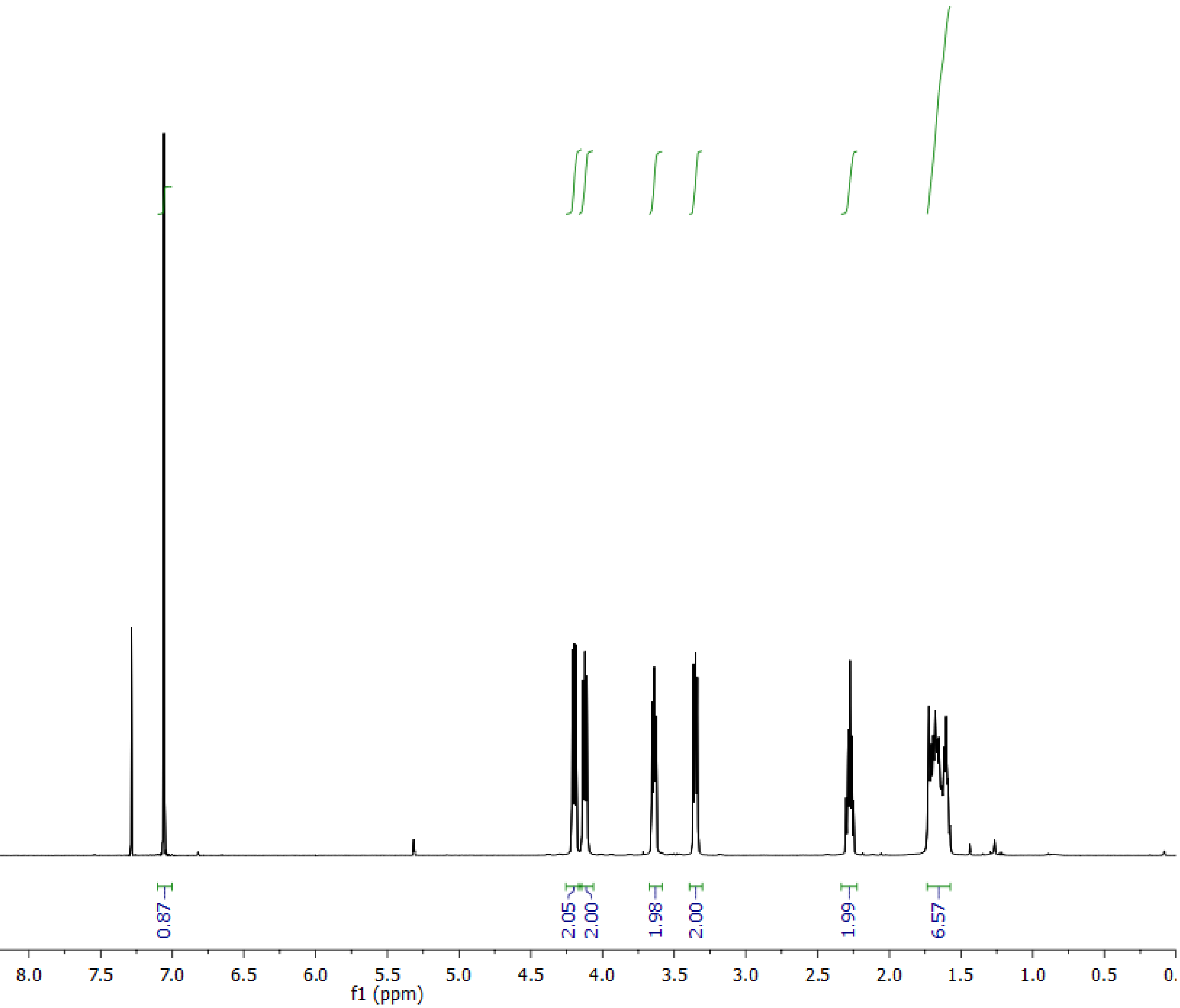

^{13}C NMR (100 MHz, $CDCl_3$)

Figure S167. ^{13}C NMR of **118**

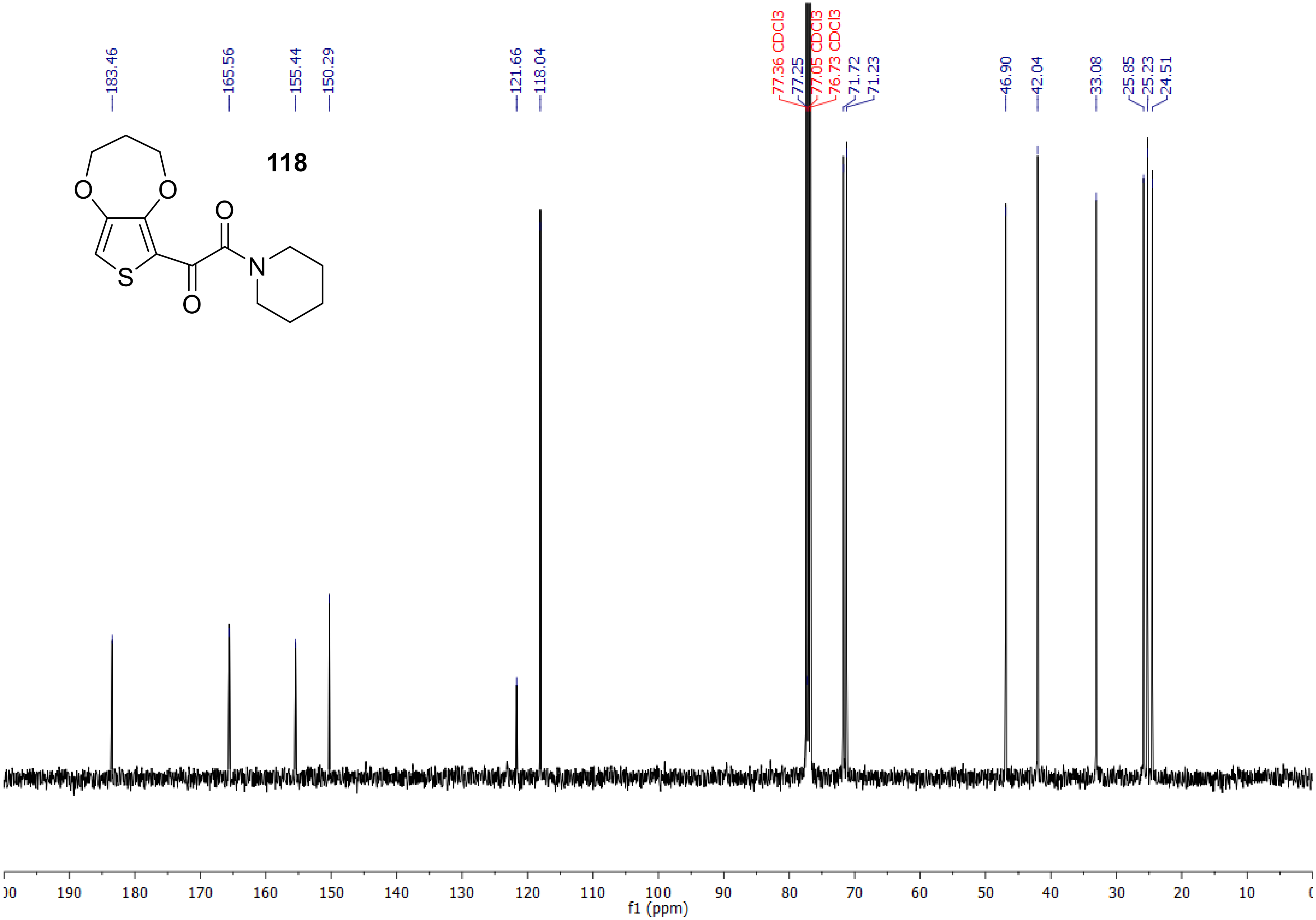

118

^{1}H NMR (400 MHz, $CDCl_3$)

Figure S168. ^{1}H NMR of **119**

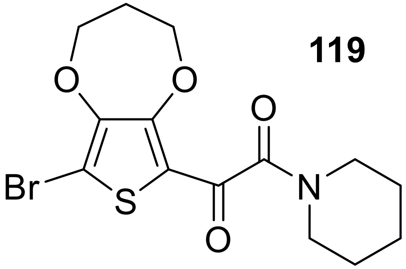

119

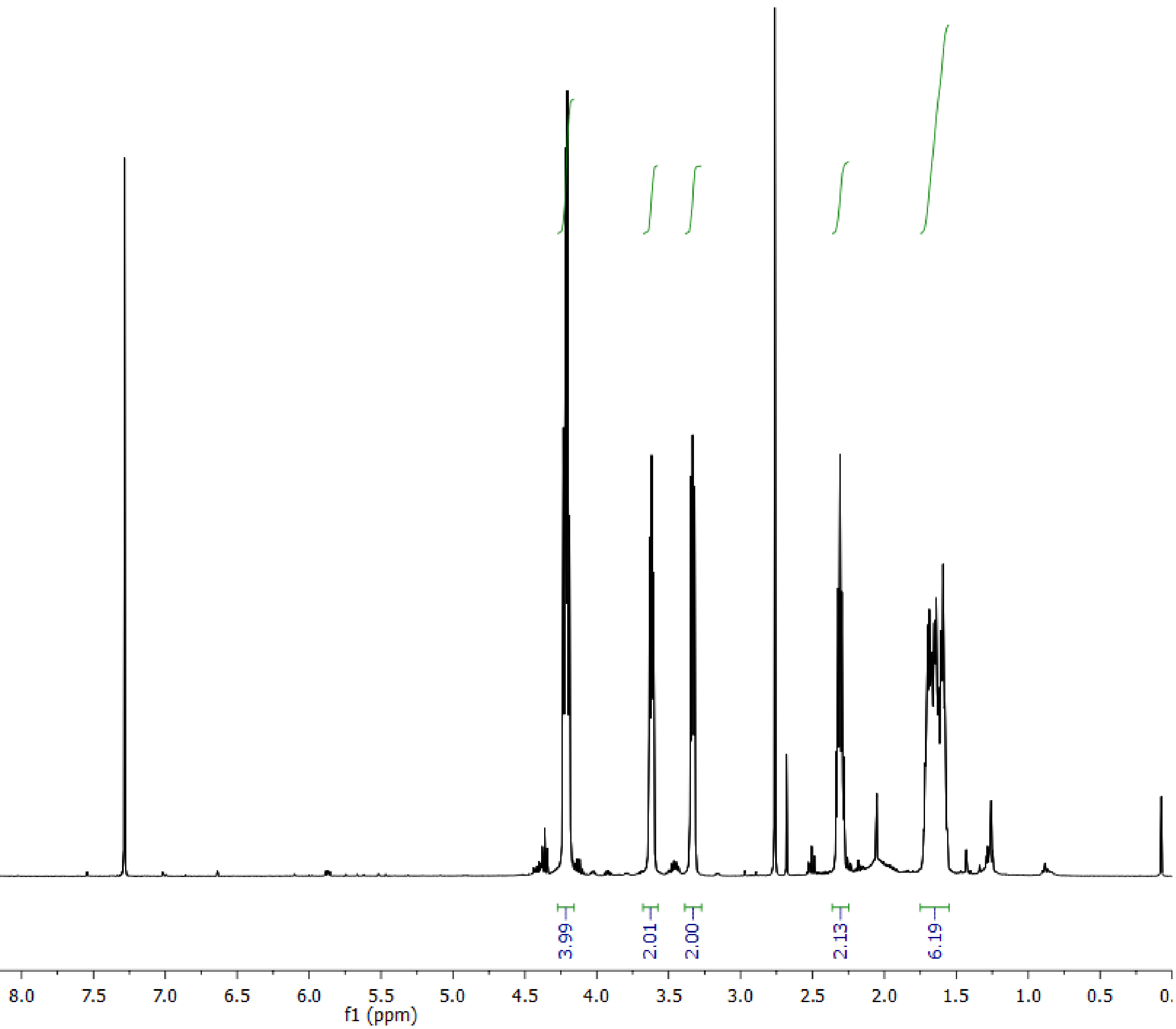

^{13}C NMR (100 MHz, $CDCl_3$)

Figure S169. ^{13}C NMR of **119**

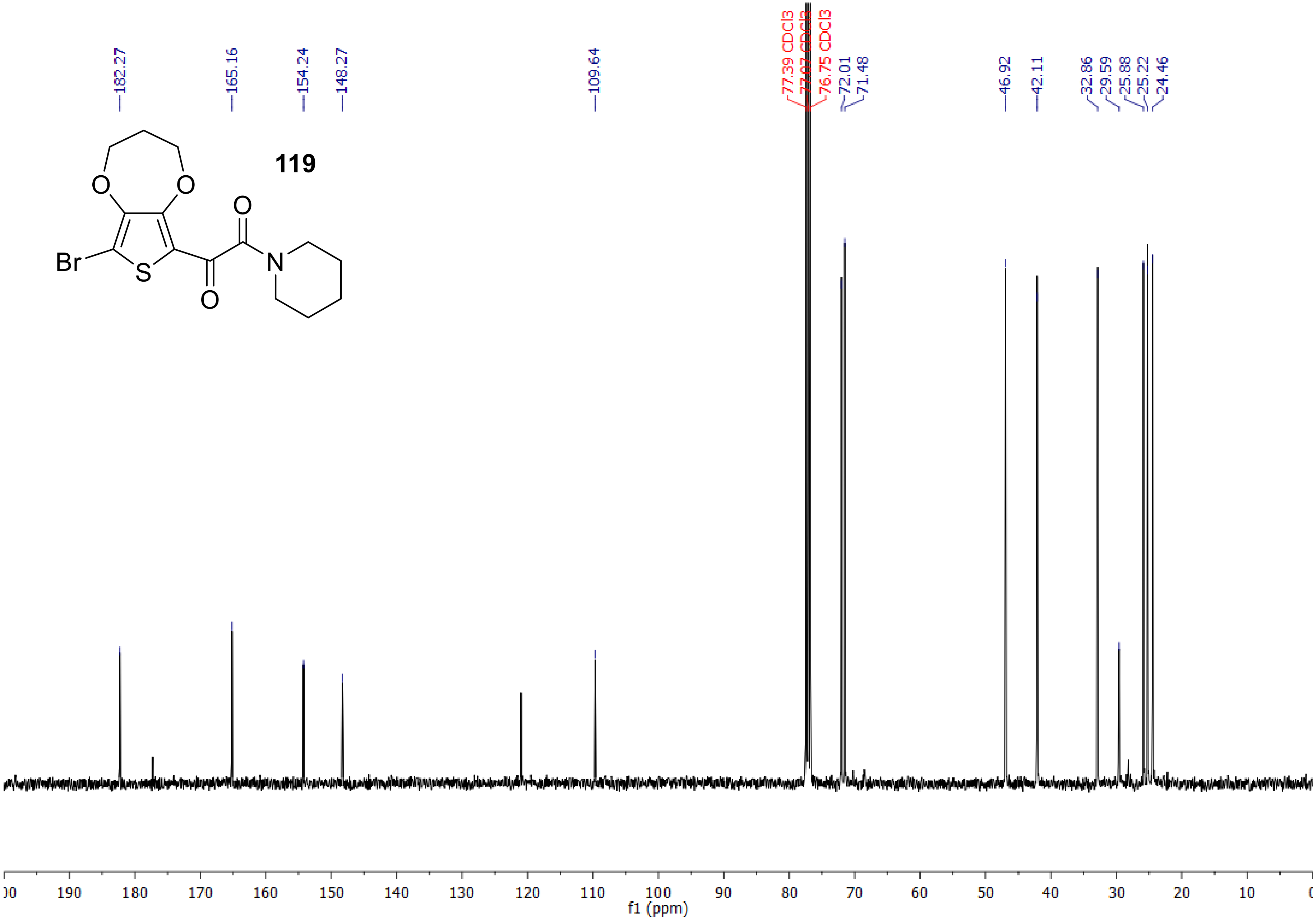

^{1}H NMR (400 MHz, $CDCl_3$)

Figure S170. ^{1}H NMR of **120**

120

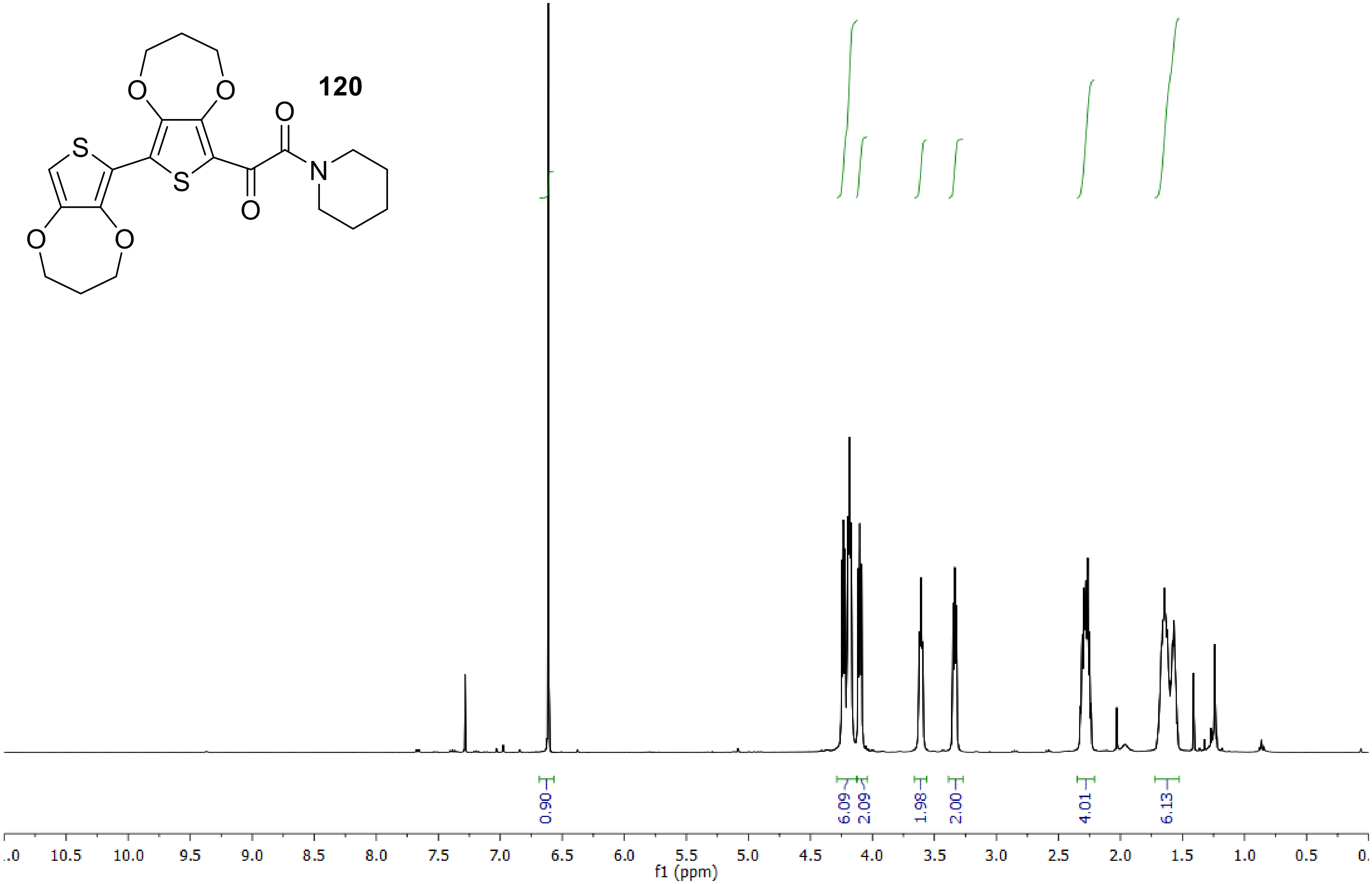

^{13}C NMR (100 MHz, $CDCl_3$)

Figure S171. ^{13}C NMR of **120**

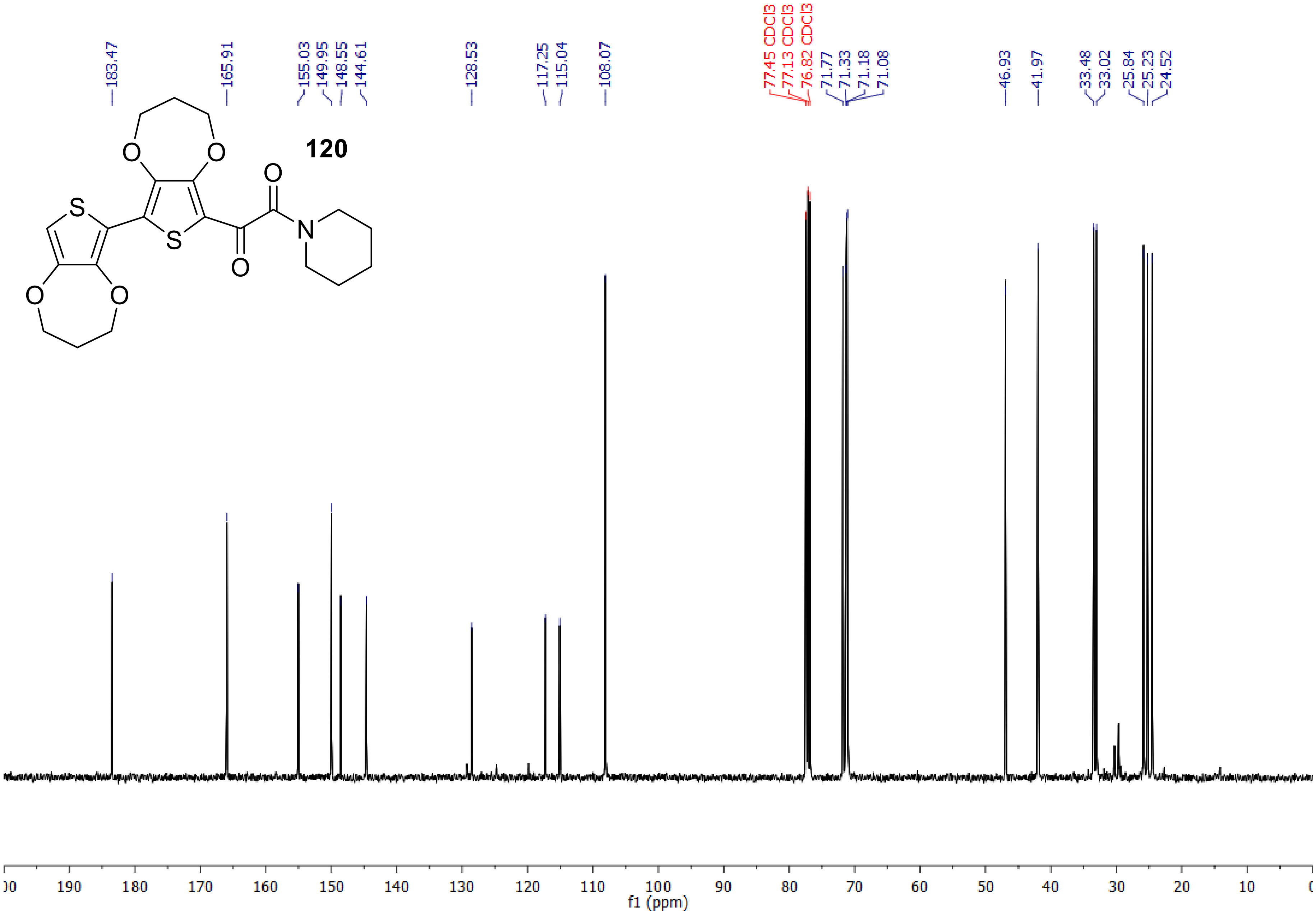

^{1}H NMR (400 MHz, $CDCl_3$)

Figure S172. ^{1}H NMR of **121**

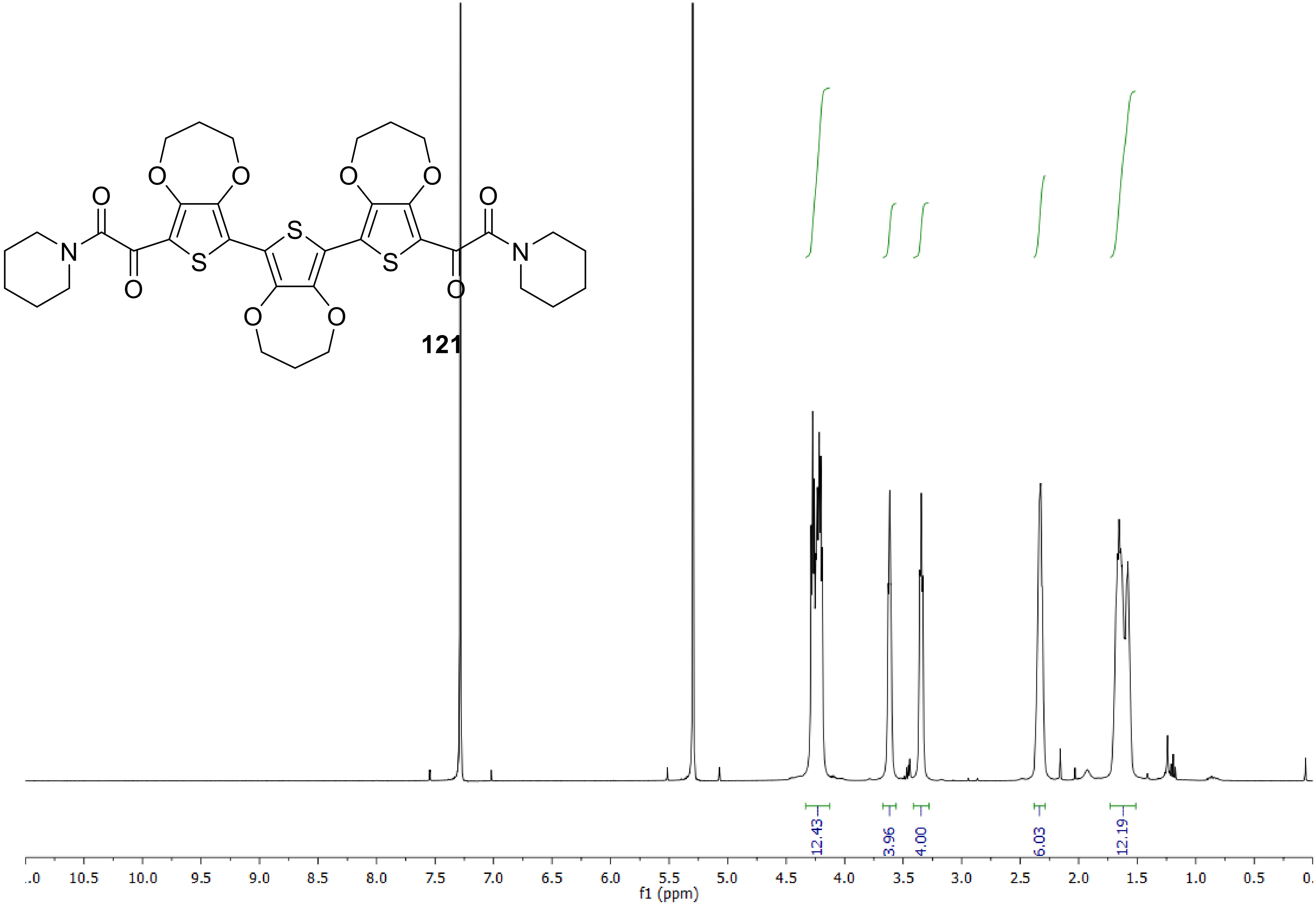

^{13}C NMR (100 MHz, $CDCl_3$)

Figure S173. ^{13}C NMR of **121**

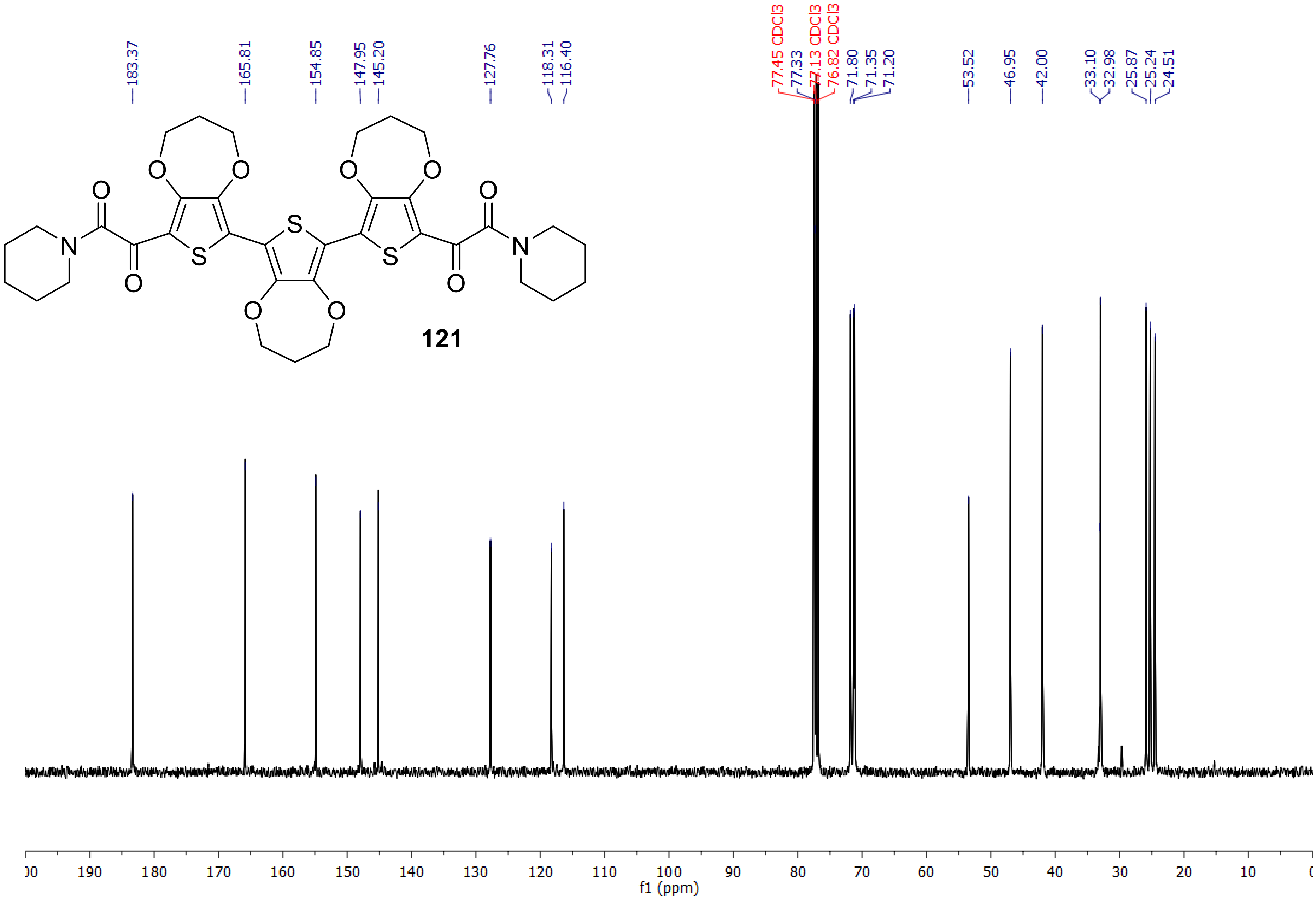

121
