# Supplementary material for: Synthesis of Hetero-bifunctional, End-Capped Oligo-EDOT Derivatives
Source: Chem. 2017 Jan 12;2(1):125–38. doi: 10.1016/j.chempr.2016.12.003 (PMC5268340; doi:10.1016/j.chempr.2016.12.003)

Chem

CellPress

Article

Synthesis of Hetero-bifunctional, End-Capped Oligo-EDOT Derivatives

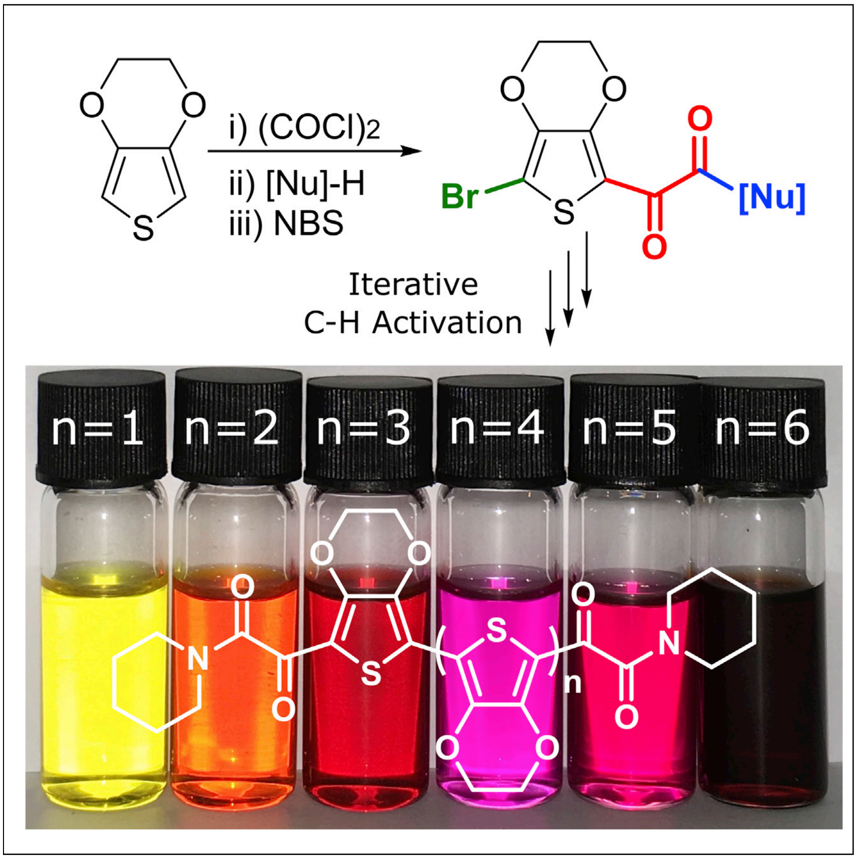

The synthesis and characterization of a series of keto-acid end-capped conjugated oligomers (n = 2–7) based around the monomer EDOT is reported. The use of direct arylation chain extension allows the synthesis of stable structures, which represent the longest reported EDOT oligomers to date with tunable properties based around the versatile end-capping group and monomer composition. These constructs can undergo subsequent derivatization, allowing them to be integrated into functional materials, such as those required for tissue engineering applications.

Christopher D. Spicer, Marsilea A. Booth, Damia Mawad, Astrid Armgarth, Christian B. Nielsen, Molly M. Stevens

m.stevens@imperial.ac.uk

HIGHLIGHTS

A keto-acid end-capping strategy has been used to create stable oligo-EDOTs

Oligomers can be synthesized in a facile manner via iterative direct arylation

Hetero-bifunctional and mixed-monomer constructs can be controllably synthesized

The keto-acid end group determines oligomer optical and electrochemical properties

3 GOOD HEALTH AND WELL-BEING

7 AFFORDABLE AND CLEAN ENERGY

Spicer et al., Chem *2*, 125–138
January 12, 2017 © 2017 The Author(s).
Published by Elsevier Inc.
http://dx.doi.org/10.1016/j.chempr.2016.12.003

CrossMark

Chem

CellPress

Article

Synthesis of Hetero-bifunctional, End-Capped Oligo-EDOT Derivatives

Christopher D. Spicer,[1] Marsilea A. Booth,[1] Damia Mawad,[1] Astrid Armgarth,[1] Christian B. Nielsen,[2] and Molly M. Stevens[1,3,*]

SUMMARY

Conjugated oligomers of 3,4-ethylenedioxythiophene (EDOT) are attractive materials for tissue engineering applications and as model systems for studying the properties of the widely used polymer poly(3,4-ethylenedioxythiophene). We report here the facile synthesis of a series of keto-acid end-capped oligo-EDOT derivatives (n = 2–7) through a combination of a glyoxylation end-capping strategy and iterative direct arylation chain extension. Importantly, these structures not only represent the longest oligo-EDOTs reported but are also bench stable, in contrast to previous reports on such oligomers. The constructs reported here can undergo subsequent derivatization for integration into higher-order architectures, such as those required for tissue engineering applications. The synthesis of hetero-bifunctional constructs, as well as those containing mixed-monomer units, is also reported, allowing further complexity to be installed in a controlled manner. Finally, we describe the optical and electrochemical properties of these oligomers and demonstrate the importance of the keto-acid in determining their characteristics.

The Bigger Picture

The production of materials that can aid the repair, regrowth, or replacement of damaged tissue is a key challenge in tissue engineering. In this context, conjugated polymers have been proposed as attractive materials for the engineering of electroactive tissues such as the heart. Although there has been much progress in the field, the use of conjugated polymers is still hindered by their high heterogeneity, stiffness, poor solubility, and lack of chemical functionality. Therefore, there is a pressing need to produce new routes to create constructs such as those reported here, which offer homogeneity, stability, ease of synthesis, and most importantly, flexibility of design. This versatility allows the incorporation of conjugated structures into the higher-order biomaterial architectures required for tissue engineering, as well as tunable solubility and material properties. It is anticipated that this report will open the door to an exciting new chapter in the use of EDOT in biology.

INTRODUCTION

Conjugated polymers (CPs) are promising materials for tissue engineering applications.[1–4] However, further developments are required in order to allow their full potential to be realized in the biomedical field. Although initial investigations have shown CPs to be able to modulate cellular growth,[5] migration,[6] and differentiation,[7,8] as well as protein adhesion and conformation,[9] difficulties remain as a consequence of their poor material characteristics, difficult processing, and lack of biodegradability.[1,2,10] Further, the production of constructs bearing reactive functionalities for integration into more complex scaffold architectures remains challenging.[2]

In order to address these issues, there is increasing interest in the use of oligomers rather than polymeric systems. Although oligomers are often more synthetically complex,[11] they offer the benefits of a defined molecular structure, improved solubility, tunability, and additional chemical functionality.[2,12] Oligomers can also act as mono-disperse model systems for studying the electronic and optical properties of the parent polymer, for which such investigations can be hindered.[13]

Poly(3,4-ethylenedioxythiophene) (PEDOT) is a particularly attractive material for tissue engineering because of its electrical and chemical stability and high conductivity when doped with polymeric ionomers such as polystyrene sulfonate.[14,15] Although the synthesis of thiophene-based oligomers has been widely reported,[11,16–19] those of EDOT (**1**; Scheme 1) have generated comparatively little interest, largely as a

© 2017 The Author(s). Published by Elsevier Inc.
This is an open access article under the CC BY license (http://creativecommons.org/licenses/by/4.0/).

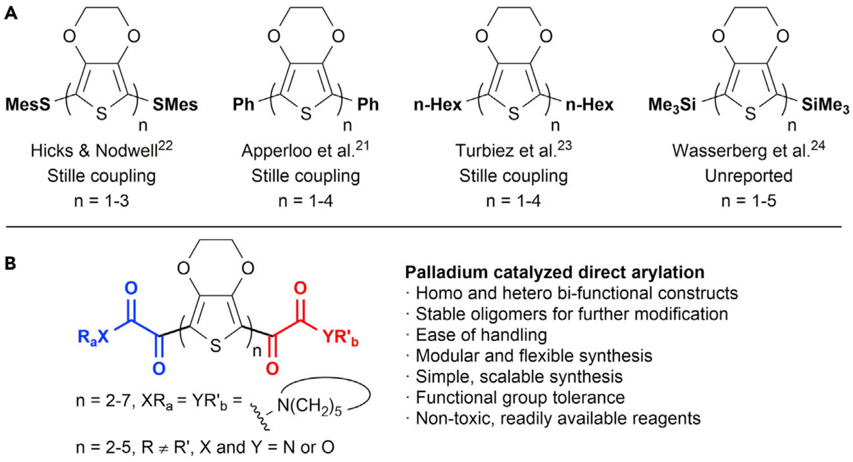

Figure 1. Previous EDOT End Caps and the Concept of This Report

(A) Previous reports of the synthesis of EDOT oligomers.

(B) Keto-acid-capped oligomers presented in this work. These oligomers were synthesized by direct arylation.

consequence of the poor oxidative stability and low solubility of the oligomers.[20,21] Mesityl,[22] phenyl,[21] *n*-hexyl,[23] and trimethylsilyl[24] capping groups have all been reported. However, longer oligomers were found to be unstable in solution, very poorly soluble, and difficult to purify, limiting their utility. Indeed, there remains only a single report on the synthesis of a pentameric species, but no synthetic details were reported[24] (Figure 1A). Furthermore, the end caps utilized offer no opportunities for further chemical derivatization and subsequent incorporation into more complex structures.

Here, we report the facile synthesis and characterization of bench-stable oligo-EDOT derivatives, up to n = 7, produced via a glyoxylation keto-acid end-capping strategy and iterative C–H activation chemistry. Importantly, this allows the production of hetero-bifunctional constructs with a wide range of functional handles for further modification (Figure 1B). These motifs allow additional integration into more challenging substrates, such as those required for tissue engineering applications.

RESULTS AND DISCUSSION

Oligomer Synthesis

Our initial designs were inspired by reports of thiophene glyoxylation with oxalyl chloride.[25] We reasoned that the intermediate glyoxylyl chloride **2** could be reacted in situ with a range of nucleophiles to generate α-functionalized EDOT derivatives (Scheme 1). Importantly, the choice of nucleophile would have little influence on aromatic stability, allowing for a range of diverse constructs to be produced. After treatment of EDOT with 1 equiv of oxalyl chloride, the intermediate chloride **2** reacted smoothly with piperidine to generate the tertiary keto-amide **3** (Scheme 1A; Figures S6 and S7) in good yield. Subsequent bromination with *N*-bromosuccinimide yielded the di-functional monomer **4** on a multi-gram scale (Figures S8 and S9).[22]

A range of functionalized monomers could be produced by this method, including secondary amines (**5**), hindered tertiary amines (**6**), esters (**7**, **8**, and **9**), and monomers bearing functional groups for further modifications (Scheme 1, route A; Figures S10–S19, S73, S74, S91, S92, S99, S100, and S129–S132). In addition, hydrolysis of

[1]Departments of Materials and Bioengineering, Institute of Biomedical Engineering, Imperial College London, London SW7 2AZ, UK

[2]Materials Research Institute and School of Biological and Chemical Sciences, Queen Mary University of London, London E1 4NS, UK

[3]Lead Contact

*Correspondence: m.stevens@imperial.ac.uk

http://dx.doi.org/10.1016/j.chempr.2016.12.003

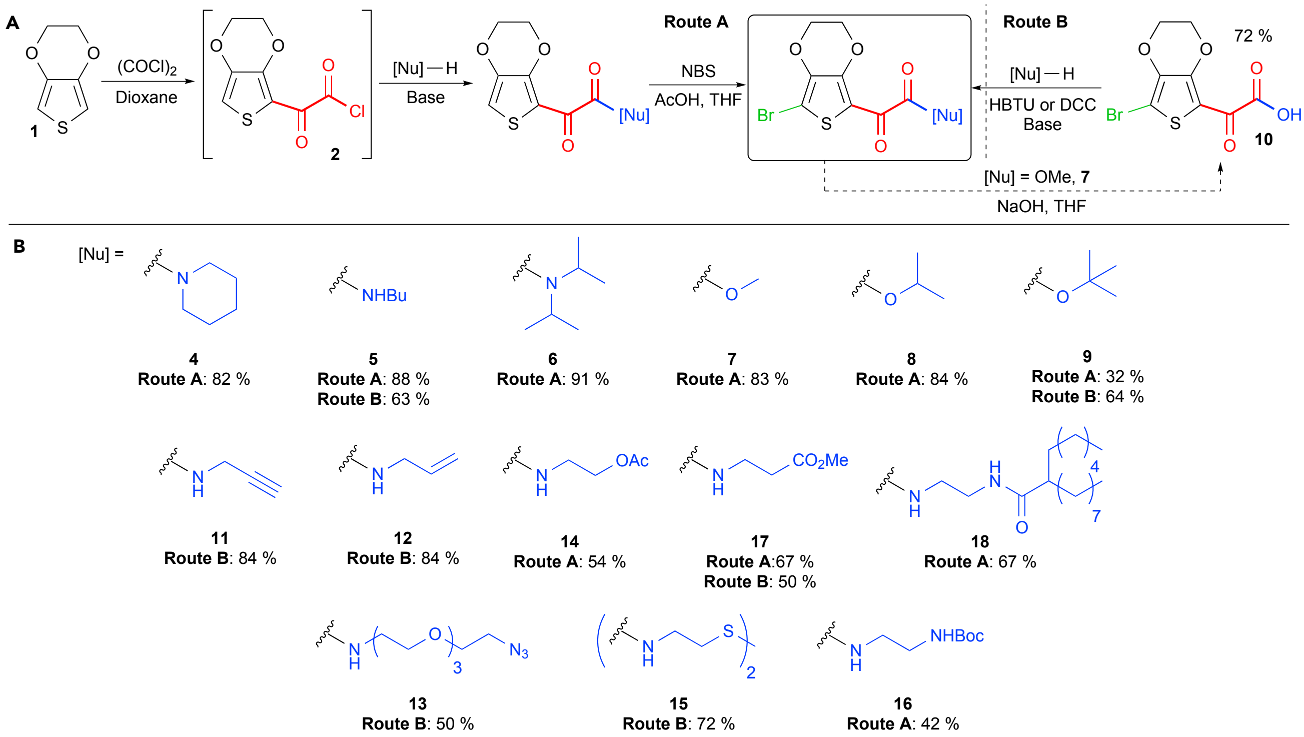

Scheme 1. EDOT Glyoxylation and Functionalization

(A) Treatment of EDOT with oxalyl chloride and subsequent treatment with the desired nucleophile generates functional end-capped derivatives, which can then undergo bromination (route A). Alternatively, amide or ester coupling can be undertaken from a common intermediate **10** to give the desired monomers (route B).

(B) Functionalized brominated monomers synthesized.

brominated-EDOT methyl ester **7** and subsequent amide or ester coupling allowed the synthesis of a range of di-functional monomers from a common intermediate **10** (Scheme 1, route B; Figures S20 and S21). Thus, monomers containing orthogonal reactive groups for further conjugation, such as alkynes (**11**), alkenes (**12**), azides (**13**), and protected alcohols (**14**), thiols (**15**), and amines (**16**), could all be produced in good yields in a simple fashion (Figures S22–S37, S125–S128, and S133–S150).[26]

Next, we investigated the chain extension of brominated monomer **4** to form dimer **19**. The most popular strategies for undertaking such reactions utilize Kumada,[27] Negishi,[28] or Stille[29] couplings. However, problems such as poor functional-group tolerance, monomer instability, and high reagent toxicity result in significant limitations, particularly for use in biological applications.[22,30,31] As such, we chose to investigate the use of direct arylation, which has emerged in recent years as a powerful tool for constructing conjugated systems.[32,33] Pleasingly, **4** was found to be partially converted to **19** in the presence of 1.5 equiv of EDOT **1** in *N*,*N*-dimethylformamide (DMF) at 130°C for 1 hr (Scheme 2A; Figures S38 and S39). Importantly, the reaction was catalyzed by a readily available combination of $Pd(OAc)_2$, pivalic acid, and potassium carbonate, thus negating the need for expensive or air-sensitive catalysts and ligands or the use of specialist techniques.[34]

Investigating the reaction further, we found yields to be increased significantly through the use of 4 equiv of EDOT, the excess of which could be readily re-isolated through column chromatography. At lower loadings, a significant amount of the

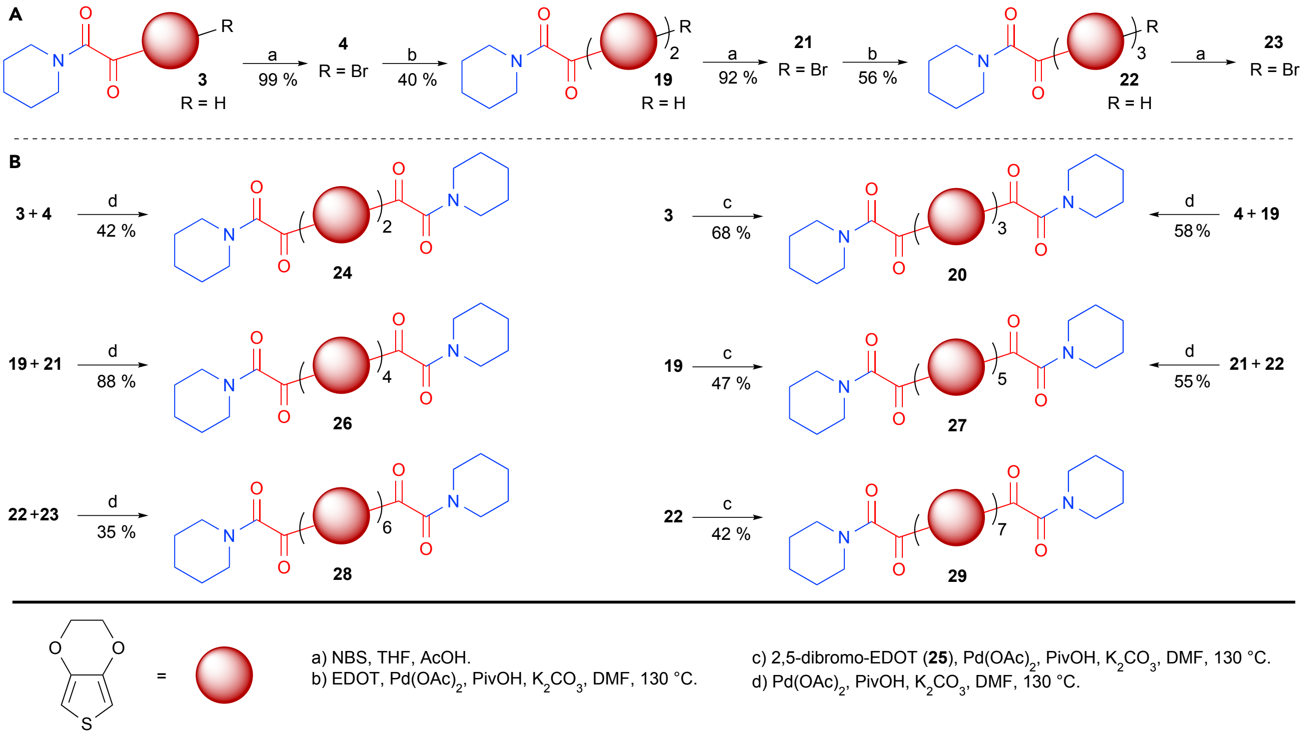

Scheme 2. Synthesis of Piperidine End-Capped Homo-bifunctional EDOT Oligomers

(A) Chain extension of mono-functional (n = 1–3) piperidine-capped oligomers.

(B) Convergent coupling to generate bifunctional piperidine end-capped constructs (n = 2–7).

symmetrical di-capped trimer **20** was produced as a result of further reaction of **19** with **4** (Figures S40 and S41). Although small amounts of this side product were still produced at higher EDOT loadings, yields were significantly lowered, and separation was readily achieved. Further iterations of bromination and direct arylation allowed the production of brominated dimer **21** and trimer **22** on a gram scale, both of which were found to be bench stable (Figures S42–S45). Bromination to form brominated trimer **23** was also possible, although its low solubility and stability prevented characterization and required its immediate use once prepared, as discussed later.

With these mono-capped building blocks in hand, we investigated the synthesis of di-capped oligomers (Scheme 2B). Heating a mixture of brominated and non-brominated monomers **4** and **3** (1.1 equiv) under the same conditions required for chain extension cleanly produced di-capped dimer **24** (Figures S46 and S47). Similarly, trimer **20** was produced from **4** and dimer **19**. Alternatively, **20** could be produced from the reaction of 2 equiv of either monomer **3** or brominated monomer **4** with 2,5-dibromo-EDOT **25** or EDOT **1**, respectively, in an optimized version of the previously discussed chain-extension side reaction.

By suitable choice of starting materials, di-capped oligomers (n = 2–5; **24**, **20**, **26**, and **27**) were all readily produced and easily isolated by column chromatography (Figures S48–S50). Extending the scope further to the use of brominated trimer **23**, used immediately without purification, allowed the synthesis of hexamer **28**, whereas coupling of trimer **22** with 2,5-dibromo-EDOT **25** allowed the synthesis of heptamer **29**, the first time the synthesis of EDOT oligomers of such lengths has

been reported (Figures S51 and S52). Oligomers up to n = 6 were found to be bench and air stable and therefore could be easily handled, purified, and analyzed; no change in structure was observed by UV-Vis or ^{1}H-NMR spectroscopy after 2 months of storage at room temperature. Heptamer **29** was produced with reduced purity (~80% as judged by ^{1}H NMR) but retained stability. Although oligomers of n = 2–5 were also found to be stable in solution, after long periods in chlorinated solvents (>2 weeks), a broadened UV-Vis absorption indicated that hexamer **28** and heptamer **29** had undergone partial degradation.

Oligomer solubility was found to decrease with increasing chain length, and aggregation in solution became significant at longer lengths. However, it remained high enough to allow manipulation in solution and the use of typical synthetic techniques such as phase extraction and column chromatography. Oligomers of n = 2–5 were soluble at concentrations of >20 mM in dichloromethane (DCM), and hexamer **28** was soluble at concentrations of >5 mM, whereas heptamer **29** could be solubilized at concentrations up to 0.5 mM. It is important to note that solubility is strongly influenced by the choice of end group and can be readily improved by the introduction of a flexible solubilizing linker to the functional group of interest, as discussed later. Finally, we analyzed oligomers **20** and **26** by inductively coupled plasma mass spectrometry (ICP-MS) to determine the levels of residual palladium present. As for other heavy metals, palladium contamination in pharmaceuticals and biomedical devices is tightly regulated because of the potential for toxic side effects. Palladium contamination was found to be at a low level of 7.4 $\pm$ 0.5 ppm for trimer **20** and 1.2 $\pm$ 0.5 ppm for tetramer **26**. Although it is difficult to make a direct comparison between a substrate intended for applications in tissue engineering and an active pharmaceutical ingredient (API), it is useful to note that these low levels of contamination are below the 10 ppm limit set by the International Council on Harmonisation of Technical Requirements for Registration of Pharmaceuticals for Human Use and the US Pharmacopeia for acceptable levels of palladium in APIs.[35] Furthermore, because no extensive effort was taken to remove palladium from the samples, it is likely that these levels could be reduced further. For example, the use of palladium chelators during purification or the use of heterogeneous catalysts would be expected to lead to a significant reduction in contamination in any structures intended for biological applications.[36–38]

Although the ability to create symmetrical oligo-EDOTs with non-functional end groups is a useful tool for modeling the properties of PEDOT, the true utility of the method described above for the synthesis of di-piperidine-capped oligomers is in the synthesis of hetero-bifunctional constructs, which can be selectively derivatized at both ends, allowing their integration into more complex architectures. To demonstrate this, we first synthesized a series of unsymmetrical oligomers capped with a piperidine motif at one terminus and diisopropylamine at the other (see Scheme S1). Coupling differently terminated oligomers as described above produced oligomers of n = 2–5 (**30–33**) in a limited number of steps (Figures S53–S59 and S93–S98).

During these experiments, a number of observations were made. Firstly, although a temperature of 130°C was required for the chain extension and oligomer synthesis with brominated piperidine-based species, for diisopropyl-functionalized oligomers, 90°C was found to be sufficient to give complete conversion within 1 hr of reaction, leading to cleaner reaction products. Indeed, for all other end-capping groups investigated during this work, 90°C was high enough to facilitate reaction.[39] Secondly, although couplings generally proceeded cleanly, the amount of side

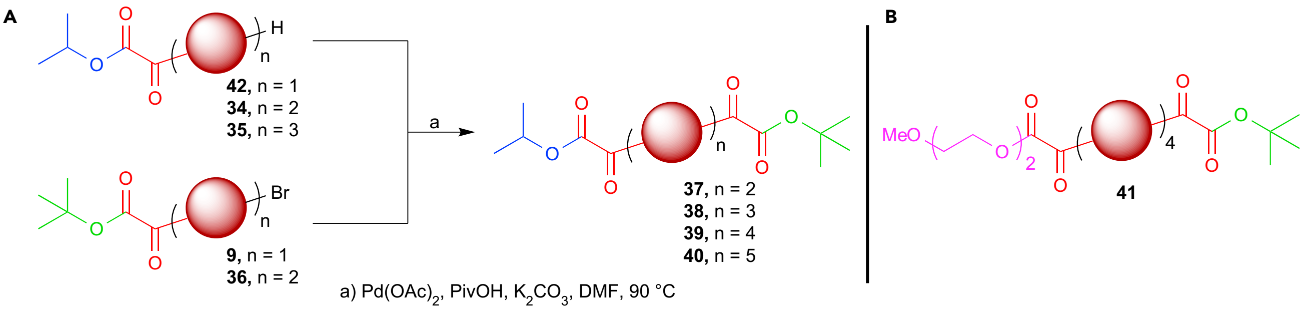

Scheme 3. Synthesis of Orthogonal-Ester-Functionalized Hetero-bifunctional Oligomers

(A) Synthesis of unsymmetrical, orthogonally protected oligo-EDOT diesters **37**–**40** with *iso*-propyl and *tert*-butyl end groups.
(B) Triethylene glycol ester-capped tetra-EDOT **41** with improved solubility.

products produced increased with increasing oligomer length. The major side product was found to stem from the instability of the brominated species, resulting in partial dehalogenation and subsequent homo-coupling and, to a lesser extent, homo-coupling of the non-brominated reaction partner. Such side reactions have been studied extensively[40] and are also known to occur during Stille and Suzuki polymerizations.[41] Although outside the scope of this work (which focuses on the use of unoptimized, simple, and cheap catalyst systems), it is likely that such products could be minimized through judicious choice of both metal and ligand.[42]

To create functional oligomers primed for further reaction and derivatization, we considered that a number of common reactive handles would not be amenable to the chain extension and bromination procedures described above.[43] It would therefore be advantageous to be able to install functionality at a late stage after oligomer synthesis. Thus, we investigated the use of orthogonal ester-protecting groups to provide latent functionality. Initial attempts to react methyl ester **7** with an excess of EDOT **1** led not only to chain extension but also to a significant amount (~40%) of ester cleavage (see Scheme S2A). However, switching to *iso*-propyl ester **8** lead to a clean conversion to dimer **34** at 90°C, followed by subsequent bromination and extension to yield trimer **35** (reaction at 130°C as described for piperidine oligomers led to complete ester cleavage; see Scheme S2B; Figures S60–S63, S101, and S102). Similarly, the orthogonally protected *tert*-butyl ester **9** could undergo iterative chain extension and bromination to yield brominated dimer **36** (Figures S64, S65, and S111–S114).

With these substrates in hand, we were able to synthesize di-capped, orthogonally protected oligomers **37**–**40** with n = 2–5 in a short number of steps and in good yields (Scheme 3A; see Scheme S2C for full details and Figures S66–S71). Although the synthesis of tetramer **39** and pentamer **40** was confirmed by mass spectrometry, the propensity of the constructs to aggregate in solution prevented analysis by ^{13}C NMR. As an alternative, constructs possessing a solubility-enhancing triethylene glycol chain could also be produced as discussed above (**41**; Scheme 3B; Figures S72 and S103–S110). Here, the significant difference in end-group polarity greatly aided purification, offering a potential means of enhancing purity during particularly difficult separations. This representative example demonstrates an important advantage of the synthesis reported in this work; because the choice of end group is an important determinant in the material properties of the synthesized constructs, simply choosing an appropriate end cap can alter factors such as the solubility of the material to reflect the desired application.

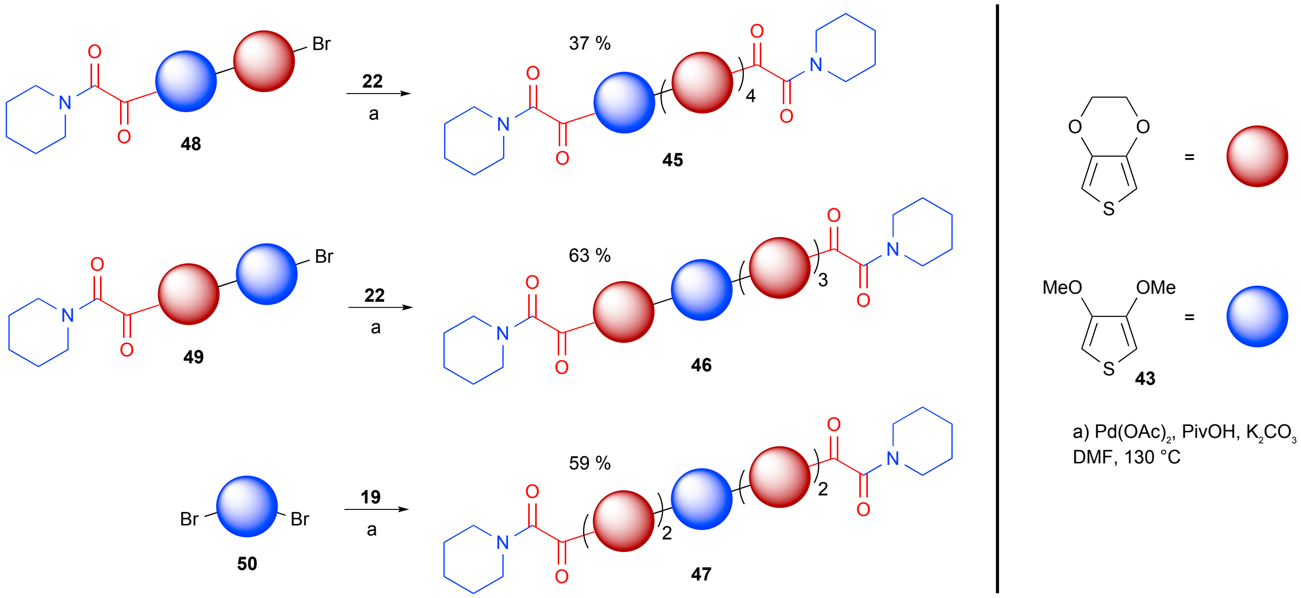

Scheme 4. Synthesis of Dimethoxythiophene-Containing Isomers

Pentameric EDOT oligomers containing a single DMT unit were synthesized for the generation of the structural isomers **45**, **46**, and **47**.

Amide coupling after sequential ester deprotection, first in the presence of trifluoroacetic acid to remove the *tert*-butyl group and then in the presence of sodium hydroxide to cleave the *iso*-propyl ester, allowed the subsequent synthesis of unsymmetrical constructs bearing reactive functionality for further modification (see Scheme S3; Figures S115 and S116). As a result of the mild amide- or ester-forming conditions required, this method is applicable to the late-stage hetero-functionalization of the oligomers reported with a wide range of reactive or functional groups, such as those shown in Scheme 1. The potential applications of this methodology are diverse. The ability to create hetero-bifunctional oligomers of a tunable length and bearing handles for further modification allows the modular synthesis of more complex structures. For example, the integration of such constructs into biologically active scaffolds[2] or the production of amphiphilic, self-assembling morphologies[44,45] offers exciting possibilities in the fields of both the material and biomedical sciences.

Finally, we wished to investigate the application of our methodology to the synthesis of mixed oligomers composed of different monomer units, which could possess interesting properties. In particular, we considered the rigidity of EDOT oligomers, which are known to lead to highly planar structures with enhanced π conjugation.[23] We reasoned that disrupting planarity in a controlled fashion could tune the properties of the resultant material. Structurally related dialkoxythiophene monomers such as 3,4-dimethoxythiophene (DMT, **43**) and 3,4-propylenedioxythiophene (ProDOT, **44**) were found to be suitable substrates for our glyoxylation and chain-extension procedures. We therefore introduced a single DMT moiety in an EDOT-pentameric structure to create three structural isomers: **45**, **46**, and **47** (Scheme 4; see Scheme S4 for full details and Figures S75–S83 and S117–S124). The simple manner in which such compounds can be created allows the rapid construction of a library of dialkoxythiophene-based constructs for investigating the effects of structure, substituents, and isomerization on the chemical and electrical properties of CPs.

A

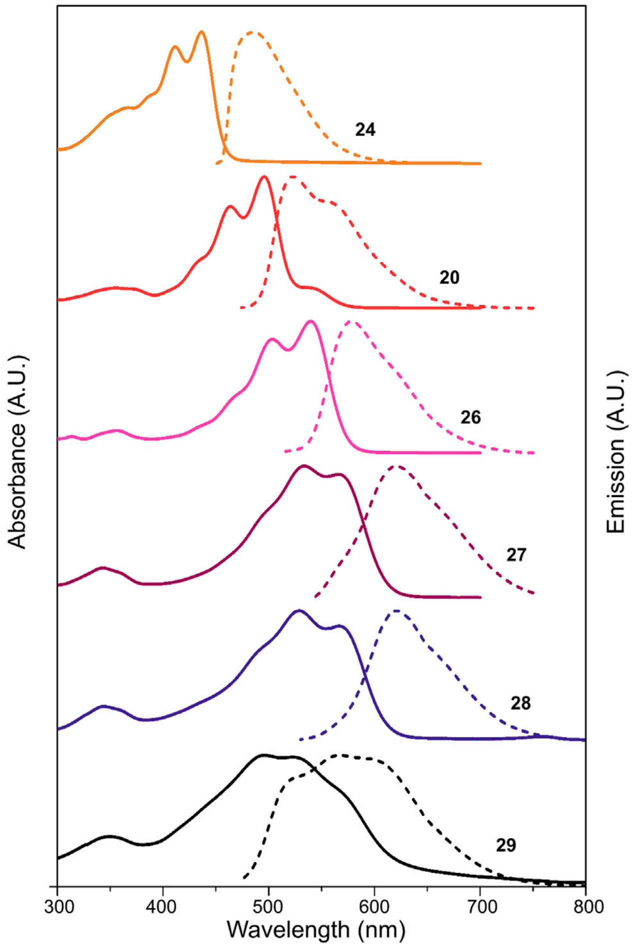

B

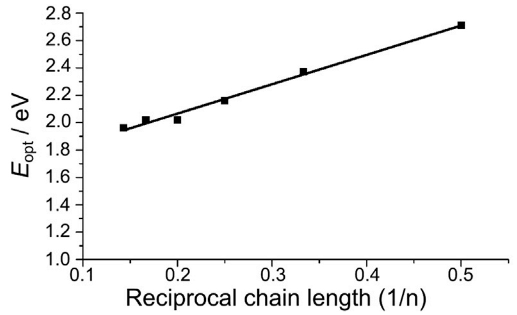

C

Entry	Compound	End-group	Monomer	Length	E_{opt}
1	**24**	Piperidine	EDOT	2	2.71 eV
2	**20**	Piperidine	EDOT	3	2.37 eV
3	**26**	Piperidine	EDOT	4	2.16 eV
4	**27**	Piperidine	EDOT	5	2.02 eV
5	**28**	Piperidine	EDOT	6	2.02 eV
6	**29**	Piperidine	EDOT	7	1.96 eV
7	**53**	-N*i*Pr_2	EDOT	5	2.02 eV
8	**54**	-NHBu	EDOT	5	1.92 eV
9	**55**	-O*i*Pr	EDOT	3	2.30 eV
10	**56**	-O*i*Pr	EDOT	5	1.88 eV
11	**45**	Piperidine	DMT/EDOT	5	2.03 eV
12	**46**	Piperidine	DMT/EDOT	5	2.06 eV
13	**47**	Piperidine	DMT/EDOT	5	2.08 eV
14	**57**	Piperidine	DMT	5	2.14 eV
15	**58**	Piperidine	ProDOT	5	2.09 eV

Figure 2. Oligomer Optical Characterization

(A) Normalized UV-Vis (solid line) and fluorescence (dashed line) spectra of di-piperidine-capped oligomers **24**, **20**, and **26**–**29**.

(B) Correlation of inverse chain length and E_{opt} for oligomers **24**, **20**, and **26**–**29** (adjusted R^2 = 0.9828).

(C) Summary of E_{opt} for a series of di-functionalized oligomers.

Oligomer Characterization

Solutions of the di-piperidine-capped oligomers described above (**24**, **20**, and **26**–**29**) in DCM were analyzed by UV-Vis and fluorescence spectroscopy. Within the range investigated, the optical properties of the materials were found to be independent of concentration, indicating that aggregation was not occurring. As expected, a gradual red shift in the onset of absorbance was observed with increasing chain length (Figure 2), although a blue shift in absorbance maxima for heptamer **29** was observed, most likely because of the presence of impurities in the sample. Furthermore, the spectra possessed well-defined vibronic structures, a widely reported feature of EDOT oligomers not shared by unsubstituted thiophene structures.[21,23,46] When compared with the parent C–H capped oligomers biEDOT **51** and terEDOT **52**, mono-piperidine-capped dimer **19** and trimer **22** displayed a large red shift in absorbance (see Figure S1). This effect was even more pronounced for the di-capped oligomers **24** and **20**. A red shift in absorbance of >100 nm indicated that conjugation of the thiophene core with the keto-acid end group, to create an acceptor-donor-acceptor triad, played a major role in influencing the properties of the synthesized oligomers, leading to a significant narrowing of the optical gap (E_{opt}).[47,48]

When compared with those of previously reported EDOT end-capped oligomers, the absorption spectra were strongly red shifted in relation to the corresponding mesityl, phenyl, hexyl, and trimethylsilyl structures highlighted in Figure 1.[21–24] The remarkably low-energy E_{opt} of the structures reported here is considered to be a consequence of the lowering in energy of the lowest unoccupied molecular orbital (LUMO) as a result of the electron-withdrawing nature of the keto-acid moiety, as discussed later. Oligomer capping with primary amines to yield secondary amides was found to result in a further lowering of E_{opt} (Figure 2C, entry 8; Figures S2 and S151–S158). This effect was enhanced through capping with more electron-poor ester groups, resulting in an E_{opt} as low as 1.88 eV for the *iso*-propyl ester di-capped pentamer **56** (Figure 2C, entry 10; Figures S84–S86 and S159–S161).

The constrained six-membered ring of EDOT is known to result in favorable attractive intramolecular S–O interactions between repeating units.[23,49] This effect is reduced upon the introduction of the more structurally flexible methoxy units of DMT. Therefore, as predicted, the introduction of a single DMT residue into an EDOT pentamer led to an increase in E_{opt} as a result of disruption of the highly planar EDOT-repeating structure. This effect was found to be position dependent such that the length of the longest continuous EDOT chain determined the degree of disruption. When compared with the pentaEDOT oligomer **27**, DMT-containing isomer **45** (four continuous residues) exhibited a ΔE_{opt} = +0.013 eV, whereas isomer **47** (two continuous residues) possessed an increased ΔE_{opt} = +0.057 eV (Figure 2C, entries 11–13). This widening of the optical gap was further enhanced in an oligomer consisting of end-capped penta-DMT **57** (ΔE_{opt} = +0.122 eV) or the analogous penta-ProDOT oligomer **58** (ΔE_{opt} = +0.44 eV) (Figure 2C, entries 14 and 15; Figures S87, S88, and S164–S173). These results support our hypothesis that the oligomer properties can be tuned through the suitable choice and positioning of alternative monomer units.

Next, we investigated the solution electrochemical properties of selected oligomers by cyclic voltammetry. Di-piperidine-capped oligomers **24**, **20**, and **26–28** (n = 2–6) were all investigated. However, because of the low solubility of EDOT-heptamer **29** and its reduced purity, weak signal intensity was observed during measurements, and therefore this structure was not further investigated. Cyclic voltammograms (CVs) demonstrated a decrease in the first oxidation potential with increasing chain length, supporting the results obtained by UV-Vis spectroscopy (Figure 3A). Linear correlations were found between the first and second oxidation potentials and the inverse chain length (Figure 3B; see Table S1). The oxidation of oligomers **24**, **20**, and **26** (n = 2–4) was electrochemically quasi-reversible, whereas pentamer **27** and hexamer **28** displayed improved electrochemical reversibility (Figure 3). Furthermore, CVs of penta-DMT **57** and penta-ProDOT **58** allowed comparison with penta-EDOT **27** (see Figure S3). As was seen for the optical gap, the first oxidation potential was found to follow the trend EDOT < ProDOT < DMT. These results further support the higher effective conjugation of EDOT oligomers and a degree of planarity disruption induced by the high torsional strain of DMT-based structures.[50] The ease with which the oxidation potentials can be tuned, through both alteration of oligomer length and monomer composition, offers intriguing possibilities for applications not only in tissue engineering but also in creating sensitive and selective organic bioelectronics.[51,52]

Finally, we undertook computational density functional theory (DFT) calculations to further probe the influence of the keto-acid end groups on oligomer properties.[53] The trends observed in the calculated HOMO-LUMO gaps during these studies

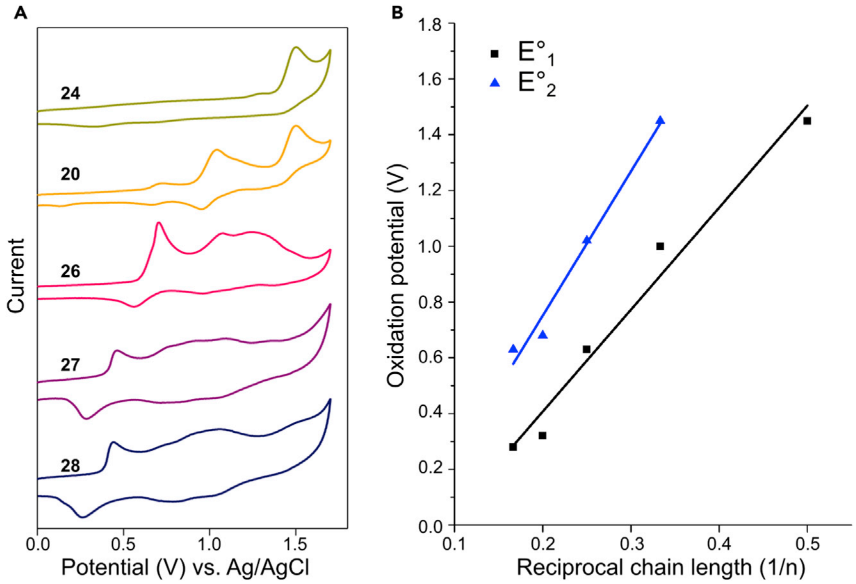

Figure 3. Cyclic Voltammetry Characterization

(A) Cyclic voltammograms of piperidine-capped oligomers **24**, **20**, and **26–28**. CVs were recorded at a scan rate of 100 mV s^{-1} with oligomer concentrations of 1 mM in DCM containing 0.1 M Bu_4NPF_6.

(B) Correlation of inverse chain length and first and second oxidation potentials for oligomers **24**, **20**, and **26–28** (adjusted R^2 = 0.9680 and 0.9725, respectively).

reproduced the structural and length dependencies observed during experimental measurements. Initial calculations on carboxy-terminated EDOT pentamer **59** validated our hypothesis that the keto-acid end group played an important role in extending π conjugation (Figure 4). This was particularly true for the LUMO—the electron-withdrawing nature of the end group led to a large orbital localization across the ketone group. Partial distribution of the LUMO across the terminal carboxyl indicated that the choice of an ester or amide linkage might influence the electrical properties of oligomeric constructs. Thus, compared with an analogous amide substrate, the presence of a more electron-deficient ester group would be expected to lower the LUMO level, leading to a decreased HOMO-LUMO gap (see Figure S4). This supports our experimental observation of a lower E_{opt} for *iso*-propyl ester di-capped oligomers than for amide-capped structures.

DFT also provided rationale for the increase in E_{opt} observed for tertiary-amide-capped structures. To accommodate the steric bulk of both the piperidine and diisopropylamine substituents, the dicarbonyl groups were found to be significantly disrupted from the antiperiplanar orientation observed for other substituents. This led to dihedral angles of as little as 131° for diisopropyl-capped dimer **60** and 142° for piperidine-capped dimer **24** (see Figures S5, S89, S90, S162, and S163). As a result, conjugation was partially disrupted, leading to an increase in the HOMO-LUMO gap, supporting the observed increase in E_{opt}. Replacement of EDOT with DMT or ProDOT offered two different mechanisms by which disruption of the expected planar configuration could potentially occur. In the case of DMT, the high torsional strain of consecutive units was found to lead to a slight twisting of the backbone for longer oligomer structures, therefore decreasing effective conjugation. In contrast, calculations predicted a slight deflection of the alkoxy substituents in the ProDOT structure (174° and 180° dihedral angle in EDOT and DMT,

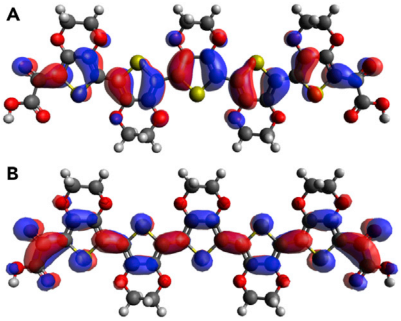

Figure 4. DFT Orbital Projections
(A) HOMO orbital distribution of carboxy-capped pentamer **59**.
(B) LUMO orbital distribution.

respectively) to accommodate an expanded seven-membered ring. The resultant cumulative decrease in electron donation from these substituents might explain the slight increase in E_{opt} observed for the ProDOT derivatives described above.

Conclusions

We have developed a glyoxylation end-capping strategy that allows the rapid installation of keto-amides and keto-esters at the end of oligomeric-EDOT chains. The resultant materials retain solubility and are bench stable, in contrast to previous reports of oligo-EDOT derivatives. These developments allow us to report the synthesis of hexa- and heptameric EDOT constructs for the first time. Furthermore, the use of iterative chain extension allows the construction of hetero-bifunctional constructs bearing orthogonally reactive handles for further modification. Characterization of the structures produced demonstrated the important role played by the keto-acid end group in determining oligomer properties. The remarkably low optical gap observed for the oligomeric structures was attributed to the important role played by the extended conjugated system, particularly in lowering the LUMO energy, as demonstrated by DFT calculations. Notably, through suitable choice of oligomer length, end group, and monomer composition, the optical, electronic, and physical properties of a construct can be readily tuned both across a wide range and with fine control. This ability to undertake a flexible and modular approach to structural design creates intriguing opportunities in the synthesis of novel materials. Work to explore the full possibilities of this powerful methodology is currently ongoing in our group for the integration of tunable conjugated materials into tissue engineering scaffolds.

EXPERIMENTAL PROCEDURES

General Method for EDOT Glyoxylation

Oxalyl chloride (850 μL, 10 mmol) was added drop-wise to a solution of EDOT (1.05 mL, 10 mmol) in dioxane (30 mL). The mixture was heated to 100°C for 1 hr and then allowed to cool to room temperature. The requisite amine (15 mmol) and base (50 mmol) were then added, and the mixture was stirred for 3 hr. After this time, the mixture was diluted with DCM (150 mL) and washed with water (100 mL), and the organics were dried with $MgSO_4$, filtered, and concentrated in vacuo. The residue was purified by flash column chromatography, and pure fractions were concentrated in vacuo.

General Method for Monomer Bromination

EDOT derivative (5 mmol) was dissolved in a mixture of tetrahydrofuran (THF, 5 mL) and acetic acid (3 mL). If solubility was poor, a further 25 mL of THF was added. The mixture was placed in the dark, and *N*-bromosuccinimide (6 mmol) was added. After being stirred for 2 hr, the mixture was either precipitated in water (150 mL), causing

precipitation of the product, which could be collected by filtration, or diluted with DCM (150 mL) and washed with saturated $NaHCO_3$ (3 × 100 mL), dried with $MgSO_4$, filtered, and concentrated in vacuo. Column chromatography was then undertaken if required, although the products were usually sufficiently pure for further use.

General Method for Chain Extension

Brominated monomer (1 mmol), pivalic acid (0.5 mmol), palladium(II) acetate (0.05 mmol), and potassium carbonate (10 mmol) were charged under nitrogen. Dry DMF (2 mL) and EDOT (4 mmol) were then added, and the mixture was heated to 90°C for 2 hr. After cooling to room temperature, the mixture was diluted with DCM (50 mL) and washed with water (2 × 50 mL) and brine (50 mL). The organics were dried with $MgSO_4$, filtered, and concentrated in vacuo. The residue was purified by flash column chromatography, and pure fractions were concentrated in vacuo.

General Method for Oligomer Synthesis

Brominated oligomer (1 mmol), hydrogen-capped oligomer (1.2 mmol), pivalic acid (0.5 mmol), palladium(II) acetate (0.05 mmol), and potassium carbonate (10 mmol) were charged under nitrogen. Dry DMF (2 mL) was added, and the mixture was heated to 90°C for 2 hr. After cooling to room temperature, the mixture was diluted with DCM (50 mL) and washed with water (2 × 50 mL) and brine (50 mL). The organics were dried with $MgSO_4$, filtered, and concentrated in vacuo. The residue was purified by flash column chromatography, and pure fractions were concentrated in vacuo.

SUPPLEMENTAL INFORMATION

Supplemental Information includes Supplemental Experimental Procedures, 173 figures, 2 tables, and 4 schemes and can be found with this article online at http://dx.doi.org/10.1016/j.chempr.2016.12.003.

AUTHOR CONTRIBUTIONS

C.D.S. performed all experiments and wrote the manuscript. M.A.B. performed CV measurements. A.A. performed UV-Vis measurements. C.D.S., M.A.B., A.A., and C.B.N. analyzed and interpreted data. C.D.S., D.M., and M.M.S. developed the ideas. All authors commented on the manuscript. M.M.S. supervised the project.

ACKNOWLEDGMENTS

Prof. John McArthur of the ICP Cross-Faculty Elemental Analysis Facility and Dr. Kersti Karu of the Mass Spectrometry Facility at University College London are thanked for running ICP-MS analyses. Prof. M. Heeney and Drs. A.G. Guex and C.S. Wood are thanked for critical evaluation of the manuscript. M.M.S. and C.D.S. acknowledge the British Heart Foundation Cardiovascular Regenerative Medicine Center (RM/13/1/30157). D.M. was supported by Marie Skłodowska-Curie Actions under the Seventh Framework Programme (FP7) through the Intra-European Marie Curie Fellowship "MultiFun CP" under grant agreement no. 328897. M.A.B. was supported by the Freemasons Foundation of New Zealand through the Royal Society of New Zealand-Rutherford Foundation. M.M.S. acknowledges support from the ERC FP7 Consolidator Grant "Naturale CG" under grant agreement no. 616417 and a Wellcome Trust Senior Investigator Award (098411/Z/12/Z) for funding. The research data supporting this publication are available online at https://doi.org/10.5281/zenodo.163505.

Received: August 11, 2016
Revised: November 9, 2016
Accepted: December 9, 2016
Published: January 12, 2017

REFERENCES AND NOTES

1. Guimard, N.K., Gomez, N., and Schmidt, C.E. (2007). Conducting polymers in biomedical engineering. Prog. Polym. Sci. *32*, 876–921.
2. Guo, B., Glavas, L., and Albertsson, A.-C. (2013). Biodegradable and electrically conducting polymers for biomedical applications. Prog. Polym. Sci. *38*, 1263–1286.
3. Balint, R., Cassidy, N.J., and Cartmell, S.H. (2014). Conductive polymers: towards a smart biomaterial for tissue engineering. Acta Biomater. *10*, 2341–2353.
4. Pashuck, E.T., and Stevens, M.M. (2012). Designing regenerative biomaterial therapies for the clinic. Sci. Transl. Med. *4*, 1–12.
5. Wong, J.Y., Langer, R., and Ingber, D.E. (1994). Electrically conducting polymers can noninvasively control the shape and growth of mammalian cells. Proc. Natl. Acad. Sci. USA *91*, 3201–3204.
6. Gumus, A., Califano, J.P., Wan, A.M.D., Huynh, J., Reinhart-King, C.A., and Malliaras, G.G. (2010). Control of cell migration using a conducting polymer device. Soft Matter *6*, 5138–5142.
7. Gilmore, K.J., Kita, M., Han, Y., Gelmi, A., Higgins, M.J., Moulton, S.E., Clark, G.M., Kapsa, R., and Wallace, G.G. (2009). Skeletal muscle cell proliferation and differentiation on polypyrrole substrates doped with extracellular matrix components. Biomaterials *30*, 5292–5304.
8. Srivastava, N., Venugopalan, V., Divya, M.S., Rasheed, V.A., James, J., and Narayan, K.S. (2013). Neuronal differentiation of embryonic stem cell derived neuronal progenitors can be regulated by stretchable conducting polymers. Tissue Eng. Part A. *19*, 1984–1993.
9. Wan, A.M.D., Schur, R.M., Ober, C.K., Fischbach, C., Gourdon, D., and Malliaras, G.G. (2012). Electrical control of protein conformation. Adv. Mater. *24*, 2501–2505.
10. Green, R.A., Hassarati, R.T., Goding, J.A., Baek, S., Lovell, N.H., Martens, P.J., and Poole-Warren, L.A. (2012). Conductive hydrogels: mechanically robust hybrids for use as biomaterials. Macromol. Biosci. *12*, 494–501.
11. Koch, F.P.V., Smith, P., and Heeney, M. (2013). "Fibonacci's route" to regioregular oligo(3-hexylthiophene)s. J. Am. Chem. Soc. *135*, 13695–13698.
12. Lin, Y., and Zhan, X. (2016). Oligomer molecules for efficient organic photovoltaics. Acc. Chem. Res. *49*, 175–183.
13. Müllen, K., and Wegner, G. (1998). Electronic Materials: The Oligomer Approach (Wiley-VCH).
14. Yamato, H., Ohwa, M., and Wernet, W. (1995). Stability of polypyrrole and poly(3,4-ethylenedioxythiophene) for biosensor application. J. Electroanal. Chem. *397*, 163–170.
15. Strakosas, X., Sessolo, M., Hama, A., Rivnay, J., Stavrinidou, E., Malliaras, G.G., and Owens, R.M. (2014). A facile biofunctionalisation route for solution processable conducting polymer devices. J. Mater. Chem. B *2*, 2537–2545.
16. Izumi, T., Kobashi, S., Takimiya, K., Aso, Y., and Otsubo, T. (2003). Synthesis and spectroscopic properties of a series of b-blocked long oligothiophenes up to the 96-mer: revaluation of effective conjugation length. J. Am. Chem. Soc. *125*, 5286–5287.
17. Kreyes, A., Amirkhani, M., Lieberwirth, I., Mauer, R., Laquai, F., Landfester, K., and Ziener, U. (2010). The longest β-unsubstituted oligothiophenes and their self-assembly in solution. Chem. Mater. *22*, 6453–6458.
18. Zhang, L., Colella, N.S., Liu, F., Trahan, S., Baral, J.K., Winter, H.H., Mannsfeld, S.C., and Briseno, A.L. (2013). Synthesis, electronic structure, molecular packing/morphology evolution, and carrier mobilities of pure oligo-/poly(alkylthiophenes). J. Am. Chem. Soc. *135*, 844–854.
19. Zhang, L., Colella, N.S., Cherniawski, B.P., Mannsfeld, S.C.B., and Briseno, A.L. (2014). Oligothiophene semiconductors: synthesis, characterization, and applications for organic devices. ACS Appl. Mater. Inter. *6*, 5327–5343.
20. Sotzing, G.A., Reynolds, J.R., and Steel, P.J. (1997). Poly(3,4-ethylenedioxythiphene) (PEDOT) prepared via electrochemical polymerization of EDOT, 2,2′-Bis(3,4-ethylenedioxythiophene) (BiEDOT), and their TMS derivatives. Adv. Mater. *9*, 795–798.
21. Apperloo, J.J., Groenendaal, L.B., Verheyen, H., Jayakannan, M., Janssen, R.A.J., Dkhissi, A., Beljonne, D., Lazzaroni, R., and Brédas, J.L. (2002). Optical and redox properties of a series of 3,4-ethylenedioxythiophene oligomers. Chemistry *8*, 2384–2396.
22. Hicks, R.G., and Nodwell, M.B. (2000). Synthesis and electronic structure investigations of alpha,omega-bis(arylthio)oligothiophenes: toward understanding wire-linker interactions in molecular-scale electronic materials. J. Am. Chem. Soc. *122*, 6746–6753.
23. Turbiez, M., Frère, P., and Roncali, J. (2003). Stable and soluble oligo(3,4-ethylenedioxythiophene)s end-capped with alkyl chains. J. Org. Chem. *68*, 5357–5360.
24. Wasserberg, D., Meskers, S.C.J., Janssen, R.A.J., Mena-Osteritz, E., and Bäuerle, P. (2006). High-resolution electronic spectra of ethylenedioxythiophene oligomers. J. Am. Chem. Soc. *128*, 17007–17017.
25. Merkul, E., Dohe, J., Gers, C., Rominger, F., and Müller, T.J.J. (2011). Three-component synthesis of ynediones by a glyoxylation/Stephens-Castro coupling sequence. Angew. Chem. Int. Ed. Engl. *50*, 2966–2969.
26. Spicer, C.D., and Davis, B.G. (2014). Selective chemical protein modification. Nat. Commun. *V5*, P4740.
27. Stefan, M.C., Bhatt, M.P., Sista, P., and Magurudeniya, H.D. (2012). Grignard metathesis (GRIM) polymerization for the synthesis of conjugated block copolymers containing regioregular poly(3-hexylthiophene). Polym. Chem. *3*, 1693–1701.
28. Xu, S., Kim, E.H., Wei, A., and Negishi, E. (2014). Pd- and Ni-catalyzed cross-coupling reactions in the synthesis of organic electronic materials. Sci. Technol. Adv. Mater. *15*, 44201.
29. Carsten, B., He, F., Son, H.J., Xu, T., and Yu, L. (2011). Stille polycondensation for synthesis of functional materials. Chem. Rev. *111*, 1493–1528.
30. Zhao, H., Liu, C.-Y., Luo, S.-C., Zhu, B., Wang, T.-H., Hsu, H.-F., and Yu, H.-H. (2012). Facile syntheses of dioxythiophene-based conjugated polymers by direct C–H arylation. Macromolecules *45*, 7783–7790.
31. Vechorkin, O., Proust, V., and Hu, X. (2009). Functional group tolerant Kumada-Corriu-Tamao coupling of nonactivated alkyl halides with aryl and heteroaryl nucleophiles: catalysis by a nickel pincer complex permits the coupling of functionalized Grignard reagents. J. Am. Chem. Soc. *131*, 9756–9766.
32. Schipper, D.J., and Fagnou, K. (2011). Direct arylation as a synthetic tool for the synthesis of thiophene-based organic electronic materials. Chem. Mater. *23*, 1594–1600.
33. Segawa, Y., Maekawa, T., and Itami, K. (2015). Synthesis of extended π-systems through C-H activation. Angew. Chem. Int. Ed. Engl. *54*, 66–81.
34. Lafrance, M., and Fagnou, K. (2006). Palladium-catalyzed benzene arylation: incorporation of catalytic pivalic acid as a proton shuttle and a key element in catalyst design. J. Am. Chem. Soc. *128*, 16496–16497.
35. United States Phamacopeia Convention. (2015). <232> Elemental Impurities - Limits (United States Pharmacopeia).
36. Torborg, C., and Beller, M. (2009). Recent applications of palladium-catalyzed coupling reactions in the pharmaceutical, agrochemical, and fine chemical industries. Adv. Synth. Catal. *351*, 3027–3043.
37. Recho, J., Black, R.J.G., North, C., Ward, J.E., and Wilkes, R.D. (2014). Statistical DoE approach to the removal of palladium from active pharmaceutical ingredients (APIs) by functionalized silica adsorbents. Org. Process. Res. Dev. *18*, 626–635.

38. Cano, R., Schmidt, A.F., and McGlacken, G.P. (2015). Direct arylation and heterogeneous catalysis; ever the twain shall meet. Chem. Sci. *6*, 5338–5346.

39. Bura, T., Blaskovits, J.T., and Leclerc, M. (2016). Direct (hetero)arylation polymerization: trends and perspectives. J. Am. Chem. Soc. *138*, 10056–10071.

40. Lombeck, F., Komber, H., Gorelsky, S.I., and Sommer, M. (2014). Identifying homocouplings as critical side reactions in direct arylation polycondensation. ACS Macro Lett. *3*, 819–823.

41. Hong, W., Chen, S., Sun, B., Arnould, M.A., Meng, Y., and Li, Y. (2015). Is a polymer semiconductor having a "perfect" regular structure desirable for organic thin film transistors? Chem. Sci. *6*, 3225–3235.

42. Tan, Y., and Hartwig, J.F. (2011). Assessment of the intermediacy of arylpalladium carboxylate complexes in the direct arylation of benzene: evidence for C-H bond cleavage by "ligandless" species. J. Am. Chem. Soc. *133*, 3308–3311.

43. Johansson Seechurn, C.C.C., Kitching, M.O., Colacot, T.J., and Snieckus, V. (2012). Palladium-catalyzed cross-coupling: a historical contextual perspective to the 2010 Nobel Prize. Angew. Chem. Int. Ed. Engl. *51*, 5062–5085.

44. Jatsch, A., Schillinger, E.-K., Schmid, S., and Bäuerle, P. (2010). Biomolecule assisted self-assembly of π-conjugated oligomers. J. Mater. Chem. *20*, 3563–3578.

45. Bell, O.A., Wu, G., Haataja, J.S., Brömmel, F., Fey, N., Seddon, A.M., Harniman, R.L., Richardson, R.M., Ikkala, O., Zhang, X., and Faul, C.F. (2015). Self-assembly of a functional oligo(aniline)-based amphiphile into helical conductive nanowires. J. Am. Chem. Soc. *137*, 14288–14294.

46. Medina, B.M., Wasserberg, D., Meskers, S.C.J., Mena-Osteritz, E., Bäuerle, P., and Gierschner, J. (2008). EDOT-type materials: planar but not rigid. J. Phys. Chem. A. *112*, 13282–13286.

47. Bredas, J.-L. (2014). Mind the gap! Mater. Horiz *1*, 17–19.

48. Kularatne, R.S., Magurudeniya, H.D., Sista, P., Biewer, M.C., and Stefan, M.C. (2013). Donor-acceptor semiconducting polymers for organic solar cells. J. Polym. Sci. Part A. Polym. Chem. *51*, 743–768.

49. Nielsen, C.B., Angerhofer, A., Abboud, K.A., and Reynolds, J.R. (2008). Discrete photopatternable pi-conjugated oligomers for electrochromic devices. J. Am. Chem. Soc. *130*, 9734–9746.

50. Lomas, J.S., Adenier, A., Gao, K., Maurel, F., and Vaissermann, J. (2002). Hydrogen bonding and steric effects on rotamerization in 3,4-alkylenedioxy-, 3-alkoxy- and 3,4-dialkoxy-2-thienyldi(tert-butyl)methanols: an NMR, IR and X-ray crystallographic study. J. Chem. Soc. Perkin Trans. *2*, 216–224.

51. Rivnay, J., Owens, R.M., and Malliaras, G.G. (2014). The rise of organic bioelectronics. Chem. Mater. *26*, 679–685.

52. Simon, D.T., Gabrielsson, E.O., Tybrandt, K., and Berggren, M. (2016). Organic bioelectronics: bridging the signaling gap between biology and technology. Chem. Rev. *116*, 13009–13041.

53. Sun, H., and Autschbach, J. (2014). Electronic energy gaps for π-conjugated oligomers and polymers calculated with density functional theory. J. Chem. Theor. Comput. *10*, 1035–1047.

Chem, Volume *2*

Supplemental Information

Synthesis of Hetero-bifunctional, End-Capped

Oligo-EDOT Derivatives

Christopher D. Spicer, Marsilea A. Booth, Damia Mawad, Astrid Armgarth, Christian B. Nielsen, and Molly M. Stevens

Table of contents

**S2**	Supplemental figures
**S5**	Supplemental schemes
**S7**	Supplemental table
**S7**	General considerations
**S9**	Cyclic voltammetry
**S9**	DFT calculations
**S10**	Amine synthesis
**S13**	Thiophene synthesis
**S16**	EDOT Functionalisation
**S21**	Monomer bromination
**S25**	Monomer manipulation
**S31**	Chain extension protocol
**S32**	Chain extension-dimers
**S36**	Dimer bromination
**S38**	Chain extension-trimers
**S40**	Trimer bromination
**S40**	Oligomer synthesis protocol
**S41**	Dimer synthesis
**S42**	Trimer synthesis
**S44**	Tetramer synthesis
**S46**	Pentamer synthesis
**S49**	Hexamer synthesis
**S49**	Heptamer synthesis
**S49**	Oligomer manipulation
**S53**	3,4-Dimethoxythiophene functionalisation
**S60**	3,4-Propylenedioxythiophene functionalisation
**S63**	References
**S64**	NMR spectra of novel compounds

S2

Figure S1. Normalised UV-Vis (solid) and fluorescence (dashed) spectra of piperidine-capped oligomers.

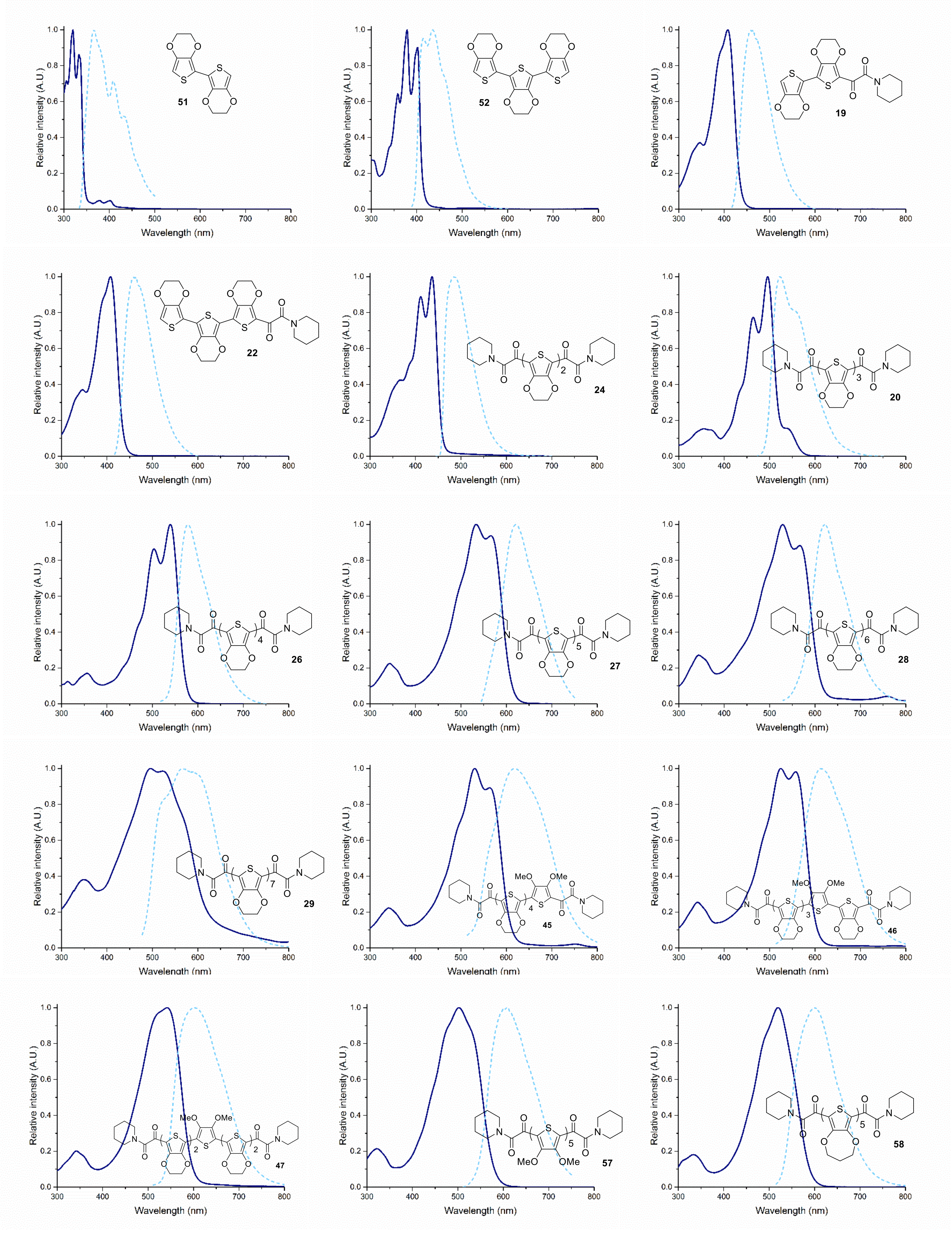

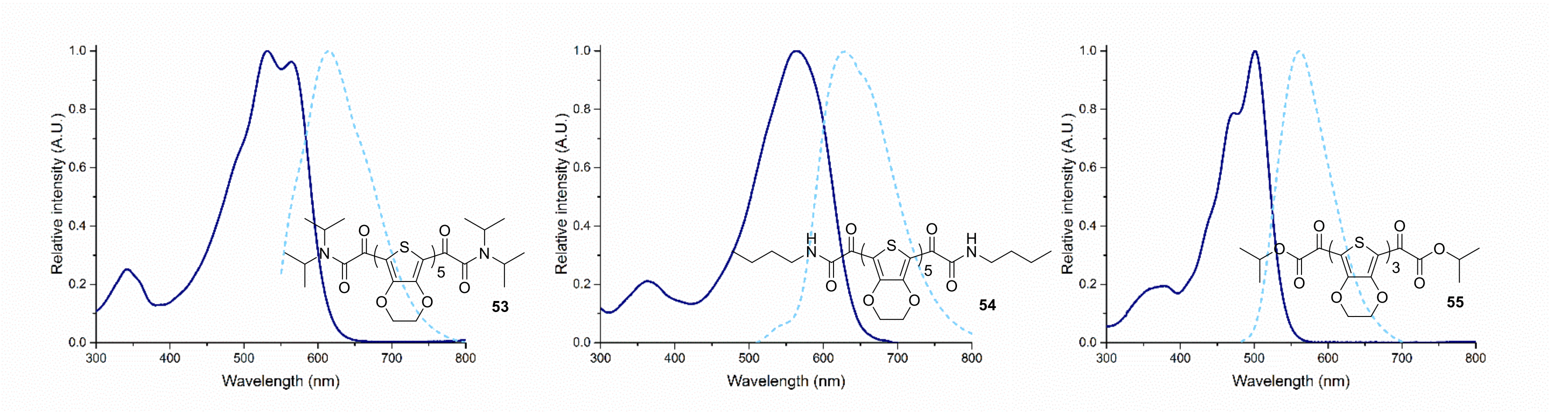

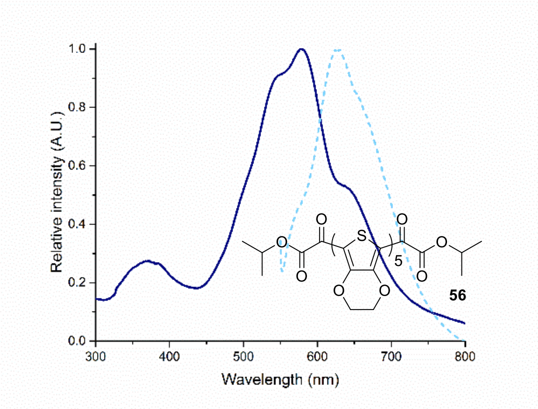

Figure S2. Normalised UV-Vis (solid) and fluorescence (dashed) spectra of diisopropylamine, *n*-butylamine ad *iso*-propanol capped oligomers.

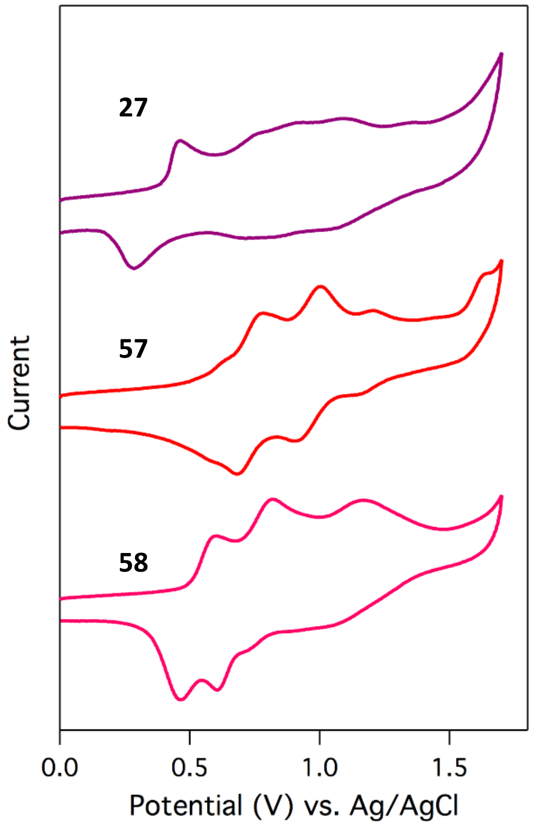

Figure S3. Cyclic voltammograms of EDOT (**27**), DMT (**57**) and ProDOT (**58**) pentamers. Voltamograms were recorded at a concentration of 1 mM in DCM containing 1 M Bu_4NPF_6.

S4

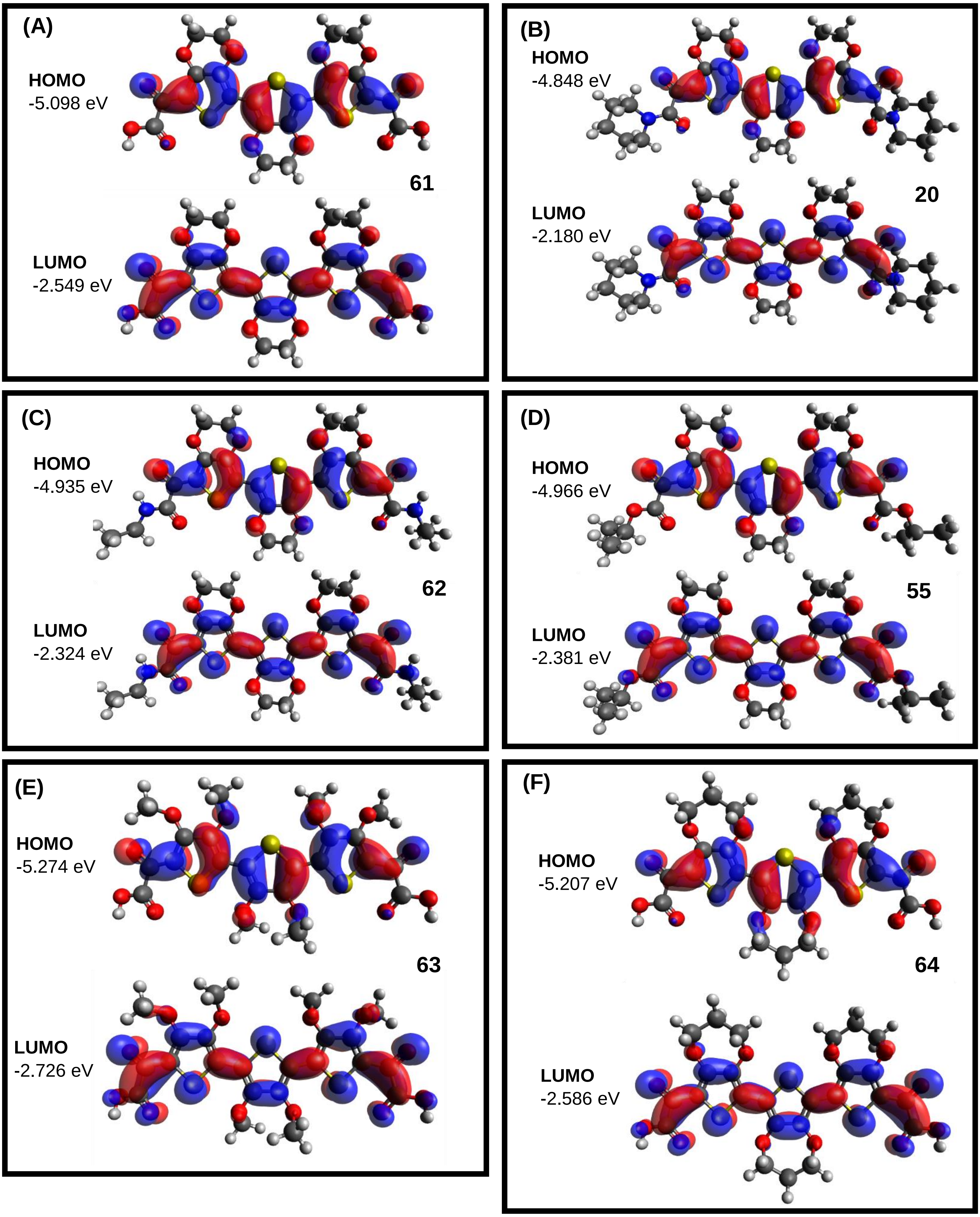

Figure S4. DFT HOMO/LUMO distributions and optimised geometries for model oligomers. Trimers were modelled in order to reduce computational time. (A) Carboxy-terminated EDOT trimer **61**. (B) Piperidine capped EDOT trimer **20**. (C) Ethylamine-capped EDOT trimer **62**. An ethyl group was modelled in place of the butyl group used experimentally to reduce computational time. (D) *Iso*-propyl ester-capped EDOT trimer **55**. (E) Carboxy-terminated DMT trimer **63**. (F) Carboxy-terminated ProDOT trimer **64.**

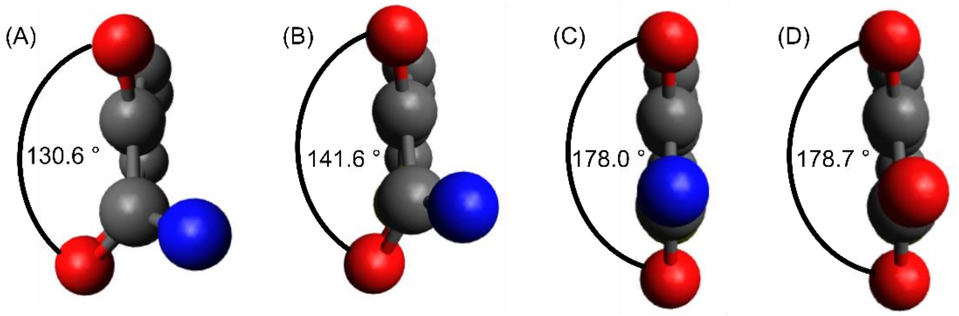

Figure S5. DFT optimised geometries demonstrating the dicarbonyl dihedral angle. Amide/ester substituents, dialkoxy-rings and further EDOT residues have been removed for visual clarity. (A) Diisopropylamine capped dimer **60**. (B) Piperidine capped dimer **24**. (C) Ethylamine capped trimer **62**. (D) *Iso*-propyl ester capped trimer **38**.

Supplemental schemes

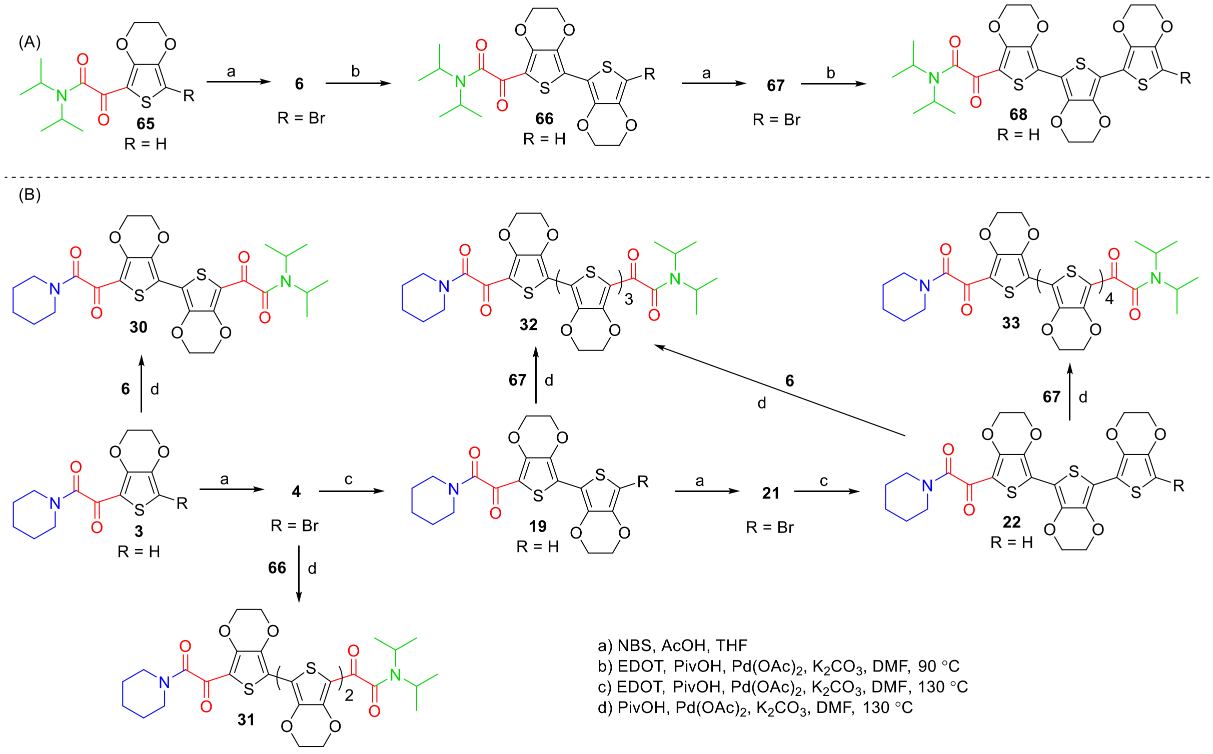

Scheme S1. (A) Synthesis of mono-capped diisopropylamine-EDOT oligomers; (B) Synthesis of unsymmetrical, bifunctional piperidine-diisopropylamine EDOT oligomers.

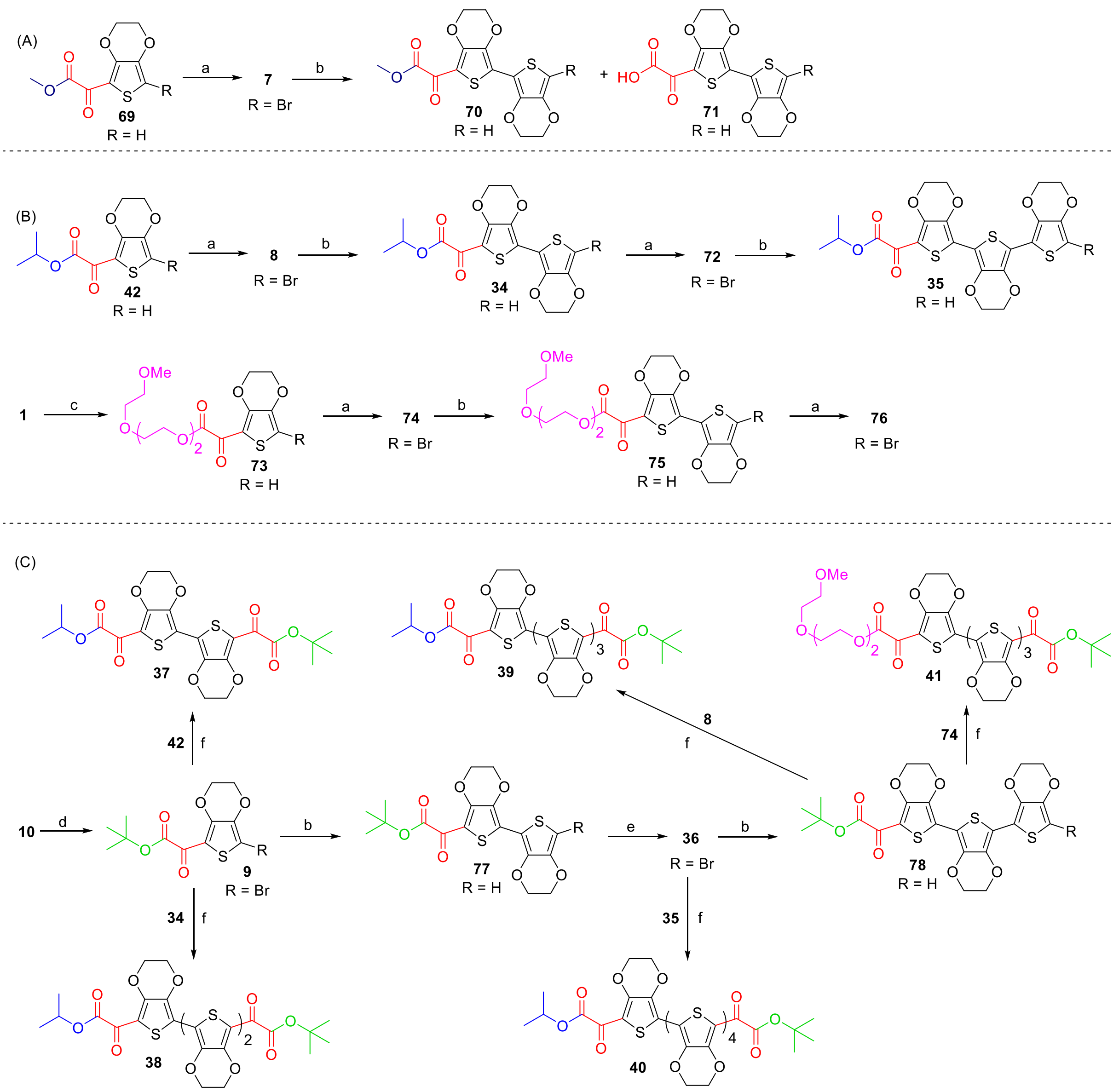

a) NBS, AcOH, THF; b) EDOT, PivOH, $Pd(OAc)_2$, K_2CO_3, DMF, 90 °C; c) i) $(COCl)_2$, dioxane, ii) HO-TEG-OMe, NEt_3; d) tBuOH, DCC, DMAP, DCM;
e) NBS, DMF; f) PivOH, $Pd(OAc)_2$, K_2CO_3, DMF, 90 °C;

Scheme S2. a) Partial methyl ester cleavage under chain extension conditions; b) Synthesis of mono-capped ester-oligomers; c) Synthesis of hetero-bifunctional di-ester oligomers.

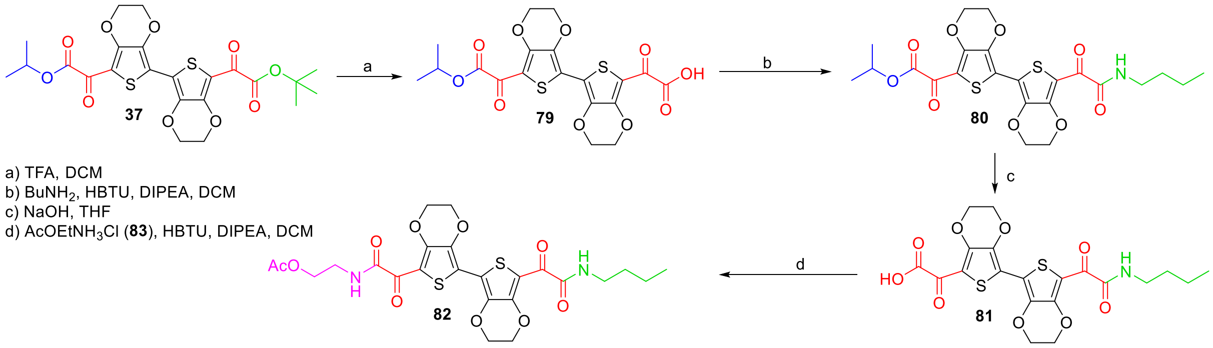

Scheme S3. Orthogonal ester deprotection and sequential amide coupling to generate hetero-bifunctional oligomer bearing reactive groups for further modification.

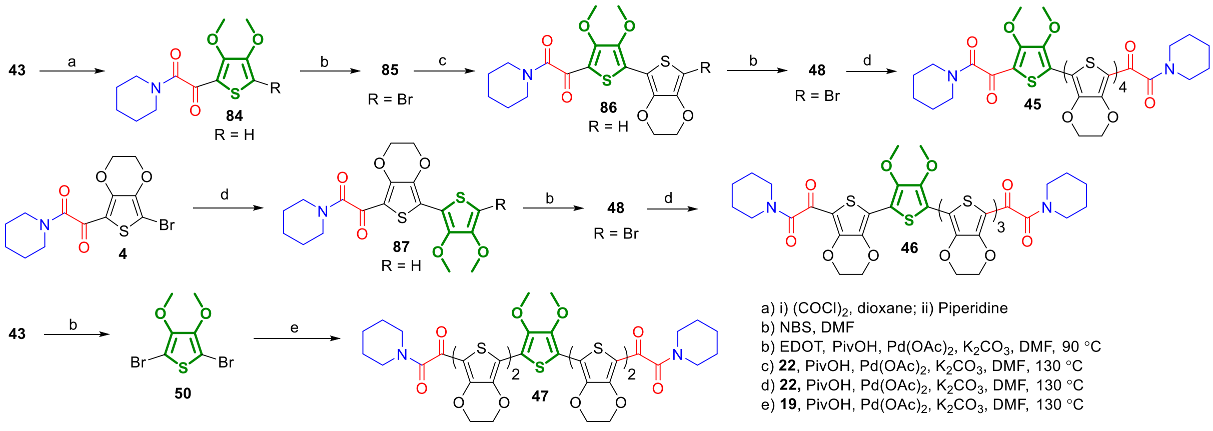

Scheme S4. Synthesis of dimethoxythiophene substituted oligomers.

Entry	Compound	Length	Monomer	$E°_1$ (V)	$E°_2$ (V)
1	**24**	2	EDOT	1.45	
2	**20**	3	EDOT	1.00	1.45
3	**26**	4	EDOT	0.63	1.02
4	**27**	5	EDOT	0.32	0.68
5	**28**	6	EDOT	0.28	0.63
6	**57**	5	DMT	0.69	0.95
7	**59**	5	ProDOT	0.48	0.76

Table S1. Summary of oligomer first and second oxidation potentials, calculated by cyclic voltammetry. Potentials are corrected by the internal standard Fc/Fc$^+$ and are vs. Ag/AgCl.

General Considerations

Proton nuclear magnetic resonance (^{1}H NMR) spectra were recorded on a Bruker AV-400 (400 MHz) spectrometer. Carbon nuclear magnetic resonance (^{13}C NMR) spectra were recorded on a Bruker AV-400 (100 MHz) spectrometer. NMR shifts were assigned using COSY, HSQC and HMBC spectra. All

chemical shifts are quoted on the δ scale in ppm using residual solvent as the internal standard (^{1}H NMR: $CDCl_3$ = 7.26; MeOD = 3.31 DMSO-d_6 = 2.50 and ^{13}C NMR: $CDCl_3$ = 77.16, MeOD = 49.00, DMSO-d_6 = 39.52). Coupling constants (*J*) are reported in Hz with the following splitting abbreviations: s = singlet, d = doublet, t = triplet, q = quartet, sept = septet, m = multiplet, app = apparent, br = broad. Melting points (m.p.) were recorded on a Zeiss Axio Imager: Z1M microscope equipped with a Linkam LTS 420 temperature controlled microscope stage and are uncorrected. Infrared (IR) spectra were recorded on a Perkin Elmer Spectrum 100 spectrophotometer with a Universal ATR Sampling Accessory. Absorption maxima ($υ_{max}$) are reported in wavenumbers (cm^{-1}). UV-Vis spectra were recorded on a Perkin Elmer Lambda 25 spectrophotometer in a quartz cuvette with a pathlength of 1 cm. All measurements were undertaken in DCM at a concentration sufficient to give an absorbance reading of 0.5-1 Au. Fluorescence spectra were recorded on a Horiba Fluorolog fluorimeter in a quartz fluorescence cuvette. All measurements were undertaken in DCM at a concentration sufficient to give a relative emission intensity of 10^{6}-2.5x10^{7} C.P.S. Low resolution mass spectra (LRMS) were recorded on an Agilent 6130 Quadrupole mass spectrometer using electrospray ionization (ESI), connected to an Agilent 1260 Infinity liquid chromatography set-up with a Phenomenex Gemini-NX-C16 column. High resolution mass spectra (HRMS) were recorded on a Waters LCT Premier (ES-ToF) spectrometer connected to an Aquity-iClass UPLC. Matrix-assisted laser desorption-ionization (MALDI) spectra were recorded on a Micromass MALDI-ToF spectrometer. Nominal and exact m/z values are reported in Daltons. ICP-MS was performed on a Varian 820-MS ICP-mass spectrometer. Samples were digested in 69 % AnalaR nitric acid (Sigma-Aldrich) and then diluted to 0.1 % analyte in 2 % nitric acid. Analysis was undertaken using the isotopes Pd-105, Pd-106, and Pd-108, using a 10 ppb palladium standard as a calibrant. Thin layer chromatography (TLC) was carried out using aluminium backed sheets coated with 60 F_{254} silica gel (Merck). Visualization of the silica plates was achieved using a UV lamp ($λ_{max}$ = 254, 302, or 366 nm), and/or ammonium molybdate (5 % in 2M H_2SO_4), and/or potassium permanganate (5 % $KMnO_4$ in 1M NaOH with 5 % potassium carbonate). Flash column chromatography was carried out using Geduran Si 60 (40-63 µm) (Merck). Mobile phases are reported as % volume of more polar solvent in less polar solvent. Anhydrous solvents were purchased from Sigma-Aldrich and used as supplied. All other solvents were used as supplied (Analytical or HPLC grade), without prior purification. Reagents were purchased from Sigma-Aldrich and used as supplied, unless otherwise indicated. 3,4-Ethylenedioxythiophene and palladium (II) acetate were purchased from Alfa Aesar. 3,4-

Dibromothiophene was purchased from Apollo scientific. Brine refers to a saturated solution of sodium chloride. Anhydrous magnesium sulfate ($MgSO_4$) was used as the drying agent after reaction workup unless otherwise stated.

Cyclic voltammetry

Cyclic voltammetry studies were performed using in-house potentiostats and a PowerLab 8/35, controlled by EChem (eDAQ). A three-electrode system was employed with a glassy carbon working electrode, and Ag/AgCl reference electrode (3M, NaCl, +0.197 V vs. SHE) and a stainless steel counter electrode. Ferrocene was added as an internal standard. Measurements were undertaken on 1 mM solutions of oligomers in DCM, containing 0.1 M Bu_4NPF_6 as an electrolyte. The spectra given in Figure 3 and supplemental Figure 2 are background subtracted. Oxidation potentials were calculated using the equation $E°_x = (E_{p,a} - E_{p,c})/2$ where $E_{p,a}$ is the anodic potential and $E_{p,c}$ is the cathodic potential.

DFT calculations

Molecules were built using the Avogadro software and geometries were manipulated to provide the lowest free energy configuration using a UFF forcefield. Single point energy and geometrical optimisation calculations were then undertaken utilising the Gaussian 9.0 software (B3LYP/6-31G*) in order to estimate the HOMO and LUMO orbital distributions. Alkyl end-groups were minimised in order to reduce computational complexity in a number of calculations.

Amine Synthesis

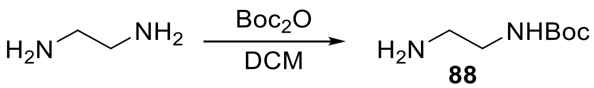

88

A solution of di-*tert*-butyl dicarbonate (3.27 g, 15 mmol) in DCM (200 mL) was added dropwise to a solution of ethylenediamine (6.01 mL, 90 mmol) in DCM (50 mL) over a period of 5 hrs. After stirring for a further 12 hrs the mixture was washed with K_2CO_3 (2 M, 2 x 200 mL), dried with $MgSO_4$, filtered and concentrated *in vacuo* to give the DP as a colourless oil. A yield of 1.74 g, 10.8 mmol (72 %) was obtained. Spectroscopic data were consistent with those previously reported.[1] ^{1}H NMR (400 MHz, $CDCl_3$): δ = 5.04 (1H, br s, -N<u>H</u>), 3.01-3.19 (2H, m, -C<u>H</u>$_2$NHBoc), 2.78 (2H, t, *J* = 5.9 Hz, -C<u>H</u>$_2$NH$_2$), 1.44 (9H, s, <u>Boc</u>) ppm;

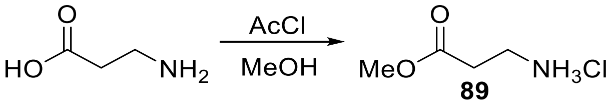

89

Acetyl chloride (14 mL, 200 mmol) was added dropwise to methanol (250 mL) at 0 ºC over a period of 15 min. After stirring for a further 15 min, β-alanine (1.78 g, 20 mmol) was added and the mixture stirred for 18 hrs. The solvent was then removed *in vacuo* and the residue triturated for 15 min in diethyl ether (2 x 100 mL) and DCM (100 mL), filtered and dried *in vacuo*. The DP was obtained as a white solid. A yield of 2.58 g, 18.6 mmol (93 %) was obtained. Spectroscopic data were consistent with those previously reported.[2] ^{1}H NMR (400 MHz, MeOD): δ = 3.76 (3H, s, -O<u>Me</u>), 3.23 (2H, t, *J* = 6.5 Hz, $\underline{H}_{\beta}$), 2.78 (2H, t, *J* = 6.5 Hz, $\underline{H}_{\alpha}$) ppm;

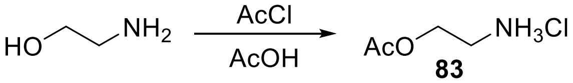

83

Acetyl chloride (5.7 mL, 80 mmol) was added to a solution of ethanolamine (2.4 mL, 40 mmol) in acetic acid (20 mL). After stirring for 18 hrs the mixture was poured into diethyl ether (200 mL). The resultant precipitate was collected by filtration, washed with diethyl ether (100 mL) and dried *in vacuo* to give the DP as a white sold. A yield of 5.2 g, 37.4 mmol (93 %) was obtained. Spectroscopic data were consistent with those previously reported.[3] ^{1}H NMR (400 MHz, DMSO-d$_6$): δ = 8.24 (3H, br s, -N$\underline{H}_3$Cl), 4.20 (2H, t, *J* = 5.2 Hz, -C$\underline{H}_2$NH$_3$Cl), 3.05 (2H, t, *J* = 5.2 Hz, -C$\underline{H}_2$OAc), 2.06 (3H, s, -O<u>Ac</u>) ppm;

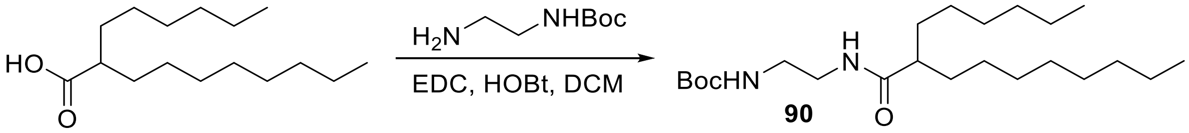

90

EDC (4.8 g, 25 mmol) and hydroxybenzotriazole (3.8 g, 25 mmol) were added to a solution of 2-Hexyldecanoic acid (5.1 mL, 16.7 mmol) and amine **88** (3.2 g, 20 mmol) in DCM (100 mL). The mixture was stirred for 18 hrs then washed with water (100 mL) and brine (100 mL). The organics were dried with $MgSO_4$, filtered and concentrated onto silica. The residue was purified by flash column chromatography eluting with 20-50 % EtOAc:Hexane. Pure fractions were concentrated *in vacuo* to give the DP as a white solid. A yield of 3 g, 7.5 mmol (45 %) was obtained. ^{1}H NMR (400 MHz, $CDCl_3$): δ = 6.28 (1H, br s, -N<u>H</u>COAlk), 4.99 (1H, br s, -N<u>H</u>Boc), 3.38 (2H, dt, *J* = 6.4, 5.1 Hz, -C$\underline{H}_2$NHCOAlk), 3.29 (2H, t, *J* = 5.1 Hz, -C$\underline{H}_2$NHBoc), 1.97-2.09 (1H, m, $\underline{H}_{\alpha}$), 1.51-1.58 (2H, m, $\underline{H}_{\beta}$), 1.35-1.51 (9H, m, <u>Alkyl</u>

and Boc), 1.15-1.35 (20H, m, Alkyl), 0.79-0.85 (6H, m, -CH_3) ppm; ^{13}C NMR (100 MHz, $CDCl_3$): δ = 177.47 (-NHCOAlkyl), 157.00 (-NHCO_2*t*Bu), 79.82 (-CMe$_3$),47.78 ($C_α$), 40.86 (-CH_2NHCOAlk), 40.39 (-CH_2NHBoc), 32.91 (Alkyl), 29.69 (Alkyl), 29.34 (Alkyl), 29.29 (Alkyl), 28.37 (Alkyl), 22.66 (Alkyl), 22.62 (Alkyl), 14.10 (-CH_3), 14.07 (-CH_3) ppm; IR ($υ_{max}$, solid): 3346, 3305, 2952, 2849, 1686, 1645, 1530, 1390, 1367, 1317, 1283, 1251, 1236, 1169 cm^{-1}; HRMS *m/z* (ESI+): Found: 399.3568 (M+H), Calc.: 399.3587; m.p. = 101-102 °C;

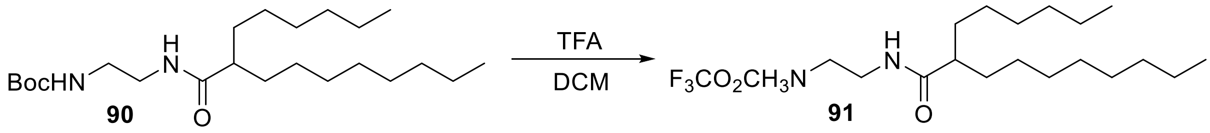

Trifluoroacetic acid (10 mL) was added to a suspension of amine **90** (3 g, 7.5 mmol) in DCM (50 mL) causing dissolution. After 3 hrs the mixture was concentrated *in vacuo* and azeotroped with toluene (2 x 50 mL) to give the DP as a white solid. NMR showed that some TFA remained in the product, but it was used in the following step without further purification. A yield of 3.09 g, 7.5 mmol (99 %) was obtained. ^{1}H NMR (400 MHz, $CDCl_3$): δ = 7.78 (3H, br s, -NH_3), 7.24-7.29 (1H, m, -NHCOAlk), 3.56-3.65 (2H, m, -CH_2NHCOAlk), 3.22-3.33 (2H, m, , -CH_2NH$_3$), 2.13-2.26 (1H, m, $H_α$), 1.38-1.55 (4H, m, $H_β$), 1.11-1.37 (20H, m, Alkyl), 0.82-0.92 (6H, m, -CH_3) ppm; ^{13}C NMR (100 MHz, $CDCl_3$): δ = 181.57 (-NHCOAlkyl), 47.32 ($C_α$), 41.07 (-CH_2NH$_3$), 38.21 (-CH_2NHCOAlk), 32.38 (Alkyl), 31.74 (Alkyl), 31.46 (Alkyl), 29.42 (Alkyl), 29.25 (Alkyl), 29.17 (Alkyl), 29.06 (Alkyl), 27.34 (Alkyl), 27.29 (Alkyl), 22.56 (Alkyl), 22.47 (Alkyl), 13.94 (-CH_3), 13.81 (-CH_3) ppm; IR ($υ_{max}$, solid): 3285, 2926, 2853, 1691, 1647, 1607, 1544, 1467, 1433, 1359, 1202, 1170, 1130 cm^{-1}; HRMS *m/z* (ESI+): Found: 299.3052 (M+H), Calc.: 299.3062; m.p. = 53-55 °C;

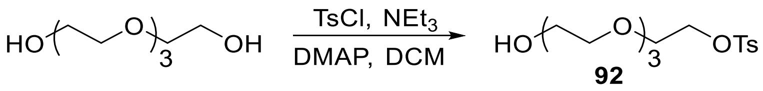

A solution of *p*-toluenesulfonyl chloride (3.8 g, 20 mmol) in DCM (200 mL) was added drop-wise over a period of 6 hrs to a mixture of tetraethylene glycol (17 mL, 100 mmol), triethylamine (3.1 mL, 22 mmol) and DMAP (244 mg, 2 mmol) in DCM (100 mL). After addition was complete the mixture was stirred for a further 10 hrs. The reaction was then washed with water (2 x 150 mL) hydrochloric acid (1 M, 2 x 150 mL), and brine (150 mL), dried with $MgSO_4$, filtered and concentrated *in vacuo* to give the DP as a

colourless oil. A yield of 6.4 g, 18.4 mmol (92 %) was obtained. The product was used in the subsequent step without further purification or analysis.

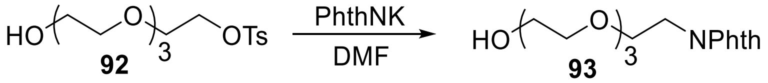

92 **93**

TEG tosylate **92** (6.4 g, 18.4 mmol) was dissolved in dry DMF (30 mL) under nitrogen and potassium phthalimide (4.1 g, 22 mmol) was added. After heating to 100 °C for 18 hrs, the mixture was cooled to rt and concentrated *in vacuo*. The residue was re-suspended in ethyl acetate (200 mL) and the organics washed with water (150 mL), hydrochloric acid (1 M, 2 x 150 mL) and brine (150 mL), dried with $MgSO_4$, filtered and concentrated *in vacuo*. The residue was purified by flash column chromatography eluting with 70-100 % EtOAc:Petrol. Pure fractions were concentrated *in vacuo* to give the DP as a colourless oil. A yield of 3.4 g, 10.5 mmol (57 %) was obtained. Spectroscopic data were consistent with those previously reported.[4] ^{1}H NMR (400 MHz, $CDCl_3$): δ = 7.79-7.86 (2H, m, Phth), 7.67-7.73 (2H, m, Phth), 3.84-3.93 (2H, m, TEG), 3.51-3.76 (14H, m, TEG) ppm;

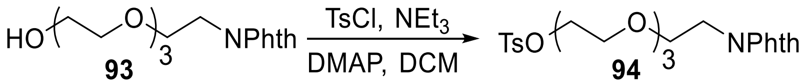

93 **94**

Protected TEG-amine **93** (3.4 g, 10.5 mmol) and DMAP (128 mg, 1.05 mmol) were dissolved in dry DCM (25 mL) under nitrogen and cooled to 0 ˚C. Triethylamine (2.9 mL, 21 mmol) and *p*-toluenesulfonyl chloride (2.4 g, 12.6 mmol) were then added and the reaction stirred for 4 hrs at rt. The mixture was then washed with water (100 mL), hydrochloric acid (1 M, 2 x 100 mL) and brine (100 mL), dried with $MgSO_4$, filtered and concentrated *in vacuo* to give the DP as a yellow oil. A yield of 4.76 g, 9.98 mmol (95 %) was obtained. The product was used in the subsequent step without further purification or analysis.

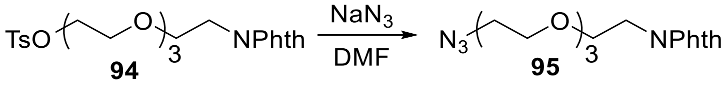

94 **95**

Sodium azide (89 mg, 1.37 mmol) was added to a solution of TEG-tosylate **94** (530 mg, 1.14 mmol) in dry DMF (5 mL) under nitrogen and heated to 70 °C for 48 hrs. After cooling to rt, the mixture was diluted with ethyl acetate (150 mL) and the organics washed with water (100 mL), hydrochloric acid (1 M, 100 mL) and brine (100 mL), dired with $MgSO_4$, filtered and concentrated *in vacuo*. The residue was

purified by flash column chromatography eluting with 50-60 % EtOAc:Hexane. Pure fractions were concentrated *in vacuo* to give the DP as a yellow oil. A yield of 1.8 g, 5.2 mmol (49 %) was obtained. Spectroscopic data were consistent with those previously reported.[5] ^{1}H NMR (400 MHz, $CDCl_3$): δ = 7.82-7.89 (2H, m, Phth), 7.69-7.75 (2H, m, Phth), 3.91 (2H, t, *J* = 5.7 Hz, TEG), 3.75 (2H, t, *J* = 5.6 Hz, TEG), 3.57-3.69 (10H, m, TEG), 3.37 (2H, t, *J* = 5.3 Hz, TEG) ppm;

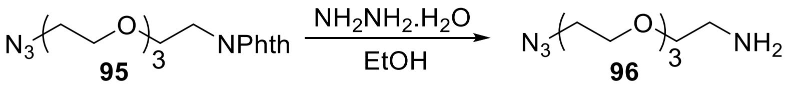

Hydrazine monohydrate (0.95 mL, 19.5 mmol) was added to a solution of TEG-azide **95** (1.7 g, 4.9 mmol) in ethanol (40 mL) under nitrogen and the mixture refluxed for 18 hrs. After cooling to rt, the resultant white precipitate was removed by filtration and the filtrate concentrated *in vacuo*. The residue was stirred in ether (20 mL) for 1 hr and the organics then filtered and concentrated *in vacuo* to give the DP as a light yellow oil. A yield of 1.06 g, 4.85 mmol (99 %) was obtained. Spectroscopic data were consistent with those previously reported.[5] ^{1}H NMR (400 MHz, $CDCl_3$): δ = 3.62-3.72 (10H, m, TEG), 3.52 (2H, t, *J* = 5.3 Hz, TEG), 3.40 (2H, t, *J* = 5.1 Hz, TEG), 2.87 (2H, t, *J* = 5.3 Hz, TEG) ppm;

Thiophene synthesis

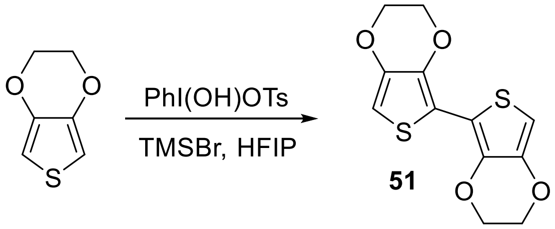

Koser's reagent (196 mg, 0.5 mmol) was added to a solution of EDOT (107 µL, 1 mmol) in HFIP (5 mL). Bromotrimethylsilane (132 mg, 1 mmol) was then added and the mixture stirred for 5 hrs. The reaction was then diluted with DCM (50 mL) and the organics washed with sat. $NaHCO_3$ (50 mL), dried with $MgSO_4$, filtered and concentrated *in vacuo*. The residue was purified by flash column chromatography eluting with 20-30 % EtOAc:Hexane. Pure fractions were concentrated *in vacuo* to give the DP as a grey solid. A yield of 65 mg, 0.23 mmol (46 %) was obtained. Spectroscopic data were consistent with those previously reported.[6] ^{1}H NMR (400 MHz, $CDCl_3$): δ = 6.29 (2H, s, ArH), 4.32-4.39 (4H, m, -OCH_2-), 4.23-4.30 (4H, m, -OCH_2-) ppm;

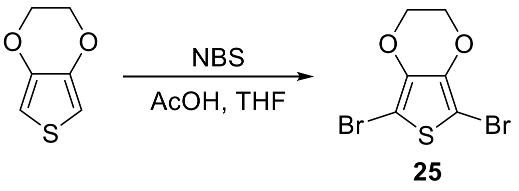

N-Bromosuccinimide (2.6 g, 14.7 mmol) was added to a solution of EDOT (1 g, 7 mmol) in acetic acid (15 mL) and THF (15 mL) in the dark and stirred for 4 hrs. The mixture was poured into water (150 mL) and the resultant precipitate collected by filtration, washed with water (2 x 50 mL) and the dissolved in DCM. The organics were dried with $MgSO_4$, filtered and concentrated *in vacuo* to give the DP as a light yellow solid. A yield of 1.9 g, 6.3 mmol (91 %) was obtained. Spectroscopic data were consistent with those previously reported.[7] ^{1}H NMR (400 MHz, $CDCl_3$): δ = 4.29 (4H, s, -OC<u>H</u>$_2$-) ppm;

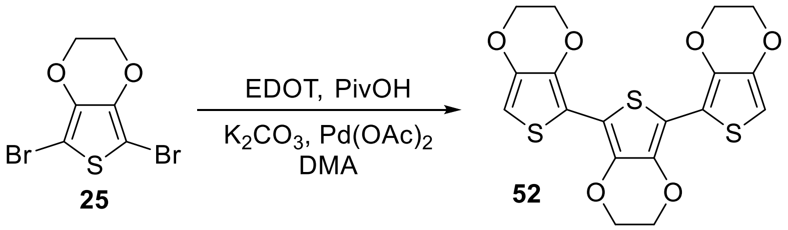

2,5-Dibromo-EDOT **25** (100 mg, 336 μmol), EDOT (143 μL, 1344 μmol), pivalic acid (10 mg, 101 μmol), palladium (**II**) acetate (4 mg, 17 μmol) and potassium carbonate (92 mg, 672 μmol) were heated to 110 ˚C in dry dimethylacetamide (500 μL) under nitrogen for 18 hrs. After cooling to rt, the mixture was diluted with DCM (50 mL), washed with water (50 mL), dried with $MgSO_4$, filtered and concentrated *in vacuo*. The residue was purified by flash column chromatography eluting with 5-20 % EtOAc:Hexane. Pure fractions were concentrated *in vacuo* to give the DP as a yellow solid. A yield of 17 mg, 40 μmol (12 %) was obtained. Spectroscopic data were consistent with those previously reported.[8] ^{1}H NMR (400 MHz, $CDCl_3$): δ = 6.30 (2H, s, Ar<u>H</u>), 4.28-4.44 (8H, m, -OC<u>H</u>$_2$-), 4.19-4.28 (4H, m, -OC<u>H</u>$_2$-) ppm;

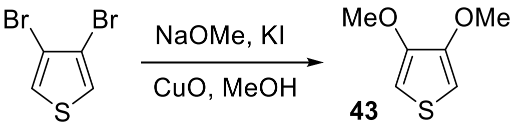

Sodium methoxide solution (25 %, 4.8 mL, 20.6 mmol) was degassed by bubbling through nitrogen for 10 min. Potassium iodide (8 mg, 0.04 mmol), copper (II) oxide (342 mg, 4.3 mmol) and 3,4-dibromothiophene (455 μL, 4.1 mmol) were added and the mixture refluxed for 18 hrs. After cooling to rt, the methanol was removed *in vacuo* and the residue diluted with water (50 mL) and extracted with

diethyl ether (2 x 50 mL). The combined organics were dried with $MgSO_4$, filtered and concentrated *in vacuo* The residue was purified by flash column chromatography, eluting with 0-5 % EtOAc:Hexane. Pure fractions were concentrated *in vacuo* to give the DP as a colourless oil. A yield of 578 mg, 4.0 mmol (98 %) was obtained. Spectroscopic data were consistent with those previously reported.[9] ^{1}H NMR (400 MHz, $CDCl_3$): δ = 6.19 (2H, s, ArH), 3.86 (6H, s, -OMe) ppm;

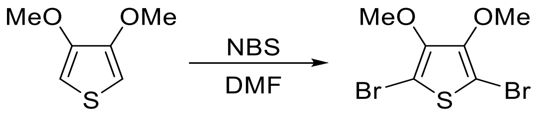

43 **50**

N-Bromosuccinimide (3.26 g, 18.3 mmol) was added to a solution of 3,4-dimethoxythiophene **43** (1 mL, 8.4 mmol) in DMF (5 mL) in the dark and stirred for 1 hr. The mixture was then diluted with diethyl ether (100 mL) and washed with water (100 mL) and brine (2 x 100 mL), dried with $MgSO_4$, filtered and concentrated *in vacuo* to give the DP as a dark brown oil. A yield of 2.7 g, 8.1 mmol (96 %) was obtained. ^{1}H NMR (400 MHz, $CDCl_3$): δ = 3.93 (6H, s, -OMe) ppm; ^{13}C NMR (400 MHz, $CDCl_3$): δ = 147.98 (ArC3), 94.80 (ArC2), 60.95 (-OMe) ppm; IR ($υ_{max}$, solid): 3002, 2939, 2882, 2830, 1566, 1495, 1450, 1424, 1345, 1211, 1204, 1152, 1040, 1009 cm^{-1}; HRMS *m/z* (CI+): Found: 302.8495 (M+H), Calc.: 302.8513;

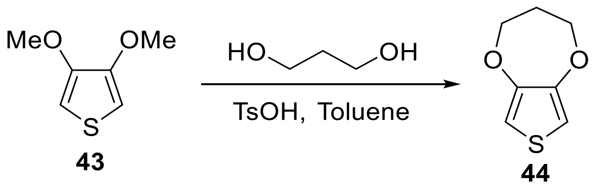

43 **44**

A mixture of 3,4-dimethoxythiophene **43** (2.06 mL, 17.4 mmol), 1,3-propanediol (6.2 mL, 87 mmol), *p*-toluenesulfonic acid monohydrate (299 mg, 1.74 mmol), and anhydrous toluene (250 mL) was degassed via argon bubbling for 30 min. The mixture was then heated to 100 °C for 48 hrs. At this point crude NMR indicated some starting material remained and so a further portion of 1,3-propanediol (5 mL, 69 mmol) and *p*-toluenesulfonic acid (299 mg, 1.74 mmol) were added and heating continued for a further 48 hrs. The mixture was then cooled to room temperature and concentrated *in vacuo*. The residue was diluted with diethyl ether (200 mL) and the organics washed with sodium hydroxide (0.5 M, 100 mL) and water (100 mL), dried with $MgSO_4$, filtered and concentrated onto silica. The residue was purified by flash column chromatography eluting with 10-20 % Et_2O:Hexane. Pure fractions were concentrated *in vacuo* to give the DP as a white solid. A yield of 1.9 g, 12.1 mmol (70 %) was obtained.

The Spectroscopic data were consistent with those previously reported.[10] ^{1}H NMR (400 MHz, $CDCl_3$): δ = 6.55 (2H, s, ArH), 4.10 (4H, t, *J* = 5.3 Hz, -OCH_2-), 2.17-2.27 (2H, m, -OCH_2CH_2-) ppm;

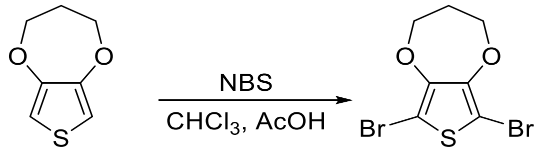

N-Bromosuccinimide (502 mg, 2.82 mmol) was added to a solution of ProDOT **44** (200 mg, 1.28 mmol) in a mixture of chloroform (50 mL) and acetic acid (10 mL) in the dark and stirred for 1 hr. The mixture was then washed with sodium hydroxide (2 M, 3 x 75 mL) and water (100 mL), dried with $MgSO_4$, filtered and concentrated *in vacuo* to give the DP as a colourless oil which solidified at -20 °C. A yield of 405 mg, 1.28 mmol (99 %) was obtained. Spectroscopic data were consistent with those previously reported.[10] ^{1}H NMR (400 MHz, $CDCl_3$): δ = 4.20 (4H, t, *J* = 5.4 Hz, -OCH_2-), 2.24-2.34 (2H, m, -OCH_2CH_2-) ppm;

EDOT functionalisation

Numbering system for functionalised-EDOT NMR assignments

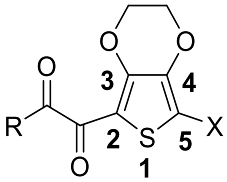

Method A: Oxalyl chloride (850 µL, 10 mmol) was added drop-wise to a solution of EDOT (1.05 mL, 10 mmol) in dioxane (30 mL). The mixture was heated to 100 ºC for 1 hr then allowed to cool to room temperature. The requisite amine (50 mmol) was then added and the mixture stirred for 3 hrs. After this time the mixture was diluted with DCM (150 mL), washed with water (100 mL), and the organics dried with $MgSO_4$, filtered and concentrated *in vacuo*. The residue was purified by flash column chromatography and pure fractions were concentrated *in vacuo*.

Method B: Oxalyl chloride (850 µL, 10 mmol) was added drop-wise to a solution of EDOT (1.05 mL, 10 mmol) in dioxane (30 mL). The mixture was heated to 100 ºC for 1 hr then allowed to cool to room temperature. The requisite amine (15 mmol) and triethylamine (7 mL, 50 mmol) were then added and

the mixture stirred for 3 hrs. After this time the mixture was diluted with DCM (150 mL), washed with water (100 mL), and the organics dried with $MgSO_4$, filtered and concentrated *in vacuo*. The residue was purified by flash column chromatography and pure fractions were concentrated *in vacuo*.

Method C: Oxalyl chloride (850 µL, 10 mmol) was added drop-wise to a solution of EDOT (1.05 mL, 10 mmol) in dioxane (30 mL). The mixture was heated to 100 ºC for 1 hr then allowed to cool to room temperature. The requisite alcohol (30 mL) and triethylamine (7 mL, 50 mmol) was then added and the mixture stirred for 3 hrs. Excess alcohol was then removed *in vacuo* and the mixture diluted with DCM (150 mL), washed with water (100 mL), and the organics dried with $MgSO_4$, filtered and concentrated *in vacuo*. The residue was purified by flash column chromatography and pure fractions were concentrated *in vacuo*.

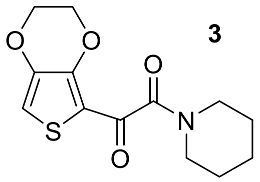

3

Method A with piperidine: Column eluted with 50-60 % EtOAc:Hexane. Yield of 2.34 g, 8.3 mmol (83 %) as a light yellow oil. ^{1}H NMR (400 MHz, $CDCl_3$): δ = 6.85 (1H, s, ArH5), 4.33-4.42 (2H, m, ArC4-OCH_2-), 4.22-4.29 (2H, m, ArC3-OCH_2-), 3.53-3.68 (2H, m, -CH_2N-), 3.30-3.42 (2H, m, -CH_2N-), 1.51-1.77 (6H, m, -$CH_2CH_2CH_2$N-) ppm; ^{13}C NMR (100 MHz, $CDCl_3$): δ = 182.17 (-COCONR_2), 165.09 (-CONR_2), 147.06 (ArC4), 141.79 (ArC3), 115.90 (ArC2), 111.76 (ArC5), 65.53 (ArC4-OCH_2-), 64.06 (ArC3-OCH_2-), 47.19 (-CH_2N-), 46.97 (-CH_2N-), 26.03 (-CH_2CH_2N-), 25.23 (-CH_2CH_2N-), 24.49 (-$CH_2CH_2CH_2$N-) ppm; IR ($υ_{max}$, solid): 3094, 2948, 2926, 2858, 1630, 1620, 1485, 1352, 1432, 1368, 1354, 1254, 1226, 1186, 1178, 1125, 1060, 1032, 1007 cm^{-1}; HRMS *m/z* (ESI+): Found: 282.0797 (M+H), Calc.: 282.0800; m.p. = 140-141 °C;

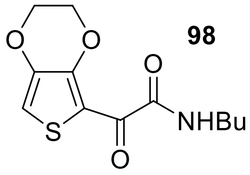

98

Method A with *n*-butylamine: Column eluted with 30-40 % EtOAc:Hexane. Yield of 2.4 g, 8.9 mmol (89 %) as a yellow solid. ^{1}H NMR (400 MHz, $CDCl_3$): δ = 7.40 (1H, br t, J = 6.8 Hz, -NH), 6.87 (1H, s, ArH5), 4.37-4.48 (2H, m, ArC4-OCH_2-), 4.17-4.31 (2H, m, ArC3-OCH_2-), 3.34 (2H, dt, $J_1 = J_2$ = 6.8 Hz, -NHCH_2-), 1.56 (2H, tt, J = 6.8, 7.3 Hz, -NHCH_2CH_2-), 1.37 (2H, tq, $J_1 = J_2$ = 7.3 Hz, -CH_2CH_3), 0.93 (3H, t, J = 7.3 Hz, -CH_3) ppm; ^{13}C NMR (100 MHz, $CDCl_3$): δ = 176.19 (-COCONHBu), 161.40 (-CONHBu), 150.17 (ArC4), 141.45 (ArC3), 115.43 (ArC5), 110.74 (ArC2), 65.57 (ArC4-OCH_2-), 63.87 (ArC3-OCH_2-), 39.15 (-NHCH_2-), 31.28 (-NHCH_2CH_2-), 21.02 (-CH_2CH_3), 14.18 (-CH_3) ppm; IR ($υ_{max}$, solid): 3366, 2952, 2924, 2871, 1682, 1619,

1531, 1472, 1450, 1440, 1416, 1378, 1359, 1291, 1236, 1183, 1175, 1075 cm^{-1}; HRMS *m/z* (ESI+): Found: 270.0810 (M+H), Calc.: 270.0800; m.p. = 85-87 °C;

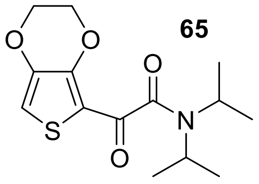

65

Method A with diisopropylamine: Column eluted with 30-50 % EtOAc:Hexane. Yield of 2.75 g, 9.2 mmol (92 %) as a yellow solid. ^1H NMR (400 MHz, $CDCl_3$): δ = 6.82 (1H, s, Ar$\underline{H}$5), 4.29-4.38 (2H, m, ArC4-OC$\underline{H}_2$-), 4.20-4.27 (2H, m, ArC3-OC$\underline{H}_2$-), 3.81 (1H, sept, *J* = 6.8 Hz, -C$\underline{H}$Me$_2$), 3.54 (1H, sept, *J* = 6.8 Hz, -C$\underline{H}$Me$_2$), 1.52 (6H, d, *J* = 6.8 Hz, -CH$\underline{Me}_2$), 1.20 (6H, d, *J* = 6.8 Hz, -CH$\underline{Me}_2$) ppm; ^{13}C NMR (100 MHz, $CDCl_3$): δ = 181.90 (-$\underline{C}$OCONR$_2$), 166.26 (-$\underline{C}$ONR$_2$), 146.74 (Ar$\underline{C}$4), 141.66 (Ar$\underline{C}$3), 116.02 (Ar$\underline{C}$2), 111.30 (Ar$\underline{C}$5), 65.14 (ArC4-O$\underline{C}$H$_2$-), 64.04 (ArC3-O$\underline{C}$H$_2$-), 50.26 (-$\underline{C}$HMe$_2$), 45.79 (-$\underline{C}$HMe$_2$), 20.56 (-CH$\underline{Me}_2$), 20.03 (-CH$\underline{Me}_2$) ppm; IR (υ_{max}, solid): 3101, 3073, 2968, 2933, 2878, 1626, 157, 1489, 1451, 1431, 1371, 1359, 1359, 1274, 1180, 1149, 1117, 1068, 1042, 1019 cm^{-1}; HRMS *m/z* (ESI+): Found: 298.1120 (M+H), Calc.: 298.1113; m.p. = 140-142 °C;

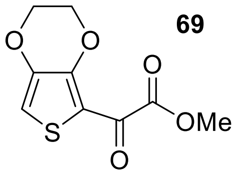

69

Method C with methanol: Column chromatography was not required to generate pure product. Yield of 2.02 g, 8.9 mmol (89 %) as a yellow solid. ^1H NMR (400 MHz, $CDCl_3$): δ = 6.87 (1H, s, Ar$\underline{H}$5), 4.35-4.42 (2H, m, ArC4-OC$\underline{H}_2$-), 4.23-4.39 (2H, m, ArC3-OC$\underline{H}_2$-), 3.93 (3H, s, -O$\underline{Me}$) ppm; ^{13}C NMR (100 MHz, $CDCl_3$): δ = 175.04 (-$\underline{C}$OCO$_2$Me), 163.38 (-$\underline{C}$O$_2$Me), 148.92 (Ar$\underline{C}$4), 141.78 (Ar$\underline{C}$3), 113.48 (Ar$\underline{C}$2), 113.20 (Ar$\underline{C}$5), 65.64 (ArC4-O$\underline{C}$H$_2$-), 63.98 (ArC3-O$\underline{C}$H$_2$-), 53.00 (-O$\underline{Me}$) ppm; IR (υ_{max}, solid): 3100, 2962, 2944, 1729, 1651, 1475, 1440, 1428, 1362, 1316, 1272, 1247, 1227, 1177, 1126, 1069, 1027 cm^{-1}; HRMS *m/z* (ESI+): Found: 229.0165 (M+H), Calc.: 229.0166; m.p. = 105-106 °C;

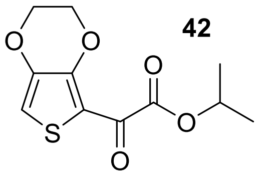

42

Method C with *i*-propanol: Column eluted with 30 % EtOAc:Hexane. Yield of 2.33 g, 9.1 mmol (91 %) as a light yellow oil which solidified on standing. ^1H NMR (400 MHz, $CDCl_3$): δ = 6.85 (1H, s, Ar$\underline{H}$5), 5.23 (1H, sept, *J* = 6.3 Hz, -C$\underline{H}$Me$_2$), 4.32-4.41 (2H, m, ArC4-OC$\underline{H}_2$-), 4.21-4.31 (2H, m, ArC3-OC$\underline{H}_2$-), 1.37 (6H, d, *J* = 6.3 Hz, -CH$\underline{Me}_2$) ppm; ^{13}C NMR (100 MHz, $CDCl_3$): δ = 176.37 (-$\underline{C}$OCO$_2$*i*Pr), 162.98 (-$\underline{C}$O$_2$*i*Pr), 148.38 (Ar$\underline{C}$4), 141.71 (Ar$\underline{C}$3), 114.02 (Ar$\underline{C}$2), 112.62 (Ar$\underline{C}$5), 70.56 (-$\underline{C}$HMe$_2$), 65.40 (ArC4-O$\underline{C}$H$_2$-), 63.99 (ArC3-O$\underline{C}$H$_2$-), 21.57 (-CH$\underline{Me}_2$) ppm; IR (υ_{max}, solid): 2991, 2941, 1731, 1636, 1488, 1439, 1356, 1314, 1246, 1225, 1180, 1104, 1062, 1016 cm^{-1}; HRMS *m/z* (ESI+): Found: 257.0485 (M+H), Calc.: 257.0484; m.p. = 74-77 °C;

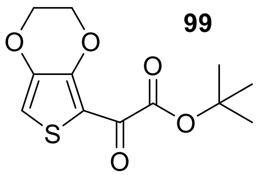

99 Method C with *t*-butanol: Column eluted with 30-50 % EtOAc:Hexane. Yield of 914 mg, 3.38 mmol (38 %) as a yellow oil which solidified on standing. ^{1}H NMR (400 MHz, $CDCl_3$): δ = 6.85 (1H, s, ArH5), 4.34-4.43 (2H, m, ArC4-OCH$_2$-), 4.21-4.33 (2H, m, ArC3-OCH$_2$-), 1.60 (9H, s, -O*t*Bu) ppm; ^{13}C NMR (100 MHz, $CDCl_3$): δ = 176.81 (-COCO$_2$*t*Bu), 162.70 (-CO$_2$*t*Bu), 148.09 (ArC4), 141.65 (ArC3), 114.14 (ArC2), 112.23 (ArC5), 84.34 (-CMe$_3$), 65.30 (ArC4-OCH$_2$-), 64.01 (ArC3-OCH$_2$-), 27.94 (-CMe$_3$) ppm; IR (υ_{max}, solid): 2980, 2935, 1721, 1632, 1488, 1452, 1436, 1359, 1337, 1251, 1228, 1173, 1155, 1127, 1064 cm^{-1}; HRMS *m/z* (ESI+): Found: 271.0643 (M+H), Calc.: 271.0640; m.p. = 64-66 °C;

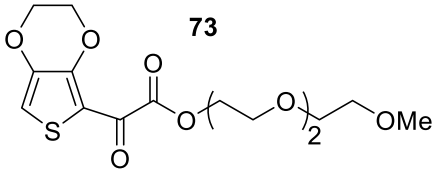

73 Method C with triethylene glycol monomethyl ether: Column eluted with 70-100 % EtOAc:Hexane. Yield of 3.3 g, 9.2 mmol (92 %) as a yellow oil. ^{1}H NMR (400 MHz, $CDCl_3$): δ = 6.85 (1H, s, ArH5), 4.43-4.47 (2H, m, TEG), 4.34-4.38 (2H, m, ArC4-OCH$_2$-), 4.21-4.25 (2H, m, ArC3-OCH$_2$-), 3.77-3.81 (2H, m, TEG), 3.65-3.69 (2H, m, TEG), 3.59-3.65 (4H, m, TEG), 3.49-3.53 (2H, m, TEG), 3.34 (3H, s, -OMe) ppm; ^{13}C NMR (100 MHz, $CDCl_3$): δ = 175.37 (-COCO$_2$R), 163.11 (-CO$_2$R), 148.78 (ArC4), 141.77 (ArC3), 113.71 (ArC2), 112.96 (ArC5), 71.87 (TEG), 70.70 (TEG), 70.59 (TEG), 70.54 (TEG), 68.55 (TEG), 65.61 (ArC4-OCH$_2$-), 65.31 (TEG), 64.02 (ArC3-OCH$_2$-), 58.97 (-OMe) ppm; IR (υ_{max}, oil): 2876, 1740, 1638, 1488, 1452, 1434, 1360, 1317, 1248, 1220, 1174, 1121, 1099, 1064, 1025 cm^{-1}; HRMS *m/z* (ESI+): Found: 361.0966 (M+H), Calc.: 361.0957;

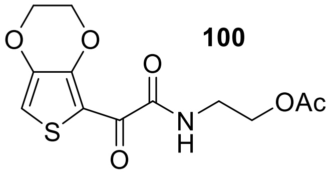

100 Method B with amine **83**: Column eluted with 50-70 % EtOAc:Hexane. Yield of 1.9 g, 6.3 mmol (63 %) as a yellow solid. ^{1}H NMR (400 MHz, $CDCl_3$): δ = 7.65 (1H, br t, *J* = 6.2 Hz, -NH), 6.89 (1H, s, ArH5), 4.41-4.47 (2H, m, ArC4-OCH$_2$-), 4.24-4.28 (2H, m, ArC3-OCH$_2$-), 4.22 (2H, t, *J* = 5.4 Hz, -CH$_2$OAc), 3.63 (2H, dt, *J* = 6.2, 5.4 Hz, -NHCH$_2$-), 2.08 (3H, s, -OAc) ppm; ^{13}C NMR (100 MHz, $CDCl_3$): δ = 175.67 (-COCONHR), 170.81 (MeCO$_2$-), 161.64 (-CONHR), 150.39 (ArC4), 141.54 (ArC3), 115.60 (ArC5), 110.64 (ArC2), 65.60 (ArC4-OCH$_2$-), 63.89 (ArC3-OCH$_2$-), 62.60 (-CH$_2$OAc), 38.44 (-NHCH$_2$-), 20.79 (MeCO$_2$-) ppm; IR (υ_{max}, solid): 3346, 3085, 2924, 1718, 1682, 1637, 1524, 1473, 1464, 1441, 1422, 1369, 1357, 1266, 1253, 1241, 1190, 1114, 1069, 1051 cm^{-1}; HRMS *m/z* (ESI+): Found: 300.0534 (M+H), Calc.: 300.0542; m.p. = 105-108 °C;

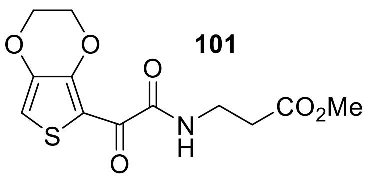

101

Method B using only 6 mmol of amine **89**: Column eluted 30-50 % EtOAc:Hexane. Yield of 1.3 g, 4.2 mmol (72 %) as a yellow solid. ^{1}H NMR (400 MHz, $CDCl_3$): δ = 7.80 (1H, br t, J = 6.4 Hz, -NH), 6.89 (1H, s, ArH5), 4.42-4.50 (2H, m, ArC4-OCH_2-), 4.23-4.29 (2H, m, ArC3-OCH_2-), 3.72 (3H, s, -CO_2Me), 3.65 (2H, dt, J_1 = J_2 = 6.4 Hz, -$NHCH_2$-), 2.64 (2H, t, J = 6.4 Hz, -CH_2CO_2Me) ppm; ^{13}C NMR (100 MHz, $CDCl_3$): δ = 175.78 (-COCONHR), 172.09 (-CO_2Me), 161.53 (-CONHR), 150.28 (ArC4), 141.52 (ArC3), 115.39 (ArC5), 110.71 (ArC2), 65.59 (ArC4-OCH_2-), 63.90 (ArC3-OCH_2-), 51.94 (-CO_2Me), 34.90 (-$NHCH_2$-), 33.58 (-CH_2CO_2Me) ppm; IR (υ$_{max}$, solid): 2938, 2856, 1716, 1683, 1634, 1620, 1606, 1546, 1467, 1435, 1375, 1361, 1217, 1196, 1171, 1155, 1132, 1069 cm^{-1}; HRMS *m/z* (ESI+): Found: 300.0550 (M+H), Calc.: 300.0542; m.p. = 120-125 °C;

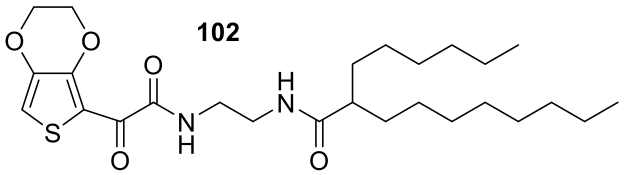

102

Method B with amine **91**: Column eluted with 60-80 % EtOAc:Hexane. Yield of 3.4 g, 6.8 mmol (68 %) as a light yellow solid. ^{1}H NMR (400 MHz, $CDCl_3$): δ = 7.82 (1H, br t, J = 5.9 Hz, -NHCOCOAr), 6.88 (1H, s, ArH5), 6.06 (1H, br t, J = 5.1 Hz, -NHCOAlk), 4.38-4.47 (2H, m, ArC4-OCH_2-), 4.28-4.37 (2H, m, ArC3-OCH_2-), 3.39-3.59 (4H, m, -$NHCH_2CH_2NH$-), 1.95-2.05 (1H, m, $H_α$), 1.48-1.63 (2H, m, $H_β$), 1.31-1.45 (2H, m, $H_β$), 1.11-1.31 (20H, m, Alkyl), 0.81-0.92 (6H, m, -CH_3) ppm; ^{13}C NMR (100 MHz, $CDCl_3$): δ = 176.84 (-NHCOAlkyl) 175.46 (-COCONHR), 162.52 (-COCONHR), 150.37 (ArC4), 141.55 (ArC3), 115.39 (ArC5), 110.61 (ArC2), 65.56 (ArC4-OCH_2-), 63.89 (ArC3-OCH_2-), 48.11 ($C_α$), 39.47 (-$NHCH_2$-), 39.41 (-$NHCH_2$-), 33.01 ($C_β$), 31.84 (Alkyl), 31.67 (Alkyl), 29.44 (Alkyl), 29.35 (Alkyl), 29.32 (Alkyl), 22.66 (Alkyl), 22.63 (Alkyl), 14.11 (-CH_3), 14.06 (-CH_3) ppm; IR (υ$_{max}$, solid): 3280, 2923, 2852, 1638, 1469, 1423, 1360, 1252, 1216, 1174, 1117, 1080, 1065 cm^{-1}; HRMS *m/z* (ESI+): Found: 495.2877 (M+H), Calc.: 495.2893; m.p. = 141-143 °C;

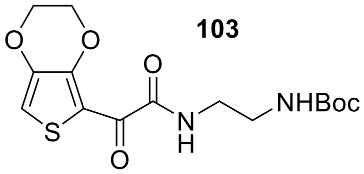

103

Method B with amine **88**: Column eluted with 30-60 % EtOAc:Hexane. Yield of 1.7 g, 4.8 mmol (48 %) as a yellow solid. ^{1}H NMR (400 MHz, $CDCl_3$): δ = 7.80 (1H, br app s, -NHCOCOAr), 6.88 (1H, s, ArH5), 4.96 (1H, br app s, -NHBoc), 4.36-4.48 (2H, m, ArC4-OCH_2-), 4.21-4.29 (2H, m, ArC3-OCH_2-), 3.49 (2H td, J = 6.6, 5.0 Hz, -$CH_2NHCOCOAr$), 3.35 (2H, dt, J_1 = J_2 = 6.6 Hz, -CH_2NHBoc), 1.43 (9H, s, Boc) ppm; ^{13}C NMR (100 MHz, $CDCl_3$): δ = 175.80 (-COCONHR), 162.09 (-COCONHR), 156.22 (-CO_2*t*Bu), 150.27

(ArC4), 141.50 (ArC3), 115.40 (ArC5), 110.70 (ArC2), 79.71 (-CMe$_3$), 65.56 (ArC4-OCH$_2$-), 63.89 (ArC3-OCH$_2$-), 39.93 (-CH$_2$NH-), 28.35 (-CMe$_3$) ppm; IR (υ_{max}, solid): 3360, 3309, 2980, 2933, 2873, 1735, 1703, 1677, 1621, 1508, 1470, 1439, 1426, 1358, 1264, 1246, 1224, 1163, 1142, 1118, 1071 cm^{-1}; HRMS *m/z* (ESI+): Found: 357.1137 (M+H), Calc.: 357.1120; m.p. = 81-83 °C;

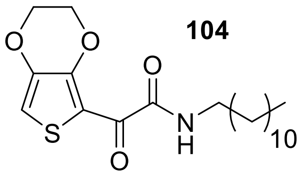

104

Method B with dodecylamine: Column eluted with 20-30 % EtOAc:Hexane. Yield of 3.6 g, 9.4 mmol (94 %) as a yellow solid. ^{1}H NMR (400 MHz, CDCl$_3$): δ = 7.41 (1H, br t, *J* = 6.1 Hz, -NH), 6.89 (1H, s, ArH5), 4.39-4.49 (2H, m, ArC4-OCH$_2$-), 4.21-4.32 (2H, m, ArC3-OCH$_2$-), 3.28-3.41 (2H, m, -NHCH$_2$-), 1.54-1.63 (2H, m, -NHCH$_2$CH$_2$-), 1.24-1.41 (18H, m, Alkyl), 0.89 (3H, t, *J* = 6.8 Hz, -CH$_3$) ppm; ^{13}C NMR (100 MHz, CDCl$_3$): δ = 176.21 (-COCONH-), 161.37 (-CONH-), 150.20 (ArC4), 141.46 (ArC3), 115.49 (ArC5), 110.77 (ArC2), 65.59 (ArC4-OCH$_2$-), 63.89 (ArC3-OCH$_2$-), 39.47 (-NHCH$_2$-), 31.91 (-NHCH$_2$CH$_2$-), 29.62 (Alkyl), 29.55 (Alkyl), 29.50 (Alkyl), 29.34 (Alkyl), 29.26 (Alkyl), 29.22 (Alkyl), 26.86 (Alkyl), 22.69 (Alkyl), 14.13 (-CH$_3$) ppm; IR (υ_{max}, solid): 3326, 2921, 2848, 1657, 1633, 1530, 1482, 1462, 1450, 1425, 1367, 1176, 1079, 1069 cm^{-1}; HRMS *m/z* (ESI+): Found: 382.2053 (M+H), Calc.: 382.2052; m.p. = 77-78 °C;

Monomer bromination

Bromination procedure A: EDOT derivative (5 mmol) was dissolved in a mixture of THF (5 mL) and acetic acid (3 mL). If solubility was poor a further 25 mL of THF was added. The mixture was placed in the dark and *N*-bromosuccinimide (6 mmol) was added. After stirring for 2 hrs the mixture was poured into water (150 mL) causing precipitation of the product. The solid was collected by filtration or centrifugation (5000 rpm, 10 min), washed with water (50 mL) and then dissolved in DCM. The organics were dried with MgSO$_4$, filtered and concentrated *in vacuo*. Column chromatography was then undertaken if required.

Bromination procedure B: Reaction procedure A was followed. After pouring into water the product was extracted with DCM (200 mL). The organics were washed with sat. NaHCO$_3$ (2 x 100 mL), dried with MgSO$_4$, filtered and concentrated *in vacuo*. Column chromatography was then undertaken if required.

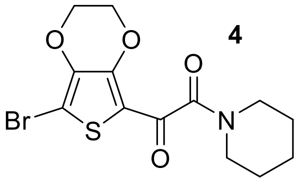

4

Method B on 3.9 mmol scale: Yield of 1.4 g, 3.9 mmol (99 %) as a yellow solid. ^{1}H NMR (400 MHz, CDCl$_3$): δ = 4.35-4.42 (2H, m, ArC4-OCH$_2$-), 4.30-4.35 (2H, m, ArC3-OCH$_2$-), 3.60-3.65 (2H, m, -CH$_2$N-), 3.34-3.39 (2H, m, -CH$_2$N-), 1.52-1.74 (6H, m, -CH$_2$CH$_2$CH$_2$N-) ppm; ^{13}C NMR (100 MHz, CDCl$_3$): δ = 180.86 (-COCON-), 164.67 (-CON-), 146.29 (ArC4), 140.27 (ArC3), 115.91 (ArC2), 102.66 (ArC5), 65.54 (ArC4-OCH$_2$-), 64.49 (ArC3-OCH$_2$-), 46.99 (-CH$_2$N-), 42.33 (-CH$_2$N-), 26.09 (-CH$_2$CH$_2$N-), 25.24 (-CH$_2$CH$_2$N-), 24.45 (-CH$_2$CH$_2$CH$_2$N-) ppm; IR (υ_{max}, solid): 3342, 1946, 2859, 1627, 1491, 1478, 1425, 1354, 1252, 1124, 1080 cm^{-1}; HRMS *m/z* (ESI+): Found: 359.9904/361.9890 (M+H), Calc.: 359.9905/361.9885; m.p. = 157-160 °C;

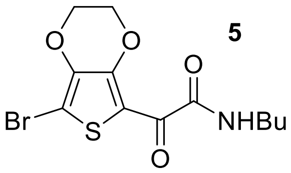

5

Method A on 18.6 mmol scale: Yield of 6.4 g, 18.5 mmol (99 %) as a yellow solid. ^{1}H NMR (400 MHz, CDCl$_3$): δ = 7.38 (1H, br t, *J* = 6.2 Hz, -NH), 4.41-4.50 (2H, m, ArC4-OCH$_2$-), 4.32-4.39 (2H, m, ArC3-OCH$_2$-), 3.36 (2H, td, *J* = 7.1, 6.2 Hz, -NHCH$_2$-), 1.58 (2H, tt, *J* = 7.6, 7.1 Hz, -NHCH$_2$CH$_2$-), 1.39 (2H, tq, *J* = 7.6, 7.3 Hz, -CH$_2$CH$_3$), 0.96 (3H, t, *J* = 7.3 Hz, -CH$_3$) ppm; ^{13}C NMR (100 MHz, CDCl$_3$): δ = 175.05 (-COCONHBu), 161.29 (-CONHBu), 149.44 (ArC4), 139.99 (ArC3), 110.71 (ArC2), 106.88 (ArC5), 65.58 (ArC4-OCH$_2$-), 64.35 (ArC3-OCH$_2$-), 39.25 (-NHCH$_2$-), 31.27 (-NHCH$_2$CH$_2$-), 20.03 (-CH$_2$CH$_3$), 13.69 (-CH$_3$) ppm; IR (υ_{max}, solid): 3308, 2954, 2930, 2869, 1659, 1526, 1545, 1480, 1456, 1442, 1418, 1367, 1355, 1267, 1247, 1229, 1142, 1084 cm^{-1}; HRMS *m/z* (ESI+): Found: 347.9906/349.9891 (M+H), Calc.: 347.9905/349.9884; m.p. = 160-164 °C;

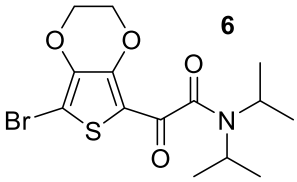

6

Method B on 10 mmol scale: Yield of 3.75 g, 9.9 mmol (99 %) as a yellow oil which solidified on standing. ^{1}H NMR (400 MHz, CDCl$_3$): δ = 4.34 (4H, app s, -OCH$_2$-), 3.81 (1H, sept, *J* = 6.6 Hz, -CHMe$_2$), 3.55 (1H, sept, *J* = 6.6 Hz, -CHMe$_2$), 1.51 (6H, d, *J* = 6.6 Hz, -CHMe$_2$), 1.21 (6H, d, *J* = 6.6 Hz, -CHMe$_2$) ppm; ^{13}C NMR (100 MHz, CDCl$_3$): δ = 180.70 (-COCON-), 165.88 (-CON-), 145.91 (ArC4), 140.15 (ArC3), 116.07 (ArC2), 102.04 (ArC5), 65.15 (ArC4-OCH$_2$-), 64.49 (ArC3-OCH$_2$-), 50.29 (-CHMe$_2$), 45.85 (-CHMe$_2$), 20.60 (-CHMe$_2$), 19.98 (-CHMe$_2$) ppm; IR (υ_{max}, solid): 3360, 3297, 2924, 2853, 1631, 1545, 1475, 1422, 1355, 1270, 1249, 1233, 147, 1085 cm^{-1}; HRMS *m/z* (ESI+): Found: 376.0246/378.0227 (M+H), Calc.: 376.0243/378.0198; m.p. = 137-139 °C;

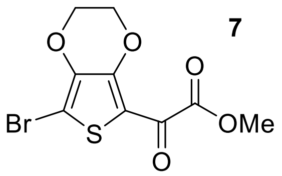

7 Method A, washing DCM with sat. $NaHCO_3$ (50 mL) before drying and concentrating. Yield of 1.42 g, 4.6 mmol (93 %) as a yellow solid. ^{1}H NMR (400 MHz, $CDCl_3$): δ = 4.40-4.45 (2H, m, ArC4-OCH_2-), 4.31-4.37 (2H, m, ArC3-OCH_2-), 3.93 (3H, s, -OMe) ppm; ^{13}C NMR (100 MHz, $CDCl_3$): δ = 173.24 (-$COCO_2$Me), 162.88 (-CO_2Me), 148.35 (ArC4), 140.33 (ArC3), 113.26 (ArC2), 104.38 (ArC5), 65.60 (ArC4-OCH_2-), 64.43 (ArC3-OCH_2-), 53.20 (-OMe) ppm; IR (υ_{max}, solid): 3360, 3296, 2923, 2852, 1680, 1632, 1544, 1475, 1421, 1357, 1248, 1235, 1089 cm^{-1}; HRMS *m/z* (ESI+): Found: 306.9282/308.9269 (M+H), Calc.: 306.9276/308.9255; m.p. = 133-137 °C;

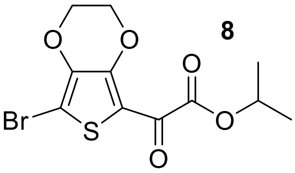

8 Method B on 13.6 mmol scale: Yield of 4.2 g, 12.5 mmol (92 %) as a yellow oil which slowly solidified on standing. ^{1}H NMR (400 MHz, $CDCl_3$): δ = 5.23 (1H, sept, *J* = 6.3 Hz, -$CHMe_2$), 4.38-4.42 (2H, m, ArC4-OCH_2-), 4.33-4.38 (2H, m, ArC3-OCH_2-), 1.38 (6H, d, *J* = 6.3 Hz, $CHMe_2$) ppm; ^{13}C NMR (100 MHz, $CDCl_3$): δ = 174.56 (-$COCO_2$*t*Bu), 162.37 (-CO_2*t*Bu), 147.83 (ArC4), 140.23 (ArC3), 113.78 (ArC2), 103.77 (ArC5), 70.95 (-$CHMe_2$), 65.39 (ArC4-OCH_2-), 64.44 (ArC3-OCH_2-), 21.58 (-$CHMe_2$) ppm; IR (υ_{max}, solid): 2982, 2934, 2876, 1735, 1713, 1641, 1480, 1472, 1430, 1356, 1314, 1246, 1221, 1174, 1131, 1099, 1075, 1035 cm^{-1}; HRMS *m/z* (ESI+): Found: 334.9583/336.9562 (M+H), Calc.: 334.9589/336.9568; m.p. = 71-73 °C;

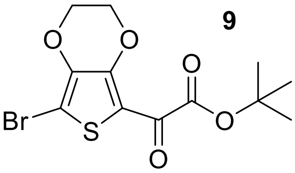

9 Method A on 1.22 mmol scale. DMF was used as solvent in place of THF/AcOH to prevent hydrolysis of the *t*-butyl group. Yield of 360 mg, 1.03 mmol (85 %) as a yellow solid. ^{1}H NMR (400 MHz, $CDCl_3$): δ = 4.37-4.41 (2H, m, ArC4-OCH_2-), 4.33-4.37 (2H, m, ArC3-OCH_2-), 1.60 (9H, s, -O*t*Bu) ppm; ^{13}C NMR (100 MHz, $CDCl_3$): δ = 175.21 (-$COCO_2$*t*Bu), 162.11 (-CO_2*t*Bu), 147.51 (ArC4), 140.18 (ArC3), 113.88 (ArC2), 103.23 (ArC5), 84.67 (-CMe_3), 65.31 (ArC4-OCH_2-), 64.45 (ArC3-OCH_2-), 27.80 (-CMe_3) ppm; IR (υ_{max}, solid): 2980, 2934, 1715, 1647, 1485, 1473, 1428, 1367, 1357, 1331, 1248, 1226, 1165, 1128, 1080, 1033 cm^{-1}; HRMS *m/z* (ESI+): Found: 348.9734/350.9714 (M+H), Calc.: 348.9745/350.9725; m.p. = 104-106 °C;

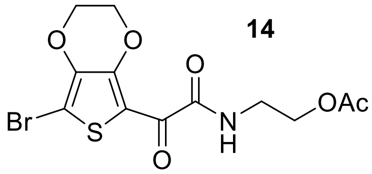

14 Method B on 1.87 mmol scale: Purified by column chromatography eluting with 50 % EtOAc:Hexane. Yield of 602 mg, 1.59 mmol (85 %) as a yellow solid. ^{1}H NMR (400 MHz, $CDCl_3$): δ = 7.64 (1H, br t, *J* = 6.3 Hz, -NH), 4.42-4.49 (2H, m, ArC4-OCH_2-), 4.30-4.40 (2H, m, ArC3-OCH_2-), 4.22 (2H, t, *J* = 5.4 Hz,

-CH2OAc), 3.62 (2H, dt, *J* = 6.3, 5.4 Hz, -NHCH2-), 2.08 (3H, s, -OAc) ppm; ^{13}C NMR (100 MHz, $CDCl_3$): δ = 174.48 (-COCONH-), 170.83 ($MeCO_2$-), 161.53 (-CONH-), 149.63 (ArC4), 140.07 (ArC3), 110.58 (ArC2), 106.99 (ArC5), 65.58 (ArC4-OCH_2-), 64.35 (ArC3-OCH_2-), 62.54 (-CH_2OAc), 38.54 (-NHCH_2-), 20.80 ($MeCO_2$-) ppm; IR ($υ_{max}$, solid): 3345, 2947, 1723, 1680, 1626, 1543, 1474, 1442, 1422, 1355, 1245, 1221, 1200, 1148, 1081, 1037 cm^{-1}; HRMS *m/z* (ESI+): Found: 377.9654/379.9630 (M+H), Calc.: 377.9647/379.9630; m.p. = 143-147 °C;

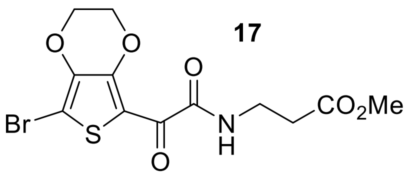

17

Method A, washing DCM with sat. $NaHCO_3$ (50 mL) before drying and concentrating. Yield of 1.72 g, 4.6 mmol (93 %) as a yellow solid. ^{1}H NMR (400 MHz, $CDCl_3$): δ = 7.79 (1H, br s, -NH), 4.40-4.49 (2H, m, ArC4-OCH_2-), 4.30-4.39 (2H, m, ArC3-OCH_2-), 3.73 (3H, s, -CO_2Me), 3.65 (2H, dt, J_1 = J_2 = 6.3 Hz, -NHCH_2-), 2.63 (2H, t, *J* = 6.4 Hz, -CH_2CO_2Me) ppm; ^{13}C NMR (100 MHz, $CDCl_3$): δ = 174.61 (-COCONH-), 172.06 (-CO_2Me), 161.40 (-CONH-), 149.50 (ArC4), 140.04 (ArC3), 110.66 (ArC2), 106.75 (ArC5), 65.56 (ArC4-OCH_2-), 64.36 (ArC3-OCH_2-), 51.97 (-CO_2Me), 34.95 (-NHCH_2-), 33.52 (-CH_2CO_2Me) ppm; IR ($υ_{max}$, solid): 3344, 2951, 1725, 1680, 1627, 1546, 1475, 1423, 1357, 1321, 1295, 1199, 1176, 1079 cm^{-1}; HRMS *m/z* (ESI+): Found: 377.9645/379.9630 (M+H), Calc.: 377.9647/379.9626; m.p. = 199-203 °C (Degrades);

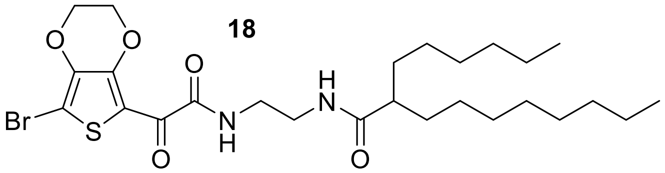

18

Method A on 3.4 mmol scale: Yield of 1.9 g, 3.3 mmol (98 %) as a yellow solid. ^{1}H NMR (400 MHz, $CDCl_3$): δ = 7.82 (1H, br t, *J* = 5.6 Hz, -NHCOCO-), 6.06 (1H, br t, *J* = 5.1 Hz, -NHCOAlk), 4.40-4.48 (2H, m, ArC4-OCH_2-), 4.30-4.38 (2H, m, ArC3-OCH_2-), 3.45-3.56 (4H, m, -NHCH_2CH_2NH-), 1.95-2.05 (1H, m, $H_α$), 1.51-1.62 (2H, m, $H_β$), 1.33-1.44 (2H, m, $H_β$), 1.15-1.32 (20H, m, Alkyl), 0.81-0.91 (6H, m, -CH_3) ppm; ^{13}C NMR (100 MHz, $CDCl_3$): δ = 176.92 (-NHCOAlkyl) 174.28 (-COCONH-), 162.37 (-COCONH-), 149.58 (ArC4), 140.06 (ArC3), 110.54 (ArC2), 106.76 (ArC5), 65.54 (ArC4-OCH_2-), 64.53 (ArC3-OCH_2-), 48.11 ($C_α$), 39.64 (-NHCH_2-), 39.32 (-NHCH_2-), 33.00 ($C_β$), 31.86 (Alkyl), 29.35 (Alkyl), 27.65 (Alkyl), 22.67 (Alkyl), 22.64 (Alkyl), 14.12 (-CH_3), 14.07 (-CH_3) ppm; IR ($υ_{max}$, solid): 3360, 3294, 2923, 2852, 1680, 1635, 1544, 1474, 1444, 1421, 1358, 1296, 1248, 1235, 1145, 1089 cm^{-1}; HRMS *m/z* (ESI+): Found: 573.1982/575.1966 (M+H), Calc.: 573.1998/575.1978; m.p. = 159-162 °C;

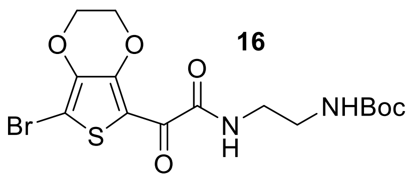

Method A on 1.96 mmol scale. DMF was used as solvent in place of THF/AcOH to prevent Boc cleavage. Purification by flash column chromatography was undertaken eluting with 30-60 % EtOAc:Hexane Yield of 746 mg, 1.71 mmol (87 %) as a yellow solid. ^{1}H NMR (400 MHz, CDCl$_3$): δ = 7.81 (1H, app s, -CONHR), 4.93 (1H, app s, -NHBoc), 4.41-4.49 (2H, m, ArC4-OCH$_2$-), 4.29-4.39 (2H, m, ArC3-OCH$_2$-), 3.49 (2H, dt, $J_1 = J_2$ = 7.1 Hz, -CONHCH$_2$-), 3.36 (2H, dt, $J_1 = J_2$ = 7.1 Hz, -CH$_2$NHBoc), 1.45 (9H, s, Boc) ppm; ^{13}C NMR (100 MHz, CDCl$_3$): δ = 174.60 (-COCONH-), 161.93 (-CONH-), 156.25 (-CO$_2$*t*Bu), 149.50 (ArC4), 140.03 (ArC3), 110.63 (ArC2), 106.75 (ArC5), 79.78 (-CMe$_3$), 65.55 (ArC4-OCH$_2$-), 64.35 (ArC3-OCH$_2$-), 40.09 (-CH$_2$NH-), 39.93 (-CH$_2$NH-), 28.36 (-CMe$_3$) ppm; IR (υ$_{max}$, solid): 2981, 2942, 2879, 1684, 1640, 1528, 1478, 1421, 1367, 1276, 1247, 1235, 1167, 1144, 1089 cm^{-1}; HRMS *m/z* (ESI+): Found: 435.0232/437.0220 (M+H), Calc.: 435.0225/437.0205; m.p. = 162-167 °C;

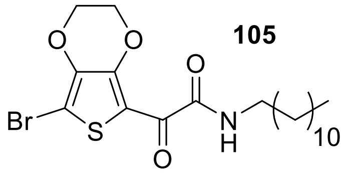

Method A on 4.2 mmol scale: Yield of 1.2 g, 2.6 mmol (62 %) as a yellow solid. ^{1}H NMR (400 MHz, CDCl$_3$): δ = 7.37 (1H, br t, J = 6.1 Hz, -NH), 4.41-4.50 (2H, m, ArC4-OCH$_2$-), 4.31-4.38 (2H, m, ArC3-OCH$_2$-), 3.34 (2H, dt, $J_1 = J_2$ = 6.9 Hz, -NHCH$_2$-), 1.52-1.66 (2H, m, -NHCH$_2$CH$_2$-), 1.20-1.38 (18H, m, Alkyl), 0.89 (3H, t, J = 6.7 Hz, -CH$_3$) ppm; ^{13}C NMR (100 MHz, CDCl$_3$): δ = 175.05 (-COCONH-), 161.26 (-CONH-), 149.41 (ArC4), 139.98 (ArC3), 110.72 (ArC2), 106.84 (ArC5), 65.57 (ArC4-OCH$_2$-), 64.35 (ArC3-OCH$_2$-), 39.54 (-NHCH$_2$-), 31.91 (-NHCH$_2$CH$_2$-), 29.62 (Alkyl), 29.54 (Alkyl), 29.49 (Alkyl), 29.34 (Alkyl), 29.21 (Alkyl), 26.84 (Alkyl), 22.69 (Alkyl), 14.13 (-CH$_3$) ppm; IR (υ$_{max}$, solid): 2919, 2869, 2849, 1628, 1468, 1435, 1424, 1356, 1264, 1222, 1211, 1077, 1064, 1042 cm^{-1}; HRMS *m/z* (ESI+): Found: 460.1165/462.1144 (M+H), Calc.: 460.1157/462.1136; m.p. = 132-134 °C;

Monomer manipulations

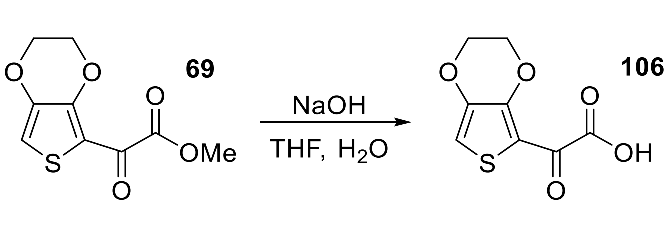

Sodium hydroxide (2 M, 5 mL, 10 mmol) was added to a solution of methoxy-EDOT **69** (0.5 g, 2.2 mmol) in THF (10 mL) and the mixture stirred for 2 hrs. The THF was then removed *in vacuo* and the residue

acidified with hydrochloric acid (1 M). The resultant precipitate was extracted with ethyl acetate (2 x 50 mL) and the organics dried with $MgSO_4$, filtered and concentrated *in vacuo* to give the DP as a yellow solid. ^{1}H NMR (400 MHz, DMSO-d_6): δ = 14.32 (1H, br s, -CO_2H), 7.36 (1H, s, ArH5), 4.34-4.42 (2H, m, ArC4-OCH_2-), 4.22-4.32 (2H, m, ArC3-OCH_2-) ppm; ^{13}C NMR (100 MHz, DMSO-d_6): δ = 178.41 (-$COCO_2H$), 165.80 (-CO_2H), 148.96 (ArC4), 142.15 (ArC3), 113.57 (ArC5), 113.22 (ArC2), 66.09 (ArC4-OCH_2-), 64.34 (ArC3-OCH_2-) ppm; IR (u_{max}, solid): 2953, 1754, 1621, 1487, 1454, 1436, 1366, 1335, 1262, 1253, 12284, 1070 cm^{-1}; HRMS *m/z* (ESI-): Found: 212.9866 (M-H), Calc.: 292.9858; m.p. = 219-221 °C (Degrades);

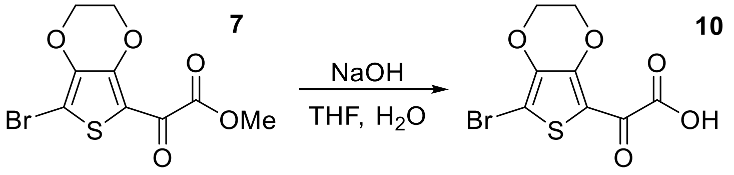

Sodium hydroxide (2 M, 15 mL, 30 mmol) was added to a solution of bromo-methoxy-EDOT **7** (2.8 g, 9.3 mmol) in THF (50 mL) and the mixture stirred for 2 hrs. The THF was then removed *in vacuo* and the residue acidified with hydrochloric acid (1 M). The resultant precipitate was collected by filtration, washed with water (2 x 50 mL) and dried *in vacuo* to give the DP as a yellow-green solid. A yield of 1.96 g, 6.7 mmol (72 %) was obtained. ^{1}H NMR (400 MHz, MeOD): δ = 4.40-4.45 (2H, m, ArC4-OCH_2-), 4.34-4.40 (2H, m, ArC3-OCH_2-) ppm; ^{13}C NMR (100 MHz, MeOD): δ = 175.42 (-$COCO_2H$), 164.33 (-CO_2H), 148.66 (ArC4), 140.65 (ArC3), 112.53 (ArC2), 103.07 (ArC5), 65.56 (ArC4-OCH_2-), 64.44 (ArC3-OCH_2-) ppm; IR (u_{max}, solid): 3257, 2949, 1749, 1615, 1479, 1451, 1438, 1372, 1357, 1336, 1269, 1167, 1083, 1005 cm^{-1}; HRMS *m/z* (ESI-): Found: 290.8956/292.8888 (M-H), Calc.: 290.8956/292.8948; m.p. = 222-227 °C;

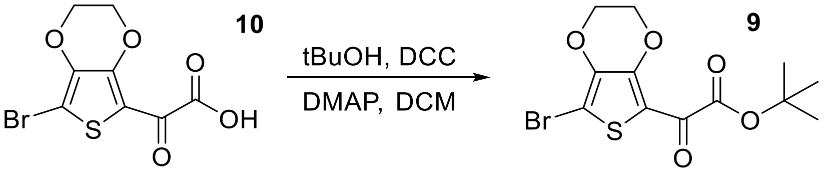

N,N'-Dicyclohexylcarbodiimide (2.12 g, 10.3 mmol) and 4-dimethylaminopyridine (83 mg, 0.68 mmol) were added to a suspension of carboxy-EDOT **10** (2 g, 6.8 mmol) in DCM (100 mL) and *tert*-butanol (5 mL). The mixture initially solubilised and then a white precipitate gradually formed. After stirring for 18 hrs the reaction was filtered and concentrated *in vacuo*. The residue was purified by flash column

chromatography eluting with 20-30 % EtOAc:Hexane. Pure fractions were concentrated *in vacuo* to give the DP as a yellow oil which solidified on standing. A yield of 1.9 g, 5.5 mmol (81 %) was obtained. Spectroscopic data were consistent with those reported above.

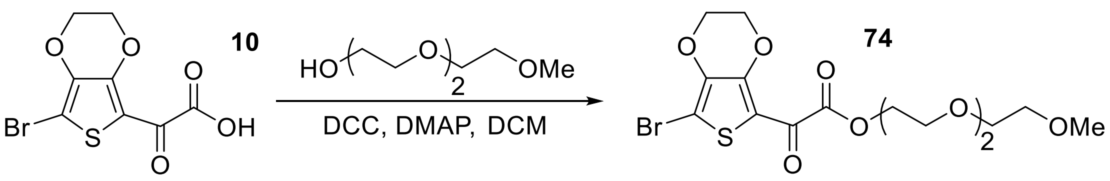

N,N'-Dicyclohexylcarbodiimide (2.32 g, 11.2 mmol) and 4-dimethylaminopyridine (92 mg, 0.75 mmol) were added to a suspension of carboxy-EDOT **10** (2.2 g, 7.5 mmol) in DCM (100 mL). The mixture initially solubilised and then a white precipitate gradually formed. After stirring for 18 hrs the reaction was filtered and concentrated *in vacuo*. The residue was purified by flash column chromatography eluting with 40-90 % EtOAc:Hexane. Product containing fractions were concentrated *in vacuo* and the residue triturated in water (3 x 70 mL), dissolved in DCM, dried with $MgSO_4$, filtered and concentrated *in vacuo* to give the DP as a yellow oil. A yield of 1.7 g, 3.88 mmol (52 %) was obtained. ^{1}H NMR (400 MHz, $CDCl_3$): δ = 4.45-4.49 (2H, m, TEG), 4.39-4.43 (2H, m, ArC4-OC$\underline{H}_2$-), 4.32-4.37 (2H, m, ArC3-OC$\underline{H}_2$-), 3.80-3.84 (2H, m, TEG), 3.67-3.72 (2H, m, TEG), 3.62-3.67 (4H, m, TEG), 3.52-3.56 (2H, m, TEG), 3.38 (3H, s, -O$\underline{Me}$) ppm; ^{13}C NMR (100 MHz, $CDCl_3$): δ = 173.65 (-$\underline{C}$OCO$_2$R), 162.59 (-$\underline{C}$O$_2$R), 148.19 (Ar$\underline{C}$4), 140.31 (Ar$\underline{C}$3), 113.54 (Ar$\underline{C}$5), 104.08 (Ar$\underline{C}$2), 71.91 (TEG), 70.74 (TEG), 70.63 (TEG), 70.58 (TEG), 68.51 (TEG), 65.59 (ArC4-O$\underline{C}$H$_2$-), 65.55 (TEG), 64.48 (ArC3-O$\underline{C}$H$_2$-), 59.02 (-O$\underline{Me}$) ppm; IR (υ_{max}, film): 2876, 1728, 1642, 1494, 1479, 1427, 1355, 1314, 1245, 1215, 1124, 1101, 1084, 1028 cm^{-1}; HRMS *m/z* (ESI+): Found: 460.9871/462.9862 (M+Na), Calc.: 460.9882/462.9861;

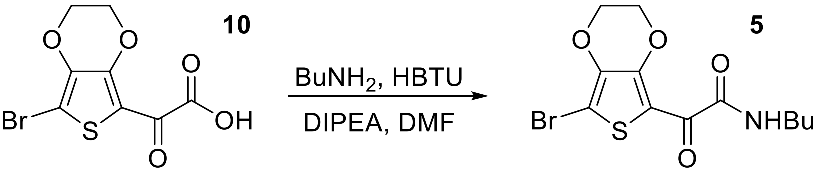

Bromo-carboxy-EDOT **10** (29 mg, 100 µmol) and HBTU (57 mg, 150 µmol) were dissolved in DMF (0.5 mL). *n*-Butylamine (12 µL, 120 µmol) and DIPEA (35 µL, 200 µmol) were added and the mixture stirred for 18 hrs. The mixture was then diluted with DCM (50 mL) and the organics washed with sat. $NaHCO_3$ (30 mL) and hydrochloric acid (1 M, 30 mL), dried with $MgSO_4$, filtered and concentrated *in vacuo*. The residue was purified by flash column chromatography eluting with 30-50 % EtOAc:Hexane. Pure

fractions were concentrated *in vacuo* to give the DP as a yellow solid. A yield of 22 mg, 63 µmol (63 %) was obtained. Spectroscopic data were consistent with those reported above.

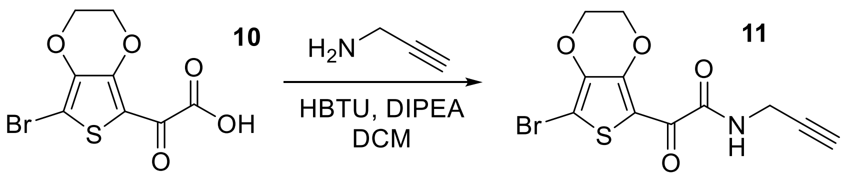

Bromo-carboxy-EDOT **10** (200 mg, 0.68 mmol) and HBTU (519 mg, 1.37 mmol) were suspended in DCM (5 mL). Propargylamine (88 µL, 1.37 mmol) and DIPEA (355 µL, 2.04 mmol) were added and the mixture stirred for 18 hrs. The mixture was then diluted with DCM (50 mL) and the organics washed with water (30 mL), dried with $MgSO_4$, filtered and concentrated *in vacuo*. The residue was purified by flash column chromatography eluting with 40-50 % EtOAc:Hexane. Pure fractions were concentrated *in vacuo* to give the DP as a yellow solid. A yield of 178 mg, 0.57 mmol (84 %) was obtained. ^{1}H NMR (400 MHz, $CDCl_3$): δ = 7.54 (1H, br s, -N<u>H</u>), 4.42-4.48 (2H, m, ArC4-OC<u>H</u>$_2$-), 4.29-4.39 (2H, m, ArC3-OC<u>H</u>$_2$-), 4.12 (2H, dd, *J* = 5.7, 2.2 Hz, -C<u>H</u>$_2$NH-), 2.30 (1H, t, *J* = 2.2 Hz, -C≡C<u>H</u>) ppm; ^{13}C NMR (100 MHz, $CDCl_3$): δ = 174.04 (-<u>C</u>OCONH-), 160.98 (-<u>C</u>ONH-), 149.66 (Ar<u>C</u>4), 140.09 (Ar<u>C</u>3), 110.55 (Ar<u>C</u>2), 107.09 (Ar<u>C</u>5), 78.08 (-<u>C</u>≡CH), 72.40 (-C≡<u>C</u>H), 65.60 (ArC4-O<u>C</u>H$_2$-), 64.35 (ArC3-O<u>C</u>H$_2$-), 29.20 (-<u>C</u>H$_2$NH-) ppm; IR ($υ_{max}$, solid): 3290, 3288, 1686, 1634, 1539, 1475, 1454, 1417, 1358, 1265, 1221, 1087 cm^{-1}; HRMS *m/z* (ESI+): Found: 329.9429/331.9406 (M+H), Calc.: 329.9430/331.9410; m.p. = 183-186 °C (Degrades);

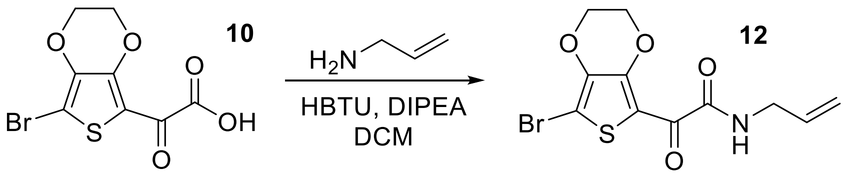

Bromo-carboxy-EDOT **10** (100 mg, 342 µmol) and HBTU (259 mg, 685 µmol) were suspended in DCM (5 mL). Allylamine (51 µL, 685 µmol) and DIPEA (238 µL, 1.37 mmol) were added and the mixture stirred for 18 hrs. The mixture was then diluted with DCM (50 mL) and the organics washed with hydrochloric acid (1 M, 30 mL), dried with $MgSO_4$, filtered and concentrated *in vacuo*. The residue was purified by flash column chromatography eluting with 20-60 % EtOAc:Hexane. Pure fractions were concentrated *in vacuo* to give the DP as a light yellow solid. A yield of 76 mg, 229 µmol (67 %) was obtained. ^{1}H NMR (400 MHz, $CDCl_3$): δ = 7.47 (1H, br s, -N<u>H</u>), 5.88 (1H, ddt, *J* = 17.2, 10.3, 5.6 Hz, -

CH=CH$_2$), 5.27 (1H, ddt, J_1 = 17.2, J_2 = J_3 = 1.5 Hz, -CH=CH$_2$), 5.22 (1H, ddt, J_1 = 10.3 Hz, J_2 = J_3 = 1.5 Hz, -CH=CH$_2$), 4.42-4.51 (2H, m, ArC4-OCH$_2$-), 4.31-4.37 (2H, m, ArC3-OCH$_2$-), 3.99 (2H, tt, J = 5.6, 1.5 Hz, -CH$_2$NH-) ppm; ^{13}C NMR (100 MHz, CDCl$_3$): δ = 174.73 (-COCONH-), 161.19 (-CONH-), 149.54 (ArC4), 140.04 (ArC3), 132.76 (-CH=CH$_2$), 117.32 (-CH=CH$_2$), 110.68 (ArC2), 106.99 (ArC5), 65.60 (ArC4-OCH$_2$-), 64.35 (ArC3-OCH$_2$-), 41.76 (-CH$_2$NH-) ppm; IR (υ_{max}, solid): 3010, 1683, 1626, 1477, 1417, 1386, 1354, 1258, 1246, 1227, 1141, 1085 cm^{-1}; HRMS *m/z* (ESI+): Found: 331.9601/333.9595 (M+H), Calc.: 331.9592/333.9572; m.p. = 140-143 °C;

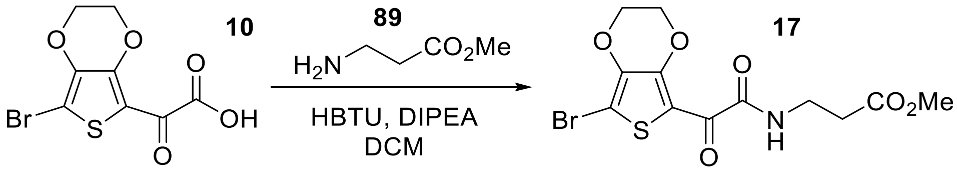

Bromo-carboxy-EDOT **10** (300 mg, 1.02 mmol) and HBTU (777 mg, 2.05 mmol) were dissolved in DCM (10 mL). Amine **89** (287 mg, 2.05 mmol) and DIPEA (889 µL, 5.10 mmol) were added and the mixture stirred for 18 hrs. The mixture was then diluted with DCM (50 mL) and the organics washed with water (30 mL), dried with MgSO$_4$, filtered and concentrated *in vacuo*. The residue was purified by flash column chromatography eluting with 40-50 % EtOAc:Hexane. Pure fractions were concentrated *in vacuo* to give the DP as a yellow solid. A yield of 194 mg, 0.51 mmol (50 %) was obtained. Spectroscopic data were consistent with those reported above.

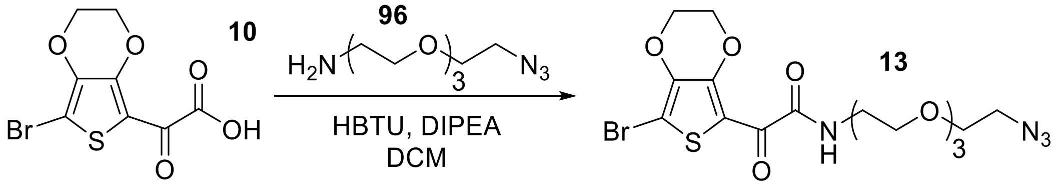

Bromo-carboxy-EDOT **10** (244 mg, 0.83 mmol) and HBTU (629 mg, 1.66 mmol) were dissolved in DCM (15 mL). Amine **96** (218 mg, 1 mmol) and DIPEA (578 µL, 3.32 mmol) were added and the mixture stirred for 5 hrs. The mixture was then diluted with DCM (50 mL) and the organics washed with water (30 mL), dried with MgSO$_4$, filtered and concentrated *in vacuo*. The residue was purified by flash column chromatography eluting with 60-90 % EtOAc:Hexane. Pure fractions were concentrated *in vacuo* to give the DP as a yellow oil. A yield of 204 mg, 0.42 mmol (50 %) was obtained. ^{1}H NMR (400 MHz, MeOD): δ = 4.41-4.45 (2H, m, ArC4-OCH$_2$-), 4.34-4.38 (2H, m, ArC3-OCH$_2$-), 3.62-3.69 (12H, m, TEG), 3.50 (2H, t, J = 5.4 Hz, -CH$_2$NH-), 3.40 (2H, t, J = 5.5 Hz, -CH$_2$N$_3$) ppm; ^{13}C NMR (100 MHz, MeOD): δ

=175.30 (-COCONHR), 162.88 (-CONHR), 149.55 (ArC4), 140.40 (ArC3), 110.63 (ArC5), 104.69 (ArC2), 70.14 (TEG), 70.09 (TEG), 70.02 (TEG), 69.88 (TEG), 69.62 (TEG), 68.84 (TEG), 65.49 (ArC4-OCH_2-), 64.35 (TEG), 50.37 (-CH_2N_3), 38.88 (-CH_2NH-) ppm; IR (υ_{max}, oil): 2926, 2875, 2102, 1682, 1638, 1478, 1443, 1419, 1357, 1238, 1107, 1080, 1067 cm^{-1}; HRMS *m/z* (ESI+): Found: 514.9980/516.9934 (M+Na), Calc.: 515.0206/517.0186;

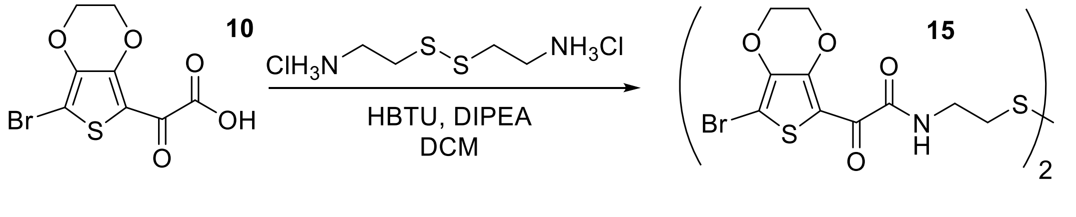

Bromo-carboxy-EDOT **10** (293 mg, 1 mmol) and HBTU (758 mg, 2 mmol) were dissolved in DCM (20 mL). Cystamine dihydrochloride (113g, 0.5 mmol) and DIPEA (872 µL, 5 mmol) were added and the mixture stirred for 3 hrs. The mixture was then diluted with DCM (10 mL) and the organics washed with water (30 mL), dried with $MgSO_4$, filtered and concentrated onto silica. The residue was purified by flash column chromatography eluting with 50-100 % EtOAc:Hexane. Pure fractions were concentrated *in vacuo* to give the DP as a yellow solid. A yield of 253 mg, 0.36 mmol (72 %) was obtained. ^{1}H NMR (400 MHz, DMSO-d_6): δ = 9.03 (2H, t, *J* = 5.9 Hz, -NH), 4.39-4.38 (4H, m, ArC4-OCH_2-), 4.29-4.39 (4H, m, ArC4-OCH_2-), 3.48 (4H, td, *J* = 6.8, 5.9 Hz, -CH_2NH-), 2.89 (4H, t, *J* = 6.8 Hz, -CH_2S-) ppm; ^{13}C NMR (100 MHz, DMSO-d_6): δ = 175.44 (-COCONR$_2$), 162.44 (-CONR$_2$), 149.74 (ArC4), 140.67 (ArC3), 110.45 (ArC2), 104.07 (ArC5), 65.77 (ArC4-OCH_2-), 64.70 (ArC3-OCH_2-), 38.62 (-CH_2NH-), 36.89 (-CH_2S-) ppm; IR (υ_{max}, solid): 2994, 1682, 1637,1626, 1528, 1477, 1418, 1350, 1279, 1244, 1168, 1142, 1087 cm^{-1}; HRMS *m/z* (ESI+): Found: 702.8389 (M+H), Calc.: 702.8371; m.p. = 232-235 °C;

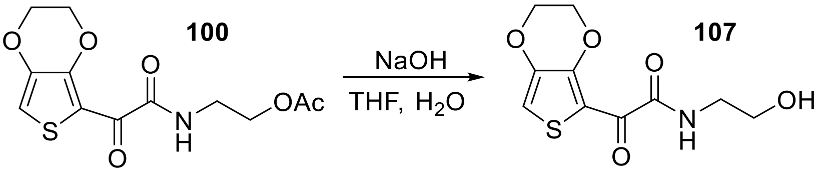

Sodium hydroxide (18 mL, 36 mmol) was added to a solution of acetoxy-EDOT **100** (2.7 g, 9 mmol) in THF (50 mL) and the mixture stirred for 2 hr. The THF was then removed *in vacuo* and the mixture diluted with DCM (100 mL). The organics were washed with hydrochlodic acid (1 M, 50 mL), dried with $MgSO_4$, filtered and concentrated *in vacuo*. The residue was purified by flash column chromatography eluting with 70-100 % EtOAc:Hexane. Pure fractions were concentrated *in vacuo* to give the DP as a

light yellow solid. A yield of 758 mg, 2.9 mmol (33 %) was obtained. ^{1}H NMR (400 MHz, DMSO-d$_6$): δ = 8.67 (1H, br t, *J* = 5.9 Hz, -N<u>H</u>), 7.25 (1H, s, Ar<u>H</u>5), 4.75 (1H, t, *J* = 5.4 Hz -O<u>H</u>), 4.34-4.41 (2H, m, ArC4-OC<u>H</u>$_2$-), 4.22-4.31 (2H, m, ArC3-OC<u>H</u>$_2$-), 3.48 (2H, dt, *J* = 5.9, 5.7 Hz, -C<u>H</u>$_2$OH), 3.24 (2H, td, *J* = 5.7, 5.4 Hz, -NHC<u>H</u>$_2$-) ppm; ^{13}C NMR (100 MHz, DMSO-d$_6$): δ = 177.75 (-<u>C</u>OCONH-), 163.21 (-<u>C</u>ONH-), 149.86 (Ar<u>C</u>4), 141.85 (Ar<u>C</u>3), 114.46 (Ar<u>C</u>5), 111.27 (Ar<u>C</u>2), 65.69 (ArC4-O<u>C</u>H$_2$-), 64.18 (ArC3-O<u>C</u>H$_2$-), 59.69 (-<u>C</u>H$_2$OH), 41.95 (-NH<u>C</u>H$_2$-) ppm; IR (υ_{max}, solid): 3491, 3330, 1672, 1631, 1618, 1533, 1472, 1446, 1418, 1370, 1359, 1176, 1075, 1065, 1052, 1033 cm^{-1}; HRMS *m/z* (ESI+): Found: 258.0435 (M+H), Calc.: 258.0436; m.p. = 152-157 °C;

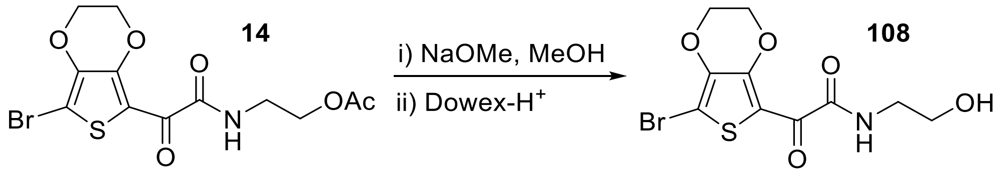

Sodium methoxide (1.14 g, 21.1 mmol) was added to a solution of Bromo-acetoxy-EDOT **14** (2 g, 5.3 mmol) in methanol (50 mL) and the mixture stirred for 1 hr, during which time a yellow precipitate formed. The reaction was neutralised with pre-activated Dowex-50WX8 and stirred for 10 min. The mixture was then filtered and the residue washed extensively with DCM (~ 150 mL). The filtrate was then concentrated *in vacuo* to give the DP as a yellow solid. A yield of 1.1 g, 3.3 mmol (62 %) was obtained. ^{1}H NMR (400 MHz, DMSO-d$_6$): δ = 8.76 (1H, br t, *J* = 6.0 Hz, -N<u>H</u>), 4.75 (1H, br s, -O<u>H</u>), 4.39-4.46 (2H, m, ArC4-OC<u>H</u>$_2$-), 4.32-4.39 (2H, m, ArC3-OC<u>H</u>$_2$-), 3.47 (2H, t, *J* = 6.1 Hz, -C<u>H</u>$_2$OH), 3.23 (2H, td, *J* = 6.1, 6.0 Hz, -NHC<u>H</u>$_2$-) ppm; ^{13}C NMR (100 MHz, DMSO-d$_6$): δ = 175.73 (-<u>C</u>OCONH-), 162.49 (-<u>C</u>ONH-), 149.70 (Ar<u>C</u>4), 140.71 (Ar<u>C</u>3), 110.45 (Ar<u>C</u>2), 103.93 (Ar<u>C</u>5), 65.77 (ArC4-O<u>C</u>H$_2$-), 64.72 (ArC3-O<u>C</u>H$_2$-), 59.58 (-<u>C</u>H$_2$OH), 42.10 (-NH<u>C</u>H$_2$-) ppm; IR (υ_{max}, solid): 2931, 2860, 1607, 1467, 1422, 1358, 1307, 1246, 1216, 1066 cm^{-1}; HRMS *m/z* (ESI+): Found: 335.9552/337.9520 (M+H), Calc.: 335.9541/337.9521; m.p. = 161-167 °C;

Chain extension protocol

Brominated monomer (1 mmol), pivalic acid (0.5 mmol), palladium (II) acetate (0.05 mmol) and potassium carbonate (10 mmol) were charged under nitrogen. Dry DMF (2 mL) and EDOT (4 mmol) were then added and the mixture heated to 90 ºC for 2 hrs. After cooling to rt the mixture was diluted with DCM (50 mL) and washed with water (2 x 50 mL) and brine (50 mL). The organics were dried with

$MgSO_4$, filtered and concentrated *in vacuo*. The residue was purified by flash column chromatography and pure fractions were concentrated *in vacuo*.

Numbering system for monofunctional-oligomer NMR assignments

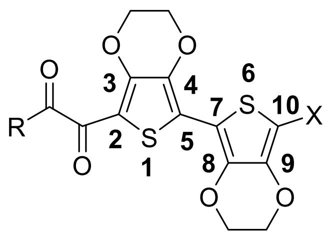

Chain extension- Dimers

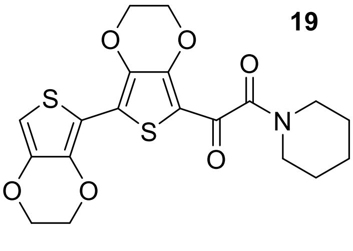

19 Run on 2.78 mmol scale at 130 ºC. Column eluted with 2 % NEt_3 in 50-90 % EtOAc:Hexane. A yield of 470 mg, 1.11 mmol (40 %) was obtained as a yellow solid. ^{1}H NMR (400 MHz, $CDCl_3$): δ = 6.48 (1H, s, ArH10), 4.36-4.45 (6H, m, -OCH2-), 4.24-4.31 (2H, m, -OCH2-), 3.65 (2H, t, J = 5.2 Hz, -NCH2-), 3.39 (2H, t, J = 5.2 Hz, -NCH2-), 1.53-1.76 (6H, m, -CH2CH2CH2N-) ppm; ^{13}C NMR (100 MHz, $CDCl_3$): δ = 181.76 (-COCON-), 165.52 (-CON-), 146.55 (ArCβ), 141.37 (ArCβ), 140.30 (ArC9), 136.11 (ArCβ), 122.92 (ArCα), 112.24 (ArCα), 108.99 (ArC7), 101.87 (ArC10), 65.50 (-OCH2-), 65.28 (-OCH2-), 64.53 (-OCH2-), 64.48 (-OCH2-), 47.04 (-CH2N-), 42.19 (-CH2N-), 26.06 (-CH2CH2N-), 25.27 (-CH2CH2N-), 24.55 (-CH2CH2CH2N-) ppm; IR ($υ_{max}$, solid): 3305, 2931, 2867, 1659, 1637, 1626, 1603, 1556, 1467, 1452, 1440, 1427, 1358, 1265, 1250, 1224, 1086, 1038 cm^{-1}; HRMS *m/z* (ESI+): Found: 422.0730 (M+H), Calc.: 422.0732; m.p. = 276-277 °C;

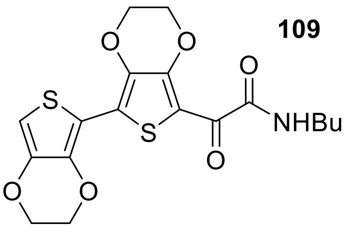

109 Run on 0.6 mmol scale. Column eluted with 2 % NEt_3 in 40-70 % EtOAc:Hexane. A yield of 158 mg, 0.39 mmol (67 %) was obtained as a yellow solid. ^{1}H NMR (400 MHz, $CDCl_3$): δ = 7.47 (1H, br t, J = 6.9 Hz, -NH), 6.49 (1H, s, ArH10), 4.47-4.56 (2H, m, -OCH2-), 4.35-4.47 (4H, m, -OCH2-), 4.20-4.33 (2H, m, -OCH2-), 3.39 (2H, dt, $J_1 = J_2$ = 6.9 Hz, -NHCH2-), 1.52-1.66 (2H, m, -NHCH2CH2-), 1.32-1.47 (2H, m, -CH2CH3), 0.95 (3H, t, J = 7.3 Hz, -CH3) ppm; ^{13}C NMR (100 MHz, $CDCl_3$): δ = 175.33 (-COCONH-), 162.03 (-CONH-), 149.94 (ArCβ), 141.40 (ArCβ), 140.74 (ArCβ), 136.21 (ArCβ), 126.41 (ArCα), 109.35 (ArCα), 108.14 (ArCα), 102.26 (ArC10), 65.59 (-OCH2-), 65.32 (-OCH2-), 64.53 (-OCH2-), 64.33 (-OCH2-), 39.15 (-NHCH2-), 31.35 (-NHCH2CH2-), 20.07 (-CH2CH3),

13.72 (-C̲H3) ppm; IR (υmax, solid): 3280, 2959, 2932, 2875, 2850, 1674, 1622, 1490, 1469, 1455, 1441, 1424, 1364, 1300, 1262, 1236, 1170, 1085, 1058, 1045 cm^{-1}; HRMS *m/z* (ESI+): Found: 410.0732 (M+H), Calc.: 410.0732; m.p. = 186-187 °C;

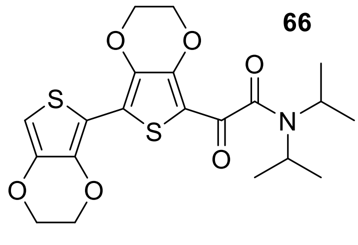
66

Run on 2.6 mmol scale. Column eluted with 2 % NEt_3 in 60 % EtOAc:Hexane. A yield of 480 mg, 1.09 mmol (41 %) was obtained as a yellow solid. ^{1}H NMR (400 MHz, $CDCl_3$): δ = 6.46 (1H, s, ArH̲10), 4.32-4.42 (6H, m, -OCH̲2-), 4.23-4.30 (2H, m, -OCH̲2-), 3.85 (1H, sept, *J* = 6.6 Hz, -CH̲Me2), 3.53 (1H, sept, *J* = 6.8 Hz, -CH̲Me2), 1.52 (6H, d, *J* = 6.8 Hz, -CHM̲e̲2), 1.19 (6H, d, *J* = 6.6 Hz, -CHM̲e̲2) ppm; ^{13}C NMR (100 MHz, $CDCl_3$): δ = 181.69 (-C̲OCON-), 166.68 (-C̲ON-), 146.19 (ArC̲β), 141.35 (ArC̲β), 140.15 (ArC̲9), 135.98 (ArC̲β), 122.43 (ArC̲α), 112.40 (ArC̲α), 109.04 (ArC̲7), 101.64 (ArC̲10), 65.25 (-OC̲H2-), 65.09 (-OC̲H2-), 64.53 (-OC̲H2-), 64.47 (-OC̲H2-), 50.24 (-C̲HMe2), 45.70 (-C̲HMe2), 20.57 (-CHM̲e̲2), 20.06 (-CHM̲e̲2) ppm; IR (υmax, solid): 2926, 2853, 1691, 1647, 1607, 1544, 1467, 1433, 1359, 1202, 1171, 1130 cm^{-1}; HRMS *m/z* (ESI+): Found: 438.1053 (M+H), Calc.: 438.1045; m.p. = 265-267 °C;

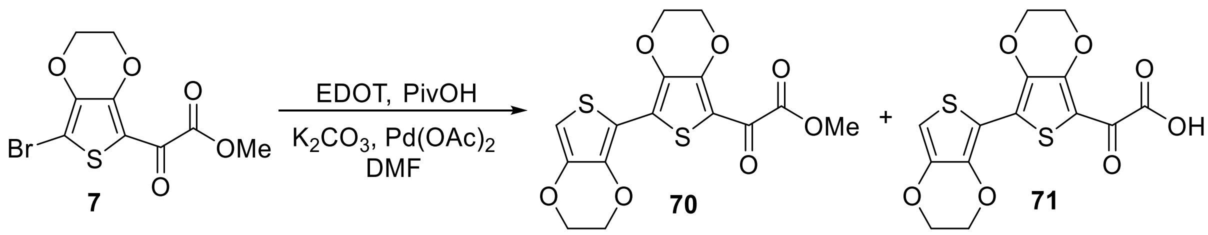

Run on 0.3 mmol scale. Analysis by LC-MS indicated that while complete chain extension of **7** had occurred, a significant degree of ester cleavage had occurred to generate a mixture of **70** and **71** (~1.5:1 ratio). Subsequent experiments were therefore undertaken with alternative esters which did not undergo cleavage under the reaction conditions.

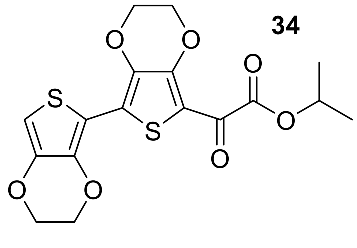
34

Run on 0.30 mmol scale. Column eluted with 40-60 % EtOAc:Hexane. A yield of 80 mg, 0.20 mmol (66 %) was obtained as a yellow oil. If the reaction was undertaken at 130 °C, ester cleavage was observed as described above for methyl ester **3**. ^{1}H NMR (400 MHz, DMSO-d_6): δ = 6.90 (1H, s, ArH̲10), 5.15 (1H, sept, *J* = 6.5 Hz, -CH̲Me2), 4.37-4.60 (6H, m, -OCH̲2-), 4.20-4.37 (2H, m, -OCH̲2-), 1.31 (6H, d, *J* = 6.5 Hz, -CHM̲e̲2) ppm; ^{13}C NMR (400 MHz, DMSO-d_6): δ = 176.57 (-C̲OCO2*i*Pr), 163.99 (-C̲O2*i*Pr), 148.67 (ArC̲β), 141.66 (ArC̲9), 141.19 (ArC̲β), 136.53 (ArC̲β), 122.87

(ArC$_\alpha$), 109.63 (ArC$_\alpha$), 108.08 (ArC7), 103.59 (ArC10), 70.58 (-CHMe$_2$), 66.07 (-OCH$_2$-), 65.14 (-OCH$_2$-), 64.71 (-OCH$_2$-), 21.74 (-CHMe$_2$) ppm; IR (υ_{max}, solid): 3345, 2980, 2939, 2880, 1731, 1704, 1646, 1637, 1489, 1468, 1438, 1357, 1312, 1263, 1244, 1216, 1175, 1104, 1057, 1028, 1010 cm^{-1}; HRMS *m/z* (ESI+): Found: 397.0435 (M+H), Calc.: 397.0416; m.p. = 168-172 °C;

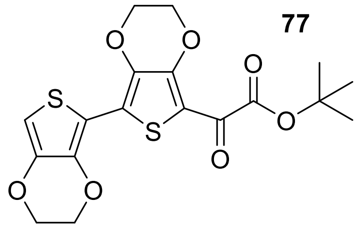

77

Run on 0.57 mmol scale. Column eluted with 2 % NEt_3 in 50-70 % EtOAc:Hexane. A yield of 190 mg, 0.46 mmol (81 %) was obtained as a yellow solid. ^{1}H NMR (400 MHz, DMSO-d$_6$): δ = 6.90 (1H, s, ArH10), 4.40-4.50 (6H, m, -OCH$_2$-), 4.25-4.32 (2H, m, -OCH$_2$-), 1.54 (9H, s, -O*t*Bu) ppm; ^{13}C NMR (100 MHz, $CDCl_3$): δ = 176.87 (-COCO$_2$*t*Bu), 163.75 (-CO$_2$*t*Bu), 148.40 (ArC$_\beta$), 141.66 (ArC$_\beta$), 141.09 (ArC9), 136.50 (ArC$_\beta$), 122.52 (ArC$_\alpha$), 109.69 (ArC$_\alpha$), 108.10 (ArC7), 103.44 (ArC10), 84.39 (-CMe$_3$), 66.05 (-OCH$_2$-), 65.97 (-OCH$_2$-), 65.15 (-OCH$_2$-), 64.71 (-OCH$_2$-), 27.97 (-CMe$_3$) ppm; IR (υ_{max}, solid): 2935, 1714, 1611, 1506, 1466, 1441, 1361, 1270, 1247, 1228, 1159, 1140, 1118, 1095, 1062 cm^{-1}; HRMS *m/z* (ESI+): Found: 411.0577 (M+H), Calc.: 411.0572; m.p. = 188-191 °C;

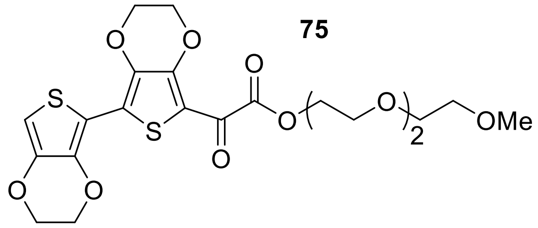

75

Run on 2.28 mmol scale. Column eluted with 2 % NEt_3 in 60-100 % EtOAc:Hexane. A yield of 270 mg, 0.59 mmol (26 %) was obtained as a yellow oil which solidified on standing. ^{1}H NMR (400 MHz, $CDCl_3$): δ = 6.48 (1H, s, ArH10), 4.43-4.48 (2H, m, TEG), 4.34-4.43 (6H, m, -OCH$_2$-), 4.19-4.31 (2H, m, -OCH$_2$-), 3.75-3.85 (2H, m, TEG), 3.66-3.71 (2H, m, TEG), 3.60-3.66 (4H, m, TEG), 3.46-3.58 (2H, m, TEG), 3.35 (3H, s, -OMe) ppm; ^{13}C NMR (400 MHz, $CDCl_3$): δ = 174.73 (-COCO$_2$R), 163.67 (-CO$_2$R), 148.30 (ArC$_\beta$), 141.36 (ArC$_\beta$), 140.58 (ArC$_\beta$), 136.13 (ArC$_\beta$), 124.17 (ArC$_\alpha$), 110.42 (ArC$_\alpha$), 108.97 (ArC$_\alpha$), 102.34 (ArC10), 71.58 (TEG), 70.74 (TEG), 70.59 (TEG), 70.55 (TEG), 68.60 (TEG), 65.59 (-OCH$_2$-), 65.35 (-OCH$_2$-), 65.20 (-OCH$_2$-), 64.49 (-OCH$_2$-/TEG), 64.48 (-OCH$_2$-/TEG), 58.98 (-OMe) ppm; IR (υ_{max}, oil): 2920, 2876, 1724, 1623, 1612, 1472, 1441, 1359, 1262, 1245, 1215, 1118, 1089, 1062, 1037, 1013 cm^{-1}; HRMS *m/z* (ESI+): Found: 501.0897 (M+H), Calc.: 501.0889; m.p. = 80-81 °C;

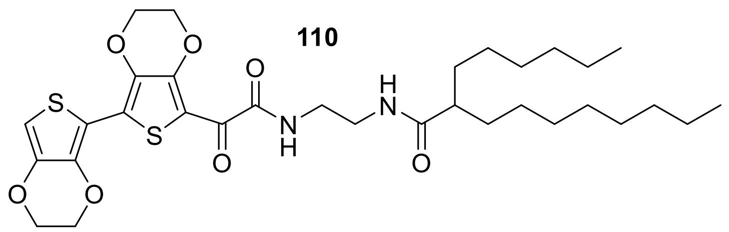

110

Run on 2.02 mmol scale. Column eluted with 2 % NEt_3 in 60-90 % EtOAc:Hexane. A yield of 290 mg, 0.46 mmol (23 %) was obtained as a yellow solid. ^{1}H NMR (400 MHz, $CDCl_3$): δ = 7.78 (1H, br t, *J* = 6.0 Hz, -NHCOCO-), 6.52 (1H, s, ArH10), 5.97 (1H, br t, *J* = 5.2 Hz, -NHCOAlk), 4.48-4.52 (2H, m, $-OCH_2-$), 4.38-4.46 (2H, m, $-OCH_2-$), 4.26-4.31 (2H, m, $-OCH_2-$), 3.49-3.59 (4H, m, $-NHCH_2CH_2NH-$), 1.95-2.05 (1H, m, H_{α}), 1.52-1.64 (2H, m, H_{β}), 1.34-1.46 (2H, m, H_{β}), 1.16-1.31 (20H, m, Alkyl), 0.82-0.90 (6H, m, $-CH_3$) ppm; ^{13}C NMR (100 MHz, $CDCl_3$): δ = 176.79 (-NHCOAlkyl) 174.56 (-COCONH-), 163.09 (-COCONH-), 150.13 (ArC_{β}), 141.40 (ArC_{β}), 140.78 (ArC_{β}), 136.27 (ArC_{β}), 109.33 (ArC_{α}), 107.97 (ArC_{α}), 102.49 (ArC10), 65.58 ($-OCH_2-$), 65.35 ($-OCH_2-$), 64.52 ($-OCH_2-$), 64.33 ($-OCH_2-$), 48.12 (C_{α}), 39.35 ($-NHCH_2-$), 39.32 ($-NHCH_2-$), 33.02 (C_{β}), 31.86 (Alkyl), 31.68 (Alkyl), 29.71 (Alkyl), 29.46 (Alkyl), 29.34 (Alkyl), 27.71 (Alkyl), 27.65 (Alkyl), 22.65 (Alkyl), 14.12 ($-CH_3$), 14.07 ($-CH_3$) ppm; IR (υ_{max}, solid): 3324, 3287, 2923, 2870, 2853, 1671, 1641, 1623, 1471, 1440, 1428, 1369, 1251, 1207, 1181, 1095, 1064 cm^{-1}; HRMS *m/z* (ESI+): Found: 635.2820 (M+H), Calc.: 635.2825; m.p. = 228-230 °C;

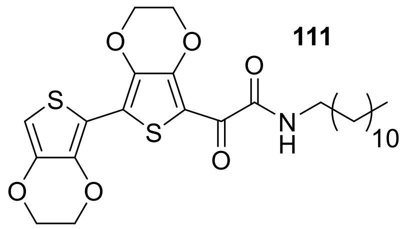

111

Run on 2.17 mmol scale. A significant amount of product was lost during work-up due to its high insolubility. The product was loaded onto silica prior to column chromatography, eluting with 2 % NEt_3 in 20-50 % EtOAc:Hexane. Yield of 210 mg, 0.40 mmol (18 %) as a yellow solid. ^{1}H NMR (400 MHz, $CDCl_3$): δ = 7.47 (1H, br t, *J* = 6.8 Hz, -NH), 6.47 (1H, s, ArC10), 4.43-4.52 (2H, m, $-OCH_2-$), 4.35-4.44 (4H, m, $-OCH_2-$), 4.20-4.30 (2H, m, ArC3-OCH_2-), 3.35 (2H, dt, $J_1 = J_2$ = 6.8 Hz, $-NHCH_2-$), 1.52-1.62 (2H, m, $-NHCH_2CH_2-$), 1.23-1.40 (18H, m, Alkyl), 0.87 (3H, t, *J* = 6.7 Hz, $-CH_3$) ppm; ^{13}C NMR (100 MHz, $CDCl_3$): δ = 175.31 (-COCONH-), 161.99 (-CONH-), 149.92 (ArC_{β}), 141.38 (ArC_{β}), 140.72 (ArC_{β}), 136.19 (ArC_{β}), 126.38 (ArC_{α}), 109.33 (ArC_{α}), 108.12 (ArC_{α}), 102.24 (ArC10), 65.58 ($-OCH_2-$), 65.31 ($-OCH_2-$), 64.52 ($-OCH_2-$), 64.33 ($-OCH_2-$), 39.44 ($-NHCH_2-$), 31.90 ($-NHCH_2CH_2-$), 29.62 (Alkyl), 29.55 (Alkyl), 29.51 (Alkyl), 29.34 (Alkyl), 29.31 (Alkyl), 26.90 (Alkyl), 22.68 (Alkyl), 14.13 ($-CH_3$) ppm; IR (υ_{max}, solid): 2919, 2850, 1677, 1624, 1493, 1469, 1432, 1364, 1301, 1171, 1090, 1058 cm^{-1}; HRMS *m/z* (ESI+): Found: 522.1988 (M+H), Calc.: 522.1984; m.p. = 180-183 °C;

Dimer bromination

Bromination procedure A: Di-EDOT derivative (1 mmol) was dissolved in a mixture of THF (10 mL) and acetic acid (2 mL). If solubility was poor a further 25 mL of THF was added. The mixture was placed in the dark and *N*-bromosuccinimide (1.2 mmol) was added. After stirring for 2 hrs the mixture was poured into water (50 mL) causing precipitation of the product. The solid was collected by filtration or centrifugation (5000 rpm, 10 min), washed with water (50 mL) and then dissolved in DCM. The organics were dried with $MgSO_4$, filtered and concentrated *in vacuo*. Column chromatography was then undertaken if required.

Bromination procedure B: Reaction procedure A was followed. After pouring into water the product was extracted with DCM (200 mL). The organics were washed with sat. $NaHCO_3$ (2 x 100 mL), dried with $MgSO_4$, filtered and concentrated *in vacuo*. Column chromatography was then undertaken if required.

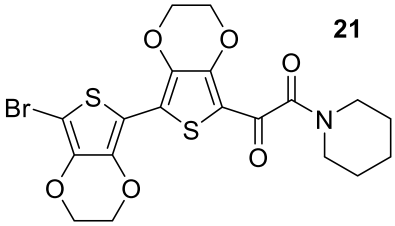

21

Method B on 0.48 mmol scale. The product was sufficiently pure for further applications. An analytical sample was obtained via column chromatography eluting with 2 % NEt_3 in 50-90 % EtOAc:Hexane. Yield of 220 mg, 0.44 mmol (92 %) as a dark green-yellow solid. ^{1}H NMR (400 MHz, $CDCl_3$): δ = 4.31-4.45 (8H, m, -OC$\underline{H}_2$-), 3.65 (2H, t, *J* = 5.2 Hz, -C$\underline{H}_2$N-), 3.39 (2H, t, *J* = 5.3 Hz, -C$\underline{H}_2$N-), 1.62-1.74 (4H, m, -C$\underline{H}_2$C$\underline{H}_2$CH$_2$N-), 1.53-1.61 (2H, m, -C$\underline{H}_2$CH$_2$N-) ppm; ^{13}C NMR (100 MHz, $CDCl_3$): δ = 181.73 (-$\underline{C}$OCON-), 165.40 (-$\underline{C}$ON-), 146.41 (Ar$\underline{C}_\beta$), 139.76 (Ar$\underline{C}_\beta$), 139.25 (Ar$\underline{C}_\beta$), 136.23 (Ar$\underline{C}_\beta$), 121.91 (Ar$\underline{C}_\alpha$), 112.48 (Ar$\underline{C}_\alpha$), 109.25 (Ar$\underline{C}_\alpha$), 90.81 (Ar$\underline{C}$10), 65.51 (-O$\underline{C}$H$_2$-), 65.22 (-O$\underline{C}$H$_2$-), 64.98 (-O$\underline{C}$H$_2$-), 64.57 (-O$\underline{C}$H$_2$-), 47.04 (-$\underline{C}$H$_2$N-), 42.22 (-$\underline{C}$H$_2$N-), 26.08 (-$\underline{C}$H$_2\underline{C}$H$_2$CH$_2$N-), 25.27 (-$\underline{C}$H$_2\underline{C}$H$_2$CH$_2$N-), 24.54 (-$\underline{C}$H$_2\underline{C}$H$_2$CH$_2$N-) ppm; IR (υ_{max}, solid): 2978, 2929, 2858, 1645, 1601, 1509, 1472, 1445, 1358, 1315, 1257, 1250, 1222, 1122, 1075 cm^{-1}; HRMS *m/z* (ESI+): Found: 499.9841/501.9814 (M+H), Calc.: 499.9837/501.9816; m.p. = 238-240 °C;

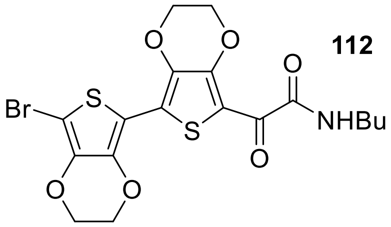

112

Method A on 2.44 mmol scale. A yield of 854 mg, 1.75 mmol (72 %) was obtained as a dark yellow solid. ^{1}H NMR (400 MHz, $CDCl_3$): δ = 7.45 (1H, br t, *J* = 6.3 Hz, -N$\underline{H}$), 4.48-4.53 (2H, m, -OC$\underline{H}_2$-), 4.38-4.46 (4H, m, -OC$\underline{H}_2$-), 4.33-4.38 (2H, m, -OC$\underline{H}_2$-), 3.38 (2H, td, *J* = 6.3, 6.1 Hz, -C$\underline{H}_2$NH-), 1.54-1.66 (2H, m, -C$\underline{H}_2$CH$_2$NH-), 1.33-1.47 (2H, m, -C$\underline{H}_2$CH$_3$), 0.96 (3H, t, *J* =

7.3 Hz, -C<u>H</u>$_3$) ppm; ^{13}C NMR (100 MHz, $CDCl_3$): δ = 175.37 (-<u>C</u>OCONH-), 161.96 (-<u>C</u>ON-), 149.81 (Ar<u>C</u>$_β$), 139.80 (Ar<u>C</u>$_β$), 139.68 (Ar<u>C</u>$_β$), 136.31 (Ar<u>C</u>$_β$), 125.39 (Ar<u>C</u>$_α$), 109.61 (Ar<u>C</u>$_α$), 108.31 (Ar<u>C</u>$_α$), 91.21 (Ar<u>C</u>10), 65.59 (-O<u>C</u>H$_2$-), 65.25 (-O<u>C</u>H$_2$-), 64.99 (-O<u>C</u>H$_2$-), 64.42 (-O<u>C</u>H$_2$-), 39.17 (-<u>C</u>H$_2$NH-), 31.34 (-<u>C</u>H$_2$CH$_2$NH-), 20.07 (-<u>C</u>H$_2$CH$_3$), 13.71 (-<u>C</u>H$_3$) ppm; IR ($υ_{max}$, solid): 3383, 2951, 2927, 2867, 1674, 1625, 1494, 1467, 1440, 1426, 1355, 1296, 1072, 1057, 1029 cm^{-1}; HRMS *m/z* (ESI+): Found: 487.9843/489.9798 (M+H), Calc.: 487.9837/489.9817; m.p. = 259-267 °C (Degrades);

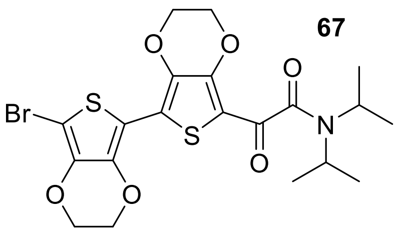

67

Method B on 0.56 mmol scale. The product was sufficiently pure for further applications. An analytical sample was obtained via column chromatography eluting with 2 % NEt_3 in 30-60 % EtOAc:Hexane. A yield of 252 mg, 0.49 mmol (87 %) was obtained as a yellow solid. ^{1}H NMR (400 MHz, $CDCl_3$): δ = 4.29-4.45 (8H, m, -OC<u>H</u>$_2$-), 3.85 (1H, sept, *J* = 6.5 Hz, -C<u>H</u>Me$_2$), 3.54 (1H, sept, *J* = 6.8 Hz, -C<u>H</u>Me$_2$), 1.52 (6H, d, *J* = 6.8 Hz, -CH<u>Me</u>$_2$), 1.20 (6H, d, *J* = 6.8 Hz, -CH<u>Me</u>$_2$) ppm; ^{13}C NMR (100 MHz, $CDCl_3$): δ = 181.67 (-<u>C</u>OCON-), 166.57 (-<u>C</u>ON-), 146.03 (Ar<u>C</u>$_β$), 139.74 (Ar<u>C</u>$_β$), 139.10 (Ar<u>C</u>$_β$), 136.11 (Ar<u>C</u>$_β$), 121.42 (Ar<u>C</u>$_α$), 112.67 (Ar<u>C</u>$_α$), 109.32 (Ar<u>C</u>$_α$), 90.52 (Ar<u>C</u>10), 65.20 (-O<u>C</u>H$_2$-), 65.10 (-O<u>C</u>H$_2$-), 65.00 (-O<u>C</u>H$_2$-), 64.57 (-O<u>C</u>H$_2$-), 50.26 (-<u>C</u>HMe$_2$), 45.74 (-<u>C</u>HMe$_2$), 20.61 (-CH<u>Me</u>$_2$), 20.07 (-CH<u>Me</u>$_2$) ppm; IR ($υ_{max}$, solid): 2971, 2933, 2872, 1711, 1637, 1606, 1470, 1438, 1362, 1289, 1270, 1238, 1209, 1179, 1162, 1115, 1079, 1045 cm^{-1}; HRMS *m/z* (ESI+): Found: 516.0.170/518.0129 (M+H), Calc.: 516.0150/518.0130; m.p. = 281-282 °C;

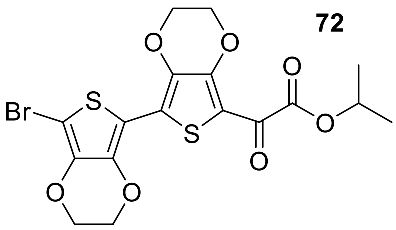

72

Method B on 0.83 mmol scale. The product was sufficiently pure for further applications. An analytical sample was obtained via column chromatography eluting with 2 % NEt_3 in 20-50 % EtOAc:Hexane. A yield of 320 mg, 0.67 mmol (81 %) was obtained as a green-yellow solid. ^{1}H NMR (400 MHz, $CDCl_3$): δ = 5.26 (1H, sept, *J* = 6.3 Hz, -C<u>H</u>Me$_2$), 4.38-4.45 (6H, m, -OC<u>H</u>$_2$-), 4.33-4.37 (2H, m, -OC<u>H</u>$_2$-), 1.40 (6H, d, *J* = 6.3 Hz, -CH<u>Me</u>$_2$) ppm; ^{13}C NMR (100 MHz, $CDCl_3$): δ = 175.60 (-<u>C</u>OCO$_2$*i*Pr), 163.31 (-<u>C</u>O$_2$*i*Pr), 147.76 (Ar<u>C</u>$_β$), 139.73 (Ar<u>C</u>$_β$), 139.39 (Ar<u>C</u>$_β$), 136.13 (Ar<u>C</u>$_β$), 122.83 (Ar<u>C</u>$_α$), 110.84 (Ar<u>C</u>$_α$), 109.23 (Ar<u>C</u>$_α$), 91.12 (Ar<u>C</u>10), 70.40 (-<u>C</u>HMe$_2$), 65.32 (-O<u>C</u>H$_2$-), 65.22 (-O<u>C</u>H$_2$-), 64.92 (-O<u>C</u>H$_2$-), 64.48 (-O<u>C</u>H$_2$-), 21.58 (-CH<u>Me</u>$_2$) ppm; IR ($υ_{max}$, solid): 2981, 2933, 2869, 1737, 1714, 1649, 1621, 1484, 1469, 1457, 1429, 1357, 1220, 1099, 1075 cm^{-1}; HRMS *m/z* (ESI+): Found: 474.9525/476.9513 (M+H), Calc.: 474.9521/476/9500; m.p. = 178-186 °C;

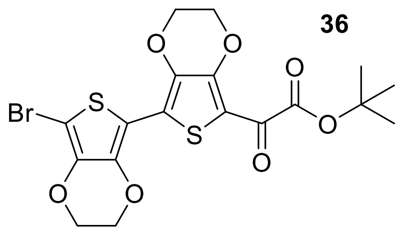

36

Method A on 1.22 mmol scale. DMF was used as solvent in place of THF/AcOH to prevent hydrolysis of the *t*-butyl group. The product was sufficiently pure for further applications. An analytical sample was obtained via column chromatography eluting with 2 % NEt_3 in 20-50 % EtOAc:Hexane. A yield of 544 mg, 1.11 mmol (92 %) was obtained as a yellow solid. ^{1}H NMR (400 MHz, $CDCl_3$): δ = 4.37-4.43 (6H, m, -OC$\underline{H}_2$-), 4.32-4.36 (2H, m, -OC$\underline{H}_2$-), 1.61 (9H, s, -$CO_2$$\underline{tBu}$) ppm; ^{13}C NMR (100 MHz, $CDCl_3$): δ = 176.36 (-$\underline{C}$OCO_2*t*Bu), 163.12 (-$\underline{C}O_2$*t*Bu), 147.47 (Ar$\underline{C}_\beta$), 139.75 (Ar$\underline{C}_\beta$), 139.31 (Ar$\underline{C}_\beta$), 136.12 (Ar$\underline{C}_\beta$), 122.41 (Ar$\underline{C}_\alpha$), 110.98 (Ar$\underline{C}_\alpha$), 109.30 (Ar$\underline{C}_\alpha$), 90.91 (Ar$\underline{C}$10), 65.27 (-O$\underline{C}H_2$-), 64.97 (-O$\underline{C}H_2$-), 64.53 (-O$\underline{C}H_2$-), 27.97 (-C$\underline{Me}_3$) ppm; IR (υ_{max}, solid): 2983, 2934, 2871, 1737, 1611, 1508, 1469, 1455, 1359, 1335, 1263, 1223, 1164, 1146, 1117, 1074 cm^{-1}; HRMS *m/z* (ESI+): Found: 488.9661/490.9648 (M+H), Calc.: 488.9657/490.9636; m.p. > 350 °C;

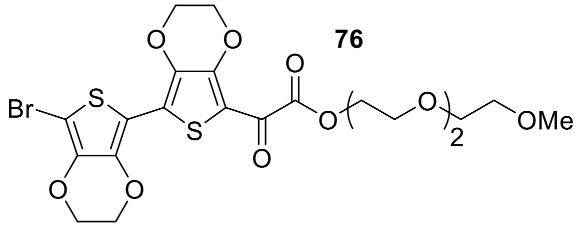

76

Method B on 0.26 mmol scale. The product was sufficiently pure for further applications. An analytical sample was obtained via column chromatography eluting with 2 % NEt_3 in 70-100 % EtOAc:Hexane. A yield of 150 mg, 0.24 mmol (93 %) was obtained as a green-yellow solid. ^{1}H NMR (400 MHz, $CDCl_3$): δ = 4.45-4.50 (2H, m, TEG), 4.36-4.45 (6H, m, -OC$\underline{H}_2$-), 4.32-4.46 (2H, m, -OC$\underline{H}_2$-), 3.79-3.85 (2H, m, TEG), 3.68-3.72 (2H, m, TEG), 3.61-3.68 (4H, m, TEG), 3.51-3.56 (2H, m, TEG), 3.36 (3H, s, -O$\underline{Me}$) ppm; ^{13}C NMR (400 MHz, $CDCl_3$): δ = 174.67 (-$\underline{C}$OCO_2R), 163.54 (-$\underline{C}O_2$R), 148.17 (Ar$\underline{C}_\beta$), 139.77 (Ar$\underline{C}_\beta$), 139.53 (Ar$\underline{C}_\beta$), 136.24 (Ar$\underline{C}_\beta$), 123.15 (Ar$\underline{C}_\alpha$), 110.62 (Ar$\underline{C}_\alpha$), 109.23 (Ar$\underline{C}_\alpha$), 91.28 (Ar$\underline{C}$10), 71.89 (TEG), 70.74 (TEG), 70.60 (TEG), 70.56 (TEG), 68.60 (TEG), 65.59 (-O$\underline{C}H_2$-), 65.30 (-O$\underline{C}H_2$-/TEG), 65.27 (-O$\underline{C}H_2$-/TEG), 64.96 (-O$\underline{C}H_2$-/TEG), 64.57 (-O$\underline{C}H_2$-), 59.01 (-O$\underline{Me}$) ppm; IR (υ_{max}, film): 2879, 2160, 1725, 1634, 1470, 1450, 1362, 1263, 1216, 1076 cm^{-1}; HRMS *m/z* (ESI+): Found: 578.9994/581.0024 (M+H), Calc.: 578.9997/580.9977; m.p. = 115-120 °C;

Chain extension-Trimers

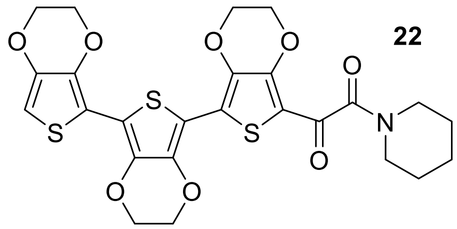

22

Run on 0.9 mmol scale at 130 ˚C. Column eluted with 2 % NEt_3 in 70-100 % EtOAc:Hexane. A yield of 284 mg, 0.51 mmol (56 %) was obtained as an orange solid. ^{1}H NMR (400 MHz, $CDCl_3$): δ = 6.37 (1H, s, Ar$\underline{H}$15), 4.36-4.49 (10H, m, -

OCH$_2$-), 4.23-4.30 (2H, m, -OCH$_2$-), 3.61-3.70 (2H, m, -CH$_2$N-), 3.36-3.40 (2H, m, -CH$_2$N-), 1.62-1.74 (4H, m, -CH$_2$CH$_2$CH$_2$-), 1.55-1.61 (2H, m, -CH$_2$CH$_2$N-) ppm; ^{13}C NMR (100 MHz, CDCl$_3$): δ = 181.43 (-COCONR$_2$), 166.64 (-CONR$_2$), 146.61 (ArC$_\beta$), 141.30 (ArC$_\beta$), 140.16 (ArC$_\beta$), 137.80 (ArC$_\beta$), 136.54 (ArC$_\beta$), 135.73 (ArC$_\beta$), 123.36 (ArC$_\alpha$), 112.96 (ArC$_\alpha$), 109.67 (ArC$_\alpha$), 106.90 (ArC$_\alpha$), 99.22 (ArC15), 65.52 (-OCH$_2$-), 65.25 (-OCH$_2$-), 64.91 (-OCH$_2$-), 64.59 (-OCH$_2$-), 47.06 (-CH$_2$N-), 42.18 (-CH$_2$N-), 26.07 (-CH$_2$CH$_2$CH$_2$N-), 25.28 (-CH$_2$CH$_2$CH$_2$N-), 24.56 (-CH$_2$CH$_2$CH$_2$N-) ppm; HRMS *m/z* (ESI+): Found: 562.0667 (M+H), Calc.: 562.0664; IR ($υ_{max}$, solid): 2924, 2862, 1650, 1607, 1510, 1466, 1433, 1313, 1254, 1223, 1176, 1121, 1101, 1077, 1059, cm^{-1}; m.p. = 319-321 °C;

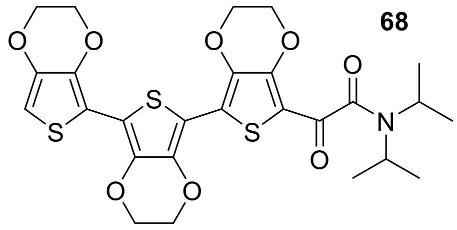

68

Run on 0.9 mmol scale. Column eluted with 2 % NEt$_3$ in 70-100 % EtOAc:Hexane. A yield of 252 mg, 0.44 mmol (48 %) was obtained as an orange solid. ^{1}H NMR (400 MHz, CDCl$_3$): δ = 6.35 (1H, s, ArH15), 4.30-4.48 (10H, m, -OCH$_2$-), 4.20-4.30 (2H, m, -OCH$_2$-), 3.85 (1H, sept, *J* = 5.7 Hz, -CHMe$_2$), 3.53 (1H, sept, *J* = 6.8 Hz, -CHMe$_2$), 1.52 (6H, d, *J* = 6.8 Hz, -CHMe$_2$), 1.20 (6H, d, *J* = 5.7 Hz, -CHMe$_2$) ppm; ^{13}C NMR (100 MHz, CDCl$_3$): δ = 181.42 (-COCONR$_2$), 166.80 (-CONR$_2$), 146.25 (ArC$_\beta$), 141.30 (ArC$_\beta$), 139.99 (ArC$_\beta$), 137.74 (ArC$_\beta$), 136.53 (ArC$_\beta$), 135.61 (ArC$_\beta$), 122.83 (ArC$_\alpha$), 112.69 (ArC$_\alpha$), 112.65 (ArC$_\alpha$), 109.67 (ArC$_\alpha$), 106.97 (ArC$_\alpha$), 99.10 (ArC15), 65.24 (-OCH$_2$-), 65.12 (-OCH$_2$-), 65.08 (-OCH$_2$-), 64.92 (-OCH$_2$-), 64.59 (-OCH$_2$-), 50.26 (-CHMe$_2$), 45.68 (-CHMe$_2$), 20.61 (-CHMe$_2$), 20.09 (-CHMe$_2$) ppm; IR ($υ_{max}$, solid): 2973, 2932, 2872, 1634, 1614, 1509 ,1466, 1431, 1358, 1267, 1077, 1062, 1043 cm^{-1}; HRMS *m/z* (ESI+): Found: 578.0968 (M+H), Calc.: 578.0977; m.p. = 218-220 °C (Degrades);

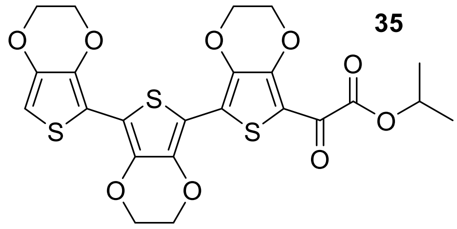

35

Run on 1.26 mmol scale. Column eluted with 2 % NEt$_3$ in 50-100 % EtOAc:Hexane. A yield of 270 mg, 0.5 mmol (40 %) was obtained as a red solid. ^{1}H NMR (400 MHz, CDCl$_3$): δ = 6.38 (1H, s, ArH15), 5.26 (1H, sept, *J* = 6.3 Hz, -CHMe$_2$), 4.37-4.50 (10H, m, -OCH$_2$-), 4.24-4.32 (2H, m, -OCH$_2$-), 1.40 (6H, d, *J* = 6.3 Hz, -CHMe$_2$) ppm; ^{13}C NMR (100 MHz, CDCl$_3$): δ = 174.97 (-COCO$_2$*i*Pr), 163.41 (-CO$_2$*i*Pr), 147.81 (ArC$_\beta$), 141.31 (ArC$_\beta$), 140.38 (ArC$_\beta$), 137.88 (ArC$_\beta$), 136.56 (ArC$_\beta$), 135.57 (ArC$_\beta$), 124.27 (ArC$_\alpha$), 113.23 (ArC$_\alpha$), 109.97 (ArC$_\alpha$), 109.88 (ArC$_\alpha$), 106.95 (ArC$_\alpha$), 99.34 (ArC15), 70.29 (-CHMe$_2$), 65.36 (-OCH$_2$-), 65.31 (-OCH$_2$-), 65.24 (-OCH$_2$-), 64.90 (-OCH$_2$-), 64.58 (-OCH$_2$-), 64.54 (-OCH$_2$-), 21.63 (-CHMe$_2$) ppm; IR ($υ_{max}$, solid): 2926,

1721, 1645, 1606, 1508, 1465, 1432, 1397, 1360, 1319, 1252, 1217, 1175, 1083, 1061, 1014 cm^{-1}; HRMS *m/z* (ESI+): Found: 537.0359 (M+H), Calc.: 537.0350; m.p. = 267-268 °C;

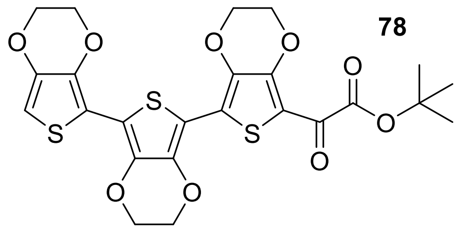

78

Run on 365 μmol scale. Column eluted with 2 % NEt_3 in 50-80 % EtOAc:Hexane. A yield of 74 mg, 135 μmol (37 %) was obtained as an orange solid. ^{1}H NMR (400 MHz, $CDCl_3$): δ = 6.37 (1H, s, ArH15), 4.36-4.49 (10H, m, -OCH_2-), 4.20-4.30 (2H, m, -OCH_2-), 1.61 (9H, s, -O*t*Bu) ppm; ^{13}C NMR (100 MHz, $CDCl_3$): δ = 176.05 (-COCO_2*t*Bu), 163.35 (-CO_2*t*Bu), 147.66 (ArC$_β$), 141.30 (ArC$_β$), 140.24 (ArC$_β$), 137.83 (ArC$_β$), 136.54 (ArC$_β$), 135.55 (ArC$_β$), 122.52 (ArC$_α$), 113.09 (ArC$_α$), 110.69 (ArC$_α$), 109.66 (ArC$_α$), 106.97 (ArC$_α$), 99.26 (ArC15), 83.89 (-CMe_3), 65.30 (-OCH_2-), 65.25 (-OCH_2-), 64.90 (-OCH_2-), 64.59 (-OCH_2-), 64.55 (-OCH_2-), 27.98 (-CMe_3) ppm; IR ($υ_{max}$, solid): 2978, 2931, 2873, 1720, 1607, 1509, 1430, 1360, 1252, 1222, 1162, 1145, 1123, 1083, 1025 cm^{-1}; HRMS *m/z* (ESI+): Found: 551.0491 (M+H), Calc.: 551.0504; m.p. = 214 °C (Degrades);

Trimer bromination

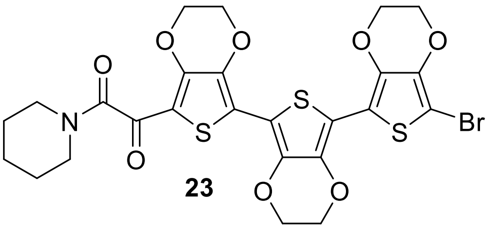

23

Run on 106 μmol scale, following 'dimer bromination procedure A'. After precipitating in water (2 x 15 mL) and collecting by centrifugation (5000 rpm, 10 min), the product was used immediately without further purification or analysis due to its instability.

Oligomer synthesis protocol

Brominated oligomer (1 mmol), hydrogen-capped oligomer (1.2 mmol), pivalic acid (0.5 mmol), palladium (II) acetate (0.05 mmol) and potassium carbonate (10 mmol) were charged under nitrogen. Dry DMF (2 mL) was added and the mixture heated to 90 ºC for 2 hrs. After cooling to rt the mixture was diluted with DCM (50 mL) and washed with water (2 x 50 mL) and brine (50 mL). The organics were dried with $MgSO_4$, filtered and concentrated *in vacuo*. The residue was purified by flash column chromatography and pure fractions were concentrated *in vacuo*.

Numbering system for bifunctional-oligomer NMR assignments

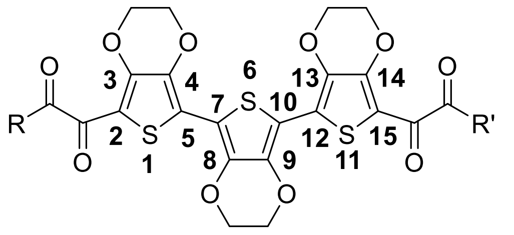

Dimer synthesis

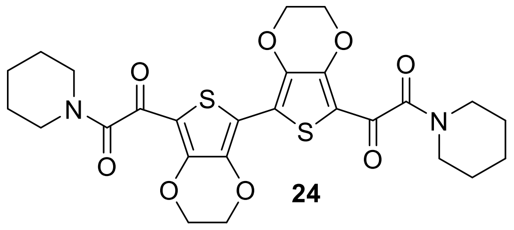

24

Run on 278 µmol scale at 130 °C with brominated piperidine-EDOT monomer **4** and piperidine-EDOT monomer **3**. Column eluted with 50-100 % EtOAc:Hexane. Yield of 65 mg, 116 mmol (42 %) as a yellow solid. ^{1}H NMR (400 MHz, $CDCl_3$): δ = 4.43 (8H, s, -OC$\underline{H}_2$-), 3.64 (4H, t, J = 5.2 Hz, -C$\underline{H}_2$N-), 3.38 (4H, t, J = 5.4 Hz, -C$\underline{H}_2$N-), 1.62-1.74 (8H, m, -C$\underline{H}_2$C$\underline{H}_2$CH$_2$N-), 1.54-1.62 (2H, m, -C$\underline{H}_2$CH$_2$N-) ppm; ^{13}C NMR (100 MHz, $CDCl_3$): δ = 182.19 (-$\underline{C}$OCONR$_2$), 165.03 (-$\underline{C}$ONR$_2$), 146.08 (Ar$\underline{C}$3), 139.05 (Ar$\underline{C}$4), 119.75 (Ar$\underline{C}_\alpha$), 115.13 (Ar$\underline{C}_\alpha$), 65.49 (-O$\underline{C}$H$_2$), 64.77 (-O$\underline{C}$H$_2$), 47.02 (-$\underline{C}$H$_2$N-), 42.28 (-$\underline{C}$H$_2$N-), 26.08 (-$\underline{C}$H$_2\underline{C}$H$_2$CH$_2$N-), 25.24 (-$\underline{C}$H$_2\underline{C}$H$_2$CH$_2$N-), 24.48 (-$\underline{C}$H$_2\underline{C}$H$_2$CH$_2$N-) ppm; IR (υ_{max}, solid): 2935, 2921, 2854, 1644, 1606, 1556, 1476, 1440, 1358, 1304, 1261, 1253, 1223, 1150, 1119, 1072, 1006 cm^{-1}; HRMS m/z (ESI+): Found: 561.1383 (M+H), Calc.: 561.1365; m.p. > 350 °C;

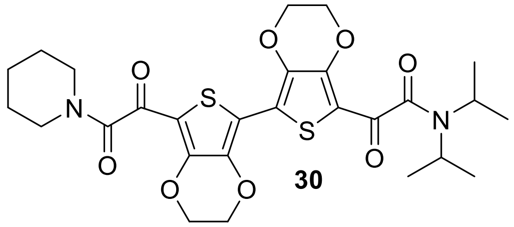

30

Run on 139 µmol scale with brominated piperidine-EDOT monomer **4** and diisopropyl-EDOT monomer **65**. Column eluted with 60-100 % EtOAc:Hexane. Yield of 59 mg, 102 µmol (74 %) as a yellow oily solid. ^{1}H NMR (400 MHz, $CDCl_3$): δ = 4.35-4.49 (8H, m, -OC$\underline{H}_2$-), 3.82 (1H, sept, J = 6.6 Hz, -C$\underline{H}$Me$_2$), 3.64 (2H, t, J = 5.3 Hz, -NC$\underline{H}_2$-), 3.55 (1H, sept, J = 6.8 Hz, -C$\underline{H}$Me$_2$), 3.38 (2H, t, J = 5.3 Hz, -NC$\underline{H}_2$-), 1.55-1.85 (6H, m, -C$\underline{H}_2$C$\underline{H}_2$CH$_2$N-), 1.52 (6H, d, J = 6.8 Hz, -CH$\underline{Me}_2$), 1.21 (6H, d, J = 6.6 Hz, -CH$\underline{Me}_2$) ppm; ^{13}C NMR (100 MHz, $CDCl_3$): δ = 182.19 (-$\underline{C}$OCON-), 182.00 (-$\underline{C}$OCON-), 166.21 (-$\underline{C}$ON^{i}Pr), 165.07 (-$\underline{C}$OPip), 146.12 (Ar$\underline{C}_\beta$), 145.69 (Ar$\underline{C}_\beta$), 138.98 (Ar$\underline{C}_\beta$), 138.94 (Ar$\underline{C}_\beta$), 119.93 (Ar$\underline{C}_\alpha$), 119.33 (Ar$\underline{C}_\alpha$), 115.43 (Ar$\underline{C}_\alpha$), 115.01 (Ar$\underline{C}_\alpha$), 65.49 (-O$\underline{C}$H$_2$), 65.08 (-O$\underline{C}$H$_2$), 64.75 (-O$\underline{C}$H$_2$), 50.31 (-$\underline{C}$HMe$_2$), 47.02 (-$\underline{C}$H$_2$N-), 45.82 (-$\underline{C}$HMe$_2$), 42.27 (-$\underline{C}$H$_2$N-), 26.07 (-$\underline{C}$H$_2\underline{C}$H$_2$CH$_2$N-), 25.25 (-$\underline{C}$H$_2\underline{C}$H$_2$CH$_2$N-), 24.49 (-$\underline{C}$H$_2\underline{C}$H$_2$CH$_2$N-), 20.63 (-CH$\underline{Me}_2$), 20.03 (-CH$\underline{Me}_2$) ppm; IR (υ_{max}, solid): 2971, 2937, 2860, 1634, 1614, 1551, 1473,

1434, 1357, 1280, 1254, 1233, 1216, 1115, 1074, 1042 cm^{-1}; HRMS *m/z* (ESI+): Found: 577.1693 (M+H), Calc.: 577.1678; m.p. = 312-314 °C;

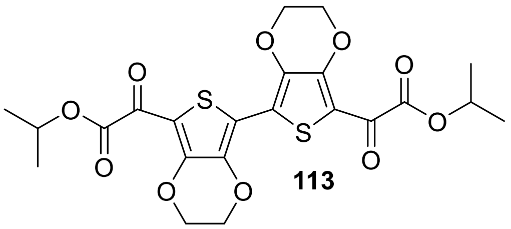
113

Run on 290 μmol scale with brominated isopropyl ester-EDOT monomer **8** and isopropyl ester-EDOT monomer **42**. Column eluted with 20-50 % EtOAc:Hexane. Yield of 44 mg, 86 mmol (30 %) as a yellow-orange solid. The product was highly insoluble in all standard NMR solvents and ^{13}C NMR analysis could not therefore be undertaken. ^{1}H NMR (400 MHz, DMSO-d$_6$): δ = 5.16 (2H, sept, *J* = 6.4 Hz, -C<u>H</u>Me$_2$), 4.48-4.57 (8H, m, -OC<u>H</u>$_2$-), 1.32 (12H, d, *J* = 6.4 Hz, -CH<u>Me</u>$_2$) ppm; IR (υ$_{max}$, solid): 2923, 2872, 2853, 1732, 1635, 1473, 1432, 1366, 1275, 1227, 1103, 1075, 1021 cm^{-1}; HRMS *m/z* (ESI+): Found: 511.0739 (M+H), Calc.: 511.0733; m.p. = 225-228 °C;

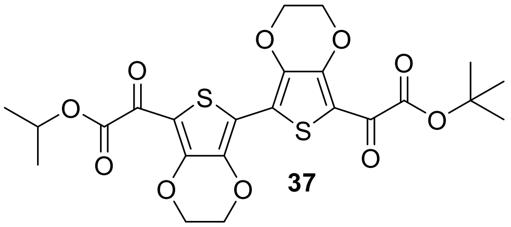
37

Run on 286 μmol scale with brominated *tert*-butyl ester-EDOT monomer **9** and isopropyl ester-EDOT monomer **42**. Column eluted with 20-50 % EtOAc:Hexane. Yield of 100 mg, 188 mmol (66 %) as a yellow solid. ^{1}H NMR (400 MHz, CDCl$_3$): δ = 5.26 (1H, sept, *J* = 6.3 Hz, -C<u>H</u>Me$_2$), 4.37-4.51 (8H, m, -OC<u>H</u>$_2$-), 1.61 (9H, s, -C<u>Me</u>$_3$), 1.40 (6H, d, *J* = 6.3 Hz, -CH<u>Me</u>$_2$) ppm; ^{13}C NMR (100 MHz, CDCl$_3$): δ = 176.86 (-<u>C</u>OCO$_2$R), 176.25 (-<u>C</u>OCO$_2$R), 162.98 (-<u>C</u>O$_2$R), 162.70 (-<u>C</u>O$_2$R), 147.40 (Ar<u>C</u>$_β$), 147.06 (Ar<u>C</u>$_β$), 139.06 (Ar<u>C</u>$_β$), 138.97 (Ar<u>C</u>$_β$), 120.63 (Ar<u>C</u>$_α$), 120.11 (Ar<u>C</u>$_α$), 113.80 (Ar<u>C</u>$_α$), 113.53 (Ar<u>C</u>$_α$), 84.47 (-<u>C</u>Me$_3$), 70.74 (-<u>C</u>HMe$_2$), 65.32 (-O<u>C</u>H$_2$), 65.22 (-O<u>C</u>H$_2$), 64.75 (-O<u>C</u>H$_2$), 27.95 (-C<u>Me</u>$_3$), 21.61 (-CH<u>Me</u>$_2$) ppm; IR (υ$_{max}$, solid): 2981, 2935, 1732, 1620, 1547, 1472, 1439, 1360, 1317, 1245, 1222, 1162, 1121, 1099, 1075, 1027 cm^{-1}; HRMS *m/z* (ESI+): Found: 525.0880 (M+H), Calc.: 525.0889; m.p. = 270-272 °C (Degrades);

Trimer Synthesis

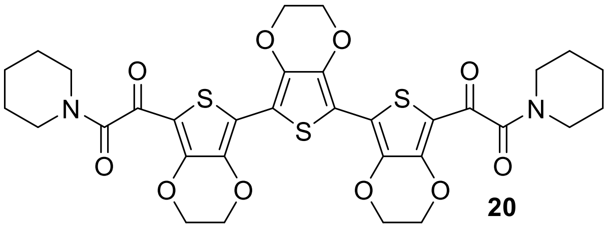
20

Run on 142 μmol scale at 130 °C with dibromo-EDOT **25** and piperidine-EDOT monomer **3**. Column eluted with 0-8 % MeOH:EtOAc. Yield of 68 mg, 97 mmol (68 %) as a red solid. Alternatively, the product could be accessed through the coupling of brominated piperidine-EDOT

monomer **4** and piperidine-EDOT dimer **19** in a 58 % yield. ^{1}H NMR (400 MHz, $CDCl_3$): δ = 4.33-4.57 (12H, s, -OC<u>H</u>$_2$-), 3.64 (4H, t, *J* = 5.2 Hz, -C<u>H</u>$_2$N-), 3.38 (4H, t, *J* = 5.4 Hz, -C<u>H</u>$_2$N-), 1.61-1.74 (8H, m, -C<u>H</u>$_2$C<u>H</u>$_2$CH$_2$N-), 1.52-1.61 (4H, m, -C<u>H</u>$_2$CH$_2$N-) ppm; ^{13}C NMR (100 MHz, $CDCl_3$): δ = 181.68 (-<u>C</u>OCONR$_2$), 165.40 (-<u>C</u>ONR$_2$), 146.41 (Ar<u>C</u>3), 139.71 (Ar<u>C</u>$_β$), 136.73 (Ar<u>C</u>$_β$), 122.00 (Ar<u>C</u>$_α$), 113.24 (Ar<u>C</u>$_α$), 110.94 (Ar<u>C</u>$_α$), 65.51 (-O<u>C</u>H$_2$), 65.22 (-O<u>C</u>H$_2$-), 64.72 (-O<u>C</u>H$_2$), 47.05 (-<u>C</u>H$_2$N-), 42.22 (-<u>C</u>H$_2$N-), 26.07 (-<u>C</u>H$_2$<u>C</u>H$_2$CH$_2$N-), 25.26 (-<u>C</u>H$_2$<u>C</u>H$_2$CH$_2$N-), 24.52 (-<u>C</u>H$_2$<u>C</u>H$_2$CH$_2$N-) ppm; IR ($υ_{max}$, solid): 2923, 2853, 1642 1622, 1614, 1486, 1431, 1361, 1308, 1251, 1217, 1117, 1096, 1065 cm^{-1}; HRMS *m/z* (ESI+): Found: 701.1299 (M+H), Calc.: 701.1292; m.p. = 315-318 °C;

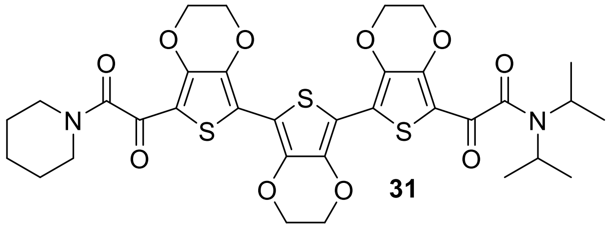

31

Run on 100 µmol scale with brominated piperidine-EDOT monomer **4** and diisopropyl-EDOT dimer **66**. Colum eluted with 60-100 % EtOAc:Hexane. Yield of 51 mg, 72 µmol (72 %) as an orange solid. ^{1}H NMR (400 MHz, $CDCl_3$): δ = 4.33-4.52 (12H, m, -OC<u>H</u>$_2$-), 3.87 (1H, sept, *J* = 6.6 Hz, -C<u>H</u>Me$_2$), 3.65 (2H, t, *J* = 5.3 Hz, -NC<u>H</u>$_2$-), 3.55 (1H, sept, *J* = 6.8 Hz, -C<u>H</u>Me$_2$), 3.40 (2H, t, *J* = 5.3 Hz, -NC<u>H</u>$_2$-), 1.62-1.78 (6H, m, -C<u>H</u>$_2$C<u>H</u>$_2$CH$_2$N-), 1.57-1.64 (2H, m, -C<u>H</u>$_2$C<u>H</u>$_2$CH$_2$N-), 1.53 (6H, d, *J* = 6.8 Hz, -CH<u>Me</u>$_2$), 1.21 (6H, d, *J* = 6.6 Hz, -CH<u>Me</u>$_2$) ppm; ^{13}C NMR (100 MHz, $CDCl_3$): δ = 181.65 (-<u>C</u>OCON-), 181.60 (-<u>C</u>OCON-), 166.55 (-<u>C</u>ON^{i}Pr), 165.39 (-<u>C</u>OPip), 146.39 (Ar<u>C</u>$_β$), 145.98 (Ar<u>C</u>$_β$), 139.74 (Ar<u>C</u>$_β$), 139.57 (Ar<u>C</u>$_β$), 136.67 (Ar<u>C</u>$_β$), 136.61 (Ar<u>C</u>$_β$), 122.07 (Ar<u>C</u>$_α$), 121.49 (Ar<u>C</u>$_α$), 113.55 (Ar<u>C</u>$_α$), 113.23 (Ar<u>C</u>$_α$), 111.05 (Ar<u>C</u>$_α$), 110.71 (Ar<u>C</u>$_α$), 65.48 (-O<u>C</u>H$_2$), 65.21 (-O<u>C</u>H$_2$), 65.17 (-O<u>C</u>H$_2$), 65.07 (-O<u>C</u>H$_2$-), 64.69 (-O<u>C</u>H$_2$-), 50.26 (-<u>C</u>HMe$_2$), 47.06 (-<u>C</u>H$_2$N-), 45.75 (-<u>C</u>HMe$_2$), 42.24 (-<u>C</u>H$_2$N-), 26.09 (-<u>C</u>H$_2$<u>C</u>H$_2$CH$_2$N-), 25.28 (-<u>C</u>H$_2$<u>C</u>H$_2$CH$_2$N-), 24.54 (-<u>C</u>H$_2$<u>C</u>H$_2$CH$_2$N-), 20.62 (-CH<u>Me</u>$_2$), 20.07 (-CH<u>Me</u>$_2$) ppm; IR ($υ_{max}$, solid): 2930, 2863, 1720, 1631, 1610, 1545, 1507, 1465, 1424, 1359, 1314, 1251, 1216, 1150, 1116, 1067, 1014 cm^{-1}; HRMS *m/z* (ESI+): Found: 717.1616 (M+H), Calc.: 717.1610; m.p. = 324-326 °C;

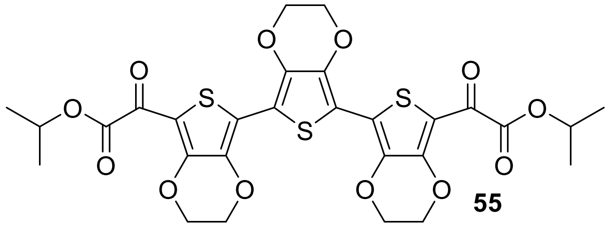

55

Run on 156 µmol scale with dibromo-EDOT **25** and isopropyl ester-EDOT monomer **42**. Column eluted with 30-80 % EtOAc:Hexane. Yield of 74 mg, 114 mmol (73 %) as a deep red solid. ^{1}H NMR (400 MHz, $CDCl_3$): δ = 5.26 (2H, sept, *J* = 6.2 Hz, -C<u>H</u>Me$_2$), 4.40-4.51 (12H, m, -OC<u>H</u>$_2$-), 1.61

(9H, s, -CMe$_3$), 1.40 (12H, d, J = 6.2 Hz, -CHMe$_2$); ^{13}C NMR (100 MHz, CDCl$_3$): δ = 175.58 (-COCO$_2$*i*Pr), 163.31 (-CO$_2$*i*Pr), 147.77 (ArC3), 139.91 (ArC$_\beta$), 136.72 (ArC$_\beta$), 122.90 (ArC$_\alpha$), 111.77 (ArC$_\alpha$), 111.20 (ArC$_\alpha$), 70.50 (-CHMe$_2$), 65.33 (-OCH$_2$), 65.24 (-OCH$_2$-), 64.66 (-OCH$_2$), 21.63 (-CMe$_2$) ppm; IR (υ$_{max}$, solid): 2938, 2858, 1716, 1683, 1634, 1621, 1471, 1427, 1375, 1360, 1314, 1299, 1217, 1197, 1172, 1133, 1117, 1069 cm^{-1}; HRMS *m/z* (ESI+): Found: 651.0677 (M+H), Calc.: 651.0665; m.p. = 293-296 °C;

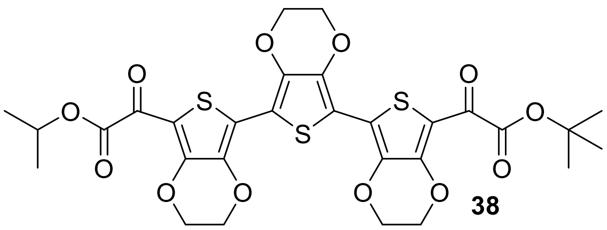

38

Run on 210 μmol scale with brominated *tert*-butyl ester-EDOT monomer **9** and isopropyl ester-EDOT dimer **34**. Column eluted with 40-80 % EtOAc:Hexane. Yield of 114 mg, 171 mmol (82 %) as a deep red solid. ^{1}H NMR (400 MHz, CDCl$_3$): δ = 5.25 (1H, sept, J = 6.2 Hz, -CHMe$_2$), 4.39-4.49 (12H, m, -OCH$_2$-), 1.61 (9H, s, -CMe$_3$), 1.39 (6H, d, J = 6.2 Hz, -CHMe$_2$) ppm; ^{13}C NMR (100 MHz, CDCl$_3$): δ = 176.26 (-COCO$_2$R), 175.58 (-COCO$_2$R), 163.35 (-CO$_2$R), 163.06 (-CO$_2$R), 147.78 (ArC$_\beta$), 147.44 (ArC$_\beta$), 139.91 (ArC$_\beta$), 139.77 (ArC$_\beta$), 136.68 (ArC$_\beta$), 122.95 (ArC$_\alpha$), 122.42 (ArC$_\alpha$), 111.87 (ArC$_\alpha$), 111.27 (ArC$_\alpha$) 111.02 (ArC$_\alpha$), 84.14 (-CMe$_3$), 70.49 (-CHMe$_2$), 65.36 (-OCH$_2$), 65.25 (-OCH$_2$-), 64.67 (-OCH$_2$), 27.96 (-CMe$_3$), 21.63 (-CMe$_2$) ppm; IR (υ$_{max}$, solid): 2981, 2934, 2876, 1736, 1613, 1469, 1434, 1359, 1335, 1265, 1244, 1221, 1163, 1135, 1117, 1097, 1069 cm^{-1}; HRMS *m/z* (ESI+): Found: 664.9289 (M+H), Calc.: 665.0821; m.p. = 256-257 °C;

Tetramer Synthesis

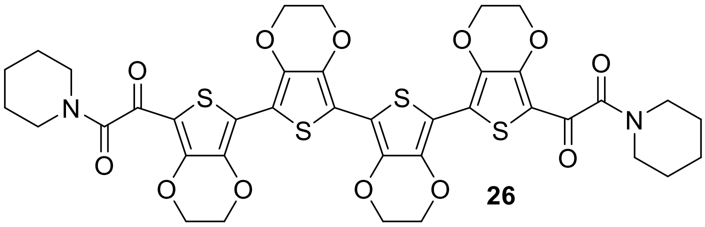

26

Run on 200 μmol scale with brominated piperidine-EDOT dimer **21** and piperidine-EDOT dimer **19**. Column eluted with 0-5 % MeOH:DCM. Yield of 148 mg, 176 μmol (88 %) as a deep red/purple solid. ^{1}H NMR (400 MHz, CDCl$_3$): δ = 4.14-4.64 (16H, m, -OCH$_2$-), 3.58-3.72 (4H, m, -NCH$_2$-), 3.29-3.48 (4H,m, -NCH$_2$-), 1.39-1.81 (12H, m, -CH$_2$CH$_2$CH$_2$N-) ppm; ^{13}C NMR (125 MHz, CDCl$_3$): 181.76 (-COCONR$_2$), 165.35 (-CONR$_2$), 146.33 (ArC$_\beta$), 142.93 (ArC$_\beta$), 139.34 (ArC$_\beta$), 136.68 (ArC$_\beta$), 121.80 (ArC$_\alpha$), 112.86 (ArC$_\alpha$), 111.91 (ArC$_\alpha$), 108.77 (ArC$_\alpha$), 65.44 (-OCH$_2$-), 64.97 (-OCH$_2$-), 64.92 (-OCH$_2$-), 64.54 (-OCH$_2$-), 47.02 (-CH$_2$N-), 42.21 (-CH$_2$N-), 26.04 (-CH$_2$CH$_2$CH$_2$N-), 25.24 (-CH$_2$CH$_2$CH$_2$N-), 24.50 (-CH$_2$CH$_2$CH$_2$N-) ppm; IR (υ$_{max}$, solid): 2931, 2853,

1652, 1601, 1574, 1488, 1463, 1428, 1358, 1308, 1279, 1263, 1250, 1219, 1135, 1116, 1108, 1067, 1022 cm^{-1}; HRMS *m/z* (ESI+): Found: 841.1229 (M+H), Calc.: 841.1232; m.p. > 350 °C;

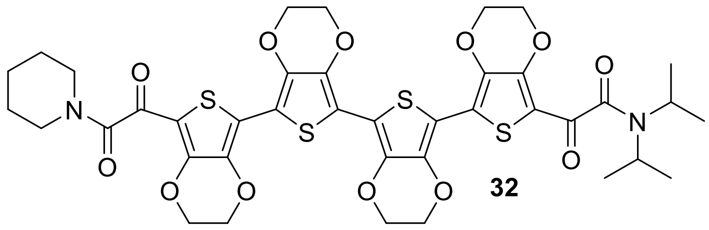

32

Run on 237 μmol scale with brominated diisopropyl-EDOT dimer **67** and piperidine-EDOT dimer **19**. Column eluted with 0-5 % MeOH:DCM. Yield of 136 mg, 158 μmol (67 %) as a deep red/purple solid. ^{1}H NMR (400 MHz, $CDCl_3$): δ = 4.18-4.66 (16H, m, -OC$\underline{H}_2$-), 3.86 (1H, sept, *J* = 6.6 Hz, -C$\underline{H}$Me$_2$), 3.64 (2H, t, *J* = 5.0 Hz, -NC$\underline{H}_2$-), 3.54 (1H, sept, *J* = 6.7 Hz, -C$\underline{H}$Me$_2$), 3.39 (2H, t, *J* = 5.4 Hz, -NC$\underline{H}_2$-), 1.60-1.74 (4H, m, -C$\underline{H}_2$C$\underline{H}_2$CH$_2$N-), 1.55-1.60 (2H, m, -C$\underline{H}_2$CH$_2$N-), 1.52 (6H, d, *J* = 6.7 Hz, -CH$\underline{Me}_2$), 1.20 (6H, d, *J* = 6.6 Hz, -CH$\underline{Me}_2$) ppm; ^{13}C NMR (125 MHz, $CDCl_3$): 181.72 (-$\underline{C}$OCONR$_2$), 181.49 (-$\underline{C}$OCONR$_2$), 166.67 (-$\underline{C}$ONR$_2$), 165.53 (-$\underline{C}$ONR$_2$), 146.52 (Ar$\underline{C}_\beta$), 141.32 (Ar$\underline{C}_\beta$), 140.27 (Ar$\underline{C}_\beta$), 137.24 (Ar$\underline{C}_\beta$), 136.07 (Ar$\underline{C}_\beta$), 122.88 (Ar$\underline{C}_\alpha$), 112.15 (Ar$\underline{C}_\alpha$), 108.94 (Ar$\underline{C}_\alpha$), 101.83 (Ar$\underline{C}_\alpha$), 65.47 (-O$\underline{C}$H$_2$-), 65.18 (-O$\underline{C}$H$_2$-), 65.10 (-O$\underline{C}$H$_2$-), 64.60 (-O$\underline{C}$H$_2$-), 50.21 (-$\underline{C}$HMe$_2$), 47.01 (-$\underline{C}$H$_2$N-), 45.66 (-$\underline{C}$HMe$_2$), 42.14 (-$\underline{C}$H$_2$N-), 26.02 (-$\underline{C}$H$_2\underline{C}$H$_2$CH$_2$N-), 25.22 (-$\underline{C}$H$_2\underline{C}$H$_2$CH$_2$N-), 24.50 (-$\underline{C}$H$_2\underline{C}$H$_2$CH$_2$N-), 20.56 (-CH$\underline{Me}_2$), 20.03 (-CH$\underline{Me}_2$) ppm; IR ($υ_{max}$, solid): 2933, 1634, 1594, 1542, 1493, 1467, 1428, 1359, 1251, 1220, 1152, 1136, 1116, 1094, 1067 cm^{-1}; HRMS *m/z* (ESI+): Found: 857.1528 (M+H), Calc.: 857.1542; m.p. > 350 °C;

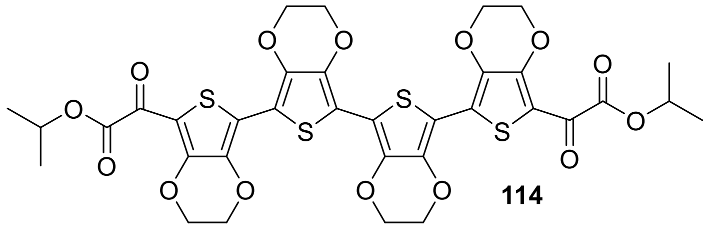

114

Run on 210 μmol scale with brominated *iso*-propyl ester-EDOT dimer **72** and *iso*-propyl ester-EDOT dimer **34**. Column eluted with 0-5 % MeOH:DCM. Yield of 122 mg, 154 μmol (74 %) as a purple solid. The low solubility of the product in $CDCl_3$ and propensity to aggregate in solution prevented the aromatic peaks being resolved in the ^{13}C NMR spectra and so analysis was not undertaken. ^{1}H NMR (400 MHz, $CDCl_3$): δ = 5.27 (2H, sept, *J* = 6.5 Hz, -C$\underline{H}$Me$_2$), 4.32-4.54 (16H, m, -OC$\underline{H}_2$-), 1.41 (12H, d, *J* = 6.5 Hz, -CH$\underline{Me}_2$) ppm; IR ($υ_{max}$, solid): 2932, 2869, 1734, 1601, 1490, 1464, 1428, 1359, 1313, 1214, 1139, 1098, 1066, 1022 cm^{-1}; HRMS *m/z* (ESI+): Found: 791.0597 (M+H), Calc.: 791.0597; m.p. > 350 °C;

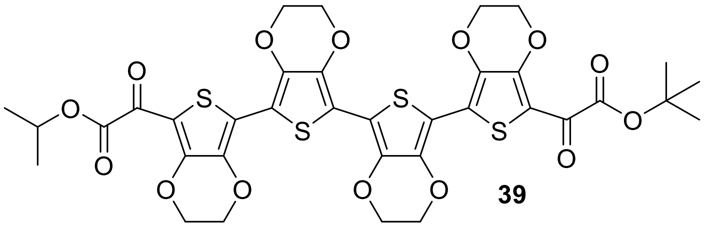

39

Run on 91 µmol scale with brominated *iso*-propyl ester-EDOT monomer **8** and *tert*-butyl ester-EDOT trimer **78**. Column eluted with 1-4 % MeOH:DCM. Yield of 63 mg, 78 µmol (86 %) as a purple solid. The product was highly insoluble in all commonly used NMR solvents and quickly aggregated in solution preventing full analysis. Thus, the purity of the construct could not be determined. However, the presence of the product as the major species was confirmed by HRMS. ^{1}H NMR (400 MHz, $CDCl_3$): δ = 5.28 (1H, sept, *J* = 6.2 Hz, -C$\underline{H}$Me$_2$), 4.34-4.58 (16H, m, -OC$\underline{H_2}$-), 1.63 (9H, s, -C$\underline{Me_3}$), 1.41 (6H, d, *J* = 6.2 Hz, -CH$\underline{Me_2}$) ppm; IR ($υ_{max}$, solid): 2979, 2938, 2869, 1722, 1605, 1439, 1454, 1434, 1361, 1221, 1139, 1092, 1069 cm^{-1}; HRMS *m/z* (ESI+): Found: 805.0746 (M+H), Calc.: 805.0753; m.p. > 350 °C;

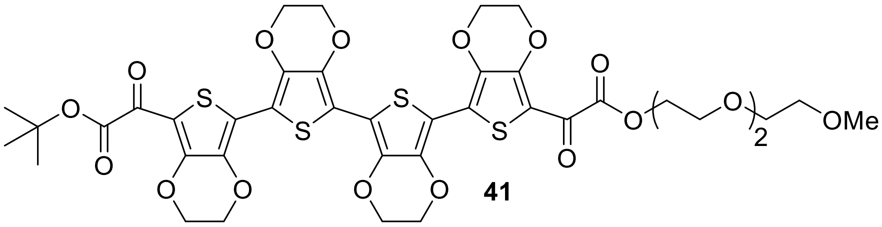

41

Run on 100 µmol scale with brominated TEG-EDOT monomer **74** and *tert*-butyl ester-EDOT trimer **78**. Column eluted with 1-5 % MeOH:DCM. Yield of 67 mg, 74 µmol (74 %) as a purple solid. Despite significantly improved solubility when compared to **39**, the aromatic peaks could not be resolved by ^{13}C NMR and so analysis was not undertaken ^{1}H NMR (400 MHz, $CDCl_3$): δ = 4.35-4.55 (16H, m, -OC$\underline{H_2}$-), 3.84 (2H, t, *J* = 5.0 Hz, TEG), 3.62-3.75 (6H, m, TEG), 3.53-3.59 (2H, m, TEG), 3.39 (3H, s, -O$\underline{Me}$), 1.60 (9H, s, -O$\underline{tBu}$) ppm; IR ($υ_{max}$, solid): 2946, 2928, 2873, 1732, 1600, 1428, 1358, 1319, 1217, 1203, 1138, 1117, 1092, 1064, 1021 cm^{-1}; HRMS *m/z* (ESI+): Found: 909.1221 (M+H), Calc.: 909.1227; m.p. > 350 °C;

Pentamer Synthesis

Aromatic peaks could not be resolved in the ^{13}C NMR spectra of all oligomers of 5 repeating units and longer, even after long scan times at elevated temperatures. Details are not therefore given for all subsequent oligomers.

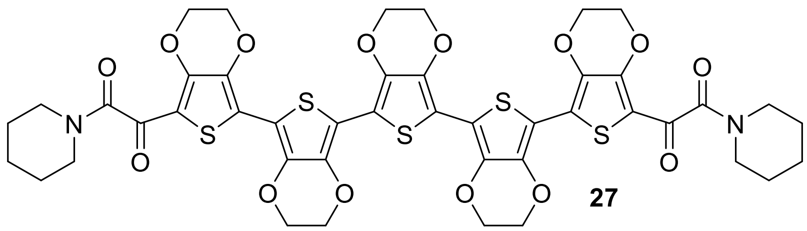

27

Run on 108 µmol scale with dibromo-EDOT **25** and piperidine-EDOT dimer **19**. Column eluted with 0-20 % MeOH:EtOAc. Yield of 58 mg, 59 µmol (55 %) as a purple solid. Alternatively, the product could be accessed through the coupling of brominated piperidine-EDOT dimer **21** and piperidine-EDOT trimer **22**. ^{1}H NMR (400 MHz, $CDCl_3$): δ = 4.40-4.49 (20H, s, -OC$\underline{H}_2$-), 3.65 (4H, t, *J* = 5.1 Hz, -C$\underline{H}_2$N-), 3.41 (4H, t, *J* = 5.3 Hz, -C$\underline{H}_2$N-), 1.65-1.73 (8H, m, -C$\underline{H}_2$C$\underline{H}_2$CH$_2$N-), 1.53-1.62 (4H, m, -C$\underline{H}_2$CH$_2$N-) ppm; IR (υ_{max}, solid): 2923, 2855, 1621, 1433, 1360, 1261, 1219, 1116, 1069 cm^{-1}; MS *m/z* (MALDI+): Found: 980.8 (M+H), Calc.: 981.1; m.p. > 350 °C;

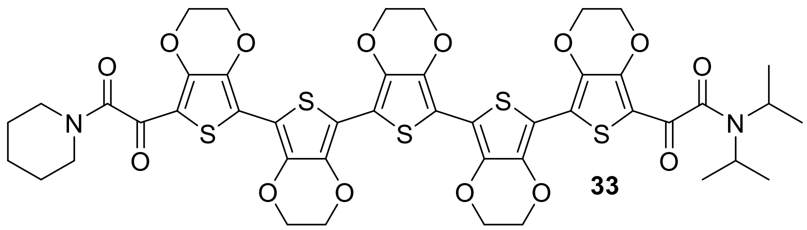

33

Run on 89 µmol scale with brominated diisopropyl-EDOT dimer **67** and piperidine-EDOT trimer **22**. Column eluted with 2-5 % MeOH:DCM. Yield of 39 mg, 39 µmol (44 %) as a purple solid. ^{1}H NMR (400 MHz, $CDCl_3$): δ = 4.19-4.56 (20H, s, -OC$\underline{H}_2$-), 3.88 (1H, sept, *J* = 6.9 Hz, -C$\underline{H}$Me$_2$), 3.66 (2H, t, *J* = 5.1 Hz, -C$\underline{H}_2$N-), 3.55 (1H, sept, *J* = 6.9 Hz, -C$\underline{H}$Me$_2$), 3.41 (2H, t, *J* = 5.4 Hz, -C$\underline{H}_2$N-), 1.63-1.74 (4H, m, -C$\underline{H}_2$C$\underline{H}_2$CH$_2$N-), 1.49-1.63 (8H, m, -C$\underline{H}_2$CH$_2$N- and –CH$\underline{Me}_2$), 1.22 (6H, d, *J* = 6.9 Hz, -CH$\underline{Me}_2$) ppm; IR (υ_{max}, solid): 2944, 2857, 1634, 1603, 1469, 1435, 1358, 1311, 1262, 1223, 1116, 1088, 1042 cm^{-1}; MS *m/z* (MALDI+): Found: 996.8 (M+H), Calc.: 997.1; m.p. > 350 °C;

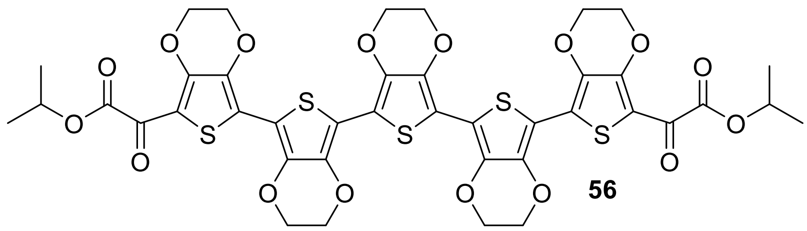

56

Run on 105 µmol scale with EDOT **1** and brominated *iso*-propyl ester-EDOT dimer **72**. Column eluted with 1-4 % MeOH:DCM. Yield of 56 mg, 64 µmol (61 %) as a purple solid. ^{1}H NMR (400 MHz, $CDCl_3$): δ = 5.27 (2H, sept, *J* = 6.2 Hz, -C$\underline{H}$Me$_2$), 4.39-4.54 (20H, m, -OC$\underline{H}_2$-), 1.41 (12H, d, *J* = 6.2 Hz, -CH$\underline{Me}_2$) ppm; IR (υ_{max}, film): 2920, 2850, 1737, 1603, 1462, 1430, 1360, 1257, 1211, 1098, 1066 cm^{-1}; MS *m/z* (MALDI+): Found: 930.6 (M+H), Calc.: 931.0; m.p. > 350 °C;

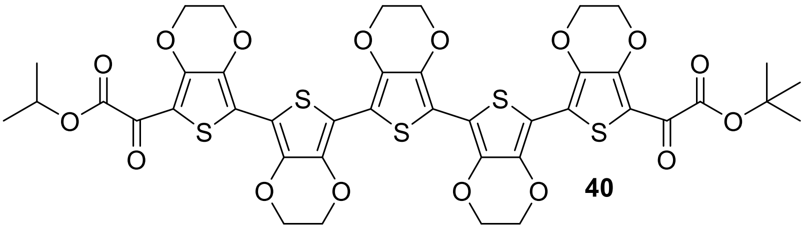

40

Run on 143 µmol scale with brominated *tert*-butyl ester-EDOT dimer **36** and *iso*-propyl ester-EDOT trimer **35**. Column eluted with 1-4 % MeOH:DCM. Yield of 56 mg, 60 µmol (42%) as a purple solid. ^{1}H NMR (400 MHz, $CDCl_3$): δ = 5.26 (1H, sept, *J* = 6.3 Hz, -C<u>H</u>Me$_2$), 4.33-4.53 (20H, m, -OC<u>H</u>$_2$-), 1.62 (9H, s, -C<u>Me</u>$_3$), 1.41 (6H, d, *J* = 6.3 Hz, -CH<u>Me</u>$_2$) ppm; IR ($υ_{max}$, solid): 2932, 1735, 1613, 1462, 1431, 1359, 1257, 1218, 1144, 1098, 1067 cm^{-1}; LRMS *m/z* (ESI+): Found: 945.0 (M+H), Calc.: 945.1; m.p. > 350 °C;

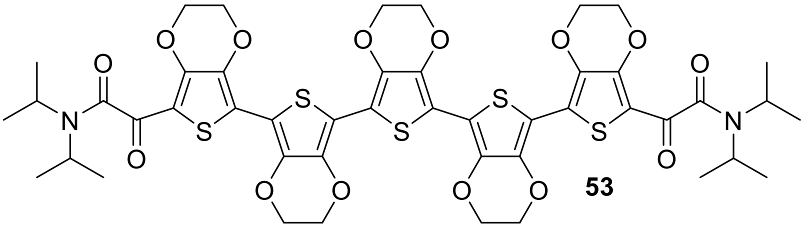

53

Run on 190 µmol scale with EDOT **1** and brominated diisopropylamine-EDOT dimer **67**. Column eluted with -4 % MeOH:DCM. Yield of 47 mg, 46 µmol (48%) as a purple solid. ^{1}H NMR (400 MHz, $CDCl_3$): δ = 4.22-4.54 (20H, m, -OC<u>H</u>$_2$-), 3.82-3.95 (2H, m, -C<u>H</u>Me$_2$), 3.50-3.60 (2H, m, -C<u>H</u>Me$_2$), 1.54 (12H, d, *J* = 6.8 Hz, -CH<u>Me</u>$_2$), 1.21 (12H, d, *J* = 7.0 Hz, -CH<u>Me</u>$_2$) ppm; HRMS *m/z* (ESI+): Found: 1013.1821 (M+H), Calc.: 1013.1787; m.p. > 350 °C;

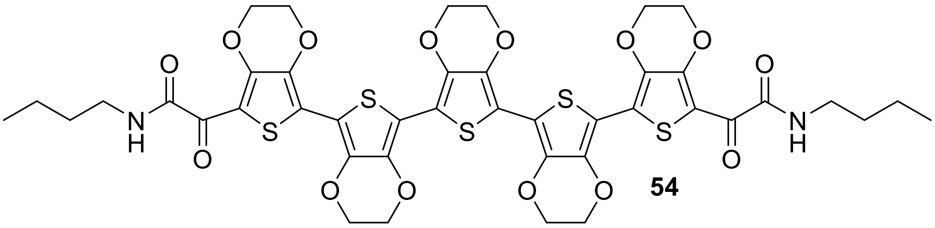

54

Run on 73 µmol scale with dibromo-EDOT **25** and *n*-butylamine-EDOT dimer **109**. Column eluted with 2-5 % MeOH:DCM. Yield of 12 mg, 12 µmol (34 %) as a purple solid. ^{1}H NMR (400 MHz, $CDCl_3$): δ = 7.46 (2H, t, *J* = 6.3 Hz, -N<u>H</u>), 4.21-4.57 (20H, m, -OC<u>H</u>$_2$-), 3.38 (4H, dt, J_1 = J_2 = 6.8 Hz, -NHC<u>H</u>$_2$-), 1.53-1.68 (4H, m, -NHCH$_2$C<u>H</u>$_2$-), 1.34-1.47 (4H, m, -C<u>H</u>$_2$CH$_3$), 0.96 (6H, t, *J* = 7.3 Hz, -C<u>H</u>$_3$) ppm; MS *m/z* (MALDI+): Found: 957.0 (M+H), Calc.: 957.1; m.p. > 350 °C;

Hexamer synthesis

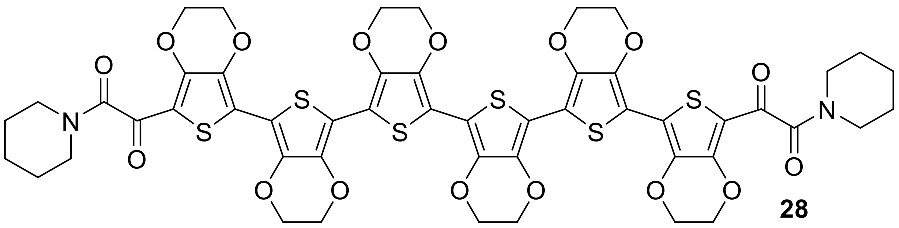

28

Run on 53 µmol scale with brominated piperidine-EDOT trimer **23** and piperidine-EDOT trimer **22**. Column eluted with 2-5 % MeOH:DCM. Yield of 21 mg, 18 µmol (35 %) as a purple solid. ^{1}H NMR (400 MHz, $CDCl_3$): δ = 4.13-4.61 (24H, s, -OC$\underline{H}_2$-), 3.56-3.74 (4H, m, -C$\underline{H}_2$N-), 3.33-3.53 (4H, m, -C$\underline{H}_2$N-), 1.65-1.78 (8H, m, -C$\underline{H}_2$C$\underline{H}_2$CH$_2$N-), 1.49-1.65 (4H, m, -C$\underline{H}_2$CH$_2$N-) ppm; IR ($υ_{max}$, solid): 2921, 2856, 1614, 1542, 1428, 1357, 1311, 1259, 1215, 1188, 1115, 1065 cm^{-1}; MS *m/z* (MALDI+): Found: 1120.8 (M+H), Calc.: 1121.1; m.p. > 350 °C;

Heptamer synthesis

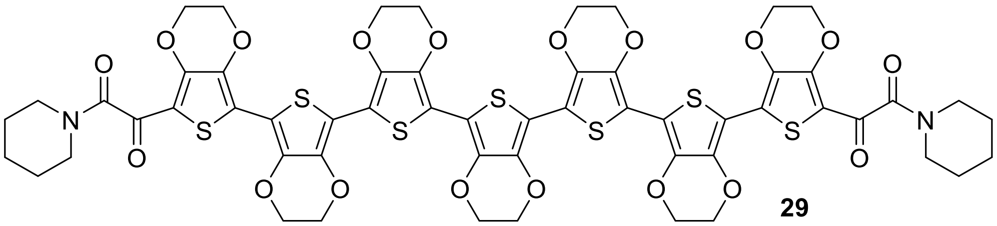

29

Run on 89 µmol scale with dibromo-EDOT **25** and piperidine-EDOT trimer **22**. Column eluted with 0-5 % MeOH:DCM. Yield of 46 mg, 37 µmol (42%) as a purple solid. ^{1}H NMR (400 MHz, $CDCl_3$): δ = 4.06-4.59 (28H, s, -OC$\underline{H}_2$-), 3.61-3.71 (4H, t, -C$\underline{H}_2$N-), 3.35-3.44 (4H, t, -C$\underline{H}_2$N-), 1.53-1.76 (12H, m, -C$\underline{H}_2$C$\underline{H}_2$CH$_2$N-) ppm; IR ($υ_{max}$, solid): 2921, 2852, 1727, 1626, 1468, 1437, 1361, 1313, 1282, 1253, 1221, 1117, 1072 cm^{-1}; MS *m/z* (MALDI+): Found: 1260.8 (M+H), Calc.: 1261.1; m.p. > 350 °C;

Oligomer manipulations

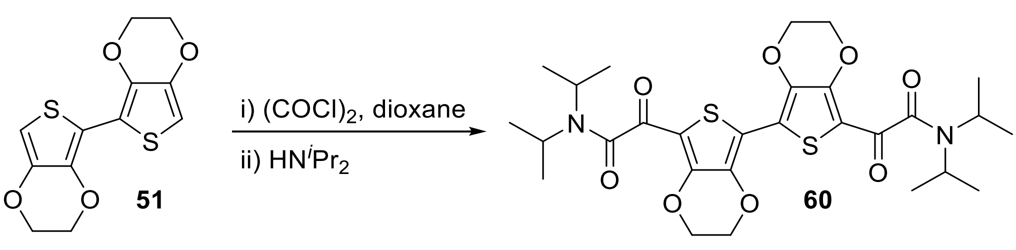

Oxalyl chloride (50 µL, 590 µmol) was added dropwise to a solution of di-EDOT **51** (10 mg, 35 µmol) in dioxane (2 mL) and heated to 100 ºC for 2 hrs. After cooling to rt, diisopropylamine (200 µL, 1425 µmol) was added and the mixture stirred for 20 min. The mixture was then diluted with DCM (20 mL) and the organics washed with water (10 mL), dried with $MgSO_4$, filtered and concentrated *in vacuo*. The residue was purified by flash column chromatography eluting with 70-100 % EtOAc:Hexane. Pure fractions were concentrated *in vacuo* to give the DP as a yellow solid. A yield of 12 mg, 21 µmol (60 %) was obtained. ^{1}H NMR (400 MHz, $CDCl_3$): δ = 4.41-4.46 (4H, m, -OC<u>H</u>$_2$-), 4.37-4.42 (4H, m, -OC<u>H</u>$_2$-), 3.83 (2H, sept, *J* = 6.6 Hz, -C<u>H</u>Me$_2$), 3.57 (2H, sept, *J* = 6.8 Hz, -C<u>H</u>Me$_2$), 1.53 (12H, d, *J* = 6.8 Hz, -CH<u>Me</u>$_2$), 1.22 (12H, d, *J* = 6.6 Hz, -CH<u>Me</u>$_2$) ppm; ^{13}C NMR (100 MHz, $CDCl_3$): δ = 181.97 (-<u>C</u>OCON-), 166.23 (-<u>C</u>ON-), 145.69 (Ar<u>C</u>3), 138.85 (Ar<u>C</u>4), 119.52 (Ar<u>C</u>$_\alpha$), 115.34 (Ar<u>C</u>$_\alpha$), 65.07 (-O<u>C</u>H$_2$-), 64.71 (-O<u>C</u>H$_2$-), 50.29 (-<u>C</u>HMe$_2$), 45.83 (-<u>C</u>HMe$_2$), 20.63 (-CH<u>Me</u>$_2$), 20.04 (-CH<u>Me</u>$_2$) ppm; IR (υ_{max}, solid): 2974, 1636, 1614, 1555, 1478, 1440, 1364, 1273, 1237, 1210, 1149, 1077 cm^{-1}; HRMS *m/z* (ESI+): Found: 593.1999 (M+H), Calc.: 593.1991; m.p. > 350 °C;

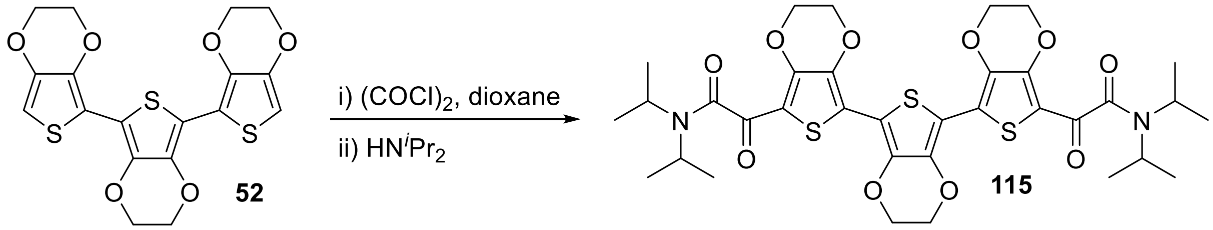

Oxalyl chloride (80 µL, 944 µmol) was added dropwise to a solution of tri-EDOT **52** (20 mg, 47 µmol) in dioxane (2 mL) and heated to 100 ºC for 2 hrs. After cooling to rt, diisopropylamine (400 µL, 2851 µmol) was added and the mixture stirred for 20 min. The mixture was then diluted with DCM (20 mL) and the organics washed with water (10 mL), dried with $MgSO_4$, filtered and concentrated *in vacuo*. The residue was purified by flash column chromatography eluting with 2 % NEt_3 in 60-100 % EtOAc:Hexane. Pure fractions were concentrated *in vacuo* to give the DP as an orange solid. A yield of 25 mg, 34 µmol (74 %) was obtained. ^{1}H NMR (400 MHz, $CDCl_3$): δ = 4.41-4.48 (8H, m, -OC<u>H</u>$_2$-), 4.35-4.41 (4H, m, -OC<u>H</u>$_2$-), 3.87 (2H, sept, *J* = 6.6 Hz, -C<u>H</u>Me$_2$), 3.55 (2H, sept, *J* = 6.8 Hz, -C<u>H</u>Me$_2$), 1.54 (12H, d, *J* = 6.8 Hz, -CH<u>Me</u>$_2$), 1.22 (12H, d, *J* = 6.6 Hz, -CH<u>Me</u>$_2$) ppm; ^{13}C NMR (100 MHz, $CDCl_3$): δ = 181.59 (-<u>C</u>OCON-), 166.56 (-<u>C</u>ON-), 145.99 (Ar<u>C</u>$_\alpha$), 139.59 (Ar<u>C</u>$_\alpha$), 136.55 (Ar<u>C</u>$_\alpha$), 121.56 (Ar<u>C</u>$_\alpha$), 113.48 (Ar<u>C</u>$_\alpha$), 110.82 (Ar<u>C</u>$_\alpha$), 65.18 (-O<u>C</u>H$_2$-), 65.07 (-O<u>C</u>H$_2$-), 64.68 (-O<u>C</u>H$_2$-), 50.25 (-<u>C</u>HMe$_2$), 45.74 (-<u>C</u>HMe$_2$), 20.62 (-CH<u>Me</u>$_2$), 20.07 (-CH<u>Me</u>$_2$) ppm; IR (υ_{max}, solid): 3361, 3297, 2925, 2852, 1634, 1547, 1470, 1424, 1358,

1297, 1268, 1255, 1235, 1209, 1090, 1070, 1042 cm^{-1}; HRMS *m/z* (ESI+): Found: 733.1931 (M+H), Calc.: 733.1923; m.p. > 350 °C;

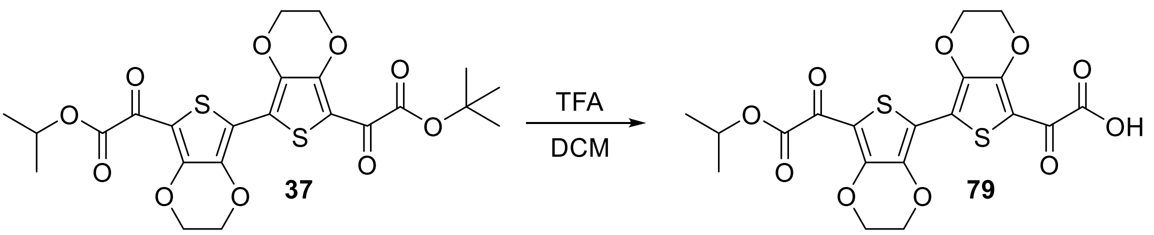

Trifluoroacetic acid (5 mL) was added to a solution of hetero-bifunctional EDOT-dimer **37** (200 mg, 382 μmol) in DCM (5 mL). After stirring for 2 hrs, complete cleavage of the *tert*-butyl ester was observed by LC-MS with no *iso*-propyl ester cleavage. The mixture was concentrated *in vacuo* and azeotroped with toluene (2 x 20 mL) to afford the DP as an orange solid which was used directly in further manipulations.

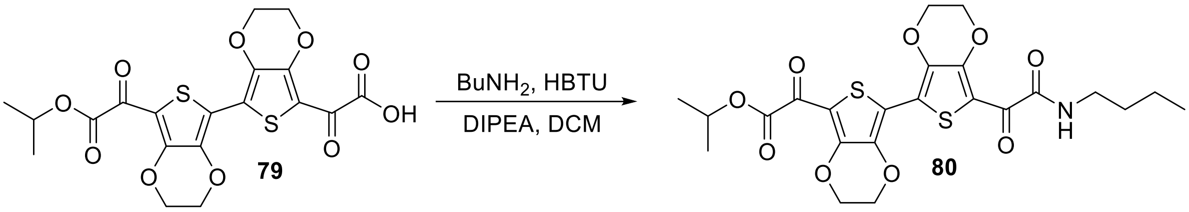

HBTU (162 mg, 427 μmol) and DIPEA (149 μL, 855 μmol) were added to a suspension of EDOT dimer **79** (100 mg, 213 μmol) in DCM (10 mL). After 5 min complete dissolution had occurred at which point *n*-butylamine (42 μL, 427 μmol) was added. After stirring for 18 hrs, the mixture was diluted with DCM (50 mL) and the organics washed with hydrochloric acid (1 M, 50 mL), dried with $MgSO_4$, filtered and concentrated *in vacuo*. The residue was purified by flash column chromatography eluting with 50-70 % EtOAc:Hexane. Pure fractions were concentrated *in vacuo* to give the DP as an orange-yellow solid. A yield of 40 mg, 76 μmol (36 %) was obtained. ^1H NMR (400 MHz, $CDCl_3$): 7.43 (1H, br t, *J* = 6.2 Hz, -N$\underline{H}$), 5.27 (1H, sept, *J* = 6.3 Hz, -C$\underline{H}$$Me_2$), 4.36-4.58 (8H, m, -OC$\underline{H}_2$-), 3.38 (2H, td, *J* = 6.9, 6.2 Hz, -C$\underline{H}_2$NH-), 1.54-1.67 (2H, m, -C$\underline{H}_2$$CH_2$NH-), 1.31-1.47 (8H, m, -C$\underline{H}_2$$CH_3$ and –CH$\underline{Me}_2$), 0.96 (3H, t, *J* = 7.3 Hz, -CH_2C$\underline{H}_3$) ppm; ^{13}C NMR (100 MHz, $CDCl_3$): δ = 176.08 (-$\underline{C}$OCO_2*i*Pr/-$\underline{C}$OCONHBu), 162.99 (-$\underline{C}O_2$*i*Pr), 161.50 (-$\underline{C}$ONHBu), 149.37 (Ar$\underline{C}_\beta$), 147.46 (Ar$\underline{C}_\beta$), 139.28 (Ar$\underline{C}_\beta$), 139.04 (Ar$\underline{C}_\beta$), 122.86 (Ar$\underline{C}_\alpha$), 120.99 (Ar$\underline{C}_\alpha$), 113.66 (Ar$\underline{C}_\alpha$), 110.83 (Ar$\underline{C}_\alpha$), 70.70 (-$\underline{C}$HMe_2), 65.52 (-O$\underline{C}H_2$-), 65.32 (-O$\underline{C}H_2$-), 64.72 (-O$\underline{C}H_2$-), 64.62 (-O$\underline{C}H_2$-), 39.25 (-$\underline{C}H_2$NH-), 31.30 (-$\underline{C}H_2$$CH_2$NH-), 21.62 (-CH$\underline{Me}_2$), 20.07 (-$\underline{C}H_2$$CH_3$), 13.72 (-$CH_2$$\underline{C}H_3$) ppm; IR ($\upsilon_{max}$, film): 2959, 2935, 2876, 1720, 1677, 1635, 1470, 1441, 1359,

1260, 1221, 1091, 1072 cm^{-1}; HRMS *m/z* (ESI+): Found: 524.1052 (M+H), Calc.: 524.1049; m.p. = 262-265 °C;

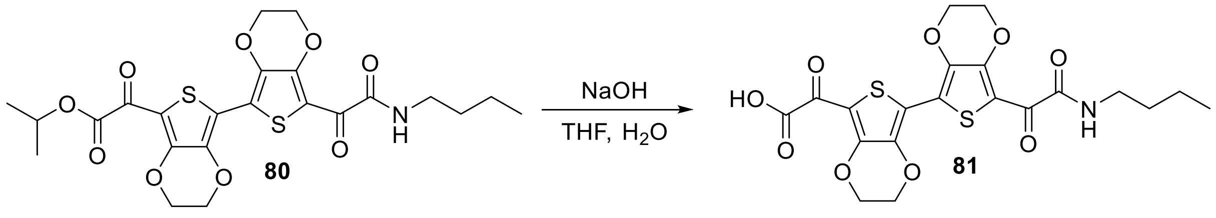

Sodium hydroxide (2 M, 3 mL) was added to a solution of hetero-bifunctional EDOT-dimer **80** (40 mg, 76 μmol) in THF (10 mL). After stirring for 2 hrs the mixture was diluted with DCM (100 mL) and washed with hydrochloric acid (1 M, 100 mL). The organics were dried with $MgSO_4$, filtered and concentrated *in vacuo* to afford the DP as an orange solid which was used directly in further manipulations.

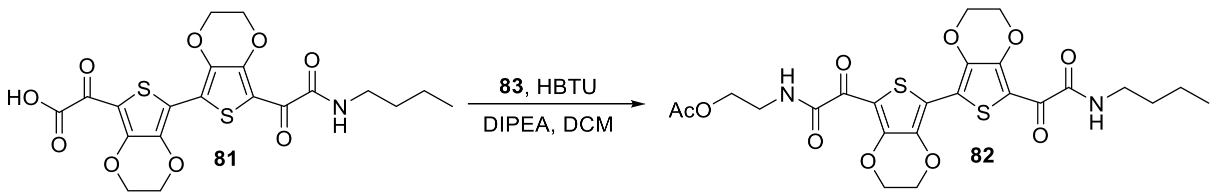

HBTU (58 mg, 152 μmol) and DIPEA (66 μL, 380 μmol) were added to a suspension of EDOT dimer **81** (36 mg, 76 μmol) in DCM (10 mL). After 5 min complete dissolution had occurred at which point amine **83** (21 mg, 152 μmol) was added. After stirring for 4 hrs, the mixture was diluted with DCM (50 mL) and the organics washed with hydrochloric acid (1 M, 50 mL) and sat. $NaHCO_3$ (50 mL), dried with $MgSO_4$, filtered and concentrated *in vacuo*. The residue was purified by flash column chromatography eluting with 50-70 % EtOAc:Hexane. Pure fractions were concentrated *in vacuo* to give the DP as an orange-yellow solid. A yield of 41 mg, 72 μmol (95 %) was obtained. ^{1}H NMR (400 MHz, $CDCl_3$): δ = 7.67 (1H, br t, J = 6.2 Hz, -N$\underline{H}$EtOAc), 7.43 (1H, br t, J = 6.4 Hz, -N$\underline{H}$Bu), 4.44-4.56 (8H, m, -OC$\underline{H}_2$-), 4.25 (2H, t, J = 5.3 Hz, -C$\underline{H}_2$OAc), 3.66 (2H, dt, J = 6.2, 5.3 Hz, -C$\underline{H}_2$CH$_2$OAc), 3.39 (2H, dt, J_1 = J_2 = 7.0 Hz, -C$\underline{H}_2$CH$_2$CH$_2$CH$_3$), 2.11 (3H, s, -O$\underline{Ac}$), 1.54-1.67 (2H, m, -C$\underline{H}_2$CH$_2$CH$_3$), 1.35-1.44 (2H, m, -C$\underline{H}_2$CH$_3$), 0.96 (3H, t, J = 7.3 Hz, -CH$_2$C$\underline{H}_3$) ppm; ^{13}C NMR (100 MHz, $CDCl_3$): δ = 176.11 (-$\underline{C}$OCONHR), 175.51 (-$\underline{C}$OCONHR), 170.8 (-O$\underline{C}$OCH$_3$), 161.80 (-$\underline{C}$ONHR), 161.52 (-$\underline{C}$ONHR), 149.61 (Ar$\underline{C}_\beta$), 149.38 (Ar$\underline{C}_\beta$), 139.33 (Ar$\underline{C}_\beta$), 139.29 (Ar$\underline{C}_\beta$), 123.14 (Ar$\underline{C}_\alpha$), 111.02 (Ar$\underline{C}_\alpha$), 110.75 (Ar$\underline{C}_\alpha$), 65.55 (-O$\underline{C}$H$_2$-), 65.51 (-O$\underline{C}$H$_2$-), 64.63 (-O$\underline{C}$H$_2$-), 62.60 (-$\underline{C}$H$_2$OAc), 39.25 (-$\underline{C}$H$_2$NH-), 38.50 (-$\underline{C}$H$_2$CH$_2$OAc), 31.31 (-$\underline{C}$H$_2$CH$_2$NH-), 20.81 (-CO$\underline{C}$H$_3$), 20.07 (-$\underline{C}$H$_2$CH$_3$), 13.70 (-CH$_2\underline{C}$H$_3$) ppm; IR (υ_{max}, solid): 3306, 2926,

2855, 1736, 1659, 1624, 1523, 1477, 1427, 1363, 1293, 1228, 1139, 1084, 1054, 1023 cm^{-1}; HRMS *m/z* (ESI+): Found: 567.1115 (M+H), Calc.: 567.1110; m.p. > 350 ˚C;

3,4-Dimethoxythiophene functionalisation

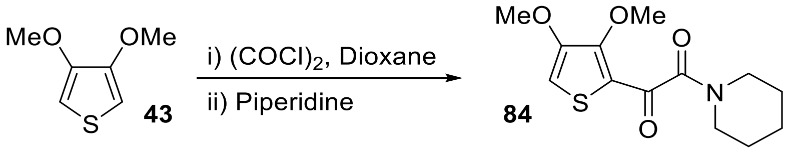

Oxalyl chloride (1.42 mL, 16.8 mmol) was added drop-wise to a solution of 3,4-dimethoxythiophene **43** (2 mL, 16.8 mmol) in dioxane (30 mL). The mixture was heated to 100 ºC for 90 min then allowed to cool to room temperature. Piperidine (8.3 mL, 84 mmol) was then added and the mixture stirred for 3 hrs. After this time the mixture was diluted with DCM (200 mL), and the organics washed with water (100 mL) and brine (100 mL), dried with $MgSO_4$, filtered and concentrated *in vacuo*. The residue was purified by flash column chromatography eluting with 20-50 % EtOAc:Hexane. Pure fractions were concentrated *in vacuo* to give the DP as a cream solid upon standing. A yield of 2.9 g, 10.2 mmol (61 %) was obtained. ^{1}H NMR (400 MHz, $CDCl_3$): δ = 6.72 (1H, s, ArH5), 3.96 (3H, s, ArC4-OMe), 3.86 (3H, s, ArC3-OMe), 3.62 (2H, dd, J = 6.3, 4.1 Hz, -NCH_2-), 3.26-3.34 (2H, m, -NCH_2-), 1.61-1.71 (4H, m, -$CH_2CH_2CH_2N$-), 1.54-1.60 (2H, m, -CH_2CH_2N-) ppm; ^{13}C NMR (100 MHz, $CDCl_3$): δ = 183.89 (-COCON-), 165.47 (-CON-), 152.32 (ArC4), 150.61 (ArC3), 123.15 (ArC2), 107.63 (ArC5), 61.00 (ArC4-OMe), 57.67 (ArC3-OMe), 46.86 (-CH_2N-), 42.02 (-CH_2N-), 25.71 (-$CH_2CH_2CH_2N$-), 25.10 (-$CH_2CH_2CH_2N$-), 24.49 (-$CH_2CH_2CH_2N$-) ppm; IR ($υ_{max}$, solid): 3078, 2934, 2865, 1631, 1491, 1453, 1427, 1398, 1370, 1301, 1281, 1264, 1254, 1240, 1213, 1145, 1124, 1052 cm^{-1}; HRMS *m/z* (ESI+): Found: 284.0951 (M+H), Calc.: 284.0957; m.p. = 93-94 °C;

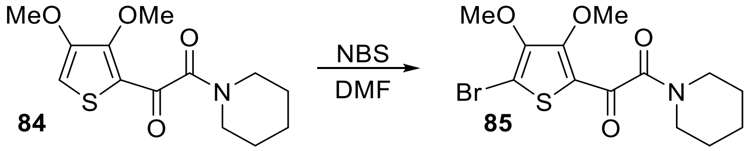

N-Bromosuccinimide (1.13 g, 6.4 mmol) was added in the dark to a solution of dimethoxythiophene derivative **84** (1.5 g, 5.3 mmol) in DMF (8 mL) (N.b. The reaction was initially run in a mixture of THF and AcOH as described above. However the reaction was extremely sluggish, even after addition of a further equivalent of NBS). After stirring for 1 hr the mixture was diluted with DCM (150 mL) and the

organics washed with water (100 mL) and sat. $NaHCO_3$ (2 x 100 mL), dried with $MgSO_4$, filtered and concentrated *in vacuo*. The residue was purified by flash column chromatography eluting with 20-50 % EtOAc:Hexane. Pure fractions were concentrated *in vacuo* and the residue dissolved in diethyl ether (100 mL). The organics were washed with brine (2 x 100 mL), dried with $MgSO_4$, filtered and concentrated *in vacuo* to give the DP as a light yellow oil. A yield of 1.8 g, 5.0 mmol (94 %) was obtained. ^{1}H NMR (400 MHz, $CDCl_3$): δ = 4.00 (3H, s, ArC4-OMe), 3.94 (3H, s, ArC3-OMe), 3.63 (2H, dd, *J* = 6.3, 4.3 Hz, -NCH$_2$-), 3.27-3.35 (2H, m, -NCH$_2$-), 1.62-1.76 (4H, m, -CH$_2$CH$_2$CH$_2$N-), 1.54-1.62 (2H, m, -CH$_2$CH$_2$N-) ppm; ^{13}C NMR (100 MHz, $CDCl_3$): δ = 182.23 (-COCON-), 164.97 (-CON-), 154.66 (ArC4), 148.56 (ArC3), 122.81 (ArC2), 112.39 (ArC5), 65.85 (ArC4-OMe), 61.18 (ArC3-OMe), 46.93 (-CH$_2$N-), 42.14 (-CH$_2$N-), 25.84 (-CH$_2$CH$_2$CH$_2$N-), 25.14 (-CH$_2$CH$_2$CH$_2$N-), 24.47 (-CH$_2$CH$_2$CH$_2$N-) ppm; IR (υ$_{max}$, oil): 2939, 2857, 1636, 1486, 1444, 1417, 1374, 1295, 1249, 1228, 1120, 1049, 1000 cm^{-1}; HRMS *m/z* (ESI+): Found: 362.0070/364.0040 (M+H), Calc.: 362.0062/364.0042;

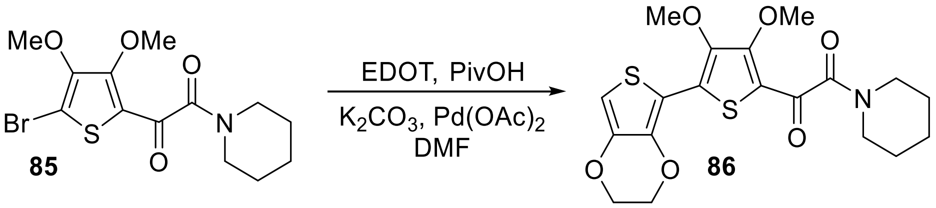

Brominated-dimethoxy thiophene **85** (0.5 g, 1.76 mmol), pivalic acid (90 mg, 0.88 mmol), palladium (II) acetate (20 mg, 0.09 mmol) and potassium carbonate (2.4 g, 17.6 mmol) were charged under nitrogen. Dry DMF (7 mL) and EDOT **1** (0.75 mL, 7.07 mmol) were then added and the mixture heated to 90 ºC for 1 hr. After cooling to rt the mixture was diluted with DCM (150 mL) and washed with water (2 x 100 mL) and brine (100 mL). The organics were dried with $MgSO_4$, filtered and concentrated *in vacuo*. The residue was purified by flash column chromatography eluting with 2 % NEt_3 in 20-70 % EtOAc:Hexane. Pure fractions were concentrated *in vacuo* and the residue dissolved in diethyl ether (100 mL). The organics were washed with brine (2 x 100 mL), dried with $MgSO_4$, filtered and concentrated *in vacuo* to give the DP as a yellow oil. A yield of 490 mg, 1.35 mmol (77 %) was obtained. ^{1}H NMR (400 MHz, $CDCl_3$): δ = 6.47 (1H, s, ArH10), 4.36-4.43 (2H, m, -OCH$_2$-), 4.21-4.30 (2H, m, -OCH$_2$-), 4.01 (3H, s, ArC4-OMe), 3.90 (3H, s, ArC3-OMe), 3.65 (2H, dd, *J* = 6.3, 4.1 Hz, -NCH$_2$-), 3.29-3.39 (2H, m, -NCH$_2$-), 1.62-1.73 (4H, m, -CH$_2$CH$_2$CH$_2$N-), 1.55-1.62 (2H, m, -CH$_2$CH$_2$N-) ppm; ^{13}C NMR (100 MHz, $CDCl_3$): δ = 183.07 (-COCON-), 165.68 (-CON-), 155.50 (ArC4), 144.10 (ArC3), 141.36 (ArC8), 140.72 (ArC9), 131.18 (ArC5), 118.96 (ArC2), 108.78 (ArC7), 102.12 (ArC10), 65.34 (ArC9-OCH$_2$-), 64.52 (ArC8-

O<u>C</u>H$_2$-), 61.01 (ArC4-O<u>Me</u>), 60.55 (ArC3-O<u>Me</u>), 47.02 (-C<u>H</u>$_2$N-), 42.07 (-C<u>H</u>$_2$N-), 25.84 (-<u>C</u>H$_2$<u>C</u>H$_2$CH$_2$N-), 25.17 (-<u>C</u>H$_2$<u>C</u>H$_2$CH$_2$N-), 24.55 (-<u>C</u>H$_2$<u>C</u>H$_2$CH$_2$N-) ppm; IR ($υ_{max}$, solid): 2926, 2856, 2228, 1638, 1595, 1572, 1533, 1500, 1469, 1432, 1395, 1364, 1324, 1282, 1262, 1248, 1221, 1183, 1150, 1137, 1114, 1078, 1051, 1015 cm^{-1}; HRMS *m/z* (ESI+): Found: 424.0880 (M+H), Calc.: 424.0889; m.p. = 82-87 °C;

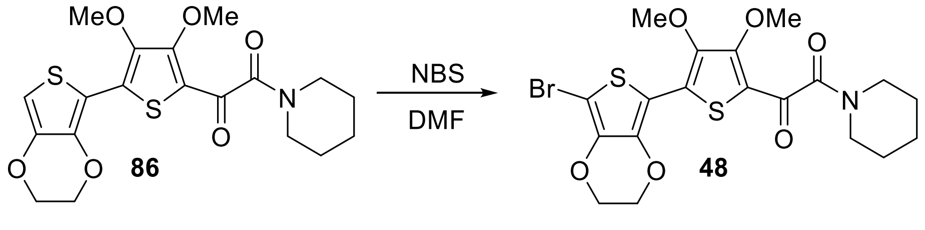

N-Bromosuccinimide (227 mg, 1.28 mmol) was added to a solution of EDOT-dimethoxythiophene dimer **86** (450 mg, 1.06 mmol) in DMF (5 mL) in the dark. After 1 hr the mixture was diluted with diethyl ether (100 mL) and the organics washed with water (100 mL) and brine (2 x 100 mL), dried with $MgSO_4$, filtered and concentrated *in vacuo*. The product was sufficiently pure for further applications. An analytical sample was obtained via column chromatography eluting with 2 % NEt_3 in 50 % EtOAc:Hexane. A yield of 410 mg, 0.82 mmol (64 %) was obtained as a yellow solid. ^{1}H NMR (400 MHz, $CDCl_3$): δ = 4.39-4.43 (2H, m, -OC<u>H</u>$_2$-), 4.34-4.38 (2H, m, -OC<u>H</u>$_2$-), 4.02 (3H, s, ArC4-O<u>Me</u>), 3.92 (3H, s, ArC3-O<u>Me</u>), 3.66 (2H, t, *J* = 5.1 Hz, -NC<u>H</u>$_2$-), 3.32-3.40 (2H, m, -NC<u>H</u>$_2$-), 1.63-1.74 (4H, m, -C<u>H</u>$_2$C<u>H</u>$_2$CH$_2$N-), 1.56-1.63 (2H, m, -C<u>H</u>$_2$CH$_2$N-) ppm; ^{13}C NMR (100 MHz, $CDCl_3$): δ = 183.05 (-<u>C</u>OCON-), 165.58 (-<u>C</u>ON-), 155.26 (Ar<u>C</u>4), 144.18 (Ar<u>C</u>3), 139.75 (Ar<u>C</u>$_β$), 139.70 (Ar<u>C</u>$_β$), 130.16 (Ar<u>C</u>5), 119.33 (Ar<u>C</u>2), 109.05 (Ar<u>C</u>7), 91.26 (Ar<u>C</u>10), 65.28 (ArC9-O<u>C</u>H$_2$-), 64.99 (ArC8-O<u>C</u>H$_2$-), 61.09 (ArC4-O<u>Me</u>), 60.57 (ArC3-O<u>Me</u>), 47.03 (-C<u>H</u>$_2$N-), 42.11 (-C<u>H</u>$_2$N-), 25.87 (-<u>C</u>H$_2$<u>C</u>H$_2$CH$_2$N-), 25.18 (-<u>C</u>H$_2$<u>C</u>H$_2$CH$_2$N-), 24.55 (-<u>C</u>H$_2$<u>C</u>H$_2$CH$_2$N-) ppm; IR ($υ_{max}$, solid): 2937, 2844, 1651, 1606, 1540, 1429, 1395, 1355, 1318, 1264, 1218, 1087, 1052 cm^{-1}; HRMS *m/z* (ESI+): Found: 501.9980/503.9965 (M+H), Calc.: 501.9994/503.9974; m.p. = 222-225 °C;

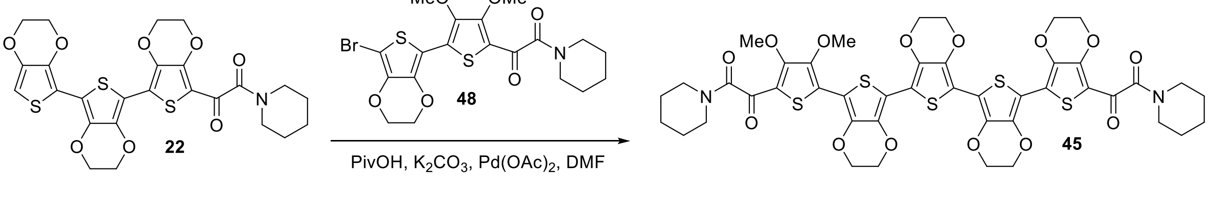

Piperidine-EDOT trimer **22** (80 mg, 142 μmol), brominated EDOT-dimethoxythiophene dimer **48** (50 mg, 159 μmol), pivalic acid (7 mg, 71 μmol), palladium (II) acetate (3 mg, 14 μmol) and potassium

carbonate (195 mg, 1420 μmol) were charged under nitrogen. Dry DMF (3 mL) was then added and the mixture heated to 130 ºC for 1 hr. After cooling to rt the mixture was diluted with DCM (50 mL) and washed with water (2 x 50 mL) and brine (50 mL). The organics were dried with $MgSO_4$, filtered and concentrated *in vacuo*. The residue was purified by flash column chromatography eluting with 2 % MeOH:DCM. Pure fractions were concentrated *in vacuo* and the residue triturated in diethyl ether (2 x 50 mL) to give the DP as a purple solid. A yield of 51 mg, 52 μmol (37 %) was obtained. ^{1}H NMR (400 MHz, $CDCl_3$): δ = 4.26-4.58 (16H, m, -OC<u>H</u>$_2$-), 4.04 (3H, s, -O<u>Me</u>), 3.96 (3H, s, -O<u>Me</u>), 3.63-3.71 (4H, m, -C<u>H</u>$_2$N-), 3.34-3.44 (2H, m, -C<u>H</u>$_2$N-), 1.65-1.74 (8H, m, -C<u>H</u>$_2$C<u>H</u>$_2$CH$_2$N-), 1.56-1.64 (4H, m, -C<u>H</u>$_2$CH$_2$N-) ppm; IR ($υ_{max}$, solid): 2927, 2857, 1622, 1434, 1394, 1359, 1316, 1218, 1151, 1068, 1057, 1013 cm^{-1}; HRMS *m/z* (ESI+): Found: 983.1295 (M+H), Calc.: 983.1318; m.p. > 350 °C;

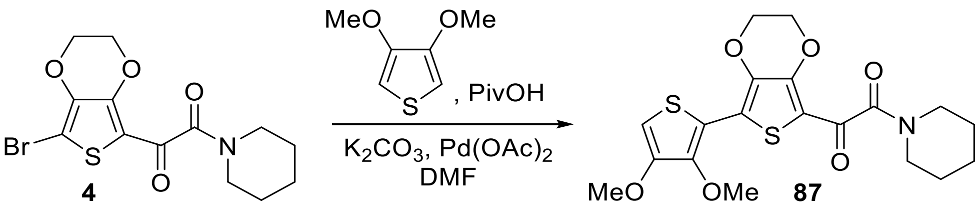

Brominated piperidine-EDOT monomer **4** (0.5 g, 1.4 mmol), pivalic acid (71 mg, 0.7 mmol), palladium (II) acetate (16 mg, 0.07 mmol) and potassium carbonate (1.9 g, 14 mmol) were charged under nitrogen. Dry DMF (5 mL) and 3,4-dimethoxythiophene **43** (0.67 mL, 5.6 mmol) were then added and the mixture heated to 130 ºC for 1 hr. After cooling to rt the mixture was diluted with DCM (150 mL) and washed with water (2 x 100 mL) and brine (100 mL). The organics were dried with $MgSO_4$, filtered and concentrated *in vacuo*. The residue was purified by flash column chromatography eluting with 2 % NEt_3 in 50-80 % EtOAc:Hexane. Pure fractions were concentrated *in vacuo* and the residue triturated in diethyl ether (2 x 100 mL) to give the DP as an orange solid. A yield of 330 mg, 0.78 mmol (56 %) was obtained. ^{1}H NMR (400 MHz, $CDCl_3$): δ = 6.32 (1H, s, Ar<u>H</u>10), 4.41 (4H, m, -OC<u>H</u>$_2$-), 3.99 (3H, s, ArC9-O<u>Me</u>), 3.88 (3H, s, ArC8-O<u>Me</u>), 3.65 (2H, t, *J* = 5.2 Hz, -NC<u>H</u>$_2$-), 3.34-3.43 (2H, m, -NC<u>H</u>$_2$-), 1.62-1.73 (4H, m, -C<u>H</u>$_2$C<u>H</u>$_2$CH$_2$N-), 1.54-1.62 (2H, m, -C<u>H</u>$_2$CH$_2$N-) ppm; ^{13}C NMR (100 MHz, $CDCl_3$): δ = 182.18 (-<u>C</u>OCON-), 165.53 (-<u>C</u>ON-), 150.53 (Ar<u>C</u>8), 146.46 (Ar<u>C</u>$_β$), 145.13 (Ar<u>C</u>9), 136.85 (Ar<u>C</u>$_β$), 122.56 (Ar<u>C</u>5), 116.42 (Ar<u>C</u>7), 112.80 (Ar<u>C</u>2), 98.09 (Ar<u>C</u>10), 65.48 (-O<u>C</u>H$_2$-), 64.51 (-O<u>C</u>H$_2$-), 60.13 (ArC9-O<u>Me</u>), 57.40 (ArC8-O<u>Me</u>), 47.02 (-C<u>H</u>$_2$N-), 42.15 (-C<u>H</u>$_2$N-), 26.03 (-<u>C</u>H$_2$CH$_2$CH$_2$N-), 25.25 (-<u>C</u>H$_2$<u>C</u>H$_2$CH$_2$N-), 24.55 (-<u>C</u>H$_2$<u>C</u>H$_2$CH$_2$N-) ppm; IR ($υ_{max}$, solid): 3107, 2931, 1636, 1605, 1548, 1504,

1471, 1445, 1403, 1363, 1314, 1262, 1251, 1224, 1208, 11187, 1091, 1041, 1003 cm^{-1}; HRMS *m/z* (ESI+): Found: 424.0877 (M+H), Calc.: 424.0889; m.p. = 214-218 °C;

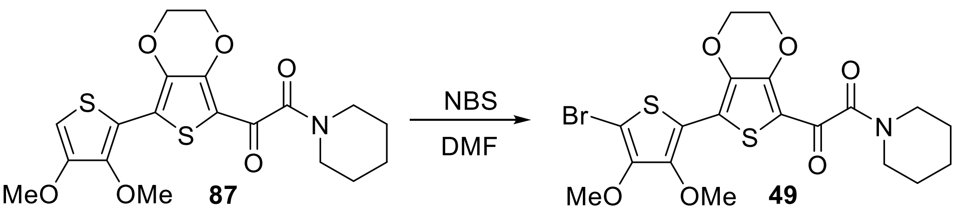

N-Bromosuccinimide (151 mg, 0.85 mmol) was added to a solution of dimethoxythiophene-EDOT dimer **87** (300 mg, 0.71 mmol) in DMF (3 mL) in the dark. After 1 hr the mixture was diluted with DCM (100 mL) and the organics washed with water (100 mL) and brine (2 x 100 mL), dried with $MgSO_4$, filtered and concentrated *in vacuo*. The product was sufficiently pure for further applications. An analytical sample was obtained via column chromatography eluting with 2 % NEt_3 in 60-90 % EtOAc:Hexane. A yield of 411 mg, 0.82 mmol (96 %) was obtained as a yellow oil. ^{1}H NMR (400 MHz, $CDCl_3$): δ = 4.34-4.43 (4H, m, -OC$\underline{H}_2$-), 3.96 (3H, s, -O$\underline{Me}$), 3.93 (3H, s, -O$\underline{Me}$), 3.61 (2H, t, *J* = 5.3 Hz, -NC$\underline{H}_2$-), 3.31-3.39 (2H, m, -NC$\underline{H}_2$-), 1.60-1.72 (4H, m, -C$\underline{H}_2$C$\underline{H}_2$CH$_2$N-), 1.51-1.59 (2H, m, -C$\underline{H}_2$CH$_2$N-) ppm; ^{13}C NMR (100 MHz, $CDCl_3$): δ = 182.13 (-$\underline{C}$OCON-), 165.37 (-$\underline{C}$ON-), 148.23 (Ar$\underline{C}_\beta$), 146.84 (Ar$\underline{C}_\beta$), 146.37 (Ar$\underline{C}_\beta$), 136.88 (Ar$\underline{C}_\beta$), 121.35 (Ar$\underline{C}_\alpha$), 116.66 (Ar$\underline{C}_\alpha$), 112.81 (Ar$\underline{C}_\alpha$), 99.82 (Ar$\underline{C}$10), 65.53 (-O$\underline{C}$H$_2$-), 64.63 (-O$\underline{C}$H$_2$-), 60.92 (-O$\underline{Me}$), 60.39 (-O$\underline{Me}$), 46.98 (-C$\underline{H}_2$N-), 42.13 (-C$\underline{H}_2$N-), 26.02 (-$\underline{C}$H$_2\underline{C}$H$_2$CH$_2$N-), 25.22 (-$\underline{C}$H$_2\underline{C}$H$_2$CH$_2$N-), 24.49 (-$\underline{C}$H$_2\underline{C}$H$_2$CH$_2$N-) ppm; IR (υ_{max}, solid): 2935, 2852, 1651, 1604, 1550, 1504, 1472, 1441, 1361, 1312, 1281, 1263, 1221, 1113, 1089, 1047, 1009 cm^{-1}; HRMS *m/z* (ESI+): Found: 501.9970/503.9957 (M+H), Calc.: 501.9994/503.9974; m.p. = 206-211 °C;

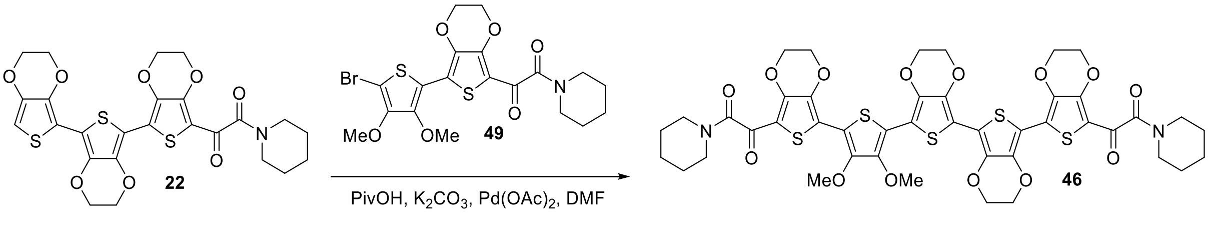

Piperidine-EDOT trimer **22** (50 mg, 89 µmol), brominated dimethoxythiophene-EDOT dimer **49** (50 mg, 100 µmol), pivalic acid (5 mg, 44 µmol), palladium (**II**) acetate (2 mg, 9 µmol) and potassium carbonate (123 mg, 890 µmol) were charged under nitrogen. Dry DMF (3 mL) was then added and the mixture heated to 130 ºC for 1.5 hrs. After cooling to rt the mixture was diluted with DCM (50 mL) and washed with water (2 x 50 mL) and brine (50 mL). The organics were dried with $MgSO_4$, filtered and

concentrated *in vacuo*. The residue was purified by flash column chromatography eluting with 2-5 % MeOH:DCM. Pure fractions were concentrated *in vacuo* and the residue triturated in diethyl ether (2 x 50 mL) to give the DP as a purple solid. A yield of 55 mg, 56 µmol (63 %) was obtained. ^{1}H NMR (400 MHz, $CDCl_3$): δ = 4.33-4.54 (16H, m, -OC<u>H</u>$_2$-), 3.94-4.10 (6H, m, -O<u>Me</u>), 3.62-3.70 (4H, m, -C<u>H</u>$_2$N-), 3.36-3.46 (2H, m, -C<u>H</u>$_2$N-), 1.63-1.75 (8H, m, -C<u>H</u>$_2$C<u>H</u>$_2$CH$_2$N-), 1.55-1.63 (4H, m, -C<u>H</u>$_2$CH$_2$N-) ppm; IR ($υ_{max}$, solid): 2924, 2872, 2855, 1732, 1634, 1601, 1468, 1427, 1359, 1248, 1217, 1138, 1117, 1066, 1021 cm^{-1}; HRMS *m/z* (ESI+): Found: 983.1321 (M+H), Calc.: 983.1318; m.p. > 350 °C;

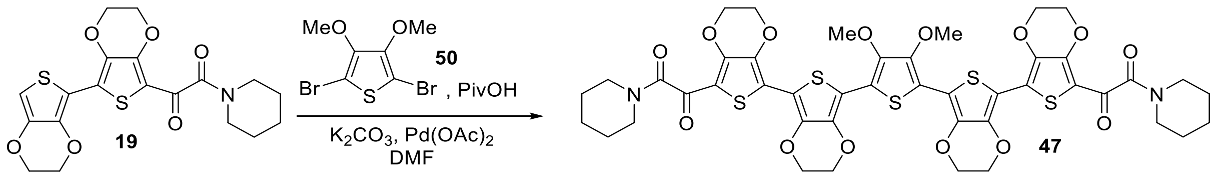

Piperidine-EDOT dimer **19** (40 mg, 95 µmol), pivalic acid (5 mg, 48 µmol), palladium (**II**) acetate (2 mg, 10 µmol) and potassium carbonate (131 mg, 950 µmol) were charged under nitrogen. Dry DMF (1.5 mL) and 2,5-dibromo-3,4-dimethoxythiophene **50** (14 mg, 48 µmol) were then added and the mixture heated to 130 ºC for 1 hr. After cooling to rt the mixture was diluted with DCM (50 mL) and washed with water (2 x 50 mL) and brine (50 mL). The organics were dried with $MgSO_4$, filtered and concentrated *in vacuo*. The residue was purified by flash column chromatography eluting with 1-5 % MeOH:DCM. Pure fractions were concentrated *in vacuo* and the residue triturated in diethyl ether (2 x 50 mL) to give the DP as a purple solid. A yield of 28 mg, 28 µmol (59 %) was obtained. ^{1}H NMR (400 MHz, $CDCl_3$): δ = 4.33-4.54 (16H, m, -OC<u>H</u>$_2$-), 4.02 (6H, s, ArC4-O<u>Me</u>), 3.65 (2H, t, *J* = 6.0 Hz, -NC<u>H</u>$_2$-), 3.40 (2H, t, *J* = 5.5 Hz, -NC<u>H</u>$_2$-), 1.62-1.75 (8H, m, -C<u>H</u>$_2$C<u>H</u>$_2$CH$_2$N-), 1.54-1.62 (4H, m, -C<u>H</u>$_2$CH$_2$N-) ppm; IR ($υ_{max}$, solid): 2931, 2854, 1650, 1602, 1540, 1488, 1463, 1421, 1358, 1309, 1249, 1217, 1135, 1116, 1067, 1022 cm^{-1}; HRMS *m/z* (ESI+): Found: 983.1467 (M+H), Calc.: 983.1466; m.p. > 350 °C;

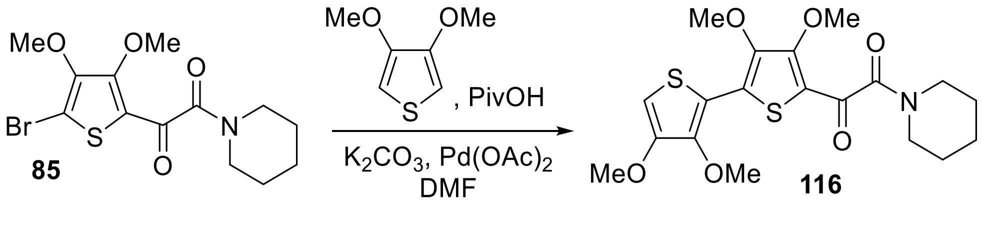

Brominated piperidine-DMT monomer **85** (400 mg, 1.1 mmol), pivalic acid (56 mg, 0.55 mmol), palladium (II) acetate (12 mg, 0.06 mmol) and potassium carbonate (1.5 g, 11 mmol) were charged under nitrogen. Dry DMF (5 mL) and 3,4-dimethoxythiophene **45** (523 µL, 4.4 mmol) were then added

and the mixture heated to 90 ºC for 1 hr. After cooling to rt the mixture was diluted with DCM (150 mL) and washed with water (2 x 100 mL) and brine (100 mL). The organics were dried with $MgSO_4$, filtered and concentrated *in vacuo*. The residue was purified by flash column chromatography eluting with 50 % EtOAc:Hexane. Pure fractions were concentrated *in vacuo* and the residue triturated in diethyl ether (2 x 100 mL) to give the DP as a yellow oil. A yield of 170 mg, 0.40 mmol (36 %) was obtained. The product was utilised directly in the subsequent formation of penta-DMT construct **57** without further purification or analysis.

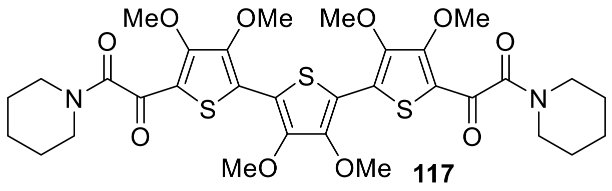

117 was obtained as a side product from the above reaction for the synthesis of **116**. The product was obtained as an orange solid. A yield of 110 mg, 156 µmol (28 %) was obtained. ^1H NMR (400 MHz, $CDCl_3$): δ = 4.00-4.01 (12H, m, $-OC\underline{H}_3$), 3.96 (6H, ArC8-$OC\underline{H}_3$), 3.65 (4H, t, J = 5.1 Hz, $-NC\underline{H}_2-$), 3.29-3.42 (4H, m, $-NC\underline{H}_2-$), 1.64-1.75 (8H, m, $-NCH_2C\underline{H}_2C\underline{H}_2-$), 1.54-1.64 (4H, m, $-NCH_2C\underline{H}_2-$) ppm; ^{13}C NMR (100 MHz, $CDCl_3$): δ = 183.27 ($-\underline{C}OCONR_2$), 165.50 ($-\underline{C}ONR_2$), 155.30 ($Ar\underline{C}_\beta$), 148.18 ($Ar\underline{C}_\beta$), 145.43 ($Ar\underline{C}_\beta$), 129.62 ($Ar\underline{C}_\alpha$), 120.75 ($Ar\underline{C}_\alpha$), 118.27 ($Ar\underline{C}_\alpha$), 109.19 ($Ar\underline{C}_\alpha$), 61.11 ($-O\underline{C}H_3$), 60.46 (ArC8-$O\underline{C}H_3$), 60.20 ($-O\underline{C}H_3$), 47.02 ($-N\underline{C}H_2-$), 42.11 ($-N\underline{C}H_2-$), 25.86 ($-NCH_2\underline{C}H_2-$), 25.17 ($-NCH_2\underline{C}H_2\underline{C}H_2-$), 24.53 ($-NCH_2\underline{C}H_2\underline{C}H_2-$) ppm; IR ($\upsilon_{max}$, solid): 2934, 2852, 1626, 1466, 1441, 1390, 1321, 1283, 1250, 1227, 1194, 1110, 1015 cm^{-1}; HRMS *m/z* (ESI+): Found: 743.1775 (M+H), Calc.: 743.1767; m.p. = 115-120 °C;

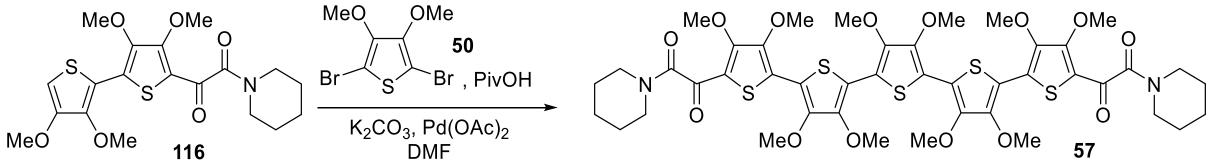

Piperidine-DMT dimer **116** (170 mg, 400 µmol), pivalic acid (20 mg, 200 µmol), palladium (**II**) acetate (9 mg, 40 µmol) and potassium carbonate (552 mg, 4 mmol) were charged under nitrogen. Dry DMF (3 mL) and 2,5-dibromo-3,4-dimethoxythiophene **50** (84 mg, 280 µmol) were then added and the mixture heated to 90 ºC for 1 hr. After cooling to rt the mixture was diluted with DCM (100 mL) and washed with water (2 x 75 mL) and brine (75 mL). The organics were dried with $MgSO_4$, filtered and concentrated *in vacuo*. The residue was purified by flash column chromatography eluting with 50-100 % EtOAc:Hexane. Pure fractions were concentrated *in vacuo* and the residue triturated in water (2 x 80 mL) to give the

DP as a red solid. A yield of 98 mg, 99 µmol (49 %) was obtained. ^{1}H NMR (400 MHz, $CDCl_3$): δ = 4.01-4.07 (24H, m, -OC$\underline{H}_3$), 3.98 (6H, s, -OC$\underline{H}_3$), 3.65-3.71 (4H, m, -NC$\underline{H}_2$-), 3.38 (4H, t, *J* = 5.3 Hz, -NC$\underline{H}_2$-), 1.65-1.75 (8H, m, -NCH$_2$C$\underline{H}_2$C$\underline{H}_2$-), 1.57-1.65 (4H, m, -NCH$_2$C$\underline{H}_2$-) ppm; IR ($υ_{max}$, solid): 2932, 2851, 1624, 1462, 1392, 1318, 1281, 1256, 1221, 1194, 1104, 1007 cm^{-1}; HRMS *m/z* (ESI+): Found: 991.1965 (M+H), Calc.: 991.1944; m.p. = 220-224 °C;

3,4-Propylenedioxythiophene functionalisation

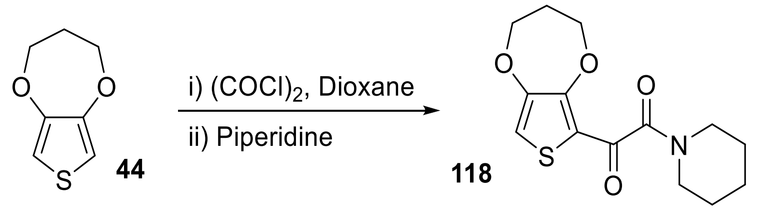

Oxalyl chloride (163 µL, 1.92 mmol) was added drop-wise to a solution of 3,4-propylenedioxythiophene **44** (300 mg, 1.92 mmol) in dioxane (10 mL). The mixture was heated to 100 ºC for 90 min then allowed to cool to room temperature. Piperidine (948 µL, 9.6 mmol) was then added and the mixture stirred for 3 hrs. After this time the mixture was diluted with DCM (200 mL), and the organics washed with water (100 mL) and brine (100 mL), dried with $MgSO_4$, filtered and concentrated *in vacuo*. The residue was purified by flash column chromatography eluting with 20-50 % EtOAc:Hexane. Pure fractions were concentrated *in vacuo* to give the DP as a white solid. A yield of 520 mg, 1.76 mmol (92 %) was obtained. ^{1}H NMR (400 MHz, $CDCl_3$): δ = 7.06 (1H, s, Ar$\underline{H}$), 4.20 (2H, t, *J* = 5.2 Hz, ArC4-OC$\underline{H}_2$-), 4.12 (2H, t, *J* = 5.3 Hz, ArC3-OC$\underline{H}_2$-), 3.58-3.68 (2H, m, -NC$\underline{H}_2$-), 3.31-3.39 (2H, m, -N$\underline{C}$H$_2$-), 2.22-2.23 (2H, m, -OCH$_2$C$\underline{H}_2$-), 1.55-1.67 (6H, m, -NCH$_2$C$\underline{H}_2$C$\underline{H}_2$-) ppm; ^{13}C NMR (100 MHz, $CDCl_3$): δ = 183.46 (-$\underline{C}$OCON-), 165.56 (-$\underline{C}$ON-), 155.44 (Ar$\underline{C}$4), 150.29 (Ar$\underline{C}$3), 121.66 (Ar$\underline{C}$2), 118.04 (Ar$\underline{C}$5), 71.72 (ArC4-O$\underline{C}$H$_2$-), 71.23 (ArC3-O$\underline{C}$H$_2$-), 46.90 (-C$\underline{H}_2$N-), 42.04 (-C$\underline{H}_2$N-), 33.08 (-OCH$_2\underline{C}$H$_2$-), 25.85 (-$\underline{C}$H$_2\underline{C}$H$_2$CH$_2$N-), 25.23 (-$\underline{C}$H$_2\underline{C}$H$_2$CH$_2$N-), 24.51 (-$\underline{C}$H$_2\underline{C}$H$_2$CH$_2$N-) ppm; IR ($υ_{max}$, solid): 3076, 2944, 2855, 1738, 1622, 1482, 1459, 1396, 1382, 1370, 1356, 1305, 1282, 1270, 1251, 1229, 1197, 1046, 1016 cm^{-1}; HRMS *m/z* (ESI+): Found: 296.0957 (M+H), Calc.: 296.0957; m.p. = 135-137 °C;

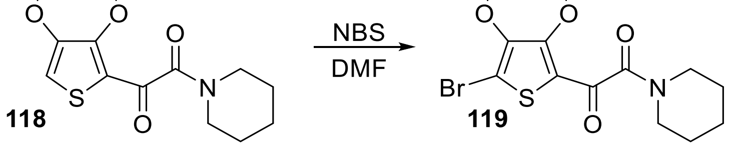

N-Bromosuccinimide (362 mg, 2.03 mmol) was added in the dark to a solution of ProDOT derivative **118** (500 mg, 1.69 mmol) in a mixture of acetic acid (15 mL) and THF (10 mL). After stirring for 18 hrs

the mixture was diluted with DCM (150 mL) and the organics washed with water (100 mL) and sat. $NaHCO_3$ (2 x 100 mL), dried with $MgSO_4$, filtered and concentrated *in vacuo*. The residue was purified by flash column chromatography eluting with 30-50 % EtOAc:Hexane. Pure fractions were concentrated *in vacuo* to give the DP as a light yellow oil. A yield of 508 mg, 1.36 mmol (81 %) was obtained. ^1H NMR (400 MHz, $CDCl_3$): δ = 4.26-4.15 (4H, m, $-OC\underline{H}_2-$), 3.62 (2H, t, *J* = 5.4 Hz, $-NC\underline{H}_2-$), 3.29-3.38 (2H, m, $-N\underline{C}H_2-$), 2.31 (2H, tt, *J* = 6.0, 4.7 Hz, $-OCH_2C\underline{H}_2-$), 1.53-1.7 (6H, m, $-NCH_2C\underline{H}_2C\underline{H}_2-$) ppm; ^{13}C NMR (100 MHz, $CDCl_3$): δ = 182.27 ($-\underline{C}OCON-$), 165.16 ($-\underline{C}ON-$), 154.24 ($Ar\underline{C}_\beta$), 148.27 ($Ar\underline{C}_\beta$), 120.99 ($Ar\underline{C}2$), 109.64 ($Ar\underline{C}5$), 72.01 ($-O\underline{C}H_2-$), 71.48 ($-O\underline{C}H_2-$), 46.92 ($-C\underline{H}_2N-$), 42.11 ($-C\underline{H}_2N-$), 32.86 ($-OCH_2\underline{C}H_2-$), 25.88 ($-\underline{C}H_2\underline{C}H_2CH_2N-$), 25.22 ($-\underline{C}H_2\underline{C}H_2CH_2N-$), 24.46 ($-\underline{C}H_2\underline{C}H_2CH_2N-$) ppm; IR ($\upsilon_{max}$, solid): 2969, 2944, 2854, 1738, 1627, 1483, 1448, 1394, 1351, 1295, 1265, 1217, 1121, 1105, 1073, 1013 cm^{-1}; HRMS *m/z* (ESI+): Found: 374.0070/376.0040 (M+H), Calc.: 374.0062/374.0036; m.p. = 87-92 °C;

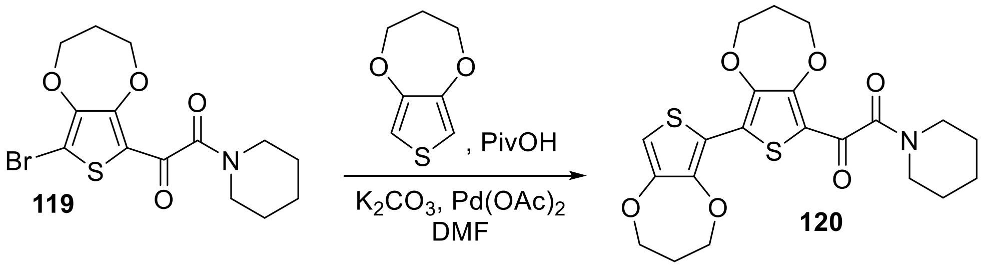

Brominated piperidine-ProDOT monomer **119** (200 mg, 0.53 mmol), pivalic acid (27 mg, 0.26 mmol), palladium (II) acetate (6 mg, 0.03 mmol), potassium carbonate (731 mg, 5.3 mmol) and ProDOT **44** (250 mg, 1.60 mmol) were charged under nitrogen. Dry DMF (4 mL) was then added and the mixture heated to 90 ºC for 1 hr. After cooling to rt the mixture was diluted with DCM (150 mL) and washed with water (2 x 100 mL) and brine (100 mL). The organics were dried with $MgSO_4$, filtered and concentrated *in vacuo*. The residue was purified by flash column chromatography eluting first with 40-80 % EtOAc:Hexane to elute the DP, then 0-10 % MeOH:EtOAc to elute SP **???** (see below). Pure fractions were concentrated *in vacuo* and the residue triturated in diethyl ether (2 x 100 mL) to give the DP as a yellow solid. A yield of 152 mg, 0.34 mmol (64 %) was obtained. ^1H NMR (400 MHz, $CDCl_3$): δ = 6.61 (1H, s, $Ar\underline{H}10$), 4.23 (2H, dd, *J* = 5.9, 4.4 Hz, $-OC\underline{H}_2-$), 4.16-4.21 (4H, m, $-OC\underline{H}_2-$), 4.10 (2H, dd, *J* = 5.8, 4.4 Hz, $-OC\underline{H}_2-$), 3.61 (2H, t, *J* = 5.3 Hz, $-NC\underline{H}_2-$), 3.28-3.37 (2H, m, $-NC\underline{H}_2-$), 2.21-2.35 (4H, m, $-OCH_2C\underline{H}_2-$), 1.60-1.72 (4H, m, $-C\underline{H}_2C\underline{H}_2CH_2N-$), 1.54-1.60 (2H, m, $-C\underline{H}_2CH_2N-$) ppm; ^{13}C NMR (100 MHz, $CDCl_3$): δ = 183.47 ($-\underline{C}OCON-$), 165.91 ($-\underline{C}ON-$), 155.03 ($Ar\underline{C}_\beta$), 149.95 ($Ar\underline{C}_\beta$), 148.55 ($Ar\underline{C}_\beta$),

144.61 (ArC_{β}), 128.53 (ArC_{α}), 117.25 (ArC_{α}), 115.04 (ArC_{α}), 108.07 (ArC_{α}), 71.77 (-OCH_2-), 71.33 (-OCH_2-), 71.18 (-OCH_2-), 71.08 (-OCH_2-), 46.93 (-CH_2N-), 41.97 (-CH_2N-), 33.48 (-OCH_2CH_2-), 33.02 (-OCH_2CH_2-), 25.84 (-$CH_2CH_2CH_2N$-), 25.23 (-$CH_2CH_2CH_2N$-), 24.52 (-$CH_2CH_2CH_2N$-) ppm; IR (υ_{max}, solid): 2969, 2922, 2864, 1738, 1631, 1603, 1535, 1495, 1470, 1439, 1421, 1398, 1357, 1319, 1285, 1267, 1250, 1216, 1180, 1079, 1047, 1023 cm^{-1}; HRMS *m/z* (ESI+): Found: 450.1059 (M+H), Calc.: 450.1045; m.p. = 184-190 °C;

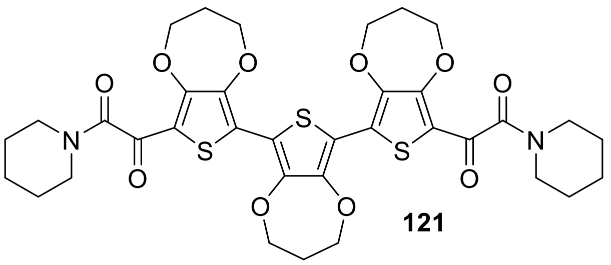

121

121 was obtained as a side product from the above reaction for the synthesis of **120**. The product was obtained as an orange solid. A yield of 20 mg, 27 µmol (10 %) was obtained. ^{1}H NMR (400 MHz, $CDCl_3$): δ = 4.15-4.34 (12H, m, -OCH_2-), 3.61 (4H, t, *J* = 5.3 Hz, -NCH_2-), 3.35 (4H, t, *J* = 5.4 Hz, -NCH_2-), 2.26-2.39 (6H, m, -OCH_2CH_2-), 1.54-1.73 (12H, m, -N$CH_2CH_2CH_2$-) ppm; ^{13}C NMR (100 MHz, $CDCl_3$): δ = 183.37 (-$COCONR_2$), 165.81 (-$CONR_2$), 154.85 (ArC_{β}), 147.95 (ArC_{β}), 145.20 (ArC_{β}), 127.76 (ArC_{α}), 118.31 (ArC_{α}), 116.40 (ArC_{α}), 71.80 (-OCH_2-), 71.35 (-OCH_2-), 71.20 (-OCH_2-), 46.95 (-NCH_2-), 42.00 (-NCH_2-), 33.10 (-OCH_2CH_2-), 32.98 (-OCH_2CH_2-), 25.87 (-NCH_2CH_2-), 25.24 (-N$CH_2CH_2CH_2$-), 24.51 (-N$CH_2CH_2CH_2$-) ppm; IR (υ_{max}, solid): 2934, 2852, 1738, 1617, 1472, 1438, 1406, 1371, 1313, 1245, 1216, 1128, 1050, 1012 cm^{-1}; HRMS *m/z* (ESI+): Found: 743.1470 (M+H), Calc.: 743.1767; m.p. = 198-202 °C;

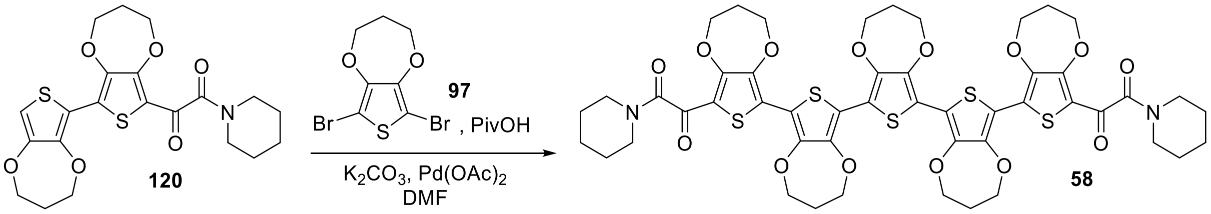

Piperidine-ProDOT dimer **120** (110 mg, 245 µmol), pivalic acid (15 mg, 123 µmol), palladium (**II**) acetate (6 mg, 25 µmol), potassium carbonate (338 mg, 2.45 mmol), and 2,5-dibromoProDOT **97** (53 mg, 171 µmol) were charged under nitrogen. Dry DMF (3 mL) was then added and the mixture heated to 130 ºC for 1 hr. After cooling to rt the mixture was diluted with DCM (100 mL) and washed with water (2 x 75 mL) and brine (75 mL). The organics were dried with $MgSO_4$, filtered and concentrated *in vacuo*. The residue was purified by flash column chromatography eluting with 1-4 % MeOH:DCM. Pure fractions were concentrated *in vacuo* and the residue triturated in diethyl ether (2 x 80 mL) to give the DP as a

red-purple solid. A yield of 77 mg, 73 µmol (60 %) was obtained. ^{1}H NMR (400 MHz, $CDCl_3$): δ = 4.17-4.35 (20H, m, -OC$\underline{H}_2$-), 3.60-3.71 (2H, m, -NC$\underline{H}_2$-), 3.33-3.43 (2H, m, -NC$\underline{H}_2$-), 2.27-2.43 (10H, m, -OCH$_2$C$\underline{H}_2$-), 1.54-1.80 (12H, m, -C$\underline{H}_2$C$\underline{H}_2$CH$_2$N-) ppm; IR ($υ_{max}$, solid): 2939, 2854, 1738, 1618, 1471, 1407, 1356, 1312, 1267, 1216, 1132, 1108, 1041 cm^{-1}; HRMS *m/z* (ESI+): Found: 1083.1930 (M+H), Calc.: 1083.1928; m.p. > 350 °C;

References

1. Aubineau, T.; Cossy, J. *Chem. Commun.*, **2013**, *49* (32), 3303.
2. Stover, J. S.; Shi, J.; Jin, W.; Vogt, P. K.; Boger, D. L. *J. Am. Chem. Soc.*, **2009**, *131* (9), 3342.
3. Liu, J.; Kolar, C.; Lawson, T. A.; Gmeiner, W. H. *J. Org. Chem.*, **2001**, *66* (17), 5655.
4. Lankshear, M. D.; Dudley, I. M.; Chan, K-M.; Cowley, A. R.; Santos, S. M.; Felix, V.; Beer, P. D. *Chem. Eur. J.*, **2008**, *14* (7), 2248.
5. Bertozzi, C. R.; Bednarski, M. D. *J. Org. Chem.*, **1991**, *56*, 4326.
6. Morimoto, K.; Nakae, T.; Yamaoka, N.; Dohi, T.; Kita, Y. *Eur. J. Org. Chem*., **2011**, *31*, 6326.
7. Turbiez, M.; Frère, P.; Roncali, J. *J. Org. Chem*., **2003**, *68* (13), 5357.
8. Goto, H. *J. Mater. Chem*., **2009**, *19* (28), 4914.
9. Goldoni, F.; Langeveld-Voss, B. M. W.; Meijer, E. W. *Synth. Commun*., **1998**, *28* (12), 2237.
10. Kim, B.; Koh, J. K.; Kim, K.; Chi, W. S.; Kim, J. H.; Kim, E. *ChemSusChem*, **2012**, *5*, 2173-2180.

^{1}H NMR (400 MHz, $CDCl_3$)

Figure S6. ^{1}H NMR of **3**

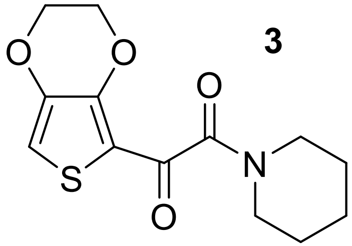

3

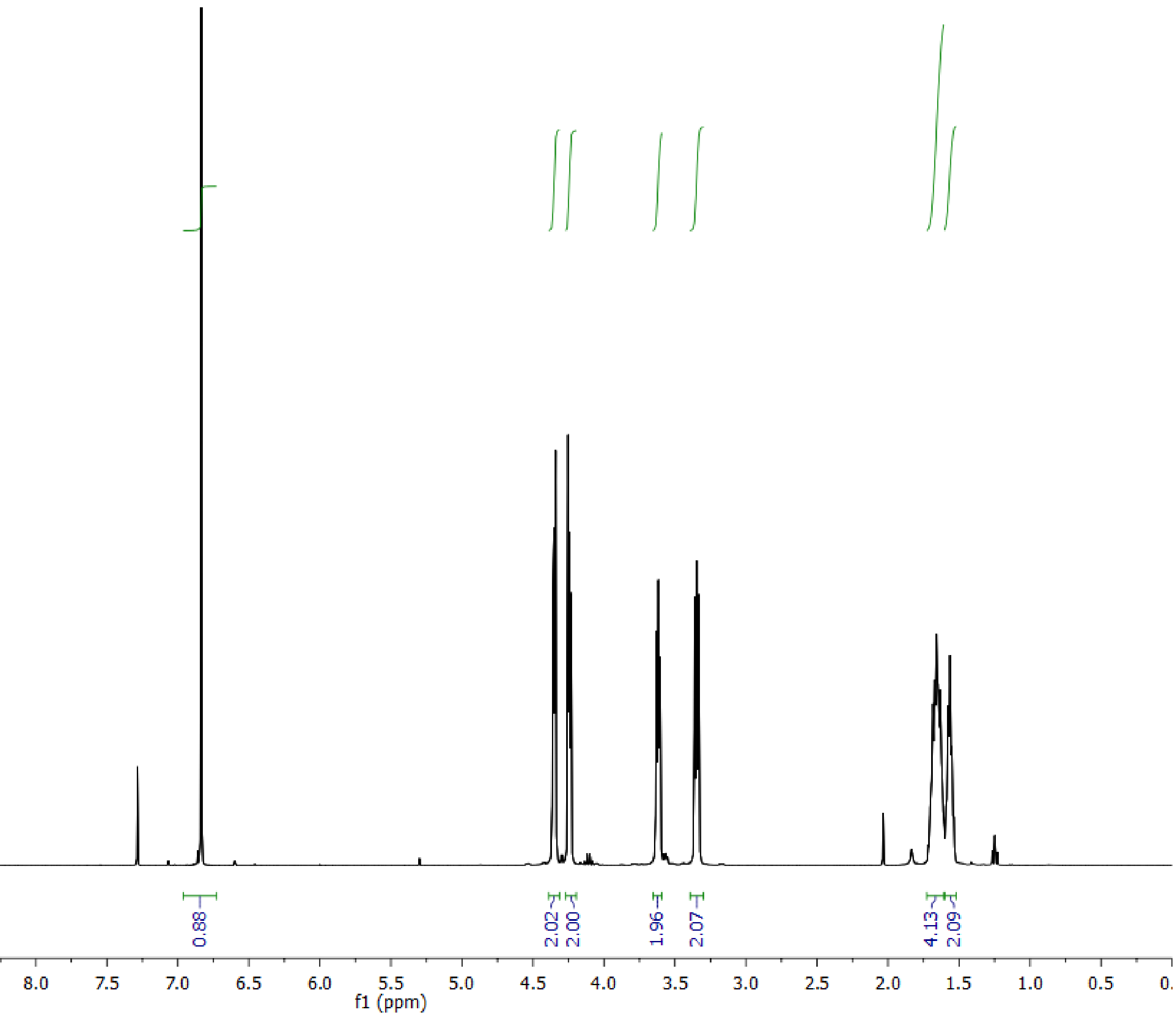

^{13}C NMR (100 MHz, $CDCl_3$)

Figure S7. ^{13}C NMR of **3**

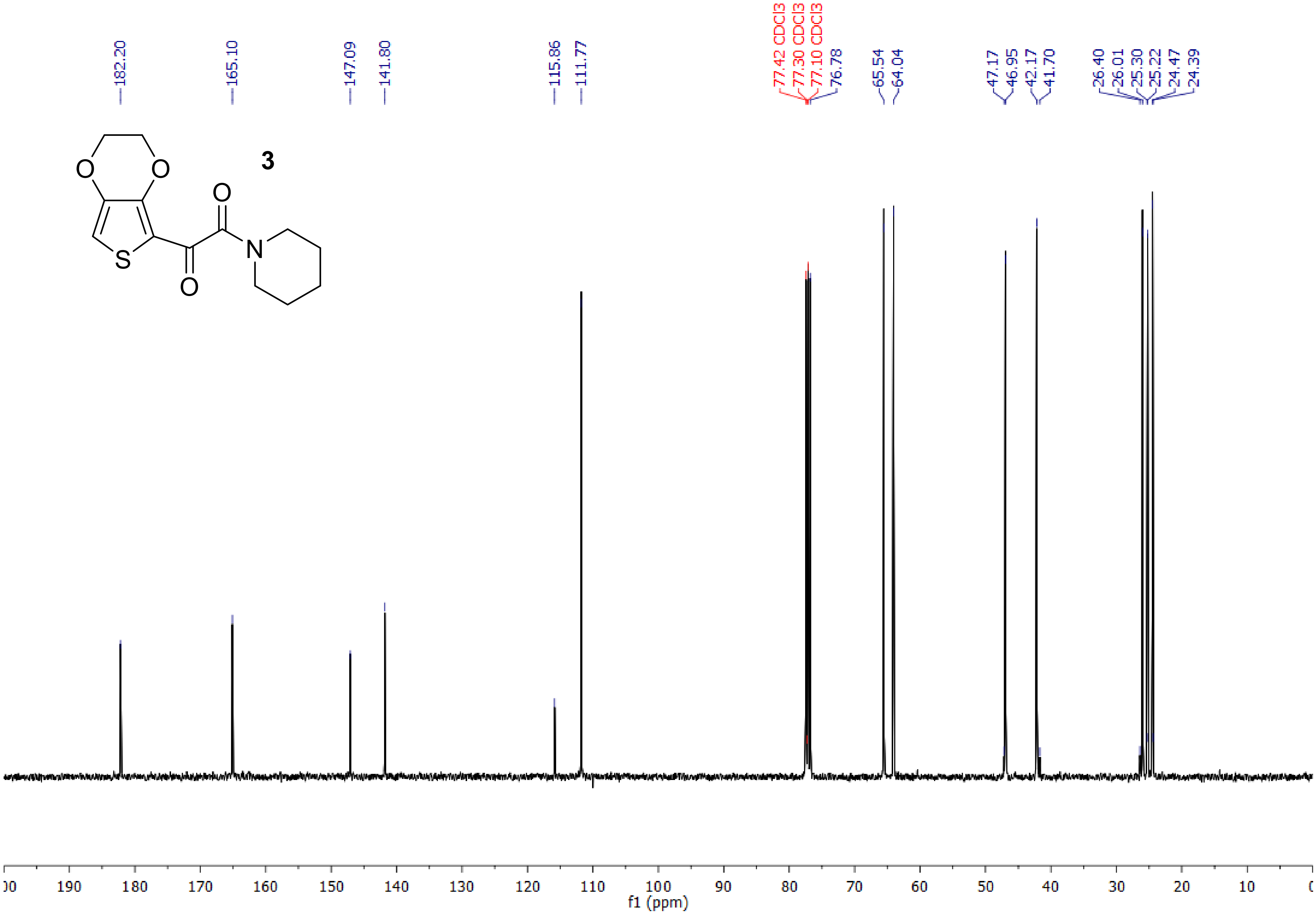

^{1}H NMR (400 MHz, $CDCl_3$)

Figure S8. ^{1}H NMR of **4**

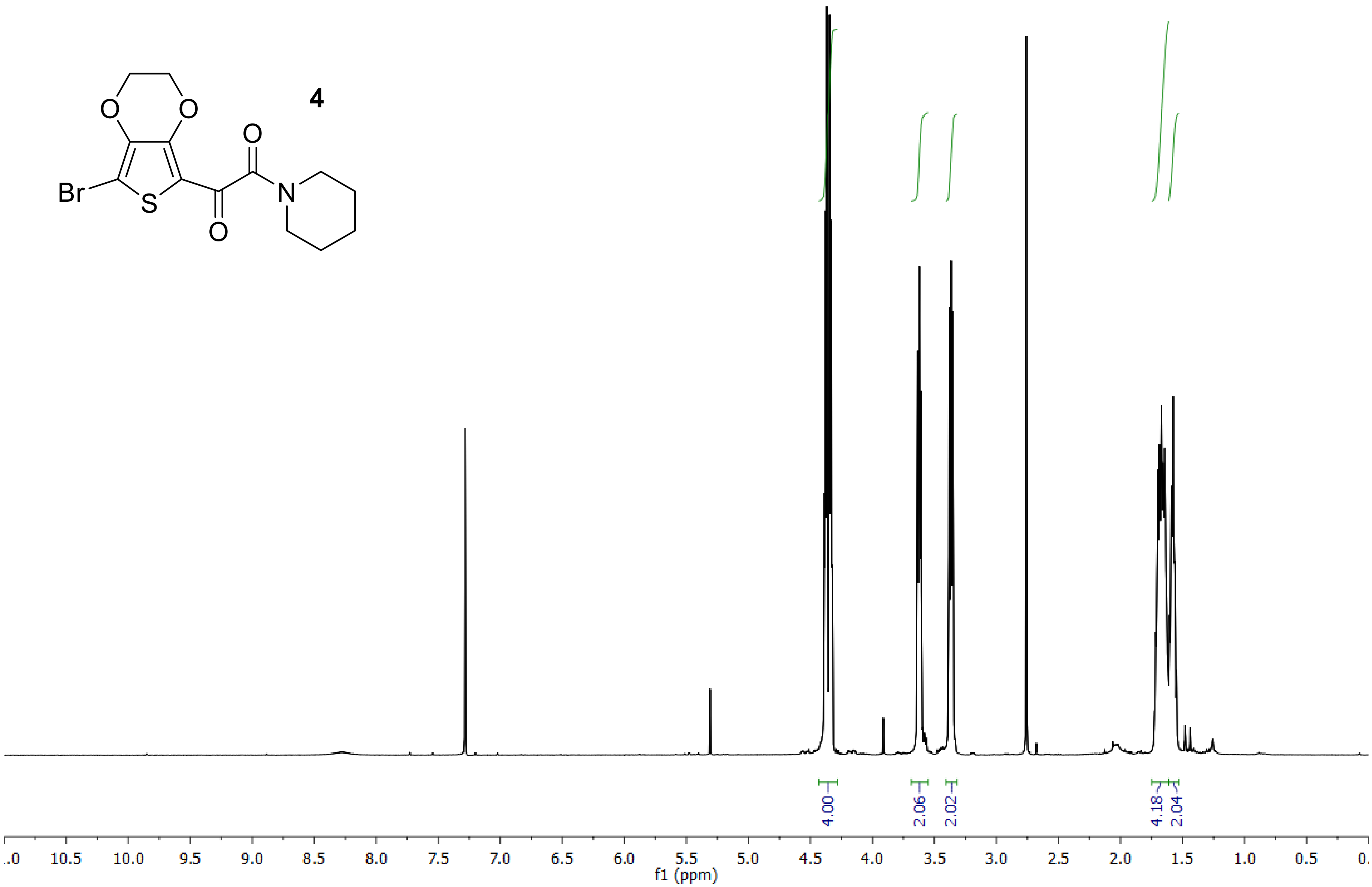

^{13}C NMR (100 MHz, $CDCl_3$)

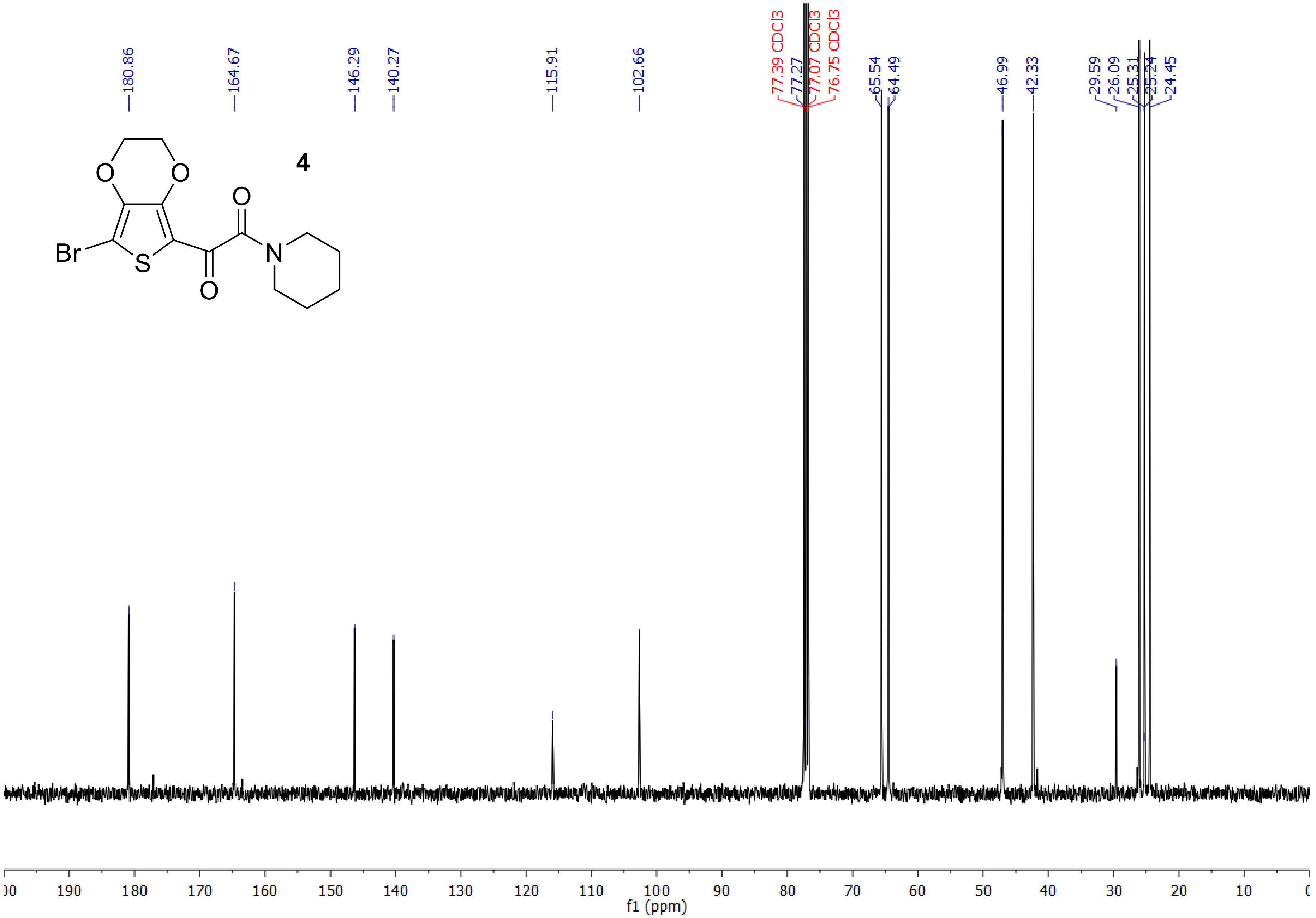

Figure S9. ^{13}C NMR of **4**

^{1}H NMR (400 MHz, $CDCl_3$)

Figure S10. ^{1}H NMR of **5**

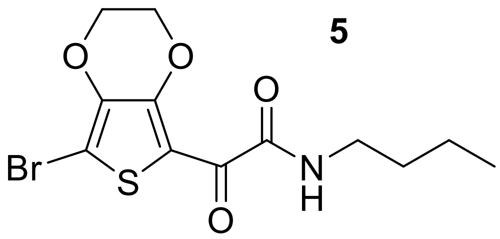

5

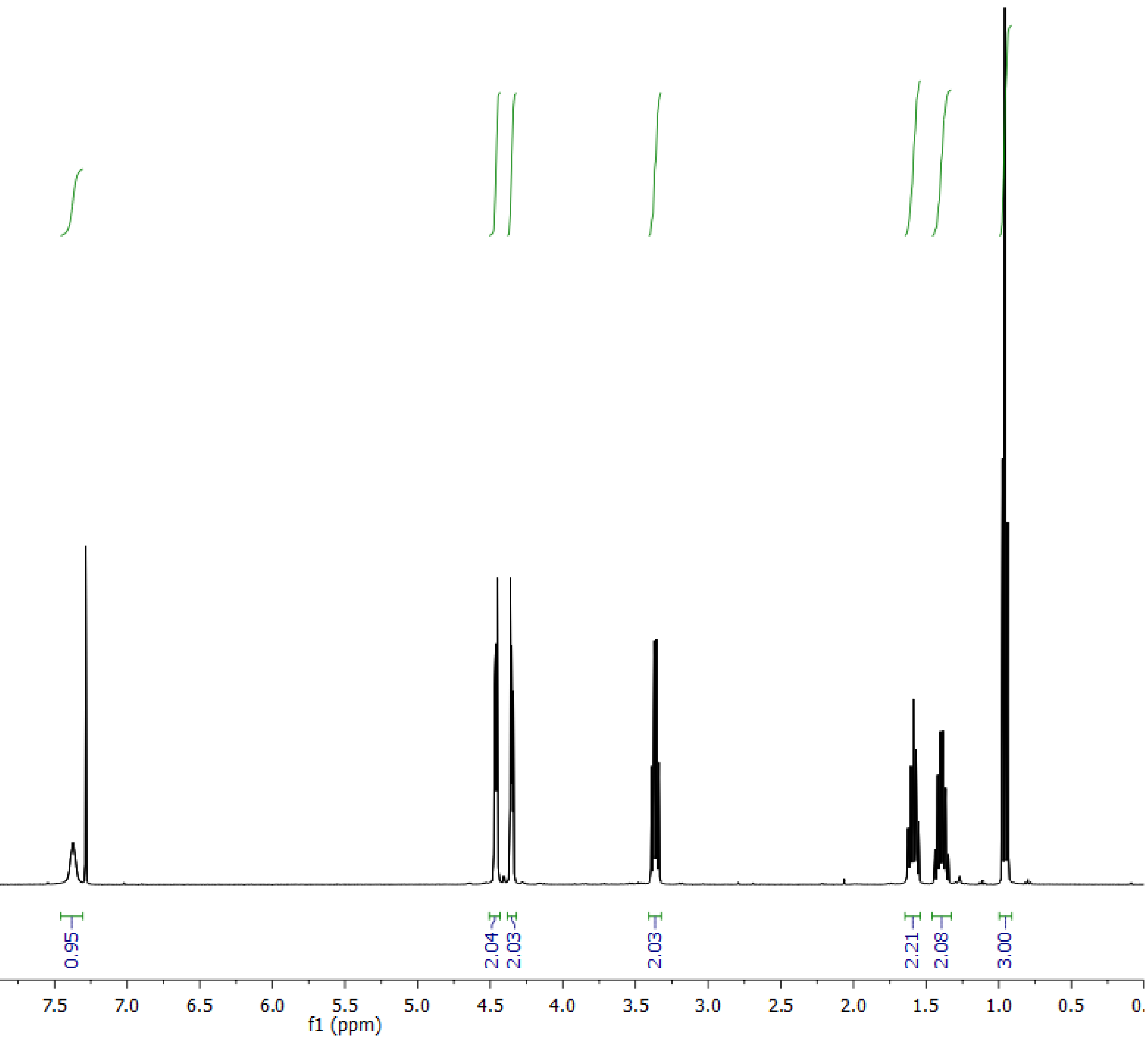

^{13}C NMR (100 MHz, $CDCl_3$)

Figure S11. ^{13}C NMR of **5**

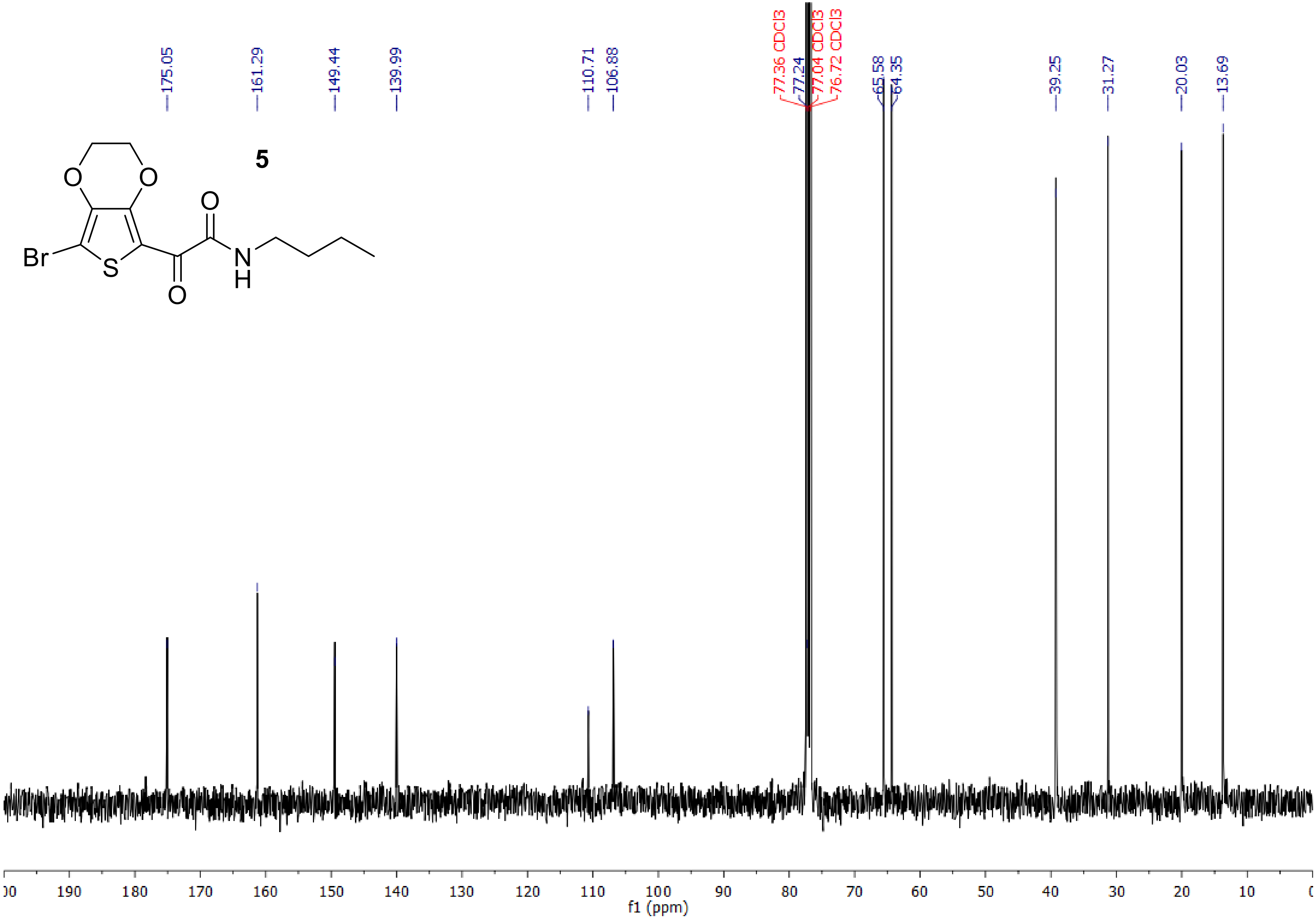

^{1}H NMR (400 MHz, $CDCl_3$)

Figure S12. ^{1}H NMR of **6**

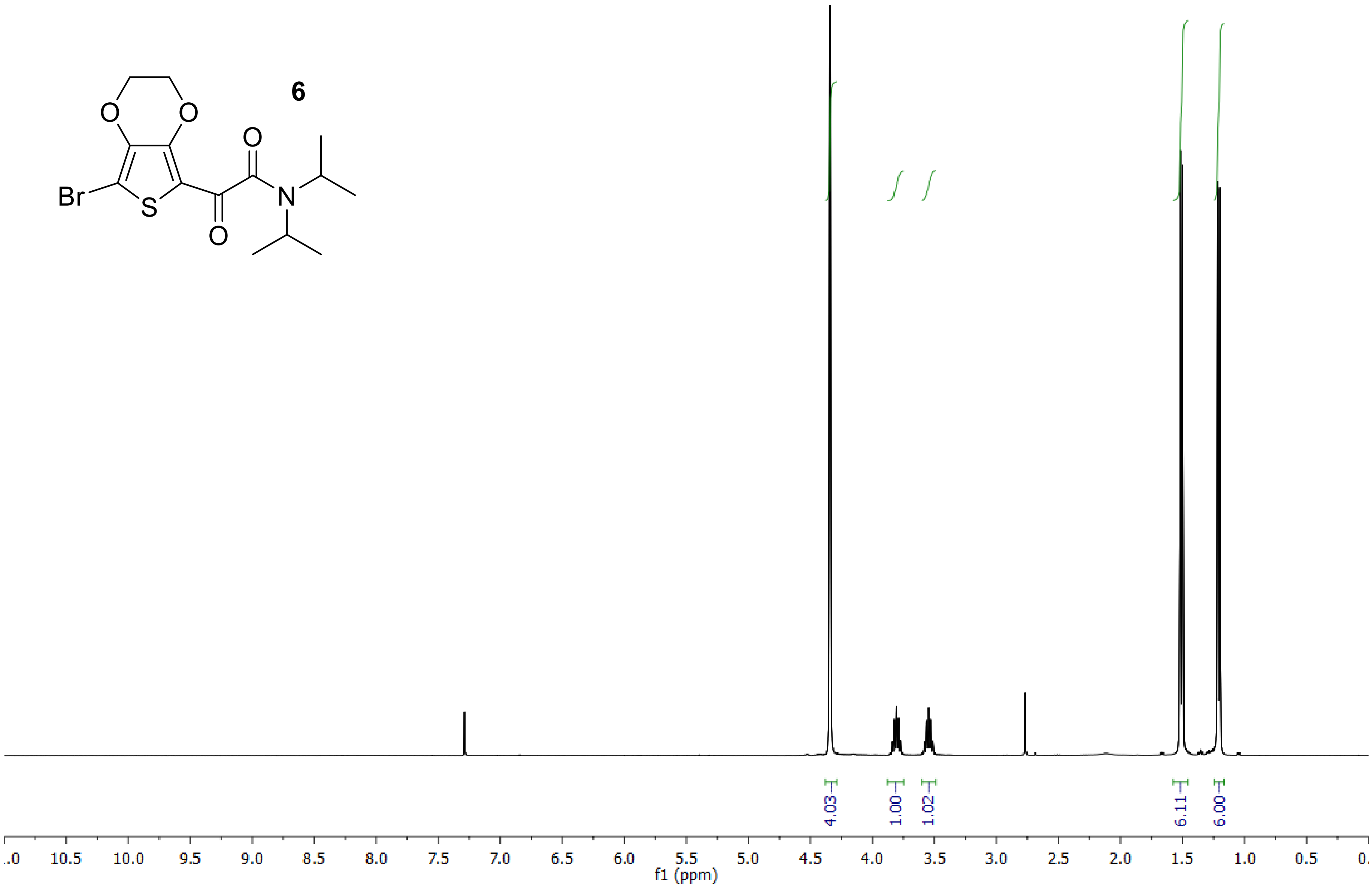

^{13}C NMR (100 MHz, $CDCl_3$)

Figure S13. ^{13}C NMR of **6**

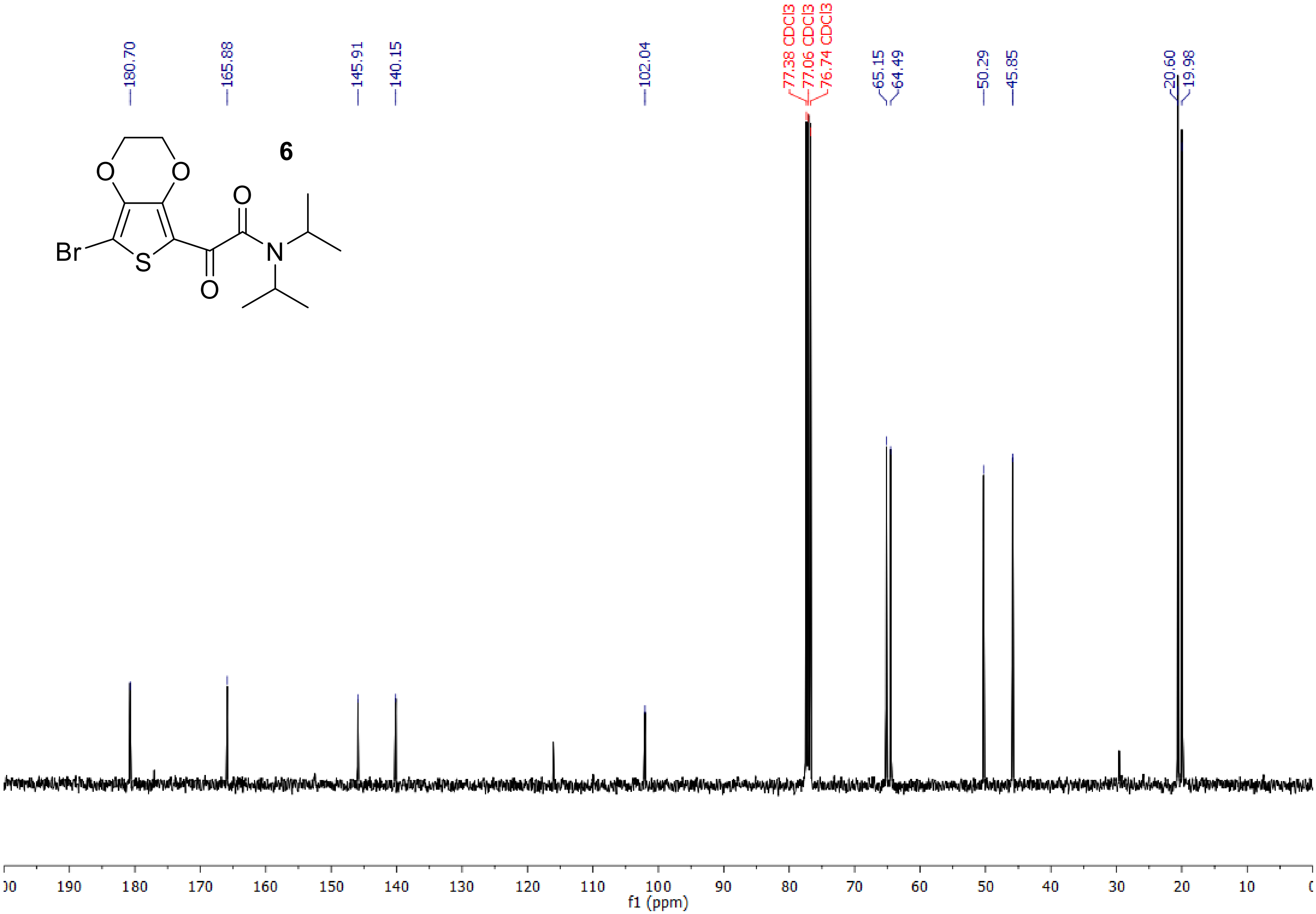

^{1}H NMR (400 MHz, $CDCl_3$)

Figure S14. ^{1}H NMR of **7**

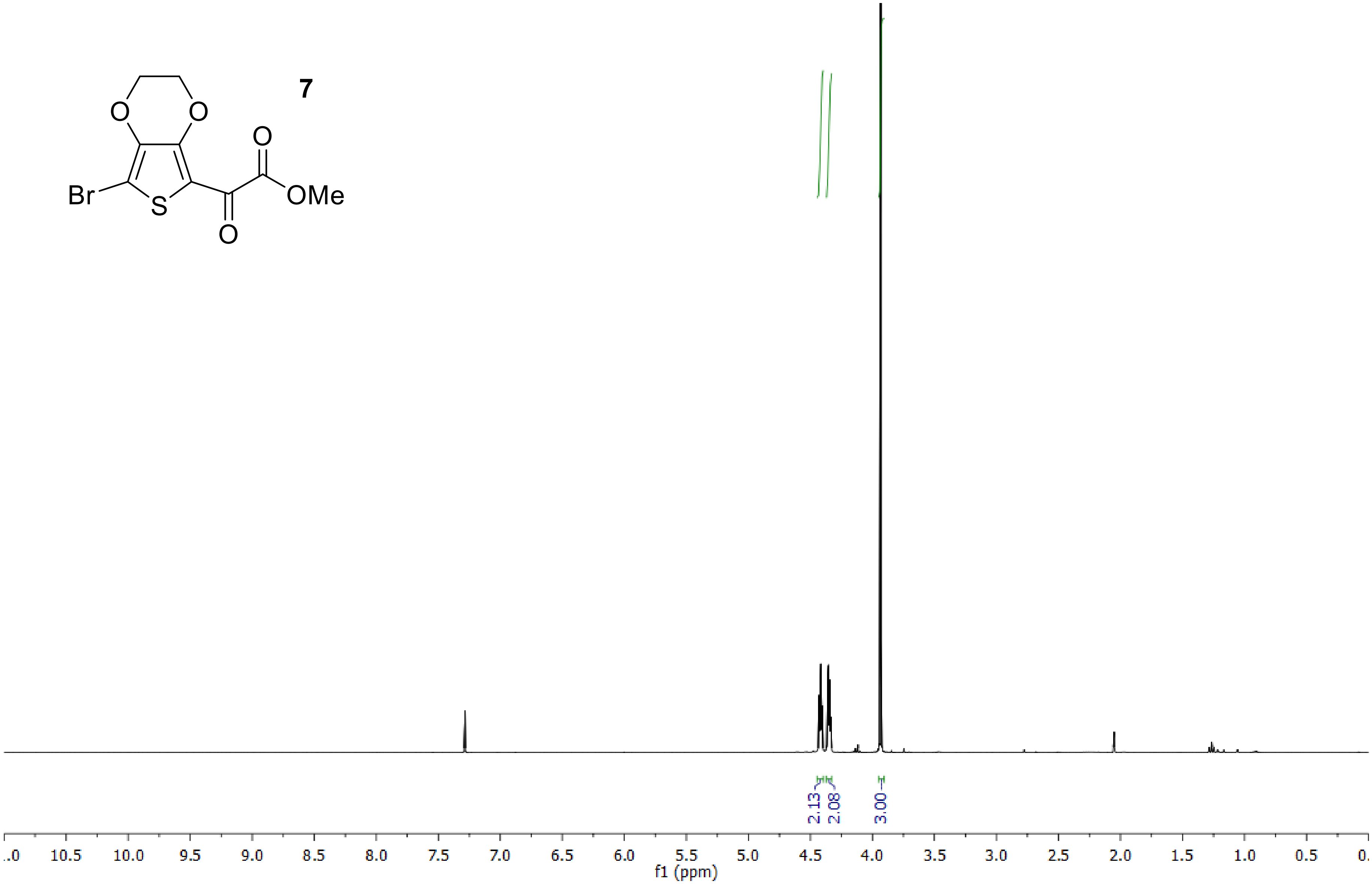

^{13}C NMR (100 MHz, $CDCl_3$)

Figure S15. ^{13}C NMR of **7**

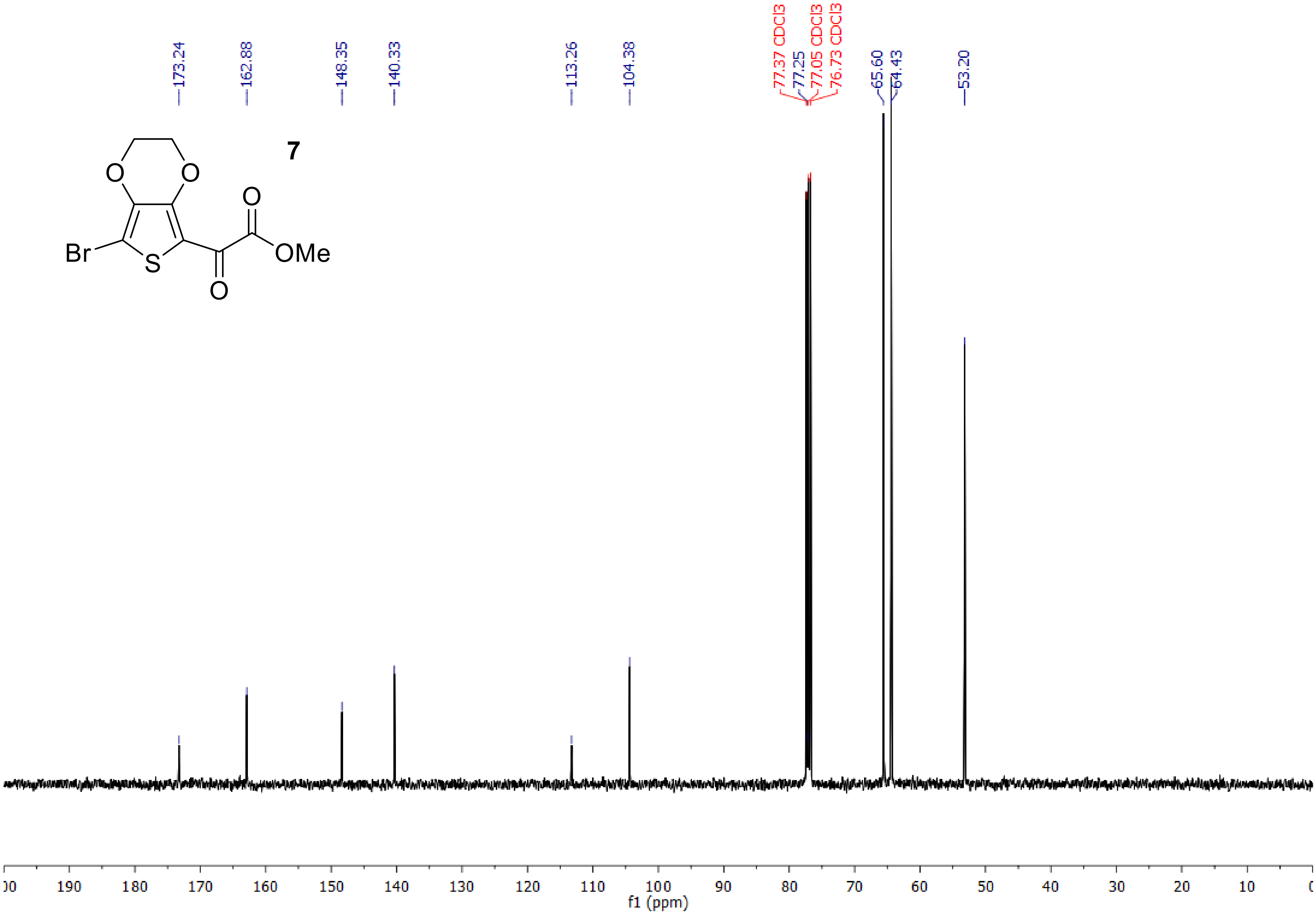

^1H NMR (400 MHz, $CDCl_3$)

Figure S16. ^1H NMR of **8**

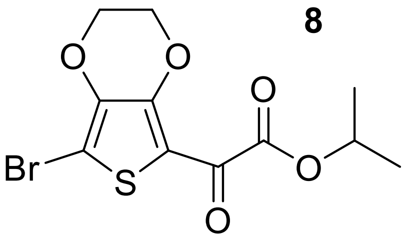

8

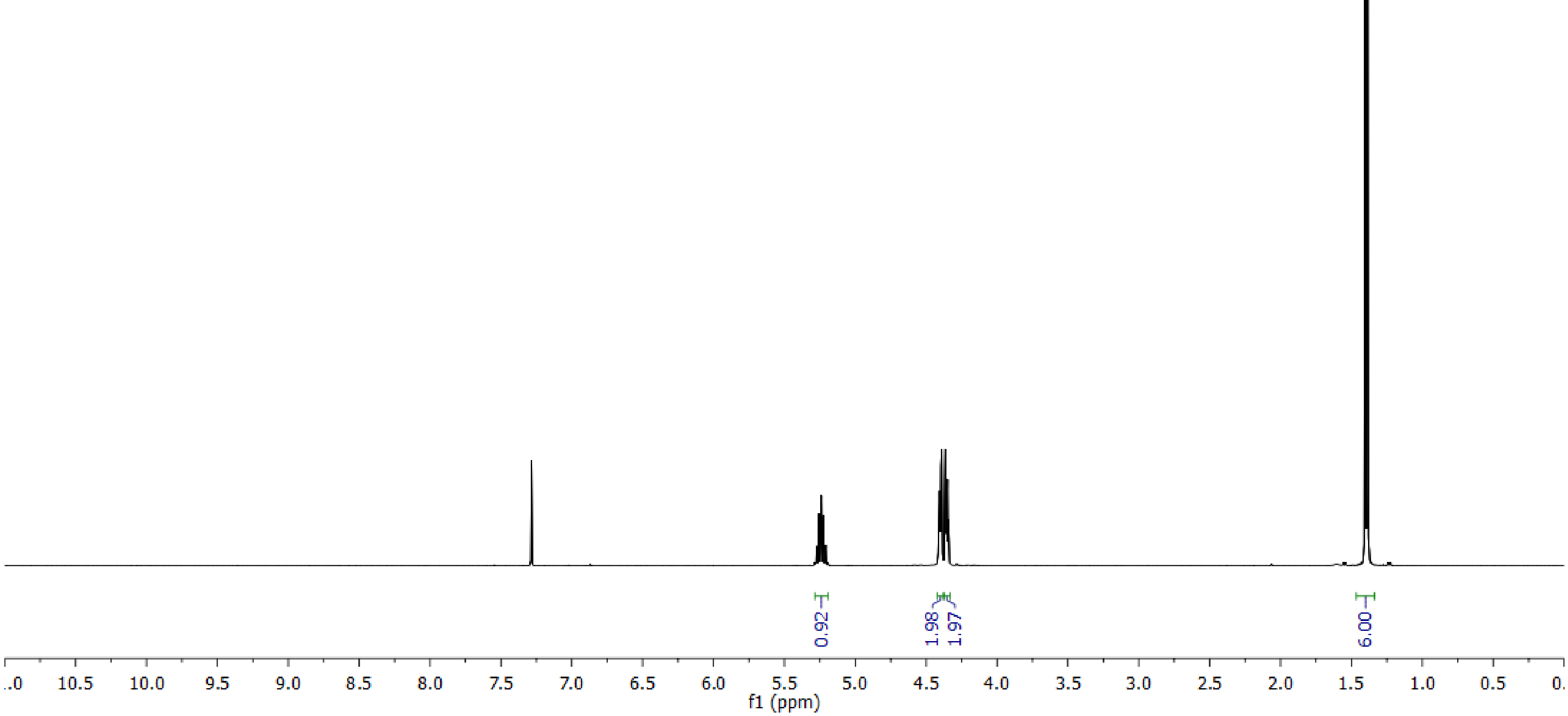

^{13}C NMR (100 MHz, $CDCl_3$)

Figure S17. ^{13}C NMR of **8**

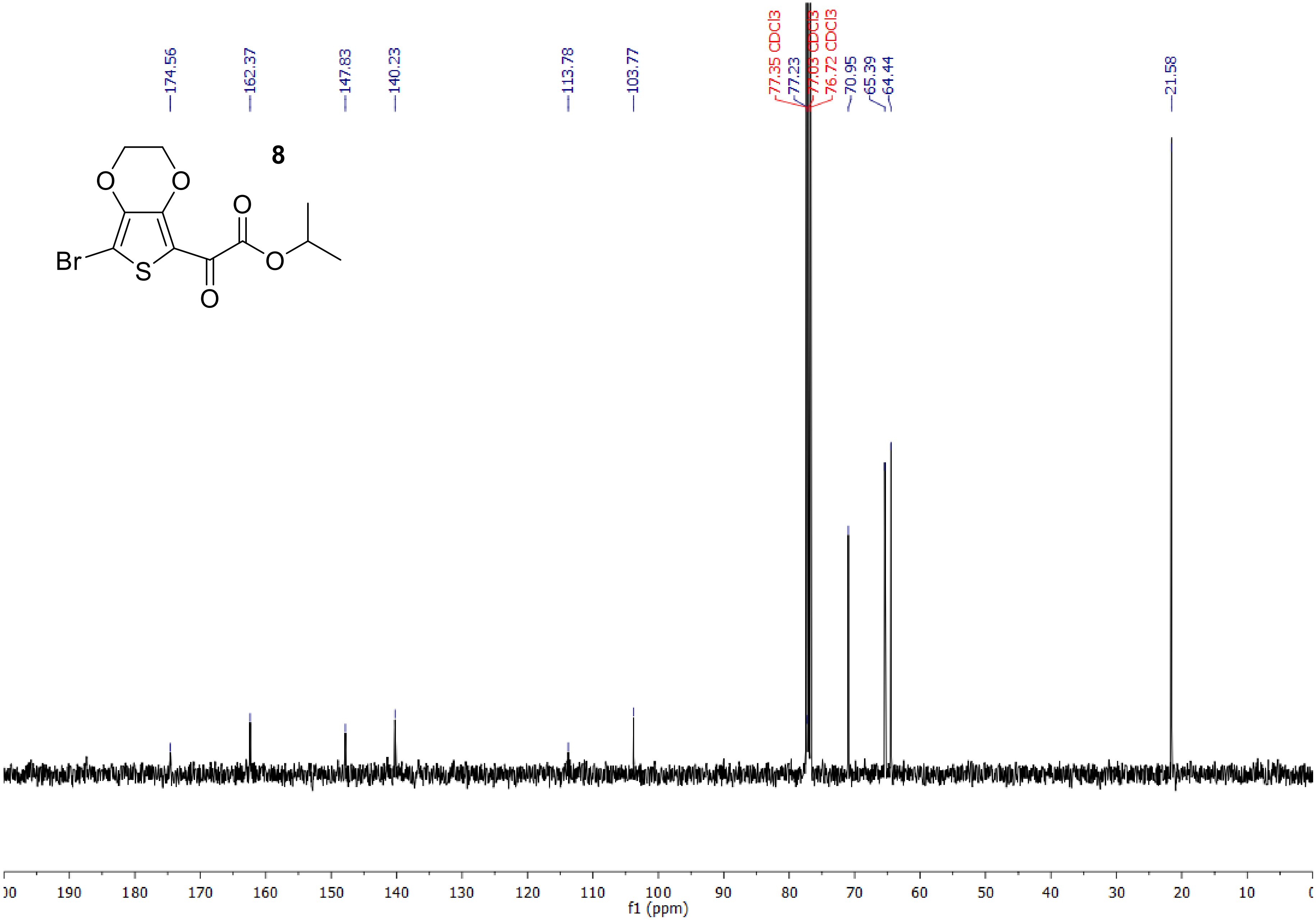

^{1}H NMR (400 MHz, $CDCl_3$)

Figure S18. ^{1}H NMR of **9**

9

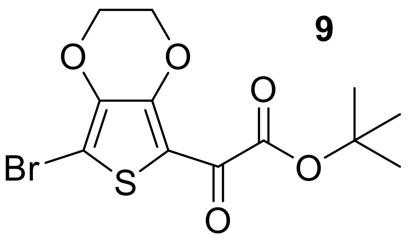

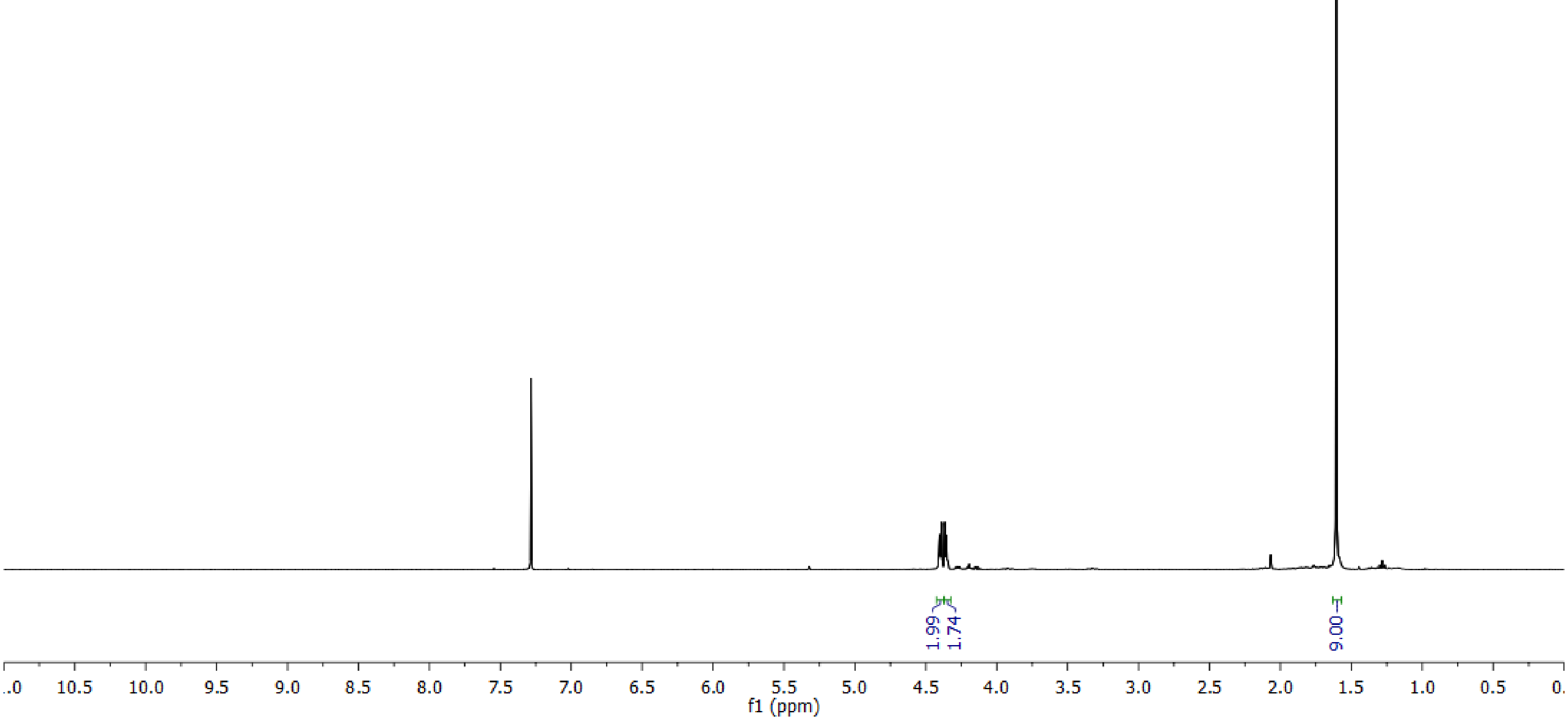

^{13}C NMR (100 MHz, $CDCl_3$)

Figure S19. ^{13}C NMR of **9**

9

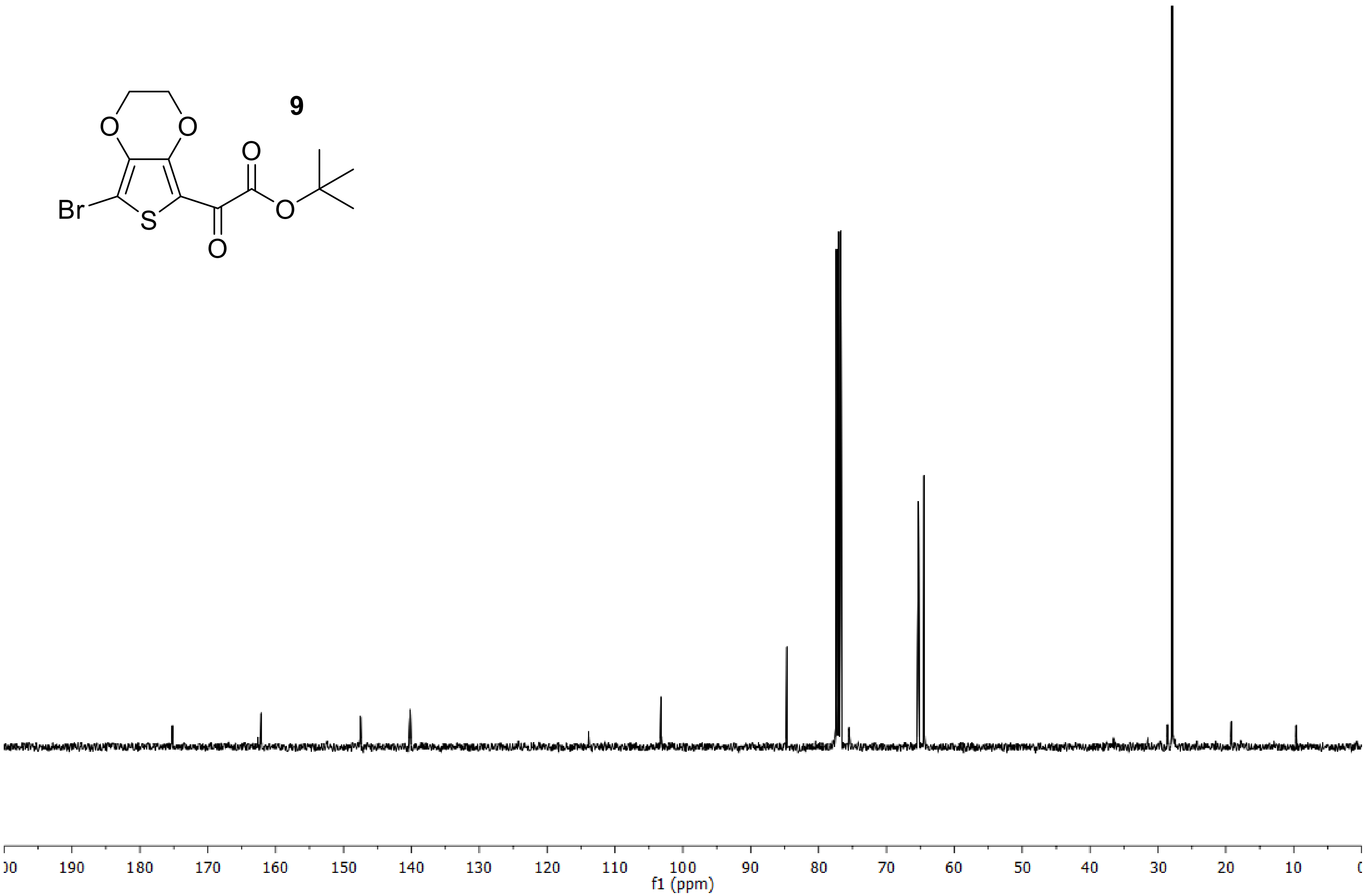

^{1}H NMR (400 MHz, MeOD)

Figure S20. ^{1}H NMR of **10**

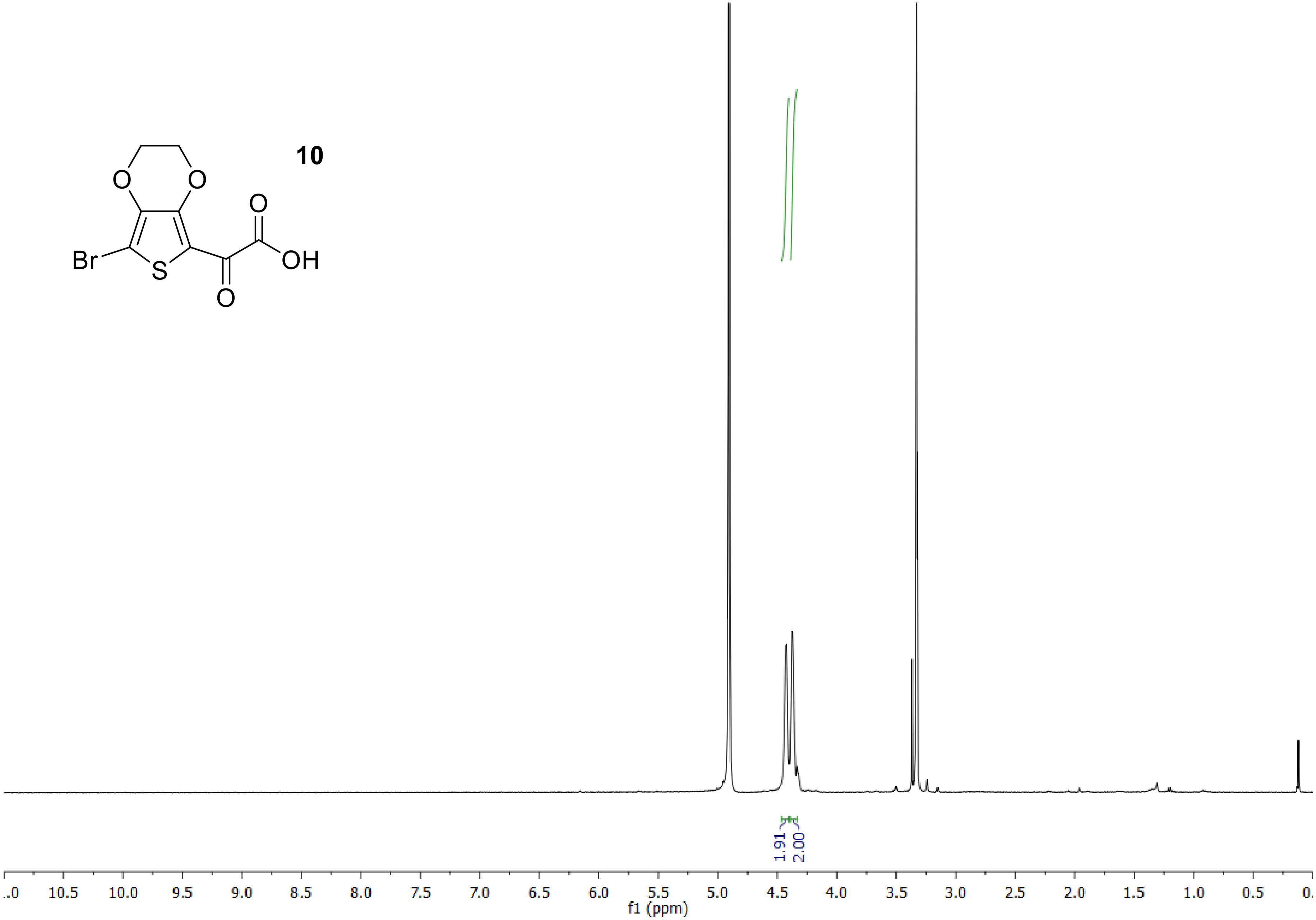

^{13}C NMR (100 MHz, MeOD)

Figure S21. ^{13}C NMR of **10**

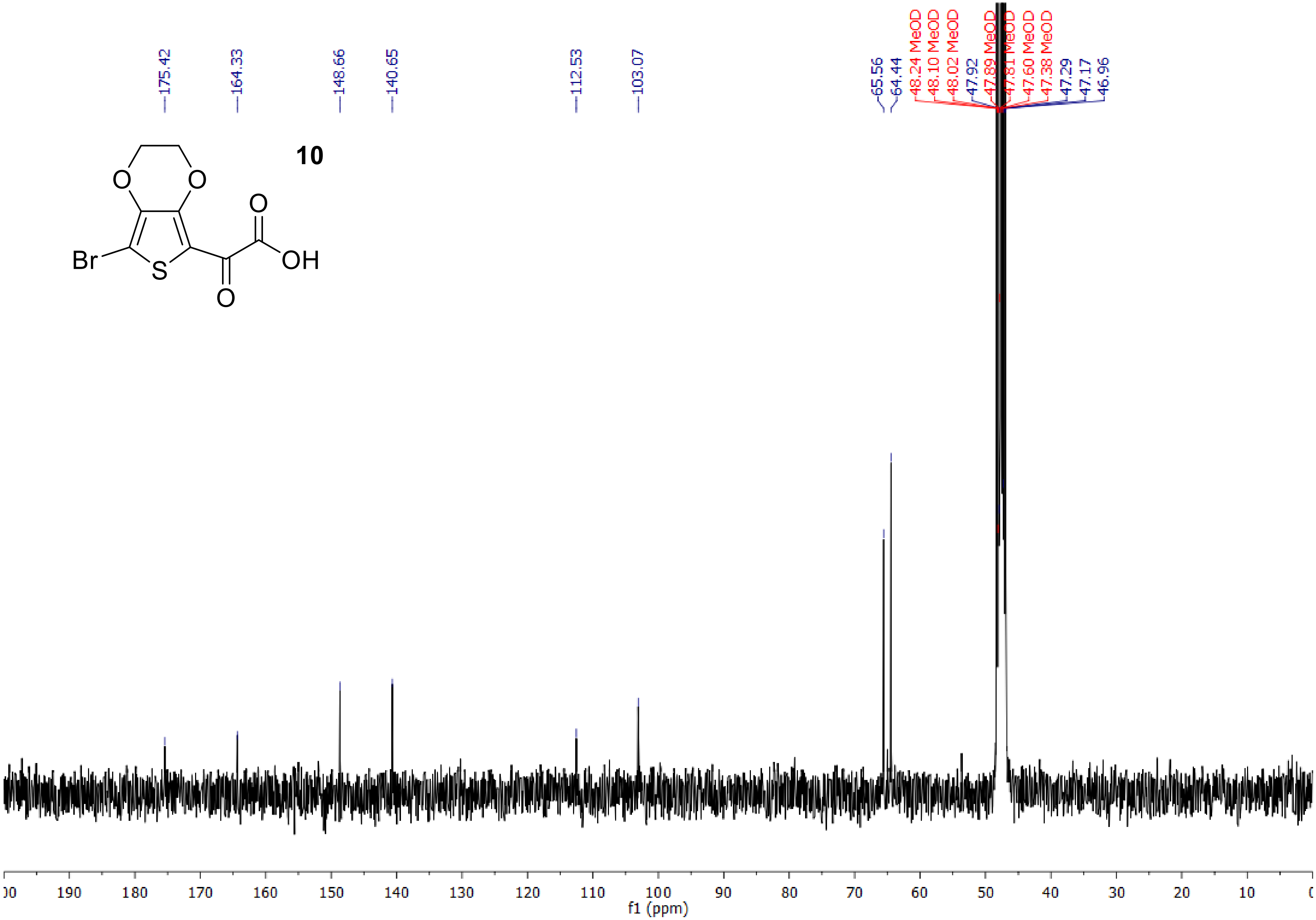

^{1}H NMR (400 MHz, $CDCl_3$)

Figure S22. ^{1}H NMR of **11**

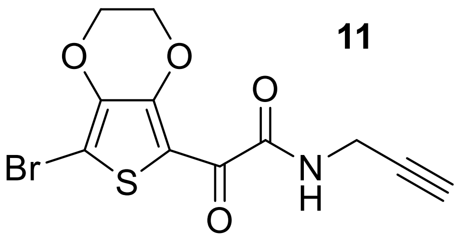

11

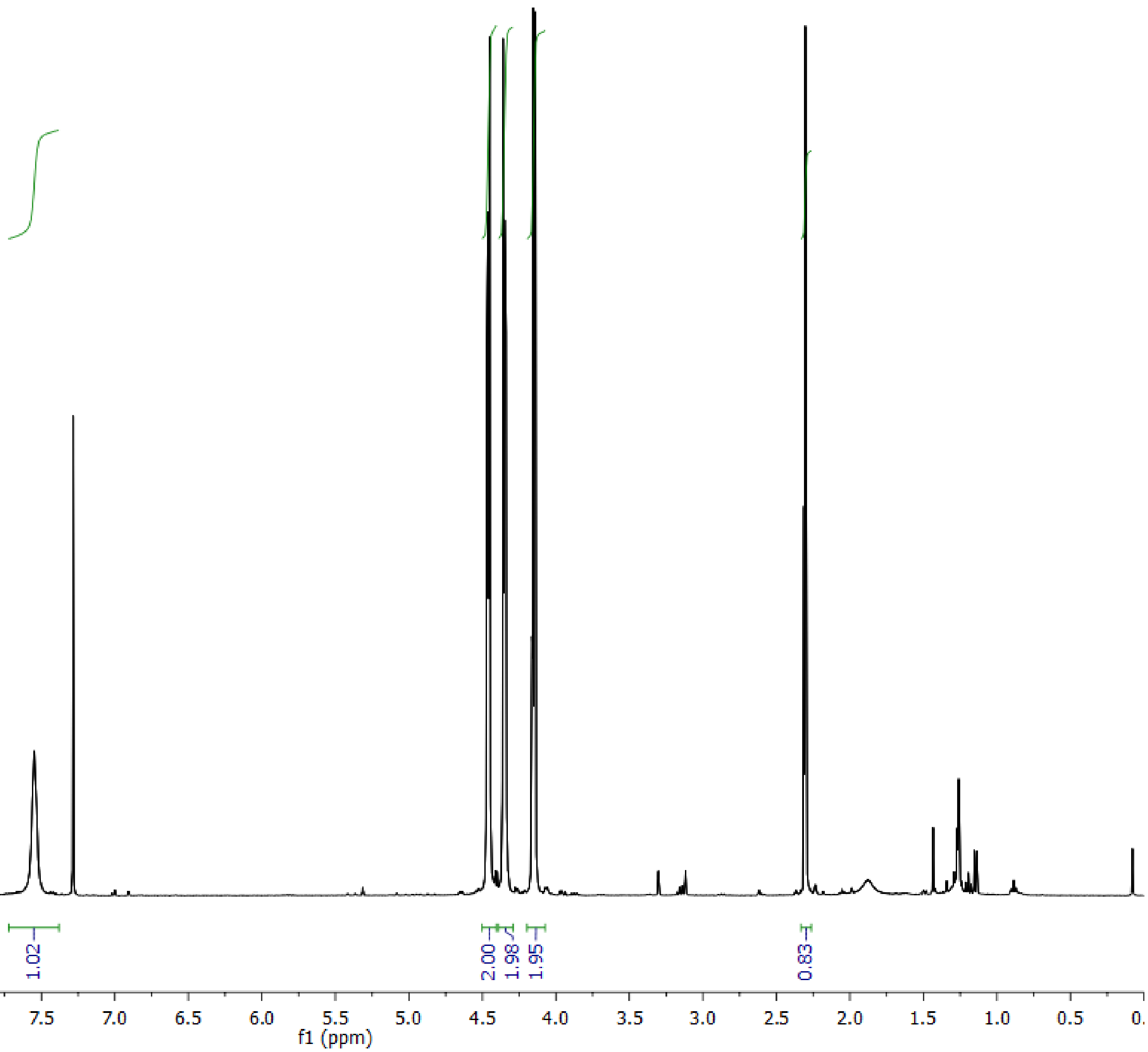

^{13}C NMR (100 MHz, $CDCl_3$)

Figure S23. ^{13}C NMR of **11**

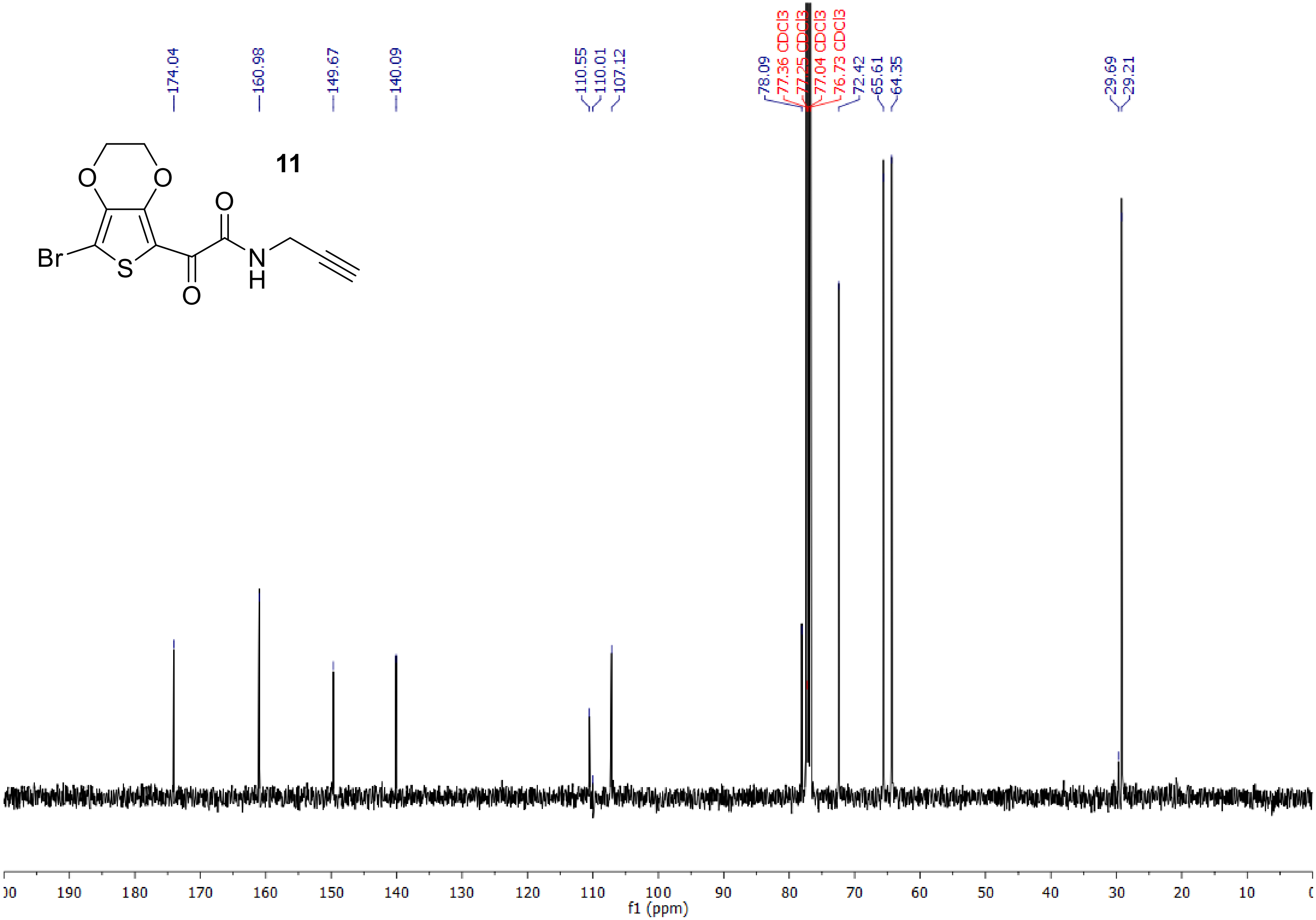

^{1}H NMR (400 MHz, $CDCl_3$)

Figure S24. ^{1}H NMR of **12**

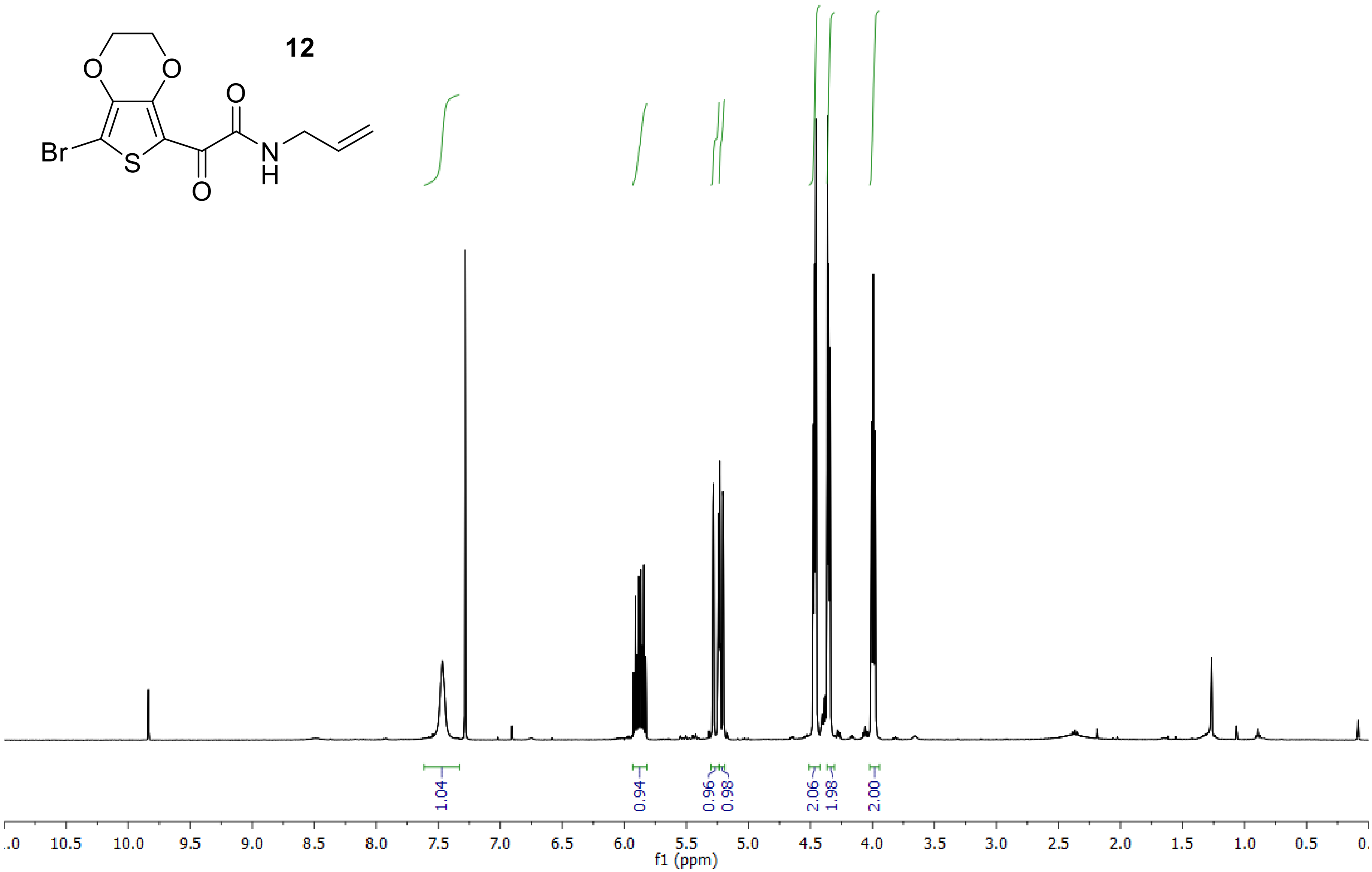

^{13}C NMR (100 MHz, $CDCl_3$)

Figure S25. ^{13}C NMR of **12**

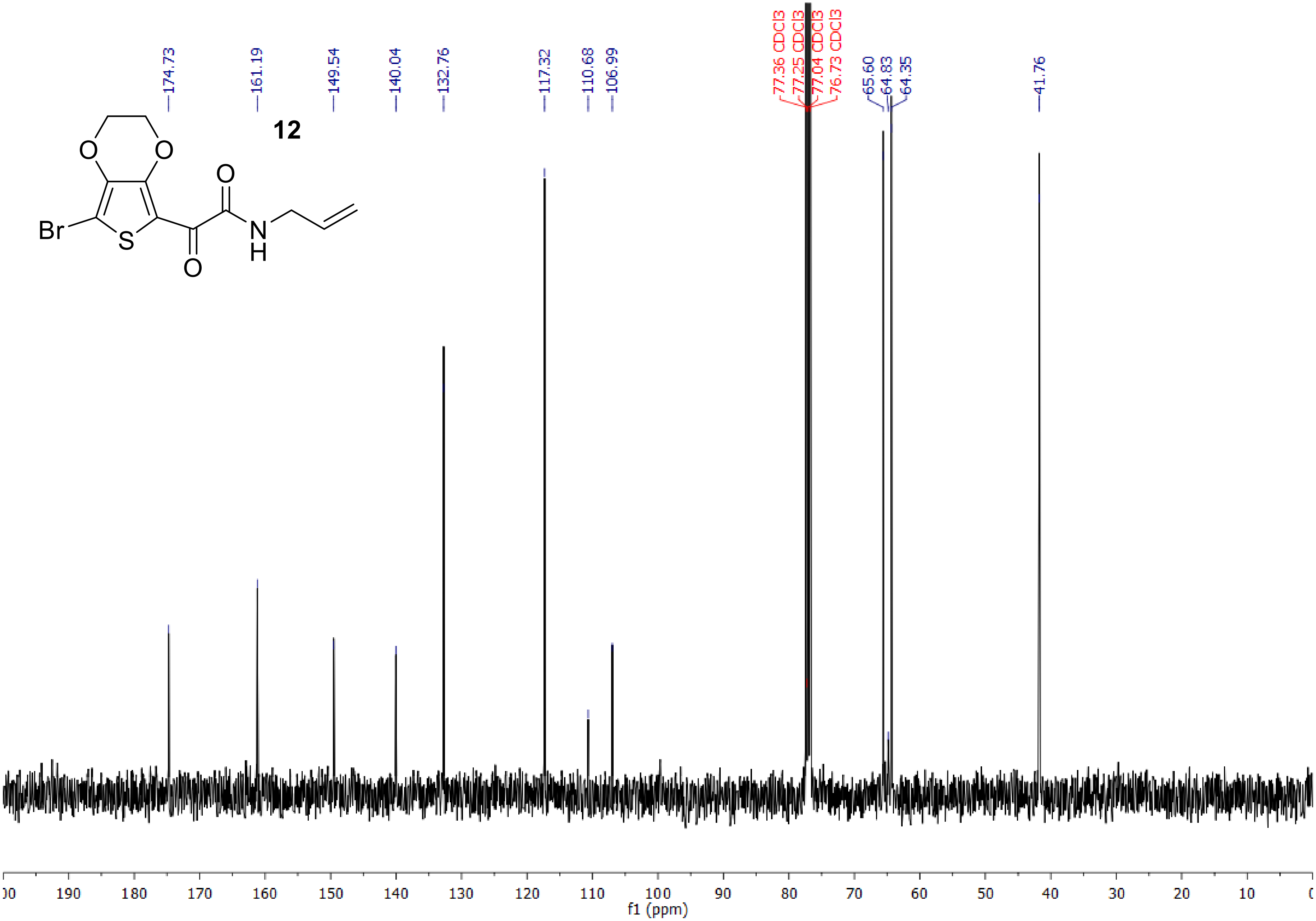

^{1}H NMR (400 MHz, MeOD)

Figure S26. ^{1}H NMR of **13**

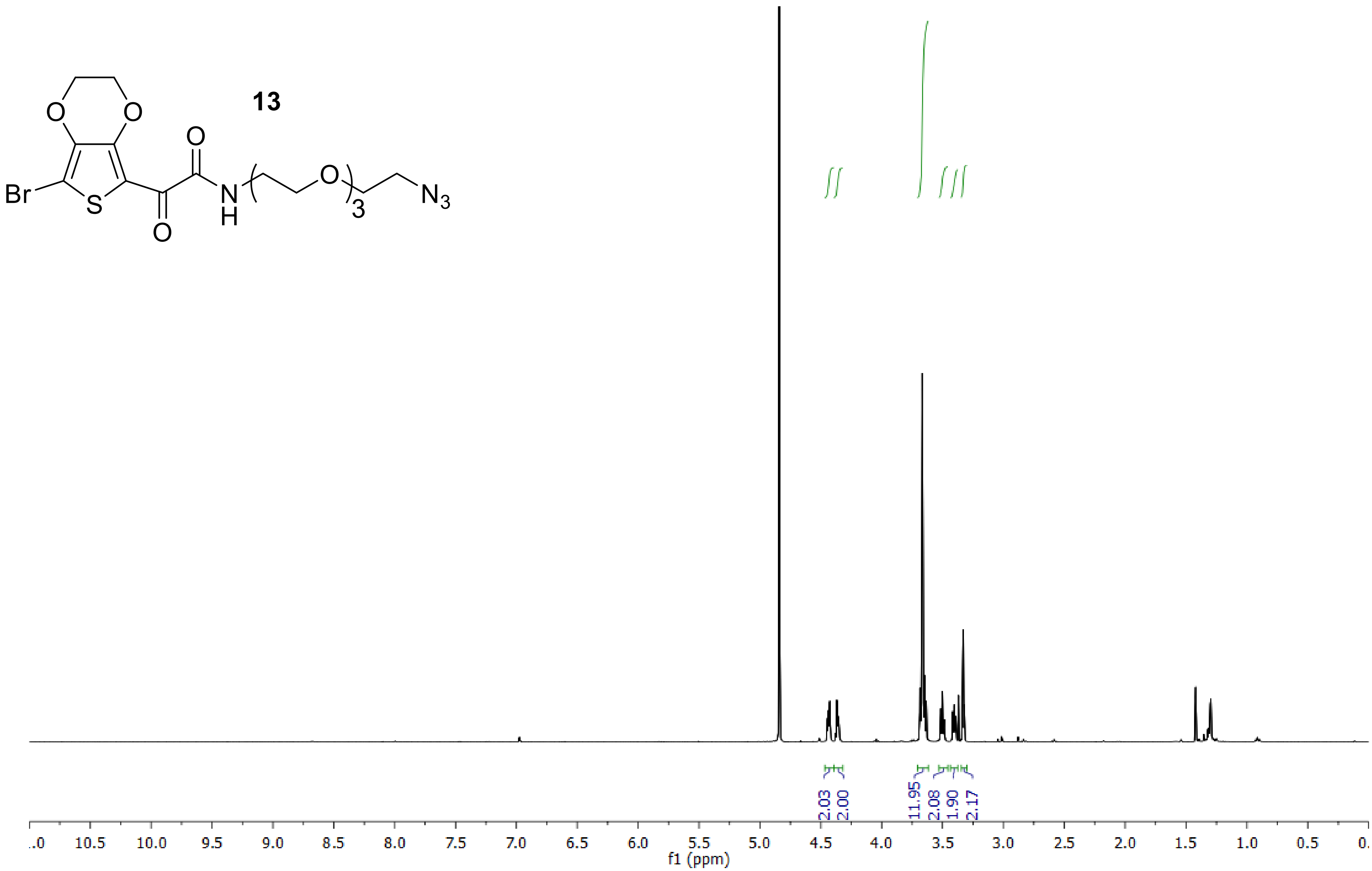

^{13}C NMR (100 MHz, MeOD)

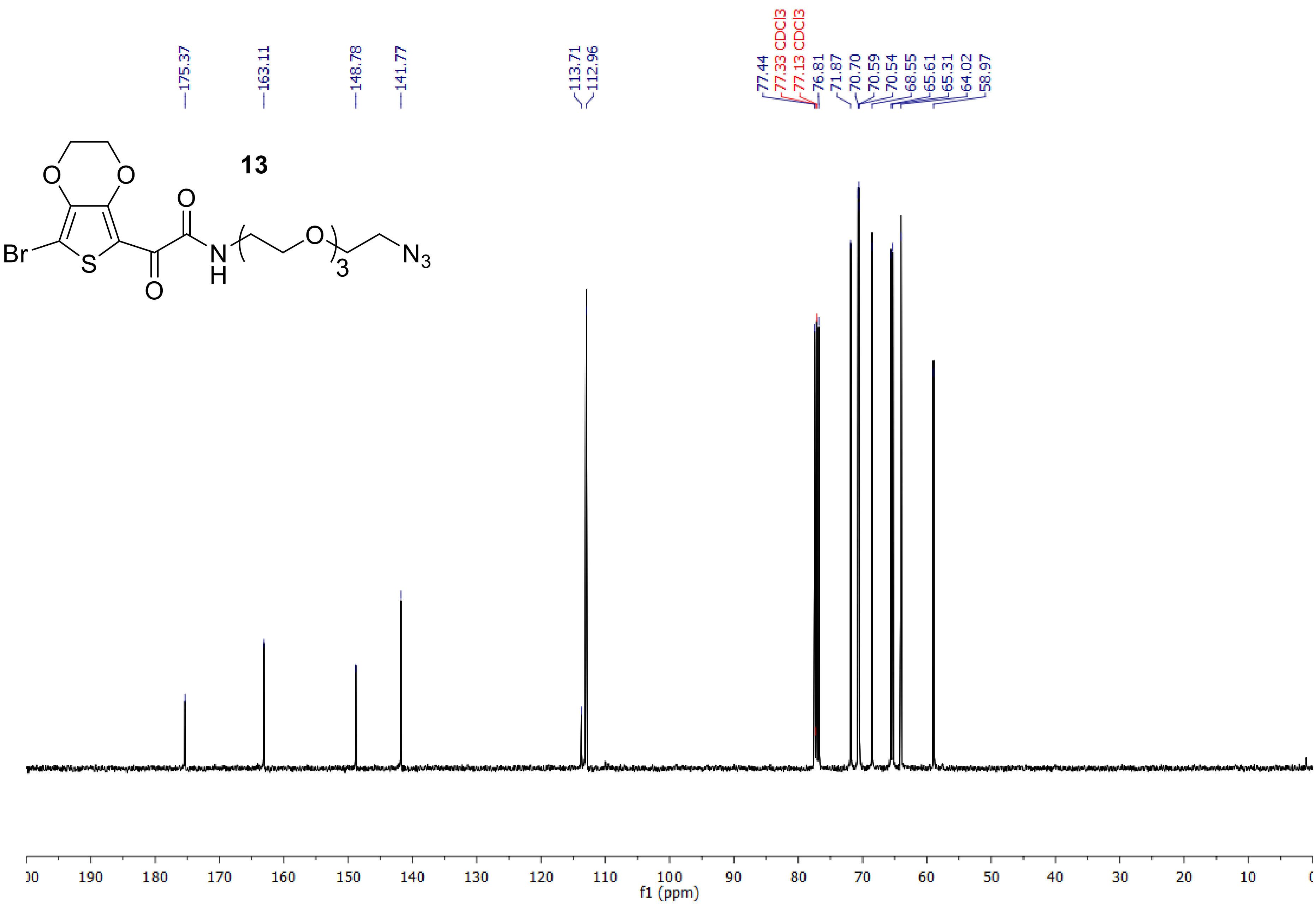

Figure S27. ^{13}C NMR of **13**

^1H NMR (400 MHz, $CDCl_3$)

Figure S28. ^1H NMR of **14**

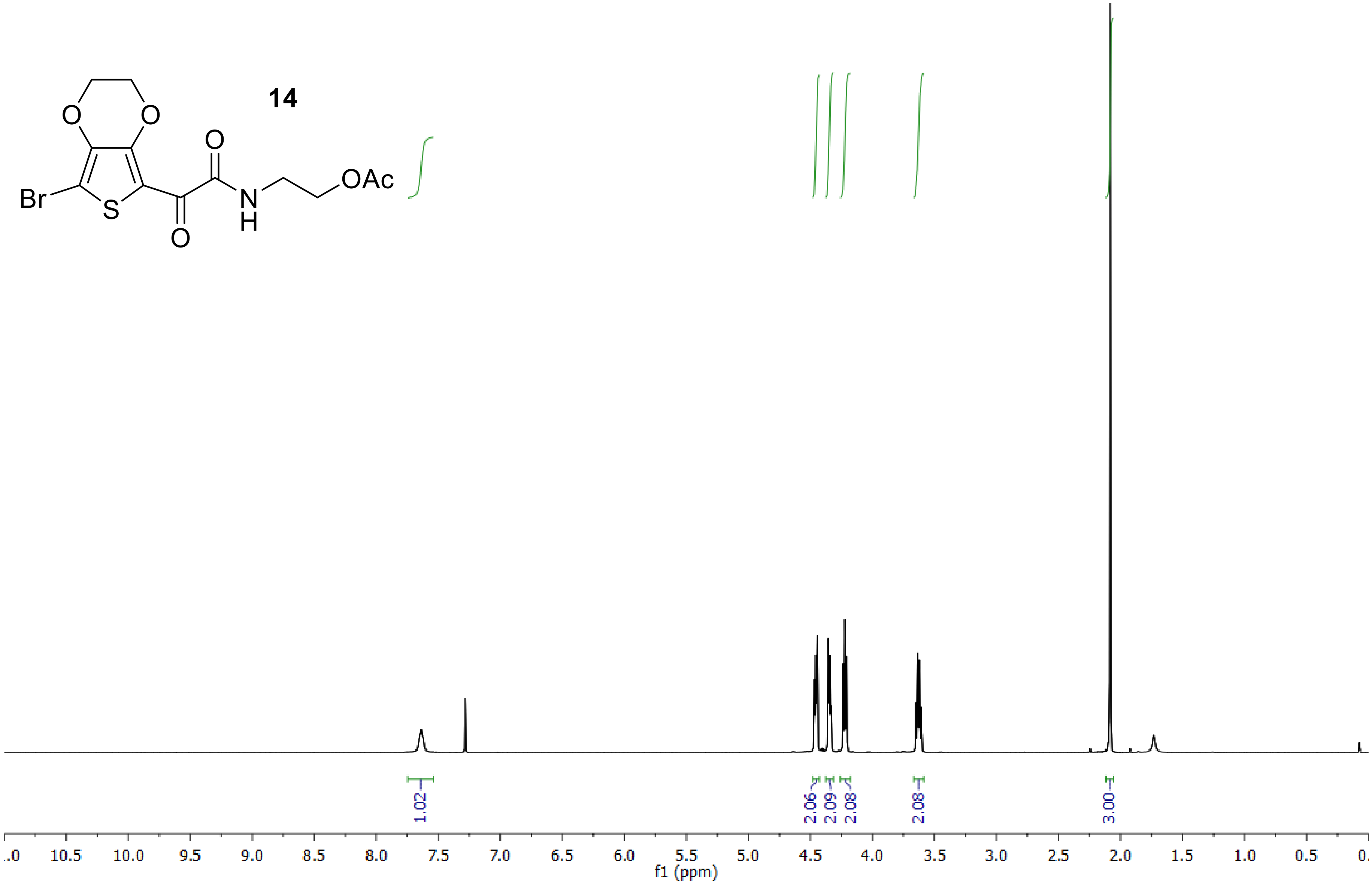

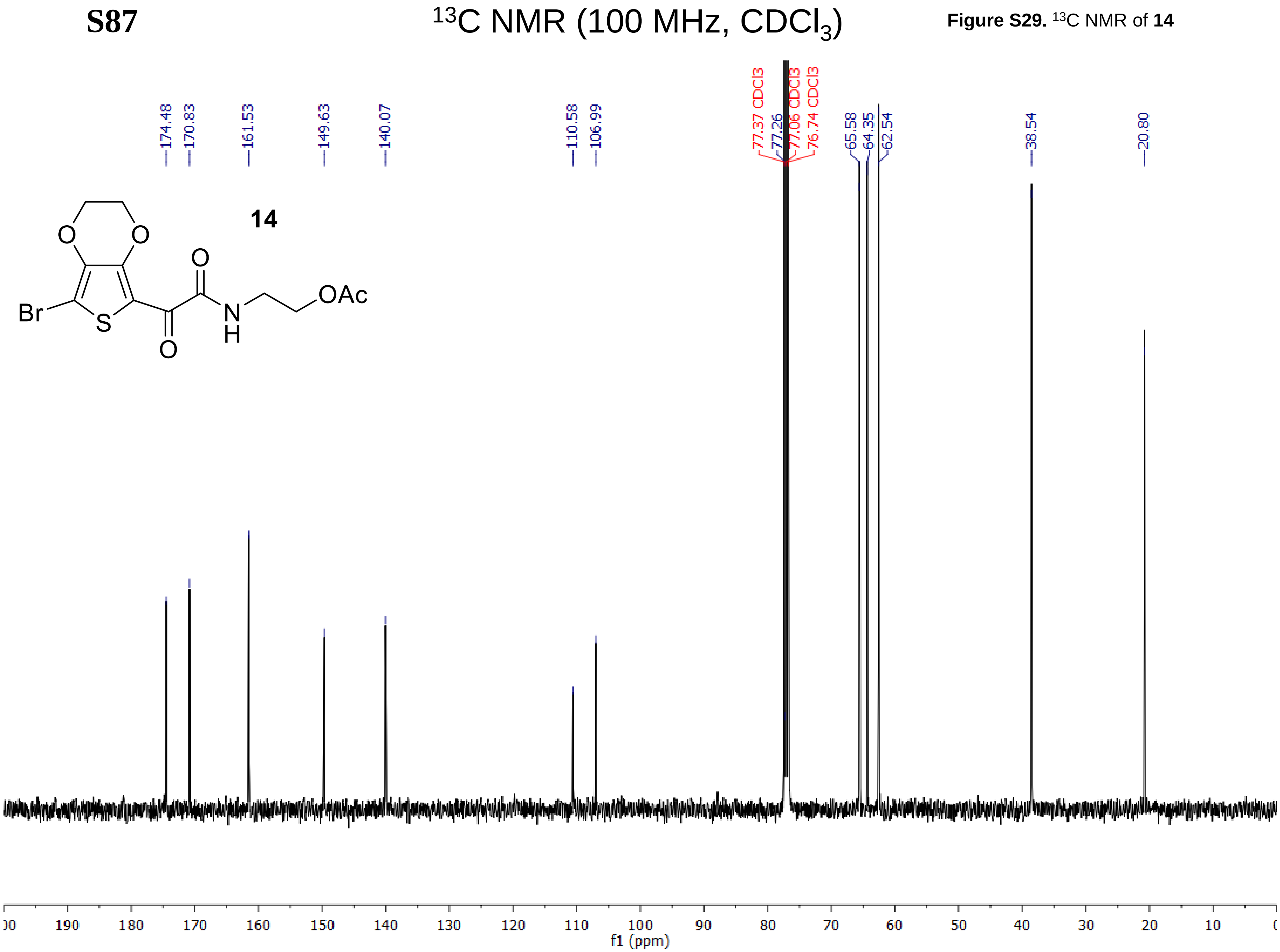

Figure S29. ^{13}C NMR of **14**

^{1}H NMR (400 MHz, DMSO-d$_6$)

Figure S30. ^{1}H NMR of **15**

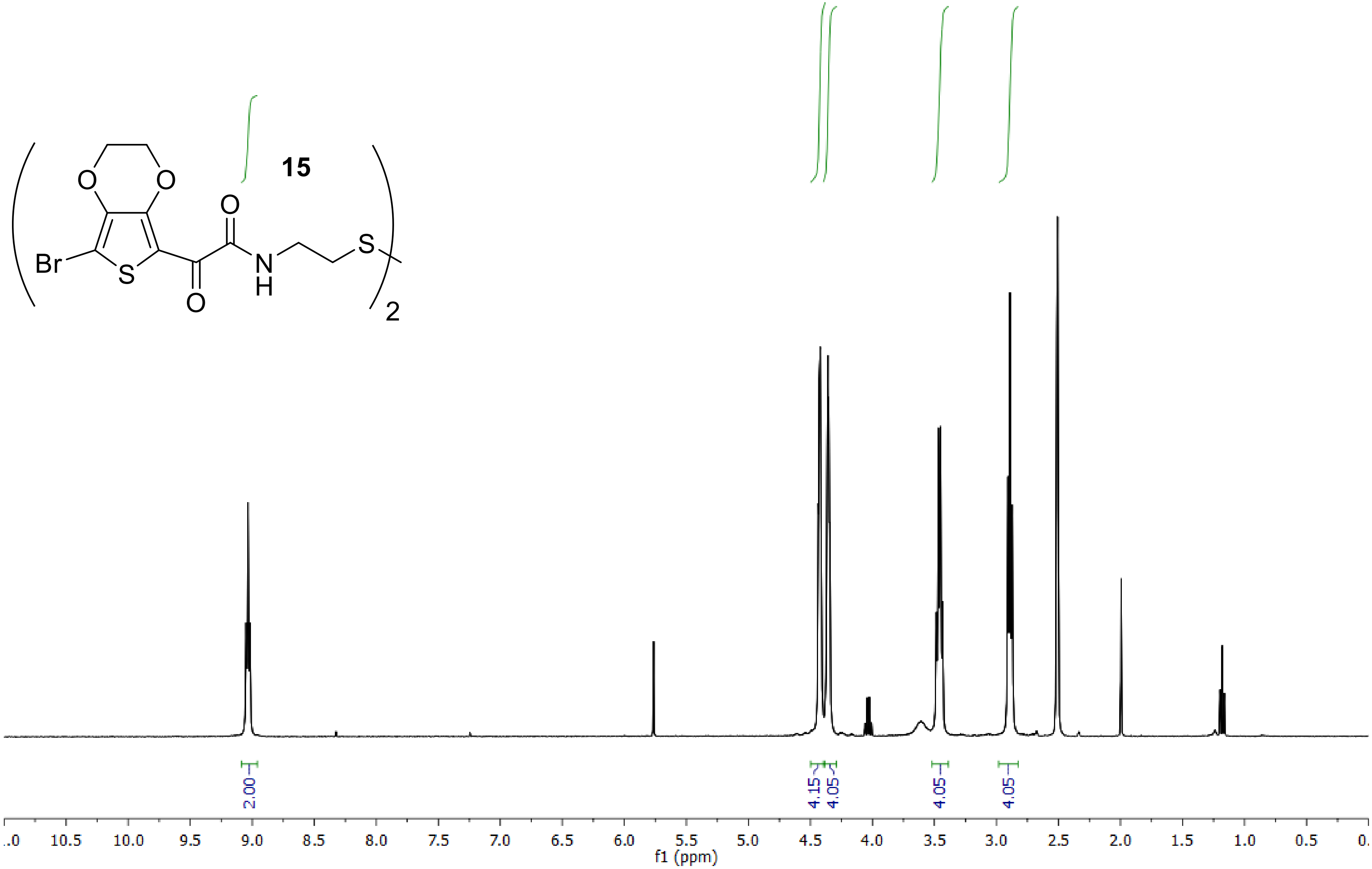

^{13}C NMR (100 MHz, DMSO-d_6)

Figure S31. ^{13}C NMR of **15**

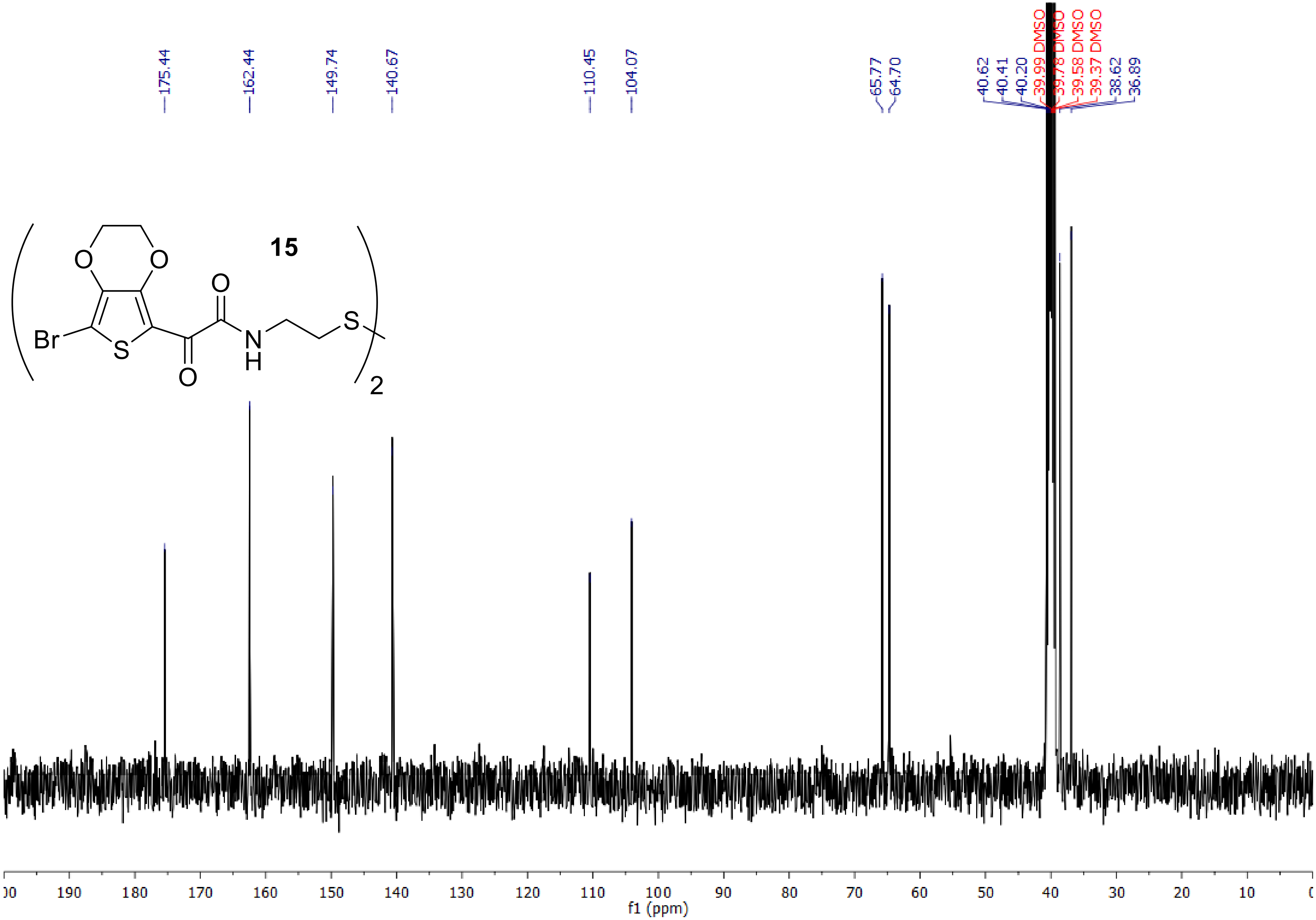

^{1}H NMR (400 MHz, $CDCl_3$)

Figure S32. ^{1}H NMR of **16**

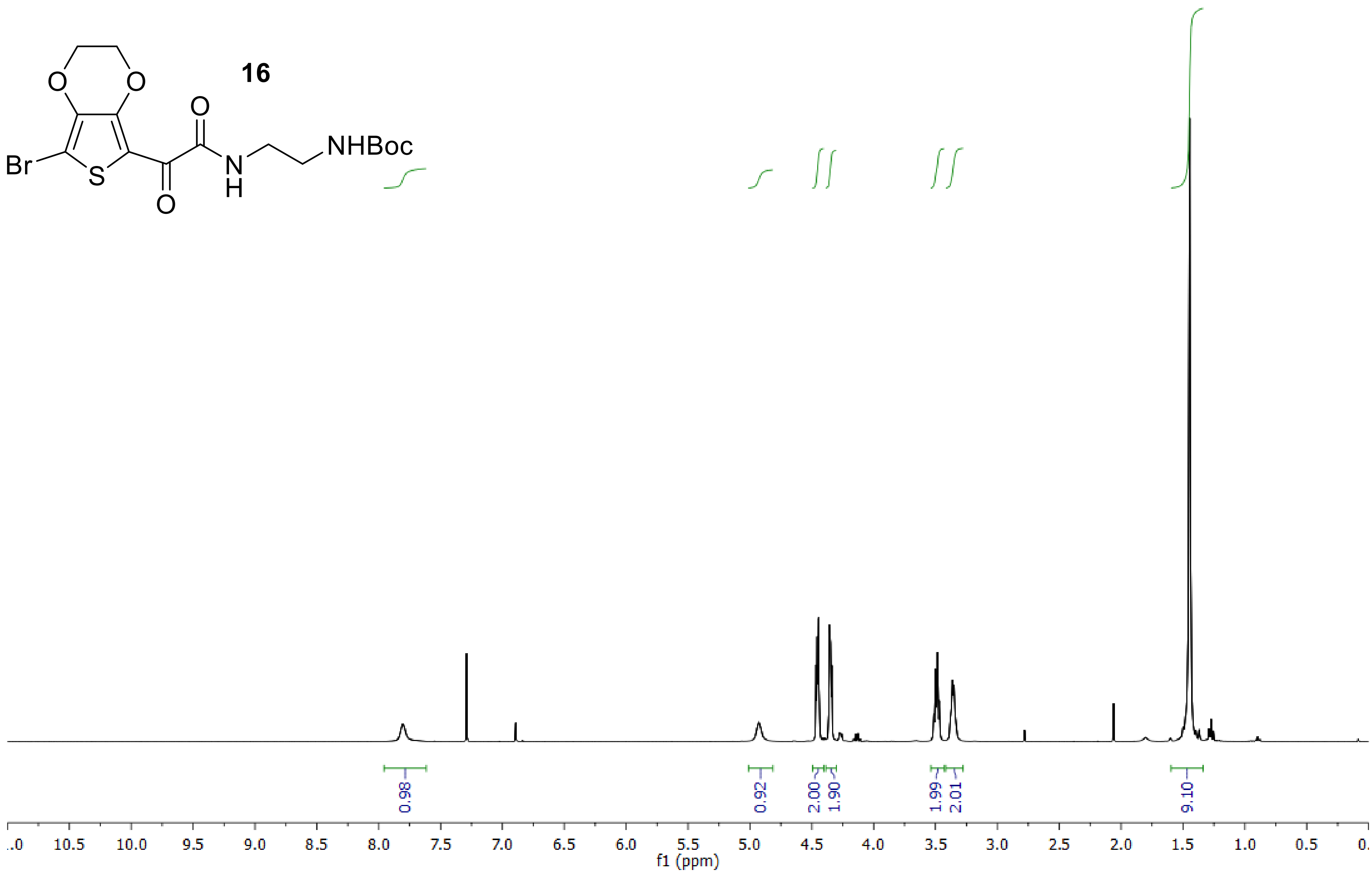

^{13}C NMR (100 MHz, $CDCl_3$)

Figure S33. ^{13}C NMR of **16**

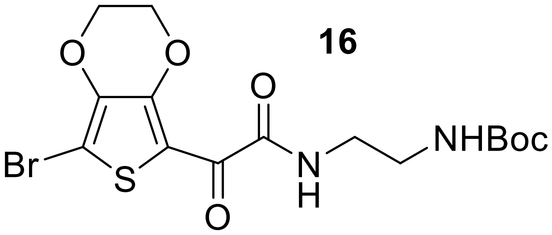

16

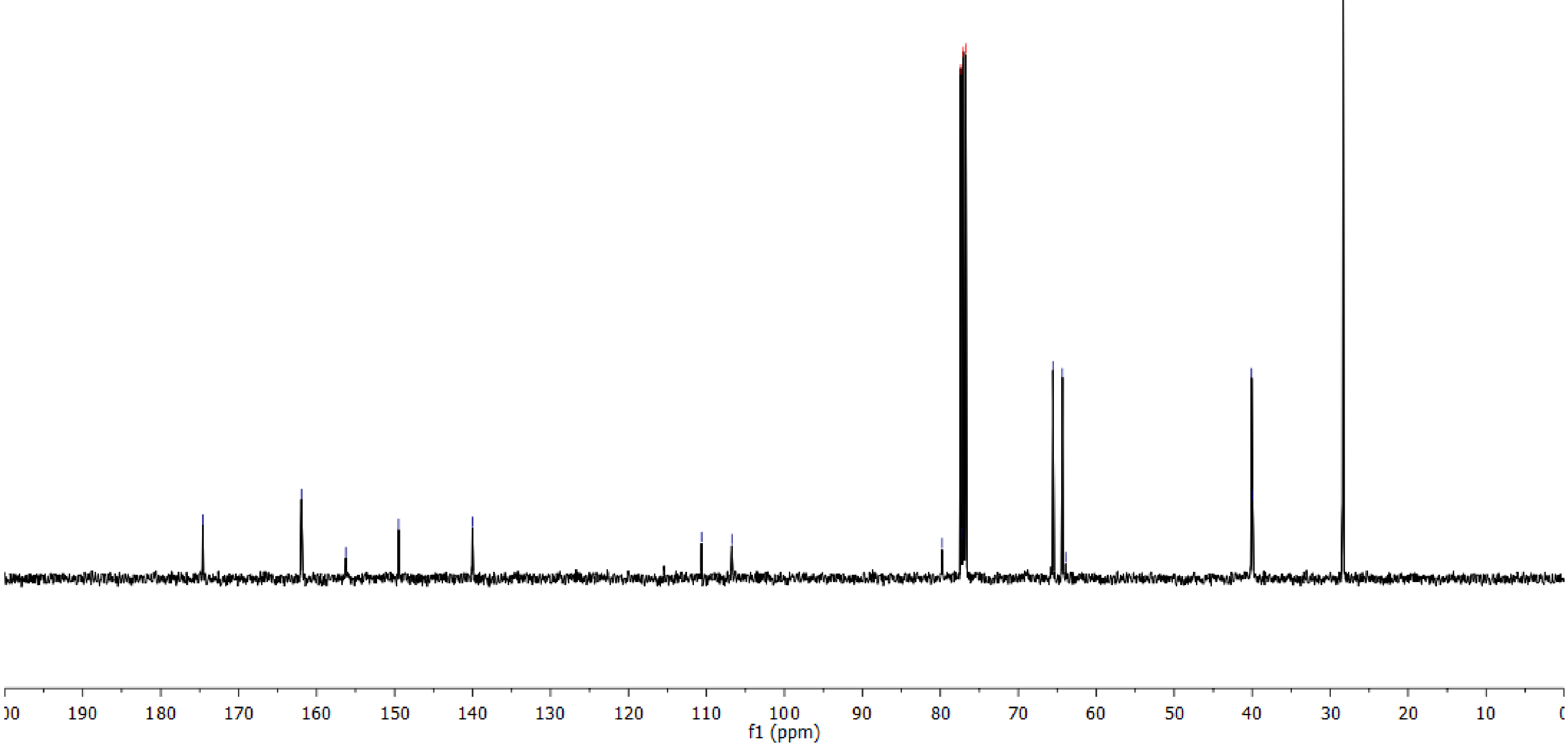

^{1}H NMR (400 MHz, $CDCl_3$)

Figure S34. ^{1}H NMR of **17**

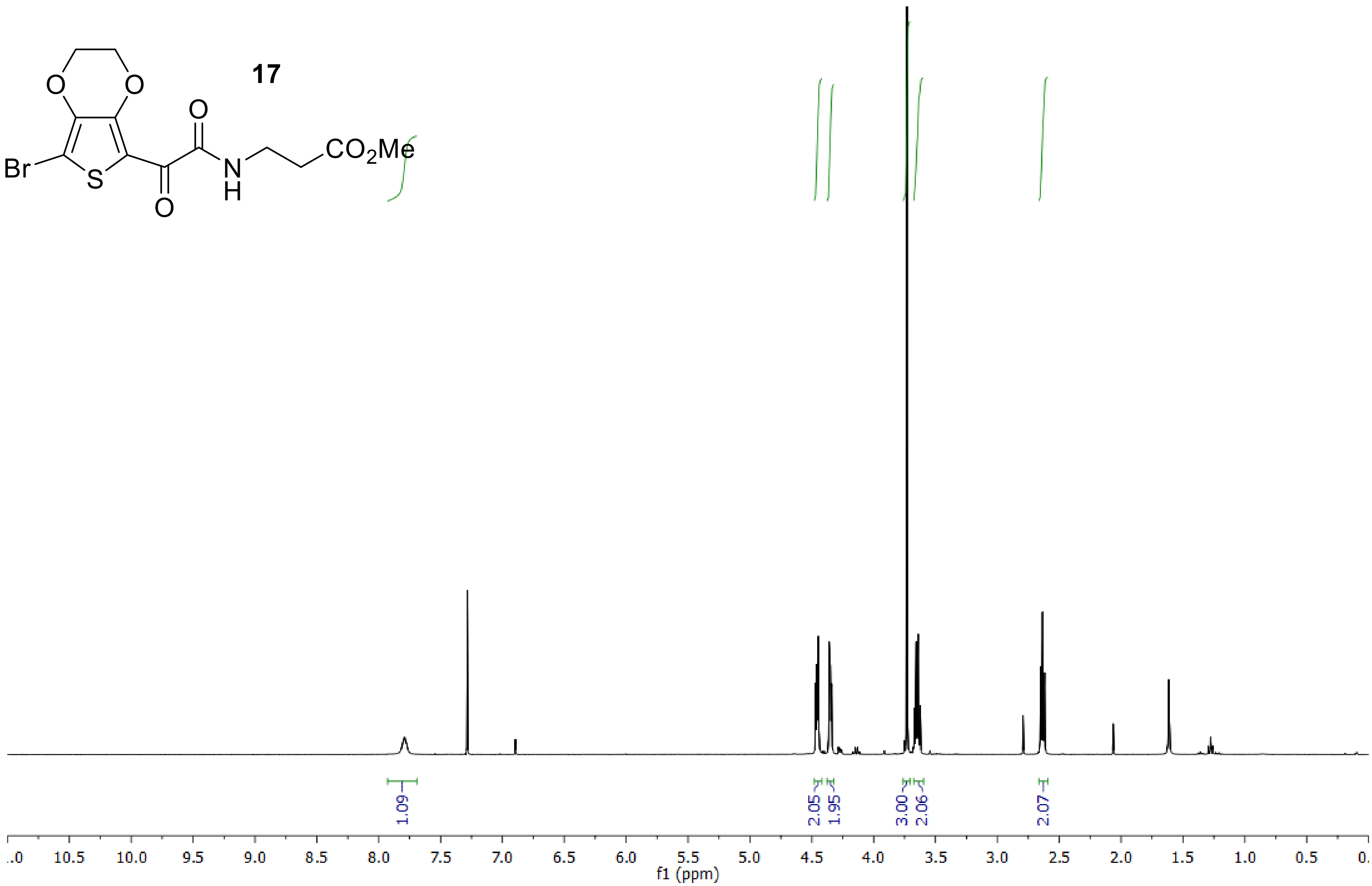

^{13}C NMR (100 MHz, $CDCl_3$)

Figure S35. ^{13}C NMR of **17**

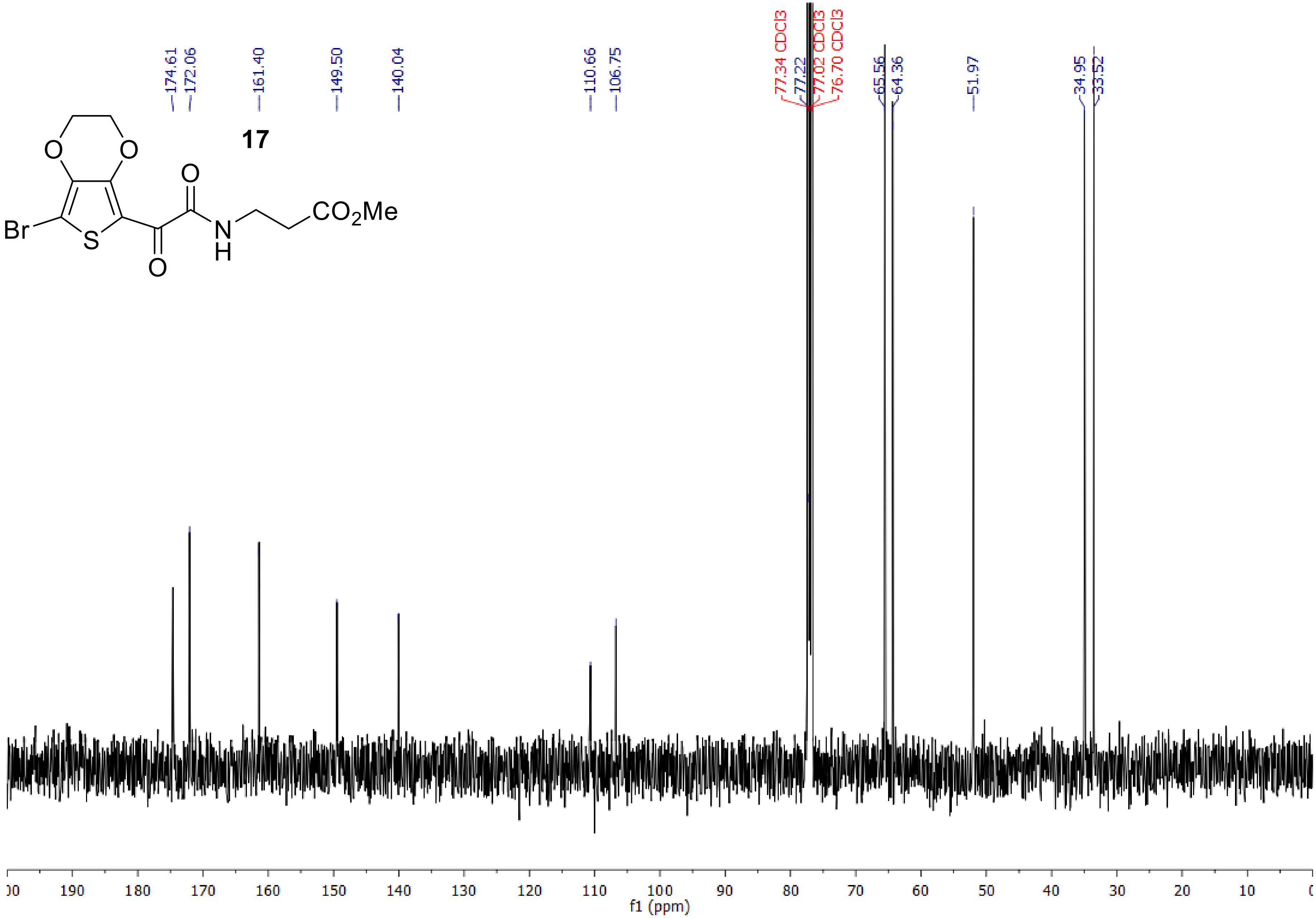

^{1}H NMR (400 MHz, $CDCl_3$)

Figure S36. ^{1}H NMR of **18**

18

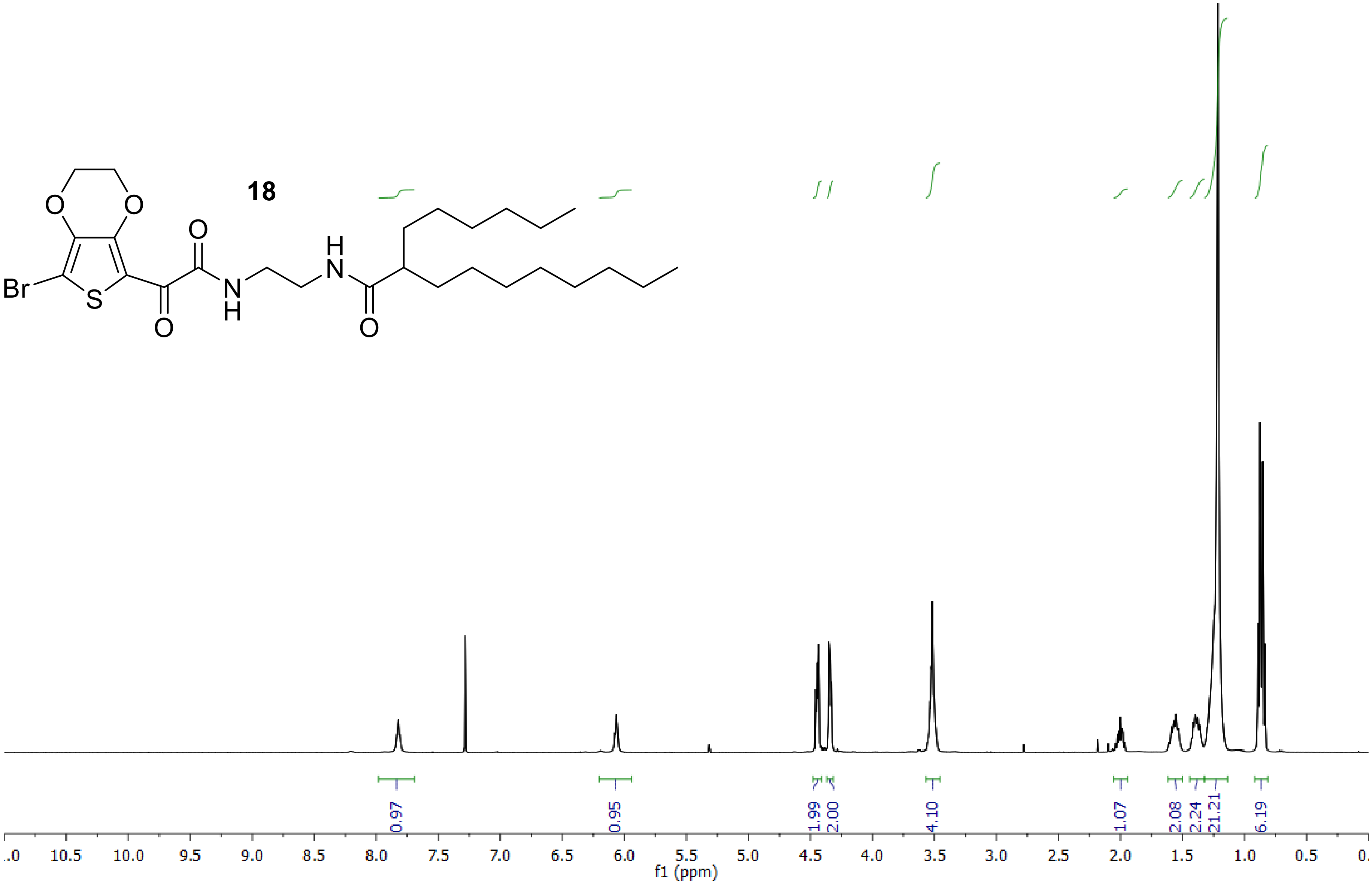

^{13}C NMR (100 MHz, $CDCl_3$)

Figure S37. ^{13}C NMR of **18**

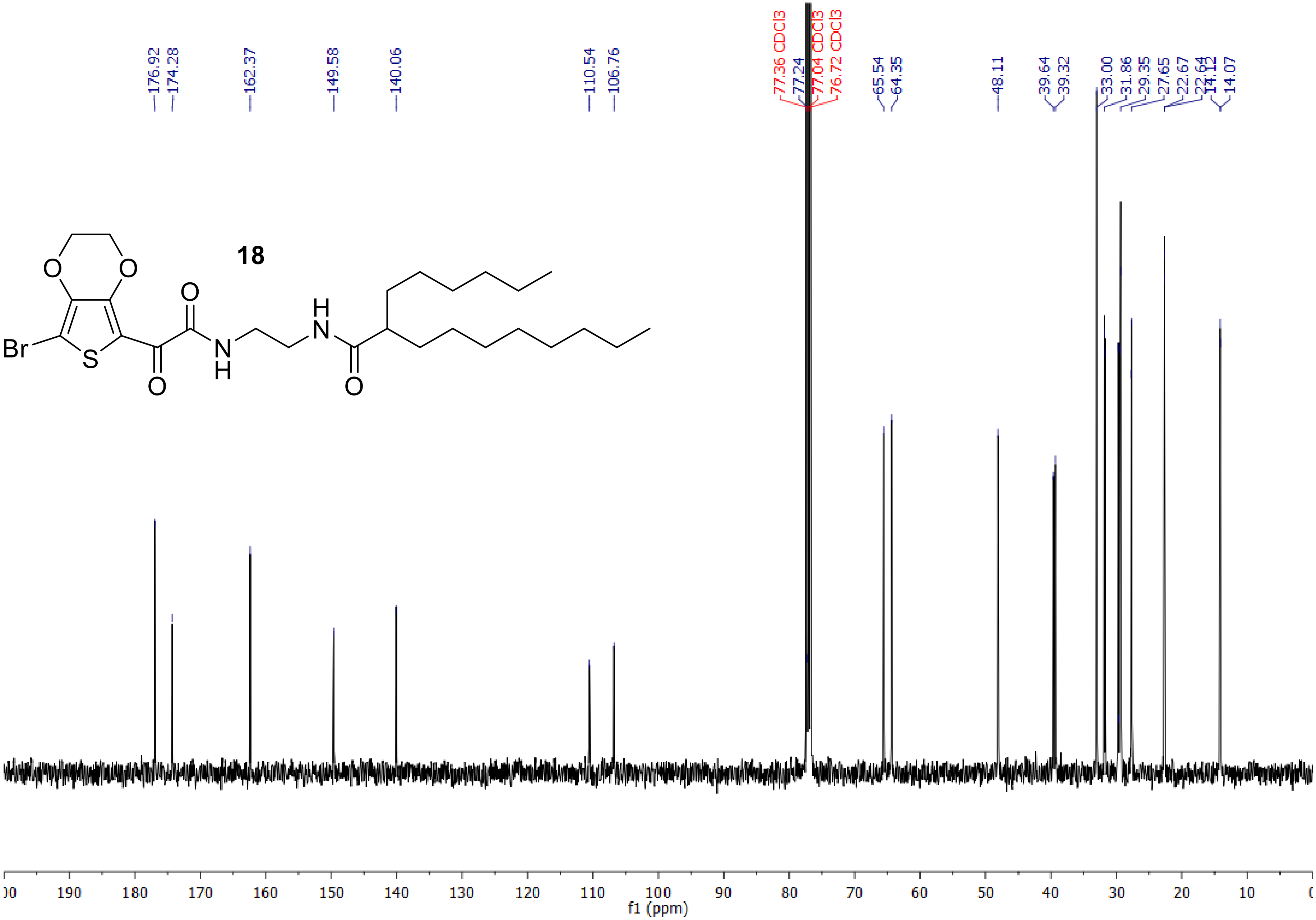

¹H NMR (400 MHz, $CDCl_3$)

Figure S38. ^{1}H NMR of **19**

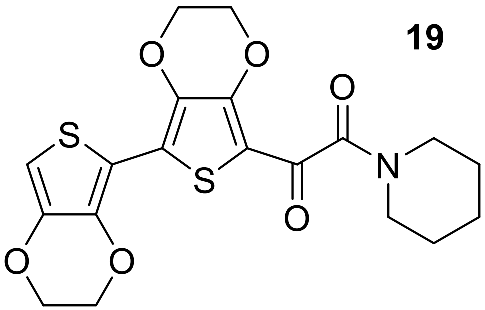

19

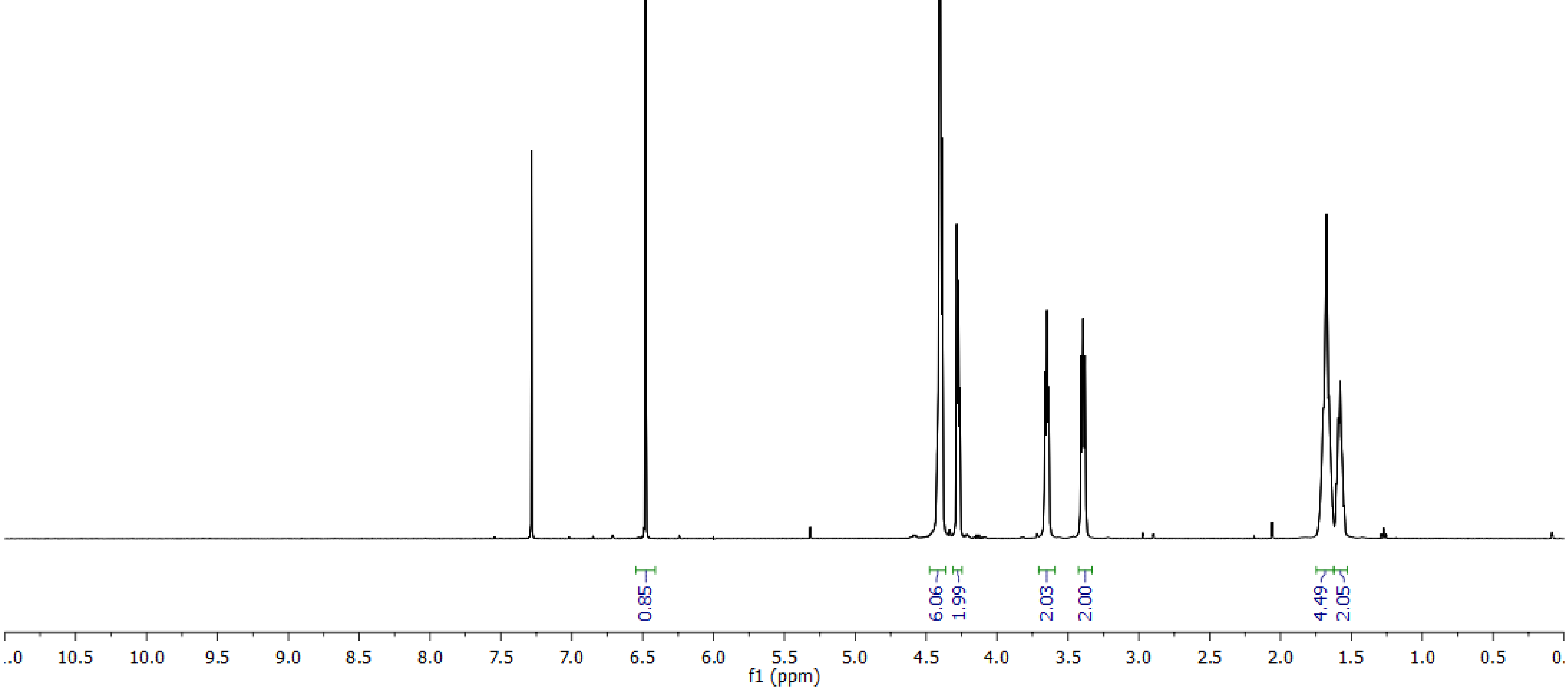

^{13}C NMR (100 MHz, $CDCl_3$)

Figure S39. ^{13}C NMR of **19**

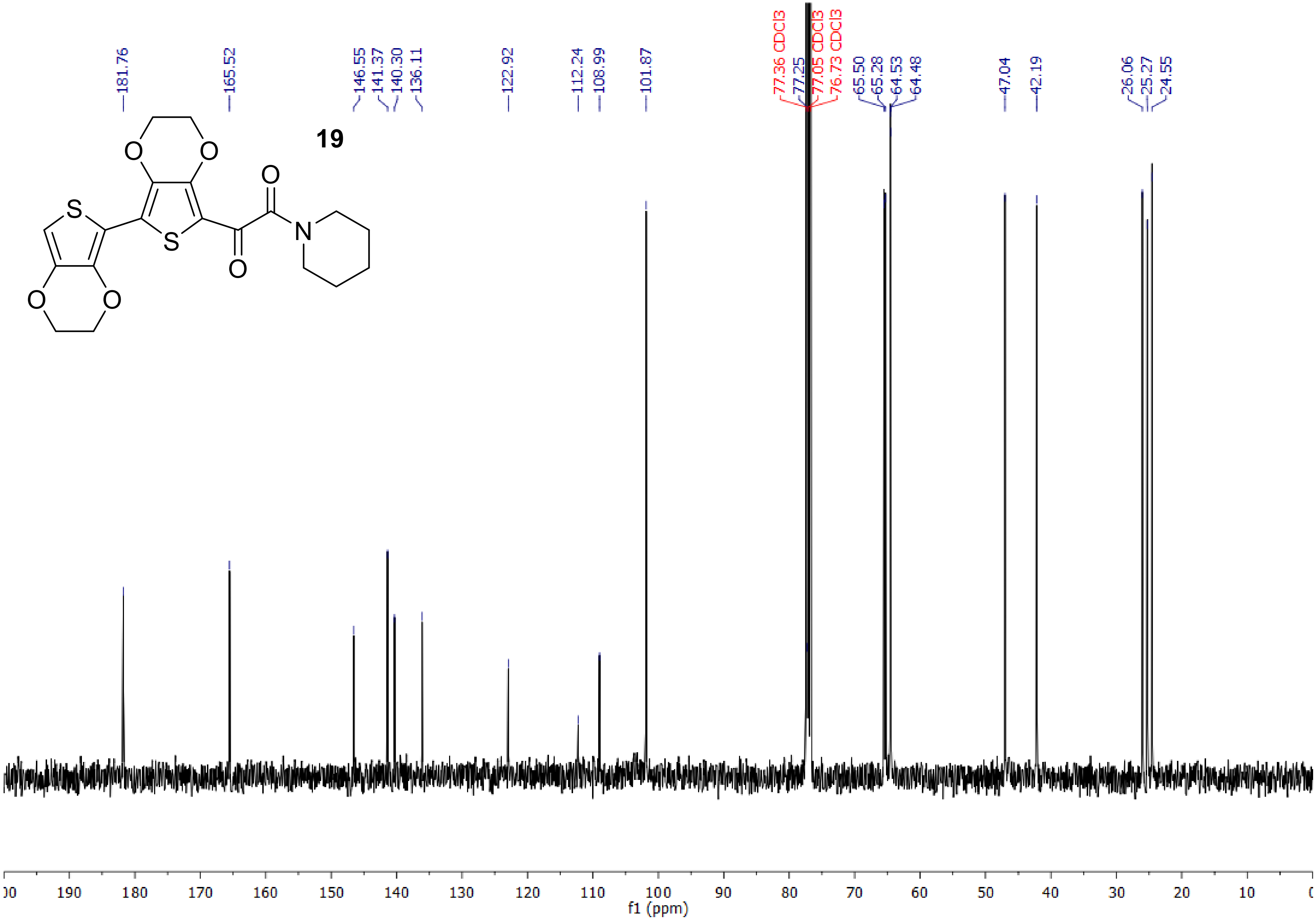

^{1}H NMR (400 MHz, $CDCl_3$)

Figure S40. ^{1}H NMR of **98**

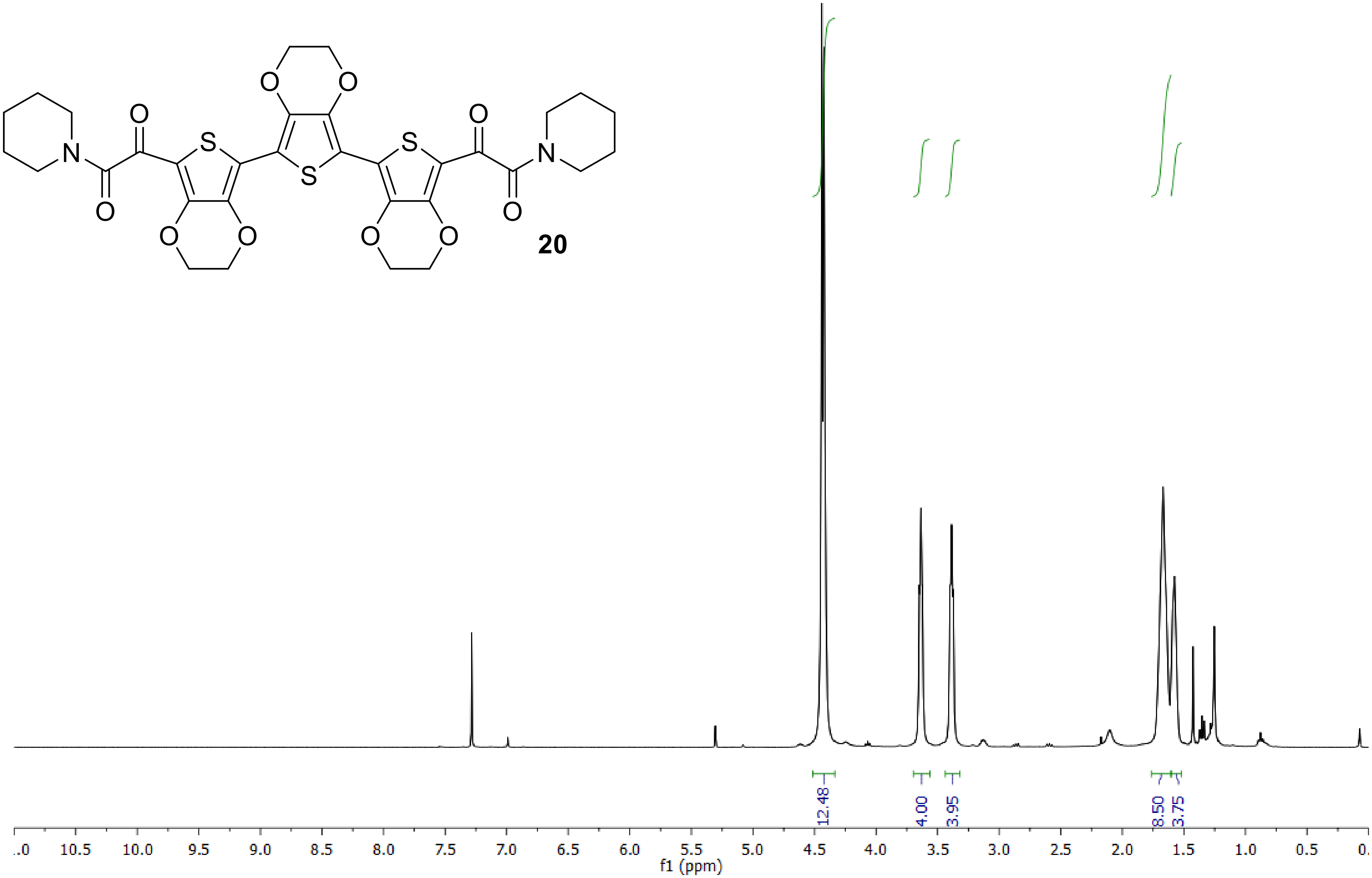

^{13}C NMR (100 MHz, $CDCl_3$)

Figure S41. ^{13}C NMR of **20**

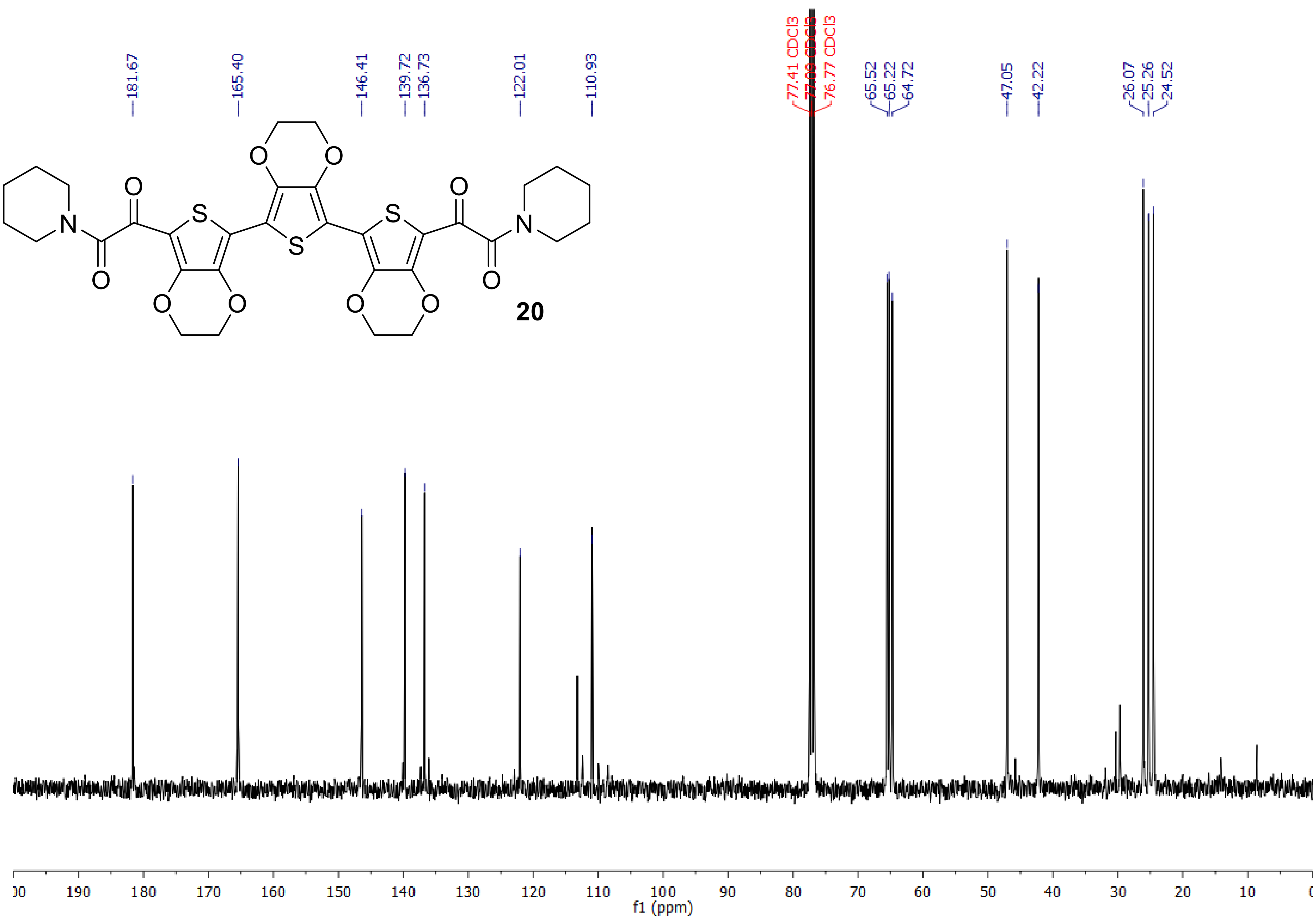

^{1}H NMR (400 MHz, $CDCl_3$)

Figure S42. ^{1}H NMR of **21**

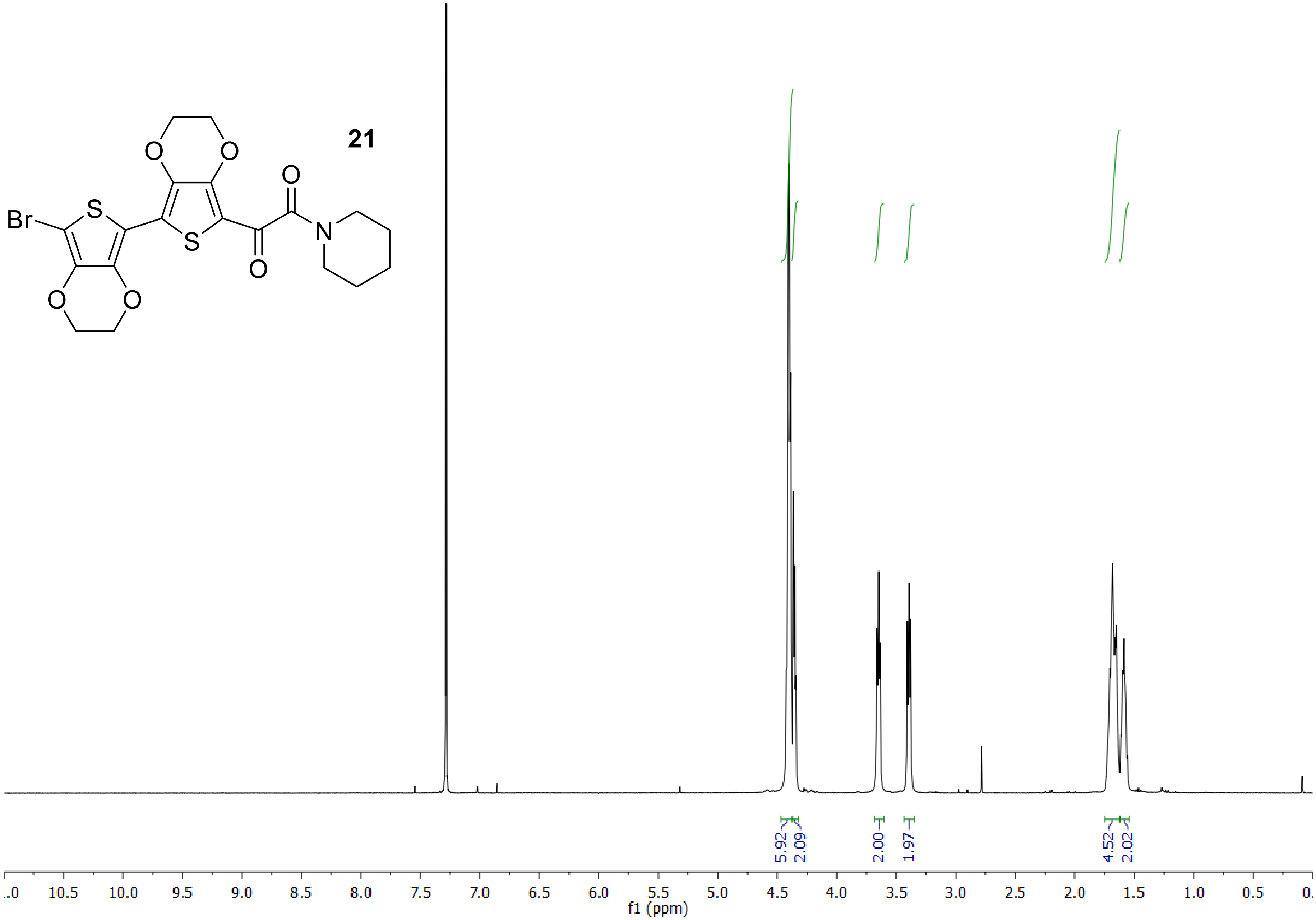

^{13}C NMR (100 MHz, $CDCl_3$)

Figure S43. ^{13}C NMR of **21**

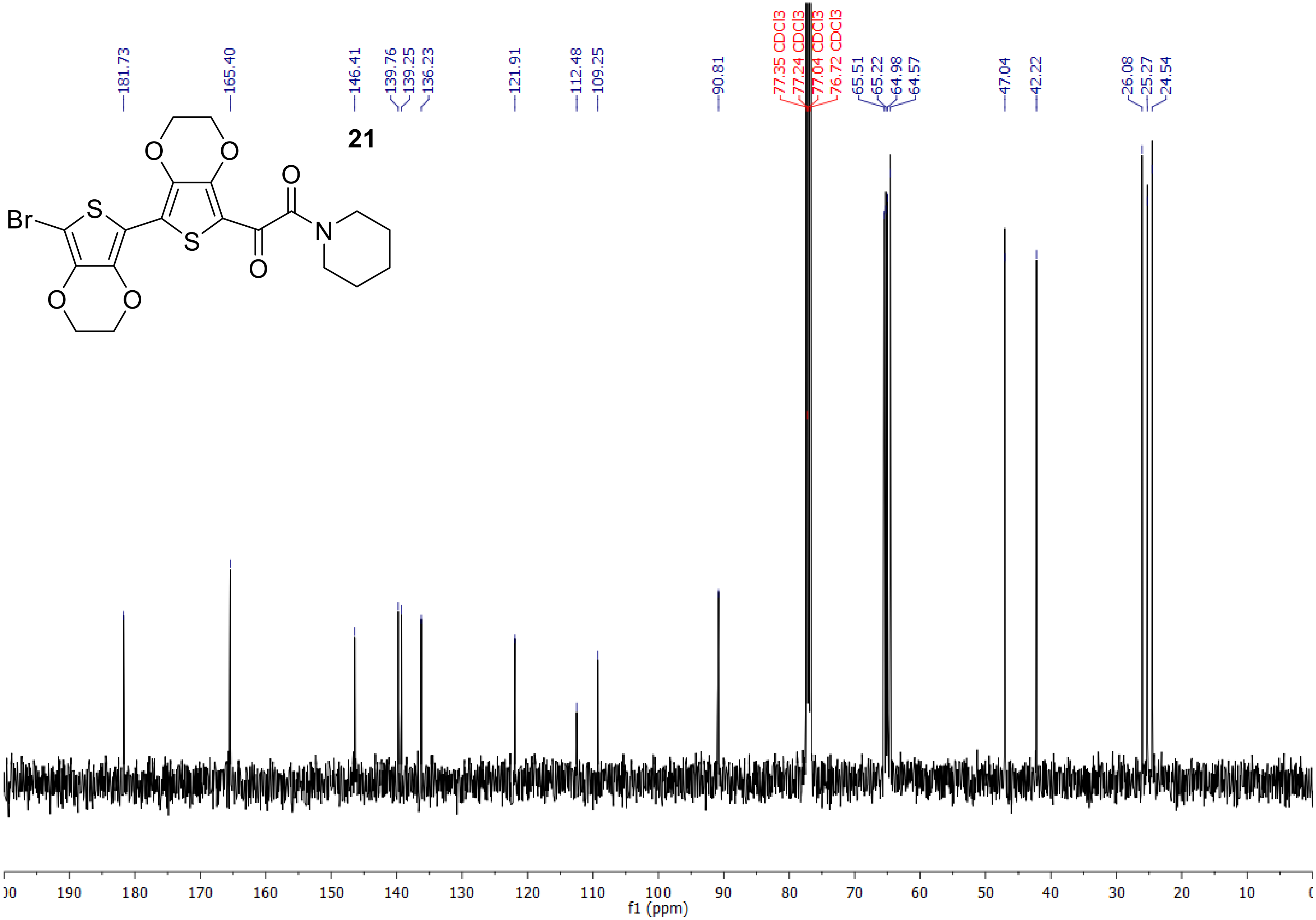

^1H NMR (400 MHz, $CDCl_3$)

Figure S44. ^1H NMR of **22**

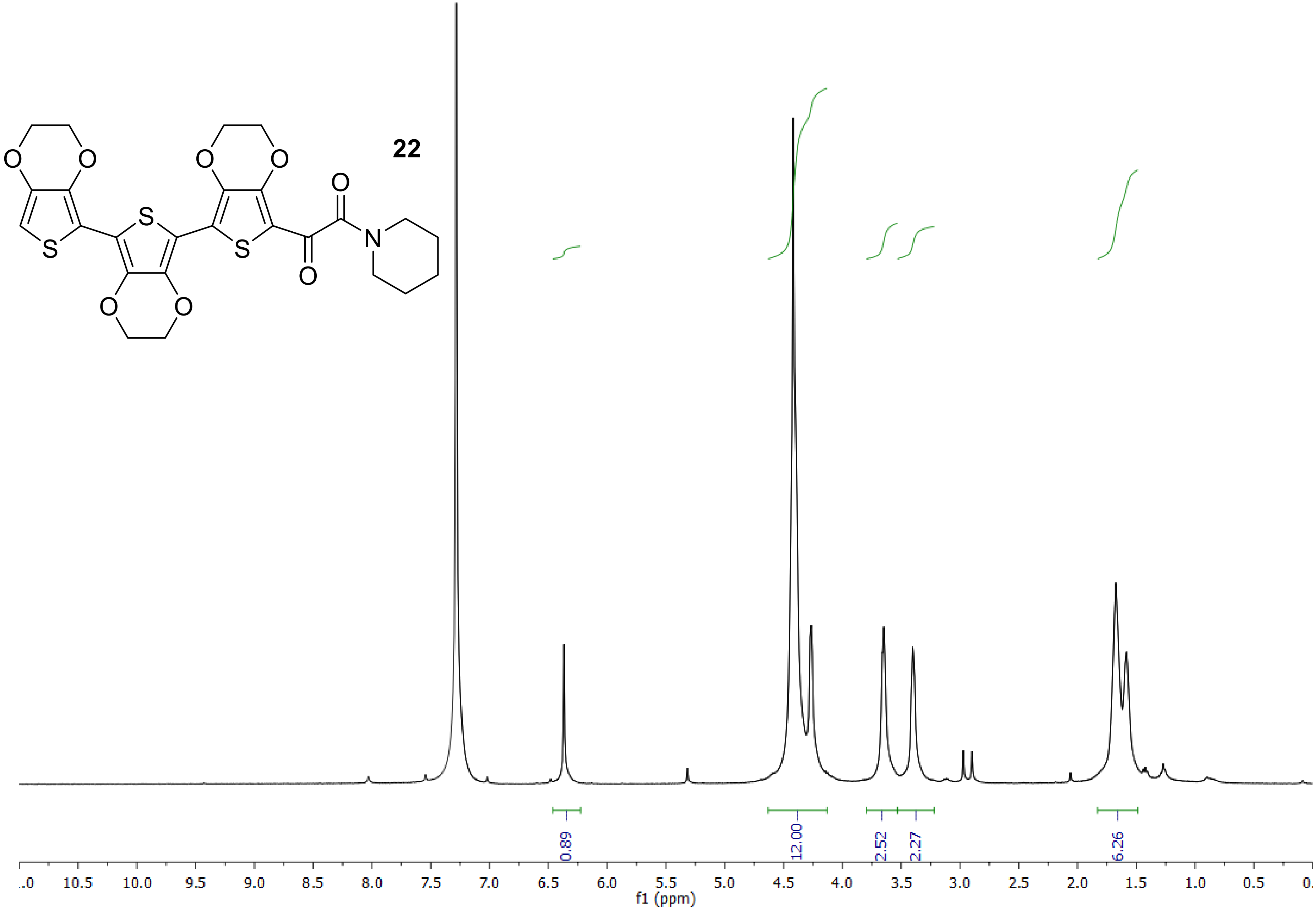

^{13}C NMR (100 MHz, $CDCl_3$)

Figure S45. ^{13}C NMR of **22**

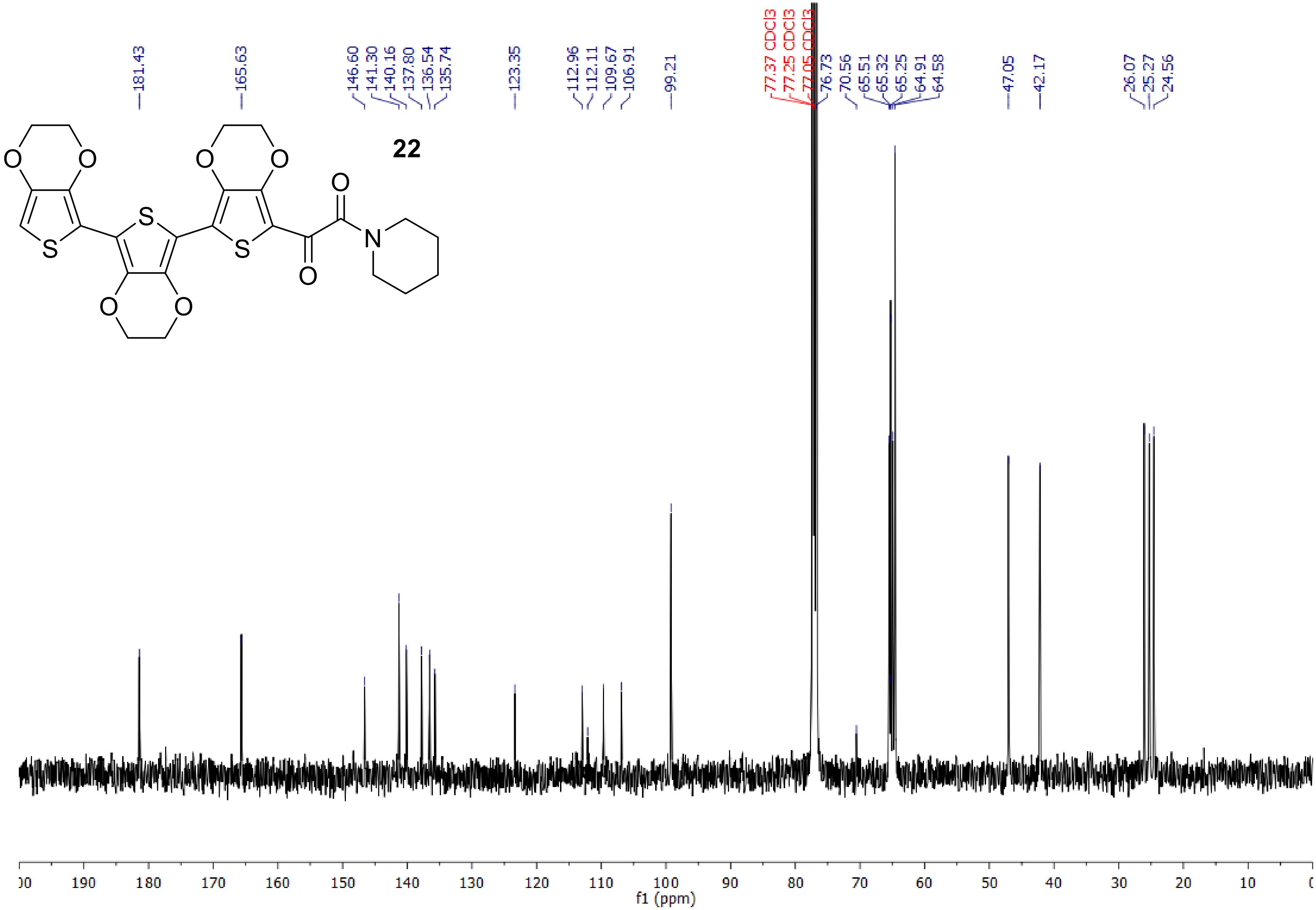

^{1}H NMR (400 MHz, $CDCl_3$)

Figure S46. ^{1}H NMR of **24**

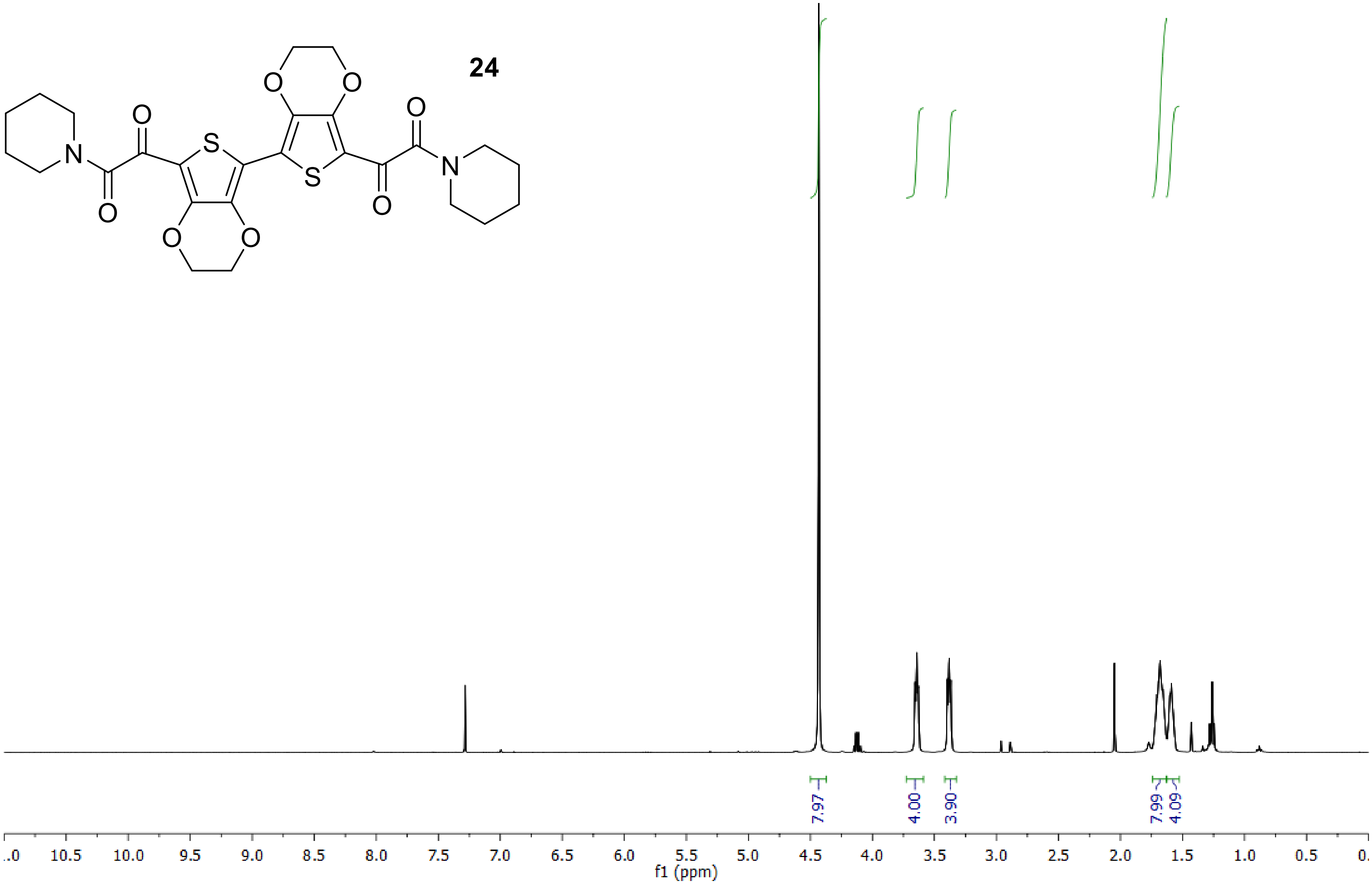

^{13}C NMR (100 MHz, $CDCl_3$)

Figure S47. ^{13}C NMR of **24**

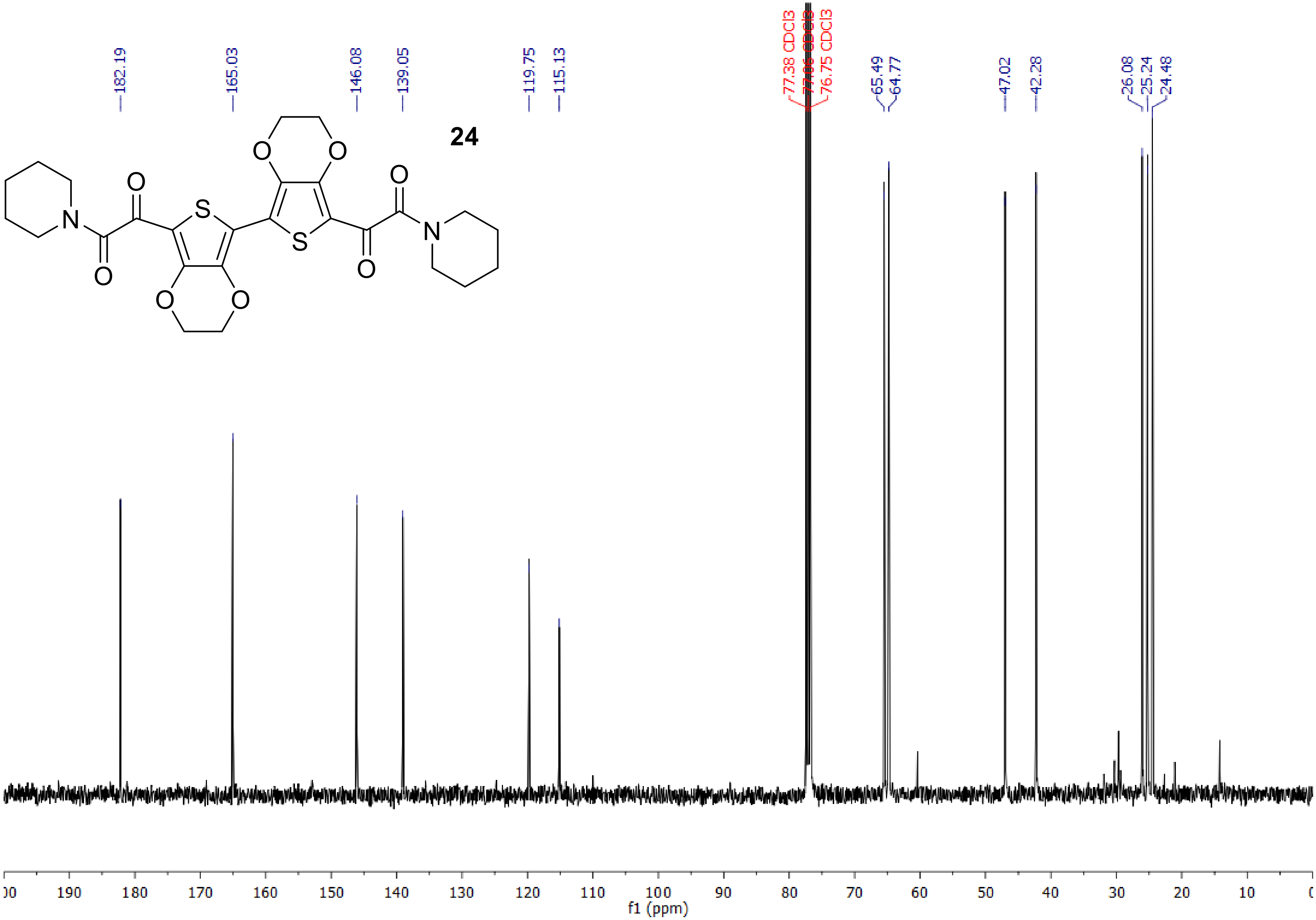

^{1}H NMR (400 MHz, $CDCl_3$)

Figure S48. ^{1}H NMR of **26**

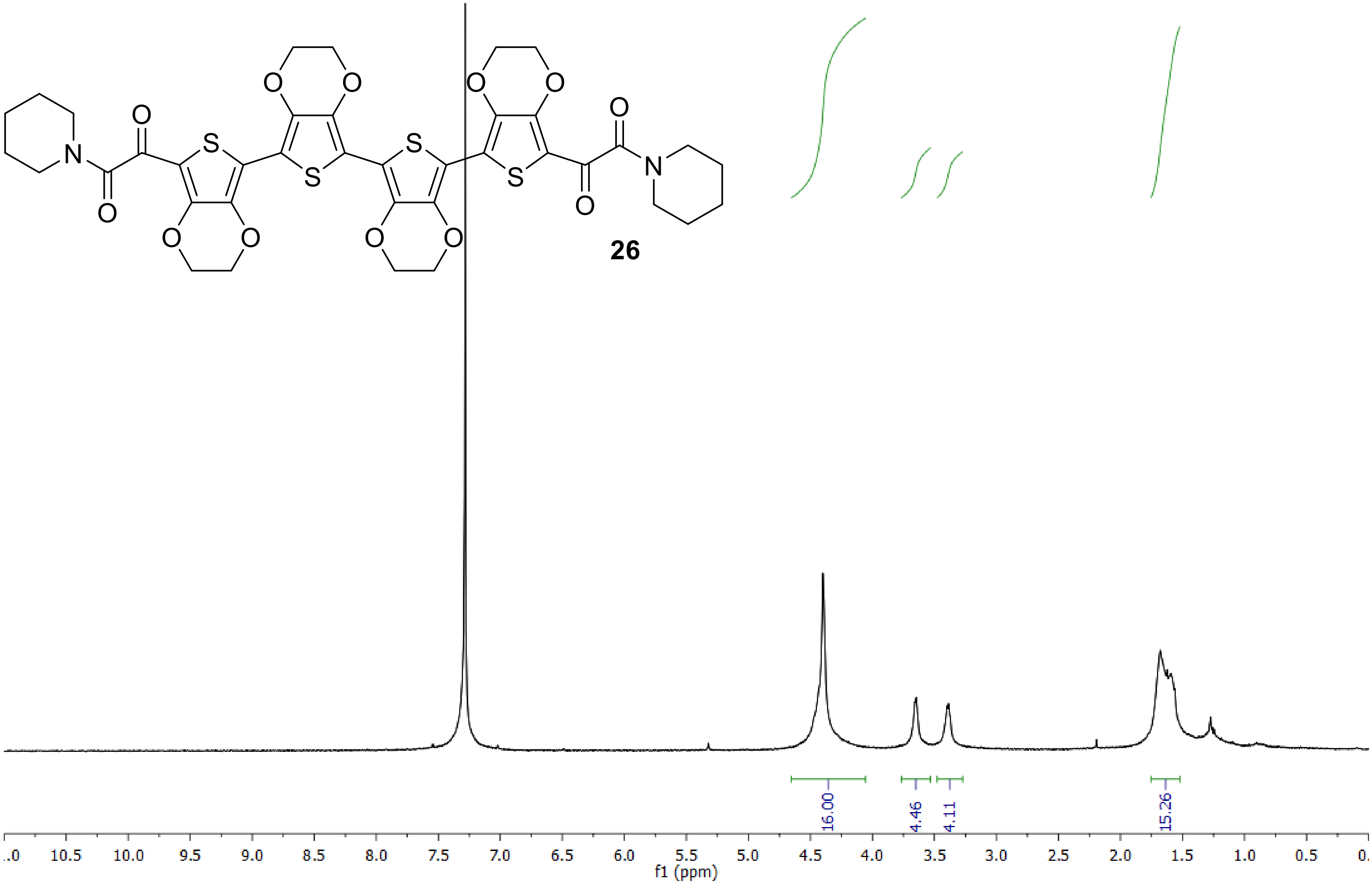

^{13}C NMR (125 MHz, $CDCl_3$)

Figure S49. ^{13}C NMR of **26**

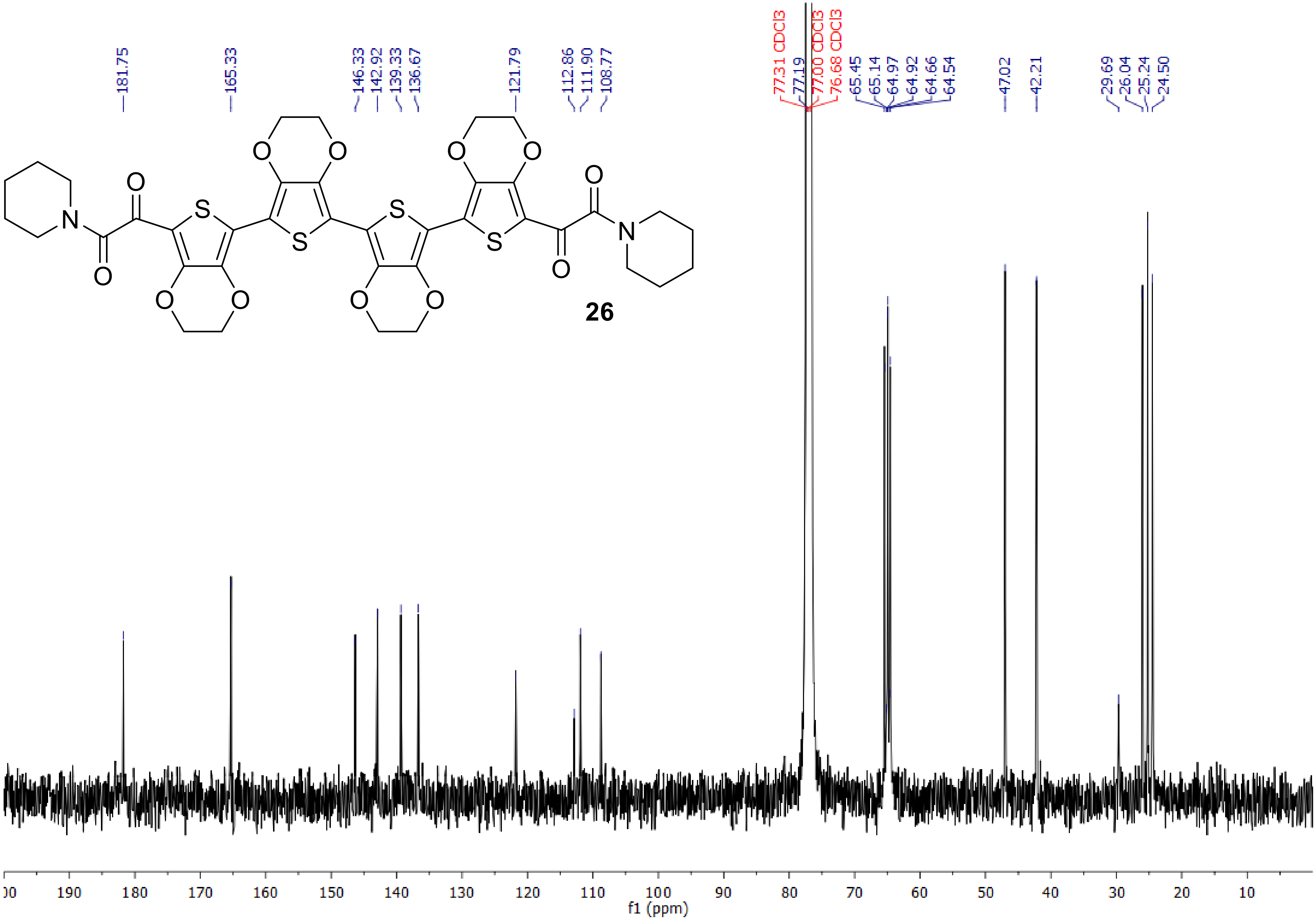

^1H NMR (400 MHz, $CDCl_3$)

Figure S50. ^1H NMR of **27**

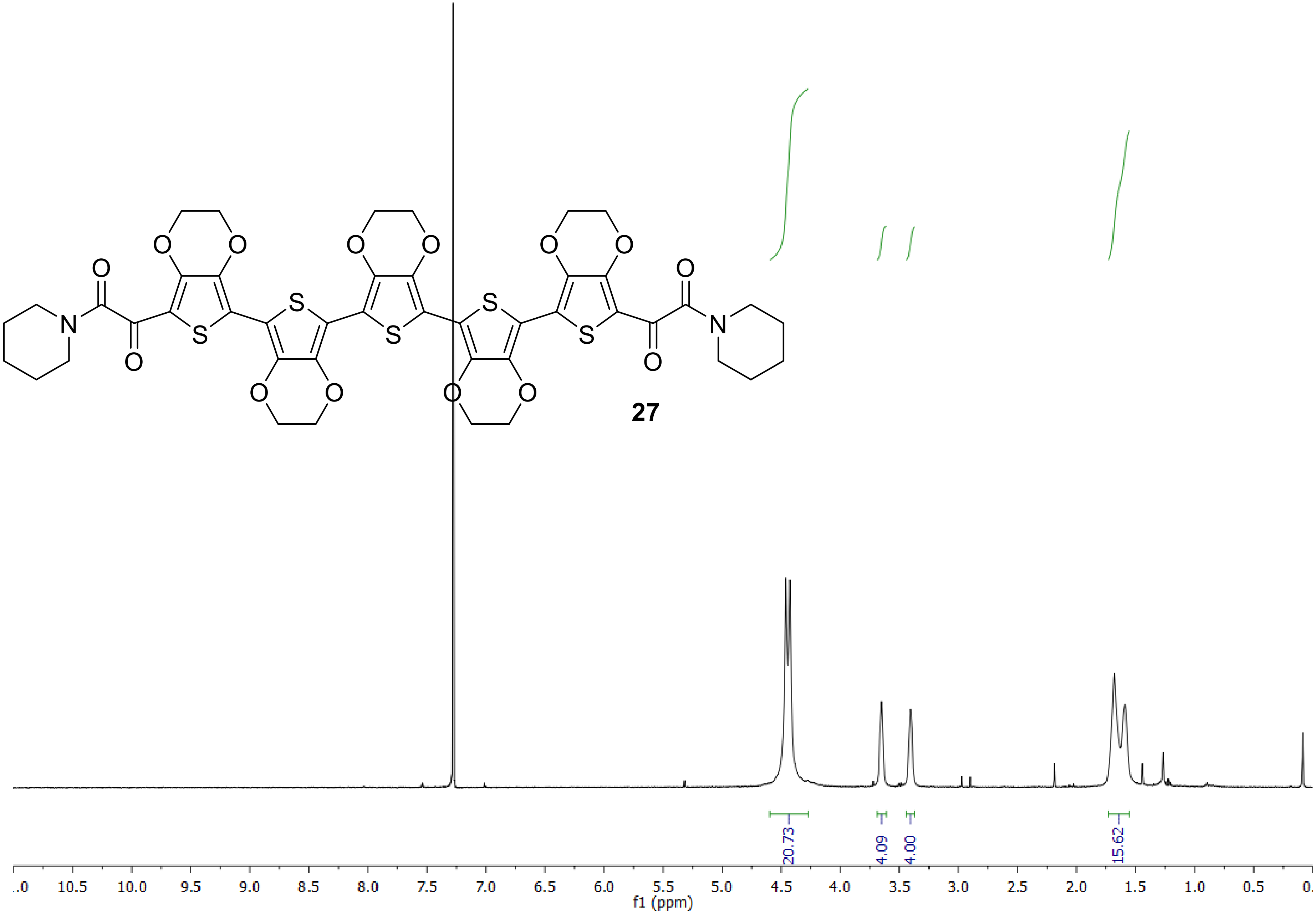

^1H NMR (400 MHz, $CDCl_3$)

Figure S51. ^1H NMR of **28**

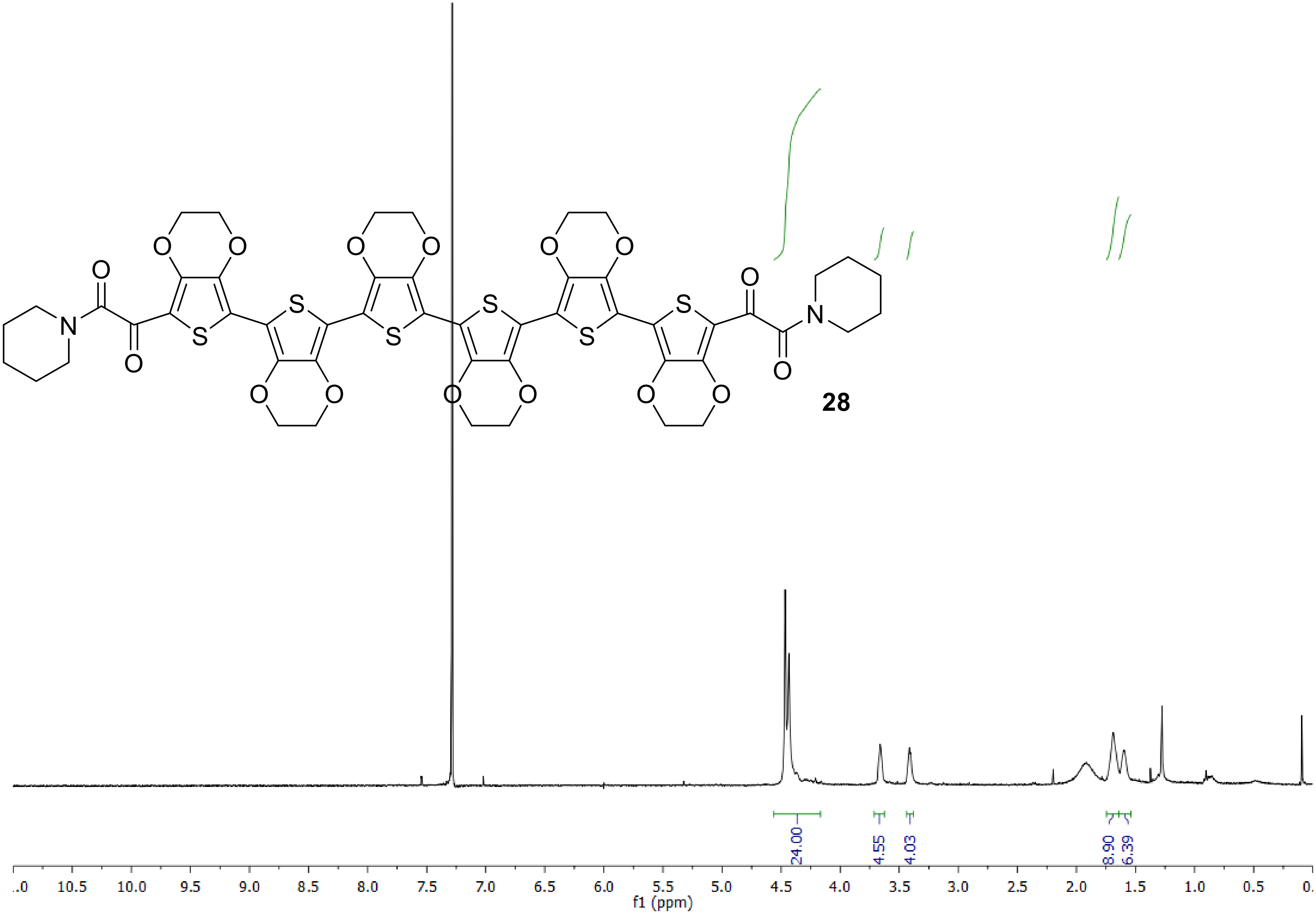

^{1}H NMR (400 MHz, $CDCl_3$)

Figure S52. ^{1}H NMR of **29**

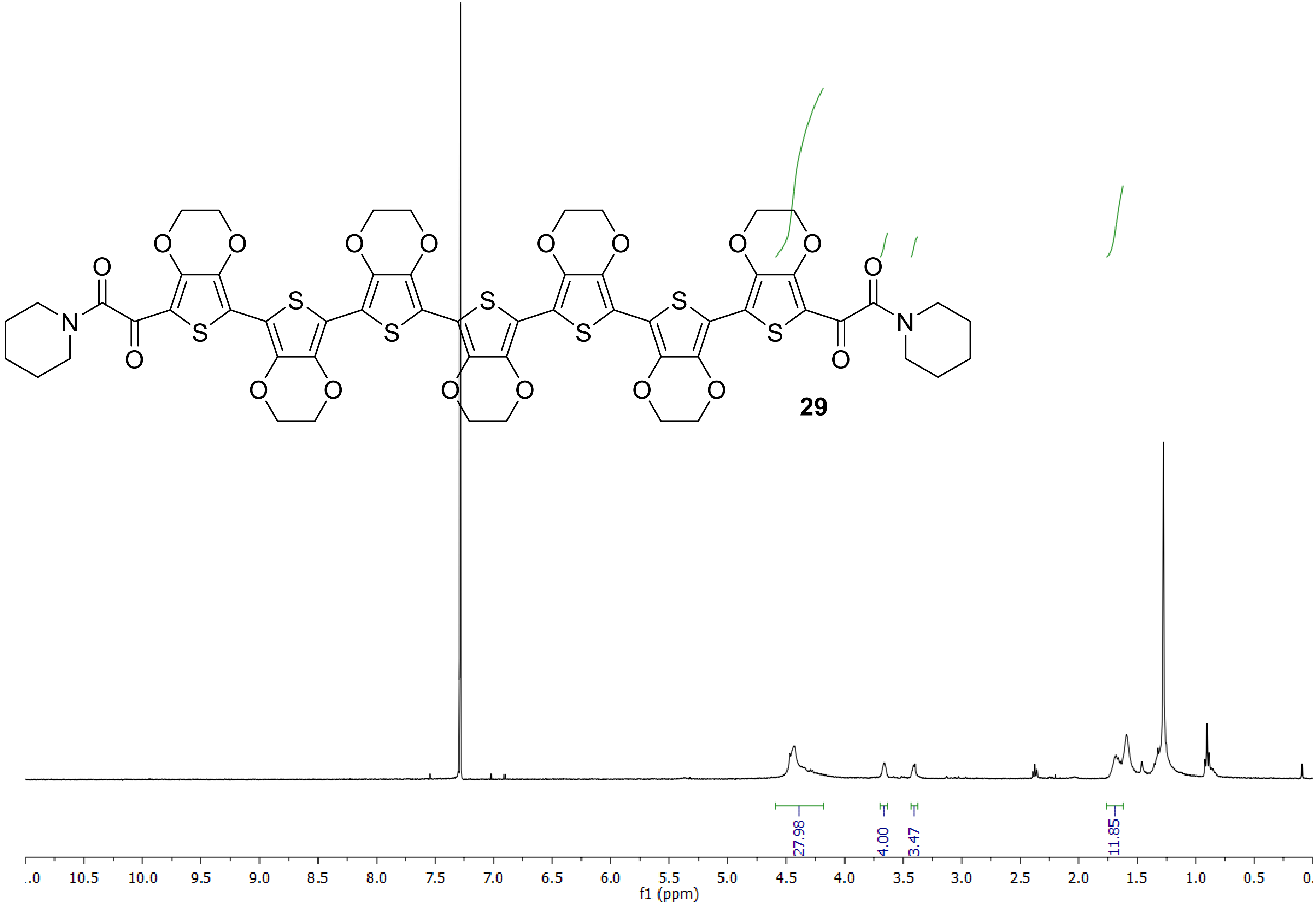

^1H NMR (400 MHz, $CDCl_3$)

Figure S53. ^1H NMR of **30**

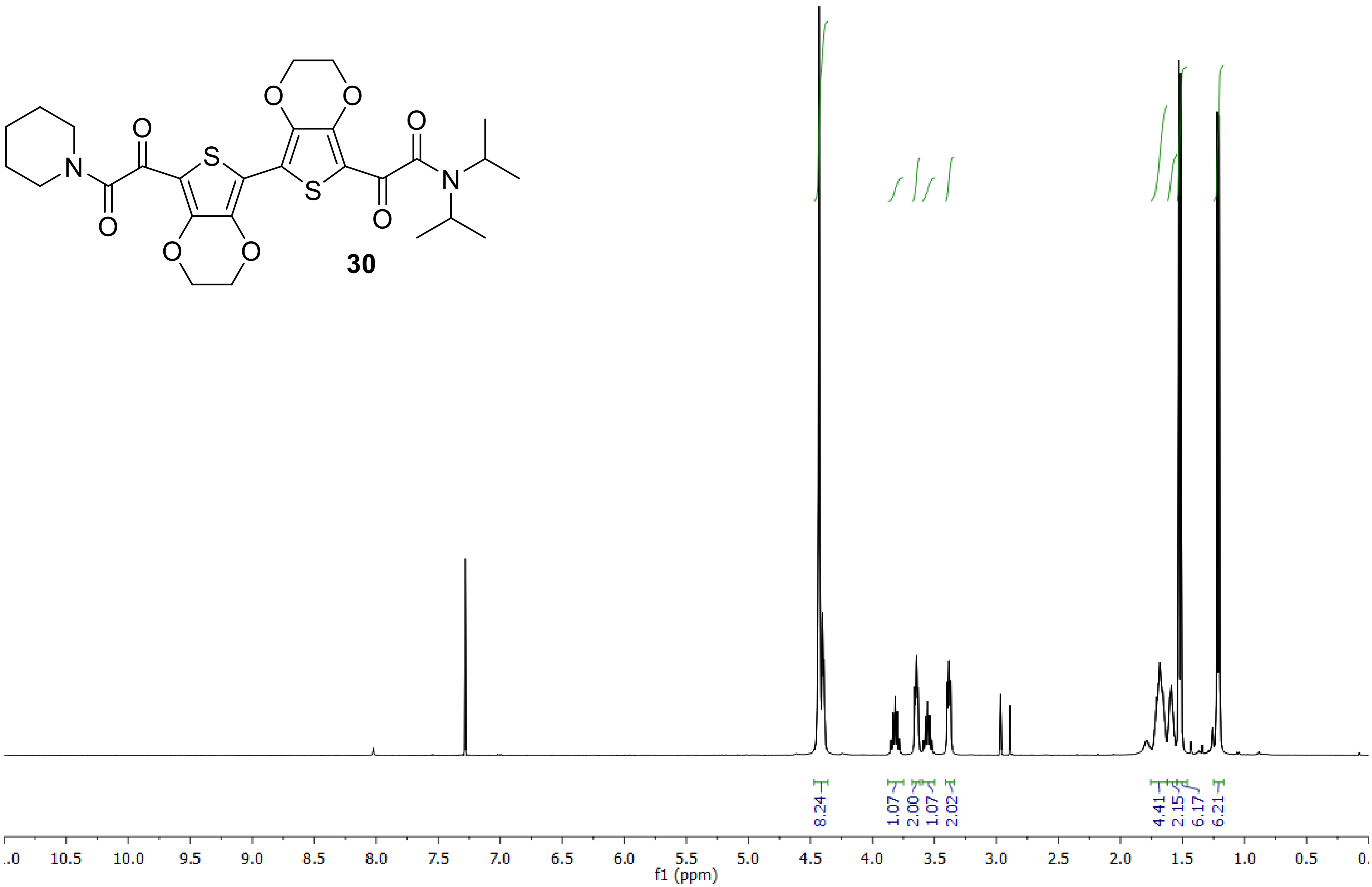

^{13}C NMR (100 MHz, $CDCl_3$)

Figure S54. ^{13}C NMR of **30**

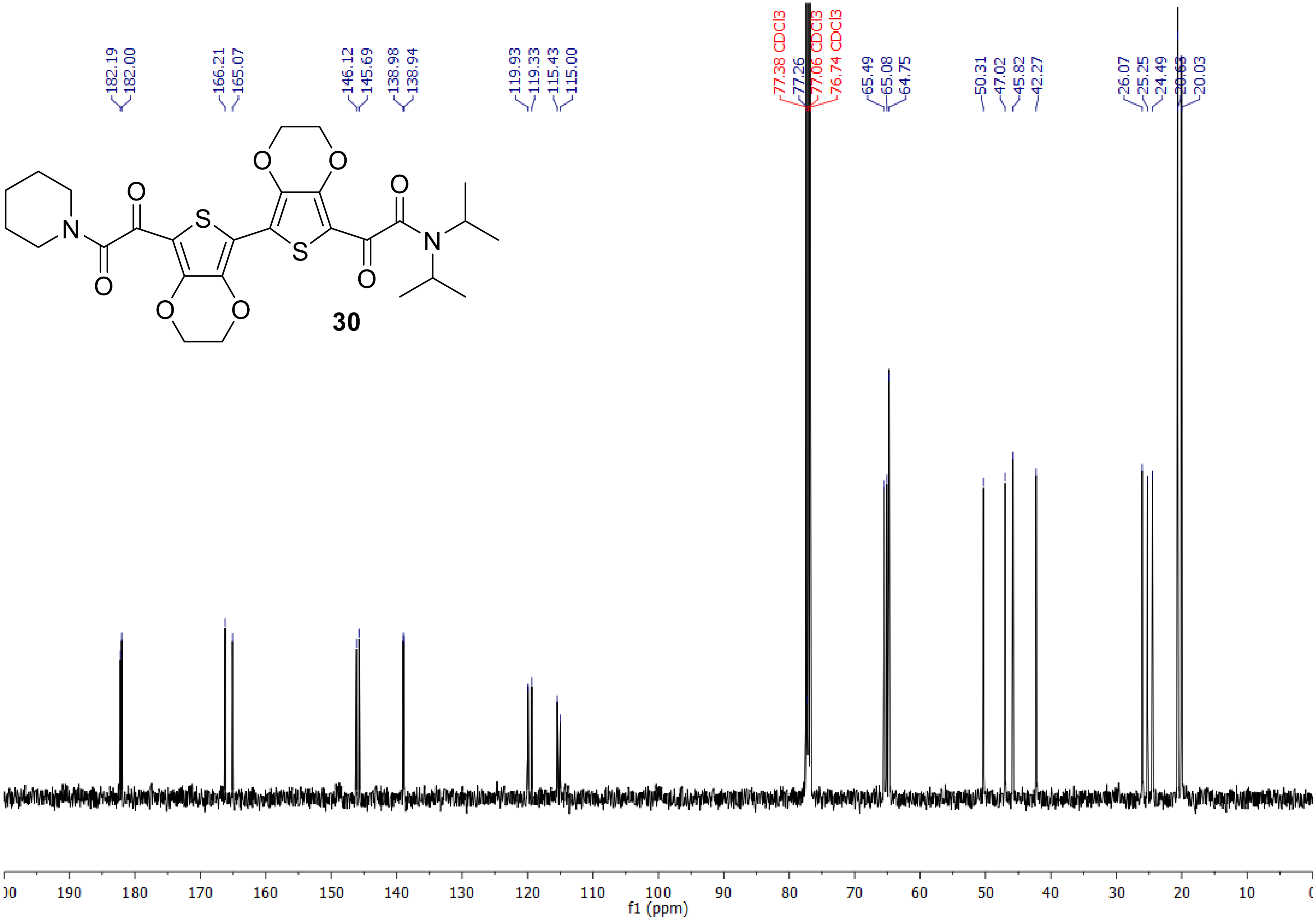

^1H NMR (400 MHz, $CDCl_3$)

Figure S55. ^1H NMR of **31**

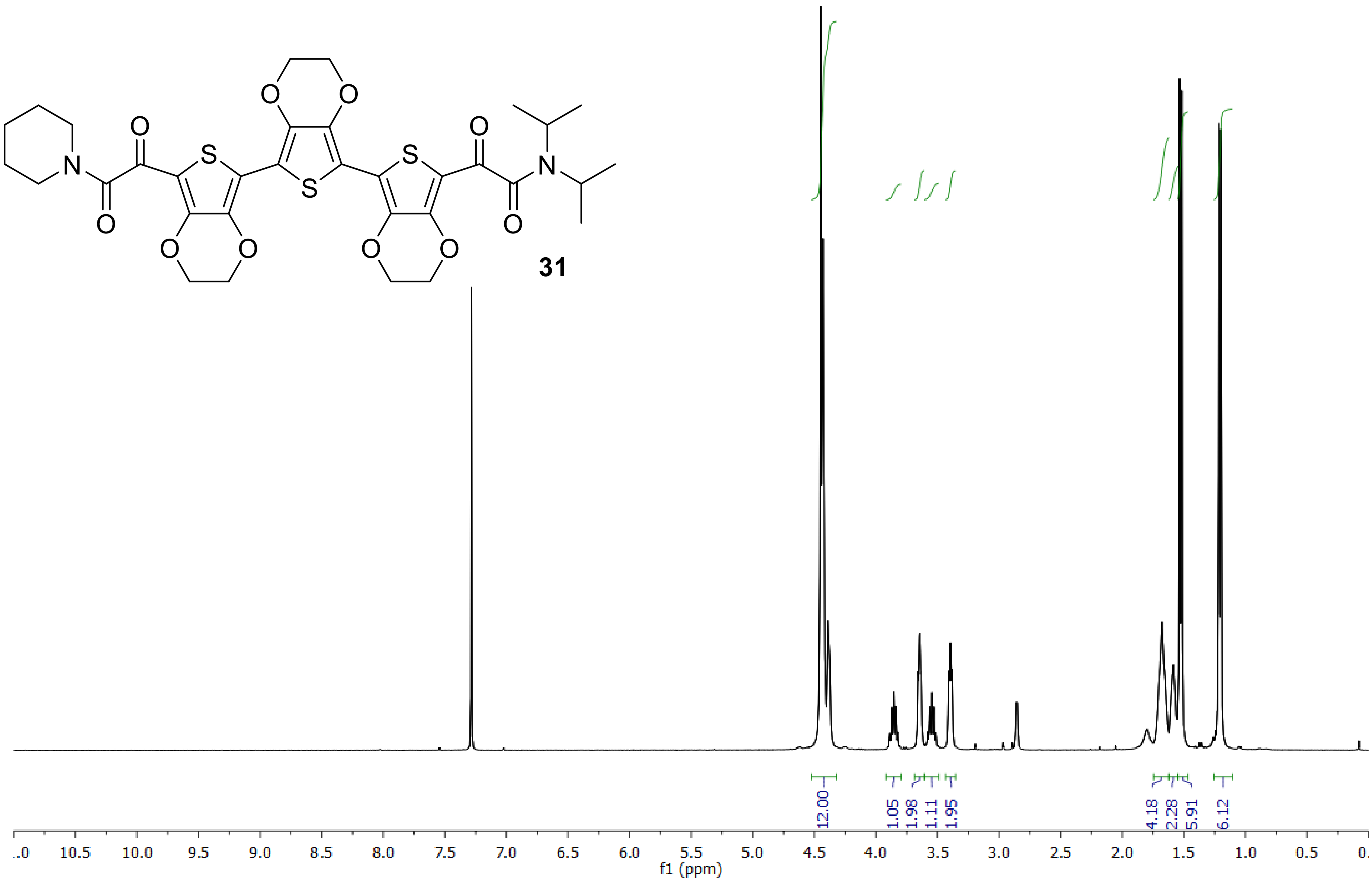

31

^{13}C NMR (100 MHz, $CDCl_3$)

Figure S56. ^{13}C NMR of **31**

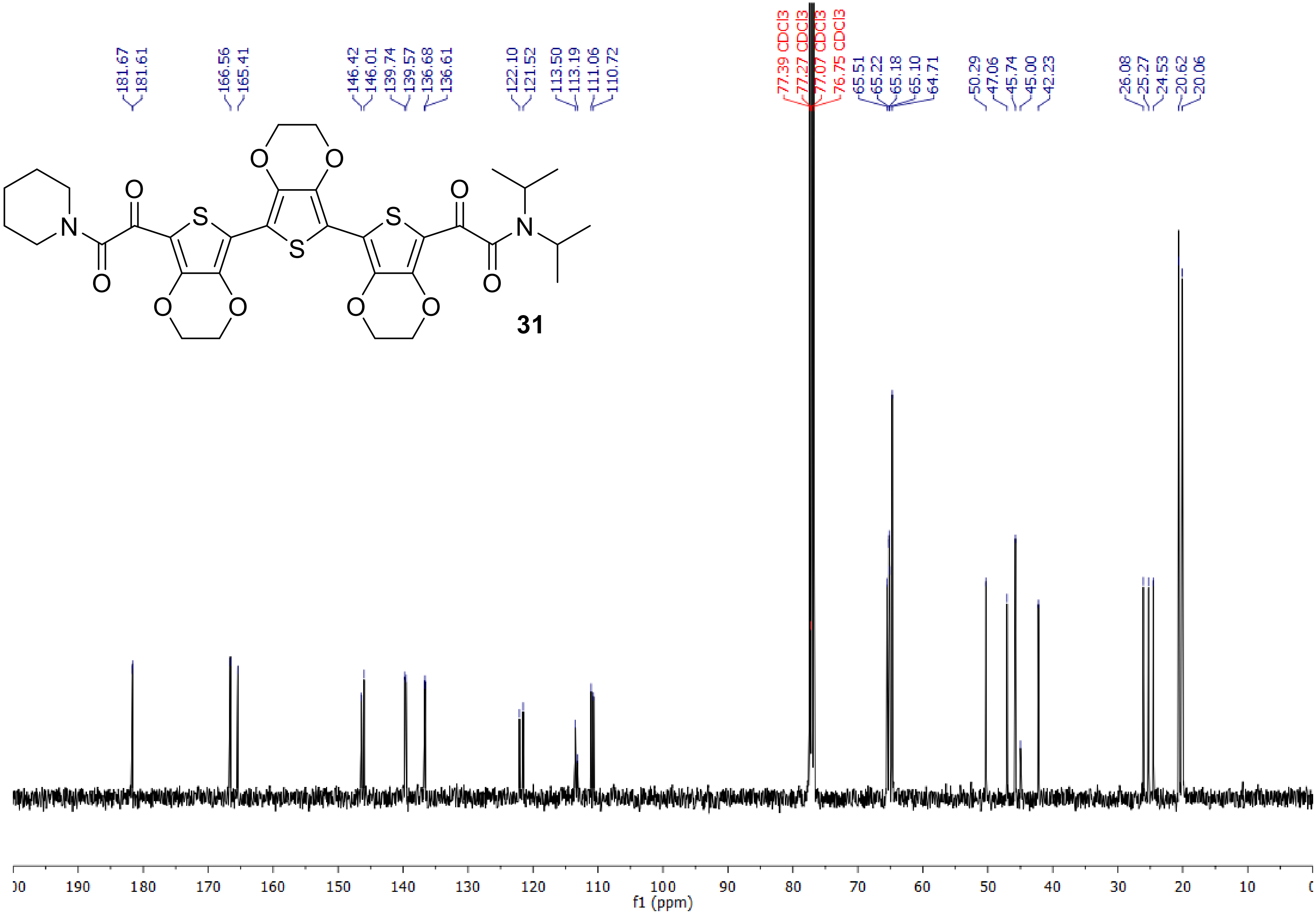

31

^{1}H NMR (400 MHz, $CDCl_3$)

Figure S57. ^{1}H NMR of **32**

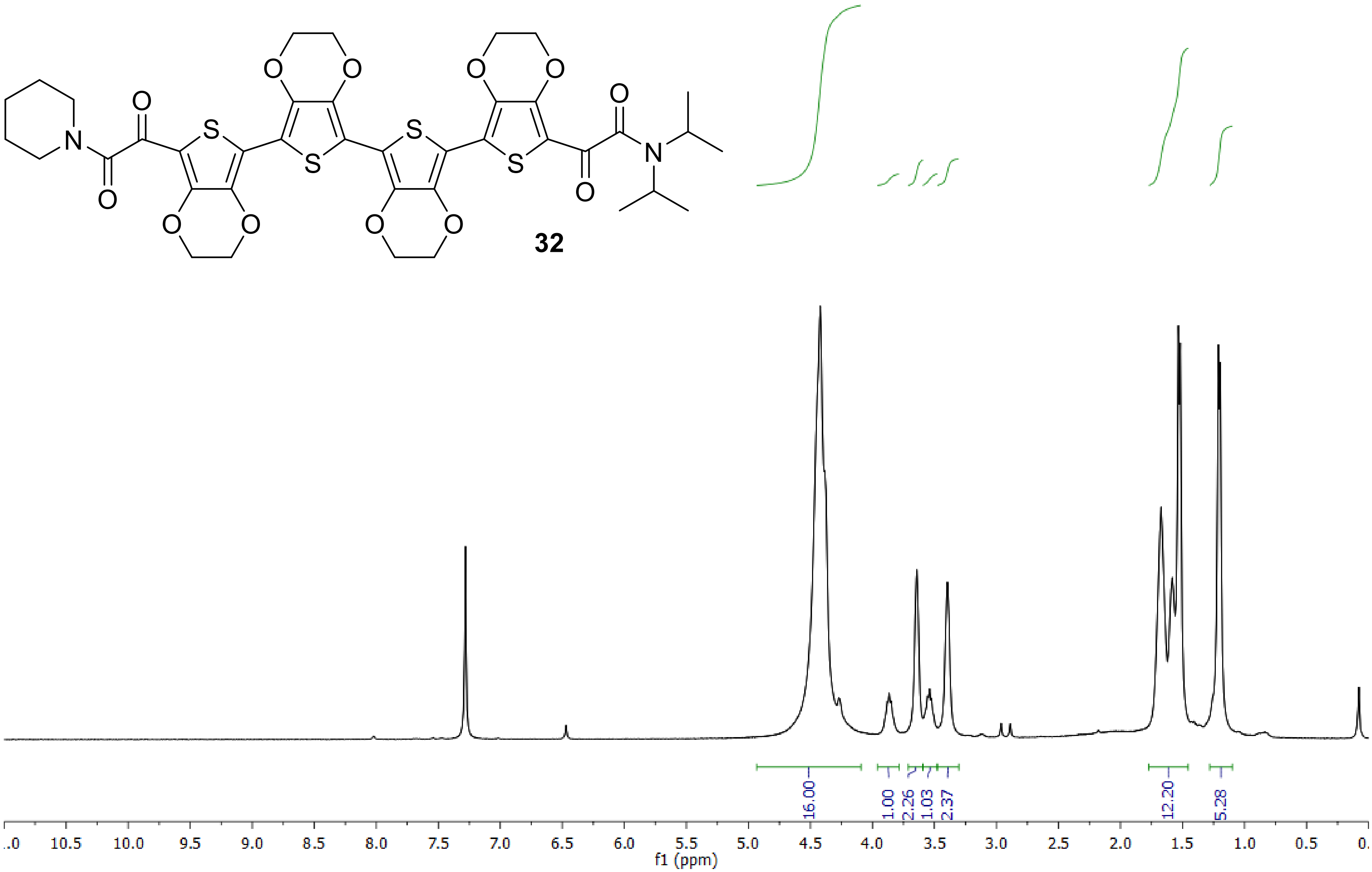

^{13}C NMR (125 MHz, $CDCl_3$)

Figure S58. ^{13}C NMR of **32**

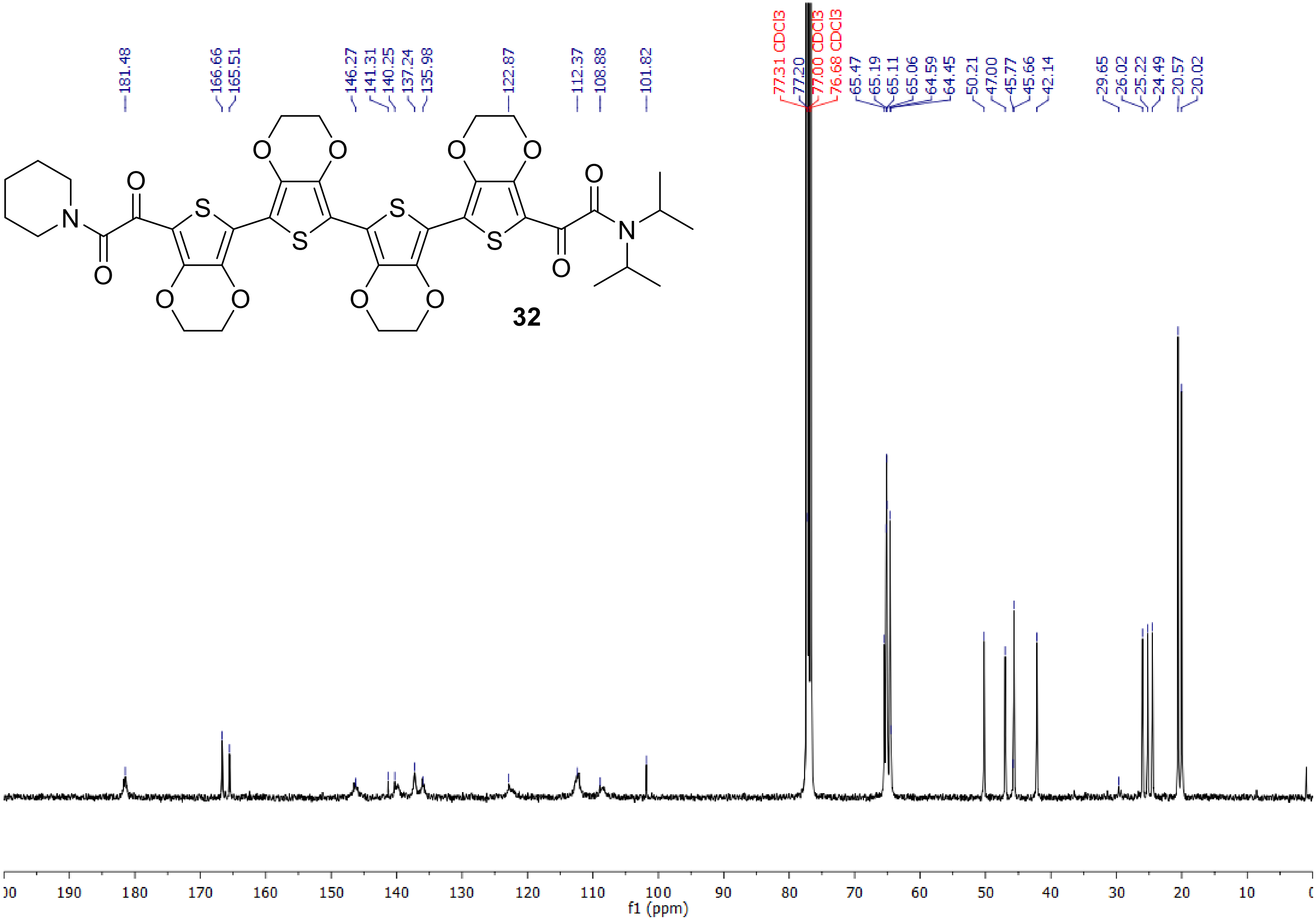

^1H NMR (400 MHz, $CDCl_3$)

Figure S59. ^1H NMR of **33**

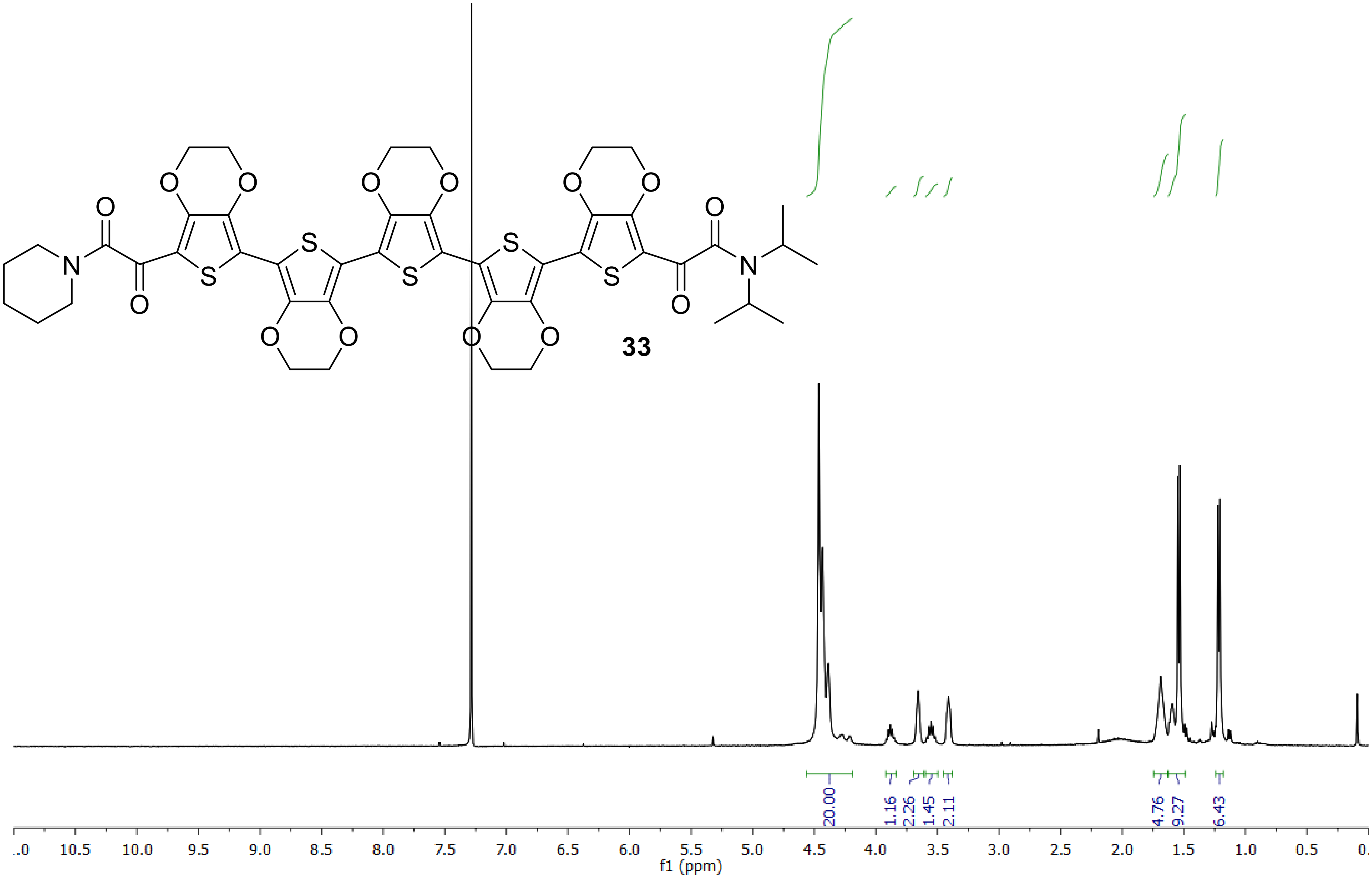

^{1}H NMR (400 MHz, DMSO)

Figure S60. ^{1}H NMR of **34**

34

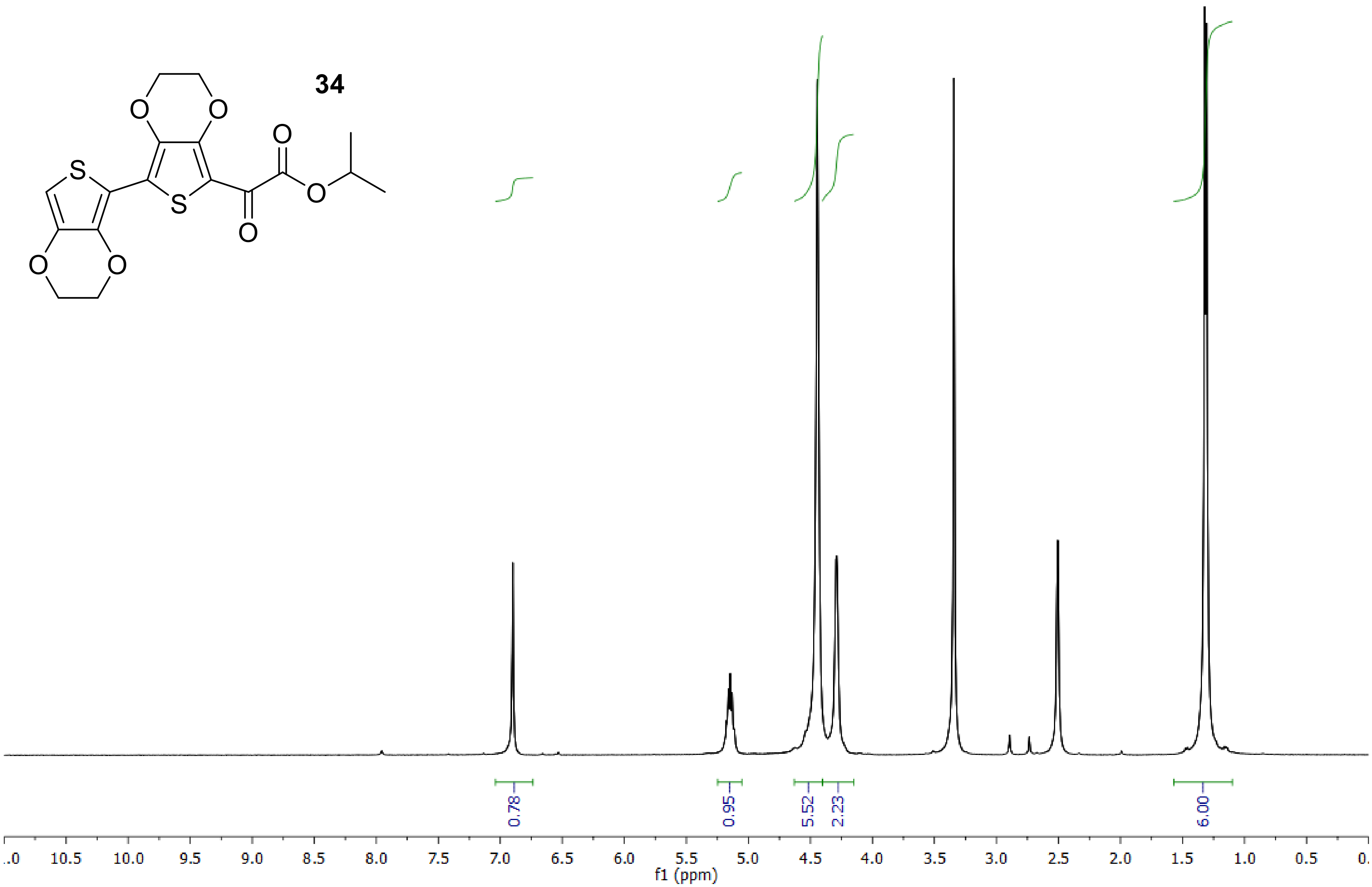

^{13}C NMR (100 MHz, DMSO)

Figure S61. ^{13}C NMR of **34**

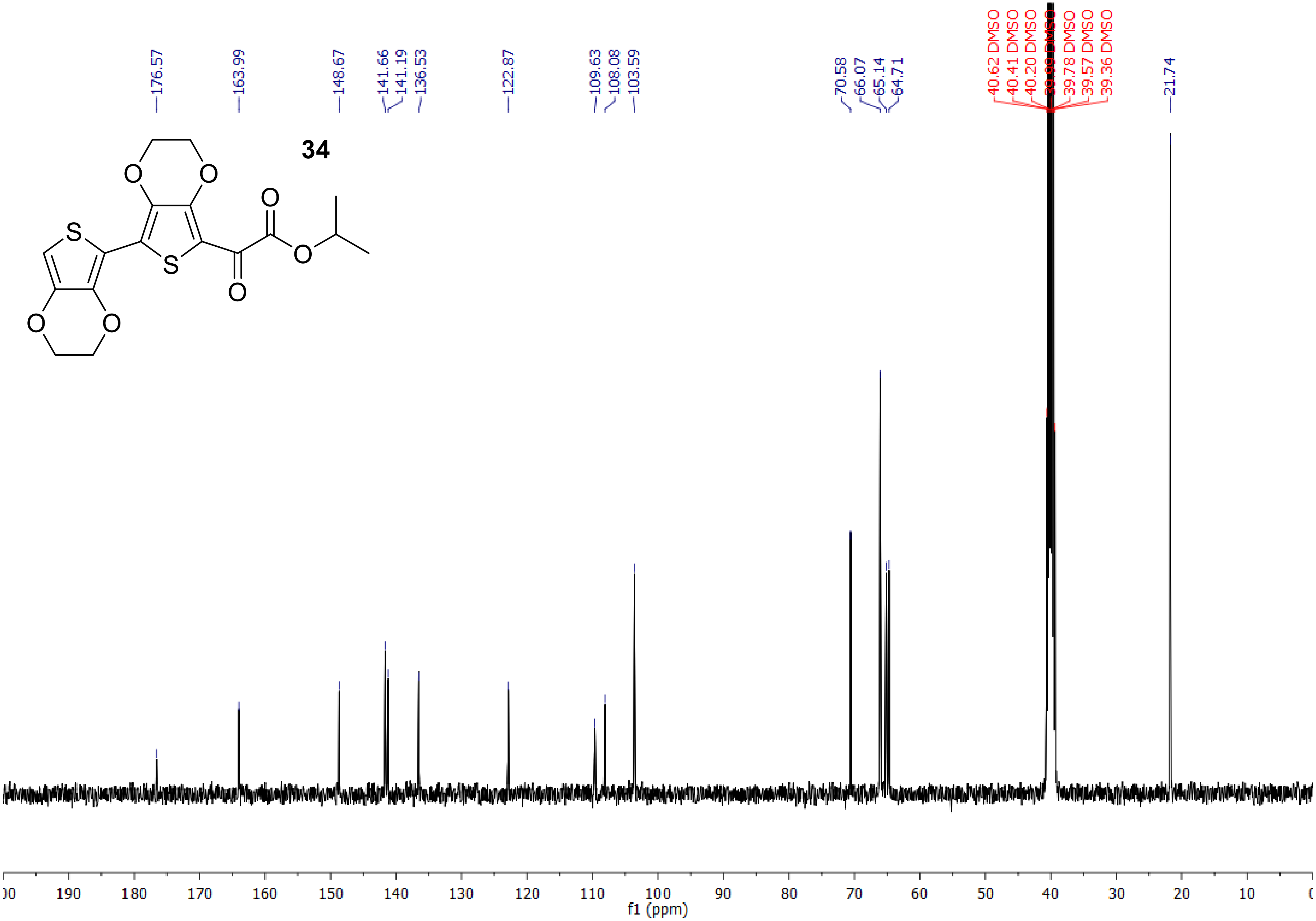

^{1}H NMR (400 MHz, $CDCl_3$)

Figure S62. ^{1}H NMR of **35**

35

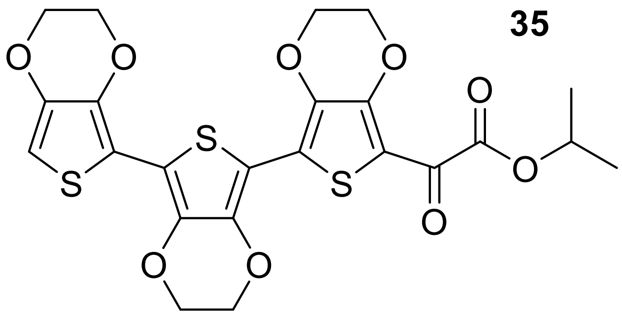

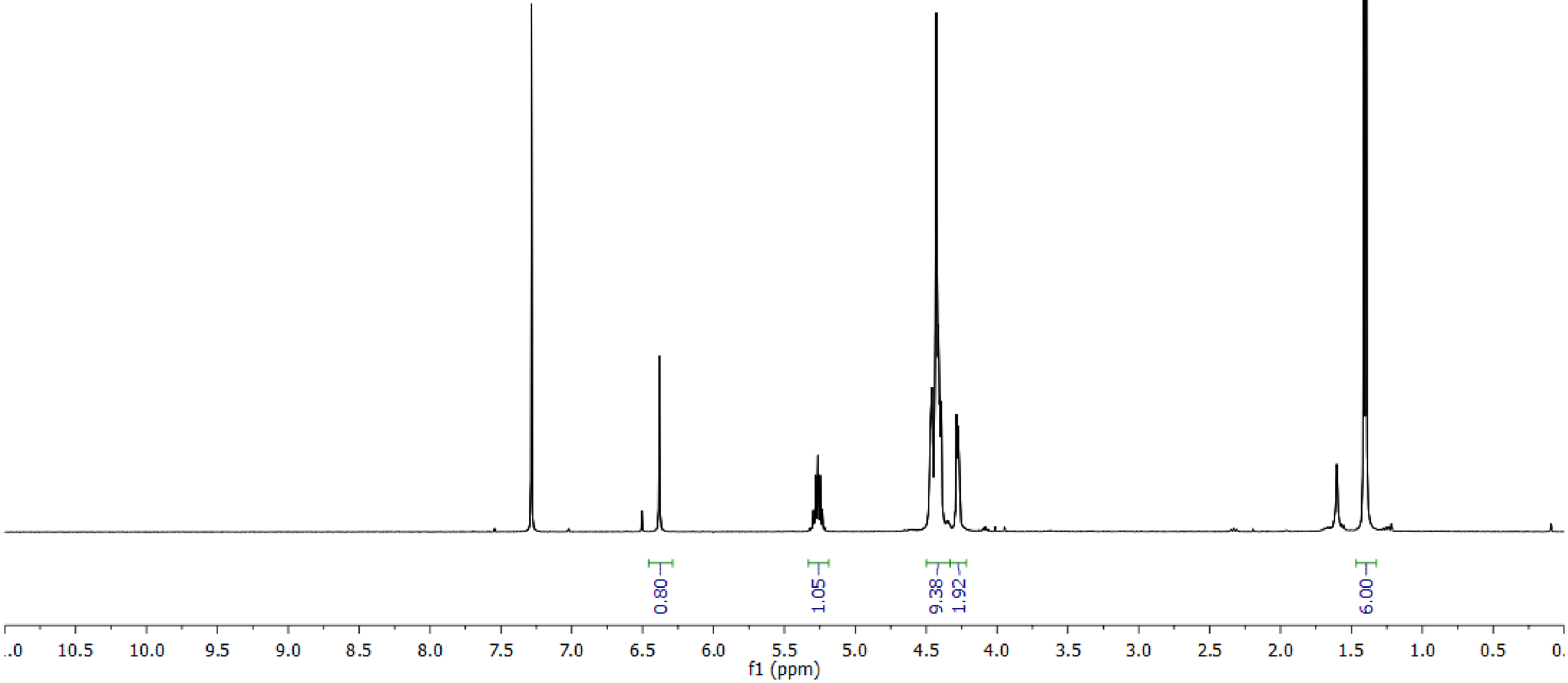

^{13}C NMR (100 MHz, $CDCl_3$)

Figure S63. ^{13}C NMR of **35**

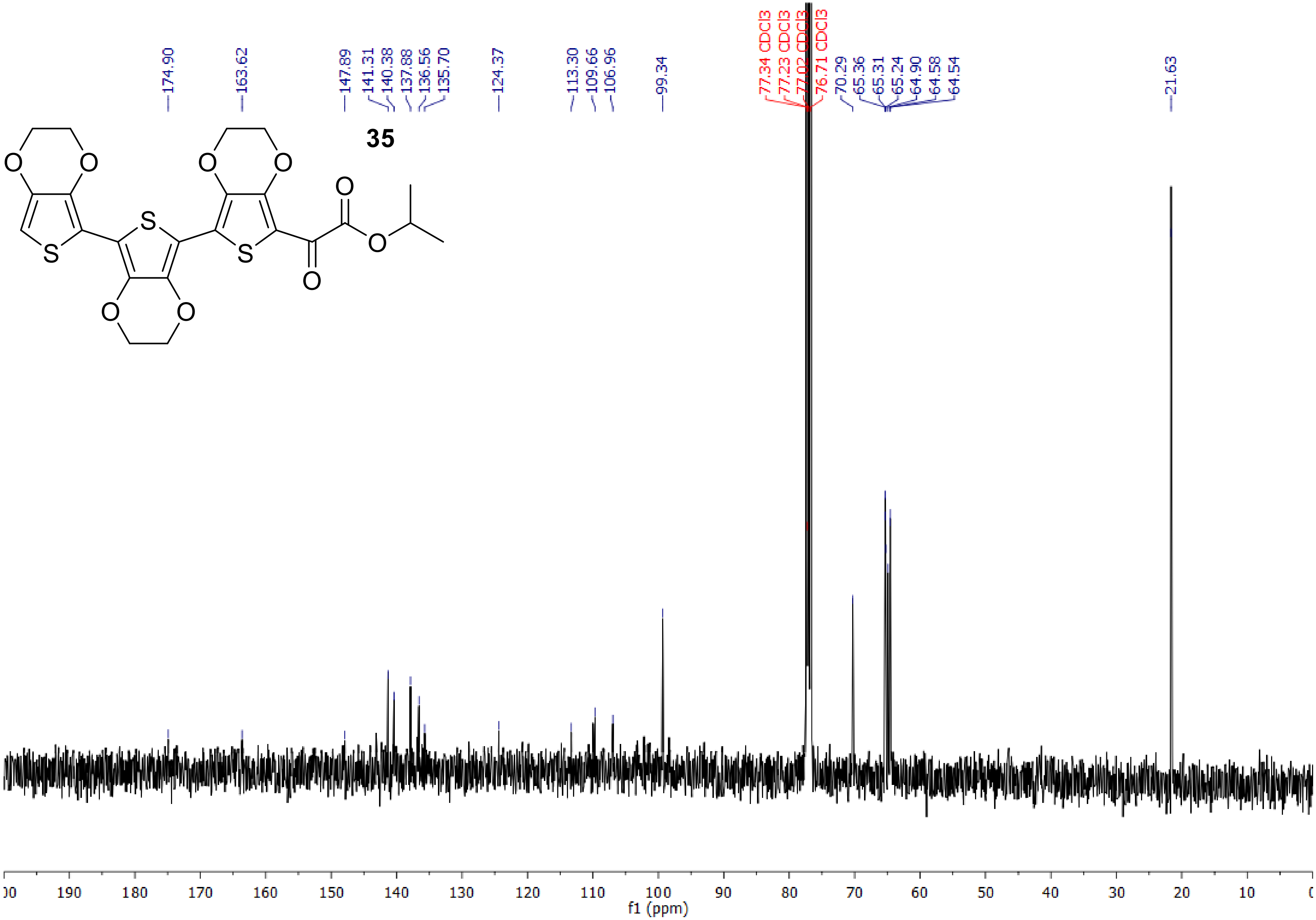

^{1}H NMR (400 MHz, $CDCl_3$)

Figure S64. ^{1}H NMR of **36**

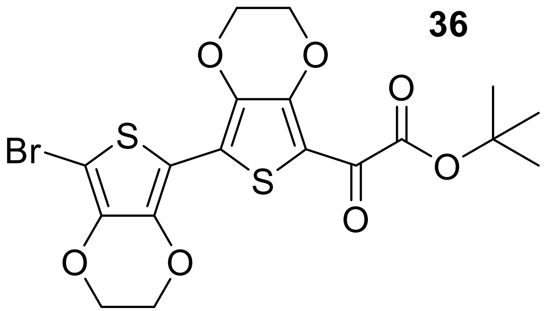

36

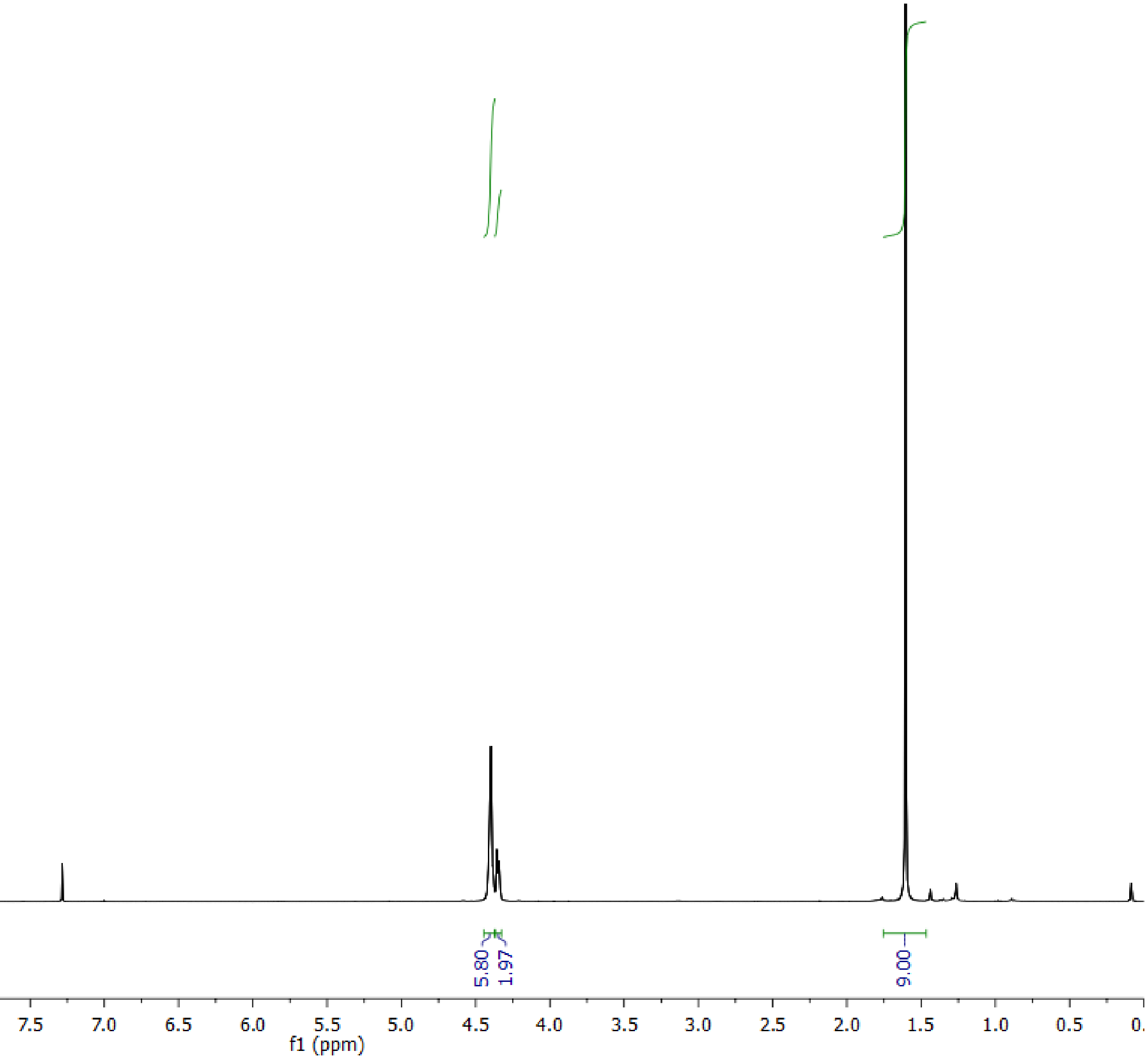

Figure S65. ^{13}C NMR of **36**

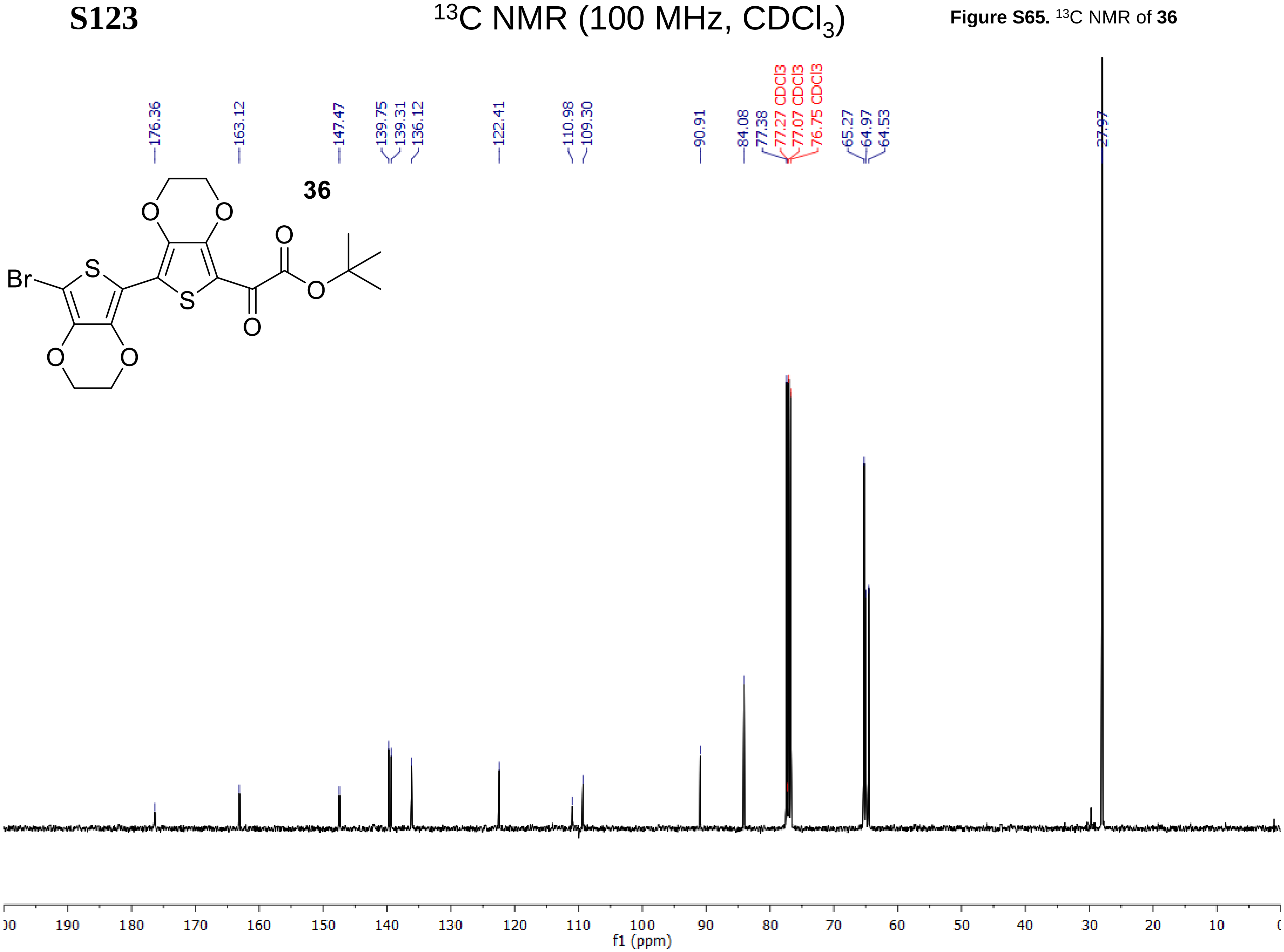

^{1}H NMR (400 MHz, $CDCl_3$)

Figure S66. ^{1}H NMR of **37**

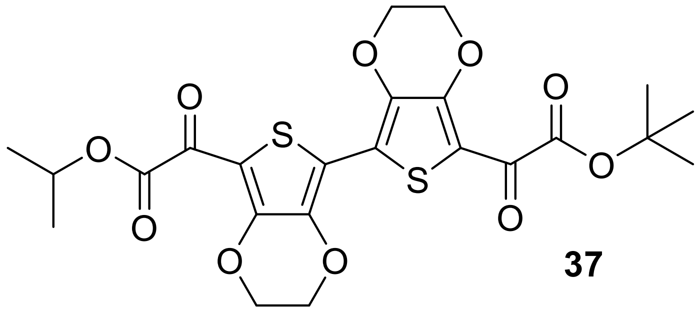

37

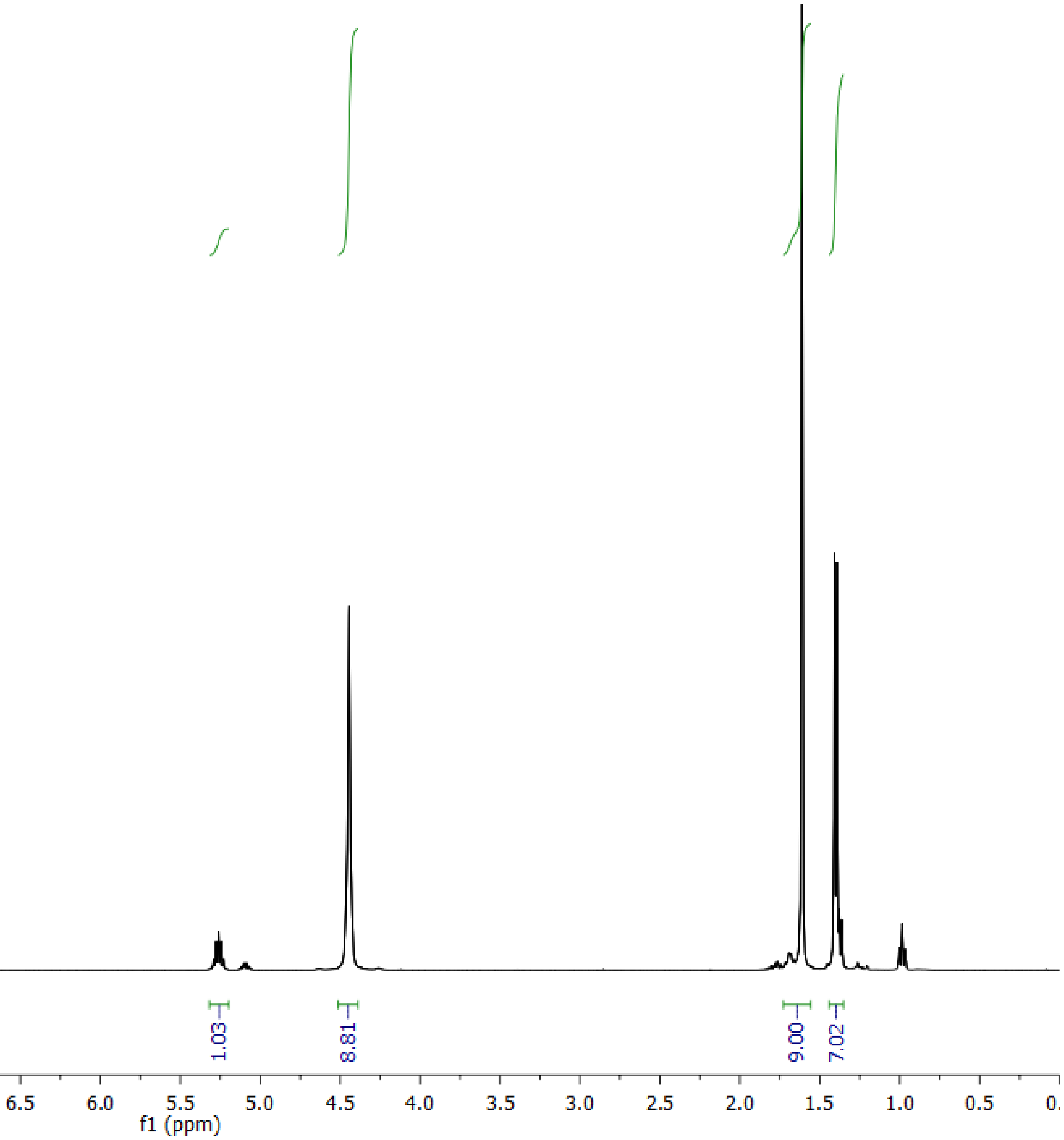

^{13}C NMR (100 MHz, $CDCl_3$)

Figure S67. ^{13}C NMR of **37**

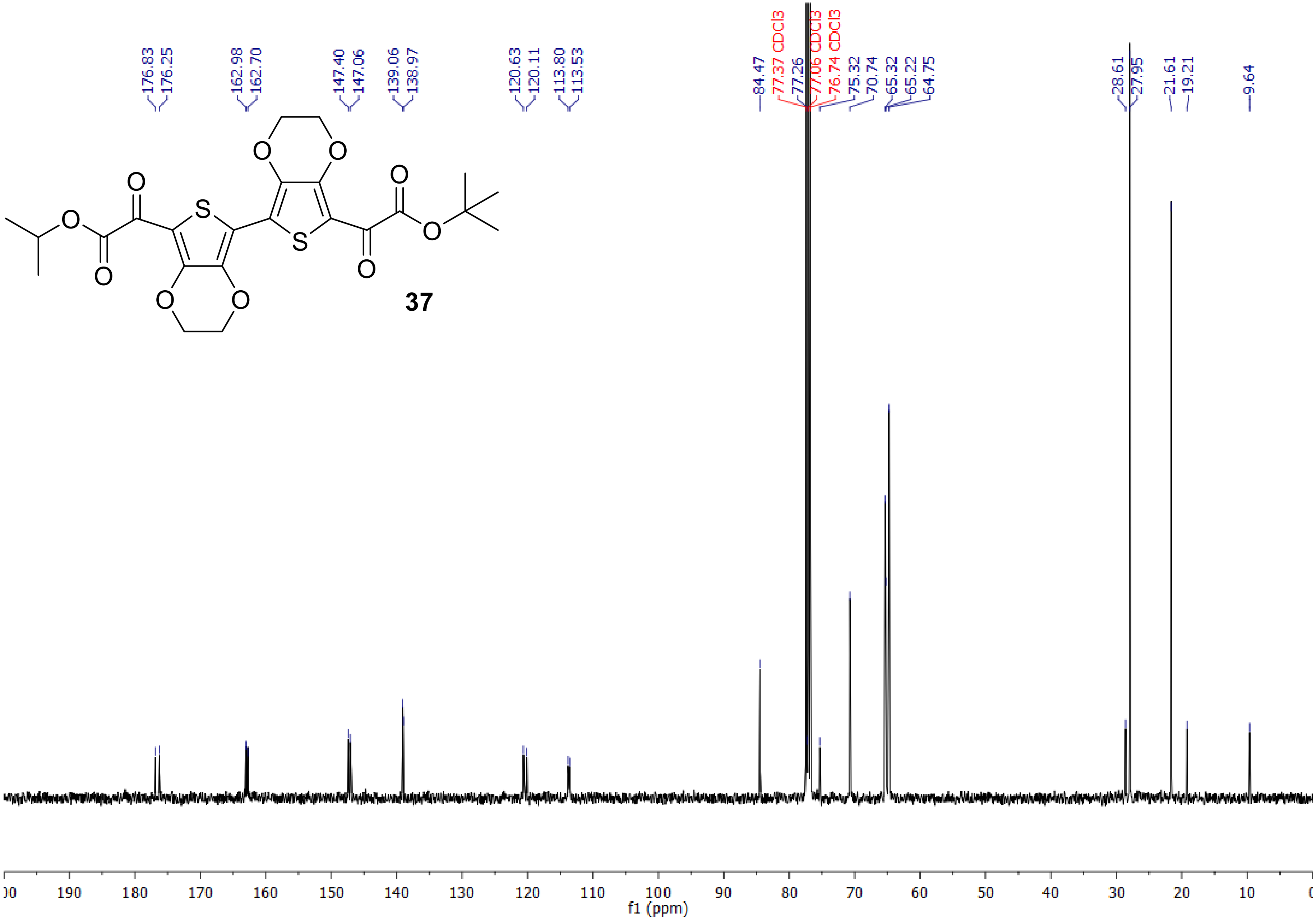

^{1}H NMR (400 MHz, $CDCl_3$)

Figure S68. ^{1}H NMR of **39**

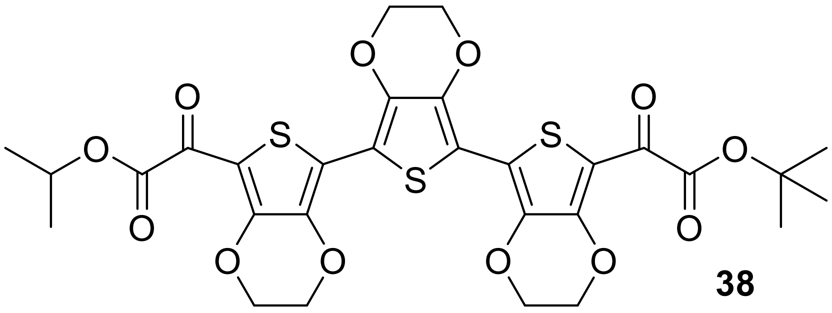

38

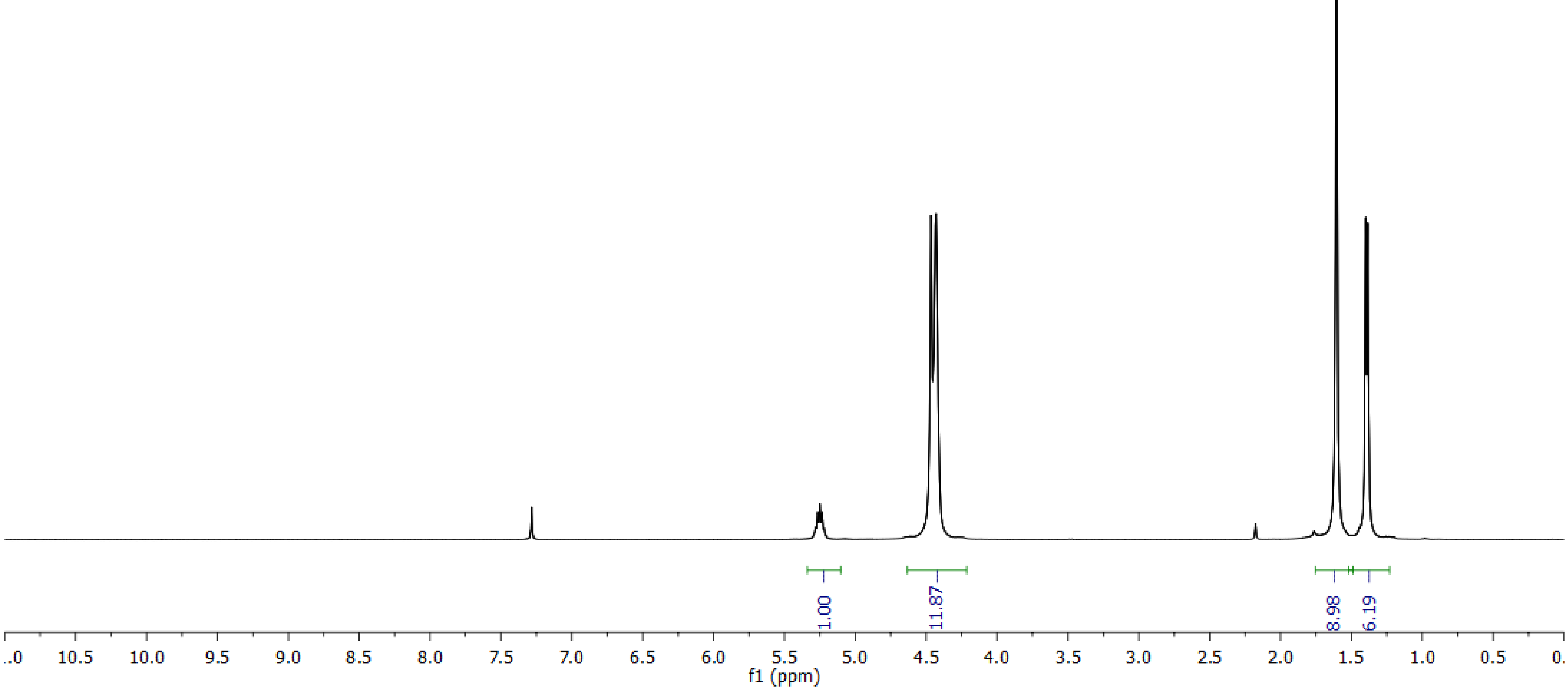

^{13}C NMR (100 MHz, $CDCl_3$)

Figure S69. ^{13}C NMR of **38**

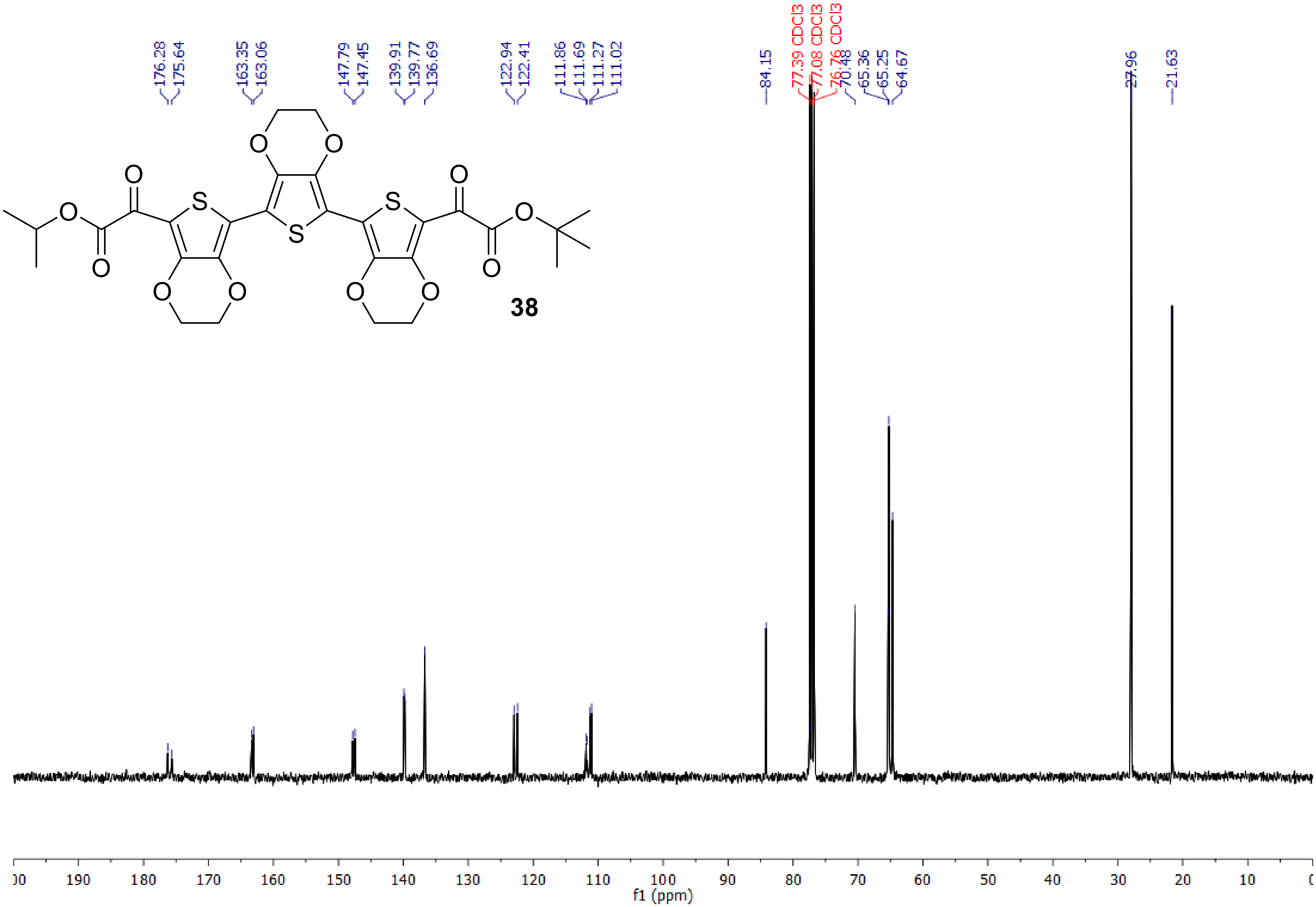

^{1}H NMR (400 MHz, $CDCl_3$)

Figure S70. ^{1}H NMR of **39**

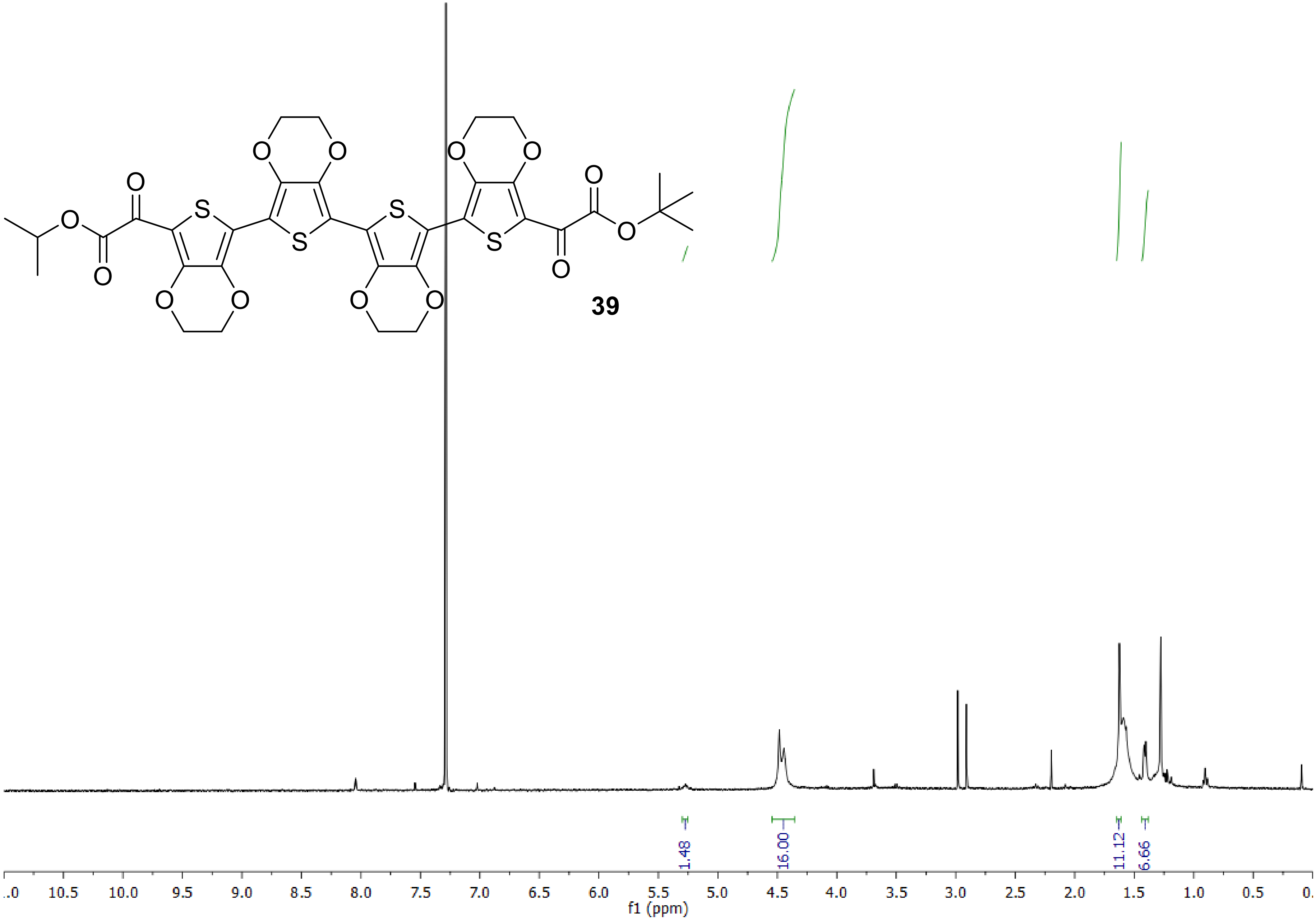

^{1}H NMR (400 MHz, $CDCl_3$)

Figure S71. ^{1}H NMR of **40**

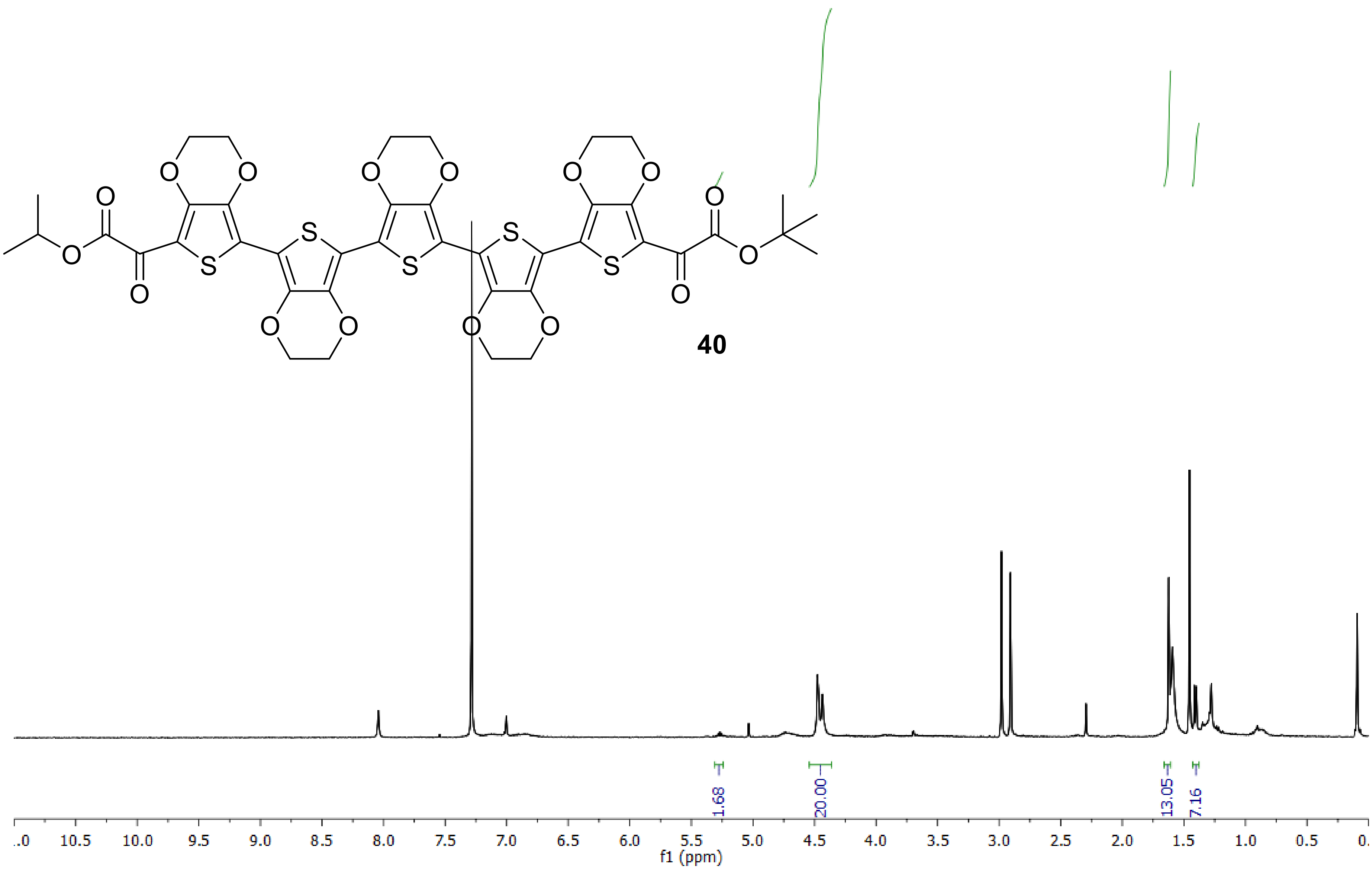

^1H NMR (400 MHz, $CDCl_3$)

Figure S72. ^1H NMR of **41**

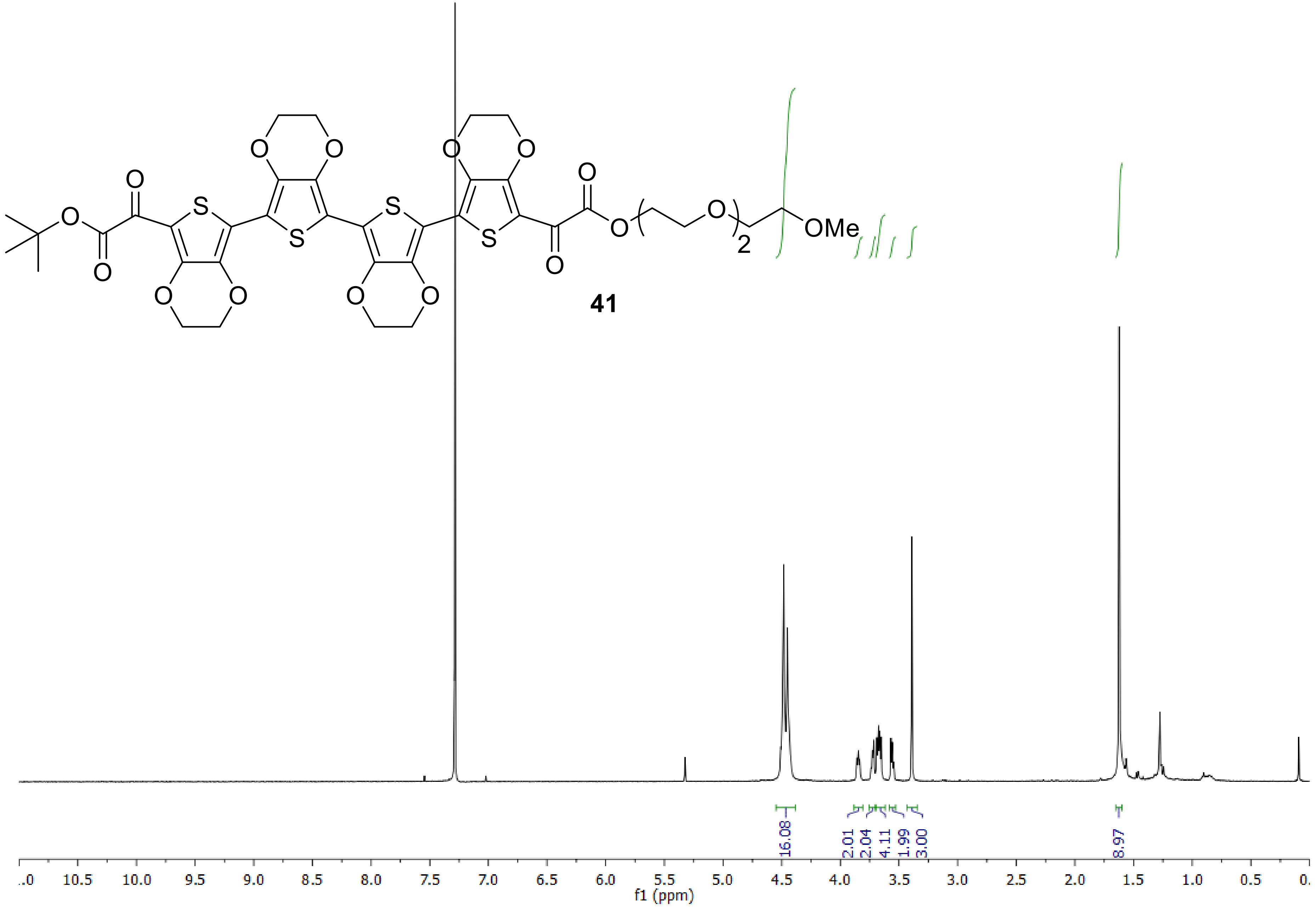

^{1}H NMR (400 MHz, $CDCl_3$)

Figure S73. ^{1}H NMR of **42**

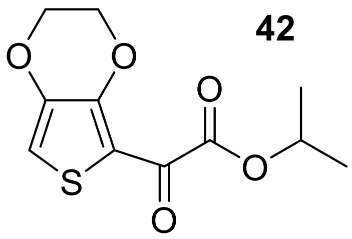

42

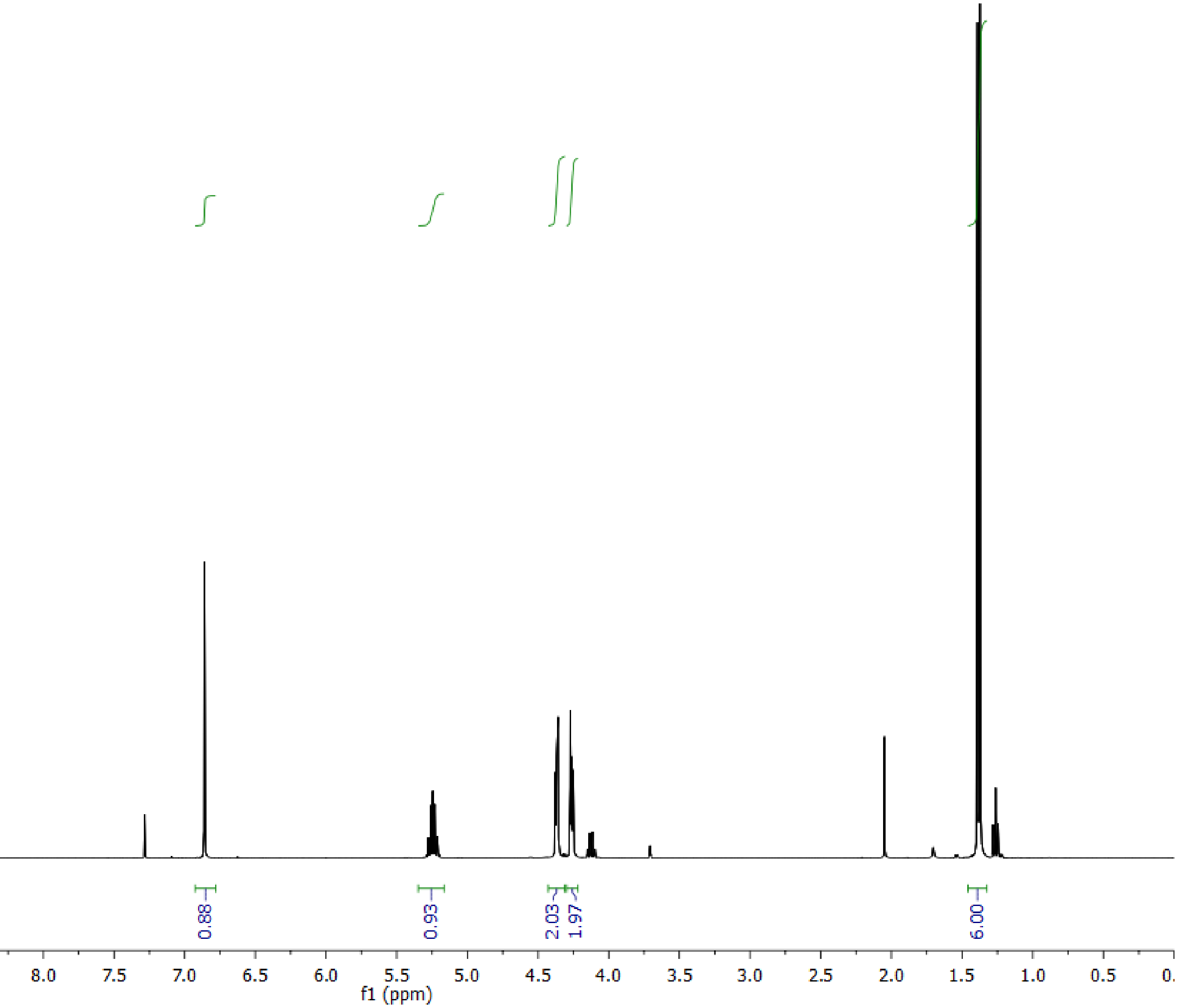

^{13}C NMR (100 MHz, $CDCl_3$)

Figure S74. ^{13}C NMR of **42**

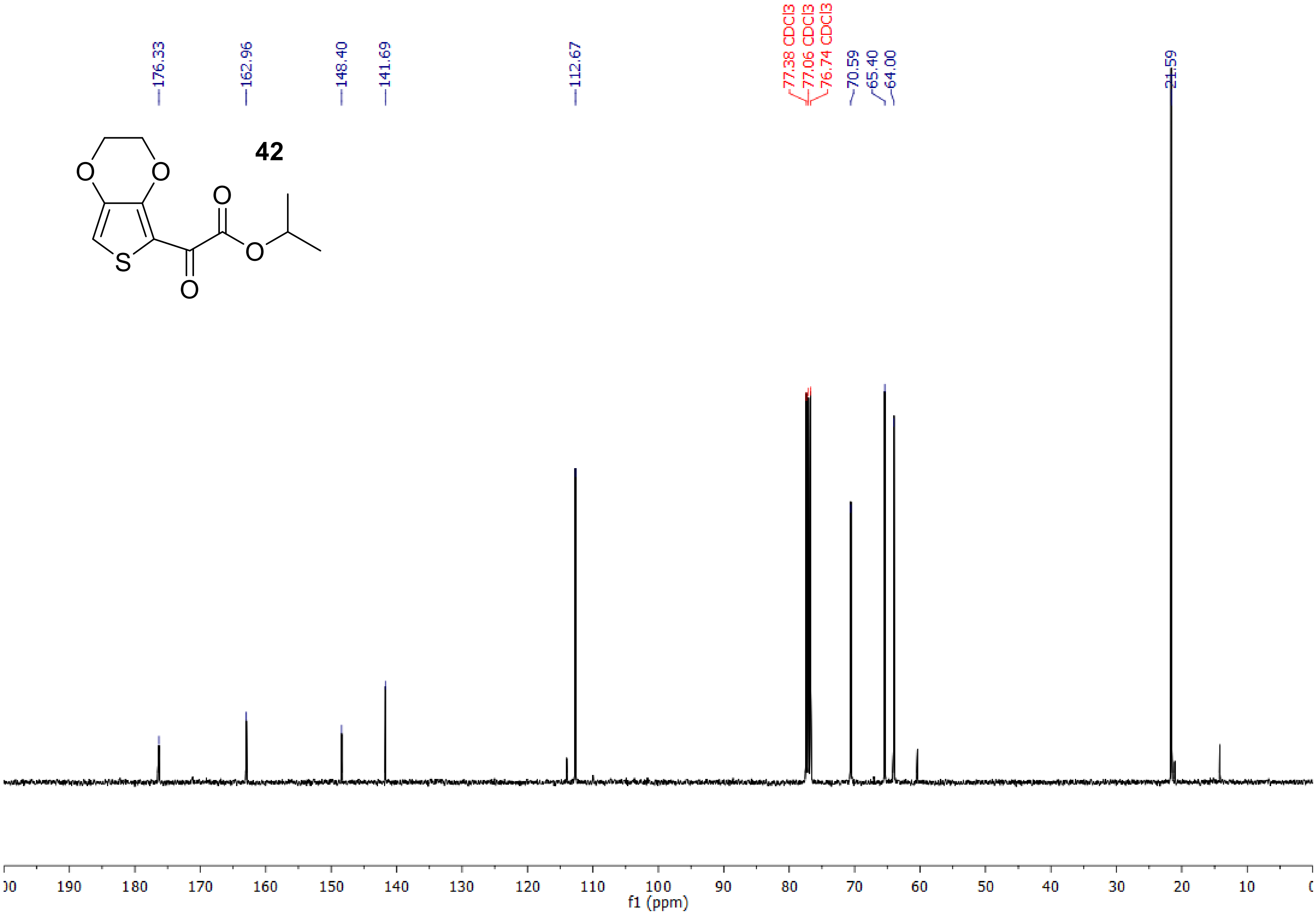

^{1}H NMR (400 MHz, $CDCl_3$)

Figure S75. ^{1}H NMR of **45**

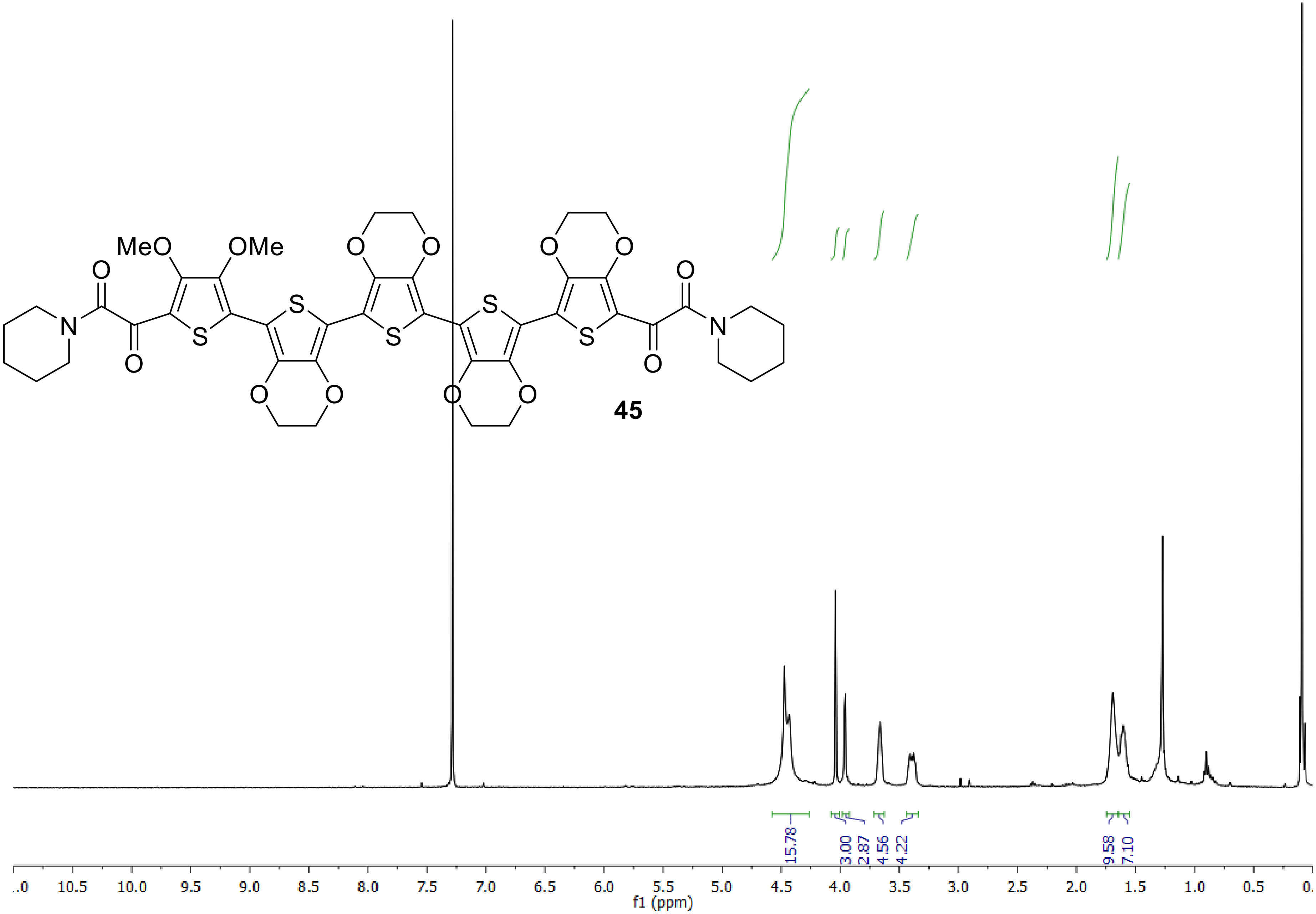

^{1}H NMR (400 MHz, $CDCl_3$)

Figure S76. ^{1}H NMR of **46**

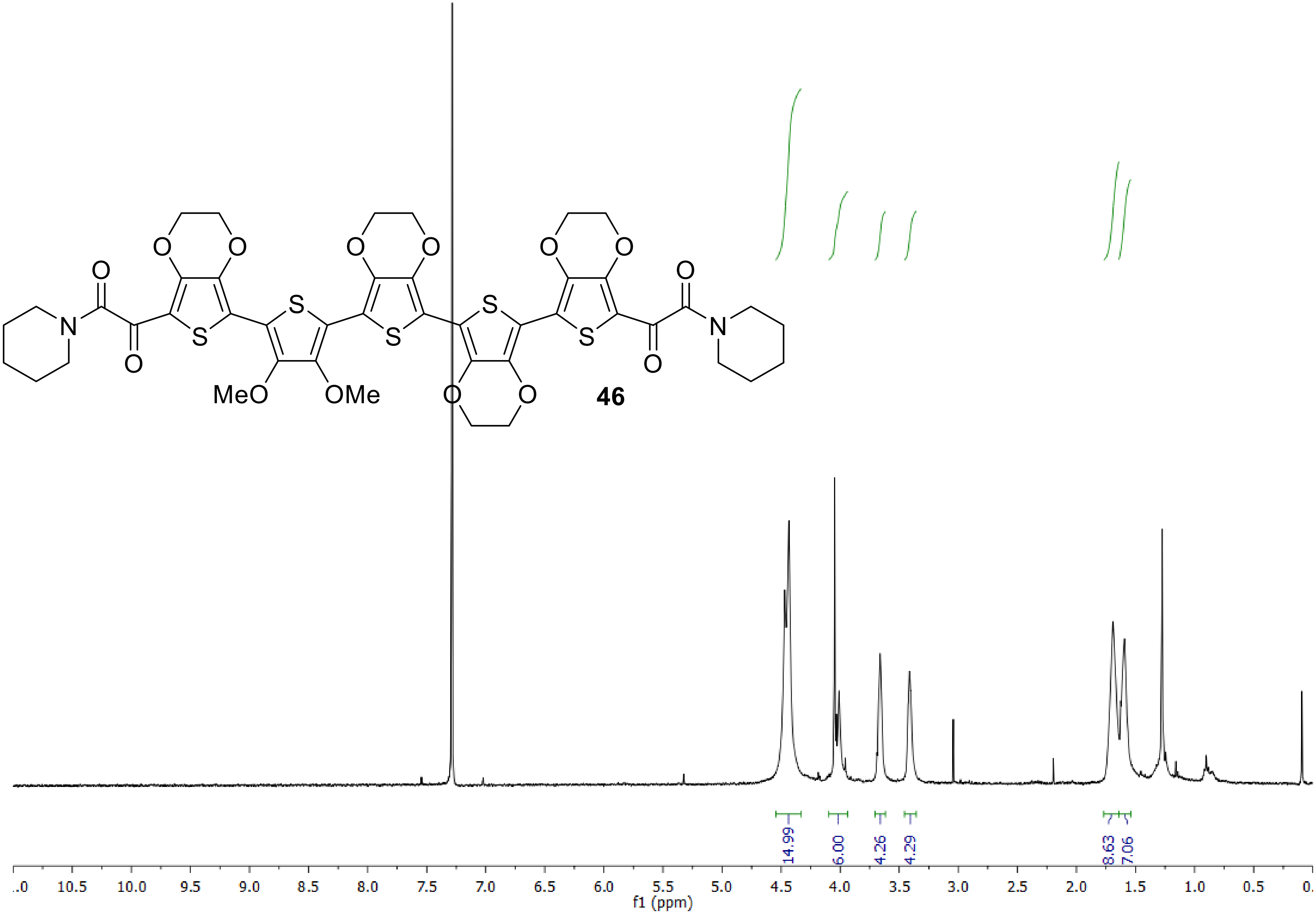

^{1}H NMR (400 MHz, $CDCl_3$)

Figure S77. ^{1}H NMR of **47**

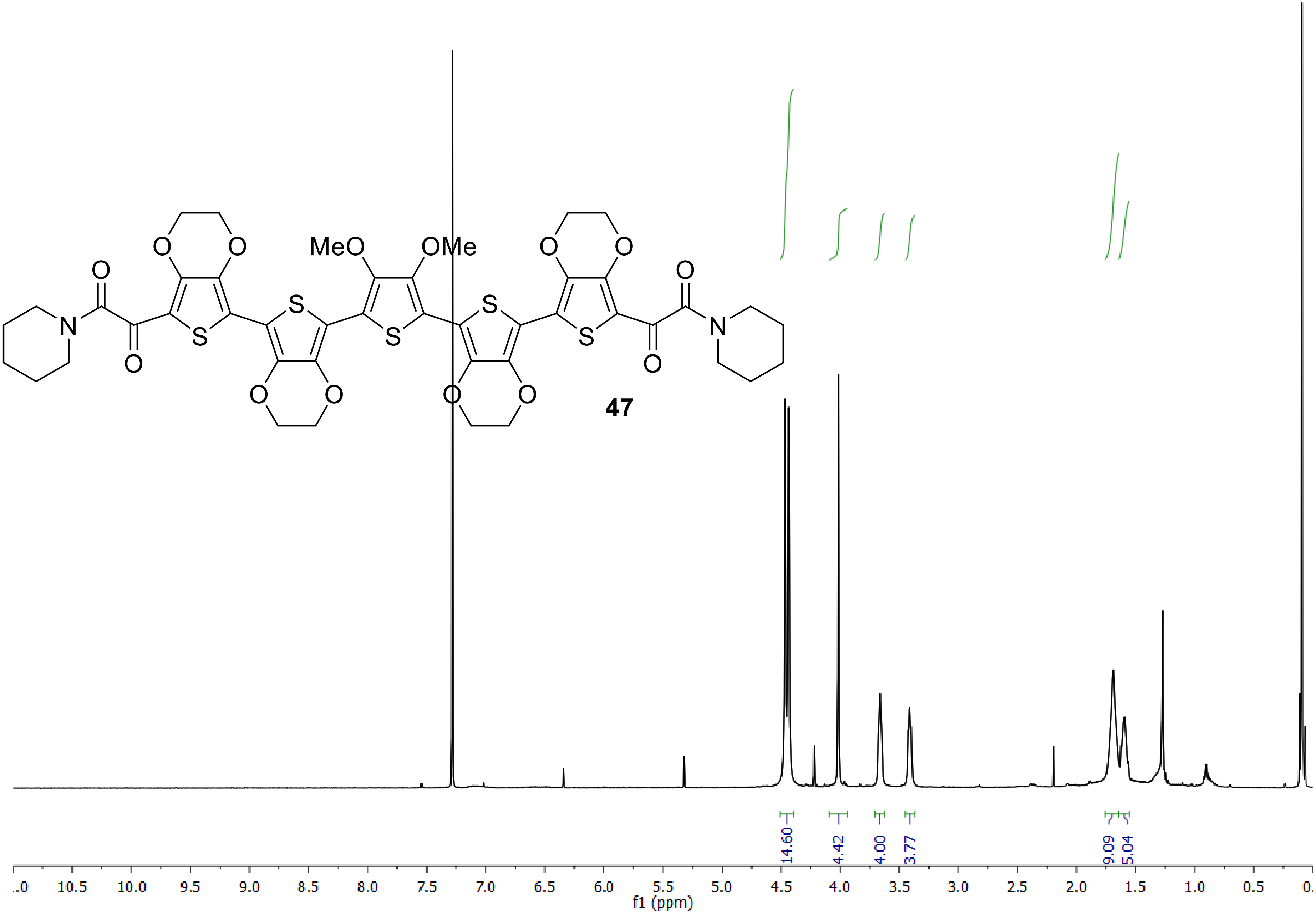

47

^{1}H NMR (400 MHz, $CDCl_3$)

Figure S78. ^{1}H NMR of **48**

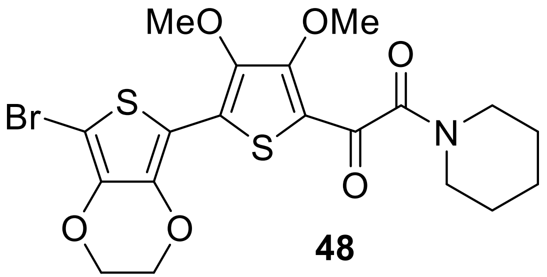

48

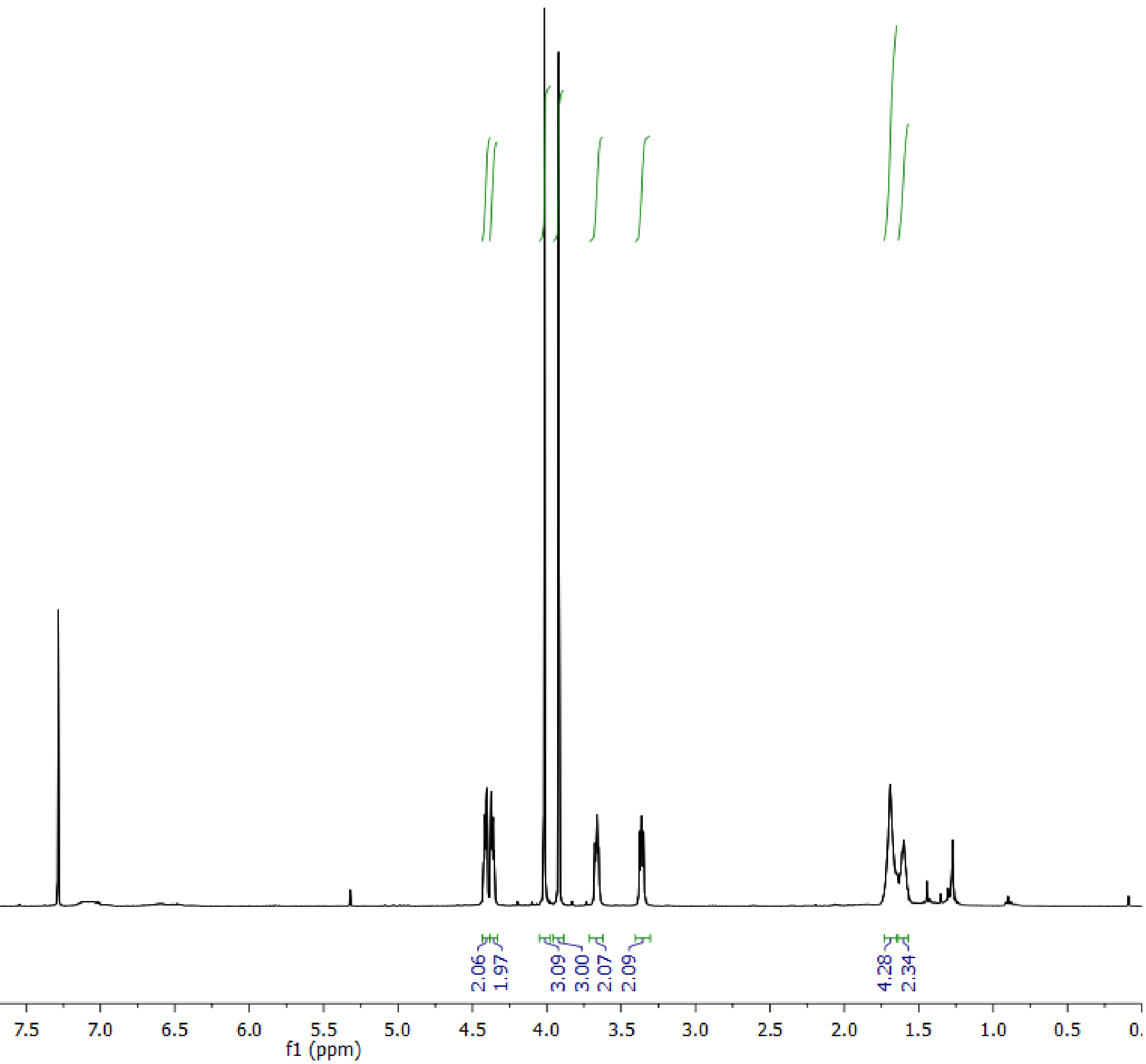

^{13}C NMR (100 MHz, $CDCl_3$)

Figure S79. ^{13}C NMR of **48**

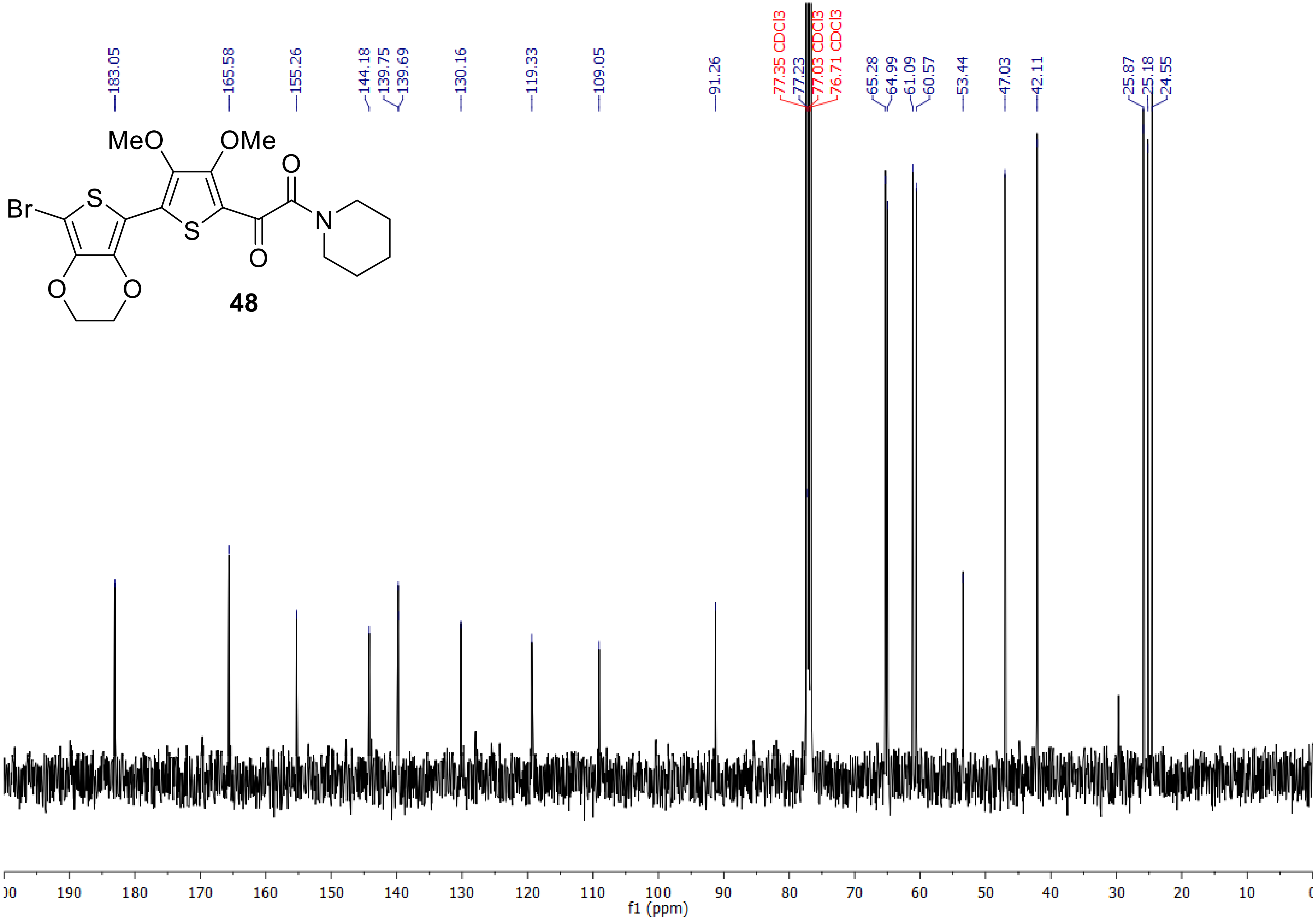

^{1}H NMR (400 MHz, $CDCl_3$)

Figure S80. ^{1}H NMR of **49**

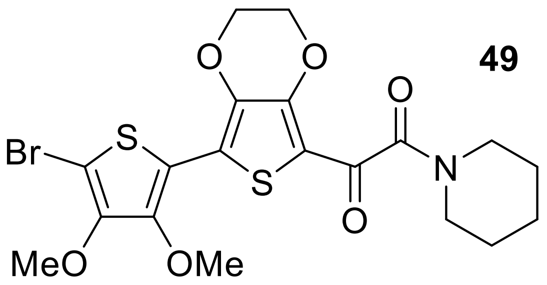

49

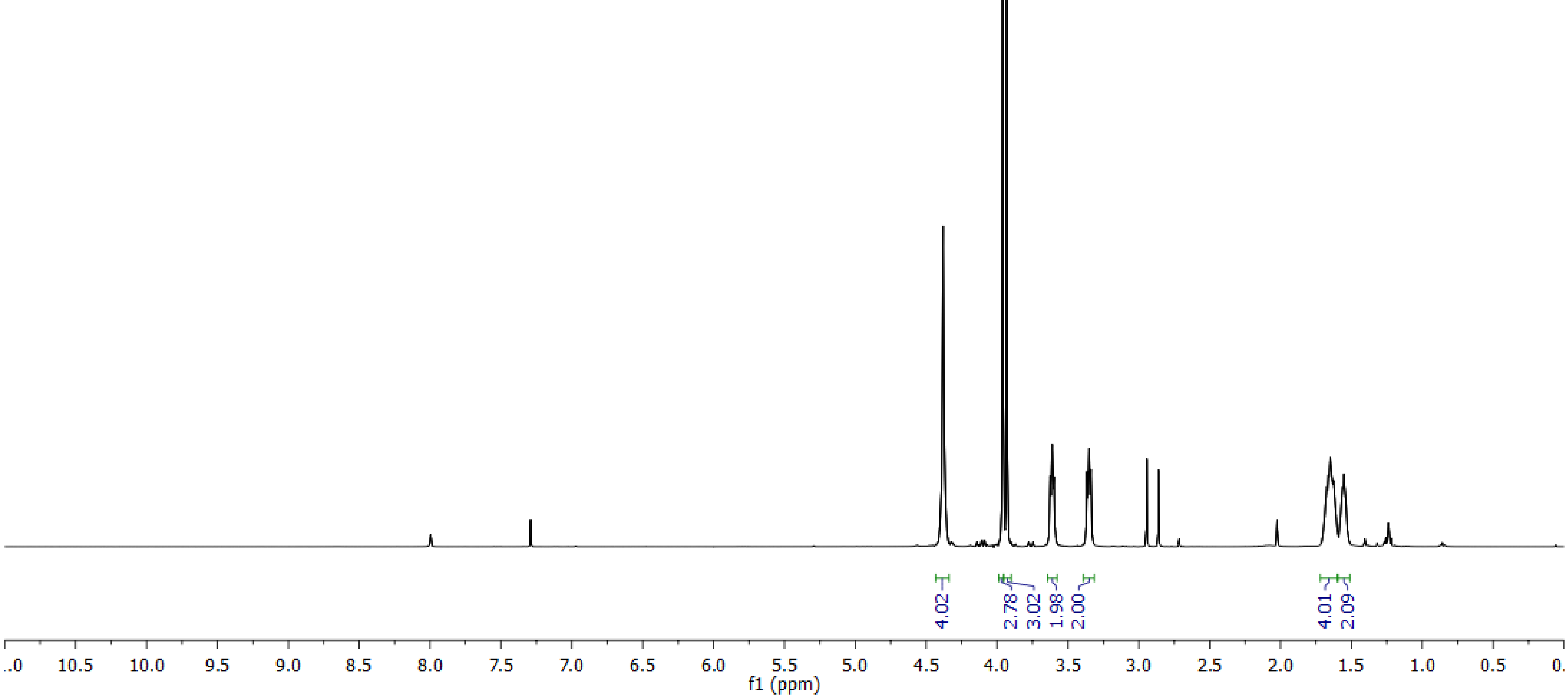

^{13}C NMR (100 MHz, $CDCl_3$)

Figure S81. ^{13}C NMR of **49**

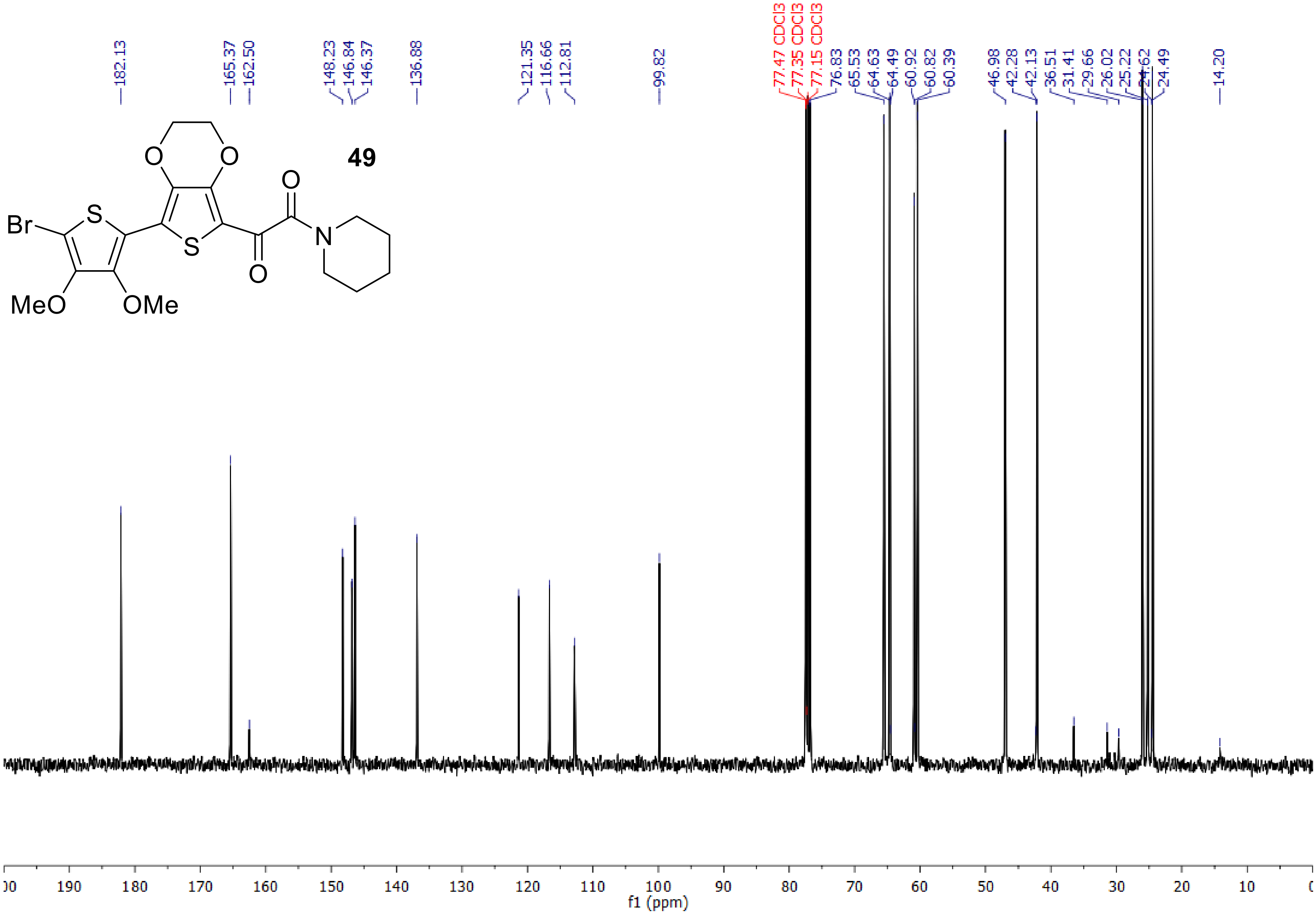

^{1}H NMR (400 MHz, $CDCl_3$)

Figure S82. ^{1}H NMR of **50**

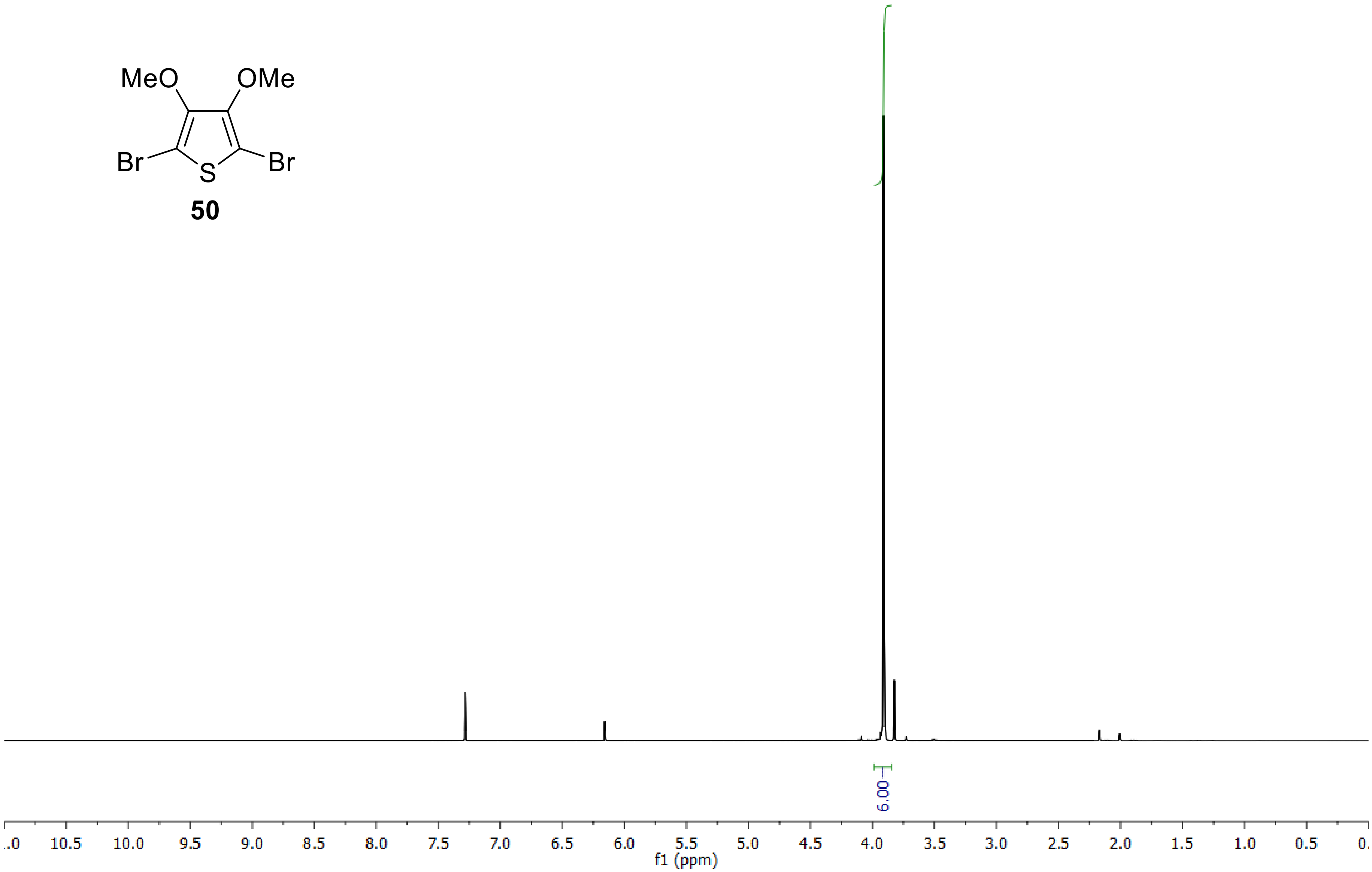

^{13}C NMR (100 MHz, $CDCl_3$)

Figure S83. ^{13}C NMR of **50**

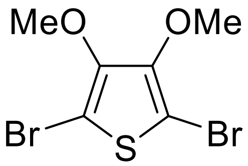

50

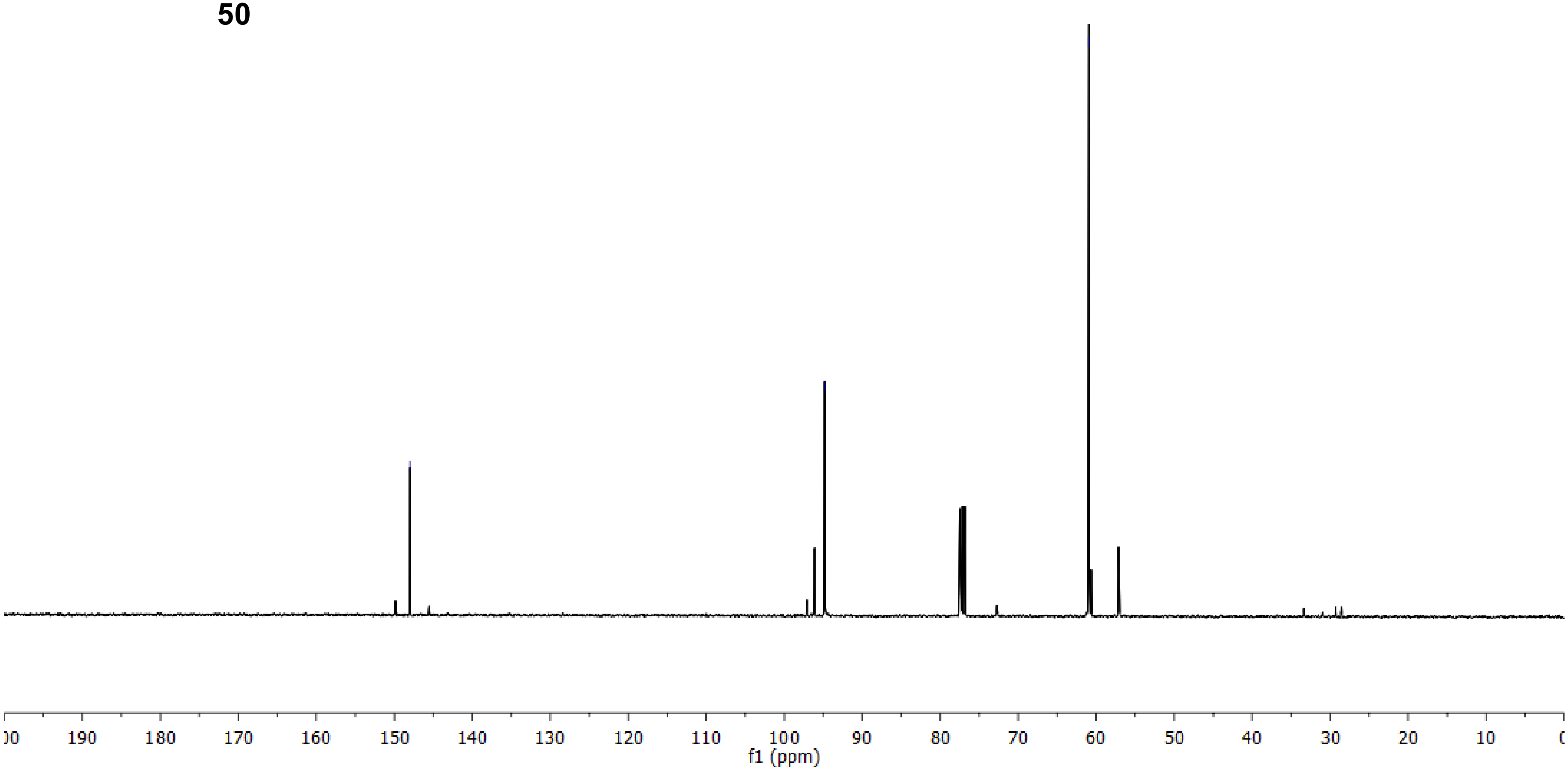

^{1}H NMR (400 MHz, $CDCl_3$)

Figure S84. ^{1}H NMR of **55**

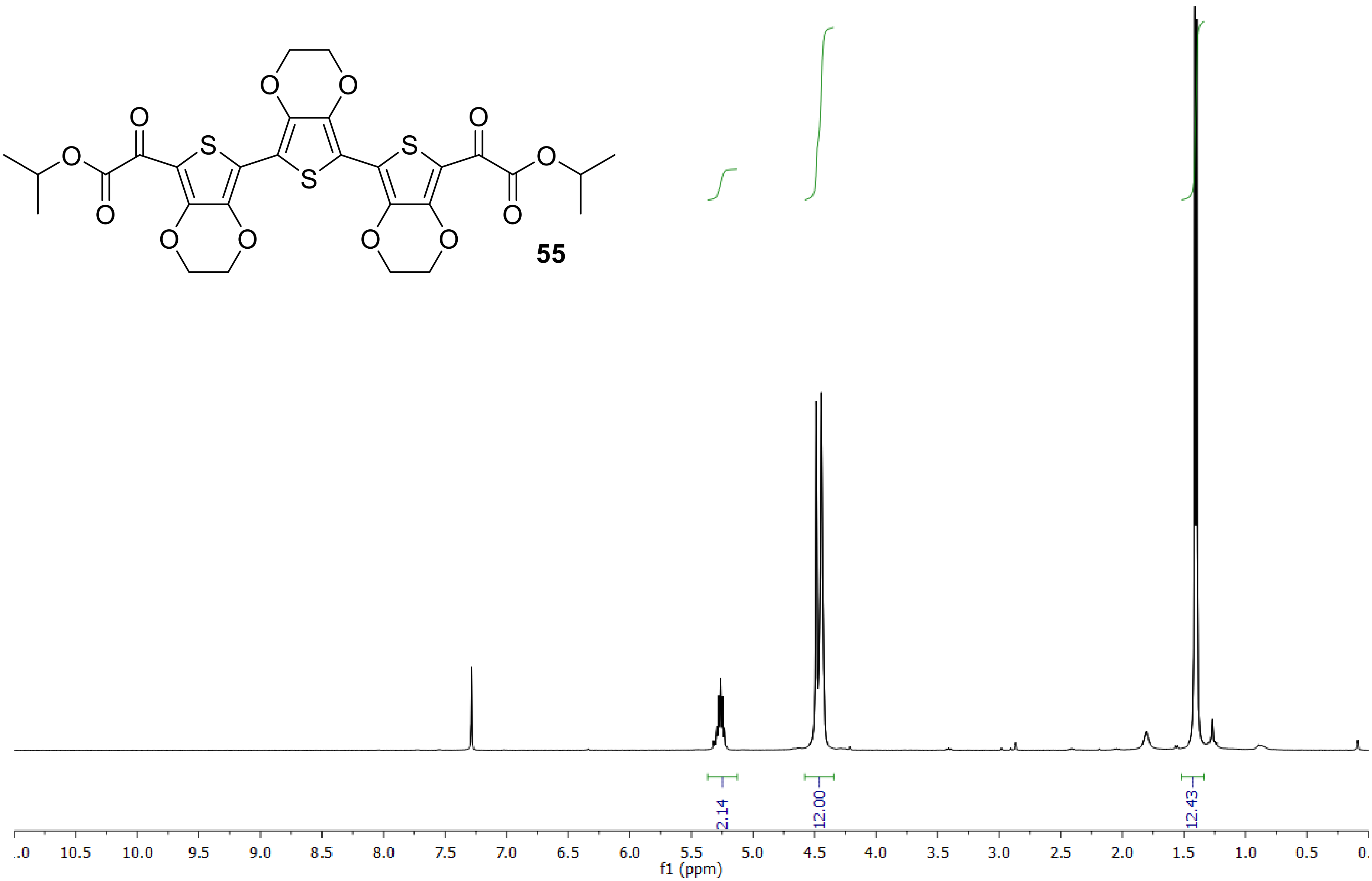

^{13}C NMR (100 MHz, $CDCl_3$)

Figure S85. ^{13}C NMR of **55**

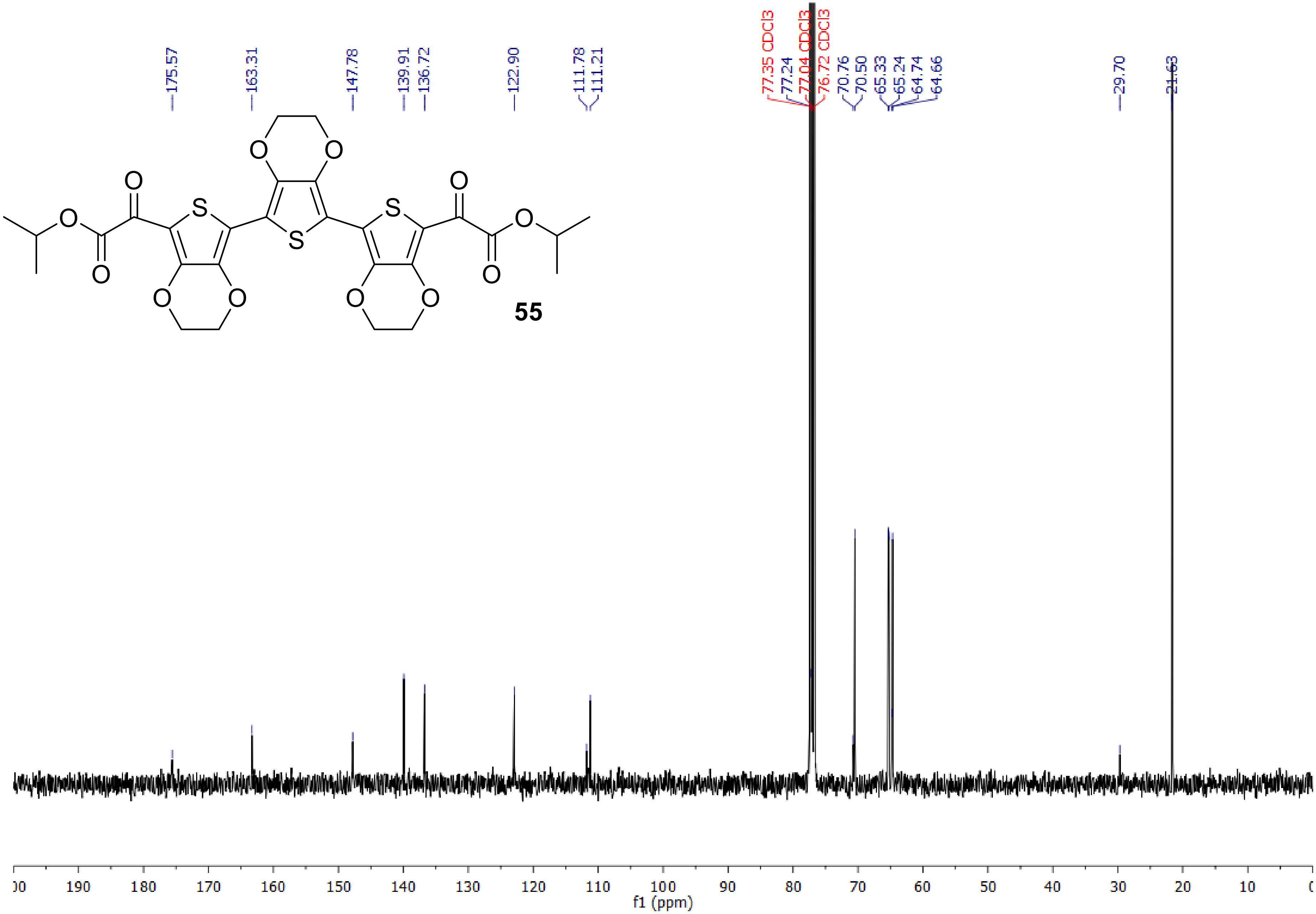

^1H NMR (400 MHz, $CDCl_3$)

Figure S86. ^1H NMR of **56**

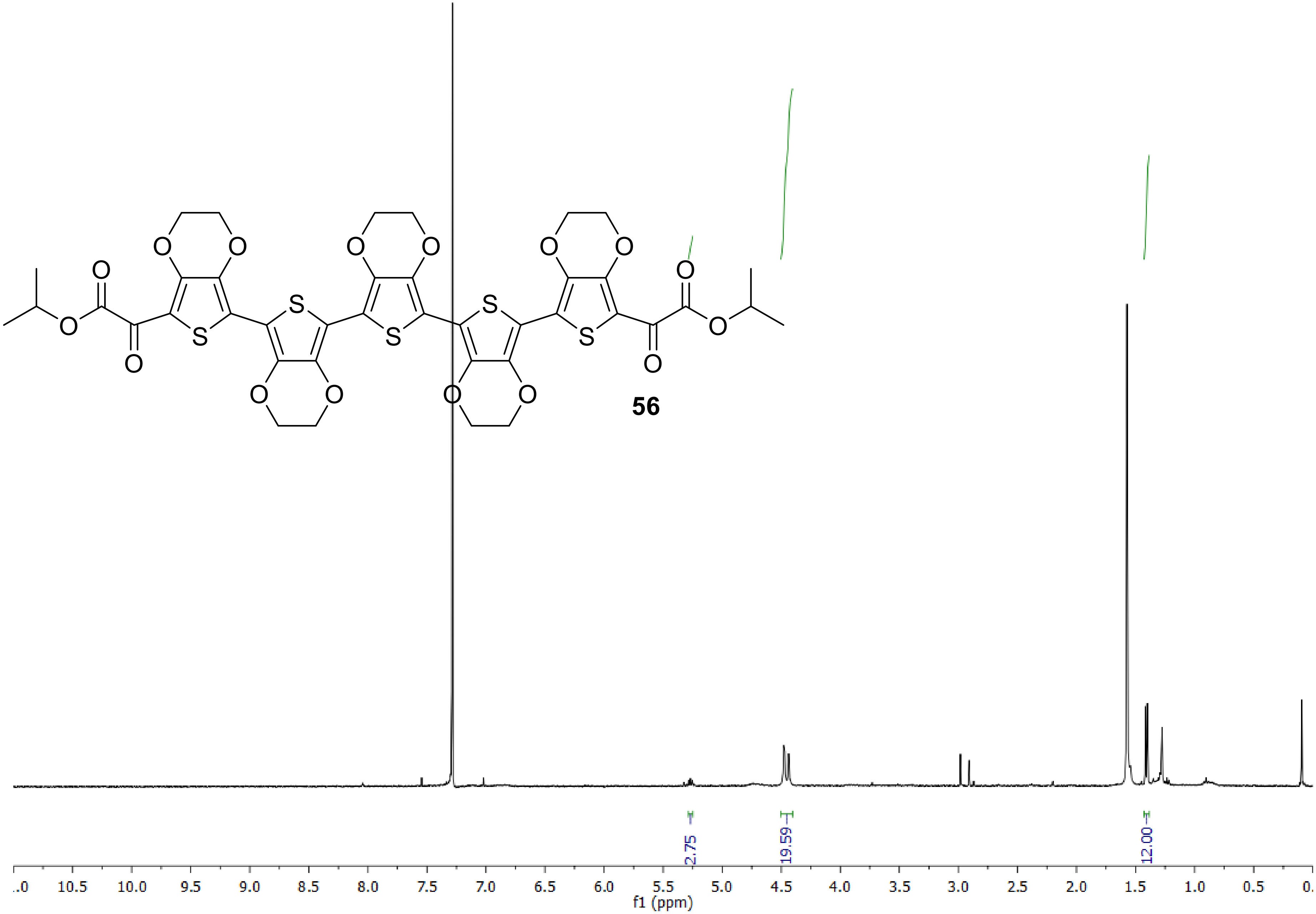

^{1}H NMR (400 MHz, $CDCl_3$)

Figure S87. ^{1}H NMR of **57**

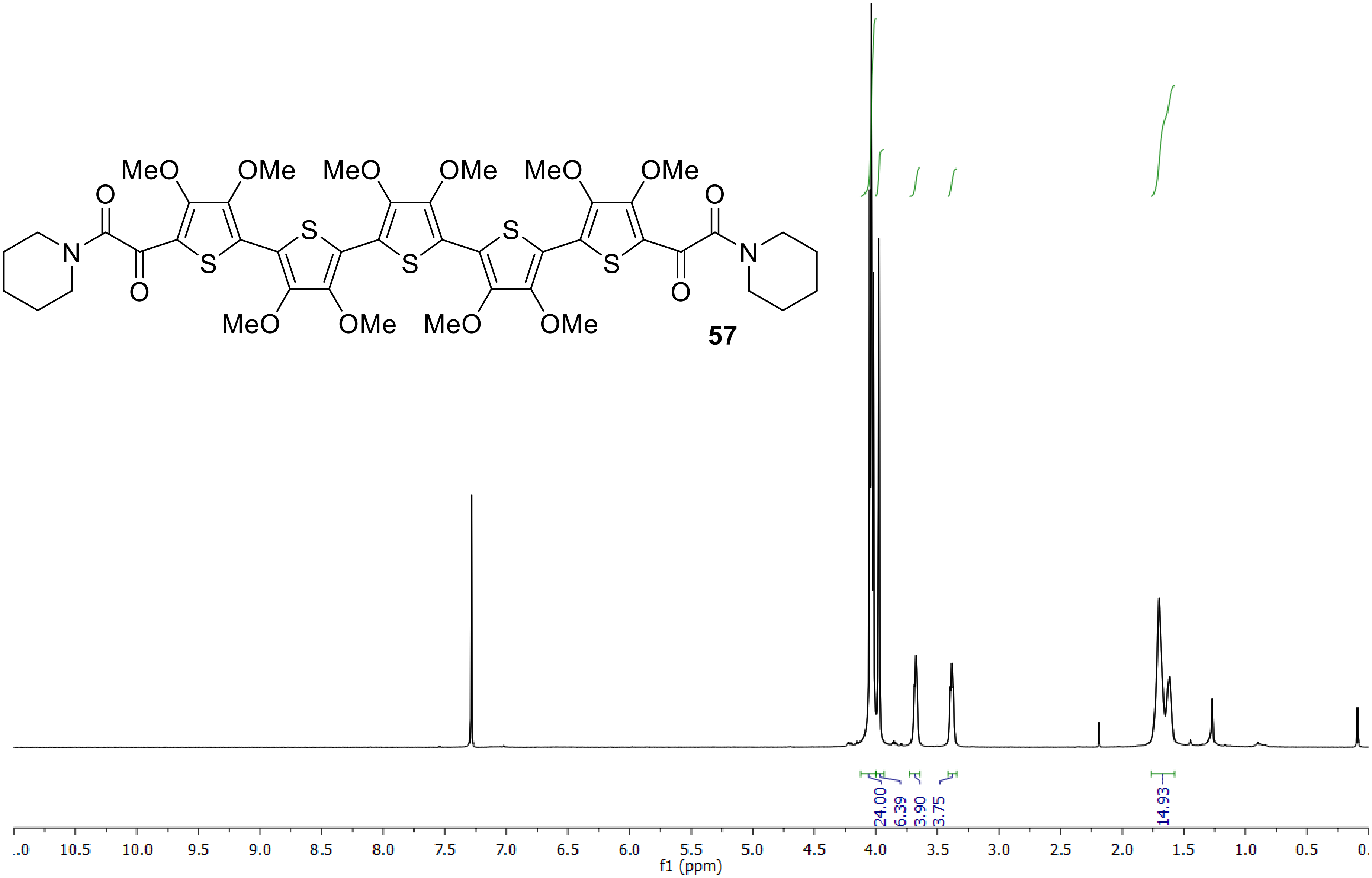

^{1}H NMR (400 MHz, $CDCl_3$)

Figure S88. ^{1}H NMR of **58**

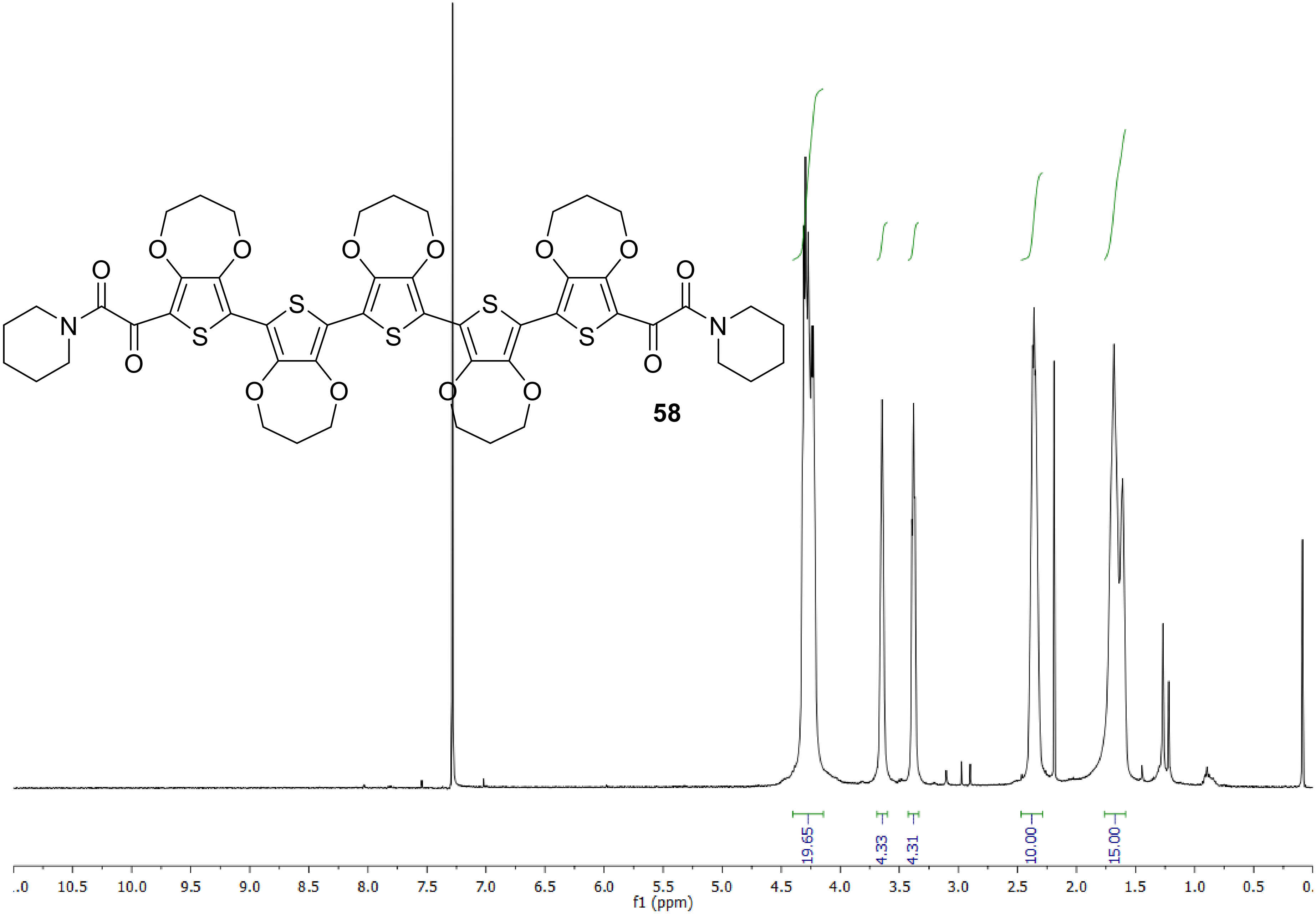

^{1}H NMR (400 MHz, $CDCl_3$)

Figure S89. ^{1}H NMR of **60**

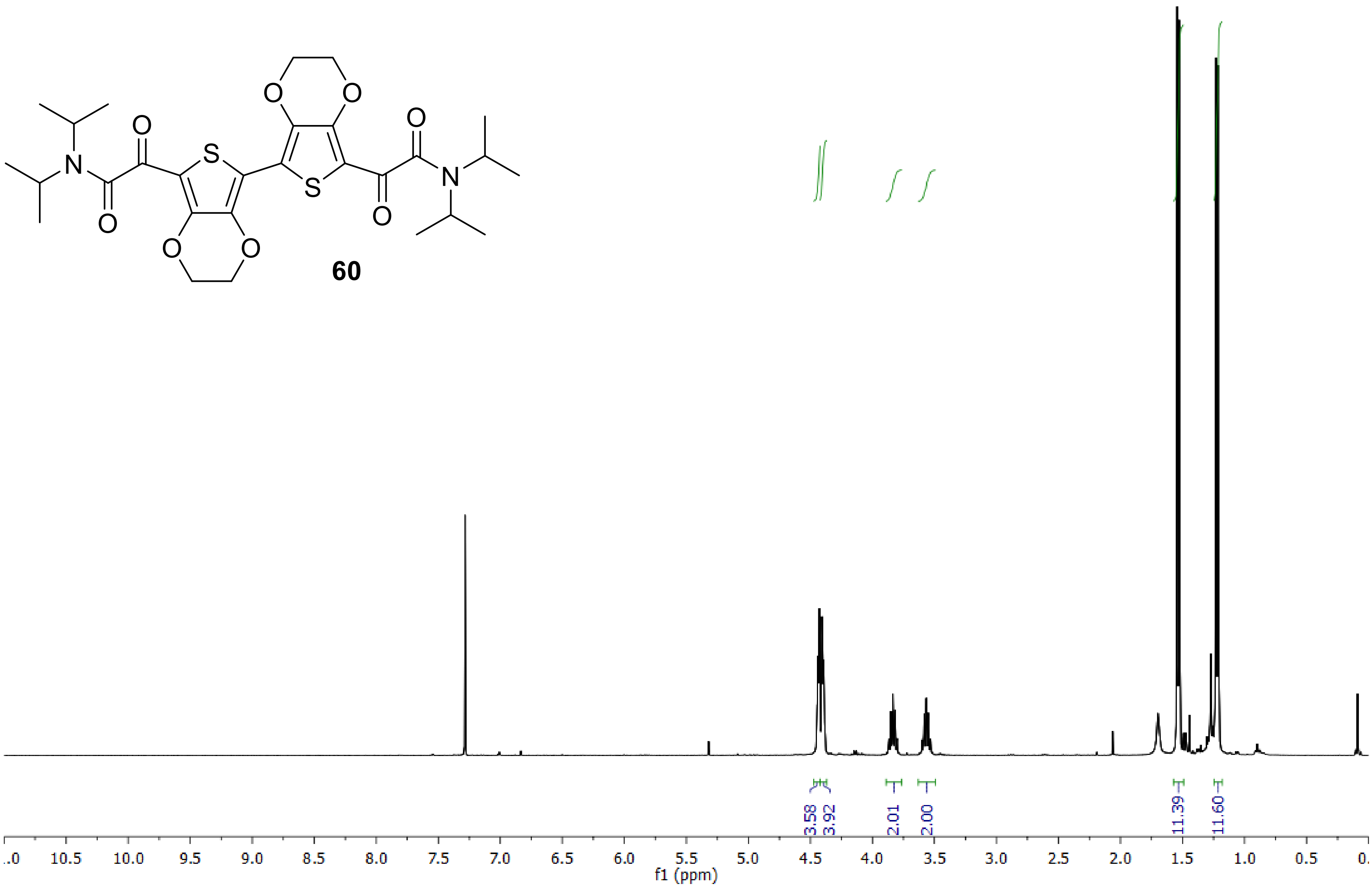

^{13}C NMR (100 MHz, $CDCl_3$)

Figure S90. ^{13}C NMR of **60**

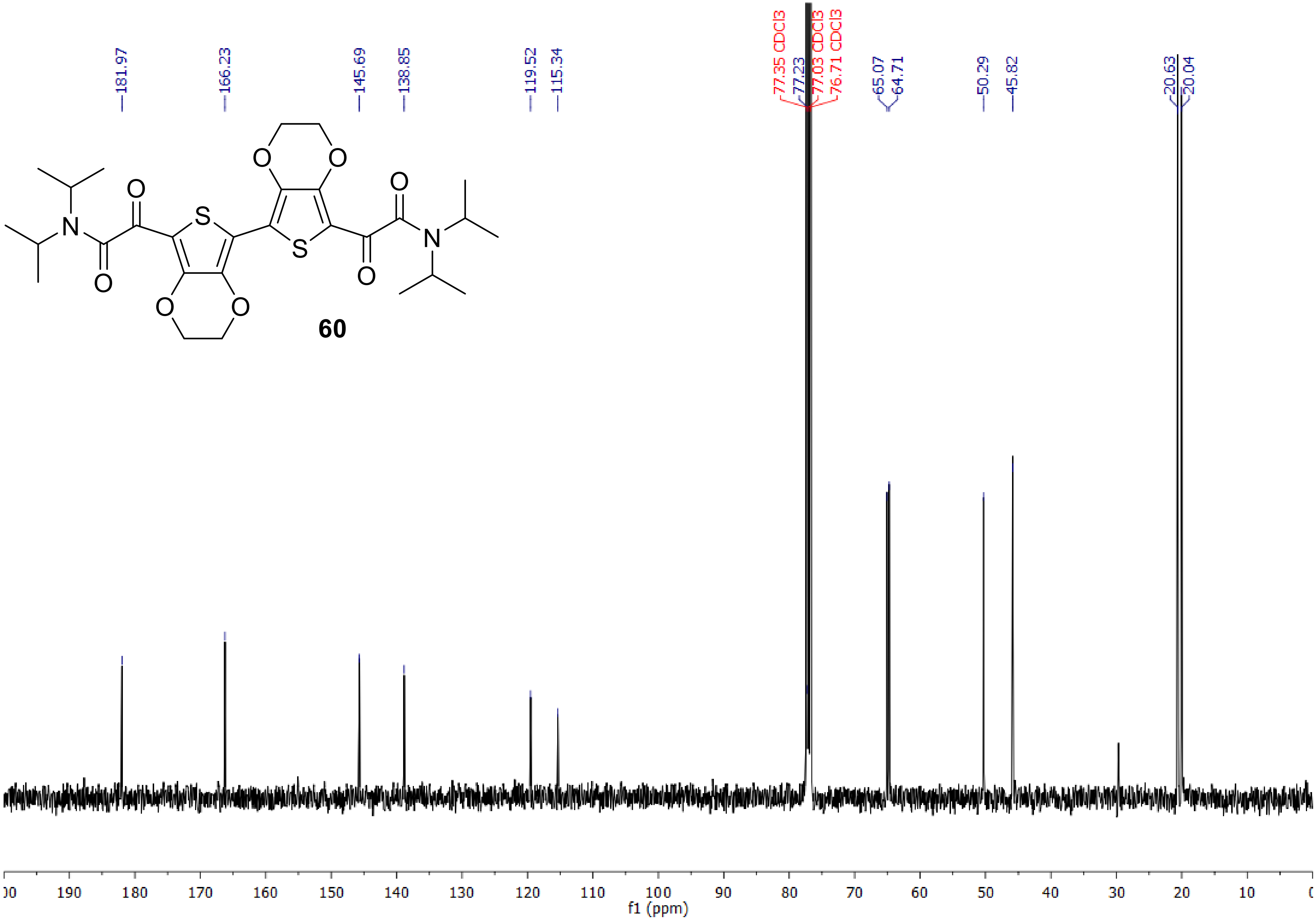

^{1}H NMR (400 MHz, $CDCl_3$)

Figure S91. ^{1}H NMR of **65**

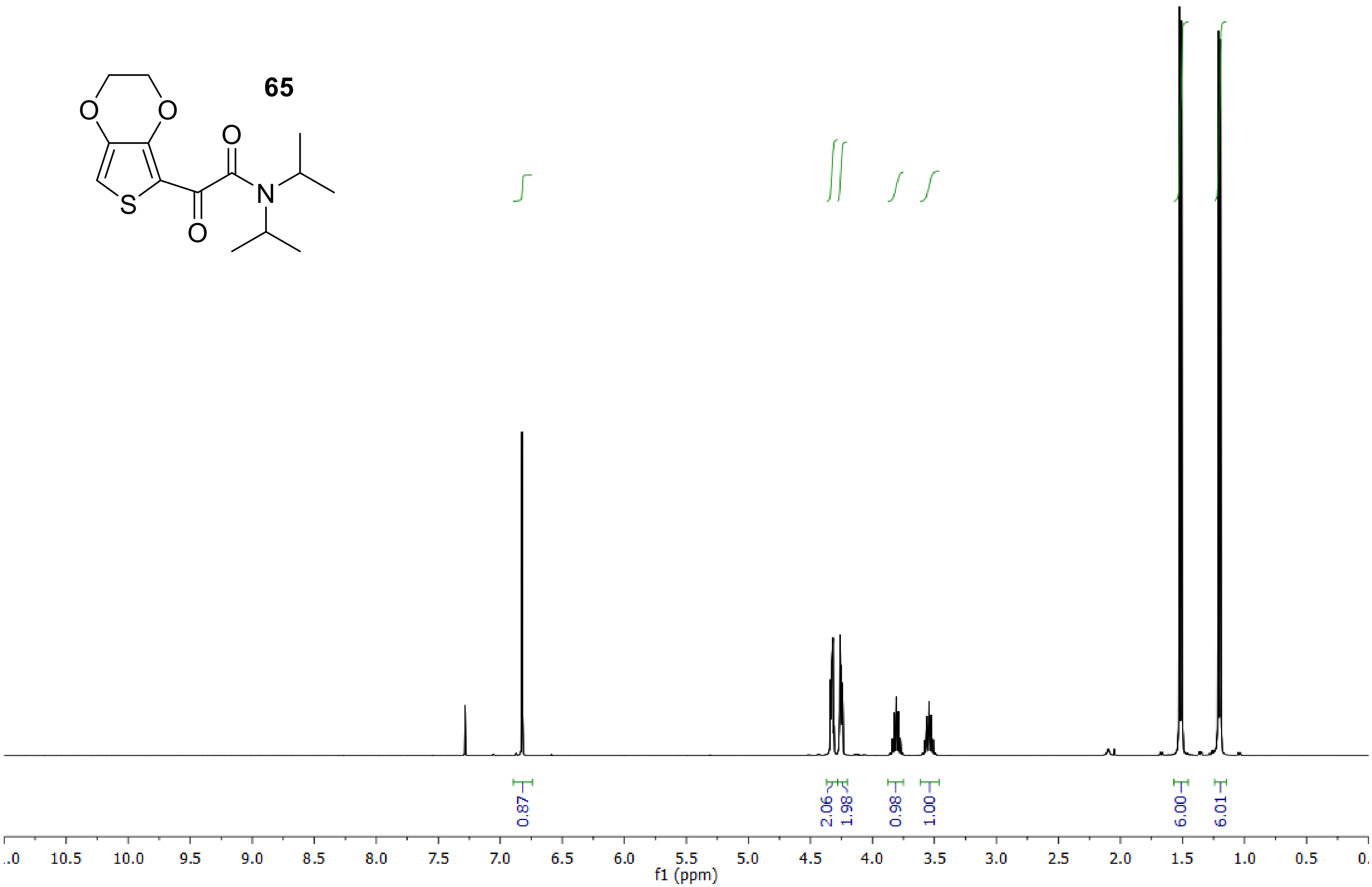

^{13}C NMR (100 MHz, $CDCl_3$)

Figure S92. ^{13}C NMR of **65**

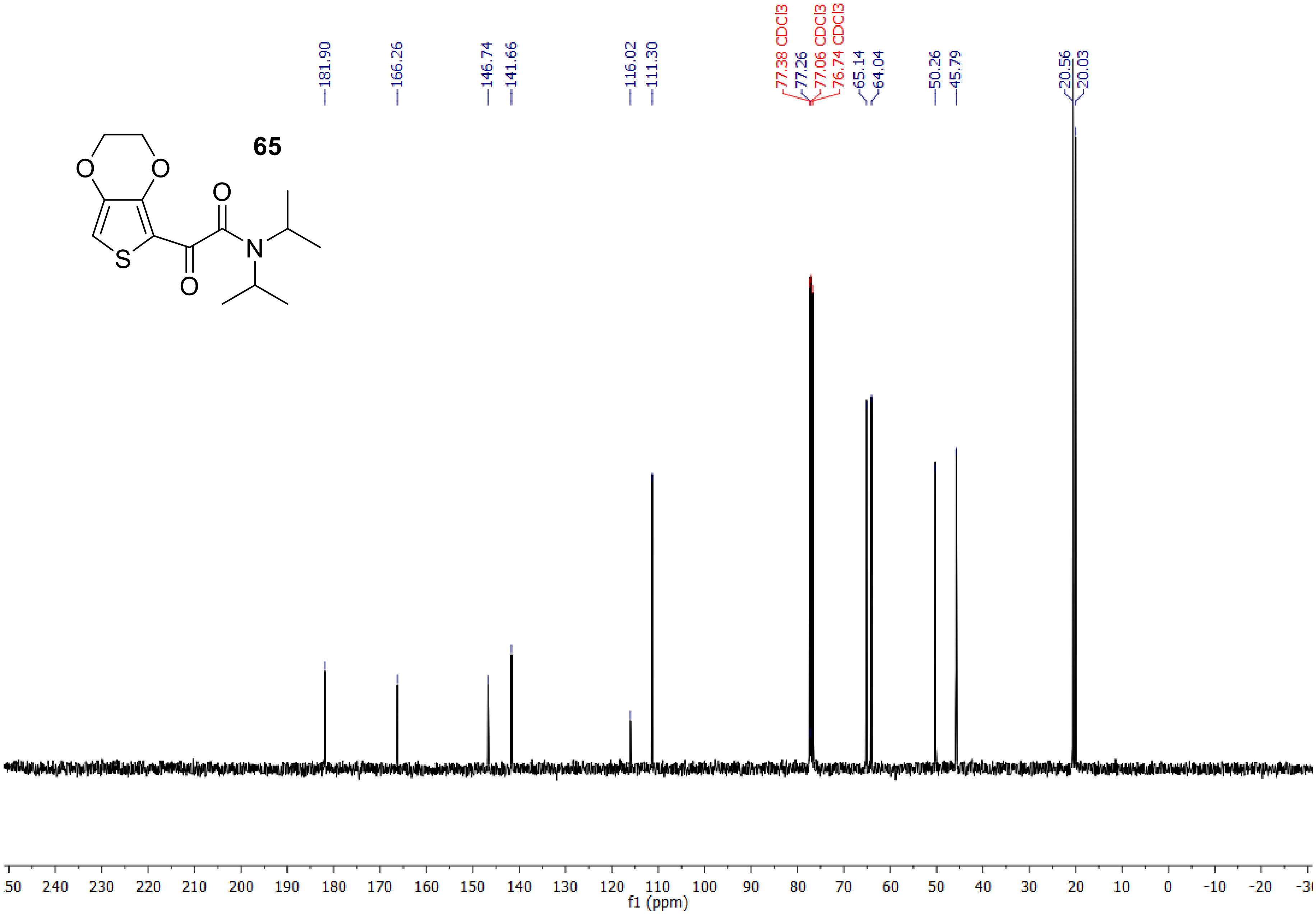

^{1}H NMR (400 MHz, $CDCl_3$)

Figure S93. ^{1}H NMR of **66**

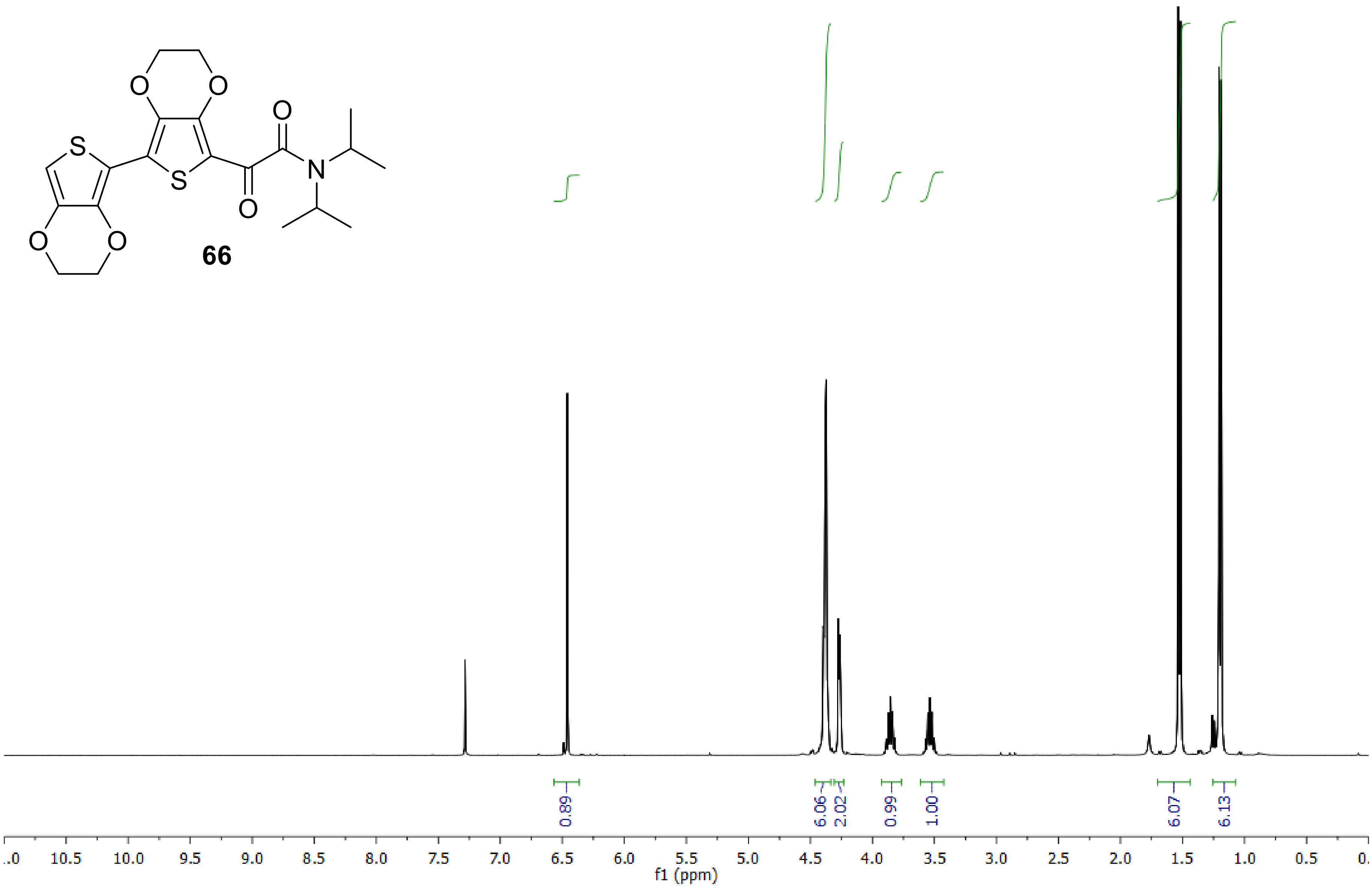

^{13}C NMR (100 MHz, $CDCl_3$)

Figure S94. ^{13}C NMR of **66**

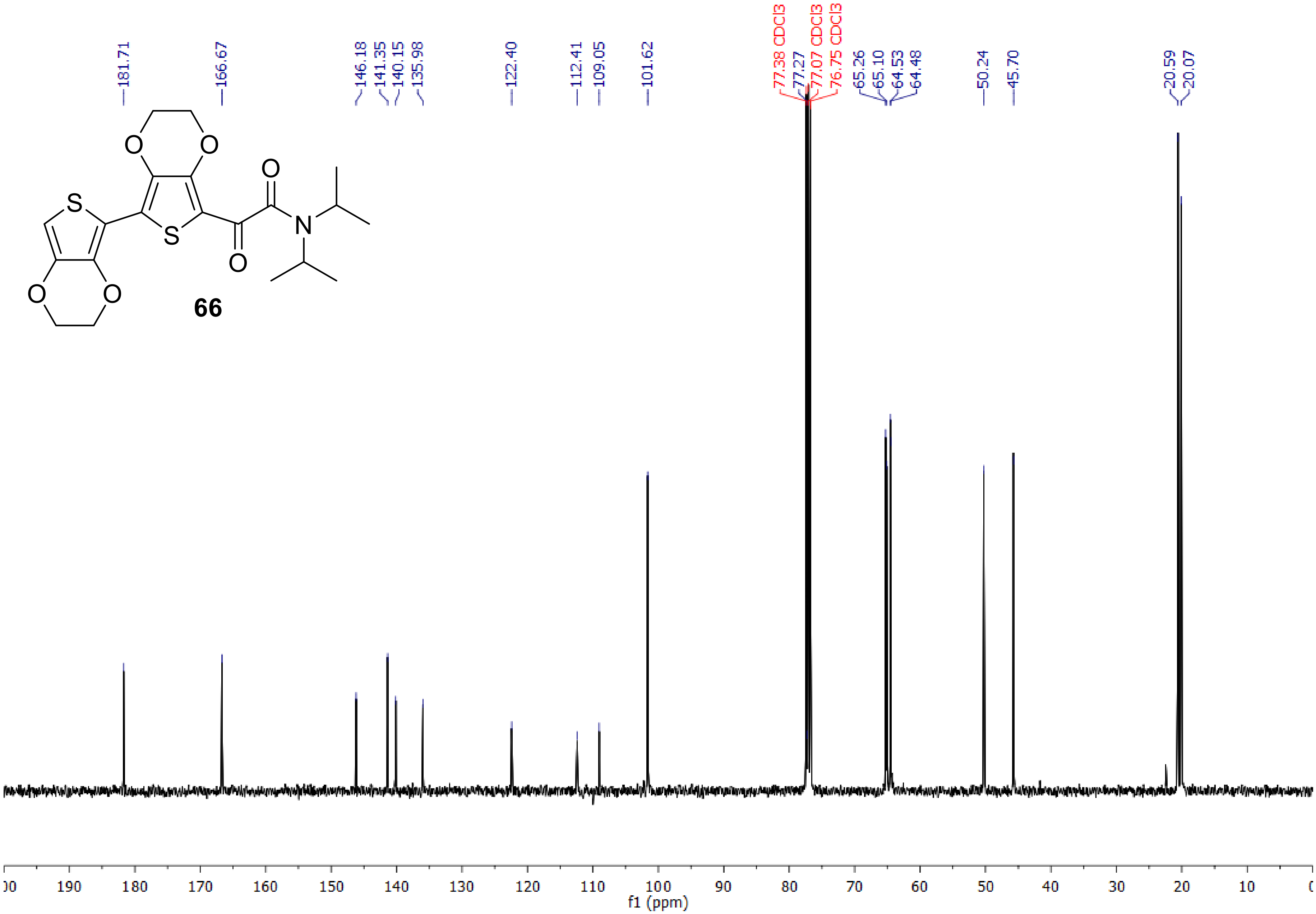

^{1}H NMR (400 MHz, $CDCl_3$)

Figure S95. ^{1}H NMR of **67**

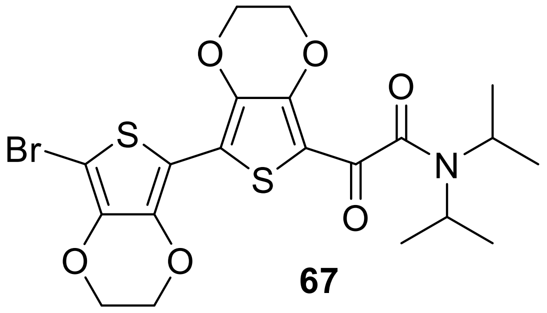

67

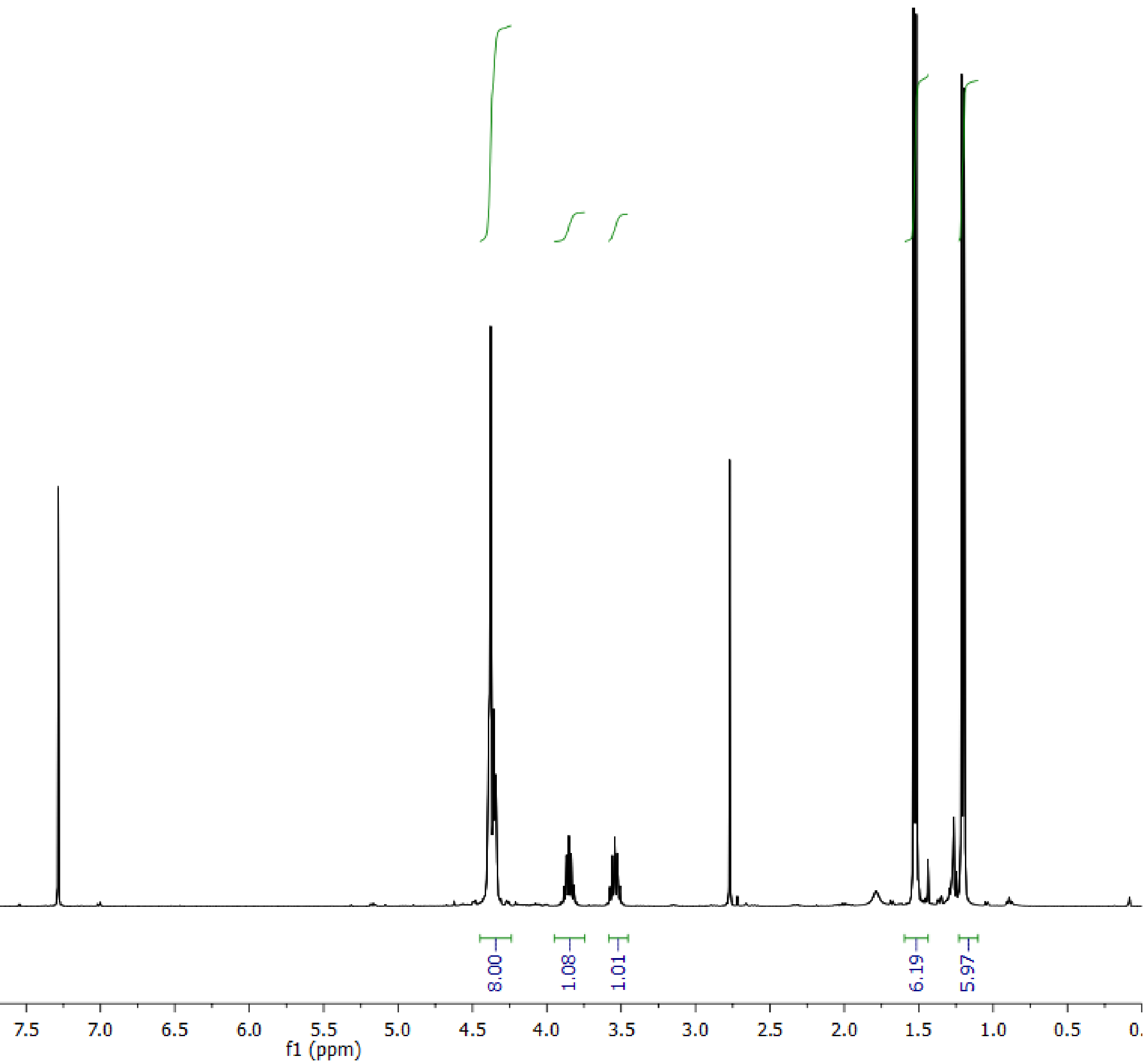

^{13}C NMR (100 MHz, $CDCl_3$)

Figure S96. ^{13}C NMR of **67**

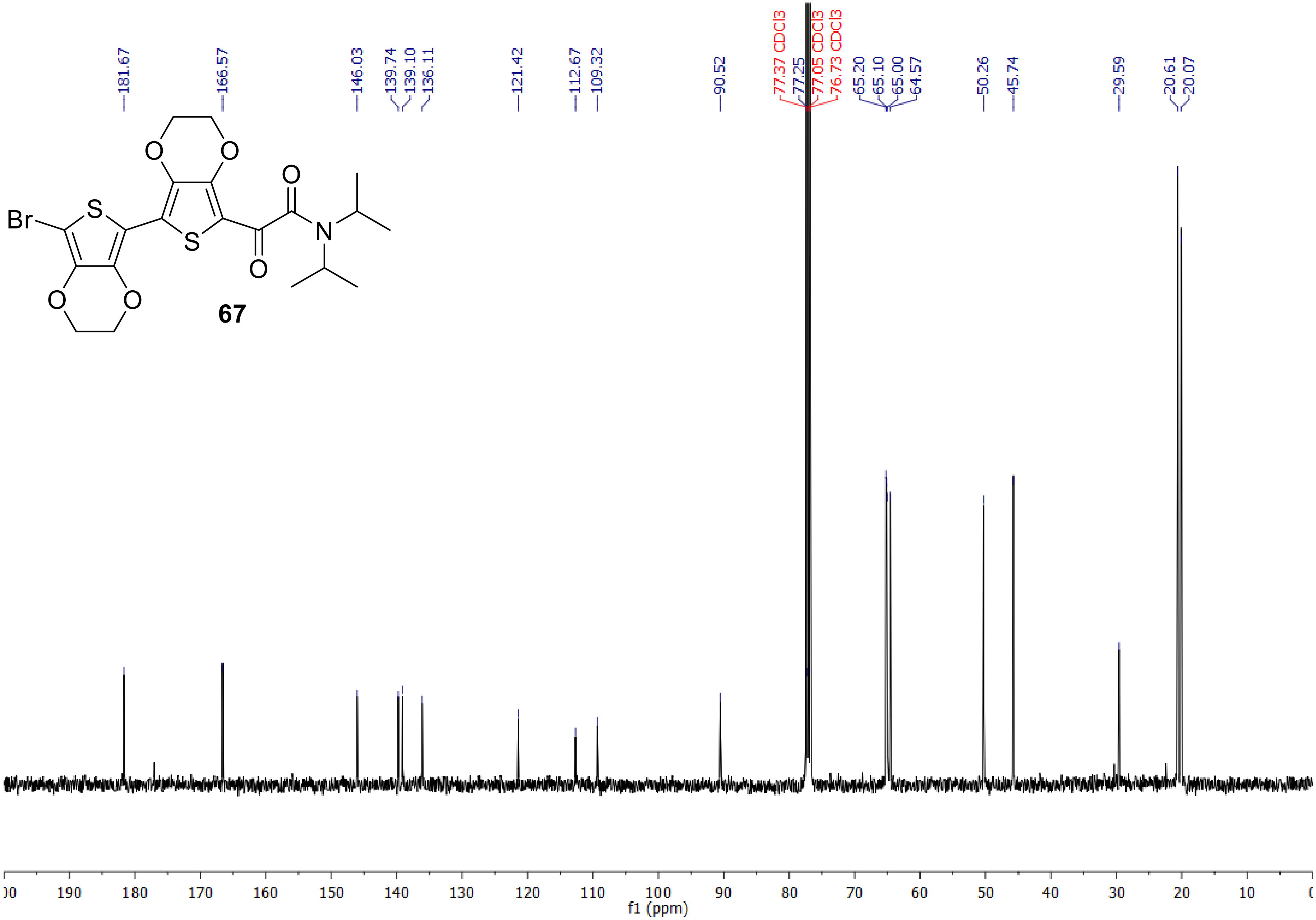

¹H NMR (400 MHz, $CDCl_3$)

Figure S97. ¹H NMR of **68**

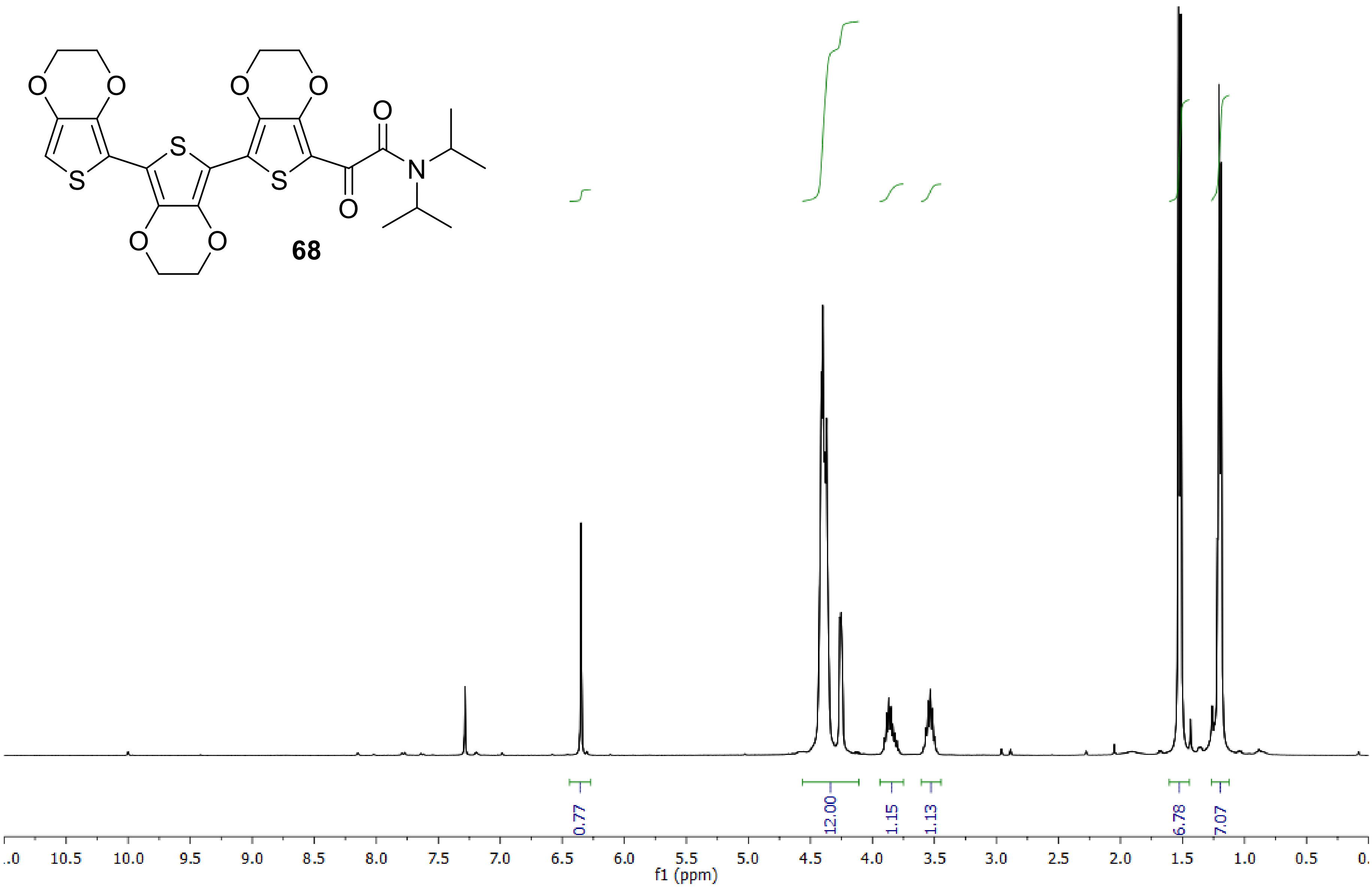

^{13}C NMR (100 MHz, $CDCl_3$)

Figure S98. ^{13}C NMR of **68**

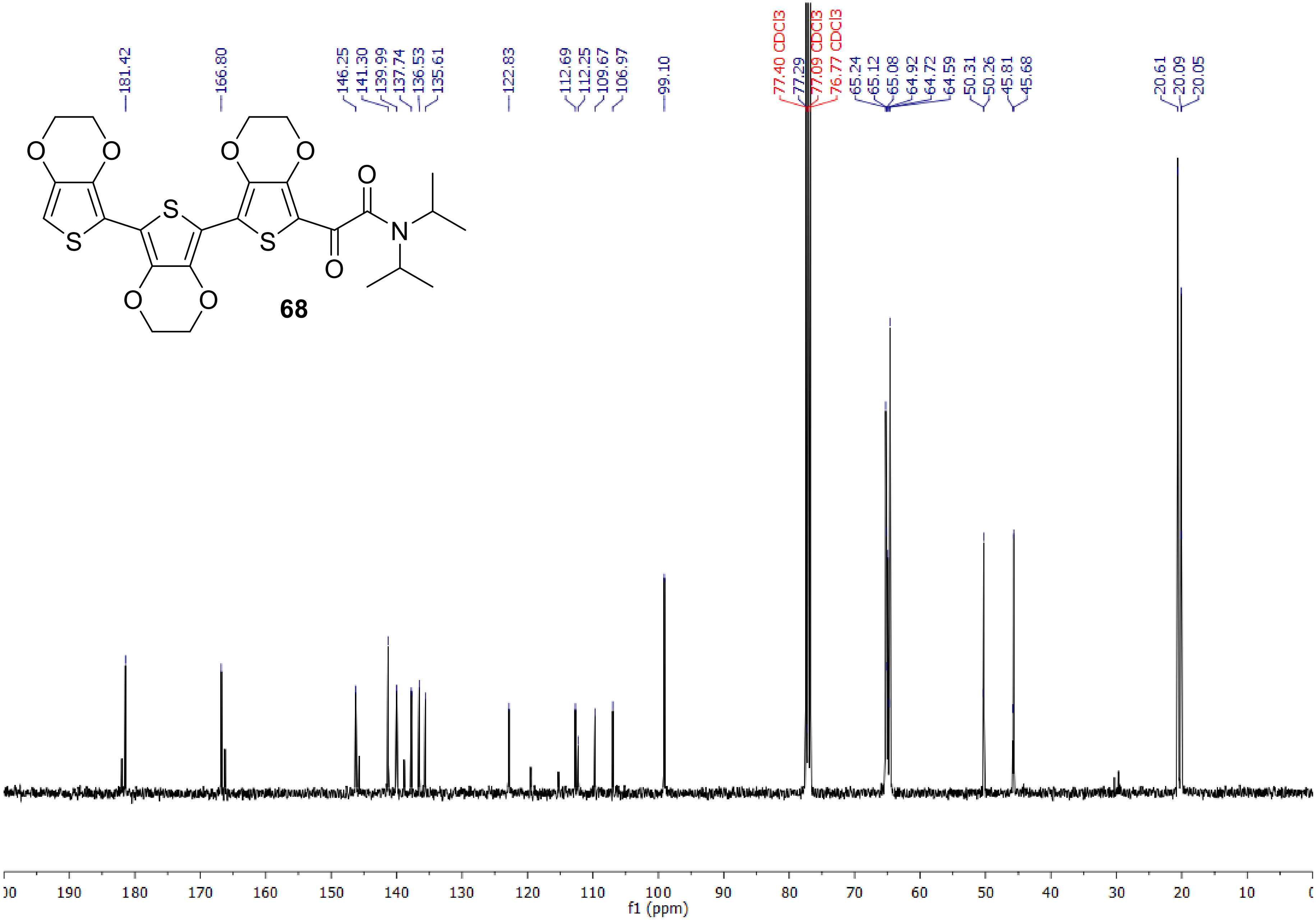

^1H NMR (400 MHz, $CDCl_3$)

Figure S99. ^1H NMR of **69**

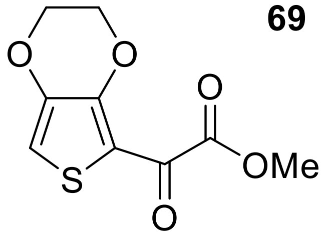

69

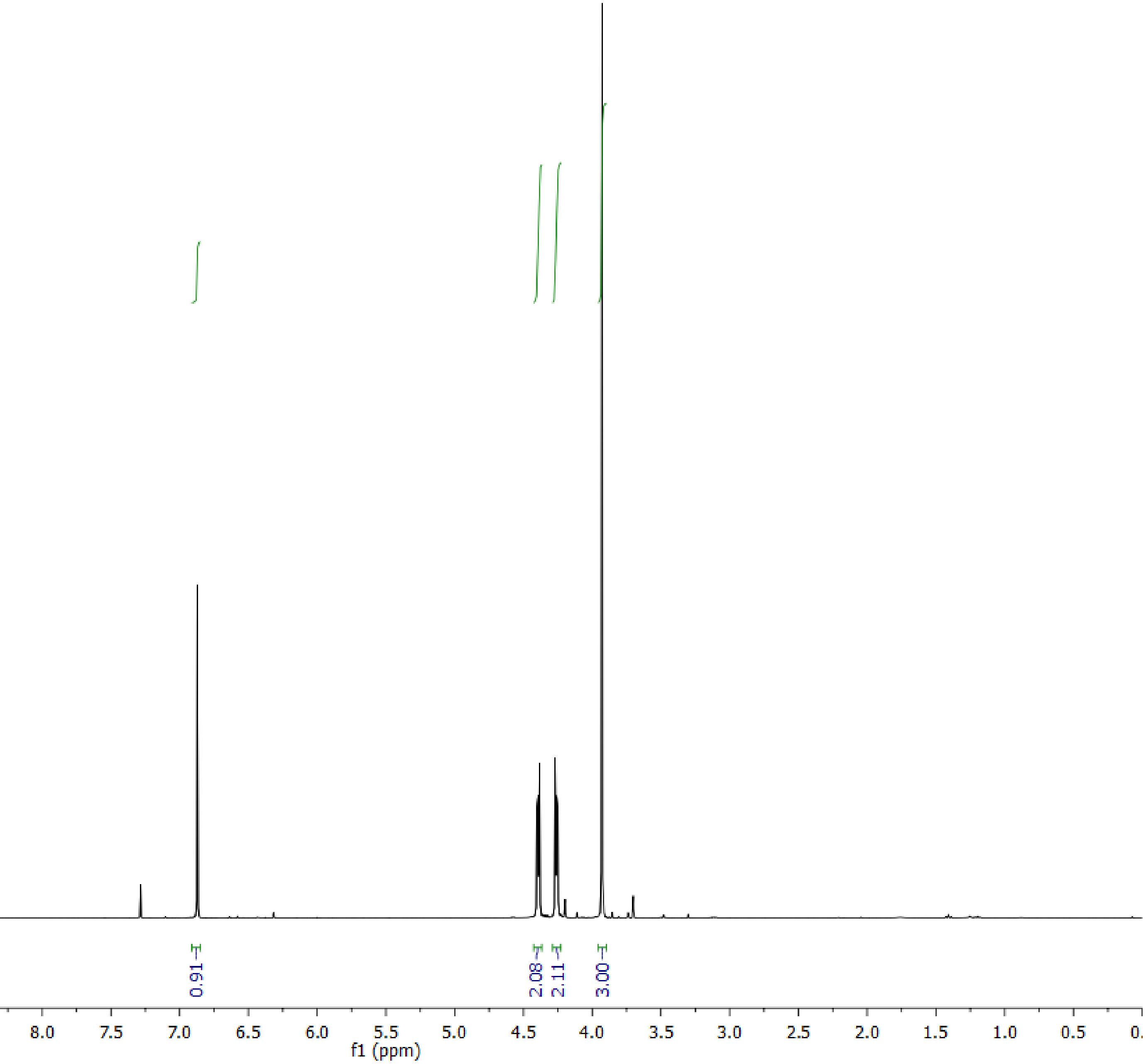

^{13}C NMR (100 MHz, $CDCl_3$)

Figure S100. ^{13}C NMR of **69**

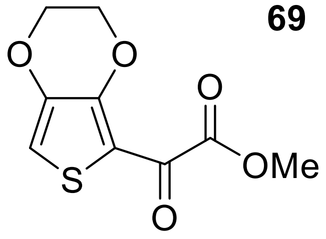

69

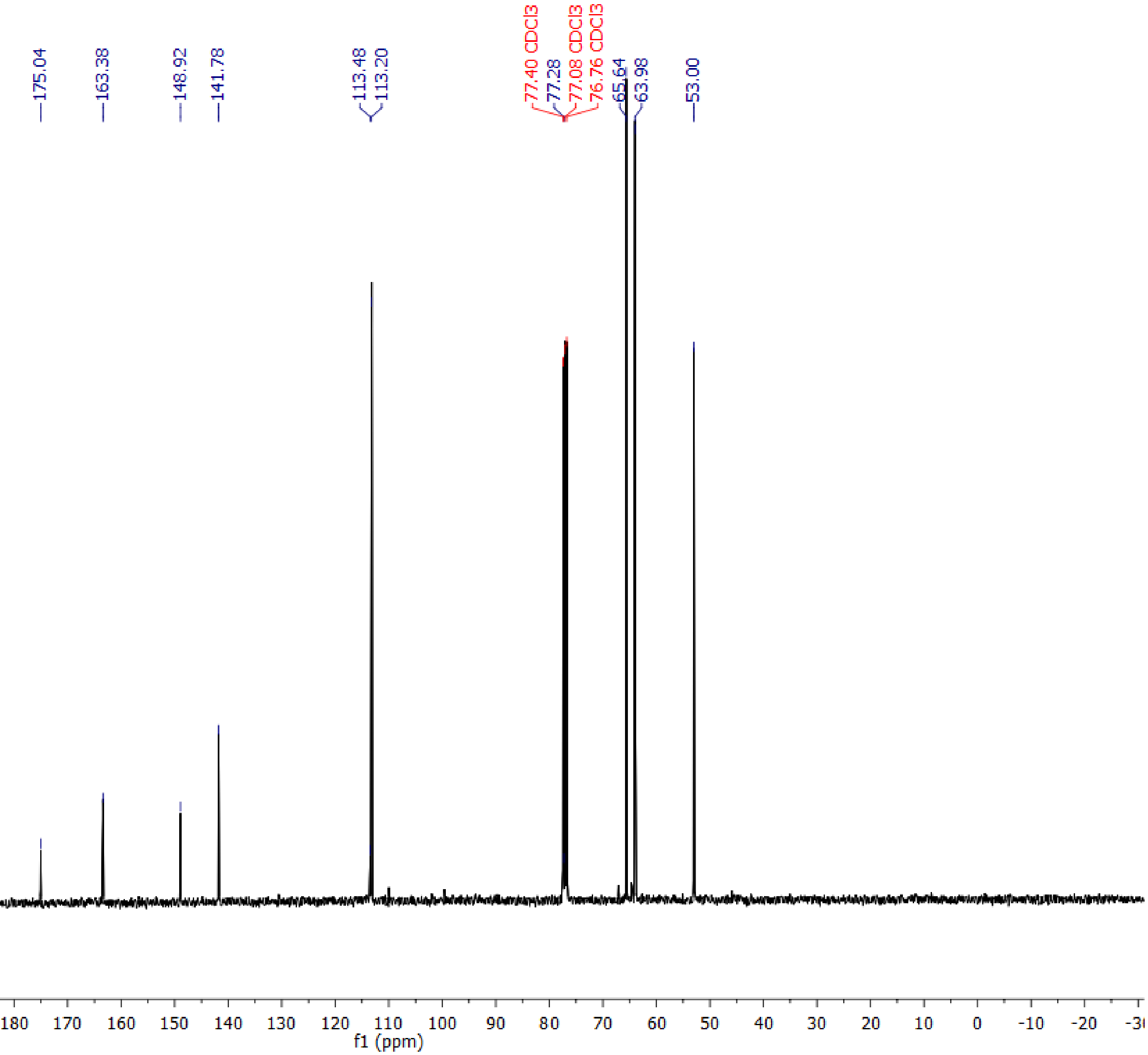

^{1}H NMR (400 MHz, $CDCl_3$)

Figure S101. ^{1}H NMR of **72**

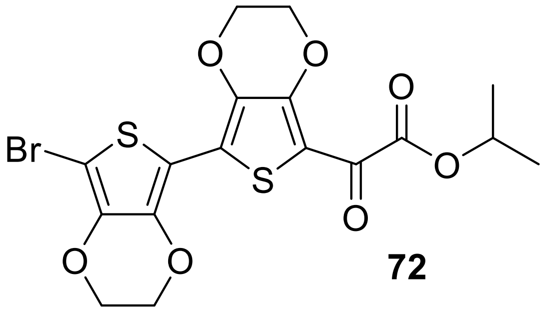

72

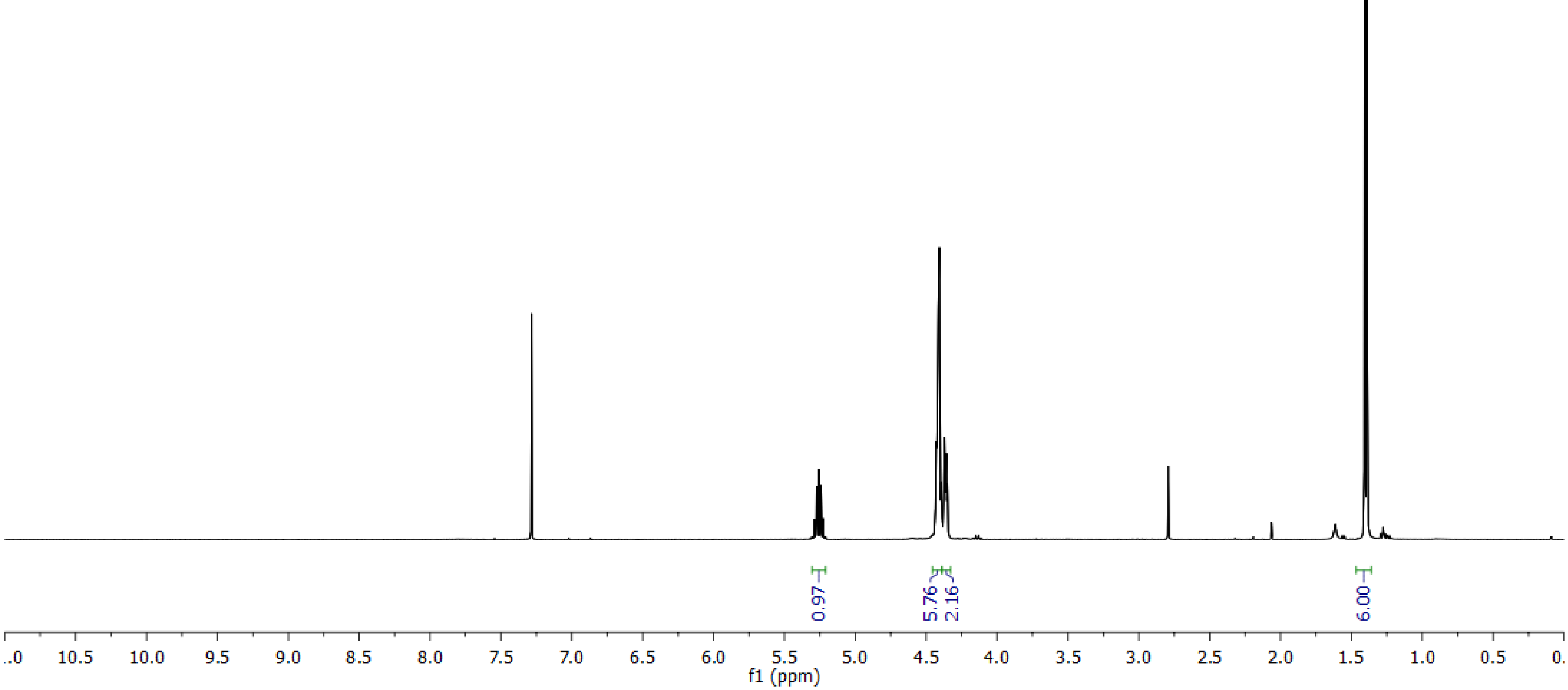

^{13}C NMR (100 MHz, $CDCl_3$)

Figure S102. ^{13}C NMR of **72**

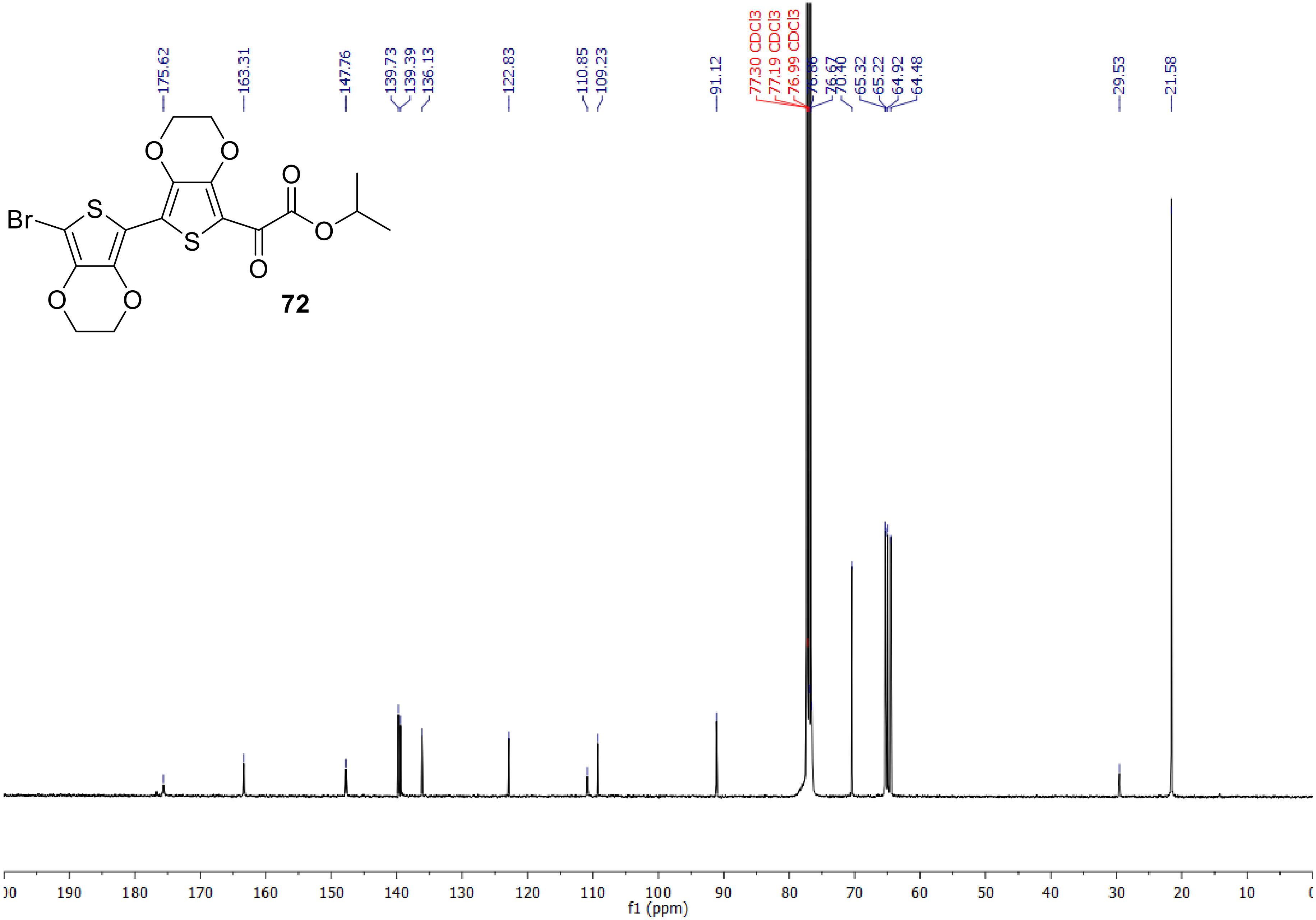

^{1}H NMR (400 MHz, $CDCl_3$)

Figure S103. ^{1}H NMR of **73**

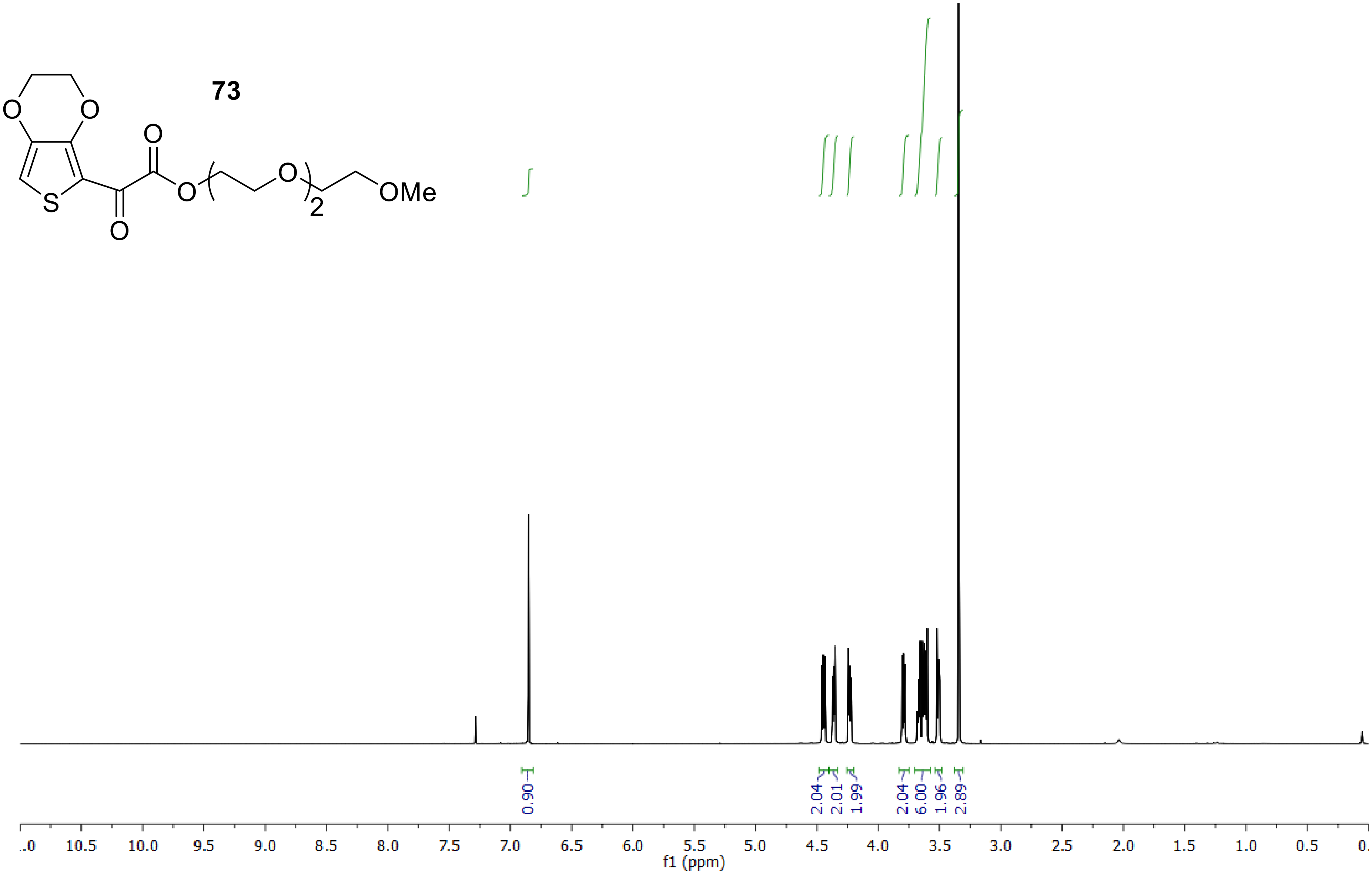

^{13}C NMR (100 MHz, $CDCl_3$)

Figure S104. ^{13}C NMR of **73**

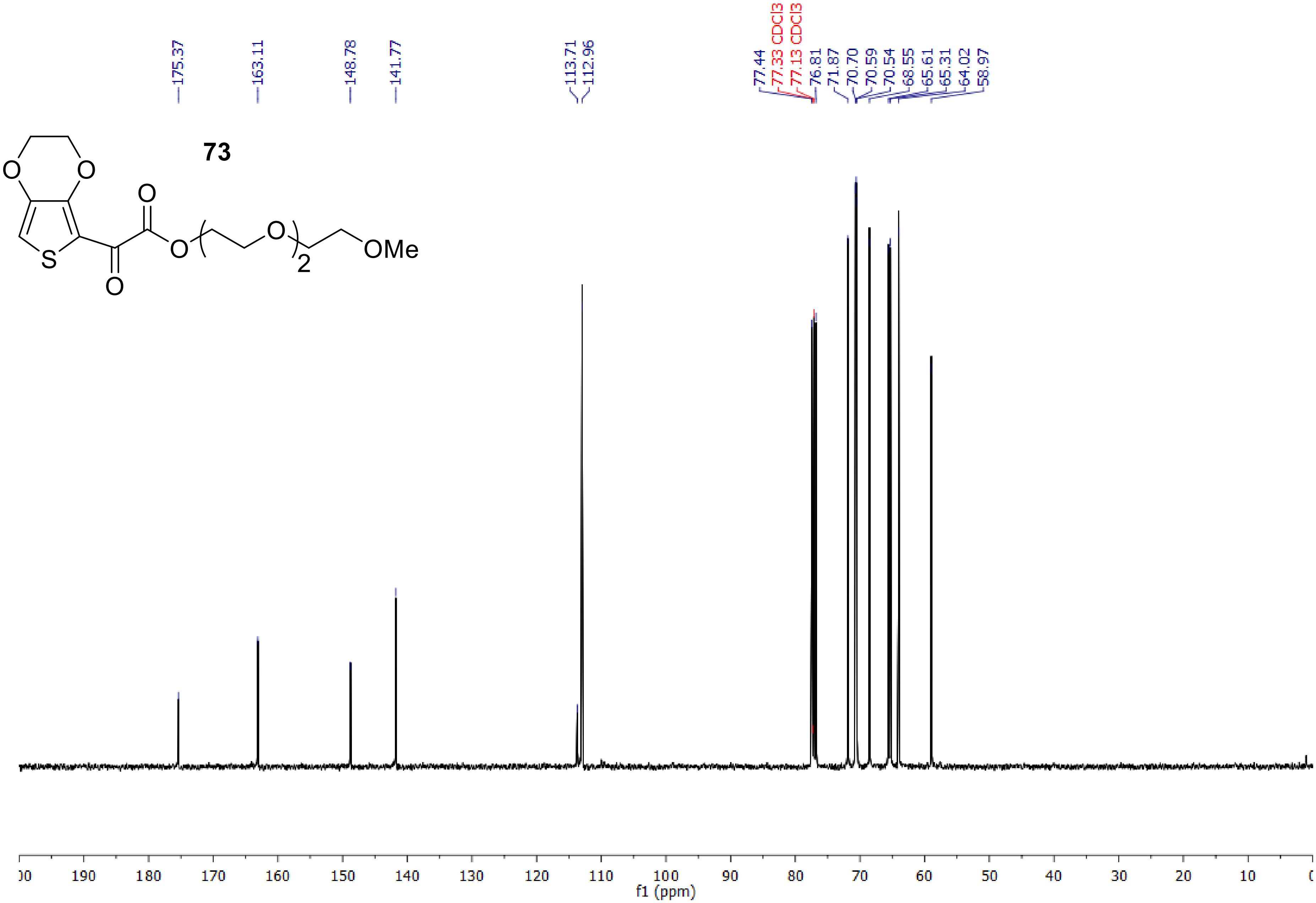

^{1}H NMR (400 MHz, $CDCl_3$)

Figure S105. ^{1}H NMR of **74**

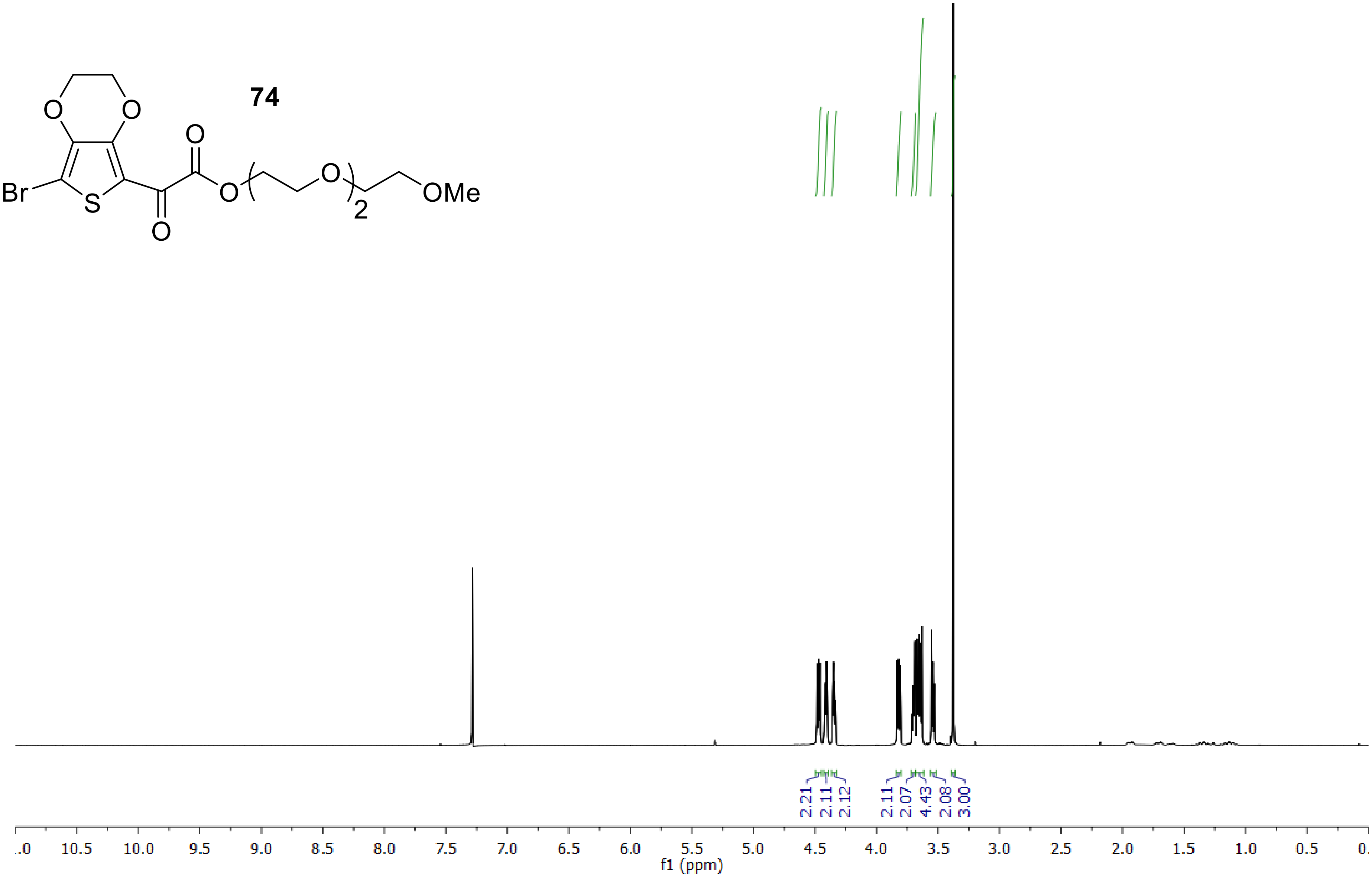

^{13}C NMR (100 MHz, $CDCl_3$)

Figure S106. ^{13}C NMR of **74**

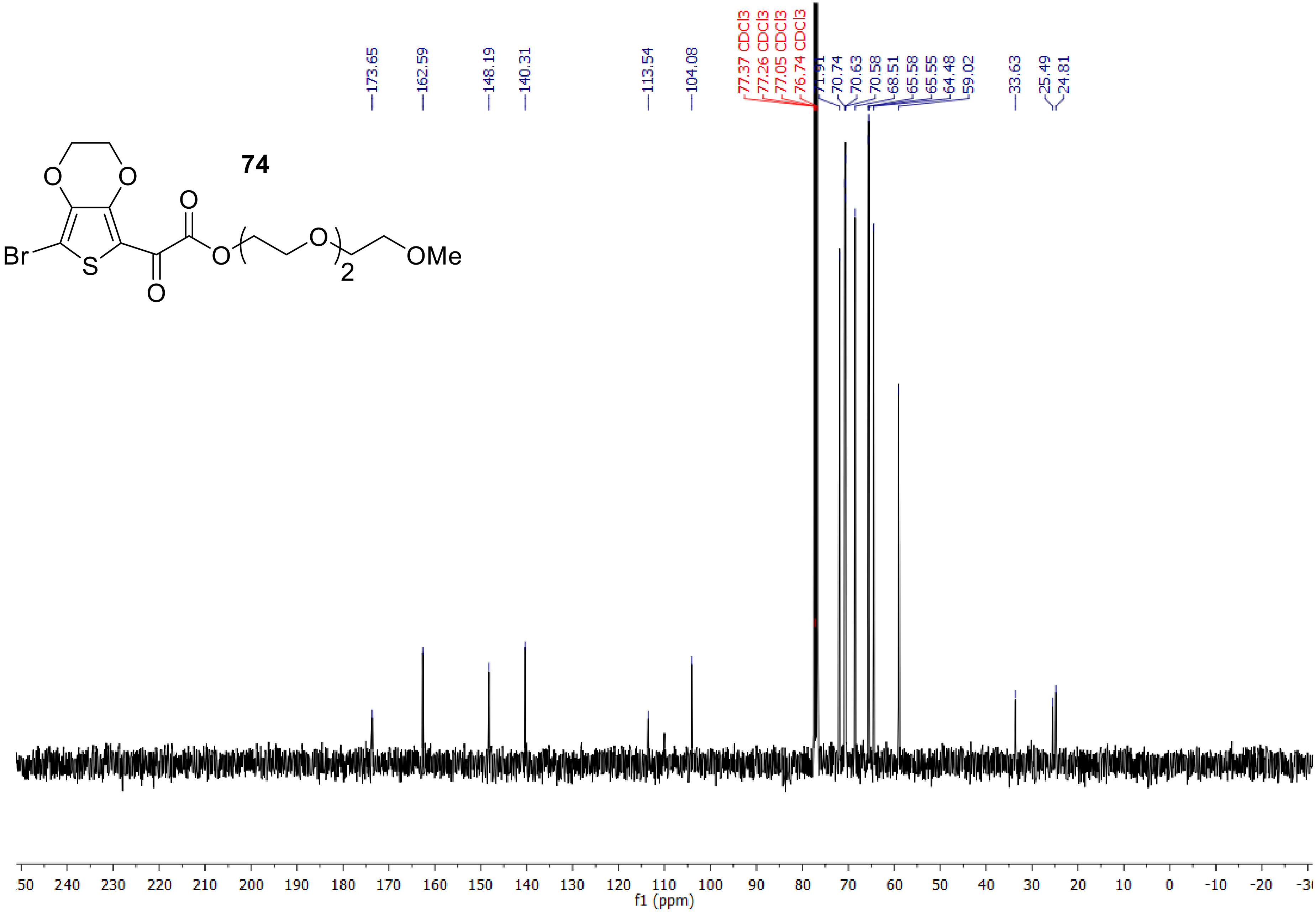

^{1}H NMR (400 MHz, $CDCl_3$)

Figure S107. ^{1}H NMR of **75**

75

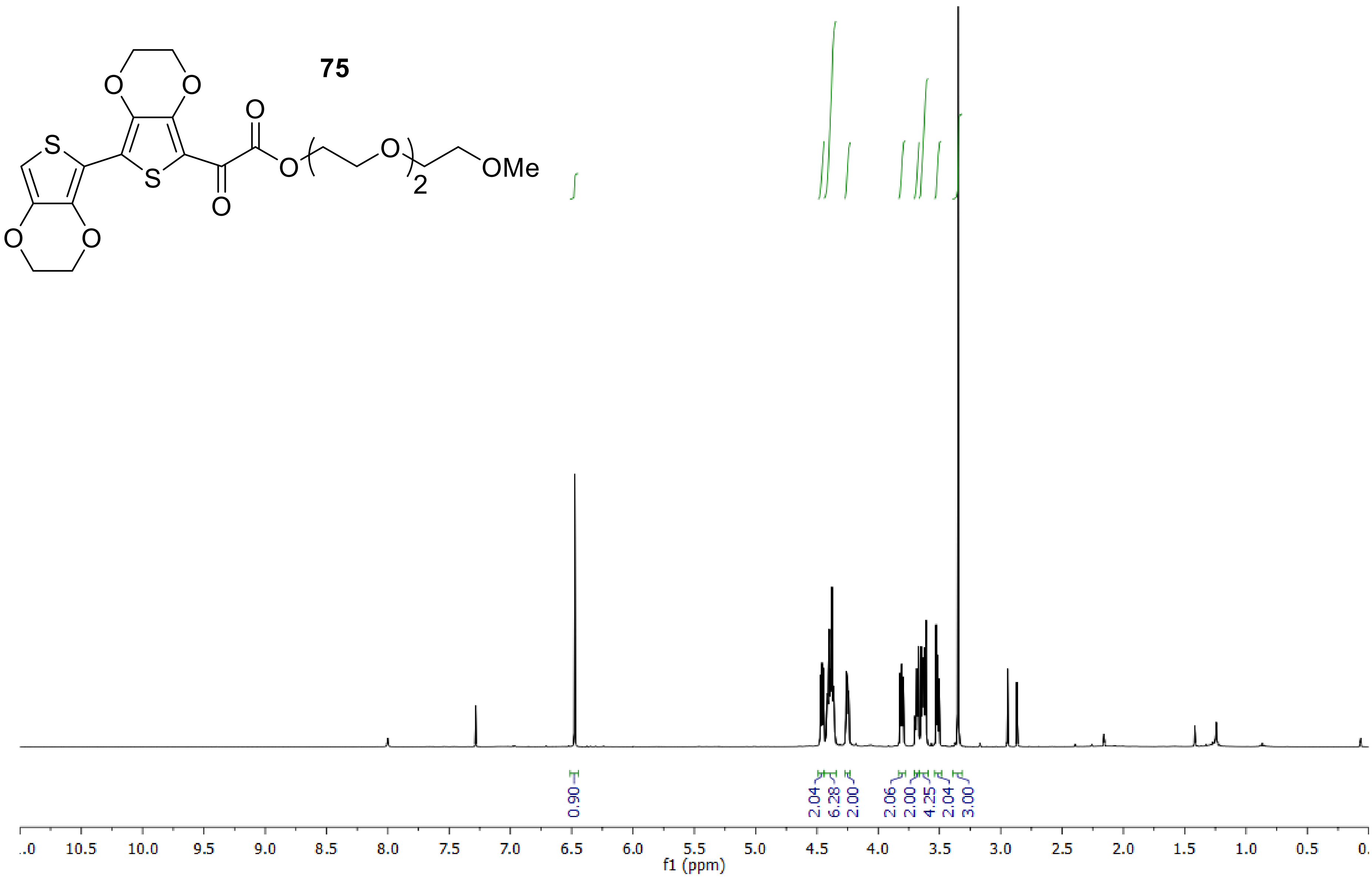

^{13}C NMR (100 MHz, $CDCl_3$)

Figure S108. ^{13}C NMR of **75**

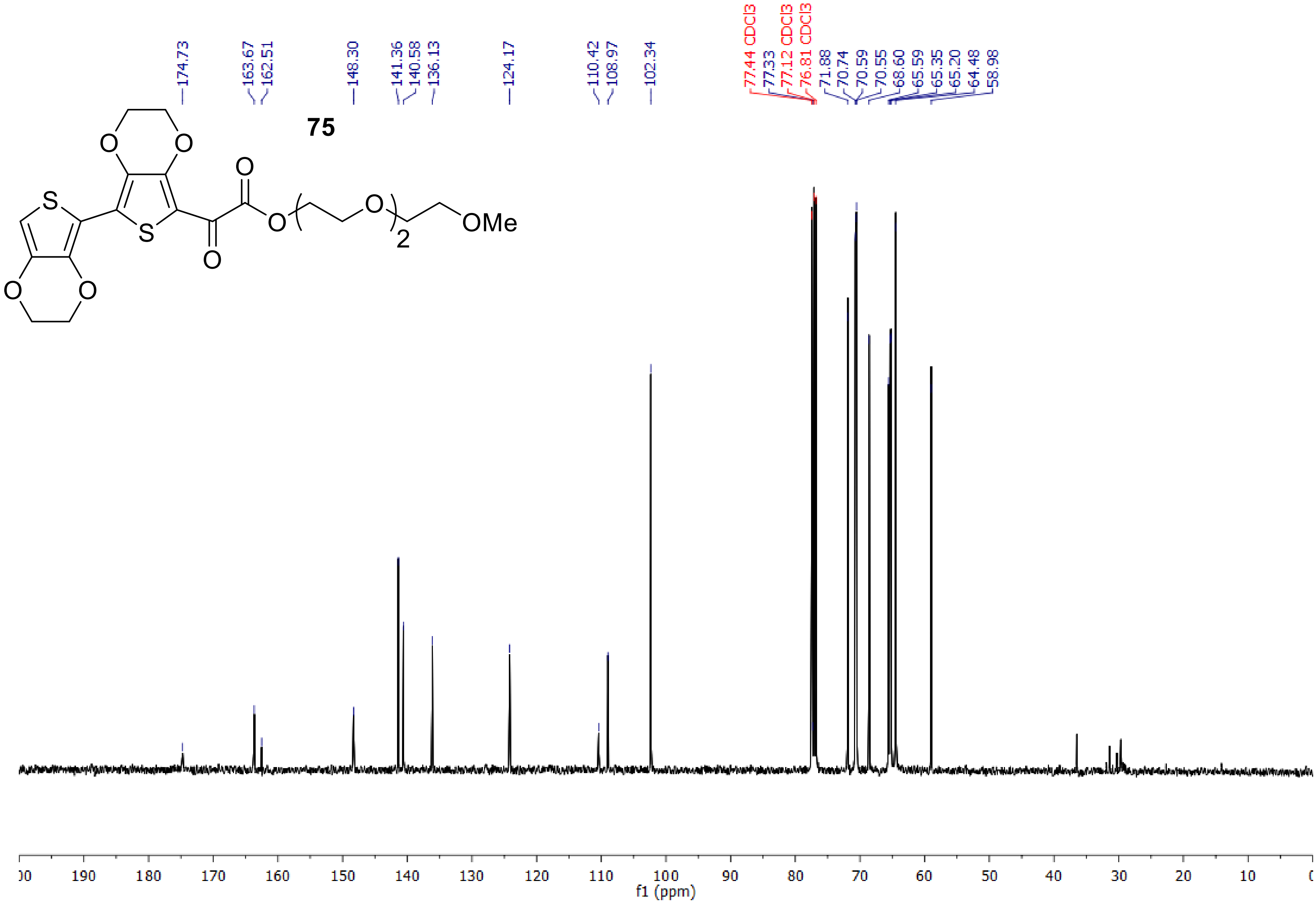

^1H NMR (400 MHz, $CDCl_3$)

Figure S109. ^1H NMR of **76**

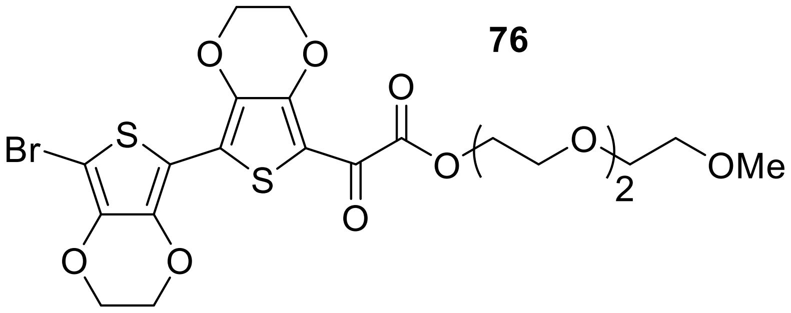

76

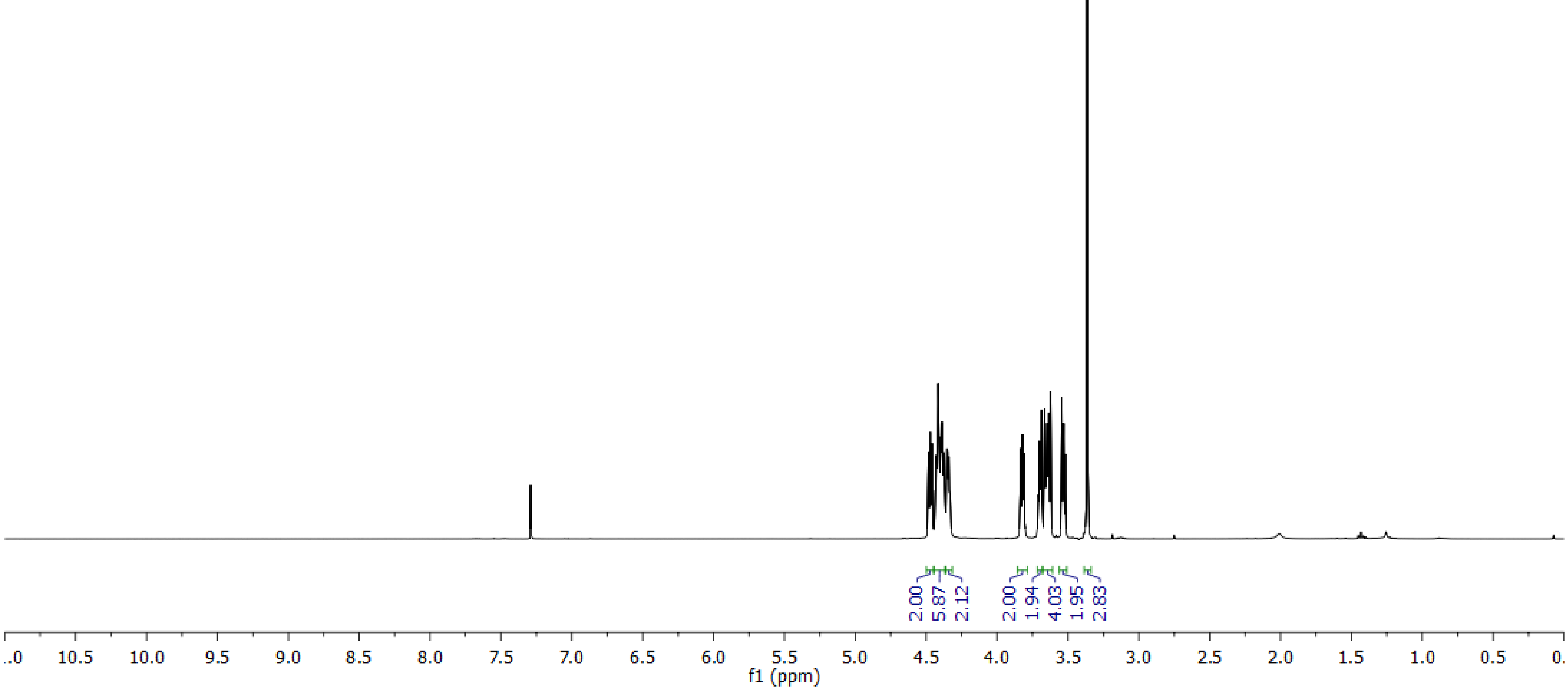

^{13}C NMR (100 MHz, $CDCl_3$)

Figure S110. ^{13}C NMR of **76**

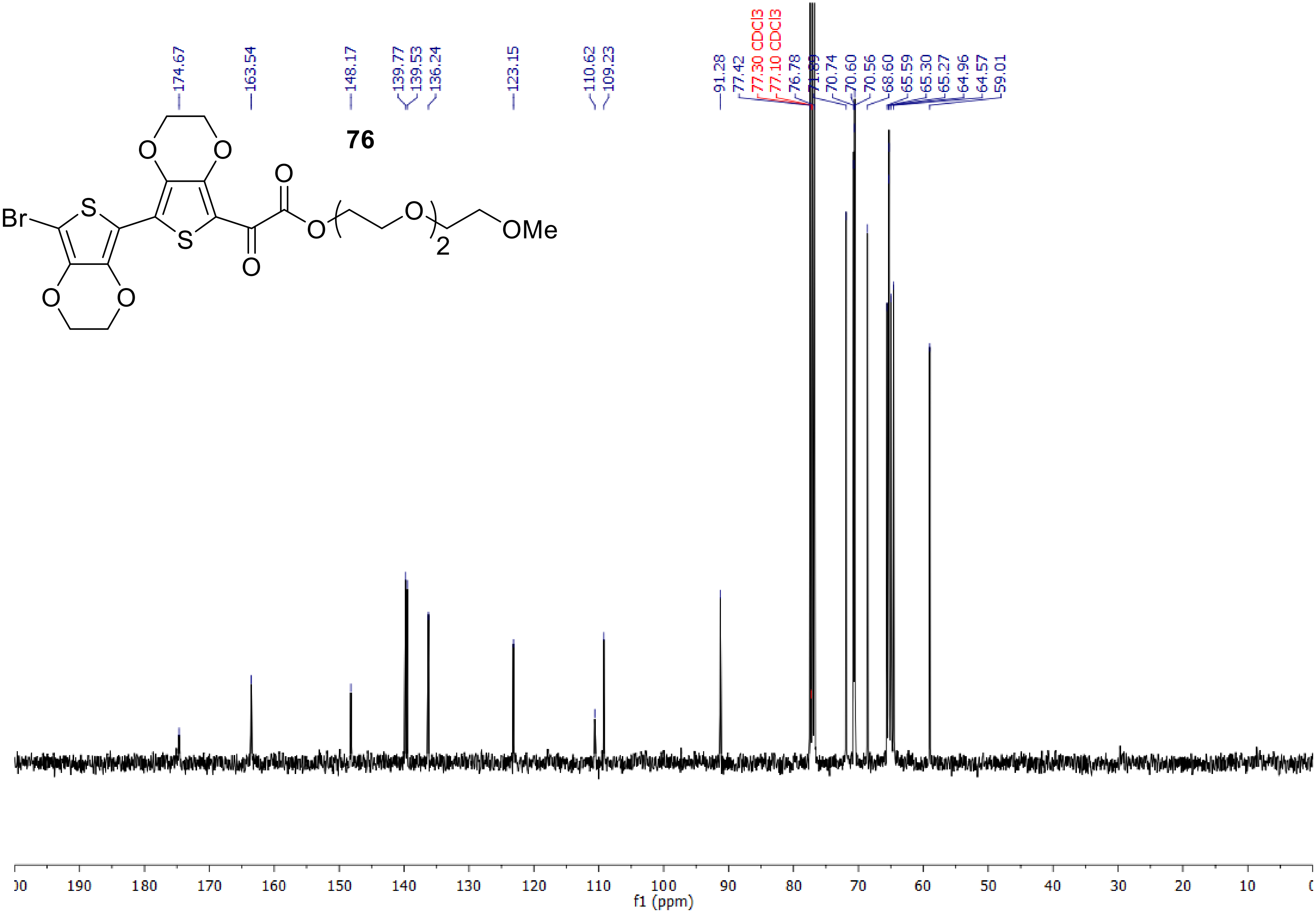

^1H NMR (400 MHz, DMSO-d_6)

Figure S111. ^1H NMR of **77**

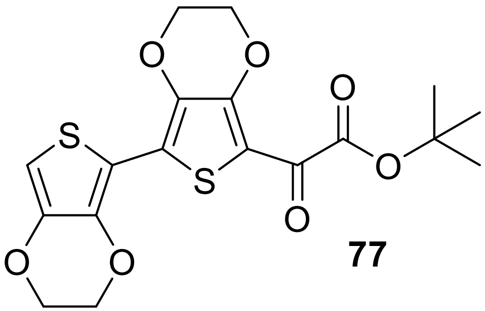

77

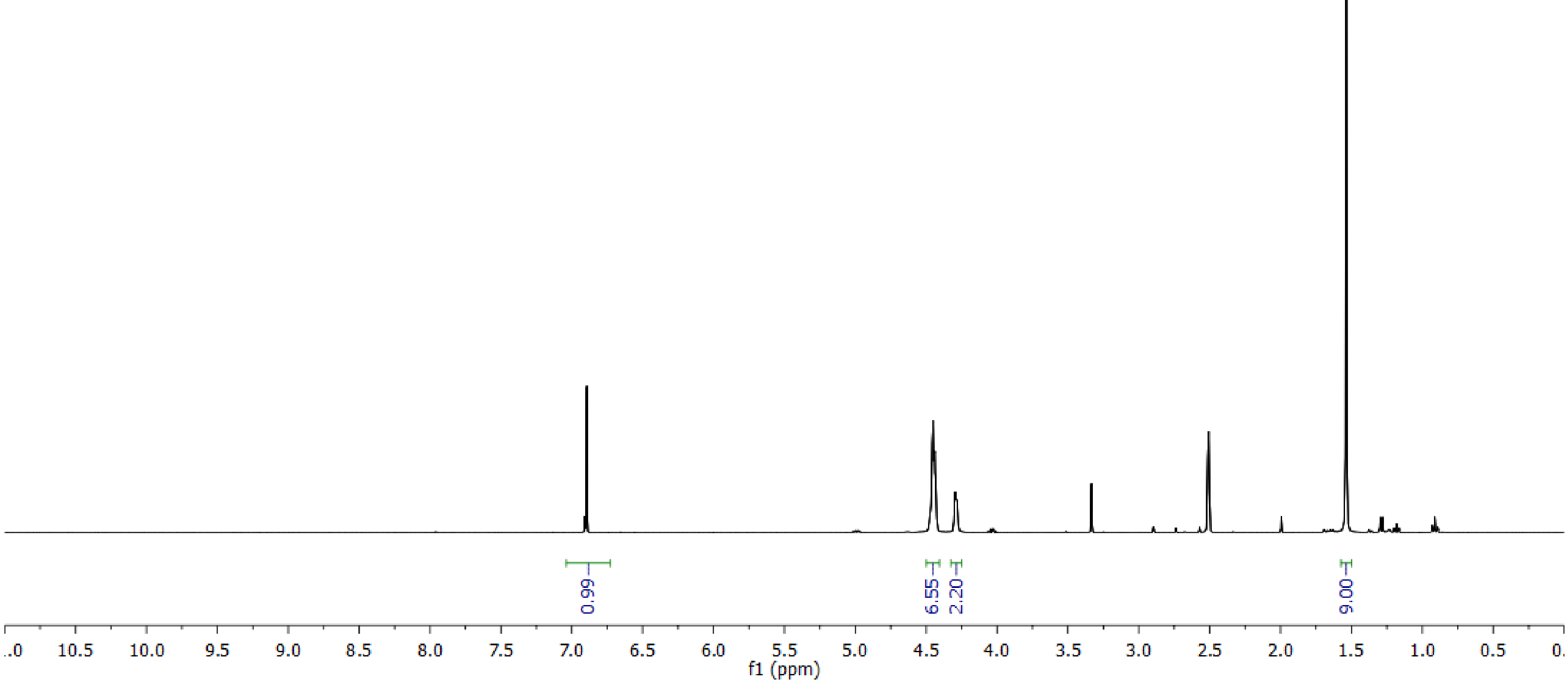

Figure S112. ^{13}C NMR of **77**

^{13}C NMR (100 MHz, DMSO-d_6)

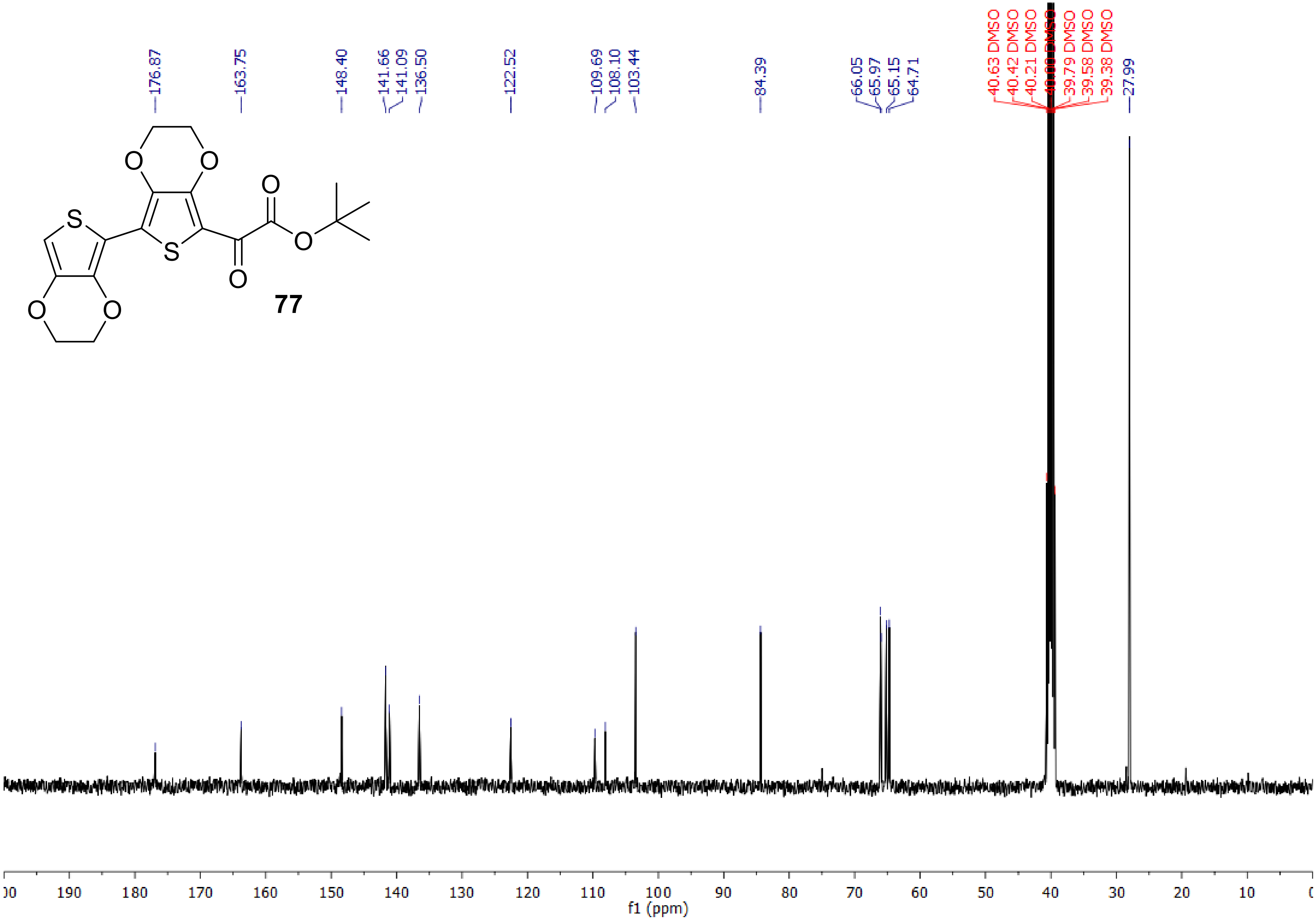

^{1}H NMR (400 MHz, $CDCl_3$)

Figure S113. ^{1}H NMR of **78**

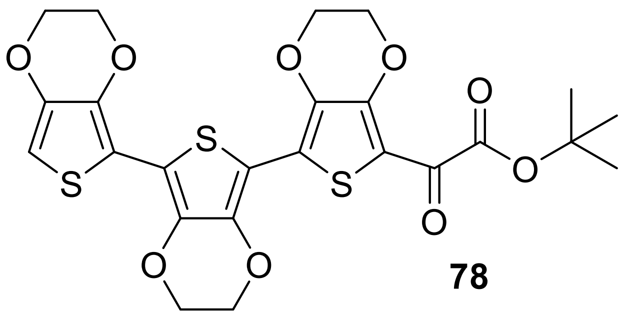

78

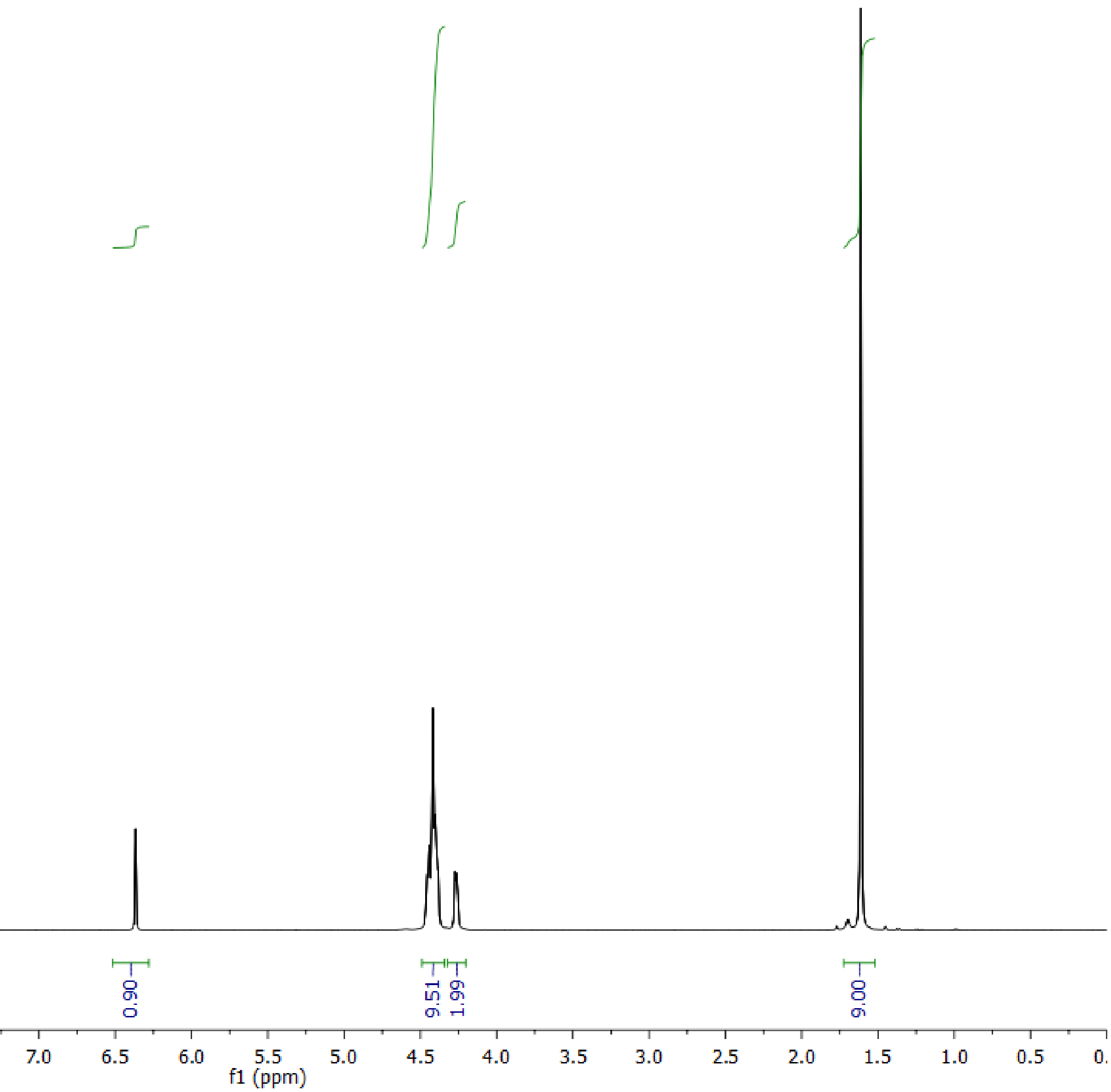

^{13}C NMR (100 MHz, $CDCl_3$)

Figure S114. ^{13}C NMR of **78**

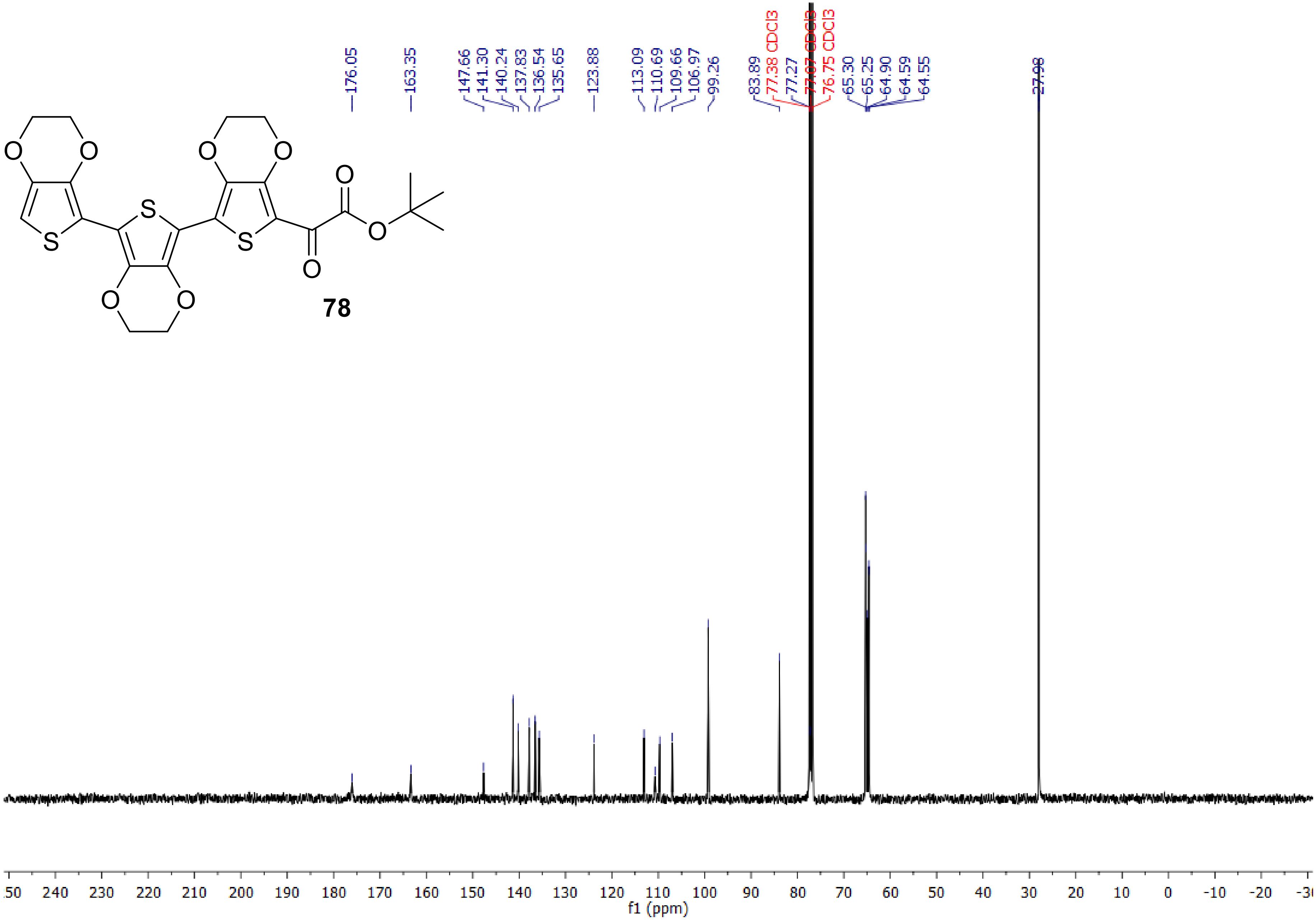

^{1}H NMR (400 MHz, $CDCl_3$)

Figure S115. ^{1}H NMR of **80**

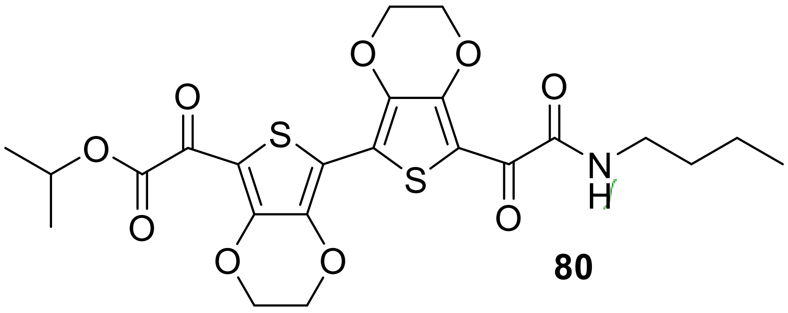

80

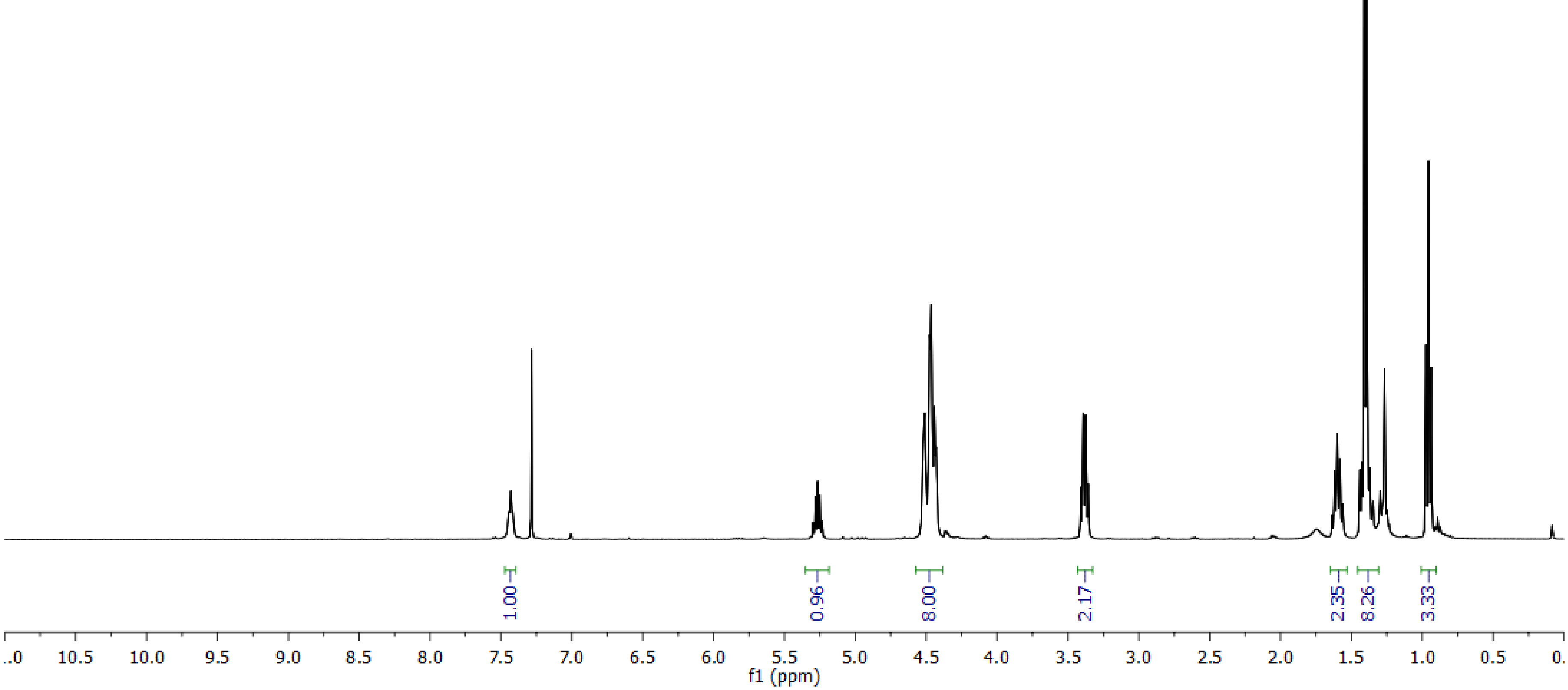

^{13}C NMR (100 MHz, $CDCl_3$)

Figure S116. ^{13}C NMR of **80**

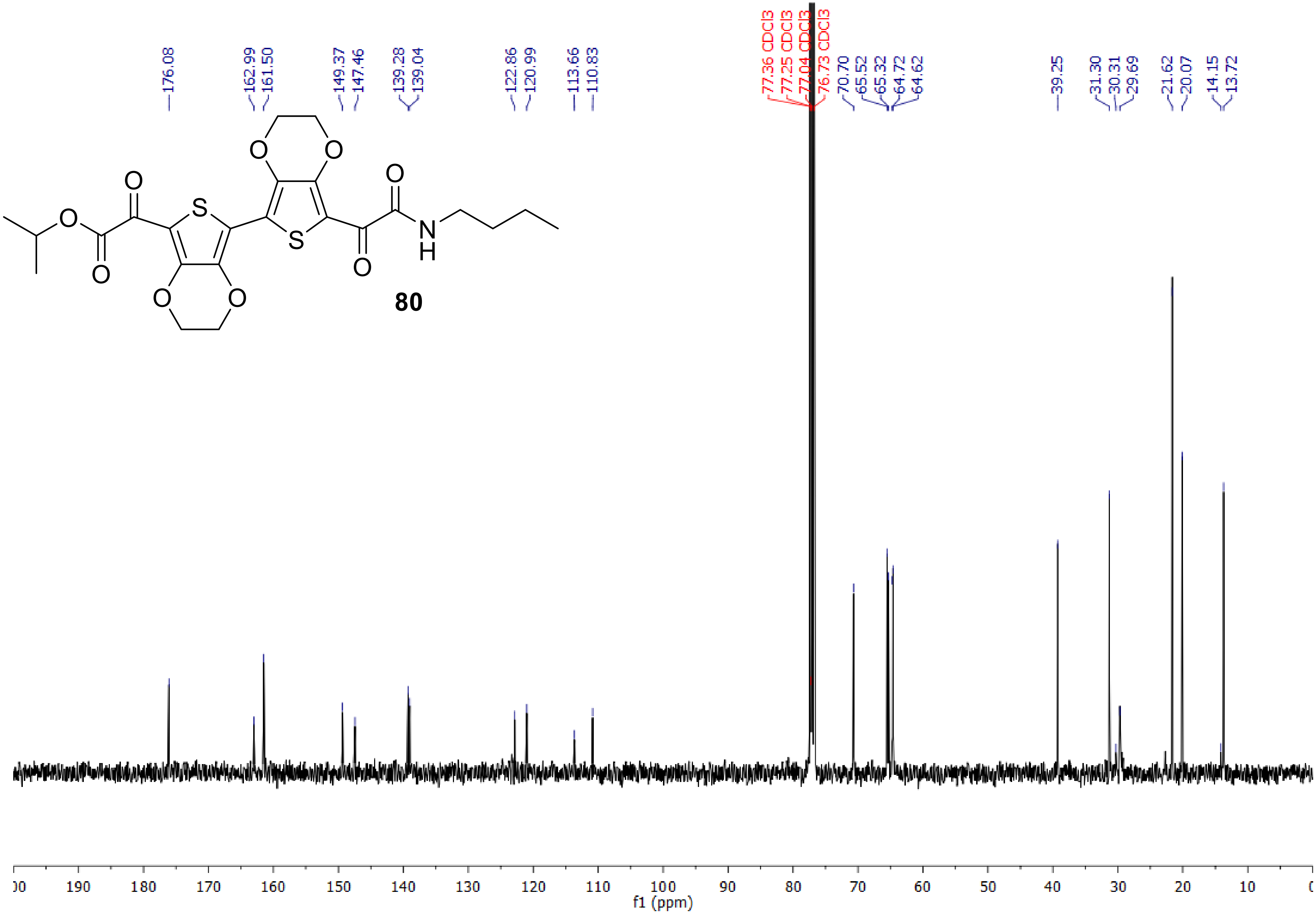

^{1}H NMR (400 MHz, $CDCl_3$)

Figure S117. ^{1}H NMR of **84**

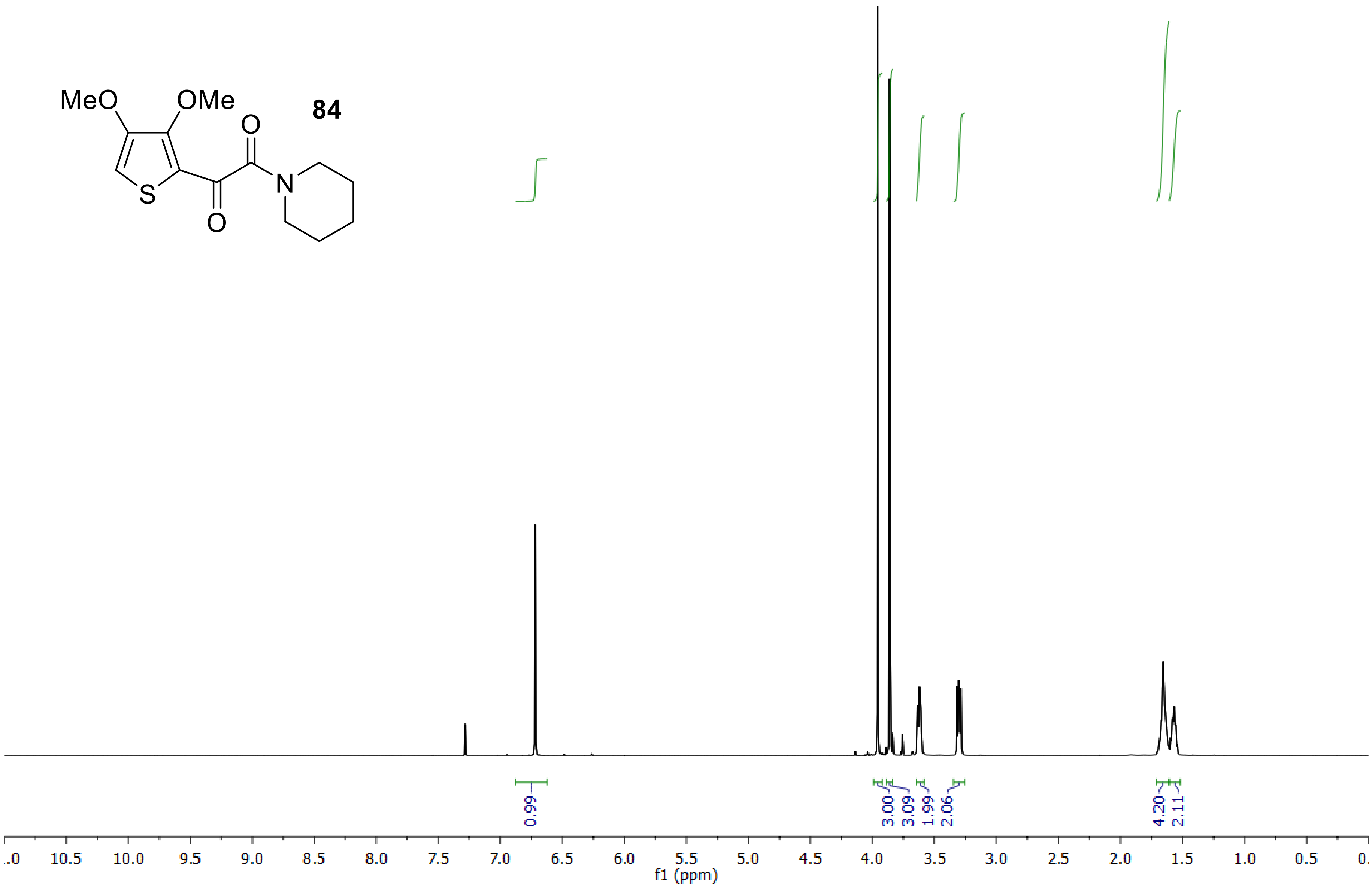

^{13}C NMR (100 MHz, $CDCl_3$)

Figure S118. ^{13}C NMR of **84**

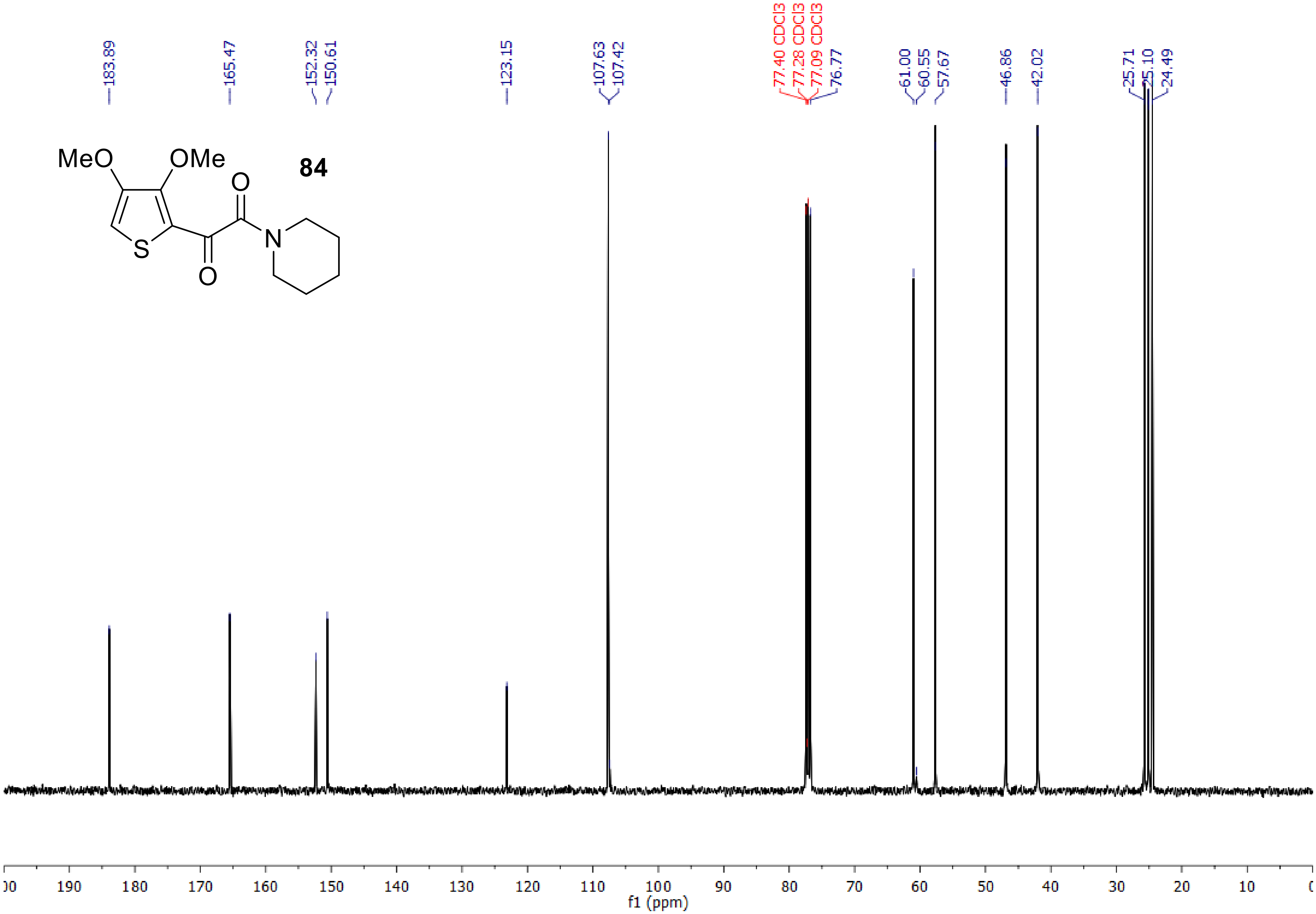

^{1}H NMR (400 MHz, $CDCl_3$)

Figure S119. ^{1}H NMR of **85**

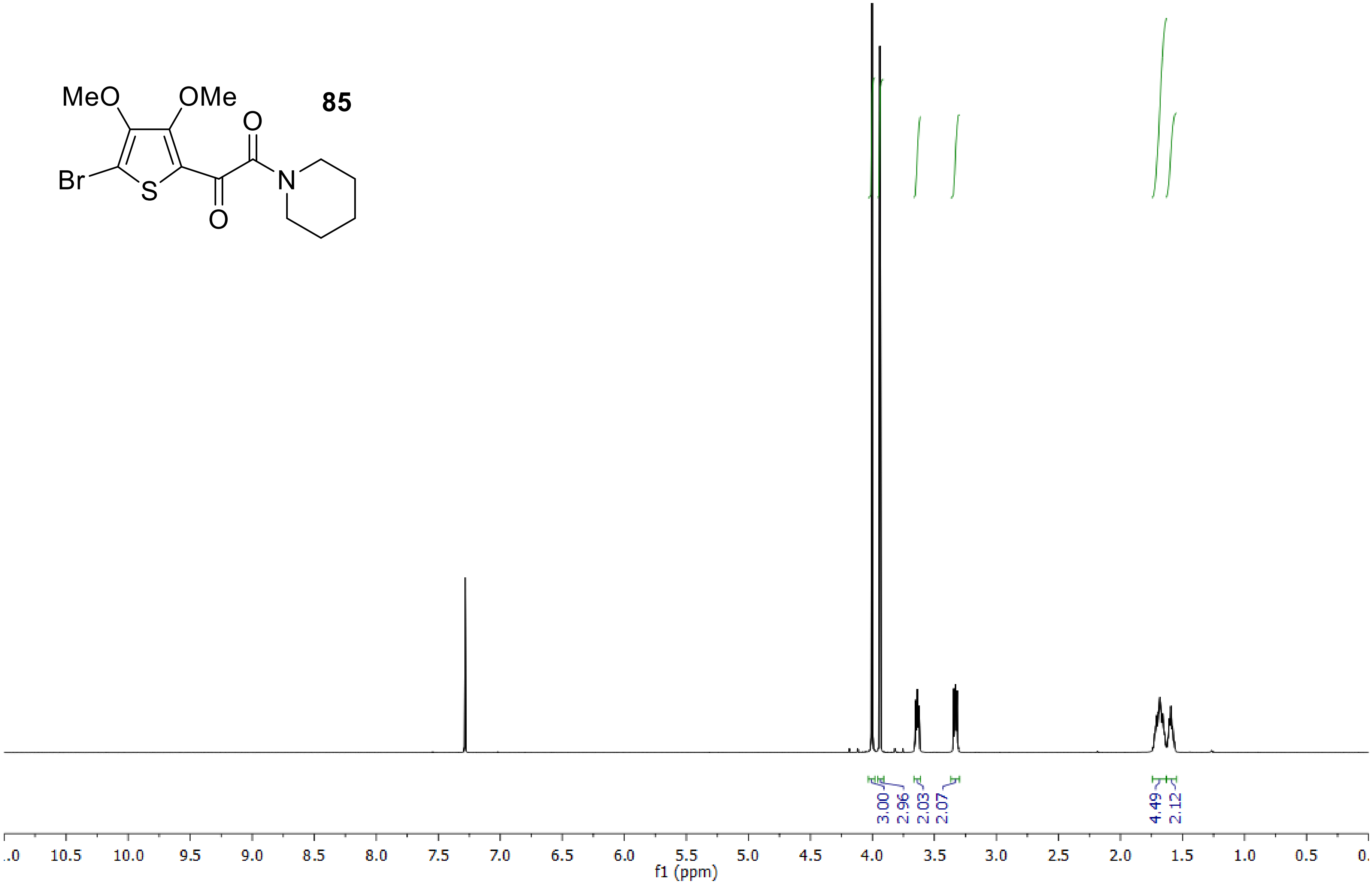

^{13}C NMR (100 MHz, $CDCl_3$)

Figure S120. ^{13}C NMR of **85**

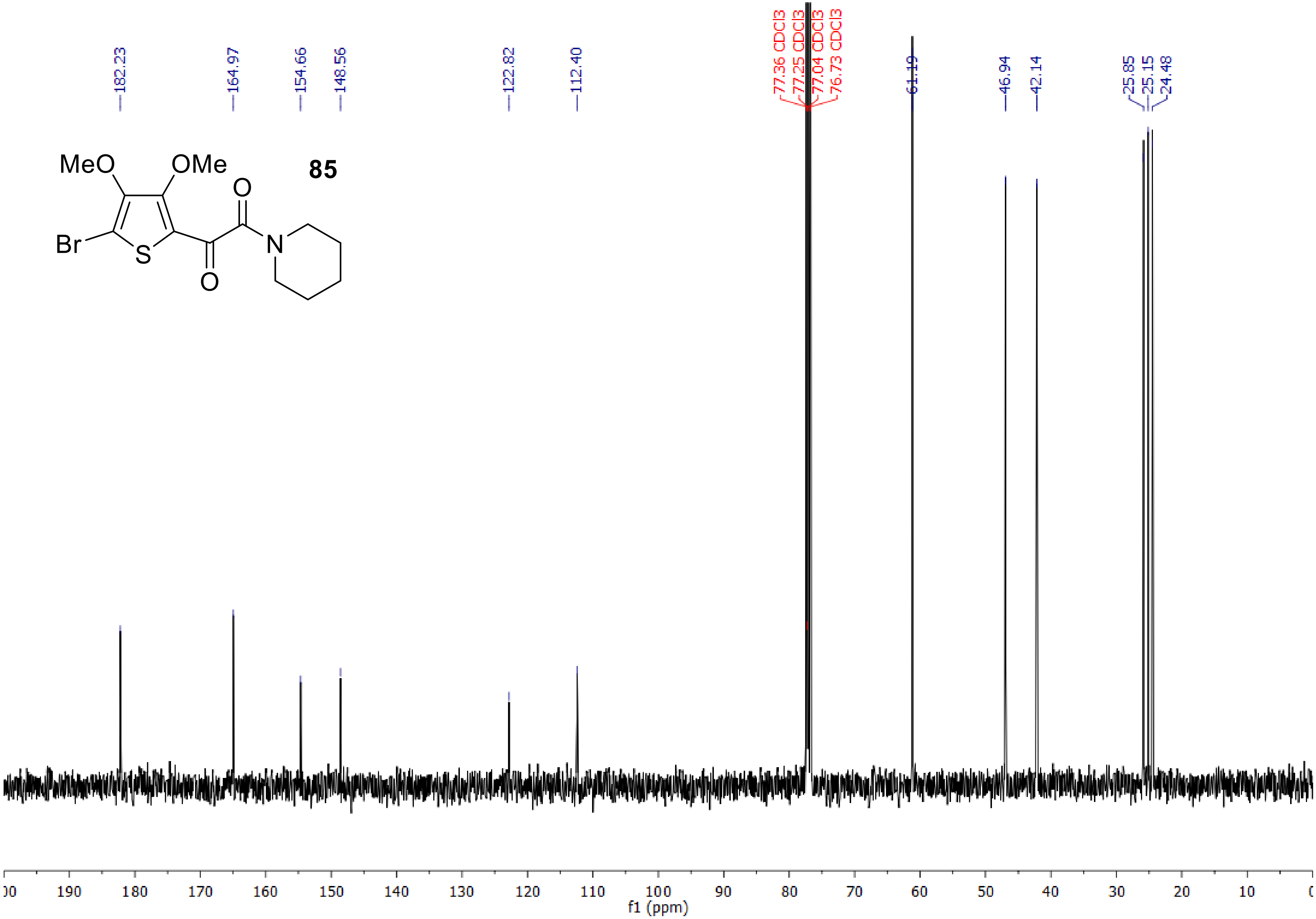

^{1}H NMR (400 MHz, $CDCl_3$)

Figure S121. ^{1}H NMR of **86**

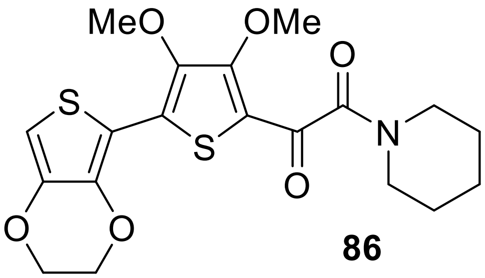

86

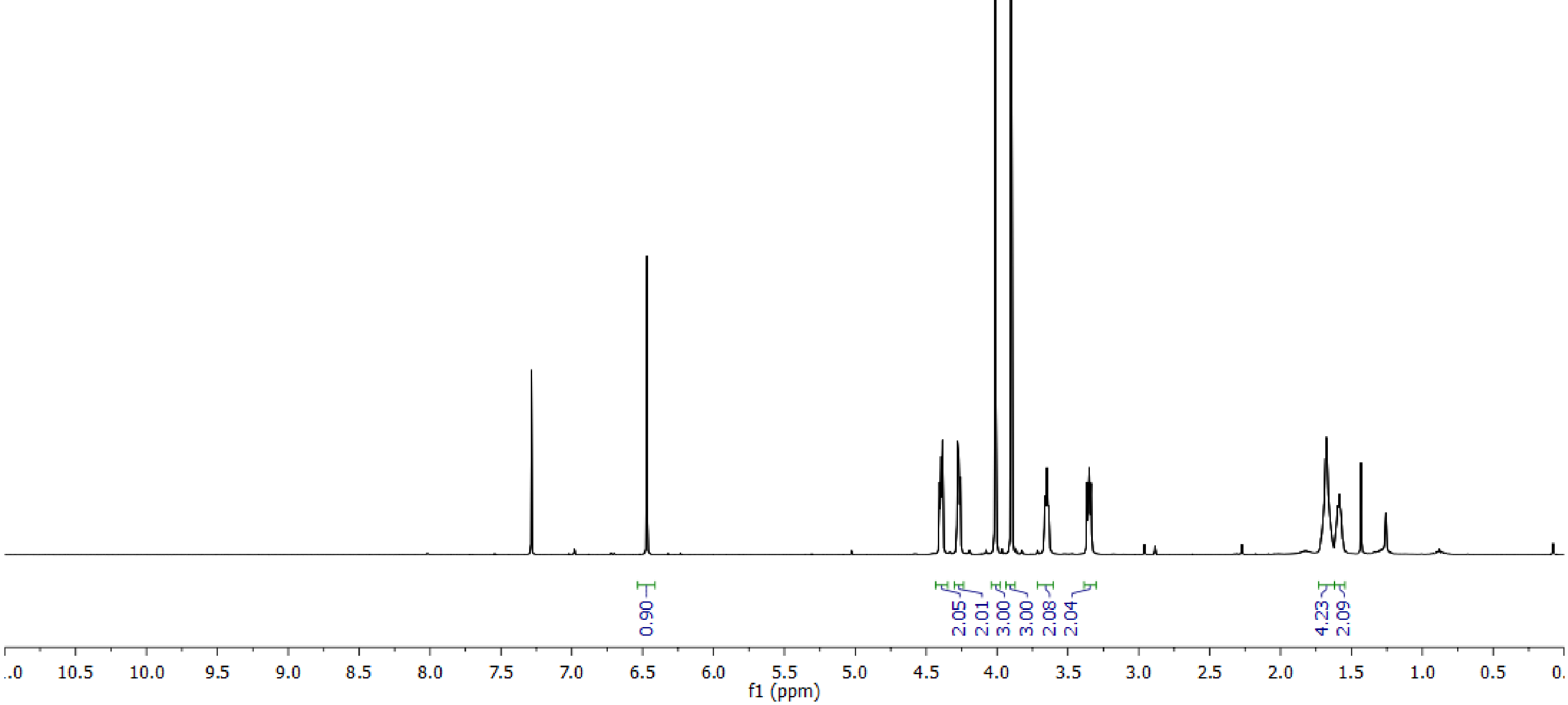

^{13}C NMR (100 MHz, $CDCl_3$)

Figure S122. ^{13}C NMR of **86**

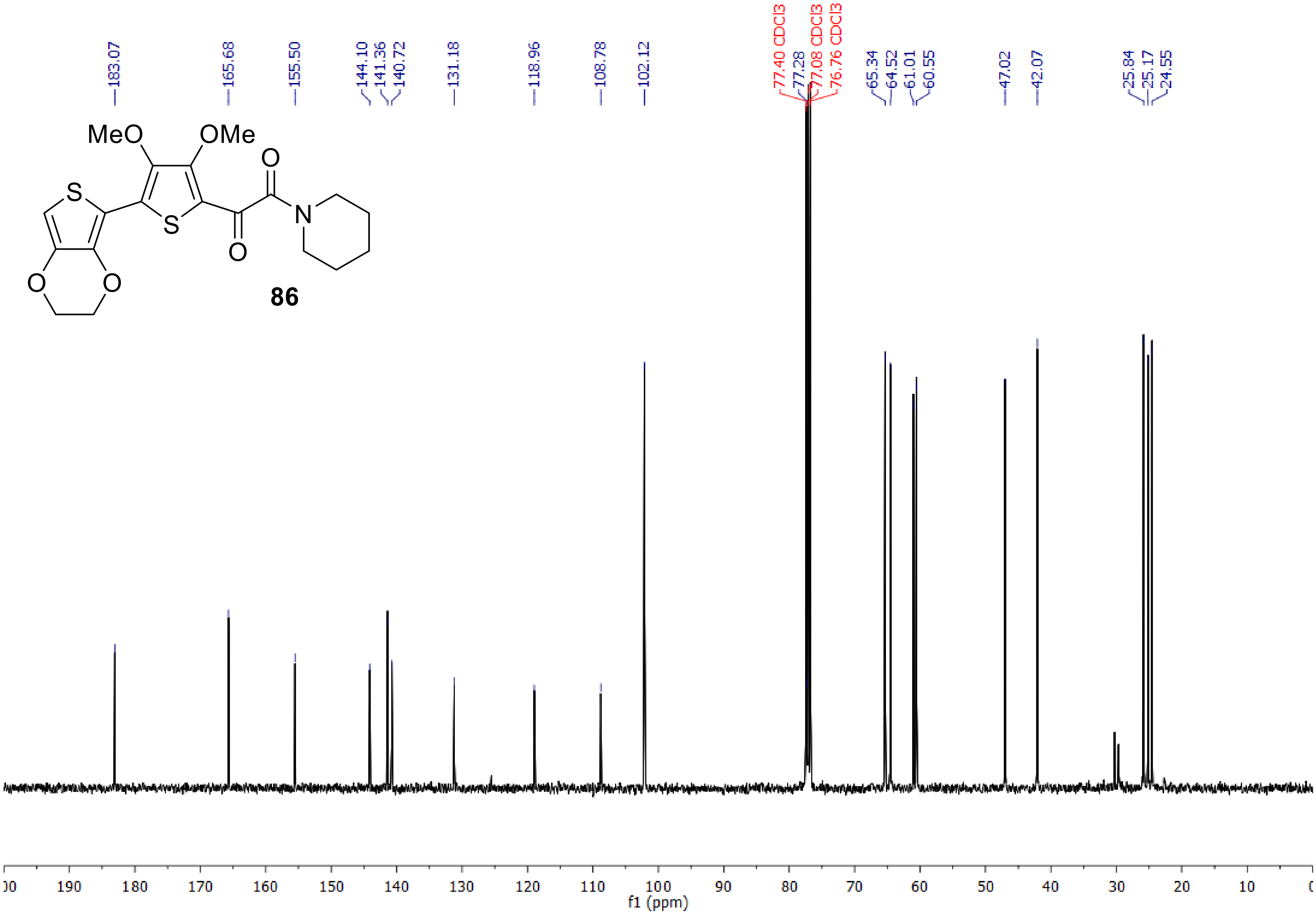

^{1}H NMR (400 MHz, $CDCl_3$)

Figure S123. ^{1}H NMR of **87**

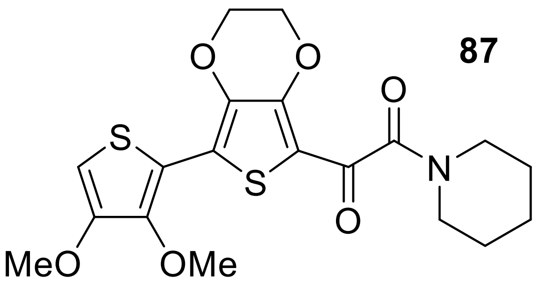

87

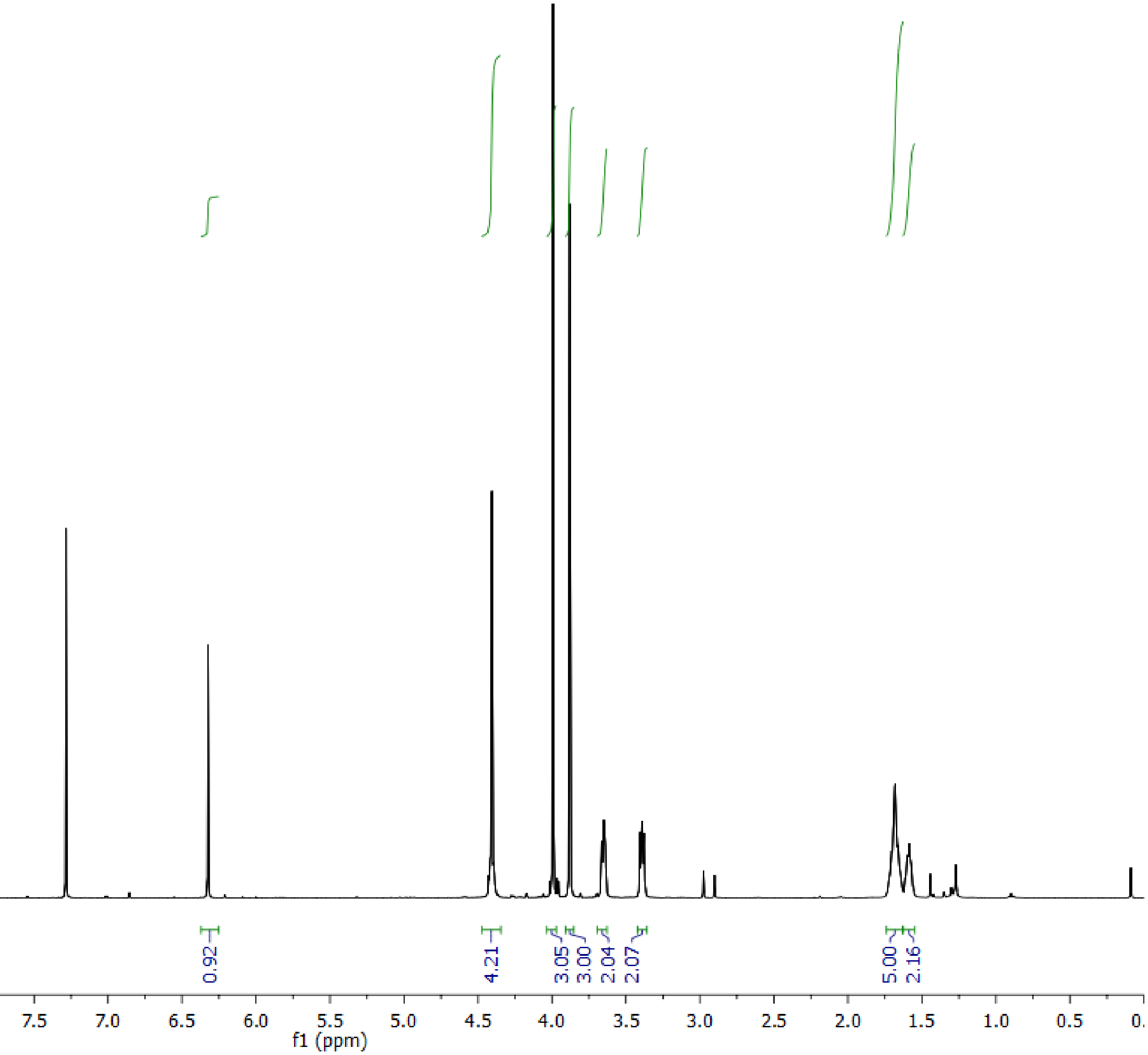

^{13}C NMR (100 MHz, $CDCl_3$)

Figure S124. ^{13}C NMR of **87**

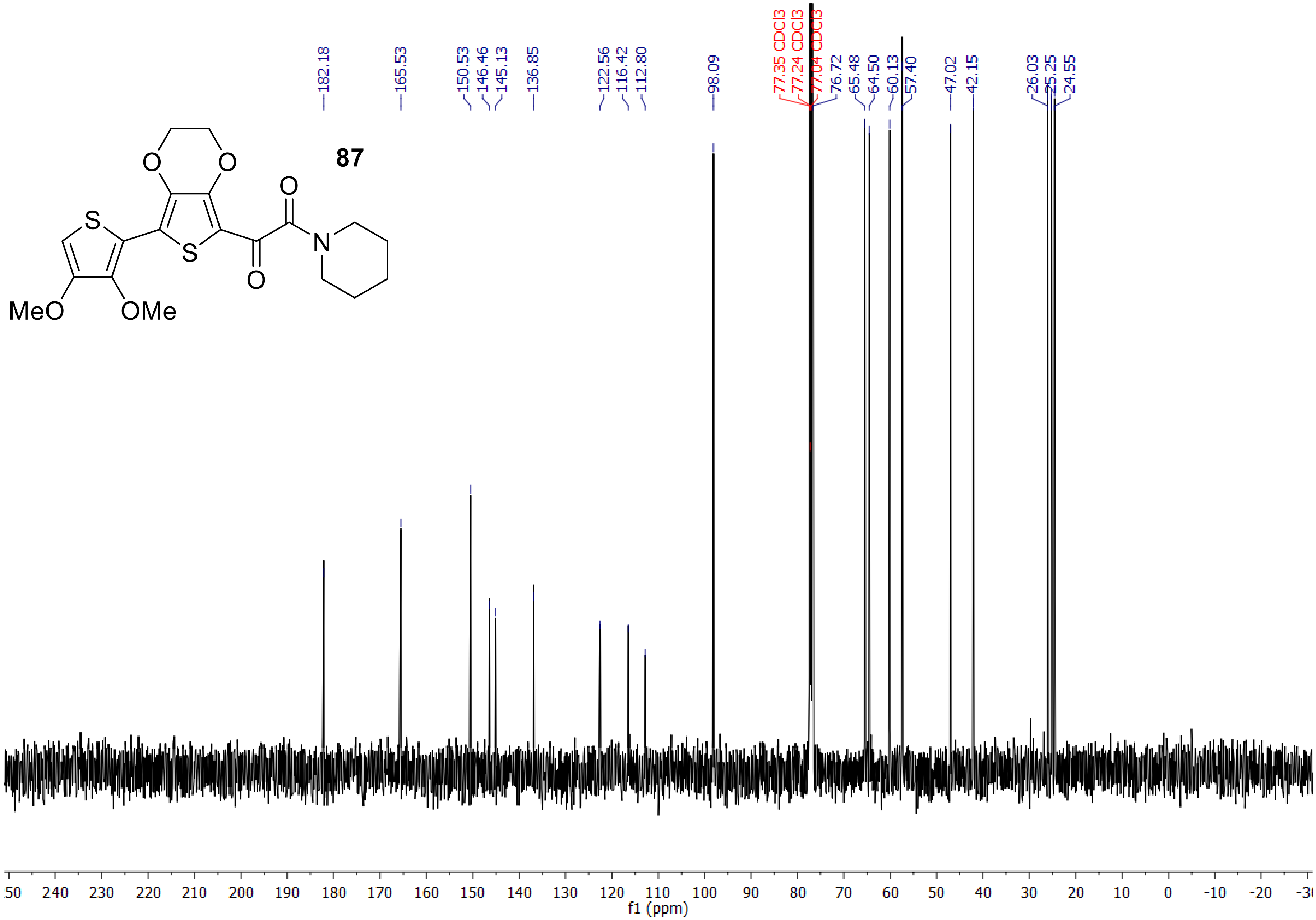

^{1}H NMR (400 MHz, $CDCl_3$)

Figure S125. ^{1}H NMR of **90**

90

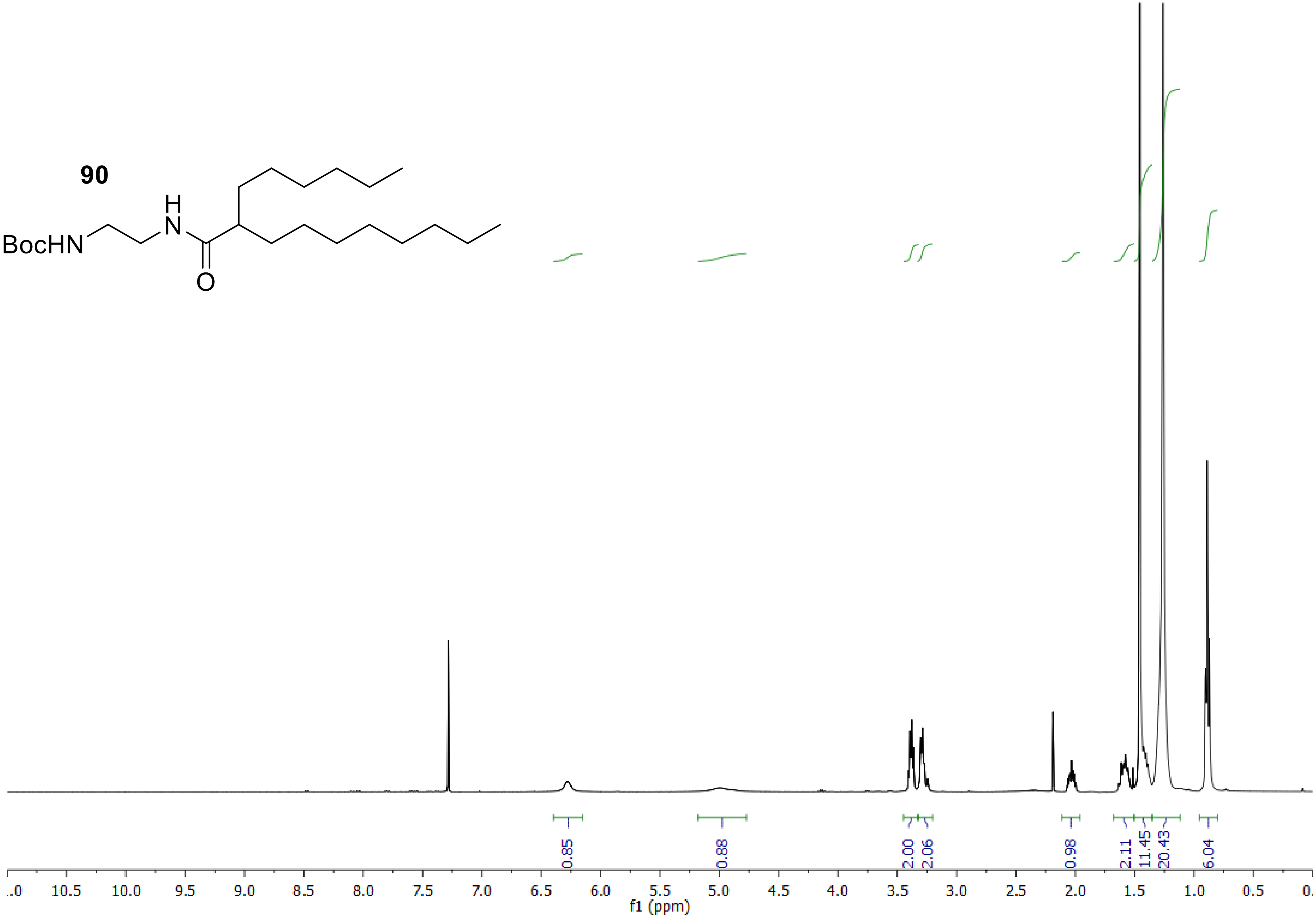

^{13}C NMR (100 MHz, $CDCl_3$)

Figure S126. ^{13}C NMR of **90**

90

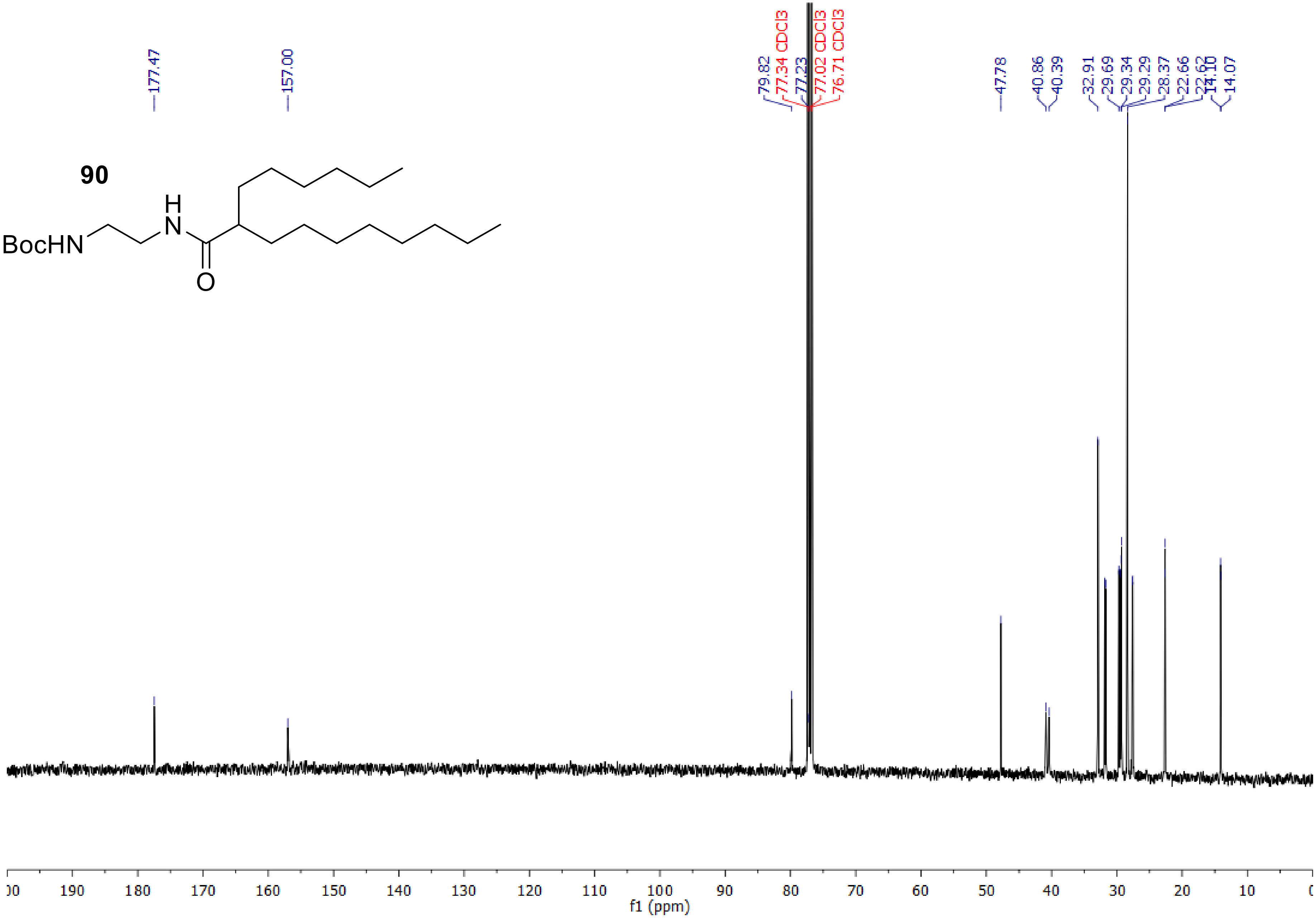

^{1}H NMR (400 MHz, $CDCl_3$)

Figure S127. ^{1}H NMR of **91**

91

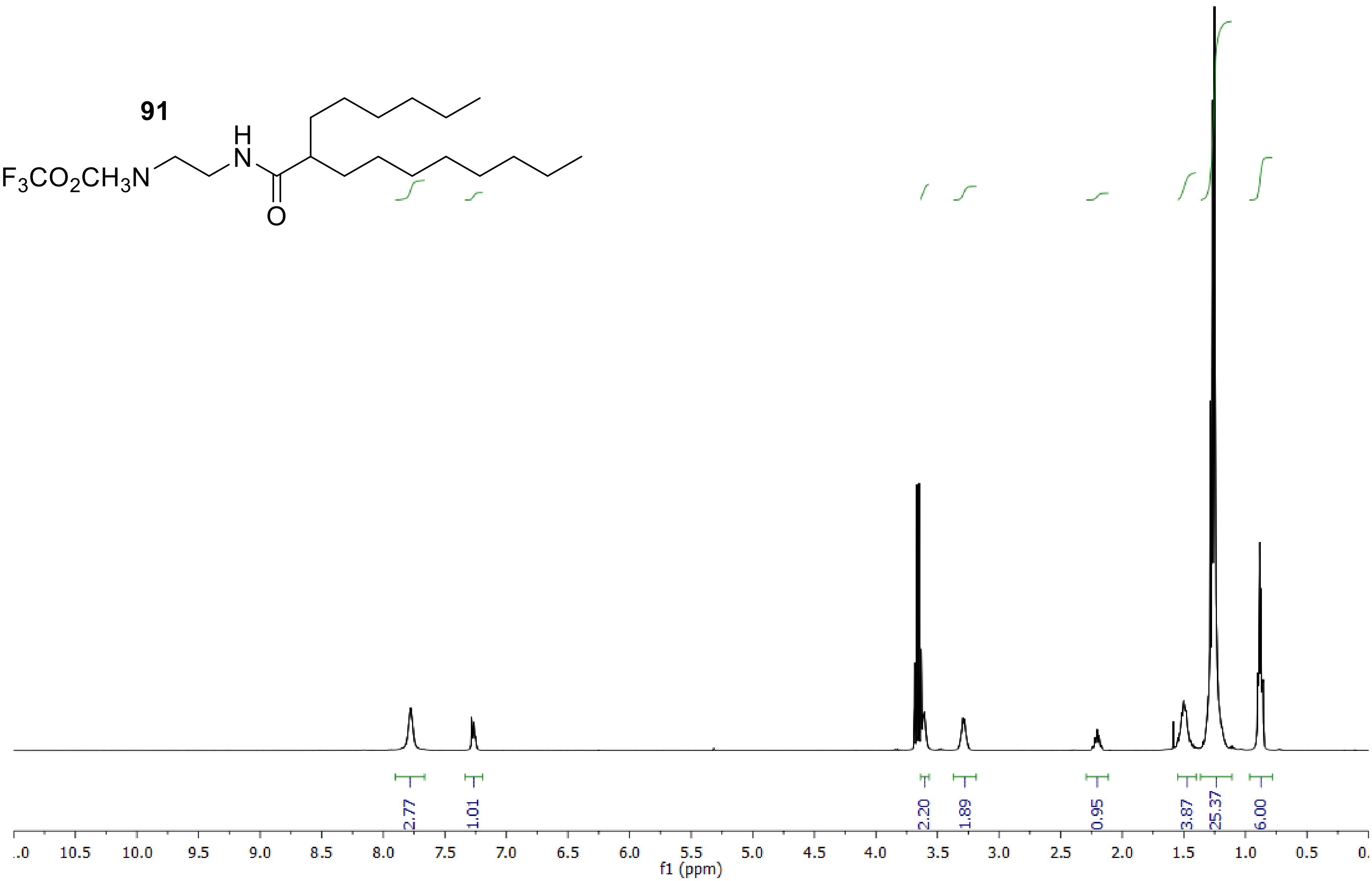

^{13}C NMR (100 MHz, $CDCl_3$)

Figure S128. ^{13}C NMR of **91**

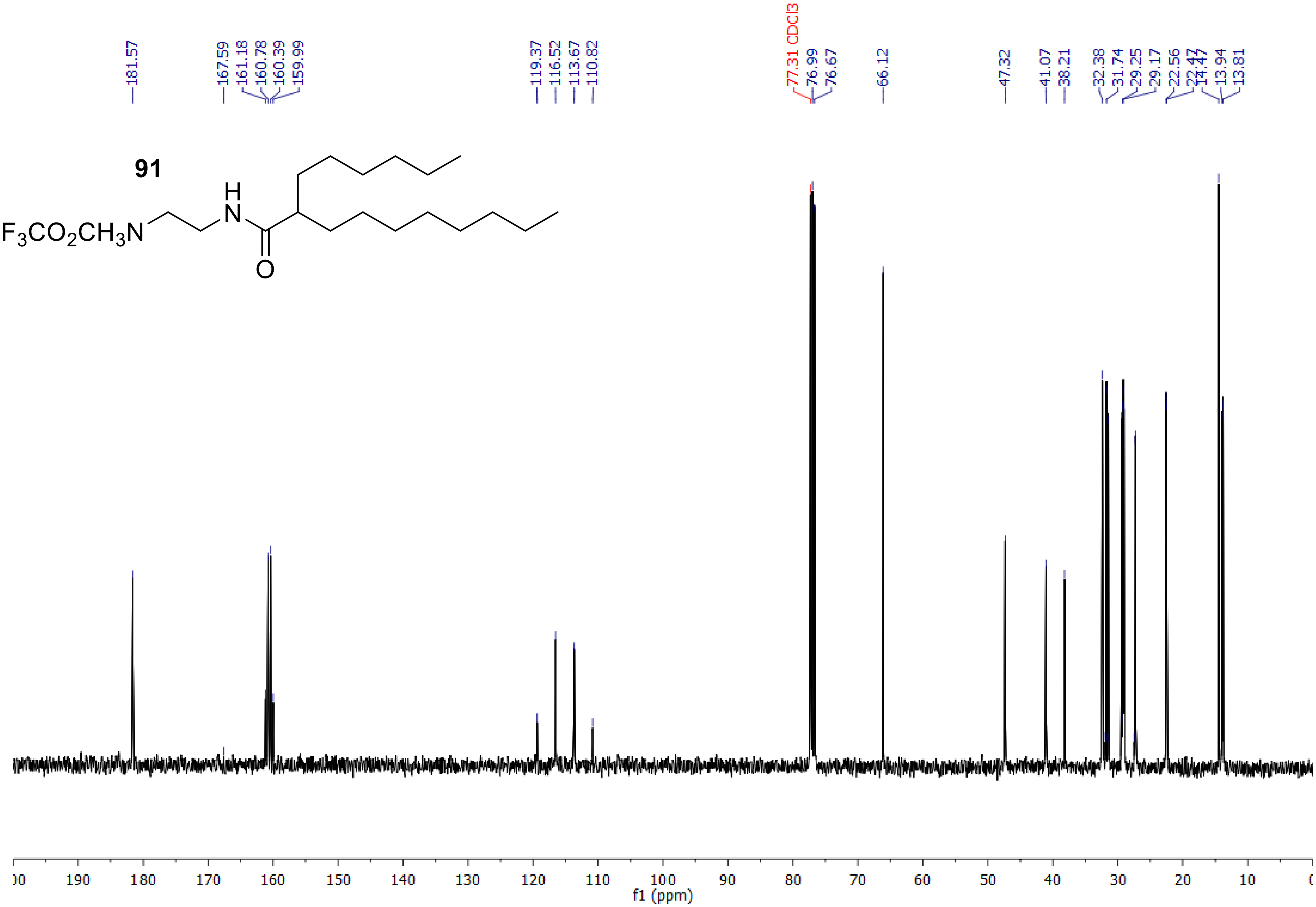

^{1}H NMR (400 MHz, $CDCl_3$)

Figure S129. ^{1}H NMR of **98**

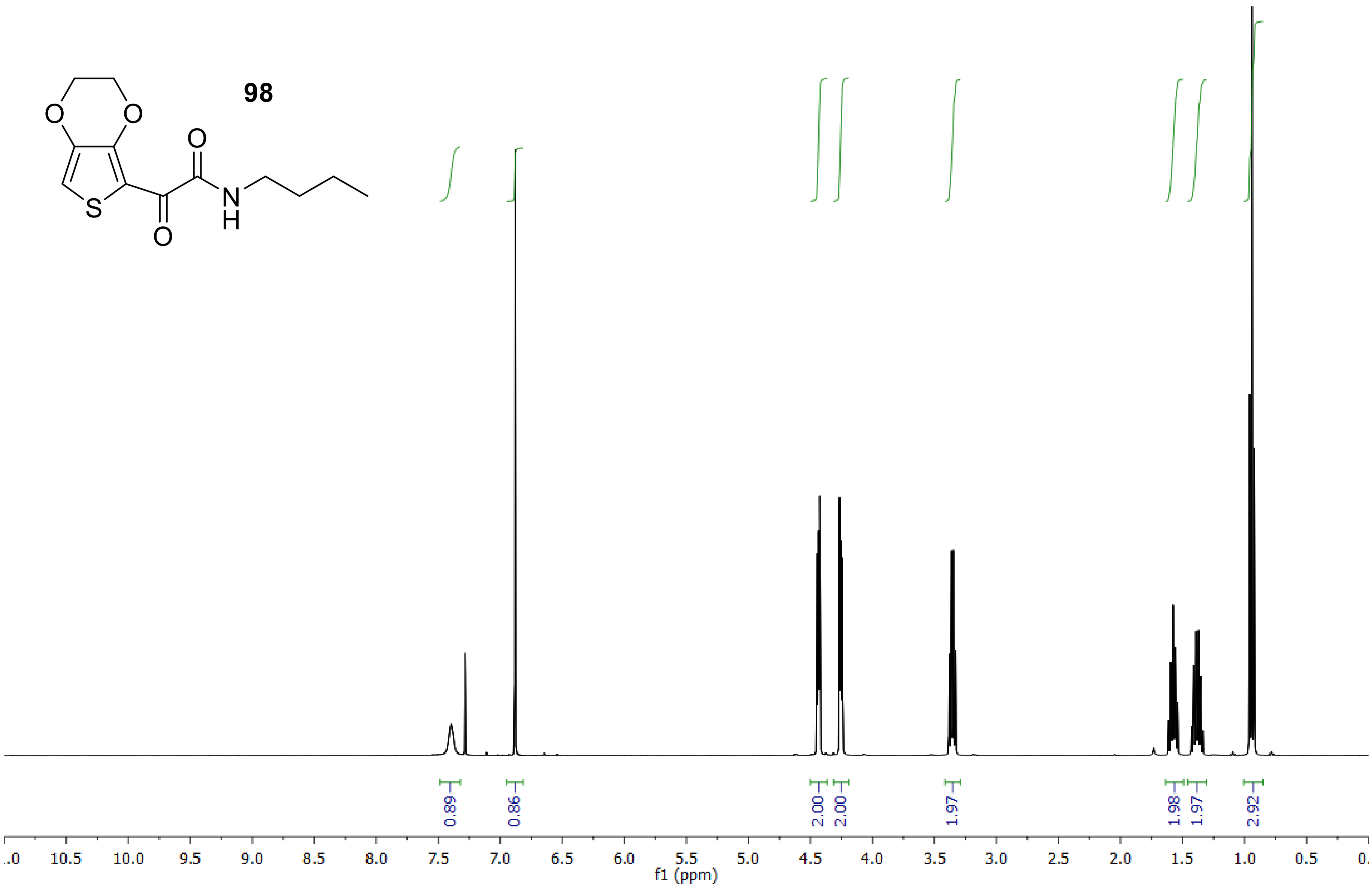

^{13}C NMR (100 MHz, $CDCl_3$)

Figure S130. ^{13}C NMR of **98**

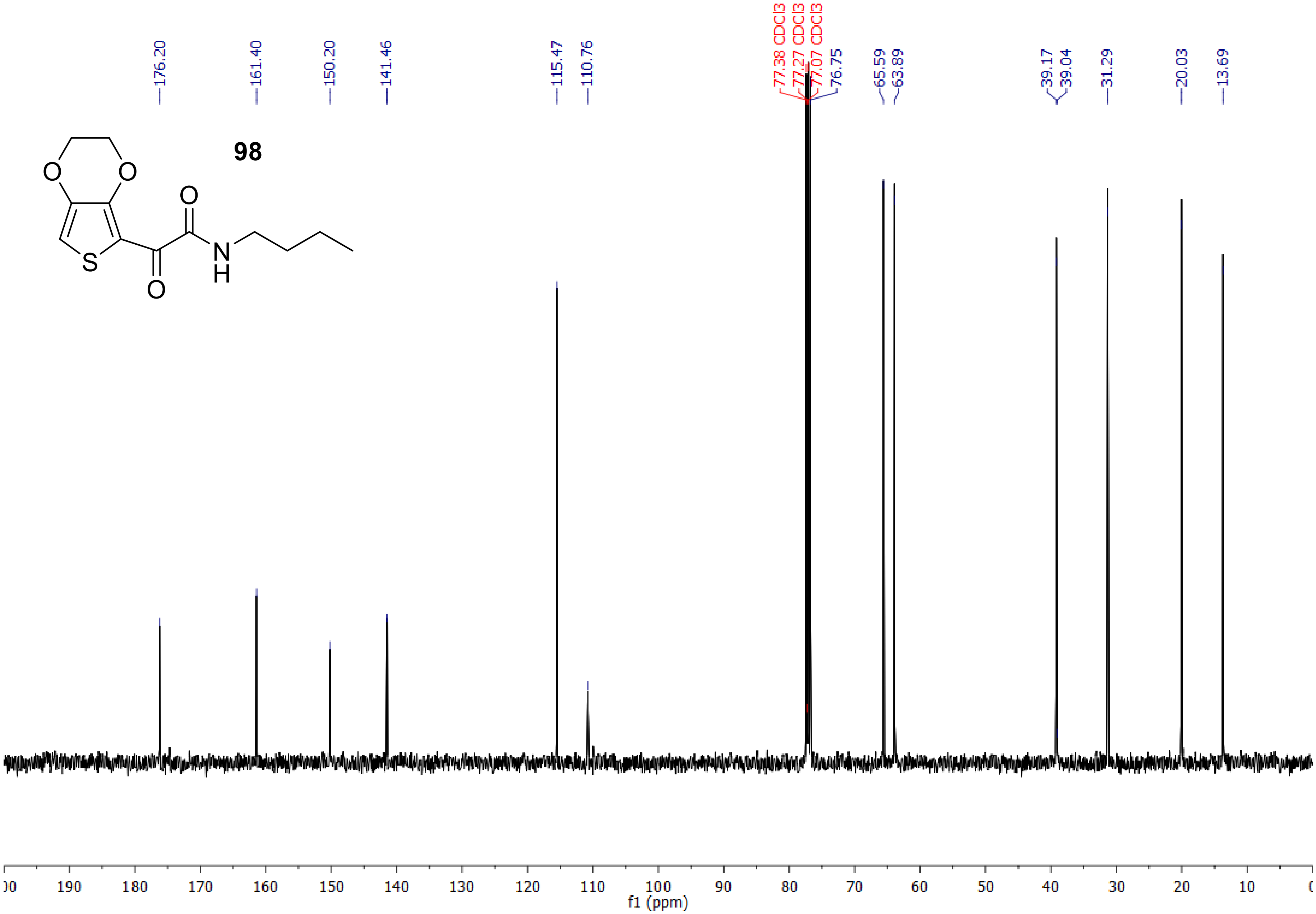

^{1}H NMR (400 MHz, $CDCl_3$)

Figure S131. ^{1}H NMR of **99**

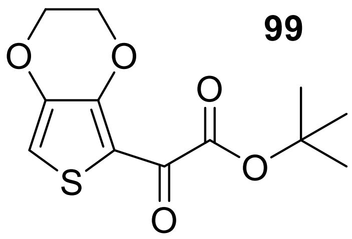

99

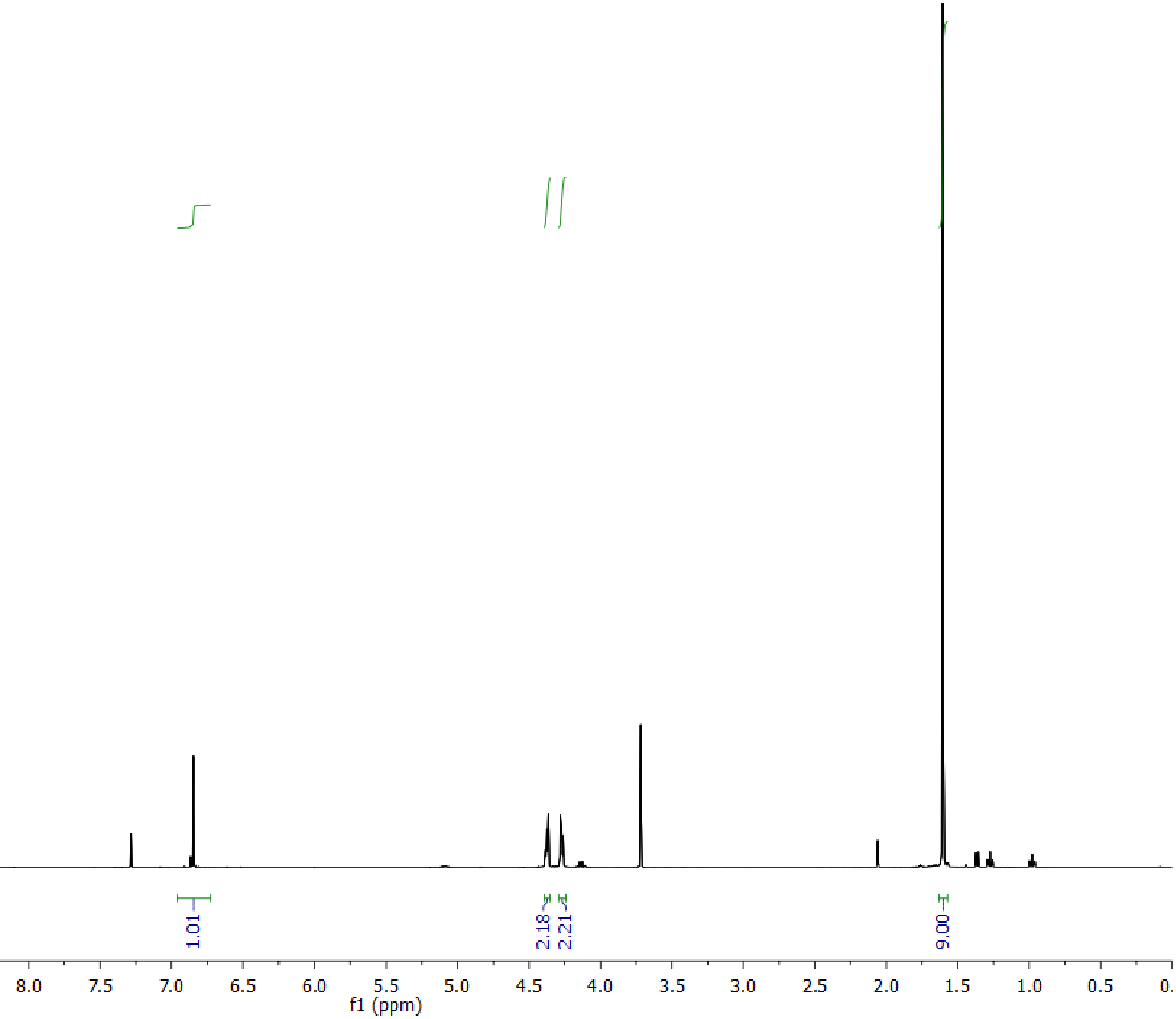

^{13}C NMR (100 MHz, $CDCl_3$)

Figure S132. ^{13}C NMR of **99**

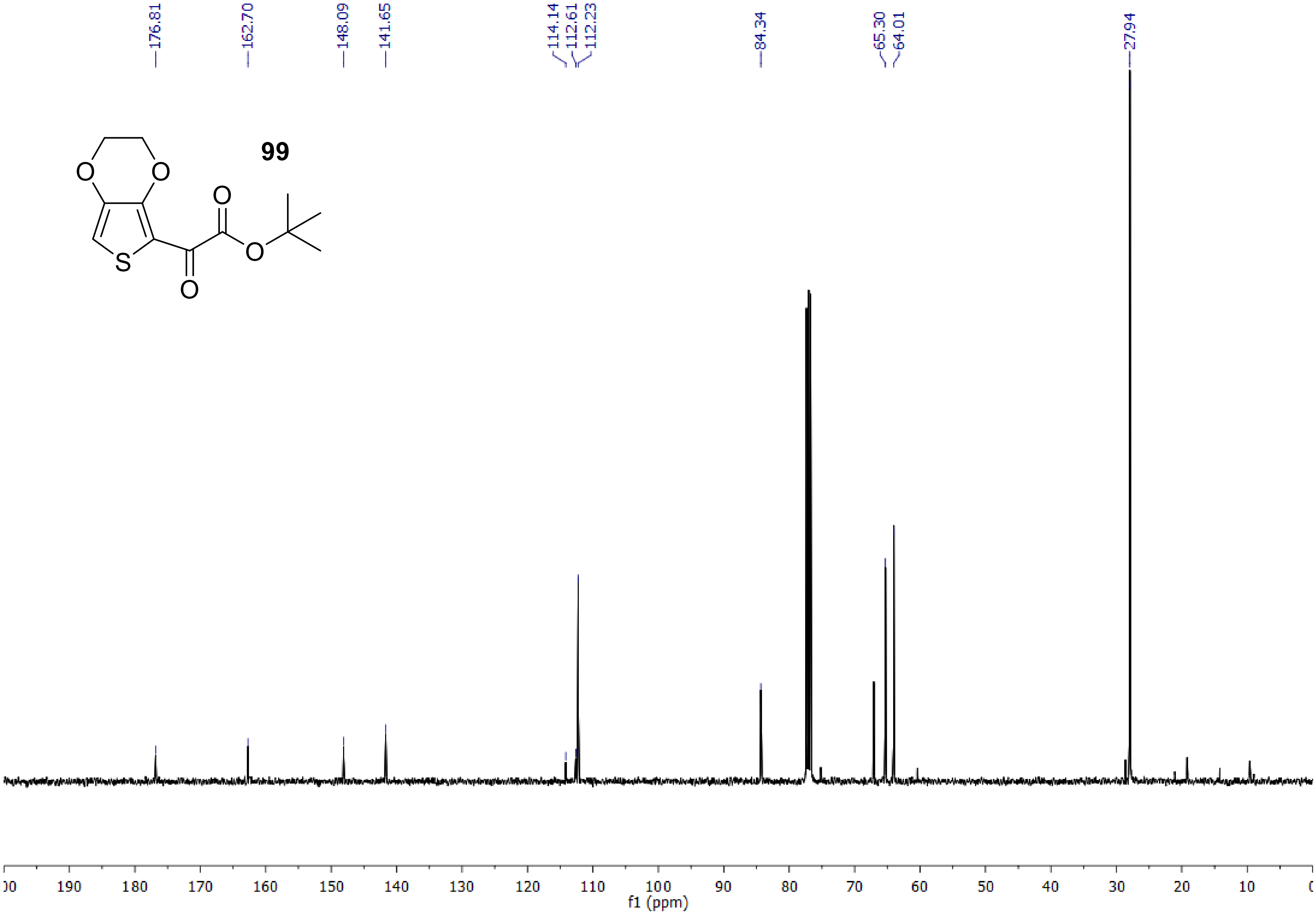

^{1}H NMR (400 MHz, $CDCl_3$)

Figure S133. ^{1}H NMR of **100**

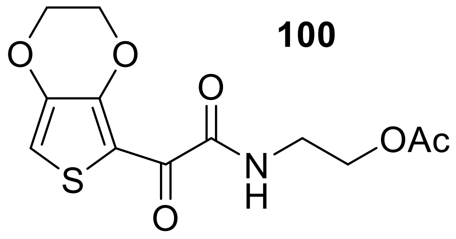

100

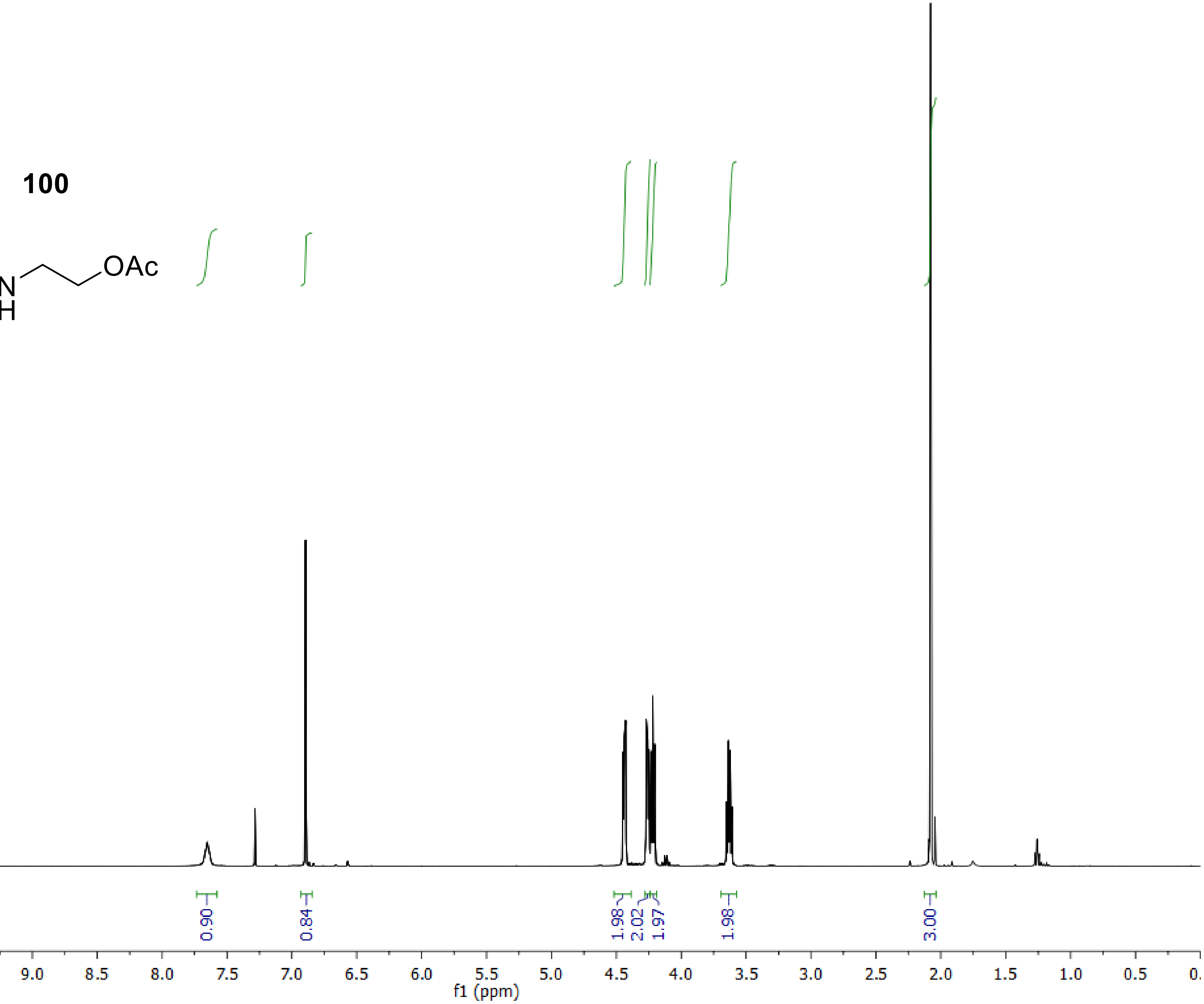

^{13}C NMR (100 MHz, $CDCl_3$)

Figure S134. ^{13}C NMR of **100**

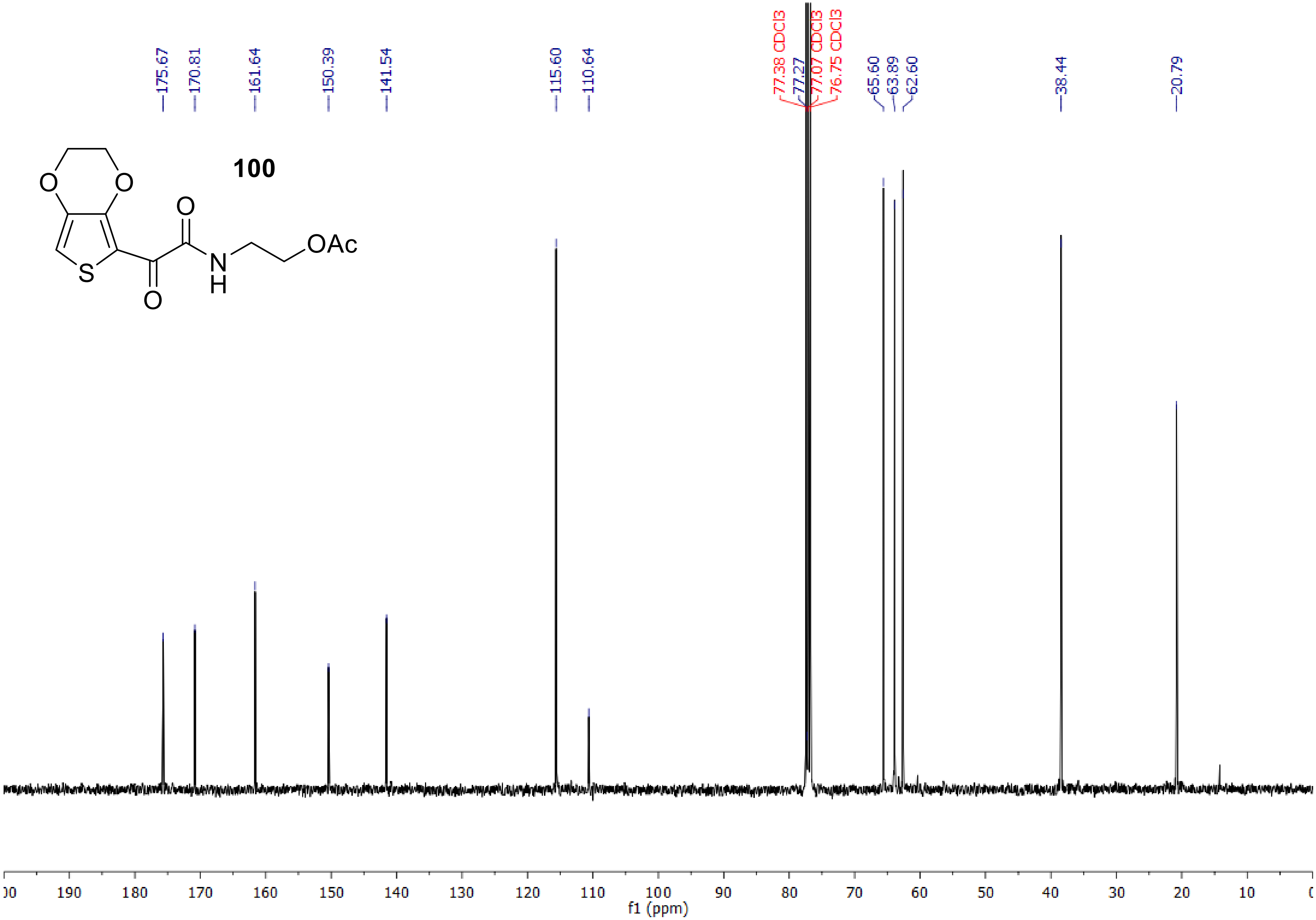

^{1}H NMR (400 MHz, $CDCl_3$)

Figure S135. ^{1}H NMR of **101**

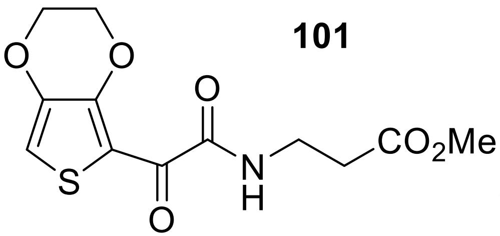

101

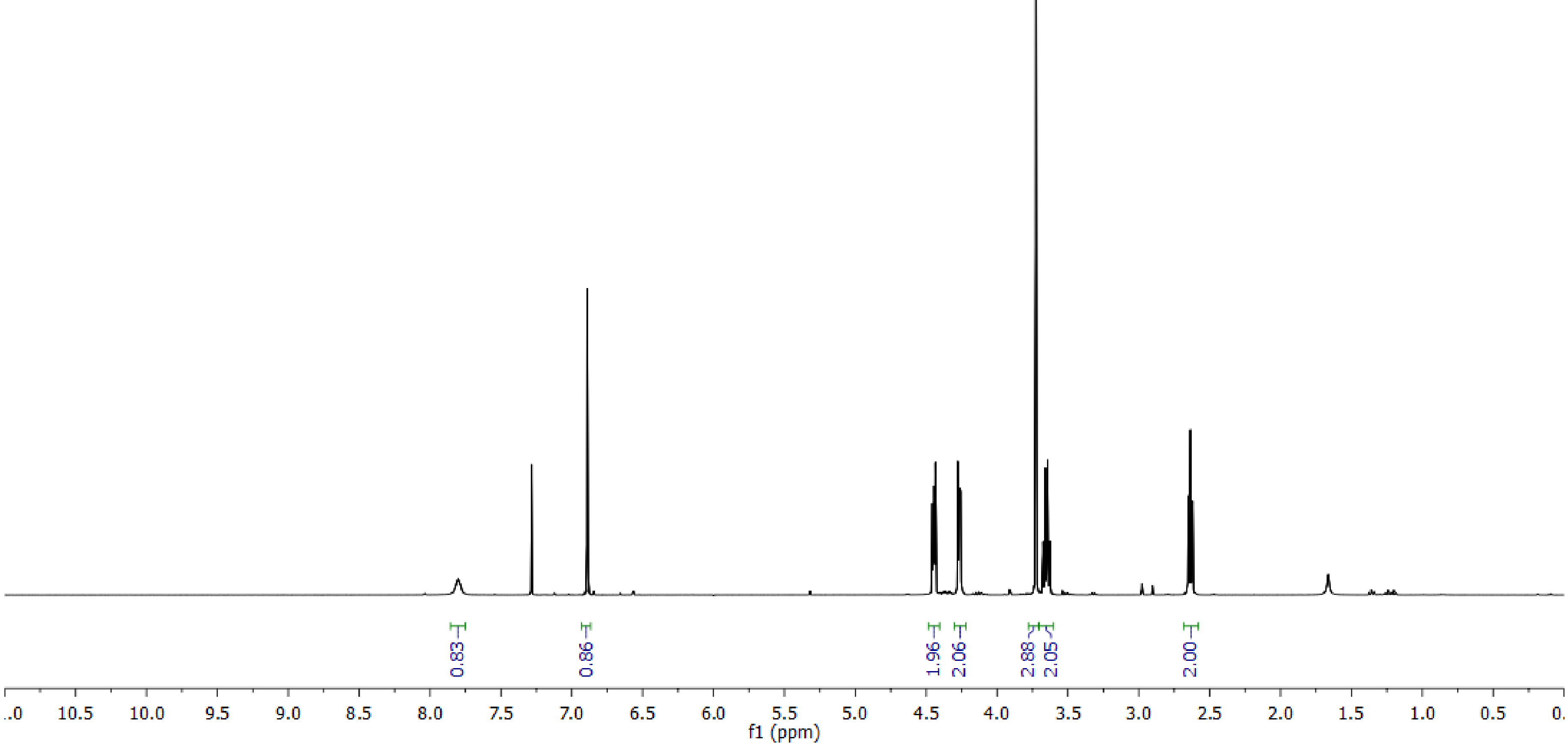

^{13}C NMR (100 MHz, $CDCl_3$)

Figure S136. ^{13}C NMR of **101**

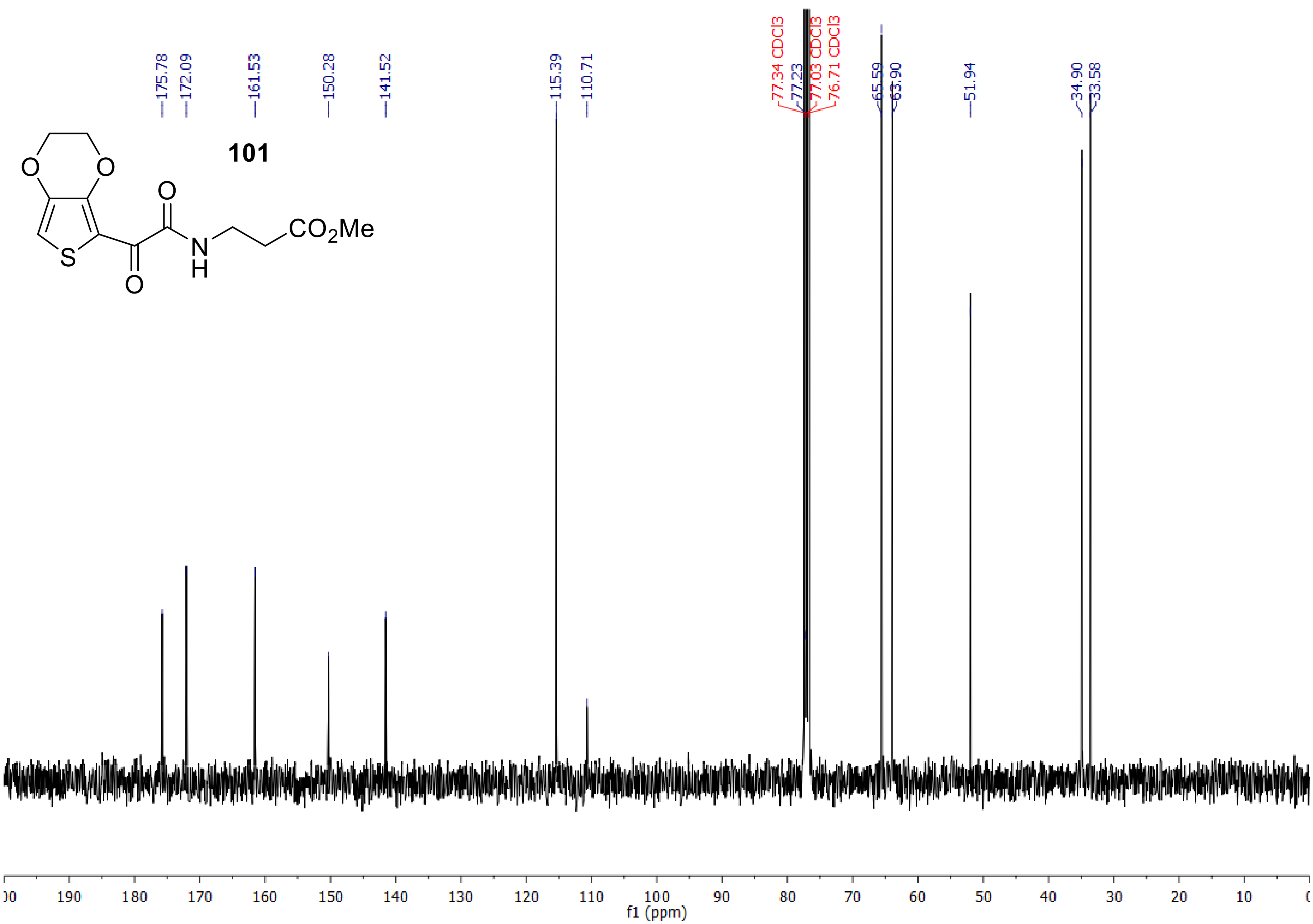

^{1}H NMR (400 MHz, $CDCl_3$)

Figure S137. ^{1}H NMR of **102**

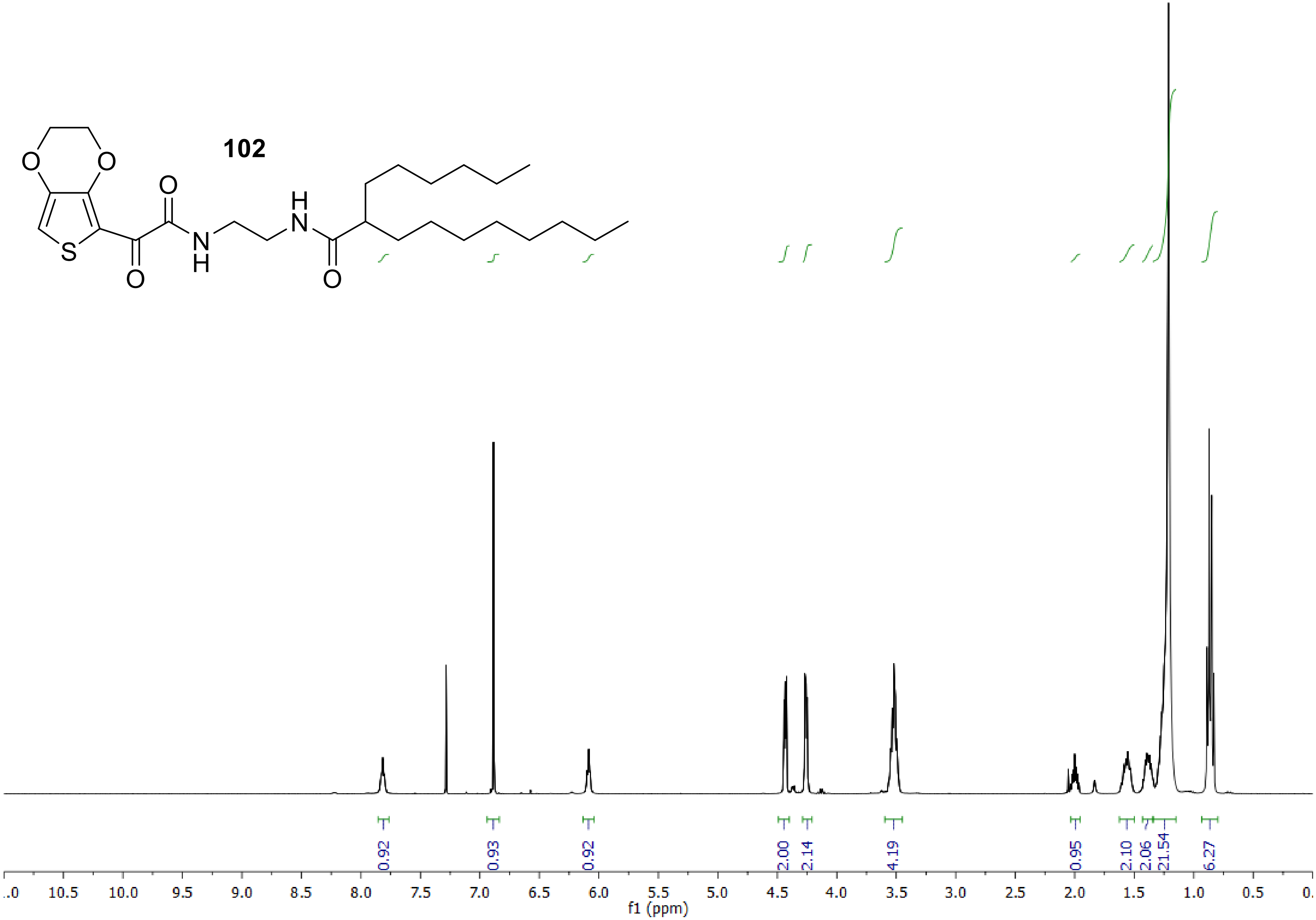

^{13}C NMR (100 MHz, $CDCl_3$)

Figure S138. ^{13}C NMR of **102**

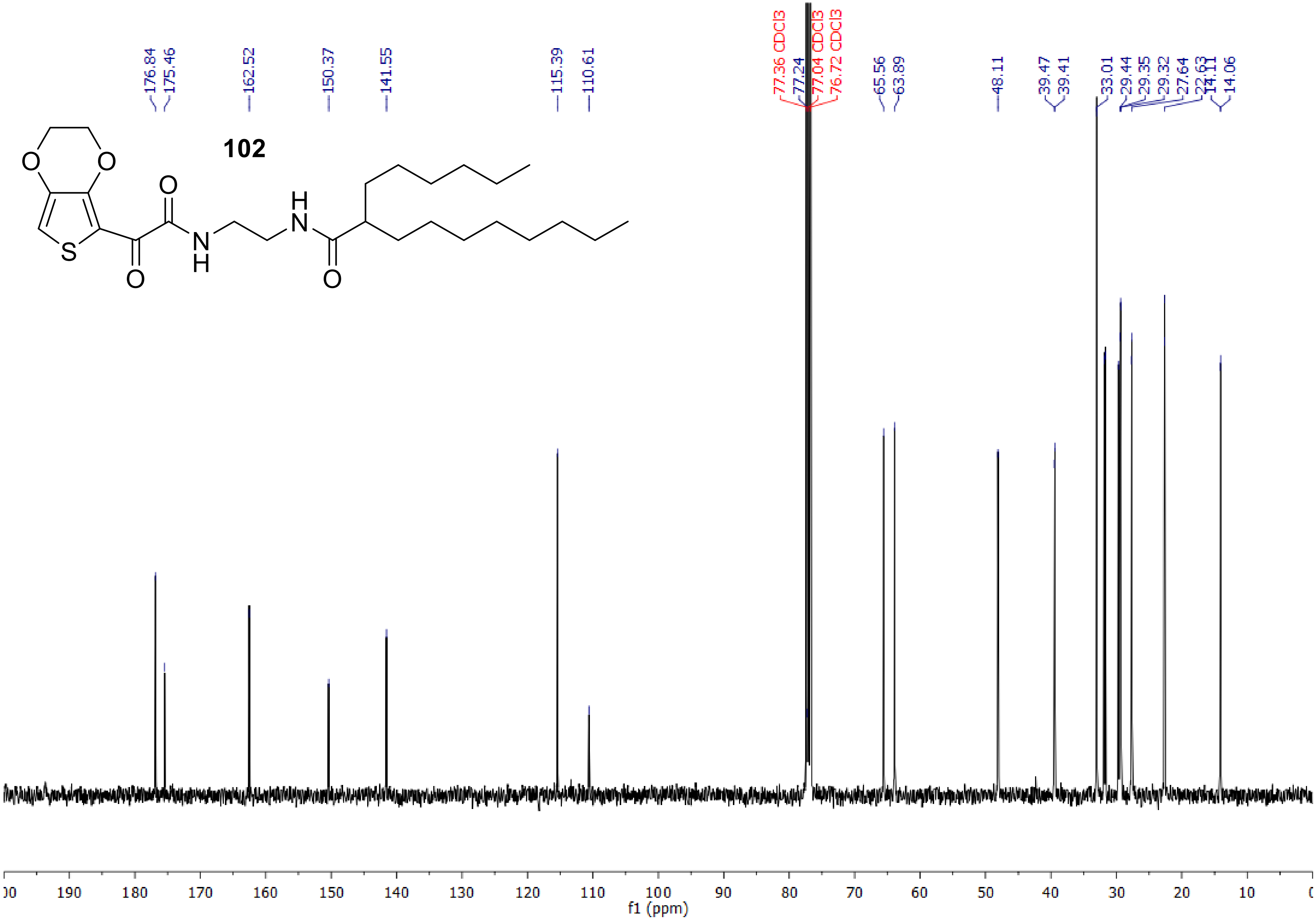

^{1}H NMR (400 MHz, $CDCl_3$)

Figure S139. ^{1}H NMR of **103**

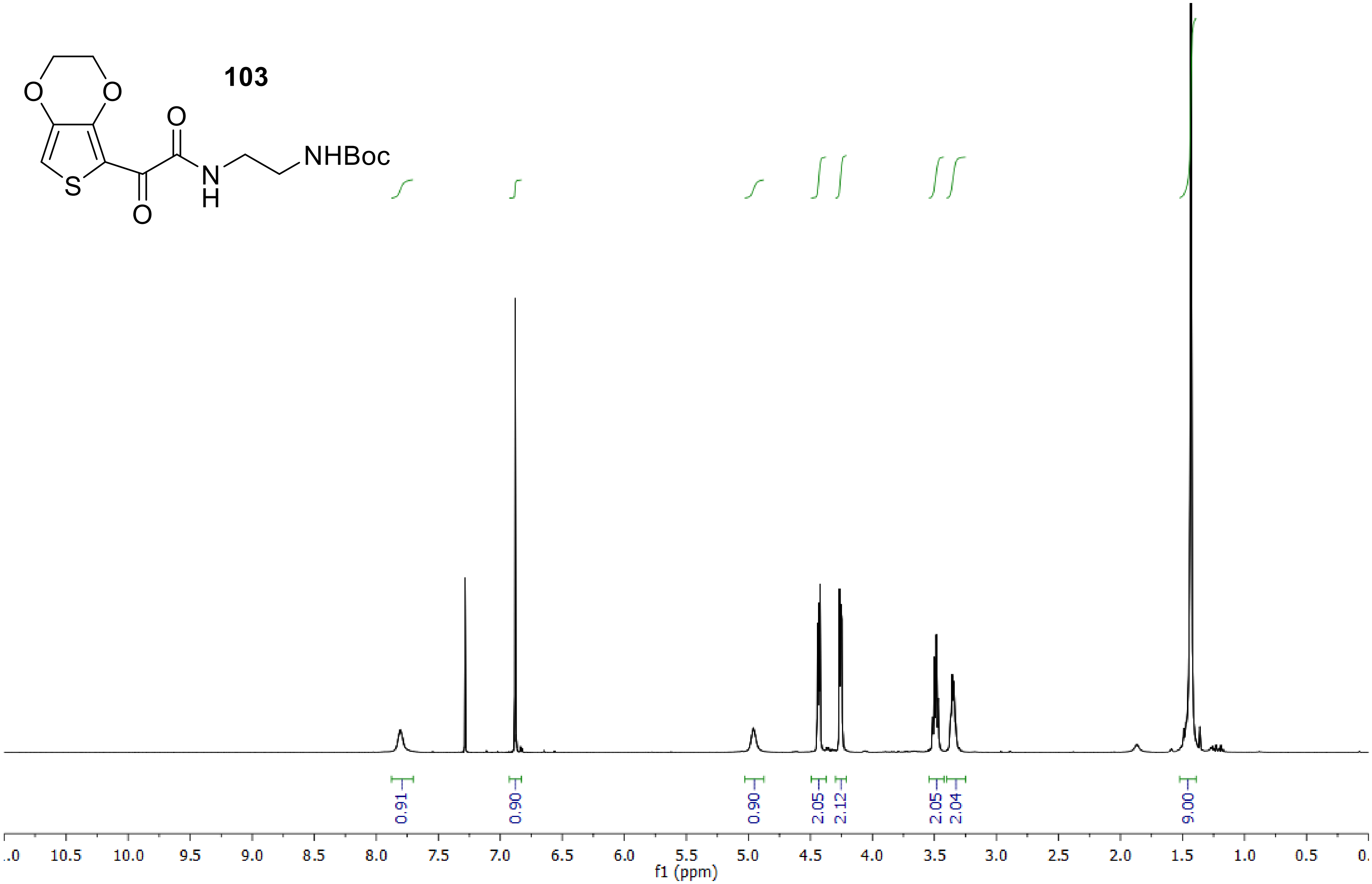

^{13}C NMR (100 MHz, $CDCl_3$)

Figure S140. ^{13}C NMR of **103**

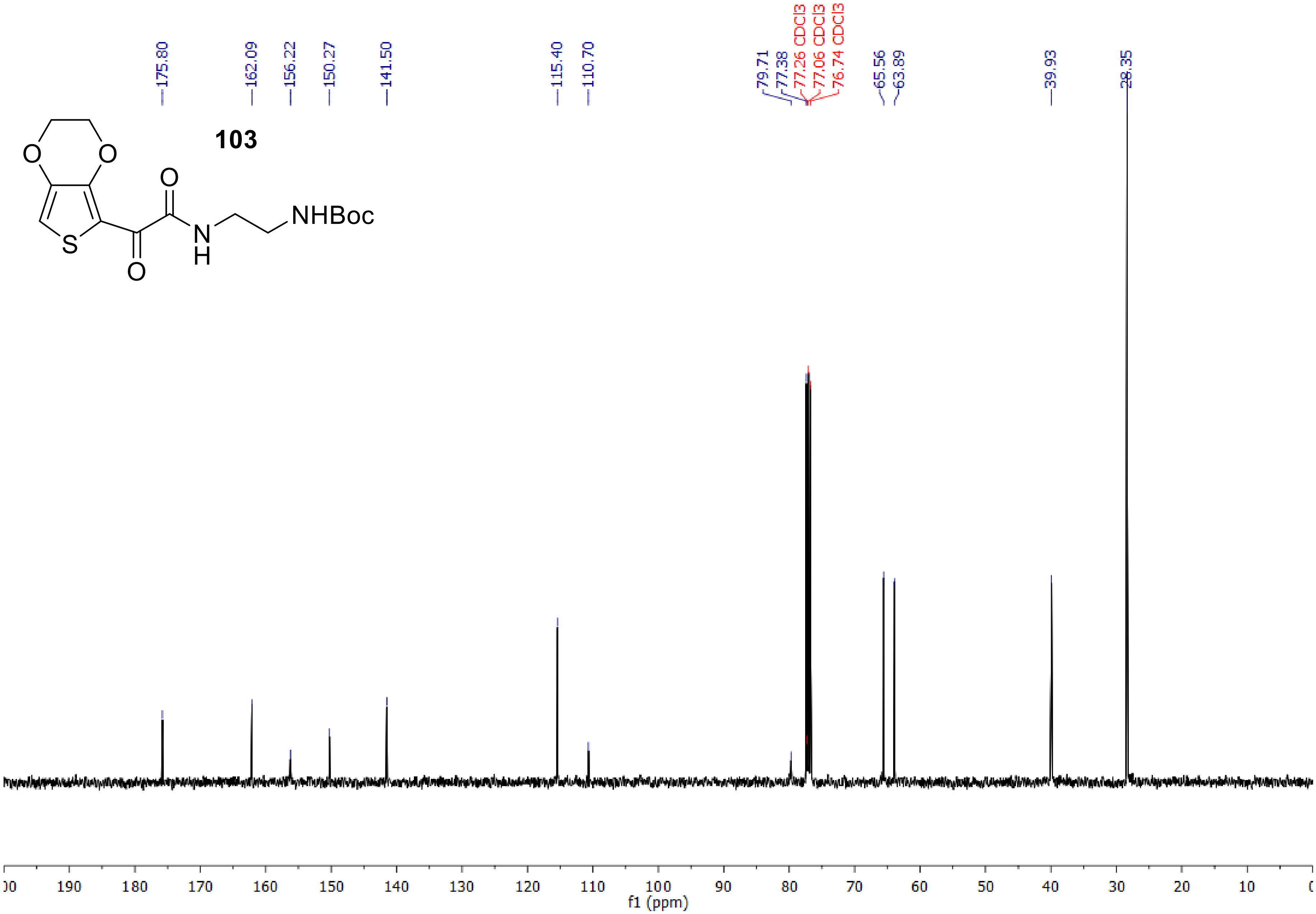

^{1}H NMR (400 MHz, $CDCl_3$)

Figure S141. ^{1}H NMR of **104**

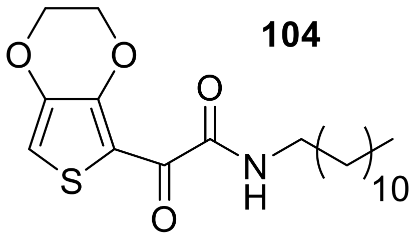

104

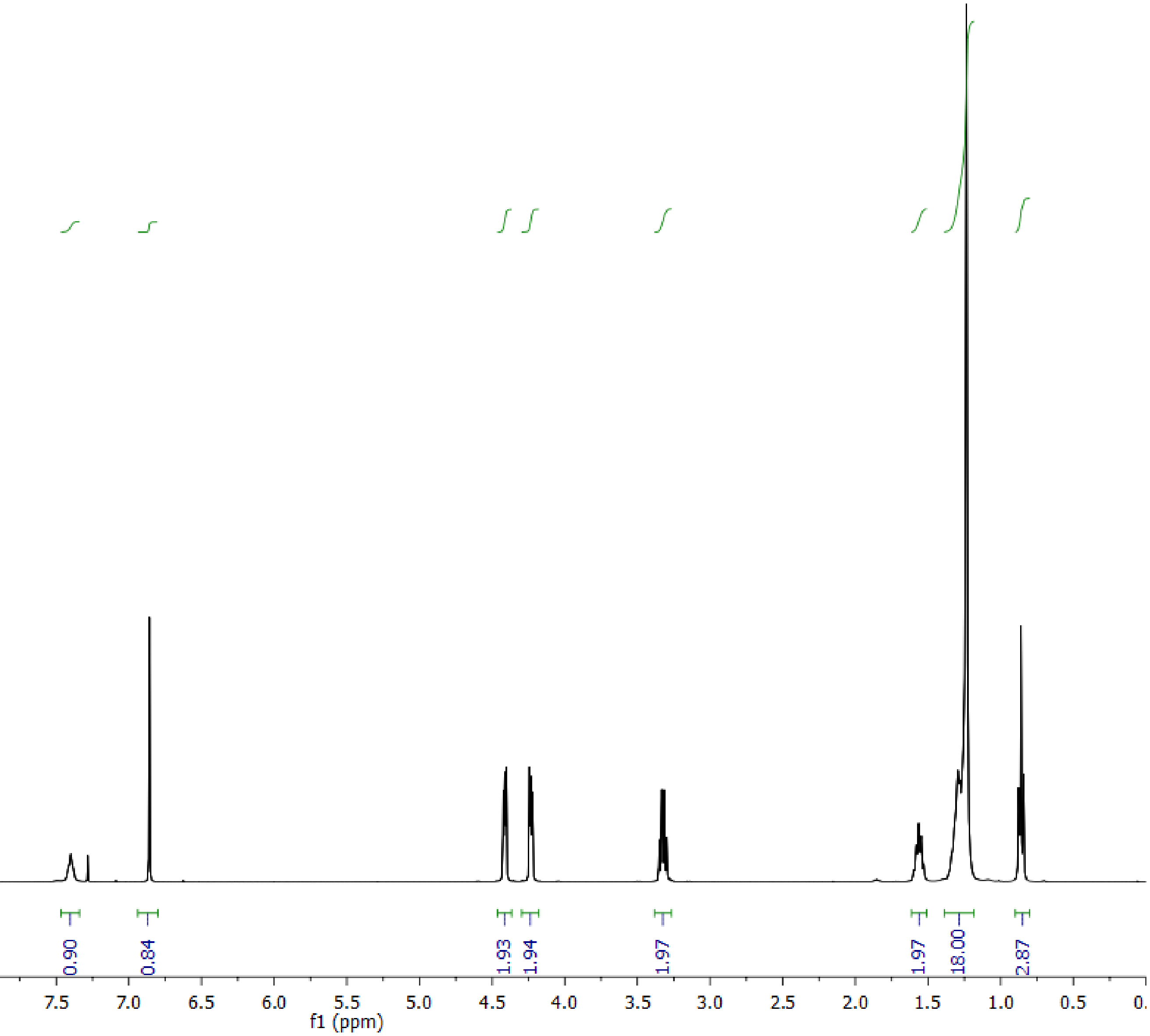

^{13}C NMR (100 MHz, $CDCl_3$)

Figure S142. ^{13}C NMR of **104**

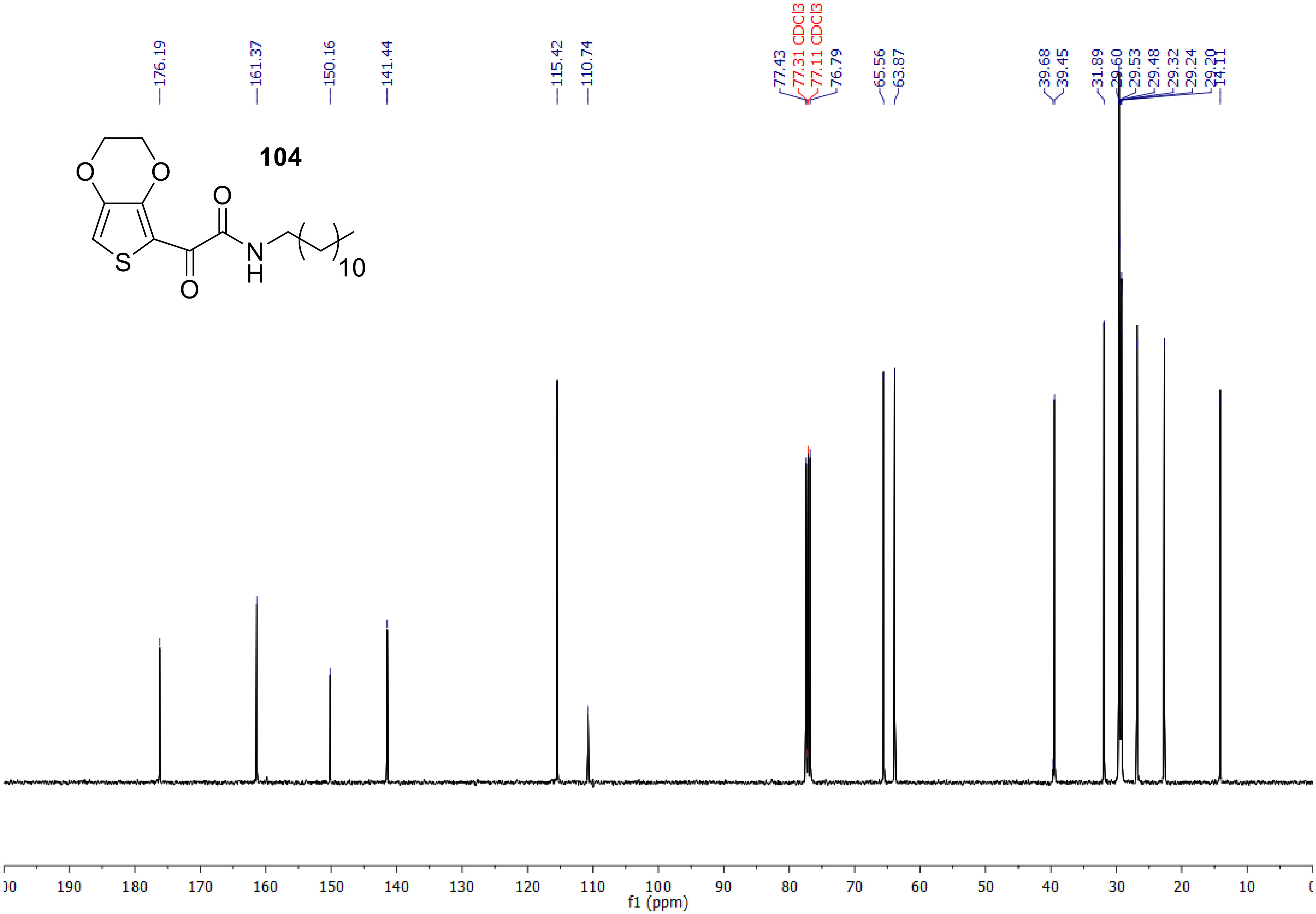

^{1}H NMR (400 MHz, $CDCl_3$)

Figure S143. ^{1}H NMR of **105**

105

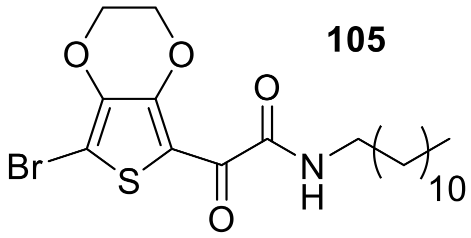

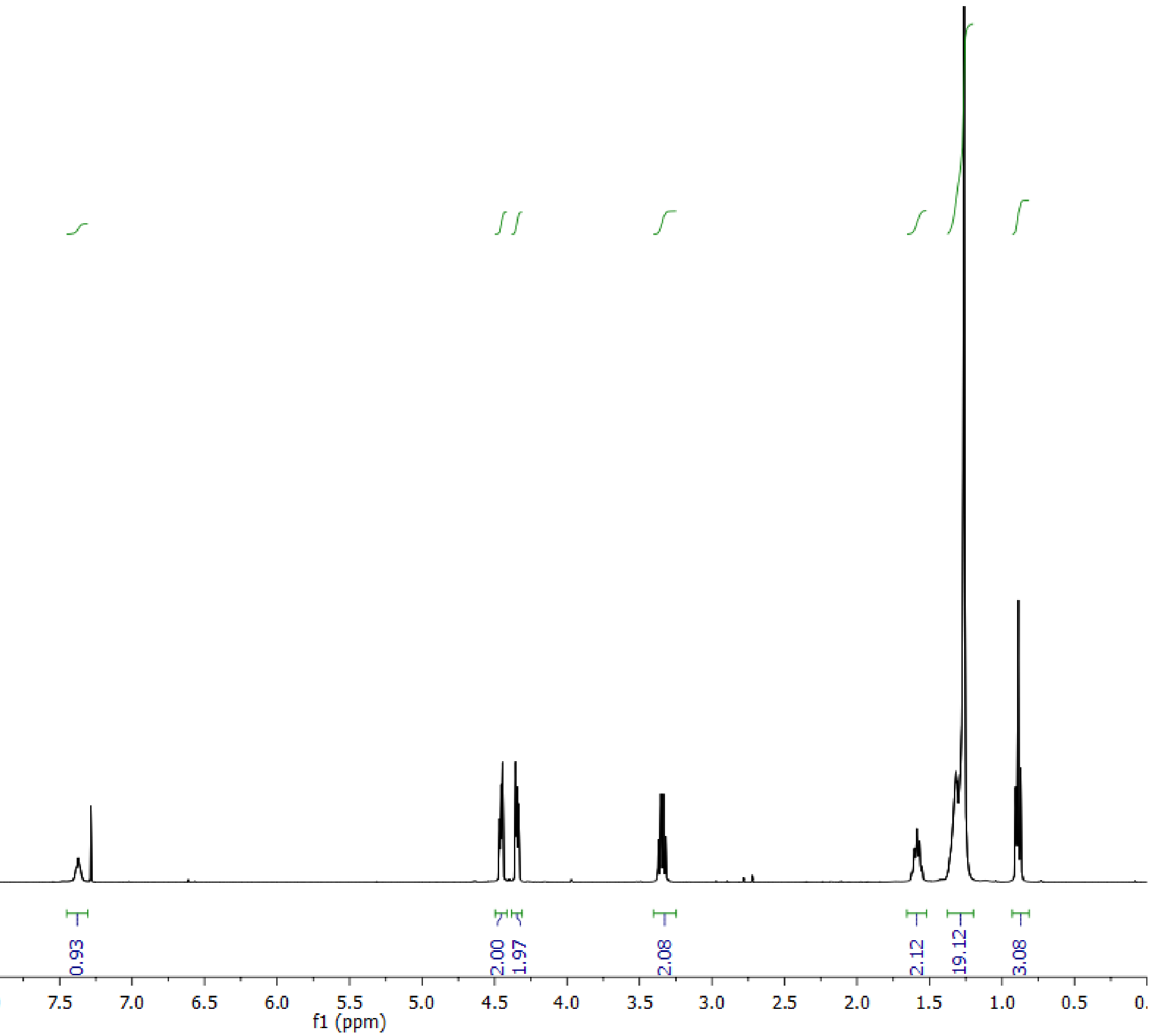

^{13}C NMR (100 MHz, $CDCl_3$)

Figure S144. ^{13}C NMR of **105**

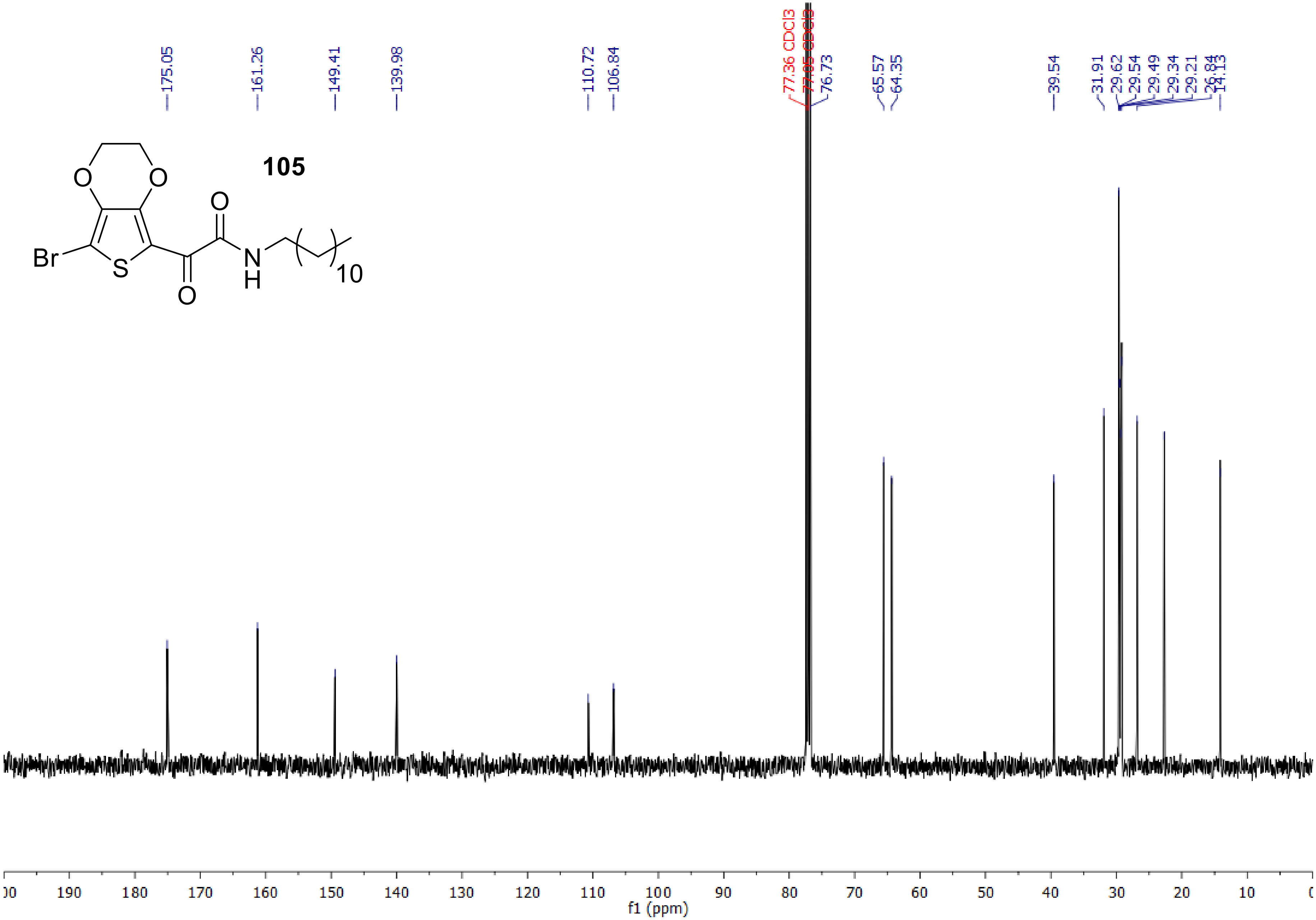

^{1}H NMR (400 MHz, DMSO-d_6)

Figure S145. ^{1}H NMR of **106**

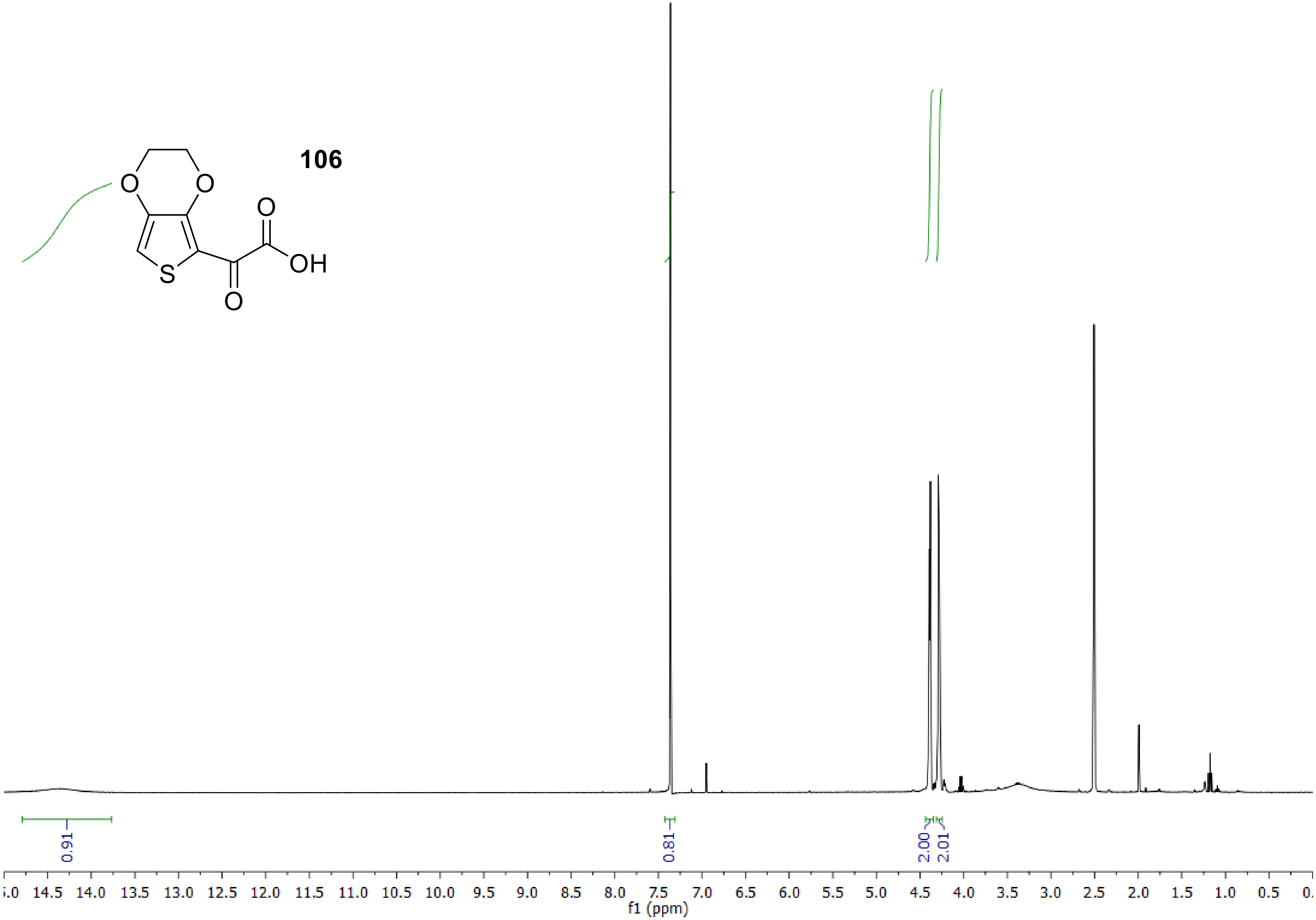

^{13}C NMR (100 MHz, DMSO-d_6)

Figure S146. ^{13}C NMR of **106**

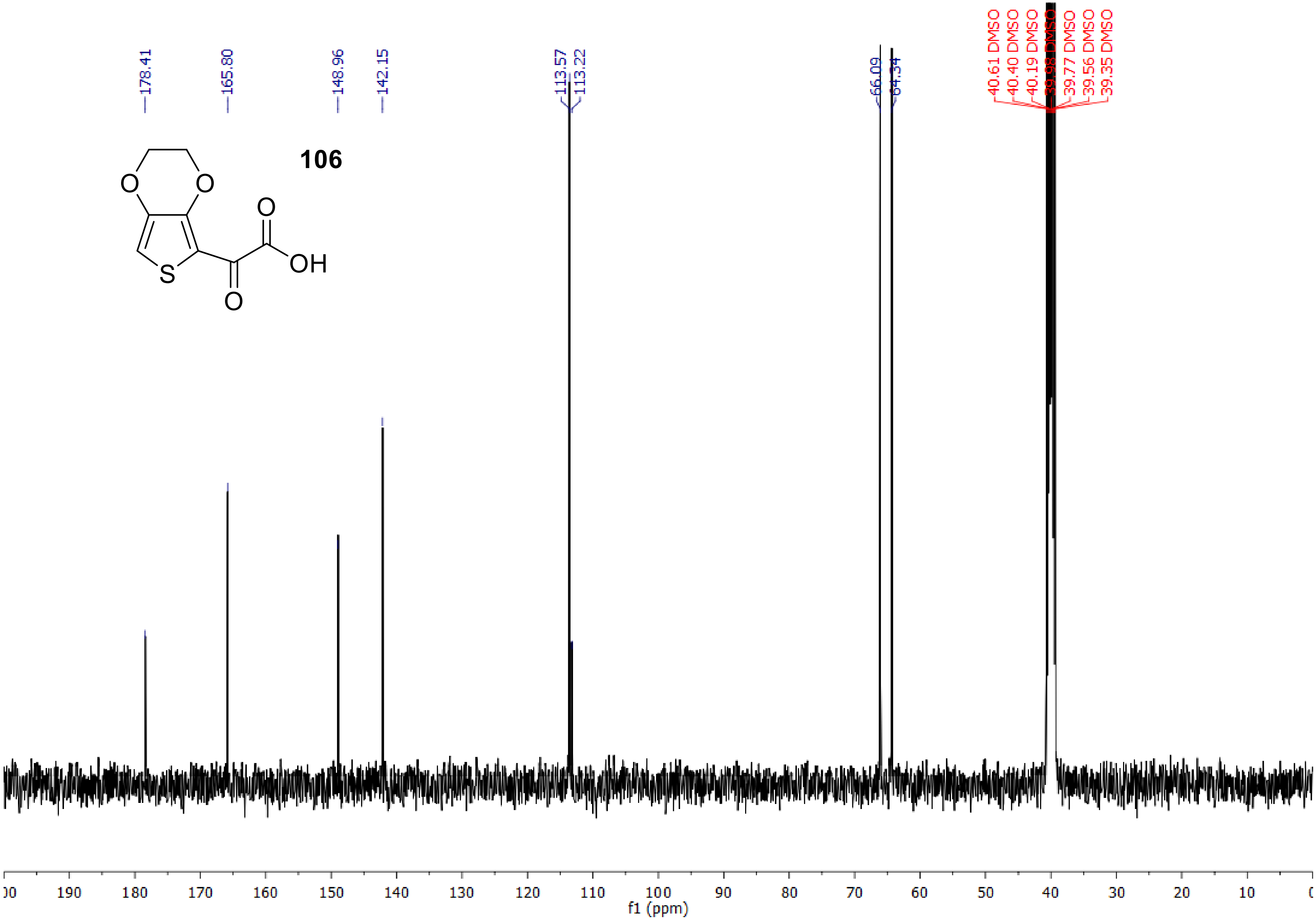

^{1}H NMR (400 MHz, DMSO-d_6)

Figure S147. ^{1}H NMR of **107**

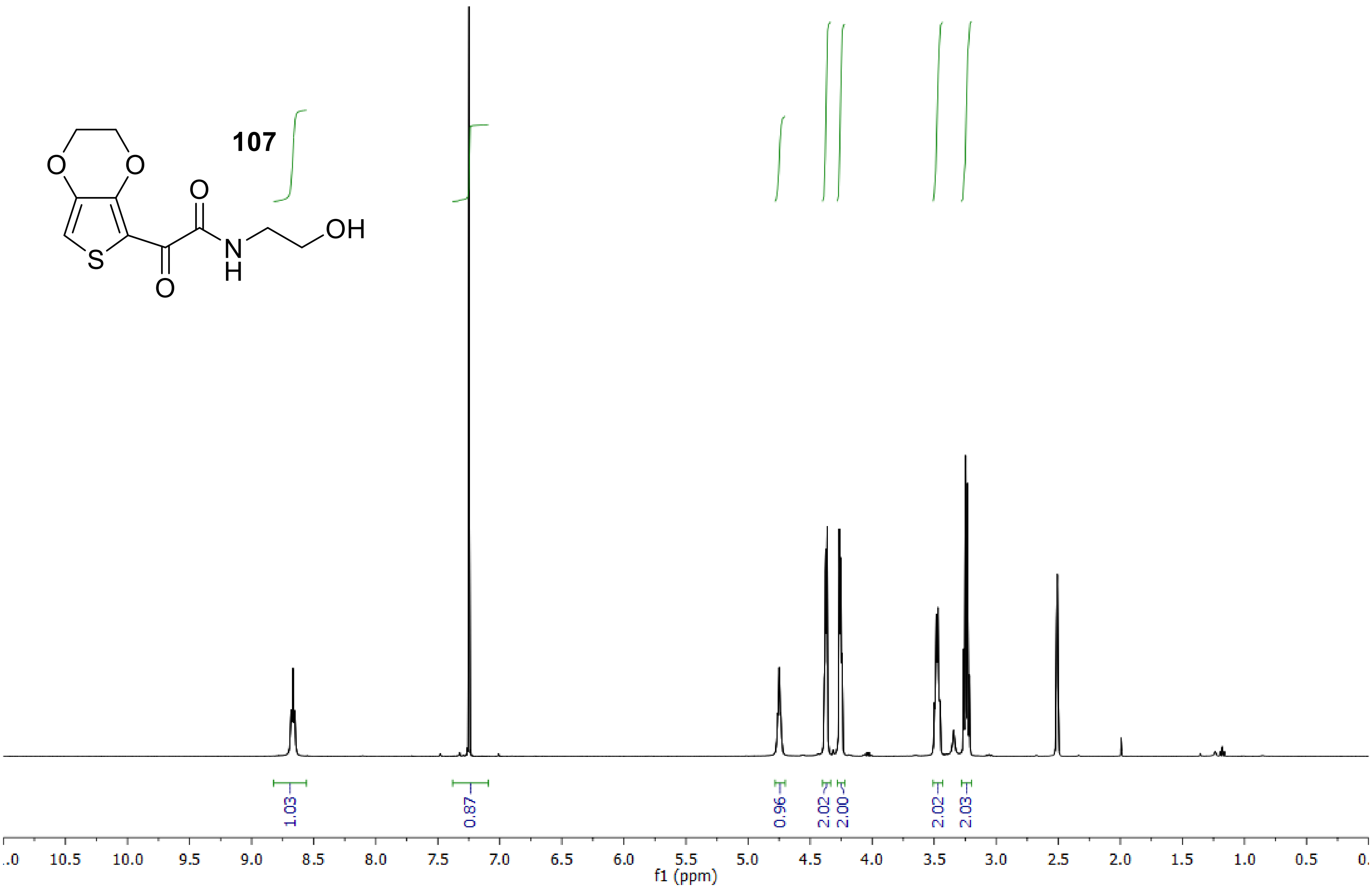

^{13}C NMR (100 MHz, DMSO-d_6)

Figure S148. ^{13}C NMR of **107**

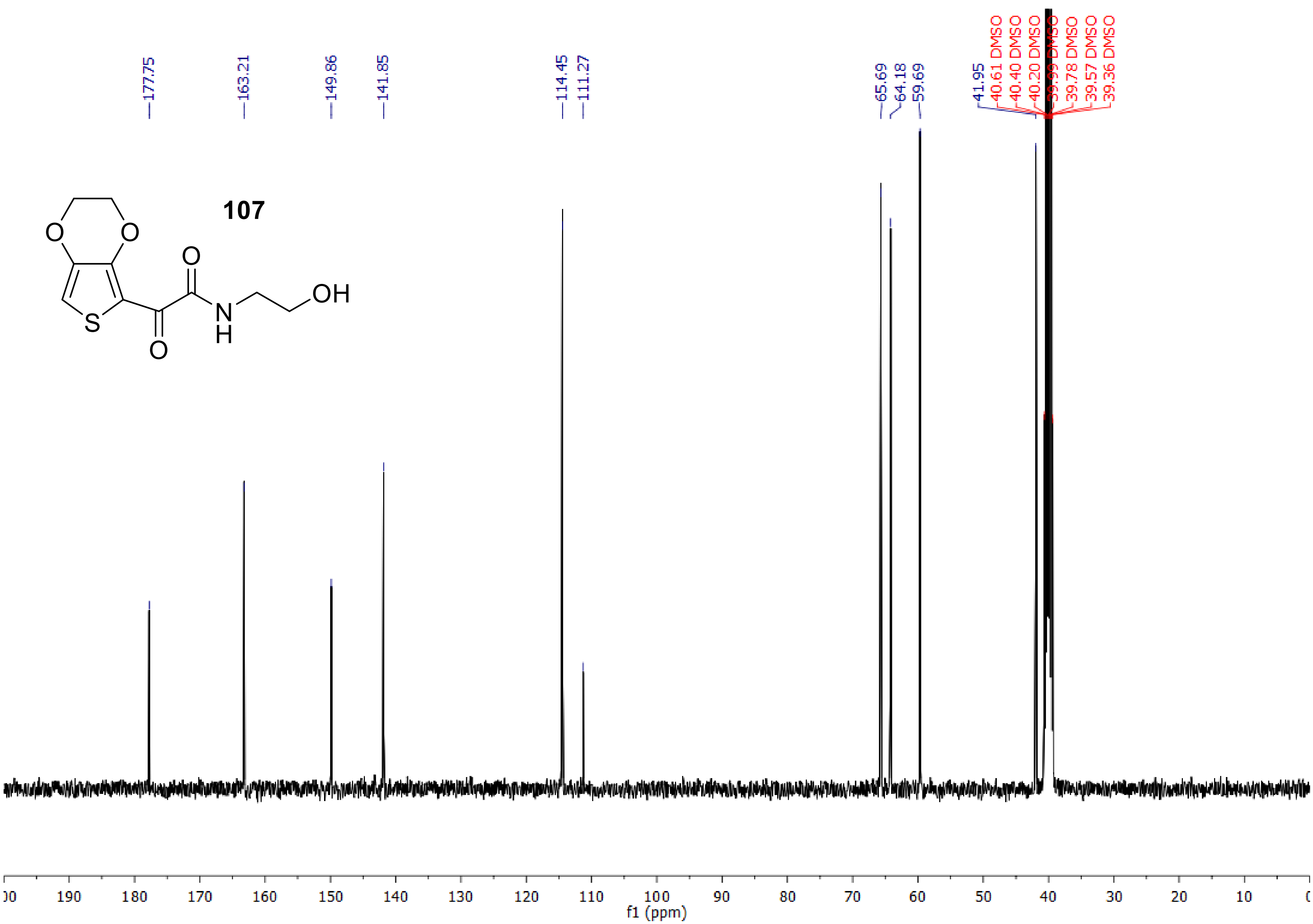

^{1}H NMR (400 MHz, DMSO-d$_6$)

Figure S149. ^{1}H NMR of **108**

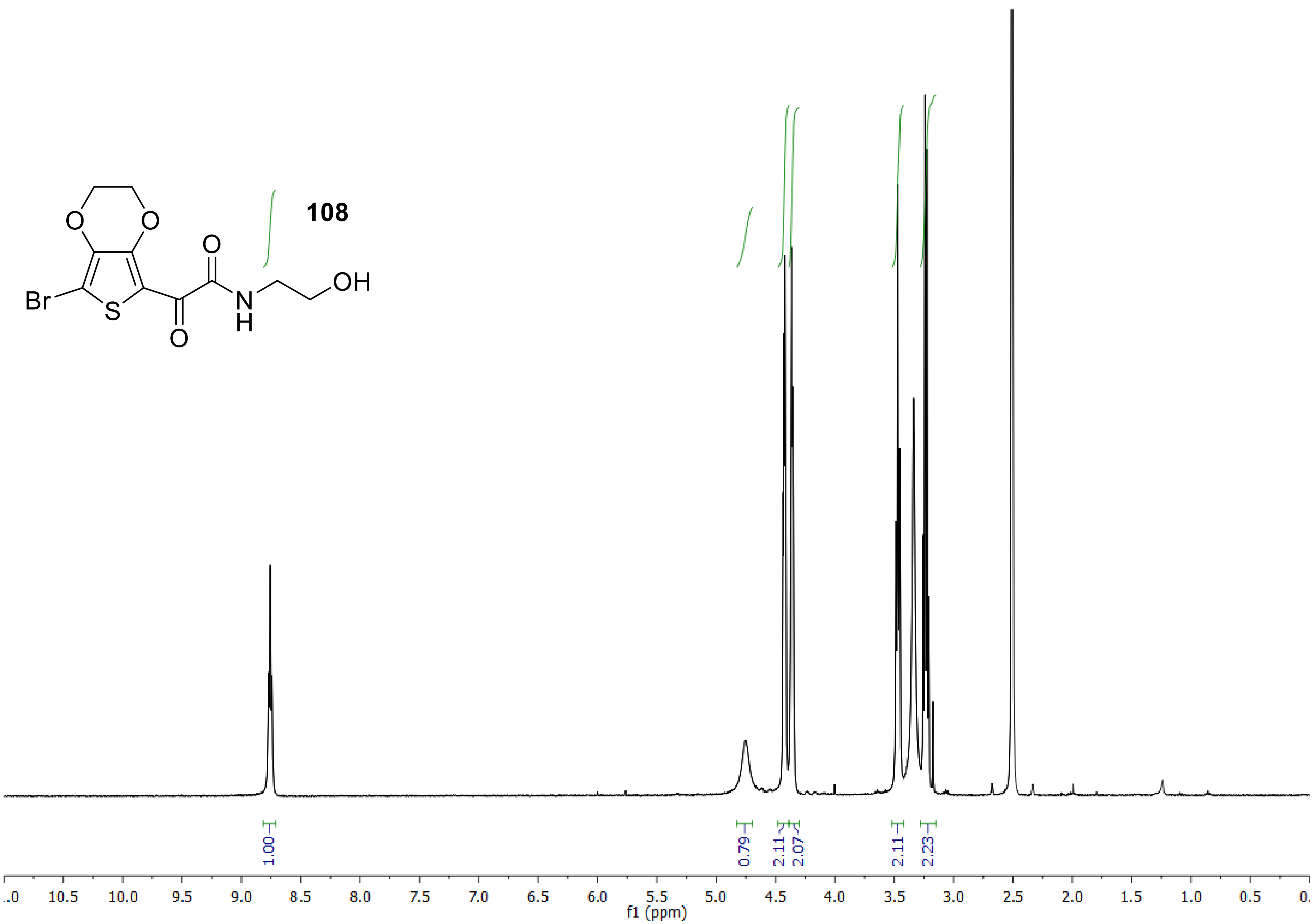

^{13}C NMR (100 MHz, DMSO-d_6)

Figure S150. ^{13}C NMR of **108**

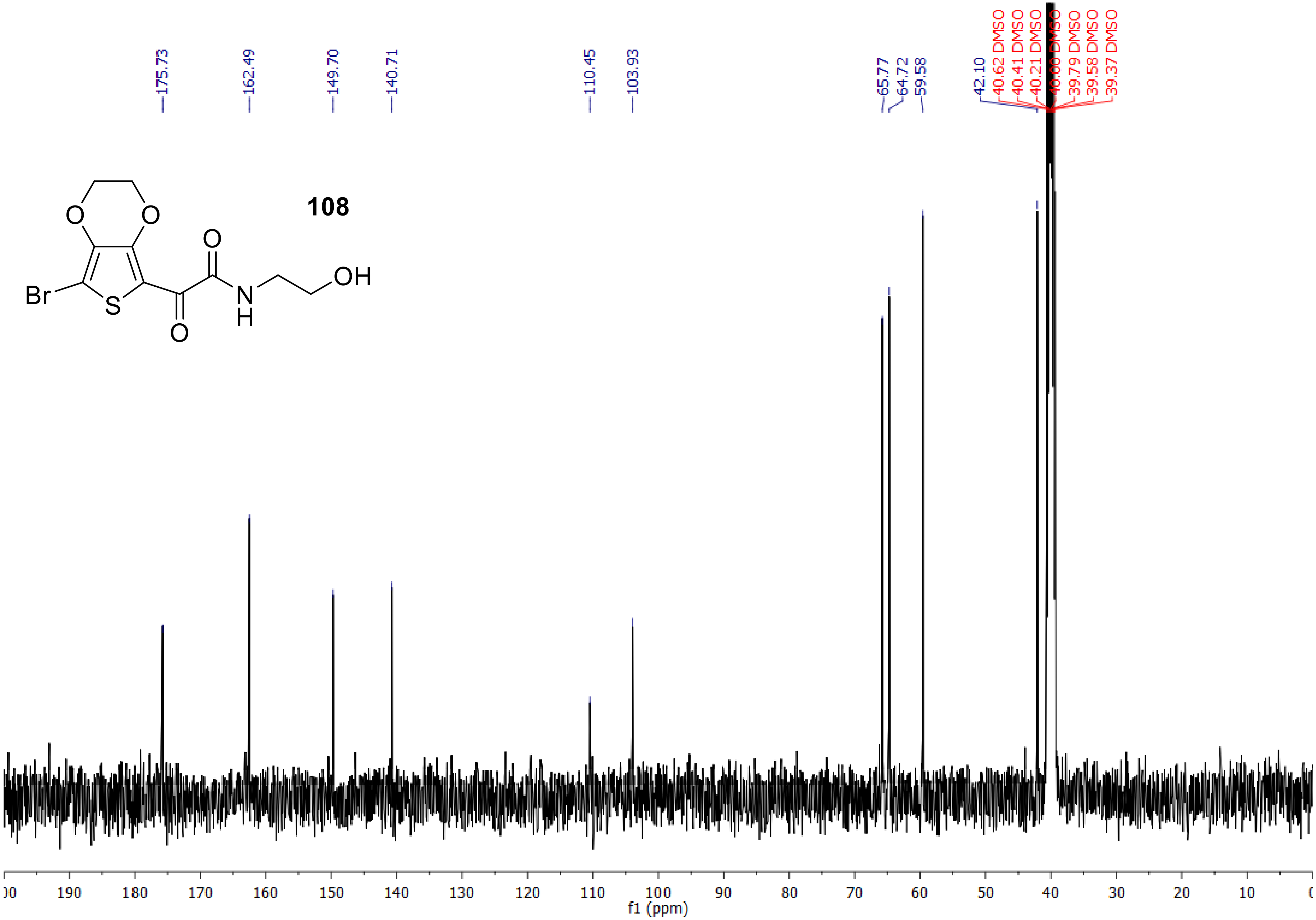

^{1}H NMR (400 MHz, $CDCl_3$)

Figure S151. ^{1}H NMR of **109**

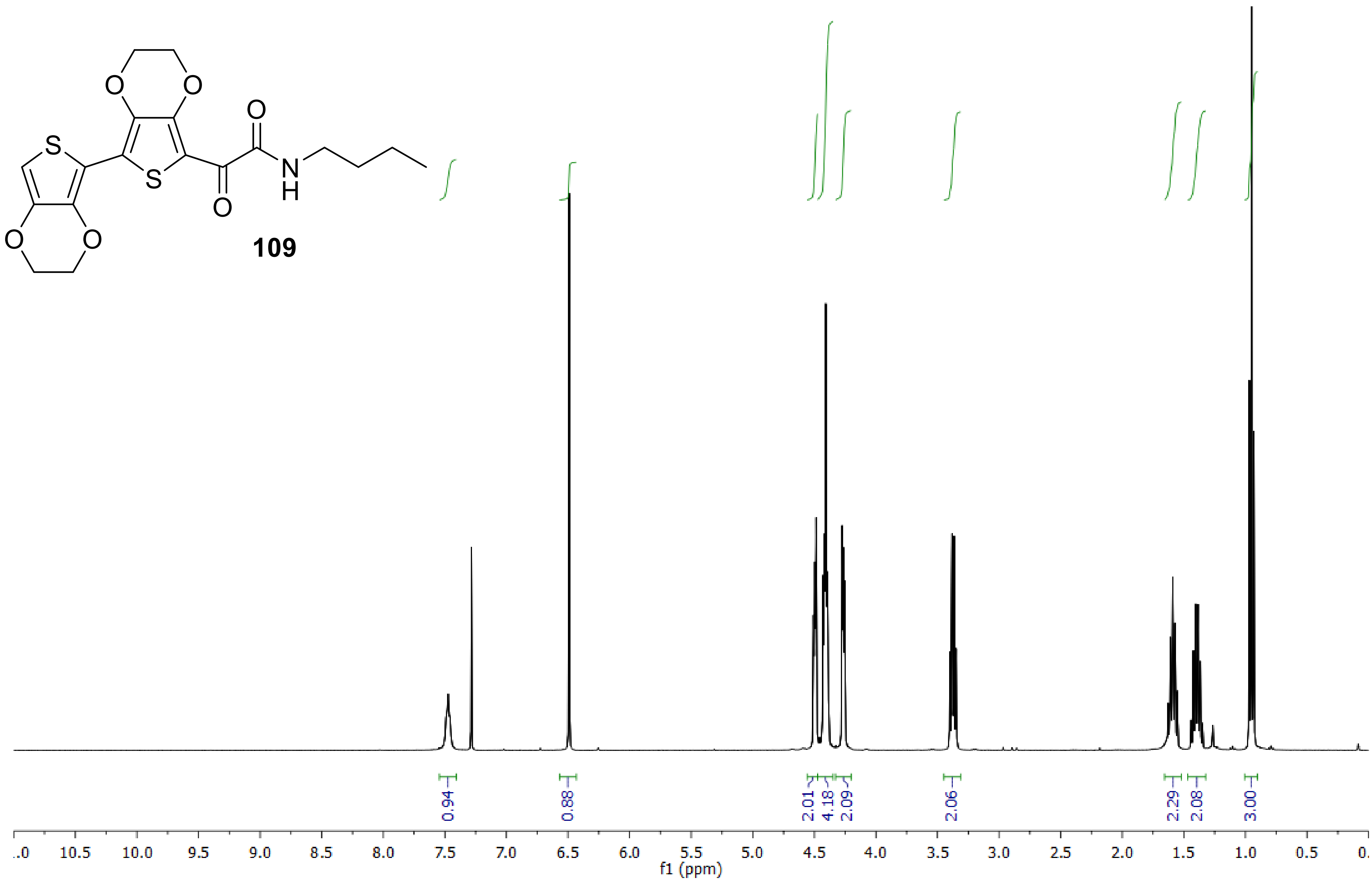

^{13}C NMR (100 MHz, $CDCl_3$)

Figure S152. ^{13}C NMR of **109**

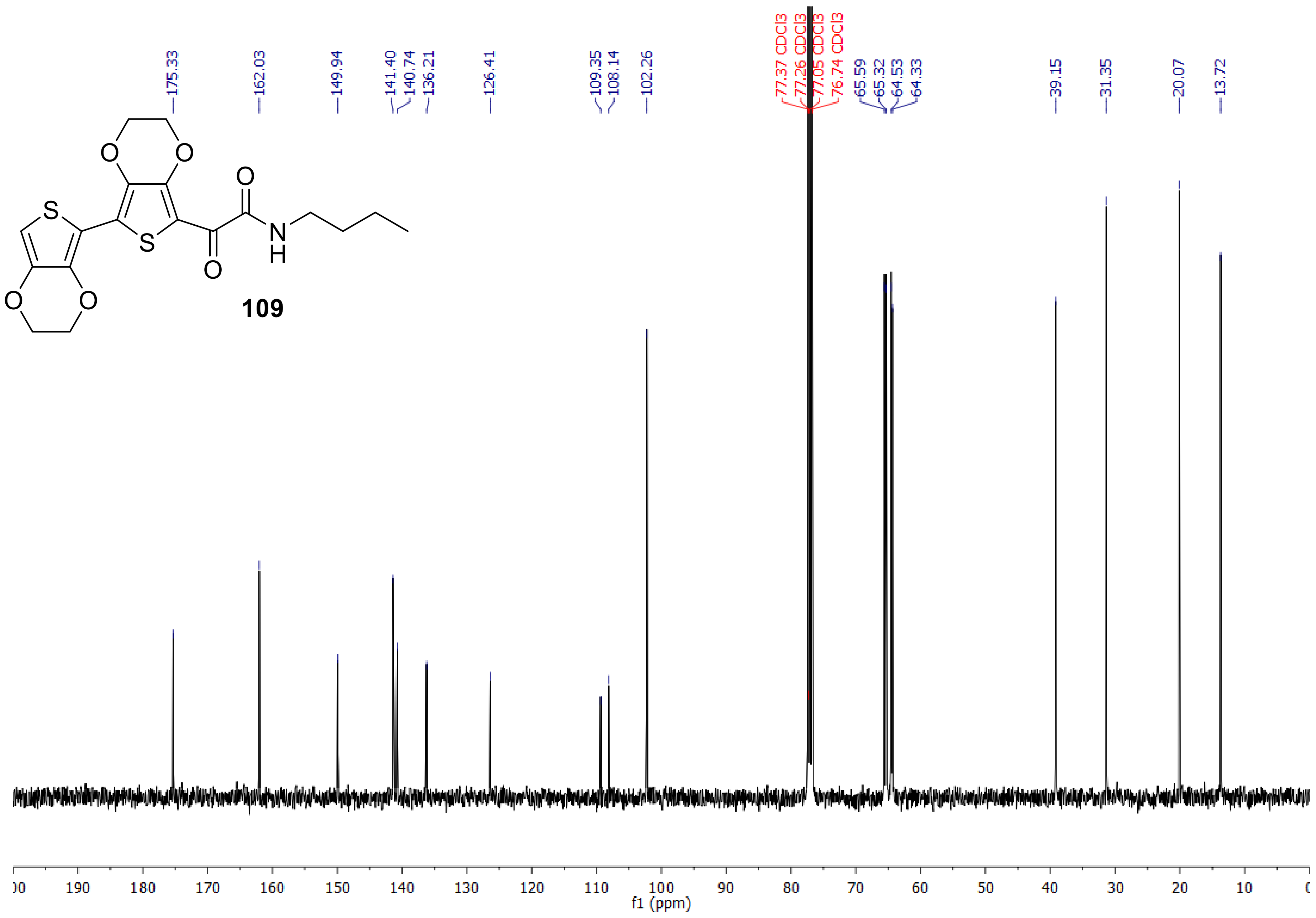

^1H NMR (400 MHz, $CDCl_3$)

Figure S153. ^1H NMR of **110**

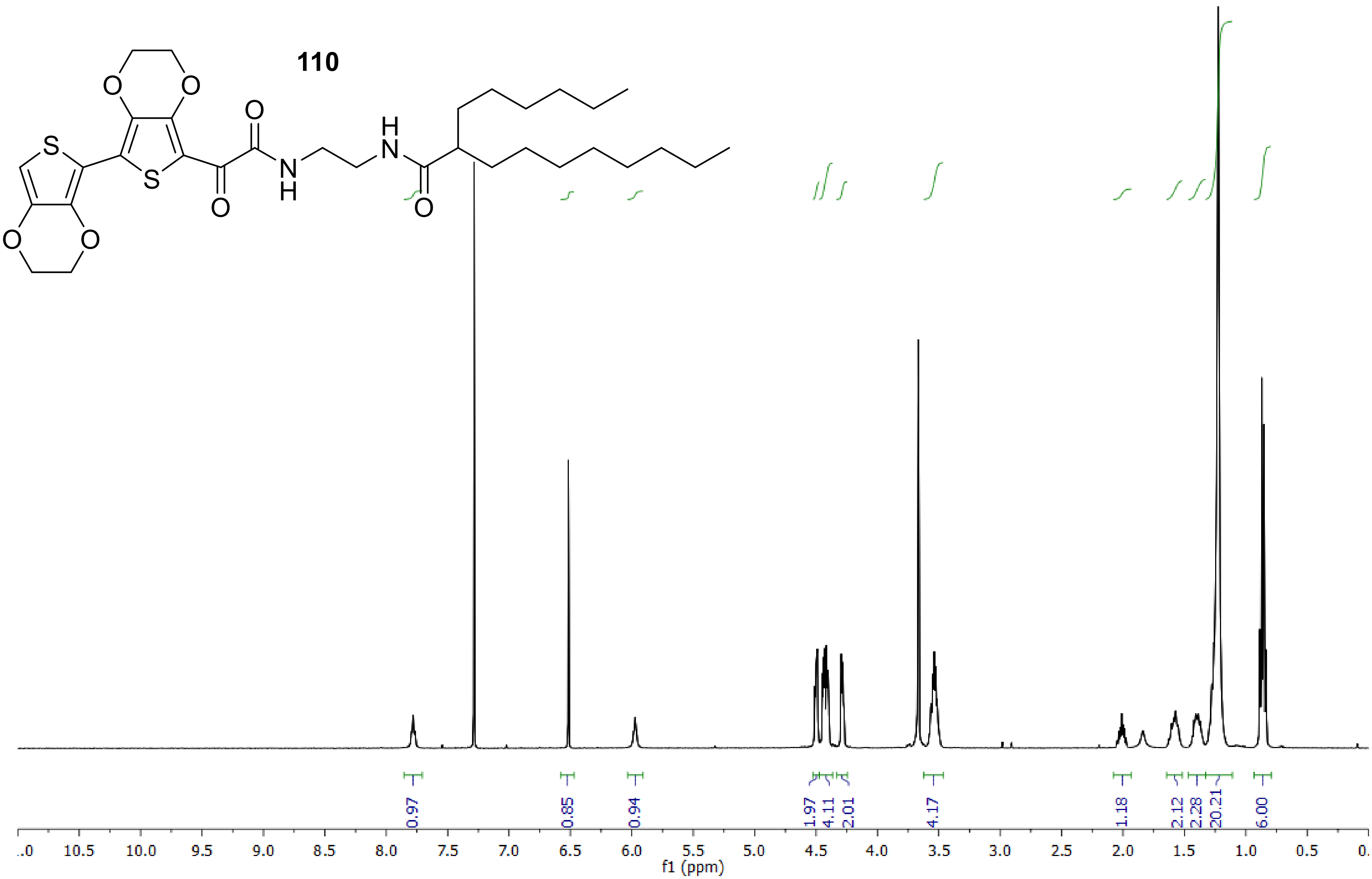

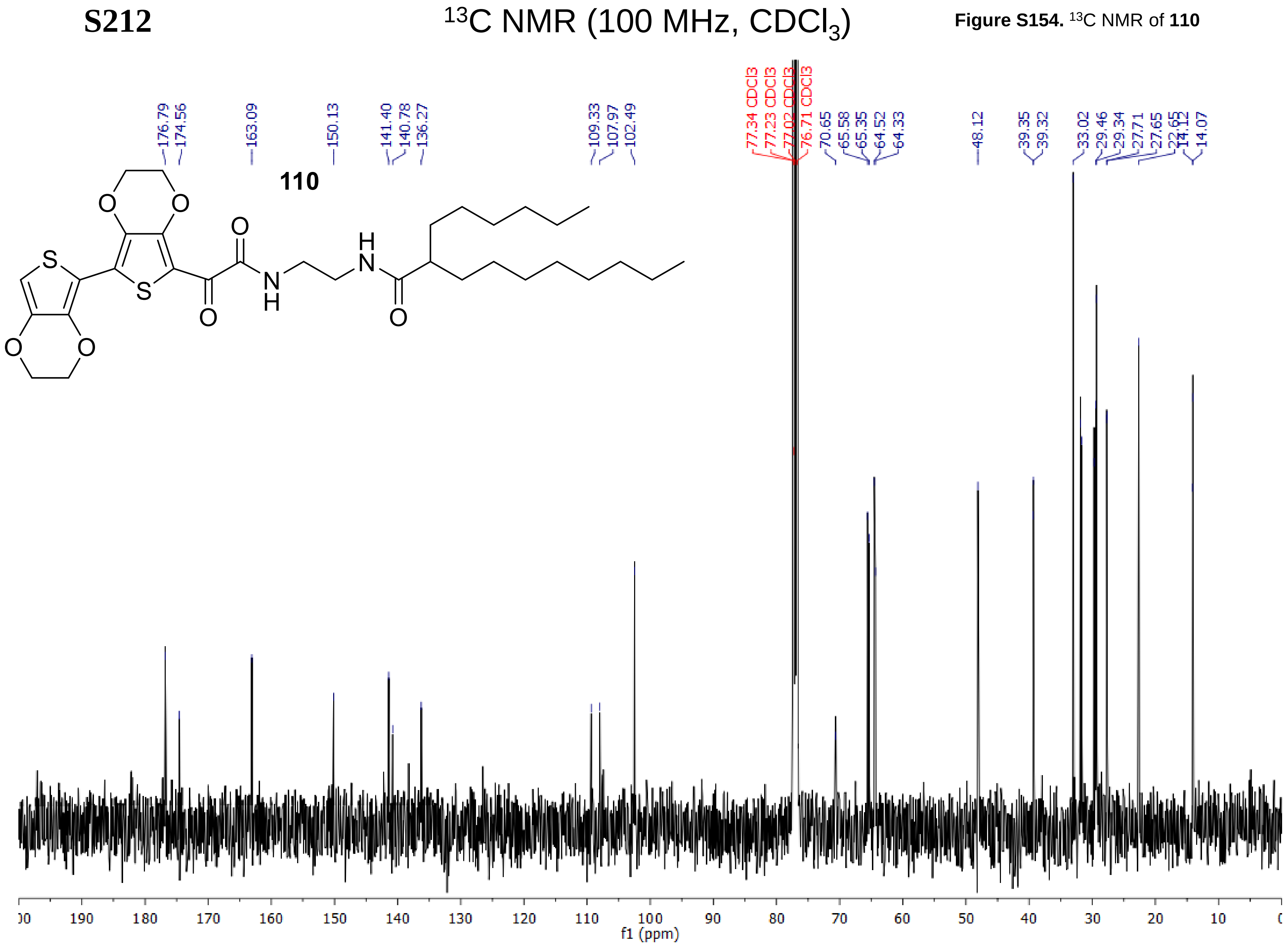

Figure S154. ^{13}C NMR of **110**

^{1}H NMR (400 MHz, $CDCl_3$)

Figure S155. ^{1}H NMR of **111**

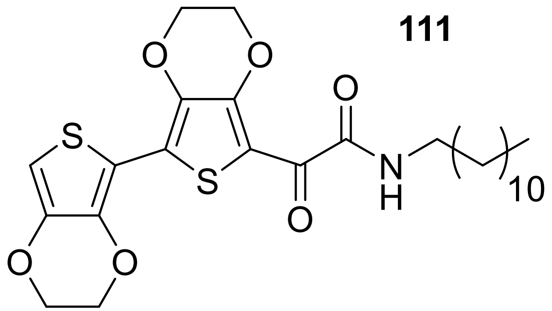

111

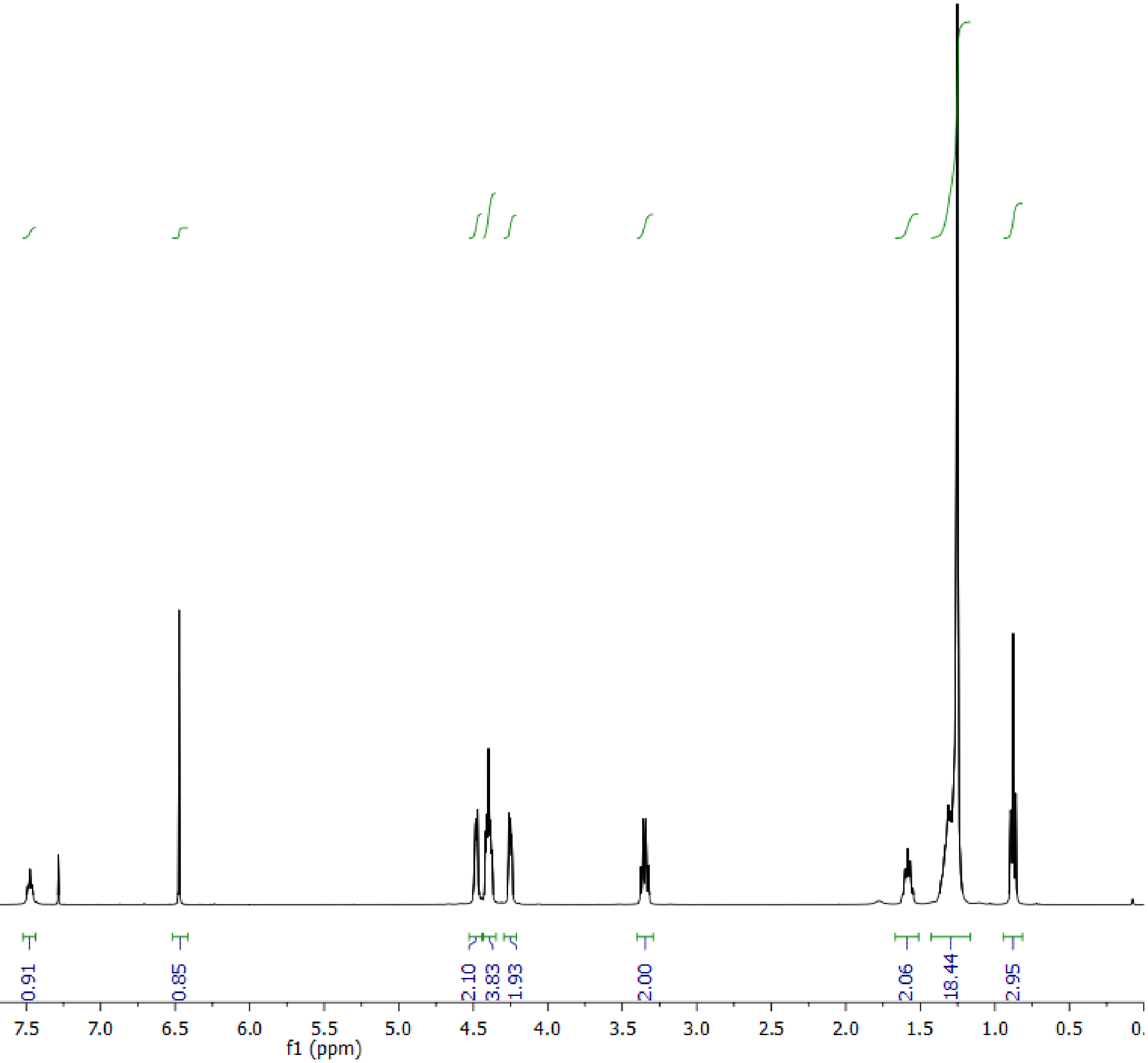

^{13}C NMR (100 MHz, $CDCl_3$)

Figure S156. ^{13}C NMR of **111**

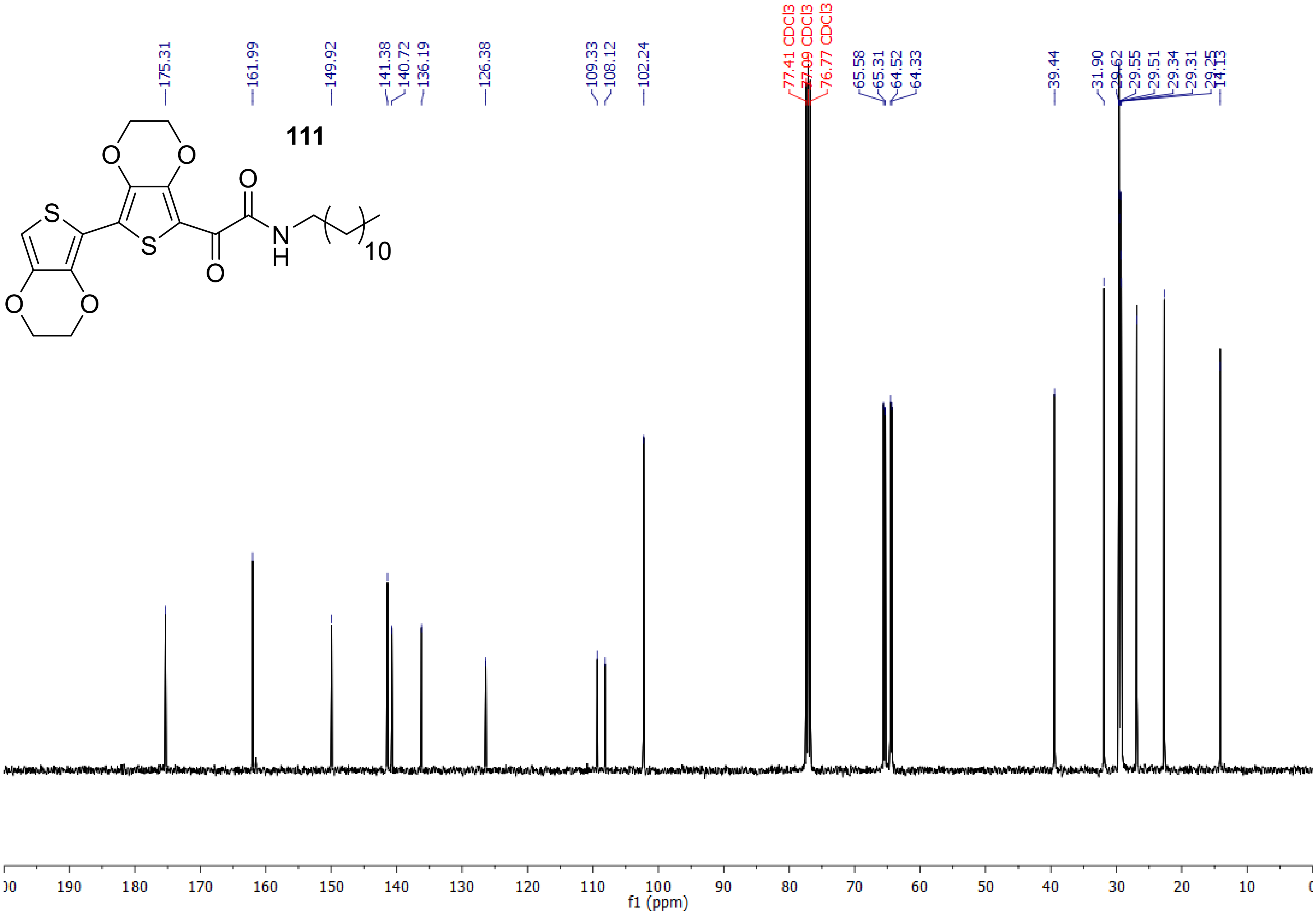

^{1}H NMR (400 MHz, $CDCl_3$)

Figure S157. ^{1}H NMR of **112**

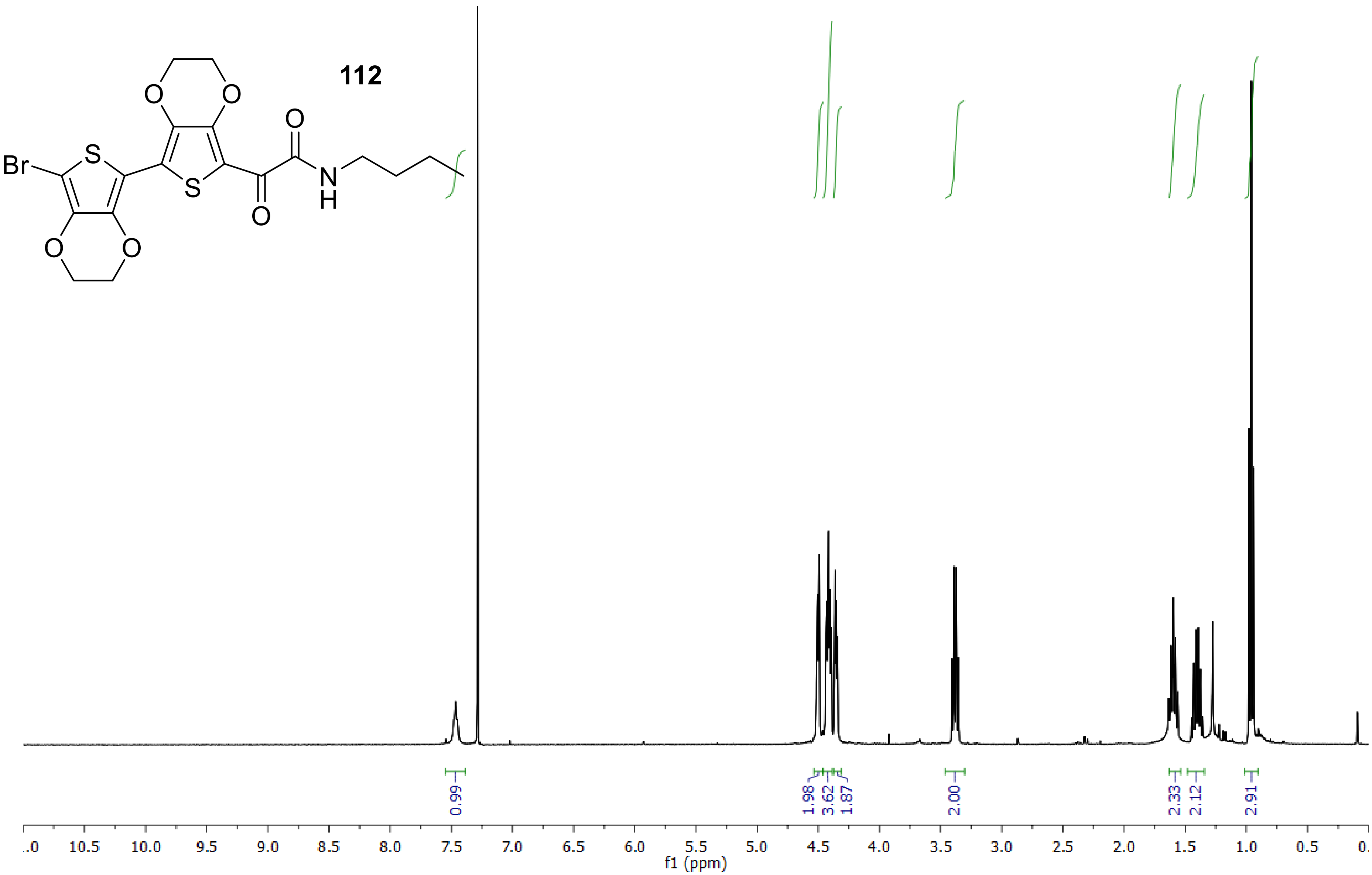

^{13}C NMR (100 MHz, $CDCl_3$)

Figure S158. ^{13}C NMR of **112**

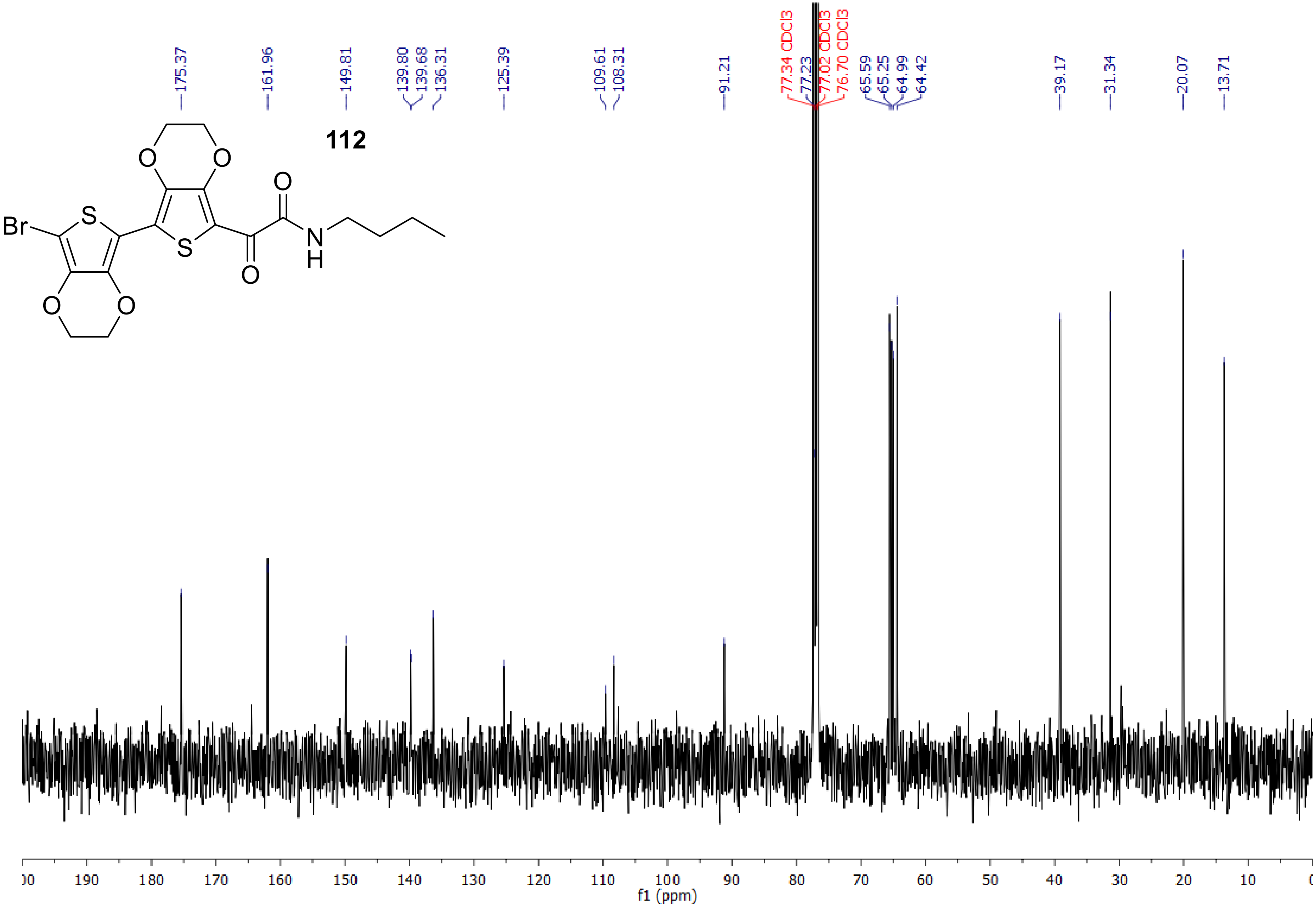

^{1}H NMR (400 MHz, DMSO)

Figure S159. ^{1}H NMR of **113**

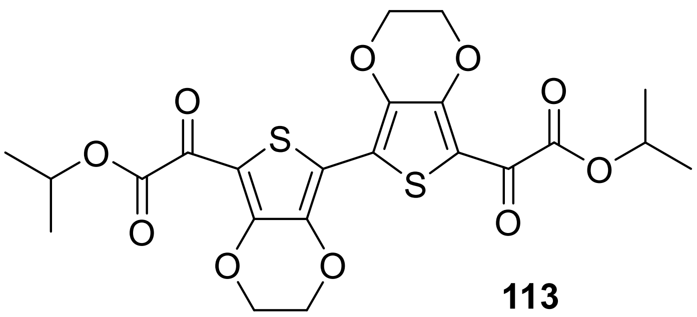

113

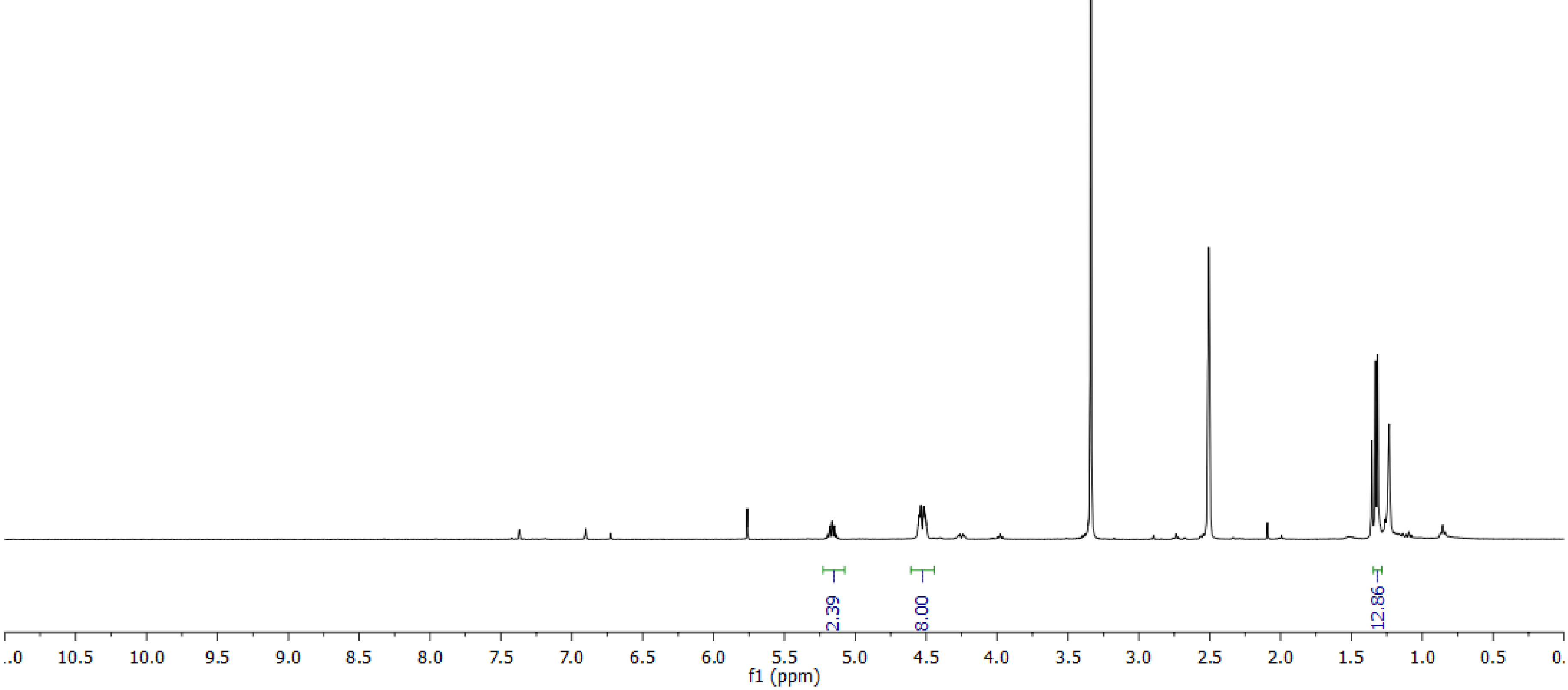

^{1}H NMR (400 MHz, $CDCl_3$)

Figure S160. ^{1}H NMR of **114**

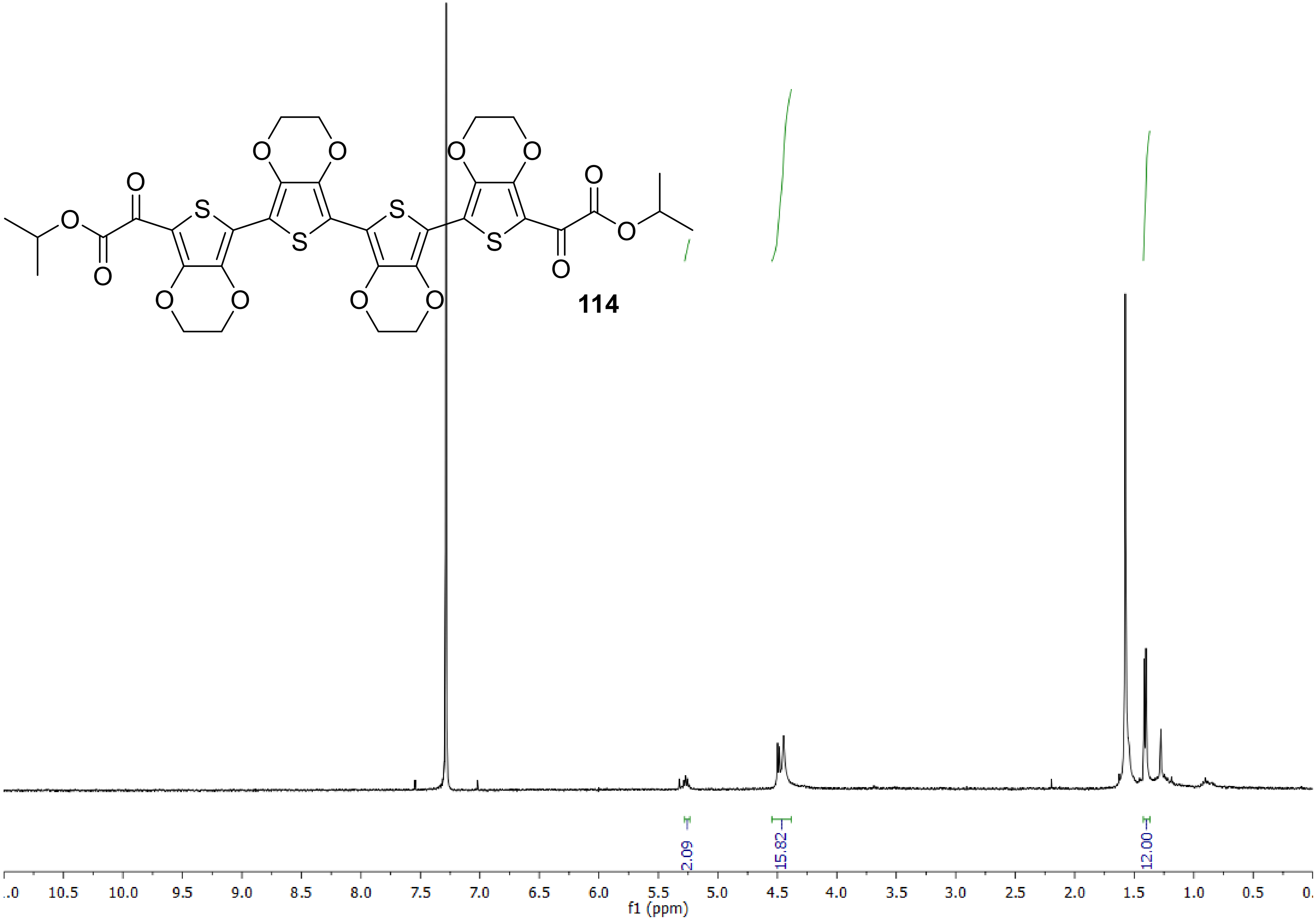

^{13}C NMR (125 MHz, $CDCl_3$)

Figure S161. ^{13}C NMR of **114**

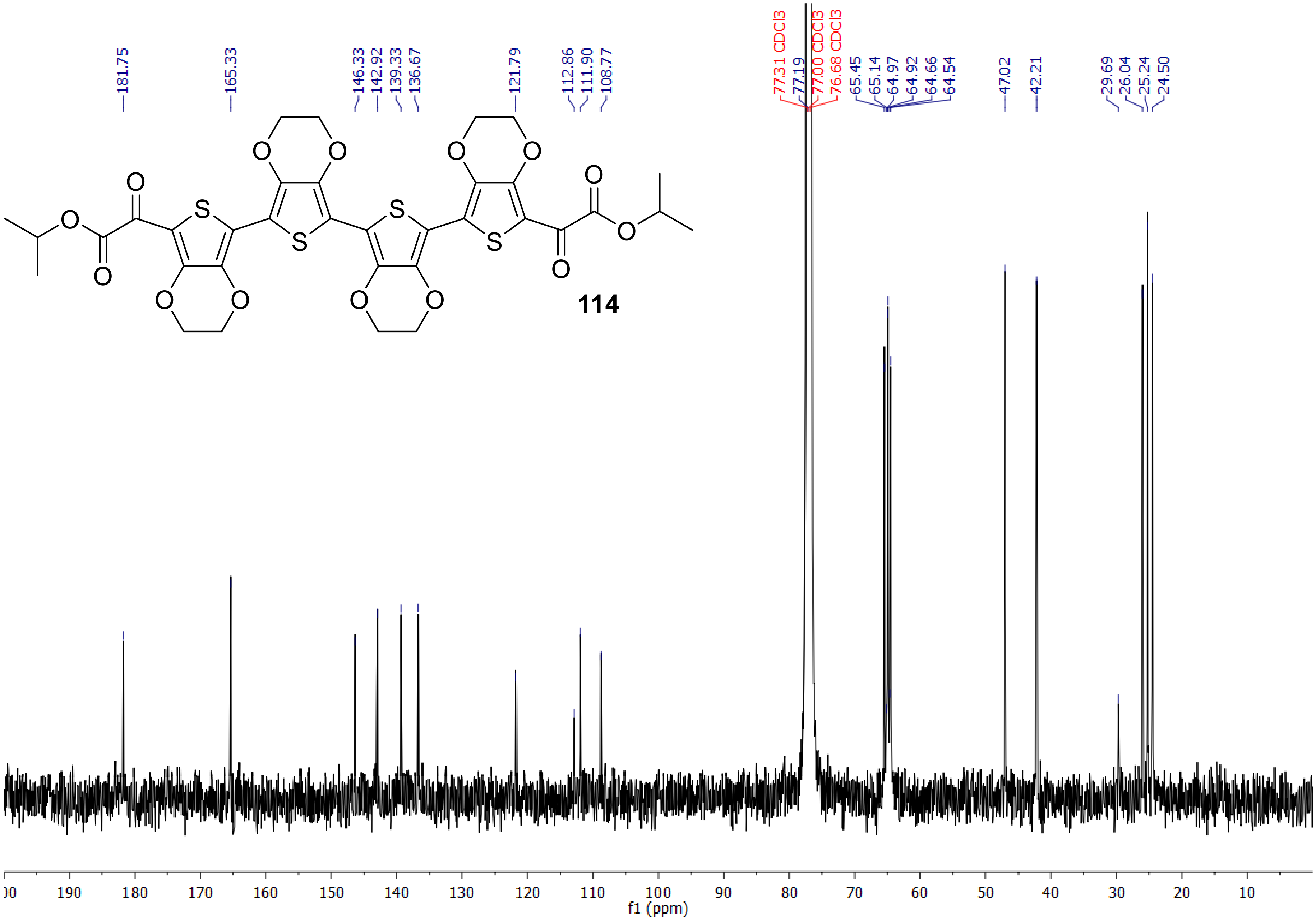

¹H NMR (400 MHz, $CDCl_3$)

Figure S162. ¹H NMR of **115**

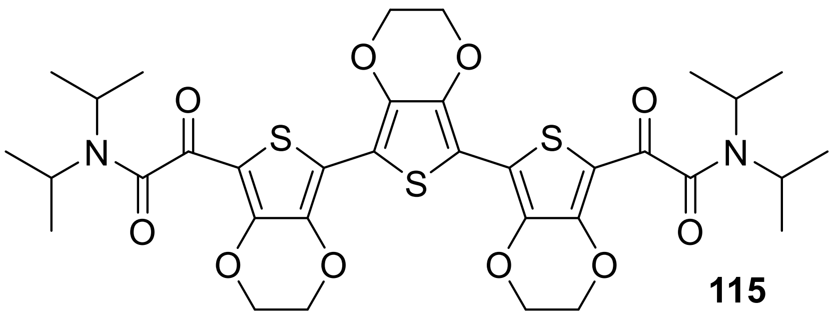

115

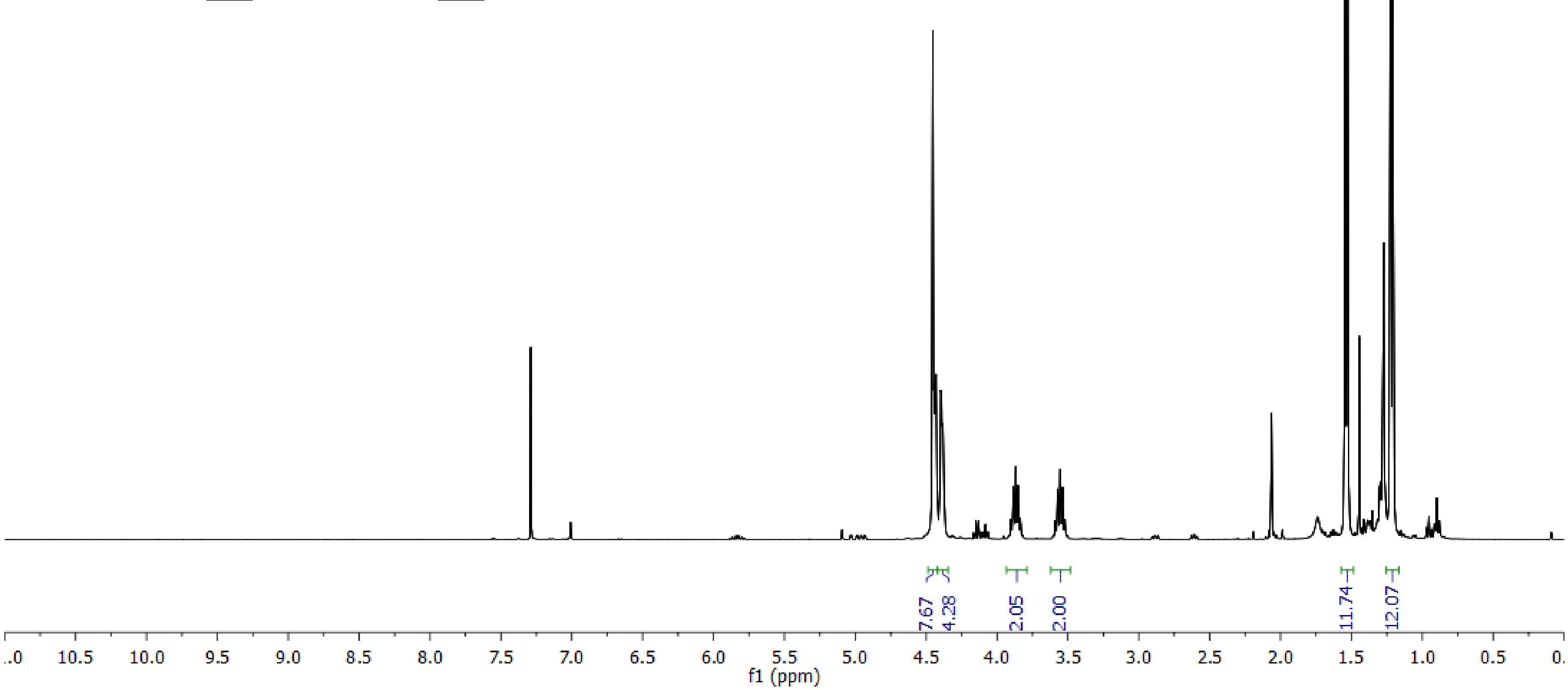

^{13}C NMR (100 MHz, $CDCl_3$)

Figure S163. ^{13}C NMR of **115**

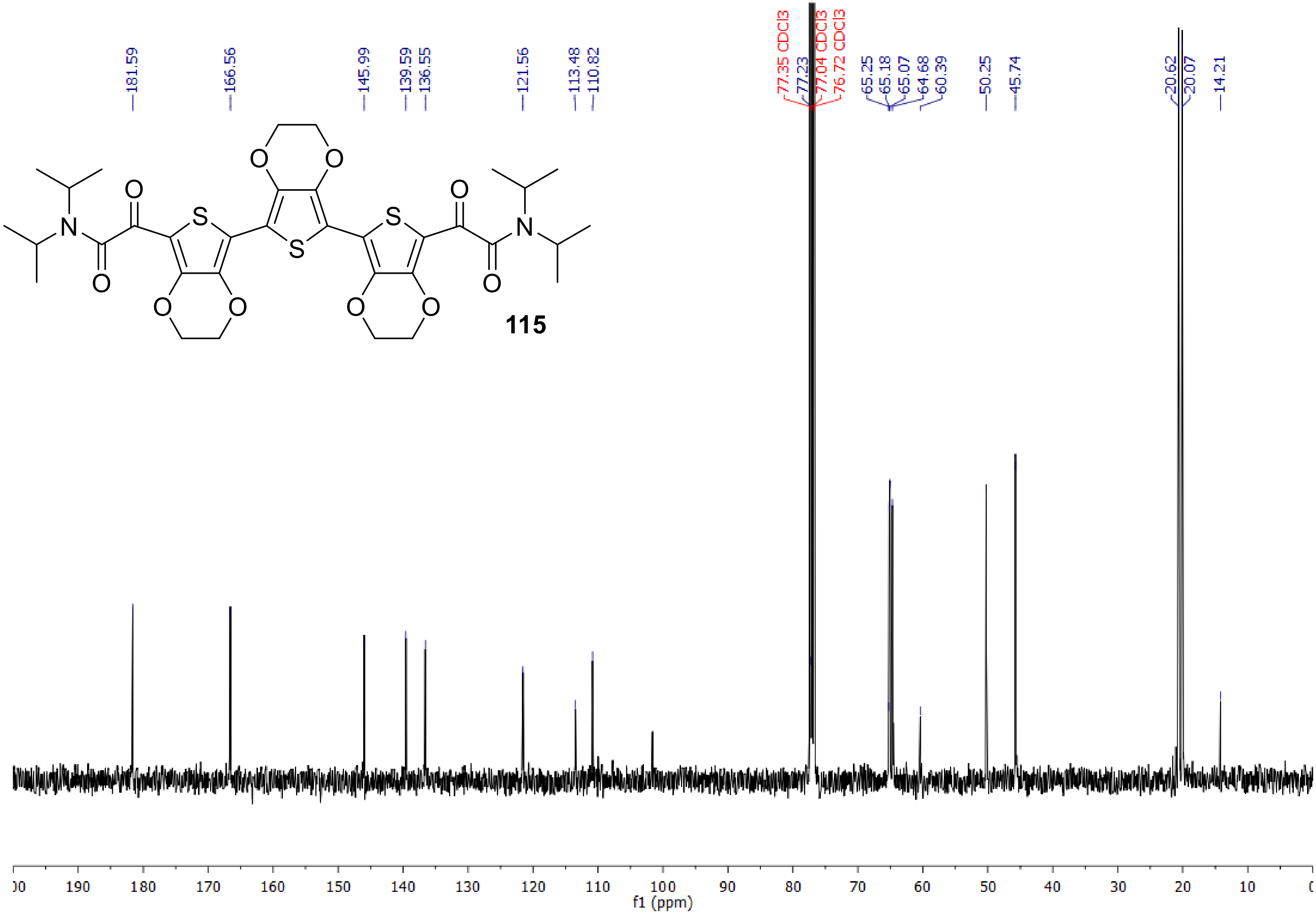

^{1}H NMR (400 MHz, $CDCl_3$)

Figure S164. ^{1}H NMR of **117**

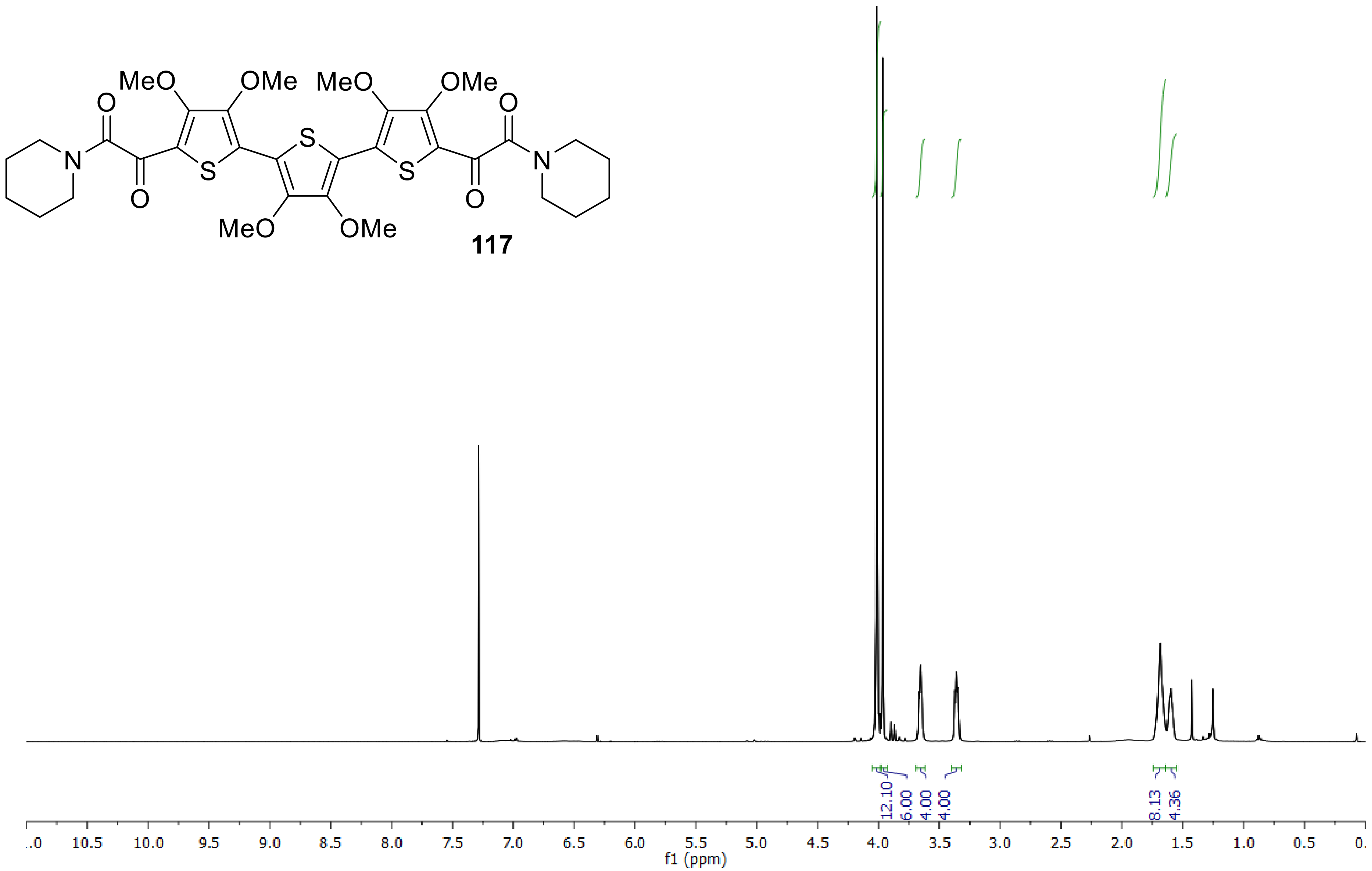

^{13}C NMR (100 MHz, $CDCl_3$)

Figure S165. ^{13}C NMR of **117**

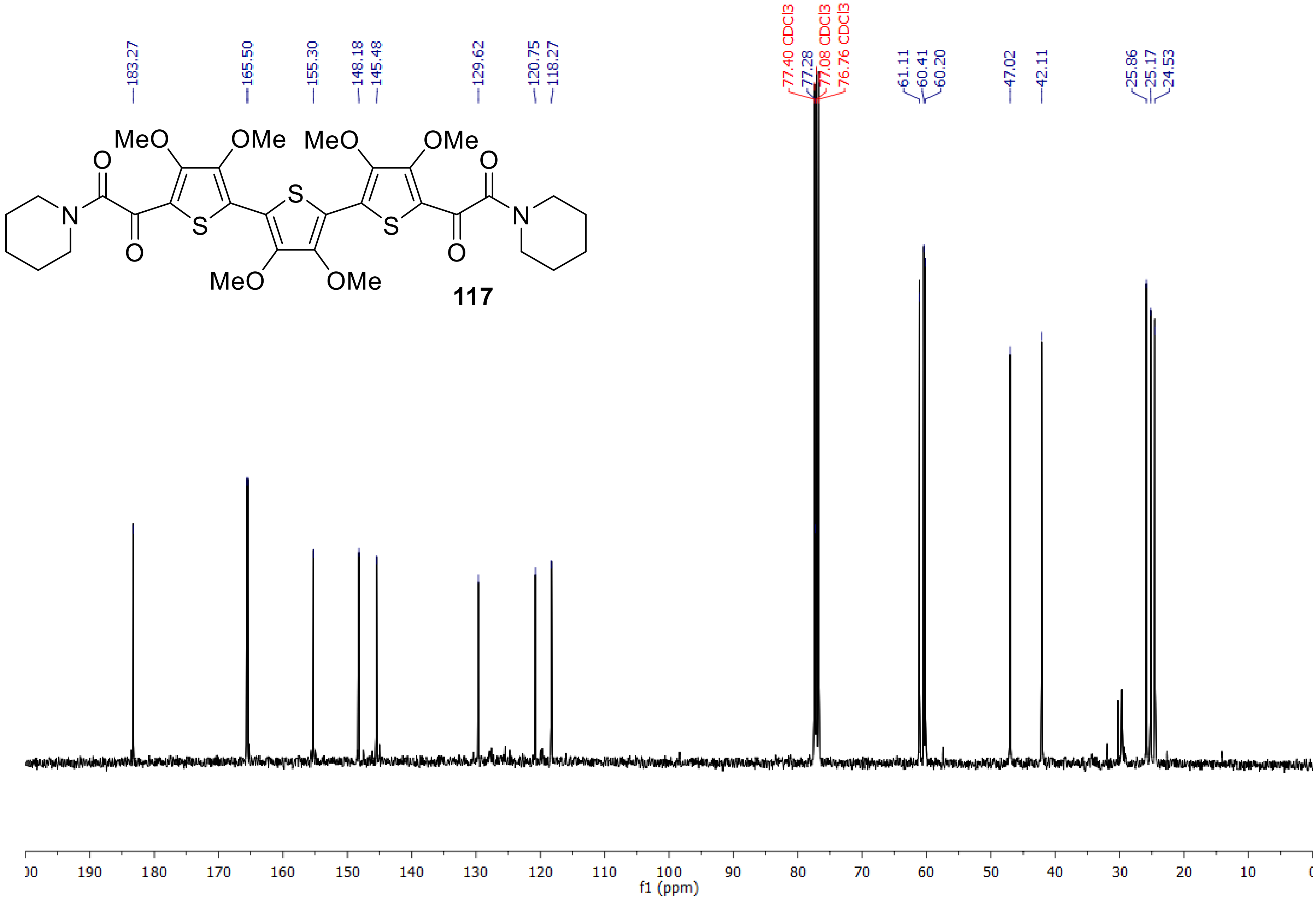

^{1}H NMR (400 MHz, $CDCl_3$)

Figure S166. ^{1}H NMR of **118**

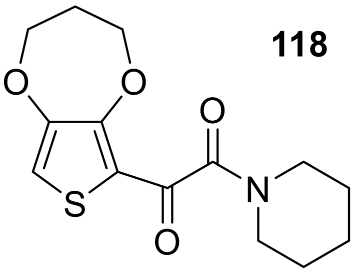

118

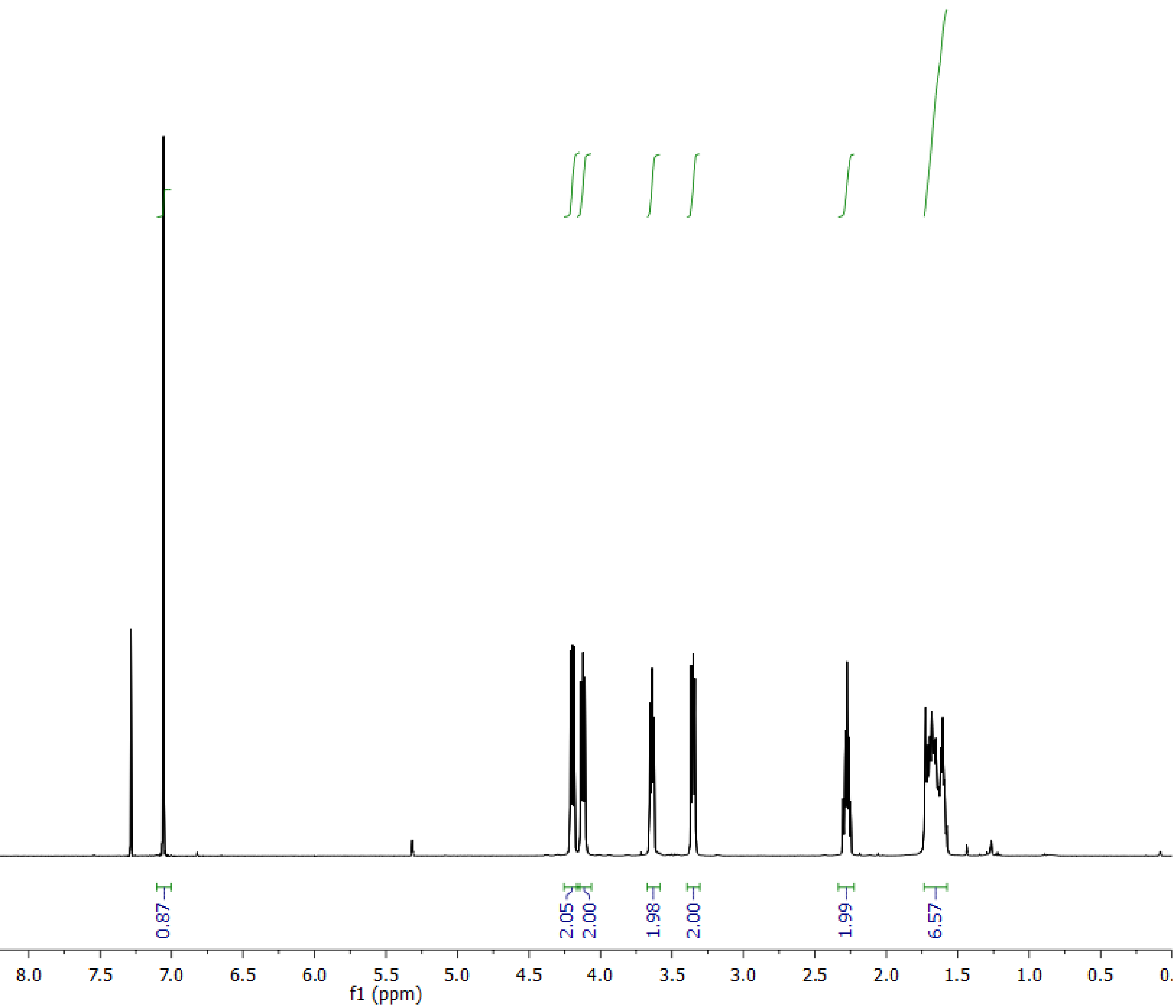

^{13}C NMR (100 MHz, $CDCl_3$)

Figure S167. ^{13}C NMR of **118**

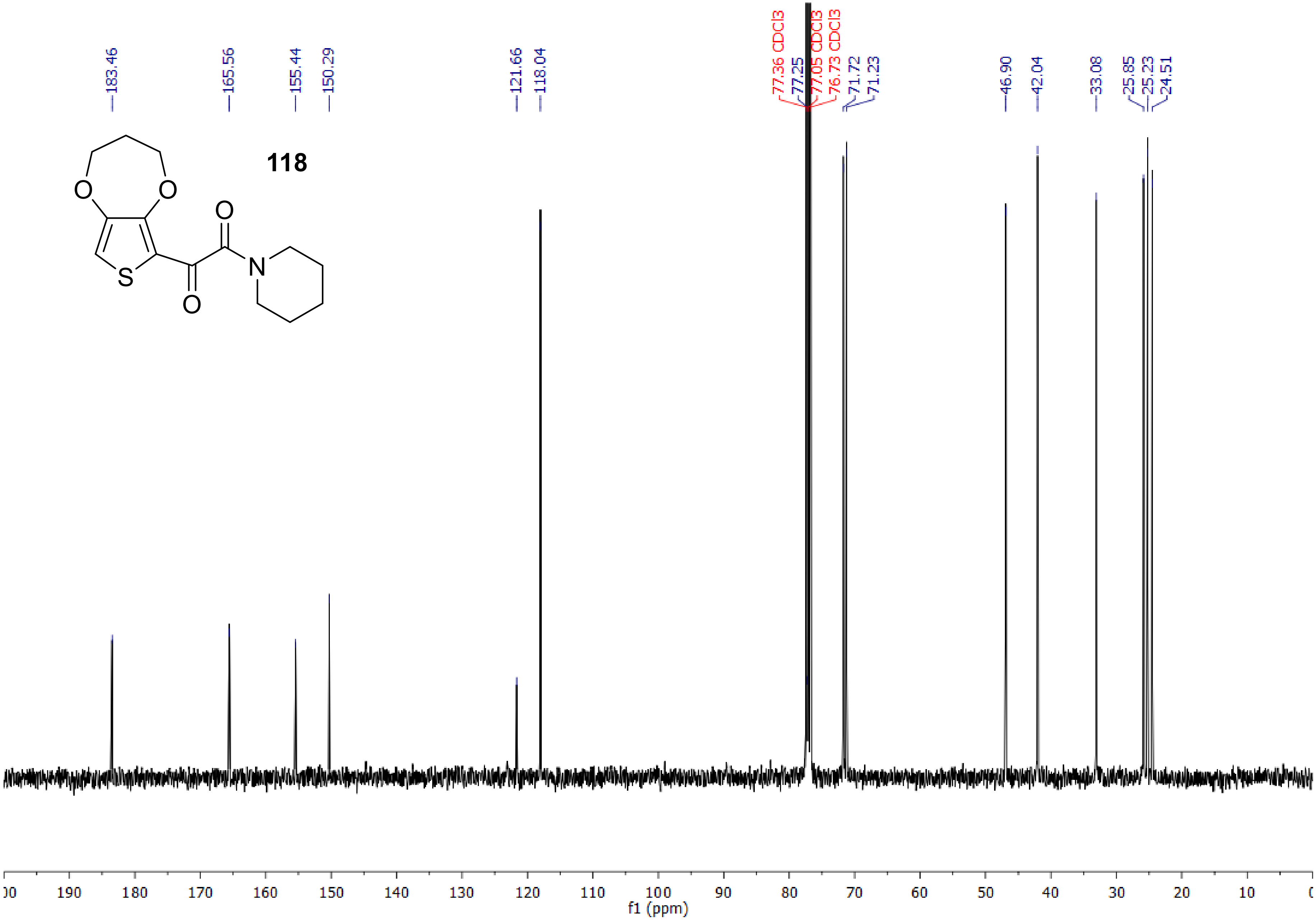

^{1}H NMR (400 MHz, $CDCl_3$)

Figure S168. ^{1}H NMR of **119**

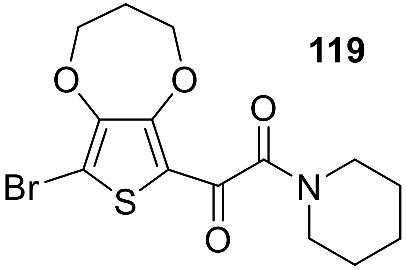

119

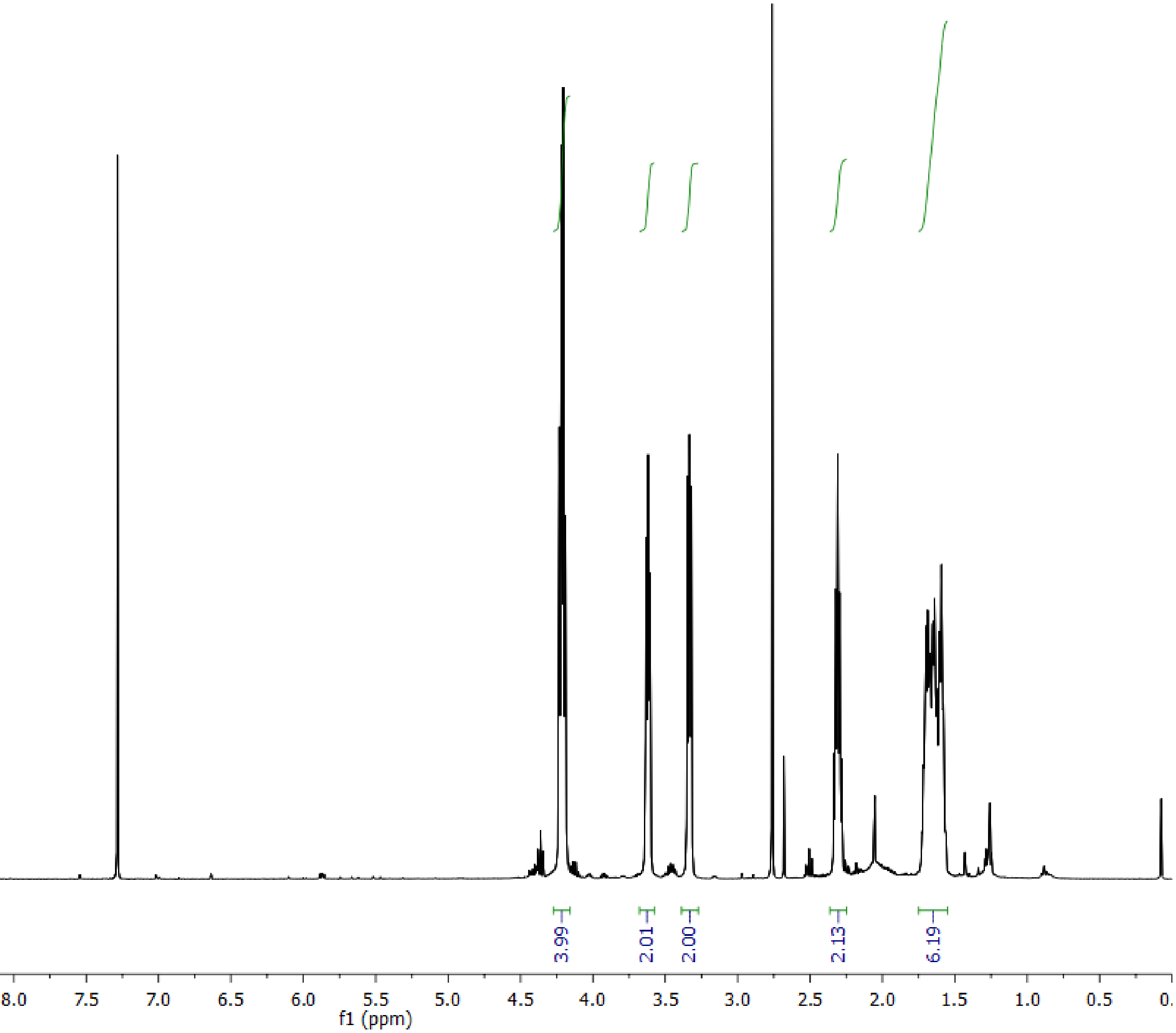

^{13}C NMR (100 MHz, $CDCl_3$)

Figure S169. ^{13}C NMR of **119**

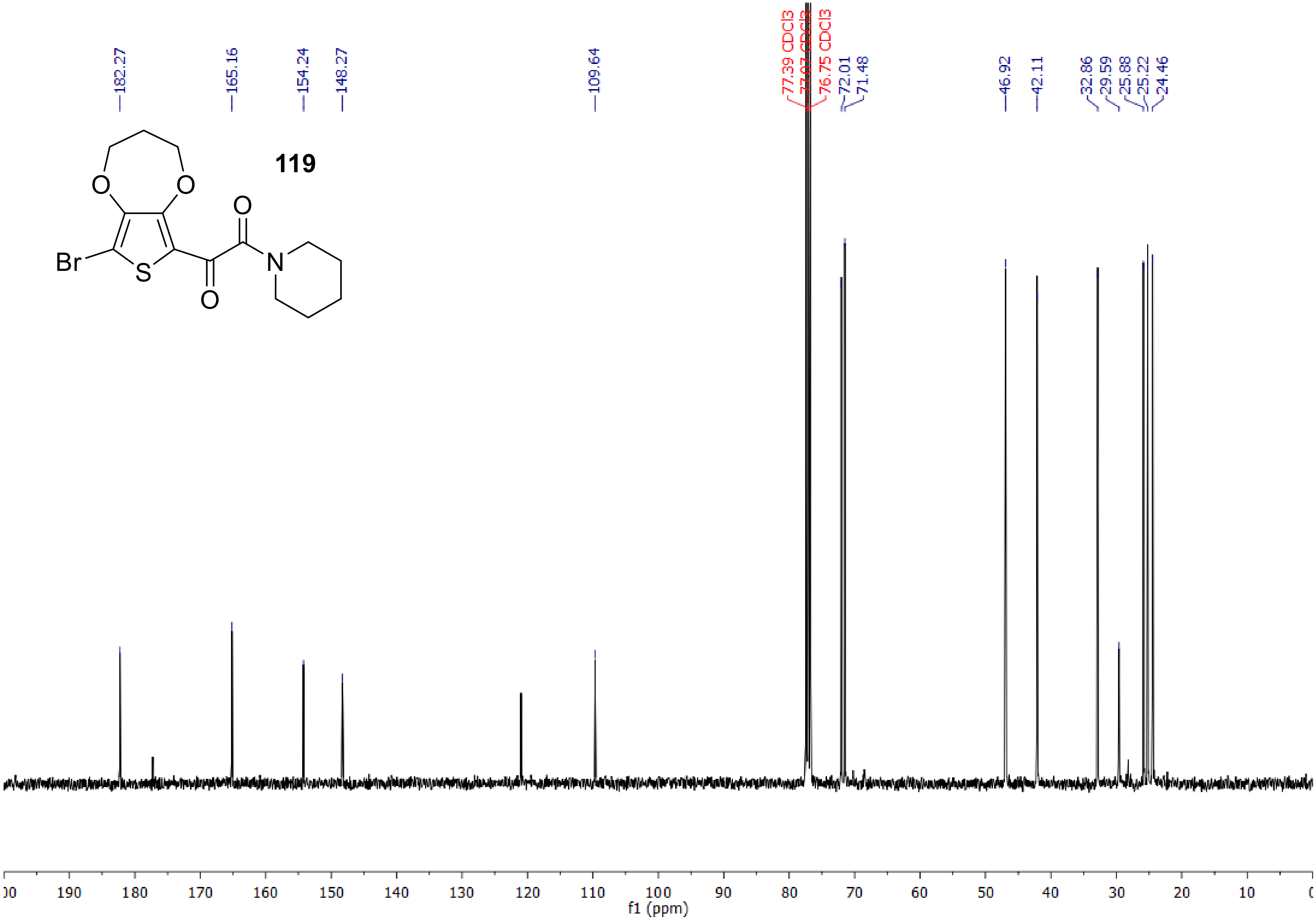

^{1}H NMR (400 MHz, $CDCl_3$)

Figure S170. ^{1}H NMR of **120**

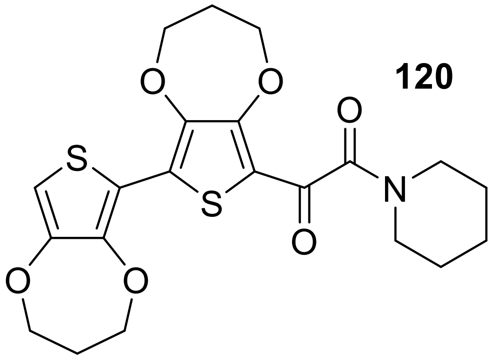

120

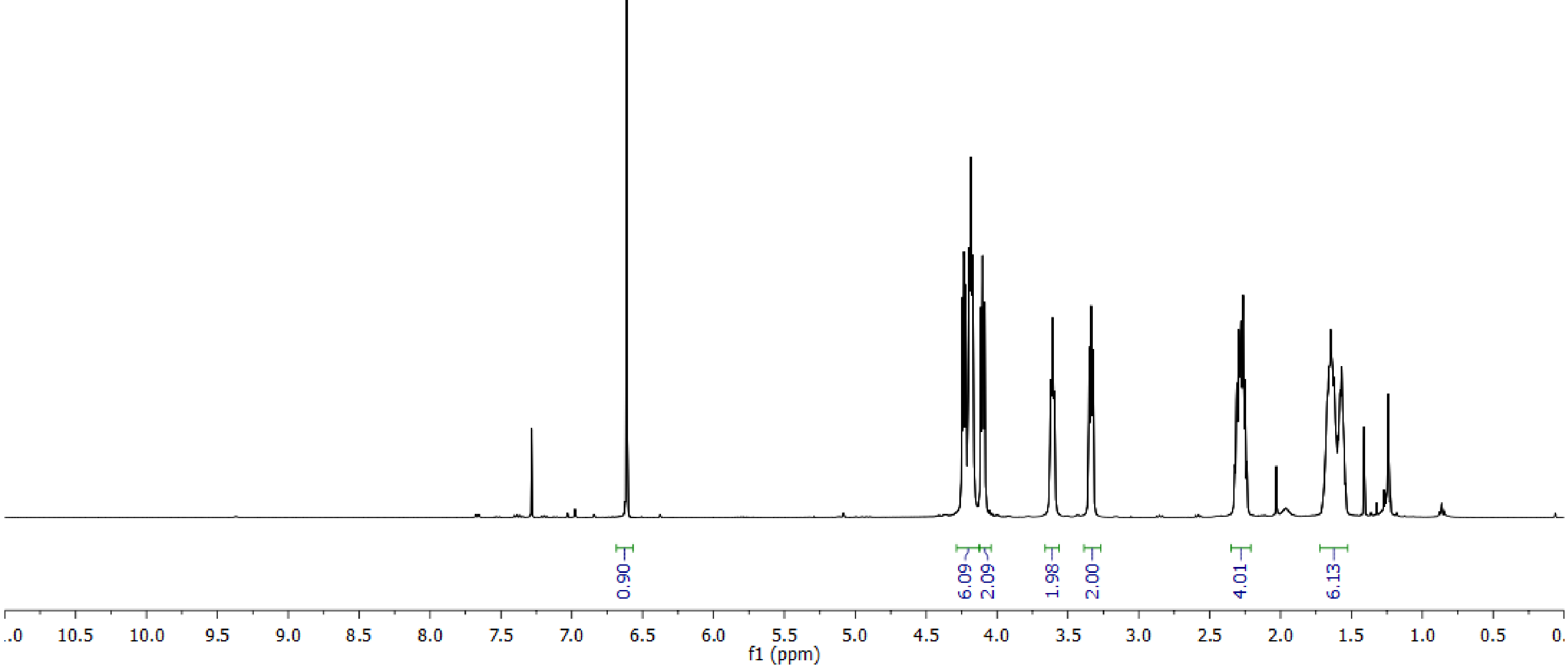

^{13}C NMR (100 MHz, $CDCl_3$)

Figure S171. ^{13}C NMR of **120**

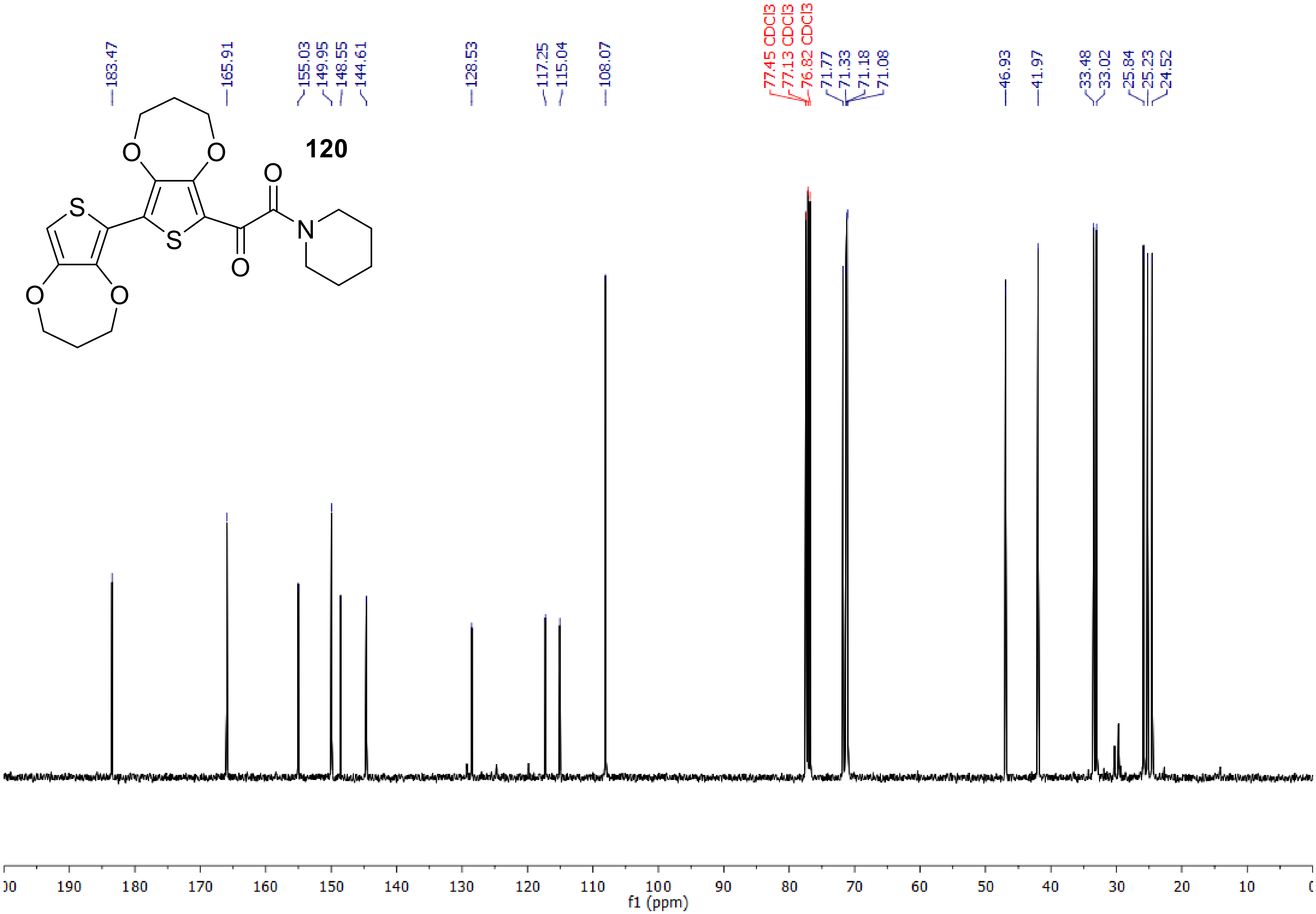

^{1}H NMR (400 MHz, $CDCl_3$)

Figure S172. ^{1}H NMR of **121**

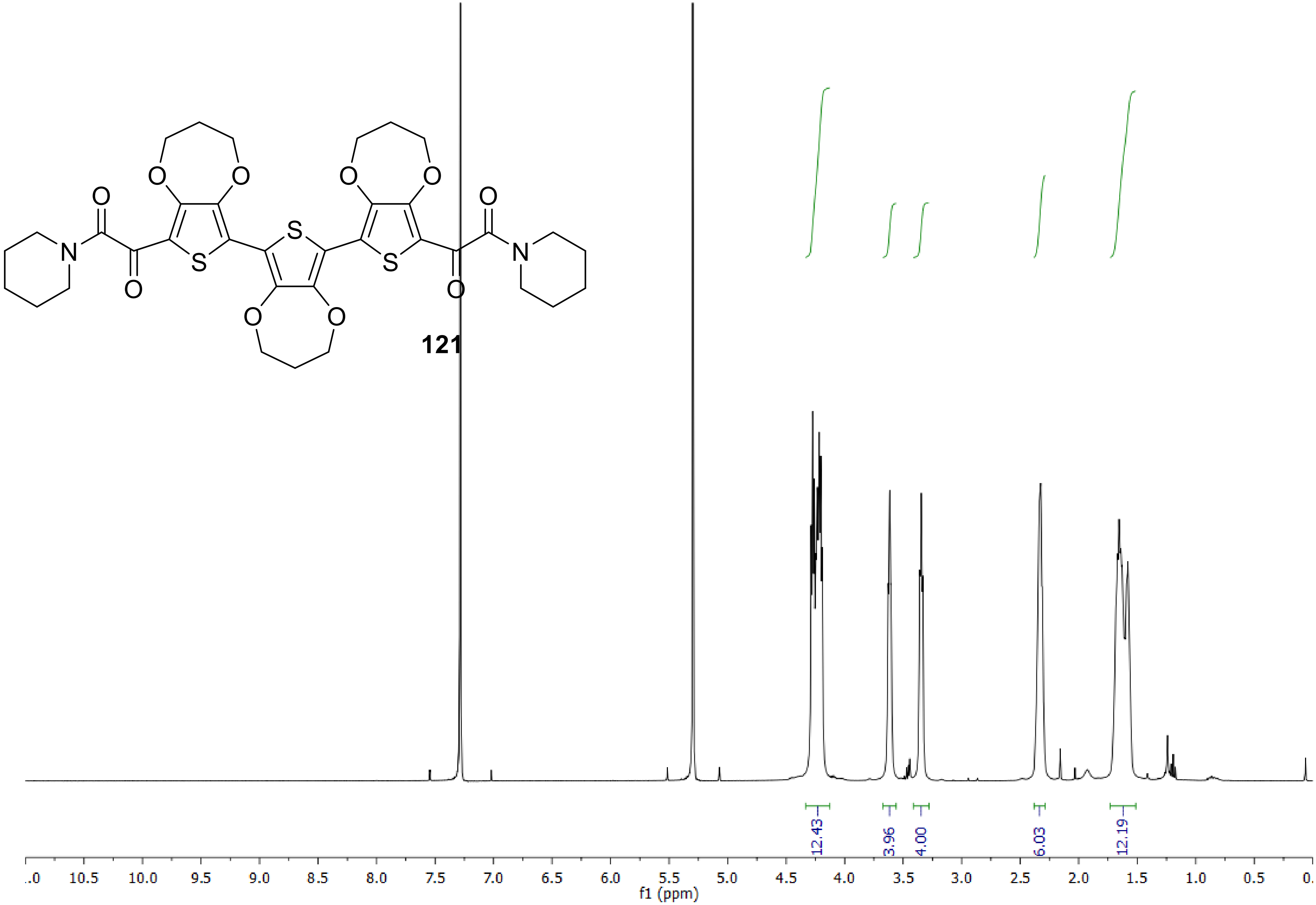

121

^{13}C NMR (100 MHz, $CDCl_3$)

Figure S173. ^{13}C NMR of **121**

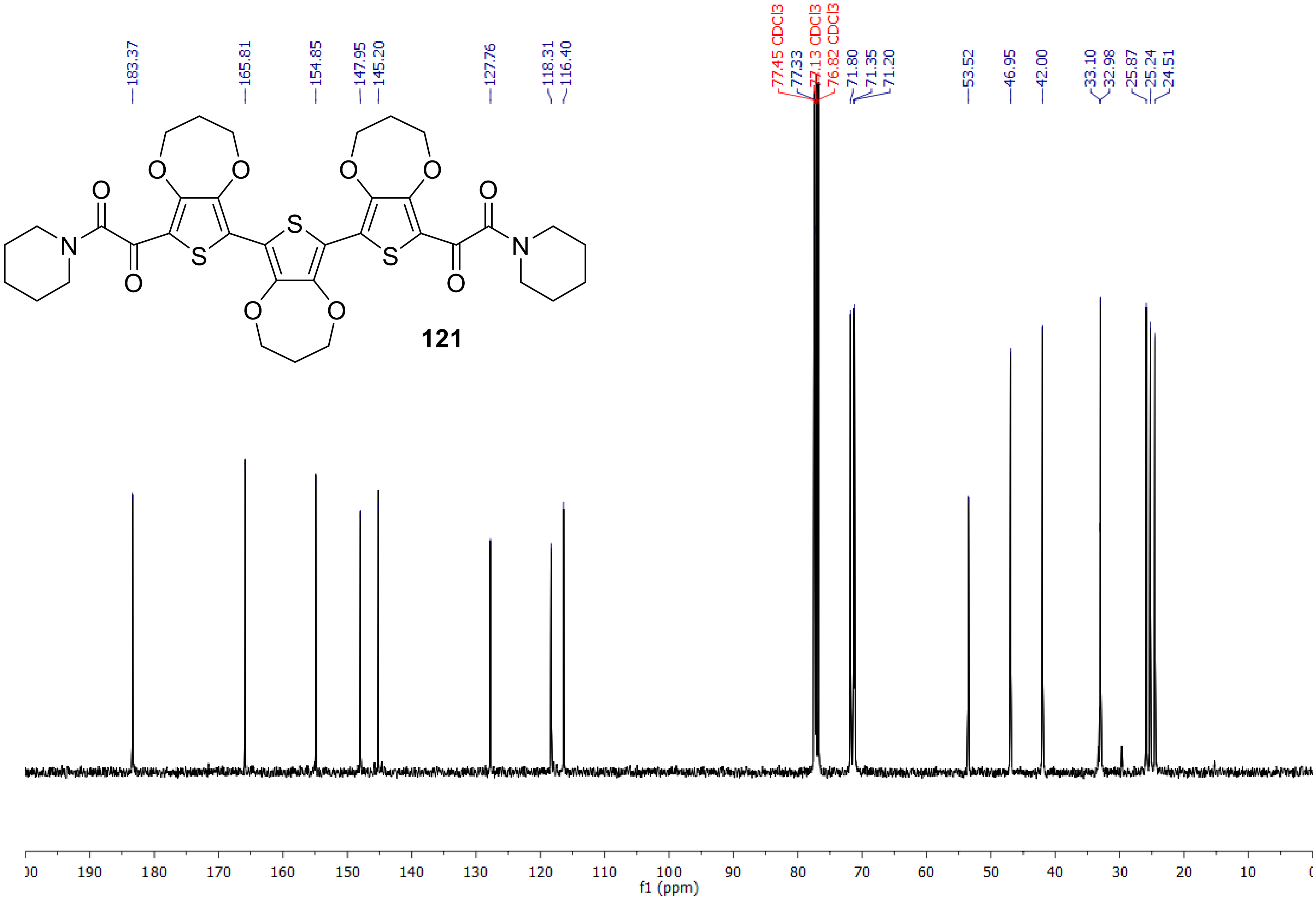

Supplement: Document S2. Article plus Supplemental Information [file mmc3.pdf]
